# Stakeholder perceptions on institutional design of digital health regulatory frameworks: insights from Kenya, Rwanda and Uganda

**DOI:** 10.1093/oodh/oqaf010

**Published:** 2025-05-09

**Authors:** Sharifah Sekalala, Shajoe J Lake

**Affiliations:** Centre for Global Health Law, School of Law, University of Warwick, Coventry CV4 7AL, United Kingdom; Centre for Global Health Law, School of Law, University of Warwick, Coventry CV4 7AL, United Kingdom

**Keywords:** digital health regulation, institutional design, Sub-Saharan Africa, HealthTech, health apps

## Abstract

Digital health holds significant promise for transforming healthcare but presents several risks to patients and providers, especially in fragmented regulatory terrains. Experts have articulated the need for clear digital health regulatory frameworks, but there is uncertainty surrounding the design of such frameworks with governments adopting varied models, spanning both formal and informal mechanisms. Using content analysis and a stakeholder dialogue with focus group discussions, we aimed to assess stakeholders’ perceptions of the benefits, costs, risks and trade-offs of different forms of regulatory frameworks in low- and middle-income countries, focusing on Kenya, Rwanda and Uganda. Stakeholders consider both formal and informal regulatory approaches to be beneficial, citing regulatory maturity, political will and financial support as key factors to consider. However, the aim of regulatory design should be patient protection, the key concern being how best to protect individuals’ and engender trust between citizens and government. Moreover, while stakeholder engagement is crucial, this should be done with a clear aim and is likely best done in the latter stages of regulation to facilitate peer review of initial regulatory efforts. Overall, context-specific, iterative strategies are key for digital health regulatory design, with patient protection, inclusive stakeholder engagement, flexible regulatory tools and enduring political and institutional support being key factors to consider.

## INTRODUCTION

### The promise of digital health

Significant advancements in computational power have fuelled the rapid expansion of digital platforms that engage consumers in health and wellness activities, collect and utilize their clinical data, and manage health outcomes, along with the quality of care [1–3]. Broadly termed digital health, this field includes innovations such as mobile health (mHealth) applications, wearable devices, online health and wellness platforms, digital equipment, telehealth and telemedicine, personalized medicine and artificial intelligence (AI) tools [2, 4–6]. These advancements have facilitated the generation and storage of complex datasets—genomic sequencing, electronic health (eHealth) records and medical imaging [1, 3, 7]. Data from millions of individuals can now be analyzed using AI, aiding physicians in diagnosing and treating illnesses.

Despite the potential benefits of digital health to broaden access to care, it presents challenges. Technological advancements have far outstripped the development of regulatory frameworks [1, 7, 8]. Digital health transformation is being driven primarily by the private sector, often with limited assurances that digital tools are rigorously validated or aligned with patient needs [1, 9]. Many digital health platforms often elude the purview of regulatory oversight [10]. Other risks like global internet outages, cyber-attacks and the potential for misinformation and automation bias may also arise from the deployment of unregulated products [10, 11]. This regulatory gap exacerbates ethical concerns, particularly regarding the potential for harm to both users and providers who rely on these tools for health-related decisions [10].

### What do we currently know about digital health regulation?

Existing research highlights the need for digital health regulatory frameworks. Some scholars note that the absence of a clear regulatory framework could result in an unregulated marketplace flooded with harmful or ineffective products, ultimately undermining public trust in digital health [12]. Proponents of the digital health equity framework, for instance, emphasize the need for regulatory frameworks to address the digital determinants of health to promote equitable access to technologies, especially for underserved populations [13–16]. Citing concerns about the long-term implications for patient privacy and quality of care in relation to the temporary relaxation of regulations to facilitate the rapid rollout of telehealth services and other digital health technologies during the COVID-19 pandemic, some scholars argue for regulatory frameworks that can adapt to emergency situations while ensuring patient safety and data security [17].

While scholars agree that digital health regulatory frameworks are essential, they are less clear about how frameworks should be designed. Broad principles for digital health regulation exist [2, 18, 19]. Some scholars have also called for legislation, while others have argued for guidelines that balance innovation with the protection of public health [11, 17]. Other scholars emphasize the need to establish policies that bridge technical and infrastructure gaps that foster the development of technologies, and to shift focus from innovation to regulation, coordination and sustainability for more truly equitable digital health systems [20, 21]. However, governments like the United Kingdom have cautioned against legislating, arguing that premature restrictions could be counterproductive [11]. Some scholars echo this scepticism, arguing that the role of law in digital health is ambivalent due to uncertainties surrounding consent and data usage laws, which implicate human rights law and could make law an enabler or obstacle to digital health regulation [22].

Au contraire, some scholars call for a whole system approach, foregrounding the multidisciplinary nature of digital health, which requires broad regulatory collaboration [23]. Some scholars agree, arguing that engaging stakeholders is crucial as these groups are often confused about the status of various digital health solutions, or can offer guidance on complex challenges introduced by digital health [12, 13, 24]. What is clear is that there is broad agreement on the need for ‘regulatory innovation’ [12].

### The value of this study

A deeper understanding of how to design regulatory frameworks is necessary. Governments and policymakers now have the critical responsibility of defining how safety and efficacy should be established and maintained. This is particularly important given the uniqueness and sensitivity of health data, and its profitability and the potential for data migration across borders [7–9]. Public administration theory on institutional design is a helpful starting point. Regulation is more than just law or policy; it implicates a range of institutions, warranting attention being paid to the broader institutional design of regulatory frameworks. Institutions, broadly defined as norms, conventions, instruments, systems and structures capable of overseeing the diverse and rapidly evolving landscape of digital health, are essential to establishing regulatory frameworks [4, 25–28]. Scholars note that low- and middle-income countries (LMICs) endure a significantly greater regulatory challenge [20]. To this end, we aimed to investigate how LMICs design their regulatory frameworks and key stakeholders’ perceptions of the benefits, costs, risks and trade-offs of these regulatory frameworks. Our study is based on data from Kenya, Rwanda and Uganda—three East African countries making strides in digital health regulation, using varied regulatory frameworks.

## METHODS

### Design

This article draws on qualitative data obtained through content analysis of eight publicly available documents, web searches and a two-day stakeholder dialogue and focus group discussions with 21 stakeholders within the digital health ecosystem in Kenya, Rwanda and Uganda. We pursued a two-phase data collection process in which we conducted a content analysis and a stakeholder dialogue with focus group discussions. We collected qualitative data on the institutional design of these countries’ frameworks and stakeholders’ perspectives on the design of the frameworks. The University of Warwick, Humanities & Social Sciences Research Ethics Committee approved this study [HSSREC 161/21–22] and the Uganda National Council for Science and Technology, where the fieldwork took place [SS1500ES]. The project methodology description in this section follows the consolidated criteria for reporting qualitative research (COREQ) [29].

### Country selection

We selected Kenya, Rwanda and Uganda as our focus countries. These three LMICs in East Africa have been making strides in organizing their digital health regulatory frameworks and have designed varied frameworks, using both formal (hard) versus informal (soft) mechanisms. These factors allowed us to gain key insights into stakeholders’ attitudes and perceptions, providing rich comparative data on benefits, costs, risks and trade-offs associated with different kinds of digital health regulatory frameworks.

### Analytical framework

The content analysis and focus group discussions were guided by an evaluation framework adapted from public administration theory [25, 26, 30, 31]. Certain evaluative criteria were additionally informed by literature on digital health regulation, as discussed in the introduction of this article [5, 9, 27, 32, 33] (Table 1). This framework was subsequently revised (Table 2), throughout the content analysis and stakeholder dialogue reported below, and in the penultimate session of the stakeholder dialogue, stakeholders validated the framework and further refined it, co-creating a tool for considering factors associated with design choices.

**Table 1:** Initial evaluative framework for conducting the content analysis

Domain	Meaning
Accountability	What foundational legal norm underpins the regulatory framework?
Administration	What organizational and human resource capacities exist to implement regulations?
Expertise	What technical and regulatory knowledge is included in the development and implementation of the framework?
Financing	What mechanisms for financing support the regulatory framework?
Participation	How are stakeholders engaged in the regulatory process?
Sponsor	What kinds of leadership arrangements support the regulatory agenda?

**Table 2:** Refined evaluative framework

Feature	Tier 1	Tier 2	Tier 3
Norm	Policies and guidelines	Hybrid	Acts of Parliament and Subsidiary Legislation
What kind of norm(s) buttress the framework?	An example of this would be guidelines, policies and standards.	An example of this would be a combination of elements from both Tiers 1 and 3.	An example of this would be legislation—an act of parliament.
Administration	Internal	Inter-Ministerial	External Agency
What level of administration supports implementation of the framework?	An example of this would be an internal team within a ministry.	An example of this would be the creation of a portfolio within ministries, including cross-sectoral teams.	An example of this would be the creation of a standalone government agency.
Expertise	Internal	External	Hybrid
How are experts engaged in digital health regulation?	An example of this would be consultation with experts within government.	An example of this would be consultation with experts external to government.	An example of this would be consultation with a combination of both internal and external experts.
Financing	Existing	Redistributed	Budgetary
What is the source of funding for digital health regulation implementation?	An example of this would be funds from existing programmes within a ministry.	An example of this would be funds appropriated to a ministry, which is then used for digital health and/or funding from international or local donors.	An example of this would be appropriation in national budget.
Participation	Laissez-faire	Ad-Hoc	Integrated
What level of engagement is there with stakeholders?	An example of this would be no established committee, but some consultation.	An example of this would be a standing committee which includes stakeholders who meet on a need basis.	An example of this would be an external, self-governed council of stakeholders.
Sponsor	Junior	Senior	Political
Where does leadership sit within government for digital health?	An example of this would be any civil servant who is ranked below C-Suite/Executive level.	An example of this would be a C-Suite/Executive-level civil servant in a ministry like the Permanent Secretary.	An example of this would be a member of the Cabinet.

### Content analysis

We analyzed publicly available documents that provided descriptive information about how each country has designed its digital health regulatory frameworks, including national (digital) health and ICT strategies, e-health policies, website descriptions, budget reports and legislation. Documents were collected between January and August 2024 by two researchers (SJL/SS). One researcher (SS) obtained some documents through in-country collaborators in Uganda and one researcher (SJL) identified other relevant documents by searching websites. Where multiple versions of similar documents were available, we used the most recent iteration. A total of eight documents were identified for our analysis (supplementary materials). All documents were available online. Once documents were collected, one researcher (SJL) extracted information about the institutional design of countries’ regulatory frameworks using our evaluative framework. We refined our framework during the content analysis to account for the different design choices countries made, engaging in an iterative process of reviewing documents and building out the framework. Our framework was validated at the stakeholder dialogue in the penultimate plenary session (Table 2).

### Focus group discussions

A 2-day stakeholder dialogue was held in Kampala, Uganda, comprising eight plenary sessions and four focus group discussions. Each focus group consisted of 8–11 participants (Table 3) and followed a semi-structured discussion guide (supplementary materials). Two researchers (SJL/HKG) led focus group discussions and recorded notes. The discussion guide, developed based on the content analysis, aimed to explore each country’s design choices. Stakeholders were invited to participate in the stakeholder dialogue based on their ability to contribute to the discussions, considering their expertise, seniority and active involvement as actors in the digital health ecosystem of the selected countries, as well as recommendations from Ugandan government stakeholders. Stakeholders included government officials, data protection officers, representatives from health and ICT ministries, health standards bodies, researchers, activists, media and international and regional organizations.

**Table 3:** Description of stakeholders

Participant characteristics	
Stakeholder	Organization type
Policymaker	
Senior Civil Servant	Ministry of Health (MoH), Uganda
Junior Civil Servant	MoH, Rwanda
Senior Civil Servant	MoH, Kenya
Junior Civil Servant	MoH, Uganda
Senior Civil Servant	MoH, Uganda
Senior Civil Servant	MoH, Uganda
Senior Civil Servant	MoH, Uganda
Junior Civil Servant	Personal Data Protection Office, Uganda
Senior Civil Servant	Office of the Data Protection Commissioner, Kenya
Academia	
Professor	University of Warwick (UoW)
Research Associate	UoW
PhD Researcher	Makerere University
Industry	
Developer	BioPharma, Uganda
Researcher	Innovation, Kenya
Civil Society	
Representative 1	Afya Na Haki, Uganda
Representative 2	Afya Na Haki, Uganda
Representative 3	Afya Na Haki, Uganda
Representative 4	CIPESA
International Institutions	
Programme Director	Africa Centres for Disease Control and Prevention
Media	
Communications Professional	Uganda
Communications Professional	Afya Na Haki, Uganda

### Data analysis

We employed thematic analysis that integrated prior knowledge with an open coding process to identify themes within the focus group discussion data. The analytical procedure adhered to Yin’s five stages of qualitative analysis: compiling, disassembling, reassembling, interpreting and concluding [34]. First, notes were read multiple times to ensure familiarity with the data and to develop a comprehensive understanding of the discussions. Thematic coding was then carried out using an abductive approach. A deductive coding framework was informed by the design features identified during the content analysis, while inductive coding enabled the emergence of new insights beyond the predefined framework. We also explored cross-country variations, identifying how contextual factors shaped design choices. Synthesizing the results of this intermediate analysis involved pinpointing dominant thematic findings that spanned different dimensions and categories of our framework. These findings were refined as the research team interpreted the results and formulated conclusions.

## RESULTS

### Content analysis

**Figure 1:**
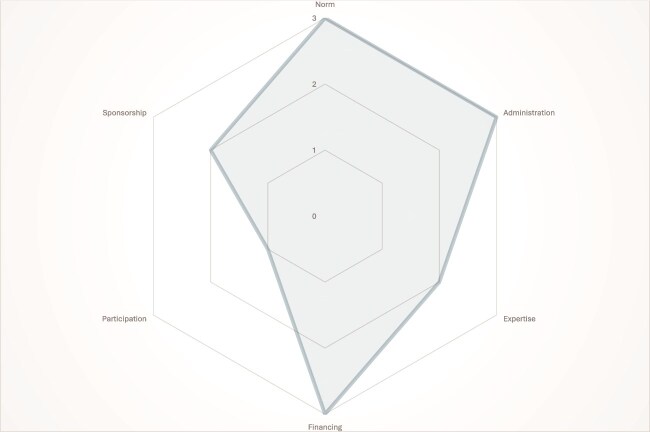
Institutional Design of Kenya’s digital Health regulatory framework

**Figure 2:**
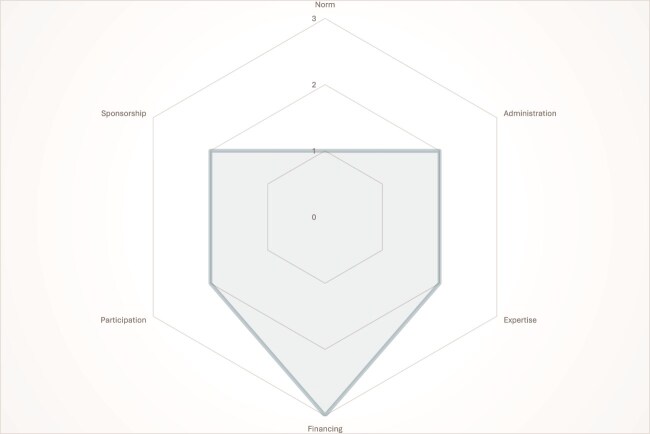
Institutional Design of Rwanda’s digital Health regulatory framework

**Figure 3:**
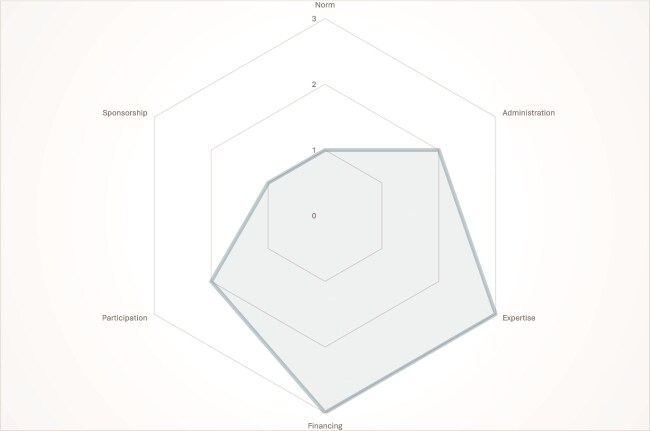
Institutional Design of Uganda’s digital Health regulatory framework

Our content analysis confirmed that while digital health regulatory frameworks’ design choices differed across countries, they shared six main overarching design features: norms, administration, participation, sponsorship, expertise and financing. These features could be broken down into three sub-features each (Table 4). We organized these sub-features on a continuum, most starting from inside government as a core, going outwards. Rather than attaching any value criteria to these sub-features, we considered them to be typologies—various ways in which these features manifest along a continuum. For example, the starting point for administration is an internal government ministry or department team, while at the end of the continuum, there is an external agency. Each country’s framework was coded along the continuum, with 0, 1, 2 and 3 being used to represent, no information found, Tier 1, Tier 2 and Tier 3, respectively (Figs 1–3). This is exemplified where Kenya was ranked at Tier 3 for having a Digital Health Agency, while Rwanda and Uganda were ranked Tier 2 for having interdepartmental teams administering the digital health strategy.

**Table 4:** Tiers within each design feature

Feature	Tier 1	Tier 2	Tier 3
Norm	Policies, Guidelines	Hybrid	Acts of Parliament
Administration	Internal	Inter-ministerial	External Agency
Expertise	Internal	External	Hybrid
Financing	Existing	Redistributed	Budgetary
Participation	Laissez-faire	Ad-hoc	Integrated
Sponsor	Junior	Senior	Political

### Focus group discussions

#### Normative frameworks are context-dependent

The normative basis of the institutional design of digital health regulatory frameworks in Kenya, Rwanda and Uganda reflects a diverse spectrum of approaches, ranging from formal legislative structures to informal guidelines. Kenya’s model, grounded in the Digital Health Act, exemplifies a formalized legislative approach. This legislation established a Digital Health Agency and a centralized Health Information Exchange to consolidate and oversee the management of citizens’ health data. Stakeholders noted that the key question when contemplating the normative anchor was: what is the best way to protect Kenyans’ health data? Stakeholders highlighted that they felt the sensitive nature of health data required not only robust governance but also public trust in the state’s handling of such data. To address these concerns, the Kenyan government anchored its regulatory framework in a formal statute to promote accountability, enforceability and public confidence.

In contrast, Uganda has adopted a less formalized approach, relying primarily on guidelines and standards. This softer regulatory strategy reflects Uganda’s current developmental stage in digital health. However, stakeholders pointed out significant shortcomings in this model, particularly its tendency to create fragmentation and uncertainty regarding industry obligations. Uganda’s guidelines have been published as a compendium. Still, stakeholders felt this informality raises concerns about the enforceability and legitimacy of the regulatory framework. Industry players highlighted that the lack of a formal normative anchor could undermine compliance and dilute the perceived authority of regulatory mechanisms.

Rwanda currently has a digital health strategy in place, but one stakeholder noted that consideration was being given to a hybrid model that would combine soft norms (guidelines and standards) with hard norms (legislation), presenting a middle ground that seeks to balance adaptability with enforceability. Stakeholders agreed that this hybrid model allows for rapid responsiveness to technological changes while simultaneously providing the stability and clarity often thought to be offered by legislation. This hybrid approach reflects an evolving understanding of the regulatory trade-offs: while informal norms excel in flexibility and ease of implementation, they risk ambiguity and non-compliance. Conversely, formal legislation, though resource-intensive to develop, offers a durable and enforceable foundation for regulation.

### Stakeholder engagement is fundamental

Stakeholders agreed that engaging a broad array of actors across the digital health ecosystem is pivotal in designing digital health regulatory frameworks. Participation in regulatory processes ranged from inclusive, multi-stakeholder platforms in some countries to more centralized, government-driven approaches in others. For example, Uganda and Rwanda had committees of stakeholders involved at various stages of the design process, with Uganda engaging stakeholders in consultation and Rwanda forming stakeholder groups and sub-groups to work on various projects within the digital health strategic plan development. Kenyan stakeholders emphasized that the need for consultation was based on dialogue with stakeholders in the banking and financial sectors who had experience with financial technology regulation. Stakeholders, however, emphasized that the substance rather than a form of engagement is what is crucial. They emphasized the need for broad and inclusive consultations with diverse stakeholders—ranging from citizens and healthcare providers to technology developers, regulators and government agencies. Inclusive engagement not only enhances compliance but also ensures that frameworks are responsive to the varied needs of affected parties.

Stakeholders, however, felt that stakeholder engagement is nuanced. During the early stages of regulatory development, consultations help establish priorities and shape legislative drafting, ensuring that initial frameworks are grounded in real-world concerns. In later phases of implementation and evaluation, more focused and in-depth involvement of stakeholders ensures that regulations remain relevant and effective in practice. Stakeholders felt a phased approach with only light consultations in the beginning was necessary, but this is context dependent as some Kenyan stakeholders note that there were legal requirements for consultation to take a specific form from inception.

Ugandan and Rwandan stakeholders underscored the necessity of incorporating cross-disciplinary expertise in the design process. Legal, technical, medical and ethical knowledge domains were identified as essential for crafting regulatory frameworks that are both comprehensive and responsive to the complexities of digital health. A regulatory framework that neglects the technical or ethical dimensions of digital health, for example, risks being both impractical and unjust. For instance, the Ugandan Ministry of Health has benefitted from laissez-faire consultation with civil society, practitioners and academia, both local and international.

The inclusion of international actors, non-profits and academic institutions was seen as another key factor, enriching the regulatory process by providing external perspectives and technical expertise. This was particularly the case for Kenya, which stakeholders felt benefitted from the expertise of overseas public health institutions. However, some stakeholders noted that the role of international actors warrants scrutiny. While their involvement can bring valuable resources and knowledge, it raises questions about sovereignty and the extent to which external influences shape domestic regulatory agendas. Kenyan stakeholders, for instance, felt they needed to own their regulatory framework and so sought to manage how they included stakeholders, engaging in laissez-faire consultation.

### Political sponsorship is an enabler of regulatory progress

Stakeholders felt that high-level political sponsorship is essential to digital health regulatory frameworks. Stakeholders highlighted the case of Kenya as an example of the transformative impact of political alignment and leadership. President Ruto’s explicit prioritization of digital health within his electoral manifesto provided a strong political mandate, propelling legislative initiatives and reinforcing the strategic importance of digital health. Stakeholders felt that this highlights how political sponsorship serves as a catalyst for regulatory progress, particularly when digital health aligns with broader national development agendas. By embedding digital health within high-level political discourse, the Kenyan government secured the necessary momentum to develop and institutionalize its Digital Health Act. Ugandan stakeholders echoed this, highlighting the difference in their experience. Stakeholders cited challenges in engaging key decision-makers, noting a significant barrier to progress due to the absence of a high-level political champion. Stakeholders noted that this was one factor influencing Uganda’s soft approach, relying mainly on senior health ministry officials to lead on digital health regulation.

Moreover, stakeholders felt timing was key, highlighting strategic events like elections or national health crises as opportunities, as was the case in Kenya with President Ruto. These moments provided entry points for advancing initiatives by linking them to pressing public concerns or political agendas. However, they cautioned that while such windows of opportunity are valuable, they must be paired with sustained advocacy and evidence-based arguments to ensure that regulatory progress is not merely episodic but integrated into a long-term vision.

### International influence

Stakeholders felt that international partnerships played a significant role in shaping the development of digital health regulatory frameworks in Kenya and Uganda. Stakeholders acknowledged the value of global standards as benchmarks for regulatory design but emphasized the necessity of adapting these standards to local socio-economic and cultural contexts, emphasizing a tension between globalization and localization in regulatory governance.

Ugandan stakeholders noted their collaboration with international experts. Uganda aimed to ensure its guidelines combined technical rigour with relevance to its unique challenges and capacities. This was achieved through leveraging their connections to academic communities that focused on digital health regulation in both Africa and Europe. Stakeholders therefore felt it important to achieve a balance between adopting international benchmarks and addressing national challenges as crucial to ensuring that regulations are not only theoretically robust but also practical within the local context.

Kenyan stakeholders cited international financial support as a key enabler of regulatory progress as it provided resources for justifying proposed legislation, building institutional capacity and sustaining long-term regulatory efforts. Stakeholders noted the requirement for legislation to be justified as financially feasible. However, stakeholders also highlighted the risks associated with financial dependencies, particularly when international partnerships fail to respect local autonomy. They felt these dependencies may create power imbalances, where donor priorities overshadow domestic needs, undermining regulatory sovereignty.

### Certainty and flexibility in regulatory tools

The trade-off between regulatory certainty and flexibility emerged as a central theme in the focus group discussions. Guidelines and standards were praised for their adaptability and ease of revision as stakeholders felt they were well-suited to the dynamic nature of digital health technologies. Stakeholders stressed that their informality allows for swift responses to technological advancements, reducing the regulatory lag often associated with formal legislative processes. However, some stakeholders noted that these tools come with inherent risks, including fragmented oversight and interpretative ambiguities, and uncertainty around enforcement. Stakeholders felt that without clear enforcement mechanisms, the effectiveness of guidelines and standards depends heavily on voluntary compliance and consistent stakeholder collaboration.

In contrast, Kenyan and Rwandan stakeholders thought acts of parliament provide a much more stable and authoritative framework with clearly defined oversight structures, like Kenya’s Digital Health Act provides. These formal legislative tools establish legal certainty, ensuring consistent application and enforcement. However, Ugandan stakeholders felt the rigidity and the resource-intensive process required for amendment made them less responsive to rapid technological changes.

Participants emphasized the importance of embedding flexibility mechanisms within legislative frameworks, such as provisions for periodic review and updates. Regular reviews, integration of new information and effective communication with stakeholders were identified as critical components of a dynamic regulatory framework. Stakeholders felt these processes ensure that regulations not only keep pace with technological change but also retain their legitimacy and enforceability.

### EXPERTISE

Stakeholders considered expertise critical, emphasizing the need to include appropriate technical, legal and ethical expertise to address the multifaceted challenges of digital health. While stakeholders felt the substance of expertise was paramount, the form and structure of expertise-based engagements were also noted as influential in achieving effective outcomes. It is noteworthy that there was significant variation in how countries structured their use of expertise. Uganda employed small, specialized committees to develop specific guidelines to enable focused and context-sensitive consultations. They also, like Kenya, drew on both domestic and international expertise, which allowed for a diverse pool of knowledge, though some stakeholders felt that this introduced coordination challenges. Rwanda centralized its expertise within a digitization directorate housed under the Ministry of Health, streamlining its governance structure and aligning digital health initiatives with broader national policies. This variation reflects different institutional capacities and strategic priorities.

Expertise was unevenly distributed across the three countries. Stakeholders noted that some countries have made significant investments in capacity building, but others face persistent resource gaps that hinder their ability to develop and implement regulatory frameworks. While stakeholders emphasized that the substance of expertise—the actual knowledge and skills brought to the table—takes precedence, they also acknowledged the significance of the form in which expertise is organized and engaged. Regular consultations, even if conducted on an ad hoc basis, were viewed as essential for keeping regulations aligned with technological advancements. Stakeholders felt that structured mechanisms, such as dedicated committees or directorates, provide continuity and institutional memory, which are critical for addressing the rapidly evolving landscape of digital health. Stakeholders advocated for a dynamic approach to expertise, incorporating regular updates, cross-disciplinary collaboration and engagement with diverse stakeholders.

### Lessons from other regulatory sectors

Insights from other regulatory sectors, particularly food and drug regulation, were identified as valuable in shaping the form of digital health regulatory frameworks. Stakeholders from Kenya felt that a phased regulatory approach that begins with flexible tools such as guidelines to address immediate needs and gradually evolves into comprehensive legislative frameworks as institutional capacity and technological maturity increase is ideal. They felt this phased approach, an iterative model of regulation, allows for continuous learning, stakeholder engagement and refinement of regulatory tools. Early-stage reliance on guidelines provides agility to respond to urgent challenges and rapid technological advancements, like in Uganda, while the eventual transition to formal legislation offers the stability and authority necessary for long-term governance. Kenyan stakeholders noted that a key factor influencing the decision to adopt legislation was the evidence they had generated from using e-Health policies to regulate digital health in the initial stages.

While lessons from sectors like food and drug regulation provide valuable structural guidance, stakeholders emphasized the unique challenges of digital health, including the rapid pace of technological evolution and the critical importance of data protection. Unlike traditional regulatory domains, digital health operates at the intersection of technology, health and data governance, requiring tailored approaches that can simultaneously manage the complexities of these overlapping spheres. Stakeholders stressed the limitations of direct analogies from other sectors. They felt regulatory solutions for digital health should address specific issues such as interoperability, data privacy and the ethical implications of AI—challenges that are less prominent in traditional regulatory sectors.

## DISCUSSION

This study provides important insights into the design choices associated with different forms of digital health regulatory frameworks, highlighting stakeholders’ perceptions on how norms, stakeholder engagement, political sponsorship, international influence and expertise shape design choices. While there are lessons to be drawn from other sectors, the findings underscore the unique challenges posed by digital health, which demand tailored, context-sensitive regulatory solutions.

The findings reveal that the institutional design of digital health regulation varies across the three countries, reflecting the interplay of national priorities, capacities and technological maturity. Kenya’s formalized legislative approach, centred on the Digital Health Act, offers a model of formal governance designed to protect sensitive health data and build public trust. What is key to note is the rationale for their decision. Stakeholders noted that they wanted a mechanism that would provide robust protection for health data and foster trust among citizens that their health data was protected. And this was legislation, which could also set up an agency to act as guardian for health data, manage health data and sue and be sued for how they provide oversight. This insight contrasts with much of the literature that focuses on balancing innovation with patient safety, raising the question as to whether that is the correct starting point or whether the starting point is how we can best protect individuals and what do we need to do that [11, 27, 33]. This is a key finding that is very relevant to institutional design and is likely context specific to digital health.

Moreover, concerns about the rigidity of this mechanism were rebutted with stakeholders emphasizing embedding flexibility mechanisms into legislation that anticipate future technological changes, as was the case in Kenya. Still, Uganda’s reliance on guidelines and standards, while it underscores the challenges of informality, including fragmentation and limited enforceability, suggests models must be context specific. Rwanda’s consideration of a mixed model, combining soft and hard regulatory tools, could also offer a promising middle ground that balances flexibility with enforceability. But again, the focus on this is likely misleading given the crucial points raised by Kenyan stakeholders about putting people first. This suggests that while countries may benefit from tailoring their normative anchors to their institutional readiness and embedding mechanisms for gradual evolution towards more formal structures, protecting individuals should be the primary focus [11, 23].

Regarding stakeholder engagement, it is apparent that the stage of engagement is the most crucial design choice, but this depends on other factors like whether there are legal requirements for stakeholder engagement and the form of that engagement. Inclusive, multidisciplinary engagement helps to ensure that regulatory frameworks address diverse needs and concerns [12, 24]. Kenya’s phased approach, informed by lessons from financial technology regulation, exemplifies how early-stage consultations can shape legislative drafting while ongoing engagement refines and evaluates frameworks during implementation. However, while the quality and inclusivity of consultations are paramount, structured mechanisms, such as Rwanda’s digitization directorate, provide continuity and institutional memory. Also, building out committees and sub-committees to ensure sustainable engagement can be useful, especially in the later stages where policymakers have enough experience with some forms of regulation and want to benefit from real-world experiences of stakeholders’ challenges. The findings underscore the need for dynamic, context-sensitive approaches to engagement that balance inclusivity with efficiency and responsiveness.

The transformative role of political sponsorship in advancing digital health regulation is evident in Kenya, where high-level support, including President Ruto’s prioritization of digital health, propelled legislative initiatives. This alignment of political will with regulatory development highlights the importance of embedding digital health within broader national development agendas to secure momentum and resources [21]. By contrast, Uganda’s limited political engagement underscores the barriers to progress in the absence of high-level sponsorship. This finding suggests that political advocacy, strategic framing and institutional readiness are critical for garnering political buy-in. Furthermore, the study highlights the importance of timing, with elections and national crises presenting windows of opportunity for advancing digital health. Sustained advocacy and evidence-based arguments are essential to ensure that these opportunities translate into durable regulatory frameworks. This confirms literature that has cited political will as a key enabler of regulatory progress. But what is key to note is the level of sponsorship that may be required and the issues associated with that. Kenyan stakeholders were confident that Presidential attention was crucial, but they also cautioned against building an episodic strategy that sought to tie everything to politics, given the tenuous nature of that approach.

The findings also reveal the complex interplay between global standards and local contexts in shaping regulatory frameworks. While international best practices and financial support provide valuable resources, their effective integration requires careful contextual adaptation to avoid undermining local autonomy. Uganda’s collaboration with international experts, for example, demonstrates how external partnerships can enhance technical rigour while remaining responsive to national challenges. However, the findings also caution against over-reliance on international actors, which risks creating power imbalances and prioritizing donor agendas over domestic needs. Balancing global expertise with local sovereignty is critical for ensuring that regulatory frameworks are both equitable and context-sensitive [21].

The trade-off between certainty and flexibility is a recurring theme in digital health governance. Guidelines and standards offer adaptability and ease of revision, which would seem to make them well-suited to rapidly evolving technologies. However, their informality risks ambiguity, fragmented oversight and weak enforcement [11, 27]. Conversely, legislation provides stability and authority, though their rigidity and resource-intensive amendment processes may make them less responsive to technological changes. Embedding flexibility mechanisms, such as provisions for periodic review, within formal legislation can help reconcile these tensions, enabling stability without stifling innovation.

The role of expertise is central to effective regulatory design. Across the three countries, diverse approaches to expertise highlight the importance of tailoring structures to institutional capacities [30]. While Uganda’s use of small, specialized committees provides targeted guidance, Rwanda’s centralization of expertise within a digitization directorate streamlines governance and aligns digital health initiatives with national priorities. The uneven distribution of expertise across the countries underscores the need for capacity building to address resource gaps. Still, the findings emphasize that while the substance of expertise takes precedence, the form in which it is organized—whether through structured committees or ad hoc consultations—affects the continuity and adaptability of regulatory frameworks. So, both form and structure should be considered when designing regulatory frameworks.

Insights from other sectors, such as food and drug regulation, provide valuable guidance for structuring digital health frameworks. The phased regulatory approach, beginning with flexible guidelines and progressing to formal legislation, offers a model of iterative governance that balances agility with authority. However, digital health’s unique challenges—rapid technological evolution, data protection and ethical considerations—necessitate tailored solutions that go beyond traditional regulatory paradigms. However, the findings illustrate the multifaceted nature of digital health governance, highlighting the need for context-sensitive, inclusive and adaptive regulatory frameworks. While lessons from other sectors and international standards provide valuable starting points, successful regulatory design requires balancing global benchmarks with local realities, certainty with flexibility and political sponsorship with stakeholder inclusion. These insights underscore the importance of iterative, multidisciplinary approaches to governance that prioritize legitimacy and responsiveness in the rapidly evolving landscape of digital health.

## Supplementary Material

supplementary_document_oqaf010

## Data Availability

The data underlying this article are available in the article and in its online supplementary material.
