# Supplementary material for: Stakeholder perceptions on institutional design of digital health regulatory frameworks: insights from Kenya, Rwanda and Uganda
Source: Oxf Open Digit Health. 2025 May 9;3:oqaf010. doi: 10.1093/oodh/oqaf010 (PMC12101727; doi:10.1093/oodh/oqaf010)
Supplement: supplementary_document_oqaf010 [file supplementary_document_oqaf010.pdf]

Supplementary Materials

The National Treasury and Economic Planning
P.O. Box 30007- 00100,
Nairobi, Kenya
Tel: +254202252299
Email: communications@treasury.go.ke
www.treasury.go.ke

BUDGET STATEMENT

FY 2023/24

Theme: "Bottom-Up Economic Transformation and Climate Change Mitigation /Adaptation for Improved Livelihoods of Kenyans"

STATEMENT DELIVERED TO THE NATIONAL ASSEMBLY ON 15TH JUNE, 2023 BY PROF. NJUGUNA NDUNG'U, CBS, CABINET SECRETARY FOR THE NATIONAL TREASURY AND ECONOMIC PLANNING, REPUBLIC OF KENYA, WHILE PRESENTING THE BUDGET POLICY HIGHLIGHTS AND REVENUE RAISING MEASURES FOR THE FINANCIAL YEAR 2023/24 BUDGET

1ST JULY, 2023 TO 30TH JUNE, 2024

© Budget Statement 2023

To obtain copies of this statement, please contact:

The National Treasury and Economic Planning

Treasury Building

P. O. Box 30007-00100

NAIROBI, KENYA

Tel: +254-20-2252-299

Fax: +254-20-341-082

The statement is available on: www.treasury.go.ke

I. INTRODUCTION

1. Mr. Speaker, it is a great honour and privilege for me to present to this august House and to the people of the Republic of Kenya, the First Budget of the Kenya Kwanza Administration under the leadership of His Excellency Honourable Dr. William Samoei Ruto, The President of the Republic of Kenya and Commander-in-Chief of the Defence Forces.

2. I do this in compliance with the Standing Order No. 241 of the National Assembly and Section 40 of the Public Finance Management Act, 2012 that requires the Cabinet Secretary for the National Treasury to make a public pronouncement of the budget policy highlights and revenue raising measures for the National Government.

3. This being the first budget following the conclusion of the General Elections held in August 2022, allow me to congratulate all the Honourable Members who were re-elected and those who are serving for the first time in this august House. I look forward to working closely with all of you as we implement the Bottom-Up Economic Transformation Agenda designed to achieve a

sustained economic recovery for improved livelihood of all Kenyans.

4. Before I proceed, **Mr. Speaker**, allow me to take this opportunity to express my deep appreciation to His Excellency, The President for appointing me as the Cabinet Secretary for the National Treasury and Economic Planning and entrusting me with the honour of overseeing the management of macroeconomic policy and the public financial management of this great Nation. I also wish to extend my sincere gratitude to His Excellency, the President for His wise counsel during the preparation of these budget policy highlights and revenue raising measures that will lay a strong foundation for our socio-economic transformation.

5. **Mr. Speaker,** the FY 2023/24 Budget has been prepared against a background of a challenging external and domestic environment. In addition to the COVID-19 Pandemic, Kenya has, over the past year, suffered a series of devastating negative and persistent shocks that include the worst drought in decades as well as the adverse spill overs from the conflict in Eastern Europe and tighter global financial market conditions. The combination of all

those negative and continuing shocks are what I describe as a perfect storm that has confronted the New Administration.

6. The extreme climate change events have emerged as key drivers of food insecurity and increased cost of living. Indeed, our people are currently confronted with increasingly higher prices of basic commodities, especially food, energy and transport. This calls for urgent and decisive interventions, especially on our supply side response.

7. **Mr. Speaker**, in fulfilment of the requirement under the Constitution on public participation, we accorded Kenyans the opportunity to share their views on issues that need to be addressed in this Budget. **Mr. Speaker,** we received from Kenyans many useful proposals and suggestions that have informed the priorities outlined in this Budget Statement. The key concerns raised by the public can be broadly clustered around five issues: **one**, the high cost of living; **two**, the high level of unemployment among the youth; **three**, high tax burden; **four,** wastage of public resources and **five,** high public debt burden.

8. **Mr. Speaker,** having listened carefully to concerns from the public and taking cognizance of the socio-economic and environmental challenges facing the country, this Budget Policy highlights seeks to provide solutions to the said concerns as we all work towards accelerating economic recovery.

9. Proactively, the Kenya Kwanza Government has begun the journey to bring down the cost of living and improve livelihoods, while at the same time fostering a sustainable inclusive economic transformation. The Government has taken the long-term sustainable approach of subsidizing production of goods instead of consumption to respond to the rising cost of living.

10. In this regard, the Government has instituted immediate interventions that are aimed at providing short-term solutions to the high cost of living while at the same time building a momentum for the long-term economic vibrancy and transformation. In particular, the Government has covered the following:

i) Through the e-voucher Fertilizer Subsidy Program, availed 6 million (50kg bags) of subsidized fertilizer worth Ksh 15

billion, retailing at a cost of Ksh 3,500 per bag, a reduction from the market price of Ksh 6,500 per bag, representing a subsidy of Ksh 3,000 per bag. So far, the Government has registered 5 million farmers nationwide and issued over 2.5 million e-vouchers to registered farmers. As a result, Kenyan farmers have been able to plant at least 200,000 additional acres of food this year and used 2 million more kilograms of seeds. This is expected to improve agricultural yields;

ii) Granted duty waiver for importation of key food products such as white maize, rice, yellow maize, soya beans, soya bean meal, assorted protein concentrate, and feed additives effective from 1st February to 6th August, 2023, in order to bridge the food stocks deficit as well as lower and stabilize food prices;

iii) As part of drought mitigation measures and the climate adaptation programme, the Government initiated a National Tree Planting Campaign that seeks to enhance our national tree cover to from 12 percent to 30 percent through a target of planting 15 billion trees by 2032. In addition, the Government has initiated new dam constructions to address

drought concerns and ensure Kenya has enough harvested water to facilitate a water management strategy for irrigation for food production;

iv) Invested in biotechnology research and uptake of drought tolerant crops to promote food security, particularly in marginalized areas. In the current year, over 250 metric tonnes of assorted drought tolerant seeds valued at Ksh 50 million have been distributed in Embu, Meru, Makueni, Machakos, Tharaka Nithi, Murang'a, Nyeri, Siaya and Busia counties;

v) Reformed the National Health Insurance Fund to meet the urgent needs of Kenyans at the bottom of the socioeconomic structure by actualizing its purpose as a social medical insurance facility. In addition, the Government has committed to deliver Universal Health Coverage to ensure that every Kenyan attains dignified healthcare at a minimal subscription fee; and

vi) Reviewed the policy in the energy sector with the aim of developing and diversifying this market further by improving sourcing and supply of cooking gas that

inevitably had resulted in high costs that also drive domestic inflation. This has allowed for additional players to be licensed to set up Liquefied Petroleum Gas (LPG) facilities. A common user bulk storage and handling facility for LPG will be constructed through a private sector participation framework to enhance market certainty and stability in the use of LPG. This will cut the cost of handling and evacuating gas from ships to the mainland hence allowing the dealers to pass through the cost reliefs to the consumers.

11. **Mr. Speaker,** in order to improve service delivery, support livelihood and businesses as well as strengthen governance institutions, the Kenya Kwanza Government has since assumption of office:

i) Established the Financial Inclusion Fund, or the Hustlers Fund in November 2022 to provide access to affordable credit to individuals and MSMEs and encourage savings. This is an intervention that will remain at the bottom of the pyramid. In order to access borrowing under the Fund, a customer only needs to register through his or her mobile

phone. Since the launch of the first product, the Hustlers Fund Personal Loan Fund, the Government has invested a total of Ksh 11.0 billion. This money has been revolving within the Fund, providing low interest loans at 8 percent per annum to 16.07 million Kenyans, of which 7.1 million are repeat customers. So far, Ksh 30.8 billion has been borrowed from this revolving fund. In addition to borrowing, the Fund is designed to encourage savings. To date, Ksh 1.5 billion has been saved as mandatory savings, while Ksh 17 million has been saved on a voluntary basis. We have witnessed a phenomenal growth in the number of transactions that currently stand at 43.5 million. The largest number of times a single customer has borrowed from the Fund is about fifty times indicating strong traction to the Fund.

ii) To further empower MSMEs, the Government on 1st June, 2023, launched the second product of the Financial Inclusion Fund, that is aimed at facilitating Kenyans access funding through groups. This follows phenomenal success of the Personal Loan product under the initial phase. This will

allow lending to groups such as chamas and saccos. The groups can now borrow loans of between Ksh 20,000 and Ksh 1.0 million;

iii) Redesigned the competence-based education curriculum to make it responsive to our education needs;

iv) Employed over 30,000 teachers in a historic and unprecedented drive to improve the national teacher - pupil ratio and enhance performance;

v) Introduced a new funding model for higher education that will make the universities and technical training fully inclusive, financially self-sufficient and capable of competing with their peers globally while contributing to our national socioeconomic transformation;

vi) Established the National Open University in order to make higher education inexpensive, affordable, accessible, inclusive and attainable to all. The University will receive its Charter before the end of June 2023;

vii) Launched several affordable housing projects across the country, translating to about 36,000 housing units in just eight months;

viii) Digitized and automated 3,570 of Government services through the e-Citizen platform that has enhanced access to Government services and brought greater convenience to citizens. The Government targets to bring onboard 5,000 services on the e-Citizen platform by the end of 2023;

ix) Launched a data protection registration system to provide digital identity to all Kenyans in order to facilitate transactions between the Government and its citizens;

x) Returned the port operations from Nairobi and Naivasha to Mombasa in order to revamp the coastal economy and give importers, especially the business community a choice on cargo clearance;

xi) Introduced a Government-to-Government arrangement for oil importation to provide a longer-term supply plan of fuel and ease the monthly demand for the US dollar in the country;

xii) Appointed four Court of Appeal judges and two judges of the Environment and Land courts, in line with the recommendation of the Judicial Service Commission, to strengthen the Judiciary. Further, the Government escalated

the actualization of the Judiciary Fund that will increase allocation of resources towards enhancing access to justice; and

xiii) Enhanced independence of the National Police Service (NPS) by granting them financial autonomy as envisaged in our constitution.

12. Mr. Speaker, while we celebrate these achievements, we are aware that a lot remains to be done in order to decisively deal with the pressing socio-economic challenges **first,** to reverse the effects of the recent past negative shocks, and **second,** improve and accelerate the betterment of the livelihoods of Kenyans. It is for this reason that, the Government is implementing the Bottom-Up Economic Transformation Agenda popularly referred to as "BETA" geared towards economic turnaround and inclusive growth. Aligned to this agenda, the theme for this year's budget is: **"Bottom-Up Economic Transformation and Climate Change Mitigation/Adaptation for Improved Livelihoods of Kenyans"**.

13. Consistent with this goal, the Government will scale up implementation of policy priorities and structural reforms under the Bottom – Up Economic Transformation Agenda as detailed in

the 2023 Budget Policy Statement and the Fourth Medium Term Plan (MTP IV) currently under development, that will guide attainment of these development objectives as well as the continuation of the Kenya Vision 2030 and the Sustainable Development Goals (SDGs).

14. The strategy will involve increasing investments in five strategic sectors that have the largest impact and linkages to the economy as well as on household welfare as the starting point. These include: Agricultural Transformation; Micro, Small and Medium Enterprise Economy; Housing and Settlement; Healthcare; and, Digital Superhighway and Creative Industry. Special focus will be placed on the interventions that: **reduce the cost of living**; **create jobs**; **achieve more equitable distribution of income**; **enhance social security**, **expand tax base for more revenues to finance development**; and **increase foreign exchange earnings**.

15. **Mr. Speaker,** we intend to implement the policy priorities within a sustainable fiscal framework. Overall, we have moderated our spending to ensure value for money and

enhanced revenue mobilization efforts to ensure that our public debt remains on a sustainable path.

16. Prioritisation is critical because resources are scarce, thus limiting our capacity and desire to cover everything at once. In this regard, the budgeting process for the priority programmes under the Bottom-Up Economic Transformation Agenda will be managed through a value chain approach. This has ensured that resources are allocated to all the components along the value chain, thereby eliminating duplication of roles, removing any funding gaps and enhancing efficient use of available budgetary resources.

17. **Mr. Speaker,** it is now my privilege and honour to present the economic and fiscal policies underpinning the Government's Bottom-Up Economic Transformation Agenda for improved livelihoods of Kenyans. Thereafter, I will broadly outline the proposed resource allocations and revenue raising measures that the Government will implement in the FY 2023/24 budget.

18. But before I proceed to elaborate on programs, let me start by highlighting the economic context in which this budget has been prepared.

II. ECONOMIC POLICY CONTEXT

Global Economy

19. **Mr. Speaker**, the FY 2023/24 Budget has been prepared against a background of increased uncertainties in the global economic outlook, reflecting continuing geopolitical tensions particularly the ongoing conflict in Eastern Europe and the pace of monetary policy tightening amidst concerns about financial sector stability in the advanced economies. As a result, global economic growth is projected to slow down to 2.8 percent in 2023 from 3.4 percent in 2022. Economic prospects are also expected to slow down across regions and countries. Nevertheless, commodity prices in the global markets, particularly of oil and food, have been easing due to improved and functioning supply chains.

Domestic Economy

20. Mr. Speaker, on the domestic scene, the economic growth for 2022 slowed down to 4.8 percent from 7.6 percent in 2021 due to the adverse impact of the multiple shocks that affected the economy. Kenya's growth in 2022 was supported by growth in the services sectors while the agricultural sector contracted for the second consecutive year due to the prolonged drought effect that also contributed to a slowdown in growth in the manufacturing as well as that of the wholesale and retail trade sectors.

21. Mr. Speaker, the economy is expected to rebound and expand by 5.5 percent in 2023 up from 4.8 percent in 2022 and maintain that pace over the medium term. This growth will be supported by a broad-based private sector led growth, including continued strong performance of the services sector and recovery in the agriculture sector due to improved weather conditions during the March – May rain season.

22. This growth outlook will be reinforced by the interventions being implemented by the Government under the Bottom-Up Economic Transformation Agenda, including provision of

subsidized fertilizer and seeds to farmers during the planting season. Further, economic activity is expected to improve and drive aggregate demand expansion as the international commodity prices ease coupled with improved exports and resilient private sector consumption as well as the ongoing public and private investment.

23. **Mr. Speaker,** the Government will continue to support economic recovery by pursuing prudent macroeconomic policies geared towards reducing debt vulnerabilities and supporting sustainable and inclusive development. Inflation rate has remained above the 7.5 percent upper bound target since June 2022, mainly due to high food and energy prices following adverse weather conditions and high global oil prices but also compounded by a pass-through effect from domestic currency depreciation. In order to anchor inflation expectations, the Central Bank of Kenya through the Monetary Policy Committee tightened monetary policy during the last one year by raising the Central Bank Rate to 9.5 percent in March 2023 from 7.5 percent in May 2022. This monetary policy action together with improved agricultural production occasioned by the long rains

and supported by the fertilizer subsidy program, and the ongoing importation of key food items particularly maize, cooking oil, rice and sugar under the duty-free window are expected to ease the domestic prices of basic food items.

24. Already, inflationary pressures are easing with inflation rate at 8.0 percent in May 2023 from the high of 9.6 percent in October 2022. Inflation rate is expected to return to the target range within the second quarter of the FY 2023/24. Credit to the private sector grew by 13.2 percent in the 12 months to April 2023, supporting economic activity. Most sectors of the economy continue to receive positive credit flows.

25. Mr. Speaker, to further anchor inflation expectations, the Central Bank of Kenya will continue to implement the reforms outlined in the White Paper on Modernization of the Monetary Policy Framework and Operations that are aimed at enhancing the effectiveness of monetary policy formulation and implementation to prevent monetary policy falling behind the curve. These reforms include refining macroeconomic forecasting frameworks in line with the changing structure of the economy,

improving the functioning of the interbank market to strengthen monetary policy transmission and continued improvement of communication on monetary policy decisions.

26. **Mr. Speaker,** the fiscal policy stance over the medium term aims at supporting the Government's Bottom-Up Economic Transformation Agenda through a growth friendly fiscal consolidation plan designed to slow down the annual growth in public debt and implement an effective liability management strategy, without compromising service delivery to citizens. This will improve the country's debt sustainability position.

27. **Mr. Speaker,** I now expound on the policy priorities of the Government for fostering economic transformation and improved livelihoods.

III. POLICY PRIORITIES AND STRUCTURAL REFORMS FOR IMPROVED LIVELIHOODS

Bottom – Up Economic Transformation Agenda

28. **Mr. Speaker,** this year's budget places special emphasis on priorities under the Bottom-Up Economic Transformation Agenda which is detailed in the 2023 Budget Policy Statement

that was approved by this House in March 2023. This Agenda is premised on the need to address the challenges that the economy is facing, stimulate economic recovery, bolster resilience while building on successes realized over time.

29. The strategy will involve increasing investments in at least five sectors envisaged to have the largest impact and linkages to the economy as well as on household welfare. The priority programmes are classified under two categories, namely, the core pillars and the enablers that aim at the creation of a conducive business environment for socio-economic transformation. **Mr. Speaker**, as indicated earlier in this Statement, these core pillars include: Agricultural Transformation; Micro, Small and Medium Enterprise (MSME) Economy; Housing and Settlement; Healthcare; and Digital Superhighway and Creative Industry.

30. In order to make these programmes feasible, the Government will implement strategic interventions under the following key enablers: Infrastructure; Manufacturing; Blue Economy; the Services Economy, Environment and Climate Change; Education and Training; Women Agenda; Youth

Empowerment and Development Agenda; Social Protection; Sports, Culture and Arts; and Governance.

31. **Mr. Speaker,** in order to realize the aspirations of the Bottom-Up Economic Transformation Agenda, the Government will:

i) Continue to maintain macroeconomic stability and enhance security to foster a secure and conducive business environment for all Kenyans and their investments;
ii) Intensify national infrastructure development and connectivity in roads, rail, port, energy and fibre optic infrastructure to lower the cost of movement of people and goods, lower the cost of doing business thereby enhancing profitability of businesses;
iii) Enhance investment in key economic sectors for broad based sustainable economic recovery by promoting: agricultural transformation, growth in manufacturing, environmental conservation and water supply, food security, climate change mitigation and adaptation, tourism recovery, and sustainable land use and management. Food security and

climate change will also become a focal point for policy going forward;

iv) Expand access to quality social services in health, education and appropriate social safety nets for the vulnerable population; and

v) Support the youth, women and persons living with disability through Government-funded empowerment programs that leverage on partnerships with private sector organizations.

32. Mr. Speaker, the Government under the support of Development Partners is implementing a program that aims at boosting revenue mobilization and reduce total expenditure as a percentage of GDP thereby providing a growth friendly fiscal consolidation plan designed to slow down the annual growth in public debt and implement an effective liability management strategy, without compromising service delivery to citizens. This fiscal consolidation plan targets to gradually reduce fiscal deficit including grants from 6.2 percent of GDP in the FY 2021/22 to 5.8 percent of GDP in the FY 2022/23 to 4.4 percent of GDP in the FY 2023/24 and further to 3.6 percent in the FY 2025/26.

33. Mr. Speaker, we are truly grateful as a Nation to our Development Partners who have over the years provided financial resources to support implementation of Government programmes, policy and structural reforms. In particular, allow me to single out the multilateral institutions, specifically the World Bank, the International Monetary Fund (IMF), the European Union, the African Development Bank, and the many bilateral donors, institutions and Governments that have walked the journey of socio- economic transformation with Kenya.

34. Mr. Speaker, Kenya has continued to successfully implement the Extended Fund Facility and the Extended Credit Facility (EFF/ECF) arrangements supported by the IMF. We concluded the 5th Review of the Program in May 2023, and agreed on: **an augmentation of access** under the program of 75 percent of quota (SDR 407.1 million, about US$ 544.3 million); **an extension of the duration** of the EFF/ECF arrangements by 10 months to April 2025 to allow sufficient time for meeting the program objectives; and **on a new 20-month Resilience and Sustainability Facility** (RSF) arrangement with access of 75 percent of quota that will run in parallel with the EFF/ECF arrangements until April 2025.

35. Once approved by the IMF Executive Board in July 2023, Kenya will immediately access **SDR 306.7 million (about US$ 410 million),** including from the augmentation of access under the ECF/EFF. This will bring the cumulative IMF financial support disbursed under the EFF and ECF arrangements to **SDR 1,509 million (about US$ 2,017 million)**.

36. The Government will also access an additional SDR 407.1 million, about US$ 544.3 million under the new 20-month Resilience and Sustainability Facility (RSF) arrangement upon successful next review. **Mr. Speaker,** when combined with the RSF support, the cumulative IMF commitment to Kenya will amount to **SDR 2.633 billion (about US$3.52 billion)** by April 2025.

37. Mr. Speaker, as a stop gap measure to ease pressure on the domestic supply of petroleum products as well as ease pressure on the Kenya Shilling arising due to the global shortage of US dollar in the domestic market, we have introduced an innovative new Government-to-Government arrangement for fuel importation through Legal Notice No. 3 of 2023 that allows for

importation of petroleum products by the private sector through the current Open Tender System (OTS).

38. To date, the monthly importation of petroleum products is approximately seven hundred and forty thousand (740,000) Metric Tons of which 60 percent is consumed in the domestic market while 40 percent is for transit market in the region. This product is currently paid for using United States (US) dollars, which puts a strain on foreign exchange availability and pressure on nominal exchange rate and ensuing exchange rate volatility in the market on a monthly basis.

39. **Mr. Speaker,** to contain the pressure of US dollar availability in the market as well as the exchange rate volatility on a monthly basis, the Government entered into a Memorandum of Understanding with Governments of oil producing countries under which Framework Agreements have been signed with petroleum exporting companies for the supply of petroleum products on extended credit period of one hundred and eighty (180) days. The objective of this arrangement is: **first,** to ease the monthly demand for US dollars in the foreign exchange market

that is driven by petroleum imports by extending time required to source for US dollars from the current five (5) days to one hundred and eighty days; **Second,** to allow space to activate the interbank foreign exchange market and thus stem currency speculation; **Third,** reduce the cost of petroleum products by leveraging on economies of scale that arise from having longer supply contracts of up to 270 days with potential suppliers; **Fourth,** avert supply disruption by safeguarding a longer-term supply of petroleum products; and, **finally,** allow a restructuring of the fuel pricing mechanism that will be dependent on market conditions. Currently, the pricing mechanism coordinates expectations of price revisions on the 15th of every month, a situation that does not reflect appropriate market conduct as well as encourage diverse market development paths in this sub-sector.

Climate Change Mitigation and Adaptation

40. Mr. Speaker, climate change has become a pressing issue globally, and like other economies, the Kenyan economy is vulnerable to its ravaging impacts. Indeed, we have just come out of the worst drought in 40 years which has claimed lives of people

and livestock, while at the same time threatened livelihood of millions of Kenyans.

41. Given our vulnerability to climate change, it is imperative that we build a more climate resilient economy. For this reason, the Government has stepped up climate adaptation and mitigation efforts including green energy, smart agriculture, de-carbonized manufacturing, e-mobility and green building, all aimed at the attainment of zero carbon by 2050.

42. The Government has committed to secure our future and to achieve a cleaner and safer environment for all Kenyans by reducing greenhouse gas emissions, halting and reversing deforestation, biodiversity loss and land degradation. In this regard, issues of climate change mitigation and adaptation have been mainstreamed in all Government programmes and across all levels of Government.

43. As part of drought mitigation measures and the climate adaptation programme, the Government is implementing a National Tree Planting Campaign which is championed by His Excellency the President and seeks to enhance our national tree

cover from 12 percent to 30 percent by 2032. This will entail planting at least 15 billion trees over the period.

44. Mr. Speaker, climate financing has emerged as an important means of implementation for climate change and for promoting sustainable development and financial sector development. To enhance financial flows from the Green Climate Fund (GCF), the Government will continue to implement the National Green Climate Fund Strategy, which provides an elaborate framework of coordinating and attracting resources from the Green Climate Fund. The Government will also implement the Financing Locally-led Climate Action (FLLoCA) Program in collaboration with County Governments and development partners to manage climate risks.

45. Further, to support our climate change efforts and catalyse additional climate finance from other official and private partners, we have agreed with the IMF on a new 20-month Resilience and Sustainability Facility (RSF) arrangement that will run in parallel with the current Program arrangements until April 2025.

46. Mr. Speaker, the Government last year committed to fast-track the enactment of the Disaster Risk Management Bill; and finalize the Public Finance Management (Disaster Risk Management Fund) Regulations. These are meant to strengthen disaster risk management in the country given that Kenya is predisposed to various disasters, some of which are exacerbated by the ravaging effects of climate change. These disasters often have a huge negative impact on the economy.

47. I wish to report that the relevant Departmental Committee of this House is in the process of considering a legislation on matters relating to Disaster Risk Management. I urge this House to prioritize this matter to enable Kenya have an overarching framework on Disaster Risk Management. I will later be submitting to this House for consideration the Public Finance Management (Disaster Risk Management Fund) Regulations, 2023 to enable establishment of the Disaster Risk Management Fund.

Policy, Legal and Institutional Reforms

48. Mr. Speaker, the Government's Bottom – Up Economic Transformation Agenda (BETA) will be underpinned by sound and appropriate policy, legal and institutional reforms, aimed at improving the business environment, increasing efficiency in public service delivery and strengthening accountability and transparency in public finance management. These reforms are outlined as follows:

Procurement Reforms

49. Mr. Speaker, on a continuous basis, the Government reviews the procurement policies and procedures to ensure that we achieve efficiency and transparency, enhance good governance and promote savings in the procurement process. This is one critical area that the Government can achieve efficiency and unlock savings given the limited financial resources and fiscal space.

50. With this in mind, **Mr. Speaker,** last year, the National Treasury committed to this House to roll out an end-to-end e-Government Procurement system (e-GP) with a pilot phase

commencing in July 2022 and a target date for full roll-out to all Ministries, Departments and Agencies in January 2023. I wish to report to this House that the process is still in progress albeit with some delays. The system will be rolled out by December 2023 to both the National and County Governments. Once rolled out, all public procurement and asset disposal processes will be managed online through the system. The system will enhance efficiency and transparency in the procurement functions. The system will also ease cost of doing business with the Government and has capabilities of monitoring and tracking of all public procurement and asset disposal.

51. Mr. Speaker, the Government remains committed to facilitate access to Government procurement opportunities and empower MSMEs owned by Women, Youth and Persons with Disabilities. This remains a key priority of the Government under the Bottom-Up Economic Transformation Agenda. For this reason, the National Treasury has reengineered the Access to Government Procurement Opportunities (AGPO) portal to enable real-time registration and monitoring. The National Treasury has also directed all procuring entities to ensure prompt

payment of all contracts successfully implemented under the AGPO to enable businesses owned by the target groups access liquidity promptly.

State Corporations Reforms

52. **Mr. Speaker,** a large number of State Corporations rely on the exchequer funding to service their payroll and other expenses. Over the years, bloated payrolls have remained a large burden to the exchequer resulting in cumulated un-serviced statutory obligations and pension liabilities running into billions of Kenya Shillings. It has also been established that in most State Corporations, a huge proportion of the staff compliment comprises of support staff with a thin compliment of technical staff, gravely affects core service delivery. To address this challenge, the State Corporations Advisory Committee (SCAC) will immediately embark on an aggressive programme to rationalize staff establishment to keep them lean and strictly adhere to the **70:30 ratio** of Technical to Support staff. In addition, the National Treasury shall hence forth only approve budgets for hiring staff for only State Corporations that have received approvals from State Corporations Advisory Committee,

the Public Service Commission (PSC) and Salary and Remuneration Commission (SRC). This requirement is in addition to confirmation of the requisite funding.

53. Mr. Speaker, to support restructuring of State Corporations, the Cabinet Approved the Privatization Bill, 2023, that seeks to repeal the Privatization Act, 2005, and streamline privatization processes and to in order to restructure State Owned Enterprises (SOEs). This was subsequently submitted to this House by the National Treasury in May 2023. The Bill, once enacted, will enable the selected State Owned Enterprises to achieve their full potential by entrenching commercial principles and reduce their reliance on exchequer funding. I urge the Honourable Members to support the Government in this endeavor.

54. Mr. Speaker, in the FY 2022/23, the Government initiated a number of reforms in Kenya Power and Lighting Company (KPLC) and Kenya Airways aimed at improving their efficiencies, reducing costs and increasing revenue. To further enhance KPLC's financial sustainability, the Government will restructure its balance sheet, mainly focusing on the huge loan balances,

payables and receivables. This will in turn address the current huge liquidity gap at KPLC. Towards this end, the Government will implement a four-point action plan focusing on:

i) Transfer of all transmission assets/lines to Kenya Electricity Transmission Company (KETRACO);

ii) Settle the outstanding Rural Electrification Schemes (RES) operations and maintenance cost deficit of Ksh 19.4 billion as of June 2022 and ensure that KPLC and Rural Electrification and Renewable Energy Corporation (REREC) enter into a commercial contract for the future Rural Electrification Schemes maintenance cost;

iii) Develop and implement a turnaround strategy that includes the reduction of system losses from the current 22.4 percent to 14.4 percent by end-June 2025; and

iv) Establish a new governance structure at KPLC to give private shareholders fair representation, reflecting the company's shareholding structure.

55. With regard to Kenya Airways, the Government's policy stance is to turnaround the Airline and position is as a Pan-African

carrier, ensure it is run with profitability and sustainability objectives eventually reducing the Airline's dependency on budgetary support.

Pending Bills for the National and County Governments

56. Mr. Speaker, the total outstanding National Government pending bills as at 31st March, 2023 amounted to Ksh 537.2 billion comprising of Ksh 450.2 billion for State Corporations, Semi-Autonomous Government Agencies and Ksh 79.3 billion for Ministries, Departments and Agencies. During the same reporting period, the County Governments reported pending bills of Ksh 159.7 billion.

57. The management of the pending bills challenge, in the public eye, will form an important benchmark upon which the overall integrity of the Government's style of financial management will be tested and judged. The delay in settling payments of pending bills has led to deterioration of financial positions of businesses in particular Micro, Small and Medium Enterprises, including businesses owned by Women, Youth and Persons with disabilities.

58. In view of the above, the National Treasury has prepared a Cabinet Memorandum requesting the Cabinet to approve the establishment of a Pending Bills Verification Committee to carry out a thorough analysis of the stock of all pending bills and advise on how the bills will be settled. Once the outstanding pending bills are cleared by the verification Committee, the National Treasury will direct all Entities to ensure strict adherence to Public Finance Management Act, 2012 and clear pending bills as a first charge on the budget of the concerned Entity in the subsequent financial year.

Financial Sector Stability and Development

59. Mr. Speaker, the banking sector remained stable and resilient in 2022. Deposits held by the sector increased by 12.3 percent while pre-tax profits increased by 22.0 percent. On the other hand, gross non-performing loans for the sector stood at 13.3 percent in December 2022, a decline from 14.7 percent in June 2022. The need to build resilience and to exploit emerging opportunities has led to increased consolidations as well as regional expansion through acquisitions. This has led to strong institutions supporting the stability of the banking sector. The

sector is therefore, expected to remain sound and stable in 2023, building on the reform initiatives and buffers built over the recent years.

60. **Mr. Speaker,** in order to address consumer protection concerns arising from unregulated digital lenders, the Central Bank of Kenya introduced the Digital Credit Providers Regulations of 2022 that were approved in May 2022. They provide regulations and guidelines for the licensing and oversight of previously unregulated Digital Credit Providers (DCPs). I am happy to report that by the end of March 2023, 32 Digital Credit Providers had been licensed while others are at different approval stages. More importantly, CBK is working with other Agencies and Regulators including the Office of the Data Protection Commissioner to ensure all Digital Credit Providers are brought into the regulatory perimeter to protect consumers.

61. **Mr. Speaker**, in an effort to transform Kenya's financial markets, the Central Bank will soon launch the upgraded Central Securities Depository code named ***"DhowCSD"***. The upgraded Central Securities Depository will improve financial market

liquidity, enhance operational efficiency in the domestic debt market, further promoting market deepening, broadening financial inclusion through expansion of digital access and position Kenya as the preferred financial hub in the region. The Central Securities Depository will also scale up services to the public, market participants and support our Diaspora investors via a versatile and highly scalable digital solution with capacity to deliver seamless investor experience and convenience.

62. **Mr. Speaker,** since the launch of the National Payments Strategy 2022 – 2025 in February 2022, the Central Bank has implemented major initiatives to improve quality, access, and affordability of payment services to Kenyans. These include implementation of mobile money interoperability, licensing of new payment service providers, and rollout of key standards that increase convenience of payment services in line with global best practice. Building on these gains, in the FY 2023/24, the Central Bank together with other stakeholders will:

i) undertake a comprehensive review of the National Payment System Act, 2011 and National Payment System Regulations,

2014, to modernize Kenya's payments legal and regulatory framework in line with emerging trends, innovations and enhance oversight for improved service delivery to Kenyans; and

ii) develop and implement an interoperable payments platform to unlock cost-effective, real-time, and retail payments across the banks, payment service providers, card schemes and other regulated financial institutions. This will further contribute to lowering the cost of these services, increase choice, competition, stability, and widespread adoption of digital payments among Kenyans.

63. Mr. Speaker, in order to reduce money laundering vulnerability, the Central Bank has reviewed and enhanced the banking sector Know Your Customer (KYC) and customer due diligence (CDD) processes. This will further strengthen the effectiveness of the banking sector anti-money laundering and combating the financing of terrorism and proliferation financing (AML/CFT/PF) risk based supervisory framework.

Deposit Insurance Reforms

64. **Mr. Speaker,** financial stability risks have increased rapidly as the resilience of the global financial system has been tested by high inflation and rising interest rates. In order to enhance protection of depositors, the Kenya Deposit Insurance Corporation is in the process of reviewing the current coverage limit of Ksh 500,000 with a view to ascertain its adequacy in protecting the Micro, Small and Medium Enterprises.

65. In addition, the Corporation has developed an Alternative Dispute Resolution (ADR) framework to address disputes between financial institutions that have been closed and their respective stakeholders, thereby fast-tracking release of available resources and winding up those banks under liquidation.

Capital Markets Developments

66. **Mr. Speaker,** the capital markets remain a critical catalyst in mobilization of savings and investments towards productive enterprise in Kenya. In order to support Micro, Small and Medium Enterprises (MSMEs) access financing via the capital markets, the Capital Markets Authority has developed the Capital

Markets (Public Offers and Disclosures) Regulations, 2023 that will enable MSMEs raise debt and equity capital via the Nairobi Securities Exchange. The Authority is also implementing the Capital Markets (Investment Based Crowdfunding) Regulations, 2022 to support Kenyan Start-ups to raise finance from both global and local investors.

67. Mr. Speaker, as part of these initiatives to support the real estate sector, the LAPTRUST IMARA Real Estate Investment Trusts (REIT) has been listed in the Nairobi Securities Exchange following the approval by the Capital Markets Authority. This will provide investors with a unique opportunity to invest in a diversified portfolio of income-generating real estate assets.

Pension Reforms

68. Mr. Speaker, pension coverage in Kenya currently stands at 22 percent of the working population leaving majority of Kenyans without a pension cover. This underscores the need to extend pension coverage to the informal sector, which constitutes the majority of our workforce. To this end, **Mr. Speaker,** the Government is working on a National Pensions Policy to address

key concerns on coverage, portability of schemes, reciprocal arrangements to address concerns of Kenyans in the diaspora and mitigate the fragmentation in the sector. In furtherance of the ideals under the Bottom-Up Economic Transformation Agenda, the National Treasury has established an innovative scheme – the Kenya National Entrepreneurs Saving Trust (KNEST) with a focus on the population in the informal segment of our economy. In order to promote uptake of the scheme, we will deliberately form strategic partnerships to facilitate voluntary pension contributions for individuals in this sector and continue to explore options to provide diverse pension solutions for self-employed individuals.

69. Mr. Speaker, to effectively administer the public service pensions, the National Treasury will invest in modern technology and digital solutions to streamline pension processes and improve service delivery. In this regard, Public Service Schemes will develop user-friendly online platforms that allow pensioners to access their pension statements, make inquiries, and update their personal information conveniently. The Government continues to implement the Public Service Super annuation Scheme with the

end goal of easing the burden of pension payment by Government.

Insurance Reforms

70. Mr. Speaker, in the past two years, the insurance sector has grown by Ksh 36 billion from a premium of Ksh 273 billion. The industry has also attracted investments from key multinational players. In order to further enhance growth of the sector; the Insurance Regulatory Authority has rolled out a Micro-insurance Framework that will contribute to increased penetration coverage for individuals with low income. The Authority will strengthen private insurance's role in the Universal Health Coverage, promote crop and livestock insurance and support insurance for MSMEs.

71. Mr. Speaker, there are no deterrent measures under the Insurance Act to motivate accountability and observance of directors' duties as well as senior managers' professional responsibilities. To address this short coming, I have submitted to this House Insurance (Amendment) Bill, 2023, to provide for offences relating to the management of an insurer. In addition,

the Bill contains other amendments aimed at enhancing the efficiency of the Insurance Regulatory Authority in regulating the insurance industry.

SACCO Societies Reforms

72. Mr. Speaker, the Sacco industry is characterized by many small and medium size SACCOs that find it difficult to effectively compete in the deposit taking and credit market. To address this challenge, the Sacco Societies Regulatory Authority (SASRA) is working on amendments to the Sacco Societies Act, 2008, that will provide for licensing and supervision of Shared Sacco Services platform. This initiative will improve efficiency and competitiveness of SACCOs through establishment of cost-sharing digital platform to enable small SACCOs achieve economies of scale while at the same time mainstreaming regulatory compliance in a cost-effective manner.

73. Mr. Speaker, 12 years ago, the Government introduced prudential regulations to secure the financial stability of SACCOs and boost savings for members. However, there has been lack of a resolution mechanism for financially distressed SACCOs. In

order to address this challenge, the SACCO Societies Regulatory Authority has proposed amendments of the SACCO Societies Act, 2008 to provide for a framework for appointment of trustees to the Deposit Guarantee Fund (DGF) for SACCOs in Kenya.

Unclaimed Financial Assets

74. Mr. Speaker, the Unclaimed Financial Assets Authority Act does not provide for claimants to designate a beneficiary of their assets or to a cause of their choice and therefore any claim must be paid to the claimant. To address this, I have proposed to amend the Unclaimed Financial Assets Authority Act so as to empower claimants to designate beneficiaries of their unclaimed assets or to a cause of their choice.

Ease Cost of Doing Business and Consumer Protection

75. Mr. Speaker, in order to ease the cost of doing business and minimise compliance costs for the Micro, Small and Medium Enterprise (MSME), the Competition Authority of Kenya will:

i) Exempt MSEs sector from merger notifications thus, enabling start-ups, digital businesses, among others. Further, the Authority will monitor and conduct surveillance audits

specifically in the manufacturing and agro-processing sectors, so as to protect MSMEs from incidences of abuse of buyer power;

ii) Implement codes of practice to ensure that MSMEs in the retail and insurance sectors are protected from powerful buyers;

iii) Screen and investigate infractions such as suspected cartels or abuse of dominance conducts such as excessive pricing, price discrimination, predatory pricing and margin squeeze to ensure a level playing field; and

iv) Actualize the initiative earlier started with the National Assembly to address the issue of price fixing by professional services to make the fees competitive and improve quality of services.

76. On consumer protection, the Authority will monitor and investigate emerging consumer issues on the utility sector, pharmaceuticals, digital financial services, e-commerce, insurance, aviation and safety of basic commodities. In addition, the Authority will seek redress through replacement or refunds to

consumers affected by dark commercial patterns, scams and fraud under the digital economy.

IV. FISCAL FRAMEWORK

77. **Mr. Speaker,** the Government fiscal policy for the **FY 2023/24** and the medium-term budget aims at undertaking a growth friendly fiscal consolidation plan to ensure debt sustainability. This will be achieved through improved revenue collection, primarily through broadening of the tax base, and containing overall expenditure to the target set, while prioritizing high impact social and investment expenditure.

78. **Mr. Speaker,** the fiscal policy targets to grow total revenues to **17.9 percent of GDP** in the FY 2023/24 and **above 18.3 percent of GDP** over the medium term. As part of the economic turnaround plan, the Government will scale up revenue collection efforts by the Kenya Revenue Authority (KRA) to **Ksh 4.0 trillion** over the medium term. In order to achieve this, the Government is undertaking a combination of both tax administrative measures and tax policy reforms.

79. Mr. Speaker, regarding tax policy, the Government will implement the National Tax Policy and finalize the Medium-Term Revenue Strategy (MTRS) for the period FY 2023/24 - 2026/27. The National Tax Policy, which is before this House, will enhance administrative efficiency of the tax system, provide consistency and certainty in tax legislations and management of tax expenditure. The Medium-Term Revenue Strategy comprises legal, administrative and policy measures to reform the tax system which is expected to boost tax revenues over the medium term.

80. On tax administration, the Kenya Revenue Authority (KRA) is implementing reforms to move towards seamless taxation by ensuring that its systems, taxpayers and businesses are integrated to allow for the automatic movement of data through machine-to-machine based processes, and where appropriate shift to real-time processing. This will enable KRA to monitor business transactions in real-time therefore sealing revenue leakages through tax avoidance and evasion.

81. On expenditure reforms, **Mr. Speaker**, the Government will sustain efforts to improve efficiency in public spending and ensure

value for money by: eliminating non priority expenditures; retiring expensive and unsustainable consumption subsidies; reducing tax exemptions; scaling up the use of Public Private Partnerships financing for commercially viable projects; and rolling out an end-to-end e-procurement system.

82. Mr. Speaker, to address the challenges of perennial low absorption of development partners funds that have denied Kenyans the full benefit of this financing, the Government will put in place policies and administrative measures to minimize payment of unnecessary fees and charges.

Revenue Projections

83. Mr. Speaker, supported by tax policy reforms and administration measures highlighted above, we project total revenue collection, including appropriation-in-aid and grants for the FY 2023/24 budget to be **Ksh 2.96 trillion,** equivalent to **18.2 percent** of GDP. Of this, ordinary revenue is projected at **Ksh 2.57 trillion** equivalent to **15.8 percent** of **GDP,** Ministerial Appropriation-In-Aid is projected at **Ksh 348.7 billion** and grants are projected at **Ksh 42.2 billion**.

Expenditure Projections

84. On the other hand, **Mr. Speaker,** total expenditure in the FY 2023/24 budget is projected at **Ksh 3.7 trillion** equivalent to **22.6 percent** of **GDP**. Of this, recurrent expenditures will amount to **Ksh 2.53 trillion** equivalent to **15.6 percent** of **GDP.** Development expenditures, including allocations to domestic and foreign financed projects, Contingency Fund, Equalization Fund and conditional capital transfers to County Governments is **Ksh 743.5 billion** equivalent to **4.7 percent** of **GDP**. Equitable share to County Governments is projected at **Ksh 385.4 billion.**

Fiscal Balance

85. **Mr. Speaker**, given the projected revenue and grants against the projected expenditure, the fiscal deficit is projected to decline to **Ksh 718.0 billion** equivalent to **4.4 percent** of GDP in the FY 2023/24 from the projected **Ksh 840.9 billion** equivalent to **5.8 percent** of GDP in the FY 2022/23. The fiscal deficit will be financed through net external financing of **Ksh 131.5 billion** equivalent to **0.8 percent** of GDP and net domestic financing of **Ksh 586.5 billion** equivalent to **3.6 percent** of GDP.

86. Mr. Speaker, our medium-term fiscal consolidation policy targets to progressively reduce the level of fiscal deficit from the expected **5.8 percent** of GDP in the **FY 2022/23** to **3.6 percent** of GDP in the **FY 2026/27**.

Public Debt Management

87. Mr. Speaker, Kenya`s public debt remains sustainable but with elevated risks of debt distress due to persistent global shocks that adversely affected liquidity ratios. The depreciation of the Kenya Shilling against major currencies and rise in interest rates have elevated cost of debt service. Further, the depreciation of currency has increased the size of public debt stock as half of the public debt is denominated in foreign currency.

88. Although the debt burden has risen, Kenya has not accumulated debt service arrears and the Government is committed to honour all public debt obligations as they fall due. Over the medium term, the revenue driven fiscal consolidation policy stance is expected to improve further the country's debt sustainability ratios and debt carrying capacity.

89. Mr. Speaker, the current limit of public debt as approved in 2020 by this House, is Ksh 10 trillion. Guided by best practice, the Government intends to change the public debt ceiling from a numerical number to a Debt Anchor in form of a ratio of public debt to GDP in present value terms. The proposed change to the public debt ceiling provides an appropriate guide for optimal level of public debt based on the country's ability to pay. This is the debt carrying capacity that can be objectively assessed as well as provide directions on information on debt accumulation or reduction from time to time.

90. In order to anchor the debt ceiling in a statute, the National Treasury developed Public Finance Management (Amendment) Bill, 2023, which has been submitted to this august House for enactment. The Bill was subjected to public participation in line with the requirements of the Constitution of Kenya on transparency and accountability in public policy formulation. I urge the Honourable Members to prioritize and consider this Bill favourably.

91. Mr. Speaker, the external debt service is projected to increase to Ksh 475.6 billion in the FY 2023/24 from Ksh 242.1 billion in the FY 2022/23, mainly reflecting redemption of the US dollars 2.0 billion Eurobond (approximately Ksh 241.8 billion). In preparation for the redemption of this maturing bond, The National Treasury and Economic Planning issued an expression of interest to bring onboard a Lead Manager to advise the Government on liability management options towards the resolution of the Eurobond 2024.

92. Mr. Speaker, I wish to assure Kenyans and investors that our fiscal position remains strong and the Government remains committed to meet all maturing obligations, as and when they fall due, including the maturing Eurobond.

Public Private Partnerships Framework

93. Mr. Speaker, due to the limited fiscal space, the Government has strengthened the Public Private Partnerships (PPP) framework to leverage on private sector expertise to deliver projects that have strong economic, commercial and environmental benefits;

aligned to our national priorities with sustainability and climate change as key considerations.

94. To ensure the sustainability of PPP projects, the Project Facilitation Fund (PFF) has been operationalised. The Fund will support project preparation, provide viability gap funding to projects as well as a source of liquidity to meet fiscal commitments and contingent liabilities that may arise from PPP projects.

95. **Mr. Speaker**, with the Fund in place and a robust legal and regulatory regime, the private sector has the necessary confidence and security to invest in PPP projects. Indeed, in the last financial year, the National Treasury successfully mobilized Ksh 15 billion by achieving financial closure of two clusters of urban roads projects under the Roads Annuity Programme. The projects are currently in the construction phase. Additional projects that have closed, include two renewable energy projects with a combined installed capacity of 70 MW.

96. Further, to contribute towards the Government's agenda on bulk water supply and food security, several high impact projects have been appraised and approved to proceed to the project

development phase under the PPP model. These projects will provide production of an additional 51,000 hectares to further strengthen the food security agenda, which is a high priority for the Kenya Kwanza administration. The bulk water will increase potable water supply by an estimated 200,000 cubic metres per day. The National Treasury and the Ministry of Water and Sanitation have developed a Water Purchase Agreement which will pave way for the private sector to develop projects in a sustainable way.

97. The National Treasury is in the process of developing a 10-year infrastructure plan that will be cross-cutting across various Ministries, to support delivery and prioritisation of projects.

98. **Mr. Speaker,** to build on this, the National Treasury plans to mobilise an estimated Ksh 100 billion in private sector capital through various priority sectors. The focus will be on priority investments and underlying key projects and programmes in each sector that deliver nationally significant infrastructure, drive growth and unlock private investment with the greatest potential benefit to the Kenyan people. These sectors will include airports,

seaports, blue economy, water, agriculture, industrialization (SEZ), energy including transmission lines and digitalization among other key sectors in the economy.

99. Inclusion of local goods, works and services will be emphasized for each project, to contribute to overall economic growth by encouraging local investments in PPP projects. To also strengthen local investments, the National Treasury will be holding a series of round tables to build local capacity in the financial sector so as to increase local financing of PPP projects.

100. The National Treasury has embraced climate resilience and adaptation framework for all Public Private Partnerships projects. This is to ensure that infrastructure projects are aligned to the realities of climate change.

101. To achieve on the PPP mandate of scaling up private sector capital, the National Treasury will leverage on the continued support from Development Partners as well as other key institutions both in the public and private sector including capital markets, pension funds and commercial banks in scaling up private sector capital under the PPP framework.

Public Investment Management

102. Mr. Speaker, in 2022, this House approved the Public Investment Management (PIM) Regulations, 2022. The objective of the Regulations is to provide the National and County Governments and their entities with a standardized approach in project cycle management to enhance transparency, accountability, prudent use of public resources and public participation.

103. The Government will continue implementing the Regulations to enhance efficiency in identification and implementation of priority social and economic investment projects. This will further curtail runaway project costs, eliminate duplications and improve working synergy among implementation actors for timely delivery of development projects.

104. In the FY 2023/24, the National Treasury will develop and roll-out the Public Investment Management Information System (PIMIS) that automates the Public Investment Management process as outlined in the Regulations to ensure that only projects

with the highest social - economic returns are undertaken and prioritized for completion.

V. RESOURCE ALLOCATIONS UNDER THE BOTTOM-UP ECONOMIC TRANSFORMATION AGENDA

105. Mr. Speaker, let me now turn to the highlights of budget allocations in the coming financial year. In recognition of the need to restrain borrowing and utilize the revenues and grants projected in the Fiscal Framework, the proposed **total programmed expenditures spending** for the **FY 2023/24** has been capped at **Ksh 3.68 trillion.**

Bottom – Up Economic Transformation Agenda

106. Mr. Speaker, as I mentioned earlier, the Government will be implementing strategic priorities under the Bottom-Up Economic Transformation Agenda that is geared towards economic turnaround and inclusive growth. The Agenda focuses on five sectors envisaged to have the largest impact and linkages to the economy as well as on household welfare. These include:

i) Agricultural Transformation;

ii) Micro, Small and Medium Enterprise Economy;

iii) Housing and Settlement;

iv) Healthcare; and

v) Digital Superhighway and Creative Industry.

107. Mr. Speaker, to ensure effectiveness and efficiency in the planned investments in the five sectors, Kenya Kwanza Administration has adopted the value chain approach to budgeting. In this regard, the Government has identified nine key value chain areas for implementation, namely: Leather, Cotton, Dairy, Edible Oils, Tea, Rice, Blue economy, Natural Resources (including Minerals and Forestry), and Building Materials.

108. This will ensure that there is no break in the cycle of resource allocations for a value chain and that adequate resources are allocated to the entities along the value chain. Of importance **Mr. Speaker,** this will eliminate duplication of both roles and budgeting for resources as well as gaps and thus ensure efficiency.

Agricultural Transformation and Inclusive Growth

109. Mr. Speaker, the Government will implement interventions to ensure food security in the country through climate change

mitigation and adaptation, thereby reducing the cost of living. As part of the country's long-term food security plan, the Government, working with the private sector, will continue to subsidize fertilizer in order to make it available and improve productivity in counties.

110. This intervention is also aimed at creating jobs as agriculture has the highest employment multiplier effect owing to its strong forward and backward linkages to other sectors of the economy.

111. Mr. Speaker, in order to support our aspirations of attaining food and nutrition security, I propose to the National Assembly to effect budget allocations of **Ksh 49.9 billion** for relevant programs in this budget. Out of this, **Ksh 5.0 billion** is proposed for the Fertilizer Subsidy Programme; **Ksh 8.6 billion** for the National Agricultural Value Chain Development Project; **Ksh 2.7 billion** for the National Agricultural and Rural Inclusivity Project; **Ksh 2.1 billion** for the Kenya Cereal Enhancement Programme; **Ksh 2.8 billion** for Emergency Locust Response; **Ksh 1.4 billion** for Small Scale Irrigation and Value Addition Programme; **Ksh 1.3 billion** for Food Production and Nutrition Security Programme;

Ksh 500 million for Agricultural Sector Development Support Programme and **Ksh 596 million** for the Food Security and Crop Diversification Project.

112. Mr. Speaker, to improve livestock production, I propose to the National Assembly to effect budget allocations of **Ksh 3.7 billion** to De-Risking, Inclusion and Value Enhancement of Pastoral Economies Programme; **Ksh 2.1 billion** for Livestock Value Chain Support Project; **Ksh 1.5 billion** for Kenya Livestock Commercialization Programme and **Ksh 166 million** for the Embryo Transfer Project.

113. I also propose to the National Assembly to effect budget allocations of **Ksh 350 million** for the Development of the Leather Industrial Park at Kenanie; **Ksh 220 million** for Livestock Production; and **Ksh 132 million** for Leather Value Chain. To enhance animal diseases control, I propose to the National Assembly to effect budget allocations of **Ksh 130 million** for Sustainable Tsetse and Trypanosomiasis Free Areas in Kenya and **Ksh 135 million** for the Establishment of Liquid Nitrogen Plant.

114. Mr. Speaker, the realization of the food and nutrition security also relies heavily on the sustainable utilization of the blue economy resources. To promote this, I propose to the National Assembly to effect budget allocations of **Ksh 2.6 billion** for the Aquaculture Business Development Project; **Ksh 3.5 billion** for Kenya Marine Fisheries & Socio-Economic Development Project; **Ksh 1.2 billion** for Marine Fish Stock Assessment, **Ksh 580 million** for Capacity Building in Deep Sea Fishing; **Ksh 142 million** for rehabilitation of Fish Landing Sites in Lake Victoria; **Ksh 141.5 million** for Aquaculture Technology Development and Innovation Transfers; **Ksh 500.7 million** for Liwatoni Ultra-Modern Fish Hub; and **Ksh 88 million** for the Development of Blue Economy Initiatives.

115. Mr. Speaker, in order to increase agricultural productivity and enhance resilience to climate change risks in targeted smallholder farming and pastoral communities in Kenya, I propose to the National Assembly to effect budget allocations of Ksh **1.5 billion** for the Climate Smart Agricultural Productivity Project; **Ksh 625 million** to Enhance Drought Resilience and Sustainable Livelihood Programme; **Ksh 318 million** towards

Ending Drought Emergencies in Kenya Project. In addition, I propose to the National Assembly to effect budget allocations of **Ksh 300 million** for Crop Insurance Scheme to reduce the vulnerabilities of Kenyan farmers to diseases and natural disasters.

116. Mr. Speaker, to ensure legitimacy of land ownership, I propose to the National Assembly to effect budget allocation of **Ksh 1.2 billion** for Processing and Registration of Title deeds; **Ksh 2.6 billion** for Settlement of the Landless; **Ksh 755 million** for Digitization of Land Registries; and **Ksh 138.3 million** for Construction of Land Registries.

Transforming the Micro, Small and Medium Enterprise (MSME) Economy

117. Mr. Speaker, to tackle the key bottleneck in MSMEs on limited access to credit, the Government rolled out in November 2022, the Financial Inclusion Fund, popularly known as the Hustlers Fund with an initial target budget allocation of **Ksh 20 billion** in the current financial year, as an intervention to correct market failure problems at the bottom of the pyramid. This program aims to lift those at the bottom of the pyramid through structured products in personal finance that includes savings,

credit, insurance and investment. This together with other affirmative action Funds like the Youth Enterprise fund, Women Enterprise Fund and UWEZO Fund and MSMEs Credit Guarantee Scheme will increase access to affordable credit to targeted persons and sectors and thereby increase their investments and allow for private sector led growth.

118. To further support MSMEs, Women and the Youth of this country, I propose to the National Assembly to effect an additional budget allocation of **Ksh 10 billion** towards the Hustlers Fund; **Ksh 300 million** to SMEs in the Manufacturing Sector, **KSh 182.8 million** for Women Enterprise Fund, **Ksh 175 million** for the Youth Enterprise Development Fund; **and Ksh 192 million** for UWEZO Fund. These allocations will inevitably guarantee access to affordable credit to the Hustlers, households and MSMEs, and therefore accord them opportunities to make their rightful contribution to nation building.

Housing and Settlement

119. Mr. Speaker, the cost of housing is a heavy burden to majority of Kenyans and is the main factor driving the

proliferation of slums in the country. The Government's commitment is to turn the housing challenge into an economic opportunity to create quality jobs for over 100,000 young people graduating from TVETs every year directly in the construction sector and indirectly through the production of building products. This will be achieved through among other measures facilitating delivery of 250,000 houses per annum and enabling affordable housing mortgages.

120. To ensure success of this initiative, I propose to the National Assembly to effect budget allocations of **Ksh 35.2 billion** for the Housing Programme. This includes: **Ksh 7.3 billion** under the Kenya Urban Programme (KenUP); **Ksh 5.0 billion** to Kenya Mortgage Refinance Company for enhancement of the company's capital as well as for on lending to primary mortgage lenders; **Ksh 3.2 billion** for Construction of Affordable Housing Units as well as **Ksh 3.3 billion** for Construction of Social Housing Units.

121. Other key proposed allocations to the Housing, Urban Development and Public Works sector includes **Ksh 5.5 billion** for

the Kenya Informal Settlement Improvement Project-Phase II; **Ksh 5.2 billion** for construction of markets; **Ksh 932 million** for maintenance of Government Pool Houses; **Ksh 1.1 billion** for the construction of Housing Units for the National Police and Kenya Prison; **Ksh 637 million** for the Kenya Municipal Programme; **Ksh 150 million** for construction of foot bridges and **Ksh 300 million** for the Development of Appropriate Building Technology.

Enhancing Quality and Affordable Healthcare

122. Mr. Speaker, the Kenya Kwanza Administration is committed and determined to realise the constitutional right to health in the shortest time possible by delivering a Universal Health Coverage (UHC) system. This entails promoting access to quality and affordable healthcare though the Universal Health Coverage programme.

123. Towards this end, **Mr. Speaker,** I propose to the National Assembly to effect budget allocation of **Ksh 141.2 billion** to the health sector to support various programmes aimed at supporting Universal Health Coverage. **Mr. Speaker,** specific proposed allocations for various activities and programmes include: **Ksh**

18.4 billion for Universal Health Coverage; **Ksh 3.7 billion** for the Kenya COVID-19 Emergency Response Project; **Ksh 4.1 billion** for Free Maternity Health Care; **Ksh 5.9 billion** for the Managed Equipment Services as well as **Ksh 1.7 billion** to provide medical cover for the elderly and severely disabled persons in our society. **Mr. Speaker,** to lower cases of HIV/AIDS, Malaria and Tuberculosis in the country, **Ksh 24.8 billion** is proposed under the Global Fund; and Ksh **4.6 billion** to enhance Vaccines and Immunizations Programme.

124. Mr. Speaker, to promote early diagnosis and management of cancer, and reduce the burden of treatment among Kenyans, I propose to the National Assembly to effect a budget allocation of **Ksh 1.9 billion** for Construction of a Cancer Centre at Kisii Level 5 Hospital; **Ksh 500 million** for strengthening of Cancer Centre Management at Kenyatta National Hospital; and an additional **Ksh 155.0 million** is proposed for the establishment of Regional Cancer Centres.

125. Further, to improve health service delivery, I propose to the National Assembly to effect budget allocations of **Ksh 21.6 billion**

for Kenyatta National Hospital; **Ksh 12.8 billion** for the Moi Teaching and Referral Hospital; **Ksh 8.8 billion** for the Kenya Medical Training Colleges; **Ksh 3.3 billion** for the Kenya Medical Research Institute; **Ksh 2.4 billion** for the construction of Kenya National Hospital Burns and Paediatrics Centre; **Ksh 1.4 billion** for the Equipping of Laboratories and Classroom at KMTC; **Ksh 1.1 billion** for the renovation of Kenyatta National Hospital; **Ksh 1.0 billion** for procurement of family planning & reproductive health commodities; **Ksh 1.0 billion** for procurement of equipment for the National Blood Transfusion Services; and **Ksh 352 million** for the Digital Health Platform.

Digital Superhighway and Creative Economy

126. Mr. Speaker, the digital superhighway will play a critical role in enabling the Government achieve the objectives of the Bottom-Up Economic Transformation Agenda as well as in enhancing revenue collection via automation of VAT systems. Digitization and automation will increase productivity and competitiveness through providing quick access to the market and reducing information assymetry as well as improving risk management. Promotion of music, theatre, graphic design, digital animation,

fashion and craft, among others will increase employment for the youth.

127. Mr. Speaker, the implementation of these interventions is expected to stimulate economic recovery while at the same time securing sustainable and inclusive growth amidst emerging challenges. But more importantly improve the quality of growth from public sector investment led growth to a dynamic private sector led growth.

128. To achieve these aspirations, I propose to the National Assembly to effect budget allocations of **Ksh 15.1 billion** to fund initiatives in the Information, Communication and Technology sector. Specifically, this proposed allocation includes **Ksh 600 million** for Government Shared Services. To fast track the development of the Konza Technopolis City, I propose to the National Assembly to effect budget allocations of **Ksh 4.8 billion** for the Horizontal Infrastructure Phase I; **Ksh 1.2 billion** for Konza Data Centre and Smart City Facilities; and **Ksh 475 million** for construction of Konza Complex Phase 1B.

129. Mr. Speaker, other proposed allocations include: **Ksh 5.7 billion** for the Construction of Kenya Advanced Institute of Science and Technology (KAIST) at Konza Technopolis; **Ksh 1.3 billion** for maintenance and rehabilitation of the National Optic Fibre Backbone Phase II Expansion Cable; and **Ksh 583 million** for maintenance and rehabilitation of Last Mile County Connectivity Network.

130. Mr. Speaker, having highlighted expenditures under the five priority areas of the Bottom–Up Economic Transformation Agenda, I now turn to other proposed areas of expenditures in this budget that will support our path towards sustainable and resilient economic growth trajectory.

Investing in Critical Infrastructure

131. Mr. Speaker, the Government will continue to expand critical infrastructure in roads, railways, sea and airports to create an enabling environment for economic recovery and employment creation. Towards this end, I propose to the National Assembly to effect budget allocations of **Ksh 244.9**

billion, to support construction of roads and bridges as well as their rehabilitation and maintenance.

132. Mr. Speaker, to expand railway transport, I propose to the National Assembly to effect budget allocations of **Ksh 37.4 billion** for the Standard Gauge Railway; **Ksh 579 million** for Rehabilitation of Locomotives; and **Ksh 889 million** for the Development of Nairobi Railway City. To facilitate movement of goods and people, I propose to the National Assembly to allocate **Ksh 1.1 billion** for Nairobi Bus Rapid Transport Project; and **Ksh 300 million** for Acquisition of Ferries for Lake Victoria.

133. I have also proposed to the National Assembly to effect budget allocations of Ksh **727 million** for construction and expansion of airports and airstrips; **Ksh 500 million** for the Smart Driving Licence; and **Ksh 2.6 million** for Infrastructure Development in Dongo Kundu Special Economic Zone.

134. Mr. Speaker, to support production of reliable and affordable energy, I propose to the National Assembly to effect budget allocations of **Ksh 62.3 billion**. Out of this, **Ksh 33.8 billion** will cater for the National Grid System; **Ksh 12.1 billion** for Rural

Electrification; **Ksh 11.4 billion** for Development of Geothermal Energy; and **Ksh 3.2 billion** for Alternative Energy Technologies.

Improving Education Outcomes

135. Mr. Speaker, the ultimate means of ensuring an equitable society is through investment in education, as part of human capital development. For this reason, the Government will continue to address inequities in our education system so as to level the playing field for all children irrespective of their background.

136. Mr. Speaker, to improve education outcomes in the country, I propose to the National Assembly to effect budget allocations of Ksh **628.6 billion** to the Education Sector. Out of the proposed allocation, **Ksh 12.5 billion** will cater for Free Primary Education; **Ksh 65.4 billion** for Free Day Secondary Education including insurance under NHIF for secondary school students; **Ksh 25.5 billion** for Junior Secondary School Capitation; **Ksh 5.0 billion** to support examinations fee waiver; **Ksh 4.8 billion** for Recruitment of 20,000 Intern Teachers; **Ksh 1.0 billion** for promotion of

teachers; **Ksh 4.9 billion** for the School Feeding Programme; **Ksh 940 million** for the provision of sanitary towels.

137. In addition, **Mr. Speaker,** I propose to the National Assembly to effect budget allocations of **Ksh 1.3 billion** for training of teachers on Competency Based Curriculum and **Ksh 400.0 million** for the Digital Literacy Programme and ICT Integration in our Secondary Schools. To support infrastructure development and ensure safe learning in our schools, I propose to the National Assembly to effect budget allocations of **Ksh 6.0 billion** for Primary and Secondary schools' infrastructure including classrooms for Junior Secondary Schools and **Ksh 1.9 billion** for construction and equipping of Technical Training Institutes and Vocational Training Centres. Further, **Ksh 1.8 billion** is proposed to increase access and improve the quality of Technical and Vocational Education and Training programs under the East Africa Skills Transformation and Regional Integration Project.

138. Mr. Speaker, I propose to the National Assembly to effect budget allocations to the education sector to include: **Ksh 316.7 billion** to Teachers Service Commission; **Ksh 97.5 billion** for

University Education; **Ksh 30.3 billion** to the Higher Education Loans Board; **Ksh 2.7 billion** for Kenya Secondary Education Quality Improvement Project and **Ksh 5.2 billion** Capitation for TVET students. **Mr. Speaker, Ksh 980 million** is proposed for Technical, Vocational Education Training and Entrepreneurship; **Ksh 1.5 billion** for promotion of Youth Employment and Vocational Training; and K**sh 749.0 million** for the Research, Science, Technology and Innovation.

Supporting Manufacturing for Job Creation

139. Mr. Speaker, in order to improve productivity in the manufacturing sector, as earlier indicated, the Government has adopted a value chain approach through the Bottom-Up Economic Transformation Agenda. This will address the bottlenecks that impede the growth of the manufacturing sector in order to create jobs and enhance the country's competitiveness.

140. To further promote local industries, I propose to the National Assembly to effect budget allocations of **Ksh 26.9 billion** under various implementing Ministries, Departments and

Agencies. Out of this, **Ksh 4.7 billion** will support Establishment of County Integrated Agro-Industrial Parks; **Ksh 3.0 billion** for construction of six Export Processing Zones flagship projects; **Ksh 3.1 billion** for Supporting Access to Finance & Enterprise Recovery (SAFER) Project; **Ksh 500 million** for the Development of SEZ Textile Park Naivasha and **Ksh 1.8 billion** for the construction of an Effluent Treatment Plant at Kenanie.

141. In addition, **Mr. Speaker,** in order to maximize the benefits from our cash crops, the Government will make further investments towards their revival and enhancement of output. In this respect, I propose to the National Assembly to effect budget allocations for the revitalization of the cash crops; **Ksh 120 million** for Cotton; **Ksh 62 million** for Coconut; **Ksh 35 million** for Cashewnut; and **Ksh 150 million** for Pyrethrum.

142. Mr. Speaker, I also propose to the National Assembly to effect budget allocations of **Ksh 100 million** for modernization of cooperative cotton ginneries; **Ksh 134 million** for National Edible Oil Crops Promotion Project; and **Ksh 270 million** for Sugar Reforms Support Project.

143. Mr. Speaker, to equip our youth with essential training and internship opportunities; I propose to the National Assembly to effect budget allocation of **Ksh 1.5 billion** for the Kenya Industry and Entrepreneurship Project; **Ksh 300 million** for the Kenya Youth Employment and Opportunities Project; **Ksh 332 million** for the Construction of Industrial Research Laboratories and **Ksh 182.9 million** for Constituency Industrial Development Centers.

Improving Security

144. Mr. Speaker, security is critical for creating a safe environment for investment and protection of our citizens and their property. For this reason, I propose to the National Assembly to effect budget allocation of **Ksh 338.2 billion** to support operations of the Kenya Defence Forces, National Police Service, the National Intelligence Service and Prison Services.

145. Mr. Speaker, the proposed allocations include: **Ksh 144.9 billion** for Kenya Defence Forces; **Ksh 98.6 billion** for National Police Service; **Ksh 44.3 billion** for the National Intelligence Service; and **Ksh 31.3 billion** for Prisons Services. I have also proposed **Ksh 8.8 billion** for leasing of police motor vehicles. **Mr.**

Speaker, to better equip the National Police Service, I have proposed an initial allocation of **Ksh 500 million** for the Police Modernization Programme.

146. Mr. Speaker, I will later be requesting this august House for enhanced funding approval once the ongoing discussion on a Comprehensive Police Modernization Programme is concluded. In addition, **Mr. Speaker,** to step up war on crime and enhance support to administration of justice, I propose to the National Assembly to effect budget allocation of **Ksh 856 million** to equip the National Forensic Laboratory.

147. Mr. Speaker, improvement of the welfare of our Disciplined Forces is at the centre of the Kenya Kwanza Administration. To improve housing accommodation for our Defence Forces, we shall fast track the delivery of staff houses through the Public Private Partnership Framework. We have already allowed Kenya Defence Forces to retain **Ksh 534 million** currently collected from rental income and apply it towards repayment of the houses once complete. In addition, **Mr. Speaker,** as you are aware His Excellency, The President appointed a Taskforce to review the

terms and conditions of the Police, Prisons and National Youth Service, we shall be presenting a funding proposal to this House once the discussions are concluded and adopted within Government.

148. Mr. Speaker, other proposed allocations include **Ksh 2.9 billion** for Group Personal Insurance for Police and Prisons Services and **Ksh 5.8 billion** for Medical Insurance for Police and Prisons Services.

Protecting the Vulnerable Groups

149. Mr. Speaker, at the heart of the Government's development agenda, is the inclusion in society and employment opportunities for vulnerable members. In this regard, I propose to the National Assembly to effect budget allocations of **Ksh 38.2 billion** for social protection and affirmative actions in this budget. Out of this proposed allocation, **Ksh 18.0 billion** will cater for cash transfers to elderly persons, **Ksh 7.9 billion** for Orphans and Vulnerable Children and **Ksh 1.2 billion** for persons living with severe disabilities. **Mr. Speaker,** to ensure the elderly and other vulnerable groups under the Cash Transfer Programme do not

take long waiting for their cash, payment of the cash transfers shall be prioritized and paid alongside the monthly Government payroll as and when it is due.

150. Mr. Speaker, the proposed allocations to the vulnerable members of our society also includes **Ksh 5.6 billion** for the Kenya Hunger Safety Net Programme and **Ksh 3.3 billion** for the Kenya Social and Economic Inclusion Project. In addition, **Ksh 900 million** will go to the Child Welfare Society of Kenya; **Ksh 400 million** for the Presidential Bursary for the orphans and **Ksh 459 million** for National Development Fund for Persons living with Disabilities. These allocations shall remain "ring-fenced" from any budget rationalization, and will be fully funded in order to achieve the desires results.

Equity, Poverty Reduction, Women and Youth Empowerment

151. Mr. Speaker, the most pressing challenge in our country at the moment is lack of job opportunities for the youth. This has been exacerbated by the hard economic times following several negative shocks explained earlier in this Statement. In order to empower the youth and support businesses owned by youth and

women, I propose to the National Assembly to effect budget allocations of **Ksh 81.9 billion** for these initiatives.

152. The proposed allocations include: **Ksh 13.1 billion** for the National Youth Service; **Ksh 602 million** for the Kenya Youth Empowerment and Opportunities Project; **Ksh 300 million** for establishment of Youth Empowerment Centers; and **Ksh 229.7 million** to implement the Vijana Vuka na Afya (VIVA) Youth Programme.

153. To support the film industry, I propose to the National Assembly to effect budget allocations of **Ksh 100 million** to strengthen the film industry in Kenya; **Ksh 49 million** for film location mapping and **Ksh 100 million** for the Kenya Film School.

154. Mr. Speaker, women participation in the key sectors of our economy is critical. For this reason, the Government will sustain increased incomes for women, through employment creation and supporting women led enterprises. We will further strengthen women participation in decision-making in our governance and political institutions as well as reduce sexual and gender-based violence provision and provision of affordable Health services.

To further empower our women, I propose to the National Assembly to effect budget allocations of **Ksh 245 million** for Strengthening Prevention and Response to Gender Based Violence in Kenya Project; and **Ksh 3.0 billion** for the National Government Affirmative Action Fund.

155. Mr. Speaker, to promote regional equity, reduce poverty and enhance social development, I propose to the National Assembly to effect budget allocations of **Ksh 53.5 billion** for the National Government Constituency Development Fund as well as **Ksh 10.9 billion** for the Equalization Fund to finance programmes in previously marginalized areas.

Stimulating Tourism growth, Sports, Culture, Recreation and Arts

156. Mr. Speaker, the Government has prioritized expanding the space for creativity, including freedom of expression and protection of intellectual property rights. We will also strengthen mainstreaming of arts and culture infrastructure and support cultural production and the creative economy. We are also cognizant of the brand value of Kenyans participating and excelling in international sports arena as of ultimate importance.

157. To support Tourism, Sports, Culture and recreation, I propose to the National Assembly to effect budget allocations of **Ksh 12.5 billion**. This includes an allocation of **Ksh 6.4 billion** under the Sports, Arts and Social Development Fund; **Ksh 4.1 billion** for the Tourism Fund and **Ksh 2.0 billion** for Tourism Promotion Fund.

Environmental Protection, Water and Natural Resources

158. Mr. Speaker, environmental conservation and response to the impacts of climate change are at the centre of the Government's socio-economic transformation agenda. To expand access to clean and adequate water for domestic and agricultural use, I propose to the National Assembly to effect budget allocations of **Ksh 43.3 billion** for Water and Sewerage Infrastructure Development; **Ksh 12.4 billion** for Water Resources Management; and **Ksh 1.6 billion** for Water Storage and Flood Control. In addition, I propose **Ksh 19.7 billion** for Irrigation and Land Reclamation and **Ksh 1.9 billion** for Water Harvesting and Storage for Irrigation.

159. Mr. Speaker, in order to support environment and water conservation and respond to climate change, I propose to the National Assembly to effect budget allocations of **Ksh 14.4 billion** for forests and water towers conservation; **Ksh 3.8 billion** for environment management and protection; **Ksh 3.6 billion** for the Kenya Financing Locally Led Climate Action Project; **Ksh 1.5 billion** for Meteorological Services. In addition, I have proposed an allocation of **Ksh 1.1 billion** for Human Wildlife Conflict Compensation; **Ksh 800 million** for Wildlife Insurance; **Ksh 400 million** for Maintenance of Access Roads and Airstrips in Parks; **Ksh 319 million** for drilling of boreholes in protected areas for provision of water for wildlife and **Ksh 226 million** for Wildlife Research Facilities.

Improving Governance and Sustaining the Fight against Corruption

160. Mr. Speaker, the Government is committed to scale up the implementation of the provisions of the 2010 Constitution, strengthen the rule of law, increase access to justice and ensure respect for human rights.

161. Mr. Speaker, to enhance good governance and scale up fight against corruption, I propose to the National Assembly to effect budget allocations of **Ksh 3.9 billion** for the Ethics and Anti-Corruption Commission; **Ksh 3.6 billion** for the Office of the Director of Public Prosecutions; **Ksh 8.0 billion** for the Criminal Investigations Services; and **Ksh 8.0 billion** for the Office of the Auditor General.

162. Additionally, to enhance the oversight and legislative role of Parliament and access to justice, I propose to the National Assembly to effect budget allocations of **Ksh 41.0 billion** for Parliament; and **Ksh 23.2 billion** for the Judiciary.

Completion of Planned Projects

163. Mr. Speaker, to ensure that value for money and timely realization of benefits accruing from budgetary allocations and implementation of planned projects, Ministries, Departments and Agencies have identified projects for completion in the course of the financial year. The projects will be fast tracked and protected from any adjustments. The National Assembly is requested to

support this endeavour, as this is consistent with the resolution of this House.

Transfers from the National Government to County Governments

164. Mr. Speaker, the County Governments will receive a proposed allocation of **Ksh 385.4 billion** as equitable share in the FY 2023/24. This is 23 percent of the FY 2019/20 shareable revenue, which is the most recent audited and approved revenue by the National Assembly. This is above the minimum threshold required under Article 203(2) of the Constitution.

165. In addition to County equitable share, the County Governments will receive additional conditional and unconditional allocations amounting to **Ksh 56.7 billion**. This brings the total allocation to the County Governments in the FY 2023/24 to **Ksh 442.1 billion**. Part of this include **Ksh 425 million** for the Transfer of Library Services being a devolved function; **Ksh 2.9 billion** as outstanding mineral royalties share to 32 County Governments as stipulated in the Mining Act, 2016; a Conditional Grant of **Ksh 100 million** per county for the Aggregated Industrial

Parks Programme, which is meant to assist the farmers in value addition and market access for their products.

Equalization Fund

166. **Mr. Speaker,** in line with Article 204(1) of the Constitution of Kenya, **Ksh 7.9 billion** has been allocated under the Equalization Fund in the financial year 2023/24. This represents **0.5 percent** of the FY 2019/20 revenue which is the most recent audited and approved revenue by the National Assembly. **Mr. Speaker,** in addition to this, we have allocated **Ksh 3 billion** to cater for part arrears from the previous years bringing the total allocations under Equalization Fund to **Ksh 10.9 billion**.

Transfer of Functions between National Government and County Governments

167. **Mr. Speaker,** the National Treasury and Economic Planning in collaboration with the Council of Governors and other institutions, developed a draft legislation to operationalize Articles 187 and 189 of the Constitution on Transfer of Functions and Cooperation between the National and the County Governments and amongst County Governments. The proposed

legislation has gone through Public Participation with County Government Executives, County Assemblies, Development Partners among others. **Mr. Speaker,** I will shortly submit the Bill for Cabinet approval and onward transmission to Parliament.

Measures to Enhance County Governments' Own Source Revenue

168. Mr. Speaker, the National Treasury and Economic Planning; State Departments of Lands, Council of Governors and other stakeholders have developed the National Rating Bill, 2022 which went through the first reading on 8th March, 2023 in the National Assembly and thereafter was submitted to the National Assembly Committee on Lands. The Bill provides for among others, standards in the way Rating and Valuation is conducted in the country; how to deal with properties cross-cutting in more than one county government; procedure for claiming and payment of Contribution in Lieu of Rates (CILOR); and timely updating of valuation rolls by the county governments. More importantly, the Bill will repeal the outdated Valuation for Rating Act, Cap 266 and Rating Act, Cap 267 and align property rating legal regime with the devolved system of governance. Our appeal

to this House is to consider and fast track this Bill to unlock revenue potential to the County Governments from property taxation as envisage in Article 203(3).

Sharing of Mineral Royalty Revenues

169. Mr. Speaker, Mining Act, 2016 assigns 70 percent of mineral royalties collected from Mining companies to the National Government; 20 percent to county governments; and, 10 percent to communities. Since 2016, county governments and communities have not received their share of these royalties. To address this matter, I have submitted a framework for sharing **Ksh 2.9 billion** among 32 County Governments for mineral royalties outstanding as at 30th June, 2022 for consideration and approval by Parliament.

170. Mr. Speaker, during the Intergovernmental Budget and Economic Council (IBEC) 17th Ordinary Session held on 31st May, 2022, IBEC considered and adopted a report and a framework for sharing of funds arising from the contravention of County Government legislation. In the next Financial Year, the National

Treasury has proposed **Ksh 108 million** to be shared among the beneficiary County Governments.

171. Mr. Speaker, enhancement of County Governments has had its own challenges. One of such challenges is multiplicity of fees and charges imposed by the County Governments that increases the cost of doing business and negatively impacts on our competitiveness as a Country. Some County Governments have imposed charges in a way that prejudices national economic policies, economic activities across County boundaries or the national mobility of goods, services, capital or Labour. As a result, the National Treasury and Economic Planning has been receiving a lot of complaints from the Ministries, Departments, State Agencies, and the private sector relating to the uncoordinated manner in which Counties are imposing multiple fees and charges.

172. Mr. Speaker, some neighbouring countries have complained that some of the charges imposed by the County Governments are against the East African Community (EAC) Customs Management Act and the EAC Common Market Protocol and may result in retaliatory measures and trade disruptions between

Kenya and other Partner States in the EAC. To address these issues, the National Treasury and Economic Planning has resubmitted the County Governments' (Revenue Raising Process) Bill, 2023 to Parliament for consideration and enactment. I urge this House to expedite the enactment of this Bill in order to promote the competitiveness of our country in doing business and open up the country for investment.

VI. TAXATION MEASURES

173. Mr. Speaker, I will now turn to tax policy measures aimed at generating revenue to finance the FY 2023/24 budget. I will begin by highlighting the key measures on custom duty as agreed by the Ministers responsible for Finance and Economic Affairs in the East African Community during the Pre-Budget consultations meeting held in Arusha in May 2023.

174. Mr. Speaker, during the EAC Pre-Budget consultations meeting, the Ministers noted the significant contribution of manufacturing sector to job creation and economic growth in the region. The Ministers therefore agreed on measures to prevent the proliferation of cheap imports into the region so as to protect

local manufacturers from unfair competition and enable them access raw materials and inputs at affordable price.

175. Mr. Speaker, the agreed custom measures shall become effective from 1st July, 2023.

176. Mr. Speaker, one of the success stories of the East African Community is the establishment of the EAC Customs Union as a single customs territory. In order to streamline importation of goods into the customs territory, the EAC region completed the comprehensive review of the Common External Tariff (CET) and this has been under implementation from 1st July 2022. The revised Tariff has a four-tariff band structure compared with the earlier three-tariff band structure. In the new structure, raw materials/inputs and capital goods are imported duty free; intermediate goods at the rate of 10 percent while finished goods which are not available in the region at 25 percent. Finally, **Mr. Speaker,** all finished goods which are available in the region in sufficient quantities, attract a CET rate of 35 percent. The revised tariff bands are meant to cater for goods with long value chain which require differentiated rates to promote investments.

177. Mr. Speaker, despite rice and wheat being among the staple food items in our homes, Kenya's production of these food items does not meet the domestic demand. In order to meet domestic demand, Kenya will import rice at a rate of 35 percent, instead of 75 percent under the EAC Common External Tariff. Further, in order to ensure that there is enough wheat to meet local demand, while at the same time protecting wheat farmers from unfair competition from imported wheat, Kenya will import wheat under the EAC Duty Remission Scheme at 10 percent instead of 35 percent. The importation of wheat will be undertaken upon the recommendation by the Ministry of Agriculture who will ensure wheat millers, as a priority, purchase local wheat before they are allowed to import.

178. Mr. Speaker, in order to support local automotive assembly in the region, the EAC Partner States agreed to continue with the importation of raw materials for manufacture of parts used in the assembly of motor vehicles especially leaf springs, radiators and wiring harness under duty remission. In addition, the region agreed to extend importation of Completely Knocked Down (CKDs) kits for assembly of motor cycles at 10 percent under duty

remission, as the region awaits the finalization of the EAC Assembly Regulations.

179. Mr. Speaker, Kenya has sufficient capacity to manufacture various products in the metal and allied sub sector thus providing employment opportunities to many Kenyans. In order to continue safeguarding this sub-sector, the EAC Ministers agreed that imported iron and steel products, shall continue attracting a duty rate of 35 percent with the corresponding specific rates for a further one year.

180. Mr. Speaker, our hardworking farmers are capable of producing enough food to satisfy domestic demand. In particular, local demand for most vegetable products has been adequately served by our farm produce over the years. To protect these farmers from cheap imports, Kenya has been allowed to extend the import duty rate of 35 percent in addition to the specific import duty rates on vegetable products for a further one year.

181. Mr. Speaker, high cost of inputs of animal feeds coupled with other factors like unfavourable weather conditions make the locally manufactured animal feeds expensive hence contribute to

food inflation. In order to promote the local manufacturing and make animal feeds affordable to farmers, Kenyans will import inputs for manufacture of animal feed duty-free under the EAC Duty Remission Scheme for one year.

182. Mr. Speaker, last year we allowed manufacturers of baby diapers to access inputs for manufacture of baby diapers duty free. This was meant to lower the cost of manufacturing these essential products. I am happy to note that many companies have responded to this incentive by increasing production. In order to continue making these products affordable for our babies, the EAC Minsters allowed Kenya to extend the duty-free importation of inputs for manufacture of baby diapers for a further one year. Moreover, to protect these manufacturers from competition from imported products, the region allowed Kenya to extend charging imported baby diapers at the rate of 35 percent for a further one year.

183. Mr. Speaker, leather industry is one of the priority value chain under the Bottom - Up Economic Transformation Agenda. In order to support local value addition and protect the industry

from unfair competition, the EAC Partner States agreed to retain the import duty on imported leather and footwear products at a rate of 35 percent. In addition, Kenya was allowed to extend the duty-free importation of raw materials and inputs for manufacture of footwear products.

184. Mr. Speaker, Kenya has adequate capacity to meet local demand for roofing tiles. In order to facilitate access to Kenyan made roofing tiles at affordable prices, Kenya was granted extension of duty-free window for importing inputs for the manufacture of roofing tiles for a further one year.

185. Mr. Speaker, in order to protect the Kenyan producers of paper and paper products from the proliferation of cheap imports, Kenya was allowed to import these goods at a rate of 35 percent for the next one year.

186. Mr. Speaker, in order to protect local production of safety matches, particleboards, plywood, among other goods of timber, Kenya was allowed to extend charging of specific rates of import duty in addition to ad valorem duty ranging from USD 120 per Metric Tonne to USD 200 per Metric Tonne on these goods. This

is also meant to curb undervaluation of the goods and safeguard revenues. Further, Kenya was allowed to charge a rate of 45 percent on imported furniture in order to protect local manufacturers and producers.

187. Mr. Speaker, Kenya has sufficient capacity to manufacture plastic and rubber products to supply both the domestic and regional market. In order to protect local manufacturers, Kenya was allowed to impose import duty rate of 35 percent on these goods for one year instead of 25 percent.

188. Mr. Speaker, in order to protect local assembly of smartphones and other mobile phones, Kenya was allowed to extend the import duty rate of 25 percent on smartphones and other telephones for cellular networks or for other wireless networks for one year. Further, Kenya was allowed to extend duty free importation of inputs for assembly of smartphones and other cellular phones.

189. Mr. Speaker, Kenya has capacity to produce billets for manufacture of wire rods and other similar products to meet the local demand. In order to protect the local manufacturers of these

goods, Kenya was allowed to charge an import duty of 10 percent on billets.

190. Mr. Speaker, I now turn to the tax measures proposed in the Finance Bill, 2023.

Amendments to the Value Added Tax Act, 2013

191. Mr. Speaker, I now embark on measures proposed under the Value Added Tax Act, 2013.

192. Mr. Speaker, the high cost of Liquefied Petroleum Gas (LPG) contributes to the escalating cost of living and continued use of firewood and charcoal which negatively affects our forest cover. In order to make LPG affordable and promote its uptake as well as encourage the use of clean sources of energy, I am proposing to the National Assembly to zero rate VAT on LPG.

193. Mr. Speaker, currently, exported services are subject to VAT at the rate of 16 percent. This has caused an increase in the cost of exported services hence affecting the competitiveness of our services in the global market. In order to increase competitiveness and encourage exportation of taxable services, I propose to the National Assembly to remove VAT on exported taxable services.

194. Mr. Speaker, currently, tea purchased for local value addition, either from the factories or tea auctions centres is vatable, affecting the cash flows of tea exporters. In order to encourage local value addition of our tea, I propose to the National Assembly to remove VAT on tea purchased from factories or tea auction centres for value addition and subsequent export. This will improve cash flows for tea exporters involved in local value addition.

195. Mr. Speaker, currently helicopters, aeroplanes and other aircrafts of unladen weight not exceeding 2,000 kgs are subject to VAT. Further, some spare parts of tariff number 8803 are expressly exempt from VAT. However, VAT exemption on any other aircraft spare parts imported by aircraft operators or persons engaged in the business of aircraft maintenance is contingent upon recommendation by the competent authority responsible for civil aviation. This process creates administrative burden on the taxpayer to comply with the requirements. **Mr. Speaker,** we have also noted that the cost of aircraft maintenance and training activities has been high prompting airlines to prefer undertaking these activities in other jurisdictions. In order to ease

the process of VAT exemption of aircrafts spare parts and encourage investment in this area, I propose to the National Assembly to remove VAT on all aircrafts, simulators for training our pilots and aircraft spare parts. This proposal to expressly exempt from VAT all aircraft and spare parts of Chapter 88 will spur the growth in the aviation sector and reduce administrative burden of accessing its VAT exemption.

196. Mr. Speaker, the international best practice is to charge VAT on all goods and services and especially those that have undergone some form of processing. This is not the case in our VAT regime as some goods that have undergone some processing are exempt from VAT while others are zero-rated. This has impacted on the tax expenditures. In order to bring down the tax expenditure and also align with the international best practice, I propose to the National Assembly to rationalize goods and services in the First and Second Schedule of the VAT Act by transferring some goods and services from zero rating to exempt status and from exempt status to taxable at the standard rate.

197. Mr. Speaker, over the years, petroleum products have received preferential tax treatment gradually transitioning from exempt status in 2013-2018 and 8 percent from 2019 to-date. This is irrespective of the fact that suppliers of petroleum products claim other input tax at the general rate of 16 percent, which means the Government has been sharing the burden of the input tax with the businesses by subsidizing the difference of the general rate of 16 percent and the preferential rate of 8 percent. As a result of these practices and outcomes, the oil markers have been in perpetual credit position and not paying VAT to the Exchequer. This practice is discriminatory as businesses in other sectors of the economy bear the input tax by recovering the same from output VAT charged on the final consumer.

198. Mr. Speaker, on reviewing the VAT rate on petroleum products to the standard rate, such businesses would at the onset recover the VAT credits that they have been carrying forward over the years and Government would end up collecting VAT after some time. **Mr. Speaker,** if the anomaly is not corrected at the earliest, the build-up of credits will continue to increase and should the Government seek to charge VAT at the standard rate

in the future, it would take years for the Government to collect the much-needed revenues from this sector since the credits have to be exhausted before the businesses start paying VAT.

199. The continued practice by Government to subsidize the cost of fuel by levying a preferential rate of 8 percent distorts the market, yet the economy should be operating on the principles of demand and supply. **Mr. Speaker,** in this respect, I propose to the National Assembly to amend the VAT Act to remove the preferential rate on the petroleum products so that the product will be subject to the standard VAT rate of 16 percent.

200. Mr. Speaker, the blue economy remains largely untapped despite its huge potential. This is particularly due to low investments in maritime activities over the years. In order to spur investment and attract international sea freight in this sector, I propose to the National Assembly to zero rate inbound international sea freight offered by registered persons. The Government will continue exploring ways of providing more incentives to promote investment in this sector.

201. Mr. Speaker, currently, machinery used by pharmaceutical companies are exempt from VAT on importation. This is meant to compliment Government's efforts to ensure that all Kenyans have access to affordable healthcare. However, we have received numerous petitions to extend the exemption to locally purchased machinery to further boost access to healthcare services. In this regard, I propose to the National Assembly to amend the VAT Act to extend the exemption to machinery and equipment purchased locally for use in the manufacture of pharmaceuticals.

202. Mr. Speaker, in order to address the escalation of tax expenditures and foster prudent utilization of fiscal resources, I propose to the National Assembly to remove VAT exemption in respect of: taxable services supplied for construction of specialized hospitals; goods and services for the construction of tourist facilities; goods worth more than Ksh 2.0 billion purchased by manufacturers; as well as plant, machinery and equipment used in the construction of plastic recycling plants.

203. Mr. Speaker, currently transfer of business as a going concern is subject to VAT at standard rate. Taxpayers have raised concern

that this discourages business reorganization and sale of business. In this regard, I propose to the National Assembly to amend the VAT Act to exempt transfer of business as a going concern. This is expected to encourage mergers and acquisitions hence boost growth of businesses as well as efficiency.

Amendments to the Excise Duty Act, 2015

204. Mr. Speaker, I will now highlight the tax measures proposed under the Excise Duty Act.

205. The Excise Duty Act provides for remittance of excise duty to the Kenya Revenue Authority (KRA) by 20th day of the following month. In this era of technology, there is no justifiable reason for waiting for twenty days to report transactions to the KRA. In order to cure the delay, I propose to the National Assembly to amend the Act to provide for remittance of the excise duty on betting and gaming activities within 24 hours after closure of transactions by the betting and gaming companies.

206. Mr. Speaker, betting, gaming, prize competition and lotteries are addictive in nature and have negative socio-economic effects to the society. To discourage participation in

these activities by Kenyans and especially school going children, I propose to the National Assembly to increase the rate of excise duty on betting, gaming, prize competition and lotteries from the current rate of 7.5 percent to 12.5 percent.

207. Mr. Speaker, there has been an increase in importation of fish into the country which has negatively affected the local fish industry. To promote and protect local fish industry which provides livelihood to many Kenyans, I propose to the National Assembly to introduce excise duty on imported fish at Ksh 100,000 per metric tonne or 10 percent of the excisable value, whichever is higher.

208. Mr. Speaker, the Government charges specific excise duty on juices per litre. However, there has been an increase in supply of juices in powder form and based on the current tax regime on juices, powdered juice is not subject to excise duty, creating unfairness in taxation of juices. In order to cure this problem and create a level playing field, I propose to the National Assembly to introduce excise duty on powdered juice at a rate of Ksh 25 per kilogram.

209. Mr. Speaker, currently, specific rates of excise duty are subject to annual adjustment for inflation. However, taxpayers are concerned with frequent changes in the specific rates of excise duty through annual adjustment creating uncertainty in prices and distorting the market. Therefore, in order to create certainty in excise tax regime, I propose to the National Assembly to repeal the provision for annual adjustment for inflation.

210. Mr. Speaker, consumption of sugar has been associated with various ailments such as diabetes which has become common in many families. To discourage consumption of sugar, I propose to the National Assembly to introduce excise duty on imported sugar at the rate of Ksh 5.0 per kilogram excluding the sugar imported or purchased locally by registered pharmaceutical manufacturers for use in the manufacture of pharmaceutical products.

211. Mr. Speaker, consumption of alcohol, betting, gaming, lottery and prize competition are extremely addictive and result to harmful repercussions in our society especially to the youth and families. In order to discourage these activities and consumption

of alcoholic beverages, I propose to the National Assembly to introduce excise duty at the rate of 15 percent of the excisable value on fees charged on the advertisements by all televisions, print media, billboards, and radio stations in promotion of alcohol, betting, gaming, lottery and prize competition.

212. Mr. Speaker, to promote financial inclusion and enhance access to internet data services, I propose to the National Assembly to reduce excise duty from 20 percent to 15 percent in respect of fees charged for telephone and internet data services and fees charged for money transfer services by banks, money transfer agencies, and other financial service providers. **Mr, Speaker**, in the same spirit, I propose to the National Assembly to reduce excise duty from 12 percent to 10 percent of the excisable value on fees charged for money transfer services by cellular phone service providers or payment service providers licensed under National Payment System. This will encourage retail transactions at a more affordable rate and promote economic activity at MSMEs level.

213. Mr. Speaker, locally produced cement has been facing stiff competition from imported cement. To protect them from unfair competition and promote this industry, I propose to the National Assembly to introduce excise duty on imported cement at a rate of 10 percent of the excisable value or Ksh 1.50 per kilogram whichever is higher.

214. Mr. Speaker, there has been an increase in the importation of furniture, especially office furniture. This has negatively affected the local carpenters and artisans considering that there is enough local capacity to make furniture. In order to protect local carpenters and artisans, I propose to the National Assembly to impose excise duty on imported furniture at the rate of 30 percent of the customs value excluding the furniture originating from EAC countries.

215. Mr. Speaker, Kenya has sufficient local capacity to produce paints, varnishes and lacquers. However, proliferation of cheap imports has reduced competitiveness of locally produced paints, varnishes and lacquers. To protect the local paint manufacturers, I propose to the National Assembly to introduce excise duty at

the rate of 15 percent of the excisable value on imported paints, varnishes, and lacquers.

Amendments to the Income Tax Act

216. Mr. Speaker, I will now highlight the changes proposed under the Income Tax Act.

217. The current interest deduction restriction based on earnings before interest, taxes, depreciation and amortization, was introduced in 2021 to discourage tax planning. This restriction currently applies to both local and foreign financial institutions. **Mr. Speaker,** I have received proposals from taxpayers who borrow loans from local financial institutions indicating that the restriction discourages their access to credit. To address this concern, I propose to the National Assembly to exclude interest on loans borrowed from local financial institutions from interest deduction restriction.

218. In addition, **Mr. Speaker,** where interest on loans borrowed from non-residents is disallowed, I propose to the National Assembly to allow businesses to carry forward that interest for

deductions in the next five subsequent years. This will align with the international best practice.

219. Mr. Speaker, in 2016, the Government introduced a simplified tax regime that requires landlords to pay tax on residential rental income at the rate of 10 percent per month. In order to enhance compliance and encourage more landlords to pay tax under this regime, I propose to the National Assembly to reduce the tax rate for this regime from 10 percent to 7.5 percent per month.

220. Mr. Speaker, in order to support start-up companies, I propose to the National Assembly to amend the Income Tax Act to defer the taxation of gains on shares offered to employees in lieu of their salaries in a start-up company for five years or till the date employment ceases or till the date of sale of the shares, whichever is earlier.

221. Mr. Speaker, upon retirement, most senior citizens have no access to medical cover and spend their little savings on healthcare services. In order to encourage employees, save for their post-retirement medical needs, I propose to the National Assembly to

provide tax relief of 15 percent of the contributions to a Post-Retirement Medical Fund or Ksh 5,000 per month, whichever is lower.

222. In addition, **Mr. Speaker,** I propose to the National Assembly to exempt from income tax the investment income earned by Post-Retirement Medical Fund. This will reduce pressure on the Government expenditure on medical care and accord the retirees access to dignified healthcare.

223. Mr. Speaker, we have noted that family members have been using exemption from capital gain tax on transfers of property between related persons as an avenue to reduce their tax liability. In order to remedy the situation, I propose to the National Assembly to change the base of capital gain tax from the sale of property to be the original cost before the transfer to the family members in instances where a property is transferred to a third party within a period of less than 5 years.

224. Mr. Speaker, despite the advantages brought about by digital platforms, the transactions usually conducted under the platforms are not in the tax net. I propose to the National

Assembly to introduce a digital asset tax at the rate of 3 percent on the value of digital asset transferred or exchanged. **Mr. Speaker,** to further create equity and fairness, I propose to the National Assembly to introduce withholding tax at the rate of 5 percent on the gross payment in respect to digital content monetisation. This will apply to incomes generated through media and digital platforms.

225. Mr. Speaker, currently non-residents pay withholding tax on payments in respect to sales promotion, marketing and advertising services. In order to harmonize taxation treatment for residents and non-residents and ensure equity, I propose to the National Assembly to introduce withholding tax on the same services to residents at the rate of 5 percent of the gross amount.

226. Mr. Speaker, resident companies currently pay 30 percent corporate tax while non-residents with permanent establishments pay 37.5 percent of their gains or profit. This creates an impression that Kenya discriminates between residents and non-residents. In order to harmonise taxation treatment for residents and non-residents companies, I propose to the National Assembly

to reduce the corporate tax rate for non-residents from 37.5 percent to 30 percent. Further, in order to ensure that there is level playing field, I propose to the National Assembly to introduce tax on repatriated profits at a rate of 15 percent which is equal to the rate charged on dividend paid to non-residents.

227. **Mr. Speaker,** businesses deduct diminution value of implements, utensils or similar articles used in the production process over a period of three years, in determination of taxable profits. In order to increase working capital to businesses, I propose to the National Assembly to amend the Income Tax Act to provide for 100 percent investment deduction of the value of these items in the first year of use.

228. **Mr. Speaker**, in 2020, the Government implemented various measures to cushion business from the adverse effects of the COVID-19 Pandemic including providing tax incentives during the period. With the easing of COVID-19 Pandemic and recovery of businesses, I propose to the National Assembly to lower the upper threshold for turnover tax from Ksh 50.0 million to Ksh 25 million and retain the lower threshold at Ksh 1.0 million. In

addition, I propose to the National Assembly that the turnover tax rate for this sector to be 3.0 percent.

229. Mr. Speaker, last year, the Government provided various tax incentives to promote local manufacturing of human vaccine. In order to further support these manufacturers, I propose to the National Assembly to exempt the manufacturers in this sector from withholding tax on interest and royalties paid to non-residents.

230. Mr. Speaker, the current PAYE tax structure has three rates and bands applicable to 3.3 million employees. The bands are as follows: 10 percent is applied to incomes up to Ksh 24,000 per month; 25 percent is applied to the next Ksh 8,333 per month while 30 percent is applied to incomes above Ksh 32,333.

231. Mr. Speaker, employees who fall under the first category and who constitute 42.5 percent of total employed workers earn a maximum of Ksh 24,000 per month implying they don't pay any tax to Government as the tax computed from this income is equal to relief of Ksh 2,400 per month under the Act. The second category of employees who constitute 37.2 percent and earn

incomes of between Ksh 24,000 and Ksh 32,333 per month, pay tax at a rate of 25 percent. The third category of employees that form 20.4 percent of total employees earn incomes above Ksh 32,333 per month and pay tax at the highest rate of 30 percent.

232. The tax structure for PAYE, **Mr. Speaker,** means that the tax burden falls on 57.6 percent of total employed workers. The tax structure is therefore regressive as an individual employee earning incomes above Ksh 32,333 per month falls within the upper tax band at the rate of 30 percent leaving no significant gap between employees who do not pay tax as a result of tax relief and those earning higher incomes. In this respect, we have to embark on the review of this taxation structure to make the tax bands more progressive sharing the tax burden more uniformly across income groups.

233. Mr. Speaker, as a start on this journey, I propose to the National Assembly to introduce two additional tax bands: a fourth band that will be applicable to employees earning incomes between Ksh 500,000 and Ksh 800,000 per month at the rate of 32.5 percent; and a fifth band that will be applicable to

employees earning incomes above Ksh 800,000 per month at the rate of 35 percent.

234. As a result of this change, the employees who will pay tax at 30 percent will be 19.5 percent while the two new tax bands will affect 26,676 employees who constitute 0.8 percent of total employed workers. **Mr. Speaker,** we shall continue to review that PAYE structure to make it more progressive.

Measures to Enhance Tax Administration

Amendments to the Tax Procedures Act, 2015

235. Mr. Speaker, currently, Kenya Revenue Authority has no visibility on the transactions of Trust largely due to lack of requirement to report to the KRA by Trustees. This makes it difficult to ascertain the tax payable on the income paid to beneficiaries of Trusts. In this regard, I propose to the National Assembly to amend the Tax Procedures Act to require all trustees administering trusts in Kenya to maintain and avail records required by the Authority.

236. Mr. Speaker, in order to enhance tax compliance by use of technology, I propose to the National Assembly to amend the

Tax Procedures Act to require taxpayers to issue electronically generated tax invoices which can be tracked and traced through an Electronic Tax Invoice Management System (eTIMS).

237. Mr. Speaker, in order to streamline the tax administration and remove pressure to abandon taxes and grant waivers of tax, I propose to the National Assembly to amend Tax Procedures Act to remove powers to abandon tax as well as waivers on penalties and interest that currently exist in the Act.

238. Mr. speaker, to encourage taxpayers to pay outstanding tax debts, I propose to the National Assembly to introduce a one-year tax amnesty on penalties and interest on the accrued tax debts up to 31st December 2022. In order to benefit from the amnesty, taxpayers will be expected to clear principal taxes between 1st September, 2023 and 30th June 2024. I urge taxpayers to take advantage of the one-year amnesty to clean their tax ledgers by paying all tax arrears.

239. Mr. Speaker, in order to enhance the power of the Commissioner General to collect taxes from non-compliant taxpayers using Agency Notices, I propose to the National

Assembly to amend Tax Procedures Act to expand the scope of Agency Notices to cover default in payment of instalment taxes and in situations where a taxpayer has made a self-assessment and submitted tax returns but has failed to pay the tax on due date.

240. Mr. Speaker, submission of manual tax information to the Commissioner General poses administrative challenges and delays decision making. I therefore propose to the National Assembly to amend the Tax Procedures Act to require the Commissioner General to provide a data management and reporting system that will allow taxpayers to electronically submit standardized transactional data on real-time basis.

241. Mr. Speaker, impersonation of Kenya Revenue Authority officers is an offence under the Penal Code and poses revenue risks. We have witnessed increased cases for impersonation of Kenya Revenue Authority officers. In order to curb this vice, I propose to the National Assembly to amend the Tax Procedures Act to provide for offences and sanctions relating to impersonation of Kenya Revenue Authority officers.

242. Mr. Speaker, in order to enhance efficiency in administration of tax refunds and boost cash-flow of businesses, I propose to the National Assembly to amend the Tax Procedures Act to allow taxpayers who overpay taxes to utilize overpaid tax to offset both outstanding tax debts and future tax liabilities. In addition, I propose to the National Assembly to amend the Tax Procedures Act to provide an additional 30 days for the determination of complex tax cases. This will provide more time for the Commissioner General to complete audit and investigation process for complex tax refunds cases.

243. Further, **Mr. Speaker,** I propose to the National Assembly to amend the Tax Procedures Act to require Kenya Revenue Authority to pay approved refunds within 6 months. In instances where approved refunds are not paid within this period, I propose to the National Assembly to further amend the Tax Procedures Act to provide for automatic offset of the approved refunds against outstanding tax liabilities in other tax heads or future tax liabilities.

Amendments to the Miscellaneous Fees and Levies Act, 2016

244. Mr. Speaker, let me now turn to the proposed amendments to the Miscellaneous Fees and Levies Act.

245. Mr. Speaker, as I had earlier stated, the Government is committed to lower the cost of Liquefied Petroleum Gas. In addition to removal of VAT on LPG, I propose to the National Assembly to remove Import Declaration Fees (IDF) and Railway Development Levy (RDL) on LPG.

246. Mr. Speaker, the Kenya Defence Forces and the National Police Service are exempted from Import Declaration Fees and Railway Development Levy in respect of equipment, machinery and motor vehicles for their official use. However, this does not extend to other goods for official use such as material supplies. In this regard, I propose to the National Assembly to extend the exemption to all goods purchased or imported for official use by the Kenya Defence Forces and the National Police Service.

247. Mr. Speaker, the aviation sector is critical in supporting tourism and transportation of passengers and cargo. Currently, some aircrafts and parts of the aircraft are exempt from Import

Declaration Fees (IDF) and Railway Development Levy (RDL). To further support this industry, I propose to the National Assembly to extend the exemption to cover all aircrafts, helicopters, aircraft engines and aircraft spare parts.

248. Mr. Speaker, currently, imported goods are subject to Import Declaration Fee at the rate of 3.5 percent and Railway Development Levy at the rate of 2 percent. On the other hand, raw materials and inputs imported by registered manufacturers are subjected to a lower rate of 1.5 percent for both Import Declaration Fee and Railway Development Levy. These differential rates pose administrative challenges and are avenues for revenue loss. In order to address these challenges, I propose to the National Assembly to harmonize these rates at 2.5 percent for Import Declaration Fee and 1.5 percent for Railway Development Levy applicable to all goods imported into the country.

Amendments to the Betting, Gaming and Lotteries Act, Cap. 131

249. Mr. Speaker, currently, the Kenya Revenue Authority is not able to enforce tax collection from betting, gaming, prize

competition and lotteries activities due to lack of an enforcement provision in the Betting, Gaming and Lotteries Act. In this regard, I propose to the National Assembly to amend this Act to allow KRA to apply the provisions of Tax Procedures Act in enforcing tax collection.

Amendments to the Employment Act

250. Mr. Speaker, in order to provide funds for development of affordable housing and associated social and physical infrastructure as well as the provision of affordable home financing strategy, I propose to the National Assembly to introduce an Affordable Housing Levy through an amendment to the Employment Act, 2007 to provide for a monthly levy payable by the employer and employee at one point five percent (1.5%) per month of an employee's gross monthly salary.

251. Mr. Speaker, given that construction is labour-intensive, we expect every housing unit constructed to create new jobs, both direct and indirect among the Professionals, Youth and MSMEs in general along the production value chain of affordable housing.

Support to Enterprises and Businesses operating in the Special Economic and Export Zones

252. Mr. Speaker, through Government support, the Special Economic Zones and the Export Processing Zones continue to position our manufacturing sector as pace setters in the region through exports of manufactured goods. Presently, **Mr. Speaker,** the goods sold from these zones to the domestic market are subjected to import duty and other applicable taxes similar to goods imported from outside the EAC region. This taxation does not differentiate whether the inputs or raw materials used to produce such goods originate from the Customs Territory or from outside the Customs Territory. In order to enhance market access, I propose to the National Assembly to amend the Export Processing Zones Act and the Special Economic Zones Act to exempt from import duty goods produced from the Special Economic Zones and Export Processing Zones that uses inputs or raw materials originating from the Customs Territory when sold into the domestic market. This will encourage enterprises operating in the zones to purchase raw materials or inputs from the domestic markets.

VII. CONCLUSION

253. In conclusion, **Mr. Speaker,** it is clear that we face many socio-economic challenges. The global recovery may take a long time before output gaps are closed. To address these challenges, the Government has initiated policy, legal and institutional reforms.

254. His Excellency the President has already led the way. Under his leadership, we have already laid a firm foundation for the Bottom - Up Economic Transformation Agenda. The policies spelt out in this budget builds a momentum for the envisaged transformation that will lead to improved livelihoods for all Kenyans. I therefore urge all Kenyans to join hands in making this transformation journey a reality.

255. Thankfully, our country is endowed with the necessary resources and human capital to confront the contemporary economic and social challenges and create a momentum for not only a higher and sustainable but also inclusive growth path, create decent jobs, reduce poverty significantly.

256. Mr. Speaker, this budget underpins the Government's resolve and commitment to fulfill its social contract to Kenyans by progressively improving their welfare.

257. At this point, **Mr. Speaker,** allow me to express my sincere gratitude to His Excellency Honourable Dr. William Samoei Ruto, The President of the Republic of Kenya and Commander-in-Chief of the Defence Forces and His Excellency the Deputy President Honurable Rigathi Gachagua for their counsel, guidance and support during the entire process of laying the foundation for the Bottom-Up Economic Transformation Agenda and in finalizing this Budget.

258. I also thank Her Excellency, Mrs. Rachael Ruto, The First Lady of the Republic of Kenya and Her Excellency, Pastor Dr. Dorcas Gachagua, the Second Lady of the Republic of Kenya for their support and contributions to the implementation of Government initiatives especially on environment and climate action, financial inclusion and women empowerment.

259. My special appreciation goes to the Prime Cabinet Secretary, the Attorney General, my fellow Cabinet Secretaries, respective

Principal Secretaries and Accounting Officers, and staff in all Government, Ministries, Departments and Agencies for their support and contributions to the FY 2023/24 budget making process.

260. I also extend my gratitude to the Chairman, of the Presidential Council of Economic Advisors, Dr. David Ndii and all the Economic Advisors under the Council for their critical inputs and guidance in supporting the Bottom-Up Economic Transformation Agenda. This is one team that used their diverse frontier knowledge and worked closely in developing the Kenya Kwanza Administration manifesto (The Plan) in the run up to the August 2022 General Elections under the guidance of His Excellency, The President and his team. More specifically, I gained a lot on fiscal matters from Dr. Kamau Thugge, Senior Adviser and Head of Fiscal Affairs and Budget Policy at the Executive Office of the President and currently the Governor Designate for the Central Bank of Kenya.

261. My sincere appreciation also goes:

- **First,** to you, **Honourable Speaker** of the National Assembly and your counterpart in the Senate, the Majority and Minority Leaders and the entire House Leadership including respective Clerks for overseeing the approval process of the budget estimates for FY 2023/24 and the related documents;

- **Second,** to Honourable Chairs and Members of the both the Budget and Appropriations Committee and the Finance and National Planning Committee and all the other Departmental Committees of this House as well as the staff of the Parliamentary Budget Office, for their constructive inputs during the approval process of this budget;

- **Third,** I recognize and appreciate the management and staff of the National Treasury and Economic Planning, who have worked tirelessly for long hours including weekends to ensure that this budget and supporting documents meet the legal deadlines;

- **Fourth,** the Kenya Revenue Authority; Central Bank of Kenya; Attorney General's Office, Commission on Revenue Allocation; Financial Sector Regulators and the various

Agencies under the National Treasury and Economic Planning for their contributions and advice during the budget process;

- **Fifth,** my gratitude goes to our Multilateral and Bilateral Development Partners for their continued technical and financial support. Further, I thank the private sector for its sustained contribution to the growth of our economy; and
- **Sixth,** I wish to appreciate the Media and non-state actors for their active engagement and participation in the FY 2023/24 budget process.

262. Mr. Speaker, I remain immensely grateful to my family for their support and inspiration as I execute my duties at the National Treasury and Economic Planning since my appointment to this docket last year.

263. Finally, **Mr. Speaker,** my utmost gratitude goes to all Kenyans for their contributions, proposals and suggestions received during the finalization of the budget proposals.

Thank You and May God Bless Kenya.

Pause

264. Mr. Speaker, before I resume my seat, you will recall that I have already submitted to this House the budget estimates and the Finance Bill, 2023, together with the accompanying documents as required by the Public Finance Management Act, 2012. Today, I further submit the following documents to this august House and request that you graciously receive them:

i) Budget Statement for the FY 2023/24;
ii) The 2023 Budget Policy Statement;
iii) Estimates of Revenue, Grants and Loans for the FY 2023/24 Budget;
iv) Financial Statement for the FY 2023/24 Budget;
v) Medium Term Debt Management Strategy 2023;
vi) Budget Highlights – The "Mwananchi" Guide for the FY 2023/24 Budget; and
vii) Statistical Annex to the Budget Statement for the FY 2023/24.

THANK YOU, HONOURABLE SPEAKER

____________________ o ____________________

THE NATIONAL TREASURY AND ECONOMIC PLANNING
JUNE 15, 2023

© Budget Statement 2023
The statement is available on: www.treasury.go.ke

REPUBLIC OF UGANDA
MINISTRY OF HEALTH

COMPENDIUM OF THE NATIONAL DIGITAL HEALTH GUIDELINES

SEPTEMBER 2024

Foreword

The compendium of the national digital health guidelines presents guidelines and standards necessary for the implementation of digital health within the health sector. These guidelines are intended to standardize the implementation of digital health across Uganda's health system. These guidelines are aligned with the Health Information and Digital Health Strategic Plan 2020/2021-2024-2025, National Community Health Strategy 2020/2021-2024-2025 and Ministry of Health Strategic Plan 2020/2021-2024-2025.

The Ministry of Health (MoH) is committed to improving the application of digital health technologies to facilitate the attainment of its overall objective of delivering high-quality health services to all citizens. This aligns with the call of Uganda Vision 2040 and the National Development Plan (NDP) III 2020/21 – 2024/25 which require sectors to adopt Information Communication Technologies (ICTs) to optimize service delivery.

This compendium, therefore, contains seven digital health guidelines namely; 1) Uganda Digital Health Enterprise Architecture, Standards and Knowledge Guidelines, 2) The Uganda Health Information Exchange and Interoperability Guidelines, 3) The Uganda Health Data Protection, Privacy and Confidentiality Guidelines, 4) The Uganda Health Data Access, Sharing and Use Guidelines, 5) Guidelines for the Introduction of Digital Health Solutions and Innovations in Uganda, 6) Guidelines for the implementation of the Electronic Medical Records System and 7) Guidelines for Implementation of Uganda's Electronic Community Health Information System (eCHIS).

These guidelines shall serve as a framework to ensure the proper governance, leadership and implementation of digital health within the country while ensuring its sustainability. All stakeholders are therefore called upon to adopt and use these guidelines that have been compiled into a compendium.

....

Dr. Henry G. Mwebesa

DIRECTOR GENERAL HEALTH SERVICE

REPUBLIC OF UGANDA
MINISTRY OF HEALTH

DIGITAL HEALTH
ENTERPRISE ARCHITECTURE, STANDARDS AND KNOWLEDGE PRODUCTS

A Guide to Standardising Digital Health in Uganda

TABLE OF CONTENTS

TABLE OF CONTENTS ii
LIST OF ACRONYMS iv
LIST OF FIGURES v
LIST OF TABLES vi
FOREWORD vii
PREFACE viii
EXECUTIVE SUMMARY x
INTRODUCTION 1
SECTION 1: DIGITAL HEALTH ENTERPRISE ARCHITECTURE 3
COMPONENT 1: DIGITAL HEALTH ARCHITECTURE VISION AND GOALS .. 4
COMPONENT 2: DIGITAL HEALTH ARCHITECTURE GOALS 6
COMPONENT 3: MoH INFORMATION MANAGEMENT PRINCIPLES 8
COMPONENT 4: ARCHITECTURE PROCESS 19
COMPONENT 5: DIGITAL HEALTH MONITORING & EVALUATION 28
COMPONENT 6: DH ENTERPRISE ARCHITECTURE GOVERNANCE MODEL 33
COMPONENT 7: DIGITAL HEALTH SECURITY ARCHITECTURE 43
Section 2: DIGITAL HEALTH STANDARDS FOR UGANDA 49
2.1. Data and Interoperability Standards 49
2.2 DIGITAL HEALTH Communication Infrastructure Standards 54
2.2.1. HARDWARE DEVICES STANDARDS 55
2.2.2. COMMUNICATION NETWORKS & CONNECTIVITY STANDARDS 58
2.2.3. APPLICATION, SOFTWARE AND TECHNOLOGIES STANDARDS 61
2.2.4. DHCI's FACILITATING RESOURCES STANDARDS 63
2.2.5. SECURITY AND PRIVACY STANDARDS 65
Section 3: DH WORKFORCE CAPACITY BUILDING FRAMEWORK 69
DH Competency Model 69
DHW JOB ROLES AND REQUIRED PROFICIENCIES 69
Proficiency Levels Required for Digital Health Workers (DHWs) in Uganda 69
CONCLUSION 71
REFERENCES 72
APPENDICES 75
APPENDIX A.1 STANDARDISATION FRAMEWORK 75
APPENDIX A.2: STANDARDISATION PROCESS 76
APPENDIX B.1: USE CASE – HIV/TB CLIENT MANAGEMENT SYSTEM ARCHITECTURE FOR IDI CLINIC, MULAGO 77

APPENDIX B.2: Use Case – Contextualising Terminology Codes for HIV Disease Management Services 84
APPENDIX C.1: DH Monitoring & Evaluation indicators and operationalisation tools 89
APPENDIX C.2: Results chain based on the results-based management strategy 93
APPENDIX C.3: Categorisation of DH Indicators 93

LIST OF ACRONYMS

DH	Digital Health
DH-ASK	Digital Health Architecture, Standards and Knowledge Products
DHEAF	Digital Health Enterprise Architecture Framework
DHIM	Division of Health Information Management
DHM&EF	Digital Health Monitoring and Evaluation Framework
eHealth	Electronic Health
HIE	Health Information Exchange
HIIRE	Health Information and Innovation Research
HIV	Human Immunodeficiency Virus
HSE	Health Sector Enterprise
IDI	Makerere University Infectious Disease Institute
ISO	International Organisation for Standardisation
ICT	Information and Communication Technology
ITU	International Telecommunications Union
LMIC	Low- and Middle-Income Country
MoH	Ministry of Health
MoICT&NG	Ministry of Information and Communication Technology, and National Guidance
NITA-U	National Information Technology Authority - Uganda
PEPFAR	U.S. President's Emergency Plan for AIDS Relief
SDG	Sustainable Development Goals
TWG	Technical Working Group
WHO	World Health Organisation

LIST OF FIGURES

Figure 1. Digital Health Enterprise Architecture Framework 3
Figure 2. Digital Health Enterprise Architecture Framework for Uganda 4
Figure 3. Digital Health Architecture Vision for Uganda 5
Figure 4. EA Requirements, Outcomes, and Goals Model 7
Figure 5. Architecture Process 20
Figure 6. Digital Health Evaluation Reference Model 29
Figure 7. Digital Health Governance Structure 33
Figure 8. Architecture Policy Development Process 39
Figure 9. Architecture Compliance Review Process 39
Figure 10. MoH Architecture Repository 43
Figure 11. Digital Health Information Security Architecture 44
Figure 12. Data Standards 49
Figure 13. Standards for DHCI 55
Figure 14. Framework for Standardisation of Uganda's Digital Health Interventions 76
Figure 15. Process of Selecting Suitable Standards for Digital Health 77
Figure 16: Goals, objectives, and requirements relationship diagram 79
Figure 17. Modelling Language for HIV/TB Client Management System Architecture 82
Figure 18. Symbols used in the CMS Architecture 82
Figure 19. Integrated HIV/TB Client Management System Model (Initial version) 83
Figure 20. Process of Contextualising Health Terminologies 84

LIST OF TABLES

Table 1. Digital Health Enterprise Architecture Goals for Uganda 7
Table 2. HSE Enterprise Principles 8
Table 3. MoH Business Architecture Principles 10
Table 4. MoH Data Architecture Principles 13
Table 5. MoH Application Architecture Principles 15
Table 6. Digital Health Technology Architecture Principles 16
Table 7. MoH Interoperability Principles 17
Table 8. MoH Security and Privacy Principles 17
Table 9. Inputs and Outputs of Preliminary Phase 20
Table 10. Inputs and Outputs of Architecture Vision Phase 21
Table 11. Inputs and Outputs of Business Architecture Phase 21
Table 12. Inputs and Outputs of Data Architecture Phase 22
Table 13. Inputs and Outputs of Application Architecture Phase 23
Table 14. Inputs and Outputs of Technology Phase 24
Table 15. Inputs and Outputs of Technology Phase 24
Table 16. Inputs and Outputs of the Migration Planning Phase 25
Table 17. Inputs and Outputs of Implementation Governance 26
Table 18. Inputs and Outputs of Architecture Change Management Phase 28
Table 19. Mapping Digital Health maturity stages with the results chain and Digital Health evaluation questions 30
Table 20. Example of Digital Health monitoring and evaluation reporting tool 32
Table 21. Proficiency Levels of DHWs (adopted from Alunyu et al., 2020) 71
Table 22. IDI EA Requirements, Outcomes and Goals 78
Table 23. Assumptions, Potential Risks and Mitigation Measures 79
Table 24. Provides the recommended coding standards that should be followed in the implementation of HIV/TB CMS. 81
Table 25. Recommended Semantic Standards to Support HIV/TB CMS 81
Table 26. Presents the guidelines for protecting and managing HIV/TB data. 81
Table 27. Use Case - Terminology Codes for HIV Disease Management Services 85

FOREWORD

The Government of Uganda recognises the use of Information and Communication Technology (ICT) in the National Development Plan III 2020/21 - 2024/25 as an enabler to improving the delivery of services to its citizens across its sectors. The Ministry of Health Strategic Plan 2020/21 – 2024/25 also recognises digital health as a key enabler for supporting the health system to deliver good health to the population. In addition, the Health Information and Digital Health Strategic Plan 2020/21 – 2024/25 guides how to use ICT to facilitate the improvement of health service provision in a bid to facilitate universal access to care, health sector efficiency, and social transformation.

The Government, Partners, and Private institutions have continued to invest in various digital health initiatives. However, there is a lack of standardization of these initiatives to address digital health challenges such as continued duplication, fragmented/siloed systems that cannot be integrated or scaled across the continuum of care, data duplication, and security and privacy of patient data.

Consequently, this standardisation guide presents several digital health artefacts that aim to standardise digitally-enabled healthcare and services in Uganda's health system including the Digital Health Enterprise Architecture that should define the "how and what" different digital health resources work together to optimise the delivery of healthcare and services. The digital health Standards also aim to provide common rules, principles, and guidelines for the use of various digital health interventions in Uganda's health system.

....

Dr. Henry G. Mwebesa

DIRECTOR GENERAL HEALTH SERVICES

PREFACE

The Ministry of Health acknowledges the potential of Information and Communication Technology (ICT) in transforming healthcare delivery by enabling information access and supporting healthcare operations, management, and decision-making. However, Uganda's health sector is characterized by a fragmented and siloed landscape of digital health interventions/pilot projects with significant barriers to effective sharing and exchange of health information within and across Uganda's health system.

The key obstacle to achieving this capability is the unavailability of health data standards for organising, representing, and encoding clinical data/information so that the data can be understood and accepted by the receiving systems. Lack of standards has also prevented the reuse of clinical data to meet the broad range of patient safety and quality reporting requirements.

Accordingly, this Standardisation Guide provides a set of digital health artefacts (DH-ASK) that should guide the standardisation of digitally-enabled healthcare and services to enhance health information management, and decision- and policy-making processes across the health sector.

The development of this standardisation guide was achieved through a participatory process spearheaded by Makerere University Health Informatics Research Group in collaboration with the Ministry of Health. We extend our profound appreciation to each of the contributors to these DH-ASK artefacts for playing a key role in standardising digital health to strengthen Uganda's health system.

....

Dr. Sarah Byakika

Commissioner Health Services

Department of Planning, Financing and Policy

ACKNOWLEDGEMENTS

The Ministry of Health expresses its profound gratitude to all Ministry of Health Departments and Programs, and members of the Health Information Innovation and Research Technical Working Group (HIIRE TWG) who provided technical inputs leading to the successful completion of this document.

Special appreciation goes to the Department of Planning, Financing and Policy for the overall guidance to ensure that the objectives of the Digital Health Architecture, Standards and Knowledge products (DH-ASK) are aligned to the priorities of the NDP III, MoH Strategic Plan 2020/21–2024/25, and the Health Information and Digital Health Strategic Plan 2020/21-2024/25.

Special gratitude is also extended to the Makerere University Health Informatics Research Group for leading and collaborating in the research and development of the DH-ASK products. Specifically, the Principal Investigator, Dr. Josephine Nabukenya, the Co-Investigators Dr. Andrew Kambugu and Dr. Mercy Rebekah Amiyo, the Researchers; Andrew Alunyu Egwar, Moses Bagyendera, Achilles Kiwanuka, Joseph Wamema and Justus Ashaba.

Finally, I would like to acknowledge the Government of the Republic of Uganda through the Makerere University Research and Innovation Fund (MakRIF) for sponsoring the research and development of the DH-ASK products. As well as thank all the development partners that provided financial and technical support for this process specifically HISP Uganda.

The Ministry of Health commits to the overall stewardship of these DH-ASK products through communication and dissemination, implementation, periodic monitoring and planned evaluations.

....

Mr. Paul Mbaka

Assistant Commissioner Health Services

Health Information Management

EXECUTIVE SUMMARY

The Standardisation Guide presents several digital health (DH) artefacts including; digital health Standards, digital health Enterprise Architecture Framework, digital health Workforce Capacity Building Framework, and digital health Monitoring and Evaluation Framework (DH-ASK). The DH-ASK artefacts will be used to standardise digitally-enabled healthcare and services in Uganda's health system.

Standardisation of the DH-ASK artefacts was derived from the requirements of an exploratory landscape study for digital health in Uganda's Health System. The landscape analysis provided an understanding of the challenges that impeded digital health standardisation and data-use-for-action in Uganda's health system. Whereas standardisation has been considered a remedy to digital health challenges in many countries; conversely, the digital health standardisation ground is troubled with numerous challenges, including a huge number of available standards, that compete, overlap and sometimes contradict one another; worse still, many of the available standards do not have implementation guidelines, which makes their implementation quite a difficult task. As such, adopting international digital health standards normally requires significant localisation to meet specific requirements of that country such as Uganda. Standardisation entails standards determination, implementation, compliance monitoring and review. Standardisation can be used to develop standards requirements for Uganda's digital health to address digital health challenges such as fragmentation/siloed (un-interoperable) systems, data duplication, security and privacy of patients' data.

The digital health Enterprise Architecture Framework aims to define the "how and what" different digital health resources work together to optimise the delivery of healthcare and services. The digital health Standards aim to provide common rules, principles, and guidelines for the use of various digital health interventions in Uganda's health system. The digital health Monitoring and Evaluation Framework aims to assess the results and impact of digital health interventions. The digital health Workforce Capacity Building Framework aims to define standardised digital health workers' skills, knowledge and appreciation of digital health in work practices.

INTRODUCTION

Standardisation has been considered as a remedy to digital health challenges in many countries (Adebesin et al., 2013; Adebesin & Kotzé, 2017; WHO & ITU, 2012). Conversely, the digital health standardisation ground is troubled with numerous challenges, including a huge number of available standards, that compete, overlap and sometimes contradict one another (Alunyu & Nabukenya, 2018; Hammond, 2017; WHO & ITU, 2012); worse still, many of the available standards do not have implementation guidelines, which makes their implementation quite a difficult task (Alunyu & Nabukenya, 2018; MoH-Uganda, 2016). As such, adopting international digital health standards normally requires significant localization to meet the specific requirements of a country such as Uganda. ***Standardisation*** entails; standards determination, implementation, compliance monitoring and review (Costello & Parker, 2020; ISO/IEC, 2019; Kim & Matney, 2018). Standardisation can be used to develop standards requirements for Uganda's digital health to address digital health challenges such as fragmentation/siloed (un-interoperable) systems, data duplication, security and privacy of patients' data.

Standardising digital health in Uganda was motivated by an exploratory landscape analysis of the digital health in Uganda's Health System (Alunyu et al., 2021; Kiwanuka et al., 2021; MoH-Uganda, 2021), which provided an understanding of the challenges that impeded digital health standardisation and data-use-for-action in Uganda's health system. The exploratory study used the HIV disease model to provide highlights of the state of the art for digital health conducted in Uganda on grounds that several global and local interventions (i.e.; Uganda Government and Development Partners) as well as enormous investments had been made to control the epidemic (Ministry of Health-Uganda, 2010). Notably, PEPfAR sponsors 80% of the resources for Uganda's HIV response, which places great emphasis on the use of data-driven approaches to facilitate decision-making ensuring appropriate interventions are implemented in relevant populations in the right way (PEPfAR, 2019).

Principally, various, though duplicated and disintegrated data-driven digital health interventions have been developed in Uganda to manage the HIV epidemic. As such, patients' health data continues to be fragmented, unsecure and lacks privacy due to sitting on several siloed/un-interoperable digital health systems. This has greatly affected ease-of-use for such health data to inform patient care, clinical decision-making and policy-making. Worse still, the digital health interventions that have been successful in one healthcare program, are most times not integrate-able with others nor adopted by other similar programs within and across the same health system (i.e., these interventions may be useful in a specific health program).

In other words, their integration into a coherent national integrated health information system for Uganda is currently challenging.

Accordingly, achieving a coherent National Integrated Digital Health Information System for Uganda would require standardisation for all digitally-enabled healthcare interventions; this is to facilitate their proper implementations to support health information exchange (HIE) within and across Uganda's health system. To this end, the contents of this Standardisation Guide report on several digital health (DH) artefacts including; digital health standards, digital health Enterprise Architecture Framework, digital health Worker Capacity Building Framework, and the digital health Monitoring and Evaluation Framework (DH-ASK). The DH-ASK artefacts aim to standardise digitally-enabled healthcare and services in Uganda's health system as stipulated in Uganda's Health Information and Digital Health Strategy (2020/21-2024/25), other aligned policies/plans including Uganda's National Development Plan III (2020/21–2024/25), Uganda Vision-2040; Health Sector Development Plan (2020/21–2024/25) on digital health development and implementation in Uganda; WHO global strategy on digital health (2020-2024) where standardising digital health is among the primary health systems strengthening building blocks; UN-SDG 3 to "ensure healthy lives and promote well-being for all at all ages", one of its targets being elimination of epidemics related to infectious diseases.

SECTION 1: DIGITAL HEALTH ENTERPRISE ARCHITECTURE

Enterprise Architecture (EA) is a description of an enterprise from an integrated business and IT perspective intended to bridge the communication gap between business and IT stakeholders and improve business and IT alignment (Kotusev, 2019; van de Wetering et al., 2021). This integrated view identifies and communicates the necessary improvements to business processes and IT assets to optimise an organisation's mission capabilities and resource utilization. EA, therefore, translates an organisation's vision and strategy into effective enterprise change by creating, communicating, and improving the key requirements, principles, and models that describe the enterprise's future state.

Figure 1 presents the Digital Health Enterprise Architecture Framework (DHEAF) for Uganda. This framework is intended to standardise the implementation of Digital Health across Uganda's health system by optimizing and transforming the often-fragmented processes, information, application systems, and technologies into an efficient and integrated environment supportive of Uganda's Health Information and Digital Health (HIDH) Strategy. The DHEAF, which comprises several components that work together, has been built on the foundations of the Uganda National eHealth Strategy 2017-2021 (MoH-Uganda, 2016) and the Uganda HIDH Strategy 2020/21-2024/25. It is aligned with the Uganda e-Government Interoperability Framework (NITA-U, 2021), Uganda e-Government Interoperability Reference Architecture (GoU, 2021a), and Government of Uganda e-Government Web Application Security Architecture Framework (GoU, 2021b). This approach supports the alignment of the Ministry of Health's Digital Health initiatives with the digital strategies of the Government of Uganda.

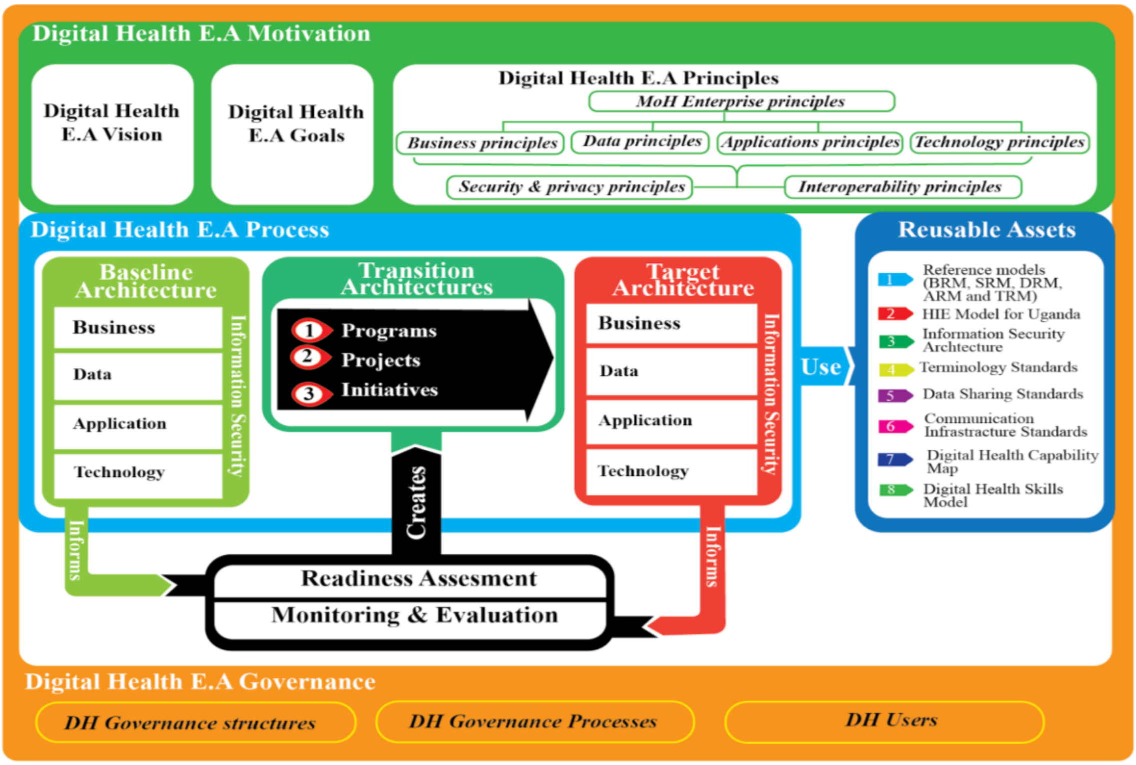

Figure 1. Digital Health Enterprise Architecture Framework for Uganda

The Digital Health Enterprise Architecture Framework (DHEAF) for Uganda is anchored on The Open Group Architecture Framework (TOGAF) version 9.2 and uses the semantic model for systems architecture defined in IEEE 1471-2000 standard. The DHEAF, incorporates best practices from several other popular Enterprise Architecture frameworks and standards, including; the Treasury Enterprise Architecture Framework (TEAF), California Enterprise Architecture Framework, Open Health Information Exchange Framework, and publications from the National Institute of Standards and Technology (NIST), Massachusetts Institute of Technology (MIT), National E-Health Transition Authority Limited (NEHTA) and Gartner.

DESCRIPTION OF DHEAF COMPONENTS

The DHEAF constitutes of seven building blocks/components that have to support each other to realise a standardised Digital Health environment where all stakeholders can seamlessly share health information. These building blocks/components are intended to reduce duplication, redundancies, and complexity as well as to promote shared Digital Health solutions while emphasizing the security and privacy of health data.

COMPONENT 1: DIGITAL HEALTH ARCHITECTURE VISION AND GOALS

Digital Health Architecture Vision

An integrated foundation for Digital Health that ensures access to healthcare in a secure, transparent, efficient, and cost-effective manner in Uganda.

Description of EA Vision

Figure 2 and **Figure 3** illustrate the Digital Health Architecture Vision for Uganda. This vision describes the dream for Uganda's health system target architecture, it is intended to create an integrated foundation for Digital Health that will ensure accessibility of healthcare in a secure, transparent, efficient, and cost-effective manner. It will serve as a model for all Health organisations that constitute Uganda's health system and will be a crucial input in all initiatives to create target architectures.

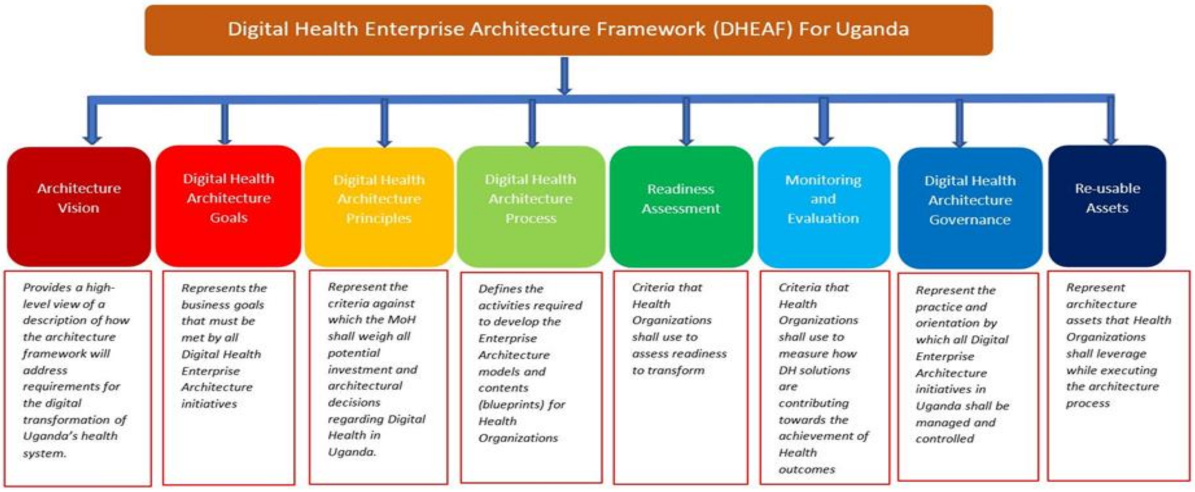

Figure 2. Description of the Eight Major Components of the Digital Health Enterprise Architecture Framework for Uganda

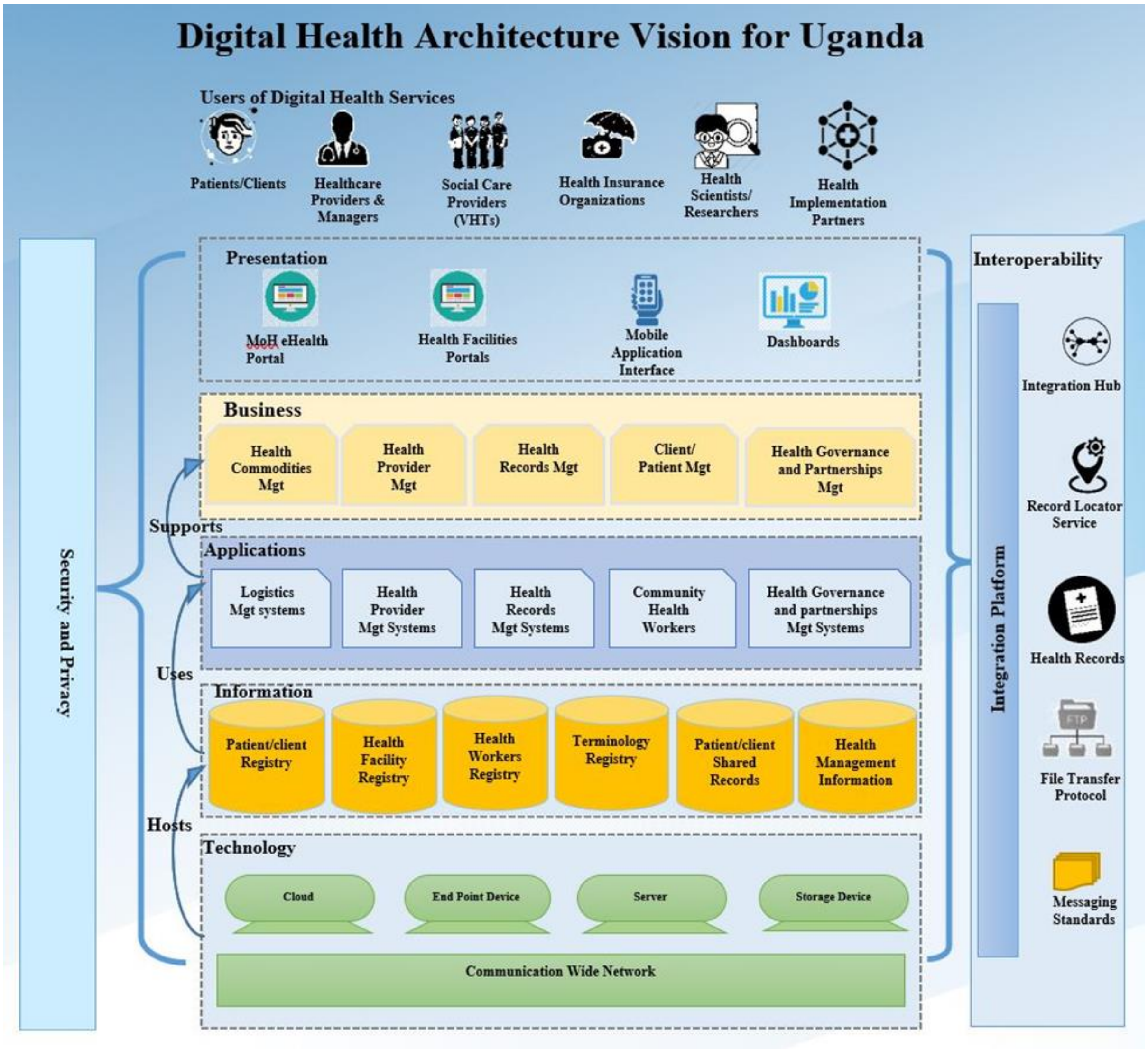

Figure 3. Digital Health Architecture Vision for Uganda (conceptualized based on TOGAF)

Digital Health users are categorised into six groups (including private healthcare organisations) and the supply chain as described hereunder:

- Patients/Clients – these are individuals that access health services
- Health Practitioners and Managers – these are individuals who are actively involved in either providing, supplying or overseeing the provision of health services
- Social Care Practitioners (CHWs) – these are individuals who deliver health support from the community to the health facility
- Health Insurance Organisations – these are organisations financing healthcare delivery through provision of insurance services
- Health Researchers – these are individuals and organisations involved in systematic investigations in the health sector
- Health Implementing Partners – these are organisations that support the Government of Uganda in the implementation of health programs.

These users will access Digital Health services from the Ministry of Health (MoH) Portal, Health facility portal, Mobile Application Interfaces, and Dashboards. Health organisations shall be allowed to implement five major categories of Health Information Systems; Logistics Management systems, Health Provider Management systems, Health Records Management Systems, Community Health Worker systems, and Health Governance and Partnership Management systems. These systems shall be interoperable with Uganda's National Health Information Systems, such as DHIS2. To facilitate this, the DHEAF defines standards for integration. All Digital Health systems shall be required to conform to the standards before they are allowed to integrate with other health systems. Integration shall be through an interoperability platform.

The Integration Platform shall provide a single logical gateway into the core of the National Health Information System and standard-based channels for synchronous and asynchronous messaging between local Health Information Systems and the National Health Information System. Critical functions of the integration platform shall include security, messaging, and auditing. The MoH shall implement an integration platform through several specialized components, including; an Integration Hub, Resource Locator Service (RLS), File Transfer Protocol (FTP), and messaging standards.

This architecture vision also depicts five registries to support the management of health data, including; the Patient/Client, Health Facility, Health Worker, Terminology, Health Shared Record, and Health Products Management registries. In addition, datasets shall be normalised, integrated, and held centrally where necessary to support business capabilities. The detailed contents of these shall be driven by the information architecture and the detailed design of core applications and core services, the definition of which will continue to evolve and mature as the new Knowledge & Information capabilities are implemented. MoH shall host all Digital Health applications and registries, endpoint devices, servers, and other storage devices as may be necessary. These will be linked together via a wide communication network.

To ensure the confidentiality, integrity, and availability of health data, Health organisations shall incorporate security governance in their everyday practices to effectively cope with the emerging security and privacy threats to health information. As such, security and privacy considerations must be factored into all the layers of the Uganda Health System, right from the technology layer to the application, data, and business layers. Security Governance must be integrated into Corporate Governance and regarded as a governance challenge that includes reporting accountability, and adequate risk management.

COMPONENT 2: DIGITAL HEALTH ARCHITECTURE GOALS

The following business goals shall guide the implementation of the DHEAF for Uganda's Health System (See **Table 1**).

Table 1. Digital Health Enterprise Architecture Goals for Uganda

**Business Goal**	**Notes**
Business Goal 1	Harnessing Digital Health to facilitate transformation of Uganda's healthcare system and improve health outcomes
Business Goal 2	Making patient care safer and more effective by making available the correct information in the right place at the right time
Business Goal 3	To ensure equitable access to quality health services, with emphasis on improving access to health services by the underserved and vulnerable populations
Business Goal 4	To standardise the use of ICT in healthcare delivery to ensure interoperability of systems and timely access to health-related information
Business Goal 5	To safeguard confidentiality, privacy, and integrity of patient/client information

DIGITAL HEALTH ENTERPRISE ARCHITECTURE REQUIREMENTS, OUTCOMES, AND GOALS RELATIONSHIPS

Figure 4 shows the relationships that exist between the Enterprise Architecture requirements, outcomes, and goals model.

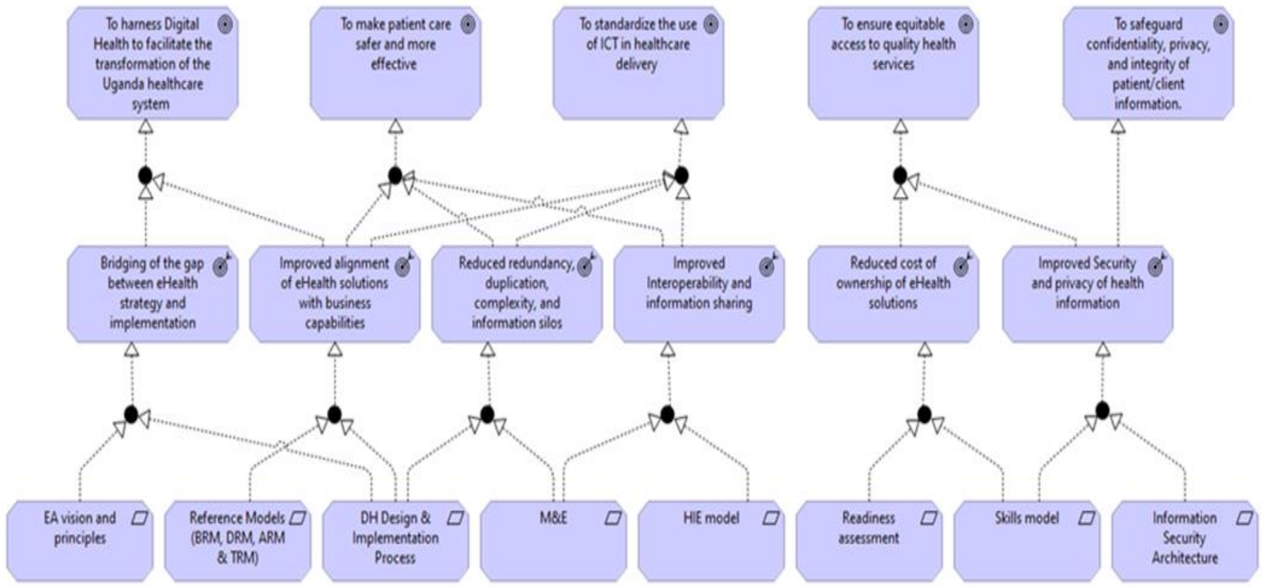

Figure 4. EA Requirements, Outcomes, and Goals Model

COMPONENT 3: MoH INFORMATION MANAGEMENT PRINCIPLES

Principles are general rules and guidelines intended to be enduring and seldom amended that inform and support how an organisation fulfils its mission (Josey et al., 2018). Principles may be established within different domains and at different levels of an enterprise. This section presents the MoH Information Management principles that shall guide the implementation and use of Digital Health in Uganda. These principles are based on best practices from TOGAF, MIT, and NEHTA (Josey et al., 2018; NEHTA, 2012) and have been informed by:

- Health Information and Digital Health Strategic Plan (2020/21-2024/25)
- Uganda National eHealth Strategy (2017-2021)
- MoH Handbook on "Strengthening Uganda's Health System through Standardising Digital Health: Requirements for Digital Health Standards and Enterprise Architecture Framework" (2021)
- Legal and Regulatory Framework for Digital Health in Uganda
- Accepted architectural practices in the Information Technology industry

The MoH Information Management Principles are structured according to The Open Group Architecture Framework (TOGAF) recommendations. Each principle has a *Name* that highlights the essence of the principle, *a Statement* that unambiguously communicates the fundamental rule, a *rationale* that gives the business benefit of adhering to the principle, and *implications* that outline the critical tasks, resources, and potential costs associated with following the principle. The main objective of the MoH Information Management Principles is to support the delivery of Digital Health solutions that are interoperable, flexible, and fit for purpose while supporting established rules and processes for communication and the use of information within Uganda's health sector. These principles are divided into two, namely; enterprise principles and architecture principles.

Component 3A: MoH Enterprise Principles

Enterprise principles are general rules that provide a basis for decision-making throughout an enterprise and dictate how the organisation intends to fulfil its mission (Josey et al., 2018). Such principles are commonly used to harmonise decision-making at the strategic level and are a crucial element of the governance strategy. The four enterprise principles presented in **Table 2** shall guide decision-making at the strategic level of Uganda's Health System. Each principle has a code that reflects the category (i.e., Health Sector Enterprise (*HSE*) and the principle number (i.e., P1).

Table 2. HSE Enterprise Principles

CODE	HSE-P1
NAME	**Uganda's health sector is an integrated and dynamic enterprise**
STATEMENT	Uganda's health sector operates as a single enterprise with decision-making flexibility at National and sub-national levels

**RATIONALE**	A single enterprise with shared strategic objectives, joint governance, integrated management processes, and consistent policies improves the implementation of enterprise-wide strategies and the coordination of the delivery of health services to citizens
**IMPLICATION**	• Optimise resource allocations to achieve common goals across the entire health sector<br>• Ensure availability and access to health information for evidence-based decision-making across the health sector<br>• Digital Health architectural designs should integrate services for efficiency while keeping the autonomy of operations of the different units of the health sector
**CODE**	**HSE-P2**
**NAME**	**Improve the safety and quality of healthcare for citizens**
**STATEMENT**	Decisions about Digital Health must aim to improve the safety and quality of healthcare
**RATIONALE**	Providing safe, high-quality, and effective healthcare is a national priority. Digital Health investment decisions should support this priority and thus minimise healthcare risks associated with inaccurate and inadequate healthcare information and processes.
**IMPLICATION**	• Apply applicable Institute of Medicine (IOM) quality principles of care to all decisions about Digital Health investments; that is, healthcare should be safe, effective, patient-centred, timely, and unbiased<br>• Decisions about Digital Health investments should be driven by the need for safe, high-quality, effective healthcare rather than by technological or other external concerns
**CODE**	**HSE-P3**
**NAME**	**Improve the efficiency of healthcare services**
**STATEMENT**	Decisions about Digital Health investments should aim to improve the efficiency of healthcare service provision
**RATIONALE**	The provision of efficient healthcare is a national priority. Digital Health investment decisions should support this priority and thus improve the healthcare delivery processes.
**IMPLICATION**	• Decisions about Digital Health investments should be driven by the need for cost-efficient healthcare delivery rather than by technological or other external concerns<br>• The applicable Institute of Medicine (IOM) quality of care principles should be applied to such decisions; that is, healthcare should be efficient
**CODE**	**HSE-P4**
**NAME**	**Security, privacy, and protection of clients' health information are core needs of Uganda's health system**
**STATEMENT**	Security, privacy, and protection of clients' health information are integral to Uganda's health system's operations and are part of Digital Health innovations. Therefore, all citizens' health information should be protected against unauthorised access and intentional and accidental modifications.
**RATIONALE**	Protection of clients' confidential health information will increase public trust in Digital Health and improve their utilization to deliver healthcare services
**IMPLICATION**	• The business context should define security and privacy requirements to be integrated into the entire Digital Health ecosystem<br>• Digital Health systems should embed national policies to minimise improper data use and security violations<br>• Apply security and privacy information management policies and monitor compliance<br>• Information security controls should be clearly defined to balance costs and mitigate risks

Component 3B: MoH Digital Health Architecture Principles

Architecture principles are a set of principles that relate to architectural work. They reflect consensus across the enterprise and embody the spirit of Enterprise Architecture. Architecture Principles govern the architecture process, affecting the Enterprise Architecture's development, maintenance, and use (Josey et al., 2018). This section provides the Digital Health architecture principles for Uganda's health system. These principles shall support the health sector in delivering Digital Health solutions that are interoperable, flexible, and fit-for-purpose and use while supporting established guidelines, policies, and processes for communication and use of information within Uganda's health system. They are not only expected to act as drivers for defining functional requirements for the architecture but will also be used as a guide when assessing Digital Health innovations.

The Digital Health Architecture principles listed in **Table 3 – Table 8** are categorised under four architecture domains: business, data, application, and technology. They comprise five *Business architecture (B) principles*, six *Data architecture (D) principles*, three *Application architecture (A) principles*, and two *technology architecture (T) principles, five Security and Privacy Principles (SP), and two Interoperability (I) principles* as discussed in the subsequent sub-sections. Each principle has a code, statement, rationale, and implication. The principal code (code) represents the type or category of principle (i.e., *Business architecture (B)*, data *architecture (D)*, *application architecture (A)* and *technology architecture (T)*, *Interoperability (I)*, *Security and Privacy (S)*, and the principle number (e.g., P1).

Table 3. MoH Business Architecture Principles

**CODE**	**B-P1**
**NAME**	**Adhere to the Architecture Principles**
**STATEMENT**	The architectural principles defined apply to all Health organisations within Uganda's healthcare systems
**RATIONALE**	• The effectiveness of a Digital Health Architecture in providing a consistent and measurable level of quality information to decision-makers depends upon all Health organisations abiding by the principles upon which that architecture was designed<br>• Without this principle, inconsistency, exclusion, and favoritism would rapidly undermine the information management required to achieve Uganda's health sector's strategic direction<br>• These principles shall guide the selection, creation, and implementation of Digital Health and provide a workable transition path to targeted architecture, maintain flexibility and enhance interoperability
**IMPLICATION**	• All Digital Health architecture developments and new solutions should align with the principles<br>• Digital Health solution owners and or leads should ensure that solutions comply with the principles
**CODE**	**B-P2**
**NAME**	**Engage with all relevant Digital Health stakeholders**
**STATEMENT**	Developers of Digital Health architecture designs, standards, and solutions must collaborate with all stakeholders
**RATIONALE**	The Digital Health ecosystem for Uganda involves a diverse and vast community of stakeholders. Therefore, an inclusive and participatory development approach is required to address the collective

	set of stakeholder requirements. Using a participatory approach provides the most significant probability of achieving a successful and acceptable Digital Health solution outcome.
**IMPLICATION**	• Digital Health stakeholders within a community should be allowed to express their opinions when submitting requirements and providing feedback to developers and implementers<br>• Sustained, ongoing engagement will be required to ensure collective appreciation and buy-in of architectural decisions
**CODE**	**B-P3**
**NAME**	**Ensure Digital Health solutions are fit-for-purpose**
**STATEMENT**	All Digital Health architecture decisions should consider the business constraints and requirements of the healthcare community
**RATIONALE**	• The adoption and uptake of Digital Health is dependent on:<br>a) how well implementers of Digital Health have practically implemented and integrated it into current healthcare community practices<br>b) the extent to which these result into overall healthcare outcomes and process improvement
**IMPLICATION**	• Digital Health Implementing Partners must support requirements management at all stages of architecture development, standards specifications, system implementation, system operations, and change management<br>• Benefits realised from all Digital Health interventions must be measurable<br>• Implementers should consider the usability of Digital Health systems when designing Digital Health solutions interventions
**CODE**	**B-P4**
**NAME**	**Support services-based approaches**
**STATEMENT**	Implementers must apply a service-oriented approach when developing Digital Health solutions
**RATIONALE**	• Service orientation delivers agility<br>• Business services are the fundamental mechanism for sharing information and are essential building blocks for building interoperable Digital Health systems. One or more application services can support a business service.
**IMPLICATION**	• Identify standard services that the other systems can re-use<br>• Business services identify information artefacts associated with service provision and use. Therefore, information models must be related to business services to determine the benefit to the health organisation.
**CODE**	**B-P5**
**NAME**	**Assess the Total Cost of Ownership of Digital Health solutions**
**STATEMENT**	The deployment of a new Digital Health capability should be based on an assessment of its business and social value as well as the implementation and operational costs
**RATIONALE**	New Digital Health capabilities are expected to support better healthcare; however, this would involve both technology and organisational change costs. Therefore, the operational cost of a solution must be identified and contained to ensure that the ongoing operation of the solution is feasible and viable.
**IMPLICATION**	• When making decisions to create a new Digital Health capability, stakeholders must consider the total cost of developing and maintaining the Digital Health solution<br>• Digital Health solutions should be acquired or developed, deployed, replaced and decommissioned at the least cost while ensuring fitness for purpose of an overall system<br>• Operational procedures and their likely cost must be identified early in selecting and developing a Digital Health solution

	• The deployment, migration, and cutover strategy for any Digital Health solution must be identified in assessing operational costs
**CODE**	**B-P6#**
**NAME**	**Ensure Supportability, Sustainability, and Continuity**
**STATEMENT**	Digital Health solutions should be supportable, sustainable, and should be able to provide the required degree of business continuity necessary for their operations
**RATIONALE**	• The reliability of the Digital Health solutions must be considered throughout their design and use. If a Digital Health solution is to be adopted and embraced, it needs to be readily supportable, sustainable, and provide a business continuity of operation exceeding routine expectations<br>• Users must be provided with the capability to continue business operations regardless of the failure of a Digital Health solutions<br>• Hardware/software failure and data corruption should not be allowed to disrupt the delivery of healthcare and services
**IMPLICATION**	• Dependency on Digital Health mandates that the risks of healthcare delivery interruption must be established in advance and managed. Periodic reviews of the Digital Health solutions should be carried out to assure healthcare continuity.<br>• Recoverability, redundancy, and maintainability should be addressed at the design time.<br>• All Digital Health solutions that are critical in healthcare delivery must be assessed to determine what level of continuity is required and the necessary recovery plan.
**CODE**	**B-P7**
**NAME**	**Support Use of Enterprise-wide Digital Health Solutions**
**STATEMENT**	The development of Digital Health solutions that are usable across Uganda's health ecosystem is preferred over the development of similar or duplicative applications which support a single Health organisation or disease domain
**RATIONALE**	Duplicative capability is expensive and proliferates conflicting data
**IMPLICATION**	• Health organisations which depend on a Digital Health solution that does not serve the entire health continuum must change over to health sector-wide solutions. This will require the establishment of an adherence policy.<br>• Health organisations shall not be allowed to develop Digital Health solutions that are similar/duplicative to health sector-wide solutions. In this way, expenditures of resources to build duplicate Digital Health solutions will be reduced.
**CODE**	**B-P8**
**NAME**	**Comply with Laws, Policies, and Regulations**
**STATEMENT**	Digital Health solutions and infrastructure should comply with applicable legislation and policies in all jurisdictions and organisations within which they operate
**RATIONALE**	• Digital Health systems should comply with all applicable legislation, regulation, and standards. Compliance is key to maintaining stakeholder trust (Quality assurance) in the services that Digital Health solutions provide.
**IMPLICATION**	• Define applicable legislative and policy requirements for all Digital Health solutions and infrastructure; otherwise, there is a risk of non-compliance. These requirements should be kept up to date to reflect changes in legislation and policy<br>• Digital Health developers and implementers must be compliant with laws, regulations, and external policies regarding the collection, retention, management and dissemination of health data

	• Particular attention needs to be given to Uganda's Data Protection and Privacy Act 2019 and the Data Protection and Privacy Regulations, 2020 which regulate how organisations collect, use, disclose and secure personal information and provide individuals with rights of access and correction. All health service providers must comply with the Data Protection and Privacy Act 2019 and any other laws that relate to data security including the National Information Security Policy. The National Information Policy outlines the minimum security controls that all public and private organisations must meet<br>• Rather than prescribing implementation mechanisms, policy requirements should be expressed in terms of obligations, permissions, prohibitions, outcomes, and performance requirements

Table 4. MoH Data Architecture Principles

**CODE**	**D-P1**
**NAME**	**Manage Health Data as an Asset**
**STATEMENT**	Health data must be managed so that its source and the times and places it is created, changed, updated, accessed, and ultimately disposed of are captured and retained.
**RATIONALE**	Health data is the foundation of decision-making within healthcare, so it must be carefully managed to ensure that we know where it is, rely upon its accuracy, and obtain it when and where we need it. Effective health data management ensures evidence-based decision-making & improved healthcare outcomes. Organizing and managing the critical data assets drives the business processes required to support healthcare service delivery.
**IMPLICATION**	• Health data components should always identify the time and place of information creation and change. Versioning and metadata are one way of capturing this.<br>• Services providing sharing or access to information may keep only the most recent or most accurate version but must acknowledge the existence of preceding versions.<br>• Uganda's health system must have a cultural transformation from "data ownership" thinking to "data stewardship" thinking.<br>• Data stewards must have the authority and means to manage the data they are accountable for. Part of the role of the data steward is to ensure data quality.<br>• Procedures must be developed and used to prevent and correct errors in Digital Health solutions.<br>• Steps to ensure the quality of data held by the Digital Health solutions should be implemented.
**CODE**	**D-P2**
**NAME**	**Ensure Data Consistency in Distributed Environments**
**STATEMENT**	A distributed Digital Health environment requires explicit support for ensuring consistency and completeness of data originating from multiple sources.
**RATIONALE**	Digital Health components and services typically span organisational and geographic boundaries affecting the reliability, availability, and performance of data processing. Atomic transactions cannot generally address the scalability, autonomy, and robustness issues in such an environment, so a process-centric approach to consistency must be adopted. This is particularly important for sporadically connected systems and long-running transactions or processes.
**IMPLICATION**	• The process for establishing consistency of information should be explicitly defined for service usage scenarios.<br>• Atomic transaction mechanisms may be used across services, but an alternate mechanism should always be provided to achieve consistency through discrete steps when information crosses organisational boundaries.

	● When developing processes, the needs of sporadically connected participants must be considered to ensure consistency.<br>● Transactional messaging is a valuable and robust mechanism that can support consistency processes but is not generally sufficient; a consistency process definition is still required.<br>● The time and place attributes of an information component (see D.P1) can be used to help establish consistency.
**CODE**	**D-P3**
**NAME**	**Ensure Data Sharing across Uganda's Health System**
**STATEMENT**	Data held by Digital Health solutions should be shareable across Health organisations. Users of Digital Health should have access to the necessary shared data required to perform their respective business functions. In addition, shared data should be centrally controlled and managed.
**RATIONALE**	· Electronically shared data will increase efficiency as Health organisations can use it, without rekeying, to create new entities.<br>· Users will rely on official sources of more accurate and timely managed data, thereby improving decision-making.
**IMPLICATION**	A common set of standards, policies, and procedures that users should adhere to should be developed to enable data sharing among Health organisations.<br>● Standard data models, data elements, and other metadata that define the shared environment should be developed and stored in a repository for easy access.<br>● This principle of data sharing held by Digital Health Solutions will continually "bump up against" the principle of data security. However, under no circumstances will the data sharing principle result in confidential data held by the Digital Health systems being compromised.<br>● All users will have to rely on electronic data made available for sharing to execute their respective tasks. This will ensure that only the most accurate and timely data is relied upon for decision-making.
**CODE**	**D-P4**
**NAME**	**Ensure Data Ownership and Responsibility**
**STATEMENT**	● Health organisations must own each data entity held by Digital Health systems. Every Health organisation should be responsible for the integrity and security of its data. Therefore, each data entity held by Digital Health Systems must have an owner.<br>● *Primary Data Source*: All health data should have an authoritative, official, primary data source, which should be the location for all "create", "update" and "delete" actions but this should be in line with data management guidelines of the health sector.
**RATIONALE**	● The lack of well-defined data ownership may lead to confusion about who can change the data held by the Digital Health systems. Identifying the Health organisation's ownership of its respective data entities avoids ambiguity and creates clear responsibility and accountability for all data.<br>● Identifying the data owners will clearly define the point of contact in the respective unit responsible and accountable for all changes in the data entities held by the Digital Health systems.<br>● For data to be managed effectively, there can be only one primary source for each data entity. This will enable a data entity to be traceable back to the source. Otherwise, inconsistent, erroneous, and out-of-date data may result.<br>● Data integrity is at its highest level when data changes are centrally done.
**IMPLICATION**	● The data owner shall be responsible for meeting quality requirements levied upon the data.<br>● Health data shall be captured once and immediately validated at the source. In addition, quality control measures must be implemented to ensure the integrity of the data.
**CODE**	**D-P5**

**NAME**	**Use Common Terminologies and Data Definitions**
**STATEMENT**	• There should be a standard, common and consistent definition of all data held by the Digital Health systems, which should be understandable and available to all users.<br>• Data held by the Digital Health systems should be defined to ensure seamless interoperability.
**RATIONALE**	• Electronic data to be exchanged across the health sector and should have a standard definition with an agreed format and meaning of the data items. A common vocabulary facilitates effective communication and enables sharing of data.<br>• Electronic data sharing also enhances metadata modelling, consistency, and quality<br>• Centralized metadata provides a single point for maintaining the metadata.
**IMPLICATION**	• Common terminologies for the services must be established. The definitions shall be used uniformly throughout the Digital Health solutions.<br>• Ambiguities resulting from multiple parochial definitions of data must give way to accepted health sector-wide definitions and understanding<br>• The MoH shall coordinate all the data standardisation initiatives

Table 5. MoH Application Architecture Principles

**CODE**	**A-P1**
**NAME**	**Promote the development of modular and component-based Digital Health solutions**
**STATEMENT**	• Digital Health solutions should be modular and component-based, aligned to business processes.<br>• They should also conform to established open standards with well-defined roles & responsibilities.<br>• Components should be independent of the physical topology of the system and scalable.
**RATIONALE**	Reduces total cost of ownership and avoids vendor lock-in
**IMPLICATION**	· Avoid proprietary solutions and technologies if possible.<br>· Consider use of the latest web services, XML and integration standards in line with GoU eGovernment Web applications security (WASA) framework.<br>· Internet-based web standards and technology should be preferred for all solutions.
**CODE**	**A-P2**
**NAME**	**Ensure ease of use of solution**
**STATEMENT**	Digital Health solutions should be easy to use. Developers of Digital Health solutions must ensure that the underlying technology is transparent to users. This will allow users to concentrate on the tasks at hand.
**RATIONALE**	• The more a user has to understand the underlying technology, the less productive that user is. Ease of use is a positive incentive for the use of a Digital Health system.<br>• It encourages users to work within the integrated information environment instead of developing isolated systems to accomplish tasks. Training shall be kept to a minimum, and the risk of misusing a Digital Health solution will be reduced.
**IMPLICATION**	• Digital Health applications shall be required to have a common "look and feel." Hence, the common look and feel standard must be designed, and usability test criteria must be developed.<br>• Guidelines for user interfaces should not be constrained by narrow assumptions about user location, language, systems training, or physical capability. Instead, factors such as linguistics,

	customer physical infirmities (visual acuity, ability to use keyboard/mouse), and proficiency in using technology have broad ramifications in determining the ease of use of an application.<br>• The Digital Health solutions should define and communicate the right of access and use.
**CODE**	**A-P3**
**NAME**	**Ensure technology independence of solutions**
**STATEMENT**	Digital Health solutions should be independent of specific technology choices. They should be able to operate on a variety of technology platforms.
**RATIONALE**	The independence of applications from the underlying technology allows applications to be developed, upgraded, and operated in the most cost-effective and timely way.
**IMPLICATION**	• Developers should adopt standards that support portability while developing Digital Health solutions.<br>• Subsystem interfaces should be developed to enable existing systems to interoperate with the new Digital Health system.<br>• Developers are encouraged to use programming software, which gives a high degree of priority to platform independence.

Table 6. Digital Health Technology Architecture Principles

**CODE**	**T-P1**
**NAME**	**Embrace Requirements-Based Change**
**STATEMENT**	Changes to Digital Health technology infrastructure should be made in response to the business needs of the health organisation.
**RATIONALE**	This principle will foster an atmosphere where the Digital Health technology infrastructure changes in response to the business needs and requirements of the health organisations, rather than having it change in response to IT changes.<br>This shall ensure that business drives changes to Digital Health. Unintended effects on business due to IT changes will be minimised. A technology change may provide an opportunity to improve the business process and, hence, change business needs.
**IMPLICATION**	• Changes to Digital Health technology infrastructure shall follow a complete examination of the proposed changes using the Digital Health Enterprise Architecture.<br>• The need for financial and technical assistance should be communicated by the business need or requirement.<br>• Change management processes conforming to this principle should be developed and implemented.
**CODE**	**T-P2**
**NAME**	**Control Technical Diversity**
**STATEMENT**	Technological diversity should be controlled to minimise the non-trivial cost of maintaining expertise and connectivity between multiple processing environments.
**RATIONALE**	There is a real, non-trivial cost of infrastructure required to support alternative technologies for processing environments. More infrastructure costs are incurred to keep multiple processor constructs interconnected and maintained. Limiting the number of supported components will simplify maintainability and reduce costs.

**IMPLICATION**	● Policies, standards, and procedures that govern the acquisition of technology must be tied directly to this principle.<br>● Technology choices for Digital Health solutions shall be constrained by the options available within the technology blueprint. Procedures for augmenting the acceptable technology set to meet evolving requirements will have to be developed and embraced.<br>● The technology blueprint of Digital Health shall only change when compatibility with current infrastructure improvement in operational efficiency or a required capability has been demonstrated.

Table 7. MoH Interoperability Principles

**CODE**	**I-P1**
**NAME**	**Re-use Digital Health components**
**STATEMENT**	Components and services that are re-usable across the health sector are preferred over proprietary solutions.
**RATIONALE**	Duplicating capability is expensive and undermines interoperability by proliferating inconsistency and ambiguity.
**IMPLICATION**	Where infrastructure components are provided for re-use within Digital Health solutions, adoption, integration, and use of these components should be preferred to duplicating their functionality through bespoke development.
**CODE**	**I-P2**
**NAME**	**Observe open standards**
**STATEMENT**	Promote adherence to open standards.
**RATIONALE**	● Adherence to open standards that will enhance interoperability of Digital Health.<br>● The attributes of open standards such as platform independence, vendor neutrality, and ability to use across multiple implementations and the model for establishing open standards are what will allow for sustainable information exchange, interoperability, flexibility, data preservation & and greater freedom from technology and vendor lock-in<br>● Adoption of open standards will facilitate the storing of electronic health data using open data file formats.
**IMPLICATION**	● Choices should comply with the World Trade Organisation (WTO) Code of Good Practice for the adoption and application of Standards. In particular, locally contextualised standards should be used where they exist.

Table 8. MoH Security and Privacy Principles

**CODE**	**SP-P1**
**NAME**	**Align security and privacy controls to the existing legal and regulatory framework**
**STATEMENT**	● Security and privacy controls should be compliant with Uganda's Data Protection and Privacy Act 2019 and Data Protection and Privacy Regulations, 2020<br>● The selection of security controls should be based on risk analysis and risk management decisions. The process for selecting new controls should consider both the degree of risk

	mitigation provided by the control and the total cost to acquire, implement and maintain the control.<br>• The selection of controls should be driven by the ability of the control to be applied uniformly across the entire health sector and to minimise exceptions.
**RATIONALE**	• Achieving a standards-based Digital Health security environment will reduce operational costs, improve interoperability and improve supportability.
**IMPLICATION**	• Health organisations should ensure that Security and privacy policies align with Uganda's National Information Security Framework.
**CODE**	**SP-P3**
**NAME**	**Incorporate Security into Architecture Design**
**STATEMENT**	Architectures should employ security measures to ensure integrity, confidentiality, and availability of IT services and applications. Security should be designed into the architecture in a scalable and efficient manner. The security architecture design should follow a modular design where the overall technology infrastructure is divided into functional layers/modules.
**RATIONALE**	The layered/modular approach allows the architecture to address the security relationship between the various functional blocks of the infrastructure. In addition, it permits designers to evaluate and implement security on a module-by-module basis instead of attempting to complete the architecture in a single phase.
**IMPLICATIONS**	Comprehensive published security standards for each layer/module of the IT architecture are a prerequisite for all security designs.
**CODE**	**SP-P4**
**NAME**	**Implement Least Privilege Standard**
**STATEMENT**	Each Digital Health solution must incorporate a standard for the least privilege; thus, the baseline is the bare minimum privileges necessary to perform functions, actions, and access.
**RATIONALE**	The baseline for functions, actions, and access is the minimum required to complete the task, thus protecting users and the data they are using from inadvertent errors, exploitation, or attempts at deliberate misuse. Adhering to the principle of least privilege reduces the risk of attackers gaining access to critical systems or sensitive data by compromising a low-level user account, device, or application.
**IMPLICATION**	• Part of the solution design should ensure the principle of least privilege is incorporated into the solution to enhance the protection of data and functionality from faults and malicious behavior.<br>• Contains compromises to their area of origin, stopping them from spreading.<br>• The scope of audits can be reduced dramatically, thus ensuring the efficient use of scarce resources.<br>• Users, clients, businesses, and the enterprise are better protected.
**CODE**	**SP-P5**
**NAME**	**Implement appropriate Privacy and Security Safeguards**
**STATEMENT**	Appropriate technical and organisational controls must be implemented to protect data and keep it confidential.
**RATIONALE**	The privacy and security of data are everyone's responsibility. The Data Protection and Privacy Act 2019 requires organisations that process personal data to ensure they maintain the security and privacy of this data. It is necessary to have policies and security safeguards implemented to protect health information, whether stored on paper or electronically.

**IMPLICATIONS**	• Access controls – Access to information should be on a need-to-know basis, with role-based access.<br>• Technical controls – Implement robust IT security controls such as password authentication, encryption, anti-malware software, patch management, firewalls, IDS, mobile device management, secure code reviews, etc.<br>• Auditing – Digital Health systems should be audited at least twice a year.<br>• Incident detection & response – continuous monitoring to identify and respond to threats.

COMPONENT 4: ARCHITECTURE PROCESS

The DHEAF includes an architecture process illustrated in **Figure 5** that addresses the Enterprise Architecture goals of the Digital Health initiatives. The cycle is enclosed by the architecture catalysts previously introduced so that all activities, deliverables, and guidelines are aligned to the constituents for the EA development to deliver a successful digital transformation initiative. The DHEAF development process is based on the TOGAF Architecture Development Model (ADM) cycle and impersonates the architecture development structure of TOGAF. The TOGAF ADM is a generic and reliable method for architecture development, designed to deal with most systems and organisational requirements (The Open Group, 2018a).

The architecture process provides for the development and implementation of Enterprise Architectures that address the goals and objectives of the health organisations. The architecture process phases include business, application, data, and technology, followed by opportunities and solutions, migration planning, implementation governance, and change management that work in conjunction with procedures laid down in the Health Information and Digital Health Strategy for Uganda. In addition, the Architecture Process emphasises requirements management at every stage and includes governance via several governance bodies highlighted in the EA governance model. It is, however, important to note that in navigating the architecture process, architects should leverage the reusable assets that are part of this framework. These assets include; reference models for Business, Data, Application, Technology, and services, Information security architecture, and Health Information Exchange (HIE) model. Others include; the Digital Health capability model, Interoperability standards, Monitoring and Evaluation model, and the DH capacity building/skills model. **Figure 5** illustrates the phases of the architecture process for Digital Health including the respective phase associated activities and expected architecture inputs and outputs. It is also necessary that the requirements are identified, stored, and input into all activities in the architecture process. Health organisations should define all the non-functional requirements of the solution they would like to implement. The architecture team should consider the domain-specific principles that derive the requirements and existing Digital Health Policies and standards when defining requirements.

Description of the phases in the Architecture process

Preliminary Phase

The Preliminary Phase prepares a health organisation for a successful EA project. It mainly focuses on the preparation and initiation activities required to meet a business directive for digital transformation. Specifically, it is concerned with determining and establishing the architecture capability needed by a health organisation and defining the principles that will constrain the digital transformation effort. Activities involved in this phase include; scoping the health organisation's units to be impacted by the digital transformation effort, establishing architecture principles, and developing a strategy and implementation plan for the productivity tools (i.e., Modelling) that will be required. **Table 9** outlines the critical inputs and outputs of the preliminary phase.

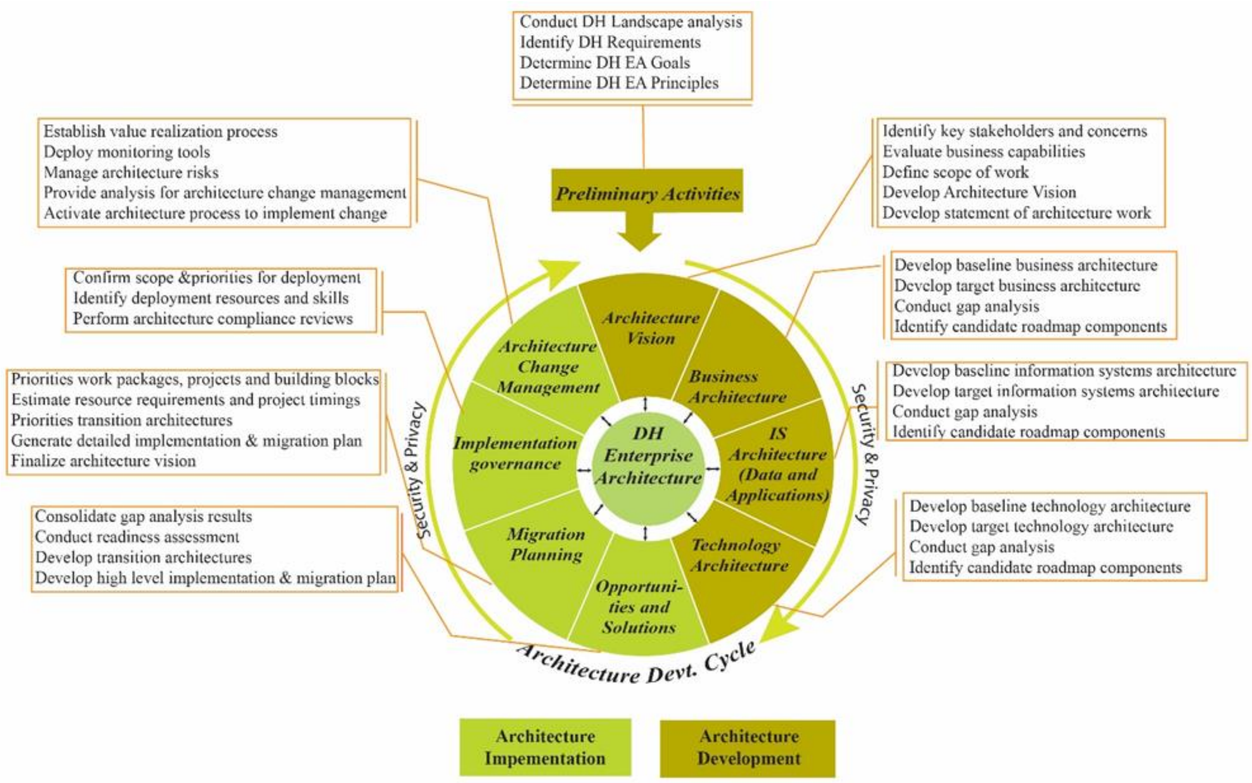

Figure 5. Architecture Process (Adopted from The Open Group,2018)

Table 9. Inputs and Outputs of Preliminary Phase

**Inputs**	• Business strategies, business goals, and business principles of the health organisation<br>• Governance and legal frameworks relevant to Digital Health<br>• Partnerships and Contracts agreements<br>• National Health Information and Digital Health Strategy for Uganda

**Outputs**	• Organisational Model for Enterprise Architecture specifying the units that will be part of the initiative<br>• Request for Architecture Work

Architecture Vision

The Architecture Vision phase defines the scope of the architecture effort and the constraints that the health organisation must deal with. The primary goal of this phase is to create a high-level aspirational vision of the business capabilities targeted by the health organisation. Therefore, critical activities of this phase should include; identifying key stakeholders and their concerns, evaluating business capabilities, defining the scope of work, and developing an Architecture Vision. Furthermore, when documenting the Architecture Vision, architects need to show the value proposition for each category of stakeholders in the digital transformation effort. Also, as part of this phase, the architects must go through all the formal processes of seeking approval of the Statement of Architecture Work Approved by the relevant authorities. **Table 10** shows key inputs and outputs of the Architecture Vision phase.

Table 10. Inputs and Outputs of Architecture Vision Phase

Inputs	• Organisational Model for Enterprise Architecture specifying the units that will be part of the initiative<br>• Request for Architecture Work
Outputs	• Approved Statement of Architecture Work<br>• Capability assessments<br>• Architecture Vision including high-level stakeholder requirements<br>• Draft Architecture definition document including;<br>○ Baseline Business, Data, Application, and Technology Architecture (high-level)

Business Architecture

This phase elaborates on a Business Architecture based on the agreed-upon Architecture Vision. Specifically, it identifies business capabilities and processes, services, and actors in the current state and those required in the future. The principal technique applied here is Business Capability Modelling (BCM), used with business process models. All process models should be associated with enterprise-level information entities to reflect what business objects are accessed or ealized as they are executed. Activities of the Business Architecture phase include; developing the baseline and target business architecture describing the product/service strategy, organisation structure, functional processes, business goals, etc. Other activities include analysing the gaps between baseline and target business architectures and identifying business architecture's candidate road map components. **Table 11** shows key inputs and outputs of the Business Architecture phase.

Table 11. Inputs and Outputs of Business Architecture Phase

Inputs	• Approved Statement of Architecture Work<br>• Capability Assessments<br>• Business principles, business goals, business drivers<br>• Architecture Vision including key refined high-level stakeholder requirements<br>• Draft Architecture definition document including;<br>o Baseline Business, Data, Application, and Technology Architectures (high-level)<br>o Target Business, Data, Application, and Technology Architectures (high-level)
Outputs	• Draft Architecture Definition Document containing content updates:<br>o Baseline Business Architecture (detailed), if appropriate<br>o Target Business Architecture (detailed with Business Capabilities, business processes, and Organisation Map as core artefacts)<br>• Draft Architecture Requirements Specification document including content updates:<br>o Gap analysis results<br>o Technical requirements<br>• Updated business requirements<br>• Business Architecture components of an Architecture Roadmap

Information Systems Architecture

The Information Security Architecture Phase is concerned with identifying and defining the *applications* and *data* considerations that support a health organisation's business architecture developed in the previous phase. In this phase, the architects create both the Data and Application Architecture, which they may design either sequentially or concurrently.

Data Architecture

Data Architecture Phase is concerned with defining the data considerations that support the Business Architecture, including; data management (i.e., collection, processing, storage, and dissemination), migration, and governance. As part of the Data Architecture, the architecture team should consider developing the Application/Organisation, Role/Application, Application Interaction, and Application Function matrices. These matrices enable the architecture team to identify user and organisational dependencies on applications. In addition, this will support future state planning by determining impacted user communities and facilitating application grouping by user type or user location. **Table 12** shows the key inputs and outputs of the Data Architecture phase.

Table 12. Inputs and Outputs of Data Architecture Phase

Inputs	• Approved Statement of Architecture Work<br>• Capability Assessments<br>• Data principles<br>• Architecture Vision<br>• Draft Architecture definition document including;<br>o Baseline Business, Data, Application, and Technology Architectures (high-level)<br>o Target Business, Data, Application, and Technology Architectures (high-level)

Outputs	• Draft Architecture Definition Document containing content updates:<br>○ Baseline Data Architecture (detailed), if appropriate<br>○ Target Data Architecture (detailed).<br>• Draft Architecture Requirements Specification document including content updates:<br>○ Gap analysis results<br>○ Data interoperability requirements<br>• Relevant technical requirements that will apply to the digital transformation effort<br>• Constraints in the Technology Architecture<br>• Data Architecture components of an Architecture Roadmap

Applications Architecture

This phase is concerned with defining the application components and or subsystems required to support the business architecture, including; services, major functions, and interfaces. It shows how the application layer components map onto the Business Architecture. However, it is important to note that this phase is not concerned with application system design but is concerned with defining what kind of systems are relevant to Uganda's health sector. As part of this phase, the architecture team is encouraged to take advantage of relevant Application Architecture resources provided by:

- The TOGAF Integrated Information Infrastructure Reference Model (III-RM) — focuses on the application-level components and services necessary to provide an integrated information infrastructure ((The Open Group, 2018b))
- The Object Management Group (OMG) — has several software models relevant to domains such as healthcare (www.omg.org)

Key activities of the Application Architecture Phase should include developing the baseline and target Application Architectures, gap analysis, and identification of Application Architecture components of the Architecture Roadmap. **Table 13** shows the key inputs and outputs of the Application Architecture phase.

Table 13. Inputs and Outputs of Application Architecture Phase

Inputs	• Approved Statement of Architecture Work<br>• Capability Assessments<br>• Application principles<br>• Architecture Vision<br>• Draft Architecture definition document including;<br>○ Baseline Business, Data, Application, and Technology Architectures (high-level)<br>○ Target Business, Data, Application, and Technology Architectures (high-level)
Outputs	• Draft Architecture Definition Document containing content updates:<br>○ Baseline Application Architecture (detailed), if appropriate<br>○ Target Application Architecture (detailed)<br>• Draft Architecture Requirements Specification document including content updates:<br>○ Gap analysis results<br>○ Application interoperability requirements<br>○ Relevant technical requirements that will apply to the digital transformation effort<br>• Constraints in the Technology Architecture<br>• Data Architecture components of an Architecture Roadmap

Technology Architecture

The technology architecture phase maps application components defined in the Application Architecture phase into a set of technology components representing software, hardware, and communication technology. These include; computing platforms, storage, networks, operating system, middleware, database systems, other system software, and deployable artefacts. As part of this phase, the architects define new technology components introduced in the digital transformation effort. Likewise, existing technology components to be supported in the target environment may need to be re-defined to ensure fit-for-purpose and interoperability. The architecture team is encouraged to take advantage of relevant Technology Architecture resources provided by the TOGAF Technical Reference Model (TRM). Key activities for this phase should include developing baseline and target Technology Architectures, gap analysis, and identification of Technology Architecture components of the Architecture Roadmap. **Table 14** shows the key inputs and outputs of the Technology Architecture phase.

Table 14. Inputs and Outputs of Technology Phase

Inputs	• Approved Statement of Architecture Work<br>• Capability Assessments<br>• Technology principles<br>• Architecture Vision<br>• Draft Architecture definition document including;<br>o Baseline Business, Data, Application, and Technology Architectures (Detailed)<br>o Target Business, Data, Application, and Technology Architectures (Detailed)
Outputs	• Draft Architecture Definition Document containing content updates:<br>o Baseline Technology Architecture (detailed), if appropriate<br>o Target Technology Architecture (detailed)<br>• Draft Architecture Requirements Specification document including content updates:<br>o Gap analysis results<br>o Technology interoperability requirements<br>o Relevant technical requirements that will apply to the digital transformation effort<br>• Constraints in the Technology Architecture<br>• Technology Architecture components of an Architecture Roadmap

Opportunities and Solutions

This phase is concerned with identifying delivery vehicles (projects, programs, or portfolios) that will successfully deliver the Target Architecture identified in the business, application, and technology architecture phases. As part of this phase, the architecture team should review the target business objectives and capabilities of the health organisation, consolidate the gap analysis results and the interoperability requirements from the Business to Technology Architecture phase, and then organize groups of building blocks to address these capabilities. The architecture team should also conduct readiness assessment exercises to evaluate the health organisation's ability to absorb change. Finally, centering on the readiness assessment results, the architecture team should develop a series of Transition Architectures (delivery vehicles) to generate a high-level Implementation and Migration plan. **Table 15** shows the key inputs and outputs of the Opportunities and Solutions phase.

Table 15. Inputs and Outputs of Technology Phase

Inputs	• Capability Assessments<br>• Enterprise Architecture Governance Model/Framework<br>• Statement of Architecture Work<br>• Architecture Vision<br>• Draft Architecture Definition Document<br>• Draft Architecture Requirements Specification Document<br>• Change requests from existing Digital Health Programs/Projects<br>• Candidate roadmap components from Business, Information system, and Technology Phases.
Outputs	• Capability Assessments including;<br>o Business Capability<br>o IT Capability<br>• Architecture Roadmap including;<br>o Identified Transition Architecture<br>o Work package portfolio<br>o Implementation recommendations<br>• High-level Implementation and Migration Plan including;<br>o Implementation and Migration Strategy

Migration Planning

Migration planning is concerned with formulating a detailed Implementation and Migration Plan that will realise some or all of the Transition Architectures identified in the opportunities and solution phase. **Table 16** shows the inputs and outputs of the migration planning phase. Phase Key activities of this phase should include:

- Coordinating the implementation and migration plan with the various management frameworks within the health organisation, including project planning and business planning. Enterprise Architecture and Operations Management.
- Prioritising all work packages, projects, and building blocks by assigning business value to each and conducting a cost/business analysis. Business value must be associated with the capabilities at this stage, and capability increments should be used to assign the business values.
- Estimating resource requirements, project timings, and availability/delivery vehicles. When estimating resource requirements, costs should be divided into capital (to create the capability) and operations and maintenance (to run and sustain the capability).
- Prioritising the transition architectures/delivery vehicle through conducting a cost/benefit assessment and Risk authentication.
- Generating a completed Implementation and Migration Plan. This should include integrating all project activities, dependencies, and the impact of change into a project plan.
- Finalising the Architecture Vision and Architecture Definition Documents, in line with the agreed implementation approach.

Table 16. Inputs and Outputs of the migration planning phase

Inputs	• Capability Assessments<br>• Statement of Architecture Work<br>• Architecture Vision<br>• Draft Architecture Definition Document<br>• Draft Architecture Requirements Specification Document<br>• Change requests from existing Digital Health Programs/Projects<br>• Architecture Roadmap<br>• High-level Implementation and Migration Plan
Outputs	• Implementation and Migration Plan (detailed), including:<br>o Implementation and Migration Strategy<br>o Project and portfolio breakdown of the implementation<br>• Finalised Architecture Requirements Specification<br>• Finalised Architecture Roadmap Re-usable

Implementation Governance

This phase is concerned with providing an architectural oversight of implementing the target Digital Health architecture for Health organisations. Key activities of this phase include;

- Confirming scope and priorities for deployment with development management. The implementation team should review the migration planning outputs and produce recommendations for deployment. It should also identify building blocks for replacement, perform a gap analysis between the DH Architecture and solutions framework and produce a gap analysis report to guide implementation decisions.
- Identifying deployment resources and skills required for the deployment team. Additionally, the deployment team should be educated about the overall architecture deliverables and expectations from specific development and implementation projects.
- Performing architecture compliance reviews for each transition architecture. The compliance review must follow the compliance review process in the Enterprise Architecture Governance model. **Table 17** shows the inputs and outputs of the Implementation governance phase.

Table 17. Inputs and Outputs of Implementation Governance

Inputs	• Capability Assessment<br>• Statement of Architecture Work<br>• Architecture Vision<br>• Architecture Definition Document<br>• Architecture Requirements Specification<br>• Architecture Roadmap<br>• Digital Health Enterprise Architecture Governance Model<br>• Implementation and Migration Plan (Detailed)
Outputs	• Compliance Assessments<br>• Change requests<br>• Deployed Architecture compliant Digital Health solutions, including;<br>o Architecture-compliant implemented system<br>o Populated Architecture Repository

	o Architecture compliance recommendations and dispensations<br>o Recommendations on service delivery requirements<br>o Recommendations on performance metrics<br>o Service Level Agreements (SLAs)<br>o Architecture Vision, updated post-implementation<br>o Architecture Definition Document, updated post-implementation

Architecture Change Management

The Architecture Change Management phase establishes procedures for managing change to the new Digital Health architecture. The goal is to ensure that the architecture achieves its original target business value. Part of this phase requires the architecture team to continuously monitor governance requests, developments in technology, and changes in the business environment. In addition, the architecture team should put in place a change management process for the implemented architecture. The change management process, once established, will help to determine:

- Circumstances under which the implemented architecture, or parts of it, will be permitted to change and the process by which that will happen;
- The circumstances under which the architecture development process will be initiated again to develop a new architecture.

Table 18 shows the inputs and outputs of the architecture change management phase. Key activities of this phase should include:

- Establishing a value realisation process. The architecture team should engage stakeholders and agree on the Key Performance Indicators (KPIs) for each transition architecture/delivery vehicle.
- Deploying monitoring tools to support:
  - The business and technology changes could impact Digital Health Enterprise Architecture for the health organisation.
  - Business value tracking; e.g., investment appraisal method to determine value metrics for the business objectives.
  - Monitoring Enterprise Architecture Capability maturity of the health organisation
  - Tracking Quality of Service (QoS) performances and usage
- Managing architecture risks and providing recommendations on how the risks can be mitigated. In performing this activity, the architecture team is encouraged to use the approved Risk Management Framework for the MoH. If this is not available, the architecture team may adopt the Risk management process part in the Digital Health Enterprise Architecture Governance Model.
- Providing analysis for architecture change management, including:
  - Conducting architecture performance reviews;
  - Assessing requests for change and reporting to ensure that the expected value realisation and Service-Level Agreement (SLA) expectations are met;
  - Undertaking a gap analysis of the performance of the architecture;

  - Ensuring that requests for change adhere to the Request for Change that is part of the Digital Health Enterprise Architecture Governance Model;
  - Documenting recommendations on change requirements to meet performance targets;
- Activating the architecture process to implement the required change. This involves the development of a new Request for Architecture Work.

Table 18. Inputs and Outputs of Architecture Change Management Phase

Inputs	• Statement of Architecture Work<br>• Architecture Vision<br>• Architecture Definition Document<br>• Architecture Requirements Specification<br>• Architecture Roadmap<br>• Request for Change due to business and technology changes<br>• Compliance Assessments<br>• Implementation and Migration Plan
Outputs	• Architecture updates<br>• Changes to architecture vision and principles<br>• New Request for Architecture Work

COMPONENT 5: DIGITAL HEALTH MONITORING & EVALUATION

Evaluation of Digital Health interventions helps to generate data that can be used as a basis for assessing whether observed changes in behavior, processes or health outcomes can be attributed to the Digital Health interventions (Lau & Kuziemsky, 2016; WHO, 2016). ***Evaluation*** is defined as a systematic and objective assessment of an intervention that aims to determine the fulfilment of objectives, efficiency, effectiveness, impact and sustainability (WHO, 2013). ***Monitoring*** is the routine collection, review, and analysis of data intended to measure implementation progress for Digital Health initiatives, and results in adjustments in intervention activities necessary to maintain or improve the quality and consistency of the Digital Health deployment.

The Digital Health Monitoring and Evaluation Framework (DHM&EF) aims to guide the process of monitoring DH implementations and evaluation of DH results in terms of outcomes and impact on healthcare in Uganda. The DHM&EF constitutes of three components; i). a *reference model* that demonstrates key aspects of DH monitoring and evaluation; ii). *performance indicators* for measuring DH performance and how these can be operationalized; and iii). a *set of guidelines* on how to conduct monitoring and evaluation of Digital Health interventions.

The Digital Health evaluation reference model (**Figure 6**) provides a basis for evaluating the performance of Digital Health implementations along the results chain (**Appendix 2**), starting with the programme inputs and activities until the impact. The model indicates the key Digital Health evaluation questions that should be answered; the key domains of performance indicators, and the data sources where the data needed to measure the indicators can be collected at each stage of the results chain.

Monitoring of programme inputs and activities answers whether the DH technology works, and it relates to the assessment of the feasibility of the DH implementation and the inputs for developing the initiative. Monitoring of DH outputs assesses how the users interact with the initiative, and it relates to the service output measures intended to capture and assess the immediate results of the DH initiative as well as the usability measures to help quantify how the users interact with it.

Evaluation of DH outcomes help to ascertain how the DH improves programme processes and service delivery; and it captures the effect of the Digital Health implementation on programme service utilization outputs or the extent to which the clients use the programme service, and intermediate population-level outcomes. The "*evaluation of impact*" focuses on establishing how improvements in service delivery impact health by capturing long-term outcomes, and impact on health that are attributed to the health intervention being supported by the DH initiative.

Digital Health Monitoring and Evaluation Reference Model

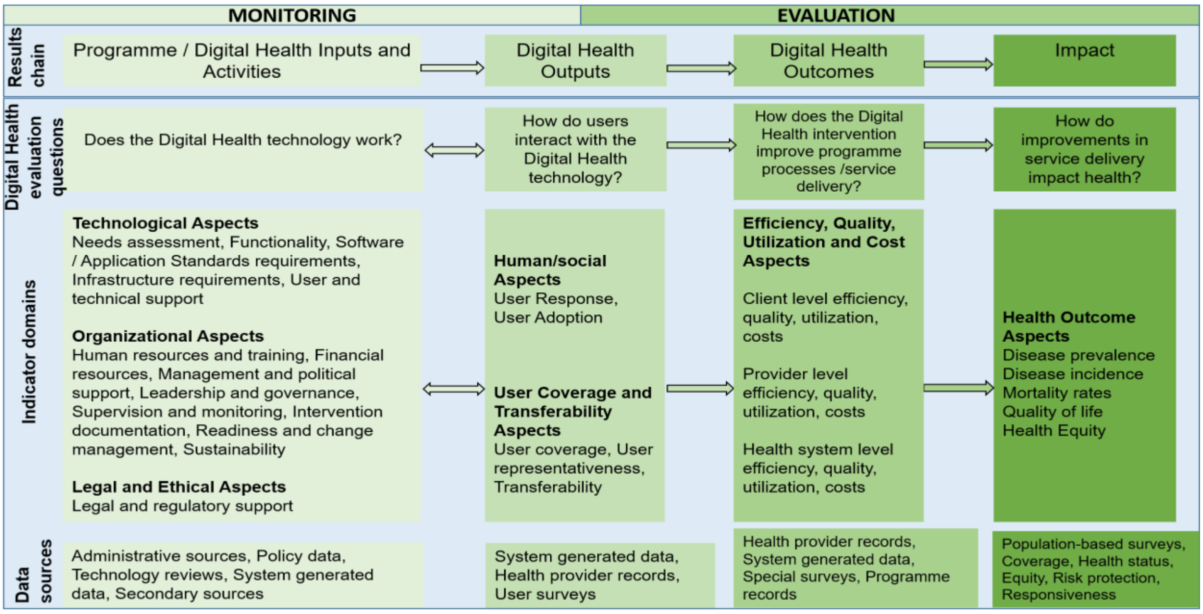

Figure 6. Digital Health Evaluation Reference Model (Adopted from Ashaba & Nabukenya, 2022)

Also, to note is a feedback loop in monitoring of the programme inputs and activities, and DH outputs. From a technical perspective, DH inputs affect the respective outputs and users' experience, while the user experience/feedback informs the DH development process. Feedback from end-user adoption and satisfaction rates loops around to further inform the DH development process and determines the quality of the technological inputs. This, in turn, affects the performance of the revised version of the DH system among end-users, making the technology development process iterative.

DHM & EF Evaluation Indicators and Operationalisation Tools

The DHM&EF Evaluation indicators and operationalisation tools (**Appendix C1**) demonstrate how the DH interventions' performance can be measured at each stage of the

results chain in the reference model. To note is that, this set of example indicators and operationalisation tools are general and non-exhaustive; henceforth requiring that priority indicators and their operationalisation tools for use to monitor and evaluate a specific DH intervention should be selected based on the relevance of the intervention and modified to reflect the specific objectives of the intervention.

DH Interventions' Evaluation Guidelines

The last component of the DHM&EF Evaluation framework provides a set of guidelines (strategies and course-of-actions) that are intended to guide/facilitate the users through a step-by-step process of planning and executing activities for monitoring and evaluation of DH interventions. These should be followed with adaptation to the DH context.

1. Establish dedicated resources and effort to ensure an effective process and such considerations should be provided from the inception of the initiative. DH implementers should plan and budget for monitoring and evaluation of the interventions during planning and costing of their DH programmes, to ensure that appropriate resources are dedicated (WHO & ITU, 2012).
2. Identify all key stakeholders of the DH initiative, involve them and consider their interests in the DH intervention while designing the monitoring and evaluation plans, and ensure their consensus on the public health need or goal that the DH addresses or supports (WHO, 2016).
3. Make use of/refer to the DH reference model, indicator domains and sub-domains as well as the example indicators as guides to think through the necessary monitoring and evaluation aspects of the DH intervention. The reference model provides a graphical representation to aid the clarification of monitoring and evaluation scopes and identification of expected causal links between inputs and activities, outputs, outcomes and impacts for DH implementations, while the indicator domains and indicators provide a non-exhaustive list of some examples of indicators that can be measured under each stage of the results chain.
4. Define the appropriate DH initiative implementation phases and timeframes to specify and harmonize the stages of the DH maturity, timing for delivery of the defined results and frequency of measuring the results. Defining the DH initiative implementation phases helps to refine and focus on the appropriate results and indicators to be monitored or evaluated at each stage of maturity of the DH intervention. Stakeholders should agree on the stage of maturity of the DH intervention and determine the appropriate monitoring or evaluation activities to avoid embarking on premature DH results (WHO, 2016). The mapping shown in **Table 19** can be used to match the six stages of maturity of a DH intervention with the results chain and DH evaluation questions.

Table 19. Mapping Digital Health maturity stages with the results chain and Digital Health evaluation questions

Stages of Digital Health Maturity	Pre-prototype	Prototype	Pilot	Demonstration	Scale-up	Integration/ sustainability

Results Chain Stage	Programme and Digital Health Inputs and Activities	Digital Health Outputs	Digital Health Outcomes	Digital Health Impact
Digital Health evaluation question	Does Digital Health technology work?	How do users interact with Digital Health?	How does Digital Health improve programme processes/ service delivery?	How do improvements in service delivery impact health?

5. Define stakeholder priorities and their projected impact of the DH intervention and outcomes required to cause the defined impact, and the respective impact and outcome measurable indicators while ensuring that the interests of all identified stakeholders are addressed. Evaluation of a DH intervention can be limited to its process and early outcomes without the need to evaluate its long-term outcomes and impact if the DH is supporting a health intervention with known efficacy based on prior research.
6. Define DH outputs required leading to each defined DH outcome and the respective DH output indicators (MoH-Uganda, 2016). The outputs should include immediate results of the DH intervention and should also capture usability measures that help quantify how users interact with the DH intervention.
7. Define DH inputs and activities and their respective measurable indicators while taking into consideration all the technical, organisational, policy and behavioral factors that influence the delivery of a working DH application. Indicators for the availability of resources required to support monitoring and evaluation functions should also be considered while defining indicators for the DH inputs and activities.
8. Define the baseline and target measures for defined indicators to clearly label the situation before the DH implementation (baseline measures) and expected achievements (target measures) after the DH implementation (MoH-Uganda, 2016). A measure of the baseline situation facilitates the ability to compare situation statuses before and after the DH implementation. It necessitates research and analysis to determine baseline measures for each indicator, while internal analysis and input from the subject-matter experts and other health experts are necessary to determine target measures (WHO & ITU, 2012).
9. Define the necessary monitoring and evaluation governance and processes/activities (MoH-Uganda, 2016; WHO & ITU, 2012). Considering that monitoring and evaluation tasks are conducted at different and various times and by various parties, their governance model must be defined to guide the available functions, roles, responsibilities, and required mechanisms to deliver them.
10. Prepare for and initiate the monitoring and evaluation process that should continue ongoing in parallel with the DH implementation (MoH-Uganda, 2016; WHO & ITU, 2012) , with monitoring or evaluation undertaken at the defined timeframes for each of the indicators. The preparation involves defining detailed monitoring and evaluation timelines and milestones and ensuring the availability of all the resources required to support the monitoring and evaluation activities including the monitoring and evaluation teams and data collection tools. Depending on the timelines or reporting frequency set for each of the identified indicators, the indicators should be reviewed, and if necessary revised at the start of each next monitoring or evaluation cycle. The review and revision ensure that data is collected on the most relevant indicators during the monitoring or evaluation cycle.

11. Execute the monitoring and evaluation data collection activities and track indicator measures according to the DH maturity stage, timing for delivery of the defined results, defined indicators, frequency of measuring the results, and defined monitoring and evaluation timelines.
12. Analyze collected data and report on the actual situation versus target DH adoption and results as per defined indicators (see example reporting tool in **Table 20**). The analysis may also include identification of causes of divergences in actual and target performance at each stage in the results chain for each timeframe of measuring results.

Table 20. Example of Digital Health monitoring and evaluation reporting tool

**Result**	**Indicator**	**Reporting Frequency**	**Baseline (Period)**	**Reporting Period 1**		**Reporting Period 2**	
				**Target**	**Actual**	**Target**	**Actual**
**Digital Health Impact**							
Impact 1	Indicator 1.1						
	Indicator 1.2						
Impact 2	Indicator 2.1						
	Indicator 2.2						
**Digital Health Outcomes**							
Outcome 1	Indicator 1.1						
	Indicator 1.2						
Outcome 2	Indicator 2.1						
	Indicator 2.2						
**Digital Health Outputs**							
Output 1	Indicator 1.1						
	Indicator 1.2						
Output 2	Indicator 2.1						
	Indicator 2.2						
**Digital Health Inputs and Activities**							
Input 1	Indicator 1.1						
	Indicator 1.2						
Input 2	Indicator 2.1						
	Indicator 2.2						

13. Engage stakeholders to agree on corrective actions to address divergence between target and actual results, and provide for management of changes in the DH implementation scope, if required, to implement agreed corrective actions (MoH-Uganda, 2016; WHO & ITU, 2012).

14. For indicators that may be unrealistic or unachievable within the required timeframes, engage stakeholders to review and agree on re-fined indicators, targets and timeframes for consideration in the next round of results measurement.

COMPONENT 6: DH ENTERPRISE ARCHITECTURE GOVERNANCE MODEL

Architecture Governance is the practice by which Enterprise Architecture and other architectures are managed and controlled at an enterprise-wide level (Josey et al., 2018). It encompasses a series of processes, cultural orientation, and a set of owned responsibilities that ensure the integrity and effectiveness of the organisation's architecture.

Figure 7 illustrates the Digital Health Enterprise Architecture Governance structure based on practices for architecture governance proposed by TOGAF (The Open Group, 2018a) and leverages the Digital Health Governance structures highlighted in the Uganda National eHealth Strategy 2017-2021(MoH-Uganda, 2016). The model highlights the governance structure and processes required to support the implementation and use of Digital Health in Uganda's Health System. The organisation structure represented in **Figure 7** comprises five levels, including; MoH Top Management, MoH Senior Management, Health Information, Innovation and Research Technical Working Group (Architecture Review Board), the Division of Health Information Management, the Digital Health Enterprise Architecture Program Office, and Digital Health Communities of Practice. Each level has specific roles and responsibilities in the development, implementation and use of Digital Health.

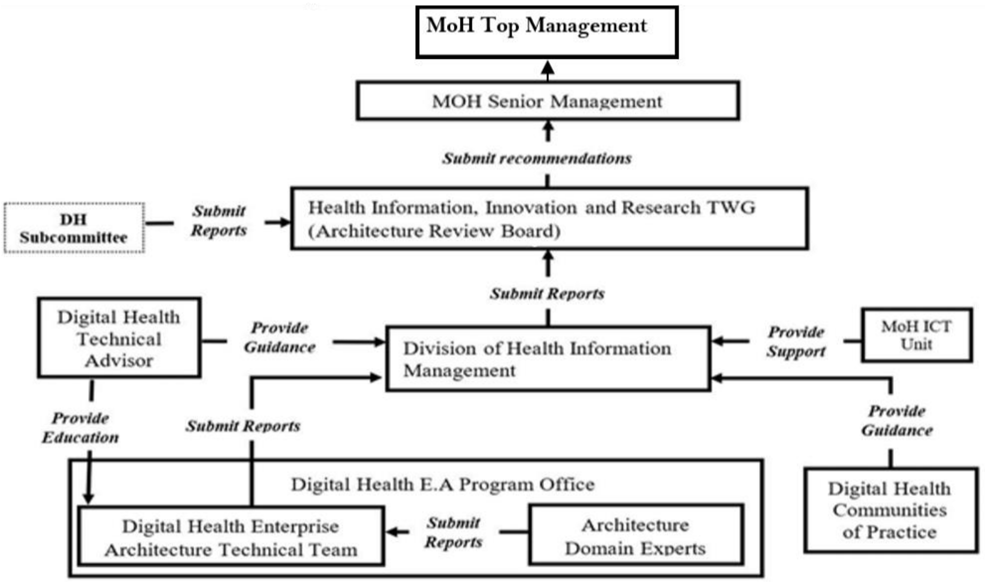

Figure 7. Digital Health Governance Structure

Description, Roles, and Responsibilities of DHEAG in Health Organisations

(1) Top Management (Exists)

MoH Top Management shall be the topmost decision-making body on all matters relating to Digital Health at the Ministry of Health. They shall be responsible for providing the governance to make Enterprise Architecture work within Uganda's health system. MoH Top

Management shall oversee all Digital Health Enterprise Architecture activities, including understanding and directing the linkage among MoH Health Sector Development Plan, MoH Digital Health Strategic Plan, business transformations, information governance transformations, and technology directions and investments across Uganda's health system. They will also act as vocal and visible champions for Enterprise Architecture initiatives within Uganda's Health sector and shall approve all project deliverables.

Roles and Responsibilities

- Approve Digital Health architecture standards, policies, and contracts.
- Approve the Digital Health Enterprise Architecture Roadmap and promote it among all Digital Health stakeholders.
- Approve Digital Health Architecture deliverables.
- Approve funding for the Digital Health Enterprise Architecture activities.
- Approve the Digital Health Enterprise Architecture framework.
- Approve the Digital Health Enterprise Architecture Metamodel and Guidelines for Enterprise Architecture introduction.
- Provide strategic direction for the Digital Health Enterprise Architecture activities.
- Ensure the alignment of the Enterprise Architecture initiatives with both the National Health Sector Development Plan and Uganda National Digital Health Strategy.

(2) MoH Senior Management (Exists)

MoH Senior Management shall be the second topmost decision-making body on all matters relating to Digital Health at the Ministry of Health. They shall be responsible for guiding to make Enterprise Architecture work within Uganda's health system. MoH Senior Management shall monitor all Digital Health Enterprise Architecture activities, including understanding and guiding the linkage among MoH Health Sector Development Plan, MoH Digital Health Strategic Plan, business transformations, information governance transformations, and technology directions and investments across Uganda's health system. They will also act as vocal and visible champions for Enterprise Architecture initiatives within Uganda's Health sector and shall endorse all project deliverables.

Roles and Responsibilities

- Endorse Digital Health architecture standards, policies, and contracts.
- Endorse Digital Health Enterprise Architecture Roadmap and promote it among all Digital Health stakeholders.
- Endorse Digital Health Architecture deliverables.
- Endorse funding for the Digital Health Enterprise Architecture activities.
- Endorse the Digital Health Enterprise Architecture framework.
- Endorse the Digital Health Enterprise Architecture Metamodel and Guidelines for Enterprise Architecture introduction.
- Monitor and provide strategic direction for the Digital Health Enterprise Architecture activities.

- Ensure the alignment of the Enterprise Architecture initiatives with both the National Health Sector Development Plan and Uganda National Digital Health Strategy.

(3) Digital Health Technical Advisor (Proposed)

The Digital Health Technical Advisor will serve as an expert who understands what it takes to develop, deliver and maintain high-quality Digital Health Enterprise Architecture. The Digital Health Technical Advisor shall be responsible for providing education to the Digital Health Enterprise Architecture program office. Additionally, the Digital Health Technical Advisor shall provide technical advice regarding Digital Health Enterprise Architecture implementation to the Division of Health Information Management.

Roles and Responsibilities

- Provide strategic direction to the Health Information, Innovation and Research (HIIRE) team to implement the DH Enterprise Architecture Framework.
- Guide the Division of Health Information (DHI), HIIRE TWG, MoH Senior Management and MoH Top Management on all issues that relate to Digital Health.
- Advocate and educate DH stakeholders on enterprise architecture and its benefits.
- Provide advice and training to all MoH internal Digital Health stakeholders on the current state of Digital Health technologies, policies, and functionalities.
- Assist with identifying and evaluating Digital Health solutions that will enhance the business processes of Uganda's health system.

(4) Health Information, Innovation and Research Technical Working Group (Exists)

The Health Information, Innovation and Research (HIIRE) Technical Working Group (TWG) is a multi-sectoral body responsible for providing technical advice to MoH and stakeholders on implementing the DHEAF framework. They will act as the Architecture Review Board (ARB) for all DH Enterprise Architecture artefacts. Within HIIRE TWG, there shall be an Architecture sub-committee responsible for providing technical advice on EA. The committee will constitute individuals knowledgeable in EA who will be responsible for providing technical advice relating to all DHEA artefacts and deliverables.

Roles and Responsibilities

- Review all architecture artefacts/deliverables and make recommendations to MoH senior Management.
- Enforce compliance to Digital Health Enterprise Architecture and grant dispensations where necessary.
- Improve maturity levels of architecture discipline within Uganda's healthcare system.
- Ensure that the adoption of the discipline of architecture-based development is across the health sector.
- Monitor and control the Architecture Contracts.
- Resolve escalated ambiguities, issues, or conflicts.
- Considering policy (e.g., schedule, Service Level Agreements (SLAs), etc.) changes where similar dispensations are requested and granted.

- Providing a fundamental control mechanism for ensuring the effective implementation of the Digital Health architectures.

(5) Division of Health Information (DHI) Management (Exists)

The Division of Health Information (DHI) Management is a National Digital Health entity within the Ministry that coordinates and oversees Digital Health investment. In addition, it is responsible for reviewing and monitoring Digital Health strategy outcomes and developing strategic recommendations and priorities for consideration by the HIIRE TWG.

Roles and Responsibilities

- Provide strategic direction to the Enterprise Architecture Program Office.
- Advocate and educate Digital Health stakeholders (Patients/Clients, Heath Practitioners and Managers, Social Care Practitioners, Health Insurance organisations, Health researchers, and Health Development Partners) on enterprise architecture and its benefits.
- Market the benefits of enterprise architecture via collaborative forums.
- Prepare an implementation plan for the Digital Health Enterprise Architecture Framework.
- Coordinate the development of segment and capability architectures across the health sector.
- Preparing and implementing the strategy for stakeholder management and engagement.

(6) Digital Health Enterprise Architecture Program Office (Proposed)

The DH Enterprise Architecture Program Office will be a permanent unit under the Division of Health Information (DHI) Management. The purpose of the program office will be to cultivate the culture of Enterprise Architecture practice throughout Uganda's health system.

Composition of Digital Health Enterprise Architecture Program Office

The composition of the Digital Health Enterprise Architecture Program Office includes:

- Manager, Enterprise Architecture
- Enterprise Architect(s)
- Domain Architect(s) (Business, Data, Application, Technology and Security)
- Enterprise Architecture Standards Specialist(s)
- Enterprise Architecture Policy Specialist(s)

Roles and Responsibilities

- Promote and support the MoH in the adoption and implementation of Enterprise Architecture practice.

- Coordinate and manage the Digital Health Enterprise Architecture Program, Digital Health Communities of Practice, Digital Health Enterprise Architecture Governance processes, and Architecture Repository.
- Ensure that DH Enterprise Architecture programs/projects are carried out consistently and successfully in compliance with standards, procedures, and strategies.
- Provide education on DHEA Framework, Standards, and Policies.
- Investigate performance issues of the Digital Health Enterprise Architecture initiatives and communicate early warning signs of the troubled areas.
- Ensure quality control, quality assurance, and process coaching. The Program office will inspect Digital Health Enterprise Architecture artefacts/ deliverables and provide coaching to Digital Health projects teams requiring additional support.
- Identify Enterprise Architecture risks and propose mitigation measures.
- Develop Enterprise Architecture Standards. They can work in conjunction with DH stakeholders to develop the standards.
- Develop Enterprise Architecture Policies. As part of their mandate to oversee that the policies are developed.

(7) Digital Health Communities of Practice (Exists)

The Digital Health Communities of Practice is a group of architects and other information technology and subject matter experts with shared interests and shared concerns and passion for Digital Health. This group will ensure the proper development and evolution of various Digital Health Enterprise Architecture artefacts and deliverables. In addition, the Digital Health Communities of Practice will purely play an advisory role in all Digital Health Enterprise Architecture initiatives.

Roles and Responsibilities:

- Influence the practice of EA by advising the Health Information, Innovation and Research (HIIRE) Technical Working Group in the development of Digital Health implementation plans.

Digital Health Enterprise Architecture Governance Processes

Architecture Governance processes are a set of activities that an organisation executes to support decision-making and accountability during the implementation of an architecture (Josey et al., 2018). The Digital Health Enterprise Architecture governance processes are intended to help identify, manage, audit and disseminate all information related to architecture contracts and implementation management. In addition, these processes are designed to ensure the monitoring of all architecture artefacts and contracts, principles, and operational-level agreements generated by Digital Health initiatives.

Architecture Policy Management and Take-on

All architectural amendments, contracts, and supporting information must come under governance through a formal process to register, validate, ratify, manage and publish new or updated content (The Open Group, 2018b). This requires that the MOH enact effective

architecture policies to guide all Digital Health Enterprise Architecture initiatives. For purposes of effective policy management and take-on, MoH should adhere to the following:

- MoH Top Management shall have the authority to issue architecture policies. This will, however, only be done after an appropriate review by MoH Senior Management and HIIRE TWG.
- MoH Top Management shall ensure timely communication of architecture policy changes. Timely communication of architectural policy changes will strengthen a culture of policy adherence among Health organisations.
- All changes to architecture policies shall be documented to track all changes. Records should specify who made the change, why they made it, and when the change was made.
- The MoH should put a central place (repository) where DH Organisations can easily access all architecture policies. Clear and well-organised architecture policies provide little benefit if the people who need them cannot access them. Therefore, MoH should ensure that all EA policies are accessible to the public through the MoH DH repository.
- The MoH should put a feedback mechanism for Digital Health stakeholders' comments on the architecture policies. A culture that does not readily accept stakeholders' comments will likely produce a substandard set of architectural policies.
- The MoH should regularly review all architecture policies. The review dates should be agreed upon when the policy is first created. A key element of effective policy management is an ongoing commitment to review and update all policies before they become outdated periodically.
- Drafting or revision of architecture policies should follow a formal process. Following a formal process results in policies that are consistent and reflect the needs of stakeholders (Hudson et al., 2019). **Figure 8** shows an 8-step process that shall be followed to develop or review architecture policies. This process borrows the best practices for stakeholder involvement highlighted in the WHO National eHealth Strategy toolkit (WHO & ITU, 2012).

Architecture Compliance Review

An *Architecture Compliance Review* is a scrutiny of the compliance of a specific project against established architectural criteria, spirit, and business objectives (The Open Group, 2018a). Confirming the compliance of individual DH projects with the Digital Health Enterprise Architecture framework is an essential aspect of Architecture Governance. Therefore, the Division of Health Information Management shall periodically carry out compliance reviews for all Digital Health initiatives to ensure conformance to the Architecture Vision, Principles, and Standards. Architecture compliance reviews should be held at appropriate project milestones. The most appropriate times are when the architecture is taking shape but when there is still time to correct any errors or shortcomings in the architecture effort. **Figure 9** shows the compliance review process for all Digital Health Projects. The compliance review process is adopted from the TOGAF and contextualised for Uganda.

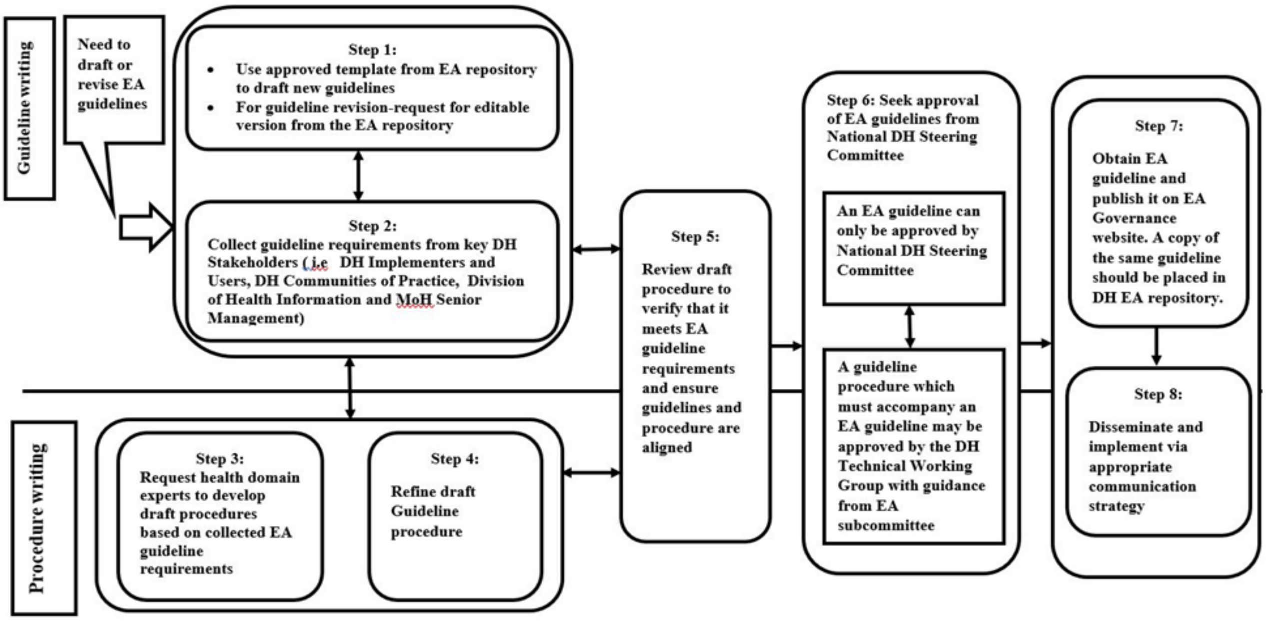

Figure 8. Architecture Policy Development Process (WHO & ITU, 2012)

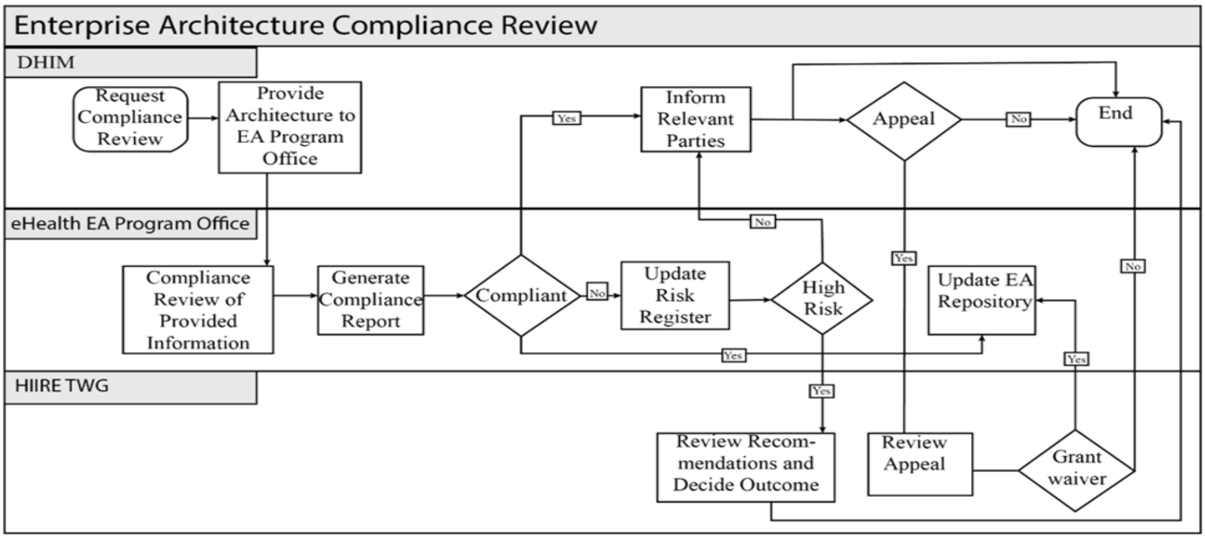

Figure 9. Architecture Compliance Review Process (The Open Group, 2018)

Description of the architecture compliance review process

The compliance review process for all Digital Health projects shall follow these steps:

1. The Division of Health Information (DHI) Management, which is the overall overseer of all Digital Health initiatives in the MoH, shall request the Digital Health Enterprise Architecture Program Office to undertake a compliance review for a Digital Health initiative that is under execution.
2. The Division of Health Information shall provide the Digital Health EA Program office with all pertinent architecture information required for the review.
3. The EA program office shall conduct the compliance review based on information provided by the DHI and any other information that may be available in the Ministry's

Digital Health Architecture repository. Compliance reviews shall be carried out every quarter (i.e., every 3 months).

4. On completion of a compliance assessment, a Compliance Review report shall be forwarded by the Digital Health EA Program Office to the DHI, who will liaise with the relevant Digital Health project to inform them of the outcome.

   - If the architecture of the Digital Health project is fully compliant, the DHI will allow the project to continue.
   - If the architecture is partially compliant, the Digital Health project shall be required to submit a reworked architecture within one month.
   - If the architecture is non-compliant, the DHI shall forward the review report to HIIRE TWG for further consideration. In this case, a risk is added to the appropriate risk register; an assessment shall be carried out to determine its likelihood and impact. If the likelihood and impact of the risk are high, HIIRE TWG may issue recommendations that may include suspension of work until the risks have been effectively mitigated.

Request for Exception (Dispensation)

There may be cases in which a Digital Health initiative or a subject area (design, operation, service level, or technology) does not comply with the DH Enterprise Architecture Framework. These non-compliance cases may either be known at the initiation stage or are revealed through the Architecture Complains reviews. In cases where the non-compliant subject area, despite best efforts utilized, cannot comply with the Digital Health EA Framework and needs more time to comply, dispensation may be requested for the subject area (The Open Group, 2018a). However, to determine a dispensation requirement, a Health organisation shall discuss the dispensation requirement with the Digital Health EA Program Office. If the request is required, the health organisation will be allowed to submit a dispensation request to the Digital Health Program Office. For all DH projects, requests for exception to any architectural activity, policy, or standard shall follow the following process:

Step 1. Submit and process Requests at Digital Health Enterprise Architecture Program Office

Requests for exceptions shall first be submitted to the Digital Health Enterprise Architecture Program Office. The submitted request shall be recorded into the Enterprise Architecture Dispensation Register and allocated a unique reference identifier. Requests shall include:

- A description of the Digital Health project and its timeline.
- A description of the specific exception sought.
- A statement of the reasons for the exemption.
- A description of the steps taken to eliminate the need for the exception in the future.

Requests for exemption shall be allowed to be submitted at any time in the project's progress. However, the turnaround time shall be determined at the project level. They shall specifically include a Request for Proposal (RFP) response evaluation to determine whether to consider a particular response. The appropriate subject matter experts should assess each request, and the assessment shall include an evaluation of the health organisation's ability to implement the Digital Health Enterprise Architecture Framework.

Any issues or risks that arise during evaluation shall be negotiated with the submitting health organisation. After the assessment, the assessor shall provide a written recommendation to the relevant governing bodies for consideration. The recommendations should include any outstanding issues and risks, and conditions on granting the dispensation. The recommendations may include a deadline for removing the need for the exception, constraints on future projects, or similar terms.

Step 2: Appeal (if necessary) request at Division of Health Information Management
If the Digital Health EA Program Office does not grant the request and a particular Digital Health stakeholder is dissatisfied, the decision may be appealed to the DHIM. The DHIM shall consider the request at the next regular meeting (as defined by MoH's Division of Health Information) or, if necessary, at a special meeting. The DHIM may request additional information to make its decision.

Step 3: Re-appeal (if necessary) at HIIRE Technical Working Group
If the DHIM does not grant the request, the Digital Health project may submit an appeal to the HIIRE Technical Working Group. The HIIRE TWG decision shall be final.

Risk Management Process
There will always be risks with any digital transformation effort (The Open Group, 2018a). Therefore, it is important that risks are classified, identified, and mitigated early enough so that they can be tracked through a formal risk management process (Josey et al., 2018). The early identification of potential problems allows the creation of plans to reduce their potential adverse impact (Barateiro et al., 2012). Therefore, risk management shall be an integral part of all Digital Health initiatives. The goal is to define prevention and control mechanisms to address the risks attached to specific activities and valuable assets. Therefore, all Digital Health organisations shall be required to implement a risk management process. Where such a process exists but is insufficient, it can be extended by best practices stipulated in ISO 31010 2019 Risk Management Framework or The Open Group Enterprise Architecture Framework. Key steps to the risk management process should include classification, identification, analysis, evaluation, and treatment. Continuous monitoring and review should also be carried out to audit the behavior of the whole environment allowing, for instance, the identification of changes in risks or the suitability of implemented risk treatment procedures and activities. Also, there should be continuous communication and consultation with key stakeholders. throughout all the steps.

Architecture Repository Management
The Architecture Repository is a software tool that stores the essential architectural inputs and outputs, including Architectures themselves, the elements of which they are composed, standards, references, principles, and the Governance Register (The Open Group, 2018a). **Figure 10** provides a structural framework for the DHIM Architecture Repository, which will allow the division to distinguish between different types of architectural assets that will be realised by the various digital transformation efforts within Uganda's health sector. Six classes of architectural information shall be held within the MoH Enterprise Architecture Repository. These include; the Digital Health Enterprise Architecture framework, Digital Health Governance framework, Digital Health Architecture Landscape, Standards, Reference

models, records of governance activity, Digital Health requirements for Uganda, and Solution Building Blocks (SBBs) supporting the Architecture Landscape.

Description of Contents of Sections of the Repository

Architecture Metamodel – stores the DH Enterprise Architecture Framework for Uganda.

Architecture Capability – stores the parameters, structures, and processes that support governance of the Architecture Repository.

Architecture Landscape – stores architectural views of the state of the MoH Enterprise Architecture descriptions at particular points in time. The Architecture Landscape shall be divided into three levels, namely; *strategic architecture*, which shows the long-term summary view of the MoH Enterprise Architecture; *segment architecture*, which provides more detailed operating models for different healthcare domains; and *capability architecture*, which shows in detail how a unit of capability is going to be realised.

Standards Information Base – stores all standards with which new architectures must comply, including data, communication and infrastructure standards, application standards, selected products and services from suppliers, or shared services already deployed within MoH.

Reference Library – stores guidelines, templates, patterns, and other forms of reference material that can be leveraged to accelerate the creation of new architectures for health organisations within the MoH.

Governance Log – stores all records of governance activity related to the Digital Health Enterprise Architecture. The Governance log holds all architecturally significant decisions made, Compliance assessments, Capability assessments, and Performance assessments.

Architecture Requirements – stores all authorised architecture requirements which have been approved by the HIIRE Technical Working Group (Architecture Review Board).

Solutions Landscape – stores Solution Building Blocks (SBBs) supporting the Architecture Landscape which have been planned or deployed by the health organisation.

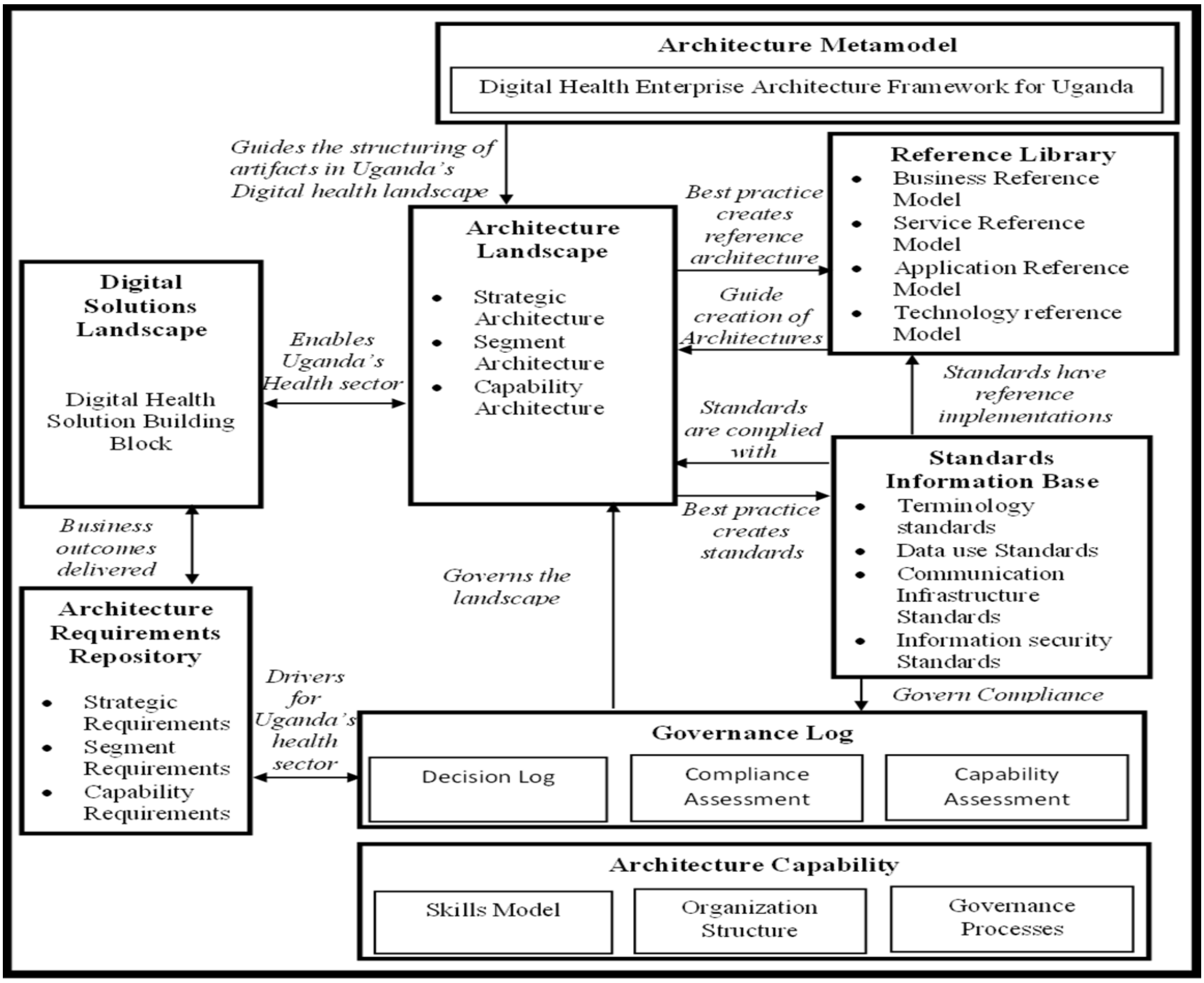

Figure 10. MoH Architecture Repository

COMPONENT 7: DIGITAL HEALTH SECURITY ARCHITECTURE

Privacy relates to the metadata that allows for geo-positioning of the digital health solution when in use. ***Confidentiality*** relates to the obligation of the digital service provider or other third-party user to protect the disclosure of either the content or metadata without explicit permissions from the client. ***Security*** relates to adoption and application of reasonable, technical and organisational measures to prevent loss, damage or unauthorised destruction and unlawful access to processing of the personal data.

The ***Digital Health Information Security Architecture*** (DHISA) intends to strengthen security regarding; *Confidentiality* (ensuring that information is not made available or disclosed to unauthorised entities), *Integrity* (protecting the accuracy and completeness of information), and *Availability* of health data/information (being accessible and usable when an authorised entity demands access) (ISO/IEC 27000 standard) held by Digital Health systems in Uganda.

The DHISA as presented in **Figure 11** is based on Sherwood Applied Business Security Architecture (SABSA) framework for enterprise security architecture and the Principles for Digital Development which outline best practices for digital health programming. SABSA is

a framework and methodology for enterprise security architecture and service management, and it integrates freely with TOGAF, the foundational framework for the Digital Health Enterprise Architecture Framework.

The DHISA comprises three main components that support each other to realize *Confidentiality*, *Integrity*, and *Availability* of health information. Additionally, DHISA includes all security considerations related to governance, technology, and operations that are required to protect the information assets of MoH. Specifically, the components are intended to ensure security of health information held by Digital Health systems by providing accountability and assurance.

1. **Security Drivers** – these are elements (internal and external) that can act as a source of information security for an organisation. DHISA highlights four key security drivers that Health organisations should focus on when identifying security requirements. Business requirements and opportunities are the key sources of internal security requirements. These have the service-level business requirements that must be met to effectively and efficiently provide services to clients. External security requirements may be derived from threats and the need to comply with legal and regulatory requirements. Privacy and confidentiality are key examples of functional requirements driven by legal requirements.

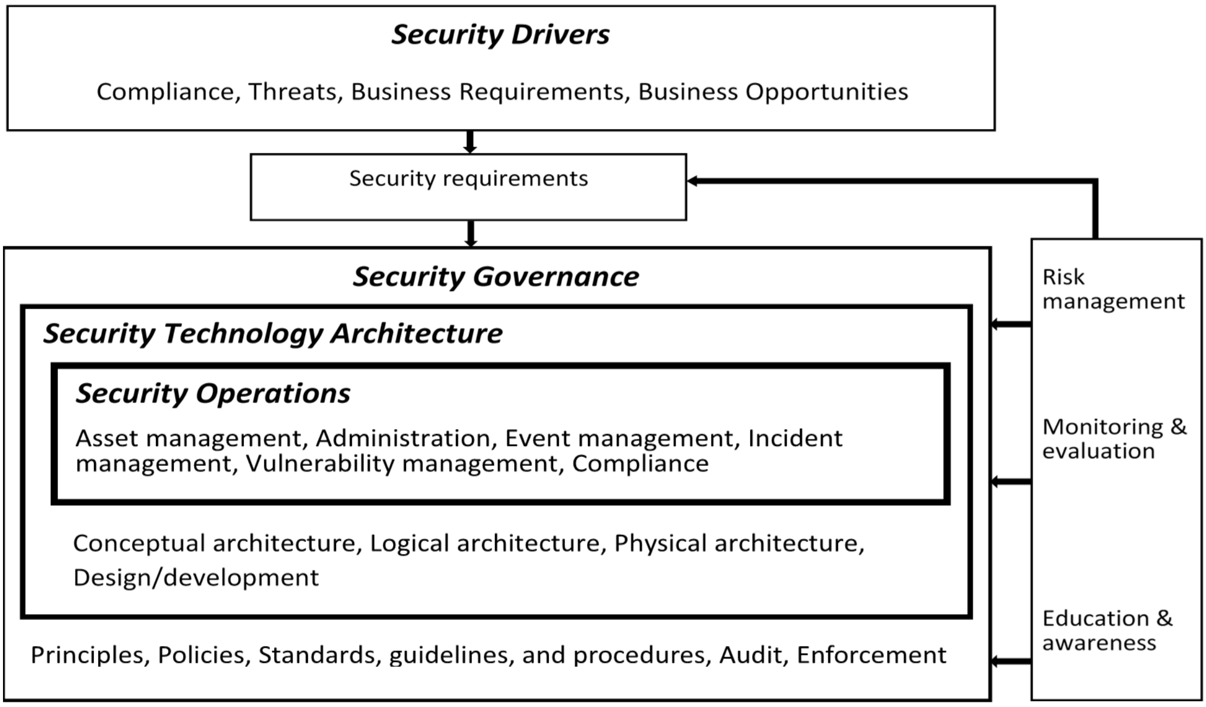

Figure 11. Digital Health Information Security Architecture

Security Requirements – these represent an information security functionality that ensures that the security properties of an Information system are being satisfied (Harris & Maymi, 2019). Security requirements drive the development of a Health organisation's security governance as well as the entire security program. Security requirements of Digital Health systems should be defined in terms of availability, integrity, and confidentiality.

Monitoring and Evaluation – this is the ongoing assessment of a Health organisation's compliance with DHISA and responding to changes that may impact any aspect of the security governance processes and policy framework.

Risk management – this is the process of determining the acceptable level of security risk at various points in the health organisations DH system and implementing the optimal level of management and technical control.

Education and awareness – these are programs that create awareness about current information risks and security practices so that DH users are empowered to protect the confidentiality, integrity, or availability of systems and data.

Security Governance – these are principles, policies, standards, guidelines, and procedures that allow the security goals to be set, expressed, and communicated throughout the different levels of a Health organisation. It grants power to entities needed to implement and enforce security and provides a way to verify the performance of these necessary security activities.

Security Technology – this is a set of conceptual diagrams and blueprints that document what the resulting security system will look like from various perspectives, including; conceptual, logical, and physical architectures.

Security Operations – these are security considerations for the day-to-day operations of the security services and supporting infrastructure. They include;

> <u>Asset management</u> – a component and process for maintaining the inventory of hardware and software assets required to support device administration, compliance monitoring, vulnerability scanning, and other elements of security operations.
> <u>Administration</u> – the process for securing the organisation's operational digital assets against accidental or unauthorised modification or disclosure.
> <u>Event management</u> – the process for the day-to-day management of the security-related events generated by a variety of devices across the operational environment, including security, network, storage, and host devices.
> <u>Incident management</u> – the process for responding to security-related events that indicate a violation or imminent threat of a breach of security policy (i.e., the organisation is under attack or has suffered a loss).
> <u>Vulnerability management</u> – the process of identifying high-risk infrastructure components, assessing their vulnerabilities, and taking the appropriate actions to control the level of risk to the operational environment.
> <u>Compliance</u> – the process of ensuring that the deployed technology conforms to the organisation's policies, procedures, and architecture.

2. **Security Governance –** establishes a governance model that Health organisations can use to assure that their DH information security strategies are aligned with and support business goals of MoH and are consistent with applicable laws and regulations through adherence to policies and internal controls, and provide assignment of responsibility.

Principles – are general rules and guidelines intended to be enduring and seldom amended that inform and support how an organisation sets about fulfilling its mission (Josey et al., 2018). All policies, standards, architectures, designs, operations, and other components of

Digital Health should always be aligned to established governance principles unless a governance body has granted an exception. Depending on the Health organisation, governing principles may be established at one or more levels. When adapting security principles to their needs, Health organisations should ensure that they are aligned with the high-level IT principles, which guide the use and deployment of all IT resources and assets across the enterprise. The DHEAF provides a list of security principles that Health organisations can adopt for their environments.

Policies – are governance tools used to enforce an organisation's guiding principles (Harris & Maymi, 2019). They define the authorizations and a program of actions adopted by an organisation to govern the use of technology in specific areas of management control and are established and maintained through standards, guidelines, and procedures in accordance with related legal and business principles. Health organisations should define a set of policies for information security and have them approved by their respective management. The information security policies should contain statements concerning definition of information security, objectives and principles to guide all activities relating to information security; assignment of general and specific responsibilities for information security management to defined roles and, processes for handling deviations and exceptions. Additionally, security policies should be reviewed at planned intervals or if significant changes occur to ensure their continuing suitability, adequacy and effectiveness.

Standards, Procedures and Guidelines – these are mandatory activities, actions, or rules. Policies should be implemented through; technical standards (mandatory directives that must be adhered to by all users), guidelines, and procedures (describe how to achieve the standard or guideline), which are distinguished as follows:

Enforcement – the process of ensuring compliance with policy. For all Health Organisations, enforcement of information security should be accomplished through a combination of technical controls, process and procedure controls, and management controls. Controls should be built into the implementation of technical standards and procedures. Management controls should provide for discretionary invocation of enforcement processes (such as disciplinary actions) as a result of security events or incidents.

Audit – an Information security audit is an organized, quantifiable technical assessment of how the organisation's security policy is employed (Onwubiko, 2009). Health organisations are encouraged to establish their own security audit frameworks and have them formally approved by relevant authorities. The framework comprises; the security policy (that defines acceptable use, technical controls, management standards and practices), audit policy (that specifies what needs to be audited), processes (organisation processes around security, procedures needed to protect valued assets), and regulatory compliance (that stipulates acceptable regulatory and security compliances).

3. **Security Technology Architecture** – focuses on sophisticated security infrastructures that support the DH applications. The model is described at four levels of abstraction; conceptual architecture, logical architecture, physical architecture and design/development.
    a. ***Conceptual Architecture*** – is a generic technical framework for policy-based security services. It decomposes policy management and security services to

specific conceptual services. The conceptual architecture should clearly state the overlap between policy management and runtime security services management splitting it into identity management, access management, and configuration management services, which represent three roles of the tactical security.

Identity management services – responsible for assigning and maintaining digital identities and associated attributes across the electronic computing environment and for deleting identities when they no longer represent valid users of the environment.

Access management services – responsible for assigning and maintaining resource access privileges across the electronic computing environment and for terminating those privileges when they are no longer required. Access management services may encompass a variety of components such as access policy definition, account creation, and access control list (ACL) maintenance.

Configuration management services – responsible for consistently setting and maintaining the security configuration across the electronic computing environment. Configuration management is where the eHealth security technology extends to include distributed components for all security services. Configuration of the various security services should include; boarder protection, threat detection, content control, auditing, cryptography, and even configuration management itself.

b. ***Logical Architecture*** – identifies discrete logical services and their relationships at the level required to determine what the health organisation needs to build or buy to construct a set of identity management services for the digital health environment. It entails;

Human resource system – provides administrative feeds to create or update internal user identities in the internal entities directory.

External identity administration system – creates or updates external user identities in the external entities directory on behalf of affiliated enterprises. User administration may be delegated to an administrator at the affiliate enterprise site.

Identity registration and vetting functions – provide the means for establishing digital identities for persons that might not go through the human resource system, such as contractors or consultants. Additional functions may be included to support special identity attributes, such as security clearances or citizenship, which may be provided by external organisations other than internal human resource system.

c. ***Physical Architecture*** – physical implementation of servers, software, network connections, etc., of identity management environment described by the logical architecture. This architecture environment should have multiple documents, including; Software component layering on servers and network topology diagrams, all network addresses of Domain Controllers in an environment, and documentation of the configuration settings for software components.

d. ***Design and Development*** – these identify the types of guidance that organisations may want to provide to those responsible for design, development, and deployment of applications. Design and development guidance may range from overall process guidelines to specific guides, templates, and tools. These may include design patterns, code samples, reusable libraries, and testing tools. All of these are aimed at effective utilization of information security architecture and effective integration into the environment. This guidance can be applied to

components or applications built in-house as well as commercial off-the-shelf (COTS) applications selected for integration.

4. **Security Operations** – these define the processes required to support a policy driven security environment. This domain consists of two key processes major categories; one category comprises the administration, compliance, and vulnerability management processes required to ensure that the technology as deployed conforms to policy and provides adequate protection to control the level of risk to the environment; while the other category consists of the administration, event, and incident management processes required to enforce policy within the environment. Further guidance on how these can be addressed can be found in ISO 27002. The security operations domain of MoH's DHISAF has a strong dependency on asset management. They entail;

Asset Management – a component and process for maintaining the inventory of hardware and software assets required to support device administration, compliance monitoring, vulnerability scanning, and other elements of security operations. Common components include a repository of hardware and software assets (including the configuration and usage information), a capability to discover assets as they are added to the network, and reporting capabilities (ISO/IEC, 2018). This information may be used for activities e.g., contract renewals, software license compliance audits, and cost reduction activities. This requires that health organisations identify all assets associated with information and processing facilities and also define appropriate protection responsibilities. An inventory of these assets should be drawn up and maintained on a regular basis. ISO/IEC 27005 provides examples of assets that might need to be considered by health organisations when identifying assets.

Security Administration – the components and processes for securing the health organisation's operational digital assets against accidental or unauthorised modification or disclosure. This is accomplished by planning, coordinating, and implementing the Digital Health systems and best practices required to create and maintain secure access to resources and protect the integrity of the system and device configurations. Security administration comprises two primary sub-components namely; 1). Identity management (responsible for the creation, modification, and termination (inactivation or deletion) of digital identities, including the workflow process for managing both identity and access management information. It is also responsible for management of authentication tokens and certificates); 2). Device configuration (responsible for technical standards instantiation at the device level. It is also responsible for ensuring that updates to the actual devices are reflected in the asset database).

SECTION 2: DIGITAL HEALTH STANDARDS FOR UGANDA

2.1. DATA AND INTEROPERABILITY STANDARDS

Figure 12 shows the contextual data and interoperability standards that shall be applied to ensure that the patient (health) data collected, information processed, shared, and stored are in formats that support both semantic and syntactic interoperability. Data and interoperability standards include medical coding, data exchange and sharing standards. These comprise of six (6) dimensions; Patient Identification, Health Information Exchange Registries, Compliance, Management and Communication of Medical Imaging Data, Health Information Systems, Digitization of Health Information, and the crosscutting Security and Privacy for Data and Training and capacity building standards.

These standards are contextualised from several resources including; the Uganda Protection and Privacy Act 2019, (HL7) Health Level Seven (Lin et al., 2012), (FHIR) Fast Healthcare Interoperability Resources (Lin et al., 2012) and (DICOM) Digital Imaging and Communications in Medicine (Greenwell & Salentine, 2018; Saripalle et al., 2019), (HL7 Service-Aware Interoperability Framework: Canonical Definition Specification, Release 2 Draft Standard for Trial Use (2012); HL7_FHIR_DCOM Standards, 2022), Interoperability Framework. The e-Government Interoperability Framework Reference Architecture (GIRA) for Uganda was also used as a reference architecture focused on the design of end-to-end interoperable digital public services. The GIRA is composed of the most salient Architecture Building Blocks (ABBs) needed to promote cross-sector interactions between Ministries Departments, Agencies (MDAs) and Local governments (LGs).

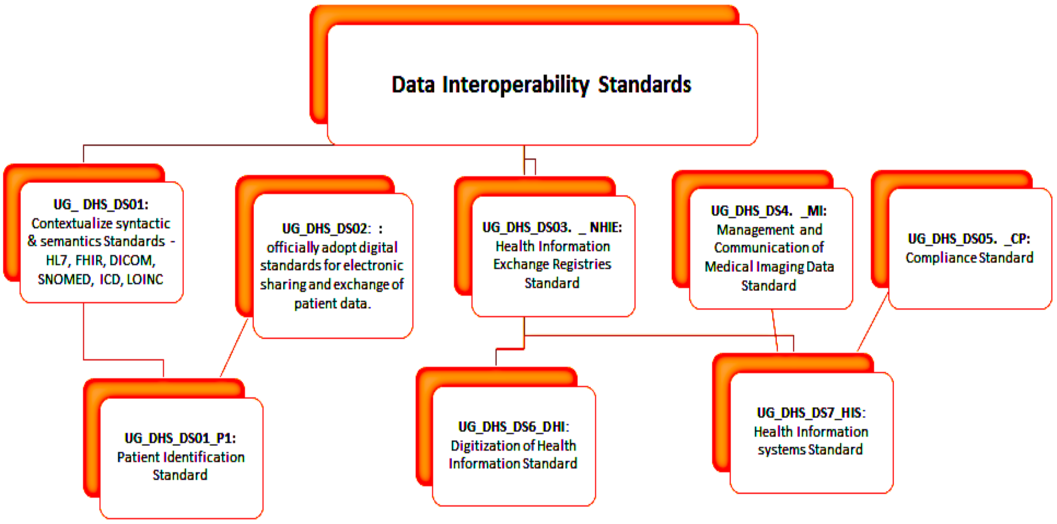

Figure 12. Contextualised Data and Interoperability Standards

UG_DHS_DS01_P1: Patient Identification

> ***Statement***: Establish a unique, standardised, comprehensive, and comprehensible Electronic Medical Record (EMR), Electronic Health Records (EHR), and Personal Health Record (PHR).

Rationale

UG_DHS.DS01_P1_Rt1: A unique identifier in Digital Health services will provide for accurate identification of a given patient across a continuum of care to address the priority business needs of patients/clients, healthcare providers, and healthcare managers by improving efficiency, effectiveness, data management and utilization, and resource planning. All health record systems must therefore adhere to standards for capturing information related to patient demography and identifiers.

Implementation Specification(s)

UG_DHS_DS01_P1_Rt1_IS1: Adopt and enforce a Unique Identifier for every Patient/Client. This may include the National Identification Number (NIN) and the Legally Resident Alien Identification Number (AIN) as the Unique Identifier of every Patient/Client or any other nationally acceptable identifier in circumstances where patients/clients do not have NINs or AINs such as children and non-registered aliens.

UG_DHS_DS01_P1_Rt1_IS2: Identify and analyze all initiatives by different stakeholders towards the design of EMR, EHR, and PHR with a view of its utilization.

UG_DHS_DS01_P1_Rt1_IS3: Design, adopt, and enforce EMR, EHR, and PHR that conform to National and international standards.

UG_DHS_DS01_P1_Rt1_IS4: Monitor and evaluate the adherence and conformance to the unique identifier standard.

UG_DHS_DS01_P1_Rt1_IS5: All eHealth systems that collect patient-level data should have provision for the following minimum identifiers (e.g., Name, date of birth, gender, NIN etc.).

UG_DHS_DS02. _ NHIE: Health Information Exchange Registries Standard

***Statement*:** Developing and maintaining comprehensive interoperable master lists of health facilities, providers, health products, and patients/clients is a necessary step toward monitoring health infrastructure and services that form a core component of the national Health Management Information System (HMIS).

Rationale

UG_DHS_DS02. _ NHIE_Rt1: For a health record system to function properly in a larger ecosystem of other components there have to be communication protocols to enhance sharing of data to realize a continuum of care.

Implementation Specification(s)

UG_DHS_DS02. _ NHIE_Rt1_IS1: Provide a protocol for electronic exchange of data in healthcare environments.

UG_DHS_DS02. _ NHIE_Rt1_IS2: Data should be readily available and accessible as and when it is required by authorised users.

UG_DHS_DS02. _ NHIE_Rt1_IS3: Shall enable healthcare planning and financial management to ensure effective collection, allocation, and use of health financial resources at all levels by health plan priorities

UG_DHS_DS02. _ NHIE_Rt1_IS4: Shall define specifications for information object definitions, data structures, and their semantics, protocols for the exchange of medical information among imaging equipment and other healthcare applications, file format, and storage of medical images.

UG_DHS_DS02._NHIE_Rt1_IS5: Shall enable the exchange of clinical data between systems to support a central patient care system as well as a more distributed environment where data resides in departmental/decentralized systems.

UG_DHS_DS02._NHIE_Rt1_IS6: Enable the exchange of clinical and administrative data among healthcare applications in the form of patient demographics, health insurance data, clinical observations, appointment schedules, and patient referrals etc.

UG_DHS_DS02._NHIE_Rt1_IS7: Strengthen healthcare professionals' human resource management to ensure effective information management, assignment, development, and accountable use of health human resources at all levels following health plan priorities.

UG_DHS_DS03. _CP: Compliance

Statement: Establish an Intelligent and Integrated monitoring mechanism to ensure compliance across the health sector. Generate data for observation and clinically relevant events and encounters. This requires having a common semantic and syntactic logical information model and structural composition.

Rationale

UG_DHS_DS03. _CP_Rt1: Without the right data standards being implemented wrong communication will be realised in the whole ecosystem. Therefore, a health record system must conform to agreed standards.

Implementation Specification(s)

UG_DHS_DS03. _CP_Rt1_IS1: Generic implementation should be harmonized with the design requirements and preferred technology platforms.

UG_DHS_DS03. _CP_Rt1_IS2: Monitor systems against specifications for the structure and semantics of health data to support a common representation of recommended standards e.g., clinical summaries, discharge notes, and radiology reports, etc.

UG_DHS_DS03. _CP_Rt1_IS3: Establish a monitoring system to support inspection and compliance monitoring of electronic data, information and knowledge management, analysis and utilization System.

UG_DHS_DS4. -_MI: Management and Communication of Medical Imaging Data

Statement: This includes all health record management in terms of imaging modalities including radiography, magnetic resonance imaging (MRI), nuclear medicine, ultrasound, tomography, echocardiography, X-ray, CT, and other modalities used in radiology, cardiology, radiotherapy, ophthalmology, and dentistry.

Rationale
UG_DHS_DS4. -_MI-_ Rt1: For comprehensive handling and managing of images (series or single), waveforms (such as those in ECG/EEG), audio (such as those in digital stethoscope), and video (such as those in the endoscope, ultrasound, etc.) data in medicine. Where required and relevant, other features of standards such as services, display, print, and workflow may be implemented.

Implementation Specification(s)
UG_DHS_DS4.-_MI-_Rt1_IS1: Data elements must serve the purpose of documentary records of various diagnostic and prescriptive data or information generated.

UG_DHS_DS4. -_MI-_ Rt1_IS2: Medical equipment procured must comply with the universal format for PACS (picture archiving and communication systems) image storage and transfer Scanned or Captured Records:

- Image: JPEG lossy (or lossless) with size and resolution not less than 1024px x 768px at 300dpi
- Audio/Video file document Management formats should comply with Long-Term Preservation

UG_DHS_DS4. -_MI-_ Rt1_IS3: Where no maximum image resolution has been prescribed, a sufficiently acceptable limit may be used to avoid unnecessarily large files that do not aid in correspondingly better interpretation or analysis.

UG_DHS_DS4. -_MI-_ Rt1_IS4: Preference in the procurement of IT hardware and software for image data management shall be from an authorised dealer licensed and accredited by NITA-U or on the PPDA Register of Providers and comply to the DCOM/ PACS Standards.

UG_DHS_DS5_HIS: Health Information systems Standard

Statement: Establish mechanisms for implementing and adhering to set guidelines for all HIS solutions in the health sector

Rationale
UG_DHS_DS5_HIS_Rt1: The HIS guidelines will support the collection, quality review, aggregation and reporting of health-related data for clinical and system-wide use in a timely, and reliable manner.

Implementation Specification(s)

UG_DHS_DS5_HIS_Rt1_IS1: Define data, information and knowledge lifecycle responsibilities and requirements (e.g., Processes, Data Ownership / Stewardship, Audit, Creation and Collection, Analysis, Retention and Disposal, Data Quality, etc.).

UG_DHS_DS5_HIS_Rt1_IS2: Develop a "Right to Use" Policy - Defining the purposes for which health data can be used by providers in the direct delivery of care to the patient (e.g., "Need to Know" rules).

UG_DHS_DS5_HIS_Rt1_IS3: Develop Data, Information and knowledge Sharing Agreements. Defining the purposes for which health data may be shared between organisations and custodians, observing terms and responsibilities.

UG_DHS_DS5_HIS_Rt1_IS4: Define requirements for data collection, storage, analysis, and reporting and dissemination mechanisms.

UG_DHS_DS5_HIS_Rt1_IS5: Identify data sources, storage and flow patterns that enable timely and accurate collection and information exchange.

UG_DHS_DS5_HIS_Rt1_IS6: Develop a reporting process that is aligned to the requirements at all relevant levels.

UG_DHS_DS5_HIS_Rt1_IS7: Develop guidelines, SOPs, and manuals for data use and utilization for ownership, usage to influence decision-making processes at different levels (clinicians, data managers, clients, researchers, policymakers, partners, etc.).

UG_DHS_DS6_DHI: Digitization of Health Information Standard

Statement: Establish an ICT infrastructure that forms the foundations for electronic communication and information/data sharing across geographical and health-sector boundaries. This includes the computing infrastructure, databases, directory services, network connectivity, and storage that underpin a national digital Health environment.

Rationale

UG_DHS_DS6_DHI_Rt1: Improving the quality of health care services in terms of efficiency, availability, accessibility, reliability and timeliness to ease interoperability of patient information across the health facilities.

Implementation Specification(s)

UG_DHS_DS5_HIS_Rt1_IS1: Establish a cost-effective and affordable ICT infrastructure to support communication and sharing of information/data across the continuum of care.

UG_DHS_DS5_HIS_Rt1_IS2: Strengthen the electronic health information system (HIS) to support evidence-based health care and decision making.

UG_DHS_DS5_HIS_Rt1_IS3: Enable electronic delivery and interventions of health services in line with the universal health coverage to all Ugandans.

UG_DHS_DS5_HIS_Rt1_IS4: Implement a National Minimum Health Care Package (UNMHCP) which includes promotive, preventative, curative, rehabilitative, and palliative care.

UG_DHS_DS5_HIS_Rt1_IS5: Shall enable electronic delivery of quality health care (mHealth, telehealth...) to all individuals in remote areas lacking needed expertise to reduce isolation, amplify the voices of the disadvantaged, and provide means to individuals to influence health systems.

UG_DHS_DS5_HIS_Rt1_IS6: Strengthen disease prevention, surveillance, and control by using hybrid ICT solutions to facilitate early detection and rapid reporting and response.

2.2 DIGITAL HEALTH COMMUNICATION INFRASTRUCTURE STANDARDS

The Digital Health Communication Infrastructure (DHCI) standards in **Figure 13** have five dimensions; hardware devices, connectivity and communication networks, applications and software, security and privacy, and facilitating resources (human resources, financial resources, and electric power). These standards are contextualised from several resources including; Uganda Data Protection and Privacy Act 2019, (ANSI/BICSI & Institute, 2018; EHRS FM R2; European Parliament, n.d.; European Parliament, Council of the European Union, 2004; European Union, 2015, 2019; Gary et al., n.d.; HHS Office of Civil Rights, 2013; HIPAA, 2013; ISO, n.d., 2017; ISO IS 17090-1 2013; ISO IS 17090-2 2008; ISO IS 22600-1 2014; ISO IS 27799 2008; ISO/IEC 27033-4:2014; ISO/HL7, n.d., n.d., p. 710781; ISO/IEEE, n.d.; ISO/TR, 2009; ITI Planning Committee, 2015; ITU-T, 2015; MoICT-Uganda, 2019; NITA-U, 2010; Singapore Ministry of Health, 2015; The Open Group, 2018; TR-42.1, 2017).

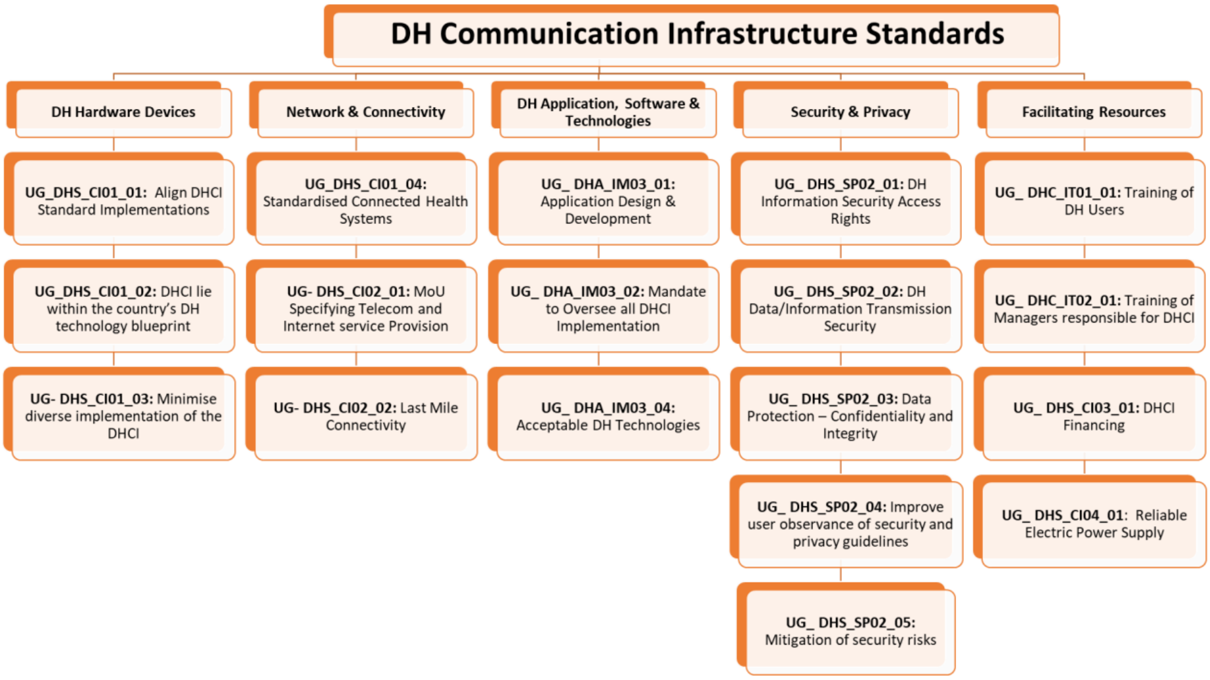

Figure 13. Contextualised DHCI Standards

2.2.1. HARDWARE DEVICES STANDARDS

UG_DHS_CI01_01: Align DHCI Standard Implementations

Statement: Critical areas and or changes in the DHCI are appropriately aligned to the country's digital health needs, Health Information and Digital Health strategy, and related policies.

Rationale

UG_DHS_CI01_01_Rt1: Changes in the DHCI are continuously researched, guided and well documented based on current DH user needs.

Implementation Specification(s)

UG_DHS_CI01_01_Rt1_IS1: MoH and participating health facilities and supporting organisations should procure and implement components of the DHCI following the MoH and related MDAs guidelines, including but not limited to;

(i) Requirements specifications for each component of the DHCI, technologies, and use, lie within Uganda's digital health technology blueprint.

(ii) The MoH and healthcare facilities/ organisations supporting health interventions procure and implement end-user ICT devices with adequate hardware (HW) and software (SW) specifications and networking devices to meet health data quality requirements.

(iii) MoH should ensure that appropriate data storage devices or data backup mechanisms are provided to all healthcare service delivery points. Additionally, MoH should ensure that each participant in the connected health

system implements the different types of data backup procedures for differential and replication.

(iv) All healthcare service delivery points implement scheduled maintenance, repair, and environmentally friendly end-of-life and disposal of DHCI waste.

UG_DHS_CI01_01_Rt1_IS2: Government policy guidelines for operationalisation of the DHCI are implemented at all levels of Uganda's health system.

UG_DHS_CI01_02: DHCI lie within the country's DH technology blueprint

Statement
Minimum specifications of HW devices restrict implementations of the DHCI within the country's DH technology blueprint that assures healthcare facilities/ organisations participating in the connected health system establish a DH communication network with quality devices, data rate, and network services.

Rationale
UG_DHS_CI01_02_Rt1: Hardware devices should be able to support all forms of approved DH applications, health data, and information being captured, processed, presented and or shared.

Implementation Specification(s)
UG_DHS_CI01_02_Rt1_IS1: ***Desktop Specifications*** **-** Processor family: I3 or Equivalent; Processor Speed: 2.2 Ghz; Cores: 2; Processor Cache: 2 mb; Processor Speed: 3 Ghz; Memory: 8 GB DDR4 Expandable; Storage: 256 GB SSD Expandable; Power Supply: 220 V; Network Controller: Ethernet and Wireless Support; Display: 17-inch Monitor, and HDMI/ VGA.
UG_DHS_CI01_02_Rt1_IS2: ***Laptop Specifications*** **-** Processor family: I3 or Equivalent; Processor Speed: 2.2 Ghz; Cores: 2; Processor Cache: 2 mb; Processor Speed: 3 Ghz; Memory: 8 GB DDR4 Expandable; Storage: 256 GB SSD Expandable; Power Supply: 220 V; Network Controller: Ethernet and Wireless Support; Ports: HDMI, USB Ports – 3; Battery Life: Minimum 5 hours; and Display: 13 - 17 Inch.
UG_DHS_CI01_02_Rt1_IS3: ***PDA/IPAD/TAB*** - Processor Speed: 2 Ghz; Memory: 4 GB; Storage: 32 GB; Display: 10 inch tablet; Camera Min: 2 MP; Battery Life: Minimum 5 Hours; Ruggedness: Min Drop height 70 cm; and Connectivity: Mobile data support, Mi 4G/LTE/GSM Connectivity, Wifi, Hotspot; SIM: Nano-SIM; DISPLAY Type: TFT capacitive touchscreen, 16M colours; Bluetooth: 5.0, A2DP, LE GPS: Yes, with A-GPS, GLONASS, GALILEO, BDS USB: 2.0, Type-C 1.0 reversible connector FEATURES: Sensors Accelerometer, gyro, compass.

UG_DHS_CI01_02_Rt1_IS4: Local **d**ata backup medium should be sufficient to meet the health facility needs

(i). National Referral Hospitals……10Tb
(ii). Regional Referral Hospitals ….....5Tb
(iii). General Hospitals ……………….2Tb
(iv). HC IV & III ………………….…..1Tb

UG_DHS_CI01_02_Rt1_IS5: Biometric Fingerprint Reader- Connection: USB 2.0; Operating System's: Microsoft Windows (32-bit and 64-bit), Linux (32-bit and 64-bit), Android; Resolution: 512 ppi; Image capture area (Platen size): 15 x 18 mm (0.6″ x 0.7″); Sensor type: Optical; Illumination: Blue LEDs; Device size: 65 x 36 x 16 mm (2.6″ x 1.4″ x 0.6″); Operating temperature: 0°C ~ +40°C; Operating humidity: 20-80 (non-condensing).
UG_DHS_CI01_02_Rt1_IS6: Raspberry Pi – Processor Speed: 2 Ghz; Memory: 8 GB DDR4; Storage: 128 GB; SSD Expandable Display: 17-inch LED Monitor; HDMI & VGA Ports; Case for Raspberry Pi 4 with Fan, Vesa Mount, USB Keyboard, USB Mouse.

UG_DHS_CI01_02_Rt1_IS7: Other ICT Devices-Printers, Scanners and Photocopiers should be fast with high-resolution prints/scans. They should all support high-quality resolution of at least 200 DPI.

UG_DHS_CI01_02_Rt2: Software running on the devices should be licensed and support installation (be compatible) of other DH applications.

Implementation Specification(s)
UG_DHS_CI01_02_Rt2_IS1: Min OS & Runtime environment -Microsoft OS: Windows 8.1 classic & later or macOS 10.14 (Mojave); Web Browser: Microsoft Internet Explorer 11 & later, Microsoft Edge or Safari 12, Google Chrome of 2010 and above, Mozila Firefox 4 and above.

Java Runtime Environment (JRE): Oracle JRE 8 up to 8u231. For macOS, JRE 8 or later versions for the smart card reader.

UG- DHS_CI01_03*:* Minimise diverse implementation of the DHCI

Statement
Reduce diversity of DHCI implementations that support healthcare programmes and interventions through procurement and implementation based on contextualised global standards and minimum specifications laid down by MoH and related MDAs.

Rationale
UG- DHS_CI01_03_*Rt1*: The MoH enforces implementation and use of interoperable DHCI and applications capable of facilitating the exchange of health data/information across all health system levels.

Implementation Specification(s)
UG- DHS_CI01_03_*Rt1_IS1:* Adopt and use ANSI/TIA-1179 - A standard for health communication infrastructure to support best practices in the design and implementation of the DHCI that facilitate intra- / inter-facility exchange.

UG- DHS_CI01_03_*Rt1_IS2:* ICT devices used for handling DH data should be housed in an environment that meets the requirements of a Tier 2 data centre (TIA-942 Telecommunications Infrastructure Standard for Data Centres[1]), including;

(i). Cooling and Temperature: Ambient room temperature of 15-32 degree Celsius; Fire detection and Suppression;

(ii). Physical Security: Two Factor authentication for access e.g., (Pin and biometric fingerprint); Secure door; Surveillance with Intrusion detection; and Fire detection and Suppression

(iii). Power supply and Backup: 48 hours backup time (Time to recover from an incident on main power supply); and Clean uninterrupted power supply.

2.2.2. COMMUNICATION NETWORKS & CONNECTIVITY STANDARDS

UG_DHS_CI01_04: Standardised Connected Health Systems

Statement

Healthcare facilities/ organisations participating in the connected health system should establish DH communication network(s) with quality devices, data rate, & suitable network services to support secure HIE.

Rationale

UG_DHS_CI01_04_Rt1: All participating health organisation/ facilities implement DHCI that meet the required hardware, software, network, and security specifications for quality health data.

Implementation Specification(s)

UG_DHS_CI01_04_Rt1_IS1: MoH, telecommunication organisations should provide fast and reliable network connectivity to all service delivery points, both LAN and the Internet (that uses the networking protocols: TCP/IP Version 4, VPN, & WLAN-802.11) to ensure reliable connectivity that supports all modes of HIE. We recommend that ANSI/TIA-1179-A be adapted to include clauses stipulating that;

(i) All health service delivery points have procured end-user ICT devices with at least min. specifications as laid down in the "**ICT INFRASTRUCTURE STANDARDS SPECIFICATION DOCUMENT**".

(ii) Communication HW, SW, computer networks, and migration middleware should conform to MoH and related MDAs defined standards to ensure interoperability between systems and devices participating in the connected health system.

(iii) Healthcare sites/healthcare organisations must ensure the reliability and security of the health data/information being exchanged over the communication network. Health data sharing policies and procedures should also ensure infrastructure capability to securely support information sharing.

---

[1] A data centre is a facility used to house computer systems and associated components, such as telecommunications and storage systems. It generally includes redundant or backup power supplies, redundant data communications connections, environmental controls (e.g. air conditioning, fire suppression and security devices)

(iv) Guidelines for DH HW, SW and Communication Network, must be fully documented and made public (identifiable) for all healthcare stakeholders to have a copy that they can follow.

(v) The mechanisms for monitoring compliance of different DH communication network implementation against MoH and related MDA's defined standards should be in place.

UG_DHS_CI01_04_Rt1_IS2: Each entity participating in the connected health system should comply with MoH's minimum specifications for connectivity devices of their health system level.

(i). **Firewall**: CPU Cores: 4 Core; Physical Network I/O ports: (1) 10GB SFP), (4) 1 Gbps RJ-45 "direct" (unswitched) ethernet (LAN)/(WAN); Storage 16 GB - upgradable; Memory: 8 GB DDR4; Console: Port 1 console port; USB Ports: 2x USB 3.0; Mounting: Optional wall mount kit; Functionality features: DNS Server, DHCP Server, Proxy, IDS/IPS, Packet capture/inspection, NAT & PAT, QoS, Software load balancer, Traffic monitoring, Traffic logging, statistics, and graphs, Traffic shaping, VLAN, Wake-on-LAN, and Web filtering.

(ii). **Switches**: (a) Managed switches - Ports: 8/ 16/ 24/ 48; Networking Interfaces: 10/100/1000 Mbps RJ45 Ports AND 1 Gbps SFP Ports; PoE: Output (RJ45 Ports) IEEE 802.3at/af; Operating Temperature: -5 to 45° C (23 to 113° F); Power Method: 100-240VAC/50-60 Hz, Universal Input preferably dual power supply; and Other Considerations include L3 support for 24/ 48 port switches, GUI for configurations to ease support or Rack Mountable. (b) Unmanaged switches - Ports: 8/ 16/ 24; Networking Interfaces: 10/100/1000 Mbps RJ45 Ports AND 1 Gbps SFP Ports; Operating Temperature: -5 to 45° C (23 to 113° F); Power Method 100-240VAC/50-60 Hz, Universal Input; and Other Considerations include Rack-mountable.

(iii). **Access Points**: (a) Indoor Access Points – Wi-Fi Standards: 802.11 a/b/g/n/ac/ac; Networking Interface: RJ45 GbE; Wireless Security: WPA-PSK, WPA-Enterprise (WPA/WPA2/WPA3, TKIP/AES); Operating Frequency: 2.4 GHz, 5 GHz; Throughput: 2.4GHz- 300 Mbps, 5GHz- 1733 Mbps; Operating Temperature: -5 to 45° C (23 to 113° F); Power Method: 100-240VAC/50-60 Hz, DC Power adapter, P.O.E; and Other Considerations: Mounting Wall/Ceiling, Mesh capability, 50 users. (b) Outdoor Access Point – Wi-Fi Standards: 802.11 a/b/g/n/ac/ac; Networking Interface: (1) RJ45 GbE; Wireless Security: WPA-PSK, WPA-Enterprise (WPA/WPA2/WPA3, TKIP/AES); Operating Frequency: 2.4 GHz, 5 GHz; Throughput: 2.4GHz- 300 Mbps, 5GHz- 1733 Mbps; Operating Temperature: -5 to 45° C (23 to 113° F); Power Method: 100-240VAC/50-60 Hz, DC Power adapter, P.O.E; and Other Considerations including Mounting Wall/Ceiling, Mesh capability, Water and dust resistant, 50 users.

(iv). Server Specifications and Rack: (a) Regional Referral Hospital – Rack Size: 42 U; Processor family: Xeon; No. of Processors: 1 or 2; Cores: 8; Processor Cache: 8 mb; Processor Speed: 3 Ghz; Power Supply: 2 flexible power supplies; Memory: 64 GB DDR4; Storage: 1 TB x 4 Disks - Raid configurable; Network Controller: 1GBps, 4ports Ethernet, 2 SFP+ 10 GBps ports; Rackmount Console with KVM Switch: 19 inch

display, LED back lit, Touchpad and keyboard, VGA and HDMI support, USB ports; and Other Considerations include Rack Mountable with rail kit, Warranty 3 years, Windows Server 2019 licence, PDU. (b) General Hospital - Rack Size: 25 U; Processor family: Xeon; No. of Processors: 1 or 2; Cores: 8; Processor Cache: 8 mb; Processor Speed: 3 Ghz; Power Supply: 2 flexible power supplies; Memory: 32 GB DDR4; Storage: 1 TB x 2 Disks - Raid configurable; Network Controller: 1 GBps, 4 ports Ethernet, 2 SFP+ 10 GBps ports; Rackmount Console with KVM Switch: 19 inch display, LED back lit, Touchpad and keyboard, VGA, HDMI support, USB ports; and Other considerations such as Rack Mountable with rail kit, Warranty 3 years, Windows Server 2019 licence, PDU. (c) Health Centr IV – Rack Size: 15 U; Processor family: Xeon; Cores: 2; Processor Cache: 2 mb; Processor Speed: 3 Ghz; Memory: 16 GB DDR4; Storage: 1 TB; Network Controller: 2 ports Ethernet; Display: 19-inch display, LED back lit, Mouse and keyboard, VGA, HDMI support, USB ports; and other considerations like the Microserver size, Warranty of atleast 3 years, Windows Server 2019 licence, and PDU. (d) Health Centre III – Rack Size: 15 U; Processor family: i7; Cores: 2; Processor Cache: 2 mb; Processor Speed: 3 Ghz; Memory: 16 GB DDR4; Storage: 1 TB; Network Controller: Ethernet; Display, KVM: 19-inch display, LED back lit, Mouse and keyboard, VGA, HDMI support, USB ports; and other considerations like Microserver size, at least a warranty 3 years, Windows Server 2019 licence, and PDU.

UG_DHS_CI01_04_Rt1_IS3: All participating healthcare organisations implement data communication networks and infrastructure that support QoS, Security and Privacy.

(i). Implementations of guided networking/transmission media should be based on Cat3 and higher, and Fiber Optic (preferred because of higher data rate and security).

(ii). Healthcare cites /devices and networks should support VPN and TCP/IP networking protocols and emerging Bring Your Own Device (BYOD).

(iii). The wireless LAN should follow the 802.11 protocol suite.

(iv). Healthcare sites should be connected to MoH designated Cloud Service(s) and Data Centre(s).

UG- DHS_CI02_01: MoU Specifying Telecom and Internet service Provision

Statement

Any form of MoU and SLA (as part of the social corporate responsibility) between telecom and Internet service providers should specify both quality and terms of service provision to Uganda's health sector.

Rationale

UG- DHS_CI02_01_Rt1: There is reliable and affordable (subsidised) connectivity to support all forms of HIE in healthcare.

Implementation Specifications

UG- DHS_CI02_01_Rt1_IS1: An MoU is established between MoH and Telecom/Internet Service Providers (ISPs) stipulating the quality and conditions for Internet services to be provided to support healthcare interventions in Uganda.

UG- DHS_CI02_01_Rt1_ *IS2:* The MoU with telecom and Internet service providers should specify and establish a mechanism for monitoring QoS for health programs in Uganda.

UG- DHS_CI02_01_Rt1_ *IS3:* *Other Arrangements.* The MoH and related MDAs have structures and a mechanism for monitoring QoS provided by Internet service providers who have agreements to connect healthcare service delivery points.

UG- DHS_CI02_02: Last Mile Connectivity

Statement

NITA-U should connect all health facilities to the National Backbone Infrastructure.

Rationale

UG- DHS_CI02_02_Rt1: Government Health facilities and Service delivery points are connected to the National Data Transmission Backbone Infrastructure.

Implementation Specifications(s)

UG- DHS_CI02_02_Rt1_IS1: MoH should determine and prioritise connectivity needs for all healthcare sites in Uganda

UG- DHS_CI02_02_Rt1_IS2: NITA-U should follow the MoH priority list for connecting healthcare facilities and service delivery points to the national Backbone.

2.2.3. APPLICATION, SOFTWARE AND TECHNOLOGIES STANDARDS

UG_ DHA_IM03_01: Application Design & Development

Statement

DH application designing and development is guided by agreed standards and guidelines as contextualised by MoH and related MDAs, which specify modes for linking (sharing) health data between applications in a secure manner.

Rationale

UG_ DHA_IM03_01_Rt1: Design, development, and deployment of DH applications are guided by standards that specify modes for linking (sharing) health data from across DH applications in a secure manner.

***Implementation Specification*(s)**

UG_ DHA_IM03_01_Rt1_IS1: MoH should develop and enforce guidelines and standards required for the development and use of standardised DH applications.

UG_ DHA_IM03_01_Rt1_IS2: Development of DH applications should demonstrate alignment with approved/contemporary software development life cycle.

UG_ DHA_IM03_01_Rt1_IS3: The application interface design and features are consistent across all DH applications being adopted/developed for use in healthcare interventions/programs in Uganda.

UG_ DHA_IM03_01_Rt1_IS4: DH applications should follow data interoperability standards

UG_ DHA_IM03_02: Mandate to Oversee all DHCI Implementation

Statement

MoH superintends the implementation and use of DH applications in public and private healthcare sites.

Rationale

UG_ DHA_IM03_02_Rt1: The MoH has mechanisms for regulating development and deployment of DH applications for healthcare services in Uganda.

Implementation Specification(s)

UG_ DHA_IM03_02_Rt1_IS1: MoH directs the acquisition of all DH applications and or owns them after rigorous testing and approval for use in the health sector.

(i). The MoH tests, approves, and maintains a database of all DH applications in the country

(ii). The MoH, where possible, should own or be a party to the Intellectual Property Rights (IPR).

(iii). The applications and or software may be supplied or availed by the Government or commercially

UG_ DHA_IM03_02_Rt2: MoH superintends DH infrastructure/ Applications implementation and use at both public and private health facilities.

Implementation Specification(s)

UG_ DHA_IM03_02_Rt12_IS1: The MoH ensures that an agreement addressing detailed protocols for data collection, transmission, processing, storage and use is signed between all healthcare sites/organisations involved.

UG_ DHA_IM03_02_Rt2_IS2: MoH supervises the implementation of guidelines on use of the DH applications and data that they hold.

UG_ DHA_IM03_04: Acceptable DH Technologies

Statement

Control the choice of DH technologies to be adopted and used by healthcare organisations, facilities, and researchers to ensure interoperable and secure collection, sharing and use of health data/information.

Rationale

UG_ DHS_IM03_04_Rt1: MoH should determine and enlist acceptable (including emerging) ICT technologies that healthcare participating entities can adopt to support healthcare processes in Uganda.

Implementation Specification(s)

UG_ DHS_IM03_04_Rt1_IS1: The MoH has a database of approved and listed ICT technologies that can be used in the country. Examples of such technologies are;

i. Internet of Things (IoT) which is a collection of devices, services, and software that integrates data received from various connected devices for patient monitoring or health management e.g., Body area Networks
ii. Big data technologies
iii. mHealth, Telemedicine, etc
iv. Blockchain technologies
v. Artificial Intelligence (AI)

UG_ DHS_IM03_04_Rt1_IS2: MoH has criteria for evaluating implementations of these technologies. The evaluation criteria should support scoring for continued use or decommissioning of such ICT technologies in Uganda's health system.

UG_ DHS_IM03_04_Rt1_IS2: The MoH has a safe migration policy and procedure for decommissioning the technologies once they have been considered outdated/inappropriate for Uganda's healthcare environment.

2.2.4. DHCI's FACILITATING RESOURCES STANDARDS

UG_ DHC_IT01_01: *Training of DH Users*

Statement

The MoH, collaborating medical training institutions, and the DH implementing partners should aim at producing an all-rounded, knowledgeable, and skilled digital health workforce capable of observing stipulated guidelines.

Rationale

UG_ DHC_IT01_01_Rt1: Ensure all rounded, knowledgeable, and skilled DH health workforce for improved service delivery.

Implementation Specification(s)

UG_ DHC_IT01_01_Rt1_IS1: Training institutions have revised/updated/developed their curricula with courses aimed at producing all-rounded DH with expertise in using DH applications and supporting CI.

UG_ DHC_IT01_01_Rt1_IS2: DH workers are trained to use existing DH applications and support CI under strict observance of health data/information security and privacy guidelines.

UG_ DHC_IT01_01_Rt1_IS3: The MoH should stipulate the required minimum DH competencies for the different digital health workforce/roles.

UG_ DHC_IT01_01_Rt1_IS4: Incorporate the basic criteria of DH competency into recruitment material and processes to help attract and hire healthcare workers with the appropriate educational background and experience to use the DH technologies.
UG_ DHC_IT01_01_Rt1_5: Develop a competency assessment tool or skills assessment based on the Competency Framework or another benchmark. The competency assessment tool can be used to complete a periodic skills assessment of the health workers' activity to identify gaps (training needs assessment).

UG_ DHC_IT02_01: Training of Managers responsible for DHCI

Statement

Leaders/managers responsible for DHCI are informed and trained on the use of DH technologies and trends.

Rationale

UG_ DHC_IT02_01_Rt1: Leaders/managers responsible for DHCI are made aware of emerging technologies and trends to support their decision-making process.

Implementation Specification(s)

UG_ DHC_IT02_01_Rt1_IS1: MoH has developed mechanisms for identifying and documentation of relevant emerging DH technologies and trends.
UG_ DHC_IT02_01_Rt1_IS2: Leaders/managers responsible for DHCI are made aware and trained to use DH technologies to support their tactical and strategic decision-making.

(i). Managers responsible for DHCI are made aware of emerging DH technologies and trends that have been identified.
(ii). Managers/leaders responsible for DHCI have the skills and knowledge required to use DH technologies in decision-making and planning.

UG_ DHS_CI03_01: ***DHCI Financing***

Statement

Adequate funds should be allocated to finance the DHCI that supports DH data collection, processing, and information sharing in Uganda.

Rationale

UG_ DHS_CI03_01_Rt1: Resources are identified and equitably allocated to the critical areas of the DHCI for efficient deployment of DH in a manner that enables the health sector levels (National & Subnational) to acquire and implement the DHCI defined for the different levels by MoH.

Implementation Specification(s)

UG_ DHS_CI03_01_Rt1_IS1: MoH should identify financial resources, including sources of funds and ensure equitable allocation to the critical areas of the DHCI.

UG_ DHS_CI03_01_Rt1_IS2: MoH should ensure the acquisition and implementation of DHCI to the different health service delivery points as defined for their levels.

UG_ DHS_CI04_01: ***Reliable Electric Power Supply***

Statement

Reliable and alternative sources of electric energy are available at all healthcare service delivery points.

Rationale

UG_ DHS_CI04_01_Rt1: Whenever possible, DH systems are powered by electric connections to the national grid and or alternative smart sources of electric energy are made available at all health service delivery points.

***Implementation Specification*(s)**

UG_ DHS_CI04_01_Rt1_IS1: The MoH and related MDAs are required to support the electrification programme of connecting all health facilities to the national power grid.

(i). MoH should engage relevant MDAs responsible for the electrification programme through the provision of documentation of priority health facilities requiring connection to the national grid.
(ii). Relevant MDAs should support the connection of priority health facilities to the national grid.

UG_ DHS_CI04_01_Rt1_IS2: MoH and related MDAs are required to define and provide (when possible) suitable alternative sources of clean energy to health facilities and community-level service delivery points (whether government, for-profit, or not-for-profit healthcare institutions) to power the DH systems to ensure their reliability.

2.2.5. SECURITY AND PRIVACY STANDARDS

UG_ DHS_SP02_01: ***DH Information Security Access Rights***

Statement

DH information security access rights are properly assigned and managed.

Rationale

UG_ DHS_SP02_01_Rt1: Appropriate information access and security clearance levels are assigned (based on the relevancy of their roles or duties) and managed for all members of the workforce and other authorised parties.

Implementation Specification(s)

UG_ DHS_SP02_01_Rt1_IS1: Designate a person responsible for overseeing adherence to security policies and procedures as required by the standard for all participating entities or associates.

UG_ DHS_SP02_01_Rt1_IS2: Regardless of medium, consistently apply and enforce levels of information protection throughout the DH device, technology, or network lifetime.

UG_ DHS_SP02_01_Rt1_IS3: The DHCI data/information security processes should be aligned with national and international security guidelines.

UG_ DHS_SP02_01_Rt1_IS4: Applicable security controls should be put in place to protect electronic data that is resident on DH devices.

UG_DHS_SP02_01_Rt1_IS5: Proper security measures are implemented and strictly enforced for the DH Applications/ISs that manage electronically protected health information to allow access only to those persons or software programs that have been granted access rights.

UG_DHS_SP02_01_Rt1_IS6: Information access management procedures are implemented for authorising access to electronically protected health information that are consistent with assigned access rights.

UG_ DHS_SP02_02: ***DH Data/Information Transmission Security***

Statement

Develop policy-based guidelines for securing the DH data/information transmission.

Rationale

UG_ DHS_SP02_02_Rt1: The MoH and related MDAs responsible for the DH standardisation have developed guidelines for data/information transmission within and between networks and devices.

Implementation Specification(s)

UG_ DHS_SP02_02_Rt1_IS1: There should be proper guidelines and documentation for approving ICT devices to be used on the DHCI network.

UG_ DHS_SP02_02_Rt1_IS2: The MoH and related MDAs responsible for the DH standardisation review security and access requirements and consequently revise them annually.

UG_ DHS_SP02_02_Rt1_IS3: Security requirements should ensure that any third-party user of the personal protected information like a health insurance company must ensure reasonable and appropriate safeguard for electronic data under their custody.

UG_ DHS_SP02_02_Rt1_IS4: Technical security measures are implemented to guard against unauthorised access to electronically protected health information that is being transmitted over an electronic communications network.

UG_ DHS_SP02_03: *Data Protection – Confidentiality and Integrity*

Statement

Enforce measures that provide for data/information confidentiality and protection are implemented and enforced across the entire health sector.

Rationale

UG_ DHS_SP02_03_Rt1: The MoH and participating entities in the health sector ensure the protection and privacy of personally identifiable data that the DHCI contains.

Implementation Specification(s)

UG_ DHS_SP02_03_Rt1_IS1: Measures that provide confidentiality and integrity of health data/information are implemented and enforced across the entire health sector.

(i). Policies and procedures are implemented to protect electronic health information from improper alteration or destruction.

(ii). Ensure confidentiality of personally identifiable data and information at all stages of the HIE.

(iii). DH users should strictly adhere to data protection, and privacy laws and guidelines regarding electronic data collection, processing, retention, and exchange.

UG_ DHS_SP02_03_Rt1_IS2: *Person or entity authentication, Audit controls:* DH systems have M&E and Audit concerning security and privacy risks faced by health data residing on them and how they can be mitigated.

(i). There are mechanisms to authenticate all nodes and keep an audit trail of all system transactions.

UG_ DHS_SP02_04: Improve user observance of security and privacy guidelines

Statement

Users are conversant and comply with DHCI relevant security and privacy laws, and guidelines including Uganda's Data Protection and Privacy Act regarding electronic data collection, processing, retention, and exchange.

Rationale

UG_ DHS_SP02_04_Rt1: There is widespread sensitisation and monitoring to improve user compliance with DHCI relevant security and privacy laws, standards/guidelines.

Implementation Specification(s)

UG_ DHS_SP02_04_Rt1_IS1: Users should be sensitised about existing relevant DHCI security and privacy laws, standards/ guidelines.

(i). DH User compliance with relevant DHCI security and privacy standards/ guidelines is improved through wide sensitization and compliance monitoring.

UG_ DHS_SP02_04_Rt1_IS2: Users observe all relevant DHCI security and privacy laws and guidelines.

UG_ DHS_SP02_04_Rt1_IS3: The MoH shall avail DH implementers and users with proper documentation of security and privacy laws, standards/guidelines including Uganda's Data Protection and Privacy Act.

UG_ DHS_SP02_05: ***Mitigation of security risks***

Statement

DH systems have mitigation plans addressing security risks faced by DHCI and the health data they contain.

Rationale

UG_DHS_SP02_05_Rt1: All entities in the health sector must mitigate, to the extent practicable, any harmful effect that may result from the violation of security policies and guidelines or requirements.

Implementation Specification(s)

UG_DHS_SP02_05_Rt1_IS1: All entities in the health sector should actively participate in the identification, documentation, and reporting of possible security risks to the DHCI.

UG_DHS_SP02_05_Rt1_IS2: There are well-documented DHCI mitigation plans and mechanisms to handle security/privacy risks identified, documented, and reported.

UG_DHS_SP02_05_Rt1_IS3: MoH has a program for periodic technical and nontechnical verification/evaluation of the DHCI security measures to ensure that the security safeguards are effectively implemented and that the safeguards are continually updated in response to new risks or deficiencies.

UG_DHS_SP02_05_Rt1_IS4: A contingency plan is developed and implemented to respond to any emergency or other occurrences like fire, vandalism, system failure, and natural disaster that damages systems that contain health data/information.

SECTION 3: DH WORKFORCE CAPACITY BUILDING FRAMEWORK

A digital health worker (DHW) is a health worker with the capabilities to use digital devices, applications, and or systems. A DH competency model with the corresponding set of standards for human resources development and training regards DH competencies is provided to facilitate health workers to become digitally competent in their work practices.

DH COMPETENCY MODEL

The competency model provides proficiency levels required for the different health work knowledge domains, and Uganda's DHW job roles and required proficiencies. The focus of the assessment criteria aims to train a DHW with the attributes defined in the DH curriculum for the African region, i.e., communicator, collaborator, professional, advocate and manager; data analyst, and technology expert (Alunyu et al., 2020).

DHW JOB ROLES AND REQUIRED PROFICIENCIES

A DHW can include all individuals working in healthcare/third-party health organisations providing healthcare, health facilities, or even in communities (CHWs) who use ICT to provide health services. Depending on their roles and responsibilities in the health sector, these persons require differing levels of knowledge and/or skills to use ICT in health to develop guidelines, standards, or policy and promote use of ICT in health, make decisions regarding deployment, and/or use ICT in the health environment. The DHW domains for training include soft skills, digital health knowledge and use skills, information technology (IT) technical skills, enterprise architecture skills, program or project management skills, and legal environment.

(i). **DH policy and regulation management**: this role entails leading the development, implementation, and maintenance of DH policy, standards, guidelines, and all manner of regulations in the DH space. Some of the job positions include health policymakers, DH policy advisors, standards specialists, etc.

(ii). **DH managers/leaders**: this includes persons in the position of authority, who oversee the implementation and use of DH. Examples include health facility administrators, heads of departments, directors, commissioners, etc.

(iii). **DH technical managers/architects**: this role includes different facets of DH including enterprise architecture, data management, IT infrastructure, Software and Application Development/Management, DH Security and privacy, etc.

(iv). **DH application and technology users**: Any HW who uses ICT to support their work routines.

PROFICIENCY LEVELS REQUIRED FOR DIGITAL HEALTH WORKERS (DHWs) IN UGANDA

Table 21 shows Four proficiency levels that are recommended for DHWs in Uganda, i.e., Basic level=1, Intermediate=2, Advanced=3, and Expert=4. Knowledge/skill domains where a health worker does not require proficiency shall be scored a zero (0) value.

Table 21. Proficiency Levels of DHWs (adopted from Alunyu et al., 2020)

Level of proficiency	Level 1 – Basic	Level 2 – Intermediate	Level 3 - Advanced	Level 4 - Expert
Brief description	This is foundational & develops the DHW's literacy level. Provides common knowledge or understanding of basic DH technology techniques and concepts e.g., types of technology, what for, how to use, etc. Key terms are use, find, identify, etc.	Training at this level aims at developing the DHW capability to independently use DH technology to complete tasks and to apply DH technology knowledge or skills in different situations. Key terms include explain, describe, and illustrate, among others	Advanced training equips the DHW with techniques to apply theory. Can perform DH technology tasks without help, it's a level of DH technology professionalism. Key terms are apply, show, propose, explain, vary, assess, etc.	This level prepares a DHW to provide guidance on specific DH technology(ies), troubleshoot and answer questions related to them or an area of expertise within the technology. The DHW becomes the consultant – "go to person" Key terms include create, integrate, propose, etc.
Expected Outcomes	i. Understand and can identify medical / DH terminologies, concepts, principles, and issues<br>ii. Can utilize a full range of DH Technologies	i. Occasionally apply knowledge to different cases with minimal guidance<br>ii. Understand and can discuss the application and implications of DH technology changes to processes, policies, and procedures<br>iii. Chooses appropriate tools for tasks<br>iv. Experiments with new processes, tools, or technologies to determine the applicability	i. Provide practical/relevant ideas and perspectives on DH technology processes or practice improvements to be implemented<br>ii. Coach others in the application DH technologies translating complex problems to solvable forms<br>iii. Support the DH technology development including references and resource materials	i. Demonstrate consistent excellence in applying DH technology expertise across multiple projects and/or health systems<br>ii. Create new technologies/application scenarios<br>iii. Explain the relevant DH technology process elements and issues about organisational issues and trends in sufficient detail
Possible competencies	i. Can use computers & other ICTs<br>ii. Can identify appropriate DH technologies<br>iii. Browse, search, filter data, information, and digital content<br>iv. Distinguish data, information, and digital content<br>v. Can understand DH & medical terminologies, identify disease codes, etc.<br>vi. Can use inbuilt security measures	In addition to level 1 competencies, can;<br>i. Evaluating data, information, and digital content<br>ii. Managing data, information, and digital content<br>iii. Interact through digital technologies<br>iv. Share through digital technologies<br>v. Engage in citizenship through digital technologies<br>vi. Collaborate through digital technologies<br>vii. Netiquette<br>viii. Managing digital identity	In addition to level 2 competencies, level 3 DHW can;<br>i. Develop DH content<br>ii. Integrate and re-elaborate on the DH digital content<br>iii. Solve technical but DH-related problems<br>iv. Shares expertise, teaching skills, and explaining concepts to others<br>v. Copyright and licenses	In addition to level 3 competencies, level 4 DHW can;<br>i. Improves or redesigns DH processes, tools, or technology<br>ii. Implement and troubleshoot complex issues on DH technology(ies) of their expertise<br>iii. Programming
Possible competency categories	i. Technology literacy & usage skills<br>ii. Literacy in medical & DH terminologies<br>iii. Information & Data Literacy<br>iv. Security & Privacy Literacy	In addition to level 1 competency categories are;<br>i. Digital communication<br>ii. DH products & services	In addition to level 2 categorization;<br>i. Regulation & compliance (implementation)<br>ii. Business processes	In addition to level 2 categorization;<br>i. Networking and Programming<br>ii. Data analytics
Example of security & Privacy application	SECURITY and PRIVACY<br>(Required at all levels of competency to protect devices, personal data, health & wellbeing and environment)			
	Protect devices, use security measures on devices & inside applications, etc.;	Protect devices, use security measures on devices, inside applications, on data/ information sharing, etc.	Protect & guide others on how to protect devices, applications, data/ information privacy, etc.	Improve or develop systems to enforce security & privacy

CONCLUSION

Notwithstanding numerous benefits that can be realised from implementing Digital Health (DH) in Uganda's health system, lack of standardisation and non-interoperability have caused most DH interventions to remain isolated and uncoordinated; hence affecting data use and utilisation in healthcare and service delivery, decision-making and informing policy. This Guide fills the gap by detailing what is needed to standardise Digital Health in Uganda's health system. Particularly, the Standardisation Guide provides a comprehensive set of Digital Health Enterprise Architecture, Standards, and Knowledge products (DH-ASK) guidelines, that if implemented as prescribed, can facilitate a sustainable Digital Health environment for Uganda's health system. Additionally, if standards for DH capacity building are implemented as prescribed, these can aid the production of competent DH practitioners that can; lead DH policy development, management and regulation; develop, implement and maintain DH systems; and proficiently use the deployed DH systems in Uganda. As such, supporting realisation of MoH's mission to provide the highest possible level of health services to all people in Uganda. MoH's next steps include among others; phased implementation and rollout of the DH-ASK Standardisation Guidelines in Uganda's health system, and effective monitoring and evaluation of the DH implementations in the country.

REFERENCES

Adebesin, F., & Kotzé, P. (2017). A process for developing an e-health standards selection method artefact using design science research. *Journal of Design Research, 15*(3–4), 258–287.

Adebesin, F., Kotze, P., Foster, R., & Van Greunen, D. (2013). A Review of Interoperability Standards in E-health and Imperatives for their Adoption in Africa. *South African Computer Journal, 50*(1), 55–72.

Alunyu, A. E., Munene, D., & Nabukenya, J. (2020). Towards a Digital Health Curriculum for Health Workforce for the African Region: A Scoping Review. *Journal of Health Informatics in Africa, 7*(1), 38–54.

Alunyu, A. E., & Nabukenya, J. (2018). *A Conceptual Model for Adaptation of eHealth Standards by Low and Middle-Income Countries. 5*(2), 10–16. https://doi.org/10.12856/JHIA-2018-v5-i2-199

Alunyu, A. E., Wamema, J., Kiwanuka, A., Moses, B., Amiyo, M., Kambugu, A., & Nabukenya, J. (2021). *Investigating the impediments to accessing reliable, timely and integrated electronic patient data in healthcare sites in Uganda.*

ANSI/BICSI, & Institute, A. N. S. (2018). *Information Communication Technology Systems Design and Implementation Best Practices for Healthcare Institutions and Facilities: ANSI/BICSI 004-2018.* BICSI. https://books.google.co.ug/books?id=6GjYvQEACAAJ

Ashaba, J., & Nabukenya, J. (2022). Beyond monitoring functionality to results evaluation of eHealth interventions: Development and validation of an eHealth evaluation framework. *Health Informatics Journal, 28*(4), 1–16. https://doi.org/10.1177/14604582221141834

Barateiro, J., Antunes, G., & Borbinha, J. (2012). Manage risks through the enterprise architecture. *2012 45th Hawaii International Conference on System Sciences*, 3297–3306.

Costello, M., & Parker, J. (2020). *Related Documents 1. A Health Interoperability Standards Development, Maintenance and Management Model for Australia, Executive Summary Report, Final, Version 1.0, 30 June 2019. 2. A Health Interoperability Standards Development, Maintenance and Management* (p. 145). http://www.jpconsulting.com.au

Council of the European Union. (2004). *List of standards and/or specifications for electronic communications networks, services and associated facilities and services* (p. 144). https://www.etsi.org/deliver/etsi_sr/002200_002299/002211/01.01.01_60/sr_002211v010101p.pdf

European Parliament. (n.d.). *eHealth – Technology for health—Think Tank.* Retrieved 27 November 2017, from http://www.europarl.europa.eu/thinktank/en/document.html?reference=EPRS_BRI(2015)551324

European Union. (2015). *eHealth Network: Refined eHealth European Interoperability Framework* (p. 24). https://ec.europa.eu/health/sites/health/files/ehealth/docs/ev_20151123_co03_en.pdf

European Union. (2019). *eHealth Network Guidelines* (p. 22). https://ec.europa.eu/health/sites/health/files/ehealth/docs/ev_20190611_co922_en.pdf

Gary, D., Mark, J., Don, M., John, R., & Helen, S.-L. (n.d.). *ISO/HL7 10781—Electronic Health Record System Functional Mode.*

GoU. (2021a). *E-Government Interoperability Framework Reference Architecture.*

GoU. (2021b). *Government of Uganda e-Government Web Application Security Architecture Framework.*

Hammond, W. E. (2017). Standards for Global Health Information Systems. *Global Health Informatics: How Information Technology Can Change Our Lives in a Globalized World*, 94–108.

Harris, S., & Maymi, F. (2019). *All-in-One CISSP® All-in-One Exam Guide.*

HHS Office of Civil Rights. (2013). *HIPAA Administrative Simplification.*

HIPAA. (2013, August 12). *HIPAA Security Rule Standards and Implementation Specifications.* HHS.Gov. https://www.hhs.gov/hipaa/for-professionals/security/laws-regulations/index.html

Hudson, B., Hunter, D., & Peckham, S. (2019). Policy failure and the policy-implementation gap: Can policy support programs help? *Policy Design and Practice*, *2*(1), 1–14. https://doi.org/10.1080/25741292.2018.1540378

ISO. (n.d.). *Publicly Available Standards.* Retrieved 23 January 2019, from https://standards.iso.org/ittf/PubliclyAvailableStandards/index.html

ISO. (2017). *Health informatics Standards.* https://www.iso.org/committee/54960/x/catalogue/

ISO/HL7. (n.d.). *ISO/HL7 10781:2015 Health Informatics—HL7 Electronic Health Records-System Functional Model, Release 2 (EHR FM).* ISO. Retrieved 14 April 2022, from https://www.iso.org/cms/render/live/en/sites/isoorg/contents/data/standard/05/77/57757.html

ISO/IEC. (2018). INTERNATIONAL STANDARD ISO / IEC Information technology—Security techniques—Information security management systems—Overview and. In *ACM Workshop on Formal Methods in Security Engineering. Washington, DC, USA* (Vol. 34, Issue 19).

ISO/IEC. (2019). *Guide 59 ISO and IEC recommended practices for standardization by national bodies. 2019.*

ISO/IEEE. (n.d.). *Novel ISO/IEEE 11073 Standards for Personal Telehealth Systems Interoperability—IEEE Conference Publication.* Retrieved 31 December 2018, from https://ieeexplore.ieee.org/abstract/document/4438177

ISO/TR. (2009). *ISO/TR 11636:2009; Health Informatics—Dynamic on-demand virtual private network for health information infrastructure.* ISO. https://www.iso.org/cms/render/live/en/sites/isoorg/contents/data/standard/05/06/50638.html

ITI Planning Committee. (2015). *Health IT Standards for Health Information Management Practices (HIT Standards for HIM Practices).*

ITU-T. (2015). *Recommendation ITU-T H.813: Interoperability design guidelines for personal health systems: Health record network (HRN) interface.*

Josey, A., Harrison, R., Homan, P., Rouse, M. F., Sante, T. Van, Turner, M., & Merwe, P. van. (2018). *The TOGAF® Standard, Version 9.2: A pocket guide* (Issue April). The Open Group.

Kim, T. Y., & Matney, S. A. (2018). Informatics-Related Standards and Standards-Setting Organizations. In *Health Informatics: An Interprofessional Approach* (2nd Edition). Elsevier Inc.

Kiwanuka, A., Bagyendera, M., Wamema, J., Alunyu, A., Amiyo, M., Kambugu, A., & Nabukenya, J. (2021). *Establishing the State of Practice about Data Standards in Monitoring Healthcare Interventions for HIV in Uganda's EMR-based Health Information Systems.* 200–211. https://doi.org/10.5220/0010264302000211

Kotusev, S. (2019). Enterprise architecture and enterprise architecture artifacts: Questioning the old concept in light of new findings. *Journal of Information Technology*, *34*(2), 102–128.

Lau, F., & Kuziemsky, C. (2016). *Handbook of eHealth evaluation: An evidence-based approach.*

Ministry of Health-Uganda. (2010). *The Second National Health Policy 2010/11 to 2019/20.* 30.

Ministry of ICT-Uganda. (2019). *Data Protection and Privacy Act, 2019.*

MoH-Uganda. (2016). *Uganda National eHealth Policy and Strategy.*

MoH-Uganda. (2021). *Strengthening Uganda's Health System through Standardizing Digital Health: Requirements for Digital Health Standards and Enterprise Architecture Framework* (1st ed.). http://library.health.go.ug/health-information-systems/digital-health/strengthening-ugandas-health-system-through-standardizing

NEHTA. (2012). *Standards-Australia.* http://www.e-health.standards.org.au/StandardsOrganisations/NEHTA.aspx

NITA-U. (2010). *ICT Policies, Strategies and Initiatives put in place in Uganda* (p. 12). http://unctad.org/Sections/un_cstd/docs/cstd2010d12_Uganda_en.pdf

NITA-U. (2021). *Uganda e-Government Interoperability Framework (e-GIF).* https://www.nita.go.ug/projects-service-portfolio/uganda-e-government-interoperability-framework-e-gif
Onwubiko, C. (2009). A security audit framework for security management in the enterprise. *Communications in Computer and Information Science*, *45*, 9–17. https://doi.org/10.1007/978-3-642-04062-7_2
PEPfAR. (2019). *The United States President's Emergency Plan for AIDS Relief—United States Department of State.* https://www.state.gov/pepfar/
Singapore Ministry of Health. (2015). *Singapore—National-telemedicine-guidelines-for-singapore.*
The Open Group. (2018a). *The Open Group Architectural Framework Standards* (Version 9.2). https://pubs.opengroup.org/architecture/togaf9-doc/arch/
The Open Group. (2018b). *The Open Group Standard (TOGAF 9.2).*
TR-42.1, T. (2017). *Cabling standard for healthcare facilities_ANSI-TIA-1179-A.*
van de Wetering, R., Kurnia, S., & Kotusev, S. (2021). The Role of Enterprise Architecture for Digital Transformations. *Sustainability*, *13*(4), 2237. https://doi.org/10.3390/su13042237
WHO (2013). *WHO evaluation practice handbook.* World Health Organization.
WHO (2016). *Monitoring and evaluating digital health interventions: A practical guide to conducting research and assessment.*
WHO & ITU. (2012). *National eHealth strategy toolkit.* International Telecommunication Union. http://www.who.int/iris/handle/10665/75211

APPENDICES

APPENDIX A.1 STANDARDISATION FRAMEWORK

Figure 14 illustrates the standardisation framework that can be used to develop standards requirements for Uganda's digital health. It constitutes three major phases (i.e., context setting, standards adaption, and standards implementation).

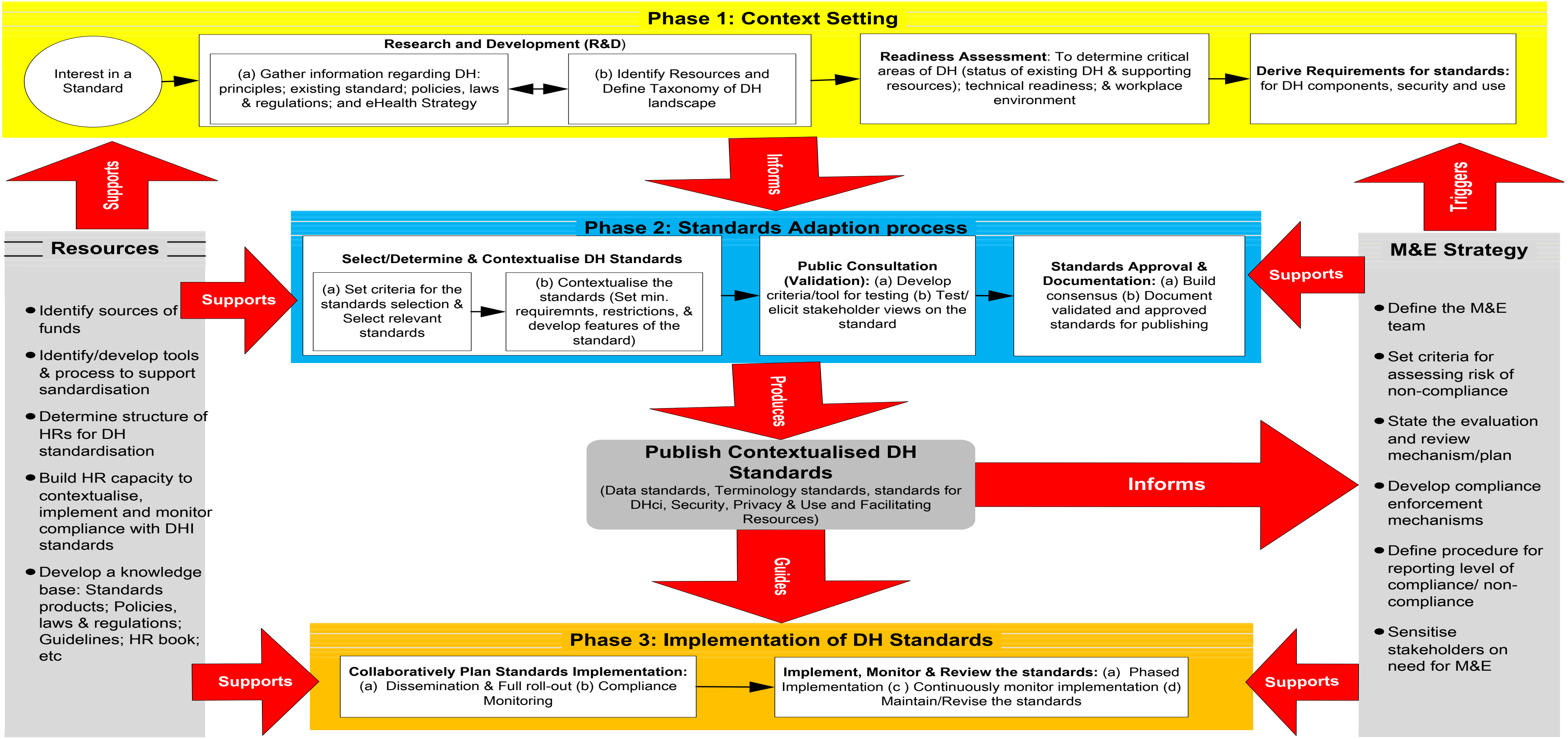

Figure 14. Framework for Standardisation of Uganda's Digital Health Interventions

APPENDIX A.2: STANDARDISATION PROCESS

Error! Reference source not found.**15** illustrates the determination process for filtering suitable Digital Health standards for possible adoption or contextualisation for Uganda's health system.

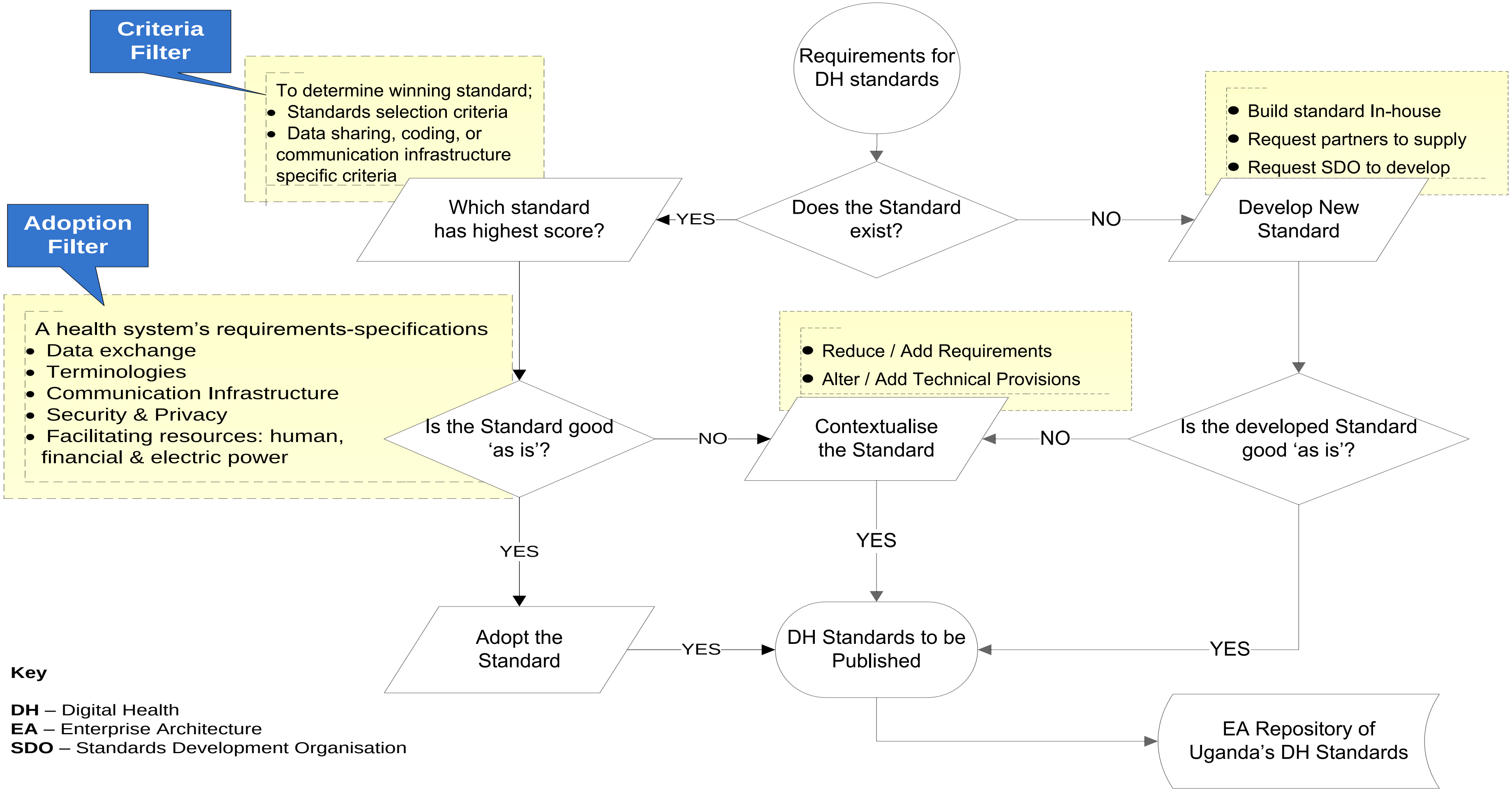

Figure 15. Process of selecting suitable standards for Digital Health

APPENDIX B.1: USE CASE – HIV/TB CLIENT MANAGEMENT SYSTEM ARCHITECTURE FOR IDI CLINIC, MULAGO

The aim of the Digital Health Enterprise Architecture Framework (DHEAF) in Section 1 is to guide the development and implementation of Digital Health Solutions in Uganda. The HIV/TB Client Management System Architecture (HIV/TB CMS) for the Infectious Disease Institute (IDI) Clinic located at Mulago National Referral Hospital is used as an instance of a real-life EA for a DH solution anchored on DHEAF.

IDI-Clinic, Mulago offers treatment to over 7,000 HIV patients and specialised clinics for special populations such as TB patients, pregnant women, adolescents, young adults, and discordant couples. Services offered by the clinic include; General clinic for HIV patients, Integrated TB-HIV clinic, Sexual and reproductive health services, Adolescents' Clinic, Discordant couples' clinic, Senior citizens clinic, Mental health clinic, non-communicable diseases clinic, Kaposi's sarcoma clinic, and Co-Pay clinic services.

To provide high-quality HIV services, the clinic implemented an electronic patient management system (ICEA) that records individual patient data, including all visits information. ICEA is linked to DHIS2, Uganda's National Health Information System, for collecting aggregated data on HIV and Navision Accountancy software to support in determining actual costs of patient care. The Clinic also uses RX solution to support pharmaceutical inventory management, including; drug ordering, stock taking, patient registration, prescription, dispensing of drugs, and audit. These solutions were, however, implemented without any kind of plan and coordination, which, resulted into several challenges including; (i) inaccurate and fragmented data; (ii) HIV/TB client management solution (ICEA) that is not fully aligned to all the client management processes at the clinic; (iii) increased workload for healthcare workers; (iv) duplication of services; and (v) inability to exchange data due to lack of standardisation.

IDI CMS Architecture Vision – to create a sustainable and interoperable digital health solution that supports the delivery of quality HIV/TB healthcare in a secure and cost-effective manner while meeting national and international healthcare standards.

The EA effort is limited to HIV/TB client management capability focusing on the following processes; Pre-test counseling, Client registration, Laboratory requisition, post-testing counseling, Baseline visits, visits, Follow-up clinical evaluation, Client referral, and Pharmacy management as seen in **Figure 16**.

EA REQUIREMENTS, OUTCOMES, AND GOALS

Table 22 summarises the enterprise architecture goals, requirements and outcomes for IDI clinic.

Table 22. IDI EA Requirements, Outcomes and Goals

**Architecture Goals**
• To make HIV/TB client care safer and more effective by making available the correct information in the right place at the right time.
• To ensure equitable access to quality healthcare services by all HIV/TB clients.

• To safeguard the confidentiality, privacy, and integrity of HIV/TB client health information.
• To ensure the seamless sharing of information to support health care delivery to HIV/TB clients.
**Architecture Requirements**
• Reduce redundancy, complexity, and duplication by optimising HIV/TB healthcare processes.
• Ensure interoperability of digital solutions that support HIV/TB client management
• Eliminate information silos
• Improve security and privacy of HIV/TB clients' data
**Expected Outcomes**
• *Improved client experience or satisfaction* - The HIV/TB CM) is implemented not as a tool for data collection but for delivering high-quality HIV/TB healthcare services to clients
• *Optimised HIV/TB client management processes* - Optimised HIV/TB healthcare processes will reduce complexity, redundancy, and duplication, making it easy to add new functions to support new requirements
• *Reduced cost*- With standards in place and improved integration and interoperability, it will be possible to re-use or integrate existing solutions rather than developing new ones, thereby reducing the cost of developing and maintaining new systems.
• Improved Security and Privacy of health data- By storing all HIV/TB clients' information in one system, it will be easy to manage access to the information

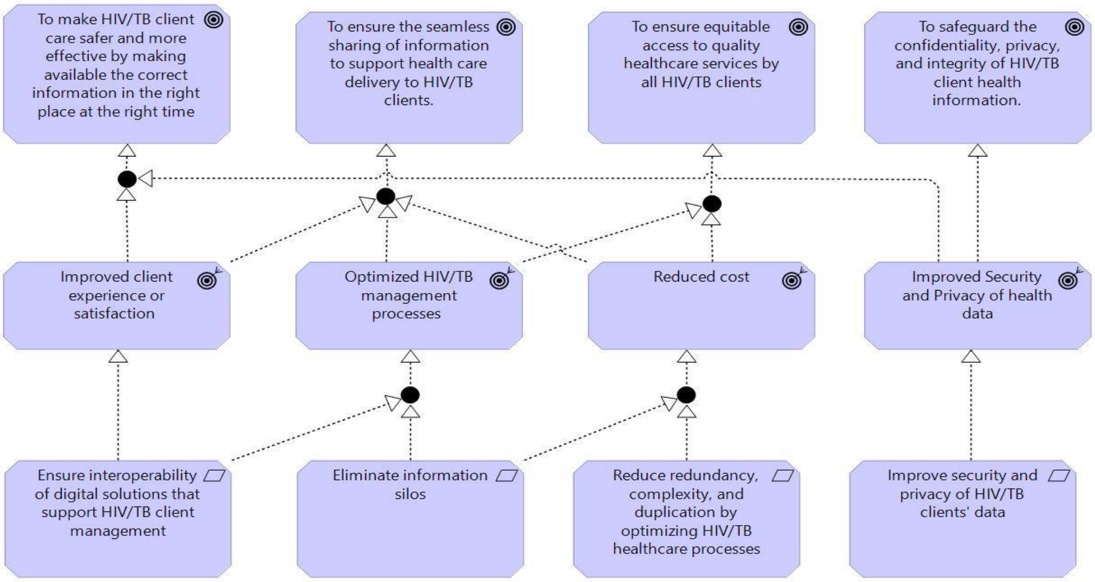

Figure 16: Goals, objectives, and requirements relationship diagram

ASSUMPTIONS, POTENTIAL RISKS, AND DEPENDENCES

Implementation of the target architecture for HIV/TB CMS will require the commitment and support of the leadership of the IDI clinic, Mulago, and all stakeholders. The benefits of the target architecture will only be realised once the architecture has been fully implemented. It is, therefore, critical to identify assumptions, anticipated risks, and dependencies to achieve this. **Table 23** summarises the assumptions, potential risks and mitigation measures to implement the architecture.

Table 23. Assumptions, Potential Risks and Mitigation Measures

**Assumptions**	
**Resources Availability**	Leadership at the Clinic, shall ensure that the required financial and human resources are in place to support the implementation of the target HIV/TB client management architecture
**Stakeholder Involvement**	Implementers of the target architecture shall ensure that key stakeholders are involved in all the stages of the implementation process.
**Leadership involvement and support**	Leadership at the Clinic, shall drive the implementation of the target architecture, and will ensure that all employees comply with the new approach to service delivery.
**Supportive technology infrastructure**	Leadership at the Clinic, shall ensure the availability of the required technology infrastructure.

**Potential Risks and Mitigation Measures**	
**Code**	**IDI_MR01**
**Risk**	***Change in leadership at IDI Clinic, Mulago.***<br>The current leadership at IDI-Clinic, believes that EA can be used as a general-purpose vehicle to transform HIV/TB client management. However, given the time it may take to have the

	target architecture implemented, changes in leadership may occur, and this may negatively affect the implementation of the target architecture
**Probability**	Medium
**Impact**	High
**Mitigation approach**	Implementers of the target architecture for HIV/TB client management should ensure that all top management members at IDI-Clinic, are involved in planning and directing the implementation of the target architecture such that if any one of them leaves, the other members can continue to support the implementation
**Code**	IDI_MR02
**Risk**	***Insufficient expertise in Enterprise Architecture***<br>EA is relatively new in Uganda, as such, the IDI-Clinic, may not have adequate staff trained in EA; which may affect implementation of the target architecture for HIV/TB Client Management
**Probability**	High
**Impact**	High
**Mitigation approach**	Management should organise in-house training in Enterprise Architecture for all the critical staff to be involved in the implementation of the target HIV/TB client management architecture
**Code**	IDI_MR03
**Risk**	***Resistance to new HIV/TB CMS.***<br>IDI-Clinic staff members who have been used to ICEA may not want to change to the new HIV/TB CMS.
**Probability**	Medium
**Impact**	High
**Mitigation approach**	Involve current users of ICEA in the development processes for the HIV/TB CMS.

DEPENDENCIES

ICT Governance – ICT Governance at the IDI-Clinic, should be strengthened to support the EA approach to digital transformation. New roles, including; Enterprise Architect, Data Standards Specialist, and Data Steward, should be added to the governance structure of the IDI-Clinic.

Knowledge about EA and Data Standards - Staff involved in the implementation of the target HIV/TB Client Management architecture should make efforts to upgrade their skills in EA, Interoperability, and data standards.

Resource mobilisation - Leadership at the IDI-Clinic, should work together to mobilise financial and human resources to support the implementation of the target HIV/TB client management architecture.

Information Management Principles – These include both the Enterprise principles (general rules intended to support decision-making at IDI-Clinic and shall dictate how the clinic plans to fulfill its mission) and Architecture principles *(business, data, application,*

technology, security and interoperability) that can be customised based on the generic DHEAF to suit IDI-Clinic CMS Architecture.

Coding Standards

Table 24. Provides the recommended coding standards that should be followed in the implementation of HIV/TB CMS.

Table 25. Recommended Semantic Standards to Support HIV/TB CMS

Standard	Description	Recommendations
International Classification of Diseases (ICD)	A standard vocabulary for diseases, health status, types of patient visits to doctors and other health providers, and external causes of injuries.	HIV/TB CMS should use ICD 10 or 11 coding for disease specification.
Logical Observation Identification Names and Codes (LOINC)	A common terminology standard for Laboratory and clinical observations	HIV/TB CMS should use LOINC to define laboratory measurements and clinical observations.
Systematized Nomenclature of Medicine-Clinical Terms (SNOMED CT)	A standardised, multilingual vocabulary of clinical terminology used by physicians and other health care providers for the electronic exchange of clinical health information.	HIV/TB CMS should use SNOMED CT for clinical information.

INFORMATION SECURITY GUIDELINES

Table 26. Presents the guidelines for protecting and managing HIV/TB data.

Guideline	Description
**Authentication**	Use a password or combination of tokens, biometrics, or multiple authentications to control access to HIV/TB CMS.
**Authorisation**	Use of role-based access controls.
**Encryption**	Ensure that shared HIV/TB data is unreadable until an authorized user decrypts them.
**Integrity**	Ensure that HIV/TB data are protected from unauthorized alteration and destruction, whether accidentally or deliberately, and that data and programs are changed only in a specified and authorized manner.
**Accountability**	Track and monitor activities associated with the access and use of the HIV/TB CMS
**Assurance and awareness**	• Ensure that existing HIV/TB data is of high quality.<br>• Monitoring and evaluation tools and procedures should be put in place to identify data quality issues that need to be addressed.<br>• Ensure that user education is continuous for crucial staff interacting with HIV/TB CMS.
**Auditing**	Track all changes in access to HIV/TB data and all changes to the data.

HIV/TB CLIENT MANAGEMENT SYSTEM ARCHITECTURE INTEGRATED MODEL

ArchiMate modelling language is used to model the HIV/TB CMS Architecture as seen in **Figure 17**. *Archi*Mate offers an integrated architectural approach that describes, analyses, and visualises the different architectural domains and their underlying relations and dependencies (Josey, 2016). It is also fully aligned with TOGAF, the foundation standard for the development of the DHEAF.

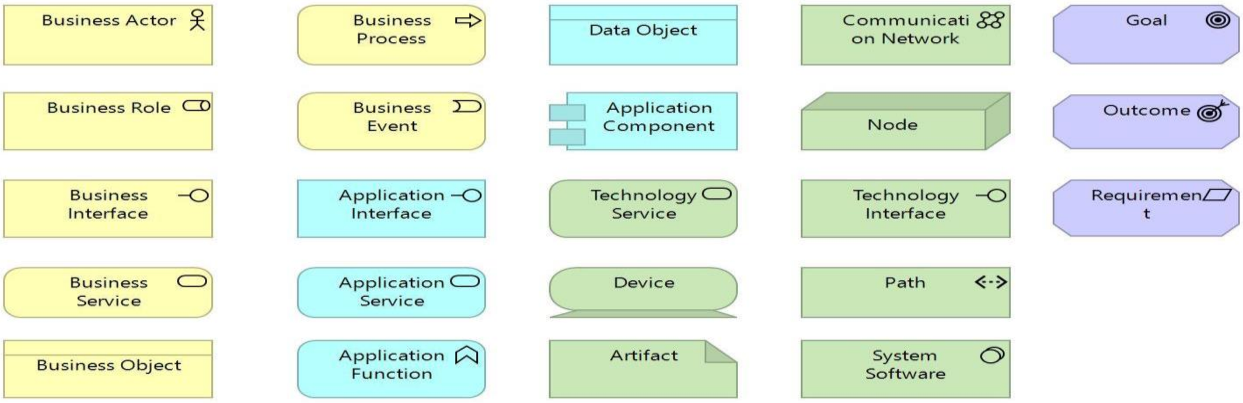

Figure 17. Modelling Language for HIV/TB Client Management System Architecture

Figure 18 below describes the key symbols used to document the HIV/TB CMS Architecture.

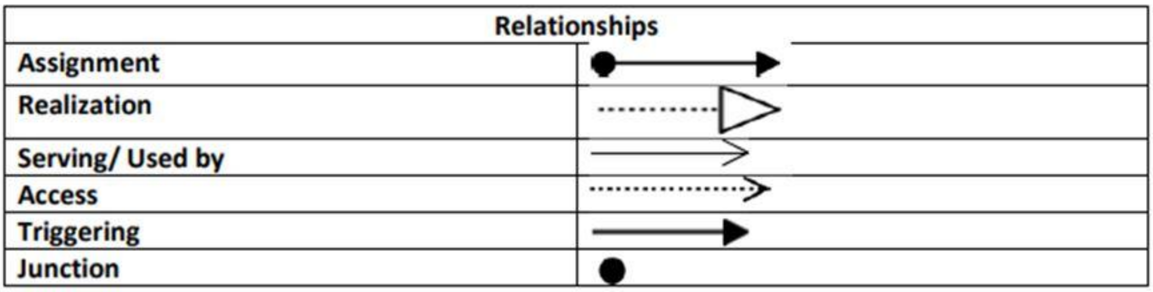

Figure 18. Symbols used in the CMS Architecture

Finally, the high-level HIV/TB Client Management System Architecture is presented in **Figure 19**.

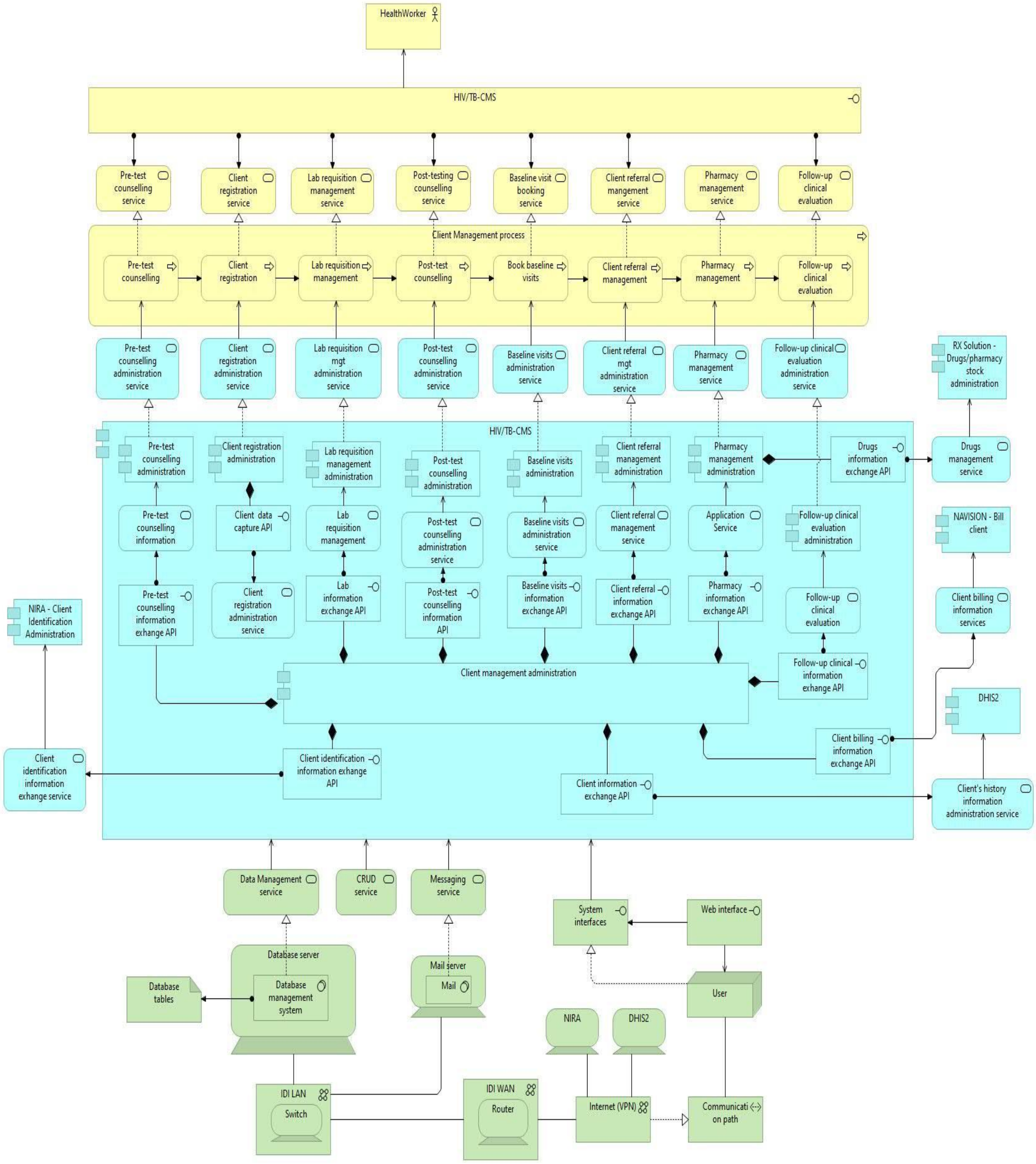

Figure 19. Integrated HIV/TB Client Management System Model (Initial version)

APPENDIX B.2: USE CASE – CONTEXTUALISING TERMINOLOGY CODES FOR HIV DISEASE MANAGEMENT SERVICES

Contextualisation of terminologies and medical codes follows a process as outlined in **Figure 20**. There has to be a need for a terminology to kick start the terminology contextualisation process. The need drives stakeholders to study the national and international health system contexts to understand the opportunities and complexities of the specific terminology. Understanding the national context will drive extraction of data sets and indicators from the national Health Information Systems. Mapping will consider the exact matches in terms of code descriptions and then unmatched codes will be contextualised using UG as a prefix to a code that is most similar in description in any of the international terminologies. Then a prefix that is serially labeled will follow as exemplified using HIV services in **Table 27**. The main terminologies that majorly suit our digital health ecosystem are Systematised Nomenclature of Medicine -- Clinical Terms (SNOMED-CT), International Classification of Diseases (ICD) and Logical Observation Identifiers Names and Codes (LOINC). SNOMED CT which is a structured clinical vocabulary for use in an electronic health record is the most comprehensive and precise clinical health terminology product in the world. ICD is designed to promote international comparability in the collection, processing, classification, and presentation of morbidity and mortality statistics. LOINC is a database and universal standard for identifying medical laboratory observations. The compiled terminologies will be validated with stakeholders and any revisions made if needed. The final set of terminologies will then be housed in an online terminology server housed at the Ministry of Health headquarters and managed by a standards manager.

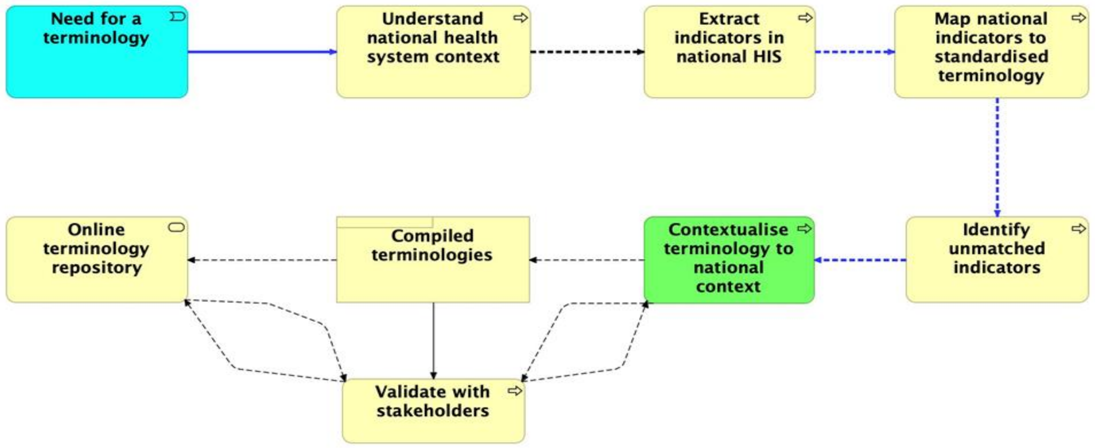

Figure 20. Process of Contextualising Health Terminologies

Terminology Codes for HIV Disease Management Services

Table 27. Use Case - Terminology Codes for HIV Disease Management Services

Terminology Code	Terminology Name	Terminology Description
Registry Codes		
Health Facility Registry	Name of Health Facility	Name of the health facility
Health Facility Registry	Code	Code of the health facility
Health Facility Registry	Level	Health system level of the health facility
Health Facility Registry	Sub-county / Division	Sub-county or division in which the health facility is located
Health Facility Registry	HSD	Health sub-district in which the health facility is located
Health Facility Registry	District	District in which the health facility is located
Health Facility Registry	Name of Health Facility	Name of the health facility
Health Facility Registry	District	District in which the health facility is located
Health Facility Registry	Hub	Hub for viral load testing
Health Worker Registry	Requesting Clinician	Clinician requesting for viral load analysis and HIV drug resistance testing
Health Worker Registry	Phone number	Phone number of the clinician requesting for viral load analysis and HIV drug resistance testing
Client Registry	NIN	National identification number of client
Client Registry	Patient's surname	Surname of the client
Client Registry	Patient's first name	First name of the client
Client Registry	Patient's contact	Telephone number of the client
Client Registry	Date of birth	Date of birth of the client
Client Registry	Sex	Sex of the client
Client Registry	Date of Birth	Date of birth of the client
Client Registry	Patient Clinic ID/ART #:	Unique identifier of client at the health facility
Client Registry	If DOB Unknown Age in Years	Age in years
Client Registry	If < 2 years, Age in Months	If the age is less than 2 years, indicate age in months
Client Registry	Sex	Sex of the client
Client Registry	NIN	National identification number of client
Client Registry	Other ID	Number of other identifications
Client Registry	Phone number	Telephone number of the client
Client Registry	If Pregnant, enter the ANC #	Antenatal care number of client

Client Registry	PNC #	Postnatal care number of client
Client Registry	Name of Exposed Person	Name of exposed person
Client Registry	Age	Age of exposed person
Client Registry	Sex	Sex of exposed person
Client Registry	Village	Village of exposed person
Client Registry	Parish	Parish of exposed person
Client Registry	Sub-county	Sub-county of exposed person
Health Worker Registry	Name of Lab Person	Name of laboratory personnel taking sample
Health Worker Registry	Phone number	Telephone number of laboratory personnel taking sample
**Viral Load Codes**		
76423-3	Date of Registration	Date of the first visit is considered the start date for a specific episode of care.
21866-9	Patient Clinic ID	Provides a unique identifier for the patient consisting of the year in which the patient was first seen at the reporting facility and the consecutive order in which the patient was abstracted. The first four numbers specify the year and the last five numbers are the numeric order in which the patient was entered into the registry database.
133932002	Supporter's name	Person in the healthcare environment to support the client
UGHIV.1137457010.0001	eMTCT	For female clients on eMTCT, write yes (Y) and then specify as “P” if Pregnant or “BF” if Breastfeeding
UGHIV.82772-6.0002	Date VL results received at facility	Date when viral load results were received at the facility as written on the result form
UGHIV.51953-9.0003	Date CD4 Sample Collected	Date CD4 collected from patient
8128-1	CD result / 100 cells	CD3+CD4+ (T4 helper) cells/100 cells in Blood
24467-3	CD4 result / volume in blood	CD3+CD4+ (T4 helper) cells [#/volume] in Blood
UGHIV.59419-3.0004	VL results at initiation of IAC (copies/ml)	HIV 1 RNA [#/volume] (viral load) in Plasma by Probe with signal amplification
UGHIV.20447-10.0005	VL results at initiation of IAC (copies/ml)	HIV 1 RNA [#/volume] (viral load) in Serum or Plasma by NAA with probe detection
UGHIV.21008-9.0006	VL results at initiation of IAC (copies/ml)	HIV 1 RNA [#/volume] (viral load) in Serum or Plasma by Probe
UGHIV.86548-6.0007	VL results at initiation of IAC (copies/ml)	HIV 2 RNA [#/volume] (viral load) in Plasma by NAA with probe detection
399445004	Date of sample collection	Date of sample collection
33882-2	Date of sample collection	Date of sample collection
UGHIV.133877005.0008	Regimen at VL test	Code of the ART regimen the patient is currently on

UGHIV.63936-10.0009	Start date of ARV Regimen	Date the client was initiated on the current regimen
UGHIV.413946010.0010	Start date of ARV Regimen	Date the client was initiated on the current regimen
104298002	Serum Crag Screening	Measurement of Cryptococcus species antibody (procedure)
31791-7	Crag Results	Cryptococcus sp Ag [Units/volume] in Serum
UGHIV.387174007.0011	Treated with Fluconazole	A client treated with fluconazole
UGHIV.95127-7.0012	TB LAM Test	TB LAM test done
UGHIV.95127-8.0013	TB LAM Results	TB LAM test result
UGHIV.45241-8.0014	TB Treatment	Is the client on TB treatment
UGHIV.409063006.0015	Restart Intensive Adherence Counselling (IAC)	“Yes” for a client who has completed 6 Intensive Adherence Counselling sessions but is not yet eligible for a Viral load re- test, and “No” for a client who is just starting Intensive Adherence Counselling.
UGHIV.409063006.0016	IAC session number	Indicate the Intensive Adherence Counselling session number
UGHIV.409063006.0017	Session date	The date the Intensive Adherence Counselling session was conducted
UGHIV.409063006.0018	Adherence assessment score (%)	The adherence assessment score in percentage
UGHIV.409063006.0019	Adherence code	The adherence assessment score (G for Good, F for Fair and P for Poor)
UGHIV.119297001.0020	VL sample collected	Sample collection for VL for clients on second line regimen
UGHIV.119297001.0021	HIVDR sample collected	Sample collection fo HIVDR
UGHIV.399445005.0022	Date of repeat VL / HIVDR sample collection	Date the repeat VL and HIVDR samples have been collected
UGHIV.59419-3.0023	Repeat VL results after IAC (Copies/ ml)	Repeat HIV-1 VL results after Intensive Adherence Counseling in copies per ml
UGHIV.56155-5.0024	Date VL Results Received at facility	Date VL results received at facility from referral lab
UGHIV.425581000.0025	HIVDR results	Results of HIVDR
UGHIV.56155-5.0026	Date HIVDR Results Received at facility	Date HIVDR results received at facility from referral lab
UGHIV.91541-3.0027	Clinical decision	Clinical decision after receiving VL and HIVDR results
UGHIV.133877005.0028	New Regimen	New treatment regimen using national codes
UGHIV.413946010.0029	Date of decision	Date clinical decision has been taken
86467-8	Comments	Narrative comments after the client's visit
406543005	Date	Date of visit

45235-9	Date of Treatment Initiation:	Date of starting HIV treatment
1C62.0	Current WHO Stage	HIV disease clinical stage 1
1C62.1	Current WHO Stage	HIV disease clinical stage 2
1C62.2	Current WHO Stage	HIV disease clinical stage 3
1C62.3	Current WHO Stage	HIV disease clinical stage 4
UGHIV.261773007.0030	How long has this patient been on the current regimen?	Duration the patient has been on current regimen
UGHIV.133877005.0031	Which treatment line is patient on?	Treatment line the client has been on
UGHIV.133877005.0032	Current Regimen	Current regimen the client is taking
77386006	Is mother pregnant?	Mother is pregnant
60001007	Is mother pregnant?	Mother is not pregnant
866041003	Is mother breastfeeding?	Mother currently breastfeeding
427099000	Patient has active TB?	Patient has active TB
UGHIV.45241-8.0033	If Yes, which phase are they on?	TB phase that the client is in
182884001	ARV Adherence	ARV adherence
UGHIV.182991003.0034	Treatment care approach	Treatment care approach as per the Differentiated Service Delivery Model
315124004	Indication for viral load testing	Indication for viral load testing
UGHIV.133877005.0035	Past Regimens	Past regimens that the client has taken as per the national guidelines
413946009	Start Date	Start date for the past regimens
413947000	Stop Date	Stop date for the past regimens
29463-7	Body weight (Kg)	Bodyweight of the client
UGHIV.387159010.0036	Patient on Rifampicin?	Patient taking rifampicin
68963-8	Date and Time of sample collection	Date of sample collection
118575009	Date and Time of Sample centrifugation	Date and Time of Sample centrifugation
20395-0	Sample type	Plasma sample type
57718-9	Sample type	DBS sample type
**CRAG Register Codes**		
406543005	Date	Date patient has come to health facility
76427-4	Date	Date patient has come to health facility

UG.64601-8.0037	Date of return after 1 week	Date of return for review after 1 week of Cryptococcal Antigen testing
UG.64601-8.0038	Date of return at 4 weeks	Date of return for review after 4 weeks of Cryptococcal Antigen testing
UG.64601-8.0039	Date of return at 3 months	Date of return for review after 3 months of Cryptococcal Antigen testing
UG.64601-8.0040	Date of return at 6 months	Date of return for review after 6 months of Cryptococcal Antigen testing

APPENDIX C.1: DH MONITORING & EVALUATION INDICATORS AND OPERATIONALISATION TOOLS

Indicator Domain	Measurement Means (Example Indicators)	Operationalisation Tools
**Monitoring Programme / Digital Health Inputs and Activities (Does the Digital Health technology work?)**		
Needs assessment	Availability of documentation of needs assessment results indicating public health needs to be addressed by the Digital Health and its alignment to strategic health objectives	Needs assessment reports
Functionality	The extent to which specified functionalities and requirements are met by the Digital Health technology<br>Proportion of data fields from the original paper-based system that are captured by the Digital Health technology<br>Proportion of the target population who report successful use of the Digital Health technology capabilities	Software testing outcomes<br>System generated data<br><br>User surveys
Software/ Application Standards requirements	Proportion of software standards requirements met by the Digital Health technology	Design specifications<br>Software audit reports
Infrastructure requirements	Proportion of target population who are able to connect and use the Digital Health technology<br>Proportion of target population with access to a power/electricity source for powering/recharging Digital Health related devices<br>The extent to which required hardware technologies are met/provided by the Digital Health intervention	User surveys<br>Infrastructure audit reports
Connectivity	Proportion of target population which can connect to the Digital Health technology signals	System generated data<br>User surveys
User and technical support	Proportion of Digital Health intervention sites or users with access to local technical support for troubleshooting	User surveys
Human Resources	Proportion of target users that have been trained on the use of the Digital Health technology	Training reports
Financial Resources	Proportion of Digital Health budget that has been funded<br>Proportion of Digital Health budget that has been timely funded	Budget Analysis reports

Management and political support	Proportion of Digital Health management stakeholders that commit their time or other resources in support of Digital Health implementation	Administrative data<br>Surveys
Leadership and governance	Availability of an institutionalized Digital Health governance structure<br>Proportion of Digital Health leadership positions that are occupied and functional	Administrative data
Sustainability	Availability of a sustainability plan for the implemented Digital Health<br>Proportion of sustainability related actions that are completed<br>Proportion of Digital Health implementation budget that is facilitated by local funding	Administrative data<br>User surveys
Intervention documentation	Availability of an established systematic process for storage and maintenance of the Digital Health intervention documentation<br>Availability and accessibility of the Digital Health initiative project charter<br>Availability and accessibility of documented key steps from the Digital Health intervention's launch to pilot to maintenance<br>and availability of an established systematic process for housing and maintaining such records	Administrative data
Readiness and change management	Availability of a change management strategy<br>Proportion of change management related actions that are completed	Document reviews<br>User surveys
Legal and regulatory support	The extent to which available laws and regulations support and guide the implementation of Digital Health	Document reviews
**Monitoring / Evaluating Digital Health Outputs (How do users interact with the Digital Health?)**		
User Coverage	Proportion of users who demonstrate proficiency in use of Digital Health technology<br>Proportion of target users observed using Digital Health system over a reference period	Observations<br>System generated data<br>User surveys
User representativeness	The proportionality of each target user category among users who demonstrate proficiency in use of Digital Health technology	User surveys
Transferability	Proportion of target population who report successful use of Digital Health technology capabilities<br>The extent to which the Digital Health intervention has been adopted in different healthcare settings and usage scenarios	User surveys<br>System generated data
User Response	Proportion of users who rate the Digital Health system as "easy to use"<br>Proportion of users who rate the Digital Health system as 'works as intended'<br>Proportion of users who report satisfaction with the content of health information received via the Digital Health system<br>The extent to which users have a demand for the Digital Health	User surveys<br>System generated data
User Adoption	Number of messages / reports / amount of data transmitted by end-users via the Digital Health technology within a reference period<br>Proportion of messages / reports transmitted via the Digital Health system that are responded to appropriately by end-users over a reference period	System generated data

	Proportion of data fields / forms / reports that are complete or left incomplete over a reference period	
**Digital Health Outcomes (How does the Digital Health improve programme processes/service delivery? i.e. Evaluation)**		
**Patient Level**		
Efficiency	Time between Digital Health system prompt received about a health intervention and seeking care from the provider<br>Number of in-person consultations with qualified healthcare providers about a health intervention by target patients as a result of accessing required services using the Digital Health initiative over a reference period	Healthcare facility records, System generated data, Special surveys, Programme records
Quality	Time spent with a healthcare provider in relation to a health intervention at the last visit<br>% of messages received through Digital Health intervention that patients are able to recall about a health intervention during patient exit interviews<br>% of target patients who report correctly adhering to prescribed care protocol in relation to a health intervention<br>% of target patients who report adverse effects from a health intervention<br>% of target patients who report facing barriers to healthcare accessibility (health equity-related barriers)	Healthcare facility records, System generated data, Special surveys, Programme records
Utilization	% of emergency events where the Digital Health system was used by patients to expedite treatment over a reference period<br>% of target patients who report receiving health information about a health intervention via the Digital Health system within a reference period<br>% of target patients who report contact with a qualified healthcare provider using the Digital Health system in relation to a health intervention over a reference period<br>% of target patients who report adequate knowledge about signs and symptoms for which they should seek care in relation to a health intervention<br>% of target patients who report adequate knowledge about the health issue relevant to a health intervention	Healthcare facility records, System generated data, Special surveys, Programme records
Costs	% changes in reported patient out-of-pocket payments for illness management over a reference period	Healthcare facility records, System generated data, Special surveys, Programme records
**Provider Level**		
Efficiency	Time for last patient counselling about a health intervention using Digital Health system<br>Time spent on health record-keeping about a health intervention over a reference period<br>Time used per individual health worker over reference period to transmit data relating to a health intervention from community-based logs to health-care facility-based information systems<br>Time taken per individual healthcare provider over a reference number of events between identification of an adverse event and provision of care (health intervention), across levels of a health system<br>Time used per individual health worker to report important adverse events	Healthcare facility records, System generated data, Special surveys, Programme records

Quality	Proportion of health workers who report adequate knowledge of the health issue relevant to a health intervention<br>Proportion of care standards relating to a health intervention observed to be met using the Digital Health initiative during a patient–provider consultation<br>Proportion of providers observed to be using the Digital Health initiative during their patient consultations	Healthcare facility records, System generated data, Special surveys, Programme records
Utilization	Proportion of targeted health workers who use the Digital Health system in relation to a health intervention through the Digital Health system over a reference period<br>Proportion of health workers observed to use the Digital Health system during their last client contact<br>Proportion of health workers who use the Digital Health system to connect with medical staff to receive real-time clinical information and decision support<br>Number of patients (average or total) attended by a health worker using the Digital Health system over a reference period	Healthcare facility records, System generated data, Special surveys, Programme records
Costs	Amount of estimated cost savings due to improvement in service delivery/efficiency/other factors related to using Digital Health	Healthcare facility records, System generated data, Special surveys, Programme records
**Health System Level**		
Efficiency	Cumulative time over a reference period for all health workers in a healthcare facility using the Digital Health system to enter data related to a health intervention<br>Cumulative time over a reference period for all health workers to transmit data about a health intervention from community-based logs to healthcare facility information systems<br>Cumulative time over a reference number of events between identification of an adverse event and provision of care (health intervention), across levels of a health system<br>Number of days over a reference period for which a healthcare facility reports stock-out of a commodity essential for provision of a health intervention	Healthcare facility records, System generated data, Special surveys, Programme records
Quality	Number of health workers observed to be providing clinical services related to the Digital Health initiative<br>% change in reported stock-out events of a commodity essential for provision of a health intervention over a reference period<br>% change in data entry errors over a reference period	Healthcare facility records, System generated data, Special surveys, Programme records
Utilization	Number of patients seeking a health intervention over a reference period<br>% of patients in a specified area who receive a health intervention through the Digital Health system over a reference period<br>% of target population who have access to a health intervention over a reference period<br>% of health-care facilities in a target geographical area that use the Digital Health initiative<br>Number of patients seeking a health intervention at a healthcare facility using the Digital Health system	Healthcare facility records, System generated data, Special surveys, Programme records
Costs	% change in costs of transporting paper forms and manual data entry over a reference period<br>% change in costs of human resources for data entry<br>% change in costs associated with timely and appropriate management of illness	Healthcare facility records, System generated data, Special surveys, Programme records

	% change in reported patient out-of-pocket payments for management of illness<br>Total population-level savings in out-of-pocket payments attributed to timely and appropriate care seeking	
**Impact (How do improvements in service delivery impact health? i.e., Evaluation)**		
Impact	Improvement in health outcomes attributed to the health intervention being supported by the Digital Health. Measures of health outcomes at this stage are defined basing on the expectations from a health intervention being supported by the Digital Health initiative. Examples of indicators include Mortality ratio, Mortality rate, Quality of life, Disease Incidence rate, Disease notification rate, Lifestyle	Population-based surveys, Coverage, Health status, Equity, Risk protection, Responsiveness

Source: Adopted from Ashaba & Nabukenya (2022)

APPENDIX C.2: RESULTS CHAIN BASED ON THE RESULTS-BASED MANAGEMENT STRATEGY

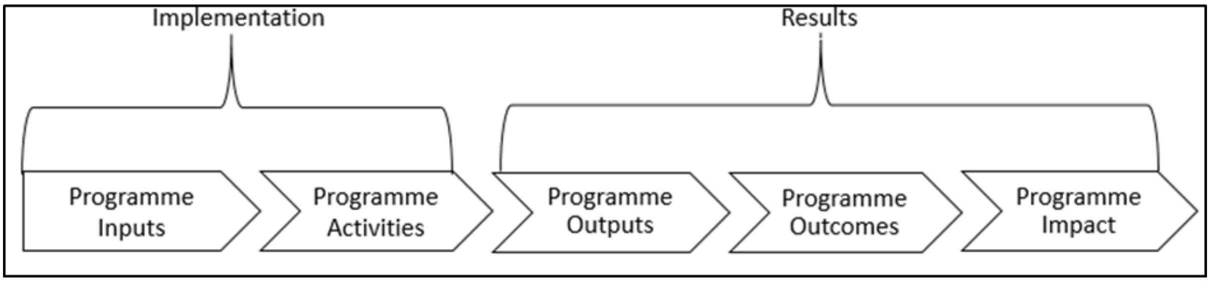

Source: UN Development Group (2011)

APPENDIX C.3: CATEGORISATION OF DH INDICATORS

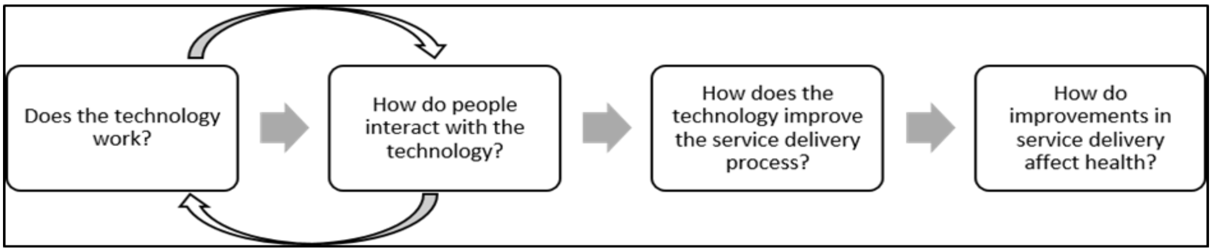

Source: WHO (2016).

REPUBLIC OF UGANDA
MINISTRY OF HEALTH

THE UGANDA HEALTH INFORMATION EXCHANGE AND INTEROPERABILITY GUIDELINES

JANUARY 2023

Foreword iii

Preface iv

Acknowledgement v

Document Reviews and Approvals vi

Abbreviations and Acronyms vii

Definitions of Terms viii

1. Introduction 1

1. 1.1 Background 1
2. 1.2 Review of Health Information Exchange 2
3. 1.3 Document Development Process 3
4. 1.4 Purpose and Scope of Document 4
5. 1.5 Target Audience 4

2. Section A: HIE & Interoperability Guidelines for Digital Health Systems 5

6. 2.1 Systems Standard Guidelines 5
7. 2.2 Communication and Connectivity Protocols 5
8. 2.3 Messaging Standards 6
9. 2.4 Vocabulary Standards 8

3. Section B: Governance Foundations for HIE and Interoperability Standards 10

10. 3.2 Standards Management 10
11. 3.3 Messaging Exchange Standards Support 11
12. 3.4 Protocols and Services for Interoperability 11
13. 3.5 Support HIE Platform Best Practices 11

4. Section C: Monitoring and Evaluating the Uganda HIE & Interoperability Guidelines 13

5. Section D: Sustaining the Health Information Exchange 14

6. Section E: References 16

Foreword

The Government of the Republic of Uganda recognises the use of Information and Communication Technology (ICT) in the National Development Plan III 2020/21 - 2024/25 as an enabler to improving the delivery of services to its citizens across its sectors. The Ministry of Health Strategic Plan 2020/21 – 2024/25 also recognises digital health as a key enabler for supporting the health system to deliver good health to the population.

In addition, the Health Information and Digital Health Strategic Plan 2020/21 – 2024/25 guides how to use ICT to facilitate improvement in the flow of information, through electronic means, to support the delivery of health services and the management of the health system in a bid to facilitate universal access to care, health sector efficiency, and social transformation.

The Government of Uganda is committed to improving the application of digital health technologies to facilitate the attainment of its overall objective of delivering high-quality health services to all citizens.

The Government, Partners, and Private institutions have continued to invest in various digital health initiatives. However, most of these initiatives remain fragmented or siloed systems with minimal exchange of health information to enable high-quality health service delivery.

The Health Information Exchange and Interoperability Guidelines present how standards defined in the Uganda Digital Health Enterprise Architecture, Standards and Knowledge Guidelines are to be applied to digital health systems. This will strengthen information exchange in the national health services network to facilitate quality health service delivery and support achieving Universal Health Coverage (UHC).

All stakeholders are therefore called upon to adopt and use these guidelines while implementing digital health initiatives.

....

Dr. Henry G. Mwebesa

DIRECTOR GENERAL HEALTH SERVICES

Preface

Interoperability guidelines are a major milestone in the journey towards quality, responsive, accessible, and cost-effective Health Information Services (HIS). A well-developed digital health information system is a fundamental and crucial component of any health system. The use of information exchange is not only a key enabler of direct patient care but also a vital tool in health program monitoring. Therefore, it requires necessary attention and well-planned investment of resources to realise its function. These Health Information Exchange and Interoperability Guidelines generally align with the goals and strategies as stipulated in the Uganda Health Information and Digital Health Strategic Plan 2020/21-2024/25 for strengthening information exchange in the national health services network to facilitate adequate health service delivery support to achieve the Universal Health Coverage (UHC).

In the past decade, health services in Uganda have seen tremendous improvement in technology support. This is a result of both technology evolution and funding in these areas which has been progressively acknowledged by the Ministry of Health (MoH), its partners in health and stakeholders. Following MoH guidance in the planning and implementation of HIS for health services, the Uganda HIE will accelerate the achievement of the expected program benefits towards strengthening health information management in both the Public and Private Sectors. MoH is committed to strengthening the coordination and quality of health information management to support the attainment of Universal Health Coverage (UHC).

All stakeholders are therefore called upon to examine the Health Information Exchange and Interoperability Guidelines, assess their involvement, and thereafter align their present and future health information exchange implementations with the guidelines laid out in this document.

....

Dr. Sarah Byakika

Commissioner Health Services

Department of Planning, Financing and Policy

Acknowledgement

The Ministry of Health expresses its profound gratitude to all departments and programs, the Health Information Innovation and Research Technical Working Group (HIIRE TWG) and the Digital Health Subcommittee who contributed technical inputs leading to the successful completion of this document. Special appreciation goes to the Ministry of Health Division of Health Information Management (DHIM) and the Information Communication Technology (ICT) Section for the overall guidance to ensure the guidelines are aligned with the Uganda Health Information and Digital Health Strategic Plan 2020/21-2024/25.

The Ministry of Health acknowledges and thanks all development, implementing and technology partners that provided financial and technical support towards the development of these guidelines, specifically CDC, MUSPH/METS, PATH, HISP and the Technical Assistance Platform (TAP). DHIM is grateful for all the support, sacrifice and contribution invested in the successful development of these guidelines.

Finally, the Ministry of Health is grateful to the Ministry of Information Communication Technology and National Guidance (MoICT&NG), National Information Technology Authority Uganda (NITA-U), Uganda Health Informatics Association (UgHIA), Makerere University and all those institutions and individuals who have not been specifically mentioned above, but who directly or indirectly contributed to the successful development and finalisation of these Health Information Exchange and Interoperability Guidelines.

....

Mr. Paul Mbaka

Assistant Commissioner Health Services

Health Information Management

Document Reviews and Approvals

These guidelines shall be reviewed once a year and the changes shall be documented and approved by the relevant authorities. Relevant sections that shall need to be added to the document shall follow the standard MoH approval processes, these shall include: -

- Assign a new version number
- Document the date of approved updates.

**Version**	**Owner**	**Author**	**Approver**	**Date of Approval** *(MMDDYY)*
01	Ministry of Health	Division of Health Information Management	Top Management Committee	18.09.2024

Abbreviations and Acronyms

API	Application Programming Interface
ANSI/TIA	American National Standards Institute / Telecommunications Industry Association
EMR	Electronic Medical Record
EHR	Electronic Health Record
HIE	Health Information Exchange
HIDH	Health Information and Digital Health Strategic Plan 2020/21-2024/25
HIM	Health Information Mediator
HIIRE	Health Information Innovation and Research
HIS	Health Information System
ICT	Information and Communication Technology
ICD	International Classification of Diseases
IFMIS	Integrated Financial Management Information System
IL	Interoperability Layer
IP	Implementing Partner
IT	Information Technology
MFL	Master Facility List
OHIE	Open Health Information Exchange
PII	Personally, Identifiable Information
SDGs	Sustainable Development Goals
TWG	Technical Working Group
VHT	Village Health Team
OSI	Open Systems Interconnection

Definitions of Terms

Health Information Exchange: is the transfer of electronic health information among information systems based on agreed and recognised standards.

Interoperability: is the ability of different information systems, devices and applications (‘systems’) to access, exchange, integrate and cooperatively use data in a coordinated manner, within and across organisational, regional and national boundaries.

Health Information System: is a system designed to manage healthcare data.

Foundational Interoperability: is the ability of one IT system to send data to another IT system in a manner that the receiving IT system does not necessarily need to be able to interpret the exchanged (received) data, it must simply be able to acknowledge the receipt of the data payload. It’s the most basic type of interoperability.

Structural Interoperability: the uniform movement of healthcare data from one system to another such that the clinical or operational purpose and meaning of the data are preserved. To achieve structural interoperability, the receiving system should be able to interpret information at the data field level. This is the intermediate level of interoperability.

Semantic Interoperability: the ability of health IT systems to exchange and interpret information and then actively use information that has been exchanged. This is the highest level of interoperability. Semantic interoperability takes advantage of both the structuring of the data exchange and the codification of the data including vocabulary so that the receiving IT systems can interpret the data.

“New” Organizational Interoperability: includes governance, policy, social, legal and organisational considerations to facilitate the secure, seamless and timely communication and use of data both within and between organisations, entities and individuals.

Digital Health: refers to the use of information and communications technologies in health service delivery.

Client Registry: is a directory that lists all clients and their relevant personal information (names, addresses, etc.). A client registry is designed to assist in uniquely identifying individuals who receive health care services by supporting the centralised storage and retrieval of the client (i.e. patient) identification data and enterprise client identifiers (ECIDs).

Electronic Health Facility Registry: this is an application that allows users to access the list of all health facilities in the country (Master Facility List (MFL)). It provides a focused service to collect, curate, store, and distribute standardised and updated health facility details and resource data to the diverse network of digital health systems in a country.

Health Worker Registry: this is an application that allows authorised stakeholders to access the list of health workers within the various health facilities and the community in the country based on agreed-upon standards. It comprises a minimum dataset on all providers of healthcare services that can be shared with all systems that need them.

Shared Health Record: It is an operational transactional subset of normalised data for a patient from various systems such as Electronic Medical Records (EMRs), Laboratory Information Management System (LIMS) etcetera. This record is queried and updated between the different institutions and systems that are authorised to do so.

Terminology Services: comprises a standardised set of terms explaining what type of service a patient receives, improves communication during clinical procedures, and enables everyone involved in the process of treatment and care to perform more efficiently for the patient's benefit.

HIE Governance: refers to the establishment and oversight of a common set of behaviours, policies, and standards that enable trusted electronic health information exchange among a set of information systems and participants.

Information Model: is a representation of concepts, tools, data flows and the relationships, constraints rules, and operations to specify how systems or data sources are linked.

OSI Model: This is a conceptual framework used to represent how applications communicate over a network.

1. Introduction

1. 1.1 Background

The Government of Uganda through the Ministry of Health (MoH) is committed to leveraging Information and Communications Technology (ICT) in transforming healthcare service delivery in Uganda by enabling information access and supporting healthcare operations, management, decision-making making and policy formulation. This is evidenced by the inclusion of digital health as one of the priorities in the Ministry of Health Strategic Plan [1], listing ICTs as a key enabler for supporting better healthcare service delivery. This has been expounded in the Uganda Health Information and Digital Health Strategic Plan 2020/21-2024/25 [2] whose goal is to strengthen the health information system by leveraging digital health to support health service delivery that achieves Universal Health Coverage (UHC) by 2030.

Health information systems have proved valuable in supporting patient care and improving population health outcomes by making data readily available for decision-making at all levels of the health sector. However, these health information systems are typically isolated within communities, hospitals, clinics, laboratories, district offices, partners, pharmacies, or other organisations. This results in fragmented health records and reports, with each component separate and unlinked from the other. Ultimately, this fragmentation may prevent timely access to necessary information leading to duplication and delayed decision making hence the need for standardised and interoperable health information exchange (HIE). HIE is the secure electronic transfer of health information among healthcare organisations using nationally recognised communication protocols and safety standards.

The implementation and use of HIE technology have influenced patient care by allowing providers direct access to health information, reducing the time to obtain this information, and increasing providers' awareness of patient interactions with the health care system.[1] From a broader provider and patient perspective, timely sharing of a patient's clinical information can improve the accuracy of diagnoses, reduce the number of duplicative tests, improve patient safety by reducing medication and medical errors, increase efficiency by eliminating unnecessary paperwork and handling, provide caregivers with clinical decision support tools for

---

[1] Dixon, Brian & Rahurkar, Saurabh & Apathy, Nate. (2020). 'Interoperability and Health Information Exchange for Public Health.'

more effective care and treatment, engage healthcare consumers regarding their personal health information, improve healthcare quality and outcomes; and reduce health-related costs. From a public health perspective, the exchange of health information fosters a positive relationship with public health agencies, improves public health surveillance, improves public health reporting, and monitoring, and increases the efficiency and quality of public health reporting.

In Uganda, several health information exchange modalities are being used to aid information exchange across systems and organisations; These range from Application Programming Interfaces (APIs), ETL (Extract, Transform, Load) tools, and OpenHIE architecture. In the past years, the HIE modalities faced challenges such as disparate and non-interoperable information technologies, the use of different formats and conventions to exchange health data, no set prior standards, lack of capacity, and a variety of governance challenges around information sharing.

Therefore, there is a need to standardise HIE bearing in mind the known limitations and preconditions as well as provide guidance on best-known practices. These are used to address a specific clinical health interoperability/HIE need such as providing the ability to seamlessly access clients' information generated by different HIS and the possibility of progressively enriching the data at different locations whenever the clients interact with health systems across the country.

The goal of HIE is to facilitate the secure and seamless exchange of patient health information in a standardised format, ensuring that relevant and timely data is available.

It is against this background that the Ministry of Health has developed Guidelines for Health Information Exchange and Interoperability.

2. 1.2 Review of Health Information Exchange

Over the past two decades, large investments in the health sector have led to an increase in the deployment of digital health solutions in Uganda. Most of these solutions are limited in scale, likely to be proprietary and operate in isolation. Lack of interoperability has been recognized as a critical obstacle to realising the potential benefits of digital health in the country.

There have been ad-hoc attempts to implement HIE use cases within the sector, such as data exchange within some aggregate reporting systems, EMRs exchanging data with the national Viral Load Information System, and surveillance mTrac system exchanging data with the

national eHMIS system, among others. However, all these were not premised on nationally endorsed HIE and Interoperability guidelines.

Given the benefits and success stories of implementing health information exchange in some African countries like Rwanda[2] where Individual Patient Care (Clinical) for HIV disease management was improved and the healthcare administration of facilities was further made more efficient.

The Government of Uganda has made considerable efforts to spell out the overall vision of how different sectors including the health sector would be exchanging information in the Uganda e-Government Interoperability Framework[3]

MoH as a custodian of the healthcare system in the country has embarked on developing its national HIE and Interoperability guidelines to ensure that health data collected is capable of being shared and facilitate primary and secondary uses of patient health information (i.e. any health data collected should be available to appropriate users to improve health outcomes).

It is also important that for any two or more heterogeneous systems to exchange data meaningfully, there must be a mutual standard for data exchange or communication between those systems. Health information exchange standards provide clear guidelines for robust and interoperable solutions.

Therefore MoH shall advocate for the use of proven free and open standards, open innovation, open data and where feasible open software, and collaborate with open software organisations to extend existing products to meet HIE needs.

3. 1.3 Document Development Process

The development of the guidelines was carried out by the Digital Health Subcommittee of the Health Information Innovation and Research (HIIRE) Technical Working Group (TWG) in consultation with various committees within MoH and other stakeholders from MDAs, Academia, as well as Development, Implementing and Technology partners.

[2] HIE, O. (2023) 'Creating a Health Information Exchange System in Rwanda'. Available at: https://ohie.org/impact-stories/creating-a-health-information-exchange-system-in-rwanda/
[3] NITAU (2021) 'Uganda e-Government Interoperability Framework (e-GIF)'. Available at: https://www.nita.go.ug/sites/default/files/2023-03/e-GIF.pdf.

Feedback from the various stakeholders was incorporated into the document during the development, validation and approval process.

Reference was made to the Uganda Digital Health Enterprise Architecture, Standards and Knowledge Guidelines [4], the African Union Health Information Exchange Guidelines and Standards [26], internationally recognised standards and guidelines governing HIE and Interoperability that include system standard guidelines, communication and connectivity protocols, messaging standards, and vocabulary standards.

The guidelines were then presented to the HIIRE TWG and the Senior Management Committee for endorsement and then to the Top Management Committee for approval.

4. 1.4 Purpose and Scope of Document

The purpose of HIE is to facilitate the secure, standardised and efficient exchange of electronic health information between digital health systems enabling seamless access to patient data for improved care coordination, patient safety, and healthcare outcomes.

Therefore, this document outlines the guidelines to develop and implement health information exchange and interoperability for digital health systems in Uganda. Whilst the health sector may use a combination of both paper-based and digital exchange solutions for interactions, this document focuses on electronic data, digital data exchange, and protocols to assist the MoH in defining and specifying a framework to guide its long-term implementation of the Health Information and Digital Health Strategic Plan (2020/21-2024/25) [2].

5. 1.5 Target Audience

The document is intended for:

i. Policymakers at all levels.
ii. System Developers and Integrators.
iii. Health and Information Practitioners.
iv. Project and Program Managers.
v. System Implementers.
vi. Researchers and Academia.
vii. Implementing and Development Partners.
viii. Information and Systems Key Stakeholders.

ix. Ministries, Departments and Agencies
x. Private Sector

2. Section A: HIE & Interoperability Guidelines for Digital Health Systems

This section describes the system standard guidelines, communication and connectivity protocols, messaging standards, and vocabulary standards for health information exchange.

6. 2.1 Systems Standard Guidelines

The MoH aspires towards a more integrated, open, and flexible digital health solution. Specifically, common global goods have been recognized across public and private entities as a fundamental course of action towards building interoperable, cost-effective, easy-to-use infrastructure components.

However, digital solutions must be tailored to national needs, circumstances, and resources. Each solution should support incremental extension into a fully integrated national health information system without substantial reengineering. It is not practical to either discard the existing systems or have only one system in place to solve the interoperability problem given that we have many siloed or fragmented systems. At the same time, the MoH encourages the use and application of newer and more modern technologies that have proven real-world application to maximise long-term benefits.

7. 2.2 Communication and Connectivity Protocols

The following communication protocols for digital health systems communications shall be implemented;

Guidelines for Communication Protocols:

1. For sensor communications, standards ISO/IEEE 11073-10101 (Nomenclature) [5], IEEE 11073-10201™ (Domain information model) [6] and IEEE 11073-20101™ (Application profile base standard) [7] shall be used for supporting interoperable communication among sensor devices and computers.
2. For secure internetworking between entities and local area networks, use transport protocols guided in Section B.

3. The health sector shall adopt ANSI/TIA-1179-A [8] for standardised connected health systems. MoH shall ensure that implementing entities procure end-user ICT devices that meet the recommended specifications for all health service delivery points as stipulated in the Uganda Digital Health Enterprise Architecture, Standards and Knowledge Guidelines.
4. Personal data including health data-sharing shall be conducted securely as stipulated in the Data Protection and Privacy Act 2029 and further elaborated in the Uganda Health Data Protection, Privacy and Confidentiality Guidelines [9].

8. 2.3 Messaging Standards

The internationally known comprehensive and robust data exchange standards shall be used. Below are the messaging standards that shall be adopted.

Messaging Standard 1: HL7/FHIR

Definition: Health Level 7 (HL7) Fast Healthcare Interoperability Resources (FHIR) [10] shall be the standard for the exchange of healthcare information. Based on modern design patterns like RESTful communication and the concept of Resources, FHIR covers a broad range of clinical use cases by providing modular building blocks that can be combined without jeopardising information integrity. The built-in methods for extending a Resource allow FHIR to provide flexibility and adaptability in the clinical environment. FHIR combines the best features of HL7v2, HL7 V3R2 CDA, and web standards (XML, JSON, HTTP, Atom, and others).

Objective: To ensure communication between systems that document and manage the care and treatment of patients in a wide variety of healthcare settings.

Guidelines:

1. For public health and clinical content messaging, FHIR and HL7 V2.5.1 and above shall be used.
2. FHIR/HL7 shall be used to facilitate the exchange of healthcare data about admission data, discharge/transfer data, clinical encounters and observations, treatments, prescriptions and dispensing, orders and results of laboratory tests, and appointment schedules among heterogeneous healthcare systems.

3. For clinical content and structure (including clinical summaries, discharge notes, laboratory, and radiology investigation), use HL7 V3R2 Clinical Document Architecture (CDA). Additionally, the HL7 Continuity of Care Document for the exchange of clinical information, including patient demographics, problems, medications, and allergies shall be used.

Messaging Standard 2: DICOM

Definition: Digital Imaging and Communications in Medicine (DICOM) [11] is a file format and transmission standard for exchanging medical images and associated information between medical imaging equipment made by different manufacturers. The DICOM standards are widely adopted in equipment and information systems used in hospitals, imaging centres, and providers' offices to produce, display, store, or exchange medical images.

Objective: To ensure medical images and associated information are stored, queried, retrieved, and transferred between imaging machinery and information systems in an interoperable manner.

Guideline:

1. For the exchange of digital medical diagnostic images, Use DICOM to facilitate the sharing of images such as radiology, radiotherapy, ophthalmology, ultrasound, digital mammography, pathology, dentistry, dermatology, computed tomography, etc, between imaging equipment and other healthcare applications. It specifies data structure as well as the protocols for the exchange of medical images, among others.

Messaging Standard 3: Aggregate Data e-Xchange (ADX)

Definition: ADX is a messaging standard that shall be used to exchange health indicators, aggregate data and metadata. It is derived from SDMX (statistical data and metadata exchange) and based on XML [12].

Objective: To support automated aggregate data exchange of health indicators, aggregate data and metadata with the national eHMIS and other health information systems.

Guidelines:

1. Aggregate Data eXchange (ADX) profile shall be used to support the exchange of health indicators, aggregate data, metadata, etc. The details for the ADX profile shall provide

a formal specification by the data owners (user departments), to guide the generation and exchange of aggregate data from systems.

2. The reporting frequency shall be determined by data owners (user departments).
3. Metadata (data element names, codes, disaggregates, etc) shall be determined by data owners (user departments).

9. 2.4 Vocabulary Standards

Vocabulary standards facilitate semantic interoperability between health providers and systems. The Ministry of Health adopted the following international vocabulary standards that cover business processes and services for the health sector such as SNOMED-CT, ICD-11, LOINC, RxNorm, and DSM.

MoH shall develop, manage and control a national healthcare data dictionary as a central repository for the terminology service. Below is guidance on the use of the contextualised standards;

Vocabulary Standard 1: SNOMED-CT

Systematized Nomenclature of Medicine - Clinical Terminology (SNOMED-CT) shall be the standard used to support coding terms and synonyms for clinical findings, symptoms, diagnoses, procedures, pharmaceuticals, etc. It has an inbuilt mechanism to cater for local extensions and different languages [13].

Vocabulary Standard 2: ICD-11

International Classification of Disease (ICD-11) shall be used for classifying diseases, health conditions, causes of death, clinical terminology, and coding standards [14].

Vocabulary Standard 3: LOINC

Logical Observation Identifiers Names and Codes (LOINC) shall be the standard used for coding laboratory test reports. LOINC codes can be integrated into the data message content such as HL7 V2.5.1 and HL7 CDA for standardising laboratory reports such as chemistry, haematology, serology, microbiology, and toxicology as well as cell counts, antibiotic susceptibilities, and others [15].

Vocabulary Standard 4: RX-Norm

RX-Norm shall be the standardized naming system to codify clinical drugs to allow computer systems to communicate drug-related information efficiently and unambiguously. RxNorm

provides normalised names for clinical drugs and links its name to many drug vocabularies commonly used in pharmacy management and drug interactions [16].

Vocabulary Standard 5: Diagnostic and Statistical Manual of Mental Disorders (DSM). Diagnostic and Statistical Manual of Mental Disorders (DSM) shall be used as the nomenclature that clinicians can reference to enhance clinical practices and as a language for communicating diagnostic information. It contains descriptions, symptoms and other criteria for diagnosing mental disorders [17].

Guidelines for Vocabulary Standards:

1. All users shall ensure they are using the latest copy/ version of the vocabulary standard adopted by MoH.
2. MoH guides that reference to international authorities behind these standards as a source e.g., WHO (ICD11), SNOMED-CT, OCL (Open Concept Library), etc.
3. All users shall ensure that all data elements are mapped to the correct codes.
4. Modalities shall be put in place for MoH and other stakeholders to ensure that the proper skill sets are in place to support the implementation of the vocabulary data elements and codes (for example, workshops facilitated by experts, online training, and community meetings, among others)
5. Public health vocabulary data to support MoH messaging system development, sharing with potential digital health system users, and dissemination of the latest vocabulary and terminology information.

3. Section B: Governance Foundations for HIE and Interoperability Standards

Governance for digital health aims to harmonise, and strengthen the capabilities and skills needed for countries to promote, innovate and scale up digital health technologies. Currently, the MoH coordinates the implementation of this digitization effort and is charged to the DHIM, Department of Planning, Financing and Policy with strategic advice and support from the HIIRE TWG. The HIIRE TWG has constituted a Digital Health sub-committee that provides technical advisory guidance and support to the HIIRE TWG in the implementation and coordination of digital health aspects like health information exchange and interoperability. The HIIRE TWG comprises representation from MoH departments, implementing and development partners, technology partners, academia, private sector, and civil society organisations.

3.1 HIE & Interoperability Development Principles

As guiding principles for the HIE & Interoperability, the following Principles for Digital Development [24] were adopted and shall be followed;

1. Design with the user
2. Understand the existing ecosystem
3. Design for scale and national adoption
4. Build for sustainability
5. Be data-driven
6. Use of appropriate open standards, open source, open data and open innovation
7. Reuse and improve
8. Address privacy and security
9. Be collaborative

10. 3.2 Standards Management

MoH shall ensure that HIE & Interoperability guidelines are followed and adhered to by all implementers of health information exchange and interoperability.

As and when the need arises, MoH shall convene working groups to continually review these standards and to evolve these standards in a thoughtful and controlled fashion.

11. 3.3 Messaging Exchange Standards Support

MoH shall lead and coordinate the establishment of a message testing environment and the development of a message validation tool to validate messaging implementations and associated vocabularies. As part of message processing, the following minimum functions shall be observed;

- Assign a unique anonymous identifier to case-based records traversing the system; this shall prevent the potential duplication of MoH unique case identifiers.
- Exclude PII as needed when sharing and re-transmitting data as may be required.
- Reformat messages into the appropriate recipient-required format (e.g. Unified Messaging and Collaboration System (UMCS), email, secure file, or web page message).
- Enforce the quality of message structure and content.

12. 3.4 Protocols and Services for Interoperability

The following interoperability service areas are critical to achieving digital health systems interoperability. Each service area as briefly described below should comply with the latest version of the corresponding RFC standard or equivalent service, when and as adopted by the MoH. The initially recommended standard for 2020 is identified below as the baseline for interoperability evolution over time.

MoH guides that all implementers of health information exchange and interoperability shall use known and standard secure transfer protocols e.g., File Transfer Protocol over Transport Layer Security (FTP-TLS) [18], Simple Mail Transfer Protocol (SMTP) over Transport Layer Security (SMTP-TLS) [19], Hypertext Transfer Protocol over Transport Layer Security (HTTP-TLS) [20], SSH Tunnels, SFTP [20], etc.

MoH recommends the use of Representational State Transfer (REST) [21] to support access (GET, POST, PUT, PATCH and DELETE) to facilitate system-to-system and user-to-system services exchange using HTTP APIs.

13. 3.5 Support HIE Platform Best Practices

- HIE platform(s) shall be housed in secure facilities with controlled access for only authorised personnel.
- The HIE platforms shall be protected from unauthorised access.
- HIE platforms shall ensure role-based access for system security purposes.

- Staff managing infrastructure related to HIE shall be trained per the training schedule with the responsibilities stipulated.
- Any HIE incident shall be reported promptly within 24 hours for further action.
- Security of HIE systems shall be implemented through the use of system logs or audit trails
- Access to the HIE systems shall be by authorised users.
- All systems required to exchange health data shall be authenticated.
- A secure, remote recovery/backup site should be available in the event of damage to, or loss of, the primary HIE platform and/or facility.
- MoH shall promote research and development in line with HIE.
- Software and system updates shall be tested and verified on a separate (but potentially scaled down) staging system before deployment and use.

4. Section C: Monitoring and Evaluating the Uganda HIE & Interoperability Guidelines

This Section outlines how to monitor and evaluate the implementation of HIE & Interoperability Guidelines and the impact of the HIE. The implementation of the HIE & interoperability guideline and its impact shall be based on the monitoring and evaluation. This is based on the Monitoring and Evaluation framework stipulated in the Uganda Health Information and Digital Health Strategic Plan 2020/21-2023/24 which is essential for compliance and effectiveness of the guideline. Therefore; the guideline shall be monitored in terms of the:-

- Dissemination of the guideline
- Compliance with the guideline
- Technical capacity building on the usage

The HIE platform shall be monitored based on the following: -

- Number of systems connected to the HIE disaggregated by prioritised patient workflows like patient flow, products and commodities, and aggregate report generation.
- Number of facilities integrated on the HIE platform such as financial institutions, health service providers, payers, and government agencies.
- Number of transactions on the health information exchange in the health information mediator including HISs.
- Number of reported and resolved complaints.
- Percentage uptime of the HIE platform.

The following considerations shall also be made;

1. There shall be routine auditing of systems connected to the HIE platform to ensure compliance with the guidelines and standards.
2. There shall be documentation of the process of resolution of cases.
3. There shall be product assurance- conformity assessment.

A checklist shall be developed to monitor the compliance of implementers with the guidelines.

4.1 Dissemination and adoption of the guidelines

The HIE & Interoperability guidelines shall be disseminated for adoption through:

1. Presentation of the guidelines to stakeholders.
2. Posting of the guidelines on the MoH websites and the electronic Library for access by the stakeholders.
3. Organising quarterly workshops to train stakeholders and innovators.

5. Section D: Sustaining the Health Information Exchange

This section outlines the approaches, strategies, tools and methods that the MoH shall adopt to sustain the health information exchange and interoperability processes, and practices to achieve the UHC goals and the Uganda HIDH Strategic Plan 2020/21 - 2024/25. To effectively implement an HIE more sustainably is not only necessary but also shall support MoH and partners to achieve their long-term impact on improved health outcomes.

Overall the Ministry of Health shall work with the Ministry of Information Communication Technology and National Guidance (MoICT&NG) and the National Information Technology Authority Uganda (NITA-U) as a means to achieve the overarching Republic of Uganda Government Enterprise Architecture (GEA), Digital Government Strategy (NITA, n.d.) and E-Government Interoperability Framework (E-GIF) goals and objectives.

This section highlights the basic tenets of sustainability: capacity building; ensuring user and stakeholder contributions are not minimised due to interruptions such as loss of funding; and creating solutions more likely to be embedded into policies, daily practices, and workflows.

A Sustainable Business Model for Uganda HIE

HIEs need high performance, high availability, reliable ICT infrastructure and compatible future technologies. MoH shall utilise its existing infrastructure and data centres provided by the Government of Uganda to host the HIEs at the national and district levels. At the facility level, MoH shall utilize the existing infrastructure to host the individual EMRs at the health facilities. MoH is encouraged to critically evaluate and address the following;

- Procurement framework as per the Public Procurement and Disposal of Public Assets Act 2003, and the NITAU Procurement of ICT Goods and Product Guidelines that

includes content that guides infrastructure procurement, disposal, replacement, upgrade, and other pursuits related to ICT infrastructure maintenance.

- Alignment with the Data Protection and Privacy Act 2019 and the Health Data Protection, Privacy and Confidentiality Guidelines.
- Budget development to encompass monetary quantification of infrastructure initial cost and subsequent operational cost.
- Linkage to the country's digital health investment framework, which possibly adopts common principles such as collaboration, digital global goods, participatory budgeting, instrument tracking plan, and overall capacity building on technical skills for digital health.
- HIE facilitates the interoperability of health information systems allowing for collaboration and information sharing within and across organisational boundaries to advance the health of individuals and communities and the effective delivery of health care to them. Implementing a fully functional HIE infrastructure and ecosystem is no mean feat and requires the presence and interplay of several fundamental components. Indeed, investment in HIE should be considered within the wider digital health ecosystem. The WHO and ITU eHealth strategy toolkit highlights the need to understand a country's "national context for eHealth" when considering digital health investment (TAP).
- Linkage with other sector information management systems as guided in the e-Government Interoperability Framework.

6. Section E: References

1. (MoH), M.o.H., *Ministry of Health Strategic Plan 2020/21 - 2024/25*. 2020, Ministry of Health (MoH): Kampala.
2. MoH, M.o.H., *Health Information and Digital Health Strategic Plan (2020/21-2024/25)*. 2023.
3. Bowen, R., et al., *HIE management and operational considerations*. J ahima, 2011. **82**(5): p. 56-61.
4. MoH, M.o.H., *Uganda Digital Health Enterprise Architecture Framework 2020/21 - 2024/25*.
5. ISO. *ISO/IEEE 11073-10101:2020*
    a. *Health informatics - Device interoperability - Part 10101: Point-of-care medical device communication - Nomenclature*. 2020-08; Available from: https://www.iso.org/standard/77338.html.
6. ISO. *ISO/IEEE 11073-10201:2020*
    a. *Health informatics - Device Interoperability - Part 10201: Point-of-care medical device communication - Domain information model*. 2020 - 05; Available from: https://www.iso.org/standard/77339.html.
7. ISO. *ISO/IEEE 11073-20101:2004*
    a. *Health informatics - Point-of-care medical device communication - Part 20101: Application profiles - Base standard*. 2004; Available from: https://www.iso.org/standard/36347.html.
8. Anixter. *ANSI/TIA-1179*. Available from: https://www.anixter.com/en_us/resources/literature/technical-references/standards/ansi-tia-1179-standard.html.
9. MoH, M.o.H., *Health Data Protection and Confidentiality Guidelines for Uganda 2020/21 - 2024/25*. 2023.
10. International, H. *HL7/FHIR*. Available from: https://hl7.org/fhir/.
11. Secretariat, D. *DICOM*. Available from: https://www.dicomstandard.org/.
12. (IHE), I.t.H.E. *Aggregate Data Exchange - HIV*. 2019; Available from: https://wiki.ihe.net/index.php/Aggregate_Data_Exchange_-_HIV.
13. International, S. *SNOMED*. Available from: https://www.snomed.org/.
14. WHO. *International Classification of Diseases 11th Revision*. Available from: https://icd.who.int/en.
15. LOINC. *The international standard for identifying health measurements, observations, and documents.*; Available from: https://loinc.org/.
16. Medicine, N.L.o. *RxNorm*. Available from: https://www.nlm.nih.gov/research/umls/rxnorm/index.html.
17. Association, A.P. *The Diagnostic and Statistical Manual of Mental Disorders, Fifth Edition*. 2013; Available from: https://g.co/kgs/4HBYbX.
18. Wikipedia. *FTPS*. Available from: https://en.wikipedia.org/wiki/FTPS.
19. Wikipedia. *SMTPS*. Available from: https://en.wikipedia.org/wiki/SMTPS#:~:text=SMTPS%20(Simple%20Mail%20Transfer%20Protocol,not%20an%20extension%20of%20SMTP.
20. Technology, N.I.o.S.a., *Guide to Secure Web Services*, C.S. Division, Editor. 2007.
21. Wikipedia. *Representational state transfer*. Available from: https://en.wikipedia.org/wiki/Representational_state_transfer.
22. TAP. *Health Information Exchange - A Model Guideline*. Technical Assistance Platform.
23. NITA, U. (n.d.). *Digital Government Strategy*. https://nita.go.ug/sites/default/files/2023-03/Digital-Strategy.pdf.

24. Principles for Digital Development *Principles for Digital Development* Available from: https://digitalprinciples.org/
25. Dixon, Brian & Rahurkar, Saurabh & Apathy, Nate. (2020). *Interoperability and Health Information Exchange for Public Health*.
26. Union, A. (2023) *African Union Health Information Exchange Guidelines and Standards*, (September 2023), pp. 1–108.

REPUBLIC OF UGANDA
MINISTRY OF HEALTH

THE UGANDA HEALTH DATA PROTECTION, PRIVACY AND CONFIDENTIALITY GUIDELINES

January 2023

DOCUMENT REVIEWS AND APPROVALS

**Version**	**Owner**	**Author**	**Approver**	**Date of Approval** *(MMDDYY)*
01	Ministry of Health	Division of Health Information Management	Top Management Committee	18.09.2024

TABLE OF CONTENTS

FOREWORD v

PREFACE vi

ACKNOWLEDGEMENT vii

ABBREVIATIONS AND ACRONYMS viii

DEFINITION OF TERMS ix

1.0 INTRODUCTION 1

2.0 PURPOSE AND SCOPE 1

3.0 OBJECTIVES 2

4.0 DEVELOPMENT METHODOLOGY 2

4.1 HEALTH DATA TO BE PROTECTED 3

5.0 HEALTH DATA MANAGEMENT COMPONENTS 3

5.1 DATA COLLECTION 3

5.2 DATA PROCESSING 4

5.3 DATA CORRECTION 4

5.4 PROCESSING PERSONAL DATA OUTSIDE UGANDA 5

5.5 DATA STORAGE 5

5.6 DATA RETENTION, ARCHIVAL AND DISPOSAL 6

5.7 DATA SECURITY BREACHES 6

5.8 RIGHT TO ACCESS PERSONAL INFORMATION 6

5.9 DATA ACCESS CONTROLS FOR THIRD PARTIES 7

5.10 DATA CLASSIFICATION 7

6.0 COMPLAINTS AND INVESTIGATIONS 7

6.1 COMPLAINT HANDLING 7

6.2 HEALTH INFORMATION EXCHANGE 7

7.0 PRINCIPLES FOR DATA PROCESSING 8

8.0 GOVERNANCE STRUCTURE 12

8.1 DATA CONTROLLER 13

8.2 DATA PROCESSOR 14

8.3 DATA PROTECTION OFFICER 16

9.0 RIGHTS OF DATA SUBJECTS 17

9.1 RIGHTS OF DATA SUBJECTS TO THEIR INFORMATION 17

9.2 GENERAL CONDITIONS FOR SUBMITTING REQUESTS 20

10. CONSENT FRAMEWORK 21

10.1 CONSENT FOR DATA COLLECTION AND PROCESSING 21

10.2 PRIVACY NOTICE FOR THE COLLECTION OR PROCESSING OF PERSONAL DATA 22

10.2.1 METHOD OF OBTAINING CONSENT 24

10.3 PROCESSING PERSONAL DATA PERTAINING TO A CHILD 25

10.4 PROCESSING PERSONAL DATA OF SUBJECTS WHO ARE SERIOUSLY ILL OR MENTALLY INCAPACITATED 26

11. DATA SECURITY, IMPACT ASSESSMENT, AND AUDIT 27

11.1 ADEQUATE SECURITY PRACTICES AND PROCEDURES 27

11.2 DATA MANAGEMENT BY DATA PROCESSORS 28

11.3 DATA PROTECTION IMPACT ASSESSMENT 29

11.4 RECORDS MAINTAINANCE 29

12. DATA SHARING OBLIGATIONS 31

12.1 DATA CONTROLLERS SHARING PERSONAL DATA 31

12.2 DATA CONTROLLERS SHARING DE-IDENTIFIED OR ANONYMIZED DATA 31

12.3 RESTRICTIONS ON SHARING, CIRCULATING, OR PUBLISHING OF PERSONAL DATA 33

12.4 LEGAL GROUNDS FOR PROCESSING PERSONAL DATA 33

13.1 GRIEVANCE REDRESSAL 34

13.2 PERSONAL DATA BREACH AND INCIDENT MANAGEMENT 35

13.3 COMPLIANCE AND GUIDELINES GOVERNANCE 35

13.4 NON-COMPLIANCE WITH THESE GUIDELINES 36

13.5 LIABILITY, PENALTIES AND REMEDIES 36

13.6 SALE OF HEALTH DATA 36

14.0 DISSEMINATION AND ADOPTION OF THE GUIDELINES 36

Appendix I: Compliance checklist for data controllers to align with the guidelines 37

Appendix II: Privacy Notice Template 39

Appendix III: Data Protection Privacy and Confidentiality Checklist 43

Appendix IV: Assessing and Improving Cybersecurity Maturity 53

Appendix V: Data Processing Agreement Template 61

FOREWORD

The Government of Uganda promotes the use of data for decision-making and policy formulation. The Ministry of Health Strategic Plan 2020/21 – 2024/25 also recognises the use of data as a key enabler for supporting the health system to deliver good health to the population. This is further articulated in the Uganda Health Information and Digital Health Strategic Plan 2020/21-2024/25.

However, the health sector must uphold the highest standards of data protection, privacy, and confidentiality through operationalising the Data Protection and Privacy Act 2019 within the sector. The Uganda Health Data Protection, Privacy, and Confidentiality Guidelines will serve as a cornerstone in ensuring the integrity and protection of health information in our nation.

In an era where digital technologies are revolutionising the way we collect, process, store, and utilise health data, a robust framework to safeguard sensitive information must be established. These guidelines provide comprehensive guidance to healthcare providers, policymakers, and stakeholders on best practices for protecting health data throughout its lifecycle.

By adhering to these guidelines, we not only fulfil our ethical and legal obligations to safeguard patient privacy but also foster trust and confidence among individuals accessing healthcare services. Furthermore, these guidelines will pave the way for innovation and collaboration in leveraging health data to improve healthcare delivery, research, and public health interventions.

All stakeholders in the health spectrum are therefore called upon to adopt the use of these guidelines while handling health-related data.

....

Dr. Henry G. Mwebesa

DIRECTOR GENERAL HEALTH SERVICES

PREFACE

This document presents Uganda's Health Data Protection, Privacy, and Confidentiality Guidelines for the Health Sector. These guidelines are intended to standardize the implementation of health data protection, privacy and confidentiality across Uganda’s health system. They are aligned with the Data Protection and Privacy Act 2019, Data Protection and Privacy Regulations 2021, National ICT Policy 2018, Health Information and Digital Health Strategic Plan 2020/2021-2024-2025, Ministry of Health Strategic Plan 2020/2021-2024-2025. The guidelines are also aligned with the Uganda Digital Health Enterprise Architecture, Standards and Knowledge Guidelines.

Health Information and supporting systems are critical assets for health service delivery. In Uganda like in many places, health data is faced with many privacy and confidentiality threats from a wide range of sources. Therefore, protecting the health data of Uganda’s health system clients is of prime importance to the Ministry of Health. These guidelines aim to support all health stakeholders in the collection, transmission and storage of health data.

These guidelines will serve as a framework to ensure the protection of personal health data, secure information access, exchange, and storage as well as timely identification and addressing of vulnerabilities.

In addition, the guidelines will ensure that health data protection, privacy and confidentiality are observed by all health practitioners (including public and private) while handling health data (from collection, processing, storage and access). All health data stakeholders are called upon to embrace the use of these guidelines while handling any form of health data.

....

Dr. Sarah Byakika

Commissioner Health Services

Department of Planning, Financing and Policy

ACKNOWLEDGEMENT

The Ministry of Health expresses its profound gratitude to all departments and programs, members of the Health Information Innovation and Research Technical Working Group, and the Digital Health Subcommittee, who contributed technical inputs leading to the successful completion of this document. Special appreciation goes to the Division of Health Information Management (DHIM) and the Information Communication Technology (ICT) Section for the overall guidance to ensure that the guidelines are aligned with the Health Information and Digital Health Strategic Plan 2020/21-2024/25.

I acknowledge and thank all development and implementing partners that provided financial and technical support for this process, specifically MUSPH/METS, HISP, IDI, CDC, The Technical Assistance Platform (TAP) and the University of Warwick. DHIM is grateful for all the support, sacrifice, and contributions that have been invested in the successful development of these guidelines.

Finally, the Ministry of Health is grateful to the Ministry of ICT, NITA-U, Uganda Health Informatics Association, Makerere University Health Informatics Research Group and all those institutions and individuals who have not been specifically mentioned above, but who directly or indirectly contributed to the successful development and finalisation of the Uganda Health Data Protection, Privacy and Confidentiality Guidelines.

....

Mr. Paul Mbaka

Assistant Commissioner Health Services

Health Information Management

ABBREVIATIONS AND ACRONYMS

DHIM	Division of Health Information Management
HIIRE	Health Information Innovation and Research
ICT	Information Communication Technology
MOH	Ministry of Health
TWG	Technical Working Group
PDPO	Personal Data Protection Office

DEFINITION OF TERMS

Anonymization	Is an irreversible process of transforming or converting personal data to a form in which a data subject cannot be identified through any means reasonably.
Biometric Data	Personal data resulting from measurements and calculations relating to the physical, physiological, or behavioural characteristics of a natural person that allow or confirm the unique identification of that natural person, such as facial images or fingerprint data.
Confidentiality	The obligations of those who receive information are to respect the privacy interests of those to whom the data relates.
Consent	Any freely given, specific, informed, and unambiguous indication of the data subject's wishes by which they, by a statement or by a clear affirmative action, signify agreement to the collection and processing of personal data relating to them.
Consent Artefact	A machine-readable document that specifies the parameters and scope of data access, sharing and use that a data subject consents to in any personal data-sharing transaction.
Consent Manager	A person who facilitates the consent process by providing tools and mechanisms for obtaining, managing, and documenting user consent in a transparent and user-friendly manner.
Data	Data means information which—<br>a) Is processed using equipment operating automatically in response to instructions given for that purpose;

	b) Is recorded with the intention that it should be processed using such equipment;<br>c) Is recorded as part of a relevant filing system or with the intention that it should be part of a relevant filing system; or<br>d) Does not fall under paragraphs (a), (b), and (c). But forms part of an accessible record.<br><br>A representation of information, facts, concepts, opinions, or instructions in a manner suitable for communication, interpretation, or processing by humans or by automated means.
Data Collector	A person or an entity that collects personal data.
Data Controller	A natural or legal person (e.g., an organization or other entity) who alone, jointly with other persons, in common with other persons, or as a statutory duty determines the purpose for and the manner in which data is processed or is to be processed.
Data Processor	A natural or legal person (e.g., an organisation or other entity) that processes personal data on behalf of the data controller.
Data Protection Officer	Is an individual employed by a data controller who understands and applies data protection regulations to the data control operations and serves as a liaison between the data controller and regulators.
Data subject	An individual from whom or in respect of whom personal information has been requested, collected, collated, processed, or stored.
De-identification	The process by which a data controller or data processor may remove or mask identifiers from personal data, or replace them with a

	fictitious name or code that is unique to a data subject but does not, on its own, directly identify the data subject.
Health Data	Personal data related to the physical or mental health of a natural person, including the provision of health care services that reveal information about their health status.
Natural persons	A living human being
Nominee	Is a natural person or legal entity who is selected or appointed by a data subject to act on their behalf in a specific capacity to access their personal data in the event of death or physical incapacity.
Personal data	Information about a person from which a person can be identified that is recorded in any form and includes data that relates to —<br>a) The nationality, age, or marital status of a person;<br>b) Education level or occupation of a person;<br>c) An identification number, symbol or other particulars assigned to a person;<br>d) Identity data; or<br>e) Other information that is in possession of, or is likely to come into the possession of a data controller and includes an expression of opinion about the individual.<br>Any information relating to an identified or identifiable natural person (i.e., a data subject).
Personal health identifier	A subset of "personal data", is the data that, alone or combined with other information could potentially identify a data subject and/or be used to distinguish one data subject from another; a

	personal health identifier could also be used to re-identify previously de-identified data and could include a data subject's demographic and location information, family and relationship information, and contact details.
Personally Identifiable Information	Any personal data that can be used to clearly identify an individual. Personally identifiable information includes names, physical and internet protocol addresses, financial information, login information , biometric identifiers, video footage, geographic location data, social media accounts, email addresses, and insurance identification number.
Privacy	An individual's right to control the acquisition, use, or disclosure of their personal information.
Protection	The process of protecting sensitive information from damage, loss, or corruption.
Pseudonymization	The processing of personal data in such a manner that the personal data can no longer be attributed to a specific data subject without the use of additional information, provided that such additional information is kept separately so as to ensure that the personal data is not attributed to an identified or identifiable natural person.
Sensitive personal data	A subset of "personal data", means such personal information that consists of information relating to an individual's (i) password; (ii) financial information such as bank account, credit card, debit card, or other payment instrument details; (iii) physical, physiological, or mental health condition; (iv) sexual orientation; (v) political opinions, religious or philosophical beliefs, data revealing racial or

	ethnic origin, or trade union membership; (vi) medical records and history; (vii) biometric information; or (viii) details provided to entities providing services and processed or stored under lawful contract or otherwise provided that any information that is freely available or accessible in a public domain or made available under any applicable law for the time being in force shall not be regarded as sensitive personal data or information for the purposes of these rules.
Third-party	A natural or legal person, public authority, agency, or body other than the data subject, data controller, or data processor, and a person who, under the direct authority of the data controller or data processor, is authorized to process personal data.

1.0 INTRODUCTION

The Ministry of Health recognises the need to harness data for improved health service ‘delivery, and it is in the process of digitising health service delivery in line with its Health Information and Digital Health Strategic Plan 2020/21 – 2024/25, Ministry of Health Strategic Plan 2020/21 – 2024/25, the National Development Plan III, and the Uganda Vision 2040, among others.

Digitisation of service delivery is recognised by the Government of Uganda as a key enabler for service optimisation. However, the Ministry of Health is cognisant of the fact that there is a need to adhere to the National Data Protection and Privacy Act 2019.

This document presents the Health Data Protection, Privacy, and Confidentiality Guidelines for Uganda that operationalises the Data Protection and Privacy Act 2019, and the Data Protection and Privacy Regulations 2021 within the health sector.

2.0 PURPOSE AND SCOPE

The guidelines seek to establish an effective, transparent, and accountable framework for managing health data protection, privacy, and confidentiality and ensuring compliance with the Data Protection and Privacy Act 2019.

These guidelines aim to ensure “Security and Privacy by Design” for the protection of personal data (both digital and paper-based) concerning the health of individuals living in the country. The guidelines shall set out the minimum standards for data protection, privacy, and confidentiality that should be followed throughout the health sector to ensure compliance with the Data Protection and Privacy Act 2019.

These guidelines are to be read along with, and not in contradiction to, any applicable law, or any instrument having the effect of any law; policies relating to information security and privacy, data retention, and data archiving; and any other policies or guidelines that may be notified from time to time.

OTHER LEGAL FRAMEWORKS

These guidelines are aligned with the following national documents adopted by the Government of Uganda; Computer Misuse Act 2011, the National Information Security Policy (framework) 2014, the Electronic Signatures Act 2011, the National Records and Archives Act 2001, the Access to Information Act (2005), and the Electronic Transactions Act 2011.

3.0 OBJECTIVES

The main objective of these guidelines is to provide guidance and establish a framework for the secure collection, processing, storage and use of personal and sensitive data within the health sector.

The specific objectives of these Guidelines include;

i. Protection of personal data, including personal health identifiers, within the jurisdiction of the national health care system by implementing adequate technical measures across the national health care system;
ii. Establish appropriate institutional mechanisms for auditing the data privacy, protection, and confidentiality controls, as needed, and support stakeholders and ecosystem partners to adopt the data protection principles established in these Guidelines;
iii. Establish that all eligible entities adhere to and comply with the applicable laws, rules, regulations, and any other standards about the protection and processing of personnel with in the health sector

4.0 DEVELOPMENT METHODOLOGY

A highly consultative approach was used in the development of the guidelines. Stakeholders from the Ministry of Health (MoH), Ministry of ICT and National Guidance, Personal Data Protection Office (PDPO), Ministry of Public Service, NITA-U, Development and Implementing partners, Civil Society Organizations, Academia, and other members of the Health Information Innovation and Research (HIIRE) Technical Working Group (TWG) collaborated in developing these guidelines.

4.1 HEALTH DATA TO BE PROTECTED

The personal data to be protected includes but not limited to;

1. Information about an individual for the provision of health services, research, or enforcement of the law. [1]
2. Information about payments or eligibility for healthcare concerning the individual.
3. A number, or particular symbol assigned to an individual to uniquely identify the individual for health purposes.
4. Information derived from the testing or examination of a body part or bodily substance, and identification of a person (e.g., a health professional) as a provider of healthcare to the individual.
5. Health data in various forms, including; electronic and paper-based data, pictures, audio, and videos.

5.0 HEALTH DATA MANAGEMENT COMPONENTS

This section of the document introduces health data management components to be protected under the Health Data Protection, Privacy, and Confidentiality Guidelines for Uganda.

5.1 DATA COLLECTION

All stakeholders involved with collecting and/ or capturing health information are required to:

a. Define the objective(s) for collecting and/ or capturing data [5] before information-gathering activities.
b. Define how the data shall be collected, transmitted, and stored.
c. Define the volume of data collected at any moment (quantity).
d. Ensure all proposed data to be collected and/ or captured, including tools, are approved by the data controller.
e. Obtain consent from the data subjects before data collection.
f. Have a data risk assessment before data collection.
g. Electronic data shall be collected per the MoH ICT Policy Guidelines

5.2 DATA PROCESSING

1. Data processing means any operation that is performed upon collected data by any means or otherwise, including:
   a. Organisation, adaptation or alteration of the information or data;
   b. Retrieval, consultation or use of the information or data
   c. Disclosure of the information by transmission, dissemination or otherwise making available, or;
   d. Alignment, combination, blocking, erasure, or destruction of the information or data
2. Before data processing, health data shall be classified using the following:
   a. Based on its sensitivity to disclosure, low-risk, medium-risk, and high-risk
   b. The criticality of information should always be classified through a risk assessment, i.e., obligatory and supplementary. The extent to which the information is essential for the ongoing provision of healthcare
   c. Based on time, it can be classified as current, future or historical
3. Data processing shall be conducted in alignment with the MoH ICT Policy Guidelines;

   a. Request to correct or delete personal data
   b. The data controller shall design a relevant form that shall be used to process any requests from data subjects.

5.3 DATA CORRECTION

a. A data subject shall, in writing, request the data controller to correct or delete their personal data which is inaccurate, irrelevant, excessive, out of date, incomplete, misleading, or obtained unlawfully; or
b. Destroy or delete a record of personal data about the data subject held by the data controller that the data controller no longer has the authority to retain.
c. Upon receipt of the request, the data controller [18] considers the request and informs the data subject in writing of its decision within seven days after receipt of the request.
d. Where the data controller is satisfied [19] [20] with the request, the data controller shall comply.

e. Where the data controller cannot comply with the request, the data controller shall, in writing, inform the data subject of the rejection, the reasons for the rejection, and any action taken as a result of the request.

f. The manner of informing the data subject about the correction made shall be determined by the data controller, taking into consideration the correction made, the nature of the personal data, and the number of persons to whom the change has to be communicated.

5.4 PROCESSING PERSONAL DATA OUTSIDE UGANDA

a. A data collector, data processor [22], or data controller shall not process or store personal data outside Uganda unless such data collector, data processor or data controller seeks authorisation from the PDPO [23]

b. That the country outside Uganda where the personal data [24] is to be processed or stored has adequate measures in place for the protection of the personal data, at least equivalent to the protection provided for by the Data Protection and Privacy Act 2019.

5.5 DATA STORAGE

a. Health information shall be stored as guided by the MoH. The Division of Health Information Management (DHIM) shall be responsible for developing the SOPs and ensuring compliance of all health stakeholders (as stated in the MoH ICT Policy Guidelines, and the relevant Data Management Guidelines)

b. Minimize data risks by ensuring that user devices only access data required to perform approved business activities at any given time (as stated in the MoH ICT Policy Guidelines).

c. The use of privately owned devices to handle health data should conform to the Ministry of Health's ICT Policy Guidelines.

d. Digital Health Information shall be encrypted to protect it from unauthorised access and theft.

e. Electronic Data storage shall be conducted as detailed in the Ministry of Health's ICT Policy Guidelines.

f. All persons and entities collecting, processing and storing health data shall be registered by the PDPO.

g. A registry of third-party entities processing data on behalf of MoH shall be maintained at MoH. [17]

5.6 DATA RETENTION, ARCHIVAL AND DISPOSAL

a. All health data shall be retained for a minimum period of 5 (five) years from the date of creation before being archived. However, organizations shall establish their policies for the retention of health data based on legal requirements, industry standards, and operational needs while observing the minimum period for data retention. These policies shall outline specific retention periods for different types of health records, such as medical records, diagnostic test results, treatment plans, and billing information.

   The retention period shall also vary depending on the purpose for which the data was collected. For example, some data may need to be retained for a longer period for continuity of care, while other data may only be needed for a specific period for billing or research purposes. Organisations should assess the ongoing need for health data and establish appropriate retention periods based on its relevance and usefulness.

b. Ministry of Health and stakeholders handling sensitive patient health information for Ugandans shall only dispose of data in line with the Ministry of Public Service, and National Records and Archives Act 2001.

c. Patients shall have the right to request for deletion or destruction of their health data under certain circumstances, such as when the data is no longer needed for the purposes for which it was collected.

5.7 DATA SECURITY BREACHES

In case of a data security breach, the Ministry of Health ICT Policy Guidelines specifically the Incident Management Plan shall take effect and all incident management processes shall be followed and applied in reference to Section 11.2.

All data breaches shall be reported to the PDPO immediately after becoming aware of it

5.8 RIGHT TO ACCESS PERSONAL INFORMATION

Where the need arises, a data subject shall have to request access to personal information from the data controller who shall grant access after thorough verification. A request for access to personal information shall be made in a prescribed form or manner.

5.9 DATA ACCESS CONTROLS FOR THIRD PARTIES

Where there is a need to allow third-party access to the information processing facilities or personal health information, MOH or the relevant Data Controller shall carry out a risk assessment to identify any requirements for specific security controls. These controls shall include;

a. The legitimacy of the data requester must be ascertained.
b. The purpose for which the data is requested must be clear to the controller.
c. The request must be formal; in writing (either digital or paper)

5.10 DATA CLASSIFICATION

Data classification shall follow Section 8.0 of The Uganda Health Data Access, Sharing and Use Guidelines.

6.0 COMPLAINTS AND INVESTIGATIONS

6.1 COMPLAINT HANDLING

Any complaint related to health data protection, privacy, and confidentiality shall be reported to the person designated as the Data Protection Officer within the institution or the overall accountable person of the respective institution.

6.2 HEALTH INFORMATION EXCHANGE

Specific guidance on health information exchange can be found in the Uganda Digital Health Enterprise Architecture, Standards and Knowledge Guidelines, and Uganda Health Information Exchange and Interoperability Guidelines. The following guidelines shall be considered for health information exchanges.

a. Each HIE participant must comply with the Data Protection and Privacy Act 2019.
b. Each HIE participant must comply with Health Data Protection, Privacy, and Confidentiality Guidelines.
c. Each HIE participant must comply with Health Data Access, Sharing and Use Guidelines.
d. Each HIE participant shall report to their Organisation Management and the Personal Data Protection Office any breaches of confidentiality.

e. Established limitations shall be placed on the use and disclosure of protected health information.

f. Protected health information shall be secured by appropriate administrative, physical, and technical safeguards.

g. Each HIE participant shall report to the Ministry of Health any use of protected health information outside the established terms and conditions.

7.0 PRINCIPLES FOR DATA PROCESSING

These Guidelines shall ensure that data controllers and data processors adhere to the following principles while processing any personal data as permitted under the provisions of the applicable law(s):

a) Data processing must be lawful, fair, and transparent to the data subject. Data controllers shall take all necessary steps to maintain transparency in processing any personal data and shall make the following information available to the Ministry of Health as may be required:

   i. The classification (Section 5.2 (2)) of personal data generally collected and the manner of such collection;
   ii. The purposes for which the personal data are generally processed;
   iii. Any classification of personal data processed in exceptional situations, or for exceptional purposes that create a risk of significant harm.
   iv. The existence of, and the procedure for, the exercise of rights of data subjects and any related contact details for the same;
   v. The grievance redressal procedure;

b) In addition to the information specified above, the data controller shall also notify the data subject, from time to time, of important operations in the processing of any personal data related to the data subject. The information provided to the data subject shall be in an intelligible form, using clear and plain language.

c) Purpose limitation: All personal data collected and processed by data controllers should be for specific, clear, and legitimate purposes specified explicitly to the data subject in

the privacy notice (Section 10.2) and consented to by the data subject. Further processing in the interest of public health archiving, scientific or historical research, or statistical purposes shall, in accordance with the relevant laws of the country such as the Data Protection and Privacy Act 2019, Uganda National Health Research Organisation Act, 2011, and Public Health Act among others.

d) Data minimization: Data collection and processing should be limited to as much data as absolutely necessary for the purposes specified. However, data minimization should not risk data quality or the quality of care. If data minimization risks the quality of care, it should be excluded from data processing.

e) Storage limitation: Personal data should be kept in a form that permits identification of data subjects for not longer than is necessary for the purposes for which the personal data are processed; personal data may be stored for longer periods in far as the personal data shall be processed solely in the interest of public health archiving, scientific or historical research, or statistical purposes, subject to implementation of the appropriate technical and organizational measures required by these Guidelines to safeguard the rights and freedoms of the data subject. Refer to Section 5.6 of these guidelines for further guidance on health data retention, archival and disposal.

f) Integrity and confidentiality: Data processing must be done in a manner that ensures appropriate security of personal data, including protection against unauthorized or unlawful processing and accidental loss, destruction, or damage, using appropriate technical and organizational measures.

g) Privacy by Design: Data controllers shall consider data protection requirements as part of the design and implementation of their systems, services, products, and business processes. The Privacy Notice issued and the principles of Privacy by Design followed by the data controllers should align with these Guidelines and applicable law. Data controllers shall prepare a Privacy Notice containing the following information:

i. Clear and easily accessible statements of their practices and policies;
ii. Type(s) of personal or sensitive personal data collected.

iii. The purpose of collection and usage of such personal or sensitive personal data;
iv. Whether personal or sensitive personal data are being shared with other data controllers or data processors; and
v. Reasonable security practices and procedures used by the data controller to safeguard the personal or sensitive personal data that is being processed.

h) The Privacy Notice referred to in Section 10.2 shall be published on the website of the data controller. In addition, the data controller shall make available a Privacy by Design statement on its website containing the following information:

i. The managerial, organizational, and business practices, and technical systems designed to anticipate, identify, and avoid harm to the data subject;
ii. The obligations of data controllers;
iii. The technology used in the processing of personal data, in accordance with commercially accepted or certified standards;
iv. The protection of privacy throughout data processing from the point of collection to deletion of the personal data;
v. The processing of personal data in a transparent manner; and
vi. The fact that the interest of the data subject is accounted for at every stage of processing of personal data.

i) Choice and consent-driven sharing: Data controllers shall give data subjects a choice to opt in/opt out of the data sharing and processing and take their consent in accordance with Section 8.1 (a) of these Guidelines before accessing, sharing, or processing any of their personal data. This consent shall be free, informed, clear, and specific with respect to the purpose(s) identified in the privacy notice issued under Section 10.2 of these Guidelines. Insofar as the sharing or disclosure of any personal data is concerned, the technical design of the consent management framework should also ensure interoperability across the entire health information exchange ecosystem. The framework should be agnostic to applications, programming languages, and platforms.

j) Empowerment of data subjects: Data controllers should strengthen the rights of data subjects about their personal data. Data subjects shall enjoy rights as specified in Section 9.0 of these Guidelines.

k) Data quality: Data controllers shall take necessary steps so that the personal data that is processed is updated, complete, accurate, not misleading, and relevant to the purpose(s) for which it is processed. All personal data should be reliable and verifiable. However, the data controller shall not be responsible for the authenticity of the personal data supplied to them by the data subject. Personal data once created cannot be erased or amended without following the due process referred to in Section 5.3 and Section 5.6 of these Guidelines. All personal data must also be traceable to its collector unambiguously.

l) Accountability: Data controllers must be able to demonstrate compliance with the above principles (Section 7.0). They shall be accountable for complying with measures that give effect to the privacy principles while processing any personal data. However, the true ownership and control of the personal data shall remain with the data subjects.

m) Classification of personal data: Special provisions Section 5.2 (2) apply to the following types of personal data, whether or not they are related to health data:

i. Information about an individual for the provision of health services, research, or enforcement of the law.
ii. Information about payments or eligibility for healthcare concerning the individual.
iii. A number or particular symbol assigned to an individual to uniquely identify the individual for health purposes.
iv. Information derived from the testing or examination of a body part or bodily substance, and identification of a person (e.g., a health professional) as a provider of healthcare to the individual.
v. Health data comes in various forms, including; electronic and paper-based data, pictures, audio and videos.
vi. Health status.
vii. Genetic or biometric data to uniquely identify a natural person;
viii. Data concerning a natural person's sexual activities or sexual orientation;
ix. Political opinions, religious or philosophical beliefs, data revealing tribal or ethnic origin;
x. Digital footprint created by users while accessing health data from an electronic system.

8.0 GOVERNANCE STRUCTURE

The Governance of health data protection, confidentiality and privacy shall follow the existing governance and management structures at the national and decentralized levels, as summarized in Figure 1.

Technical oversight is the mandate of the Division of Health Information Management (DHIM) under the Department of Planning, Financing and Policy which guides and coordinates all stakeholders involved in health data collection, processing, use and storage. This function is similarly decentralized at the Local Government level, as summarized in Figure 1.

The main task of the Health Information Innovation and Research Technical Working Group (HIIRE TWG) is reviewing and giving advice on Health Information Systems (HIS) and Digital Health policy and strategic related issues from the user departments and other stakeholders.

National Governance & Management Structures
Top Management
Senior Management
Health Information, Innovation and Research Technical Working Group (HIIRE TWG)
Department of Planning, Financing and Policy (Division of Health Information)

Local Government Level Governance & Management Structures
Technical Planning Committee
Extended District Health Management Team
District Health Team
District Health Office (Health Facilities)

The Ministry of Health together with the Personal Data Protection Office (PDPO) shall be responsible for monitoring the application of these Guidelines in order to protect the fundamental rights and freedoms of natural persons in relation to the collection, processing, use and storage of their personal data and to facilitate the free flow of personal data within and outside the country in relation to health service delivery.

The PDPO shall represent the supervisory authority in any dispute or conflict arising from infringement of the provisions of these Guidelines.

At the national and subnational levels, a Data Protection Officer (DPO) shall be designated who shall, in addition to the functions identified under these Guidelines, communicate with regulators and external stakeholders on matters concerning data privacy and serve as an escalation point for decision-making on health data governance and other matters concerning health data.

The roles of the data controller, data collector, data processor, data protection officer, and data protection authority are described below;

8.1 DATA CONTROLLER

Technical and organizational measures: The data controller shall implement appropriate technical and organizational measures to ensure and to be able to demonstrate that data processing is performed in accordance with these Guidelines and any other applicable regulation. Appendix I provides a compliance checklist based on the Data Protection and Privacy Regulations 2021 compliance checklist. The data controller shall consider the nature, scope, context, and purposes of data processing as well as the risks and likelihood and severity of these risks as they relate to the rights and freedoms of data subjects to determine the technical and organizational measures. Those measures shall be reviewed and updated where necessary.

i. Taking into account the latest technological advancements, the cost of implementation, and the nature, scope, context, and purposes of data processing as well as the risks and likelihood and severity of these risks on the rights and freedoms of natural persons posed by the processing, the data controller shall, both at the time of the determination of the means for processing and at the time of the processing itself, implement appropriate technical and organizational measures, such as pseudonymization, that are designed to implement data protection principles, such as data minimization, in an effective manner and to integrate the necessary safeguards into the processing in order to meet the requirements of these Guidelines and protect the rights of data subjects.
ii. The data controller shall implement appropriate technical and organizational measures (including physical measures) for ensuring that, by default, only personal data that is necessary for each specific purpose of the processing is processed. That obligation applies to the amount of personal data collected, the extent of its processing, the period of its storage, and its accessibility. In particular, such measures shall ensure that by

default personal data is not made accessible to an indefinite number of natural persons without the data subject's intervention.

iii. Code of conduct: Adherence to approved codes of conduct or approved certification mechanisms, as approved by the PDPO may be used as an element by which to demonstrate compliance of the data controller with the obligations set forth herein.

iv. Data breach notification: The data controller must notify the PDPO of any personal data breach immediately after becoming aware of it.

v. In the notification, the data controller must detail the nature of the breach, who has the most information on the breach, and the consequences and measures taken to address the breach.

vi. If the data breach poses a high risk to the rights and freedoms of data subjects, the data controller shall communicate the breach to the data subjects within 24 hours.

vii. Adherence to Guidelines requirements: Where data processing is to be carried out on behalf of a data controller, the data processor shall use only data processors providing sufficient guarantees to implement appropriate technical and organizational measures in such a manner that data processing shall meet the requirements of these Guidelines and thus protect the rights of the data subject.

8.2 DATA PROCESSOR

i. Adherence to Guidelines requirements: Data processing shall be carried out on behalf of the Data Controller. The data processor shall only implement appropriate technical and organizational measures after obtaining sufficient guarantees from the data controller and ensuring that data processing shall meet the requirements of these Guidelines and thus protect the rights of the data subject.

ii. Authorization: The data processor shall not engage another data processor without prior specific or general written authorization of the data controller. In the case of general written authorization, the data processor shall inform the data controller of any intended changes concerning the addition or replacement of other data processors, thereby allowing the data controller to object to such changes.

iii. Health Data sharing agreement: Health Data processing by a data processor shall be governed by a data sharing agreement as per the Uganda Health Data Access, Sharing

and Use Guidelines and any other applicable law that is binding on the data processor with regard to the data controller and that sets out the subject matter and duration of the processing, the nature and purposes of the processing, the type of personal data, the categories of data subjects, and the obligations and rights of the data controller and the data processor.

iv. Data sharing agreement applicability: Where a data processor engages another data processor for carrying out specific processing activities on behalf of the data controller, the same data protection obligations as described in Section 6.2 (iii) shall be imposed on that other data processor by way of a data sharing agreement or other legal contract under the applicable country law, in particular providing sufficient guarantees to implement appropriate technical and organizational measures in such a manner that the processing shall meet the requirements of these guidelines. Where that other data processor fails to fulfil its data protection obligations, the initial data processor shall remain fully liable to the data controller for the performance of that other data processor's obligations.

v. Adherence to code of conduct: Adherence of a data processor to an approved code of conduct or approved certification mechanisms, as approved by the PDPO, may be used as an element by which to demonstrate sufficient guarantees as referred to in Section 6.2 (i & iv).

vi. Legal basis: Without prejudice to an individual data-sharing agreement between the data controller and the data processor, the data-sharing agreement or other legal contracts in Section 6.2 (iii & iv) may be based, in whole or in part, on standard contractual clauses referred to in Section 6.2 (vii).

vii. Authority: The PDPO may lay down standard contractual clauses for the matters referred to in Section 6.2 (iii & iv) and in accordance with the governance procedures referred to in Section 6.2 (ii).

viii. Format: The data sharing agreement or other legal contracts referred to in Section 6.2, Bullets (iii) and (iv) shall be in writing, including in electronic format.

ix. Data processor as data controller: Without prejudice to Section 8.2 (ii–iv), if a data processor infringes these Guidelines by determining the purposes and means of processing, the data processor shall be considered to be a data controller in respect of that processing.

8.3 DATA PROTECTION OFFICER

a. Knowledge and expertise: The DPO shall be responsible for understanding data protection regulations and how they apply to the data controller, data processor and data collector; advising people in the organization about their responsibilities, conducting data protection training, conducting audits and monitoring of Guidelines/regulation compliance, and serving as a liaison with regulators.
b. Engagement: The data controller, data processor and data collector shall ensure that the DPO is involved, properly and in a timely manner, in all issues that relate to the protection of personal data.
c. Access: Data subjects may contact the DPO about all issues related to the processing of their personal data and to the exercise of their rights under these Guidelines.
d. Code of conduct: The DPO shall be bound by secrecy or confidentiality concerning the performance of their tasks, in accordance with Data Protection and Privacy Act 2019.
e. Conflict of interest: The DPO may fulfil other tasks and duties. The data controller, data processor, or data collector shall ensure that any such tasks and duties do not result in a conflict of interest.
f. Independence: The DPO shall be able to act independently, without any interference or influence from the data controller, data processor or data collector.
g. Key tasks: The DPO shall have at least the following tasks:
    i. Inform and advise the data controller, the data processor or the data collector of their obligations pursuant to these Guidelines and the Data Protection and Privacy Act 2019.
    ii. Educate and train employees who execute the Guidelines;
    iii. Monitor awareness-raising and training of staff involved in data processing operations;
    iv. Monitor compliance with these Guidelines, with Uganda data protection laws, and with the policies of the data controller or data processor or data collector in relation to the protection of personal data, including the assignment of responsibilities, and the related audits;

v. Provide advice where requested about the data protection impact assessment and to monitor its performance pursuant to Section 9 (iii);

vi. Guide any assessment of these Guidelines and initiate a Guidelines revision/update.

vii. Risk management: The DPO shall, in the performance of their tasks, have due regard for the risks associated with data processing operations, considering the nature, scope, context, and purposes of the data processing.

viii. Support: The data controller, data processor and data collector shall support the DPO in performing the tasks referred to in these Guidelines by providing the resources necessary to carry out those tasks, including access to personal data and data processing operations, and to maintain the data controller's expert knowledge.

ix. Supervision: The DPO shall directly report to the institutional management structures in place. He shall execute his roles/duties without interference from the data controller, the collector and the data processor as referenced in Data Protection and Privacy Regulations 2021. The DPO shall not be dismissed or penalized by the data controller or data processor or data collector for performing the tasks of a DPO.

9.0 RIGHTS OF DATA SUBJECTS

9.1 RIGHTS OF DATA SUBJECTS TO THEIR INFORMATION

Data subjects can request the following from data controllers:

a. Confirmation and access

Data subjects can obtain from data controllers the following information:

i. A confirmation as to whether the data controller has processed any personal data of the data subject;

ii. The personal data that has been processed or a summary of the same;

iii. A summary of processing activities carried out on such personal data; and

iv. Any information provided under the notice issued in accordance with Section 10.1 (a) of these Guidelines in relation to such processing.

b. The data controller shall provide the information under Section 9.1 (a) in a clear and concise manner that is comprehensible to a reasonable person.
c. The data subject shall also have the right to access, in one place, the identities of all the data controllers with whom their personal data has been shared and the categories of personal data that have been shared.
d. Correction and erasure:

   Data subjects can, with regard to the purposes for which their personal data are processed, rectify any inaccurate or misleading personal data, complete any incomplete personal data, and update any out-of-date personal data as outlined in Section 5.3 and Section 5.6. That is;

   i. A data subject shall have the right to initiate modification or correction of personal data stored in an electronic or paper system.
   ii. A data subject shall provide lawful justification and specify the type and data elements that should be modified or corrected. This justification shall be documented by the data controller for future reference

When data subjects request that their personal data be corrected or erased, the following rules shall apply:

i. The data may be corrected or erased if the storage of the personal data violates data protection principles or if the personal data is no longer necessary for the purpose for which it was processed.
ii. If the storage of personal data for a certain period of time is mandated by law, it cannot be erased.
iii. Data subjects can request the data controller to delete the uploaded personal data stored in their personal health record.
iv. Personal data can be blocked and restricted, rather than erased, in such instances where the law prohibits erasure as it would impair the legitimate interests of the data subject or entities that act as information providers (e.g., by collection, storing, or distributing health records).

v. Where erasure is not possible without disproportionate effort due to the specific type of storage, overwriting, or anonymization, then other methods (s) of removal of the personal data from live systems can be used.

vi. Erasure or destruction of any personal data shall be handled with the utmost care, in accordance with applicable laws, regulations, policies, standards, and guidelines, policies relating to information security, and guidelines relating to data retention and archival, as may be notified from time to time. Where data processors are erasing or destroying any personal data on behalf of the data controller, such erasure or destruction shall be in accordance with their contractual terms of service, and a notification of the erasure or destruction shall be required.

vii. Data erasure should be documented and must include information about the reason for data erasure, how it was erased/destroyed, when, and by whom.

e. Restriction or objection to disclosure: Subject to applicable law, data subjects can restrict or object to the disclosure of their personal data by the data controller.

f. Automated individual decision-making: Data subjects shall have the right to be informed in case a decision to be based solely on automated processing, including profiling, would produce legal effects concerning them or that similarly significantly affect them.

g. Data subjects shall have the right not to be subject to a decision based solely on automated processing, including profiling, that produces legal effects concerning them or that similarly significantly affects them.

h. Section 9.1 shall not apply if the decision:

i. Is necessary for the entering into, or performance of, a contract between the data subject and data controller;

ii. Is authorised by a law to which the data controller is subject, and which also lays down suitable measures to safeguard the data subject’s rights and freedoms and legitimate interests; or

iii. Is based on the data subject’s explicit consent (Section 8.1 (a)).

9.2 GENERAL CONDITIONS FOR SUBMITTING REQUESTS

a. All requests under Section 9.1 above shall be made by the data subject or a legally acceptable representative in writing, either electronically or physically on paper, to the designated officer of the data controller (referred to as a DPO),

b. The request shall be made with the necessary information in regard to the identity of the data subject, and the data controller shall acknowledge receipt of such request. All requests shall be addressed by the data controller within five (5) working days and in compliance with applicable laws, regulations, and these Guidelines.

c. In the event that any request for correction, completion, update, or erasure of any personal data is accepted by the data controller, such a data controller shall take the necessary steps to notify all relevant entities or individuals to whom such personal data may have been disclosed regarding the relevant correction, completion, update, or erasure, particularly where such action may have an impact on the rights and interests of the data subject or decisions made regarding them. The data controller shall also notify the data subject once the relevant personal data has been corrected, completed, updated, or erased within five (5) working days after the action of the request.

d. In the event that any request for correction, completion, update, or erasure of personal data is rejected, the data controller shall provide to the data subject the reasons, in writing, for such refusal within five (5) working days. If the data subject is not satisfied with such reasons, they may require that the data controller take reasonable steps to indicate, alongside the relevant personal data, that the same is disputed by the data subject.

e. In the event of the death of the data subject, the nominee of the data subject may have access to the personal data of the data subject only if such access by such person was specifically consented to by the data subject. If the deceased data subject did not consent, access shall be denied to those who request the personal data of the deceased data subject.

f. The data controller shall not impose any restrictions on the method or channel of raising requests by data subjects under Section 9.1 above.

g. The data controller shall maintain records of all requests received under Section 9.1 above, irrespective of their fulfilment status.

h. Data corrections and modifications should be subject to strict oversight. A request should be lawful and valid. If the modification is clinical, it should be reviewed by a clinician to

ensure the correction being requested is reasonable and appropriate. If the correction is nonclinical (e.g., demographic information), there shall be a policy or process for permitting the correction without the oversight requirements. The correction should also be documented as to why, how, when, and by whom the data was corrected.

10. CONSENT FRAMEWORK

Data controllers can collect personal data, which shall be limited as specified in this section of the Guidelines.

Consent principles: The consent framework under these Guidelines incorporates the following principles in relation to the processing of personal data by data controllers:

a. Data subjects should own their personal data based on the data subjects' rights and determine how their data can be shared and used.
b. Specifically, in the case of electronic consent, data controllers should make use of appropriate technological means to prevent security breaches and to guarantee the integrity of access permissions given by data subjects. Such technological means must conform with the Uganda Data Protection and Privacy Act 2019 and other applicable international standards, as may be notified for the implementation of technical and organizational security measures from time to time.

10.1 CONSENT FOR DATA COLLECTION AND PROCESSING

a. Data controllers can collect or process personal data only with the consent of the data subject or other valid justifications for processing data, except when data sharing does not require the consent of the data subject, as referenced in **Data Protection and Privacy Act 2019 Part III Section 7B I-III**. It is the responsibility of the data controller to ensure that the consent given by the data subject is valid. The consent of the data subject shall be considered valid only if the data controller is able to demonstrate that the data subject consented to the processing of their personal data, and the consent is:
    i. Free from coercion, with regard to whether it complies with the standards set out under the Data Protection and Privacy Act 2019;

ii. Informed, with regard to whether the data subject has been provided with the necessary information by way of notice, as set out in Section 8 (a, b) of these Guidelines;

iii. Specific, where the data subject can give consent for the processing of their personal data for a particular purpose;

iv. Clearly given; a specific, informed and unambiguous indication of the data subject's wish which he or she, by a statement or by clear affirmative action, signifies agreement to the collection or processing of personal data relating to him or her; and

v. Capable of being withdrawn at any time, and the ease of such withdrawal is comparable to the ease with which consent may be given.

b. The purposes for the collection or processing of personal data shall be limited to those that may be specified by the PDPO and such purposes shall be related to the health of an individual or maybe such other incidental purposes that a data subject can reasonably expect for the purpose, context, and circumstances in which the personal data was collected or processed.

c. In addition to the conditions mentioned in Section 8.1 (a) of these Guidelines, the consent of a data subject with respect to the collection or processing of any personal or sensitive personal data shall be obtained only after informing the data subject of the purpose of the data processing and any processing that is likely to cause significant harm to the data subject.

10.2 PRIVACY NOTICE FOR THE COLLECTION OR PROCESSING OF PERSONAL DATA

a. All data controllers must give a clear and conspicuous Privacy Notice (Appendix II) to data subjects:

i. Prior to the collection of personal data from the data subject;

ii. At the time the data controller changes its privacy policies or procedures; and

iii. Prior to the collection or further processing of personal data of the data subject for any new or previously unidentified purpose.

b. Any entity collecting health data shall ensure that a Privacy Notice is displayed within their premises, particularly at service points.

c. It is clarified that for the purpose of Section 8.1,(a) above, all data controllers must obtain fresh consent from data subjects in accordance with the consent framework of these Guidelines.

d. The privacy notice shall contain the following information:

    i. The extent to which the personal data is to be processed, such as
        - Data controller sharing data with data processor for joint purposes,
        - Data controller sharing data with data processor for use by the data processor, and
        - Data controller using data for its own purposes;

    ii. The nature and categories of personal data being collected by the data controller;

    iii. The methods or mechanisms by which the personal data is collected by the data controller;

    iv. The identity and contact details of the data controller collecting the personal data;

    v. The right of the data subject to withdraw their consent, and the procedure for such withdrawal;

    vi. The individuals or entities along with their contact details, including other data controllers or data processors, with whom personal data may be shared, if applicable;

    vii. The period of time for which the personal data shall be retained, or where the period of retention is not known, then the criteria for determining such period;

    viii. The existence of and the procedure for the exercise of rights of the data subject as referred to in Section 7 of these Guidelines; and

    ix. The mechanism by which a data subject may contact the data controller in relation to complaints, inquiries, and clarifications regarding the policies, practices, and procedures employed in the collection, storage, transmission, or any other aspect of processing of personal data.

e. The privacy notice shall be clear, concise, and easily comprehensible to a reasonable person and shall be available in as many languages in which the services of the data controller are intended to be provided.

10.2.1 METHOD OF OBTAINING CONSENT

a. The consent of the data subject, as referred to in Sections 10.0 and 9.0 of these Guidelines, for collection or further processing of personal data, may be obtained electronically or physically on paper, either directly from the data subject or through a consent manager, as the case may be. Where the consent is received physically on paper, then such consent may be converted to electronic format by the consent manager or the data controller.

b. Where consent is obtained through a consent manager as set out above, then such consent manager shall:

  i. Not access, process, or store, in any manner whatsoever, the personal data shared with any data controller pursuant to any consent obtained through such consent manager;

  ii. Maintain a record of all consents shared and revoked, as the case may be, and maintain a log of consents/consent transactions in a manner that enables the audit and review of any use of personal data.

  iii. Where the data subject has revoked their consent, it shall be the duty of the consent manager to notify the data controller of such revocation, as applicable.

c. It is clarified that electronic consent is the digital equivalent of a physical letter of permission given by the data subject which, when presented, allows the consent manager or data controller to collect the personal data or further process the personal data that has already been collected from the data subject for a particular purpose, as the case may be

d. Insofar as further processing of personal data pursuant to Section 10.1 (i), (ii), and (iv) above is concerned, if such processing is done through electronic consent, then a consent artefact shall be generated to initiate the sharing of personal data. If the data subject provides consent for data access and sharing that takes place between a data controller and data processor, the consent artefact shall then be shared with the data controller and data processor through the consent manager.

e. Subject to the provisions of this Guideline and the Data Protection and Privacy Act 2019, guidelines and technical specifications may be set out by the PDPO in relation to consent

obtained by data controllers for the collection and further processing of personal data of data subjects.

10.3 PROCESSING PERSONAL DATA PERTAINING TO A CHILD

a. The data controller(s) should obtain the consent of the parent or guardian of a child prior to processing the child's personal data.

b. A valid proof of relationship and proof of identity of the parent or guardian must be submitted to the data controller in order to verify the consent of the parent or guardian for processing the personal data of the child as set out in Section 10.3, (a) above.

c. Under the following situations, the child shall provide assent, and the parent shall not be the minor's personal representative under these Guidelines. These situations are:

    i. When the minor is the one who assents to care and the consent of the parent is not required under the Data Protection and Privacy Act 2019 or other applicable laws;

    ii. When the minor obtains care at the direction of a court or a person appointed by the court; or

    iii. when and to the extent that, the parent agrees that the minor and the health care provider may have a confidential relationship. For example, a child can restrict a parent's access to their sexual health data according to the country law determining the age of medical consent for the child.

d. Even under the above exceptional situations (Section 10.3, (c), the parent may have access to the medical records of the minor when the Data Protection and Privacy Act 2019 or other applicable law requires or permits such parental access. Parental access would be denied when the Data Protection and Privacy Act 2019 or other law prohibits such access. If the Data Protection and Privacy Act 2019 or other applicable law is silent on a parent's right of access in these cases, the licensed healthcare provider may exercise their professional judgement to the extent allowed by law to grant or deny parental access to the minor's medical information. Finally, a provider may choose not to treat a parent as a personal representative when the provider reasonably believes, in their professional judgment, that the child has been or may be subjected to domestic violence, abuse, or neglect, or that treating the parent as the child's personal representative could endanger the child.

e. Data controller(s) shall not process the personal data of a child in a manner that is likely to harm the child.
f. Data controller(s) should ensure that the processing of the personal data of a child takes place only in such a manner that is in the best interests of the child.

10.4 PROCESSING PERSONAL DATA OF SUBJECTS WHO ARE SERIOUSLY ILL OR MENTALLY INCAPACITATED

Processing personal data of data subjects who are seriously ill or mentally incapacitated or in response to a life-threatening or severely health-threatening medical emergency:

a. When it is necessary to collect data from a seriously ill or mentally incapacitated subject, or in response to a life-threatening or severely health-threatening medical emergency a nominee shall represent the data subject until the subject becomes able to give consent.
b. At the time a data subject opts to participate in the data collection and processing, such data subject should name a nominee;
c. The nominee referred to in Section 10.4, (a) above shall be authorized to give valid consent on behalf of the data subject in the event the data subject becomes seriously ill or mentally incapacitated, or where the data subject is facing a life-threatening or severely health-threatening medical emergency and is unable to give valid consent.
d. In the event that the data subject has not named a nominee under Section 10.4 (a) above, then any adult family member of the data subject can give valid consent on behalf of the data subject.
e. In the event that there is no family member or nominee, an authorized person may consent on behalf of the data subject.
f. In the event that the data subject has named a nominee under Section 10.4 (a) but the nominee is unable to give consent for any reason, then the health care professional can use their best judgment until such nominee, family member, or data subject is able to give consent.
g. Consent can be given by a family member of the data subject, as set out in Section 10.4 (d) above, only where there is proof of relationship with the data subject.

11. DATA SECURITY, IMPACT ASSESSMENT, AND AUDIT

11.1 ADEQUATE SECURITY PRACTICES AND PROCEDURES

i. Data controllers shall implement such security practices and standards and have a comprehensive documented information security program and information security policy(ies)/guidelines that contain managerial, technical, operational, and physical security control measures that are commensurate with the data/information assets being protected by them (Appendix III) both in paper and electronic format. These security practices, standards, and policies shall be reviewed periodically.

ii. In the event of a data/information security breach, data controllers shall be required to demonstrate, as and when called upon to do so, to the PDPO and the Ministry of Health, that they have implemented security control measures as per their documented information security program and information security policies.

iii. Data controllers shall implement necessary security safeguards, including the use of de-identification and encryption methods, to protect the integrity of the personal data collected and to prevent the misuse of, unauthorized access to, modification of, disclosure of, or destruction of personal data. The necessary safeguards shall factor in the nature, scope, and purpose of processing the personal data, the risks associated with such processing, and the likelihood and severity of harm that may result from such processing. Data controllers shall undertake a review of their security safeguards periodically and take corrective measures accordingly.

iv. Data controllers shall implement the Uganda Digital Health Enterprise Architecture, Standards and Knowledge Guidelines as well as any other standards as may apply to them. Several relevant resources (e.g., International Organization for Standardization, US National Institute of Standards and Technology, and others) are included in these Guideline’s bibliography that might be applicable for adequately securing, risk assessing, and implementing the data controller's infrastructure. Appendix IV shows how to assess and improve cybersecurity maturity.

v. As permitted under the provisions Data Protection and Privacy Act 2019, the standards in relation to security practices and procedures mentioned above may be certified or audited on a regular basis through an independent auditor duly approved by the Personal

Data Protection Office. This audit shall be carried out by the auditor at least once a year or when the data controller undertakes a significant upgrade of its processes, computer resources, or systems.

vi. If and when an entity is implementing or involved in the national health care system and is acting as a data controller in this regard, the DPO shall undertake a periodic review of the entity's security safeguards and take appropriate measures to update such safeguards, if required.

vii. Any person having access to health data shall be subjected to mandatory training once a year or access to head data systems shall be automatically revoked.

11.2 DATA MANAGEMENT BY DATA PROCESSORS

i. The data controller should conduct appropriate due diligence covering data privacy and security before engaging with any data processor.

ii. The data controller may not engage, appoint, use, or involve a data processor to process personal data on its behalf without a contract entered into by the data controller and such data processor.

iii. The data controller shall require its data processors to execute confidentiality agreements and nondisclosure agreements covering data protection and privacy responsibilities. Such agreements shall be reviewed, updated, and renewed periodically. The data controller may require that the confidentiality requirements under such agreements continue for a specified period even after the contractual period ends.

iv. Subject to the Data Protection and Privacy Act 2019, the agreements referred to in Section 11.2 (iii) shall align with these Guidelines. These agreements shall ensure that data processors adhere to the same level of data protection that is adhered to by the data controller.

v. The data controller shall require its data processors to limit their access only to the personal data necessary for the fulfilment of their employment/contractual duties and for public health, research, and statistical purposes based on a "need-to-know" principle, as the case may be.

vi. The data processor and any employee of the data controller shall only process personal data in accordance with the instructions of the data controller and treat it confidentially.

vii. The data processor may not engage, appoint, use, or involve another data processor in processing on its behalf, except with the authorization of the data controller and unless permitted in the contract referred to in Section 9.2 (ii) above.

viii. The data controller shall ensure that training and awareness materials around data protection and privacy are developed for its employees and data processors. Role-based training for individuals or teams that consider the nature of the processing and their roles shall be developed. Data privacy training and awareness programs shall be conducted on a periodic basis (at a minimum, annually) for all employees and data processors. Attendance records for such training shall be maintained for documentation and audit purposes.

11.3 DATA PROTECTION IMPACT ASSESSMENT

i. The data controller shall carry out a data protection impact assessment before it undertakes any processing involving new technologies or any other processing that carries a risk of significant harm to data subjects.

ii. The data protection impact assessment shall contain, among other things, a detailed description of the proposed processing operation, the purpose(s) of the processing, the nature of personal data being processed, an assessment of the potential harm, and measures for managing, minimizing, mitigating, or removing such risk of harm.

iii. The data controller shall consult the supervisory authority prior to processing where a data protection impact assessment under Section 11.3 (i–ii) indicates that the processing would result in a high risk in the absence of measures taken by the data controller to mitigate the risk.

11.4 RECORDS MAINTAINANCE

i. Data controllers shall maintain accurate and up-to-date records to document the important operations in the data life cycle, including collection, transfer, and erasure of personal data. These records shall cover the following:

   a. Details of the ecosystem partners;
   b. Purposes of the processing;
   c. Description of the categories of data subjects;
   d. Description of the categories of personal and sensitive personal data; and

e. Categories of recipients to whom the personal data is disclosed/transferred, including to data processors.

In addition to the records referred to in Section 11.4, (i) and elsewhere in these Guidelines, data controllers shall also maintain accurate and up-to-date records of the periodic review of security safeguards conducted under Section 11.1, data protection impact assessments conducted under Section 11.3, and requests received under Section 9.0 of these Guidelines.

Audit

i. Data controllers should maintain a strict audit trail of all processing of any personal data, at all times. This is the record of how personal data is processed by the data controller and should be maintained in a manner that enables the audit and review of any use of personal data.

ii. Data controllers should ensure that periodic audits of their data processors are conducted by third parties in accordance with relevant standards and certifications, as may be specified by the PDPO, to verify that such data processors process all personal data appropriately in compliance with all privacy notices, contracts/confidentiality agreements, these Guidelines, and any policy relating to information security as may be notified from time to time.

iii. If the data controller decides to update any personal data in accordance with Section 9.0 of these Guidelines, then the original personal data and an audit trail of the change shall be made available to the data subject. However, the updated personal data with a new version number shall be considered active.

12. DATA SHARING OBLIGATIONS

The data sharing obligations described in these Guidelines are an extension of the Uganda Health Data Access, Sharing and Use Guidelines and a Data Processing Agreement (Appendix V).

12.1 DATA CONTROLLERS SHARING PERSONAL DATA

i. Any personal data processed by a data controller may be shared with a data processor in response to a request made by such data processor for personal data pertaining to the data subject, only where consent of the data subject is obtained in accordance with Section 10. Consent framework, (a,b) of these Guidelines.

ii. Where a data processor makes a request to access any personal data under Section 12.1 (i), above, the data controller shall verify the constituents shared with it, including whether such consent has been revoked by the data subject. Where the consent is valid, it shall share such data with the data processor strictly in accordance with Section 10, (a,b) of these Guidelines.

iii. A data controller shall maintain a record of all consent obtained under these Guidelines, pursuant to which personal data has been shared by such data controller under these Guidelines in a manner that enables the audit and review of such data sharing.

iv. Where the data subject has provided their consent for the sharing of their personal data under these Guidelines, it shall not be used, disclosed, or shared by the data controller or any data processor in any other manner, or for any other purpose, except as provided in Section 10, (a,b) of these Guidelines.

v. A data controller should comply with permissible data-sharing scenarios outlined in the Uganda Health Data Access, Sharing and Use Guidelines, which does require data subject consent and complies with the legal justifications for data sharing outlined in Section 12.4 of these Guidelines. For example, data sharing between health care providers for treatment purposes should not require consent of data subjects and complies with Section 12.4 (iv) (vital interests of the data subject)

12.2 DATA CONTROLLERS SHARING DE-IDENTIFIED OR ANONYMIZED DATA

i. Data controllers may make anonymized or de-identified data in an aggregated form available as per the procedure set out in Section 12.2 (ii) to facilitate health and clinical

research, academic research, archiving, statistical analysis, policy formulation, development and promotion of diagnostic solutions, and such other purposes as may be specified by the PDPO.

ii. The PDPO shall set out a procedure through which any entity seeking access to anonymized or de-identified data under these Guidelines shall be required to provide relevant information such as its name, purpose of use, and nodal person of contact. Subject to approval being granted under this procedure, the anonymized or de-identified data under these Guidelines shall be made available to such entity on such terms as may be stipulated herein.

iii. Any entity that is provided access to de-identified or anonymized data shall not, knowingly or unknowingly, take any action that has the effect of re-identifying any data subject or of causing such data to no longer be anonymized.

iv. The data controller that is undertaking to anonymize or de-identify data under these Guidelines shall be responsible for ensuring compliance with the procedure for such anonymization or de-identification as set out in these Guidelines under Section 12.2 and any noncompliance shall be dealt with as per Section 13.4.

v. The de-identification or anonymization of data by a data controller shall be done in accordance with technical processes and anonymization protocols that may be specified by the PDPO or the organisation ICT policy. The data controller should protect an anonymized database in the same way that it protects a database with personally identifiable information.

vi. The technical processes and anonymization protocols referred to in Section 12.2, (v) shall be periodically reviewed by the PDPO, and such review shall have regard to the nature and sensitivity of the data being processed, the risks of re-identification of data subjects, and the robustness of the anonymization protocols.

vii. Any attempt to re-identify individuals or to generate information (e.g., facial images or comparable representations) that could allow the identities of data subjects to be readily ascertained should be strictly prohibited (and subject to penalty) except where expressly authorized by law. Reasonable steps should be taken to prevent the identity of data subjects being leaked or determined through indirect means such as metadata, website URLs, and email subject lines .

12.3 RESTRICTIONS ON SHARING, CIRCULATING, OR PUBLISHING OF PERSONAL DATA

a. Any personal data of the data subject shall not be published, displayed, or posted publicly by any person or entity unless permitted by any other law.

b. A database or record of any data that has been processed under these Guidelines shall not be made public, unless such database or record is in an anonymized/de-identified and aggregated form and is processed in accordance with the terms specified in Section 12.2, (i) of these Guidelines.

12.4 LEGAL GROUNDS FOR PROCESSING PERSONAL DATA

The processing of personal data (Section7.0) is permitted under one or more of the following legal grounds:

i. Explicit consent (Section 10, (a,b)): the explicit consent of a patient must be freely given and comes with an obligation to not exceed the intent of the consent, so should be considered carefully.

ii. Performing a contract: a data controller may be able to process data for performing a contract (e.g., a company is allowed to process personal data for salary processing and a care provider may be able to exchange data with payers for fulfilling insurance contracts).

iii. Legal obligations: a data controller may be required to process the data to comply with legal obligations (e.g., reporting to authorities in a legal obligation).

iv. Vital interests of the data subject: the vital interests of the data subject may present a lawful justification for data processing (e.g., in case of a public health emergency or medical emergency scenario).

v. Public interest: public interest in the area of public health (e.g., protecting against serious infectious diseases such as HIV/AIDS and tuberculosis or ensuring high standards of quality and safety for health care, medicinal products, and medical devices) on the basis of Public Health Act 1935, which provides for suitable and specific measures to safeguard the rights and freedoms of the data subject. Public interest shall also include use of personal data for public health archiving, scientific or historical research, or statistical purposes. Processing of data for public health interest shall not include

processing of personal data for other purposes by third parties, such as employers or insurance and banking companies.

vi. Legitimate interests, where these interests do not conflict with data subject rights or other protections: legitimate interests can be claimed but they must be justified (i.e., the interests must be confirmed as legitimate); for example, a pharmacy claiming that they have a legitimate interest to sell patient diagnosis data to maintain profit levels may not be claimed as a legitimate interest.

vii. If a processing activity or purpose does not fit into any of the legal justifications described above, the data processing may be considered unlawful.

13. GRIEVANCE REDRESSAL, INCIDENT MANAGEMENT, AND GUIDELINES COMPLIANCE

13.1 GRIEVANCE REDRESSAL

i. Data subject(s) with inquiries and questions about the processing of their personal data may approach a designated officer of the data controller (i.e., data protection officer) in writing, either through email or any other electronic means or physically on paper, as may be specified. The details of the DPO shall be provided on the official notice board / website/ approved platform of the data controller along with the format and process for filing inquiries/questions. Where feasible, the data controller may designate the DPO as the grievance redressal officer mentioned in Section 13.1, (ii) below.

ii. A complaint can be made by the data subject regarding any contravention of these Guidelines that has caused or is likely to cause harm to such data subject. The data controller shall have in place procedures and effective mechanisms to redress the grievances of data subjects efficiently and in a speedy manner. For this, the data controller shall designate a grievance redressal officer and publish their name and contact details on its website or on the official notice board. The grievance redressal officer shall redress the grievances of the data subject expeditiously but within one month from the date of receipt of the grievance. Acknowledgment of receipt of a grievance shall be provided to the data subject within 7 working days.

iii. In the event that a complaint is not resolved by the grievance redressal officer of the data controller as referred to under Section 13.1 (ii) above, the data subject shall be notified

and the matter may be referred to the PDPO in writing, or through email or any other electronic means provided under the grievance portal of PDPO website (https://www.pdpo.go.ug/file-complaint).

13.2 PERSONAL DATA BREACH AND INCIDENT MANAGEMENT

To address data security breaches, the following shall be undertaken:

i. The data controller shall formulate and implement a personal data breach management procedure, which shall be publicly displayed. The data controller shall also ensure that any instance of noncompliance with the provisions of these Guidelines, or any instance of unauthorized or accidental disclosure, acquisition, sharing, use, alteration, destruction of, or loss of access to personal data that compromises the confidentiality, integrity, or availability of personal data to a data subject is promptly notified to relevant entities as required by the Data Protection and Privacy Act 2019.

ii. The data controller shall also notify any incidents referred to in Section 13.2, (i), and the actions taken pursuant to such incidents, to the PDPO in a timely manner as mentioned in Section 8.3,

iii. Without prejudice to the foregoing, in the event of a personal data breach, the person(s) responsible for such breach shall be liable in accordance with the provisions of applicable laws.

13.3 COMPLIANCE AND GUIDELINES GOVERNANCE

i. The DPO of the PDPO together with the DPO of the Ministry of Health shall ensure adherence to these Guidelines and shall be responsible for compliance with all Uganda applicable laws in force.

ii. All individuals and entities who are covered by these Guidelines must comply with its requirements and, where requested, demonstrate such compliance.

iii. The compliance checklists developed (Appendix I and III) shall be used for the purpose of monitoring the compliance of stakeholders to these guidelines.

iv. These Guidelines may be revised from time to time. A copy of these Guidelines together with any significant revisions shall be made publicly available through the Ministry of Health official communication channels like e-Library or the Knowledge Management Portal.

13.4 NON-COMPLIANCE WITH THESE GUIDELINES

Where any person to whom these Guidelines is applicable is found to be in violation of any of its provisions, such a person may not be permitted to participate in the national healthcare enterprise and further action shall be taken by the PDPO.

13.5 LIABILITY, PENALTIES AND REMEDIES

Shall follow the Data Protection and Privacy Act 2019 and any other relevant laws concerning penalties (e.g., administrative, financial, criminal) liabilities and judicial remedies.

13.6 SALE OF HEALTH DATA

The sale or offer to sell health data by a person or an entity shall be prohibited. **Section 37** of the Data Protection and Privacy Act 2019 shall be followed in case of non-compliance.

14.0 DISSEMINATION AND ADOPTION OF THE GUIDELINES

The Health Data Protection, Privacy and Confidentiality Guidelines shall be disseminated for adoption.

The dissemination and adoption of these guidelines shall happen at national, sub-national and community levels as guided by the MoH. Some of these methods are;

a. Presentation of the guidelines to stakeholders at all levels.
b. Posting of the guidelines on the Ministry of Health websites, WhatsApp groups, Radio etc for access by the stakeholders.
c. Organising quarterly workshops to sensitise stakeholders

APPENDIX I: COMPLIANCE CHECKLIST FOR DATA CONTROLLERS TO ALIGN WITH THE GUIDELINES

1. Lawful basis and transparency

   - Conduct an information audit to determine what information you process and who has access to it.
   - Have a legal justification for your data processing activities.
   - Provide clear information about your data processing and legal justification in your privacy guidelines.

2. Data security

   - Take data protection into account at all times, from the moment you begin developing a product to each time you process data.
   - Encrypt, pseudonymize or anonymize personal data wherever possible.
   - Create internal security guidelines for your team members and build awareness about data protection.
   - Know when to conduct a data protection impact assessment and have a process in place to carry it out.
   - Have a process in place to notify the authorities and your data subjects in the event of a data breach.

3. Accountability and governance

   - Designate someone responsible for ensuring guidelines compliance across your organization.
   - Sign a data processing agreement between your organization and any third parties that process personal data on your behalf.
   - Appoint a data protection officer (if necessary).

4. Privacy Rights

   Ensure that it is easy for customers to:

   - Request and receive all the information you have about them.
   - Correct or update inaccurate or incomplete information.
   - Request to have their personal data deleted.
   - Ask you to stop processing their data.

- Receive a copy of their personal data in a format that can be easily transferred to another organisation.
- Object to you processing their data.
- Protect their rights if you make decisions about people based on automated processes.

APPENDIX II: PRIVACY NOTICE TEMPLATE

Sample: Department/Organization Privacy Notice

[Insert name of department/organization] is part of the [insert name of organization/ministry],]. This privacy notice shall explain how "[Insert name of department/organization] uses the personal data it collects from you when you use "[Insert name of department/organization] system.

1. **What data do** Insert name of department/organization] **collect?**

   "[Insert name of department/organization] collects the following data:

   - Personal identifiers (e.g., name, email address, phone number).
   - [Add any other data "[Insert name of department/organization] collects].

2. **How do** "[Insert name of department/organization] **collect your data?**

   You directly provide "[Insert name of department/organization] with most of the data Insert name of department/organization] collect. "[Insert name of department/organization] collect data and process data when you:

   - Register online or place an order for any of "[Insert name of department/organization] products or services.
   - Voluntarily complete a customer survey or provide feedback on any of "[Insert name of department/organization] message boards or via email.
   - Use or view "[Insert name of department/organization] website via your browser's cookies.
   - [Add any other data "(Insert name of department/organization) collects].
   - Insert name of department/organization] may also receive your data indirectly from the following sources:
   - [Add any indirect source of data your Insert name of department/organization has].

3. **How will** Insert name of department/organization] **use your data?**

   Insert name of department/organization] collects your data so that it can:

   - Process your order and manage your account.
   - Email you with special offers on other products and services it thinks you might like.
   - [Add how else Insert name of department/organization] uses data].

   If you agree, Insert name of department/organization] will share your data with the following partner Department(s)/Organization(s) so that they may offer you their products and services:

- [List organizations that will receive data].

When Insert name of department/organization] processes your order, it may send your data to, and also use the resulting information from, National Digital Health Authority reference agencies to prevent fraudulent activities.

4. **How do** Insert name of department/organization] **store your data?**

Insert name of department/organization] securely stores your data at [insert the location and describe security precautions taken].

Insert name of department/organization] will keep your [insert type of data] for [insert time period]. Once this time period has expired, Insert name of department/organization] will delete your data by [insert how you delete user data].

5. **What are your data protection rights?**

Insert name of department/organization] would like to make sure you are fully aware of all of your data protection rights. Every user is entitled to the following:

- Confirmation and access: You have the right to request Insert name of department/organization] for copies of your personal data. Insert name of department/organization] may charge you a small fee for this service.
- Correction and erasure: You have the right to request that Insert name of department/organization] correct any information you believe is inaccurate. You also have the right to request Insert name of department/organization]to complete the information you believe is incomplete. You have the right to request that Insert name of department/organization] erase your personal data, under certain conditions.
- Restriction or objection to disclosure: You have the right to request that Insert name of department/organization] restricts the processing of your personal data, under certain conditions. You have the right to object to Insert name of department/organization] processing of your personal data, under certain conditions.
- Automated individual decision-making: You have the right to request Insert name of department/organization] not to subject you to a decision based solely on automated processing, including profiling, which produces legal effects concerning you or similarly significantly affects you unless you provide explicit consent by accepting our privacy notice.

If you make a request, Insert name of department/organization] have one month to respond to you. If you would like to exercise any of these rights, please contact us by any of the following means:

Email us at:

Call us at:

Or write to us at:

6. **What are cookies?**

Cookies are text files placed on your computer to collect standard internet login information and visitor behavior information. When you visit Insert name of department/organization] website, Insert name of department/organization] may collect information from you automatically through cookies or similar technology. For further information, visit allaboutcookies.org.

7. **How do** Insert name of department/organization] **use cookies?**

Insert name of department/organization] uses cookies in a range of ways to improve your experience on our website, including:

- Keeping you signed in.
- Understanding how you use [Insert name of department/organization] website to improve user experience.
- [Add any uses your [Insert name of department/organization] has for cookies].

8. **What types of cookies do [**Insert name of department/organization] **use?**

There are a number of different types of cookies; however, Insert name of department/organization] website uses:

- Functionality cookies [Insert name of department/organization] uses these cookies so that Insert name of department/organization] recognize you on its website and remember your previously selected preferences. These could include what language you prefer and location you are in. A mix of first-party and third-party cookies are used. The first-party cookies are created by the domain while the third-party cookies are created by domains other than the [Insert name of department/organization] is visiting at the time, and are mainly used for tracking and online-advertising purposes
- Advertising cookies – **[**Insert name of department/organization] uses these cookies to collect information about your visit to **[**Insert name of department/organization] website, the content you viewed, the links you followed, and information about your browser, device, and internet protocol address. The **[**Insert name of department/organization] sometimes shares some limited aspects of this data with third parties for advertising purposes. The **[**Insert name of department/organization] may also share data collected through cookies with **[**Insert name of department/organization] advertising partners. This means that when you visit another website, you may be shown advertising based on your browsing patterns on **[**Insert name of department/organization] website.

- [Add any other types of cookies your company uses].

9. **How to manage cookies**

You can set your browser not to accept cookies, and the website "allaboutcookies.org" tells you how to remove cookies from your browser. However, in a few cases, some of **[**Insert name of department/organization] website features may not function as a result.

10. **Privacy policies of other websites**

[Insert name of department/organization] website contains links to other websites. **[**Insert name of department/organization] privacy notice applies only to our website, so if you click on a link to another website, you should read their privacy notice.

11. **Changes to our privacy policy**

[Insert name of department/organization] keeps its privacy notice under regular review and places any updates on this web page. This privacy notice was last updated on [insert date in DD/MM/YYYY format].

12. **How to contact us**

If you have any questions about our **[**Insert name of department/organization]'s privacy notice, the data it holds on you, or you would like to exercise one of your data protection rights, please do not hesitate to contact **[**Insert name of department/organization]:

Email at:

Call at:

Or write to at:

13. **How to contact the appropriate authority**

Should you wish to report a complaint or if you feel that **[**Insert name of department/organization] has not addressed your concern in a satisfactory manner, you may contact the grievance redressal officer via:

Email:

Address:

APPENDIX III: DATA PROTECTION PRIVACY AND CONFIDENTIALITY CHECKLIST

Governance and Policy/Guidelines		Recommendations
Data Protection, Privacy and Confidentiality Policy/Guidelines	Does legislation or a guidance exist that covers the use of personally identifiable information (PII) for different purposes?	
Policy/Guidelines sections	Do the guidelines contain sections to ensure the privacy, confidentiality, and protection of PII? For example:<br>● Clearly defined roles and access levels for all persons with authorized access to PII.<br>● Clearly defined standard procedures or methods that must be followed when accessing PII.<br>● Information on the data life cycle (collection, storage, backup, use, transmission, release, disposal, etc.)<br>● Information on data breaches and breach investigations.<br>● Clearly defined training standards for organizations/entities.	
Policy/Guidelines distribution	Is the guidelines document distributed to stakeholders and relevant organizations? For example:<br>● Ministry to health staff at the national level.<br>● Health staff at regional/district/local facilities.<br>● Patients/data subjects at facilities.<br>● Academic researchers involved with PII data.<br>● Staff from donor organizations.<br>● Multilateral institution staff.	
Policy/Guidelines availability	Are the guidelines for ensuring confidentiality and security of PII readily accessible to all staff members? For example:<br>● Document available in multiple formats (hard copies, electronic).	

	● Document distributed to all staff members.<br>● Document easily accessible to all staff members at the facility.	
Policy/Guidelines development	Are stakeholders involved in the development of the guidelines document and its update process? For example:<br>● Health professionals (medical/nursing practitioners, public health specialists, other health professionals).<br>● Information technology specialists (data entry staff, analysts, managers, programmers).<br>● Patient advocacy groups.<br>● Legal experts.<br>● Human rights advocates and ethicists.<br>● Government officials.<br>● Business representatives.<br>● Support service staff (cleaners, security guards).	
Policy/Guidelines governance structure	Is there a governance structure such as an advisory committee in place to provide oversight for the appropriate collection, use, and dissemination of data, including regular review of the guidelines document and security practices?	
Covered uses of PII	Are there guidelines in the document on the uses of PII? For example:<br>● Individual health care<br>● Public health practice (including monitoring and evaluation)<br>● Human subjects research (with consent)<br>● Exceptional statutory purposes<br>● Other uses of PII	
Information security management	Are there guidelines within the document that require organizations to designate an information security manager? Is there a written description of the information security manager's responsibilities?	
Security breach management	Are there guidelines in the document for identifying and managing a security breach? For example:	

	• Definition of a security breach and its consequences<br>• Organizational structure and delineation of roles, responsibilities, and levels of authority<br>• Requirements for reporting security breaches<br>• Prioritization or severity ratings of security breaches<br>• Reporting and contact forms in the event of a breach	
Risk assessment	Are there guidelines in the document for risk assessments? If so, are these mandated assessments performed regularly?	
Review of security practices	Are security practices reviewed by independent auditors at regular intervals? Are information security and its management reviewed at regular intervals?	
Policy updating - advancements	Are there guidelines on how often software and hardware technologies are reviewed? Is the guidelines document regularly updated for advancements? For example: advancements in database software, web servers, email clients, firewalls, file servers, and backup devices	
Data Collection and Storage		
Interview privacy	Does your organization take measures for protecting patient privacy while collecting information (during the interview with the patient), such as:<br>• Minimizing the exchange of verbal information.<br>• Using a partition or curtain when using open rooms.<br>• Using a separate room with a soundproof barrier.<br>• Using cover sheets on paper documents.<br>• Using a computer screen guard that provides visual privacy?	
Inventory of paper-based	Is there an inventory of paper-based tools currently and previously used by the program?	

	Which paper-based tools should continue to be used as the level of disruption evolves?	
Protect paper-based data from unauthorized access	Are there measures in place to protect PII stored on paper from unauthorized access? For example:<br>● Locked cabinet<br>● Locked room<br>● Locked windows<br>● Bars/grills on doors or windows<br>● Alarm system<br>● Video monitoring<br>● Security guard or other authorized staff control access	
Protect paper-based tools from natural risks	Are there measures in place to protect PII stored on paper from natural risks? For example:<br>● Fire<br>● Water damage<br>● Animal damage (such as mice or termites)	
Inventory of database(s) with PII	Does your organization manage an updated inventory of database(s) and files containing PII, including their backups?	
Inventory electronic equipment with PII	Is there an inventory of electronic equipment (servers, desktop computers, laptop computer, tablets, smartphones, and other mobile devices) containing database(s)/files with PII?	
Protect electronic devices from natural risks	Are there measures in place to protect PII stored on electronic devices from natural risks? For example:<br>● Power failure (backup generator)<br>● Power surge (surge protector)<br>● Water damage (furniture and shelves to raise devices off floor)<br>● Fire (smoke detector, fire extinguisher)<br>● Extreme humidity (dehumidifier)<br>● Particulates—e.g., sand, ash, etc. (window and door screens, air purifier)	
Protect electronic devices from unauthorized access	Does your organization take measures for protecting PII stored on electronic devices from theft or destruction? For example:	

	● Locked cabinet<br>● Locked room<br>● Locked windows<br>● Bars/grills on doors or windows<br>● Alarm system<br>● Video monitoring<br>● Security guard or other authorized staff control access	
Antivirus	Does your organization take measures for protecting electronic devices against viruses? For example:<br>● Installing official antivirus software on all types of devices: servers, desktop computers, laptop computer, and other mobile devices such as smartphones<br>● Mechanism to update the antivirus signature in real time<br>● Mechanism to frequently perform full scan of the device<br>● Automated scan for all new files	
Firewall	Are there measures in place to protect networks against intrusion? For example:<br>● Adequate router configuration<br>● Installation of hardware or software firewall<br>● Anti-malware software<br>● Monitoring computers, servers, and network firewall logs	
Password policy	Is there a strong password policy in place? For example:<br>● All software/database(s) containing PII are password-protected.<br>● Passwords masked when entered into computer applications.<br>● There are constraints on the password (minimum length, combination of lowercase letters, uppercase letters, numbers, and special characters).<br>● Passwords must be changed frequently.<br>● Revocation of privilege as soon as employment is terminated.<br>● All files containing PII are password-protected.	

Anonymization	Are there measures in place to remove personal identifiers from data sets whenever possible (e.g., for analysis, statistics, external reporting)?	
Encryption	Are there procedures in place to ensure PII is always encrypted?	
Backup	Are there measures in place to securely back up data? For example:<br>● Adequate frequency for backups<br>● Backups on a distant, separate site<br>● Encryption of backups (especially with PII)<br>● Regular tests for restoral using backed-up data<br>● Secure storage of backed-up data media	
Multiple network connections	Are there guidelines in place for computers containing PII data that are connected to more than one network? For example:<br>● Use of built-in encryption on these devices<br>● Use of virtual private networks<br>● Remote desktop software<br>● Multiple network interface cards<br>● Network bridge	
Authorization and Access Control		
Data access by staff	Has staff member access to data been defined? For example, access by:<br>● Health professionals (medical practitioners, nursing practitioners, public health specialists, other health professionals)<br>● Access by information technology staff (data clerks, analysts, managers, programmers)<br>● Administrative staff<br>● Professional service providers<br>● Volunteers<br>● Academic or other researchers<br>● Support services (cleaners, security guards)<br>● Bilateral donor staff<br>● Multilateral institution staff	
Levels of access	For roles where access to data has been defined, are levels of access specified for using data for different purposes? For example:	

	• Individual health care use<br>• Public health practice use	
System security controls	Are there requirements in place to ensure system security controls are independently validated?	
Session timeout	Are there procedures in place to "timeout" or lock user sessions after a specified period of inactivity on software applications that contain PII?	
Data Transmission, Sharing, and Release		
Data release	Are there guidelines in place for data release? For example:<br>• Class of use for which data may be released.<br>• Specific data elements by which data may be released.<br>• Entities or organizations to whom data may be released.<br>• Requirements for how recipients will protect the confidentiality of received data.<br>• Specifications for time limitations on the use of released data.<br>• Data quality standards that must be met before data release.<br>• Clearly defined individual(s) who are authorized to release data.<br>• Clear procedures for handling data requests that are not covered under the data release policy.	
Protect paper-based transmission	Are there procedures in place to protect paper-based documents with PII when they leave a site? For example:<br>• The amount and sensitivity of information contained in any piece of correspondence remains minimal.<br>• Documents are put in a sealed envelope.<br>• Mail marked "confidential" is only opened by the addressee.<br>• Documents are carried in a locked suitcase.<br>• Documents are carried only by authorized person(s).	

	● The person in charge of receiving the confidential mail must contact the sender to notify them that the mail was received.	
Protect electronic transmission	Are there procedures in place to protect data (password protection, encryption, acknowledgement of receipt) when information transits between systems/persons through:<br>● Email<br>● Intranet, a local-area network, wide-area network<br>● Internet (via web browser)<br>● File transfer protocol<br>● Tape, optical media<br>● Flash drive/memory stick/memory card<br>● External hard drive?	
Data release conditions	Are there conditions that must be met before releasing PII? For example:<br>● Verification that consent was obtained<br>● Confirmation that data was reviewed for accuracy<br>● Removal of direct patient identifiers from released records<br>● Request reviewed to verify the minimum amount of data needed to satisfy the purpose<br>● Acquisition of formal approval for data release	
Data release procedures - purpose	Are there procedures in place for data release (and/or data sharing) that control access to, and use of, individual-level information, covering the following cases:<br>● Individual health care (including sharing patient records with another facility when the patient is referred or transferred)?<br>● Sharing patient records with the patient directly (including sharing laboratory results with the patient via mail/email/SMS, etc.)<br>● Public health practice, including monitoring and evaluation or case reporting<br>● Human subjects research (with consent)<br>● Exceptional statutory purposes?	

Data release procedures - recipients	Are there procedures in place to protect PII when releasing it? For example:<br>● Asking the recipient to sign a confidentiality agreement.<br>● Asking the recipient for documentation of security training.<br>● Assessment of recipient organization security (review of procedural, electronic, and physical security controls).<br>● Recipient agreement to destroy information after data release purpose has been fulfilled.<br>● Internal steering group review and approval.<br>●	
Data anonymization	Are there procedures in place to explore alternatives to using identifiable data before sharing data, such as using anonymized or coded data?	
Data Disposition		
Data disposition procedures	Are there procedures in place to securely archive PII on paper-based tools when no longer in use?	
Disposition of paper-based information	Are there measures in place to securely dispose of PII stored on paper (e.g., forms, registers) and no longer used, such as:<br>● shredding documents containing PII with a cross-cutting shredder before disposing of them<br>● keeping records of documents destroyed?	
Data disposition procedures	Are there procedures in place to archive PII stored in electronic devices when no longer in use?	
Disposition of electronic-based information	Are there measures in place to securely dispose of PII stored electronically and no longer used, such as:<br>● eliminating emails sent or received containing PII<br>● sanitizing or destroying hard drives or mobile devices (including USB keys) that	

	contain PII before the computer/mobile device is reassigned to non-program staff members, sent off-site for repair, sold, or sent to garbage<br>● keeping records of documents destroyed<br>● asking partnering organizations to do the same?	
Data archival	Are there guidelines included on archiving data? For example:<br>● how often data must be archived<br>● approved storage locations of archived data<br>● approved media for archiving data<br>● roles responsible for archiving data	
Data migration	Are there procedures in place that require that data be periodically migrated to newer technologies as they become available?	
Data Disposal	Are there requirements for data retirement and record destruction? For example:<br>● data retirement schedule<br>● certification/verification of data disposal/destruction	

APPENDIX IV: ASSESSING AND IMPROVING CYBERSECURITY MATURITY

This appendix was adapted from the United States Department of Defense's Cybersecurity Maturity Model Certification, which measures cybersecurity maturity with up to three levels and aligns a set of processes and practices with the type and sensitivity of information to be protected and the associated range of threats. The model consists of maturity processes and cybersecurity best practices from multiple cybersecurity standards, frameworks, and other references. Please note that 110 CMMC2 controls originate from NIST SP 800-171 r2.

Recommended practices for each of the maturity practice areas:

1. ACCESS CONTROL (AC)

Level 1

AC.L1-3.1.1 – **Authorized Access Control** – Limit information system access to authorized users, processes acting on behalf of authorized users, or devices (including other information systems).

AC.L1-3.1.2 – **Transaction & Function Control** – Limit information system access to the types of transactions and functions that authorized users are permitted to execute.

AC.L1-3.1.20 – **External Connections** – Verify and control/limit connections to and use of external information systems.

AC.L1-3.1.22 – **Control Public Information** – Control information posted or processed on publicly accessible information systems.

Level 2

AC.L2-3.1.3 – **Control CUI Flow** – Control the flow of CUI in accordance with approved authorizations.

AC.L2-3.1.4 – **Separation of Duties** – Separate the duties of individuals to reduce the risk of malevolent activity without collusion.

AC.L2-3.1.5 – **Least Privilege** – Employ the principle of least privilege, including for specific security functions and privileged accounts.

AC.L2-3.1.6 – **Non-Privileged Account Use** – Use non-privileged accounts or roles when accessing non-security functions.

AC.L2-3.1.7 – **Privileged Functions** – Prevent non-privileged users from executing privileged functions and capture the execution of such functions in audit logs.

AC.L2-3.1.8 – **Unsuccessful Logon Attempts** – Limit unsuccessful logon attempts.

AC.L2-3.1.9 – **Privacy & Security Notices** – Provide privacy and security notices consistent with applicable Controlled Unclassified Information (CUI) rules.

AC.L2-3.1.10 – **Session Lock** – Use session lock with pattern-hiding displays to prevent access and viewing of data after a period of inactivity.

AC.L2-3.1.11 – **Session Termination** – Terminate (automatically) user sessions after a defined condition.

AC.L2-3.1.12 – **Control Remote Access** – Monitor and control remote access sessions.

AC.L2-3.1.13 – **Remote Access Confidentiality** – Employ cryptographic mechanisms to protect the confidentiality of remote access sessions.

AC.L2-3.1.14 – **Remote Access Routing** – Route remote access via managed access control points.

AC.L2-3.1.15 – **Privileged Remote Access** – Authorize remote execution of privileged commands and remote access to security-relevant information.

AC.L2-3.1.16 – **Wireless Access Authorization** – Authorize wireless access prior to allowing such connections.

AC.L2-3.1.17 – **Wireless Access Protection** – Protect wireless access using authentication and encryption.

AC.L2-3.1.18 – **Mobile Device Connection** – Control connection of mobile devices.

AC.L2-3.1.19 – **Encrypt CUI on Mobile** – Encrypt CUI on mobile devices and mobile computing platforms.

AC.L2-3.1.21 – **Portable Storage Use** – Limit use of portable storage devices on external systems.

2. AWARENESS AND TRAINING (AT)

Level 2

AT.L2-3.2.1 – **Role-Based Risk Awareness** – Ensure that managers, system administrators, and users of organizational systems are made aware of the security risks associated with their activities and of the applicable policies, standards, and procedures related to the security of those systems.

AT.L2-3.2.2 – **Role-Based Training** – Ensure that personnel are trained to carry out their assigned information security-related duties and responsibilities.

AT.L2-3.2.3 – **Insider Threat Awareness** – Provide security awareness training on recognizing and reporting potential indicators of insider threat.

3. AUDIT AND ACCOUNTABILITY (AU)

Level 2

AU.L2-3.3.1 – **System Auditing** – Create and retain system audit logs and records to the extent needed to enable the monitoring, analysis, investigation, and reporting of unlawful or unauthorized system activity.

AU.L2-3.3.2 – **Event Review** – Ensure that the actions of individual system users can be uniquely traced to those users so they can be held accountable for their actions.

AU.L2-3.3.3 – **Event Review** – Review and update logged events.

AU.L2-3.3.4 – **Audit Failure Alerting** – Alert in the event of an audit logging process failure.

AU.L2-3.3.5 – **login2Correlation** – Correlate audit record review, analysis, and reporting processes for investigation and response to indications of unlawful, unauthorized, suspicious, or unusual activity.

AU.L2-3.3.6 – **Reduction & Reporting** – Provide audit record reduction and report generation to support on-demand analysis and reporting.

AU.L2-3.3.7 – **Authoritative Time Source** – Provide a system capability that compares and synchronizes internal system clocks with an authoritative source to generate time stamps for audit records.

AU.L2-3.3.8 – **Audit Protection** – Protect audit information and audit logging tools from unauthorized access, modification, and deletion.

AU.L2-3.3.9 – **Audit Management** – Limit management of audit logging functionality to a subset of privileged users.

4. CONFIGURATION MANAGEMENT (CM)

Level 2

CM.L2-3.4.1 – **System Baselining** – Establish and maintain baseline configurations and inventories of organizational systems (including hardware, software, firmware, and documentation) throughout the respective system development life cycles.

CM.L2-3.4.2 – **Security Configuration Enforcement** – Establish and enforce security configuration settings for information technology products employed in organizational systems.

CM.L2-3.4.3 – **System Change Management** – Track, review, approve, or disapprove, and log changes to organizational systems.

CM.L2-3.4.4 – **Security Impact Analysis** – Analyze the security impact of changes prior to implementation.

CM.L2-3.4.5 – **Access Restrictions for Change** – Define, document, approve, and enforce physical and logical access restrictions associated with changes to organizational systems.

CM.L2-3.4.6 – **Least Functionality** – Employ the principle of least functionality by configuring organizational systems to provide only essential capabilities.

CM.L2-3.4.7 – **Nonessential Functionality** – Restrict, disable, or prevent the use of nonessential programs, functions, ports, protocols, and services.

CM.L2-3.4.8 – **Application Execution Policy** – Apply deny-by-exception (blacklisting) policy to prevent the use of unauthorized software or deny-all, permit-by-exception (whitelisting) policy to allow the execution of authorized software.

CM.L2-3.4.9 – **User-Installed Software** – Control and monitor user-installed software.

5. IDENTIFICATION AND AUTHENTICATION (IA)

Level 1

IA.L1-3.5.1 – Identification

Identify information system users, processes acting on behalf of users, or devices.

IA.L1-3.5.2 – Authentication
Authenticate (or verify) the identities of those users, processes, or devices, as a prerequisite to allowing access to organizational information systems.

Level 2

IA.L2-3.5.3 – **Multi Factor Authentication** – Use multifactor authentication for local and network access to privileged accounts and for network access to non-privileged accounts.

IA.L2-3.5.4 – **Replay-Resistant Authentication** – Employ replay-resistant authentication mechanisms for network access to privileged and non-privileged accounts.

IA.L2-3.5.5 – **Identifier Reuse** – Prevent the reuse of identifiers for a defined period.

IA.L2-3.5.6 – **Identifier Handling** – Disable identifiers after a defined period of inactivity.

IA.L2-3.5.7 – **Password Complexity** – Enforce a minimum password complexity and change of characters when new passwords are created.

IA.L2-3.5.8 – **Password Reuse** – Prohibit password reuse for a specified number of generations.

IA.L2-3.5.9 – **Temporary Passwords** – Allow temporary password use for system logins with an immediate change to a permanent password.

IA.L2-3.5.10 – **Cryptographically-Protected Passwords** – Store and transmit only cryptographically protected passwords.

IA.L2-3.5.11 – **Obscure Feedback** – Obscure feedback of authentication information.

6. INCIDENT RESPONSE (IR)

Level 2

IR.L2-3.6.1 – **Incident Handling** – Establish an operational incident-handling capability for organizational systems that includes preparation, detection, analysis, containment, recovery, and user-response activities.

IIR.L2-3.6.2 – **Incident Reporting** – Track, document, and report incidents to designated officials and/or authorities both internal and external to the organization.

IR.L2-3.6.3 – **Incident Response Testing** – Test the organizational incident response capability.

7. MAINTENANCE (MA)

Level 2

MA.L2-3.7.1 – **Perform Maintenance** – Perform maintenance on organizational systems.

MA.L2-3.7.2 – **System Maintenance Control** – Provide controls on the tools, techniques, mechanisms, and personnel used to conduct system maintenance.

MA.L2-3.7.3 – **Equipment Sanitization** – Ensure equipment removed for off-site maintenance

is sanitized of any CUI.

MA.L2-3.7.4 – **Media Inspection** – Check media containing diagnostic and test programs for malicious code before the media are used in organizational systems.

MA.L2-3.7.5 – **Nonlocal Maintenance** – Require multifactor authentication to establish nonlocal maintenance sessions via external network connections and terminate such connections when nonlocal maintenance is complete.

MA.L2-3.7.6 – **Maintenance Personnel** – Supervise the maintenance activities of personnel without required access authorization.

8. MEDIA PROTECTION (MP)

Level 1

MP.L1-3.8.3 – **Media Disposal** – Sanitize or destroy information system media containing Federal Contract Information before disposal or release for reuse.

Level 2

MP.L2-3.8.1 – **Media Protection** – Protect (i.e., physically control and securely store) system media containing CUI, both paper and digital.

MP.L2-3.8.2 – **Media Access** – Limit access to CUI on system media to authorized users.

MP.L2-3.8.4 – **Media Markings** – Mark media with necessary CUI markings and distribution limitations.

MP.L2-3.8.5 – **Media Accountability** – Control access to media containing CUI and maintain accountability for media during transport outside of controlled areas.

MP.L2-3.8.6 – **Portable Storage Encryption** – Implement cryptographic mechanisms to protect the confidentiality of CUI stored on digital media during transport unless otherwise protected by alternative physical safeguards.

MP.L2-3.8.7 – **Removable Media** – Control the use of removable media on system components.

MP.L2-3.8.8 – **Shared Media** – Prohibit the use of portable storage devices when such devices have no identifiable owner.

MP.L2-3.8.9 – **Protect Backups** – Protect the confidentiality of backup CUI at storage locations.

9. PERSONNEL SECURITY (PS)

Level 2

PS.L2-3.9.1 – **Screen Individuals** – Screen individuals prior to authorizing access to organizational systems containing CUI.

PS.L2-3.9.2 – **Personnel Actions** – Ensure that organizational systems containing CUI are protected during and after personnel actions such as terminations and transfers.

10. PHYSICAL PROTECTION (PE)

Level 1

PE.L1-3.10.1 – **Limit Physical Access** – Limit physical access to organizational information systems, equipment, and the respective operating environments to authorized individuals.

PE.L1-3.10.3 – **Escort Visitors** – Escort visitors and monitor visitor activity.

PE.L1-3.10.4 – **Physical Access Logs** – Maintain audit logs of physical access.

PE.L1-3.10.5 – **Manage Physical Access** – Control and manage physical access devices.

Level 2

PE.L2-3.10.2 – **Monitor Facility** – Protect and monitor the physical facility and support infrastructure for organizational systems.

PE.L2-3.10.6 – **Alternative Work Sites** – Enforce safeguarding measures for CUI at alternate work sites.

11. RISK ASSESSMENT (RA)

Level 2

RA.L2-3.11.1 – **Risk Assessments** – Periodically assess the risk to organizational operations (including mission, functions, image, or reputation), organizational assets, and individuals, resulting from the operation of organizational systems and the associated processing, storage, or transmission of CUI.

RA.L2-3.11.2 – **Vulnerability Scan** – Scan for vulnerabilities in organizational systems and applications periodically and when new vulnerabilities affecting those systems and applications are identified.

RA.L2-3.11.3 – **Vulnerability Remediation** – Remediate vulnerabilities in accordance with risk assessments.

12. SECURITY ASSESSMENT (CA)

Level 2

CA.L2-3.12.1 – **Security Control Assessment** – Periodically assess the security controls in organizational systems to determine if the controls are effective in their application.

CA.L2-3.12.2 – **Plan of Action** – Develop and implement plans of action designed to correct deficiencies and reduce or eliminate vulnerabilities in organizational systems.

CA.L2-3.12.3 – **Security Control Monitoring** – Monitor security controls on an ongoing basis to ensure the continued effectiveness of the controls.

CA.L2-3.12.4 – **System Security Plan** – Develop, document, and periodically update system security plans that describe system boundaries, system environments of operation, how security requirements are implemented, and the relationships with or connections to other

systems.

13. SYSTEM AND COMMUNICATIONS PROTECTION (SC)

Level 1

SC.L1-3.13.1 – **Boundary Protection** – Monitor, control, and protect organizational communications (i.e., information transmitted or received by organizational information systems) at the external boundaries and key internal boundaries of the information systems.

SC.L1-3.13.5 – **Public-Access System Separation** – Implement subnetworks for publicly accessible system components that are physically or logically separated from internal networks.

Level 2

SC.L2-3.13.2 – **Security Engineering** – Employ architectural designs, software development techniques, and systems engineering principles that promote effective information security within organizational systems.

SC.L2-3.13.3 – **Role Separation** – Separate user functionality from system management functionality.

SC.L2-3.13.4 – **Shared Resource Control** – Prevent unauthorized and unintended information transfer via shared system resources.

SC.L2-3.13.6 – **Network Communication by Exception** – Deny network communications traffic by default and allow network communications traffic by exception (i.e., deny all, permit by exception).

SSC.L2-3.13.7 – **Split Tunneling** – Prevent remote devices from simultaneously establishing non-remote connections with organizational systems and communicating via some other connection to resources in external networks (i.e., split tunneling).

SC.L2-3.13.8 – **Data in Transit** – Implement cryptographic mechanisms to prevent unauthorized disclosure of CUI during transmission unless otherwise protected by alternative physical safeguards.

SC.L2-3.13.9 – **Connections Termination** – Terminate network connections associated with communications sessions at the end of the sessions or after a defined period of inactivity.

SC.L2-3.13.10 – **Key Management** – Establish and manage cryptographic keys for cryptography employed in organizational systems.

SC.L2-3.13.11 – **CUI Encryption** – Employ FIPS-validated cryptography when used to protect the confidentiality of CUI.

SC.L2-3.13.12 – **Collaborative Device Control** – Prohibit remote activation of collaborative computing devices and provide indication of devices in use to users present at the device.

SC.L2-3.13.13 – **Mobile Code** – Control and monitor the use of mobile code.

SC.L2-3.13.14 – **Voice over Internet Protocol** – Control and monitor the use of Voice over Internet Protocol (VoIP) technologies.

SC.L2-3.13.15 – **Communications Authenticity** – Protect the authenticity of communications

sessions.

SC.L2-3.13.16 – **Data at Rest** – Protect the confidentiality of CUI at rest.

14. SYSTEM AND INFORMATION INTEGRITY (SI)

Level 1

SI.L1-3.14.1 – **Flaw Remediation** – Identify, report, and correct information and information system flaws in a timely manner.

SI.L1-3.14.2 – **Malicious Code Protection** – Provide protection from malicious code at appropriate locations within organizational information systems.

SI.L1-3.14.4 – **Update Malicious Code Protection** – Update malicious code protection mechanisms when new releases are available.

SI.L1-3.14.5 – **System & File Scanning** – Perform periodic scans of the information system and real-time scans of files from external sources as files are downloaded, opened, or executed.

Level 2

SI.L2-3.14.3 – **Security Alerts & Advisories** – Monitor system security alerts and advisories and take action in response.

SI.L2-3.14.6 – **Monitor Communications for Attacks** – Monitor organizational systems, including inbound and outbound communications traffic, to detect attacks and indicators of potential attacks.

SI.L2-3.14.7 – Identify Unauthorized Use – Identify unauthorized use of organizational systems.

APPENDIX V: DATA PROCESSING AGREEMENT TEMPLATE

[This appendix contains a sample data processing agreement between a data processor and a department/unit of the Ministry of Health. It includes guidelines that the data processor shall follow to maintain data protection, privacy and confidentiality and to ensure the rights of data subjects.]

This Data Processing Agreement ("Agreement") forms part of the Contract for

Services ("Principal Agreement") between

____

____

____

(the "[Department/Unit of the Ministry of Health]")

And

____

____

____

(the "Data Processor")

(together as the "Parties")

WHEREAS

(A) The [Department/Unit of the Ministry of Health] acts as a Data Controller.

(B) The [Department/Unit of the Ministry of Health] wishes to subcontract certain Services, which imply the processing of personal data, to the Data Processor.

(C) The Parties seek to implement a Data Processing Agreement that complies with the requirements of the current legal framework in relation to data processing and with Data Protection and Privacy Act 2019 regarding the processing of personal data and on the free movement of such data.

(D) The Parties wish to lay down their rights and obligations.

IT IS AGREED AS FOLLOWS:

1. Definitions and Interpretation

1.1 Unless otherwise defined herein, capitalized terms and expressions used in this Agreement shall have the following meaning:

1.1.1 "Agreement" means this Data Processing Agreement and all Schedules;

1.1.2 "[Department/Unit of the Ministry of Health] Personal Data" means any personal data processed by a Contracted Processor on behalf of [Department/Unit of the Ministry of Health] pursuant to or in connection with the Principal Agreement;

1.1.3 "Contracted Processor" means a Subprocessor;

1.1.4 "Data Protection Laws" means Data Protection and Privacy Act 2019 and, to the extent applicable, the data protection or privacy laws of any other country;

1.1.8 "Data Transfer" means:

1.1.8.1 a transfer of [Department/Unit of the Ministry of Health] Personal Data from the [Department/Unit of the Ministry of Health] to a Contracted Processor; or

1.1.8.2 an onward transfer of [Department/Unit of the Ministry of Health] Personal Data from a Contracted Data Processor to a Subcontracted Data Processor, or between two establishments of a Contracted Processor,

In each case, where such transfer would be prohibited by Data Protection Laws (or by the terms of data transfer agreements put in place to address the data transfer restrictions of Data Protection Laws);

1.1.9 "Services" means the [__________________] services the [Department/Unit of the Ministry of Health] provides.

1.1.10 "Subprocessor" means any person appointed by or on behalf of the Data Processor to receive and process Personal Data on behalf of the [Department/Unit of the Ministry of Health] in connection with the Agreement.

2. Processing of [Department/Unit of the Ministry of Health] Personal Data

2.1 Processor shall:

2.1.1 comply with all applicable Data Protection Laws in the Processing of [Department/Unit of the Ministry of Health] Personal Data; and

2.1.2 not process [Department/Unit of the Ministry of Health] Personal Data other than on the relevant [Department/Unit of the Ministry of Health] documented instructions;

2.2 The [Department/Unit of the Ministry of Health] instructs Data Processor to process [Department/Unit of the Ministry of Health] Personal Data.

3. Processor Personnel

Data Processor shall take reasonable steps to ensure the reliability of any employee, agent, or contractor of any Contracted Processor who may have access to the [Department/Unit of the Ministry of Health] Personal Data, ensuring in each case that access is strictly limited to those individuals who need to know or access the relevant [Department/Unit of the Ministry of Health] Personal Data, as strictly necessary for the purposes of the Principal Agreement, and to comply with Applicable Laws in the context of that individual's duties to the Contracted Processor, ensuring that all such individuals are subject to confidentiality undertakings or professional or statutory obligations of confidentiality.

4. Security

4.1 Taking into account the state of the art, the costs of implementation, and the nature, scope, context, and purposes of Processing as well as the risk of varying likelihood and severity for the rights and freedoms of natural persons, Data Processor shall in relation to the [Department/Unit of the Ministry of Health] Personal Data implement appropriate technical and organizational measures to ensure a level of security appropriate to that risk, including, as appropriate, the measures referred to in [Relevant Country Policy/Guidelines].

4.2 In assessing the appropriate level of security, Data Processor shall take into account the risks that are presented by Processing, in particular from a Personal Data Breach.

5. Subprocessing

5.1 Data Processor shall not appoint (or disclose any [Department/Unit of the Ministry of Health] Personal Data to) any Subprocessor unless required or authorized by the [Department/Unit of the Ministry of Health].

6. Data Subject Rights

6.1 Taking into account the nature of the Processing, Data Processor shall assist the [Department/Unit of the Ministry of Health] by implementing appropriate technical and organizational measures, insofar as this is possible, for the fulfilment of the [Department/Unit of the Ministry of Health] obligations, as reasonably understood by [Department/Unit of the Ministry of Health], to respond to requests to exercise Data Subject rights under the Data Protection Laws.

6.2 Data Processor shall:

6.2.1 promptly notify [Department/Unit of the Ministry of Health] if it receives a request from a Data Subject under any Data Protection Law in respect of [Department/Unit of the Ministry of Health] Personal Data; and

6.2.2 ensure that it does not respond to that request except on the documented instructions of [Department/Unit of the Ministry of Health] or as required by applicable laws to which the Data Processor is subject, in which case Data Processor shall to the extent permitted by applicable

laws inform [Department/Unit of the Ministry of Health] of that legal requirement before the Contracted Processor responds to the request.

7. Personal Data Breach

7.1 Data Processor shall notify [Department/Unit of the Ministry of Health] without undue delay upon Data Processor becoming aware of a Personal Data Breach affecting [Department/Unit of the Ministry of Health] Personal Data, providing [Department/Unit of the Ministry of Health] with sufficient information to allow the [Department/Unit of the Ministry of Health] to meet any obligations to report or inform Data Subjects of the Personal Data Breach under the Data Protection Laws.

7.2 Data Processor shall cooperate with the [Department/Unit of the Ministry of Health] and take reasonable commercial steps as are directed by [Department/Unit of the Ministry of Health] to assist in the investigation, mitigation, and remediation of each such Personal Data Breach.

8. Data Protection Impact Assessment and Prior Consultation

Data Processor shall provide reasonable assistance to the [Department/Unit of the Ministry of Health] with any data protection impact assessments, and prior consultations with supervising authorities or other competent data privacy authorities, which [Department/Unit of the Ministry of Health] reasonably considers to be required by Data Protection and Privacy Act 2019 or equivalent provisions of any other Data Protection Law, in each case solely in relation to Processing of [Department/Unit of the Ministry of Health] Personal Data by, and taking into account the nature of the Processing and information available to, the Contracted Processors.

9. Deletion or Return of [Department/Unit of the Ministry of Health] Personal Data

9.1 Subject to this paragraph, Data Processor shall promptly and in any event within ten (10) business days of the date of cessation of any Services involving the Processing of [Department/Unit of the Ministry of Health] Personal Data (the "Cessation Date"), delete and procure the deletion of all copies of those [Department/Unit of the Ministry of Health] Personal Data.

9.2 Data Processor shall provide written certification to [Department/Unit of the Ministry of Health] that it has fully complied with this paragraph (9.1-.9.2) within ten (10) business days of the Cessation Date.

10. Audit Rights

10.1 Subject to this paragraph, Data Processor shall make available to the [Department/Unit of the Ministry of Health] on request all information necessary to demonstrate compliance with this Agreement, and shall allow for and contribute to audits, including inspections, by the [Department/Unit of the Ministry of Health] or an auditor mandated by the [Department/Unit of

the Ministry of Health] in relation to the Processing of the [Department/Unit of the Ministry of Health] Personal Data by the Contracted Processors.

10.2 Information and audit rights of the [Department/Unit of the Ministry of Health] only arise under paragraph 10.1 to the extent that the Agreement does not otherwise give them information and audit rights meeting the relevant requirements of Data Protection Law.

11. Data Transfer

11.1 The Data Processor may not transfer or authorize the transfer of Personal Data to other countries without the prior written consent of the [Department/Unit of the Ministry of Health]. If Personal Data processed under this Agreement are transferred to or from another country, the Parties shall ensure that the Personal Data are adequately protected.

12. General Terms

12.1 Confidentiality. Each Party must keep this Agreement and information it receives about the other Party and its business in connection with this Agreement ("Confidential Information") confidential and must not use or disclose that Confidential Information without the prior written consent of the other Party except to the extent that:

(a) disclosure is required by law;

(b) the relevant information is already in the public domain.

12.2 Notices. All notices and communications given under this Agreement must be in writing and shall be delivered personally, sent by post to the mailing address, or sent by email to the email address set out in the heading of this Agreement or at such other address as notified from time to time by the Parties changing address.

13. Governing Law and Jurisdiction

13.1 This Agreement is governed by the laws of [_______________.]

13.2 Any dispute arising in connection with this Agreement, which the Parties will not be able to resolve amicably, will be submitted to the exclusive jurisdiction of the courts of [_________________], subject to possible appeal to [___________________________________].

IN WITNESS WHEREOF, this Agreement is entered into with effect from the date first set out below.

[Department/Unit of the Ministry of Health]

Signature _______________________________

Name: _________________________________

Title: ________________________________

Date Signed: __________________________

Data Processor

Signature _____________________________

Name _________________________________

Title __________________________________

Date Signed ___________________________

REPUBLIC OF UGANDA
MINISTRY OF HEALTH

THE UGANDA HEALTH DATA ACCESS, SHARING AND USE GUIDELINES

SEPTEMBER 2023

DOCUMENT REVIEWS AND APPROVALS

**Version**	**Owner**	**Author**	**Approver**	**Date of Approval** *(MMDDYY)*
01	Ministry of Health	Division of Health Information Management	Top Management Committee	18.09.2024

Table of Contents

Foreword **iv**

Preface **v**

Acknowledgement **vi**

Abbreviations **vii**

Definition Of Terms **viii**

1.0 Introduction 1

2.0 Rationale 2

3.0 Purpose 2

4.0 Overall Objective 2

4.1 Specific Objectives 3

5.0 Development Methodology 3

6.0 Target Audience and Applicability 3

7.0 Scope of the Guideline 4

8.0 Classifying Data to Facilitate Data Access, Sharing, and Use 5

8.1 Classification Framework 6

9.0 Governance Structure 7

9.1 Roles and Responsibilities 8

10.0 Data Ownership 10

11.0 Procedures for Accessing, Sharing and Using Individual-level/Personal Data 11

11.1 Procedures for Accessing Data from Ministry of Health Data Repository 13

11.2 Procedures for Research-Generated Health Information 13

11.3 Conducting Research or any Population-Based Survey on Health 14

11.4 Data Access, Sharing and Use in Public Health Emergencies 14

11.5 Secure and Ethical Cross-Border Data Transfers 15

12. 0 Data Management 16

12.1 Measures for Confidentiality and Protection in Data Sharing 18

12.2 Data use and sharing agreement 20

12.3 Contents of the Agreement 20

13.0 Compliance and Guidelines Governance 22

13.1 Non-Compliance and Repercussions with these Guidelines 22

13.2 Liability, Penalties and Remedies 22
13.3 Sale of Health Data 23
14.0 Dissemination and Adoption of the Guidelines 23
15.0 Conclusion 23
Appendix 1: Generic Data Access, Sharing, and Use Agreement Template 26
Appendix 2: Health Data Access, Sharing, and Use Compliance Checklist 39

Foreword

The Government of Uganda promotes the use of data for decision-making and policy formulation. The Ministry of Health Strategic Plan 2020/21 – 2024/25 also recognises the use of data as a key enabler for supporting the health system to deliver good health to the population. This is further articulated in the Uganda Health Information and Digital Health Strategic Plan 2020/21-2024/25.

The Uganda Health Data Access, Sharing and Use Guidelines are intended to standardize the implementation of health data access, sharing and use across Uganda's health system. The guidelines are aligned with the Data Protection and Privacy Act 2019 to ensure the operationalisation of the law within the health sector.

These guidelines will serve as a framework to ensure secure information access, exchange, and use as well as timely identification and addressing of vulnerabilities.

In addition, the guidelines will ensure that health data is accessible while protecting the Privacy and Confidentiality of those from whom data was obtained. These guidelines shall apply to all the health practitioners both in the public and private sectors while collecting, processing, archiving, accessing and sharing data. All stakeholders in the health spectrum are therefore called upon to embrace the use of these guidelines while handling any form of the health-related data.

....

Dr. Henry G. Mwebesa

DIRECTOR GENERAL HEALTH SERVICES

Preface

This document presents Uganda's Health Data Access, Sharing and Use Guidelines for the Health Sector. The guidelines are aligned with the Data Protection and Privacy Act 2019, Data Protection and Privacy Regulations 2021, National ICT Policy 2018, Health Information and Digital Health Strategic Plan 2020/2021-2024-2025, and Ministry of Health Strategic Plan 2020/2021-2024-2025.

Health Information and supporting systems are critical assets for health service delivery. In Uganda like in many places, health data is faced with many privacy and confidentiality threats from a wide range of sources. Therefore, protecting the health data of Uganda's health system clients is of prime importance to the Ministry of Health. These guidelines provide comprehensive guidance to healthcare providers, policymakers, and stakeholders on best practices for sharing and using health data throughout its lifecycle.

By adhering to these guidelines, we not only fulfil our ethical and legal obligations to safeguard patient privacy but also foster trust and confidence among individuals accessing healthcare services. Furthermore, these guidelines will pave the way for innovation and collaboration in leveraging health data to improve healthcare delivery, research, and public health interventions.

All stakeholders in the health spectrum are therefore called upon to adopt the use of these guidelines while handling health-related data.

....

Dr. Sarah Byakika

Commissioner Health Services

Department of Planning, Financing and Policy

Acknowledgement

The Ministry of Health expresses its profound gratitude to all departments and programs, members of the Health Information Innovation and Research Technical Working Group and the Data Management Subcommittee who contributed technical inputs leading to the successful completion of this document. Special appreciation goes to the Division of Health Information Management (DHIM) and the Information Communication Technology (ICT) Section for the overall guidance to ensure that the guidelines are aligned with the Health Information and Digital Health Strategic Plan 2020/21-2024/25.

I acknowledge and thank all development and implementing partners that provided financial and technical support for this process, specifically MUSPH/METS, HISP Uganda, IDI and the Centers for Disease Control and Prevention (CDC). DHIM is grateful for all the support, sacrifice and contribution that has been invested in its successful development.

Finally, the Ministry of Health is grateful to the Ministry of Information Communication Technology and National Guidance, National Information Technology Authority Uganda (NITA-U), Personal Data Protection Office, National Identification & Registration Authority, Uganda Health Informatics Association, Makerere University Health Informatics Research Group, and all those institutions and individuals who have not been specifically mentioned above, but who directly or indirectly contributed to the successful development and finalization of these guidelines.

....

Mr. Paul Mbaka

Assistant Commissioner Health Services

Health Information Management

Abbreviations

**DPIA**	Data Protection Impact Audit
**DUA**	Data Use Agreement
**GRO**	Grievance Redressal Officer
**HIE**	Health Information Exchange
**HIS**	Health Information System
**HISP**	Health Information System Programs
**ICT**	Information and Communications Technology
**IPR**	Intellectual Property Rights
**MoH**	Ministry of Health
**PII**	Personal Identifiable Information

Definition Of Terms

Anonymization	Is an irreversible process of removing or transforming personally identifiable information (PII) to a form in which a data subject cannot be identified through any means reasonably likely to be used to identify such data subject.
Biometric Data	Personal data resulting from measurements and calculations relating to the physical, physiological, or behavioural characteristics of a natural person that allow or confirm the unique identification of that natural person, such as facial images or fingerprint data.
Confidentiality	The obligations of those who receive data and/or information to respect the privacy interests of those to whom the data relates.
Consent	Any freely given, specific, informed, and unambiguous indication of the data subject's wishes by which they, by a statement or by a clear affirmative action, signify agreement to the processing of personal data relating to them.
Consent Artefact	A readable document that specifies the parameters and scope of data sharing and access that a data subject consents to in any personal data sharing transaction.
Data	Data refers to raw, unorganized facts or observations that are collected, stored, and processed to create meaningful information. Data can be in the form of text, images, numbers, audio, video
Data Collector	Person or an entity that collects data.

Data Controller	A natural or legal person (e.g., an organisation or other entity) who alone, jointly with other persons or in common with other persons or as a statutory duty determines the purpose for and the manner in which data is processed or is to be processed.
Data Processor	Person/entity other than an employee of the data controller that processes data on behalf of the data controller.
Data Protection Officer	Is an individual employed by a data controller who understands and applies data protection regulations to the data control operations and serves as a liaison between the data controller and regulators.
Data Subject	is an individual from whom or in respect of whom personal information has been requested, collected, collated, processed, or stored.
De-identification	The process by which a data controller or data processor may remove or mask identifiers from personal data, or replace them with a fictitious name or code that is unique to a data subject but does not, on its own, directly identify the data subject.
Disclosure	Refers to the act of revealing or making information known, often in a formal or public context for transparency, accountability, or legal compliance.

Data Sharing	The practice of making data available to others for various purposes, including analysis, research, collaboration, and decision-making. It involves providing access to data sets, information, or digital resources to individuals, organizations, or the public. Data sharing is a function of deciding what data, in which form, by what means and to whom it needs to be shared.
Data Access	Data access refers to the function of providing access to the shared data to the correct entities.
Encryption	The process of transforming data/value into code to prevent unauthorized access.
Health Data	Personal data related to the physical or mental health of a natural person, including the provision of health care services that reveal information about their health status.
Natural Person	Any person with rights and responsibilities defined by international human rights conventions
Notification	A legally defined process of informing about an intention to access and use personal data
Personal Data	Any information about a person from which the person can be identified and/or includes an expression of opinion about the individual.
Personal Health Identifier	A subset of “personal data”, is the data that could alone or combined with other information potentially identify a data subject and/or be used to distinguish one data subject from another; a personal health identifier could also be used to re-identify

	previously de-identified data and could include a data subject's demographic and location information, family and relationship information, and contact details.
Personally Identifiable Information	Any data that can be used to identify an individual. Personally identifiable information includes but is not limited to names, physical and internet protocol addresses, financial information, login information, biometric identifiers, video footage, geographic location data, social media accounts, email addresses, and insurance identification numbers.
Privacy	An individual's right to control the acquisition, use, or disclosure of their personal information.
Processing	Any operation performed on personal data, such as collecting, creating, recording, structuring, organizing, storing, retrieving, accessing, using, seeing, sharing, communicating, disclosing, altering, adapting, updating, combining, erasing, destroying, or deleting personal data; or restricting access or changes to personal data; or preventing destruction of the data.
Protection	Refers to the practices, policies, and measures implemented to safeguard and manage the privacy, integrity, and confidentiality of personal and sensitive information.
Pseudonymization	The processing of personal data in such a manner that the personal data can no longer be attributed to a specific data subject without the use of additional information, provided that such additional information is kept separately so as to ensure that the personal data is not attributed to an identified or identifiable natural person.

Sensitive Data	This means that the stored data have confidential and/or personal data, therefore, processing such data is limited.
Sensitive Personal data	Refers to information that, if disclosed, could result in harm to individuals or organizations.
Third-party	A natural or legal person, public authority, agency, or body other than the data subject, data controller, or data processor, and a person who, under the direct authority of the data controller or data processor, is authorized to process personal data.

1.0 Introduction

The world has experienced exponential growth in the demand for access to health-related data, spurred by the recognition of its pivotal role in driving innovation and enhancing service delivery across healthcare systems. This heightened demand has been further fueled by rapid advancements in Information Communication Technologies (ICTs), which offer unprecedented opportunities to augment and optimize initiatives aimed at harnessing health data for various purposes.

Data sharing is an important way to increase the ability of researchers, academia, scientists and policy-makers to analyze and translate data into meaningful reports, knowledge and informed decision-making. Currently, health data analytics activities require fetching, integrating and/or triangulating data from different sources using rigorous scientific techniques and advanced technologies. Indeed, the ultimate goal of doing these tasks is to generate evidence that crosses national and sub-national lines. Most importantly, timely data sharing, with recognition of the interests of investigators and respective institutions who collect the data, is essential for expedited translation of research results into health policy to improve national and subnational health programs in Uganda.

In Uganda, the generation and use of data for evidence-informed decision-making have been the health sector's priority for a long time. The Ministry of Health (MoH) has been implementing the Health Information and Digital Strategic Plan with the objective of maximizing the availability, accessibility, quality, and use of information for decision-making processes. To enable data access, sharing, and use, the national adoption of key technical standards is needed. This guideline has adopted the Uganda Health Information Exchange and Interoperability Guidelines, the Uganda Health Data Protection, Privacy, and Confidentiality Guidelines, the Data Protection and Privacy Regulations 2020, the Data Protection and Privacy Act 2019, The National Information Security Policy (framework) 2014, The National Records and Archives Act 2001 and The Access to Information Act (2005)

2.0 Rationale

The landscape of health data management in Uganda faces significant challenges due to the absence of comprehensive standards for data access, sharing, and use. The lack of these standards at all levels of the health system leads to suboptimal utilization of data, breach of personal privacy and confidentiality, and misunderstanding of data ownership. In light of these challenges, formulating data access, sharing, and use guidelines is essential to address gaps, enhance data utilization, and ultimately improve health outcomes across Uganda.

3.0 Purpose

The guidelines seek to establish an effective, transparent, and accountable framework for managing health data access, use and sharing in Uganda.

By establishing these guidelines, several important aspects will be addressed:

a) Maximizing health data use for decision-making
b) Improving health data transparency and accountability
c) Minimizing duplication of efforts
d) Clarifying ownership of health data and information
e) Foster a collaborative approach to health data management
f) Improving individual health data privacy, confidentiality, and security
g) Facilitate collaboration among researchers, policymakers, and healthcare practitioners, fostering a conducive environment for health research.

4.0 Overall Objective

The overall objective of this guideline is to define a set of principles, requirements, strategies and procedures that guide health and health-related data sources or owners to enhance data access, sharing and use within and between the institutions and individuals who own and/or deal with health and health-related data.

4.1 Specific Objectives

a. Promote and ensure the health administrative units and health facilities, research institutes, academia, agencies, researchers and development partners share health data and evidence as openly as possible;
b. Facilitate clear and transparent health data access and sharing practices using standard procedures among relevant stakeholders
c. Define procedures for health-related data classifications for better data access and sharing practice;
d. Define the roles and responsibilities of health data owners, controllers and users during data storage, access and sharing processes

5.0 Development Methodology

A consultative approach was used in the development of the guidelines. Stakeholders from the Ministry of Health (MoH), Ministry of ICT and National Guidance, Personal Data Protection Office (PDPO), Ministry of Public Service, NITA-U, NIRA, Development and Implementing partners, Civil Society Organizations, Academia, and other members of the Health Information Innovation and Research (HIIRE) Technical Working Group (TWG) collaborated in developing these guidelines.

6.0 Target Audience and Applicability

These guidelines are intended for both the public and private sector targeting a wide range of stakeholders involved in the data management within Uganda's health ecosystem. This includes:

a. General Public
b. Healthcare Providers: Clinicians, nurses, and other healthcare professionals responsible for collecting and managing health data.
c. Data Managers: Personnel responsible for organizing, storing, and maintaining health data within health facilities and institutions.
d. Policymakers: Individuals involved in developing health policies and strategies at national, regional, and local levels.

e. Researchers: Scientists and researchers conducting studies using health data for epidemiological, clinical, or public health research.
f. Technical Personnel: IT specialists and developers involved in designing and implementing health information systems.

7.0 Scope of the Guideline

These guidelines typically cover issues like data ownership, intellectual property rights, permitted uses of the data, data security, and data sharing duration. The scope of this guideline are as follows: Covers the access, use and sharing of data only, excluding biological samples

a. Covers the access, use and sharing of health data in the context of the Public and Private Sector i.e. Ministry of Health services and programs.
b. Provides a mechanism to share other health data than those already being collected, processed, anonymized, analyzed, and shared.
c. The guideline will not be applied retrospectively to data sharing agreements already in place, except where the same is being renewed or there are amendments to such data sharing agreements.
d. Data access is governed by the data processing obligations listed in the Uganda Health Data Privacy, and Confidentiality guideline.

8.0 Classifying Data to Facilitate Data Access, Sharing, and Use

Data classification by data sources/owners is one of the first steps in moving towards a data-sharing paradigm. Data elements are classified based on the sensitivity and impact of the sharing of that data to the users. Data held by data sources can be of the following types.

Sn	Sensitivity Level	Classification and Dissemination Methods	Data and Information Types
1	**Low Sensitivity**<br>Data elements with minimal risk or impact if accessed or disclosed.	**Classification: Public Data**<br>This type of data is freely accessible to the public. It can be freely used, reused, and redistributed without repercussions.	• Aggregate outline/institution-based health information data.<br>• Surveys and surveillances<br>• Program-specific evaluations<br>• Approved reports and published documents.
2	**Moderate Sensitivity**<br>Data elements containing identifiable or sensitive health information requiring protection.	**Classification: Internal-only Data**<br>Data that is strictly accessible to internal organizational personnel or internal employees who are granted access.	• Ongoing/unpublished research works/papers (manuscripts)<br>• unpublished/ unendorsed or non-finalized works e.g. Textual/print documents, audio, video, images, graphical or cartographical materials),<br>• Biological specimen with no unique identifier (to prevent uncontrolled depletion)<br>• Uniquely identified data of routine and survey data
3	**High Sensitivity**<br>Data elements with significant privacy or security implications, requiring stringent safeguards and access controls.	**Classification: Confidential Data**<br>Access to confidential data requires specific authorization and/or clearance. This may contain identifiable or sensitive health information requiring protection.	• Biologic specimen with unique identifier<br>Aggregated survey results (e.g. aggregated to the household level and with additional disaggregation based on different indicators)

			• Registries for health workers, clients and health products<br>• Financial statements and accounts.
**4**	**Severe Sensitivity**<br>Information or data that, if disclosed or accessed without proper authorization, are likely to cause severe harm or negative impacts and/or damage to affected people and/or humanitarian actors and/or impede the conduct of the work of a response.	**Classification: Restricted Data**<br>Restricted data includes data that, if compromised or accessed without authorization, could lead to criminal charges and massive legal fines or cause irreparable damage to the organisation. This may have significant privacy or security implications, requiring stringent safeguards and access controls.	• Physical/recorded or biologic data of individuals with unique identifiers.<br>• Geographic position data of military health facilities linked to military bases and training facilities.<br>• Raw survey data, e.g. individual survey responses at HH-level data<br>• Personal data of beneficiaries (i.e. Beneficiary lists, patient records, etc.)<br>• Line lists/line listing data

8.1 Classification Framework

To effectively classify health data, organizations should adopt a structured approach:

1. **Data Inventory**: Catalog all types of health data collected and processed by the organization.
2. **Sensitivity Assessment**: Evaluate the sensitivity and risk associated with each type of health data.
3. **Access Controls**: Implement access controls based on data sensitivity to limit who can view and process the data.
4. **Policy Implementation**: Develop and enforce policies and procedures for handling, protecting, and sharing health data.
5. **Regular Review and Update**: Periodically review and update data classifications to reflect new types of health data and changing regulatory requirements.

9.0 Governance Structure

The Governance of health data access, sharing and use shall follow the existing governance and management structures at the national and decentralized levels, as summarized in Figure 1.

Technical oversight is the mandate of the Division of Health Information Management (DHIM) under the Department of Planning, Financing and Policy which guides and coordinates all stakeholders involved in health data collection, processing, use and storage. This function is similarly decentralized at the Local Government level, as summarized in Figure 1.

The main task of the Health Information Innovation and Research Technical Working Group (HIIRE TWG) is reviewing and giving advice on Health Information Systems (HIS) and Data and Digital Health policy and strategic related issues from the user departments and other stakeholders.

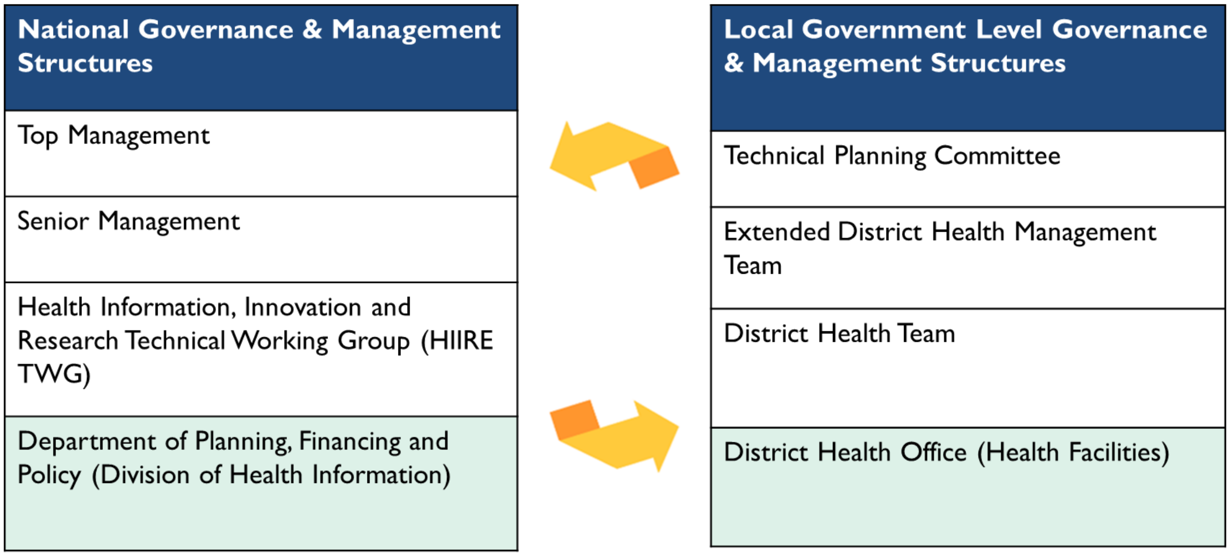

The Ministry of Health together with the Personal Data Protection Office (PDPO) shall be responsible for monitoring the application of these Guidelines in order to protect the fundamental rights and freedoms of natural persons in relation to collection, processing, storage, access, sharing, and use of their personal data and to facilitate the free flow of personal data within and outside the country in relation to health service delivery.

The PDPO shall represent the supervisory authority in any dispute or conflict arising from infringement of the provisions of these Guidelines.

At the national and subnational levels, a Data Protection Officer (DPO) shall in addition to the functions identified under these Guidelines, communicate with regulators and external stakeholders on matters concerning data privacy and serve as an escalation point for decision-making on health data governance and other matters concerning health data.

The roles and responsibilities of the data controller, data processor, and Ethics and Data Sharing Coordination Committee are described below;

9.1 Roles and Responsibilities

1. Data Controller

The controller at the national level shall be the Director General of the Ministry of Health; at District/City level, the District Health Officer/City Health Officer shall be the data controller for all public and private health facilities in the district; and at the facility level, the data controller shall be the Health Facility In charge.

a. The data controller shall implement appropriate technical and administrative measures to ensure data access, sharing and use are performed in accordance with these guidelines and other related guidelines.

b. The controller shall consider the nature, scope, context, and purposes of data collection, processing and confidentiality risks at varying likelihood and severity of impact on the rights and freedoms of data subjects, and this shall determine technical and administrative measures to be put in place.

c. Upon receiving a request for data, the data controller shall consider the intent and extent of data processes, which have varying likelihood and severity of risks to natural persons. The controller shall determine the means of collection, data processing, and he or she will also implement appropriate technical and administrative measures, such as pseudonymization and minimization, including other integral safeguards that meet the requirements of these guidelines to share and use data securely.

d. The controller shall implement appropriate technical and administrative measures (including physical measures) to ensure that, upon receipt of a request for access and data sharing, the following are taken into consideration:
   i. That a data sharing agreement *(Sample Template in Appendix 1)* is duly reviewed and approved.
   ii. Ensure that the data sharing agreement clearly stipulates the type of personal data collected, the extent of their processing, the period of their storage, and their accessibility
   iii. Only personal data that are necessary for each specific purpose shall be processed
   iv. Ensure that consent is provided before access to personal data of a natural person is provided.
   v. Ensure adherence to the Data Protection and Privacy Act 2019.

e. In the event of any personal data breach, the Data Protection, Privacy, and Confidentiality Guidelines shall be followed to guide what is to be done in case of a data breach. This will include reporting all data breaches to the Personal Data Protection Office immediately after becoming aware of it.

2. Data Processor

a. The primary role of a data processor is to process data according to the instructions provided by the data controller or as defined in a data processing agreement *(Template provided in the Uganda Health Data Protection, Privacy and Confidentiality Guidelines)*. This may involve tasks such as data collection, recording, structuring, storage, retrieval, modification, use, transmission, and erasure.
b. The processor shall not reassign the role to another processor without prior specific or general written authorization of the controller.
c. In the case of general written authorization, the processor shall inform the controller of any intended changes concerning the addition or replacement of other processors, thereby giving the controller the opportunity to object to such changes.
d. Processing shall be governed by the approved data processing agreement, that shall clearly stipulate the subject matter, and duration of the processing, the nature and purpose of the

processing, the type of personal data, the categories of data subjects, and the obligations and rights of the controller.

e. When a processor engages another processor for carrying out specific processing activities on behalf of the controller, the same data protection obligations as set out in a data processing agreement or other legal contract between the controller and the processor shall be imposed on that other processor by way of a data processing agreement or other legal contract under the Uganda Data Protection and Privacy Act 2019. Should that other processor fail to fulfill his or her data protection obligations, the initial processor shall remain fully liable to the controller for the performance of that other processor's obligations.
f. The data processing agreement or other legal contracts shall be in writing, including in electronic form.

3. Ethics and Data Sharing Coordination Committee

The role of the Ethics and Data Sharing Coordination Committee is to oversee the ethical aspects of collecting, storing, processing, and sharing health data for scientific research and healthcare service delivery. This committee reviews and approves such activities to ensure they comply with ethical standards. Even when data processing meets legal requirements, controllers and processors must implement safeguards to mitigate ethical risks. These risks include processing individuals' health data who haven't consented, using techniques that infringe upon data subject rights, and sharing data with entities outside the country.

The other roles and responsibilities of the data collector and the data protection officer are further described in **Section 8** of The Uganda Health Data Protection, Privacy and Confidentiality Guidelines.

10.0 Data Ownership

Data ownership shall remain the property of the research institute, agency, department, ministry, health facility, health system clients or any entity that generated/collected it in line with established norms.

Institutions shall develop data management and sharing plans at the proposal stage. These plans shall outline the strategies for collecting, managing, storing, accessing, sharing and use research data throughout the research project lifecycle. Approval from relevant approval bodies shall be obtained before the commencement of the research, ensuring that data-sharing practices align with ethical and legal standards.

Significant portion of health-related research/surveys is funded by private and foreign institutions, these funders shall require researchers to make their data available in national databases and repositories for reuse. Institutions engaged in health-related surveys/research shall establish and maintain up-to-date data-sharing plans. These institutional plans shalloutline the procedures and protocols for collecting, storing, accessing, sharing, and use of research/survey data.

11.0 Procedures for Accessing, Sharing and Using Individual-level/Personal Data

1) Any health worker or health institution that suspects or comes across epidemic-prone diseases that are regarded as reportable by health authorities shall immediately notify the concerned authorities.
2) All relevant institutions shall timely notify/report vital events information that are regarded as reportable by relevant health bodies.
3) Any holder of individual-level data has the responsibility to prevent illegal use, unauthorized access, data loss and modification.
4) Only individuals with a role and responsibility assigned to them by the health institution shall have access to individual-level data and shall have access to the data only for the purpose of care of the individual.
5) Any one owning individual-level data shall maintain privacy, and confidentiality and shall not share the data to any entity unless permitted by or in compliance with a specific law of the country.
6) Any health service recipient has the right to access or obtain his/her information stored in the health facility, which renders him/her health care.

7) The usage, sharing and publicizing individual-level data is only permissible for the health care of the individual and other aims permitted by the law of the country.

8) Unless by order of a court or required by the health professionals' ethics committee, the original copy of the individual-level record shall not be taken out of the health institution. If the data is kept or has backup in digital format or Electronic Medical Record (EMR), it is permissible to consent with the individual, in a de-identified manner and generally in a manner, which doesn't put the confidentiality of the data at stake.

9) Individual-level data shall be stored in a secured place, which does not allow entry to and access to data other than authorized individuals.

10) If a particular health facility is closed out, handed over or closed down, the data under its custody shall be transferred to a health facility which has replaced it or to the health administrative unit which has been overseeing it or has been reporting to.

11) The holder of individual data can share and permit its use apart from the purpose of health care to the individual if consent is obtained from the individual in writing and the purpose is properly stated.

12) Any data owner/source can share individual-level personal data with a third party or permit its use without the consent of the service recipient/individual under the following conditions:

    a. When sharing the data and use by a third party is required by legal enforcement bodies, or when the law requires it;
    b. Sharing the data to next of kin or caregiver of the individual in the interest of care of the individual and if an individual is not in a state or condition to consent:
    c. When the use or sharing of the data to a third party is necessary to prevent the public or the individual from any harm;
    d. When the use of the data or sharing with a third party is essential for the purpose of refunding from health insurance schemes;
    e. When required by the health care provider regulators and for court purposes and when ordered by the appropriate court and regulatory bodies;
    f. The use or sharing/disclosure of the data is deemed essential for public health.

11.1 Procedures for Accessing Data from the Ministry of Health Data Repository

To obtain data from the Ministry of Health data repository, the following steps shall be followed

Step 1: First, a formal data sharing/access request shall be submitted to the Ministry of Health's Director General's (DG) Office in writing, and the DG shall forward the request to the Assistant Commissioner of Health Services ACHS, Division of Health Information Management (DHIM).

Step 2: ACHS DHIM shall inform data requesters to submit detailed data-sharing requests by filling out the MoH's data request form. Then the ACHS DHIM shall evaluate the data request in accordance with the MoH's eligibility for data sharing/access and other relevant criteria and advise the DG accordingly.

Step 4: The DG shall approve or reject the data access/sharing request. Once the data request has been approved, the data sharing agreement template shall be filled out, signed, and stamped by the two parties (DG's Office and the data requesting institution/person.)

In case the data request is rejected, the data requester shall be duly notified within a reasonable time frame usually within five working days.

Step 5: An officially delegated individual will be authorized to access data based on the objectives of the data request. The ACHS DHIM shall be responsible for the overall data governance, and management and shall be accountable to the Director General. The DHIM unit will be responsible for the overall management of the national health and health-related data.

11.2 Procedures for Research-Generated Health Information

Research on health is conducted by individuals, institutions or organizations. It is intended to influence policy formulation, introduce new approaches to treatment or health management and to prove or disprove a hypothesis but all in all, to generally lead to betterment in health care. Efforts to utilize research findings have been minimal. Researchers conduct their research and there is nobody to encourage the use of the findings. For example, findings of research done as part of fulfilment of a PhD or Master's degree, are hardly used because they are not disseminated and the researchers do not know how to share this information.

11.3 Conducting Research or any Population-Based Survey on Health

Step 1: The researcher(s) shall write to the Uganda National Council for Science and Technology (UNCST), copied to the Uganda National Health Research Organization (UNHRO) of the Ministry of Health, indicating the following:

1. Study to be done
2. Reasons for conducting the study
3. Intended beneficiaries of the study
4. The geographical area that the study will cover
5. In case biological samples are to be taken, this shall be stated and the specific samples (blood, urine, spinal fluid, etc) and the study subjects indicated
6. Duration of the study

Step 2: Submission to the appropriate Institutional Review Board (IRB) shall be done.

Step 3: Following IRB approval, the researcher shall submit to the National Council for Science and Technology, the institution shall use its criteria to determine whether to give the authority or not.

Step 4: The National Council for Science and Technology shall give the response to the researcher(s) in writing, copied to the Uganda National Health Research Organization (UNHRO).

11.4 Data Access, Sharing and Use in Public Health Emergencies

During public health emergencies, such as pandemics or outbreaks, data access, sharing and use guidelines become even more critical to ensure effective response efforts while safeguarding individual rights and privacy. These are the procedures to be followed in Uganda.

a) Access to relevant health data during public health emergencies shall be facilitated promptly to support timely decision-making and response efforts.

b) Authorized government agencies, public health institutions, and designated healthcare providers shall have expedited access to necessary data for surveillance, monitoring, and epidemiological investigations.

c) Access requests shall undergo streamlined review processes to minimize delays while ensuring compliance with ethical and legal standards.

d) Data collected during public health emergencies shall be used solely for purposes related to outbreak detection, response planning, and intervention implementation.

e) Healthcare providers and public health officials shall adhere to established protocols and guidelines governing the use of emergency health data.

f) Data use shall be guided by principles of necessity, proportionality, and respect for individual privacy rights.

g) Measures shall be in place to ensure the accuracy, integrity, and confidentiality of data used for decision-making and policy formulation during emergencies.

h) Mechanisms for secure data sharing shall be established to facilitate real-time exchange of information while protecting sensitive data from unauthorized access or disclosure.

i) Data sharing agreements shall be in place to define the scope, purpose, and limitations of shared data, as well as to address issues related to data ownership, intellectual property rights, and confidentiality.

11.5 Secure and Ethical Cross-Border Data Transfers

When data needs to leave Uganda and be transferred to another country, additional guidance and precautions shall be implemented to ensure the security, integrity, and ethical handling of the data. Observe the following for such data transfers:

1) Shall conduct a thorough analysis of the legal and regulatory requirements in both the originating and receiving countries. Uganda's data transfer requests shall be in accordance with the Data Protection and Privacy Act 2019.
2) Ensure adherence to other national and international laws, treaties, and agreements governing cross-border data transfers.
3) Obtain necessary approvals from relevant regulatory bodies before initiating data transfers outside the country.
4) Data Sharing Agreements (DSAs) shall be signed between the originating and receiving countries.
5) Respect for personal or individual autonomy and rights of individuals.
6) Shall utilize secure data transfer methods like secure file transfer protocols (SFTP) and virtual private networks (VPNs) for data transmission.

12. 0 Data Management

This section provides a comprehensive overview of the utilization, analysis, publication, release, and retention/destruction of shared data.

The Ministry of Health, partners, and researchers will develop data management plans that encompass the following components:

a. **Data Description:** This element describes the following:
    i. Information to be gathered; for example, PII or non-PII.
    ii. Nature of the data (i.e., whether sensitive or restricted).
    iii. Scope, which defines the data elements that are accessible in a subset of tasks.
    iv. Scale, which is a description of measurements of the data to be produced.
b. **Existing Data Sets**: A common guide on how to assess existing data sets for purposes of clarity and determining whether the current data sets are adequate for gathering information about program indicators.

c. **Formats:** Specifications of "authorised or acceptable" formats to ensure processing, sharing, storage, and retrieval are more efficient Reference must be made in this plan to other guidelines on interoperability and security.
d. **Description and Standards of Metadata**: An in-depth description of data by means of elaborating on the method of creation, standards used, purpose, time and date of creation, and authorship.
e. **Storage and Backup:** A detailed description of how and where copies of data files will be stored to ensure confidentiality and safety. Further guidance on data storage shall be obtained from **Section 5.5** of The Uganda Health Data Protection, Privacy and Confidentiality Guidelines.
f. **Roles and Responsibilities Matrix**: A section of the data management plan elaborates on the roles and responsibilities of data controllers, data collectors, data processors, and data protection officers in the different stages of the data life cycle.
g. **Intellectual Property Rights (IPR) and data co-ownership**: A common agreement on which parties hold IPR to the data and other information created or collected and how to obtain permission to use or disseminate data.
h. **Archiving Selection and Retention Period**: A description of how the data will be selected for archiving, how long the data will be stored, and plans for the eventual transition or termination of data sharing in the future. Data retention, archival and disposal shall follow **Section 5.6** of The Uganda Health Data Protection, Privacy and Confidentiality Guidelines.
i. **Archiving and Preservation**: An outline of the procedures in place or envisioned for long-term archiving and preservation of the data, including succession plans for the data.
j. **Ethics and Privacy**: Provisions on how an informed consent process will be carried out while ensuring that personal information remains confidential and is only made available for secondary analysis as stipulated by legal processes. Managing of consent under this section shall follow the Consent Framework detailed in **Section 10** of The Uganda Health Data Protection, Privacy and Confidentiality Guidelines.
k. **Data Breach Management:** Non-compliance will be handled using the channels outlined under the governance structure. Personal data breach and incident management shall follow **Section 13.2** of The Uganda Health Data Protection, Privacy and Confidentiality Guidelines. The designated Data Protection Officer (DPO) and the Grievance Redressal

Officer (GRO) shall undertake monitoring compliance functions, and any matters concerning dispute resolution shall be channeled through the supervisory authority.

12.1 Measures for Confidentiality and Protection in Data Sharing

The steps that will be taken to ensure the confidentiality and protection of shared data are:

a. Technical standards
   In electronic data exchange, modern technologies that promote an integrated, open, and flexible digital health solution will be used. The choice of exchange/data sharing system shall be guided by fundamental elements in relation to its ability to integrate, its cost-effectiveness, sustainability, and the ease of use of infrastructure components. Technical standards shall follow Section 2 of the Uganda Health Information Exchange and Interoperability Guidelines.

b. Confidentiality and security
   To protect the privacy and security of health data, the following minimum requirements shall be implemented
   i. **Data anonymization:** Organizations and projects shall consider putting in place procedures to explore alternatives for using PII before sharing; for example, coding data, replacing original names or values with non-identifiable data, or removing unnecessary variables that may indicate individuals.
   ii. **Protect electronic transmission**: Procedures to protect data transiting between systems/persons shall be password protected and encrypted, and a receipt acknowledgement mechanism shall be implemented. The communication and connectivity protocols shall follow **Section 2.2** of The Uganda Health Information Exchange and Interoperability Guidelines.
   iii. **Data release procedures and purposes:** Provisions for protecting PII when releasing data shall be in line with data sharing principles and compliance with national and international jurisdictional laws and policies. The applicable principles are:

1. Data of public health importance shall be openly shared subject to respecting appropriately established restrictions and in accordance with international standards of ethical research conduct.
2. Sharable public health data will remain available within a minimum agreed time delay at a favorable cost, if any, for all users in support of equity to access.
3. Individuals who produce, share, and use data are stewards of the data and have the responsibility for ensuring authenticity and preserving the quality and integrity of the data, including respect for data sources, through maintenance of confidentiality/privacy by ensuring appropriate citation and acknowledgment of the original work and data repository.
4. Where necessary, data shall be labeled “sensitive” or “restricted” following proper justification and within clearly defined procedures. In any event, should sensitive or restrictive data be made available, it shall be on the least restrictive basis possible.

iv. **Data release procedures**—Recipient

Procedures for recipient individuals or partner organizations to protect PII shall be carried out under defined conditions stated as follows:

1. Have the recipient sign a confidentiality agreement.
2. Ask the recipient for documentation on security procedures, such as training, assessments, internal governance structures, and procedures for electronic and physical security controls.
3. Request the recipient to comply with the agreement to destroy information after the purpose of the data release has been fulfilled.
4. Confirm verification/evidence of access to the bare minimum amount of data and time needed to satisfy the purpose.

v. **Disposition of electronic-based information**

Provisions to securely dispose of information after the expiration of the minimum agreed period shall be put in place. This can be achieved by doing the following:

1. Eliminating e-mails sent or received containing PII
2. Sanitizing or destroying hard drives or mobile devices (including USB keys) that contain PII before the computers/mobile devices are reassigned to non-program staff members, sent offsite for repair, sold, or disposed of
3. Keeping records of documents destroyed
4. Asking partnering organizations to action items [i–ii] above

Overall, the disposal of health data shall follow **Section 5.6** of The Uganda Health Data Protection, Privacy and Confidentiality Guidelines.

12.2 Data use and sharing agreement

A data-sharing agreement is a formal contract that documents the data being shared and how the data can be used. Such an agreement serves two purposes. First, it protects the entity providing the data and ensures it will not be misused. Second, it prevents miscommunication on the part of the provider of the data and the entity receiving the data by ensuring that any questions about data use are discussed. Before any data are shared, the provider and data requester shall discuss data sharing and data use issues and develop a collaborative understanding that will be documented in an agreement. **Appendix I** provides a template that is customizable for institutions and agencies in need of sharing data under a legally binding framework.

12.3 Contents of the Agreement

The agreement between MoH and the party interested in accessing data must at least comprise but not be limited to the following components:

1. **Period of agreement** that clearly defines when the data controller will give the data or samples to the data requester how long the data requester will be able to use the material and what will happen to the data afterwards (i.e., deleted, destroyed, or returned).
2. **Intended use** of the data that states as specifically as possible how the data requester will use the data, including the studies that will be performed, questions that will be asked, and the expected outcomes. In addition, this section of the agreement shall

address whether or not the data requester can use the data to explore additional research questions without the provider's approval.

3. **Constraints on the use** of the data, which lists any restrictions on how the data/Biological Specimens or findings can be used, including whether or not the data requester is required to document how the data are used if the data requester can share, publish, or disseminate data/biological specimen findings and reports without prior approval from data controller, if the data requester can share, sell, or distribute data findings on any part of the database, and if the data requester publishes a report based on the data who the report will belong to.
4. **Data confidentiality** which describes the required processes that the data requester must use to ensure that data remains confidential. Because some data may contain information that can be linked to individuals, it is important to put safeguards in place to ensure that sensitive information remains private. Personal information shall remain confidential and not be disclosed verbally or in writing to an unauthorized third party by accident or otherwise.
5. **Data security** which describes the methods that the data requester must use to maintain data security. Hard copies of data shall be kept in a locked cabinet or room, and electronic copies shall be password-protected or kept on a secure disk. Biological Specimens shall be kept in a secure location protected from unauthorized persons. This section will note who at the data requester agency will have access to the data, how it will be protected, and what will happen to the data at the end of the sharing period.
6. **Methods of data sharing** that identify the way in which data will be transferred from the provider to the data requester (i.e., physically or electronically, encrypted or not).
7. **Financial costs** of data sharing, which clarifies who will cover the monetary costs of sharing the data. This may include transportation or postal costs.
8. **Termination of agreement** - If one of the agreements will not be fulfilled by the data requester, MoH has the full right to terminate the agreement with/without notice within 2 weeks.

13.0 Compliance and Guidelines Governance

1. The DPO of the PDPO together with the DPO of the Ministry of Health shall ensure adherence to these Guidelines and shall be responsible for compliance with all Uganda applicable laws in force.
2. All individuals and entities who are covered by these Guidelines must comply with its requirements and, where requested, demonstrate such compliance.
3. The compliance checklist developed (**Appendix II**) shall be used for the purpose of monitoring the compliance of stakeholders to these guidelines.
4. These Guidelines may be revised from time to time. A copy of these Guidelines together with any significant revisions shall be made publicly available through the Ministry of Health official communication channels like e-Library or the Knowledge Management Portal.

13.1 Non-Compliance and Repercussions with These Guidelines

Non-compliance with these guidelines may have several adverse consequences, including:

1. Compromised Data Integrity: Failure to adhere to data management standards may result in inaccuracies and inconsistencies in health data, undermining its reliability and usability.
2. Breach of Confidentiality: Improper handling of health data may lead to breaches of confidentiality and privacy, violating individuals' rights and confidentiality regulations.
3. Ineffective Data Sharing: Lack of adherence to standardized data sharing protocols may impede collaboration and data exchange efforts, hindering informed decision-making and research.
4. Legal Ramifications: Non-compliance with relevant legislation and regulations governing health data management may result in legal sanctions and penalties within the laws of Uganda.
5. Where any person or entity to whom these Guidelines is applicable is found to be in violation of any of its provisions, such a person or entity may not be permitted to participate in the national health care enterprise and further action shall be taken by the PDPO.

13.2 Liability, Penalties and Remedies

Shall follow the Data Protection and Privacy Act 2019 and any other relevant laws concerning penalties (e.g., administrative, financial, criminal) liabilities and judicial remedies.

13.3 Sale of Health Data

Sale or offer to sell health data by a person or an entity shall be prohibited. **Section 37** of the Data Protection and Privacy Act 2019 shall be followed in case of non-compliance.

14.0 Dissemination and Adoption of the Guidelines

The Uganda Health Data Access, Sharing and Use Guidelines shall be disseminated for adoption.

The dissemination and adoption of these guidelines shall happen at national, sub-national and community levels as guided by the MoH. Some of these methods are;

a. Presentation of the guidelines to stakeholders at all levels.
b. Posting of the guidelines on the Ministry of Health websites, WhatsApp groups, Radio etc for access by the stakeholders.
c. Organising quarterly workshops to sensitise stakeholders

15.0 Conclusion

Ensuring adoption and adherence to The Uganda Health Data Access, Sharing, and Use guidelines in a country requires a multi-faceted approach that combines education and training, research collaboration, enforcement, and monitoring. Here are some proposed effective strategies to encourage adoption and adherence to consider for inclusion in the guideline:

1. **Stakeholder Engagement and Collaboration:** Engage stakeholders from various sectors, including government agencies, healthcare providers, researchers, patient advocacy groups, and technology vendors. Involve them in the development of guidelines to foster a sense of ownership and collective responsibility. Regularly communicate and collaborate with stakeholders to address concerns, gather feedback, and ensure their active participation in implementing the guidelines.
2. **Education and Training:** Provide comprehensive education and training programs to all stakeholders, including healthcare professionals, researchers, data custodians, and administrators. Train them on the importance of health data access, sharing, and use, as well as the guidelines and policies in place. Offer ongoing training

opportunities to keep them informed about emerging best practices, legal requirements, and technological advancements.

3. **Clear communication and awareness campaigns:** Develop clear and accessible communication channels to disseminate information about the guidelines, their purpose, and their benefits. Launch awareness campaigns targeting healthcare professionals, patients, and the general public to increase understanding and promote a culture of responsible data access and sharing. Use various mediums, such as websites, newsletters, seminars, and social media, to reach a wide audience.
4. **Incentives and Recognition:** Offer incentives and recognition programs to motivate and reward individuals and organizations that demonstrate exemplary adherence to the guidelines. This can include public recognition, awards, funding opportunities, or access to exclusive resources. Incentives can encourage compliance and promote a positive environment for health data access, sharing, and use.
5. **Enforcement and Accountability:** Establish mechanisms to enforce compliance with the guidelines. This can involve conducting regular audits, assessments, and inspections to ensure adherence to data governance policies. Implement sanctions or penalties for non-compliance, such as fines, loss of privileges, or reputational consequences. Transparently communicate the consequences of non-compliance to deter violations and reinforce accountability.
6. **Technical Solutions and Infrastructure:** Develop or enhance technical solutions and infrastructure to facilitate secure health data access, sharing, and use. This can include implementing robust data security measures, standardized data formats, interoperability frameworks, and secure data-sharing platforms. Provide healthcare providers and researchers with user-friendly tools and technologies that comply with the guidelines and streamline data management processes.
7. **Monitoring and Evaluation:** Continuously monitor and evaluate the implementation of the guidelines to assess their effectiveness and identify areas for improvement. Collect feedback from stakeholders, conduct surveys, and analyze data to measure the level of adoption, identify challenges, and address gaps.

Regularly review and update the guidelines based on lessons learned and emerging trends.

8. **International Collaboration and Benchmarking:** Collaborate with other countries and international organizations to share best practices, and lessons learned, and benchmark against global standards. Learn from successful implementations in other jurisdictions and adapt relevant strategies to the local context. Engage in international discussions and initiatives to align guidelines with evolving global norms.

By combining these strategies, the country can promote a culture of adherence to The Health Data Access, Sharing, and Use Guidelines. It requires a holistic approach that addresses education, communication, incentives, enforcement, technological infrastructure, and ongoing evaluation to ensure sustained adoption and compliance.

Appendix 1: Generic Data Access, Sharing, and Use Agreement Template

[insert name of the department/unit]

DATA ACCESS, SHARING, AND USE AGREEMENT

For [insert study title and timeframe]

Background and Purpose

This Data Access, Sharing, and Use Agreement ("Agreement") is entered into between the [insert name of the department/unit], by and through its [insert sub-unit name, if any] and [insert name of the proprietor of the data] (also referred to herein as "Participating Entity"), individually referred to as "party" and collectively referred to as the "parties". This Agreement establishes the basic terms and conditions for the sharing, protection, and use of certain data as defined within the context of [insert survey name] supported by [insert formal name of protocol]. The [insert name of the department/unit] may release data to entities and partners with an approved Data Use Agreement (DUA) for purposes authorized by the Participating Entity. All persons with data access must sign this agreement outlining the terms and conditions for using the data. It also sets forth the expectations for each party's roles and responsibilities in this regard. Through this Agreement, the Participating Entity authorizes [insert sub-unit name] to use, store, process, and maintain the above-referenced data within the specified system as defined below and sets forth the expectations for each party's roles and responsibilities. A DUA is specific to the individual project, and all projects require annual review by [insert name of the department/unit] and a designated representative from the Participating Entity.

The [insert name of the department/unit] conducts a detailed review of every application for access to data and makes a determination on a case-by-case basis. Requests for confidential (identifiable) data will be granted only if the project meets the criteria, a valid rationale for the project not using de-identified information is provided, and final approval from the Participating Entity is obtained.

Approved applicants has to complete the data requestor information form (Table 1) and are held to the highest ethical standards and must agree to the stipulations detailed in this DUA.

Authorities

The [insert name of the department/unit] is authorized by the [insert the name of the act/law authorizing the department/unit] to maintain active surveillance of diseases through epidemiologic and laboratory investigations and data collection, analysis, and distribution and to participate with other countries in cooperative endeavours related to certain health care–related activities.

The Uganda Data Protection and Privacy Act 2019 governs the collection of health care, surveillance, historical, statistical, and research data to prevent or mitigate a serious and imminent threat to public health, public safety, or the life of the data subject or another individual. In addition, the Policy/Act provides protections as required by Uganda's law around transferring such data outside of Uganda.

Table 1: Data Requestor Information

Name	
Title	
Organization / Center / Division / Agency / Affiliation	
Mailing address	
Telephone number (including country code)	
Email address	
Contact person (if different from requestor)	
Contact person's telephone number (if different from requestor)	
Contact person's email address (if different from requestor)	
Does this application update a previous Data Use Agreement?	• Yes<br>• No<br>• Not applicable

Description of Parties and Key Points of Contact

The focus, scope, and goals of the data sharing set forth in this Agreement are aligned with efforts of the [insert name of the department/unit] and the Participating Entity for data use and access for the [insert data types] in Uganda. Accordingly, the parties, systems, and points of contact are provided below.

The Participating Entity in Uganda represents and warrants that it has the authority to enter into this Agreement as contemplated by this Agreement and that doing so will not violate any law or regulation applicable to the Participating Entity in Uganda or any agreement or arrangement to which the Participating Entity in Uganda is a party.

Each party will identify an individual to serve as the focal point to coordinate communication and activities related to sharing [insert data types] data and the coordination of the terms of this Agreement. Appendix A lists the named individuals designated as Key Points of Contact for performance of the terms of this Agreement. As part of this Agreement, both parties agree to notify each other within five (5) days when a change in Key Points of Contact occurs.

Project Summary

Project title	
Purpose of project	
Brief description of project (e.g., background, rationale, objectives, and methods)	
Duration of project	• 0–3 months<br>• 4–6 months<br>• 7–9 months<br>• 10–12 months<br>• ≥13 months
Is data custodian* for project same as requester?<br><br>If no, please indicate the name of the data custodian and their relationship to the requestor's organization.	• Yes<br>• No (explain)

* The data custodian is responsible for observance of all conditions of use and for establishment and maintenance of physical and electronic security arrangements to prevent unauthorized use. This individual must have the legal authority to keep the information confidential and maintain confidentiality. If the custodian is changed, the organization must promptly notify the [insert name of the department/unit] and the Participating Entity within five (5) days of change.

Supporting Documentation for Data Access	
Is release letter or letter of support from the participating entity attached?<br><br>If no, please explain why not.	• Yes<br>• No (explain)
Is release letter or letter of support from another agency or partner needed?<br><br>If yes, is letter attached?	• Yes<br>• No<br>• Not applicable<br>• Yes

	• No
Has project been approved by the appropriate Human Subjects Review Board(s) (e.g., Institutional Review Board)?<br><br>If yes, please provide documentation and details of review and approval.<br><br>If no, please explain why not.	• Yes (explain)<br>• No (explain)

Data Files Requesting and Specifications		
File name		
Format	• CSV<br>• Excel<br>• Access	• SAS<br>• Other—please specify:
Timeframe for data file requested (e.g., data range in month/day/year format)		
Geographic area for data file requested		
Variables requested		
If requesting confidential (non-anonymized, not de-identified, sensitive) data, please provide a few sentences of a rational for needing these data.		
File name		
Format	• CSV<br>• Excel<br>• Access	• SAS<br>• Other—please specify:
Timeframe for data file requested (e.g., data range in month/day/year format)		
Geographic area for data file requested		

Variables requested	
If requesting confidential (non-anonymized, not de-identified, sensitive) data, please provide a few sentences of a rational for needing these data.	
File name	
Format	• CSV<br>• Excel<br>• Access<br>• SAS<br>• Other—please specify:
Timeframe for data file requested (e.g., data range in month/day/year format)	
Geographic area for data file requested	
Variables requested	
If requesting confidential (non-anonymized, not de-identified, sensitive) data, please provide a few sentences of a rational for needing these data.	

Terms of Agreement

Generally, the parties will adhere to a shared vision for maintaining the integrity of the [insert data types] and respecting that proprietorship of the information remains with the Participating Entity in Uganda.

The following outlines the necessary activities, roles, and responsibilities to ensure the successful management and availability of [insert data types].

Data

No data processed, stored by, or submitted to [insert name of the department/unit] for that purpose under this Agreement by the Participating Entity in Uganda will include personally identifiable information (PII) (e.g., participant names, addresses, etc.) unless all parties agree to release PII.

MAKE NOTE OF EXCEPTIONS

Consent

As per the survey protocol under which the information was originally gathered, [insert name of the department/unit] accepts data submitted by the Participating Entity in Uganda with the understanding that all individual participants have agreed to participate in the source survey and, as such, have provided their informed, voluntary consent consistent with applicable laws, regulations, and policies.

IF DIRECTLY COLLECTING, WILL NEED TO DEVELOP CONSENT FORM IN LINE WITH COUNTRY'S DATA PROTECTION ACT

Data Submission

As technology and connectivity vary, [insert name of the department/unit] will collaborate with the Participating Entity in Uganda to determine the best approach for the secure transfer of [insert data types]. It is anticipated that a technology such as that provided by [insert name of the department/unit]'s secure file transfer protocol or similar service will be utilized. [Insert name of the department/unit] will work with the Participating Entity prior to data transfer to determine specific protocols, credentials, and transmission requirements/alternatives. In no case will [insert name of the department/unit] accept data via insecure transfer methods, as defined by [insert name of the department/unit], including, but not limited to Dropbox, Google Drive, or email. Consistent with the laws of Uganda, _____ will accept responsibility for the security of the information provided only after it has been received via the agreed-upon method.

Data Quality, Error Notification, and Correction

[Insert name of the department/unit] will use its best efforts to provide feedback within a reasonable period to the Participating Entity in Uganda to resolve identified data incompleteness, inaccuracies, and inconsistencies. The Participating Entity in Uganda will research and resolve the issues and provide corrections and clarifications to [insert name of the department/unit] through the processes defined under Data Submission. Also, should the Participating Entity proactively identify any errors or have concerns about the data in use, they are asked to contact the [insert name of the department/unit] programmatic point of contact for the relevant survey and data steward/owner as defined in Appendix A.

Data Security, Storage, and Transmission

[Insert name of the department/unit] shall establish appropriate administrative, technical, procedural, and physical safeguards in accordance with Uganda's data protection act; e.g., Republic of Uganda No. 4 of 2019: Data Protection and Privacy Act], [insert name of the department/unit] security policies, and the [insert name of the national standard-setting authority, e.g., National Institute of Standards and Technology] Risk Management Framework, including formal System Security Authorization for any data or health information system that [insert name of the department/unit] creates in Uganda. Once data is received and accepted by [insert name of the department/unit] using the submission process outlined above, [insert name of the department/unit] accepts responsibility for the secure storage, transmission, and use of the information as outlined herein to the extent required by the laws of Uganda. Except as required by the laws of Uganda and as set forth in this Agreement, data submitted by the Participating Entity in Uganda residing in the [insert name of the department/unit] data system will not be physically removed or transmitted outside the established system security boundary without first obtaining prior written approval from the Participating Entity in Uganda. Any such approval must include the scope, purpose, and protections specific to the removal action.

Data Use

The following additional terms and uses apply to [insert name of the department/unit] under the terms of this Agreement:

1. [Insert name of the department/unit]'s use includes the right to translate the [insert data types] into any language.
2. Except as required by the laws of Uganda and as set forth in this Agreement, the [insert name of the department/unit] will not share with or export to other centers and offices within or organizations external to [insert name of the department/unit] any data without the express written permission of the Participating Entity.
3. Except as required by the laws of Uganda and as set forth in this Agreement, [insert name of the department/unit] will not release any data and/or results to the public domain without prior written agreement from the Participating Entity.
4. As part of accessing and using [insert data types], [insert name of the department/unit] may develop reports and analyses focused on the [insert response type] in Uganda. [Insert name of the department/unit] may invite collaborators and co-authors from other centers and offices within [insert name of the department/unit] and the participating entities to design, analyze, and co-author such reports and analyses. Also, [insert name of the department/unit] will share developed reports and analyses with the Participating Entity for comment before publication.
5. Except as may be required by the laws of Uganda, and as set forth in this Agreement, [insert name of the department/unit] agrees it will not present any findings or analyses arising from the use of the submitted data in any public forum, or prepare or submit such findings or analyses for publication, without first providing any such findings or analyses to the Participating Entity that provided the data on which these findings or analyses are based for such Participating Entity's review to ensure that the findings or analyses do not contain any confidential or proprietary information.
6. To the extent any analyses, reports, or publications are developed using data submitted by the participating entity, [insert name of the department/unit] will acknowledge the contribution of data by the Participating Entity in Uganda.
7. To the extent required by the laws of Uganda and consistent with this Agreement, [insert name of the department/unit] will use all reasonable efforts to maintain the confidentiality, integrity, and ensure the security of the [insert data types] within the parameters defined herein. However, there shall be no obligation of confidentiality where: (i) the information is publicly available, or becomes publicly available, other than by action or omission of the receiving party; or (ii) the information was already known to the receiving party (as evidenced by its written records) prior to its receipt; or (iii) the information was received from a third party not in breach of an obligation of confidentiality owed to the other party.

Will the study results be used for publication and/or presentation?<br><br>If yes, please provide publication and presentation information and intended timing of this information.	• Yes (explain)<br>• No<br>• Not applicable
Have at least two co-authors / collaborators from the [insert name of Participating Entity] been identified and notified of this project?	• Yes

If no, please explain why not.	• No (explain)
Is the requested data needed for work being performed under contract / grant / cooperative agreement with the [insert name of the department/unit]?<br><br>If yes, please provide the contract / grant / cooperative agreement number and point of contact information.	• Yes (explain)<br>• No<br>• Not applicable

Data Security and Confidentiality

The release of information that may lead to the identification of individuals or be traced back to an individual record is prohibited. However, statistical and research results based on the data provided by the [insert name of the department/unit] and the Participating Entity pursuant to this DUA may be released. Any person(s) who access, disclose, or use confidential data in a manner or for a purpose not authorized by this Agreement may be subject to civil and criminal sanctions contained in applicable federal and state statues.

Only the listed data custodian or authorized users listed on this Agreement may access data.

Describe where data will be stored and how data will be accessed by authorized users (e.g., stored in a locked filing cabinet, password protected, residing on a physically secure server, zipped [compressed], 256-bit encrypted, on a cloud system, on an encrypted external hard drive).

Data Access Use by Others

Except as may be required by the laws of Uganda, and as set forth in this Agreement, access to data submitted by the Participating Entity in Uganda in conjunction with the Participating Entity shall be restricted to authorized [insert name of the department/unit] employees and contractors who require such access in order to perform their duties in accordance with the uses of the information as authorized in this Agreement. Such personnel shall be advised of (1) the confidential nature of the information, (2) safeguards required to protect the information, and (3) any administrative, civil, and criminal penalties for noncompliance contained in applicable laws of Uganda

The parties signing this agreement assert that they are fully aware of the policies for handling confidential (non-anonymized, sensitive) data and agree to protect these data in accordance with these policies. The parties agree not to share any confidential data with any person or other [insert name of the department/unit] employee/contractor unless that person has co-signed this Agreement.

The parties signing this Agreement agree to use the requested data files solely for the project described above and agree to the above-listed data access conditions and storage requirements. The parties also agree that any information or aggregate data derived from confidential data to be disseminated or published must be reviewed by the designated person(s) within the [insert name of the department/unit] or the Participating Entity. Data will only be disseminated and published in aggregate, summary form so no one individual is identified from the data. The parties also agree that they will not disclose or publish any results based on the data without a review and approval from the [insert name of the department/unit] and the Participating Entity.

Please identify the individuals who will have access to or be using the data, describe their role and work they will perform in the project, and have them sign the DUA.

Your signature below says that you have read and agree to comply with the terms of this DUA as well as with the provisions set out on this form. It is your responsibility as a user of the data to protect the confidentiality of the data and to prevent unauthorized use of or access to it.

Requester name		
Signature		Date
Printed name		
signature		Date
Description / role in project		
Printed name		
signature		Date
Description/role in project		
Printed name		

Signature	Date
Description / role in project	
Printed name	
Signature	Date
Description / role in project	
Printed name	
Signature	Date
Description / role in project	

Data Destruction

Consistent with Uganda laws, applicants must make provisions for the destruction of records at the conclusion of their project, or when the data are no longer required. Maintaining the privacy of the individuals whose personal information is included in the data is required to preserve the integrity of the data sharing process.

Data that have been shared with the [insert name of the department/unit] consistent with this agreement will be archived, stored, protected, or disposed of in accordance with relevant Uganda requirements. In the event the [insert data types] reach the end of their active lifecycle, they will be retired in compliance with formal system retirement, data destruction, and records management requirements in accordance with Uganda law, agency policy, applicable System Security Authorization, and [insert name of the national standard authority]. All data not retained as per Uganda management requirements will be destroyed using [insert name of designated authority; e.g., Ministry of Information Communication Technology and National Guidance]–authorized physical and/or electronic methods applicable to sensitive information.

Upon written request and to the extent possible, a copy of all [insert data types] will be created and returned to the Participating Entity prior to the above removal. The specifics (e.g., media, format, transport method) of this action will be determined by joint agreement between the parties when/if

needed, but must minimally comply with all relevant [insert name of the department/unit] information security and records management controls.

> Please detail below the manner and timeline for data destruction according to requesting agency's / institution's / organization's policies. If following a data destruction policy set by the requesting agency / institution / organization, please attach that policy to this DUA.

Privacy Questions and Incident Response

Should the Participating Entity in Uganda have [insert data types] privacy-related questions, the need to request privacy or security-related information, or the need to notify [insert name of the department/unit], the data steward, or Ministry of Health with privacy and security-related concerns or a potential identified data breach, the Participating Entity should contact the [insert name of the department/unit] Point of Contact as provided by [insert name of the department/unit] protocol. If necessary, general inquiries may also be made to the Points of Contact listed in Appendix A.

In the event of a suspected incident (e.g., loss, theft, compromise) affecting the security of data provided by the Participating Entity in Uganda, the [insert name of the department/unit] director will ensure that formal notice is provided to [insert name of the department/unit] Cybersecurity Program Office and the Participating Entity within one (1) hour of event identification. Cybersecurity Program Office personnel, with full assistance from the [insert name of the department/unit] team, will then follow the established agency process for formal incident investigation, remediation, response, and communication as appropriate to all affected parties.

Agreement Term and Termination

This Agreement takes effect upon its signature by both parties and does not have a predefined end date but may be terminated by either party with sixty (60) days advance written notice to the other party.

The rights and obligations of the parties as set forth in this Agreement that are intended by their nature to survive its expiration or earlier termination shall survive indefinitely. This includes, but is expressly not limited to, the obligations of security, confidentiality, and usage of submitted data by [insert name of the department/unit].

This Agreement sets forth the entire agreement of the parties with respect to the subject matter hereof. In the event that any portion of this Agreement is held to be invalid for any reason, the remainder of this Agreement will remain in full force and effect.

The terms of this Agreement can be changed only by a written modification to the Agreement by the agency signatories (or their designated representatives) to this Agreement or by the parties adopting a new agreement in place of this Agreement. The [insert name of the department/unit] Information System Security Officer will be included in coordinating adoption of new terms.

Signatures and Concurrence

On behalf of both parties, the undersigned individuals hereby attest that they are authorized to enter into this Agreement and agree to all the terms specified herein.			
Signature		Date	
Printed name			
Title	Director/head]		
Signature		Date	
Printed name			
Title	[Participating Entity]		
Signature		Date	
Printed name			
Title	[Data Steward or Designee]		
Signature		Date	
Printed name			
Title	[Data System Owner/Designee]		

Appendix A – Key Points of Contacts

Role	Name	Title	Affiliation	Contact Information (Phone with Country Code and Email)
Data Steward (or designee)				
Data System Owner (or designee)				

[Participating Entity] in Uganda				
[Department / Unit] Programmatic Point of Contact				

Appendix 2: Health Data Access, Sharing, and Use Compliance Checklist

1. Legal and Regulatory Compliance

- **Data Protection and Privacy Act 2019 Compliance**: Ensure all data access and sharing practices comply with the Data Protection and Privacy Act 2019.
  - Privacy Rule
  - Security Rule
  - Breach Notification Rule
- **Country-Specific Guidelines**: Verify adherence to any relevant country-specific health data regulations.

2. Data Governance

- **Data Ownership**: Clearly define data ownership and stewardship responsibilities.
- **Data Classification**: Classify data according to sensitivity and confidentiality.
- **Data Access Policies**: Develop and implement data access policies.
- **Data Use Policies**: Develop and implement data use policies.

3. Data Security

- **Access Controls**: Implement robust access control mechanisms.
  - Role-based access
  - Multi-factor authentication
- **Encryption**: Encrypt data in transit and at rest.
- **Security Audits**: Conduct regular security audits and assessments.
- **Incident Response Plan**: Develop and maintain an incident response plan.

4. Data Sharing

- **Consent Management**: Ensure proper consent is obtained and documented for data sharing.
- **Data Sharing Agreements**: Establish and enforce data sharing agreements with third parties.
- **Data Minimization**: Share only the minimum necessary data.
- **Third-Party Compliance**: Ensure third parties comply with all relevant regulations and standards.

5. Data Use

- **Purpose Limitation**: Use data only for the specified purposes for which consent was obtained.
- **Anonymization/De-identification**: Anonymize or de-identify data when possible to minimize risk.
- **Data Quality and Integrity**: Ensure data quality and integrity are maintained.
- **Monitoring and Auditing**: Continuously monitor and audit data use practices.

6. Patient Rights

- **Right to Access**: Facilitate patients' right to access their own health data.
- **Right to Correction**: Allow patients to request corrections to their data.
- **Right to Deletion**: Ensure compliance with requests for data deletion where applicable.
- **Right to Information**: Provide clear information about data use, sharing, and protection practices.

7. Training and Awareness

- **Employee Training**: Conduct regular training on data protection and privacy policies.
- **Awareness Programs**: Implement programs to raise awareness about data privacy and security.

8. Documentation and Record Keeping

- **Policy Documentation**: Maintain up-to-date documentation of all relevant policies and procedures.
- **Access Logs**: Keep detailed logs of data access and sharing activities.
- **Compliance Records**: Retain records of compliance with regulatory requirements.

9. Risk Management

- **Risk Assessments**: Conduct regular risk assessments to identify and mitigate potential risks.
- **Mitigation Plans**: Develop and implement risk mitigation plans.
- **Continuous Improvement**: Continuously improve data protection measures based on risk assessments and audit findings.

10. Technology and Infrastructure

- **Secure Systems**: Use secure systems and technologies for data storage and processing.
- **Backup and Recovery**: Implement robust data backup and recovery solutions.
- **Regular Updates**: Ensure systems and software are regularly updated and patched.

11. Communication and Transparency

- **Transparency**: Be transparent about data access, sharing, and use practices.
- **Stakeholder Engagement**: Engage with stakeholders (e.g., patients, healthcare providers) to ensure understanding and trust.

12. Compliance Reporting

- **Internal Reporting**: Develop internal reporting mechanisms for compliance issues.
- **External Reporting**: Ensure timely reporting to regulatory bodies as required.

REPUBLIC OF UGANDA
MINISTRY OF HEALTH

GUIDELINES FOR THE INTRODUCTION OF DIGITAL HEALTH SOLUTIONS AND INNOVATIONS IN UGANDA

JANUARY 2023

Table of Contents

Foreword iv

Preface v

Acknowledgement vi

Abbreviations vii

Definition of Key Terms viii

1.0 Introduction 2

1.1 Context 2

1.1.1 The Uganda Health Systems Structure 2

1.1.2 Ministry of Health Governance and Management Structures 4

1.1.3 Data Flow Within the Uganda Healthcare System 5

1.2 Situation Analysis 6

1.3 Purpose of Document 7

1.4 Goal 7

1.5 General Objective 7

1.5.1 Specific Objectives 7

1.6 Scope 8

1.7 Target Audience 8

1.8 Methodology 8

1.9 Underpinning Guiding Principles 9

1.10 Policy, Reference Guidelines and International Standards 9

1.11 Revision and Updates 9

2.0 The Digital Health Solutions and Innovation Approval Process 9

2.1 Registration of the Digital Health Solution and Innovations 10

2.2 Presentation to the Digital Health Subcommittee 10

2.2.1 Field Visit/Proof of Concept 11

2.3 Presentation to the Health Information Innovation and Research Technical Working Group (HIIRE TWG) 12

2.4 Presentation to the Senior Management Committee (SMC) 13

2.5 Presentation to the Health Policy Advisory Committee (HPAC) 13

2.6 Presentation to Top Management Committee 14

3.0 General Considerations 15

4.0 Digital Health System Implementation Framework 15

5.0 Monitoring, Evaluation and Dissemination 19

5.1 Dissemination and Adoption of the Guidelines 19

Appendix 1: Digital Health Systems Assessment Criteria 20
7. 22

Foreword

Ministry of Health is committed to improving the application of digital health technologies to facilitate the attainment of its overall objective of delivering high-quality health services to all citizens in Uganda. This is in line with the call of Uganda Vision 2040 and the National Development Plan (NDP) III which require sectors to adopt Information Communication Technologies (ICTs) to Optimise service delivery.

To further the application of ICTs within the health sector, the Ministry of Health (MoH) developed the Uganda Health Information and Digital Health Strategic Plan 2020/21-2024/25 which promotes digital health innovations and the application of digital health solutions within the health sector.

However, the health sector in Uganda is characterised by a fragmented landscape of digital health solutions, innovations and pilot projects resulting in duplication of efforts and resources, and digital health systems that are never scaled.

These guidelines standardise introducing digital health solutions and innovations in the Ugandan health sector. The overall purpose is to realise interoperable, secure digital health solutions that adhere to existing legal and guiding frameworks. This will also result in digital health solutions and innovations that can be integrated into national health systems to promote scalability and sustainability. This will deliver a safer, better connected, and more sustainable healthcare system.

....

Dr. Henry G. Mwebesa

Director General, Health Services

Preface

Digital Health is a vital component of a health system and is a critical precursor for optimising health service delivery to achieve Universal Health Coverage (UHC). The Ministry of Health is mandated to provide high-quality and accessible health services to all people in Uganda.

The purpose of these guidelines is to standardise the implementation of digital health solutions and innovations to realise interoperable, secure digital solutions that adhere to the existing legal and guiding frameworks in Uganda.

In the past ten years, between 2015 and to date, various actors have supported numerous digital health innovations and solutions, mostly independent of the national health information system, resulting in the duplication of, and limited integration of the various health information platforms, which these guidelines aim to rectify going forward. I call upon all actors to follow these guidelines as new digital health solutions and innovations are being introduced into the country to avoid further fragmentation of systems.

....

Dr. Sarah Byakika

Commissioner Health Services

Department of Planning, Financing and Policy

Acknowledgement

The Ministry of Health expresses its profound gratitude to all departments and programs, the Health Information Innovation and Research Technical Working Group (HIIRE TWG) and the Digital Health Subcommittee who contributed technical inputs leading to the successful completion of this document. Special appreciation goes to the Division of Health Information Management (DHIM) and the Information Communication Technology (ICT) Section for the overall guidance to ensure that the guidelines are aligned with the Uganda Health Information and Digital Health Strategic Plan 2020/21-2024/25.

I acknowledge and thank all development and implementing partners that provided technical support for this process, specifically UNICEF, CDC, MUSPH/METS, USAID and HISP. DHIM is grateful for all the support, sacrifice and contributions that have been invested in its successful development.

Finally, the Ministry of Health is grateful to the Ministry of Information Communication Technology and National Guidance (MoICT&NG), National Information Technology Authority Uganda (NITA-U), and all those institutions and individuals who have not been specifically mentioned above, but who directly or indirectly contributed to the successful development and finalisation of these Guidelines for the Introduction of Digital Health Solutions and Innovations in Uganda.

....

Mr. Paul Mbaka

Assistant Commissioner Health Services

Health Information Management

Abbreviations

DHTR	Digital Health Tools Registry
DHIS2	District Health Information System
DHS	Digital Health Subcommittee
HC	Health Centre
HIIRE	Health Information Innovation and Research
HMIS	Health Management Information System
HSDP	Health Sector Development Plan
HSS	Health Systems Strengthening
ICT	Information Communication Technology
NDP	National Development Plan
PFP	Private for Profit
PNFP	Private Not for Profit
TWG	Technical Working Group
WHO	World Health Organization

Definition of Key Terms

Digital Health – Digital health is the systematic application of information and communications technologies, computer science, and data to support informed decision-making by individuals, the health workforce, healthcare organisations, and health systems, to strengthen resilience to disease and improve health and wellness.

Digital Health EcoSystem – The combined set of digital health components representing the enabling environment, foundational architecture and Information Communication Technology capabilities available in a given context or country.

Digital Health Intervention – A discrete technology functionality – or capability – designed to achieve a specific objective addressing a health system's challenges.

Digital Health Application – The software, information and communications technology (ICT) systems or communication channels that deliver or execute the digital health intervention and health content.

Electronic Medical Record (EMR) – An electronic record of health-related information on an individual that can be created, gathered, managed and consulted by authorised clinicians and staff within one healthcare organisation

Electronic Health Record (EHR) – An electronic record of health-related information on an individual that conforms to nationally recognised interoperability standards and that can be created, gathered, managed and consulted by authorised clinicians and staff across more than one healthcare organization.

Health Information – Health information is any personal information, whether oral or recorded in any form or medium, that; is created or received by a health care provider, health planner, public health authority, employer or the employer's agent, life insurer, school or university, or health care clearinghouse; and relates to the past, present, or future physical or mental health or condition of an individual, the provision of health care to an individual, or the past, present, or future payment for the provision of health care to an individual.

Health Information Management - A process which involves the collection, analysis, storing, protecting and ensuring of the quality of client/patient health information. This

information can be either paper-based, a combination of paper and digital (hybrid) or increasingly, a fully electronic health record.

Health Information System (HIS) – Any organised effort to systematically collect, maintain and disseminate data relevant to the performance of a health system or any of its parts.

Health System – The complete universe of all activities that serve to maintain or improve the health and longevity of a population in a specific geography.

Interoperability – The ability of different information technology systems and software applications to communicate, exchange data, and use the information that has been exchanged. The integration allows two applications to exchange information, interoperability allows many.

Personal Health Record (PHR) – An electronic record of health-related information on an individual that conforms to nationally recognised interoperability standards and that can be drawn from multiple sources while being managed, shared and controlled by the individual record owner.

User Department or Programme - The official Ministry of Health departments and programmes as per the documented Ministry of Health macrostructure.

1.0 Introduction

Health Information System is one of the core building blocks of any health system according to the World Health Organization (WHO). The daily business of health relies on information and communication and, increasingly, on the technologies that enable it, at every health service delivery level world over. In an increasingly digital world, spurred by technological advances, economic investment, and social and cultural changes, there is growing recognition that inevitably the health sector ought to integrate Information Communication Technology (ICT) into its operations. The Ugandan healthcare system, through its ongoing health sector reforms, aims to improve health outcomes. As part of these reforms, the Ministry of Health (MoH) developed and is implementing the Ministry of Health Strategic Plan 2020/2021-2024/2025. This was to address the key challenges facing Uganda's health system, set out priorities and key areas on which to focus health investment in the medium term, for both public and private partners, to optimally contribute to the attainment of both the health sector goals and the national goals as outlined in the National Development Plan III. The Ministry of Health recognises the potential of ICTs in transforming healthcare delivery by enabling information access and supporting healthcare operations, management, and decision-making. In light of the above, the Uganda Health Information and Digital Health Strategic Plan 2020/21-2024/25 with a vision of A health sector in Uganda driven by evidence and leveraging digital health to improve efficiency in service delivery.

1.1 Context

1.1.1 The Uganda Health Systems Structure

Uganda has an organised national health system and health delivery in place within the strategic framework and focus. The national health system comprises both the private and public sectors. The private health sector comprises Private Not for Profit (PNFP), and Private for Profit (PFPs). The public sector includes government health facilities and health services departments of different ministries. Some of the Ministry of Health functions have been delegated to national autonomous institutions like the National Drug Authority.

Health services delivery is decentralised within national, districts and health sub-districts. The lowest level of healthcare delivery is at the community level with volunteers in villages facilitating health promotion, service delivery, community participation, and empowerment. These are preceded by Health Centre IIs providing a first level of interaction between the

formal health sector and communities. These provide preventive, promotive, and curative services. The next level is the Health Centre IIIs which provide in-patient, simple diagnostic, and maternal health services. The next levels are Health Centre IVs and general hospitals, which provide broad services such as surgeries and blood transfusions, research, and training. The regional referral hospitals that are next in the hierarchy provide a higher level with more specialised clinical services and involve teaching and research. These are followed by national referral hospitals which are most comprehensive as they provide the highest level of specialist services in addition to all the other clinical services.

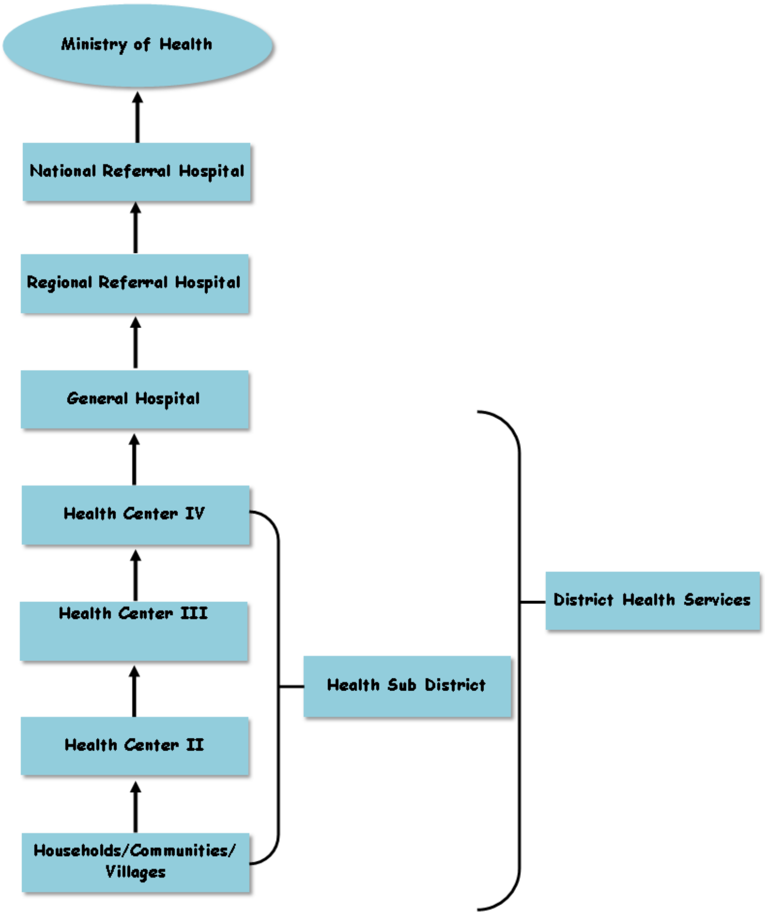

Figure 1: The Uganda Health Care System Structure

1.1.2 Ministry of Health Governance and Management Structures

The Ministry of Health governance structures derive their mandates from the corresponding policies and laws of Uganda. The health governance structures at the national and MoH include:

At the national level

- Cabinet: The cabinet is responsible for the overall leadership in the health sector.
- Parliament: The Parliamentary Health Committee provides overall oversight in the health sector.

At the Ministry of Health level, the structures include the Senior Top Management Committee (STMC), Top Management Committee (TMC), Health Policy Advisory Committee (HPAC), Senior Management Committee (SMC), Technical Working Groups (TWGs) and Departments.

Figure 2 outlines the strategic roles, structures and composition of the MoH governance and management structures. Further details can be obtained from the Implementation Guidelines for the Ministry of Health Governance and Management Structures, 2022.

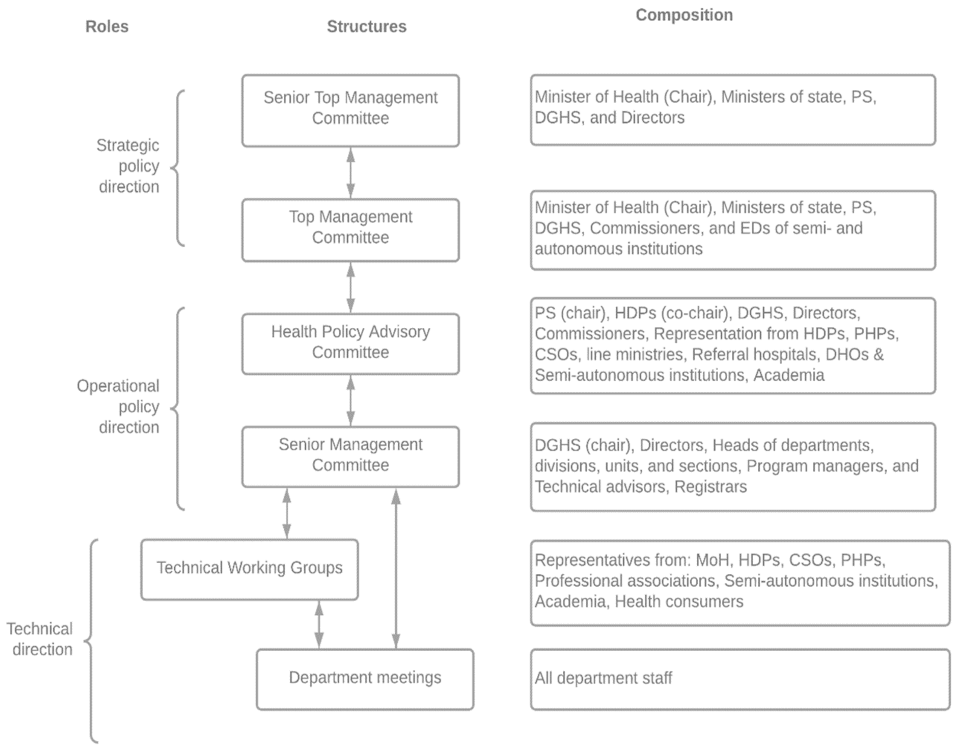

Figure 1: The MoH Governance and Management Structures[1]

The individual departments are responsible for the implementation of interventions within their respective technical areas. The departments are further broken down into divisions and divisions into units. A breakdown of the various departments within MoH is detailed in **Figure 2**.

[1] Implementation Guidelines for the Ministry of Health Governance and Management Structures, 2022.

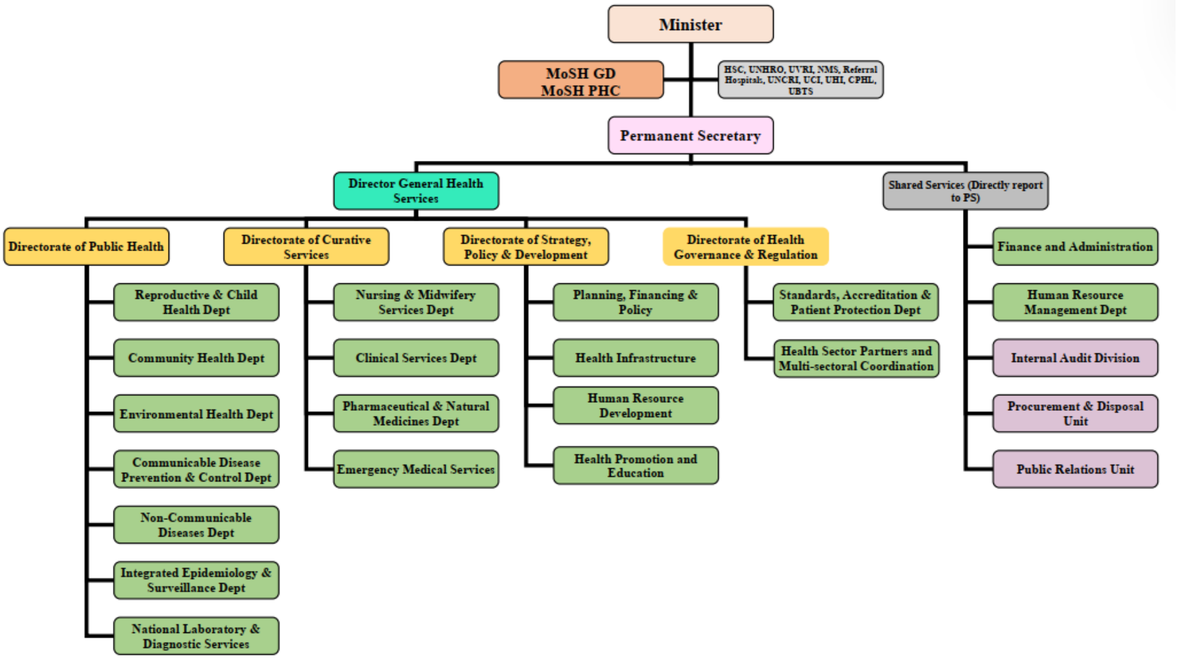

Figure 2: The Ministry of Health Macro Structure[2]

1.1.3 Data Flow Within the Uganda Healthcare System

All decisions at every level of healthcare service delivery are premised on data generated within the health sector in addition to other related sectors. As such, there is a need for data sharing across healthcare delivery at different levels for purposes of planning and decision-making. In Uganda, all health facilities must report a set of standard data defined within the national Health Management Information System (HMIS) to the Ministry of Health at various predetermined intervals (weekly, monthly, quarterly, and annually) to facilitate planning and policy formulation at the national level. Data to be collected is defined based on the health information needs of the country and it flows from the very first healthcare delivery level (community level) (***see Figure 1***) through the different subsequent levels and finally to the national level (*Ministry of Health*). Information technology as an enabler for health data management has been adopted to support the various data handling processes as shown in ***Figure 3***.

[2] Ministry of Health Strategic Plan 2020/21-2024/25

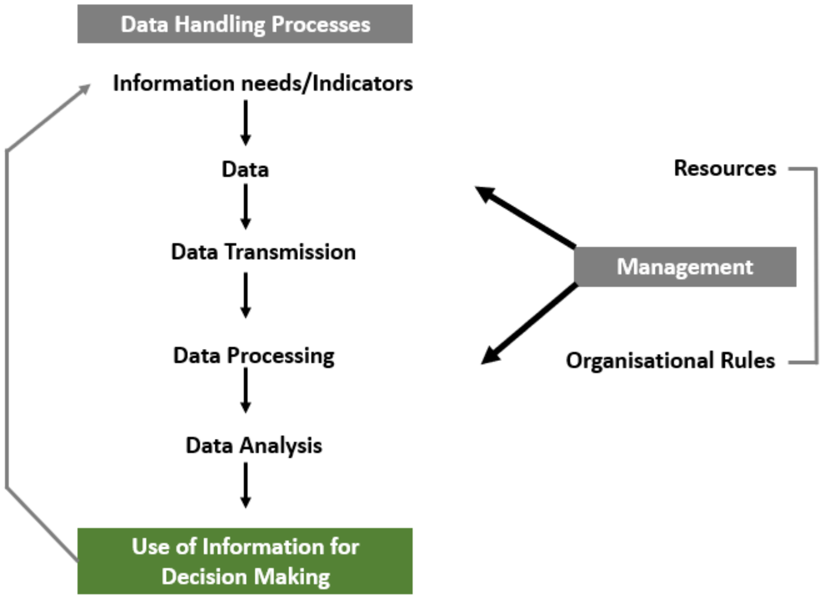

Figure 3: Data Handling Processes within the Uganda Healthcare System

For transmission, processing and analysis, Uganda adopted the District Health Information Software (DHIS2) to power its electronic Health Management Information System (eHMIS). Digital solutions implemented at the data collection stage are required to transmit the defined HMIS data set to eHMIS/DHIS2.

1.2 Situation Analysis

Digital health solutions and innovations are increasingly being adopted in Uganda to improve the quality of patient care, health data management and health program management. While international donors and health agencies have increased investment in ICT to support Health Systems Strengthening (HSS) in recent years, these investments have often resulted in a proliferation of pilot efforts rather than efforts to scale projects. Digital health systems and applications are often developed and implemented ad hoc, to meet the immediate requirements of specific activities or projects, with a limited focus on scalability. As a result, solutions that are successful in one locale are often unable to be integrated with other data systems or adopted by other, similar programs. The majority of the innovations have focused on the adoption of new products or services by individuals and their market-driven diffusion to the population at large. These innovations have typically emerged from negotiations between diverse stakeholders who compete to impose or at least prioritise their preferred version of that innovation. Thus, while many digital health interventions have succeeded in terms of adoption by a substantial number of end users, they have generally failed to gain the level of *acceptance* required for their integration into national health systems that would promote

sustainability and population-wide application. For example, by 2010, there were over 50 eHealth innovations in Uganda by almost as many donors, a situation that has been referred to as" *Pilotitis*" which is the act of continuously pursuing small healthcare projects but never scaling them, leading to duplication and short-lived benefits. This tremendous duplication of effort has not only led to a waste of resources but has also resulted in more complicated health systems. Rather than improve information flows among stakeholders, this series of non-integrated health systems has created disjointed "information islands" that have become barriers to effective communication. Furthermore, the useful lifespan of many projects is often determined by the availability of limited donor funding, and thus many cease to operate once initial development funding ends.

1.3 Purpose of Document

The guidelines seek to establish an effective, transparent and accountable framework for introducing digital health solutions and innovations.

1.4 Goal

To enhance healthcare delivery, patient outcomes, and system efficiency through the effective, equitable, and ethical integration of digital health technologies

1.5 General Objective

To standardise the processes of introducing digital health solutions and innovations into the Ugandan health sector that are capable of being integrated into the national health systems to promote scalability and sustainability.

1.5.1 Specific Objectives

1. Functionalise an appropriate governance framework for the introduction of digital health solutions and innovations in Uganda.
2. Define and standardise the processes for introducing, scaling, and sustaining digital health solutions and innovations.
3. Ensure interoperability of the digital health solutions and innovations with other Ministry of Health data systems.
4. Ensure adherence of digital health solutions and innovations to the existing legal and guiding frameworks in Uganda.

1.6 Scope

All digital health solutions and innovations being introduced into Uganda's health sector (private and public health sectors). This shall include donations of digital health solutions by donors and other stakeholders. The scope shall also include procurement of digital health solutions for both the private and public sectors. For the public sector, the Public Procurement and Disposal of Public Assets Act 2003 shall apply while for the private sector, the procurement guidelines as per the organisational policy shall apply.

1.7 Target Audience

This document is intended for;

1) The Ministry of Health Departments and Programs
2) Local Governments
3) Health workers
4) Technology and Implementing Partners
5) Development Partners and Donors/Funders
6) Health Policymakers
7) Researchers
8) Digital Health Solution Providers
9) Innovators
10) Academia
11) Procurement entities
12) Other relevant stakeholders.

1.8 Methodology

A highly consultative approach was used in the development of these guidelines. Stakeholders from various entities such as the Ministry of Health (MoH) Department and Programmes, Local Governments (LGs), Development Partners, Implementing Partners, Technology and Implementing Partners, Academia, Innovators, Technology Providers and other members of the Health Information Innovation and Research Technical Working Group (HIIRE TWG) supported the generation of a draft which was reviewed and later validated.

The validated guidelines were endorsed by the Ministry of Health's HIIRE TWG and the Senior Management Committee (SMC), and approved by Top Management for implementation within the health sector.

1.9 Underpinning Guiding Principles

1. Client Centered
2. Equity
3. Efficiency
4. Transparency and Accountability

1.10 Policy, Reference Guidelines and International Standards

These guidelines are premised on the following existing frameworks;

1. Public Procurement and Disposal of Public Assets Act 2003
2. The National Health Policy (NHP) III
3. Ministry of Health Strategic Plan 2021/2025
4. Uganda Health Information and Digital Health Strategic Plan 2020/21 - 2024/2025
5. Guidelines and Standards for Acquisition of IT Hardware and Software for Ministries, Departments and Agencies
6. Uganda Digital Health Enterprise Architecture, Standards and Knowledge Guidelines
7. Uganda Health Data Protection, Privacy and Confidentiality Guidelines

1.11 Revision and Updates

These guidelines shall be reviewed and any proposed changes documented annually to maintain relevance and/or responsiveness to an evolving healthcare ecosystem and context. Relevant sections that will need to be added to the document shall follow the standard MoH approval processes. A new version number and date of approved updates shall be documented.

2.0 The Digital Health Solutions and Innovation Approval Process

All digital health solutions and innovations implemented in Uganda must adhere to the Uganda Digital Health Enterprise Architecture, Standards and Knowledge Guidelines. The set standards within the guidelines are based on the categories under which they fall. These broad categories include Electronic Medical Records Systems (EMR), Community Health Information Systems (CHIS), Laboratory Management Information Systems (LMIS), Radiology Information Management Systems, general electronic Health Management Information Systems, and Logistics Management Information Systems amongst others. To ensure adherence to the standards and accurate curation of all the solutions in the country, they are all required to undergo an approval process as elaborated below; -

2.1 Registration of the Digital Health Solution and Innovations

The initial stage in the approval process entails registration of the digital health solution and innovation with the Digital Health Subcommittee (DHSC) of the HIIRE TWG and will follow the following criteria.

1. The registration of the solution or innovation shall be initiated by the Ministry of Health Departments and Programmes and all other relevant stakeholders including innovators through the DHSC email dhs@health.go.ug.
2. Alternatively, registration shall also be initiated through the electronic portal known as the Digital Health Tools Registry (DHTR) which shall be accessed using the URL dhtr.go.health.ug. The link will be also accessed through the Ministry of Health website where it will be published.
3. The registration process shall be guided by the **Digital Health Systems Assessment Criteria (DHSAC) as detailed in Appendix 1**. At this stage, preliminary information about the solution or innovation being registered shall be captured. It is necessary to add here the mandatory details of the information that shall be provided at registration to further aid the assessment of the solution or innovation. Failure to provide the mandatory information shall result in non-qualification for the next stage. i.e. the form submission via the electronic portal shall be automatically validated against mandatory fields, uncompleted forms will not allowed to be submitted. Forms submitted via email shall be checked for completeness by the DHSC Secretariate team.
4. A confirmation or acknowledgement of successful form submission shall be received within **24 hours** in case the DHTR electronic portal is used. In case of email submission, an acknowledgement email or confirmation of submission will be received within **5 working days**.

2.2 Presentation to the Digital Health Subcommittee

Successfully registered solutions shall be scheduled for presentation to the DHSC and the following criteria will be followed;

1. Sections A, B and C of the **DHSAC** shall be fully completed by the digital health solutions providers or innovators before being scheduled.
2. A presentation template shall be shared with the solutions provider or innovator by the DHSC secretariate team or through download from the DHTR electronic portal.

3. A presentation scheduled shall be generated indicating the date, time and means of presentations and shall be shared with the solutions provider or innovator through the registered email.
4. A minimum of a week's notice shall be provided to allow for adequate preparations by the solutions provider or innovator.
5. All solutions or innovations shall be assessed based on **Digital Health Systems Assessment Criteria** (Appendix 1).
6. The DHSC shall seek clarifications concerning the solution or innovation up to **1-week** post-presentation to enable the team to produce a comprehensive assessment report.
7. The DHSC technical report shall be produced within **2 weeks** by the secretariate team and shared with members for further input and review.
8. Review and input by members of the DHSC shall be done within **1 week** after sharing the initial draft of the report by the DHSC secretariate team.
9. The final technical assessment report by the DHSC shall be shared with the solutions providers or innovators within **3 weeks** post presentation of the digital health by the solutions provider or innovator.
10. The report shall document clear recommendations and next steps for the solutions provider or innovators including;
    a. Recommendation for endorsement of the solution or innovation by the relevant User Department or Programme in writing if there is a need.
    b. Whether the solution or innovation has been endorsed for presentation to the HIIRE TWG or not. In case further clarifications or presentations are needed to be made to the DHSC, steps 3 to 9 under section 2.2 shall be followed.
11. In a situation where the DHSC determines that the solution passes the Digital Health Systems Assessment Criteria, a decision shall be made if a site visit is required if necessary, or endorsement to present to HIIRE TWG shall be granted. Solutions that do not fulfil the requirements shall be required to redo a presentation after effecting the recommendations of the DHSC.

2.2.1 Field Visit/Proof of Concept

In case a field visit is recommended, the Digital Health Subcommittee members shall then make a field visit to at least a site where the solution or innovation being assessed is being implemented. At this stage, the aim is to correlate the functionality as purported in the presentation to the DHSC to the actual operation in the real working environment. The field

visit report shall be compiled and shared with the DHSC secretariat team within five days of the field visit. The field visit report will incorporated within the earlier DHSC report to have a complete report.

Solutions that shall be found to be performing below expectation and not in line with the stated functionalities shall be requested to effect the recommendations from the DHSC before being endorsed to move to the next stage of evaluation.

All digital health solutions or innovations not endorsed by the HIIRE TWG shall not be presented to SMC.

2.3 Presentation to the Health Information Innovation and Research Technical Working Group (HIIRE TWG)

1. Recommendations from the Digital Health Subcommittee concerning the evaluated digital health solution or innovation shall be shared with the HIIRE TWG before scheduling the solution for presentation to the HIIRE TWG.
2. A User Department or Programme shall lead the presentation to the HIIRE TWG and introduce the solution, solution providers and innovators to the HIIRE TWG members before proceeding to present the proposed solution or innovation.
3. No presentation of the digital health solution or innovation shall be made to the HIIRE TWG in the absence of the User Department or Programme.
4. The HIIRE TWG shall only approve digital health solutions or innovations for the pilot.
5. The approved digital health solution or innovation for the pilot shall be communicated officially to all stakeholders concerned.
6. In case of scale or formal adoption of digital health solutions or innovations, the HIIRE TWG shall only endorse the solution or innovations for presentation to the Senior Management Committee (SMC).
7. The HIIRE TWG recommendations and endorsement shall be documented in the meeting minutes and shared with the secretariat team of the SMC before the solution can be presented to the SMC.
8. All digital health solutions or innovations not endorsed by the HIIRE TWG shall not be presented to SMC.

2.4 Presentation to the Senior Management Committee (SMC)

Digital health solutions or innovations that need to be scaled or formally adopted shall be presented to the SMC. Also, solutions that need to be piloted but have a policy implication shall be presented to the SMC following HIIRE TWG recommendations.

1. Recommendations by the HIIRE TWG shall be implemented first by the digital health solutions provider, innovator, user department or programme before the endorsed solution or innovation is presented to the SMC.
2. A User Department or Programme shall lead the presentation to the SMC and introduce the solution, solution providers and innovators to the SMC members before proceeding to present the proposed solution or innovation.
3. No presentation of the digital health solution or innovation shall be made to the SMC in the absence of the User Department or Programme.
4. The SMC shall endorse the digital health solution or innovation for presentation to the Health Policy Advisory Committee (HPAC) in case of policy and financial implications.
5. Otherwise, the proposed digital health solution or innovation shall be endorsed for presentation to the Top Management Committee for approval.
6. All recommendations by the SMC shall be implemented by the solution provider, innovator, user department or programme before proceeding to the next stage of approval.
7. The SMC recommendations and endorsement shall be documented in the meeting minutes and shared with the secretariat team of the HPAC or SMC before the solution can be presented to the next approval level.
8. All digital health solutions or innovations not endorsed by the SMC shall not be presented to HPAC or Top Management.

2.5 Presentation to the Health Policy Advisory Committee (HPAC)

In case the proposed digital health solution or innovation has a policy or financial implication, this will require endorsement by HPAC

1. Recommendations by the SMC shall be implemented first by the digital health solutions provider, innovator, user department or programme before the endorsed solution or innovation is presented to the HPAC.

2. A User Department or Programme shall lead the presentation to the HPAC and introduce the solution, solution providers and innovators to the HPAC members before proceeding to present the proposed solution or innovation.
3. No presentation of the digital health solution or innovation shall be made to the HPAC in the absence of the User Department or Programme.
4. HPAC shall endorse the digital health solution or innovation for presentation to the Top Management for approval.
5. All recommendations by the HPAC shall be implemented by the solution provider or innovator before proceeding to the next stage of approval.
6. The HPAC recommendations and endorsement shall be documented in the meeting minutes and shared with the secretariat team of the Top Management Committee before the solution can be presented to the next approval level.
7. All digital health solutions or innovations not endorsed by the HPAC shall not be presented to Top Management.

2.6 Presentation to Top Management Committee

1. Recommendations by the SMC or HPAC shall be implemented first by the digital health solutions provider, innovator, department or programme before the endorsed solution or innovation is presented to the Top Management Committee.
2. A User Department or Programme shall lead the presentation to the Top Management Committee and introduce the solution, solution providers and innovators to the Top Management Committee members before proceeding to present the proposed solution or innovation.
3. No presentation of the digital health solution or innovation shall be made to the Top Management Committee in the absence of the User Department or Programme.
4. The Top Management Committee shall approve the digital health solution or innovation for procurement, scale or formal adoption.
5. All recommendations by the Top Management Committee shall be implemented by the solution provider or innovator, department and programme before procurement, scaling or implementation of the solution or innovation.
6. The Top Management Committee recommendations and approval shall be documented in the meeting minutes.

7. All digital health solutions or innovations not approved by the Top Management Committee shall not be procured, scaled or implemented.
8. The approved digital health solution or innovation shall be communicated officially to all stakeholders and recognised as a Ministry of Health official system.

3.0 General Considerations

1. All systems being implemented at various healthcare levels without official approval will be required to undergo steps 2.0 to 2.6 as part of the approval process.
2. For purposes of formal adoption or transition, and scale-up of the proposed digital health solution or innovation, the following supporting documentation will be needed in addition to the Digital Health Systems Assessment Criteria (DHSAC) documentation earlier defined in section Appendix 1.
    a. System Transition Plan
    b. System Sustainability Plan
    c. System Capacity Building Plan.
    d. System Specification Documentation
    e. System User Requirements Documentation
    f. System Scale-Up Plan
3. Further supporting documentation besides what is mentioned above may be needed by the various approving structures to make an informed decision. It will be upon the digital health solutions provider, innovator, relevant User Department or Programme to provide the needed information promptly.
4. All procurement of digital health solutions for the public sector shall follow the Public Procurement and Disposal of Public Assets Act 2003 while those of the Private Sector shall follow the procurement guidelines as per institutional policy.

4.0 Digital Health System Implementation Framework

Various entities will have different roles and responsibilities during the approval process and this is detailed below;

SN	Entity	Responsibility
1	Top Management Committee	3. Strategic leadership, guidance, and oversight.<br>4. Approval of digital health systems or innovations for procurement and use in Uganda as official tools.<br>5. Monitoring and Supervision of digital health systems and innovations.
2	Health Policy Advisory Committee (HPAC)	1. Strategic leadership, guidance, and oversight.

		2. Ensure that digital systems are aligned with existing policies and laws.<br>3. Monitoring and Supervision of digital health systems and innovations.<br>4. Endorsement of digital health solutions or innovations for presentation to the Top Management Committee
3	Senior Management Committee (SMC)	1. Strategic leadership, guidance, and oversight.<br>2. Ensure digital systems are aligned with the needs of the departments and programmes.<br>3. Monitoring and Supervision of digital health systems and innovations.<br>4. Endorsement of digital health solutions or innovations for presentation to the Health Policy Advisory Committee or Top Management Committee.
4	Health Information, Innovation and Research Technical Working Group (HIIRE TWG)	1. Approval of digital health solutions or innovations for piloting.<br>2. Monitoring and Supervision of digital health systems<br>3. Give technical guidance and quality assurance for the proposed digital health solutions and innovations.<br>4. Ensure adherence of digital health systems and innovations to standards and guidelines.<br>5. Review and endorse proposed standards, guidelines, System Transition Plans, System Sustainability Plans, System Capacity Building Plans, System Specification Documentation, System User Requirements Documentation and System Scale-Up Plans.<br>6. Endorsement of digital health solutions or innovations for presentation to the Senior Management Committee
5	MoH/Department of Planning, Finance and Policy	1. Strategic leadership, guidance, and oversight.<br>2. Ensure digital systems are aligned with the needs of the departments and programmes.<br>3. Monitoring and Supervision of digital health systems and innovations.<br>4. Coordinate implementation of digital health solutions and innovations<br>5. Prioritise areas of implementation in line with the Digital Health and Health Information Strategic Plan 2020/21 – 2024/25<br>6. Coordinating and Managing access to digital health systems<br>7. Resource mobilisation and funding for implementation of digital health systems.

6	Division of Health Information Management	1. Coordinate implementation of digital health solutions and innovations<br>2. Recommend digital health systems for pilot and use following a technical evaluation<br>3. Prioritise areas of implementation in line with the Digital Health and Health Information Strategic Plan 2020/21 – 2024/25<br>4. Managing access to digital health systems<br>5. Coordinate and ensure data and system management with the support of stakeholders.<br>6. Quality assurance of digital health solutions and innovations<br>7. Coordinating integration of digital health system with other system<br>8. Advise on changes and modifications to the digital health systems<br>9. Supervision of implementation teams.<br>10. Coordinate the development of standards and guidelines.<br>11. Coordinate capacity-building programmes with stakeholders<br>12. Coordinate the development and implementation of sustainability and transition plans for digital health solutions with stakeholders.<br>13. Ensure appropriate governance of digital health solutions and innovations<br>14. Resource mobilisation and funding for implementation of digital health systems.<br>15. Monitoring and Supervision of digital health systems and innovations.
7	MoH/IT Section	1. Provide hosting infrastructure for approved digital health systems<br>2. Security of digital health systems and hosting environment<br>3. Ensure data and system backup<br>4. Coordinate with external entities in case of outsourced hosting<br>5. Coordinating and Managing access to system hosting environments/backend.<br>6. Define and Specify infrastructure requirements for hosting health solutions<br>7. Guide on<br>8. Recommend digital system hosting strategies
8	MoH/User Departments	1. Participate in the digital health system user requirements specification<br>2. Programmatic guidance on the functionality of the digital health tools<br>3. Participate and sign off on user acceptance tests for the digital health systems or workflows.

9	Partners	4. Technical assistance for the implementation of digital health systems and innovations<br>5. Resource mobilisation and funding for implementation of digital health systems.<br>6. Capacity building of MoH and Local governments on management and use of the digital health systems.<br>7. Participate in the development of standards and guidelines.<br>8. Monitoring and evaluation of the digital health system<br>9. Participate in the roadmap of the development of digital health systems within the health sector<br>10. Optimise implementation and adaptation of the approved digital health systems
10	Local Governments	1. Support implementation of the approved digital health systems at the local government level<br>2. Coordination of implementation at the local government level (districts and cities) with partners<br>3. Supervision of implementation teams<br>4. Participate and sign off on user acceptance tests.<br>5. Implement the digital health system sustainability plan with the support of MoH and stakeholders.
11	Health Facilities	1. Ensure data quality assurance and assessment.<br>2. Support health workers on the use of approved digital health systems<br>3. Conduct capacity building of the health workers on the approved digital health system.<br>4. Generate a list of users of the digital health system for approval by the Division of Health Information Management<br>5. Support the implementation of the approved digital health system at national and subnational levels.<br>6. Supervision of health workers within their catchment area
12	Digital Health Subcommittee	1. Review and assess digital health systems with a view of alignment with national Digital Health and Health Information Strategic objectives.<br>2. Review existing digital health solutions versus new and/or parallel developments with a view to minimising duplications.<br>3. Technically review proposed investments to determine and advise on potential benefits.<br>4. Technically review and refine business case requirements to determine and advise on costs, benefits, timelines, and resources.<br>5. Technically review to determine availability and commitment of project sponsor.<br>6. Technically review to determine project management and project governance needs.<br>7. Provide recommendations for approval of business case/digital health solutions.

5.0 Monitoring, Evaluation and Dissemination

This Section outlines how the monitoring and evaluation of the Guidelines for the Introduction of Digital Health Solutions and Innovations in Uganda will be conducted. This shall be based on the Monitoring and Evaluation framework stipulated in the Uganda Health Information and Digital Health Strategic Plan 2020/21-2023/24 which is essential for compliance and effectiveness of the guideline. Therefore; the guideline shall be monitored in terms of the: -

- Dissemination of the guideline
- Compliance with the guideline

All records related to the endorsement and approval of digital health solutions and innovations shall be documented by the secretariat teams of the relevant endorsing and approving structures.

The status of the digital health solutions or innovations will be updated by the Digital Health Subcommittee secretariat team on the electronic Digital Health Tools Registry to enable easy reference and retrieval of information.

A checklist shall be developed to monitor the compliance of implementers with the guidelines.

5.1 Dissemination and Adoption of the Guidelines

The Guidelines for the Introduction of Digital Health Solutions and Innovations in Uganda shall be disseminated for adoption through:

1. Presentation of the guidelines to stakeholders.
2. Posting of the guidelines on the MoH websites and the electronic Library for access by the stakeholders.
3. Organising quarterly workshops to train digital health solutions providers, innovators and other relevant stakeholders.

Appendix 1: Digital Health Systems Assessment Criteria

FOR GOD AND MY COUNTRY

THE REPUBLIC OF UGANDA

MINISTRY OF HEALTH

Digital Health Systems Assessment Criteria (DHSAC)

Version 1.1

Table of Contents

The assessment criteria is made up of four core components. Sections A and B will provide the assessors the context required to understand your product and support your evidence. The core assessment criteria is defined in section C1-D1. Section D details the key Usability and Accessibility principles required.

The core criteria in Section C and D will determine the overall success of the assessment of your product or service.

A. Company Information (Non-assessed Section)

Information about your organisation and contact details.

Code	Question	Options
A1	Provide the name of your company	Free text
A2	Provide the name of your product	Free text
A3	Provide the type of product	App | Wearable | Software as a Service (SaaS) | Other
A4	Provide the name and job title of the individual who will be the key contact at your organisation	Free text

A5	Provide the key contact's email address	Free text
A6	Provide the key contact's phone number	Free text
A7	Provide the registered address of your company	Free text
A8	In which country is your organisation registered?	Free text

7.

B. Value Proposition – (Non-assessed Section)

Please set out the context of the clinical, economic or behavioural benefits of your product to support the review of your technology. This criterion will provide the context of the product undergoing assessment.

Where possible, please provide details relating to the specific technology and not generally to your organisation.

Code	Question	Options	Supporting information
B1	Who is this product intended to be used for?	Patients | Diagnostics | Clinical Support | Infrastructure | Workforce | Other	
B2	Clearly define the problem that is intended to		

	be solved by the proposed product.		
B3	Provide a clear description of what the product is designed to do and of how it is expected to be used	Free text	This question is a context question and therefore a high-level summary is required.
B4	Describe clearly the intended or proven benefits for users and confirm if / how the benefits have been validated	Free text	This question is a context question and therefore a high-level summary is required.<br><br>If your product has had an evaluation or undergone clinical trials include this information.
B5	Have you scanned the health sector or carried out a landscape assessment to find out if there are similar existing solutions and what could be unique about the solution being proposed	Yes | No<br><br>Free text	
B6	Please attach one or more user journeys which were used in the development of this product<br><br>Where possible please also provide your data flows	Provided | Not available	This question is a context question, and it is expected that existing documentation will be provided.<br><br>Data flows enable the assessor to understand how data moves through a product.

C. Technical Questions - Assessed Sections

C1 - Data Protection

Establishing that your product collects, stores and uses data (including personally identifiable data) compliantly.

This section applies to the majority of digital health technology products however there may be some products that do not process any held patient data or any identifiable data. If this is the case, the Data Protection Officer or another suitably authorized individual should authorise this data protection section to be omitted from the assessment.

Code	Question	Options	Supporting information	Scoring criteria
C1.1	Does your product have access to any personally identifiable data or MoH-held patient data?	Yes | No	The Data Protection and Privacy Act 2019 applies to the processing of personal data.	To pass, the developer must confirm that they are compliant with the Data Protection and Privacy Act 2019.
C1.2	Please confirm where you store and process data (including any third-party products your product uses)	Uganda only | Outside of Uganda	Individual organisations within the health sector are accountable for the risk-based decisions that they must take.	Individual organisations within the health sector are accountable for the risk-based decisions that they must take.<br><br>Due consideration should be taken where data is processed and stored outside of Uganda.

C1.3	If you process or store data outside of the Uganda, please name the country and set out how the arrangements are compliant with current legislation.	Free text	Should comply with the Data Protection and Privacy Act 2019	To pass, the developer must demonstrate that the country in which data is processed or stored is compliant with current legislation or the organisation's policy (should this differ)

C2 - Technical Security

Establishing that your product meets industry best practice security standards and that the product is stable.

Dependent on the digital health technology being procured, it is recommended that appropriate contractual arrangements are put in place for problem identification and resolution, incident management and response planning and disaster recovery.

Please provide details relating to the specific technology and not generally to your organisation.

Code	Question	Options	Supporting information	Scoring criteria
C2.1	Please provide the summary report of an external penetration test of the product that included Open Web Application Security Project (OWASP) Top 10 vulnerabilities from within the previous 12-month period.	Provided | No evidence available	The OWASP Foundation provides guidance on the OWASP top 10 vulnerabilities.	To pass, the developer must evidence that the product has undergone an external penetration test that included the OWASP top 10 vulnerabilities.<br><br>The penetration testing/summary report must demonstrate there are no vulnerabilities that score 7.0 or above using the Common Vulnerability Scoring System (CVSS).

C2.2	Please confirm whether all custom code had a security review.	Yes - Internal code review | Yes - External code review | No | No because there is no custom code		To pass, the developer must confirm that an internal or an external custom code security review has been undertaken. An external review is preferable; however, an internal code review would meet the baseline requirement.
C2.3	Please confirm whether logging and reporting requirements have been clearly defined.	Yes | No	To confirm yes to this question, logging (e.g., audit trails of all access) must be in place. It is acknowledged that not all developers will have advanced audit capabilities.	To pass, the developer must confirm yes that logging and reporting requirements have been clearly defined.
C2.4	Please confirm whether the product has been load tested	Yes | No	Load testing should be performed.	To pass, the developer must confirm yes that load testing has been performed.

C3 - Interoperability Criteria

Establishing how well your product exchanges data with other systems.

To provide a seamless care journey, it is important that relevant technologies in the health system are interoperable, in terms of hardware, software and the data contained within. Good interoperability reduces expenditure, complexity and delivery times on local system integration projects by standardising technology and interface specifications and simplifying integration. It allows it to be replicated and scaled up and opens the market for innovation by defining the standards to develop upfront.

Please provide details relating to the specific technology and not generally to your organisation.

Code	Question	Options	Supporting information	Scoring criteria
C3.1	Does your product expose any Application Programme Interfaces (API) or integration channels for other consumers?	Yes | No		To pass, developers must demonstrate that they have API's that are relevant to the use case for the product, are documented and freely available and that third parties have reasonable access to connect.<br><br>If the product does not have API's and there is a legitimate rationale for this considering the use case of the product then the MoH can accept this rationale.
C3.2	If yes, please provide detail and evidence:<br>● The API's (e.g., what they connect to) set out the healthcare standards of data interoperability e.g., Health Level Seven	Free text		

	International (HL7) / Fast Healthcare Interoperability Resources (FHIR)<br>● Confirm they are documented and freely available<br>● Third parties have reasonable access to connect<br><br>If no, please set out why your product does not have APIs.			
C3.3	Do you use any unique identifier to identify patient record data?	Yes | No | No because product does not identify patient record data		To pass, developers should detail how patients are uniquely identified within the system.
C3.4	Does your product have the capability for read/write operations with electronic health records (EHRs) using industry standards for secure interoperability (e.g. OAuth 2.0, TLS 1.2)	Yes | No | No because the product does not read/ write into EHRs		To pass, developers should confirm that the product has the capability to read/write into EHRs using industry standards for secure interoperability.<br><br>If a product does not use industry standards, then a legitimate rationale should be set out and the security, usability and appropriateness of the methodology should be considered.

D. Key Principles for Success

The core elements defined in this section will form part of the overall review of the product or service and is a key part to ensuring that the product or service is suitable for use. The assessment will set a compliance rating and where a product or developer is not compliant highlight areas that the organisation could improve on with regards to following the core principles.

D1 - Usability and Accessibility - Scored Section

Establishing that your product has followed best practice.

Code	Question	Options	Supporting information	Weighted score	Scoring criteria
D1.1	**Understand users and their needs in context of health care**<br><br>Do you engage user (including MoH user departments) in the development of the product?	Yes | No | Working towards it	User Department Report	10%	Developers should be awarded 10% if they demonstrate that user need has been taken in account through user research, search data, analytics or other data to understand the problem.<br><br>The submission should confirm that the developer has considered, and tested user needs with appropriate stakeholders (stakeholders will differ depending on the product) and that as the product continues to iterate user engagement has continued.<br><br>If the developer selects working towards it and/or can only partially evidence the requirement, for example user need has only partially been considered or it is not considered on an ongoing basis they should be awarded 5%.
D1.1.1	If yes or working towards it, how frequently do you consider user needs in your product development and what methods do you	Free text			

	use to engage users and understand their needs?				If the developer selects no to this question or cannot provide evidence that user need has been considered, they should be awarded 0%.
D1.2	**Work towards solving a whole problem for users**<br><br>Are all key user journeys mapped to ensure that the whole user problem is solved, or it is clear to users how it fits into their pathway or journey?	Yes | No | Working towards it	User Department Report	10%	Developers should be awarded 10% if they attach supporting information showing that the product solves a whole user problem or that it is clear to users how it fits into their pathway or journey.<br><br>If the developer selects working towards it and can provide evidence that goes some way to explaining how the whole user problem is solved or only partially explains how the product fits a user journey, they should be awarded 5%.<br><br>If the developer selects no to this question or cannot provide evidence that shows the user journey or how the product fits into the pathway or journeys, they should be awarded 0%.
D1.2.1	If yes or working towards it, please attach the user journeys and/or how the product fits into a user pathway or journey	Provided | No evidence available			
D1.3	**Make the service simple to use**<br><br>Do you undertake user acceptance testing to validate usability of the system?	Yes | No | Working towards it	User Acceptance Testing	10%	Developers should be awarded 10% if they attach supporting information showing user acceptance testing to validate usability of the product.<br><br>If the developer selects working towards it and can provide evidence that goes some way to demonstrate that user acceptance testing is being

D1.3.1	If yes or working towards it, please attach information that demonstrates that user acceptance testing is in place to validate usability.	Provided | No evidence available			used to validate usability of the system, they should be awarded 5%.<br><br>If the developer selects no to this question or cannot provide evidence that shows user acceptance testing to validate usability of the system, they should be awarded 0%.
D1.5	**Create a team that includes multi-disciplinary skills and perspectives**<br><br>Does your team contain multidisciplinary skills?	Yes | No | Working towards it		2.5%	Developers should be awarded 2.5% for confirming they have a multi-disciplinary team.<br><br>If the developer selects working towards it or no to this question, they should be awarded 0%.
D1.6	**Use agile ways of working**<br><br>Do you use agile ways of working to deliver your product?	Yes | No | Working towards it		2.5%	Developers should be awarded 2.5 % if they confirm they use agile ways of working.<br><br>If the developer selects working towards it or no to this question, they should be awarded 0%.
D1.7	**Iterate and improve frequently**<br><br>Do you continuously develop your product?	Yes | No | Working towards it		2.5%	Developers should be awarded 2.5% if they confirm they continually develop their product.<br><br>If the developer selects working towards it or no to this question, they should be awarded 0%.

D1.8	**Define what success looks like and be open about how your service is performing**<br><br>Do you have a benefits case that includes your objectives and the benefits you will be measuring and have metrics that you are tracking?	Yes | No| Working towards it		2.5%	Developers should be awarded 2.5% for confirming that the benefit case includes objectives and metrics that can be tracked.<br><br>If the developer selects working towards it or no to this question, they should be awarded 0%.

D2 - Cost - Scored Section

Establishing the total cost of ownership

Code	Question	Options	Supporting information	Weighted score	Scoring criteria
D2.1	**Understand the total cost of ownership**<br><br>Do you have financial support or a funding agency supporting the solutions development	Yes | No | Working towards it		5%	Developers should be awarded 5% if they attach supporting information showing that the product is already being funded.<br><br>If the developer selects working towards it and can provide evidence that goes some way to explaining the organisations being engaged in financing the solution, they should be awarded 2.5%.<br><br>If the developer selects no to this question or cannot provide evidence of funding they should be awarded 0%.
D2.1.1	If yes, please provide detail and evidence:				
D2.1.2	If no, what cost is the Government of Uganda expected to meet? (Capital and Operation expenditure)	Free text			

D3 – Sustainability- Scored Section

Establishing the sustainability of the product.

Code	Question	Options	Supporting information	Weighted score	Scoring criteria
D3.1	**Understand the sustainability of the product.**<br><br>Do you have a sustainability plan in place?	Yes | No | Working towards it		5%	Developers should be awarded 5% if they attach supporting information detailing the sustainability plan.<br><br>If the developer selects working towards it and can provide evidence that goes some way to explaining the sustainability plans or work towards sustainability, they should be awarded 2.5%.<br><br>If the developer selects no to this question or cannot provide evidence of sustainability, they should be awarded 0%.
D3.1.1	If yes, please provide detail and evidence:				

Supporting Documentation

Please ensure that when providing evidence, documents are clearly labelled with the name of your company, the question number and the date of submission.

Possible documents to be provided are:

- B6 - User journeys and data flows
- C2.1- External Penetration Test Summary Report
- D1.2.1 - User Journeys and/or how the product fits into a user pathway or journey
- D1.3 - Supporting information showing user acceptance testing to validate usability
- D2.1.1 - Supporting information showing available funding support for the product.
- D3.1.1 - Supporting information detailing the sustainability plan.

REPUBLIC OF UGANDA
MINISTRY OF HEALTH

GUIDELINES FOR THE IMPLEMENTATION OF THE ELECTRONIC MEDICAL RECORDS SYSTEM

DIGITALIZATION OF HEALTH FACILITY OPERATIONS

CLINICAL, ADMINISTRATION & FINANCIAL

JANUARY, 2024

DOCUMENT REVIEWS AND APPROVALS

Version	Owner	Author	Publish Date
1	Ministry of Health	Division of Health Information Management	18/09/2024

Table of Contents

Foreword **vi**

Preface **vii**

Acknowledgement **viii**

Abbreviations and Acronyms **ix**

Key Definitions **x**

1.0 Introduction **1**

1.1 Background 1

1.2 Goal 2

1.3 Purpose 2

1.4 General Objective 2

1.5 Specific Objectives 2

1.6 Scope 2

1.7 Methodology 3

1.8 Guiding Principles Used to Develop this Document 3

1.9 Policy, Reference Guidelines and International Standards 3

1.10 Revision and Updates 4

2.0 User Requirements, Design and Development **4**

2.1 Overall Architecture 4

2.2 Personas/Actors of EMRS 4

2.3 System Functions 6

2.4 Design Process 7

2.4.1 EMRS Design Principles 8

2.5 EMRS Development Process 9

2.6 System Testing 10

2.7 EMRS Upgrades 11

2.8 EMRS Branding 11

3.1 Implementing EMRS **12**

3.1.1 Public Sector Pre-Implementation Requirements. 12

3.1.2 Private Sector Pre-Implementation Requirements. 13

3.2 EMRS Deployment Phase. 13
3.2.1 Health Facility EMRS Work Plan 13
3.2.2 EMRS Deployment 13
3.2.2.1 Local Area Network and Software Configuration and Installation 14
3.2.2.2 EMRS Deployment End User Training 14
3.3 EMRS Post-Implementation Support 18
3.3.1 Continuous Capacity Building and Mentorship 18
3.3.2 Escalation of System Issues. 19
3.4 Transitioning from Other EMRS to MoH Recommended EMRS 20
3.5 EMRS Change Management 21
4.0 Data Management & Reporting 23
4.1 EMRS Data Management 23
4.2 EMRS Reporting 25
4.3 Private Sector EMRS Specific Requirements 27
5.0 Governance Framework 29
5.1 EMRS Implementation Arrangements 30
6.0 Monitoring and Evaluating the Guidelines 35
6.1 Implementation Guideline Use Monitoring 35
6.2 EMRS Deployment Monitoring 36
6.3 Evaluating the EMRS 38
6.4 Dissemination and Adoption of the Guidelines 39
Appendix A: Readiness Assessment 40
Appendix B: Hospital Walkthrough and ICT Assessment Tool 42
Appendix C: Sample Training Schedule 44
Appendix D: Sample EMRS Deployment Budget 46
Appendix E: EMRS Rollout Checklist 47
Appendix F: Standard Operating Procedure for EMR System Downtime 53
Appendix G: EMRS Support Structure 59
Appendix H: System Change Request Form 61
Appendix I: EMRS Guidelines Use Monitoring Framework 63
Appendix J: EMRS Deployment Monitoring Framework 64

Appendix K: EMRS Evaluation Framework 67

Foreword

This document presents Electronic Medical Records System (EMRS) implementation guidelines for the health sector. These guidelines are intended to standardize the implementation of the EMRS across Uganda's health system. These guidelines are aligned with the Health Information and Digital Health Strategic Plan 2020/2021-2024-2025 and Ministry of Health Strategic Plan 2020/2021-2024-2025.

The Ministry of Health (MoH) is committed to improving the application of digital health technologies to facilitate the attainment of its overall objective of delivering high-quality health services to all citizens. This aligns with the call of Uganda Vision 2040 and the National Development Plan (NDP) III 2020/21 – 2024/25 which require sectors to adopt Information Communication Technologies (ICTs) to optimize service delivery.

The EMRS therefore, is critical for improving the quality of health service delivery at the health facility level through the use of embedded decision support tools and passive data capture. These guidelines aim to support all stakeholders in the implementation and scale-up of the EMRS within the healthcare ecosystem.

These guidelines shall serve as a framework to ensure the proper governance and leadership, rollout and maintenance of the EMRS, while ensuring its sustainability.

All stakeholders are therefore called upon to adopt and use these guidelines while implementing the EMRS.

....

Dr. Henry G. Mwebesa
DIRECTOR GENERAL HEALTH SERVICES

Preface

The Electronic Medical Records System (EMRS) implementation guidelines are a major milestone in the journey towards quality, responsive, accessible, and cost-effective healthcare service delivery at the health facility level. A well-developed digital health information system is a fundamental and crucial component of any health system.

The use of the EMRS is not only a key enabler of direct patient care but also a vital tool in health program monitoring. Therefore, it requires the necessary attention and well-planned investment of resources to realise its function. The EMRS implementation guidelines generally align with the goals, and strategies stipulated in the Uganda Health Information and Digital Health Strategic Plan 2020/21-2024/25.

In the past decade, health service delivery in Uganda has registered tremendous improvement. This can be partially attributed to increased funding, technology evolution and use in the health sector, a transformation which has been progressively acknowledged by the Ministry of Health (MoH), its partners and other key stakeholders. The EMRS shall be implemented as a digital job-aid for health workers with reporting as a passive process hence improving the quality of health service delivery at the health facility level.

All stakeholders are therefore called upon to examine the EMRS implementation guidelines, assess their involvement, and thereafter align their present and future Standard Operating Procedures with the guidelines laid out in this document.

....

Dr. Byakika Sarah

Commissioner, Health Services

Department of Planning, Financing and Policy

Acknowledgement

The Ministry of Health expresses its profound gratitude to all divisions, departments and programs, members of the Health Information Innovation and Research Technical Working Group who contributed technical inputs leading to the successful completion of this document. Special appreciation goes to the staff within the Division of Health Information Management (DHIM), Information Communication Technology (ICT) Section and the Clinical Services Department for the overall guidance to ensure that the guidelines are aligned with the Health Information and Digital Health Strategic Plan 2020/21-2024/25 and the Ministry of Health Strategic Plan 2020/21-2024/25.

I acknowledge and thank all development and implementing partners that provided financial and technical support for this process, specifically CDC, USAID, METS and SITES. DHIM is grateful for all the support, sacrifice and contribution invested in the successful development of these guidelines.

Finally, the Ministry of Health is grateful to the Local Governments including health facilities and all those institutions and individuals who have not been specifically mentioned above, but who directly or indirectly contributed to the successful development and finalization of these EMRS implementation guidelines.

....

Mr. Paul Mbaka

Assistant Commissioner Health Services

Division of Health Information Management

Abbreviations and Acronyms

BPR	Business Processes Re-Engineering
BUHIC	Build Uganda's Health Workers ICT Capacity
DHIS2	District Health Information Software version 2
ELMIS	Electronic Logistic Management Information Systems
EMR	Electronic Medical Record
EMRS	Electronic Medical Record System
ERP	Enterprise Resource planning
HL7	Health Level 7
HMIS	Health Management Information System
HW	Health Worker
ICD	International Classification of Diseases
ICT	Information and Communication Technology
LAN	Local Area Network
M&E	Monitoring and Evaluation
SDP	Service Delivery Point
SMS	Short Message System
SNOMED CT	Systematized Nomenclature of Medicine - Clinical Terms
SOP	Standard Operating Procedure
SRS	System Requirements Specification

Key Definitions

Term	Definition
**Business Process Re-engineering (BPR)**	A business management strategy focusing on the analysis and design of workflows, and business processes within an organization.
**Electronic Medical Record (EMR)**	An electronic record of health-related information on an individual that can be created, gathered, managed, and consulted by authorized clinicians and staff within one health care organization.
**Electronic Medical Records System (EMRS)**	A single system or collection of integrated systems that automate both clinical and administrative processes in the health facility settings.
**Functional requirement**	A requirement that describes in detail the programmatic or project needs and/or requested behavior of a system or component. It specifies what the finished system or component is expected to do and how a user shall interact with it.
**Health Facility**	Refers to Dispensary, Health Center, Hospital, Clinic etc.
**Health Level 7 (HL7)**	A flexible standard by which various health care systems communicate with each other; it is typically used for transmission of patient level data.
**International Classification of Diseases (ICD)**	A statistical classification system used to assign diagnostic and procedural codes in order to produce coded data for statistical analysis, epidemiology, reimbursement and resource allocation.
**Interoperability**	Ability for a system to securely communicate and exchange data in an accurate, reliable, and meaningful way with another information system so that the clinical or operational purpose and meaning of the data are preserved and unaltered.
**Management Information System (MIS)**	A computer-based system that provides managers with the tools to organize, evaluate and efficiently manage departments within an organization.
**Non-functional requirement**	A requirement that specifies criteria that can be used to judge the operation of a system, rather than specific behaviors.

1.0 Introduction

1.1 Background

The Ministry of Health (MoH) is committed to improving the application of digital health technologies to facilitate the attainment of its overall objective of delivering high-quality health services to all citizens. This aligns with the call of Uganda Vision 2040 and the National Development Plan (NDP) III 2020/21 – 2024/25 which require sectors to adopt Information Communication Technologies (ICTs) to optimize service delivery.

The Ministry of Health has prioritised digital health interventions as laid out in the Ministry of Health Strategic Plan 2020/21 - 2024/25 (HSP). The plan advocates for "Acceleration of health research, innovation and technology development" as a means of improving health service delivery. This has further been detailed in the Uganda Health Information and Digital Health Strategic Plan 2020/2021 - 2024/2025. The vision of the strategic plan is a health sector in Uganda driven by evidence and leveraging digital health to improve efficiency in service delivery.

Digitising health facility operations is aimed at reducing the burden of reporting stemming from the use of paper-based tools and subsequent data quality issues. Significant improvements in health service delivery has been reported in health facilities utilising the Electronic Medical Records System (EMRS). The main achievements registered so far are reduced reporting burden and improved data quality.

However, the processes related to implementation such as service workflow re-engineering, functionalization of health analytics, meaningful information exchange, and governance of EMRS have been mostly undefined to date. As Uganda continues on her journey to implement EMRS nationwide, there is a need for implementation guidelines to direct operationalization and support the achievement of intended results.

1.2 Goal

Health facility planning and operations driven by evidence, leveraging digital health solutions to improve health service delivery.

1.3 Purpose

The guidelines seek to establish an effective, transparent and accountable framework for the implementation of the Electronic Medical Records System.

1.4 General Objective

To standardize the processes and implementation of the Electronic Medical Records System in the health sector.

1.5 Specific Objectives

1. Formulate and functionalise an appropriate governance framework for the implementation of EMRS.
2. Define and standardise the processes for introducing, scaling, transitioning and sustaining the EMRS.
3. To define the minimum functional and nonfunctional requirements for specific digital tools and systems in health facilities.
4. Ensure interoperability of the EMRS with other Ministry of Health data systems to facilitate efficient referral, commodity tracking, reporting and surveillance.
5. Establish appropriate change management strategies to foster the use of the EMRS.

1.6 Scope

All health service delivery points within the health facilities in both the public and private sectors shall follow these guidelines and procedures in their operations. These guidelines are intended for the following audiences:

a. The Ministry of Health Departments and Programs
b. Local Government

c. Public and Private Health Facilities
d. Facility Health Workers
e. Technology and Implementing Partners
f. Development Partners and Donors/Funders
g. Researchers
h. All other Relevant stakeholders

1.7 Methodology

A highly consultative approach was used in the development of the EMRS guidelines. Stakeholders from various entities such as the Ministry of Health (MoH), Local Governments (LGs), Development Partners, Technology and Implementing Partners, Academia and other members of the Health Information Innovation and Research Technical Working Group (HIIRE TWG) supported the generation of a draft which was reviewed and later validated.

The validated guidelines were endorsed by the Ministry of Health's HIIRE TWG and the Senior Management Committee (SMC), and approved by Top Management for implementation within the health sector.

1.8 Guiding Principles Used to Develop this Document

1. Client Centered
2. Equity
3. Privacy and Integrity
4. Efficiency
5. Transparency and Accountability

1.9 Policy, Reference Guidelines and International Standards

These guidelines are premised on the following existing frameworks;

- The National Health Policy (NHP) III
- Ministry of Health Strategic Plan 2021/2025
- Uganda Health Information and Digital Health Strategic Plan 2020/21 - 2024/2025
- Uganda Digital Health Enterprise Architecture, Standards and Knowledge Guidelines

1.10 Revision and Updates

These guidelines shall be reviewed and any proposed changes documented annually to maintain relevance and/or responsiveness to an evolving healthcare ecosystem and context. Relevant sections that shall need to be added to the document shall follow the standard MoH approval processes. A new version number and date of approved updates shall be documented.

2.0 User Requirements, Design and Development

This section details the process for the generation of user requirements that inform the system functionality and subsequent iterative system development and/or enhancement. For the Public Sector, the Division of Health Information Management (DHIM) shall coordinate the user requirements, system design and development process and provide overall guidance during this phase with oversight from the HIIRE TWG. During this phase, the MoH ICT Unit shall be engaged to assess and guide on the system hosting requirements as well as other technical inputs such as security. For the Private Sector, health facility ownership will guide the user requirements, design and development process.

2.1 Overall Architecture

The EMRS is part of the wider Uganda Health Information System architecture ensuring the interoperability of the EMRS with other Ministry of Health data systems to facilitate referrals, commodity tracking, reporting, analytics, and surveillance (including linkage to the eHMIS).

The EMRS fits within the overall enterprise architecture under the applications segment as further described in the Uganda Digital Health Enterprise Architecture, Standards and Knowledge Products Guidelines.

2.2 Personas/Actors of EMRS

The delivery and management of health services at the health facility level involves multiple actors, who play different but complementary roles. The following subsections describe the characteristics and roles of each of the personas of the EMRS.

a) Client

The client is the primary recipient of health services at the health facility level. Health facility clients fall into one or more cohorts defined by sex, age group, pregnancy status, health and nutrition status et cetera. These characteristics should be taken into account when enrolling health facility clients into specific health facility services. Health facility services include but are not limited to casualty and emergency, clinical consultation, radiology, laboratory, pharmacy and dispensary, mortuary, outpatient services including but not limited to immunization, maternal and child health, dental, eye, counseling, family planning, health promotion, nutrition, Antenatal Care (ANC), Postnatal care (PNC), and inpatient services but not limited to maternity, intensive care, among others. Caregivers of clients receiving health services are also included in this group. The clients shall be registered within the EMRS and services delivered to them tracked longitudinally.

b) Health Workers

The Health Worker is the primary agent of health service delivery at the health facility level. Their main role is to provide health facility services while passively collecting key program data. Health Workers shall utilize the EMRS to deliver quality health services and capture and use data for operational decisions such as tracking commodities, defaulters and referrals to higher-level health facilities. Health Workers include Clinicians, Nurses, Records managers, Radiologist, Laboratory Technicians, Surgeons, Consultants, and Supply Chain In charges among others.

c) Health Facility Supervisors

Health Facility Supervisors are responsible for managing the delivery of health services at the health facility level. Health facility supervisors oversee service delivery points within their area of assignment and provide support to other health workers. The involvement in the day-to-day health facility operations is supervisory and aimed at assessing and promoting service quality. Other tasks include training, mentoring and coaching health workers and also performing routine health data quality checks. Health facility supervisors shall access EMRS dashboards and supervisor views constituting key information necessary to provide insights on how best they can supervise and support the health workers.

d) Local Government

The Local Government shall be responsible for the management of healthcare programs at the local government level. The Local Government shall lead and coordinate the implementation of the EMRS at local government level (districts and cities). The Local Government shall access the EMRS through dashboards and reports for routine performance monitoring and evaluation, program monitoring, support supervision and reporting to enable evidence-based decisions.

e) Ministry of Health

The national-level team at the Ministry of Health is charged with the responsibility of system administration, technical programming aspects, supervision, resource mobilization, and planning. Escalation of any issue concerning the EMRS shall follow the application hierarchy right from the health workers at the health facility level to the national level.

2.3 System Functions

The software requirements and system functionality documentation shall be developed and maintained as the Electronic Medical Records System Software Requirements Specification document. The document shall be updated quarterly based on user feedback, design sessions, User Acceptance Tests, logged system issues, user requirements review, consultative engagements with key stakeholders, following Health Management Information System (HMIS) revision among others.

Overall, the patient workflows within the EMRS shall follow the Uganda Clinical Guidelines.

Below is a summary of key functionalities as a minimum and that shall further be customized according to the user requirements documentation.

Patient Registration and Management	• Registration of patients or clients.<br>• Managing new and already existing patients in the system.<br>• Assign a unique identifier to new patients.

Client Referral	● Internal referrals within the facility from one unit to another or amongst the different consultants with-in the same unit.<br>● Referrals to facilities below the level of the referring facility, at the same grade or a grade above.
Stock Management	● Commodity dispensing, adjustments and stock status monitoring in stores, pharmacy, laboratory and wards.
Disease Surveillance	● Reporting suspected cases of notifiable diseases and events of public health interest.
Analytics and Reporting	● Generating real-time case-based and aggregate client reports and analytics to drive evidence-based decision-making.
Data & Process Validation	● Validation rules that shall enable the verification of data and processes to ensure adherence to set standards before a user is able to save a record.<br>● Management of patient records.
Decision Support	● Functionality to ensure that the health worker's decision-making capability is improved.
System administration and Security	● Configuring the system with organizational hierarchies, users, user roles, commodities and security mechanisms.<br>● Should be interoperable with approved Ministries, Department and Agencies information systems.<br>● Ensure health data protection, privacy and confidentiality.

2.4 Design Process

A strong system design is crucial to the development and functionalization of the EMRS to produce real-time quality information for patient information management and decision-making. Through the design process, developers and programme teams understand the healthcare processes, persona user stories, unique data use requirements for each persona, the different healthcare workflows and

how they are delivered at the health facility level, the way users interface with the EMRS, and how to continue to build for interoperability.

The human-centred design approach and analytical development methods will be used to understand the user requirements while considering the bottlenecks and challenges that users face.

The human-centred design takes a bottom-up approach and focuses on understanding an intervention's users or stakeholders. It seeks to understand how people do their work, how they perceive their health services, and how they experience challenges and may envision solutions.

While the analytical approach focuses on breaking down the process into the elements necessary to solve it and takes a top-down approach. An analytical approach will often require an expert mindset (consultant/technology firm) to collect information about how the intervention works in a linear, causal process. The outcome is information-oriented, resulting in a user requirements document or report.

Therefore a participatory approach that allows users and stakeholders to contribute to the design and development process will be used in a blended manner, that is to say, utilizing both human-centered design and the analytical approach.

For the Public Sector, the Division of Health Information Management will lead the design and development process working with other relevant partners, departments and programs such as the Clinical Services Department, Maternal and Child Health Department, Expanded Programme on Immunization, AIDS Control Program, Tuberculosis and Leprosy Program, Environmental Health Department, Malaria Control Division, Nutrition Division among others.

For the Private Sector, health facility ownership will lead the design and development process following established guidelines and standard operating procedures.

2.4.1 EMRS Design Principles

The EMRS design principles shall guide every step throughout the EMRS design and development processes.

1. **Usability:** Design and develop the EMRS application in a way that shall minimize the complexity for end users interacting with it.
2. **Scalability:** Should be capable of continuing to perform as the EMRS scales both vertically and horizontally over time in terms of enrolling end-users, data management and use.

3. **Availability:** Ensure both system and data is available to digital health applications and their end users whenever needed (within the bounds of confidentiality) to guarantee client safety and business continuity.
4. **Interoperability**: Foster interoperability of the EMRS with other relevant digital health systems as per the digital health enterprise architecture.
5. **Collaboration:** Adopt a governance approach that includes multi-sectoral stakeholders in the decision-making and management of EMRS design and development.
6. **Open standards:** Use internationally accepted standards that promote interoperability for data, workflows, and technology.
7. **Data quality and integrity:** Follow accepted data standards and create measures to uphold the integrity and reliability of data captured, processed or stored by the EMRS.
8. **Ethics:** Equity, safe use, data privacy and protection: These principles are vital for ensuring the social inclusion of the EMRS.
9. **Access control:** Enforce mechanisms to restrict access to both the system and data stored within the system.

2.5 EMRS Development Process

The development process will follow a streamlined Software Development Life Cycle (SDLC). A Systems Requirements Specifications (SRS) document detailing both functional and non-functional requirements will be developed in line with the user requirements gathered.

The SRS document will then inform the Systems Design Document (SDD). Both documents will be presented to the stakeholders including target user departments or units for validation and sign-off.

The System development process will, where possible, employ locally available resources to reduce reliance on external sources. It is, however, recognized that some desired technical skills and capacity may not be available within the Ministry of Health, sponsoring or implementing organisation or in the country and will be outsourced according to the Government of Uganda procurement guidelines where applicable or otherwise according to the sponsoring entity's procurement guidelines.

To ensure a user-centered design approach and quality software, the Agile development model will be applied during the development process with extensive consultation and interaction with the end users. Key stakeholders will be engaged during these steps from the Ministry of Health relevant

departments and other line state agencies including but not limited to the Ministry of Information Communication Technology and National Guidance (MoICT&NG), National Information Technology Authority Uganda (NITAU), National Identification and Registration Authority (NIRA), Uganda Bureau of Statistics, and Local Governments to ensure interoperability and that other key requirements for the system are addressed.

2.6 System Testing

The EMRS testing will comprise both internal system tests that will focus on technical aspects of the system and User Acceptance Testing (UATs) where feedback on the developed/improved system shall be solicited.

a) Internal system tests

The Internal system tests will focus on technical aspects that will ensure system performance such as integration testing, unit testing, and functional and non-functional testing among others. These tests will be conducted by the technology partners, the MoH ICT Section and DHIM. A report on internal tests will be developed, maintained and signed off by the Ministry of Health (DHIM).

b) User Acceptance Testing

User Acceptance Testing (UAT) will be part of the software development process that focuses on real-world testing by the intended end users.

UATs will be conducted for every software developed, revised workflows, new features, integrations, system enhancements or upgrades.

A UAT report will be compiled and shared with relevant stakeholders through existing governance structures, the report will incorporate feedback from users and the proposed system improvements.

A meeting with relevant stakeholders will be held to discuss the report and a road map to address the issues raised will be agreed upon.

The User Acceptance Testing report will be signed off once all the raised issues are addressed and validated by the user departments. For the Public Sector, the Division of Health Information Management will be responsible for the sign-off of UATs while for the Private Sector, the ownership of the health facilities will sign off the UTAs.

2.7 EMRS Upgrades

The EMRS shall be updated from time to time to ensure enhanced system performance. System upgrades shall be categorised into major upgrades and minor upgrades.

All upgrades shall be tested comprehensively to minimise system downtime. For major upgrades, the EMRS shall be upgraded annually to the most stable update available and the version shall be updated in the software inventory system.

All health facilities implementing EMRS shall have the same version of the system and therefore a mechanism shall be put in place to ensure uniformity in rolling out updates for EMRS across all health facilities.

2.8 EMRS Branding

Public Sector EMRS shall be branded to have a national outlook consistent with the national information systems for purposes of ownership.

The EMRS shall have the following branding;

1. The Uganda Emblem and Flag
2. White Background
3. Link to the Ministry of Health's Health Information System Privacy Notice
4. MoH Contact Details
    a. Website - https://www.health.go.ug
    b. Email - hmissupport@health.go.ug
    c. Address - Ministry of Health, P.o.Box 7272, Plot 6 Lourdel Road, Kampala - Uganda

The office in charge of communication at the Ministry of Health shall guide further on the EMRS branding to ensure a national outlook.

EMRS in the Private Sector shall be branded following the guidance on branding by the ownership of the health facilities where the EMRS is to be deployed.

3.1 Implementing EMRS

3.1.1 Public Sector Pre-Implementation Requirements.

This section guides the requirements and activities that need to be done before implementation activities start.

1. **EMRS National Rollout Roadmap**

   The rollout of EMRS shall be aligned to the National Digitization Roadmap developed and updated by the Ministry of Health. The roadmap details areas of priority, timelines, rollout plan and geographical coverage.

2. **Implementation Approval by MoH**

   Implementation of the EMRS in the public sector shall be approved by the Ministry of Health to ensure alignment with the National Digitization Roadmap.

   The Ministry of Health is responsible for providing overall coordination of all parties involved in the process, including but not limited to taking the lead in inception, reviewing progress, continuing implementation efforts, and monitoring compliance with all EMRS to be implemented. All parties intending to implement EMRS should seek guidance and approval from MoH before engaging Local Governments. As part of the approval process, the implementation plan shall be presented to the EMRS Working Group responsible for coordinating the implementation of the EMRS.

   Private sector implementers of the EMRS shall be guided by the MoH on how best to achieve alignment with the National Digitization Roadmap and for further technical support.

3. **Readiness Assessment**

   A readiness assessment using the Readiness Assessment Tool (**Appendix A**) for the proposed health facilities shall be conducted to check the readiness of the facility to support the rollout of the EMRS. This shall check if resources are planned to support the rollout of the EMR in the selected health facility and this includes but is not limited to; finances, human resources, hardware, and software. A detailed hospital walk-through focused on the Information Communication Technology (ICT) needs for the health facility utilising the ICT assessment

template in **Appendix B** shall also be conducted to quantify the ICT gaps and inform the budgeting process.

4. **National Trainer of Trainers**

   A national team shall be comprehensively trained following an updated standard training guide on the use and support of the EMRS before the rollout of the EMRS at the national and sub-national levels. This is to ensure there are adequate human resources at the national level to support further training and mentorship at the health facilities that have adopted the use of the EMRS. A comprehensive database of national trainers shall be maintained and updated regularly to include trainers from the health facility levels to avoid attrition of the Trainer of Trainers.

3.1.2 Private Sector Pre-Implementation Requirements.

Private Sector pre-implementation requirements shall be guided by the ownership of the health facilities which shall also determine the choice of EMR to be deployed.

3.2 EMRS Deployment Phase.

3.2.1 Health Facility EMRS Work Plan

The health facility with the support of the MoH and partners shall develop a detailed EMRS deployment work plan and budget following the sample training schedule in **Appendix C** and sample EMRS deployment budget in **Appendix D** detailing how the health facility shall roll out and coordinate the deployment of the EMRS within the health facility.

The work plan shall clearly indicate when the deployment shall be scheduled, the resources to support the deployment, and key persons leading on activities among others. The plan shall also include how the entire exercise shall be supervised and supported including pre and post-deployment support.

3.2.2 EMRS Deployment

In this phase, the EMRS shall be introduced to the National and Regional Referral Hospital Management, Local Government leadership and Health Facility Teams Leadership . This stage shall include training of health teams and the deployment of the EMRS shall strictly observe 100% digitisation of the health facility for efficient health service delivery, reporting, and accountability.

3.2.2.1 Local Area Network and Software Configuration and Installation

Deployment of the EMRS shall include the following installation of the computing infrastructure including the Local Area Network (LAN) and computing devices like servers, deploying and configuring the new EMRS software in its target environment and EMRS software acceptance testing.

EMRS deployment end-user training shall not commence until all the needed computing infrastructure i.e LAN has been installed within the health facility and tested for functionality.

The computing infrastructure installations shall be signed off the health facility in charge once successful tests have been conducted with a test report shared with the health facility in charge and the Ministry of Health IT Section or the respective private sector health facility ownership.

Once the computing infrastructures have been installed, the next step is the configuration and installation of the EMRS software. The installation shall include server and software installation. This shall be done before the commencement of the EMRS deployment training.

The health facility IT staff shall be trained on how to manage the installed computing infrastructure before handover of the same to the health facility administration.

3.2.2.2 EMRS Deployment End User Training

The EMRS deployment training shall include the following: basic computer training, EMRS software end-user training and ICT governance training for managers. This shall follow the processes below;

a) Entry Engagements

National and Sub-national level leadership engagement to introduce the program, seek political support, and discuss resource mobilization shall be held. The leadership to be targeted at the various levels shall include but not be limited to the following Hospital Directors, CAO, RDC, LC5, DHO, DISO, DPC, District Biostats, and Health Assistants among others that shall apply to all Local Governments. This shall apply to similar structures for cities and municipal councils. Where regional and local government level partners exist within the Regions and Local Governments, these shall be part of the engagements as well.

For the Private Sector, engagements shall be guided by the health facility ownership.

b) Device Custody and Management Plan:

Information Communication Technology devices procured by MoH or partners shall be declared to the national and sub-national leadership before hand over to the facilities. At the national and regional levels, the inventory shall be captured within the health facility inventory list. While at the local government level, the ICT equipment shall be captured within the local government and health facility inventory lists for purposes of tracking.

All devices shall be engraved to enable easy identification and tracking, the engravement numbers for the ICT equipment shall be captured within the inventory lists.

The ICT devices handed over shall be distributed according to the Health Facility EMRS Digitisation Plan and ensure all service points are well equipped. The devices shall also be redistributed depending on the need of the health facilities to ensure rational use of the ICT equipment.

Sensitive equipment like servers shall be maintained by ICT National level teams, Hospital Information Technology staff and Local Government ICT staff. Any other cadre shall seek clearance for example Partner ICT staff shall seek clearance from the health facility leadership.

Both private and public health facilities shall plan and budget for maintenance costs associated with the continued use of the EMRS.

c) On-Boarding Training

1. **Pre -Training/Preparation Phase**

   The training team shall ensure that all requirements detailed in the EMRS deployment checklist (**Appendix E**) are met ahead of the training.

2. **Regional and Local Government Level Training of Trainers**

   The trainer of trainers and supervisor training shall follow the updated standard training guide. Participants to be trained as ToTs and supervisors shall include, IT Officers, Biostatisticians, Medical Records Officers, Clinicians and Partner

Representatives. ToTs and Supervisors shall be trained on the EMRS for a **minimum of five (5) days** following the training guide and training scheduled (**Appendix C)**.

3. **Training of Health Workers**

The health workers' training shall follow the established training guide that shall entail all the service areas in EMRS appropriate for the level of the health facility. Health workers shall be trained on service areas for a minimum of five (05) days and on the digital aspect for a minimum of eight (08) days in class not exceeding 65 health workers each.

The eight days training on digital aspects shall include the basic ICT training aligned to the Build Uganda's Health Workers ICT Capacity (BUHIC) curriculum. The ICT basic training is aimed at providing basic computer skills needed for users to use a computer. Where possible, the ICT basic training shall be conducted prior to the training of health workers on EMRS by the national or local government level teams.

The training on health workers shall be conducted on a training instance that shall be easily distinguishable from the production or live system. Health Workers shall be availed with training credentials to the training instance once training on the digital aspect commences.

Health workers shall be subjected to a mandatory test before and after training on the EMRS to assess the extent of knowledge transfer. A minimum of 70% shall be obtained by a health worker before successful completion of the training.

Failure by the health workers to obtain the mandatory 70%, the health workers shall be re-trained on the service areas and the EMRS and subjected to a second test.

Failure by a health worker to obtain the mandatory 70% pass mark on a second attempt, the health worker shall be monitored closely and mentored during their use of the EMRS within the health facility.

d) On-Boarding to the Production EMRS

1. **Configuration and Health Workers Activation in the Production/Live Instance**

   Upon completion of training of health workers or targeted system users, their accounts shall be configured with their user profiles in the production/live instance of the EMRS and their training accounts deactivated in the demo/training system.

2. **Tooling and Reintegration into the Facility Structure**

   The trained users shall be equipped with the necessary work tools including but not limited to the digital job aids, EMRS user guide, links to important resources like the Ministry of Health E-Learning platform among others for reference purposes and further support.

   The trained health workers or targeted system users shall switch to the use of the EMRS within five (5) days after the successful completion of the EMRS training.

   After the five (5) days of transition, the HMIS manual registers shall only be used as a backup mechanism in the event the EMRS is non-functional as per the Standard Operating Procedures of the EMRS Downtime as indicated in **Appendix F**. Once the EMRS functionality has been restored, all the data captured using the manual registers shall be transferred as the Standard Operating Procedure.

e) Deployment Phase Closure and Signoff

Once the training has been concluded or aborted, the health facility leadership shall be briefed on the progress, findings and recommendations of the deployment phase.

An activity report shall be generated by the technical team supporting the EMRS deployment and signed off by the health facility in-charge or any other person delegated by the health facility in-charge. A copy shall be kept at the health facility and another shared at the national level by the team lead within 7 days of the closure of the activity.

All accountabilities shall be retired and outstanding issues settled within 14 days after exit from the local government or health facility.

3.3 EMRS Post-Implementation Support

3.3.1 Continuous Capacity Building and Mentorship

This section includes modalities of conducting continuous capacity building and mentorship post-deployment of EMRS. Relevant courses targeted at building health workers' capacity post-EMRS deployment training shall be uploaded on the Ministry of Health eLearning platform to enable virtual training. This shall be accessed https://elearning.health.go.ug/. The portal shall be kept with updated EMRS training courses like basic ICT skills courses i.e. Build Uganda's Health Workers ICT Capacity (BUHIC) and EMRS courses for health workers to access online for purposes of refresher training. This shall be part of the sustainability plan to ensure the building of health workers' capacity.

The online training portal shall be supplemented by the following activities;

a) **Support Supervision and Mentorship**

   A team from the National Level guided by reports generated from the EMRS shall make support supervision visits to targeted EMRS-implementing health facilities quarterly. Intern, implementing health facilities shall conduct quarterly support supervision to targeted health facilities under their supervision.

   The Ministry of Health Standard HMIS Support Supervision tool shall be used for this purpose and findings thereafter discussed with all stakeholders with clear action points and well-defined timelines.

   The health facility administration shall routinely perform support supervision and give feedback on areas of improvement and areas of good performance.

b) **Quarterly Performance Review Meetings**

   The EMRS performance review meeting shall be integrated into the local government and health facility quarterly performance review meetings to review the general performance of facilities, EMRS performance, uptake, data quality gaps identification, and develop improvement plans.

c) **Refresher Training**

   1. One full day monthly training for a minimum of three months consecutively after the

initial deployment of the EMRS shall be conducted and thereafter a minimum of three quarterly refreshers shall be held. The training shall majorly be informed by the system changes, knowledge gaps identified in terms of system utilization, staff changes, poor performance as guided by the monthly report, complex workflows and modules as identified by the Health Workers (System Users) or their supervisors, and new modifications in existing workflows among others.

2. Thereafter yearly refresher training is recommended to address any area of interest as may be pointed out by the national and subnational levels or informed by changes to the system.
3. Training on any new or updated workflow or system shall be scheduled on a need basis.

d) Health Information System Support Teams

Further capacity building of the health facility staff shall be built through the Health Information Systems (HIS) support teams. The HIS Support is a multi-sectoral approach to harness resources relating to infrastructure, human resources, and overall capacity for the proper functioning of digital health solutions.

Health facilities shall leverage support from both the government and partners to address their capacity gaps. The HIS Support teams shall be linked to the national level through the levels of support.

3.3.2 Escalation of System Issues.

System users shall utilise the support structures in place and communication channels to escalate any system issue, downtime or feature that is needed for continued improvement of the EMRS. The support structure is 5 tier (0-4) to support the various levels of EMRS implementation with the escalation procedures in **Appendix G.**

The tier three (3), expert technical support at the national level shall be engaged through official communication channels established and elaborated below;

1) MoH Call Center

The call center shall be the first point of contact for all help requests that originate from the end users of the EMRS at different levels. In the event that the call center team is unable to

resolve the issue, they shall be responsible for escalating tickets to the respective support teams. Issues shall be logged in the call center Customer Relationship Management (CRM) software for tracking and management until they are resolved. The current available toll-free number is 0800-100-066.

2) **Help Desk**

The Ministry of Health help desk shall be utilised to support the help desk functions of logging and tracking the EMRS end-user requests that come in, delegation to a responsible person for resolution, escalation of complex issues and managing the feedback to the end users. The Ministry of Health help desk shall be accessed through the URL:https://helpdesk.health.go.ug/

3) **Emails**

EMRS end users shall request help or inquire about EMRS through the official HIS support email address is hmissupport@health.go.ug

4) **Health Information System Community of Practice**

HIS Community of Practice online platform accessed through https://cop.health.go.ug/ shall be utilised to solve common system issues. Some of the system user issues could have been solved in the past and the solutions exist on the HIS Community of Practice webpages. Any new system issue shall be posted and solutions proposed by stakeholders to facilitate knowledge sharing and promote practical solutions.

Other information channels like phone calls, and social media like WhatsApp and Text messages shall be utilised to escalate system issues for users to be able to get the needed support.

3.4 Transitioning from Other EMRS to MoH Recommended EMRS

Transitioning of health facilities using the organization or disease-specific or non-comprehensive EMRS shall follow sections 3.1 to 3.2 with the following emphasis;

1) Local Government engagement meetings shall involve the affected health facility supervisors and any partners supporting the specific EMRS to be discontinued.
2) An inventory of the already issued devices used in the rollout of the EMRS to be discontinued shall be handed over to the local government management for inclusion in the local government inventory and tracking.

3) The health facilities shall work with technical teams on the ground to ensure the database from the EMRS to be discontinued is backed up and a copy kept by the health facility on an encrypted device. A copy of the same shall be kept at the Ministry of Health data centre as a backup mechanism.
4) Private health facilities shall ensure that the data from the EMRS to be discontinued is backed up and safely kept following the ICT policy or backup procedures in place.
5) In some cases, with clearance from the Ministry of Health for public health facilities, the old EMRS may be kept functional and operational depending on the unique need the EMRS is servicing i.e it may be specialized and may not warrant replacement within a specific clinic of speciality. In that case, two EMRS shall be utilized within the health facilities until further guided by the Ministry of Health.

3.5 EMRS Change Management

This guideline ensures standardized methods; processes and procedures are used for all changes, and maintain proper balance between the need for change and the potential detrimental impact of changes. Effective change management is very crucial in Health facility settings for controlling changes to EMRS and other supporting systems within the live Health facility environment.

Changes to all information processing facilities, systems, software, or procedure shall be strictly controlled according to formal change management procedures.

1. **Change Initiation:** All system changes shall be initiated by filling in the system change request form (**Appendix H**) form, which shall be signed by the respective heads of department.
2. **Change Authorization:** Authorization for any changes, whether urgent or not, shall be given by the head of the Division of Health Information Management. If satisfied that the reason for the change is sound and that there is no adverse effect as a result of the change.
3. **Testing of Changes:** All testing shall be done from a test environment. No change shall be applied to the live/production environment without having been tested in the test environment.
4. **Change approval:** The concerned user departments shall check the system to see whether the results produced are as expected and a user acceptance testing (UAT) form shall be signed off.

5. **Change Scheduling:** To minimize disruption to the normal working operations of the health facility, no changes shall be effected in the system during normal working hours unless absolutely necessary. All changes shall be scheduled during weekends, public holidays or after business working hours.
6. **Change Communication:** All planned system changes shall be communicated in advance to the concerned parties to minimize business disruption and inconveniences. For the avoidance of doubt, no scheduled system change should be effected in the live EMRS during a busy business cycle.
7. **Change Recovery and Safety Measures:** Before effecting any change in the production environment, a backup on a clearly labelled storage media shall be taken and kept for good. In the event that the change made produces unexpected results, the backup taken before the change was effected has to be used to restore the system to the point before the change.
8. **Change Documentation and Tracking:** All system changes, whether approved or not shall be documented and filed. The documentation shall include a duly completed and signed-off "change request form", the test results and any other comments/documents used.

4.0 Data Management & Reporting

Effective data management for EMRS is critical to ensure data accuracy, security, privacy, and accessibility while maintaining compliance with legal and regulatory standards.

Compliance with the Personal Data Protection and Privacy Act 2019 is critical as the EMRS collects individual-level data which is classified as personal data according to the law. To ensure compliance with the law, the Uganda Health Data Protection, Privacy and Confidentiality Guidelines shall be followed while implementing the EMRS in the health facilities. Below is further guidance on specific activities;

4.1 EMRS Data Management

1. **Data Entry and Standardization**
   a. All data entered into the EMRS shall on a minimum conform to the primary national Health Management Information System (HMIS) tools approved by the Ministry of Health.
   b. Health facilities shall implement regular audits and training programs for users to ensure accurate and timely data entry.
2. **Data Security**
   a. The EMRS shall implement a Role-Based Access Control (RBAC) to limit access to sensitive data based on job roles and responsibilities.
   b. User credentials (username and password) shall not be shared with any other person besides the account holder.
   c. Regular security assessments shall be conducted periodically to identify and mitigate vulnerabilities in the system.
   d. Only health facility staff shall have access to the individual level data or records for clients within their health facility. The district and national levels shall have access to system reports with the exception of the time during responses to public health emergencies like outbreaks for surveillance purposes. However, only access to relevant data shall be granted.
   e. Official or approved emails shall be used to share EMRS reports to avoid disclosing sensitive information.

f. **Section 3** of the Ministry of Health ICT Policy Guidelines shall be followed to ensure system and data security.

3. **Data Backup and Recovery**
   a. Automated backups for the EMRS at the health facilities shall be established with offsite storage for disaster recovery. The backups shall be conducted on a daily basis during the off-peak hours to minimise system interruption.
   b. All system and data backups shall be tested on a monthly basis to ensure they are functional.
   c. The data backup, recovery and disaster recovery plan shall follow **Section 9** Ministry of Health ICT Policy Guidelines.

4. **Data Sharing**
   a. All data requests shall be addressed to the Director General Ministry of Health.
   b. The Uganda Health Data Access, Sharing and Use Guidelines shall be followed to ensure data is shared securely while maintaining data protection, privacy and confidentiality.
   c. Any health data requests for research and innovation purposes shall require administrative clearance by the Ministry of Health and this shall follow the Uganda Health Data Access, Sharing and Use Guidelines.

5. **Data Retention, Archiving and Disposal**
   a. The data retention, archiving and disposal shall follow **Section 5.6** of the Uganda Health Data Protection, Privacy and Confidentiality Guidelines.

6. **Data Audit Trails**
   a. All EMRS shall maintain detailed audit logs that record all system activities, including data access, modifications, and deletions. Logs shall be tamper-proof and regularly reviewed.
   b. For purposes of accountability, mechanisms shall be implemented to track and address unauthorized access or suspicious activity identified in audit logs.

7. **Incident Management and Response**
   a. A data breach shall be reported to the Personal Data Protection Office and the Ministry of Health immediately after becoming aware of it.
   b. Management of data security breaches shall be in accordance with Section 5.7 of the Uganda Health Data Protection, Privacy and Confidentiality Guidelines.

c. Further incident management and response shall be handled according to **Section 10.0** of the Ministry of Health ICT Policy Guidelines.

8. **Health Information Exchange**

   a. EMRS shall ensure compliance to the Uganda Health Information Exchange and Interoperability Guidelines for seamless data sharing across the healthcare providers. By adhering to established standards and guidelines, the EMRS guarantees that patient information is exchanged accurately, efficiently, and confidentially. This commitment to the health information exchange standards as stated in the Uganda Digital Health Enterprise Architecture, Standards and Knowledge Guidelines not only enhances coordination and continuity of care but also promotes trust and reliability in the management of electronic medical records.

   b. The EMRS implemented within the health facilities shall be integrated with DHIS2 for seemingless reporting. The health facilities shall adhere to the reporting timelines as detailed in **Section 4.3** of these guidelines below.

   c. To achieve a single source of truth, the EMRS shall integrate with the National Health Information Exchange (NHIE) Registries i.e National Health Facility Registry, National Health Products Registry, National Health Workers Registry and the National Client Registry.

4.2 EMRS Reporting

The reporting requirements shall be based on the approved national HMIS tools. The reports shall be for purposes of synthesis, monitoring of usage and functionality. The data shall be pushed from the EMRS to the electronic Health Management Information System (Uganda eHMIS/DHIS2) on a monthly basis following the schedule below;

1. All data captured in the EMRS shall be synchronised or pushed automatically daily to the EMRS servers based at the health facilities..
2. The HMIS reports generated from the EMRS shall be pushed to Uganda eHMIS/DHIS2 every 7th of every month following.

3. Local Government and health facility level health managers shall review and validate the pushed EMRS datasets in Uganda eHMIS/DHIS2 from 8th - 15th of every month for purposes of ensuring completeness and accuracy.
4. Uganda eHMIS/DHIS2 shall be locked for entry or validations of data past 15th of every month to allow for analysis of consistent data for the reporting period.

All the synchronised data shall be accessed through the Uganda eHMIS/DHIS2. For real time data from the EMRS, dashboards (https://emrs-dashboards.health.go.ug/) shall be utilized to access indicator performance for various administrations at various levels.

Annual, Quarterly and Monthly performance reports indicating various indicators as per the National HMIS reporting format shall be produced and accessed within Uganda eHMIS/DHIS2. The expected reports with the frequency to be submitted by the EMRS is detailed in the Table xx below;

Table xx: National HMIS Reports to be submitted through the EMRS

Sn	Report Code	Report Description	Frequency
1	HMIS 033b	Weekly Epidemiological Surveillance	Weekly
2	HMIS 097b	VHT/ICCM Quarterly Report	Quarterly
3	HMIS 097c	VHT/ICCM Monthly Report	Monthly
4	HMIS 104	NTDS MDA Implementation Report	Bi-Annual
5	HMIS 105	OPD Monthly Report	Monthly
6	HMIS 105a	Specialized Outpatient monthly report	Monthly
7	HMIS 105B.1	Child Immunization Addendum Report	Monthly
8	HMIS 105C	Palliative Care Monthly Report	Monthly
9	HMIS 106a	HIV Quarterly Report	Quarterly
10	HMIS 107a	Sub County Annual Population Projection Report	Annual
11	HMIS 107c	Health Facility Human Resource Inventory	Annual

12	HMIS 107	Health Unit Population and Annual Report	Annual
13	HMIS 108a	Specialised Inpatient Monthly Report	Monthly
14	HMIS 108	IPD Monthly Report	Monthly
15	HMIS 110	District Health Facility WASH Report	Quarterly
16	HMIS 127b	Visceral Leishmaniasis Inpatient Monthly Report	Monthly
17	HMIS 128b	Visceral Leishmaniasis Outpatient Monthly Report	Monthly
18	HMIS MAL 002	Malaria Vector Monthly Reporting Form	Monthly

4.3 Private Sector EMRS Specific Requirements

Implementation of the EMRS in the private sector shall enhance the efficiency and quality of healthcare services, streamline patient record management and offer private healthcare providers with a robust, secure, and user-friendly platform to access and update patient information in near real-time. The EMRS shall not only reduce administrative burdens like reporting but also facilitate better coordination and continuity of care, ultimately leading to improved patient outcomes.

Below are the specific requirements besides the general guidance on EMRS implementation to be adhered to by the Private Sector.

1. Certification of the Private Sector EMRS by the Ministry of Health to ensure system security, and compliance to standards i.e. Uganda Digital Health Enterprise Architecture, Standards and Knowledge Guidelines, Uganda Clinical Guidelines and other relevant guidelines.
2. The certified EMRS shall integrate with the national electronic Health Management Information System (eHMIS)/DHIS2 to facilitate mandatory reporting into the national reporting system. The certified EMRS shall also integrate with the national core health registries i.e. National Health Facility Registry, National Client Registry, National Health Productions Registry and National Health Workers Registry.
3. The certified EMRS shall produce at a minimum the national HMIS reports to enable reporting to the national eHMIS/DHIS2 as outlined in Section 4.2 of the guidelines.

4. All Private Sector certified EMRS shall submit birth and death notifications to the national reporting system (electronic Health Information Management System/DHIS2)
5. The Private Sector certified EMRS shall submit client-level data to the Ministry of Health Data central reporting system (i.e. eHMIS/DHIS2 and National Data Warehouse) during public health emergencies such as pandemics, outbreaks, or other public health emergencies. The Government of Uganda retains the authority to request or sanction a copy of the health data by the EMR for purposes of epidemiological and reporting purposes.
6. EMRS and its health data shall not be hosted on the cloud outside of Uganda without the approval of the Personal Data Protection Office and the National Information Technology Authority Uganda (NITAU). This shall be done in accordance with the Data Protection and Privacy Act 2019.
7. All processed health data by the EMRS shall be owned by the Government of Uganda but held in trust by the data controller in this case the ownership of the health facility.
8. The health facility clients reserve the right to access their health data or records at no cost besides printing costs.
9. Health data access by Non-Government third parties shall be authorized by the Ministry of Health and shall follow the Uganda Health Data Access, Sharing and Use Guidelines.
10. The EMRS health data shall be used for research and innovation purposes in accordance with the National Guidelines for Research involving Humans as Research Participants. Ministry of Health shall provide administrative clearance for the use of the EMRS health data for research and innovation.
11. Compliance to the guidelines by the private sector health facilities shall be monitored through health facility self-assessments every quarter submitted to the Ministry of Health. External assessment shall be conducted annually by the Ministry of Health.

5.0 Governance Framework

The Governance of EMRS shall follow the existing governance and management structures at the national and decentralized levels, as summarized in Figure 3.

Technical oversight is the mandate of the Division of Health Information Management (DHIM) under the Department of Planning, Financing and Policy which guides and coordinates all stakeholders involved in health data collection, processing and storage. This function is similarly decentralized at the Local Government level, as summarized in Figure 3. The main task of the Health Information Innovation and Research Technical Working Group (HIIRE TWG) is reviewing and advising on Health Information System (HIS) and Digital Health policy-related and strategic related issues from the user departments and other stakeholders.

The Ministry of Health (MoH) has Technical Working Groups (TWGs) in its governance and management structures which act as advisory committees to Departments and the Senior Management Committee. The TWGs serve as structures to deliberate on, review and advise on policy, strategic and technical issues within the Ministry of Health.

National governance and management structures	Local Government level governance & management structures
Top Management Committee	Technical Planning Committee
Department of Planning, Financing and Policy<br>Division of Health Information Managment (DHIM)	District Health Team
Health Information, Innovation and Research (HIIRE) TWG	Extended District Health Management Team

Figure 3: Governance Structure

The Health Data Collaborative, a subcommittee of the HIIRE TWG shall be charged with the coordination of the implementation of the EMRS. Specifically, under the Health Data Collaborative (HDC) Subcommittee, the Health Sector Digitization Coordination Working Group supports the HDC in the coordination of the EMRS implementation within the country. The day to day operations

shall be handled under the EMR Implementation Technical Working Group of the Health Sector Digitization Coordination Working Group.

All the other HIIRE TWG subcommittees like the Data Management Subcommittee and Digital Health Subcommittees shall provide technical input towards the implementation of the EMRS in the country.

The Digitisation Steering Committee chaired by the Permanent Secretary of the Ministry of Health has been established as a special committee to ensure fast tracking of the implementation of the EMRS and other digital initiatives in the country.

Governance of the Private Sector EMR implementation shall be under the leadership of the ownership of the health facilities and will interface with the Governance Structure illustrated in Figure 3 through the HIIRE TWG.

5.1 EMRS Implementation Arrangements

EMRS implementation shall require that all the responsibilities of all stakeholders are spelt out in order to streamline the implementation arrangements and strengthen the governance framework.

The table below spells out the different responsibilities of different entries at different levels.

SN	Entity	Level	Responsibility
1	Top Management	National	● Strategic leadership, guidance and oversight.<br>● Approval of EMRS for use in the health sector.<br>● Monitoring and Supervision of EMRS implementation
2	Senior Management Committee (SMC)	National	● Strategic leadership, guidance and oversight.<br>● Endorsement of EMRS for use in the health sector.<br>● Monitoring and Supervision of EMRS implementation
3	Health Information, Innovation and Research (HIIRE) Technical Working Group	National	● Approve EMRS for piloting<br>● Recommend EMRS to SMC for full scaleup<br>● Monitoring and Supervision of EMRS implementation<br>● Give technical guidance and quality assurance<br>● Ensure the EMRS conform to set standards<br>● and guidelines.<br>● Review and recommend developed EMRS standards, and guidelines for approval by the SMC.
4	MoH/Planning, Finance, Policy Department	National	● Coordinate implementation of EMRS<br>● Recommend EMRS for pilot and use following a technical evaluation.<br>● Prioritize areas of implementation in line with the Digital Health and Health Information Strategic Plan<br>● Coordinate and ensure EMRS data and system management with the support of stakeholders.<br>● Quality assurance of EMRS<br>● Coordinating integration of EMRS with other system<br>● Authorizing changes and modification to the EMRS<br>● Supervision of implementation teams.<br>● Develop standards and guidelines for EMRS implementation<br>● Coordinate capacity building programmes with stakeholders on EMRS<br>● Coordinate the development and implementation of a sustainability plan for EMRS with stakeholders.
5	MoH/IT	National	● Provide hosting infrastructure for EMRS<br>● Security of EMRS and hosting environment<br>● Ensure data and EMRS backup<br>● Recommend hosting strategies for EMRS<br>● Coordinate with external entities in case of outsourced hosting for the EMRS

			● Coordinating and Managing access to EMRS hosting environments/backend.<br>● Coordinating and Managing access to EMRS
6	MoH/User Departments	National	● Participate in the EMRS requirements specification<br>● Programmatic guidance on functionality of the EMRS<br>● Participate and sign off on user acceptance tests for EMRS<br>● Resource mobilization and funding for the EMRS
7	Ministry of ICT&NG	National	● Strategic leadership and guidance.<br>● Advise and guide on technologies for EMRS technologies.<br>● Monitoring and Supervision of digital health initiatives.<br>● Resource mobilisation and funding for EMRS implementation<br>● Assess the gaps in HR capacity.
8	Ministry of Energy and Mineral Development	National	● Strategic leadership and guidance on energy solutions.<br>● Advise and guide on clean energy to power EMRS.<br>● Plan and ensure that health facilities are connected to the national electrical grid.<br>● Monitoring and Supervision of power sources for health facilities.<br>● Resource mobilization and funding for power solutions for EMRS implementation<br>● Guide and advise on power backup solutions for the EMRS implementation.
9	Partners	National/ Local Government	● Technical assistance for implementation of EMRS<br>● Resource mobilization and funding for implementation of EMRS<br>● Capacity building of MoH and Local governments on management and use of the EMRS.<br>● Participate in the development of standards and guidelines for the EMRS implementation.<br>● Support monitoring and evaluation of the EMRS.<br>● Participate in the EMRS implementation roadmap.<br>● Support the optimisation, implementation and adaptation of the EMRS

10	Local Governments	District/Cities/ Municipality/ Subcounty	● Lead the implementation of the EMRS at the local government level<br>● Coordination of implementation at local government level (districts and cities) with partners<br>● Supervision of implementation teams<br>● Participate and sign off on EMRS user acceptance tests.<br>● Implement the EMRS sustainability plan with the support of MoH and stakeholders.<br>● Resource mobilization.<br>● Performance Monitoring and evaluation
11	Health facilities	Regional/ District/Cities/ Municipality/ Subcounty	● Ensure periodic data quality assurance and assessment.<br>● Support health workers on the use of EMRS<br>● Conduct capacity building of the health workers on EMRS<br>● Generate list of users of EMRS for approval by the Department Planning, Financing and Policy.<br>● Supervision of health workers within their catchment area
12	Digitization Coordination Working Group	National	● Guiding on EMRS implementation modalities<br>● Coordinate EMRS stakeholders.<br>● Joint planning, monitoring and evaluation of the EMRS<br>● Make recommendations on the development and implementation of the EMRS.
13	EMR Implementation Technical Working Group		● Discuss the implementation experiences for the different EMRS implementing health facilities.<br>● Resolve operational challenges affecting EMRS implementation.<br>● Document EMRS issues, new system requirements and features and share with the Division of Health Information Management.<br>● Review EMRS performance reports for implementing health facilities.<br>● Make recommendations on the development and implementation of the EMRS.
14	Technology developers	National	● Development of EMRS and other functional-based health tools based on user requirements.<br>● Testing of EMRS instances and training of users<br>● Conduct installation and customisation of EMRS in health facilities

			• Coordination of EMRS upgrade, maintenance and scalability<br>• Ensure user friendly, reliable and robust EMRS environments.
15	Private sector	National	• Software and hardware provision to support EMRS implementation.<br>• Training and support services<br>• Financial investment<br>• Research, innovation and development<br>• Market competition and improvement<br>• Comply with EMRS national guidelines
16	Academia	National	• Research<br>• Training<br>• Monitoring and Evaluation<br>• Come up with Innovations to address EMRS implementation challenges.

6.0 Monitoring and Evaluating the Guidelines

Monitoring and Evaluation is vital to ensure the successful implementation and smooth operation of EMRS. As part of Monitoring and Evaluation, quality assurance exercise shall run throughout the life cycle of the EMRS implementation, especially during installation and configuration of the EMRS infrastructure (software and hardware), training of staff on EMRS and deployment of the EMRS to ensure compliance with the national EMRS implementation guidelines. Compliance with the guidelines shall be monitored through health facility self-assessments every quarter submitted to the Ministry of Health. External assessment shall be conducted annually by the Ministry of Health. Furthermore, it is necessary to track and evaluate the implementation and functioning of the system in order to understand how well the implementation objectives have been met and the effect of EMRS on the day-to-day health facility operations.

6.1 Implementation Guideline Use Monitoring

Monitoring the use of the Guidelines for Implementing the EMRS shall be based on a specific framework designed to track compliance and effectiveness. Important to note is that dissemination and training on the use of the guidelines is a key requirement before monitoring its use can become a practical reality. Therefore, the guidelines shall be monitored using three major parameters, namely, dissemination, training, and compliance. These are described further below and shall be tracked and assessed to measure guideline use. The guideline-use monitoring framework consists of the parameters or components, key monitoring questions, result statements, indicators, and means of verification.

- **Dissemination:** Refers to broadcasting the guidelines to a target audience through printed or electronic media such as print, electronic documents, or other forms of media as appropriate. Copies of the guidelines shall be printed and distributed at national and sub-national levels. The electronic copy of the guideline shall be uploaded on the Ministry of Health Knowledge Management Portal for public access and consumption.
- **Training:** Refers to orienting and/or sensitizing key stakeholders on the key components of the guideline document including its value and their roles and responsibilities to ensure awareness and accelerate compliance.

- **Compliance:** Refers to the state of implementing the EMRS in accordance with established guidelines or standards, or the process of becoming so (compliant).

Refer to **Appendix I** for a detailed Guideline-Use Monitoring Framework.

6.2 EMRS Deployment Monitoring

The EMRS implementation is anchored within the overall Uganda Health Information and Digital Health Monitoring and Evaluation Framework. Notably, it is critical to continue monitoring EMRS deployment nationwide to ensure that the software is working as it should, every activity is implemented as planned, and that external factors are not affecting the potential effectiveness of the EMRS intervention.

Findings from deployment monitoring activities shall be synthesized, disseminated, and remedial action taken to address identified challenges. This shall ensure optimization of EMRS implementation across board and foster successful evaluation efforts. Consequently, four (4) key parameters of EMRS deployment monitoring shall be considered, namely, functionality, stability, fidelity, and quality. They are described further below and shall be tracked and assessed to ensure that the intervention is “doing things right”.

Similar to guideline-use monitoring, the EMRS deployment monitoring framework shall consist of parameters or components, key monitoring questions, result statements, indicators, and means of verification.

- **Functionality:** *Refers to the degree to which EMRS provides functions that meet stated and implied needs when used under specified conditions, or the ability of EMRS to support the desired intervention.* For instance, the desired functionality of the EMRS application based on agreed upon user requirements. Monitoring functionality shall be done pre- and post-deployment to ensure adequate system functionality at all times. Pre-deployment monitoring shall entail testing and providing feedback on various aspects including but not limited to skip patterns, validation checks, form schedules, form content, user interface design, data export/import functionality, data accuracy, and dashboard calculations. Post-deployment and/or at a later maturity stage (i.e., where the EMRS applications have been fully designed and only enhancements are required to meet evolving policy changes and information needs), basic system functionality monitoring shall be conducted before introducing the EMRS to

new users (e.g., a different cadre of health workers) and new geographic areas that might pose different levels of connectivity or when using new technologies.

- **Stability:** *Refers to the likelihood that EMRS functions shall not change or fail during use or the ability of EMRS to remain functional under both normal and anticipated peak conditions for data loads*. Monitoring stability shall be done concurrently with functionality monitoring both pre- and post-deployment. Initial (pre-deployment) system stability monitoring shall be conducted during quality assurance testing sessions before the EMRS is declared ready for deployment (field-ready). Post-deployment, stability shall be monitored to mitigate any events that may result in improper delivery of the EMRS intervention such as unexpected crash or stop, slow response times especially during peak times or when overloaded, and any erratic performance hindering management and use of EMRS. Considering that the EMRS heavily relies on physical (non-cloud-based) servers based at the health facilities for operation, server outage shall be monitored. Considering stability is a critical aspect to ensure successful implementation of EMRS intervention, continued stability monitoring processes shall be automated and systems configured accordingly to enable proactive and timely reactive response.
- **Fidelity:** *Refers to a measure of whether or not an intervention is delivered as intended in terms of technical and user perspectives.* For instance, the technical fidelity of EMRS (i.e., functionality and stability) and the enabling environment such as any external barriers that might cause it not to function as intended, and compliance of EMRS end-users to stipulated data use and system administration standard operating procedures. Monitoring fidelity of EMRS intervention shall occur throughout implementation with the required level of effort gradually decreasing depending on the time it takes to identify and resolve issues. Standard monitoring procedures and reporting mechanisms shall be established and automated to aid timely decision-making.
- **Quality:** *Refers to the measure of excellence, value, conformance to specifications, conformance to requirements, fitness for purpose and ability to meet or exceed expectations.* For instance, the standards related to capabilities of EMRS end-users and content of the inputs used for the programmatic interventions that eEMRS is designed to deliver such as algorithms for decision support, data collection forms, and reports. This content should be of

the highest quality possible informed by existing practice, literature and formative research in the local context to increase effectiveness of the EMRS intervention. EMRS quality monitoring shall focus on ensuring (a) data quality and regularity by checking for outliers of non-compliant users; and (b) quality of content to be delivered is as expected and inline with existing standards and appropriate for participating communities.

Refer to **Appendix J** for a detailed EMRS Deployment Monitoring Framework.

6.3 Evaluating the EMRS

The evaluation shall entail any measures that shall be taken and analysis performed to assess;

a) The interaction of users and/or the health system with the EMRS intervention and strategies
b) Changes attributable to the implementation of EMRS.

Any commissioned evaluations focused on EMRS shall be designed to ensure assessment and evidence generation on its usability, effectiveness, value for money and affordability at the very least, depending on the degree of maturity i.e, early or middle or late stage. The following evaluation components shall constitute the EMRS evaluation framework in addition to the evaluation types:

- **Feasibility:** Assess whether EMRS works as intended in various contexts and health facility levels across Uganda.
- **Usability:** Assess whether the EMRS is used as intended.
- **Effectiveness:** Assess whether the EMRS achieves the intended results in an uncontrolled (non-research) setting.
- **Implementation research:** Assess the uptake, institutionalization, and sustainability of EMRS in Uganda, including policies and practices.

Depending on EMRS degree of maturity and evidence needs, several evaluation types categorized as formative or summative may be commissioned and/or conducted.

- **Formative evaluations:** Studies aimed at informing the design and development of effective intervention strategies conducted before or during implementation of an intervention.
- **Summative evaluations:** Studies conducted at the end or a certain phase of the intervention to determine the extent to which expected outcomes have been achieved.

Refer to **Appendix K** for a high-level EMRS Evaluation Framework.

6.4 Dissemination and Adoption of the Guidelines

The framework shall guide the collection of information regarding the implementation of the EMRS to facilitate reporting, feedback, and dissemination.

a) Dissemination and adoption of the guidelines

The EMRS implementation Guidelines shall be disseminated for adoption through:

1. Presentation of the guidelines to stakeholders.
2. Posting of the guidelines on the MoH websites and the electronic Library (Knowledge Management Portal) for access by the stakeholders.
3. Organising quarterly workshops to train stakeholders and innovators.
4. Leveraging on EMRS activities like refresher training etc to disseminate the guidelines.

Appendix A: Readiness Assessment

No.	Service Area e.g. Registration, Eye Clinic, etc.	No. of Available Computers	Is service Area Using the System (Yes/No)	Challenges Faced in that Area	Support Given	No. Trained / Mentored	Next Steps

	Server Assessment											
SN	Type	HDD	RAM	CPU	OS (Windows, Linux)	Service Provider	Support period	Warranty period	Life span	Version of OS	Virtualization Technology	Software applications	Is the server connected to a Backup system?

Server Purpose		
Hardware	Manufacturer	
	Model	
	Support Level	
Operating System		
OS Version		
RAM		
CPU	Type	
	# of Processors	
	Cores/Processor	
	Speed	

Internal Disk	Type, Speed, Size, and # of Disks	
	Total Raw Size (GB)	
	Useable Size (GB)	
	Disk Configuration	
	Free Space (GB)	
External Disk	Type, Speed, Size, and # of Disks	
	Total Raw Size (GB)	
	Useable Size (GB)	
	Disk Configuration	
	Free Space (GB)	
Installed Software		

Appendix B: Hospital Walkthrough and ICT Assessment Tool

Introduction

Health Facility Details	
**Local Government**	
Sub-county/Division	
Village/Ward	
Name Facility	
Facility Level	
Name of Health Facility Director/MS	
Tel No. of Incharge	
Name of IT or Data person	
Tel No. of Contact person	
Delete all the information in read and replace with information in that hospital	

Service Points

Information on Point of service areas (inpatient, administration,wards service areas etc)										
POINT OF SERVICE (Ward name eg Male surgical ward, Pead ward)	No. of beds (where applicable)	Description of work area (e.g consultation, administrative)	Does area requisition commodities from store (Yes/No)	How many health workers are supposed to sit/work in this room	No of existing functional desktops in the room	No. of functional existing Laptops in the room	No. of existing Tablets in the room	No. of Required Computers	No. of Required Laptops	No. of Required Tablets

Wireless LAN Assessment

Wireless/ LAN Access Points								
**Location (Department, Section,)**	**LAN Availability**	**Existing Wireless Points**	**No. of Users**	**Status**	**Indoor (Existing)**	**Outdoor (Existing)**	**Comments**	**No. of Switches Needed**

Appendix C: Sample Training Schedule

TENTATIVE TRAINING SCHEDULE REGIONAL REFERRAL

1. MEETING ADMINISTRATORS {
2. MEETING HEAD OF UNITS
3. TRAINING OPD MODULES
4. TRAINING IPD MODULES
5. RADIOLOGY
6. LAB
7. PHARMACY
8. STORES
9. MORGUE

Date	Module	Department	Time
	• Patient Management<br>• Nursing (administering treatment etc)	Records & Nurses	2:00Pm
	Consultation	Doctors & Clinicians	**Two sessions**<br>Morning from 09:00Am – 12:00pm<br><br>Afternoon Session from 2pm – 4pm
	Radiology	Radiographer	9:00 am – 12 pm
	Lab	All Lab Staff	**Two Sessions**<br>Morning<br>From 9Am – 12Pm<br><br>Afternoon<br>From 2Pm – 4Pm
	• Inpatient & Theatre<br>• Ward Management (NICU, Labour Monitoring, ICU)	Doctors<br>Clinicians<br>Theatre Staff<br>Nurses	**Two Sessions**<br>Morning<br>From 9Am – 12Pm<br><br>Afternoon<br>From 2Pm – 4Pm
	Pharmacy & Dispensing	Pharmacists & Dispensers	**Two Sessions**<br>Morning<br>From 9Am – 12Pm<br><br>Afternoon<br>From 2Pm – 4Pm

	Supply chain Management	All Store Staff	Two Sessions<br>Morning<br>From 9Am – 12Pm
			Afternoon<br>From 2Pm – 4Pm
	Employee Portal (All Staff)	All Ward In-Charges	**Two Sessions**<br>Morning<br>From 9Am – 12Pm<br><br>Afternoon<br>From 2Pm – 4Pm
	Support & Customization Set and Identifying the Focal Person	Hospital IT and Trainers	**Two Sessions**<br>Morning<br>From 9Am – 12Pm<br><br>Afternoon<br>From 2Pm – 4Pm
	Feedback meeting with all the Staffs	Hospital IT and Trainers<br>All Staff	8:30Am

Appendix D: Sample EMRS Deployment Budget

Detailed Budget and Model for Software Deployment for a Single Health Facility							
**1.9**	**Health facility**	**Unit Cost**	**Units**	**Unit of Measure**	**Frequency**	**Unit of Frequency**	**Amount**
1.9.1	**Project Initiation** (Definition of Scope, Team Charter, Project plan, Hospital Workflow Workshop, Project Role and Responsibilities, Computer & Networking Infrastructure Assessment)	175000	2	Persons	1	# of days	350,000
1.9.2	**Solution Design** (Requirements Gathering, Gather Information about end users and Approvers, Price and Service Lists, Inventories, Services Packaging & Insurance Categorization)	175000	2	Persons	2	# of days	700,000
1.9.3	**Staff Training** (Front Office and Outpatients Staff Training, Inpatient Staff Training, Administrative Staff Training, Management Training)	175000	3	Persons	8	# of days	4,200,000
1.9.4	**Implementation** (Server Installation & Configuration, End User Computer Customization, Network Configuration)	175000	2	Persons	3	# of days	1,050,000
1.9.5	**Customization & Realization** (Template Branding, HR & User profiles creation, Accounts Customization, Pricing and Service Packaging, Inventory and Assets Registration, Insurance Customization)	175000	3	Persons	5	# of days	2,625,000
1.9.6	**System Testing** (Outpatient Mock Operation, Inpatient Mock Operation, Billing and Accounting & Human Resource optimization)	175000	2	Persons	1	# of days	350,000
1.9.7	**Project Closure** (Sign-off)	175000	2	Persons	1	# of days	350,000
1.9.8	Fuel	5500	190	Kms/Liters	1		1,045,000
1.9.9	Communication	100000	2	Persons	1	# of days	200,000
1.9.14	**Subtotal**						**10,870,000**

Appendix E: EMRS Rollout Checklist

No	Question	RESPONSE
1	MoH EMRS rollout team	1.<br>2.<br>3.
2	Local Government	
3	Sub County	
4	Facility name	
5	Level	
6	Authority	
7	ED/Director/MS/In-charge	Name:<br>Role:<br>Phone Contact:
8	EMR system used	ð Clinic Master<br>ð eAFYA

Description

The EMRS Rollout planning checklist is intended to aid the trainers in planning for EMRS implementation. It can be used to plan for the EMR system go-live event and to identify any issues that need to be addressed beforehand.

Instructions

Keep updating it daily. A copy to be attached as part of the report.

	Section	Check(☒)	Date	Comment
**1.0**	**Section 1: Team Debrief and movement**	☐		
1.1	Discuss the rollout roadmap and movement plan	☐		
1.2	Team code of conduct	☐		
1.3	Discuss the expected output from the teams (Daily activity reports)	☐		
1.4	Required documents are in place (introduction letter, attendance forms, printed checklist, infrastructure tool etc)	☐		
**2.0**	**Section 2: Inception**			
2.1	Engage the hospital Management which includes the administration, the local government health officials by calling and sending them an email.	☐		
2.2	Hold a meeting with Director/DHO to explain the rollout and required support	☐		
2.2a	Get the hospital Organogram, Staff List (Departmental Staff Lists), Units List (OPD & IPD Units), Hospital Layout (should this be available), Departmental Heads Contact information (name, email, tel, designation, unit) to design training agenda	☐		
2.3	Determine the rollout strategy with hospital head	☐		
2.4	Set date for demo to management and kick off meeting	☐		
2.5	Get Implementing Partner Support List from the hospital	☐		
2.6	Reached out to the district for support (Health facilities)	☐		

2.7	Reached out to Implementing Partners for Support	☐		
**3.0**	**Section 3: Hospital Walkaround and Assessment**	☐		
3.1	Identify and document the different service points, with data points and computers already in place	☐		
3.2	LAN/WLAN Coverage (OPD & IPD): ☐20% ☐40% ☐ 60% ☐ 80% ☐100%	☐		
3.3	Available power options at the facility: ☐Umeme ☐Solar ☐Generator ☐Others…	☐		
3.4	Server/ server room in place and functional	☐		
3.5	IT Equipment in place (Computer, laptops)	☐		
3.6	Count and document the beds in the wards	☐		
3.7	Identify and document the unit stores in the facility	☐		
3.8	Identify and document the hospital workflow	☐		
3.8	Find out the baselines for all units	☐		
3.9	Find out and document the clinic days	☐		
3.10	Look at the physical security (Door locks, burglar proof) and make recommendations	☐		
3.11	Evening Team meetings to review the days' activity and plan for the next day	☐		
**4.0**	**Section 4: Training schedule:**			
4.1	Work with the focal person to Come up with a training schedule	☐		

4.2	Circulate the schedule to the staff by using the staff communication platform/notice board.	☐		
4.3	Set up training room fully loaded with computers			
**5.0**	**Section 5: Entry Kick off Meeting**			
5.1	Set up an EMR **implementation kick off meeting** with the Key stakeholders include but are not limited to: Local Government Health Officials, Hospital Directors/Medical Superintendents, Hospital Administrators, Senior Nursing Officers, Records Personnel, Hospital/Local Government IT Office, a Store representative and all heads of departments<br><br>***Key Agenda Items for the meeting include: identifying a project focal person at the hospital, Confirmation of equipment delivery and distribution plan, Scope of system deployment, Proposed deployment timelines & activity work plan, Roles of the stakeholders involved and a demo of the system***	☐		
**6.0**	**Section 6: Basic ICT Skills Training**			
6.1	Users who need the basic ICT Skills training have been identified	☐		
6.2	Training has been Scheduled	☐		
6.3	Training has been carried out	☐		
**7.0**	**Section 7: EMR System training**	☐		
7.1	Users have been trained according to the schedule by module/cadre.	☐		
7.2	Appointments with users who are not always available at the hospital have been made.	☐		
**8.0**	**Section 8: IT Equipment Installation and distribution/redistribution**			

8.1	Distribution list for the new equipment (Desktop/laptops) has been made with support from the hospital administration.	☐		
8.2	Desktop/laptop setup in different service points	☐		
8.3	Desktop/laptop configuration	☐		
8.4	Connect the computers to the network	☐		
**9.0**	**Section 9: System setup (get support from IT and installation team)**			
9.1	The server room and server have been set up and configured	☐		
9.2	The system has been set up on the Server	☐		
9.3	Thin client server has been setup and configuration (Where applicable)	☐		
9.4	Servers have a power backup	☐		
**10.0**	**Section 10: Go-Live preparation**			
10.1	User accounts have been created for all users	☐		
10.2	Every user has completed the training necessary to use the EMRS	☐		
10.3	Every user has completed basic computer navigation, keyboarding, and other applicable training; provide refresher if necessary	☐		
10.4	The practice support team has been trained and is aware of their roles/functions for go live.	☐		
10.5	Every user has a user ID and password, and they remember them.	☐		
10.6	Every user has been assigned the required rights.	☐		

10.7	Everyone can log on and has the correct privileges.	☐		
10.8	The EMR system has been customized ☐ Stores ☐Lab ☐ Radiology	☐		
10.9	Data has been entered (Stock, supply categories)	☐		
10.10	All service points have the required computers and the EMRS application can be accessed.	☐		
10.11	Communication channels like WhatsApp groups have been set up where users can be supported from.	☐		
**11.0**	**Section 11: Go-Live**			
11.1	Go-live meeting held with staff to communicate the change and go-live date			
11.2	Units to go live fast have been communicated			
**12.0**	**Section 12: Post Go-Live**			
12.1	User Hand Holding and mentorship on workstations	☐		
12.2	EMR System User support	☐		
**13.0**	**Section 13: Closure Meeting with HODs**			
13.1	Shared a report and showing the performance so far.	☐		
13.2	Discussed how the HODs are going to support the users in absence of the MOH team.	☐		

Appendix F: Standard Operating Procedure for EMR System Downtime

Republic of Uganda
Ministry of Health

Standard Operating Procedure (SOP) for Handling Electronic Medical Record (EMR) System Downtime

Objective:

This Standard Operating Procedure (SOP) outlines the protocol to be followed by all health workers when an Electronic Medical Record (EMR) System is down to ensure continuity of care, patient safety, and data integrity.

Key definitions

- *Downtime:* Is a period during which production or business processes come to a halt due to application/ system unavailability, technical glitch, network outage or natural disaster [Adam Marget et al]
- *Uptime:* Is a measure of an application/system's availability to its end users
- *Downtime procedure:* refers to a predefined set of steps that healthcare organisations follow during system or network downtime.

Types of downtime

Planned vs. Unplanned Downtime

Unplanned downtime (also known as unscheduled downtime) is when a lapse in operations occurs because of an unplanned machine, network outage, power outage,

natural disaster or server error. It’s outside your control and doesn’t abide by the institution's schedule.

Planned downtime (scheduled downtime) is when you schedule these down periods at a convenient time to the institution and minimize any negative impact for the users. It’s scheduled, proactive maintenance that allows you to install upgrades and perform routine maintenance in order to ensure optimal functionality of machines and services. This can include replacing old or outdated machine parts, performing regular system updates and patches, and a wide range of other tasks intended to increase the reliability of services.

Types of Planned Downtime

a) Fixed downtime: This adheres to a set schedule- you determine a specific start and stop time for maintenance/ recovery to occur.

b) Flexible downtime: Provides for a window of time during which downtime shall happen, though the exact start time is unknown

Causes of Downtime

There are several causes of downtime. Some of the main causes are explained below:

a) Human Error: Regardless of whether accidental or due to negligence, human error is one of the most common causes of unplanned downtime.

b) Hardware/Software Failure: Obsolete hardware or software increases the chances of application failure and system outage.

c) Device Misconfiguration: Device misconfiguration is another major cause of unplanned downtime.

d) Bugs: Bugs in a server's operating system can impact its performance as well as lead to security issues.

e) Cybersecurity Threats: Cyber threats, including sophisticated ransomware and phishing attacks, are one of the most dangerous and common causes of IT downtime.

f) Natural Disasters: Natural disasters, such as hurricanes, floods and earthquakes, can disrupt power supply and communication or even damage hardware.

How to Prepare for EMRS Downtime

1. Be prepared for EMRS downtime (Be expectant)
2. Train for downtime from the beginning.
3. Create an incidence response downtime plan
4. Practice for an outage
5. Designate a person to document during EMRS downtime
6. Run backups and store backups in different locations
7. Know who to contact in the event of a data breach

Scope:

This SOP applies to all health facilities with an EMR system for patient/client care management.

Responsibilities:

1. **Clinical Staff:**
    a. Adheres to downtime procedures and documentation protocols outlined in this SOP.
    b. Continues to provide patient care using the relevant paper-based processes and registers during downtime.
    c. Communicates effectively with other health workers and patients/clients to ensure continuity of care.
2. **IT Officer/EMRS Focal Person:**

a. Monitors system performance and initiates downtime protocols when necessary.
b. Documents and reports EMR system downtime to the national IT support team using the relevant ticketing system (EMRS Help Desk) or official communication channels in place.
c. Coordinates with the national IT support team and vendors to troubleshoot and resolve downtime issues.

3. **Health Facility In charge**
    a. Oversees the implementation and maintenance of EMR system downtime procedures.
    b. Ensures staff training and readiness for EMR system downtime.
    c. Coordinates with national IT support team and EMR system vendors for technical support and resolution of downtime issues.

Procedure:

1. Initiating Downtime Protocol:
    i. Upon identification of an EMR system failure or outage, the IT Officer/EMRS Focal Person shall assess the situation and determine if downtime procedures need to be activated.
    ii. If downtime procedures are required, the IT Officer/EMRS Focal Person shall initiate the downtime protocol and notify clinical staff via the designated communication channels immediately.
2. Notification and Communication:
    i. Clinical staff shall be notified immediately of EMR system downtime through the health facility's designated communication system (e.g., WhatsApp group, phone call, email, text alerts).
    ii. Clear communication shall be provided regarding the expected duration of downtime and alternative documentation procedures.
3. Transition to Downtime Procedures:
    i. Clinical staff shall transition to paper-based Health Management Information System (HMIS) documentation (registers, forms and reports) and manual processes for patient/client care during downtime.
    ii. HMIS pre-printed paper tools shall be made available at all points of care for use during downtime.
4. Patient Identification and Safety:

    i. Clinical staff shall verify patient identification using standard paper protocols to ensure patient safety during downtime.
    ii. Allergies, medications, and other critical patient information shall be verified verbally with patients or caregivers during downtime.
5. Documentation and Record Keeping:
    i. Clinical staff shall document patient care activities, assessments, medications, and other relevant information on the HMIS paper-based tools.
    ii. Data captured on the paper tool shall be transferred to the EMRS within 24 hours once the system is restored.
6. Continuity of Care:
    i. Clinical Staff shall collaborate effectively with the EMRD focal person to ensure continuity of care and smooth transitions between shifts during downtime.
    ii. Critical patient information shall be communicated verbally during handoffs and shift changes to ensure seamless care delivery.
7. Monitoring and Resolution:
    i. The IT Officer/EMRS Focal Person shall monitor the status of the EMR system and work with the national IT support staff and vendors to resolve downtime issues promptly.
    ii. Regular updates shall be provided to the clinical staff regarding the progress of downtime resolution and expected system restoration times.
8. Post-Downtime Review:
    i. Following the resolution of EMR system downtime issues, a post-downtime review shall be conducted to assess the effectiveness of downtime procedures and identify opportunities for improvement.
    ii. Lessons learned from EMR system downtime incidents shall be documented, and recommendations for system enhancements or staff training shall be implemented as appropriate.
9. Training:
    i. All clinical staff shall receive training on EMR downtime procedures during orientation and regularly scheduled training sessions.
    ii. Updates and refresher training on EMR system downtime procedures shall be provided as needed to ensure staff readiness and competence.
10. Documentation and Recordkeeping:

i. Documentation of EMR system downtime incidents, including timelines, actions taken, and resolutions, shall be maintained at the health facility or using the systems' issues tracker/ticketing system.
ii. Incident reports and documentation logs shall be reviewed periodically for quality assurance and compliance purposes.

11. Document Review and Approval:

Version	Owner	Author	Publish Date	Name/Title	Signature
1	Ministry of Health	DHIM	12/01/2023	xxx	
2					

12. Dissemination:

This SOP shall be disseminated to all EMR system-implementing health facilities and relevant stakeholders and made accessible through the Ministry of Health's Knowledge Management Portal (KMP). A copy shall also be made available on the Health Facilities' notice boards.

13. Contact the Ministry of Health

Any questions, inquiries or emergencies to raise to the Ministry of Health shall be through the;

a) The Call Center Toll-Free 0800-100-066
b) E-mail: Email: info@health.go.ug
c) Address: Plot 6, Lourdel Road, Nakasero P.O Box 7272, Kampala Uganda.
d) Website: www.health.go.ug/

Appendix G: EMRS Support Structure

Support Tiers	Description	Support team & Resources	Support needs
**Tier 0:**<br><br>*Self-help or end-user*	Users identify issues, retrieve support information and attempt to resolve the issue.<br><br>If they fail to resolve the issue, they report the issue to tier 1 support	**Support team**<br><br>- End users<br>- Peer support<br>**Resources**<br><br>- SOPs<br>- User manual<br>- FAQs<br>- Community of practice<br>- Support channels	This level shall require self-support resources to be created, maintained, and updated to support users.<br><br>Tier 1 personnel shall receive and respond to requests through the available channels.
**Tier 1:**<br><br>**Basic technical support**	Support for basic user issues such as solving usage problems and simple user requests that need HIS team involvement.<br><br>If no solution is available, tier 1 personnel escalate incidents to a higher tier.	**Support teams**<br><br>- Records Assistants<br>- Health Information Assistants (HIA)<br>- Medical Records Assistants<br>- Data Clerks<br>**Resources**<br><br>- SOPs<br>- FAQs<br>- Community of practice<br>- User manual<br>- Support channels	Lower-level technical personnel shall be trained to solve known problems and respond to requests using standard guidelines and procedures
**Tier 2:**<br><br>**Experienced technical support**	Experienced and knowledgeable technicians assess issues and provide solutions for problems that cannot be handled by tier 1.<br><br>If no solution is available, tier 2 support escalates the incident to tier 3.	**Technical teams**<br><br>- Regional Referral Biostats<br>- Regional Bio-stat representative<br>- HMIS HSD managers<br>- District Biostatisticians<br>- IP HMIS managers<br><br>**Resources**<br><br>- SOPs<br>- Technical and end-user manuals<br>- Support channels	Support personnel with technical working experience and knowledge of the solution, not necessarily the engineers or programmers of the solution.

**Tier 3:**<br><br>**Expert technical support**	Highly experienced or knowledgeable or national experts and technicians assess issues and provide solutions for problems that cannot be handled by tier 2.<br><br>They duplicate problems and understand possible causes and attempt to provide solutions to the problem.<br><br>If no solution is available, tier 3 support escalates the incident to tier 4.	**Technical teams**<br><br>- MoH HMIS Support Team<br>- HIS support teams<br>- Consultants<br>**Resources**<br><br>- SOPs<br>- Technical manuals<br>- HIS Support channels	Support personnel with high technical working experience, expert knowledge of the solution but may not necessarily be the engineers or programmers who designed and created the solution.
**Tier 4:**<br><br>**Developer technical support**	This is the highest technical resource available for problem resolution.<br><br>The technicians attempt to duplicate problems and define root causes, using product designs, code, or specifications and make new fixes.	**Technical teams**<br><br>- Consultants<br><br>**Resources**<br><br>- Official emails<br>- HIS Support channels	Highly skilled product specialists, and may include the creators, chief architects, or engineers who created the product or solution.

Appendix H: System Change Request Form

THE REPUBLIC OF UGANDA
MINISTRY OF HEALTH

Ministry of Health Information and Communications Technology Section System Change Request Form

<table>
<tr><td colspan="4">Change Request ID: ___________________ Report ID: ____________________</td></tr>
<tr><td colspan="4">Originator/Requester (Name, Position and Contact):</td></tr>
<tr><td colspan="4">Department/Consultant/Partner:</td></tr>
<tr><td>Problem Statement: Provide a brief description of the requested change, for upgrades indicate current and target builds/versions</td><td colspan="3"></td></tr>
<tr><td rowspan="2">Where & When Changed Requested:</td><td>Date</td><td>Location</td><td>Time</td></tr>
<tr><td></td><td></td><td></td></tr>
<tr><td>Description of Problem: Provide a detailed description of the problem, circumstances leading to the requested change</td><td colspan="3"></td></tr>
<tr><td>Supporting Information: Provide screenshots of an error or printout of an error in a document / report</td><td colspan="3"></td></tr>
<tr><td>Reasons and Justification: Describe the reason why the change has been requested and the justification for the request.</td><td colspan="3"></td></tr>
<tr><td>Affected Areas:
According to the Perception of the Requester</td><td>System / Workflow Affected</td><td>Subsystem / Workflow Affected</td><td>Documentation Affected</td></tr>
</table>

Downtime Implication: *Will the change cause/require system downtime*			
Alternate Actions: *Describe alternatives to the change according to the Perception of the Requester*			
Priority to Implement: *Describe priority assigned by the requester*			
Approvals			
Change Requester *(Role, Name and signature)*			
Head User Department/Division/Section *(Department Name and Signature)*			
Head Health Information Management Division *(Name and Signature)*			
Head ICT Section *(Role, Name and signature)*			
Assignment			
Engineer Assigned to execute *(Role, Name)*			
Engineer Notes			

Appendix I: EMRS Guidelines Use Monitoring Framework

Guideline-Use Monitoring Framework
**Parameter 1:** Dissemination
**Key Monitoring Questions:**<br>*1.1 What is the version of the current guideline document?*<br>*1.2 Has the current guideline document been disseminated?*<br>*1.3 If yes under [1.2] above, through which channels?*<br>*1.4 What is the proportion of target entities possessing at least one copy of the guideline document? – disaggregated by the National, District, Health Facility, Partners.*
**Indicator:**<br>*% of target entities who received at least one copy of the guideline document, disaggregated by: (a) National; (b) District; (c) Health Facility; (d) Partners*
**Means of Verification:**<br>*Reports (e.g., dispatch, supportive supervision)*
**Result Statement:**<br>*Existing version of the Guidelines for Implementing the Uganda electronic Community Health Information System disseminated to key stakeholders nationwide.*
**Parameter 2:** Training
**Key Monitoring Questions:**<br>*2.1 Has training on the current version of the guidelines been conducted?*<br>*2.2 If yes under [2.1], what was the proportion of individuals trained on the various components of the guideline document? – disaggregated by (a) National; (b) District; (c) Health Facility; (d) Partners*
**Indicator:**<br>% of target entities oriented on guideline document, disaggregated by: (a) National; (b) District; (c) Health Facility; (d) Partners
**Means of Verification:**<br>Reports (e.g., training, supportive supervision)
**Result Statement:**<br>*Key stakeholders nationwide oriented on the existing version of the Guidelines for Implementing the Uganda electronic Community Health Information System.*
**Parameter 3:** Compliance
**Key Monitoring Questions:**<br>*3.1 Are the various target groups implementing EMRS in accordance with established guidelines? Please provide comment by target group and key area as appropriate*<br>● ***Target Group:*** *(a) National; (b) District; (c) Health Facility; (d) Community Health Workers; (e) Partners*<br>● ***Key Component:*** *(a) EMRS User Requirements, Design and Development; (b) Implementing EMRS; (c) System and Data Access; (d) Governance Structure (e)Monitoring and Evaluation*
**Indicator:**<br>*Target entities are compliant with existing versions of the guidelines – disaggregated by (a) National; (b) District; (c) Health Facility; (d) Partners.*<br>*Use Likert scale: 1-5, where 1 = strongly agree, 2 = agree, 3 = neutral, 4
**Means of Verification:**<br>Reports (e.g., design workshop, training, supportive supervision)

**Result Statement:**<br>*Key stakeholders nationwide are compliant with existing Guidelines for Implementing the Electronic Medical Records System.*

Appendix J: EMRS Deployment Monitoring Framework

**EMRS Deployment Monitoring Framework**
**Result Statement:**<br>*By 2025, the health sector has institutionalized the use of patient-level digital systems at the point of care.*
**Parameter 1: Functionality**
**Key Monitoring Questions:**<br>*1.1 Does the technology or system work?*<br>*1.2 Does the technology or system operate as intended?*<br>*1.3 Does the technology or system perform its intended functions effectively?*<br>*1.4 Is the technology effectively adapted to the local context in terms of language, literacy, modifications for network coverage, etc?*
**Indicator List:**<br>1. *% service points of care with a functional computing device/computer/laptop at the time of deployment*<br>2. *% service points of care within a health facility with access to a power source for powering of the EMRS*<br>3. *# of alternative functional power sources in place*<br>4. *% end-users with access to local technical support for troubleshooting*<br>5. *% devices that are not currently operational (misplaced/broken/not working)*<br>6. *% end-users who are literate in the language used by the digital health intervention*<br>7. *% data fields or elements from original paper-based system that are captured by the technology*<br>8. *# hours of initial training on the use of the EMRS attended by end-users*<br>9. *# hours of refresher training on the use of the EMRS attended by end-users*
**Means of Verification:**<br>*Reports (e.g., data/performance reviews, supportive supervision, regional/national telecommunication reports, surveys)*
**Parameter 2: Stability**
**Key Monitoring Questions:**<br>*2.1 Does the system consistently operate as intended?*
**Indicator List:**<br>1. *# hours of system/server downtime over reference period (i.e., last 1 month or 3 months)*<br>2. *% end-users reporting successful synchronization with server over reference period (i.e., last 1 month or 3 months)*<br>3. *% end-users reporting failed synchronization with server over reference period (i.e., last 1 month or 3 months)*
**Means of Verification:**<br>*Reports (e.g., system-generated/audit trail)*
**Parameter 3: Fidelity**
**Key Monitoring Questions:**<br>*3.1 How do people interact with technology or systems?*<br>*3.2 Do the realities of the field implementation alter the functionality and stability of the system, changing the EMRS intervention from that which was intended?*<br>*3.3 Has the digital health system or technology been widely adopted?*<br>*3.4 Do the users find the technology easy to use?*<br>*3.5 Do the end-users find the health data/information received from the EMRS intervention useful?*<br>*3.6 Are the end-users able to communicate with the EMRS as intended?*

3.7 Are the end-users responsive to the information received through the system?

Indicator:

1. *% end-users that pass the assessment test during the training phase.*
2. *% end-users who demonstrate proficiency in use of the EMRS*
3. *% intended end-users observed using the EMRS*
4. *# transmissions sent by intended end-users over reference period (i.e., last 1 month or 3 months)*
5. *% end-users who rate the EMRS as "easy to use"*
6. *% end users who rate the EMRS as "transmits information as intended"*
7. *% end-users who report satisfaction with the content of health data/information received via the EMRS*
8. *% end-users motivated/intending to use the EMRS*
9. *# forms/amount of data transmitted by end-users via EMRS within a reference period (i.e., last 1 month or 3 months)*
10. *% data fields or elements/forms that are left incomplete over reference period (i.e., last 1 month or 3 months)*

Means of Verification:

Reports (e.g., system-generated/audit trail, design workshop, training, data/performance reviews, supportive supervision)

Parameter 4: Quality

Key Monitoring Questions:

4.1 Is the content and the delivery of the EMRS intervention of high enough quality to yield intended outcomes?
4.2 How does EMRS improve service delivery?
4.3 How do improvements in service delivery affect health outcomes?

Indicators:

Client-Level;

1. *# minutes (reported or observed) between EMRS prompt received about programmatic intervention and seeking care from provider (e.g., HW, midwife, nurse, clinician)*
2. *# days duration of illness episode: disaggregated by illness/condition*
3. *# minutes spent with HW in relation to health intervention at the last visit*
4. *% target individuals or caregivers who report contact with a qualified health-care provider using EMRS in relation to a programmatic intervention over reference period*
5. *% target individuals or caregivers who report adequate knowledge about signs and symptoms*
6. *% target individuals or caregivers who report adequate knowledge about the health issues relevant to a programmatic intervention*
7. *% changes in reported individual level out-of-pocket payments for illness management over reference period (through managing the illness by phone-based consultation instead of visiting a health care facility, e.g., travel cost)*

Provider-Level;

1. *# minutes (reported or observed) for last client counseling about a health intervention using EMRS*
2. *# minutes or hours (reported or observed) spent on health record-keeping about a health intervention over reference period*
3. *# minutes (reported or observed) used per individual HW to report important adverse events (e.g., stock-outs)*
4. *# of HWs who report adequate knowledge of the health issue relevant to a health intervention*

5. *% care standards relating to a health intervention observed to be met using EMRS during client-provider consultation*
6. *% HWs observed to be using EMRS during their patient consultations*
7. *% target HWs who use EMRS in relation to relevant health interventions through tablets over reference period*
8. *Amount of cost savings (estimated) due to improvement in service delivery/efficiency/other factors.*
9. *# clients (average or total) attended by a HW using EMRS over reference period*

Health System-Level;

1. *# minutes (cumulative) over reference period for all HWs using EMRS to enter data related to a health intervention*
2. *# days over reference period for which a HW reports stock-out of a commodity essential for provision of a health programmatic intervention*
3. *% change in reported stock-out events of a commodity essential for service delivery over reference period, disaggregated by relevant health interventions*
4. *% change in data entry errors over reference period*
5. *% target end-users who receive training on management and use of EMRS to deliver quality service delivery, disaggregated by initial and refresher training*
6. *# individuals seeking health care over reference period, disaggregated by relevant health interventions*
7. *% individuals in a specific geographical area who receive health care through EMRS over reference period, disaggregated by relevant health interventions*
8. *% change in costs of transporting HMIS paper forms and manual data entry over reference period*
9. *% change in costs of human resources for data entry*
10. *% change in costs associated with timely and appropriate management of illness*
11. *% changes in reported individual out-of-pocket payments for management of illness*
12. *Total population-level savings in out-of-pocket payments attributed to timely and appropriate care seeking*

Means of Verification:
Reports (e.g., system-generated/audit trail, design workshop, training, data/performance reviews, supportive supervision)

Appendix K: EMRS Evaluation Framework

EMRS Evaluation Framework		
**Formative**	**Evaluation Type:** Process Evaluation<br>**Objective:** Measure outputs attributed to EMRS intervention activities and inputs; done either as a one-time assessment and/or continuously.<br>**Key Question(s):**<br>● Is the EMRS intervention operating as intended?	**Timeline:**TBD
	**Evaluation Type:** Implementation Evaluation<br>**Objective:** Monitor the fidelity of the EMRS intervention holistically or technology system.<br>**Key Question(s):**<br>● Is EMRS implementation occuring in accordance with the original and existing implementation guidelines and standard operating procedures and/or protocols?	**Timeline:**TBD
**Summative**	**Evaluation Type:** Performance or Outcome Evaluation<br>**Objective:** Measure the effectiveness of EMRS intervention activities on immediate and intermediate changes in key outcomes, including knowledge, service provision, utilization and coverage.<br>**Key Question(s):**<br>● Are the health services available? What is EMRS intervention's effect on changes in service delivery?<br>● Are the health services being utilized?<br>● Did EMRS increase coverage of the relevant health interventions? Is the target population being reached?	**Timeline:**TBD
	**Evaluation Type:** Impact Evaluation<br>**Objective:** Measure the long-term net effects or impact of the intervention on key health outcomes, including mortality, morbidity, and disease risk, at the health facility level or higher.<br>**Key Question(s):**<br>● Were there improvements in disease or mortality patterns, or health-related behaviors?	**Timeline:**TBD
	**Evaluation Type:** Economic Evaluation<br>**Objective:** Determine a probable value for money from investment made in EMRS roll-out.<br>**Key Question(s):**<br>● What is the incremental cost-effectiveness of the EMRS intervention as compared to existing services?	**Timeline:**TBD

REPUBLIC OF UGANDA
MINISTRY OF HEALTH

GUIDELINES FOR THE IMPLEMENTATION OF THE UGANDA ELECTRONIC COMMUNITY HEALTH INFORMATION SYSTEM (eCHIS)

January 2023

DOCUMENT REVIEWS AND APPROVALS

Version	Owner	Author	Approval Date
1	Ministry of Health	Division of Health Information Management	18/09/2024

Table of Contents

Foreword **vi**

Preface **vii**

Acknowledgement **viii**

Abbreviations and Acronyms **ix**

Key Definitions **x**

1.0 Introduction **2**

1.1 Background **2**

1.2 Vision for eCHIS 2

1.3 Purpose 2

1.4 Targeted User of eCHIS 3

1.5 Methodology 4

1.6 Guiding Principles Used to Develop this Document 4

1.7 Policy, Reference Guidelines and International Standards 4

1.8 Revision and Updates 4

2.0 eCHIS User Requirements, Design and Development **5**

2.1 Overall Architecture 5

2.2 Personas of eCHIS 6

2.3 System Functions 9

2.4 Design Process 10

2.4.1 eCHIS Design Principles 11

2.5 eCHIS Development Process 11

2.6 System Testing 12

3.0 Implementing eCHIS **14**

3.1 Pre-Implementation Requirements. 14

3.2 eCHIS Deployment Phase 15
3.2.1 District eCHIS Work Plan 15
3.2.2 eCHIS Deployment 15
3.3 eCHIS Post-Implementation Support 17
3.3.1 Continuous Capacity Building and Mentorship 17
3.4 Transitioning from Other Community Health Applications to eCHIS 19
4.0 System and Data Access 20
5.0 Governance Structure 21
5.1 Implementation Arrangements 22
6.0 Monitoring and Evaluating the Guidelines 25
6.1 eCHIS Implementation Guideline Use Monitoring 25
6.2 eCHIS Deployment Monitoring 25
6.3 Evaluating the electronic Community Health Information System 27
6.4 Reporting, Dissemination and Adoption of the Guidelines 28
References 30
Appendix 1: eCHIS Readiness Assessment Checklist 31
Appendix 2: Device Management Agreement Template for CHW Supervisor 32
Appendix 3: Device Management Agreement Template for CHWs 36
Appendix 4: CHW Solar System Agreement Template 42
Appendix 5: eCHIS Deployment Checklist 46
Appendix 6: Sample TOT Training Schedule 48
Appendix 7: Sample CHW Training Schedule 50
Appendix 8: eCHIS Deployment Budget Template 51
Appendix 9: Change Review Form 52
Appendix 10: Monitoring and Evaluation Checklist - Community Level 53

Appendix 11: eCHIS Deployment Monitoring Framework **55**

Appendix 12: eCHIS Guidelines Use Monitoring Framework **59**

Appendix 13: eCHIS Evaluation Framework **60**

Appendix 13: Standard Operating Procedure for eCHIS Downtime **61**

Foreword

This document presents electronic Community Health Information System (eCHIS) implementation guidelines for the health sector. These guidelines are intended to standardize the implementation of the eCHIS across Uganda's health system. These guidelines are aligned with the Health Information and Digital Health Strategic Plan 2020/2021-2024-2025, National Community Health Strategy 2020/2021-2024-2025 and Ministry of Health Strategic Plan 2020/2021-2024-2025.

The Ministry of Health (MoH) is committed to improving the application of digital health technologies to facilitate the attainment of its overall objective of delivering high-quality health services to all citizens. This aligns with the call of Uganda Vision 2040 and the National Development Plan (NDP) III 2020/21 – 2024/25 which require sectors to adopt Information Communication Technologies (ICTs) to optimize service delivery.

The eCHIS therefore, is critical for improving the quality of health service delivery at the community level through the use of embedded decision support tools and passive data capture. These guidelines aim to support all stakeholders in the implementation and scale-up of the eCHIS within the healthcare ecosystem.

These guidelines shall serve as a framework to ensure the proper governance and leadership, rollout and maintenance of the eCHIS while ensuring its sustainability.

All stakeholders are therefore called upon to adopt and use these guidelines while implementing the eCHIS.

....

Dr. Henry G. Mwebesa

DIRECTOR GENERAL HEALTH SERVICES

Preface

The electronic Community Health Information System (eCHIS) implementation guidelines are a major milestone in the journey towards quality, responsive, accessible, and cost-effective healthcare service delivery at the community level. A well-developed digital health information system is a fundamental and crucial component of any health system.

The use of the eCHIS is not only a key enabler of direct patient care but also a vital tool in health program monitoring. Therefore, it requires the necessary attention and well-planned investment of resources to realise its function. The electronic Community Health Information System (eCHIS) implementation guidelines generally align with the goals, and strategies stipulated in the Uganda Health Information and Digital Health Strategic Plan 2020/21-2024/25.

In the past decade, health service delivery in Uganda has registered tremendous improvement. This can be partially attributed to increased funding, technology evolution and use in the community health space, a transformation which has been progressively acknowledged by the Ministry of Health (MoH), its partners and other key stakeholders. The eCHIS shall be implemented as a digital job-aid for community health workers with reporting as a passive process hence improving the quality of health service delivery at the community level.

All stakeholders are therefore called upon to examine the eCHIS implementation guidelines, assess their involvement, and thereafter align their present and future Standard Operating Procedures with the guidelines laid out in this document.

....

Dr. Byakika Sarah

Commissioner, Health Services

Department of Planning, Financing and Policy

Acknowledgement

The Ministry of Health expresses its profound gratitude to all divisions, departments and programs, members of the Health Information Innovation and Research Technical Working Group and Health Data Collaborative Subcommittee who contributed technical inputs leading to the successful completion of this document. Special appreciation goes to the staff within the Division of Health Information Management (DHIM), Information Communication Technology (ICT) Section and the Community Health Department for the overall guidance to ensure that the guidelines are aligned with the Health Information and Digital Health Strategic Plan 2020/21-2024/25 and the National Community Health Strategy 2020/21-2024/25.

I acknowledge and thank all development and implementing partners that provided financial and technical support for this process, specifically UNICEF, PATH, Living Goods, BRAC, Malaria Consortium and Medic. DHIM is grateful for all the support, sacrifice and contribution invested in the successful development of these guidelines.

Finally, the Ministry of Health is grateful to the Local Governments and all those institutions and individuals who have not been specifically mentioned above, but who directly or indirectly contributed to the successful development and finalization of these eCHIS implementation guidelines.

....

Mr. Paul Mbaka

Assistant Commissioner Health Services

Health Information Management

Abbreviations and Acronyms

DHIM	Division of Health Information Management
eCHIS	Electronic Community Health Information System
HDC	Health Data Collaborative
HIIRE	Health Information Innovation and Research Technical Working Group
ICT	Information Communication Technology
MoH	Ministry of Health
SDLC	Software Development Life Cycle
SRS	Systems Requirements Specifications
TWG	Technical working group
UAT	User Acceptance Testing

Key Definitions

Community - A Specific group of people, usually living in a defined geographical area, who share common values, norms, culture, and customs, and are arranged in a social structure according to relationships which the community has collectively developed over a period of time.

Community Health Worker (CHW) - Frontline public health workers and aides living and working in the community. They are selected and trained, on a variety of tasks such as home visits, sanitation, first aid, MNCH and family planning activities, TB and HIV/AIDS care etc.

Village Health Team (VHT) - The Village Health Team is a voluntary community-based (village) structure whose members are selected by the people themselves to promote the health and well-being of the people in their areas of residence/jurisdiction. It is the lowest health delivery structure and serves as a Health Centre I.

Digital Health - Digital health is the systematic application of information and communications technologies, computer science, and data to support informed decision-making by individuals, the health workforce, healthcare organizations, and health systems, to strengthen resilience to disease and improve health and wellness.

Community Health Information System (CHIS) - This is an integrated information system that supports health service delivery at the community level. The system comprises both hard copy and electronic health management information systems.

Electronic Community Health Information Systems (eCHIS) - The electronic Community Health Information System (eCHIS) is a digital job aid for community health workers which also digitizes the paper-based health information management system and programme workflows.

Workflows - A sequence of steps or processes from initiation to completion during health service delivery.

Data Element - A basic unit of information that has a unique meaning and subcategories (data items) of distinct value for example gender, race, and geographic location.

Indicator - A quantitative or qualitative metric that provides information to monitor performance, measure achievement and determine accountability.

1.0 Introduction

1.1 Background

The Ministry of Health Strategic Plan 2020/21 - 2024/25 stipulates two service delivery platforms; the Facilities and the Community. The community platform supported by Community Health Workers is responsible for household production and modifying the social determinants of health.

The National Community Health Strategy 2020/21 - 2024/25 and the Uganda Health Information and Digital Health Strategic Plan 2020/21 - 2024/2025 both stipulate strategic direction towards digitizing community health workflows to improve health service delivery and data quality. This is aimed at reducing the burden of reporting stemming from the use of paper-based tools and subsequent data quality issues. Significant improvements in community service delivery have been reported in districts implementing the electronic Community Health Information System (eCHIS). The main achievements registered so far are (1) reduced reporting burden and (2) improved data quality.

However, the processes related to implementation such as service workflow re-engineering, functionalization of health analytics, meaningful information exchange, and governance of eCHIS have been mostly undefined to date. As Uganda continues on her journey to implement eCHIS nationwide, there is a need for implementation guidelines to direct operationalization and support the achievement of intended results.

1.2 Vision for eCHIS

Community programming in Uganda driven by evidence, leveraging digital health solutions to improve health service delivery.

1.3 Purpose

The guidelines seek to establish an effective, transparent and accountable framework for implementing the electronic Community Health Information System.

General Objective

To standardize the processes and implementation of the electronic Community Health Information System in the health sector.

Specific Objectives:

1. Formulate and functionalize an appropriate governance framework for the implementation of eCHIS.
2. Define and standardize the processes for introducing, scaling, transitioning and sustaining the eCHIS.
3. Define and aggregate functionality existing in different program and function-specific digital tools into a single government-owned electronic community health information system.
4. Ensure interoperability of the eCHIS with other Ministry of Health data systems to facilitate efficient referral, commodity tracking, reporting and surveillance.
5. Establish appropriate change management strategies to foster the use of the eCHIS.
6. Establish standard data use strategies and tools.

1.4 Targeted User of eCHIS

The targeted Users of eCHIS include the following:

- The Ministry of Health Departments and Programs
- Local Government Leadership
- District Health Teams
- Facility Health workers
- Community Health Workers
- Technology and Implementing Partners
- Data Managers and M&E-related staff involved in the management of HMIS data
- Development Partners and Donors/Funders who provide resources for implementation.
- Health Policymakers
- Community Health Program/Project Managers and for
- Researchers
- Other relevant Ministries, Departments and Agencies.

1.5 Methodology

A highly consultative approach was used in the development of the eCHIS guidelines. Stakeholders from various entities such as the Ministry of Health (MoH), Local Governments (LGs), Development Partners, Technology and Implementing Partners, Academia and other members of the Health Information Innovation and Research Technical Working Group (HIIRE TWG) supported the generation of a draft which was reviewed and later validated.

The validated guidelines were endorsed by the Ministry of Health's HIIRE TWG and the Senior Management Committee (SMC), and approved by Top Management for implementation within the health sector.

1.6 Guiding Principles Used to Develop this Document

1. Client Centered
2. Equity
3. Privacy and Integrity
4. Efficiency
5. Transparency and Accountability

1.7 Policy, Reference Guidelines and International Standards

These guidelines are premised on the following existing frameworks;

- The National Health Policy (NHP) III
- Ministry of Health Strategic Plan 2021/2025
- National Community Health Strategy 2020/21 - 2024/25
- Uganda Health Information and Digital Health Strategic Plan 2020/21 - 2024/2025
- Community Acceleration Roadmap

1.8 Revision and Updates

These guidelines shall be reviewed and any proposed changes documented annually to maintain relevance and/or responsiveness to an evolving healthcare ecosystem and context. Relevant sections that will need to be added to the document shall follow the standard MoH approval processes. A new version number and date of approved updates shall be documented.

2.0 eCHIS User Requirements, Design and Development

This section details the process for the generation of user requirements that inform the system functionality and subsequent iterative system development and/or enhancement. The Division of Health Information Management (DHIM) shall coordinate the user requirements, system design and development process and provide overall guidance during this phase with oversight from the HIIRE TWG. During this phase, the MoH ICT Unit shall be engaged to assess and guide on the system hosting requirements as well as other technical inputs such as security.

2.1 Overall Architecture

The eCHIS is part of the wider Uganda Health Information System architecture ensuring interoperability of the eCHIS with other Ministry of Health data systems to facilitate referrals, commodity tracking, reporting, analytics, and surveillance (including linkage to the HMIS).

The eCHIS fits within the National Digital Health Enterprise Architecture indicated in Figure 1 below. The eCHIS falls under the applications segment within the architecture. For more details, reference can be made to the Uganda Digital Health Enterprise Architecture[1] , Standards and Knowledge Products Guidelines.

[1]Uganda Digital Health Enterprise Architecture, Standards and Knowledge Product Guidelines

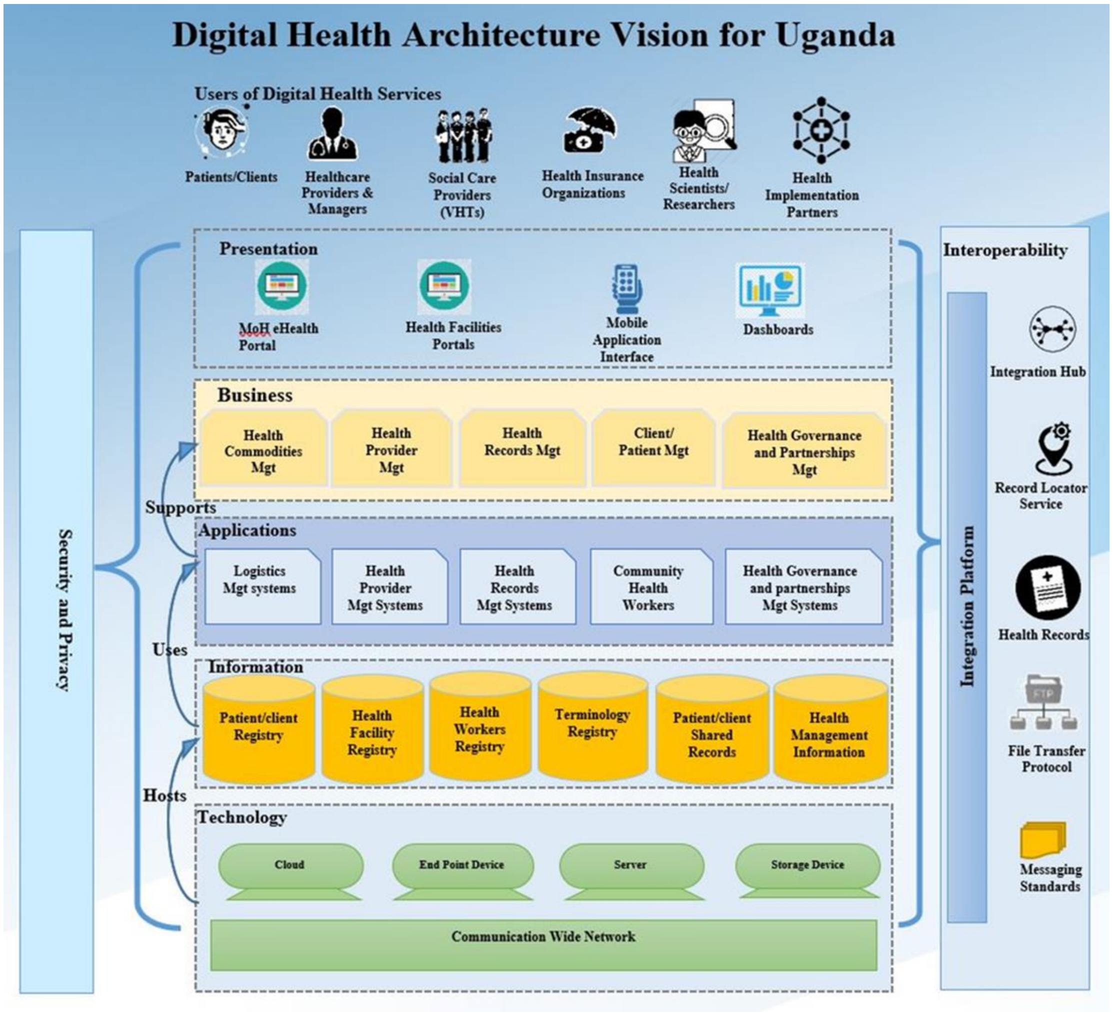

Figure 1: Uganda Digital Health Enterprise Architecture

2.2 Personas of eCHIS

The delivery and management of community health services involves multiple actors as depicted in Figure 2, who play different but complementary roles. The following subsections describe the characteristics and roles of each of the personas of the eCHIS.

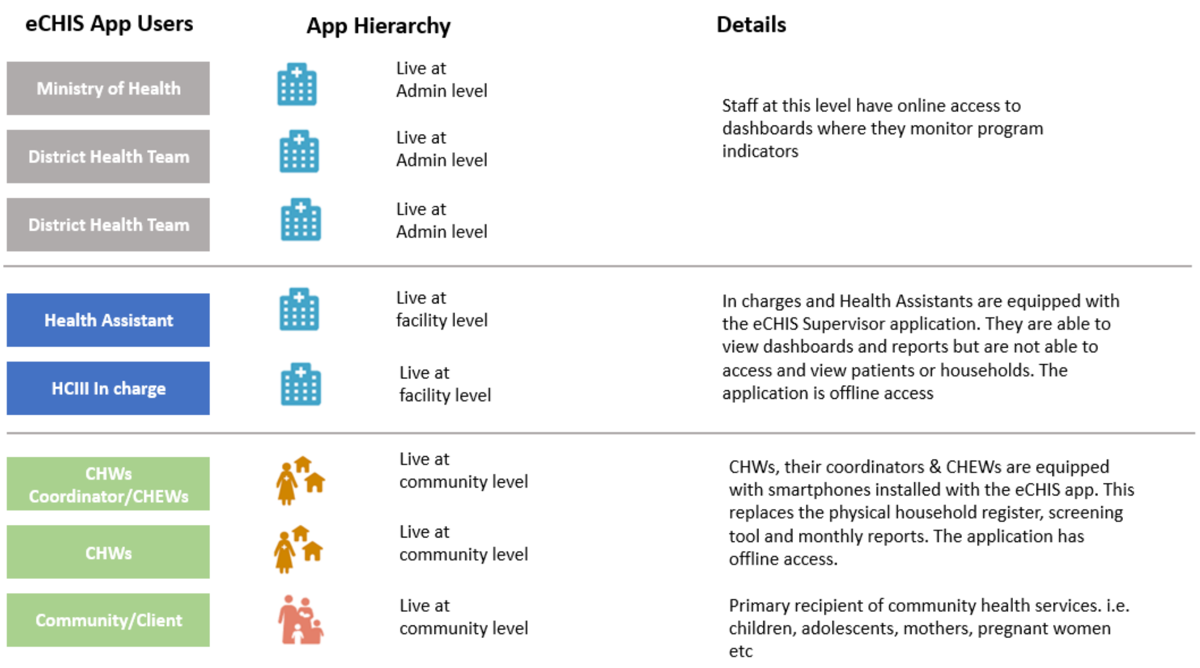

Figure 2: eCHIS User Personas

a) Client

The client is a member of the community and a primary recipient of community health services. Community members fall into one or more cohorts defined by sex, age group, pregnancy status, health and nutrition status et cetera. These characteristics should be taken into account when enrolling community members into specific community health services. Clients are normally organized into households, with each household having one member as the household head. Community health services include but are not limited to immunization, health/nutrition assessments and referrals, family planning, health promotion, nutrition, pregnancy mapping, Antenatal care (ANC), and Postnatal care (PNC). Caregivers of clients receiving health services are also included in this group. The clients shall be registered within the eCHIS and services delivered to them tracked longitudinally.

b) Community Health Workers

The Community Health Worker is the primary agent of community health service delivery at the household level. Their main role is to provide community health services while passively collecting key program data. CHWs are normally members of the communities in which they

work, hence, an intimate familiarity with those communities is assumed. CHWs shall utilize the eCHIS to deliver quality health services, capture and use data for operational decisions such as tracking defaulters and referrals to health facilities.

c) CHW Coordinators/Community Health Extension Workers (CHEWs)

CHW Coordinators/CHEWs are responsible for coordinating CHW activities at the parish level. They oversee several CHWs within villages located in their area of jurisdiction (Parish). The CHW Coordinators/CHEWs are CHWs but with a better understanding of the programmatic requirements, reporting needs and overall functionality of the eCHIS. These deliver mentorship and coaching to fellow CHWs and are the first line of support (to CHWs) in terms of troubleshooting issues or overcoming challenges associated with the eCHIS.

d) CHW Supervisors

CHW Supervisors are responsible for supervising and overseeing the delivery of CHW activities. The CHW Supervisors are full-time salaried government employees charged with the management of health service delivery at the community level. CHW supervisors -oversee a larger geographical area than CHWs, usually an entire catchment area of the health facility comprising more than one village. CHW supervisors occasionally visit clients in their households too. However, their visits are typically supervisory and are aimed at assessing and promoting service quality. CHW supervisors link to and mediate between CHWs and health facilities. In particular, they are responsible for distributing commodity supplies to CHWs, as well as verifying and escalating reports of notifiable diseases or events of public health interest. Their other tasks include training, mentoring and coaching CHWs and performing routine community health data quality checks. CHW Supervisors shall access eCHIS dashboards and supervisor views constituting key information necessary to provide insights on how best they can supervise and support the CHWs.

e) District Health Team

The District Health Team (DHT) is responsible for the management of healthcare programs at the district level. The DHT shall access the eCHIS through dashboards and reports for routine performance monitoring, program monitoring and reporting to enable evidence-based decisions.

f) Ministry of Health

The national-level team at the Ministry of Health is charged with the responsibility of system administration, technical programming aspects, supervision, resource mobilization, and planning. Escalation of any issue concerning the eCHIS shall follow the application hierarchy right from the CHW to the national level.

2.3 System Functions

The eCHIS shall provide the following key functionalities as a minimum and can further be customized according to the user requirements.

Household Registration	• Registration of households and household members.<br>• Updating household and household member data.
Client Referral	•Referring clients from the community level to the health facility level and vice versa.
Reminder Services	•Reminder messages should be sent to the clients and notifications to the CHWs and health facilities.
Stock Management	• Commodity dispensing, adjustments and stock status monitoring.
Surveillance	• Reporting suspected cases of notifiable diseases and events of public health interest.
Analytics and Reporting	• Generating case-based and aggregate client reports and analytics to drive evidence-based decision-making.
Data & Process Validation	• Validation rules that shall enable the verification of data and processes to ensure adherence to set standards before a user is able to save a record.
Decision Support	•Functionality to ensure that the CHW's decision-making capability is improved.

System administration and Security	• Configuring the system with organizational hierarchies, users, user roles, commodities and security mechanisms.

2.4 Design Process

A strong system design is crucial to the development and functionalization of the eCHIS to produce real-time quality information for decision-making. Through the design process, developers and programme teams understand the healthcare processes, persona user stories, unique data use requirements for each persona, the different healthcare workflows and how they are delivered in the community, the way users' interface with the eCHIS technologies, and how to continue to build for interoperability.

The human-centred design approach and analytical development methods shall be used to understand the user requirements while considering the bottlenecks and challenges that users face.

The human-centred design takes a bottom-up approach and focuses on understanding an intervention's users or stakeholders. It seeks to understand how people do their work, how they perceive their health services, and how they experience challenges and may envision solutions.

The analytical approach focuses on breaking down the process into the elements necessary to solve it and takes a top-down approach. An analytical approach shall often require an expert mindset (consultant/technology firm) to collect information about how the intervention works in a linear, causal process. The outcome is information-oriented, resulting in a user requirements document or report.

Therefore a participatory approach that allows users and stakeholders to contribute to the design and development process shall be used in a blended manner, that is to say, utilizing both human-centered design and the analytical approach.

The Division of Health Information Management shall lead the design and development process working with other relevant partners, departments and programs such as the Community Health Department Maternal and Child Health Department, Expanded Programme on Immunization, AIDS Control Program, Tuberculosis and Leprosy Program, Environmental Health Department, Malaria Control Division, and Nutrition Division.

2.4.1 eCHIS Design Principles

The eCHIS design principles shall guide every step throughout the eCHIS design and development processes.

1. **Usability:** Design and develop the eCHIS application in a way that shall minimize the complexity for end users interacting with it.
2. **Scalability:** Should be capable of continuing to perform as the eCHIS scales both vertically and horizontally over time in terms of enrolling end-users, data management and use.
3. **Availability:** Ensure both system and data is available to digital health applications and their end users whenever needed (within the bounds of confidentiality) to guarantee client safety and business continuity.
4. **Interoperability**: Foster interoperability of the eCHIS with other relevant digital health systems as per the digital health enterprise architecture.
5. **Collaboration:** Adopt a governance approach that includes multi-sectoral stakeholders in the decision-making and management of eCHIS design and development.
6. **Open standards:** Use internationally accepted standards that promote interoperability for data, workflows, and technology.
7. **Data quality and integrity:** Follow accepted data standards and create measures to uphold the integrity and reliability of data captured, processed or stored by the eCHIS.
8. **Ethics:** Equity, safe use, data privacy and protection: These principles are vital for ensuring the social inclusion of the eCHIS.
9. **Access control:** Enforce mechanisms to restrict access to both the system and data stored within the system.

2.5 eCHIS Development Process

The development process shall follow a streamlined Software Development Life Cycle (SDLC). A Systems Requirements Specifications (SRS) document detailing both functional and non-functional requirements shall be developed in line with the user requirements gathered.

The SRS document shall then inform the Systems Design Document (SDD). Both documents shall be presented to the CHIS implementation thematic working group for validation through target user departments for validation and sign-off.

The System development process shall, where possible, employ locally available resources to reduce reliance on external sources. It is, however, recognized that some desired technical skills and capacity may not be available in the country and shall be outsourced according to the Government of Uganda procurement guidelines and where possible according to the development partner procurement guidelines.

To ensure a user-centred design approach and quality software, the Agile development model shall be applied during the development process with extensive consultation and interaction with the end users. Key stakeholders shall be engaged during these steps from the Ministry of Health relevant departments and other line state agencies including but not limited to the National Identification and Registration Authority, Parish Development Model Secretariat, and Uganda Bureau of Statistics to ensure interoperability and that other key requirements for the system are addressed.

2.6 System Testing

The eCHIS testing shall comprise both internal system tests that shall focus on technical aspects of the system and User Acceptance Testing (UATs) where feedback on the developed/improved system shall be solicited.

a) Internal system tests

The Internal system tests shall focus on technical aspects that shall ensure system performance such as integration testing, unit testing, and functional and non-functional testing among others. These tests shall be conducted by the technology partners, the MoH ICT Section and DHIM.

b) User Acceptance Testing

User Acceptance Testing (UAT) shall be part of the software development process that focuses on real-world testing by the intended end users.

UATs shall be conducted for every software developed, revised workflows, new features, integrations, system enhancements or upgrades.

A UAT report shall be compiled and shared with relevant stakeholders through existing governance structures such as the CHIS Implementation thematic working group of the Health Data Collaborative (HDC) Subcommittee and the HIIRE TWG, the report shall incorporate feedback from users and the proposed system improvements.

A meeting with relevant stakeholders shall be held to discuss the report and a road map to address the issues raised shall be agreed upon.

The User Acceptance Testing report shall be signed off once all the raised issues are addressed and validated by the user departments. The Division of Health Information Management shall be responsible for coordinating the sign-off of UATs.

All system changes requested by User Departments and Programmes shall be documented using the Change Review Form (Appendix 9)

3.0 Implementing eCHIS

3.1 Pre-Implementation Requirements.

This subsection guides the key requirements and sub-tasks that need to be done before activity implementation.

1. **eCHIS National Rollout Roadmap**

   A national rollout roadmap shall be developed and updated by the Ministry of Health detailing priority areas, timelines, how the program shall run, and geographical coverage.

2. **Implementation approval by MoH**

   The Ministry of Health is responsible for providing overall coordination of all parties involved in the process, including but not limited to leading inception, reviewing progress, continuing implementation efforts, and monitoring compliance with all electronic community health systems to be implemented. All parties intending to implement eCHIS should seek guidance and approval from MoH before engaging districts. As part of the approval, the implementation plan shall be presented to the Community Data Thematic Working Group of the Health Data Collaborative responsible for coordinating the implementation of the eCHIS.

3. **Readiness Assessment**

   A readiness assessment using the eCHIS deployment checklist (Appendix 1) for the proposed Local Government shall be conducted to check the readiness of the Local Government and the Ministry of Health (MoH) to support the rollout of the eCHIS. This shall check if resources are planned to support the rollout of the eCHIS in the selected Local Government and this includes but is not limited to; finances, human resources, hardware, software, approved CHW lists, and draft device management agreements among others.

   The user shall sign the device management agreement (Appendix 2, 3, and 4) upon receipt of the device (Tablet/Smart Mobile Phone).

   a) Shadowing of CHWs

   The training team and supervisors shall supervise the trained CHWs on their first day of household registration. This shall also serve the purpose of practical knowledge enhancement for the CHW as well as building confidence in the use of the eCHIS. A minimum of five (05)

households shall be registered on the initial day by the newly trained CHW to their administrative units and health facilities.

As part of the preparation by the DHT, a minimum of two CHWs per village shall be selected for the case of VHTs and other CHWs shall be selected as per the national CHW selection criteria and a list made available to the National Team prior to district entry activities.

3.2 eCHIS Deployment Phase

3.2.1 District eCHIS Work Plan

The district shall develop a work plan detailing how it shall roll out and coordinate the program, resource mobilization approaches to partner engagement and support, and how it shall supervise and oversee program implementation including pre- and post-deployment support as well as the sustainability plan.

3.2.2 eCHIS Deployment

In this phase, the eCHIS shall be introduced to both the District Health Team, Health Facility Supervisors and the Community Health Workers. This stage shall include training of supervisors and CHWs, onboarding and activation of CHWs.

The deployment of the eCHIS shall strictly observe 100% digitisation of the CHWs within a health facility catchment area for purposes of ensuring complete electronic reporting for the health facility on the eHMIS (DHIS2).

For fully digitized facility catchment areas, all partners intending to work with the existing CHWs shall support them in the management and use of the eCHIS application.

Deployments shall be fully standardized and follow the cascaded activities below;

b) Entry Engagements

A meeting aimed at District leadership engagement to introduce the program, seek political and support, and discuss resource mobilization shall be held. The district leadership to be targeted for the district engagement shall include but not be limited to the following (DHT, CAO, RDC, LC5, DISO, DPC, DCDO). This shall apply to similar structures for cities and

municipal councils. Where Community level partners exist within the Local Governments, these shall be part of the engagements as well.

c) **Device Custody and Management Plan:**

Devices distributed in the district if procured by MoH or partners shall be handed over to the Local Governments who shall ensure they are captured within the district or city inventory list for purposes of tracking. Device agreements (Appendix 2, 3, and 4) shall be signed off by the beneficiary while issuing out the devices to CHWs. A copy of the agreement shall be shared with the CHW and another copy maintained by the Local Government for record purposes.

d) **On-Boarding Training**

1. **Pre -Training/Preparation Phase**

   The training team shall ensure that all requirements in the eCHIS deployment checklist are met ahead of the training (Appendix 5).

2. **Training of Trainers and Supervisors**

   Training shall follow the updated standard training guide. Participants to be trained as ToTs and supervisors shall include the Facility In charges, CHW Supervisors or Health Assistants, Health Inspectors, DHT and Partner Representatives. ToTs and Supervisors shall be trained on the eCHIS for a **minimum of three (3) days** following the **Appendix 6** schedule for TOT training.

3. **Training of CHW**

   The CHW training shall follow the established training guide that shall entail all the service areas in eCHIS. CHWs to be digitized shall be trained in health service areas for a minimum of **two (2) days**, and on the digital aspect for a minimum of **four (04) days** in class not exceeding 65 CHWs each.

   CHWs shall be trained on a training instance whose icon shall be different from the production or live system. CHWs shall be availed of generic logins to the training instance once training on the digital aspect commences.

CHWs shall be subjected to a mandatory test on the eCHIS to assess the extent of knowledge transfer. A **minimum of 80%** shall be obtained by a CHW before they complete the training.

Failure by the CHW to obtain the mandatory 80%, the CHW shall be retrained on the service areas and the eCHIS and subjected to a second test.

Failure by a CHW to obtain the mandatory 80% pass mark on a second attempt, the CHW shall be replaced immediately.

e) Onboarding on the eCHIS

1. **Configuration and CHW activation in the production instance**

   Upon completion of the training of CHWs, the CHW devices shall be configured with user profiles for the production instance of the eCHIS after uninstalling the training application as detailed in the trainer's manual.

2. **Tooling and Reintegration into the District Structure**

   The users shall be equipped with the necessary work tools including but not limited to digital job aids, eCHIS user guide, and Sick Child Job Aids (SCJA).

   CHWs shall continue to use the manual CHW registers alongside the eCHIS for a period of a maximum of one month before completely using the eCHIS alone.

   Manual registers shall only be used as a backup mechanism in the event the eCHIS is non-functional. Once the eCHIS functionality has been restored, all the data captured using the manual registers shall be transferred.

3.3 eCHIS Post-Implementation Support

3.3.1 Continuous Capacity Building and Mentorship

This subsection includes modalities of conducting continuous capacity building and mentorship post-deployment of eCHIS.

a) **Bi-Annual National Supportive Supervision**

A team from the National Level guided by reports generated from the eCHIS shall make supportive supervision visits to the district on a bi-annual basis. An approved HMIS and CHW Support Supervision tool shall be used to document findings. The findings and remedial action plan entailing clear action points and well-defined timelines shall be discussed with all stakeholders thereafter.

b) **Quarterly Performance Review Meetings**

The data quality performance review meetings shall be utilized to review community health performance, identify data quality gaps and craft improvement plans.

c) **Supportive Supervision:**

1. Routine supervision of the CHWs by the District Health Team (DHT) shall be conducted.
2. The supportive supervision by the Health Assistant or CHW Supervisor shall be conducted monthly to support the CHWs close performance and data quality gaps identified.

d) **Peer mentorship by CHW Coordinators**

The CHW coordinator shall support/mentor the peer CHWs on at least a Bi-monthly basis should the CHWs have any queries or challenges.

e) **Refresher Training:**

1. One full day of monthly training for a minimum of three months consecutively after the initial deployment of the eCHIS shall be conducted and thereafter a minimum of three quarterly refreshers shall be held. The training shall focus majorly on areas of poor performance as guided by the monthly report, complex workflows and modules as identified by the CHWs or their supervisors, and new modifications in existing workflows.
2. Thereafter yearly refresher training is recommended to address any area of interest as may be identified at the national and subnational levels or aesthetic changes to the system.
3. Training on any new/updated workflow or system enhancement/upgrade shall be

scheduled on a need basis.

3.4 Transitioning from Other Community Health Applications to eCHIS

Transitioning of CHWs already enrolled on an organizational or disease-specific community health application shall follow sections 3.1 to 3.2 with exceptions indicated below;

1) Existing CHWs already enrolled on the organizational or disease-specific community health application shall be considered for the transition.
2) In villages where more than one CHW is already digitized, efforts shall be made to identify another CHW for equipping and training. In case of resource constraints, the already digitised CHWs shall be transitioned and a plan to equip and digitise the remaining CHWs shall be agreed upon by the MoH, district and implementing partners.
3) District engagement meetings shall involve health facility supervisors as part of integrating CHWs from partner structures into Local Government structures.
4) An inventory of the already issued devices shall be handed over to the district management for inclusion in the district inventory and tracking.
5) Training of CHWs on the service areas and the digital aspect shall be for a minimum of **three (3) days**.

4.0 System and Data Access

System access credentials shall be required for User personas detailed in Figure 2. A unique username and password shall be generated for each user and it shall be the responsibility of the individual to keep their password safe. A request for a password change shall be sent to hmissupport@health.go.ug in case a user forgets her/his password or the need for renewal or the absence of an automated password reset.

Only CHWs shall be permitted to enter and update individual-level records using the eCHIS. Supervisors at the health facility level shall have access to supervisor workflows that shall enable adequate supervision of CHWs. The rest of the management teams shall have access to the interactive analytics dashboards.

The system user roles and permissions shall be automatically enforced by the eCHIS and shall require no manual intervention.

Partner organizations shall send system and data access requests to the Division of Health Information Management Head for approval with hmissupport@health.go.ug in copy. Organizations already having eHMIS (DHIS2) access may be able to access the quarterly (097B) and monthly (097C) reports.

The 097C report shall be pushed from the eCHIS automatically to eHMIS on a monthly basis, specifically, every 7th day of the month. The 097B report shall be pushed to the eHMIS quarterly. The MoH IT Section shall ensure the automated data synchronization process while the DHIM shall ensure data quality mechanisms are in place to maintain high-quality data.

Overall, the data access, sharing and use shall follow the Uganda Health Data Access Sharing and Use Guidelines.

To ensure data protection, the Uganda Health Data Protection, Privacy and Confidentiality Guidelines shall be observed once system and data access has been granted.

5.0 Governance Structure

The Governance of eCHIS shall follow the existing governance and management structures at the national and decentralized levels, as summarized in Figure 3.

Technical oversight is the mandate of the Division of Health Information Management (DHIM) under the Department of Planning, Financing and Policy which guides and coordinates all stakeholders involved in health data collection, processing and storage. This function is similarly decentralized at the Local Government level, as summarized in Figure 3. The main task of the Health Information Innovation and Research Technical Working Group (HIIRE TWG) is reviewing and advising on Health Information System (HIS) and Digital Health policy- and strategic-related issues stemming from the user departments and other stakeholders.

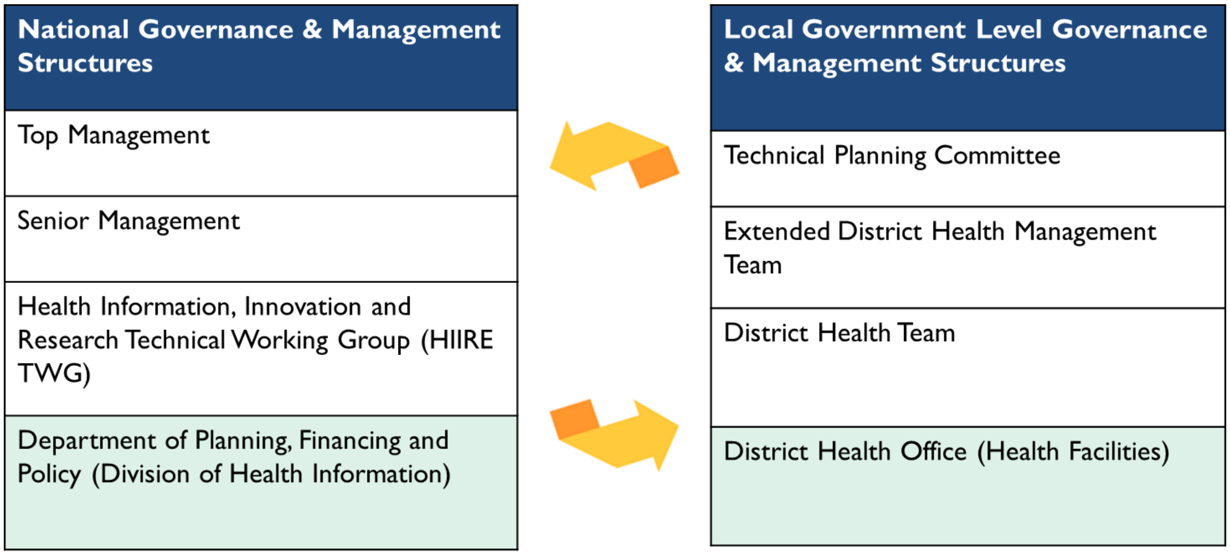

Figure 3: Governance Structure

The Division of Health Information Management (DHIM) together with other user departments shall be responsible for monitoring the application of these Guidelines to ensure compliance.

The Health Data Collaborative (HDC) Subcommittee of the HIIRE TWG specifically its CHIS Implementation thematic working group shall be charged with the coordination of the eCHIS implementation nationwide. Other HIIRE TWG subcommittees like the Digital Health, Data

Management, and Analytics Subcommittees shall provide technical input towards the implementation of the eCHIS through the Health Data Collaborative Subcommittee.

5.1 Implementation Arrangements

SN	Entity	Level	Key Responsibilities
1	Top Management	National	● Strategic leadership, guidance and oversight.<br>● Approval of systems for use in Uganda as official tools and for scale.<br>● Monitoring and Supervision of systems.
2	Senior Management Committee (SMC)	National	● Strategic leadership, guidance and oversight.<br>● Endorsement of systems for use in Uganda as official tools.<br>● Monitoring and Supervision of systems.
3	Health Information, Innovation and Research (HIIRE) Technical Working Group	National	● Approve systems for piloting.<br>● Recommend systems to SMC for full scaleup.<br>● Monitoring and Supervision of systems.<br>● Provide technical guidance and quality assurance.<br>● Ensure adherence of systems to standards and guidelines.<br>● Review and recommend developed standards, and guidelines for approval by the SMC.
4	Health Data Collaborative (HDC)		● Promote and facilitate greater alignment of investments (both domestic and external resources)<br>● Identify challenges and obstacles that impede performance and opportunities to improve data systems for increased effectiveness and value for money.<br>● Report back to the HIIRE TWG on findings and recommendations.
5	Community Data Thematic Working Group.	National	● Guide on eCHIS implementation modalities.<br>● Coordinate stakeholders.<br>● Participant in the joint planning, monitoring and evaluation of the CHIS.<br>● Recommend best-practice to aid improved development and implementation of the CHIS.

6	MoH/Planning, Finance, Policy Department	National	● Coordinate implementation of eCHIS.<br>● Recommend systems for pilot and use following a technical evaluation.<br>● Recommend system hosting strategies.<br>● Prioritize areas of implementation in line with the Health Information and Digital Health Strategic Plan.<br>● Coordinate and Manage access to systems.<br>● Coordinate and ensure data and system management with the support of stakeholders.<br>● Quality assurance of eCHIS.<br>● Coordinate integration of community health information systems with other systems.<br>● Authorize changes and modifications to the system.<br>● Supervise implementation teams.<br>● Develop Health Information System standards and guidelines and monitor use or compliance.<br>● Coordinate capacity building programmes with stakeholders.<br>● Coordinate the development and implementation of a sustainability plan for CHIS with stakeholders.
7	MoH/IT	National	● Provide and manage hosting infrastructure for systems.<br>● Ensure security of systems and respective hosting environments.<br>● Ensure data and system backup.<br>● Coordinate with external entities in case of outsourced hosting.<br>● Coordinate and Manage access to system hosting environments/backend.
8	MoH/User Departments	National	● Participate in the requirement gathering and design workshops and validation of system requirements specifications.<br>● Provide programmatic subject matter and guidance on functionality of the tool including its design.<br>● Participate in eCHIS user acceptance tests and sign off requirements, workflows and data points
9	Partners	National/ District	● Provide technical and financial assistance for implementation of community health information systems.<br>● Support resource mobilisation for the implementation of a community health information system.<br>● Support capacity-building efforts of MoH and Local governments on management and use of the CHIS.

			● Participate in the development of standards and guidelines.<br>● Support monitoring and evaluation of the community health information system.<br>● Participate in the roadmap development and implementation of CHIS with MoH.<br>● Support optimisation, implementation and adaptation of the CHIS.
8	Local Governments	District/Cities/<br>Municipality/<br>Subcounty	● Lead the implementation of the eCHIS at the local government level.<br>● Coordinate implementation of eCHIS at local government level (districts and cities) with partners.<br>● Supervise implementation teams.<br>● Participate in user acceptance tests and sign off requirements, workflows, and data points<br>● Implement the CHIS sustainability plan with the support of MoH and stakeholders.<br>● Support resource mapping and mobilization efforts towards scaling and sustaining eCHIS at district level.<br>● Conducte eCHIS deployment monitoring, assess performance and address key issues within the district's capacity or sphere of influence, else, share feedback upwards for timely resolution by the responsible MoH entity.
10	Health facilities	District/Cities/<br>Municipality/<br>Subcounty	● Ensure data quality assessment and assurance .<br>● Support community health workers on the management and use of eCHIS.<br>● Conduct capacity building of the community health workers on management and use of eCHIS and devices.<br>● Generate list of users of CHIS for approval by the MoH Department of Planning, Finance and Policy.<br>● Support eCHIS implementation at community level.<br>● Supervise community health workers within<br>● their catchment area.

6.0 Monitoring and Evaluating the Guidelines

This section outlines how eCHIS deployments and the use of eCHIS implementation guidelines shall be monitored and evaluated.

6.1 eCHIS Implementation Guideline Use Monitoring

Monitoring the use of the Guidelines for Implementing the Uganda electronic Community Health Information System shall be based on a specific framework designed to track compliance and effectiveness. Important to note is that dissemination and training on the use of the guidelines are key requirements before monitoring its use can become a practical reality. Therefore, the guidelines shall be monitored using three major parameters, namely, dissemination, training, and compliance. They are described further below and shall be tracked and assessed to measure guideline use. The guideline-use monitoring framework consists of the parameters or components, key monitoring questions, result statements, indicators, and means of verification.

- **Dissemination:** Refers to broadcasting the guidelines to a target audience through printed or electronic media such as print, electronic documents, or other forms of media as appropriate. Copies of the guidelines shall be printed and distributed at national and sub-national levels. The electronic copy of the guideline shall be uploaded on the Ministry of Health online Knowledge Management Portal for public access and consumption.
- **Training:** Refers to orienting and/or sensitizing key stakeholders on the key components of the guideline document including its value and their roles and responsibilities to ensure awareness and accelerate compliance.
- **Compliance:** Refers to the state of implementing the eCHIS in accordance with established guidelines or specifications, or the process of becoming so (compliant).

Refer to **Appendix 11** for a detailed Guideline-Use Monitoring Framework.

6.2 eCHIS Deployment Monitoring

The eCHIS implementation is anchored within the overall Uganda Health Information and Digital Health Monitoring and Evaluation Framework. Notably, it is critical to continue monitoring eCHIS deployment nationwide to ensure that the software is working as it should, that every activity is implemented as planned, and that external factors are not affecting the potential effectiveness of the eCHIS intervention.

Findings from deployment monitoring activities shall be synthesized, socialized, and remedial action taken to address identified challenges. This shall ensure the optimization of eCHIS implementation across the board and foster successful evaluation efforts. Consequently, four (4) key parameters of eCHIS deployment monitoring shall be considered, namely, functionality, stability, fidelity, and quality. They are described further below and shall be tracked and assessed to ensure that the intervention is “doing things right”.

Similar to guideline-use monitoring, the eCHIS deployment monitoring framework shall consist of parameters or components, key monitoring questions, result statements, indicators, and means of verification.

- **Functionality:** *Refers to the degree to which eCHIS provides functions that meet stated and implied needs when used under specified conditions, or the ability of eCHIS to support the desired intervention.* For instance, the desired functionality of the eCHIS application based on agreed upon user requirements. Monitoring functionality shall be done pre- and post-deployment to ensure adequate system functionality at all times. Pre-deployment monitoring shall entail testing and providing feedback on various aspects including but not limited to skip patterns, validation checks, form schedules, form content, user interface design, data export/import functionality, data accuracy, and dashboard calculations. Post-deployment and/or at a later maturity stage (i.e., where the eCHIS applications have been fully designed and only enhancements are required to meet evolving policy changes and information needs), basic system functionality monitoring shall be conducted before introducing the eCHIS to new users (e.g., a different cadre of health workers) and new geographic areas that might pose different levels of connectivity or when using new technologies.

- **Stability:** *Refers to the likelihood that eCHIS functions shall not change or fail during use or the ability of eCHIS to remain functional under both normal and anticipated peak conditions for data loads*. Monitoring stability shall be done concurrently with functionality monitoring both pre- and post-deployment. Initial (pre-deployment) system stability monitoring shall be conducted during quality assurance testing sessions before eCHIS app is declared ready for deployment (field-ready). Post-deployment, stability shall be monitored to mitigate any events that may result in improper delivery of the eCHIS intervention such as unexpected crash or stop, slow response times especially during peak times or when overloaded, and any erratic performance hindering management and use of eCHIS. Considering that the eCHIS heavily relies on physical (non-cloud-based) servers for operation, server outage shall be monitored. Considering stability is a critical aspect to ensure successful implementation of eCHIS intervention, continued stability monitoring processes shall be automated and systems configured accordingly to enable proactive and timely reactive response.

- **Fidelity:** *Refers to a measure of whether or not an intervention is delivered as intended in terms of technical and user perspectives.* For instance, the technical fidelity of eCHIS (i.e., functionality and stability) and the enabling environment such as any external barriers that might cause it not to function as intended, and compliance of eCHIS end-users to stipulated data use and system administration standard operating procedures. Monitoring fidelity of eCHIS intervention shall occur throughout implementation with the required level of effort

gradually decreasing depending on the time it takes to identify and resolve issues. Standard monitoring procedures and reporting mechanisms shall be established and automated to aid timely decision-making.

- **Quality:** *Refers to the measure of excellence, value, conformance to specifications, conformance to requirements, fitness for purpose and ability to meet or exceed expectations.* For instance, the standards related to the capabilities of eCHIS end-users and content of the inputs used for the programmatic interventions that eCHIS is designed to deliver such as algorithms for decision support, data collection forms, and SMSs (FamilyConnect). This content should be of the highest quality possible informed by existing practice, literature and formative research in the local context to increase the effectiveness of the eCHIS intervention. eCHIS quality monitoring shall focus on ensuring (a) data quality and regularity by checking for outliers of non-compliant users; and (b) the quality of content to be delivered is as expected and in line with existing standards and appropriate for participating communities.

Refer to **Appendix 13** for a detailed eCHIS Deployment Monitoring Framework.

6.3 Evaluating the electronic Community Health Information System

The evaluation shall entail any measures that shall be taken and analysis performed to assess;

a) The interaction of users and/or the health system with eCHIS intervention and strategies
b) Changes attributable to the implementation of eCHIS.

Any commissioned evaluations focused on eCHIS shall be designed to ensure assessment and evidence generation on its usability, effectiveness, value for money and affordability at the very least, depending on the degree of maturity i.e., early or middle or late stage. The following evaluation components shall constitute the eCHIS evaluation framework in addition to the evaluation types:

- **Feasibility:** Assess whether eCHIS works as intended in various contexts across Uganda.
- **Usability:** Assess whether eCHIS is used as intended.
- **Effectiveness:** Assess whether eCHIS achieves the intended results in an uncontrolled (non-research) setting.
- **Implementation research:** Assess the uptake, institutionalization, and sustainability of eCHIS in Uganda, including policies and practices.

Depending on the eCHIS degree of maturity and evidence needs, several evaluation types categorized as formative or summative may be commissioned and/or conducted.

- **Formative evaluations:** Studies aimed at informing the design and development of effective intervention strategies conducted before or during implementation of an intervention.
- **Summative evaluations:** Studies conducted at the end or a certain phase of the intervention to determine the extent to which expected outcomes have been achieved.

Refer to **Appendix 13** for a high-level eCHIS Evaluation Framework.

6.4 Reporting, Dissemination and Adoption of the Guidelines

The framework shall guide the collection of information regarding the implementation of the eCHIS to facilitate reporting, feedback, and dissemination.

a) Dissemination and adoption of the guidelines

The eCHIS implementation Guidelines shall be disseminated for adoption through:

1. Presentation of the guidelines to stakeholders.
2. Posting of the guidelines on the MoH websites and the electronic Library for access by the stakeholders.
3. Organising quarterly workshops to train stakeholders and innovators.
4. Leveraging eCHIS activities like refresher training etc to disseminate the guidelines

b) Reporting

The reporting requirements shall be based on the approved 097B HMIS report. The reports shall be for purposes of synthesis, monitoring of usage and functionality. The data shall be pushed from the eCHIS to the electronic Health Management Information System (eHMIS/DHIS2) every month following the schedule below;

1. All data captured in the eCHIS mobile application shall be synchronised or pushed by the CHWs to the eCHIS servers by the 5th of every month.
2. All the data received on the eCHIS servers tagged to fully saturated health facilities (health facilities whose catchment areas are fully digitised or CHWs are fully equipped with the eCHIS) shall be pushed to eHMIS/DHIS2 every 7th of every month following the 097B reporting format.
3. District and health facility level health managers shall review and validate the pushed eCHIS datasets in eHMIS/DHIS2 from the 8th - 15th of every month for purposes of ensuring completeness and accuracy.

4. The system shall be locked for entry or validations of data past the 15th of every month to allow for analysis of consistent data for the reporting period.

All the synchronised data shall be accessed through the eHMIS/DHIS2. For real-time data from the eCHIS, dashboards (https://echis-dashboards.health.go.ug/) shall be utilised to access indicator performance for various administrative at various levels.

Monthly performance reports indicating various indicators as per the 097B reporting format shall be produced and shared with stakeholders.

References

1. (MoH), M.o.H., *Ministry of Health Strategic Plan 2020/21 - 2024/25*. 2020, Ministry of Health (MoH): Kampala.
2. MoH, M.o.H., *Health Information and Digital Health Strategic Plan (2020/21-2024/25)*. 2023.
3. MoH, M.o.H., *Uganda Digital Health Enterprise Architecture Framework 2020/21 - 2024/25*.
4. (MoH), M.o.H., *National Community Health Strategic Plan 2020/21 - 2024/25*. 2020, Ministry of Health (MoH): Kampala.

Appendix 1: eCHIS Readiness Assessment Checklist

	Item	Response	
1	Approved Budget Available	Yes	No
2	ICT Equipment (Phones & Tablets) Quantified and Procured		
3	Approved Device Agreement Available		
4	Updated eCHIS Application Tested and ready for installation		
5	eCHIS Application Installation Guide Available		
6	eCHIS Training Guide Available		
7	CHW Training Schedule Approved		
8	Field Deployment Team Approved		
9	Official Invitation letter Signed to Support Deployment		

Appendix 2: Device Management Agreement Template for CHW Supervisor

DISTRICT LOCAL GOVERNMENT SUPERVISOR TABLET AGREEMENT

THE REPUBLIC OF UGANDA
MINISTRY OF HEALTH

MM/YY

SIGNATURE PAGE

This agreement has been approved by the Ministry of Health and passed for use by the district local governments to guide ownership and management of tablets given to Community Health Worker (CHW) supervisors.

Approved by:

Name: ………………………………………….,

Title: (Approving Authority)

Signature:.. Date: dd/mm/yy

Reviewed by:

1. Name: …………………………………………………………………….

   Title: The Chief Administrative Officer, xxx District Local Government

   Signature: …………………………………. Date: …………………………………

2. Name:..

   Title: The District Health Officer, xxx District Local Government

   Date: ………………………………………………………..

The **Tablet** Agreement is between the CHW supervisor noted below and the District Local Government (DLG) Health Office.

Name: ________________ Sub-county: ___________________Parish………..

Tablet Serial #: ____

Mobile#: ____

The Tablet is for use by supervisors to assist in the assessing CHW performance and other DLG Health office operations.

1. Ownership

This **Tablet (Device Name)**, **remains the property of DLG Health Office**. The purchase price of the Tablet is xxxx Uganda Shillings.

2. Supervisor Responsibility

The CHW supervisor shall assume responsibility for the Tablet and agree to handle the asset as follows:

a. Ensure proper use and safety of the asset at all times. Anything that happens to the asset shall be attributed to the remuneration of the supervisor.

b. The Tablet shall be used in its provided protective cover at all times, and the team member shall not eat or drink while handling the asset or any of its accessories

c. Declare any defects/ damages observed on the asset before commencement of service and while in the field to your supervisor

d. Use the keypad lock and use a PIN code to lock the Tablet, so that if the Tablet is subsequently stolen or lost, a PIN code must be used to unlock it

e. Return the asset in good working condition as qualified by DLG Health Office at the end of the contract. Failure to return the asset in good working condition shall result in reduced or in extreme cases no remuneration to the supervisor.

The Tablet is registered with the above noted serial number.

3. Loss or Damage of Tablet

In case of theft or loss, supervisor must:

a. Contact the Police within 24 hours of discovery of the occurrence and report the incident. Ensure that you receive an incident reference number from the police.

b. Report the theft/loss to DLG Health Office within 24 hours of discovery of the occurrence, with the date of the theft or when the Tablet was lost, the Police station to which it was reported and the incident number.

c. A block shall be placed on the Tablet to ensure that it cannot be used.

4. Replacement Procedure and Penalty

All staff qualify for one replacement of the Tablet as stated in procedures below:

a) All claims for replacements and repairs are supposed to be submitted in writing to Line managers accompanied with a Police letter (To whom it may concern Letter).

b) All claims are subject to an Internal investigation led by the IT department and the Victim/subject should provide supporting evidence (when they are in position to) for example a Police report (with case number and findings).

c) Staff can have only 1 replacement provided they have not miss handled or neglected the Asset. This conclusion is based on the internal investigation led by line the manager.

d) Staff shall bear the cost of any further replacement as follows provided damage/loss is established to have risen from staff negligence.

- 2nd replacement—staff pays 50% of asset cost
- Further replacements—Staff pays 100% of asset cost

5. Repair

a. Staff can have only repair provided they have not miss handled or neglected the Asset. This conclusion is based on the internal investigation led by line the manager
b. Staff shall bear the cost of repairs provided damage is established to have risen from staff negligence. The cost of repair shall be determined by the DLG Health Office based on the extent of damage.
c. Tablets in need of repair should be returned to headquarters for repair or replacement. Tablets come with a one-year warranty, and DLG shall work with the manufacturer to get the Tablet repaired. Please note that manufacturers' warranties do not cover damage caused by misuse or neglect.

6. Data and Airtime

The xxxx (organization) with funding from xxxx (development partner) shall provide the supervisors with sufficient data and airtime from mm/yy to mm/yy. Thereafter, the DLG

will send sufficient airtime and internet data bundles each month to the line registered which must be used exclusively to transmit and review data for the DLG health applications.

7. Loss of Tablet Privileges

DLG reserves the right to get the Tablet or pass the costs on to the employee if:

- A specific event in a policy is contravened with financial cost to DLG (e.g. knowing a Tablet has been stolen and failing to report it, using data bundle for personal calls or gaming) or
- if a repeat event occurs (e.g. loss or damage a second time to Tablet).

8. Support with the tablet

Should there be any queries on the use of the Tablet or mobile application, please contact IT support at **0800100066**

9. End of Relationship

The supervisor agrees that upon termination of the relationship with DLG, should she/ he not return the Tablet, or should the Tablet be returned in an unsatisfactory condition, the cost of replacement shall be deducted from any final monies owing, or the supervisor shall otherwise reimburse DLG.

10. Legal avenue

This Agreement shall be governed by the laws of the Republic of Uganda.

I confirm that I have read this Agreement and agree to the terms.

CHW supervisor: **For District Local Government:**

Name: …………………………..
Name: ………………………………..

Signature ______________________
Signature: ____________________

Appendix 3: Device Management Agreement Template for CHWs

DISTRICT LOCAL GOVERNMENT CHW MOBILE PHONE AGREEMENT

THE REPUBLIC OF UGANDA
MINISTRY OF HEALTH

MM/YY

SIGNATURE PAGE

This agreement has been approved by the Ministry of Health and passed for use by the district local governments to guide ownership and management of mobile phones given to Community Health Workers (CHWs).

Approved by:

Name: …………………………………………………………

Title: (Approving Authority)

Signature:... Date: dd/mm/yy

Reviewed by:

1. Name:…………………………………

   Title: The Chief Administrative Officer, ………………………………… District Local Government

   Signature: ………………………………….. Date: …………………………………

2. Name:

   Title: The District Health Officer, ……………………………….. District Local Government

   Date: ……………………………………………………..

This Mobile Phone Agreement is between the CHW noted below and District Local Government (DLG)

CHW Name: ______________________________ Village: ____________________
Parish: ___________________________ Sub County: _________________________________
Supervisor: ____
Phone Serial #: ____
Mobile #: ____

The Ministry of Health shall purchase Android mobile telephones (a "Mobile") for use by CHWs to assist in the execution of their day to day community health activities and improve communications. The mobile phone shall then become the property of the DLG. The CHW shall purchase and register a sim card to be used in the phone

1. Ownership

This phone remains the property of DLG for the first 3 years from the date of issuance to the CHW.
Before the 3 years, the phone shall be subject to the damage/loss policy spelled out in section 4 of this agreement.
After 3 years of consistent service and outstanding performance, ownership of the phone shall transfer to the CHW.
The ownership of the phone shall not be transferred to any other user other than the intended user without prior notice and information of the supervisors.

2. CHW Responsibility

The CHW shall be responsible for the safekeeping, proper use, and eventual signing off of the phone from DLG after 3 Years from the issue date. The CHW shall take good care of the mobile phone to ensure that the device is not damaged, lost or stolen. The CHW is required to keep the mobile telephone clean and in serviceable condition at all times, and report all irregularities immediately to the supervisor within 24 hours. The CHW is required to keep the phone charged and turned on at all times.

For security of the phone device, the CHW shall use the keypad lock and use a PIN code of their choice to lock the mobile phone.

3. Refundable Deposit

Prior to receiving the new Phone, a CHW must have paid a refundable deposit of UGX 30,000 to the xxxxx (Sponsoring entity) which is supposed to be as a security deposit. The deposit is to be used for repair of the phone in case it is damaged/mishandled within the 3 Years period from the date of issuance of the phone.

The deposit shall be reimbursed to the CHW when they choose to exit or exit anyway from the DLG CHW Voluntary assignment before the lapse of 3 Years, only if the device is returned in full working condition.

Full working condition shall be defined as;

- The phone screen is not damaged and the sensor is functional
- The phone can charge

4. Loss or Damage of Phone

In case of theft or loss, CHW must:

a. Contact the Police within 24 hours, report the incident and obtain an incident number for referral.
b. Report the theft/loss to the Health Assistant or CHW Supervisor, within 24 hours of discovery of the occurrence. The CHW Supervisor or health assistant shall investigate, asking the CHW to produce a police report before replacement. This shall then be communicated to the Technical officer who shall channel communication to the tech team.
c. The phone then shall be blocked to ensure that it cannot be used.

d. A replacement shall be issued, when the DLG team has all the required information pertaining to the police report and the follow up procedures.

5. Management of Repairs

Mobile phones in need of repair shall be returned to the CHW Supervisor, the CHW Supervisor shall send them to DLG Health Offices for repair or replacement. Phones come with a one-year warranty, and DLG shall work with the manufacturer to get the phone repaired. Please note that manufacturers' warranties do not cover damage caused by misuse or neglect.

Should your phone freeze/Not work due to technical error, DLG shall provide a replacement phone under this Agreement. In cases of personal misuse or mishandling as CHW you shall pay for the cost of repair, up to a maximum of UGX 30,000.

6. CHW phone Replacement

a. A CHW is entitled to a one-time phone replacement for a reported damage/loss occurring within the first 3 Years, provided it is not arising from personal negligence
b. In the event that the device damage/loss is out of personal negligence, the CHW shall bear the cost of second replacement as follows
    - 2nd time loss-50% of the device cost
    - 3rd time loss – 100% of the device cost
c. In the event that following an initial replacement, loss or damage occurs again and another replacement is required for the handset, the CHW shall be required to pay for the full cost of the phone as stated in clause 1 of this agreement.

The violation of the above could also lead to potential termination of the CHW by DLG

3. Data and Airtime

CHWs shall be provided with sufficient internet data for a duration of 3 Years to support for monthly synchronization of the data collected by the CHWs

4. Support with the phone

All queries on the use of the mobile device or mobile application, should be directed to IT support at **0800100066 o**r your Health Assistant or CHW Supervisor.

5. End of Relationship

In the event of any termination initiated by the CHW or DLG within the stated period (first 3 Years), the CHW agrees to return the phone with all its accessories and shall be refunded the initial deposit of UGX 30.000. In case the returned mobile phone is in an unsatisfactory condition, the CHW automatically forfeits the initial 30,000shs deposit as a recoup to the DLG. After the lapse of 3 Years, the CHW shall retain the phone and all its accessories and shall not be getting the refund of UGX 30,000.

6. Legal Provisions

This Agreement shall be governed by the laws of the Republic of Uganda.

I confirm that I have read this Agreement and agree to the terms.

CHW Name: ________________________

Signature ________________________

Date ___________________________

In presence of:

Name: ________________________ Name:________________________

Title: District Health Officer Title: Supervisor

Signature:________________________ Signature: ________________________

Date:________________________
Date: ________________________

**Guaranteed by (LC 1):**
Name ___________________________
Mobile#: _____________________________

Signature __________________________

Appendix 4: CHW Solar System Agreement Template

DISTRICT LOCAL GOVERNMENT

eCHW SOLAR SYSTEM AGREEMENT

THE REPUBLIC OF UGANDA
MINISTRY OF HEALTH

This solar system agreement is between the Community Health Worker (CHW) noted below and District Local Government (DLG)

CHW Name: ____________________________ Village: ___________________

Parish: _________________________ Sub County: _______________________________

Supervisor: ____

Solar system Serial #: ____

Mobile #: ____

xxxxxx (Name of entity supporting) has purchased solar chargers and lighting systems for use by CHWs to assist in charging of mobile devices and lighting at night while delivering services to the community. The solar system, which is an accessory to the mobile phones shall then become the property of the DLG.

1. **Solar system Ownership**

This xxxxx (specific name of the solar system) solar charging and lighting system, with a x year battery life and cost value of xxxxxx Uganda Shillings, **remains the property of DLG for the first 3 years from the date of issuance to the CHW**.

Before the 3 years, the solar charger and lighting system shall be subject to the damage/loss policy spelled out in sections 3, 4 and 5 of this agreement.

After 3 years of consistent service and outstanding performance, ownership of the solar system shall transfer to the CHW.
The ownership of the solar system shall not be transferred to any other user other than the intended user without prior notice and information of the supervisors.

2. CHW Responsibility

The CHW shall be responsible for the safekeeping, proper use, and eventual signing off of the solar system from DLG after 3 years from the issue date. The CHW shall take good care of the solar system to ensure that the device is not damaged, lost or stolen. The CHW is required to keep the solar charger in serviceable condition at all times, and report all irregularities immediately to the supervisor within 24 hours. The CHW shall be required to avail the solar charger for inspection during supervision home visits. The CHW is required to keep the solar system charged at all times when reasonable. Below are good practices to ensure that the solar is charged most of the time.

1) Ensure that the solar panel is installed on the roof away from trees for easy access to the sun
2) Phone charging should be done during the day when the sun is out and the solar system battery is charging
3) Avoid charging and lighting at the same time in the night as this shall quickly drain the solar system battery
4) While lighting at night, try to use the light of lowest intensity and high intensity when you must. Lighting at high intensity quickly drains the solar charger battery
5) Ensure that the battery is installed inside the house to avoid damage by rain or sun light

3. Loss or Damage of solar system

In case of damage, theft or loss, CHW must:

a. Contact the Police within 24 hours, report the incident and obtain an incident number for referral.
b. Report the theft/loss to the Health Assistant, within 24 hours of discovery of the occurrence. The health assistant shall investigate, asking the CHW to produce a police report before replacement. This shall then be communicated to the Technical officer who shall channel communication to the technical team.
c. A replacement shall be issued, when the DLG team has all the required information pertaining to the police report and the follow up procedures.
d. A replacement shall only be possible if there are spare solar chargers to facilitate replacement and if the loss was not out of negligence.

4. Management of Repairs

Solar systems in need of repair shall be returned to the health Assistant, the HA shall send them to DLG Health Offices for repair. Solar systems come with a 2-year warranty, and DLG shall work with the manufacturer to get the solar system repaired. Please note that manufacturers'

warranties do not cover damage caused by misuse or neglect. For solar chargers that can not be repaired, these shall be replaced depending on the available spare solar chargers.

In cases of personal misuse or mishandling as a CHW you shall pay for the cost of repair, up to a maximum of UGX 30,000.

5. **CHW solar system replacement**
   a. A CHW is entitled to a one-time solar system replacement for a reported damage/loss occurring within the first 2 years of warranty, provided it is not arising from personal negligence. Should the solar system not work due to technical error, DLG shall provide a replacement solar system under this Agreement while leveraging on the warranty provided by the manufacturer if this occurs within 2yrs from date of purchase. This replacement within the warranty period shall be achieved through xxxx (supporting development partner) with support from the xxxx (implementing partner).
   b. In the event that the device damage/loss is out of personal negligence, the CHW shall bear the cost of replacement as follows
      - 1st time loss - 50% of the solar system cost
      - 2nd time loss-100% of the solar system cost

The violation of the above could also lead to potential termination of the CHW by DLG

3. **Support with the solar system**

All queries on the use of the mobile device or mobile application, should be directed to IT support at **0800100066** or your Health Assistant or CHW Supervisor.

4. **End of Relationship**

In the event of any termination initiated by the CHW or DLG within the stated period (first 3 years), the CHW agrees to return the solar system with all its accessories. In case the returned solar system is in an unsatisfactory condition, the CHW pays up to a maximum of UGX 30,000 towards repair of the system.

5. **Legal Provisions**

This Agreement shall be governed by the laws of the Republic of Uganda.

I confirm that I have read this Agreement and agree to the terms.

CHW Name: ________________________ Signature ________________________

Date ___________________________

Guaranteed by (LC or Current CHW):

Name ___________________________
Mobile#: _____________________________

Signature __________________________

Appendix 5: eCHIS Deployment Checklist

	Item	Responsible person	Status	Notes
1	Phones/tablets labeled		Done/ In Progress/Not Done	
2	SIM cards activated with enough credit for training		Done/ In Progress/Not Done	
3	The presentation for the training prepared		Done/ In Progress/Not Done	
4	Agenda of training (printed)		Done/ In Progress/Not Done	
5	Training guides (printed)		Done/ In Progress/Not Done	
6	Device agreements (printed)		Done/ In Progress/Not Done	
7	Pre-printed test (only for user training)		Done/ In Progress/Not Done	
8	Practice Exercises (printed)		Done/ In Progress/Not Done	
9	Banners/ tear drops/posters for partners		Done/ In Progress/Not Done	
10	MOUs (printed)		Done/ In Progress/Not Done	
11	Signing sheets (Printed)		Done/ In Progress/Not Done	
12	Camera/phone for taking pictures		Done/ In Progress/Not Done	
13	Invitation letter signed by MOH		Done/ In Progress/Not Done	
14	Printed invitation letter		Done/ In Progress/Not Done	
	**Preparation of the training room**			
1	The trainers arrive an hour before the training. Assure that it is possible to have access to the room one hour before the training.		Done/ In Progress/Not Done	
2	Plug in the extension cords (at least two)		Done/ In Progress/Not Done	
3	Prepare two projectors, one to present the presentation and one to show the application screen.		Done/ In Progress/Not Done	
4	Prepare the tables and chairs.		Done/ In Progress/Not Done	
5	Prepare source of internet		Done/ In Progress/Not Done	
6	Alternate power source		Done/ In Progress/Not Done	

7	Ensure devices are charged		Done/ In Progress/Not Done	
	**Important points for the day of training!**			
1	Count phones and make sure you have enough for each user. Distribute mobile users.		Done/ In Progress/Not Done	
2	During Digital App Training CHWs to Come with their working Registers (HMIS (Day 2 of DAT)		Done/ In Progress/Not Done	
3	Make sure you lock the door during each break to ensure the security of the devices.		Done/ In Progress/Not Done	
4	At the beginning and end of each day of training and before participants take their lunch, ask participants about the battery status of their devices. Charge those who do not have much load.		Done/ In Progress/Not Done	
5	Charge the devices at the end of each day of training.		Done/ In Progress/Not Done	
6	Provide Group photo for the record		Done/ In Progress/Not Done	
7	All Training Teams converge and meet at the end of each day to update days progress		Done/ In Progress/Not Done	
	Other Logistics			
1	Training Refreshments and Meals		Done/ In Progress/Not Done	
2	Stationary		Done/ In Progress/Not Done	
3	Participants Transport Refund		Done/ In Progress/Not Done	
4	Training Teams Transport		Done/ In Progress/Not Done	
5	PPEs (Masks and Sanitizers)		Done/ In Progress/Not Done	

Appendix 6: Sample TOT Training Schedule

Day 1	Time	Day 2	Time	Day 3	Time	Day 4	Time
Arrival and Registration	8:30 AM	Arrival and Registration	8:30 AM	Arrival and Registration	8:30 AM		8:30 AM
Opening Prayer	9:00 AM	Opening Prayer	9:00 AM	Opening Prayer	9:00 AM	Opening Prayer	9:00 AM
Introductions		Recap of Day 1	9 :10am - 9:30am	Recap of Day 2	9 :10am - 9:30am	Recap of all	9 :10am - 9:30am
Opening Remarks		iCCM Assessments and treatments Tasks	9:30am - 10:00am		9:30am - 10:00am		9:30am - 10:00am
Climate Setting				Stock monitoring - ICCM trainers			
**TEA BREAK**	**10:00 am - 10:30 am**	**TEA BREAK**	**10:00am - 10:15am**	**TEA BREAK**	**10:00am - 10:15am**	**TEA BREAK**	**10:00am - 10:15am**
**Session 1**		iCCM	10:15am - 11:30am		10:15am - 11:30am		10:15am - 11:30am
Navigating the SmartPhone	**10:30 am - 11:30am**	*RMNCAH Family planning Pregnancy Registration ANC*	11:30am - 1:00pm	Stock monitoring	11:30am - 1:00pm	Troubleshooting	11:30am - 1:00pm
Accessing the Play store							
Navigating the CHW app.	11:30am - 11:45am						
Registering a new HH & WASH	11:45am - 1:00pm						
**Lunch Break**	**1:00pm - 2:00pm**	**Lunch Break**		**Lunch Break**		**Lunch Break**	
**Session 2: Service Delivery Areas**		*Delivery, PNC, Death*	2pm - 3pm	Supervisor Views	2pm - 3pm	closing remarks and planning	2pm - 3pm
Registering a new	2:00pm -4:00pm	RMNCAH -	3pm -4pm	DashBoards	3pm -4pm	Evening	4:00 PM

HH_*Cont*		Practice -				Tea and Departure	
Evening Tea and Departure	4:00 PM						

Appendix 7: Sample CHW Training Schedule

	Morning	Mid-Morning	Early Afternoon	Late Afternoon	Lead Entity
Sun 16th	Travel Day: District				
Mon 17th	District Engagements		Introducing eCHIS/Project		
Tue 18th	CHW App Walk through for District Leaders		Feedback Session with District Leaders		
Wed 19th	TOT - Health Service Areas for District Trainers, Digital Trainers and Health Assistants				
Thu 20th					
Fri 21st	TOT - eCHIS for District Trainers, Health Service Trainers and CHW Supervisors/Health Assistants				
Sat 22nd					
Sun 23rd	REST DAY				
Mon 24th	Health Service Refresher Training for CHWs - Sub County Based, led by MoH Trainers, CHW Supervisors/Health Assistants and Project Staff				
Tue 25th					
Wed 26th	eCHIS Digital App Trainings for CHWs - Subcounty-based, led by Project staff, CHW Supervisors/Health Assistants				
Thu 27th					
Fri 28th					
Sat 29th					
Sun 30th	REST DAY				
Mon 31st	Project Core Team Supporting District Kick-off and Going Live				
Tue 1st					
Wed 2nd	Project Core Team departs districts				

Appendix 8: eCHIS Deployment Budget Template

SN	Items	People	Days	Frequency	Rate	Amount (Ugx)	Notes
Training of district trainers (TOT) on e-CHIS							
1	Transport Refund of district team	45	5	1	30,000	6,750,000	
2	SDA district team	45	5	1	20,000	4,500,000	
3	DSA National facilitators (Travel day)	8	1	1	175,000	1,400,000	To Facilitate the Training , extra day is Travel Date
3	DSA National facilitators	8	5	1	135,000	5,400,000	Standard SDA minus Meals
4	Fuel for national facilitators	71	5	4	5,660	8,037,200	Calculated at 7 Kms per litre
5	Refreshments & Meals	45	5	1	45,000	10,125,000	As per standard rates for Meals and Refreshments
6	Stationary	45	1	1	5,000	225,000	
7	Airtime for coordination	1	1	1	50,000	50,000	
8	Hall Hire	1	5	1	200,000	1,000,000	
9	Airtime for Participants	53	1	1	30,000	1,590,000	
	**Sub Total**					**37,487,200**	
	Training of CHWs						
10	Transport Refund - CHWs	570	6	1	20,000	68,400,000	
11	DSA national facilitators	5	7	1	175,000	6,125,000	To Facilitate the Training , extra day is Travel Date
12	District SDAs	24	6	1	20,000	2,880,000	3 Trainers per 65 CHWs
13	Fuel for district facilitators	20	6	8	5,660	5,433,600	
14	Fuel for national facilitator	71	3	2	5,660	2,411,160	
15	Meals and Refreshments	646	6	1	45,000	174,420,000	To allow for connection in the training for the eCHIS
16	Stationary	570	1	1	3,000	1,710,000	
17	Hall Hire	8	6	1	100,000	4,800,000	Training shall be spread closer to the sub counties , 64 CHWs per training location
18	Airtime for Participants	599	1	1	30,000	17,970,000	
	**Subtotal**					**266,179,760**	
	Total					**0**	

Appendix 9: Change Review Form

THE REPUBLIC OF UGANDA
MINISTRY OF HEALTH

Change Review Form

Section A

**System**	**eCHIS -**
**Workflow/Workflow changes**	•
**Change Type**	New<br>Pending<br>Improvement
**Changes in Release**	•
**Items Being Changed**	List **ALL** items being changed i.e. user interface, workflow, health indicators

Installation Instructions:.

Section B

Attachments

Documentation Updates:

Signoff - MoH departments	*Name*	*Signature*	*Date*

Appendix 10: Monitoring and Evaluation Checklist - Community Level

General Details

District Name

____

Name of CHW

____

Duration using eCHIS? (months)

____

Highest Level of education

- ◯ PLE
- ◯ O'level
- ◯ A level
- ◯ None

Details

Do you meet with the Facility in the Last Quarter?

- ◯ Yes
- ◯ No

When was your last training on eCHIS? (Months)

____

When was your last Service Area Training? (Months)

____

Are you using paper-based Tools Alongside eCHIS?

- ◯ Yes
- ◯ No

Did you receive Data Bundles or Airtime last month?

- ◯ Yes
- ◯ No

If yes, how much?

____

Did you experience any technical issues while using the eCHIS?

- ◯ Yes
- ◯ No

Describe the Technical issues faced with eCHIS

____

Other Details

List other challenges faced with the eCHIS

____

List the suggested Solutions to some of these challenges

Appendix 11: eCHIS Deployment Monitoring Framework

eCHIS Deployment Monitoring Framework
**Result Statement:**<br>*By 2025, the health sector has institutionalized the use of patient-level digital systems at the point of care.*
**Parameter 1: Functionality**
**Key Monitoring Questions:**<br>*1.1 Does the technology or system work?*<br>*1.2 Does the technology or system operate as intended?*<br>*1.3 Does the technology or system perform its intended functions effectively?*<br>*1.4 Is the technology effectively adapted to the local context in terms of language, literacy, modifications for network coverage, etc?*
**Indicator List:**<br>1. *% CHWs with mobile phone/tablet signal at the time of deployment (i.e., training)*<br>2. *% CHWs with current access to a power source for recharging a mobile phone/tablet*<br>3. *% end-users with access to local technical support for troubleshooting*<br>4. *% devices that are not currently operational (misplaced/broken/not working)*<br>5. *% mobile devices that are operational in the language of end-users*<br>6. *% end-users who are literate in the language used by the digital health intervention*<br>7. *% data fields or elements from original paper-based system that are captured by the technology*<br>8. *# hours of initial training on the use/deployment of the eCHIS attended by end-users*<br>9. *# hours of refresher training on the use/deployment of the eCHIS attended by end-users*
**Means of Verification:**<br>*Reports (e.g., data/performance reviews, supportive supervision, regional/national telecommunication reports, surveys)*
**Parameter 2: Stability**
**Key Monitoring Questions:**<br>*2.1 Does the system consistently operate as intended?*
**Indicator List:**<br>1. *# hours of system/server downtime over reference period (i.e., last 1 month or 3 months)*<br>2. *% end-users reporting synchronization with server over reference period (i.e., last 1 month or 3 months)*<br>3. *% end-users reporting late synchronization with server over reference period (i.e., last 1 month or 3 months)*
**Means of Verification:**<br>*Reports (e.g., system-generated/audit trail)*
**Parameter 3: Fidelity**
**Key Monitoring Questions:**<br>*3.1 How do people interact with technology or system?*<br>*3.2 Do the realities of the field implementation alter the functionality and stability of the system, changing the eCHIS intervention from that which was intended?*<br>*3.3 Has the digital health system or technology been widely adopted?*<br>*3.4 Do the users find the technology easy to use?*<br>*3.5 Do the end-users find the health data/information received from the eCHIS intervention useful?*<br>*3.6 Are the end-users able to communicate with the eCHIS as intended?*<br>*3.7 Are the end-users responsive to the information received through the system?*

Indicator:

1. *% end-users that pass the assessment test during the training phase.*
2. *% end-users who demonstrate proficiency in use of the eCHIS*
3. *% intended end-users observed using the eCHIS*
4. *# transmissions sent by intended end-users over reference period (i.e., last 1 month or 3 months)*
5. *% end-users who rate the eCHIS as "easy to use"*
6. *% end users who rate the eCHIS as "transmits information as intended"*
7. *% end-users who report satisfaction with the content of health data/information received via the eCHIS*
8. *% end-users motivated/intending to use the eCHIS*
9. *# forms/amount of data transmitted by end-users via eCHIS within a reference period (i.e., last 1 month or 3 months)*
10. *% data fields or elements/forms that are left incomplete over reference period (i.e., last 1 month or 3 months)*

Means of Verification:

Reports (e.g., system-generated/audit trail, design workshop, training, data/performance reviews, supportive supervision)

Parameter 4: Quality

Key Monitoring Questions:

4.1 Is the content and the delivery of the eCHIS intervention of high enough quality to yield intended outcomes?

4.2 How does eCHIS improve service delivery?

4.3 How do improvements in service delivery affect health outcomes?

Indicators:

Client-Level;

1. *# minutes (reported or observed) between eCHIS prompt received about programmatic intervention and seeking care from provider (e.g., CHW, midwife, nurse, clinician)*
2. *# days duration of illness episode: disaggregated by illness/condition*
3. *# minutes spent with CHW in relation to health intervention at the last visit*
4. *% target individuals who report receiving health information about a programmatic intervention via their mobile phone within reference period*
5. *% target individuals or caregivers who report contact with a qualified health-care provider using eCHIS in relation to a programmatic intervention over reference period*
6. *% target individuals or caregivers who report adequate knowledge about signs and symptoms*
7. *% target individuals or caregivers who report adequate knowledge about the health issues relevant to a programmatic intervention*
8. *% changes in reported individual level out-of-pocket payments for illness management over reference period (through managing the illness by phone-based consultation instead of visiting a health care facility, e.g., travel cost)*

Provider-Level;

1. *# minutes (reported or observed) for last client counseling about a health intervention using eCHIS*
2. *# minutes or hours (reported or observed) spent on health record-keeping about a health intervention over reference period*
3. *# minutes (reported or observed) used per individual CHW over reference period to transmit data relating to a health intervention from community-based logs to health-care facility-based information systems*
4. *# minutes (reported or observed) used per individual CHW to report important adverse events (e.g., stock-outs)*
5. *# of CHWs who report adequate knowledge of the health issue relevant to a health intervention*
6. *% care standards relating to a health intervention observed to be met using eCHIS during client-provider consultation*
7. *% CHWs observed to be using eCHIS during their patient consultations*
8. *% target CHWs who use eCHIS in relation to relevant health interventions through their mobile phones over reference period*
9. *Amount of cost savings (estimated) due to improvement in service delivery/efficiency/other factors.*
10. *# clients (average or total) attended by a CHW using eCHIS over reference period*

Health System-Level;

1. *# minutes (cumulative) over reference period for all CHWs using eCHIS to enter data related to a health intervention*
2. *# days over reference period for which a CHW reports stock-out of a commodity essential for provision of a health programmatic intervention*
3. *% change in reported stock-out events of a commodity essential for service delivery over reference period, disaggregated by relevant health interventions*
4. *% change in data entry errors over reference period*
5. *% target end-users who receive training on management and use of eCHIS to deliver quality service delivery, disaggregated by initial and refresher training*
6. *# individuals seeking health care over reference period, disaggregated by relevant health interventions*
7. *% individuals in a specific geographical area who receive health care through eCHIS over reference period, disaggregated by relevant health interventions*
8. *% change in costs of transporting HMIS paper forms and manual data entry over reference period*
9. *% change in costs of human resources for data entry*
10. *% change in costs associated with timely and appropriate management of illness*
11. *% changes in reported individual out-of-pocket payments for management of illness*
12. *Total population-level savings in out-of-pocket payments attributed to timely and appropriate care seeking*

**Means of Verification:**<br>*Reports (e.g., system-generated/audit trail, design workshop, training, data/performance reviews, supportive supervision)*

Appendix 12: eCHIS Guidelines Use Monitoring Framework

**Guideline-Use Monitoring Framework**
**Parameter 1:** Dissemination
**Key Monitoring Questions:**<br>*1.1 What is the version of the current guideline document?*<br>*1.2 Has the current guideline document been disseminated?*<br>*1.3 If yes under [1.2] above, through which channels?*<br>*1.4 What is the proportion of target entities possessing at least one copy of the guideline document? – disaggregated by the Ministry of Health, District, Health Facility, Partners.*
**Indicator:**<br>*% of target entities who received at least one copy of the guideline document, disaggregated by: (a) Ministry of Health; (b) District; (c) Health Facility; (d) Partners*
**Means of Verification:**<br>*Reports (e.g., dispatch, supportive supervision)*
**Result Statement:**<br>*Existing version of the Guidelines for Implementing the Uganda electronic Community Health Information System disseminated to key stakeholders nationwide.*
**Parameter 2:** Training
**Key Monitoring Questions:**<br>*2.1 Has training on the current version of the guidelines been conducted?*<br>*2.2 If yes under [2.1], what was the proportion of individuals trained on the various components of the guideline document? – disaggregated by (a) Ministry of Health; (b) District; (c) Health Facility; (d) Partners*
**Indicator:**<br>% of target entities who received at least one copy of the guideline document, disaggregated by: (a) Ministry of Health; (b) District; (c) Health Facility; (d) Partners
**Means of Verification:**<br>Reports (e.g., training, supportive supervision)
**Result Statement:**<br>*Key stakeholders nationwide oriented on the existing version of the Guidelines for Implementing the Uganda electronic Community Health Information System.*
**Parameter 3:** Compliance
**Key Monitoring Questions:**<br>*3.1 Are the various target groups implementing eCHIS in accordance with established guidelines? Please provide comment by target group and key area as appropriate*<br>● ***Target Group:*** *(a) Ministry of Health; (b) District; (c) Health Facility; (d) Community Health Workers; (e) Partners*<br>● ***Key Component:*** *(a) eCHIS User Requirements, Design and Development; (b) Implementing eCHIS; (c) System and Data Access; (d) Governance Structure (e)Monitoring and Evaluation*
**Indicator:**<br>*Target entities are compliant with existing version of the guidelines – disaggregated by (a) Ministry of Health; (b) District; (c) Health Facility; (d) Community Health Workers; (e) Partners.*<br>*Use Likert scale: 1-5, where 1 = strongly agree, 2 = agree, 3 = neutral, 4
**Means of Verification:**<br>Reports (e.g., design workshop, training, supportive supervision)
**Result Statement:**

Key stakeholders nationwide are compliant with existing Guidelines for Implementing the Uganda electronic Community Health Information System.

Appendix 13: eCHIS Evaluation Framework

<table>
<tr><th colspan="3">eCHIS Evaluation Framework</th></tr>
<tr><td rowspan="2">Formative</td><td>Evaluation Type: Process Evaluation
Objective: Measure outputs attributed to eCHIS intervention activities and inputs; done either as a one-time assessment and/or continuously.
Key Question(s):
● Is the eCHIS intervention operating as intended?</td><td>Timeline:TBD</td></tr>
<tr><td>Evaluation Type: Implementation Evaluation
Objective: Monitor the fidelity of the eCHIS intervention holistically or technology system.
Key Question(s):
● Is eCHIS implementation occuring in accordance with the original and existing standard operating procedures and/or protocols?</td><td>Timeline:TBD</td></tr>
<tr><td rowspan="3">Summative</td><td>Evaluation Type: Performance or Outcome Evaluation
Objective: Measure the effectiveness of eCHIS intervention activities on immediate and intermediate changes in key outcomes, including knowledge, service provision, utilization and coverage.
Key Question(s):
● Are the health services available? What is eCHIS intervention’s effect on changes in service delivery?
● Are the health services being utilized?
● Did eCHIS increase coverage of the relevant health interventions? Is the target population being reached?</td><td>Timeline:TBD</td></tr>
<tr><td>Evaluation Type: Impact Evaluation
Objective: Measure the long-term net effects or impact of the intervention on key health outcomes, including mortality, morbidity, and disease risk, at community level or higher.
Key Question(s):
● Were there improvements in disease or mortality patterns, or health-related behaviors?</td><td>Timeline:TBD</td></tr>
<tr><td>Evaluation Type: Economic Evaluation
Objective: Determine a probable value for money from investment made in eCHIS roll-out.
Key Question(s):
● What is the incremental cost-effectiveness of the eCHIS intervention as compared to existing services?</td><td>Timeline:TBD</td></tr>
</table>

Appendix 13: Standard Operating Procedure for eCHIS Downtime

Electronic Community Health Information System (eCHIS) Downtime Standard Operating Procedure (SOP)

1. Purpose

This SOP outlines the procedures to follow when the eCHIS system is unavailable, ensuring continuity of patient care and data integrity for community health workers (CHWs) in Uganda.

2. Scope

This SOP applies to all CHWs, including Village Health Teams (VHTs) and Community Health Extension Workers (CHEWs), using the eCHIS system for health service provision at the community level.

3. Problem Statement

In Uganda, many eCHIS system users (CHWs) face challenges in synchronising their field activities, including various assessments, due to server unavailability and network traffic which may last for more than a week or more. As a result, CHWs often resort to using paper-based HMIS paper forms as an alternative which poses a high risk of data inconsistencies. They then transfer the data to the eCHIS system once it is restored or available. Currently,Over 60% of the CHWs have reported this issue across the districts implementing eCHIS.

4. Definitions

Synchronisation Issue: A situation where CHWs cannot synchronise (send) offline eCHIS data with the central server due to network unavailability, Network traffic or technical problems.

Downtime: A period when a system is unavailable for use, whether due to a planned outage, unexpected failure, or maintenance.

Data Integrity: The accuracy, consistency, and reliability of data throughout its lifecycle, ensuring it remains intact during downtime and subsequent synchronisation.

ODK Form: A digital form used as an alternative data capture tool when the eCHIS system is unavailable. It is designed to be easily integrated with the eCHIS server once the system is restored.

Health Facility Supervisors: Individuals responsible for overseeing the work of CHWs, ensuring data is captured accurately, and resolving or escalating system issues promptly.

DHT: A group of Digital health officials at the district level responsible for monitoring and managing the performance of health systems, including eCHIS, and coordinating with higher authorities and technical teams for issue resolution.

MOH: The technical team responsible for maintaining and troubleshooting the eCHIS system, providing solutions, and implementing workarounds during downtime.

5. Responsibilities

CHWs: Responsible for capturing patient data using alternative methods during downtime and ensuring data is transferred to the eCHIS system once available.

Health Facility Supervisors: Must replicate and document reported issues within a day and either resolve the problem or escalate it to the DHT through the toll free/ helpline 0800 203 033 as soon as they register 5 and more cases.

DHT: Required to provide a solution within two days of escalation from the supervisor or communicate the need to use alternative systems.

Implementing Partners and Biostatisticians: Support data management and ensure data integrity during downtime and recovery periods.

MOH: Expected to resolve the issue within a week or provide a workaround. If the issue requires more time for investigation, they must communicate this to the DHT.

6. Downtime Procedures

6.1 Identification and Reporting

Immediate Reporting: CHWs should immediately report synchronisation or downtime issues to their supervisors.

Replication and Escalation: Health Facility Supervisors must replicate the reported issue within one day. If the issue is unresolved, it must be escalated to the DHT.

6.2 Data Capture during Downtime

Alternative Data Capture: CHWs shall use the ODK form, developed as a temporary measure when the eCHIS system is down, instead of paper-based HMIS forms.

Data Integrity: The ODK form ensures data is captured accurately and can be easily integrated into the eCHIS system once it is restored.

6.3 Data Synchronisation Post-Downtime

Prompt Synchronisation: Upon restoration of the eCHIS system, CHWs should promptly transfer data from the ODK forms to the eCHIS system.

Verification: Supervisors should verify that all data has been successfully synchronised.

6.4 Communication Protocol

Level 1: CHWs to Health Facility Supervisors: CHWs report issues immediately. Supervisors have one day to replicate and resolve or escalate the issue.

Level 2: Health Facility Supervisors to DHT: If escalated, the DHT must provide a solution within two days or communicate the use of alternative systems.

Level 3: DHT to MOH: Medic is responsible for resolving the issue within a week. If the issue requires further investigation, Medic must inform the DHT, providing an estimated timeline and potential workarounds.

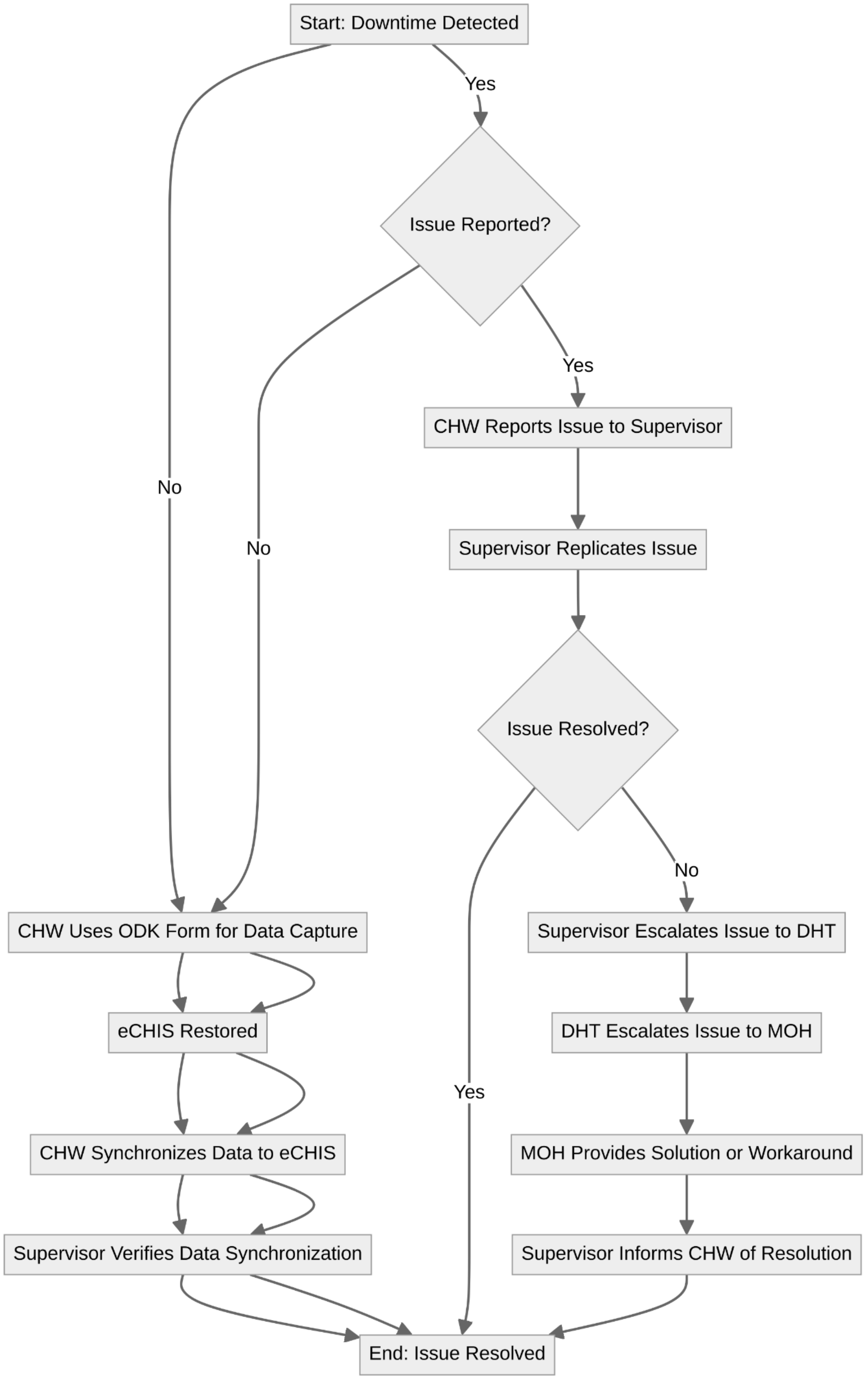

Fig 1 : Communication and Resolution Process flow

7. Training

Initial Training: CHWs shall receive comprehensive training on eCHIS data synchronisation procedures, the use of HMIS paper forms, and the ODK form.

Refresher Training: Monthly refresher training sessions shall be conducted to ensure CHWs remain proficient in these procedures.

8. Monitoring and Review

Monitoring Tools: The DHT will utilise tools like Watchdog, Kibana, and Grafana to monitor eCHIS performance, identify potential issues proactively, and prevent downtime.

Review and Improvement: This SOP will be reviewed periodically to incorporate feedback from CHWs, Health Facility supervisors, and other stakeholders, ensuring it remains effective and responsive to the needs of the health system.

THE REPUBLIC OF UGANDA

THE NATIONAL BUDGET FRAMEWORK PAPER FY 2023/24 – FY 2027/28

MINISTRY OF FINANCE, PLANNING AND ECONOMIC DEVELOPMENT

DECEMBER 2022

TABLE OF CONTENTS

TABLE OF CONTENTS 1
1.1 INTRODUCTION 3
Legal and Policy Framework 3
1.2 MEDIUM TERM MACROECONOMIC POLICY FRAMEWORK 5
Real GDP Growth 5
Inflation 7
Interest Rates 8
Exchange Rate 9
Employment 9
External Sector Developments 10
Economic Growth Strategy in FY2023/24 13
1.3 MEDIUM TERM FISCAL FRAMEWORK 14
Fiscal Strategy 14
Domestic Revenue 16
Government Expenditure and net lending 16
External Borrowing 16
Domestic Borrowing 17
Debt Repayments 17
Interest Payments 17
1.3 STATEMENT OF RESOURCE FOR THE ANNUAL BUDGET FY 2023/24 17
1.3.1 RESOURCE ENVELOPE FOR FY 2023/24 18
1.5 UPDATE ON DEBT SUSTAINABILITY 19
1.6 COMPLIANCE WITH THE CHARTER FOR FISCAL RESPONSIBILITY 20
1.7 FISCAL RISK STATEMENT AND MITIGATION MEAUSURES 21
1.8 BUDGET STRATEGY FY 2023/24 26
1.9 POLICY PROPOSALS FOR FY 2023/24 BUDGET 45

Part 2: Details of Proposed Programme Plans and Expenditure

Agro-Industrialization.. 50

Mineral Development.. 64

Sustainable Petroleum Development.. 74

Manufacturing.. 84

Tourism Development... 89

Natural Resources, Environment, Climate Change, Land and Water Management 101

Private Sector Development.. 122

Sustainable Energy Development... 133

Integrated Transport Infrastructure and Services... 145

Sustainable Urbanization and Housing... 162

Digital Transformation.. 174

Human Capital Development... 181

Innovation, Technology Development and Transfer.. 218

Public Sector Transformation.. 227

Community Mobilization and Mindset Change.. 246

Governance and Security... 255

Regional Balanced Development... 281

Development Plan Implementation... 289

Administration of Justice.. 298

Legislation, Oversight and Representation.. 320

Annexes

Annex 1: Programme Allocations For 2023/24.. 331

Annex 2: Medium Term Expenditure Framework (MTEF) FY 2022/23 – FY 2024/25 332

Annex 3: Sub SubProgramme Allocations For FY 2023/24.................................... 375

Annex 4: Approved Budget and Quarter One Outturns For FY 2022/23 by Sub Subprogramme.. 395

Annex 5: Local Government Indicative Planning Figures For FY 2023/24................ 412

Annex 6: Allocation Criteria For Transfers to Local Government FY 2023/24.......... 422

Part 1: Medium Term Macroeconomic Plan and Fiscal Framework

1.1 INTRODUCTION

Legal and Policy Framework

The National Budget Framework Paper (NBFP) for FY 2023/24 has been prepared in line with Section 9 (3) and Section 9 (5) of the Public Finance Management Act, 2015 (Amended) which requires the Minister of Finance, Planning and Economic Development to seek approval by Cabinet and submit it to Parliament by 31st December.

The theme of the National Budget Framework Paper for FY2023/24 is "***Full Monetisation of the Ugandan Economy through Commercial Agriculture, Industrialisation, Expanding and Broadening Services, Digital Transformation and Market Access".*** This is anchored on the overall East Africa Community's agenda of ***"Accelerating Economic Recovery and Enhancing Productive Sectors for Improved Livelihoods"*** *and* in agreement with the Sustainable Development Goals.

For purposes of continuity in the realization of our development agenda for socio-economic transformation for all Ugandans, the above budget theme is the same as that of this FY 2022/2023.

The National Budget Framework Paper takes into consideration the National Development Plan III, the Charter for Fiscal Responsibility, and requirements of Gender and Equity Responsiveness and balanced development.

The preparation of the National Budget Framework Paper for the FY 2023/24 involved extensive budget consultations both at the national and local government levels. This included inter-ministerial budget reviews to get consensus on the priority programme interventions for the fiscal year 2023/24 and rationalise expenditures, including sequencing of multiyear interventions to fit within the constrained budget. Further discussions were held with the Private Sector and Development Partners, in order to

understand the socio-economic challenges they face today, the actions necessary to harness opportunities in the third National Development Plan (NDPIII),the NRM Manifesto 2021 – 2026 and the business and economic recovery programes

The NBFP for FY 2023/24 has two parts: -

1. Part 1 sets out the Government's Economic Growth Strategy, Medium Term macroeconomic forecast, Medium Term Fiscal Framework and Forecast, compliance with the Charter for Fiscal Responsibility, the Resource Envelope for FY 2023/24, Policy measures, Medium Term Expenditure Framework FY 2023/24 and Fiscal Risks and Mitigation measures ;
2. Part 2 provides details of the proposed programme plans, interventions and expenditures in line with the budget strategy and the NDPIII reprioritized Programme Implementation Action Plans

1.2 MEDIUM TERM MACROECONOMIC POLICY FRAMEWORK

The National Development Plan III has a key focus of attaining sustainable industrialization for inclusive growth, employment, and sustainable wealth creation. Government remains committed to supporting initiatives which accelerate and sustain inclusive economic growth without compromising macroeconomic stability, debt sustainability and green growth. This will be through the following measures: focus expenditure on interventions that have high multiplier effects on the economy; import replacement and export promotion; job creation; enhance domestic revenue mobilization and public debt management. Further still, Government will continue to use coherent, cohesive, and well-coordinated fiscal and monetary policies to support economic recovery and to minimise the impact of external economic shocks on Uganda's economy. Table 1 provides the key macroeconomic assumptions underlying the macroeconomic policy framework for FY 2023/24 and the medium term.

Table 1: Key Macroeconomic Assumptions (Interest rate, exchange rate and employment missing)

Macroeconomic Assumptions	Outturn	Outturn	Proj Outtu	Proj	Proj	Proj	Proj
	2020/21	2021/22	2022/23	2023/24	2024/25	2025/26	2026/27
**Nominal GDP at Market Price**	148,310	162,721	184,254	208,356	233,286	261,232	292,542
Real GDP growth	3.5%	4.7%	5.3%	6.0%	6.5%	7.0%	7.2%
Headline Inflation (period aver	2.5%	3.4%	8.3%	7.2%	5.5%	5.0%	4.9%
Core Inflation (period average)	3.5%	3.2%	7.5%	6.8%	5.0%	4.4%	4.5%

Source: Ministry of Finance, Planning and Economic Development

Real GDP Growth

Since the onset of COVID-19 pandemic, Uganda's economic growth trajectory declined from 6.4% in FY 2018/19 to 3.5% in FY 2020/21. However, the economy has been on a steady recovery from the negative effects of the pandemic following the full re-opening of the economy, Government supportive measures to the private sector and the prudent

fiscal and monetary policies. The GDP growth rate has revamped to 4.7% in FY 2021/22 up from 3.5% in FY 2020/21 as shown in Fig.1 due to higher growth rates in industry and services sectors.

Figure 1 shows Uganda's economic growth trajectory since FY 2019/20.

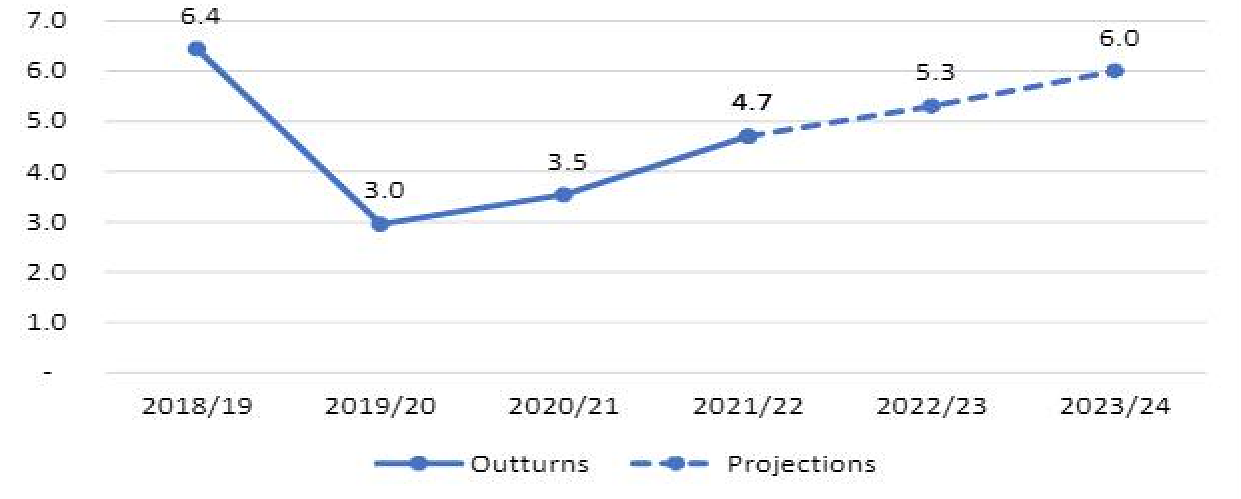

Figure 1: GDP growth rates at market prices

Source: UBOS and Ministry of Finance, Planning and Economic Development

The services sector grew by 4.1% in FY 2021/22 from 2.8% registered in FY 2020/21. This was on account of strong growth in real estate activities and health-related services. In addition, wholesale & retail trade activities, and education services recovered following the full reopening of the economy.

The industry sector expanded by 5.1% from 3.5% the previous fiscal year, due to robust growth in the manufacturing subsector. This was mostly due to increasing activity in meat processing and preservation, grain milling, and pharmaceuticals. Construction, mining, and quarrying sub-sectors also contributed to industrial sector growth.

There was modest growth in the agriculture, forestry, and fishing sector to 4.4% from 4.3% the previous financial year. This was on account of recovery in fishing activities which grew by 0.8% from -8.8% in FY

2020/21; growth in livestock output by 8.3% from 7.8% in FY 2020/21 and sustained growth in food and cash crop production.

In FY 2023/24, economic growth is expected to strengthen to 5.3%, supported by Government initiatives like the Parish Development Model and EMYOOGA; the pickup in oil sector construction activities; growth in regional trade; and a rebound in agricultural production owing to Government interventions into agricultural productivity.

Over the medium term, economic growth is projected to average between 6% and 7%, driven by anticipated increase in productivity within agriculture and manufacturing sectors-supported by Government interventions; recovery in the private sector activity; public infrastructure investments and operations in the oil and gas sector.

Inflation

Annual headline inflation in November 2022 reduced to 10.6%, from 10.7% registered in October 2022. This was mainly driven by a reduction in annual inflation for energy, fuel & utilities as well as the core basket. Particularly, prices for petrol, diesel, transport services, firewood, paraffin and propane gas declined compared to the previous month as shown in Figure 2.

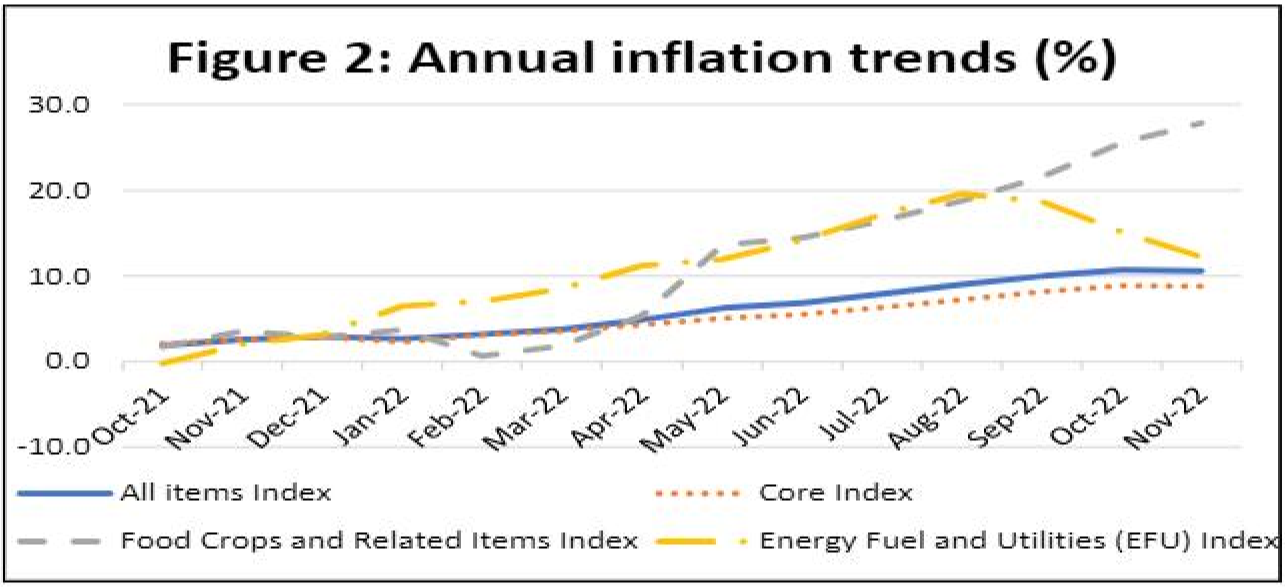

Source: Uganda Bureau of Statistics

For FY 2022/23 and 2023/24, annual headline inflation is projected to average 8.3% and 7.2% respectively and is expected to reduce to the 5% target over the medium term.

Compared to our regional neighbours, annual headline inflation continued on an upward trend for Rwanda, increasing to 33.8% in November from 31.0% the previous month. This was mainly driven by a surge in the prices for food and non-alcoholic beverages. On the other hand, Kenya's annual headline inflation slightly declined to 9.5% in November from 9.6% in October, as fuel and food prices eased. Tanzania's inflation remained at 4.9%-the same rate recorded the previous month.

Interest Rates

The Commercial Bank's Shilling denominated lending rates increased to a weighted average of 18.42% in October 2022 from 18.24% in September 2022, attributed to a rise in the Central Bank Rate from 6.5% in April to 10% in November 2022.

Interest rates (yields) on Treasury Bills marginally reduced to 11.39%, and 13.27% for the 91-day and 182-day tenors, respectively in November 2022 from 11.47%, and 13.68%, respectively in October 2022. However, the yields increased to 15.40% in November 2022 from 15.25% in October 2022 for the 364-day tenor.

Exchange Rate

The Ugandan Shilling appreciated by 1.6% percent against the US$ in November 2022, trading at an average rate of UShs 3,760.23/US$ compared to UShs 3,822.27/US$ in October 2022, as supply for the dollar outstripped its demand during the month. This recent appreciation was mainly due to higher export earnings and tourism receipts. However, on an annual basis, the US Dollar depreciated by 5.9% from UShs 3,551.52/US$ in November 2021 to UShs 3,760.23/US$ in November 2022.

Going forward, the shilling is expected to depreciate against the US Dollar due to the global strengthening of the US Dollar following the increase in the policy rate by the federal reserve of USA. The higher interest rate in USA results in higher demand for the US$ as international investors shift assets into USA to benefit from better interest rates. This is in addition to other reasons like perceived safety (reduced risk) and better economic growth prospects in the USA.

Employment

The National Labour Force Survey (NLFS) carried out in 2021 revealed that the working age population (14-64 years) was estimated at nearly 23 million of the total population with more females (52 percent) than males (48 percent). Among the working age population, 87 percent (20m) were working including other subsistence work. About 40.2 percent of the

working population was in subsistence agricultural work an increase from 39.9 percent in UNHS 2019/20.

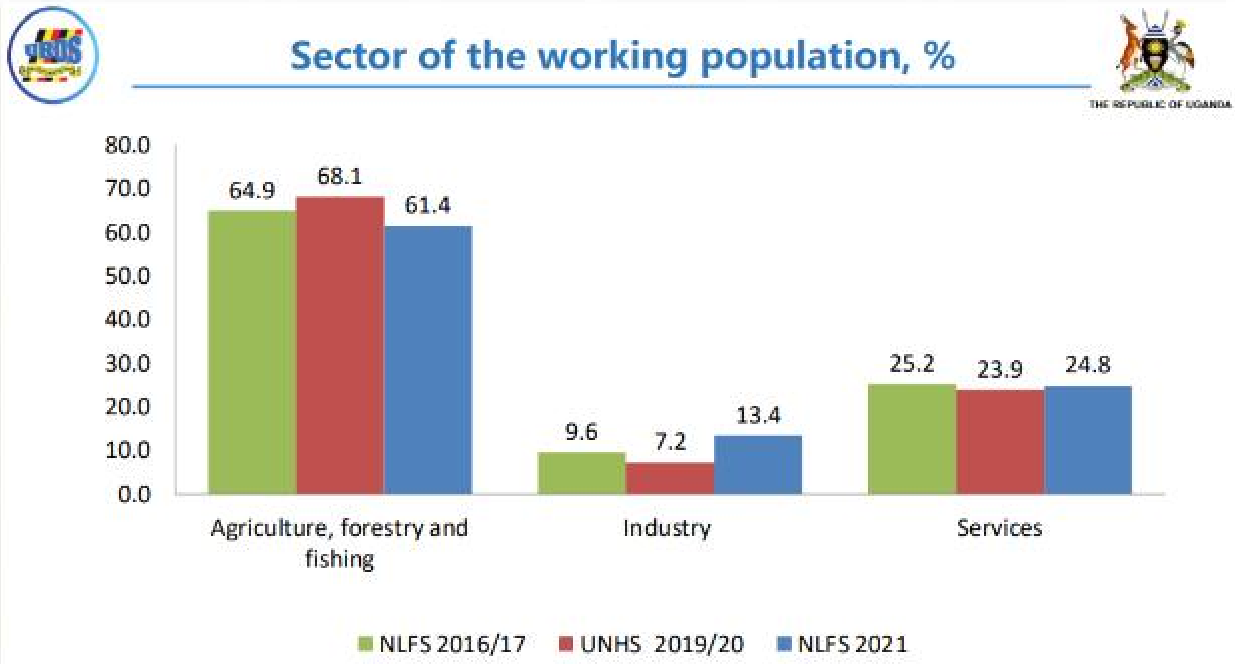

Source: UBOS

The Uganda National Household Survey FY 2019/20 and the National Labour Force Survey of 2021 show a shift in proportion of the working population from agriculture to industry and services sectors.

The proportion of the working population employed in Agriculture reduced from 68.1 percent to 61.4 percent while it increased to 13.4 percent and 24.8 percent from 7.2 percent and 23.9 percent in Industry and Services respectively.

External Sector Developments

The merchandise trade deficit widened to US$ 301.58 million in October 2022 because the import bill grew faster than export receipts. The Merchandise Export receipts increased by US$ 63 million to US$ 348.8 million in October 2022 from US$ 286.3 million in October 2021, mainly due to higher coffee exports, maize, fish and its products, among others. On the other hand, the merchandise imports increased by US$ 119

million to US$ 650.48 million in October 2022 from US$ 531.50 million in October 2021, on account of higher import bill for petroleum products, machinery equipment, vehicles & accessories, chemical and related products, among others.

The EAC remained the major destination of Uganda's exports accounting for 55.6% (USD 193.9 million) followed by European Union with a share of 17.7% (USD 61.8 million). Within EAC, 82.5% of our exports went to South Sudan, Democratic Republic of Congo, and Kenya.

External Sector-Annual Basis

Uganda's current account deficit declined by 6.42 percent from US$ 3,837 million in FY 2020/21 to US$ 3,590.8 million in FY 2021/22. This development was largely on account of an increase in budget support grants and a pickup in tourism activity following the disruptions occasioned by the COVID-19 pandemic. Table 2 shows a summary of the current account and its components for the last 5 Financial Years.

Table 2: Selected Balance of Payments flows (USD Millions)

	FY 2017/18	FY 2018/19	FY 2019/20	FY 2020/21	FY 2021/22
**Current account**	-1,798.6	-2,631.7	-2,512.5	-3,837.0	-3,590.8
**Trade Balance**	-2,447.0	-3,464.5	-3,744.8	-4,941.8	-4,623.9
Exports of Goods and Services	5,487.0	6,271.9	5,358.1	6,701.5	5,579.6
Imports of Goods and Services	7,934.0	9,736.5	9,102.9	11,643.4	10,203.5
**Primary income**	-926.4	-915.9	-633.5	-680.2	-778.5
**Secondary income**	1,574.8	1,748.7	1,865.7	1,785.1	1,811.6
o/w Remittances	1,252.4	1,369.1	1,291.6	1,154.3	1,133.9
**FDI Inflows**	929.0	1,217.4	997.5	920.0	1,218.4

Source: Bank of Uganda

From table 2, Uganda's trade deficit narrowed from US$ 4,941.8 million in FY 2020/21 to US$ 4.623.9 million in FY 2021/22. This was as a result of a reduction in the import bill. The decrease in the import bill was explained by a decline registered for government project imports, textiles

& textile products as well as machinery equipment and vehicles & accessories during the year.

Just like the imports, there was a reduction in exports-mainly attributed to a significant decline in maize and tobacco receipts. The reduction in maize export quantities was explained by the restrictions imposed by the Kenyan government, citing high levels of aflatoxins in Uganda's maize

Remittances to Uganda fell marginally by 1.8 percent from US$ 1,154.3 million in FY 2020/21 to US$ 1,133.9 million in FY 2021/22, as economies of migrant-hosting nations continued to recover from the effects of the COVID-19 pandemic.

FDI inflows on the other hand increased by 32.4 percent from US$ 920 million in FY 2020/21 to US$ 1,218.4 million in FY 2021/22. This was largely attributed to the increased investments in the oil sector given the completion of the Final Investment Decision.

Trade between Uganda and EAC Partner States

Table 3a: Merchandise Trade Balance between Uganda and EAC Partner States (USD Million)

	FY 17/18	FY 18/19	FY 19/20	FY 20/21	FY 21/22
Exports	1,579.3	1,254.6	1,107.7	1,264.3	1,544.6
Imports	798.6	1,147.0	1,301.0	2,103.2	1,132.4
Merchandise Trade Balance	780.7	107.5	-193.4	-838.9	412.2

Source: Bank of Uganda

Table 3b: Merchandise Trade Balance by country (USD Million)

	FY 17/18	FY 18/19	FY 19/20	FY 20/21	FY 21/22
Kenya	338.0	-176.1	-237.1	-226.8	-137.4
Burundi	-21.1	33.5	38.0	39.5	86.8
Rwanda	233.3	157.2	3.5	2.6	-4.6
South Sudan	356.5	410.1	325.4	442.1	624.3
Tanzania	-126.1	-317.1	-323.2	-1,096.3	-156.8
**EAC**	**780.7**	**107.5**	**-193.4**	**-838.9**	**412.2**

Source: Bank of Uganda

In FY 2021/22, Uganda traded at a surplus with EAC Partner States due to both a growth in exports to the region and a reduction in imports. The country traded at surpluses with South Sudan (US$ 624.3 million) and Burundi (US$ 86.8 million) and deficits were recorded for the other countries. There was a significant decline in the deficit with Tanzania from US$ 1,096.3 million to US$ 156.8 million.

Economic Growth Strategy in FY2023/24

Economic growth strategy in the short to medium term has a dual objective of building a self-sustaining economy to withstand future shocks and to harness resources for inclusive economic growth. These are to be attained while maintaining a stable macroeconomic environment. A self-sustaining economy will be achieved by: undertaking evidence-based actions for policy implementation; allowing market based economic dynamics and close coordination between fiscal and monetary policies to counter cyclical measures and augmenting investment in the Parish Revolving Fund (PRF) to avail liquid capital to the vulnerable households without collateral to access credit in the financial market. This will enhance growth in private and public investments, hence increased employment creation and mobilisation of more domestic revenue. Furthermore, inclusive growth will be attained by the economic policy in the Financial Year 2023/24 and in the future will therefore seek to achieve the following;

i) Ensure peace and stability through enhanced security and Macroeconomic stability, with critical coordination of both the monetary and fiscal policy measures.

ii) Mitigate the impact of internal and external shocks through building buffers that cushion the country against economic shocks and maintaining a competitive environment that supports continuous supply of goods and services.

iii) Mobilisation of resources to sustain the Parish Development Fund (PDF) and strict follow up on resource use for effective investment outcomes.

iv) Maximization of returns to public investments through implementing reforms geared towards strengthening Public Investment Management Systems.

1.3 MEDIUM TERM FISCAL FRAMEWORK

Fiscal Strategy

The overarching goal of the fiscal strategy is to attain inclusive economic growth, while maintaining a stable macroeconomic environment and preserving debt sustainability. This will be attained through continued investment in public infrastructure for inclusive growth and implementation of the Domestic Revenue Mobilisation Strategy (DRMS) which targets revenue to GDP growth of 0.5% every fiscal year.

In accordance with the Charter for Fiscal Responsibility (FY2021/22-2025/26), the total debt in nominal terms is to be maintained below 50% of the GDP, while the fiscal balance including grants shall not exceed 3.0 percent of non-oil GDP by FY 2025/26. To attain the required fiscal balance, Government is harnessing revenue mobilisation while ensuring that the rise in recurrent spending matches the efficiency in revenue mobilisation. Furthermore, Government is prioritizing spending on

sectors with high investment multipliers and households in the subsistence economy in line with objective of full monetization of the economy.

Table 4 below shows the fiscal framework for FY2023/24 and the medium term.

Table 4: Medium Term Fiscal Framework

Projected fiscal operations (Shs bn)	Budget.	Proj.	Proj.	Proj.	Proj.	Proj.
	2022/23	2023/24	2024/25	2025/26	2026/27	2026/27
**Total revenue and grants**	27,719	31,022	35,510	43,136	51,238	59,879
Revenue	25,551	28,831	33,394	41,301	49,681	58,555
Tax revenue	23,755	26,810	31,044	35,056	41,072	47,839
Non-tax revenue (including AIA)	1,796	2,021	2,298	2,572	2,827	3,541
o/w Appropriation in Aid	776	1,038	1,166	1,306	1,463	1,636
Grants	2,169	2,191	2,117	1,835	1,558	1,325
Budget support	78	40	30	31	0	0
Project grants	2,090	2,152	2,087	1,804	1,558	1,325
**Expenditures and net lending**	37,472	37,247	41,727	50,206	57,749	64,090
Current expenditures	22,244	19,118	21,157	25,186	27,920	25,613
Wages and salaries	6,363	6,426	7,384	8,709	10,574	6,194
Interest payments and commitment fees	4,692	6,136	5,812	6,029	6,160	7,194
Other current spending	11,189	6,557	7,961	10,447	11,187	12,224
Development expenditures	14,268	17,929	20,370	24,820	29,092	38,269
External	6,418	8,044	9,317	11,869	13,257	14,357
Domestic	7,850	9,886	11,053	12,951	15,835	23,912
**Overall balance**	**-9,753**	**-6,225**	**-6,217**	**-7,070**	**-6,511**	**-4,211**
Primary balance	-5,061	-8,416	-8,334	-8,905	-8,069	-5,535
Financing	9,753	6,225	6,217	7,070	6,511	4,211
External financing (net)	4,745	5,891	4,068	6,110	7,444	6,312
Disbursement	7,157	8,344	7,231	10,065	11,699	13,032
Budget financing loan	2,531	2,452	0	0	0	0
Concessional project loans	3,290	3,078	3,435	3,933	5,666	11,557
Non-concessional loans	298	2,813	3,795	6,132	6,033	1,475
Amortisation (-)	-2,412	-2,453	-3,163	-3,955	-4,255	-6,721
Domestic financing (net)	5,008	334	2,149	959	-933	-2,101
Bank financing	2,520	-460	1,072	-426	-2,315	-3,470
Bank of Uganda	-7,965	-10,050	-8,331	-7,209	-9,093	-10,233
o/w: domestic refinancing	-8,008	-8,799	-8,331	-5,402	-5,401	-5,400
Commercial banks	10,486	9,590	9,404	6,782	6,778	6,763
o/w: securities for fiscal purposes	2,478	791	1,072	1,380	1,377	1,363
o/w: securities for domestic amoritisation	8,008	8,799	8,331	5,402	5,401	5,400
Non-Bank financing	2,488	794	1,077	1,386	1,382	1,369
Errors and omissions/gap	0	0	0	0	0	0

Notes: figures net of Heavily Indebted Poor Countries (HIPC) debt relief unless stated otherwise. In the outer years, projections for project grants, external development expenditure, and concessional project loans may change when due to forecasted new projects not yet allocated in the MTEF.

Source: Ministry of Finance, Planning and Economic Development

Domestic Revenue

In FY 2023/2024, domestic revenues are projected to amount to UShs 28,831.1 billion (13.8% of GDP), from an estimate of UShs 25,550.7 billion. This translates into nominal growth in revenues of UShs 3,280 billion. 93% of domestic revenues will be obtained from tax revenues (UShs 26,810.2 billion) while the remainder will be obtained from Non-Tax Revenue (UShs 2,020.8 Billion). This rise is attributed to gains on account of higher economic growth, and projected revenue gains due to implementation of the Domestic Revenue Mobilisation Strategy (DRMS).

Over the medium term, domestic revenues are projected to grow by 0.5% of GDP in line with the target set out in the Charter for Fiscal Responsibility and the DRMS).

Government Expenditure and net lending

Expenditure and net lending in FY 2023/24 are projected to amount to UShs 37,247.4 billion (see table 4 above). This is slightly less than UShs 37,471.9 billion in the Approved Budget of FY 2022/23. Over the medium term, current expenditures will average 10.8% of GDP while development expenditures will average 8.5% of GDP.

External Borrowing

A total of UShs 8,343.8 billion is projected as external financing in FY 2023/24. Of this, UShs 2,452.1 billion will be obtained as budget financing loans and UShs 5,891.7 billion from project loans. Majority of

project loans (UShs 3,078.3 billion) will be attained under concessional terms.

Domestic Borrowing

Government borrowing from the domestic market for fiscal purposes in FY 2023/24 is projected at UShs 1,585.0 billion compared to UShs 5,007.9 billion in FY 2022/23, which is equivalent to 1% of GDP. This is in line with Government's policy decision to maintain domestic borrowing to no more than 1% of GDP in order to avoid crowding out of the private sector.

Debt Repayments

External debt repayments (amortization) are projected to amount to UShs 2,453.2 billion compared to UShs 2,412 Billion in FY 2022/23. Over the medium term, external debt payments are projected to increase due to the increase in commercial loans over the last few years. Going forward, Government's financing strategy is to reduce borrowing on commercial terms and focus more on concessional borrowing.

Interest Payments

Interest payments are projected to amount to UShs 6,135.5 billion, equivalent to 2.9% of GDP. Of this, UShs 5,227.6 billion is projected for domestic interest payments while the remaining amount equivalent to UShs 907.9 billion will be foreign interest payments and commitment fees. Over the medium term, interest payments are projected to average 2.3% of GDP.

1.4 STATEMENT OF RESOURCE FOR THE ANNUAL BUDGET FY 2023/24

Total resources available for Government expenditure will be obtained from both domestic and external sources. Domestic resources comprise of tax and non-tax revenue as well as borrowing from the domestic market while external resources include budget and project support grants and loans.

1.5 RESOURCE ENVELOPE FOR FY 2023/24

The preliminary resource envelope for FY 2023/2024 is projected at Ushs. 49,988.7 billion, compared to Ushs. 48,130.7 billion for FY 2022/2023 as disaggregated by source in Table 5. This reflects an increase of Ushs. 1,858.0 billion. It should however, be noted that whereas the resource envelope has increased by Shs 1,858.0 billion, the discretionary resource envelope reduced by Shs 2,533 billion due to the projected increase in the interest obligations and obligation to settle Bank of Uganda redemptions.

The total resource envelope of UShs 49,988.7 billion is comprised of domestic revenues equivalent to UShs 28,831.1 billion, budget support amounting to Ushs 2,491.6 billion, domestic borrowing amounting to UShs1,585.0 billion, external project support worth UShs 8,043.6 billion, domestic refinancing (roll-over) of Ushs. 8,798.9 billion, and local revenue for local government (AIA) of Ushs. 238.5 billion (see table 5 below).

Table 5: Projected Resource Envelope for FY 2023/24 UShs. Billion)

S/N	Source	FY 2022/23	FY 2023/24	Variance
1	Domestic Revenues	25,550.8	28,831.1	3,280.2
2	Petroleum Fund	-	-	-
3	Budget Support	2,609.2	2,491.6	(117.6)
4	Domestic Financing (Domestic Borrowing)	5,007.9	1,585.0	(3,422.9)
5	Project Support (External Financing)	6,716.2	8,043.6	1,327.4
6	Domestic Refinancing (Roll-over)	8,008.0	8,798.9	790.9
7	Local Revenue for Local Government	238.5	238.5	-
	**Total Resource Inflows (1+2+3+4+5+6+7)**	**48,130.7**	**49,988.7**	**1,858.0**
8	External Debt Repayments (Amortization)	*(2,412.3)*	*(2,453.2)*	(41.0)
9	Project Support (External Financing)	(6,716.2)	(8,043.6)	(1,327.4)
10	Domestic Refinancing (Roll-over)	(8,008.0)	(8,798.9)	(790.9)
11	Domestic Arrears	(661.90)	(200.00)	461.9
12	Appropriation in Aid (AIA)-Local Revenue	(238.50)	(238.50)	-
13	**GoU MTEF: Resource Envelope Less External Debt Repayments, Project Support, Domestic Refinancing, Arrears and AIA**	**30,093.84**	**30,254.48**	**160.6**
14	Interest Payments	(4,691.9)	(6,135.5)	(1,443.6)
15	Less Domestic Debt Payment (BoU)	-	(1,250.7)	(1,250.7)
16	**GoU Discretionary Resources (MTEF less Interest Payments)**	**25,401.9**	**22,868.3**	**(2,533.7)**

Source: Ministry of Finance, Planning and Economic Development

Government discretionary resources (resource envelope less amortization, domestic debt payment (BOU), interest payments, external project support, domestic refinancing, domestic arrears, and local revenue) is equivalent to UShs. 22,868.3 billion.

1.6 UPDATE ON DEBT SUSTAINABILITY

The stock of public debt increased from US$ 19.54 billion in June 2021 to US$ 20.99 billion in June 2022. As a share of GDP, public debt increased from 46.9% to 48.4% over the same period. This represents an increase of 7.4% compared to 27.45% the previous financial year. This is as a result of Government's deliberate policy of fiscal consolidation aimed

at ensuring that debt remains within the set threshold of 50% of GDP in the medium term. Figure 3 below shows the evolution public debt over the recent years.

Figure 3: Evolution of Public Debt from FY2009/10 – FY2021/22

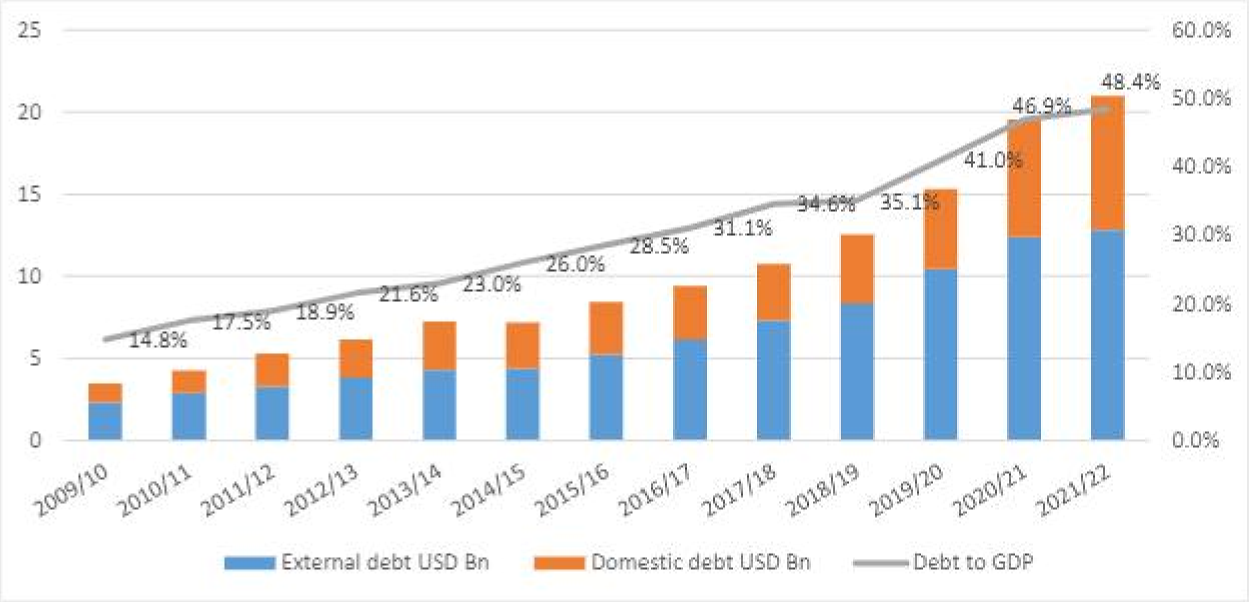

Source: Ministry of Finance, Planning and Economic Development

In line with the fiscal target under the charter for fiscal responsibility, public debt is projected to peak at 53.1 percent in June 2023 and decline to below 50 percent by FY2025/26.

1.7 COMPLIANCE WITH THE CHARTER FOR FISCAL RESPONSIBILITY

The Charter for Fiscal Responsibility (CFR) for the period FY 2021/22 to FY 2025/26 presents Government's strategy for operating a fiscal policy which is consistent with sustainable fiscal balances to maintain the public debt within sustainable levels.

The budget for FY 2023/24 is in line with the CFR. The Charter has 3 fiscal objectives namely: (i) total public debt is reduced to below 50% of GDP by FY 2025/26; (ii) the overall fiscal balance including grants should gradually adjust to a deficit not exceeding 3% of non-oil GDP by FY 2025/26 and (iii) a maximum of oil revenue of 0.8% of the preceding year's estimated non-oil GDP outturn shall be transferred to the consolidated fund for budget operations. The balance shall be transferred to the Petroleum Revenue Investment Reserve (PRIR) for investment in accordance with the PFMA as amended.

The FY 2023/24 projected fiscal deficit as percent of GDP and public debt to GDP ratio are all within the set targets in the Charter for Fiscal Responsibility.

1.8 FISCAL RISK STATEMENT AND MITIGATION MEASURES

The risk framework is categorised into: macroeconomic risks, risks related to public debt and Natural disasters.

a. Macroeconomic Risks

Changes in macroeconomic assumptions create risks to both revenue and expenditure projections as they play a key role in the formulation of the budget. This section assesses two important sources of macroeconomic risks:

i. Uncertain global financial conditions

The global financial tightening in a bid to curb the rising inflation has led to capital flight from developing to developed economies which are considered more risk-free. Therefore, the monetary policy tightening in developed economies, especially in the USA may lead to a significant depreciation of the shilling against the US Dollar which will affect the cost

of external debt servicing and government imports. In addition, it may also lead to a significant increase in domestic interest rates due to the exit of offshore investors from the domestic market to other markets in developed economies which are perceived to be safe.

ii. Estimation of macro indicators (Growth, Revenue performance, budget execution and variation)

Volatility and optimism bias in growth projections can have negative effects on tax revenues and public debt. Although revenue performance since the start of FY 2022/23 has posted surpluses, the slowdown in overall economic growth could hinder the attainment of the revenue target set out in the budget. In addition, the frequent outbreaks of diseases like Ebola could lead to further re-prioritization of resources within programs which might affect budget execution and attainment of set fiscal objectives. These challenges could negatively affect economic growth outturns going forward and in turn affect tax revenues and public debt.

iii. Volatile Commodity Prices (imported inflation)

Uganda is a net importer of petroleum products, therefore an increase in the global crude oil prices leads to imported inflation. Specifically, an increase in crude oil prices directly feeds through to the country's domestic fuel pump prices which leads to a rise in Energy, Fuel and Utilities inflation as well as core inflation. This consequently affects real GDP growth.

b. Risks related to public debt

Risks associated with external and domestic debt, include: -

1) Refinancing risks due to a high composition of short term instruments in the financing mix

2) Materialization of contingent liabilities: Contingent liabilities are payment obligations that only arise if a particular event occurs. Currently, the main contingent liabilities stem from loan guarantees and public corporations' debt.
3) Increased cost of debt especially if we are increasingly borrowing on non-concessional or commercial terms
4) Interest rate risk: Commercial loans are sometimes contracted at a variable interest rate, which is linked to benchmark lending rates such as LIBOR and EURIBOR. Variable rate loans expose future debt service to upward movements in the benchmark lending rates.
5) Foreign exchange rate risk: Borrowing more externally increases Uganda's exposure to foreign exchange rate risk. In the event of sharp and sustained deprecation, external debt service would increase drastically posing a significant impact on sustainability of the debt.

c. Institutional risks

These could stem from the shortages in technical capacity to:

1) Appropriately project revenues which would lead to revenue shortfalls during the implementation of the budget which would cause disruptions and therefore necessitate the need to borrow more to fund expenditure or expenditure revisions such as budget cuts.
2) Failure by institutions responsible for supervision, monitoring and evaluation to identify loopholes during the implementation of Government programs thus leading to cost and time overruns.

d. Natural disasters

Due to climate change, rising deforestation and wetlands degradation, the country is increasingly becoming more susceptible

to adverse weather conditions and natural disasters such as droughts, floods, landslides, crop and livestock epidemics and earthquakes, among others. These disasters pose a risk for economic growth and social welfare and can have significant consequences on the Budget in case unplanned or emergency funding is required. Results from the macro-fiscal model (UGAMOD) which analyses the interlinkage between the macroeconomic and climate variables shows that as temperatures continue to rise, there are significant losses to GDP growth particularly in the agriculture sector, capital stock & consumption and can result in high debt levels. Despite the PFM Act 2015 providing for a contingencies fund to cater for such unforeseen occurrences, these could be of greater magnitude than the provision, hence posing a fiscal risk.

e. Insecurity

Security threats both at home and in the region which may destabilise economic activity hence affecting trade. Government will therefore continue to support the security agencies to counter the threats and maintain peace in the country.

Effective analysis and management of fiscal risks is a crucial component of sound public financial management, and Uganda intends to build on the progress it has registered in this respect in recent years. While several external risks to fiscal plans are not within our control, the government is expanding its assessment and monitoring of fiscal risks and putting mitigating measures in place to reduce their impact on the public finances. Some of the mitigation measures are listed below.

1. Regarding the technical capacity in institutions, Government continues to strengthen the technical capacity of institutions so that they can efficiently supervise and implement government programmes.
2. Government remains committed to encourage concessional borrowing in order to reduce the cost of debt. It also continues to improve its financing mix so as to reduce the refinancing risks.
3. Regarding contingent liabilities government maintains a proactive policy stance to mitigate contingent liability risks by ensuring that;
   (a) All borrowing by public corporations and sub-national governments, and government-issued guarantees, are approved by the Minister of Finance.
   (b) All public corporations as well as entities requesting guarantees, are required to be financially sound, as determined by MoFPED.
   (c) All projects to be funded must be in line with the National Development Plan and sector priorities.
   (d) Only economically and financially viable projects are approved for financing and investment, the Ministry of Finance adopted a Public Investment Management System for review and approval of projects. The Framework emphasises gradualism in project development and approval process i.e., it introduces four stages of mandatory approval at concept, profile, prefeasibility and feasibility stage.

1.9 BUDGET STRATEGY FY 2023/24

Context

The Economic Strategy in the short to medium term has the twin objectives of restoring economic activity to pre-pandemic levels and subsequently accelerating the pace of Socioeconomic Transformation. Economic recovery will be achieved by boosting aggregate demand by restoring domestic consumption, renewing private and public investment, and enhancing export promotion.

The current fiscal year 2022/23 has focused on supporting and sustaining economic recovery by mitigating and strengthening the resilience of economy against the adverse impacts of the various shocks. These shocks include:

i. Covid-19 pandemic which disrupted supply networks, low demand for exports, Foreign Direct Investments (FDI) and Remittances have been negatively affected. However, the pandemic also reinforced the need to boost domestic production using locally available raw materials for exports and for import replacement;

ii. Increase in commodity prices leading to imported inflation particularly for fuel, raw materials, other essential commodities, etc;

iii. Increase in interest rates, which has increased the cost of borrowing, and reduced access to global capital due to the reversal of capital flow to advanced economies;

iv. Capital flight from developing to developed economies which are considered risk free.

v. Climate Change Effects which have resulted in a gradual withdrawal of financing to non-renewable energy projects.

The strong and timely interventions of the Government in dealing with the various shocks has allowed economic activities to continue to expand, albeit at a slow pace. Other performance indicators of the economy remain positive although currently under strain.

Objective of the Budget Strategy for FY 2023/24

The overall objective of the FY 2023/24 Budget Strategy is to restore the economy back to the medium-term growth path of 6-7 percent per annum and improve competitiveness of the economy. In the medium-term, increasing the wealth of households and eliminating poverty, particularly using the Parish Development Model and small and medium enterprises economic recovery programs is key for socioeconomic transformation. In addition, diversifying the economy and Uganda's exports, are key to achieving the planned economic growth trajectory.

The Budget Strategy has taken into consideration the key findings of the NDP III mid term review which include:

i. Lower than expected economic growth in the first two years of NDP i.e 4% below the planned target of 5.2%;
ii. The need to achieve middle income status;
iii. An expansionary monetary policy, resulting from unforeseen additional spending by Government to mitigate the effects of the COVID-19 pandemic which has led to an increase in the fiscal deficit from 7.8% to 9.1% of GDP;
iv. Lower allocations to programs than envisaged in the NDP III, which has impacted on the ability of programs to meet set targets.
v. Failure to complete any of the 69 core projects in the NDP 111.

Population

In addition Uganda's population has sharply grown from 15 million in 1986 to 43.7 million people today with a growth rate of 3.2 % and a life expectancy of 64.7 years (UBOS 2022). This is on account of provision of improved access to quality social services, peace and security, among others. The rising population however is exerting pressure on Government's capacity to provide the required quality social services for all as well as employment opportunities. Therefore, we need policy action to sustain the quality and scope of services to ensure an economically active and productive population.

Employment, Incomes, Poverty, and Inequality

Under the Third National Development Plan, Government targeted to create 2.5 million jobs over five years, with an estimated annual average of about 512,000 jobs. Most of these jobs were expected from the services sector (1.313 million).

Agriculture was expected to be the second-highest contributor to jobs (796,411 jobs), followed by industry, whose largest share of jobs was expected to arise primarily from manufacturing. While there was a loss of jobs in the year 2020/21 there was job recovery the following year with jobs increasing from 258,286 in FY2020/21 to 345,039 in FY 2021/22. More is expected to be achieved with Government interventions to revitalise the economy, especially with promotion of enterprise development along value chains that attract high labour demand and help private business to grow and thrive.

The private sector contributes about 83 percent of the total formal sector jobs, currently estimated at 2.3 million, and producing approximately 80% to Uganda's GDP. Private sector employment is concentrated in agriculture (including forestry, livestock and fishing, accounting for about 69.4 percent, manufacturing at 5.6 percent, trade and repairs at 8.9

percent, hotels and restaurants at 2.0 percent, transport and communication at 2.3 percent, construction at 2.0 percent as well as education at 3.0 percent).

Other critical sectors where recorded employment opportunities exist include mining and quarrying, utilities, posts and telecommunications, financial intermediation, insurance, business services, health and social works, and community and personal services. These sectors employ approximately 1.9 million people, representing 12% of the working population. The budget strategy therefore provides focused actions to ensure these sectors continue to grow to sustain their job creation capacity.

Overall, Uganda's income per capita increased from USD 936 in FY2020/2021 to USD 1,052 in FY2021/22 in real terms. This improvement was partly attributed to increased Government-targeted expenditure to support economic recovery to offset the impact of shocks such as COVID-19 as well as private sector investments.

However, poverty and inequality remain a critical development challenge for the country. Whereas poverty decreased marginally to 20.3 % in FY2019/20 from 21.4 % in FY2016/17 (UNHS, 2019/2020, the incidence in income poverty increased during the COVID-19 from 19 % pre-pandemic to 22 % during the pandemic. The effective implementation of the Parish Development Model as well as other Government affirmative action interventions, are expected to narrow the gap in the short and medium term

Over the remaining period of NDPIII, Government shall restore the balance between infrastructure and human capital development to spur economic growth and development. Government shall sustain economic recovery and build economic and enterprise resilience by focusing on six

strategic intervention areas which will take priority next financial year, namely:

i. *Peace and security under the Governance and Security Programme;*
ii. *Roads (Maintenance of both tarmac and marram roads) under the Integrated Transport Programme;*
iii. *Electricity (Construction of Sub Stations and Transmission lines and Ayago Hydro power station in the medium term) under the Sustainable Energy Development Programme;*
iv. *Railway (Development of SGR and rehabilitation of the Meter Gauge Railway) under the Integrated Transport Programme;*
v. *Irrigation particularly the small scale solar powered irrigation under Agro Industrialisation Programme;*
vi. *Industrial Parks through building infrastructure and connecting them to electricity under the Manufacturing Programme;*
vii. *Support to medical schools and science-based research and development under the Human Capital Development Programme;*
viii. *Full implementation of PDM and scaling up EMYOOGA under the Private Sector Development Programme;*
ix. *Oil and Gas development; and*
x. *Enhancing support to UDB and UDC under Private Sector Development.*

The above have been identified as priorities by His Excellency the President over and above other key investments in fundamentals such as Human Capital Development (Health, Education and Water for Human Consumption) among others. Therefore, Government expenditures will have to be constrained to fit within the existing resources for Financial Year 2023/24 including statutory obligations

Human Capital Development

Human Capital Development comprises three sub-programs and these include: Health, Education and Water for human consumption and the budget intervention areas will be as follows:

Health

The Health Sub-program aims at migrating the population from a predominantly curative health care system to a preventive healthcare promoting system. This will involve the following interventions:

i. Addressing staffing gaps at all levels of service delivery (HCIIIs, HCIVs and the regional hospitals)
ii. Timely supplies of drugs to address stock-outs as well as provision of relevant medical equipment
iii. Operationalizing Mulago Super Specialized Hospital and all existing HCIVs and HCIIIs.
iv. Piloting the Community Health Extension Workers (CHEWs) strategy to create a paradigm shift from a predominantly curative disease-oriented health care system to a preventive health care promoting health system.
v. Strengthening the Regional Technical Supervisory structures.

Education

The Education Sub-Program will meanwhile focus on the following areas which are guiding the planning and budgeting process for FY 2023/2024:

i. Development of a complete National Vocational Qualifications Framework
ii. Promotion of Science, Technology, Engineering, and Mathematics (STEM/STEI) in Education, Research, and incubation to transform it into goods and services for national growth and societal well-being

iii. Enhancing the capacity of the Skilling institutions to cope with the growing demands of the world of work
iv. Improved data management and evidence-based planning an upgrade of EMIS to include tracking enrolment, drop-out, and retention, and uniquely identifying learners, teachers, and institutions
v. Increased uptake of the digitalization strategy in the programme through roll-out and maintenance of systems such as Teacher Effectiveness and Learners' Achievements system (TELA), E-inspection, Teacher Management Information System (TMIS), and E-learning
vi. Improving the overall staffing level in the Education sub-program
vii. Infrastructure development and equipment of medical schools to meet international accreditation

Water

This sub-program will focus on improving access to safe and clean water for human consumption as well as sanitation in both rural and urban areas through:

i. Construction and expansion of large, medium and small piped water systems in both urban and rural growth centers
ii. Rehabilitation and maintenance of piped water systems,
iii. Strengthening and integration of the water user committees,
iv. Construction of public and community sanitation facilities
v. Promotion of motorized water supply systems through construction of solar-powered schemes.

Governance and Security

Government is mindful of improving security and good governance is instrumental for economic prosperity. Next financial year, Government will aim at the following interventions:

Security

i. Prioritise strengthening of security and intelligence capability systems to protect persons and property as well as maintaining peace.
ii. Enhancing performance of security personnel (Uganda Peoples Defence Forces, Uganda Police and Uganda Prisons Services);
iii. Ensuring availability of the required military infrastructure.
iv. Addressing the welfare of personnel in uniform (UPDF, Uganda Police Force, Uganda Prisons Service);
v. Salary enhancement and harmonization across the public service including security personnel;

Governance:

This sub-program will focus on interventions that will:

i. Finalize and lead to the implementation of the Uganda National Action Plan on Human Rights;
ii. Enhance the demand for accountability and value for money;
iii. Fast track and monitor the implementation of government programmes;
iv. Undertake the National civic education programme;
v. Strengthen business processes and case management systems in institutions
vi. Improve on monitoring and evaluation of Government of Uganda development projects
vii. Support efforts on the fight against corruption.
viii. Restructuring of Government.

Integrated Transport

Under this Program, Government will invest on Railway Development/Rehabilitation and Road Maintenance as follows:

Railway Development and Rehabilitation

Government aims at starting the construction of the Standard Gauge Railway (SGR). Coupled with the SGR is finalizing the rehabilitation of the Meter Gauge Railway on the following lines:

i. The Malaba – Gulu line,
ii. Completion of the Gulu – Pakwach line,
iii. The Malaba – Kampala line, and the
iv. Kampala - Kasese line

Road Maintenance and Transport Infrastructure

Road infrastructure development is key in enhancing economic growth and building competitiveness. To further develop Transport Infrastructure, key interventions next year will include:

i. Maintenance of existing road networks;
ii. Operationalising the Hoima International Airport;
iii. Continued upgrading of selected strategic roads from gravel to bituminous surface;
iv. The maintenance of national, districts and community roads; and the rehabilitation and upgrade of aerodromes, and support to the aviation school.

Water Transport

Development/Construction of ferries will be undertaken and:

i. Conduct a hydrographic survey and produce navigation charts of Lakes Victoria, Kyoga, Bunyonyi, Albert, Bisina, Edward, George, R. Nile;

ii. Completion of Bukungu-Kagwara-Kaberamaido (BKK) Ferries and their landing sites
iii. Two ferries for Lake Bunyonyi and their landing sites
iv. Procurement and delivery of rescue boats

Private Sector Development

Growth of an economy depends on the strength and buoyancy of its private sector to create jobs and increase incomes as well as improved living standards. In FY 2023/24, Government will promote:

i. Efforts to lower production and trade costs to promote competitive production of quality tradable goods.
ii. Access to affordable capital through Uganda Development Bank (UDB) and the Uganda Development Cooperation (UDC).
iii. Enterprise groups, Small and Medium-Scale Enterprises (SMEs) shall be financed through EMYOOGA, the Parish Development Model and related Government schemes.
iv. Access to regional and global markets as a key element in strategically building resilience of the economy.
v. Increasing Private Sector investments in key growth areas; and achieving value-added growth in exports.

Agro-Industrialization

Under this Program, Government will focus on Climate Change, Food Security Management and Irrigation. This will also be enhanced by the Parish Development Model. Therefore, resources will be directed towards the following areas:

Food Security Management

To boost food production and productivity for households and large scale farms, Government will redirect resources towards:

i. Provision of improved quality of inputs as well as post-harvest handling and storage of agricultural products;
ii. Enhanced production, storage, agro-processing and value addition;
iii. Increased market access;
iv. Supporting competitiveness of agricultural enterprise groups while guaranteeing quality agricultural products for the domestic, regional, and global market destinations.
v. The Parish Development Model, agricultural insurance and related grants, to pursue increased mobilization, access, and utilization of Agricultural Financing.
vi. Agricultural research through the National Agricultural Research Organization (NARO), National Animal Genetics and Resources Centre and Data Bank (NAGRC&DB) and the academia to target mainly the development of climate resilient crops and animal species.
vii. Diseases and Pest Control will also be prioritized in the budget of agriculture to support production and productivity.
viii. Large-scale mechanization.

Irrigation

To boost production and productivity through irrigation and help address climate change among others, emphasis will be put on:

i. Increasing the volume of and access to water available for both agricultural and industrial production.
ii. Expanding investments that will increase productive arable land in areas through small-scale solar-powered and medium-term irrigation schemes to improve production and productivity for small-scale producers; industry and increased exports.

iii. Mobilization and support of farmer groups to increase uptake of the available irrigation infrastructure.
iv. Construction of both community and private water infrastructure such as valley tanks and dams at the Parish level in line with the PDM framework.

The Parish Development Model (PDM)

Government shall deepen implementation of the Parish Development Model (PDM), beyond the Financial Inclusion pillar. Government shall maintain disbursement of UShs. 100M per parish supported by data collection and management, as well as enterprise development and fast tracking of interventions for efficient value chain development.

Through the implementation of this model, government will ensure that SACCOS, EMYOGA, and other investment groups are coordinated in order to strengthen their linkage with business development services in a sustainable way.

Sustainable Energy Development (Electricity)

Government interventions for efficient energy shall focus on electricity generation, transmission, distribution and investment in renewable energy. Specific undertakings will include:

i. Prioritizing rural electrification to the remaining sub-counties;
ii. Electricity connections for upcoming industrial zones and factories in different parts of the country;
iii. Construction of additional power sub-stations to boost and regulate the generation, transmission and distribution;
iv. Empower the Uganda Electricity Generation Company Limited (UEGCL) and Uganda Electricity Distribution Company Limited

(UEDCL) to manage the generation and distribution of power respectively throughout the Country.

In this regard, Government has commenced on the buy-out of ESKOM concession at the Kira/Nalubaale Power Station, and on the same note, the existing UMEME concession for distribution of power is scheduled to expire by the year 2025. Government shall, therefore, ensure availability of funds for investments under UEGCL and UEDCL to seamlessly fill the gap that shall be caused by the exit of UMEME upon expiry of the existing concession in 2025.

Development Plan Implementation and Regional Development

Under these two programs, plans that reflect specific local advantages that need to be harnessed for local economic development will be designed and implemented to guide regional development as follows:

i. Growth Corridors covering the Gulu-Hoima- Kasese and the Arua-Gulu-Soroti- Malaba axes will be designed and developed to complete nationwide industrial development, in addition to the Malaba-Kampala- Mbarara Corridor.
ii. Transport and Power infrastructure will be extended to key growth areas, including tourism sites, to facilitate production and value addition.
iii. Fast-tracking the establishment of e-mobility (electric vehicle) charging infrastructure in readiness for energy transition from fossil energy to renewable energy and support the electric motorcycle and motor vehicles industry.

Administration of Justice and Rule of Law

For timely administration and access to justice by all, Government will:

i. Continue to further develop appropriate infrastructure for Administration of Justice. Special focus will be on construction and equipping of courts and additional Justice Centres.
ii. Strengthen human resource through recruitment of additional Judicial Officers and relevant non-judicial Officers to further extend Judicial services nearer to the people.
iii. Strengthen Courts to resolve disputes in special areas including land, Commercial, Family disputes, Environment, Standards, Utilities and Tax disputes
iv. Enhance support to the Office of the Directorate of Public Prosecutions
v. Address welfare, housing and security concerns of judicial officers
vi. Automation of business processing across the Judiciary to improve service delivery
vii. Advance the use of ICT and other technologies. Focus will be on rolling out Electronic Court Case Management Information System (ECCMIS) to additional Courts, procurement and installation of Court Recording and Transcription Systems, and Video Conferencing Systems to enhance capacity of Courts to handle cases online;
viii. Promote Alternative Dispute Resolutions (ADR) and other case management interventions, such as Plea-Bargaining, Small Claims Procedure; and Mediation.
ix. Operationalise more High Court Circuits and strengthen the Inspectorate functions in the Administration of Justice System;
x. Strengthen prevention, detection/investigation and adjudication of corruption.

Mineral Development

In FY 2022/2023, the key focus area will seek to invest in mineral exploration, mineral data analysis, value addition and revenue harnessing from mineral activities through;

i. Finalization of exploration activities for geothermal and other priority minerals including iron and steel target areas,
ii. Investing in seismology activities for earthquake monitoring to provide early warning information,
iii. Licensing of proven mineral deposits and
iv. Establishment of mineral regulation infrastructure including weighbridges to increase Non-Tax Revenue and data collection.
v. Maintaining existing investments in exploration and quantification for steel and minerals in Karamoja, as well as incentives for import replacement of iron and steel.
vi. Support to UDC for processing of Uganda iron ore.

Sustainable Development of Petroleum Resources (Oil and Gas)

1.8.1 The key area of focus for FY 2023/2024 will be on harnessing the petroleum potentials holistically for medium to long term benefits through:

i. Fast tracking readiness activities for the construction of the East African Crude Oil Pipeline (EACOP) and the National oil refinery. Government will complete acquisition of the right of way for the refined products pipeline, negotiations with potential investors and identification suitable funding options for the refinery and associated infrastructure.
ii. Promoting the use of alternative clean sources of energy for cooking including Liquified Petroleum Gas (LPG) targeting all cities, municipalities and other urban settings, and energy saving

cookstoves for rural areas. This will be done through provision of free start up kits for LPG and cook stoves.

iii. Continued preparatory work for the petrochemical industry (Kabaale Petro-based Industrial Park).
iv. Capitalization of UNOC

Tourism Development

To sustain further growth of Tourism, the strategy is to focus on the following areas:

i. Rolling out the global destination brand as well as domestic marketing promotions for Uganda to increase competitiveness, attractiveness and identity as the **"Pearl of Africa"**.
ii. Enhance digital destination marketing through social media marketing, mobile marketing, online public relations, content marketing, virtual reality, video and affiliate marketing collaboration with the tourism and travel trade partners should be enhanced.
iii. Positioning Uganda as a global and regional centre for Meetings, Incentives, Conference and Exhibitions (MICE)
iv. Strengthen enforcement and adherence to tourism standards through Licensing, grading and classification of tourism facilities.

Climate Change, Natural Resources, Environment and Water Management

To safeguard the environment as well address the increasing effects of climate change, Government shall prioritize:

i. Fast-tracking the implementation of the National Climate Policy,

ii. Land use planning
iii. Environmental conservation, restoration and protection of degraded water catchment areas and forest cover
iv. Promotion of ecosystem-based approach to support sustainable development and utilization of water resources of ecological zones.

Digital Transformation

In support of Digital Transformation, broadband connectivity will be extended to the parish level and last mile connectivity to expand access to affordable high-speed internet through the National Backbone Infrastructure will be implemented. In addition, development and commercialization of local ICT products and establish and support regional ICT incubation hubs will be supported, and communities and SMEs will be trained to enhance digital literacy.

Manufacturing

To support Manufacturing and Industrialisation Programs, Government shall fast-track implementation of the industrialization policy through increased investment and growth of the manufacturing sector as a vehicle to increase local production and job creation for better or quality employment opportunities. Equally, local industries will be supported to acquire appropriate technologies and product certification as well as access to cheap and affordable electricity. Government will also support efforts to set up a buffer stock for textile manufacturers.

Sustainable Land Use, Housing and Urbanization

Under this Program, resources will be allocated towards:

i. Completion of the ongoing rehabilitation and development of key urban infrastructure in all major cities and municipalities. This includes

urban roads, street lighting, drainage systems, markets and public parks, among others.

ii. Further strengthen the land administration system through registration and titling to ensure timely provision of land required for various development purposes.
iii. Support housing development through the dissemination of free prototype housing plans.
iv. Improving physical planning and development through designation and gazetting of infrastructure corridors as well as development and enforcement of Physical Development Plans for all cities, municipalities, town councils, rural growth centres and rural settings.
v. Development of decent housing facilities starting with institutions such as Uganda Police, Uganda People's Defense Forces, schools and health facilities.

Community Mobilization and Mind-Set Change

In order to enhance social protection over the entire human life cycle of all Ugandans, Government will undertake the following:

i. Continue with the implementation of the Parish Development Model while targeting enterprises that will foster income generation and improved living standards for all
ii. Continue to support provision of social services for all, e.g., primary health care, universal primary and secondary education etc.
iii. Enhancing support for inclusive businesses recovery and growth;
iv. Concessional financing to enhance support for the youth, women, PWDs, the elderly, etc;
v. Recovery of UWEP and YLP funds from the beneficiaries to sustain and make affordable and accessible the availability of the revolving funds;
vi. Regulation of the externalization of labour through the Ministry of Gender, Labour and Social Development;

vii. Strengthening occupational safety and health.

Public Sector Transformation:

Government will under this Program carry out the following:

i. Restructure the current government functional architecture to support the delivery results under the Programme approach;
ii. Reconfigure and Rollout the Human Resource Management System in support of the Programme approach;
iii. Update the Teacher Licensing & E-Certification System;
iv. Rollout e-recruitment system to District Service Commissions (DSCs); and
v. Implement the Local revenue enhancement management plans.

Innovation, Technology Development and Transfer will continue to be implemented as planned, with resources already earmarked for promotion of science and research.

Legislation, Oversight and Representation

The Legislature Program will focus on the following intervention areas in line with NDPIII:

i. Timely enactment of legislation to support implementation of NDP III interventions and other government policies and programme to properly address the needs of the people of Uganda.
ii. Strengthening the oversight role by building capacity in PFM reforms for improved service delivery.
iii. Improve transparency and compliance with accountability rules and regulations especially on parliamentary recommendations.

iv. Strengthen stakeholder engagement and participation in the legislative process to ease implementation of enacted legislations.

1.9 POLICY PROPOSALS FOR FY 2023/24 BUDGET

Implementation of the Parish Development Model

Effective implementation of all the Pillars of the Parish Development Model (PDM), beyond the Financial Inclusion pillar is a key component of the Budget Strategy for FY 2023/24

Public Investment Management.

The percentage of projects that are underpinned by a cost-benefit analysis out of the total number of projects entering the Public Investment Plan (PIP), although still low, has improved from 10% by FY 2015/16, to 37% for FY 21/22.

The impact of the Government's recent reforms are visible, but are being overshadowed by challenges in critical areas including; low execution rates on donor and own-budget projects; lengthy implementation delays; cost and time overruns on projects; high commitment fees in case of externally funded projects; shortened life span of projects due to poor operation and maintenance of created physical assets; low capacity of some MDAs and, continued non-compliance of many projects to the guidelines.

The latest World Bank economic analysis for Uganda (2022) says deepened, accelerated reforms for public project management will support a strong economy over the long-term. MoFPED has also resolved not to permit any unstudied project to access the budget.

Revenue Collection and Management of Government Expenditure

In FY 2023/2024, domestic revenues are projected to amount to **UShs. 28.831 Trillion**. This translates into nominal growth in revenues of **UShs. 2.580 Trillion (equivalent to 12.8%)** up from **UShs. 25.550.69 Trillion** projected revenues for FY 2022/2023. This increase is attributed to gains on account of growth in the economy, and revenue gains from revenue enhancement measures after finalizing both tax policy and tax administration.

Government will step up efforts to implement the Domestic Revenue Mobilisation Strategy (DRMS) including; closing all gaps leading to revenue losses such as under declarations, falsification of documents, review of exemptions to put in place clear criteria for beneficiaries, sunset clauses to define cut off points, and carryout a cost-benefit analysis to ensure that the beneficiaries deliver their promised deliverables through effective monitoring of the exemptions.

The Uganda Revenue Authority (URA) is also going to step up taxpayer education, door-to-door field visits, identification of unregistered taxpayers and enrol them, effective administration of VAT, income tax, customs, and rental tax.

All these and other efforts will widen the tax base and improve our tax collection effort without introducing new taxes and tax rates that tend to fall on the same group.

Therefore, Government will pursue enhanced tax policy administration to improve domestic revenue to finance the national policy commitments, considering the rising public debt and its implications.

Restructuring and Rationalization of Government Agencies (and implication on public administration)

Cabinet approved the report on Rationalization of Government Agencies and Public Expenditure (RAPEX) to improve on service delivery, minimize duplication as well as save on public expenditures. Government, guided by the Ministry of Public Service, will fast track the implementation of the roadmap, which is already in progress.

Implementation of the program approach to planning and budgeting

Following the NDPIII Mid-Term Review, the Budget is fully aligned to the Program Based Budgeting approach and respective Vote interventions will be implemented in line with the Program Implementation Action Plans (PIAPs). It is against this that all Indicative Planning Figures and expenditure ceilings have been be issued.

Regional Integration

Expansion of the EAC bloc to include the Democratic Republic of Congo in March 2022 has opened greater cross-border investment opportunities for Uganda.

With the Africa Continental Free Trade Area (AfCFTA) now operational, it is expected to boost private sector potentials for promoting regional growth and competitiveness with other economic blocks such as the South African Development Cooperation (SADC), Economic Organization for West African States (ECOWAS) and the Maghreb region for North Africa.

However, the main challenges that remain yet unaddressed include the monetary union of the EAC bloc and non-tariff barriers such as Customs and administrative entry procedures, technical barriers to trade, sanitary and phyto-sanitary measures, charges on imports, transport, clearing and

forwarding. This will be one of the prime areas of discussion with my Colleague Finance Ministers of the EAC in April 2023.

Program Allocations for FY 2023/2024

Following the NDPIII Mid-Term Review and overall reprioritization of the Budget to inform resource allocations for FY 2023/2024, the Program Ceilings were issued based on the following criteria:

i. Ceilings for wages and wage related costs such as salary enhancement;
ii. Fixed Costs to cater for the minimum required routine operations for Program Votes;
iii. On-going NDPIII flagship projects and other priorities under multi-year commitments;
iv. NDPIII priorities under the Policy Commitments such as the Standard Gauge Railway, Meter Gauge Railway and Road Maintenance;
v. Discretionary resources to finance other NDPIII priorities;
vi. Protected Votes;
vii. Projects exiting the PIP without further extension;
viii. Merging of some Government Institutions including abolition of some Subventions; and
ix. Other Government Transfers (OGTs) to the Local Governments.

Part 2: Details of Proposed Program Plans and Expenditure

This section provides details of proposed programme plans and expenditures for the 20 Programmes defined in the NDP III Every Programme summary comprises of five subsections; P1 – P5.

i. P1 provides an overview of Programme Expenditures and defines the programme strategy and linkage to the NDP III.

ii. P2 highlights the projected programme performance. For each Programme outcome, it sets out outcome indicators and projections and further details the projections for the Sub Programme Intermediate outcomes and indicators.

iii. P3 defines the Medium-Term Budget allocations by Sub Programme and Vote.

iv. P4 provides information on the Programme priorities in line with the interventions for FY 2023/24.

v. P5 narrates the gender and equity issues and interventions for FY 2023/24.

AGRO-INDUSTRIALIZATION

Foreword

Within the framework of the Agro-Industrialization Program (2021 to 2025), the Agro-Industrialization Programme aims to increase commercialization and competitiveness of agricultural production and agro processing, in order to increase household incomes, improve food security and the quality of life for all Ugandans as envisaged in the National Development Plan (NDPIII) and the country's path to Vision 2040.

In response to the NDPIII targets and the Parish Development Model Framework (Pillar 1: Production, Storage, Processing & Value Addition), MAAIF through a detailed consultative process developed the Agricultural Value Chain Development Strategy (AVCDS) as its tool for planning and implementation of interventions across the whole value chain. For agro-industrialisation to take root, and have more agro-based industries; supplied with adequate and quality raw materials, agricultural planning and practice of the various commodities is following the value chain approach.

The AVCDS presents a harmonised position that the Agricultural Sector and other actors in the Agro-industrialisation Programme of NDP III, seek to pursue in this five-year term of Government. It captures, re-states and emphasizes WHAT needs to be done at the various components of the Agricultural value chains. It also defines the relevant strategies (HOW) to be pursued by all actors in the Agro Industrialisation Programme of the NDP III in this term of Government. After defining the WHAT to do, the HOW to do it, it further states the WHO to do the what (various programme actors) in a coordinated and collaborative manner.

His Excellency the President also guided the Ministry of Agriculture, Animal Industry and Fisheries on issues of focus and Strategy for the Development of the Agriculture Sector. These include: Production, multiplication, certification and distribution of seed and stocking material; Pest and Disease Control; Mechanization and Irrigation; Farmer Mobilization and Education; Partnerships with big commercial farmers for the production of strategic commodities to meet national and international demand; and Special intervention/affirmative action for the fisheries sub-sector and aquaculture development.

I therefore hope that the interventions planned under the FY 2023/24 will guide the implementation of the Agro-industrialization as well as the Parish Development Model (PDM).

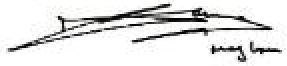

Maj. Gen. David Kasura-Kyomukama

Permanent Secretary

AGRO-INDUSTRIALIZATION

Abbrevations and Acronyms

ACRONYM	ACRONYM NAME
AGI	Agro-Industrialisation
DDA	Dairy Development Authority
NARO	National Agricultural Research Organisation

AGRO-INDUSTRIALIZATION

P1: PROGRAMME OVERVIEW

Snapshot of Medium Term Budget Allocations

Table P1.1 Overview of Programme Expenditure and Medium Term Allocations (Ush Billion)

Billion Uganda Shillings		FY2022/23		FY2023/24	MTEF Budget Projections			
		Approved Budget	Spent by End Sep	Proposed Budget	2024/25	2025/26	2026/27	2027/28
Recurrent	Wage	203.824	55.528	205.716	216.002	237.602	261.362	261.362
	Non Wage	240.606	22.257	160.086	163.287	195.945	264.526	264.526
Devt.	GoU	459.940	22.445	328.560	328.560	394.272	551.981	551.981
	ExtFin	545.440	77.052	804.967	777.348	482.582	302.882	302.955
	**GoU Total**	**904.370**	**100.229**	**694.362**	**707.849**	**827.819**	**1,077.869**	**1,077.869**
	**Total GoU+Ext Fin (MTEF)**	**1,449.811**	**177.282**	**1,499.329**	**1,485.197**	**1,310.401**	**1,380.750**	**1,380.824**
	A.I.A	0.000	0.000	0.000	0.000	0.000	0.000	0.000
	**Grand Total**	**1,449.811**	**177.282**	**1,499.329**	**1,485.197**	**1,310.401**	**1,380.750**	**1,380.824**

Programme Strategy and linkage to the National Development Plan

The design of the third National Development Plan (NDPIII) focuses on programs as the guiding framework for the implementation of the priority interventions. The NDP III consists of 15 Programmes and these integrate the efforts of the various sectors. The Agro-industrialization (AGI) program is the primary program to which the agricultural sector contributes. However, the agricultural sector also contributes to and also derives support from other programs.

The National Development Plan III seeks to consolidate and build on the investment in production and productivity, through promoting Agro industrialization as one of its major programmes. The goal of the programme is to increase household incomes from Agro-industry, mainly through increasing commercialization and competitiveness of agricultural production and Agro-processing.

The AGI program strategic objective include;
Increasing agricultural production and productivity;
Improving post-harvest handling and storage of agricultural products;
Increasing agro-processing and value addition;
Increasing market access and competitiveness of agricultural products in domestic and international markets;
Increasing the mobilization, access, and utilization of agricultural finance; and
Strengthening agriculture sector institutional capacities for agro-industrialization.

P2: Highlights Of Programme Projected Performance

Table P2.1 Programme Outcomes Indicators

AGRO-INDUSTRIALIZATION

**Programme Outcome**	Improved post-harvest management					
**Programme Objectives contributed to by the Intermediate Outcome**						
Improve post-harvest handling and storage						
			**Performance Targets**			
**Programme Outcome Indicators**	**Base Year**	**Base Line**	**2023/24**	**2024/25**	**2025/26**	**2026/27**
Post-harvest losses for priority commodities (%)	2017/18	37%	18%	15%	13%	12%
**Programme Outcome**	Increased storage capacity					
**Programme Objectives contributed to by the Intermediate Outcome**						
Improve post-harvest handling and storage						
			**Performance Targets**			
**Programme Outcome Indicators**	**Base Year**	**Base Line**	**2023/24**	**2024/25**	**2025/26**	**2026/27**
Storage capacity (MT)	2017/18	550000	1350000	1500000	1650000	1900000
**Programme Outcome**	Increased processed agricultural products					
**Programme Objectives contributed to by the Intermediate Outcome**						
Increase agro-processing and value addition						
			**Performance Targets**			
**Programme Outcome Indicators**	**Base Year**	**Base Line**	**2023/24**	**2024/25**	**2025/26**	**2026/27**
Agriculture Value added in current prices (billion Ugx)	2017/18	27993	36650	39640	42640	45,640
Export value of priority agricultural commodities (USD Billion)	2017/18	1.65	2.2	2.35	2.5	2.75
Manufacturing value added as a proportion of GDP	2017/18	8.3	9.6	10.0	10.4	10.6
**Programme Outcome**	Increased agricultural exports					
**Programme Objectives contributed to by the Intermediate Outcome**						
Increase market access and competitiveness of agricultural products in domestic and international markets						
			**Performance Targets**			
**Programme Outcome Indicators**	**Base Year**	**Base Line**	**2023/24**	**2024/25**	**2025/26**	**2026/27**
Share of agricultural exports to total exports (%)	2017/18	26%	33%	35%	37%	38%
**Programme Outcome**	Improved quality and standards of agricultural products					
**Programme Objectives contributed to by the Intermediate Outcome**						
Increase market access and competitiveness of agricultural products in domestic and international markets						
			**Performance Targets**			
**Programme Outcome Indicators**	**Base Year**	**Base Line**	**2023/24**	**2024/25**	**2025/26**	**2026/27**
Value of agricultural imports ($ billion)	2017/18	1.2	1.0	0.95	0.85	0.7

AGRO-INDUSTRIALIZATION

**Programme Outcome**	Increased production volumes of agro-enterprises					
**Programme Objectives contributed to by the Intermediate Outcome**						
Increase production and productivity						
			**Performance Targets**			
**Programme Outcome Indicators**	**Base Year**	**Base Line**	**2023/24**	**2024/25**	**2025/26**	**2026/27**
% change in production volumes in priority agricultural commodities	2017/18	10%	30.4%	31.2%	32.4%	34%
% change in yield of priority agricultural commodities	2017/18	0	15.4%	17.2%	19.6%	22.8%
Agricultural Real GDP growth rate (%)	2017/18	3.8%	5.0%	5.1%	5.2%	5.4%
Export value of priority agricultural commodities (USD Billion) - Coffee	2017/18	0.492	0.951	1.087	1.225	1.357
Export value of priority agricultural commodities (USD Billion) - Diary	2017/18	0.077	0.11	0.126	0.144	0.171
Export value of priority agricultural commodities (USD Billion) - Fish	2017/18	0.146	0.184	0.21	0.256	0.326
Export value of priority agricultural commodities (USD Billion) - Maize	2017/18	0.125	0.1	0.105	0.11	0.125
Export value of priority agricultural commodities (USD Billion) - Meat	2017/18	0.003	0.004	0.005	0.006	0.007
Export value of priority agricultural commodities (USD Billion) - Tea	2017/18	0.091	0.094	0.108	0.143	0.188
Export value of priority agricultural commodities (USD Billion) - Total	2017/18	0.935	1.4	1.6	1.8	2.0
**Programme Outcome**	Increased Water for Production Storage and utilization					
**Programme Objectives contributed to by the Intermediate Outcome**						
Increase production and productivity						
			**Performance Targets**			
**Programme Outcome Indicators**	**Base Year**	**Base Line**	**2023/24**	**2024/25**	**2025/26**	**2026/27**
% of water for production facilities that are functional	2017/18	86.7%	89%	89.2%	89.7%	89.9%
Area under formal irrigation (ha)	2017/18	15,147	23000	25000	27000	30000
Cumulative water for production storage capacity (Mcm)	2017/18	39.3MCM	55%	57MCM	59MCM	63MCM
**Programme Outcome**	Increased food security					
**Programme Objectives contributed to by the Intermediate Outcome**						
Increase production and productivity						
			**Performance Targets**			
**Programme Outcome Indicators**	**Base Year**	**Base Line**	**2023/24**	**2024/25**	**2025/26**	**2026/27**
% of food secure households	2017/18	69%	87.3%	89.84%	89.96%	92.38%

AGRO-INDUSTRIALIZATION

**Programme Outcome**	Increased employment and labour productivity in agro-industry					
**Programme Objectives contributed to by the Intermediate Outcome**						
Increase production and productivity						
			**Performance Targets**			
**Programme Outcome Indicators**	**Base Year**	**Base Line**	**2023/24**	**2024/25**	**2025/26**	**2026/27**
Labour productivity in agriculture (USD)	2017/18	2212	1000	1100	1210	1420
Number of jobs created in the agro-industrial value chain	2017/18	75000	100000	100000	105000	115000
Proportion of households dependent on subsistence agriculture as a main source of livelihood (%)	2017/18	68%	39%	38%	35%	32%
**Programme Outcome**	Increased access and utilization of agricultural finance					
**Programme Objectives contributed to by the Intermediate Outcome**						
Increase the mobilization, equitable access and utilization of Agricultural Finance						
			**Performance Targets**			
**Programme Outcome Indicators**	**Base Year**	**Base Line**	**2023/24**	**2024/25**	**2025/26**	**2026/27**
Proportion of farmers that access agricultural finance	2017/18	33%	16.1%	18.9%	21.7%	24.7%
Share of agricultural financing to total financing	2017/18	2%	13.2%	14.6%	16.6%	19.0%
**Programme Outcome**	Improved service delivery					
**Programme Objectives contributed to by the Intermediate Outcome**						
Strengthen institutional coordination for improved service delivery						
			**Performance Targets**			
**Programme Outcome Indicators**	**Base Year**	**Base Line**	**2023/24**	**2024/25**	**2025/26**	**2026/27**
Level of satisfaction with service delivery in agroindustry	2017/18	20%	63.0%	65.0%	68%	73%

Table P2.2: Intermediate Outcomes Indicators

**Sub-Programme Name:**	**Institutional Strengthening and Coordination**					
**Intermediate Outcome Indicators:**	Institutional coordination strengthened for improved service delivery					
			**Performance Targets**			
**Indicators**	**Base Year**	**Base Line**	**2023/24**	**2024/25**	**2025/26**	**2026/27**
Existence of inclusive institutionalized mechanism for mutual accountability and peer review, %	2017/18	0	100%	100%	100%	100%
Level of satisfaction with service delivery in agro-industry (%)	2017/18	20%	51.3%	52.8%	54.3%	56.3%
Evidence-based policies with supportive institutions and corresponding human resource	2017/18	71.1%	100%	100	100	100

AGRO-INDUSTRIALIZATION

**Sub-Programme Name:**	**Agricultural Production and Productivity**					
**Intermediate Outcome Indicators:**	Increased adoption of Agricultural research technologies					
			**Performance Targets**			
**Indicators**	**Base Year**	**Base Line**	**2023/24**	**2024/25**	**2025/26**	**2026/27**
Quantity of invasive aquatic weeds cleared (tons)	2017/18	2000	45000	50000	55000	61500
Volume of fish stock in the major water bodies (MT)	2017/18	661378	970000	1100000	1193622	1288622
Incidence of crop diseases and pests (FAW, Caterpillars, desert locust. BBW, Maize lethal necrosis, coffee leaf rust, cassava mosaic) (%)	2017/18	30%	10%	8%	7.4%	6.2%
Percentage of farmers accessing labour saving technologies by type (Tractors, bulldozers and DAP)	2017/18	11.3%	20.3%	21.7%	23.1%	24.5%
Percentage of farmers with access to water for Agricultural production	2017/18	11.0%	22.1%	22.9%	24.1%	25.5%
Proportion of farmers adopting improved agricultural technologies, %	2017/18	22.8%	33%	35.4%	37.8%	39.9%
Proportion of farmers enrolled on the E-voucher Management System (accessing inputs)	2017/18	2.0%	6.5%	6.9%	7.5%	8.3%
Proportion of farmers practicing sustainable land management practices (%)	2021	30%	34.5%	35.2%	35.9%	37.1%
Proportion of farming households accessing agricultural extension services, %	2017/18	11.7%	34.1%	35.4%	36.7%	38.8%
Area under formal irrigation (ha)	2017/18	19392	23762	27424	31186	35048
Number of districts free from state controlled animal vectors and diseases (considering 136 districts)	2017/18	82	102	112	120	128
**Sub-Programme Name:**	**Storage, Agro-Processing and Value addition**					
**Intermediate Outcome Indicators:**	Improved Post-Harvest Handling and Storage of Agricultural Products					
			**Performance Targets**			
**Indicators**	**Base Year**	**Base Line**	**2023/24**	**2024/25**	**2025/26**	**2026/27**
Storage Capacity (MT)	2017/18	550000	1350000	1500000	1600000	1750000
Post-harvest losses for priority commodities (%)	2017/18	37%	18%	15%	12%	9%
**Intermediate Outcome Indicators:**	Increased agro-processing and value addition					
			**Performance Targets**			
**Indicators**	**Base Year**	**Base Line**	**2023/24**	**2024/25**	**2025/26**	**2026/27**
Agriculture Value added in current prices (billion Ugx)	2017/18	27993	35240	36650	38005	39415
**Sub-Programme Name:**	**Agricultural Market Access and Competitiveness**					
**Intermediate Outcome Indicators:**	Increased market access and competitiveness of agricultural products in domestic and international markets					
			**Performance Targets**			
**Indicators**	**Base Year**	**Base Line**	**2023/24**	**2024/25**	**2025/26**	**2026/27**
Quantity of Domestic Coffee Consumption	2017/18	0.5	0	0.73	0.76	0.79

AGRO-INDUSTRIALIZATION

Sub-Programme Name:	Agricultural Market Access and Competitiveness					
Intermediate Outcome Indicators:	Increased market access and competitiveness of agricultural products in domestic and international markets					
	Performance Targets					
Indicators	Base Year	Base Line	2023/24	2024/25	2025/26	2026/27
Percentage of lint classed on the top 3 grades	2017/18	79%	83%	84%	85%	86%
Export value of priority agricultural commodities (USD Billion)	2017/18	1.65	2.2	2.35	2.5	2.6
Value of agricultural imports ($ million)	2017/18	931.1	1000	950	900	850
Sub-Programme Name:	Agricultural Financing					
Intermediate Outcome Indicators:	Increased mobilisation, access and utilisation of agricultural finance					
	Performance Targets					
Indicators	Base Year	Base Line	2023/24	2024/25	2025/26	2026/27
Proportion of farmers accessing agriculture financing	2017/18	2%	16.1%	18.9%	21.7%	24.5%

P3: Medium Term Budget Allocations by Sub-Programme and Vote

Table P3.1: Proposed Budget Allocations and Medium Term Projections by Sub-Programme

Billion Uganda Shillings	FY2022/23	2023/24	Medium Term Projections			
	Approved Budget	Proposed Budget	2024/25	2025/26	2026/27	2027/28
01 Institutional Strengthening and Coordination	183.001	177.583	200.971	219.548	256.884	261.884
02 Agricultural Production and Productivity	1,116.177	1,234.492	1,226.139	976.252	1,020.156	982.468
03 Storage, Agro-Processing and Value addition	62.120	25.812	31.535	63.101	43.101	75.789
04 Agricultural Market Access and Competitiveness	81.804	61.442	26.553	51.500	60.610	60.683
**Total for the Programme**	**1,449.811**	**1,499.329**	**1,485.197**	**1,310.401**	**1,380.750**	**1,380.824**

Table P3.2: Proposed Budget Allocations and Medium Term Projections by Vote

Billion Uganda Shillings	FY2022/23		2023/24	Medium Term Projections			
	Approved Budget	Spent by End Sep	Proposed Budget	2024/25	2025/26	2026/27	2027/28
010 Ministry of Agriculture, Animal Industry and Fisheries	564.386	80.270	618.197	680.725	716.508	621.423	621.497
011 Ministry of Local Government	49.233	4.630	0.260	0.269	0.310	0.384	0.384
015 Ministry of Trade, Industry and Co-operatives	10.150	0	9.840	10.037	12.044	16.260	16.260
019 Ministry of Water and Environment	205.038	3.691	466.165	377.780	101.294	141.377	141.377
021 Ministry of East African Community Affairs	0.251	0.005	0.250	0.255	0.306	0.413	0.413
108 National Planning Authority (NPA)	0.937	0	0.800	0.816	0.979	1.322	1.322

AGRO-INDUSTRIALIZATION

Billion Uganda Shillings	FY2022/23		2023/24	Medium Term Projections			
	Approved Budget	Spent by End Sep	Proposed Budget	2024/25	2025/26	2026/27	2027/28
121 Dairy Development Authority (DDA)	17.239	1.719	13.218	13.594	15.870	20.252	20.252
122 Kampala Capital City Authority (KCCA)	7.188	0.026	0.350	0.357	0.428	0.578	0.578
125 National Animal Genetic Resource Centre and Data Bank (NAGRC&DB)	93.169	14.836	80.356	80.800	96.358	132.432	132.432
142 National Agricultural Research Organization (NARO)	116.783	22.382	101.371	103.843	120.537	153.676	153.676
152 National Agricultural Advisory Services (NAADS)	58.065	1.950	33.815	34.581	41.150	54.625	54.625
154 Uganda National Bureau of Standards (UNBS)	1.110	0.457	0.940	0.959	1.151	1.553	1.553
155 Cotton Development Organization	7.733	1.079	5.533	5.698	6.627	8.383	8.383
160 Uganda Coffee Development Authority (UCDA)	64.940	4.195	47.505	48.719	57.399	74.686	74.686
601 Local Governments 01	245.493	40.759	120.729	126.765	139.442	153.386	153.386
**Total for the Programme**	**1,449.811**	**177.282**	**1,499.329**	**1,485.197**	**1,310.401**	**1,380.750**	**1,380.824**

P4: PROGRAMME PRIORITIES IN LINE WITH INTERVENTIONS FOR FY2023/24

Programme Priorities FY2023/24	NDP III Programme Intervention
Construction of storage facilities and installation of value addition equipment for 362 beneficiary ACCEs under the Matching Grants Scheme.<br>Support capacity building for Farmer Organizations by Enterprise Uganda and Uganda Cooperative Alliance to manage their business enterprises effectively and to scale up their operations.<br>Procure 28 sets of maize milling equipment<br>Procure 5 sets of Rice milling equipment<br>Procure small-scale cassava value addition equipment (Chippers, solar dryers & milling machines etc.)<br>Rehabilitate and equip Mbale dairy processing factory- Phase II<br>Provision of equipment for 01 micro to small scale milk dairy processing facility (250 to 500 Ltrs/batch)<br>Provision of 10 sets of milk coolers & matching generators<br>Promote and develop cottage industries in production of market grade livestock products (Processing and value addition for eggs, dairy products, meat products and pelleted/blocked/canned feed<br>Support training and incubation at Entebbe Dairy Training School<br>Silk products promoted as alternative fibre for the textile industry in Uganda. The textile industry employs mostly women and youth who will therefore be the greatest beneficiaries.	Establish post-harvest handling, storage and processing infrastructure including silos, dryers, warehouses, and cold rooms of various scale and capacities at subcounty, district and zonal levels.

AGRO-INDUSTRIALIZATION

Programme Priorities FY2023/24	NDP III Programme Intervention
Construction supervision and monitoring the Atari Irrigation Scheme<br>Construction supervision and monitoring the Acomai Irrigation Scheme<br>Design, Construct and develop 400 water facilities (Valley tanks, dams and on farm water Harvesting and storage infrastructure) of capacity (7,500 to 50,000) cubic meters in all the four regions (65 Districts sites)<br>Construct of medium to large-scale fishponds in the various water catchment areas in different parts of the country with Agriculture mechanisation equipment and machinery.<br>Provide logistical support for operations of the irrigation schemes (Mubuku, Doho, and Odina)<br>Construction, Rehabilitation and Operationalization of Small Scale Mechanized Water and Irrigation Facilities in selected areas.	Increase access and use of water for agricultural production
Support the equipping and operations of the new Zonal Agriculture Mechanization Centre of Kiryandongo, Kiruhuru, Abim, Buwama, West Nile, Busoga and Agwata i.e. contract staff salaries, fuel for maintenance and general staff welfare<br>Provide logistical and maintaining the operations of the fleet of Heavy Earth moving equipment that stationed in Namalere and Regional Centres<br>A total of 200 assorted tractors & matching implements procured & equitably distributed to all regions of Uganda for agricultural mechanization in an effort to boost agricultural production towards commercialization.<br>Purchase additional sets of earth moving equipment and Spare parts for heavy equipment and additional sets of heavy (earth moving) equipment: Hydraulic excavators, Drilling Rigs, bulldozers with rippers, Chain/Wheel Loaders, Water Bowsers, Dump Trucks, Pick Ups, Service Vans, Tandem Rollers, procured<br>Construction and Equipping of the 3 additional Regional Agricultural Mechanization Centers in Eastern (Mbale), Northern (Agwata), Western (Bushenyi/Mbarara) and Central (Kiryandongo, Buwama) Abim, Buwama, West Nile and Busoga	Increase access to and use of agricultural mechanisation
Vaccine and pharmacological products development for humans and livestock: Anti-tick vaccine, FMD and ASF, bioactive compounds.<br>Development of high yielding resilient crop varieties and animal breeds<br>Determining bio-active compounds resident in agro biodiversity resources for aesthetic and pharmaceutical applications.<br>Develop technological innovations to enhance soil health for suitable yields (Bio-fertilizer, fertilizer optimiser tools)	Strengthen agricultural research and technology development

AGRO-INDUSTRIALIZATION

Programme Priorities FY2023/24	NDP III Programme Intervention
Construction of National Metrology Laboratory at UNBS to calibrate equipment<br>Facilitate Inspection and auditing of planting materials of fruits and vegetables.<br>Carry out inspections and Pest Monitoring in places of production for FFVs, Fumigation facilities and agro-chemical shops, seed crops<br>Strengthening traceability and investigating, Noncompliance due to Harmful organisms and Chemical residues<br>50 new inspectors recruited to strengthen inspection, certification and regulation of inputs in zones and border posts<br>Namalere laboratories for testing for pesticide formulations, residues and contaminants equipped<br>Operationalize the Plant health and Chemical residue testing laboratory<br>Inspection and certification of coffee for export to ensure quality compliance to international standards, physical and sensory analysis of coffee to evaluate coffee quality levels, field visits to enforce coffee quality along the coffee value chain to ensure compliance, and conducting Q- Robusta and Q-Arabica courses in collaboration with Coffee Quality Institute of America)<br>Inspection and certification of farms (Poultry, piggery, Beef cattle), abattoirs, processing facilities (meat, eggs, hides & skin, animal feed), and livestock products export trade establishments<br>Veterinary inspection, certification and enforcement of sanitary and welfare compliance and adherence in animal product processing establishments and at ports of entry or exit supported<br>Inspection, registration, licensing and certification of dairy products and dairy inputs providers<br>Completion of the International accreditation of the National Dairy Analytical Laboratory<br>Procure Laboratory equipment and reagents to support analysis and milk sample testing<br>Residue analyses conducted and Quality control measures implemented for maintenance of Uganda beehive products (Honey,<br>beeswax and propolis, among others) quality on the Local, Regional and International markets	Strengthen enforcement and adherence to product quality requirements including; food safety, social and environmental standards, grades, etc.
Community mobilisation and information sharing through monthly barazas and dialogues.<br>Farmer group capacity building on enterprise value chain development	Strengthen farmer organizations and cooperatives

AGRO-INDUSTRIALIZATION

Programme Priorities FY2023/24	NDP III Programme Intervention
Programme actors will identify, profile, and partner with largescale commercial farmers (especially those with big chunks of land) to produce on a large scale, identified high-value commodities with high national and international demand. The smallholder farmers will be supported to work with co-operatives, which will collaborate with the large commercial farmers. The smallholder farmers will in effect be helped to produce qualitatively and sustainably for the market.	Strengthen linkages between public and private sector in agro-industry
Regional Animal Disease Control Centre in Kiruhura District and Zonal Animal Disease control Centre in Gwot Apwoyo constructed.<br><br>Disease surveillance, monitoring and investigations for BSE, FMD, CBPP, PPR and zoonoses carried out in the country; Surveys to assess acaricide and antimicrobial use at farm level to contain resistance development undertaken.<br><br>Animal movement regulation and control along gazetted major stock routes in Eastern, Northern, Central and Western regions<br><br>Holistic farmer education for effective disease prevention and control targeting FMD, Anthrax, African Swine Fever and Peste desPetits Ruminants. 60 Districts in the cattle corridor and 16 District with high pig population are targeted.<br><br>Residue analyses conducted and Quality control measures implemented for maintenance of Uganda beehive products (Honey,beeswax and propolis, among others) quality on the Local, Regional and International markets.<br><br>Inspection of crop pests and disease of mother gardens and nurseries to ensure pest and disease-free planting materials in at least 80districts across the country conducted.<br><br>Plant clinic diagnostic mini labs equipped to feed into the national plant health laboratory.<br><br>380 extension service providers/ToTs trained in management and control of crop pests and diseases<br><br>Demonstrations of treating cattle with trypanocidals in Trypanosomiasis high risk areas and live-bait technology in controlling vectors Bunyoro, Karamoja, Teso, Busoga, Bukedi, Lango, Acholi, Buganda, West Nile and Rwenzori	Strengthen systems for management of pests, vectors and diseases:

AGRO-INDUSTRIALIZATION

Programme Priorities FY2023/24	NDP III Programme Intervention
Community mobilisation and information sharing through all inclusive monthly barazas and dialogues<br>leveraging the wide media space (TV. Radio, newspapers, and social media) to make the farming conversation dominant conducted.<br><br>E-Extension systems for real-time information sharing and monitoring of extension services delivery rolled out to 60 districts across the country.<br><br>Commodity specific enterprise farmer guides/manuals developed.<br><br>The National Agricultural Extension Bill formulated.	Strengthen the agricultural extension system
Namalere laboratories for testing for pesticide formulations, residues and contaminants equipped<br>National Seed Laboratory in Namalere constructed to support seed regulation and certification<br>Strengthening traceability and investigating, Noncompliance due to Harmful organisms and Chemical residues.<br>Intensify inspection and certification of input dealers, importers and exporters of agricultural inputs and enforce adherence to quality assurance regulations including facilitation of border post agricultural inspectors)<br>Facilitate agricultural inspection services along the value chains to ensure compliance to sanitary standards.<br>Local farmer associations supported to undertake multiplication of improved seed and planting materials.<br>100 Nursery Operators trained on good agricultural practices for production of seed and planting materials.<br>20 new inspectors recruited to strengthen inspection, certification and regulation of inputs in zones and border posts.<br>Starter feeds and fingerlings distributed to 120 model Fish Farmer Aquaculture Cooperatives i.e. 1 model farmer of cooperative society<br>Inspection and certification of farms (Poultry, piggery, Beef cattle), abattoirs, processing facilities (meat, eggs, hides & skin, animal feed), and livestock products export trade establishments.<br>Out grower schemes for the feedlots and Abattoirs for meat export (Technical support, provision of parent stock/seed, training) developed through partnerships with big commercial farmers.	Strengthen the agricultural inputs markets and distribution systems to adhere to quality standards and grades

P5: GENDER AND EQUITY ISSUES AND INTERVENTIONS FOR FY2023/24

Women tend to be responsible for procuring and providing food in households and are the primary workers engaged in subsistence agriculture. They make up an average of 43 percent of the agricultural workforce in developing countries with 79 [illegible]

AGRO-INDUSTRIALIZATION

per cent of women in the least developed countries who are economically active and agriculture is their primary economic activity. Ownership of farmland was found to be in favour of men where over 72 per cent of the interviewed men owned their farmland, whereas only 8 per cent of women owned the same.

Gender mainstreaming is recommended in the sector plans, strategies, budgets and programmes and projects at both the national and Local Government Levels. The implementation of the gender mainstreaming strategy for Uganda agricultural sector is a multisectoral approach in the areas of agricultural productivity, investment, training and market access. It is therefore recommended that a programme to empower women smallholder farmers to have control and a joint venture between MAAIF and MFPED on agricultural credit facilities that specifically address the different needs of men and women be developed. Additionally, the post-harvest process which is a critical process of the agricultural value chain is handled 98 percent by men. Access to agricultural technologies was lower for both men and women but with men at 46 percent and women at 19 percent. This recommended Programme will therefore enable women to access agricultural technologies and inputs.

MINERAL DEVELOPMENT

Foreword

The Mineral Development Programme Implementation Action Plan sets out the key outputs together with their actions that will be delivered under the Minerals Programme in response to NDP III outcomes and targets within the period 2020/21 to 2024/25.

The goal of this Programme is to increase the exploitation and value addition to selected resources for job rich industrialization, with the following objectives;
1. Increase exploration and quantification of priority minerals and geothermal resources across the country;
2. Increase adoption and use of appropriate and affordable technology along the value chain;
3. Strengthen the legal and regulatory framework as well as the human and institutional capacity;
4. Increase investment in mining and value addition; and
5. Expand mineral based processing and marketing.

The programme in turn contributes to the first objective of the NDP III, which is to 'Enhance Value Addition in Key Growth Opportunities.

Minerals have a significant general value to an economy from both a financial and an employment standpoint. This implies that investing in mineral development will lower the cost of production and boost the supply of locally manufactured products like cement, iron and steel, and fertilizers.

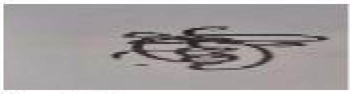

Irene Batoebe

Permanent Secretary

MINERAL DEVELOPMENT

Abbrevations and Acronyms

ACRONYM	ACRONYM NAME
ASMs	Artisanal Small-Scale Miners
Bn	Billion
CDAs	Community Development Agreements
CRMs	Certified Reference Materials
DGSM	Directorate of Geological Survey and Mines
DPs	Development Partners
DWRM	Directorate of Water Resources Management
FDI	Foreign Direct Investment
ICGLR	International Conference on the Great Lakes Region
Kg	Kilograms
Kms	Kilometres
LC	Leadership Council
M& E	Monitoring and Evaluation
Mn	Million
MoES	Ministry of Education and Sports
MoICT&NG	Ministry of Information and Communication Technology & National Guidance
MoSTI	Ministry of Science, Technology and Innovation
MoTIC	Ministry of Trade, Industry and Cooperatives
MoWE /MWE	Ministry of Water and Environment
MoWT	Ministry of Works and Transport
NITA-U	National Information Technology Authority
OSH	Occupational Safety and Health
PBFP	Programme Budget Framework Paper
PIAP	Programme Implementation Action Plan
PPPs	Policy, Plan and Programmes
PPPU	Public and Private Partnership Unit
PWG	Program Working Group
R & D	Research and Development
SDG	Sustainable Development Goals
TWG	Technical Working Group
UBOS	Uganda Bureau of Statistics
UCMP	Uganda Chamber of Mines and Petroleum
UDB	Uganda Development Bank

MINERAL DEVELOPMENT

ACRONYM	ACRONYM NAME
UDC	Uganda Development Corporation
UEPB	Uganda Export Promotion Board
UFZA	Uganda Free Zones Authority
UNFC	United Nations Framework Classification for Resources
USD	United States Dollars
UWA	Uganda Wildlife Authority

MINERAL DEVELOPMENT

P1: PROGRAMME OVERVIEW

Snapshot of Medium Term Budget Allocations

Table P1.1 Overview of Programme Expenditure and Medium Term Allocations (Ush Billion)

Billion Uganda Shillings		FY2022/23		FY2023/24	MTEF Budget Projections			
		Approved Budget	Spent by End Sep	Proposed Budget	2024/25	2025/26	2026/27	2027/28
Recurrent	Wage	0.000	0.000	5.500	5.775	6.353	6.988	6.988
	Non Wage	6.991	0.101	11.000	11.220	13.464	18.176	18.176
Devt.	GoU	17.420	0.162	22.000	22.000	26.400	36.960	36.960
	ExtFin	7.375	0.000	0.000	0.000	0.000	0.000	0.000
	**GoU Total**	**24.411**	**0.263**	**38.500**	**38.995**	**46.217**	**62.124**	**62.124**
	**Total GoU+Ext Fin (MTEF)**	**31.786**	**0.263**	**38.500**	**38.995**	**46.217**	**62.124**	**62.124**
	A.I.A	0.000	0.000	0.000	0.000	0.000	0.000	0.000
	**Grand Total**	**31.786**	**0.263**	**38.500**	**38.995**	**46.217**	**62.124**	**62.124**

Programme Strategy and linkage to the National Development Plan

The goal of and theme of NDPIII are "Increased Household Incomes and Improved Quality of Life of Ugandans" and "Sustainable Industrialization for inclusive growth, employment and wealth creation" respectively.The programme in turn contributes to the first objective of the NDP III, which is to 'Enhance Value Addition in Key Growth Opportunities. Goal: To increase the exploitation and value addition to selected mineral resources for job rich industrialization

P2: Highlights Of Programme Projected Performance

Table P2.1 Programme Outcomes Indicators

**Programme Outcome**	Reduced importation of mineral products					
**Programme Objectives contributed to by the Intermediate Outcome**						
Expand mineral processing and marketing						
	Performance Targets					
**Programme Outcome Indicators**	**Base Year**	**Base Line**	**2023/24**	**2024/25**	**2025/26**	**2026/27**
Value of imported inorganic fertilizers (USD Mn)	2019/20	26	10.1	7.5	7	6
Value of imported Iron and Steel (USD Mn)	2019/20	370	136	97	90	80
Volume of imported inorganic fertilizers (tonnes)	2019/20	75000	30982	18750	17000	15000
Volume of imported Iron and Steel (tonnes)	2019/20	700000	200000	125000	100000	95000

MINERAL DEVELOPMENT

**Programme Outcome**	Increased investment in the sector					
**Programme Objectives contributed to by the Intermediate Outcome**						
Explore and quantify priority mineral resources across the country						
			**Performance Targets**			
**Programme Outcome Indicators**	**Base Year**	**Base Line**	**2023/24**	**2024/25**	**2025/26**	**2026/27**
Contribution of processed minerals to total manufactured exports (%)	2019/20	5%	6.6%	7.1%	7.5%	8%
Value of investment (UGX Bn)	2019/20	185	200	300	400	500
Value of investment into the exploration and processing of selected minerals (Bn USD)	2019/20	0.8	1.7	2	3	5
**Programme Outcome**	Competitive mining sector					
**Programme Objectives contributed to by the Intermediate Outcome**						
Explore and quantify priority mineral resources across the country						
			**Performance Targets**			
**Programme Outcome Indicators**	**Base Year**	**Base Line**	**2023/24**	**2024/25**	**2025/26**	**2026/27**
Share of the global investment in mining, %	2019/20	0.3%	0.5%	0.8%	1%	1.2%
**Programme Outcome**	Increased mineral production					
**Programme Objectives contributed to by the Intermediate Outcome**						
Increase adoption and use of appropriate and affordable technology along the value chain						
			**Performance Targets**			
**Programme Outcome Indicators**	**Base Year**	**Base Line**	**2023/24**	**2024/25**	**2025/26**	**2026/27**
Volume of minerals produced by type (tonnes) - Copper	2019/20	0	1500	2000	2500	3000
Volume of minerals produced by type (tonnes) - Gold	2019/20	450	900	1000	1200	1500
Volume of minerals produced by type (tonnes) - Graphite	2019/20	0	0	0	0	0
Volume of minerals produced by type (tonnes) - Iron Ore	2019/20	9000	15000	20000	22000	25000
Volume of minerals produced by type (tonnes) - Limestone (Mn tons)	2019/20	1	2	2	2	3
Volume of refined Gold exports (USD Bn)	2019/20	1	1	1	2	2
**Programme Outcome**	Increased mineral revenue earnings					
**Programme Objectives contributed to by the Intermediate Outcome**						
Increase adoption and use of appropriate and affordable technology along the value chain						
			**Performance Targets**			
**Programme Outcome Indicators**	**Base Year**	**Base Line**	**2023/24**	**2024/25**	**2025/26**	**2026/27**
Contribution of mining sector to GDP (%)	2019/20	1.4%	2.5%	3%	4%	5%
NTR (UGX Bn)	2019/20	12	16	20	25	30

MINERAL DEVELOPMENT

**Programme Outcome**	Sustainable mining practices adopted					
**Programme Objectives contributed to by the Intermediate Outcome**						
Increase adoption and use of appropriate and affordable technology along the value chain						
	**Performance Targets**					
**Programme Outcome Indicators**	**Base Year**	**Base Line**	**2023/24**	**2024/25**	**2025/26**	**2026/27**
Carbon emissions per value added	2019/20	0	0	0	0	0
**Programme Outcome**	Increased mineral beneficiation facilities					
**Programme Objectives contributed to by the Intermediate Outcome**						
Increase adoption and use of appropriate and affordable technology along the value chain						
	**Performance Targets**					
**Programme Outcome Indicators**	**Base Year**	**Base Line**	**2023/24**	**2024/25**	**2025/26**	**2026/27**
Number of mineral beneficiation facilities	2019/20	4	7	8	8	10
**Programme Outcome**	Increased investment in the sector					
**Programme Objectives contributed to by the Intermediate Outcome**						
Increase investment in mining and value addition						
	**Performance Targets**					
**Programme Outcome Indicators**	**Base Year**	**Base Line**	**2023/24**	**2024/25**	**2025/26**	**2026/27**
Contribution of processed minerals to total manufactured exports (%)	2018/20	5%	6.6%	7.1%	7.5%	8%
**Programme Outcome**	Increased employment in the sector					
**Programme Objectives contributed to by the Intermediate Outcome**						
Increase investment in mining and value addition						
	**Performance Targets**					
**Programme Outcome Indicators**	**Base Year**	**Base Line**	**2023/24**	**2024/25**	**2025/26**	**2026/27**
Number of people employed mineral sector (million)	2019/20	1	2	3	4	5
**Programme Outcome**	Effective regulatory framework					
**Programme Objectives contributed to by the Intermediate Outcome**						
Strengthen the legal and regulatory framework as well as the human and institutional capacity						
	**Performance Targets**					
**Programme Outcome Indicators**	**Base Year**	**Base Line**	**2023/24**	**2024/25**	**2025/26**	**2026/27**
No. of Legal and regulatory framework in place	2019/20	1	1	1	1	1
Proportion of licensees adhering to requirements (%)	2019/20	70%	75%	80%	90%	100%

MINERAL DEVELOPMENT

Programme Outcome	Skilled and competitive human resource					
**Programme Objectives contributed to by the Intermediate Outcome**						
Strengthen the legal and regulatory framework as well as the human and institutional capacity						
	**Performance Targets**					
**Programme Outcome Indicators**	**Base Year**	**Base Line**	**2023/24**	**2024/25**	**2025/26**	**2026/27**
Geoscientists trained	2019/20	108	180	200	230	250
No. of skilled human resource	2019/20	800	1300	1500	2000	2500
**Programme Outcome**	Functional and sustainable physical infrastructure					
**Programme Objectives contributed to by the Intermediate Outcome**						
Strengthen the legal and regulatory framework as well as the human and institutional capacity						
	**Performance Targets**					
**Programme Outcome Indicators**	**Base Year**	**Base Line**	**2023/24**	**2024/25**	**2025/26**	**2026/27**
No. of functional laboratory techniques	2019/20	15	25	30	35	40
No. of functional seismological stations	2019/20	5	12	15	15	15
No. of geophysical techniques	2019/20	4	7	7	8	10
No. of regional offices and beneficiation centers	2019/20	4	7	8	8	10

Table P2.2: Intermediate Outcomes Indicators

**Sub-Programme Name:**	**Mineral exploration, development and value addition**					
**Intermediate Outcome Indicators:**	Sustainable mining practices adopted					
	**Performance Targets**					
**Indicators**	**Base Year**	**Base Line**	**2023/24**	**2024/25**	**2025/26**	**2026/27**
Carbon emissions per value added	2019/20	0	0	0	0	0
**Intermediate Outcome Indicators:**	Functional and sustainable physical infrastructure					
	**Performance Targets**					
**Indicators**	**Base Year**	**Base Line**	**2023/24**	**2024/25**	**2025/26**	**2026/27**
No. of effective geophysical techniques	2019/20	4	7	7	8	10
No. of functional laboratory techniques	2019/20	15	25	30	30	35
No. of functional regional offices and beneficiation centers	2019/20	4	7	7	8	10
No. of functional seismological stations.	2019/20	5	12	15	15	15
**Intermediate Outcome Indicators:**	Increased employment in the sector					
	**Performance Targets**					
**Indicators**	**Base Year**	**Base Line**	**2023/24**	**2024/25**	**2025/26**	**2026/27**
Number of people employed in the mineral sector (million)	2019/20	1	2	3	4	5

MINERAL DEVELOPMENT

Sub-Programme Name:	Mineral exploration, development and value addition					
**Intermediate Outcome Indicators:**	Increased mineral beneficiation facilities					
	Performance Targets					
**Indicators**	**Base Year**	**Base Line**	**2023/24**	**2024/25**	**2025/26**	**2026/27**
Number of mineral beneficiation facilities	2019/20	4	7	8	8	10
**Intermediate Outcome Indicators:**	Functional and sustainable physical infrastructure					
	Performance Targets					
**Indicators**	**Base Year**	**Base Line**	**2023/24**	**2024/25**	**2025/26**	**2026/27**
No. of effective geophysical techniques	2019/20	4	7	7	7	8
No. of functional laboratory techniques	2019/20	15	25	30	30	35
No. of functional regional offices and beneficiation centers	2019/20	4	7	8	8	10
No. of functional seismological stations.	2019/20	5	12	15	15	15

P3: Medium Term Budget Allocations by Sub-Programme and Vote

Table P3.1: Proposed Budget Allocations and Medium Term Projections by Sub-Programme

Billion Uganda Shillings	FY2022/23	2023/24	Medium Term Projections			
	Approved Budget	Proposed Budget	2024/25	2025/26	2026/27	2027/28
01 Mineral exploration, development and value addition	29.851	38.500	38.995	46.217	62.124	62.124
**Total for the Programme**	**31.786**	**38.500**	**38.995**	**46.217**	**62.124**	**62.124**

Table P3.2: Proposed Budget Allocations and Medium Term Projections by Vote

Billion Uganda Shillings	FY2022/23		2023/24	Medium Term Projections			
	Approved Budget	Spent by End Sep	Proposed Budget	2024/25	2025/26	2026/27	2027/28
017 Ministry of Energy and Mineral Development	29.851	0.250	38.500	38.995	46.217	62.124	62.124
**Total for the Programme**	**31.786**	**0.263**	**38.500**	**38.995**	**46.217**	**62.124**	**62.124**

P4: PROGRAMME PRIORITIES IN LINE WITH INTERVENTIONS FOR FY2023/24

Programme Priorities FY2023/24	NDP III Programme Intervention
Finalize the development of Local Content strategy for mining	Develop and implement training and apprenticeship programmes in areas identified under 6.3.3 human resource requirements of the programme;
Implement the International Conference on the Great Lakes Region (ICGLR) Regional Certification Mechanism (RCM	Domesticate appropriate regional and international treaties, conventions, agreements, protocols which support good governance in the mining industry

MINERAL DEVELOPMENT

Programme Priorities FY2023/24	NDP III Programme Intervention
Adequate and reliable infrastructure services of water, roads and ICT extended to mining and minerals processing zones	Extend transport, energy, water and ICT infrastructure to mining areas and mineral processing facilities/industries;
To have Increased employment, business activity and incomes associated with infrastructure projects along the major mining areas	Implement local content in public procurement to use and develop existing mineral potential, particularly in the major upcoming projects like highways connecting Kampala to the neighbouring cities, Ayago hydro-power generation plant, and regional markets like South Sudan, Democratic Republic of Congo and Rwanda.
Increased domestic production of mineral-based products	Increase public investment in priority mineral processing;
1.To continue promoting the use of PPPs in mining value chain<br>2.Establish a prospectus of bankable projects<br>3.Establish and support partnerships with the private sector to increase mineral<br>based products	Introduce incentive packages to attract investment in priority mineral value chain;
Register and develop a database for artisanal and small scale miners	Provide incentives for acquisition of appropriate and clean technology
Engage in multi-lateral negotiations to increase the minerals export market	Streamline the process for acquisition and dissemination of minerals market information;
Institutional rearrangement through restructuring<br><br>Procure mineral certification, trading, testing, inspection, regulation and<br>enforcement equipment<br>Staffing and training<br><br>Regulations to enforce the Mining and Minerals Act, 2022 developed for the mineral sector	Strengthen capacity to monitor, inspect and enforce health, safety and environmental provisions;
1.Drilling of additional eight (8) Temperature GradientHoles (TGH) , 4 each at Kibiro and Panyimur prospectsand update the conceptual models of the two areas..<br>2.Logging of Twenty four (24) TGH at Kibiro and Panyimur geothermal<br>prospects.<br>3.Designing and siting eight (8) exploration wells at Kibiro and Panyimur<br>geothermal prospects.<br>4.Environmental and Social Impact Assessment (ESIA) for drilling of Exploration Wells at Kibiro and Panyimur.<br>5.Detailed surface studies on Ihimbo, and Kanangorok geothermal sites	Undertake a detailed exploration and quantification of minerals and geothermal resources in the country
Incentivize local companies to produce and supply mineral products to infrastructure projects.<br><br>Register and recognize companies supplying required materials for infrastructure projects (Purpose to make this a supplier data base)	Undertake feasibility studies in priority mineral value chains to guide investment
To continue promoting the use of PPPs in mining value chain	Undertake PPPs to invest in mineral value addition;

MINERAL DEVELOPMENT

P5: GENDER AND EQUITY ISSUES AND INTERVENTIONS FOR FY2023/24

Increase adoption and use of appropriate and affordable technology along the value chain, especially for ASMs.
Formalization of Artisanal and Small-scale Miners (ASMs) that include women, men , youth , people living with disabilities (PWDs)
Creation of awareness for Artisanal and Small-scale Miners on cross cutting issues . the interventions are 1.Continued Staff training and sensitization on gender and equity issues and the value of engendering the budget to cater for all Ugandans
2.Sustain a good performance rating mark of the gender and equity provision

SUSTAINABLE PETROLEUM DEVELOPMENT

Foreword

The Sustainable Development of Petroleum Resources Programme aims to attain equitable value from the petroleum resources and spur economic development in a timely and sustainable manner. Key expected results include: reducing the volume and value of imported petroleum and petroleum products, increasing revenue from oil and gas industry and its contribution to GDP as well as creating more employment opportunities for Ugandans along the petroleum value chain.

This program contributes to the 3rd National Development Plan (NDPIII 2020/21 – 2024/25) objectives 1, 2 and 4 which are; Objective 1: Enhance value addition in key growth opportunities, Objective 2: Strengthen the private sector to create jobs and Objective 4: Enhance productivity & social well-being of the population.

Sustainable development of petroleum resources is one of the NDPIII priority areas and is critical for enhancing value addition to oil and gas resources as one of the key growth opportunities. It facilitates the resource-based industrialization agenda through exploitation of the available oil resources. Sustainable exploitation of petroleum resources is important in order to maximize returns for current and future generations. The realization of the results of this programme will be through six objectives stated in the NDPIII to maximize the country's returns from the oil and gas resources.

Further, the Programme will facilitate the resource-based industrialization agenda thereby contributing to export, employment and improved quality of life for the current and future generations. This will require emphasis on human capital development, conservation of the natural environment and strategic investment of the petroleum revenues to achieve equitable socio-economic and infrastructure transformation in the country.

The need for sustainable exploitation of petroleum resources is further stressed in the Sustainable Development Goals, the Africa Agenda 2063, and the EAC Vision 2050, which calls for all countries in the region to emphasize access, capacity, efficiency and sustainability of natural resources. Additionally, the Uganda Vision 2040 envisages the commercialization of oil and gas in a feasible and sustainable manner.

I would like to extend my sincere appreciation to the Programme Technical Leadership and all stakeholders that contributed to the development of this Action Plan. I also wish to recognize and appreciate the Programme Secretariat for the extra work invested in preparing and finalizing this Programme Budget Framework Paper.

I wish to call upon all the contributing Ministries, Departments, Agencies and Partners to be focused and work together as we ensure the sustainable utilisation of the petroleum resources for economic development and transformation of the Country.

For God and My Country

Irene Bateebe

PERMANENT SECRETARY

SUSTAINABLE PETROLEUM DEVELOPMENT

Abbrevations and Acronyms

ACRONYM	ACRONYM NAME
JVP	Joint Venture Partners
LG	Local Government
PAU	Petroleum Authority of Uganda
PSD	Private Sector Development
SDPR	Sustainable Development of Petroleum Resources
UNOC	Uganda National Oil Company

SUSTAINABLE PETROLEUM DEVELOPMENT

P1: PROGRAMME OVERVIEW

Snapshot of Medium Term Budget Allocations

Table P1.1 Overview of Programme Expenditure and Medium Term Allocations (Ush Billion)

Billion Uganda Shillings		FY2022/23		FY2023/24	MTEF Budget Projections			
		Approved Budget	Spent by End Sep	Proposed Budget	2024/25	2025/26	2026/27	2027/28
Recurrent	Wage	28.718	5.361	32.418	34.039	37.443	41.187	41.187
	Non Wage	743.925	93.275	352.440	359.483	431.318	582.173	582.173
Devt.	GoU	96.673	0.000	154.352	154.352	185.222	259.311	259.311
	ExtFin	0.000	0.000	0.000	596.135	909.642	431.221	0.000
	**GoU Total**	**869.316**	**98.636**	**539.210**	**547.874**	**653.983**	**882.671**	**882.671**
	**Total GoU+Ext Fin (MTEF)**	**869.316**	**98.636**	**539.210**	**1,144.008**	**1,563.625**	**1,313.893**	**882.671**
	A.I.A	0.000	0.000	0.000	0.000	0.000	0.000	0.000
	**Grand Total**	**869.316**	**98.636**	**539.210**	**1,144.008**	**1,563.625**	**1,313.893**	**882.671**

Programme Strategy and linkage to the National Development Plan

Sustainable Development of Petroleum Resources is one of the 18 programmes of the NDP III. The programme was selected given its potential to transform Uganda's economy and being critical for enhancing value addition to oil and gas resources as one of the key growth opportunities.

The Programme aims to attain equitable value from the petroleum resources and spur economic development in a timely and sustainable manner in the implementation of the NDPIII through the following objectives;

i. To ensure sustainable production and utilization of the Country's oil and gas resources;
ii. Strengthen policy, legal and regulatory frameworks as well as institutional capacity of oil and gas industry;
iii. Enhance local capacity to participate in oil and gas operations; and
iv. To promote private investment in oil and gas industry.
v. Enhance Quality Health, Safety, Security and Environment (QHSSE)
vi. Improve security of supply of refined petroleum products

P2: Highlights Of Programme Projected Performance

Table P2.1 Programme Outcomes Indicators

**Programme Outcome**	Increased participation of the local companies in the oil and gas industry					
**Programme Objectives contributed to by the Intermediate Outcome**						
To enhance local capacity to participate in oil and gas operations						
			**Performance Targets**			
**Programme Outcome Indicators**	**Base Year**	**Base Line**	**2023/24**	**2024/25**	**2025/26**	**2026/27**
Number of contracts awarded to local companies	2020	100	250	350	400	450
Number of local Companies on National Suppliers Database	2020	150	300	400	500	600

SUSTAINABLE PETROLEUM DEVELOPMENT

**Programme Outcome**	Improved safety in oil and gas industry					
**Programme Objectives contributed to by the Intermediate Outcome**						
To enhance Quality, Health, Safety, Security, Social and Environment (QHSSSE)						
			**Performance Targets**			
**Programme Outcome Indicators**	**Base Year**	**Base Line**	**2023/24**	**2024/25**	**2025/26**	**2026/27**
Lost time injury frequency rates (LTIFR)	2020	0	0	0	0	0
Total Recordable Injury frequency rate (TRIFR)	2020	0	0	0	0	0
Zero tolerance to fatalities	2020	0	0	0	0	0
**Programme Outcome**	Increased revenue from oil and gas resources					
**Programme Objectives contributed to by the Intermediate Outcome**						
To ensure sustainable production and utilization of the country's oil and gas resources						
			**Performance Targets**			
**Programme Outcome Indicators**	**Base Year**	**Base Line**	**2023/24**	**2024/25**	**2025/26**	**2026/27**
mount of revenue from oil and gas sector (UGX Bn)	2020	62	256	300	400	500
**Programme Outcome**	Increased contribution of the oil and gas sector to employment					
**Programme Objectives contributed to by the Intermediate Outcome**						
To ensure sustainable production and utilization of the country's oil and gas resources						
			**Performance Targets**			
**Programme Outcome Indicators**	**Base Year**	**Base Line**	**2023/24**	**2024/25**	**2025/26**	**2026/27**
Contribution of the Oil & Gas to GDP (%)	2020	2%	5%	6%	8%	10%
Number of Ugandans employed in the oil and gas and related industries	2020	3400	20000	30000	40000	50000
**Programme Outcome**	Increased days of Security Stock levels of refined petroleum products					
**Programme Objectives contributed to by the Intermediate Outcome**						
To improve security of supply of refined petroleum products						
			**Performance Targets**			
**Programme Outcome Indicators**	**Base Year**	**Base Line**	**2023/24**	**2024/25**	**2025/26**	**2026/27**
Number of days of Stock levels in the country	2020	1	10	12	13	15
**Programme Outcome**	High Quality Supply of Refined Petroleum products					
**Programme Objectives contributed to by the Intermediate Outcome**						
To improve security of supply of refined petroleum products						
			**Performance Targets**			
**Programme Outcome Indicators**	**Base Year**	**Base Line**	**2023/24**	**2024/25**	**2025/26**	**2026/27**
Level of quality compliance of refined petroleum products, %	2020	95	98%	99%	100%	100%

SUSTAINABLE PETROLEUM DEVELOPMENT

**Programme Outcome**	Increased private investment in the oil and gas sector						
**Programme Objectives contributed to by the Intermediate Outcome**							
To promote private investment in oil and gas industry							
	**Performance Targets**						
**Programme Outcome Indicators**	**Base Year**	**Base Line**	**2023/24**	**2024/25**	**2025/26**	**2026/27**	
FDI in the oil and gas sector (UGX Billions)	2020	300	1500	2000	4000	5000	
Number of licenses issued	2020	5	10	12	13	15	
Private sector Investment in oil and gas to GDP (%)	2020	100	90%	80%	75%	70%	
**Programme Outcome**	Increased investment in the oil and gas industry						
**Programme Objectives contributed to by the Intermediate Outcome**							
To strengthen policy, legal, regulatory and institutional framework for the oil and gas industry							
	**Performance Targets**						
**Programme Outcome Indicators**	**Base Year**	**Base Line**	**2023/24**	**2024/25**	**2025/26**	**2026/27**	
Proportion of investment in oil and gas to GDP (%)	2020	2%	5%	6%	7%	8%	
**Programme Outcome**	Sustainable management of oil and gas resources						
**Programme Objectives contributed to by the Intermediate Outcome**							
To strengthen policy, legal, regulatory and institutional framework for the oil and gas industry							
	**Performance Targets**						
**Programme Outcome Indicators**	**Base Year**	**Base Line**	**2023/24**	**2024/25**	**2025/26**	**2026/27**	
Level of compliance to Environmental standards (%)	2020	100%	100%	100%	100%	100%	
Level of compliance to Health and safety standards (%)	2020	100%	100%	100%	100%	100%	
**Programme Outcome**	Skilled local human resource employed in the oil and gas sector						
**Programme Objectives contributed to by the Intermediate Outcome**							
To strengthen policy, legal, regulatory and institutional framework for the oil and gas industry							
	**Performance Targets**						
**Programme Outcome Indicators**	**Base Year**	**Base Line**	**2023/24**	**2024/25**	**2025/26**	**2026/27**	
Number of Ugandans employed in the oil and gas and gas related industries	2020	1500	5000	6000	7000	8000	

Table P2.2: Intermediate Outcomes Indicators

**Sub-Programme Name:**	**Upstream**					
**Intermediate Outcome Indicators:**	Increased contribution of the oil and gas industry to employment					
	**Performance Targets**					
**Indicators**	**Base Year**	**Base Line**	**2023/24**	**2024/25**	**2025/26**	**2026/27**
No. of Ugandans employed as professionals in the oil and gas sector	2020	300	1000	1500	2000	3000

SUSTAINABLE PETROLEUM DEVELOPMENT

**Sub-Programme Name:**	**Upstream**					
**Intermediate Outcome Indicators:**	Increased investment in the oil & gas industry					
	**Performance Targets**					
**Indicators**	**Base Year**	**Base Line**	**2023/24**	**2024/25**	**2025/26**	**2026/27**
Industry contribution to GDP, %	2020	2%	5%	6%	8%	10%
Level of growth of investment in downstream infrastructure	2020	5%	10%	12%	14%	15%
**Intermediate Outcome Indicators:**	Increased revenue from oil and gas resources					
	**Performance Targets**					
**Indicators**	**Base Year**	**Base Line**	**2023/24**	**2024/25**	**2025/26**	**2026/27**
Increase Oil and Gas revenue	2020	2	5	6	8	10
**Sub-Programme Name:**	**Midstream**					
**Intermediate Outcome Indicators:**	Increased investment in the oil & gas industry					
	**Performance Targets**					
**Indicators**	**Base Year**	**Base Line**	**2023/24**	**2024/25**	**2025/26**	**2026/27**
Industry contribution to GDP. %	2020	2%	5%	6%	8%	10%
Level of growth of investment in downstream infrastructure	2020	5%	10%	12%	13%	15%
**Sub-Programme Name:**	**Downstream**					
**Intermediate Outcome Indicators:**	High Quality Supply of Refined Petroleum Products					
	**Performance Targets**					
**Indicators**	**Base Year**	**Base Line**	**2023/24**	**2024/25**	**2025/26**	**2026/27**
Level of quality compliance of refined petroleum products	2020	95%	98%	99%	100%	100%
**Intermediate Outcome Indicators:**	Increased days of Security Stock levels of refined petroleum products					
	**Performance Targets**					
**Indicators**	**Base Year**	**Base Line**	**2023/24**	**2024/25**	**2025/26**	**2026/27**
Number of days of Stock levels in the country	2020	5	10	12	13	15

P3: Medium Term Budget Allocations by Sub-Programme and Vote

Table P3.1: Proposed Budget Allocations and Medium Term Projections by Sub-Programme

Billion Uganda Shillings	FY2022/23	2023/24	Medium Term Projections			
	Approved Budget	Proposed Budget	2024/25	2025/26	2026/27	2027/28
01 Upstream	77.672	119.213	125.713	134.329	161.580	165.580
02 Midstream	770.683	382.492	978.688	1,313.457	967.893	532.672
03 Downstream	20.960	37.504	39.607	115.839	184.420	184.420
**Total for the Programme**	**869.316**	**539.210**	**1,144.008**	**1,563.625**	**1,313.893**	**882.671**

SUSTAINABLE PETROLEUM DEVELOPMENT

Table P3.2: Proposed Budget Allocations and Medium Term Projections by Vote

Billion Uganda Shillings	FY2022/23		2023/24	Medium Term Projections			
	Approved Budget	Spent by End Sep	Proposed Budget	2024/25	2025/26	2026/27	2027/28
006 Ministry of Foreign Affairs		0	0.695	0.709	0.851	1.148	1.148
008 Ministry of Finance, Planning and Economic Development	720.350	92.390	281.350	286.977	344.372	464.903	464.903
013 Ministry of Education and Sports		0	1.000	1.020	1.224	1.652	1.652
017 Ministry of Energy and Mineral Development	84.940	0.034	160.045	756.965	1,102.249	697.754	266.533
139 Petroleum Authority of Uganda (PAU)	63.720	6.136	94.415	96.605	112.911	145.817	145.817
150 National Environment Management Authority (NEMA)		0	0.500	0.510	0.612	0.826	0.826
154 Uganda National Bureau of Standards (UNBS)		0	0.900	0.918	1.102	1.487	1.487
506 Uganda High Commission in Tanzania, Dar es Salaam	0.305	0.076	0.305	0.305	0.305	0.305	0.305
**Total for the Programme**	**869.316**	**98.636**	**539.210**	**1,144.008**	**1,563.625**	**1,313.893**	**882.671**

P4: PROGRAMME PRIORITIES IN LINE WITH INTERVENTIONS FOR FY2023/24

Programme Priorities FY2023/24	NDP III Programme Intervention
-Capitalization of UNOC undertaken by MoFPED	Capitalize UNOC to execute its mandate as an investment arm of government in oil and gas industry.
-Conclude and implement the refinery pending agreements that include the Implementation agreement, the crude supply agreement and shareholders' agreement.<br>-Conclude the pending schedules in the EACOP agreements.	Complete the relevant oil and gas project commercial agreements
-Development of Kingfisher Development Area (KFDA) and Tilenga projects supervised.<br>-Kingfisher and Tilega projects (RAP, Development and Production activities)supervised.<br>-Field Development Reports pertaining to Tilenga and KFDA reviewed and relevant aspects updated.<br>-4 development and production work programs and budgets for 2023 reviewed and approved.<br>-3 Reservoir models, subsurface and enhanced oil recovery studies reports reviewed.<br>-Third party validation and audits of upstream policies, procedures and related documents conducted.<br>-UNOC's annual oil and gas reserves assessment and report compiled, independent audits of UNOC's annual oil and gas resources conducted.	Construct the Central Processing Facilities (CPFs) for Tilenga and Kingfisher projects;

SUSTAINABLE PETROLEUM DEVELOPMENT

Programme Priorities FY2023/24	NDP III Programme Intervention
-Develop a promotional and investment strategy and plan for the oil and gas industry.<br>-Commence preparations for the 11th East African Petroleum Conference (EAPCE' 25)<br>-Promote the country's petroleum potential at foru (4) international conferences.	Develop and implement a marketing and promotional strategy for oil and gas projects.
Continue to pursue the feasible financing mechanisms.	Develop and implement a sustainable financing strategy
-Implementation of the Oil and Gas Disaster contingency and preparedness plan.	Develop and implement an oil and gas disaster preparedness and contingency plan;
Environmental and social management plan implemented.	Develop and implement environmental and social management plan
Develop and implement the environmental QHSSE standards	Develop and implement oil and gas QHSSSE systems and standards;
-Develop 5 codes of practice and standards for rail wagons for LPG transportation, water vessels for petroleum products.<br>-Terms of Reference for Regulations of refined products on the lake prepared.<br>-Improve operations of the National Petroleum Information System (NPIS) through system upgrade.	Develop operations standards of transportation of petroleum products on Lake and Rail
-Jinja Storage Terminal 9JST) restocked and managed.<br>-JST equipped with specialized engineering inspection tools.<br>-JST equipment maintained.<br>-Oil Jetty and a 1.5km, 8 inches dual refined products pipeline constructed.<br>-Bulk Trading scaled up to importation of 15 million liters of petroleum products.<br>-Undertake marketing of sales for the stocked volumes.	Develop strategic regional storage terminals for petroleum products
-Develop 10 standards and codes of practice for Base oils, lubricants, LPG product and Accessories.<br>-Monitor petroleum quality compliance in at least 80% of all retail stations in Uganda.<br>-Undertake LPG Central Storage Designs and ESIA.<br>-LPG cylinder kits procured and distributed	Development of standards for storage infrastructure and other facilities
-Undertake a detailed feasibility study and preliminary design and route studies for the pipeline.<br>-Conclude the Inter-Governmental Agreement between Uganda and Tanzania.	Development of the Natural Gas Pipeline from Tanzania to Uganda to support EACOP, Iron Ore Industry and other industrial and domestic uses
-Environmental and social annual report of the oil and gas industry produced.<br>-Quarterly environmental compliance monitoring reports produced.<br>Simulation exercise on emergency preparedness and response undertaken.<br>-Training, drills, and exercises on oil spill preparedness and response undertaken.<br>-ESIA for UNOC led projects undertaken.<br>-QHSSE Management system secured and deployed.	Establish QHSSSE governance and assurance framework;

SUSTAINABLE PETROLEUM DEVELOPMENT

Programme Priorities FY2023/24	NDP III Programme Intervention
-Localize international accreditation to the local training centers	Fast-Track Skilling (e.g., apprenticeship), Training and International Accreditation of Ugandans including women, youth and PWDs for employment and service provision in the development/phase of the oil and gas sector.
Communication strategy for the oil and gas industry implemented.<br>-4 branding and awareness initiatives executed.<br>-8 stakeholder engagements undertaken.<br>-8 engagements with CSOs conducted.<br>-Awareness and communication strategy of LPG usage conducted country wide.	Implement a communication strategy to deal with public anxiety and managing expectations;
Implement the National Content Policy recommendations to ensure participation of Ugandans in the Oil and Gas industry with emphasis on the gender and equity matters.	Operationalize the National Content policy to enhance local Content and participation of nationals including women and youth in the oil and gas sector
-Finalize the development of the agricultural development strategy for farmers along the EACOP and commence its implementation.<br>-Establish and operationalize the industry enhancement center.<br>-Enact a law establishing the Local Content Fund Develoment	Provide SMEs both technical (training) and financial support to enhance their participation in tendering and of delivery of contracts. (Direct and indirect participants in the oil and gas value chain)
-National Petroleum Policy (NPP) development completed.<br>-NPP implementation plan developed.<br>-NPP M&E framework developed.<br>-Decommissioning and Fiscal Metering regulations developed.<br>-Decommissioning strategy developed.<br>-Five (5) standards and codes for upstream operations developed.<br>-Conclude and update the midstream tariff, third party, decommissioning and metering regulations.<br>-	Review, update relevant policies, and harmonize conflicting laws and regulations;
-Produce and publish the country's Annual petroleum resources report.<br>-Program represented t Uganda's EITI Multistakeholder Group (MSG)	Strengthen governance and transparency in the oil and gas Sector.
-Establishment of Petroleum Geo-science Laboratory.<br>-Establish a National Petroleum Data Repository.<br>-A modern core store center equipped.<br>-1 Real Time Operations/Monitoring Center (RTOC/RTMC) set up and operationalized.<br>-A functional offsite data backup and Disaster Recovery System (DRS) set up.<br>-Data management hardware and software applications for subsurface and surface facilities acquired and integrated.<br>-Refinery construction completed.<br>-Feasibility studies for Integrated complex completed for fertilizers and other petrochemicals completed.	Undertake construction and operationalisation of infrastructure projects in the Albertine Region to ease movement of goods, labour and provision of services

SUSTAINABLE PETROLEUM DEVELOPMENT

Programme Priorities FY2023/24	NDP III Programme Intervention
-Conclude data acquisition in the Moroto-Kadam basin.<br>-Commence data acquisition in the Kyoga basin.<br>Two field development plans reviewed and the relevant aspects updated.<br>-Petroleum Resource assessment undertaken and annual resources reported.<br>-Implementation of the licensing strategy for the 3rd Licensing round.<br>-3 submitted work programs and budgets under exploration licenses monitored.<br>-Surface geological field studies undertaken.	Undertake further exploration and ventures of the Albertine Graben

P5: GENDER AND EQUITY ISSUES AND INTERVENTIONS FOR FY2023/24

The Programme aims to attain equitable value from the petroleum resources and spur economic development in a timely and sustainable manner and transform the well being of all Ugandans with consideration of the youth, women, men as well as the disabled.
The program focuses on crating gainful employment of all Ugandans and creating a balance between women, men and the youth.

MANUFACTURING

Foreword

The Budget Framework Paper for FY 2023-24 was prepared in pursuance of the programme mandate, formulation of policies that promote and ensure expansion and diversification of trade, cooperatives and environmentally sustainable industrialization which is in line with full monetization of Uganda's economy through commercial agriculture, industrialization, expanding and broadening services, digital transformation and market access as the theme for the 2023-24 Budget Strategy.

This Manufacturing Budget Framework Paper for FY 2023-24 is informed by and is responsive to the Third National Development Plan, Sustainable Development Goals, undertakings agreed during the Programme Review, the Presidential Directives and National Resistance Movement Manifesto Commitments. The Programme Budget Framework Paper for Financial Year 2023-24 pays attention to harnessing the existing synergies within the Programme.

The Programme aims at the following key priorities,

1. Review and formulate trade related polices to refocus on supporting exports.
2. Sensitize farmers/producers on trade procedures and standards with emphasis on exporting and supporting the refurbishment of storage facilities.
3. Improve quality and standard concerns by expanding the services of Uganda National Bureau Standards.
4. Establishment of Border Export Zones at key strategic border points to increase cross border trade.
5. Enhance value addition and industrialization.
6. Promotion of Cooperative Movement by mobilizing collective resources through cooperatives and strengthen supervisory and management of Cooperatives through District Commercial Extension Services etc.

I am once again happy to present to you the Budget Framework Paper for Manufacturing Programme for FY 2023-24. I look forward to your continued cooperation and support as we continue striving to improve service delivery for the development of Uganda.

For God and My Country

Geraldine Ssali

Permanent Secretary

MANUFACTURING

Abbrevations and Acronyms

ACRONYM	ACRONYM NAME
AfCFTA	African Continental Free Trade Area
AGOA	African Growth Opportunities Act
CFTA	Continental Free Trade Area
COMESA	Common Market for Eastern and Southern Africa
EPZs	Export Processing Zones
FTA	Free Trade Area
G&E	Gender and Equity
MSME	Micro, Small and Medium Enterprise
MTAC	Management Training and Advisory Centre
SADC	Southern African Development Community
SME	Small and Medium Enterprises
TEXDA	Textile Development Agency
TICAD	Tokyo International Conference of African Development
UMA	Uganda Manufacturers Association
UNCE	Uganda National Commodity Exchange
UNIDO	United Nations Industrial Development Organisation
UWRSA	Uganda Warehouse Receipt System Authority
WTO	World Trade Organisation

MANUFACTURING

P1: PROGRAMME OVERVIEW

Snapshot of Medium Term Budget Allocations

Table P1.1 Overview of Programme Expenditure and Medium Term Allocations (Ush Billion)

Billion Uganda Shillings		FY2022/23		FY2023/24	MTEF Budget Projections			
		Approved Budget	Spent by End Sep	Proposed Budget	2024/25	2025/26	2026/27	2027/28
Recurrent	Wage	2.201	0.497	2.026	2.127	2.340	2.574	2.574
	Non Wage	106.054	4.985	32.533	33.184	39.821	53.758	53.758
Devt.	GoU	89.415	0.000	0.226	0.226	0.271	0.379	0.379
	ExtFin	221.251	0.000	233.615	0.000	0.000	0.000	0.000
	**GoU Total**	**197.670**	**5.483**	**34.785**	**35.537**	**42.431**	**56.711**	**56.711**
**Total GoU+Ext Fin (MTEF)**		**418.920**	**5.483**	**268.400**	**35.537**	**42.431**	**56.711**	**56.711**
	A.I.A	0.000	0.000	0.000	0.000	0.000	0.000	0.000
	**Grand Total**	**418.920**	**5.483**	**268.400**	**35.537**	**42.431**	**56.711**	**56.711**

Programme Strategy and linkage to the National Development Plan

Manufacturing programme strives to formulate policies that promote and ensure expansion and diversification of trade, cooperatives and environmentally sustainable industrialization which is in line with full monetization of Uganda's economy through commercial agriculture, industrialization, expanding and broadening services, digital transformation and market access as the theme for the 2023-24 Budget Strategy.

The Programme in line with Third National Development Plan (NDP 111) aims at ensuring the following key priorities, review and formulate trade related polices to refocus on supporting exports, sensitize farmers/producers on trade procedures and standards with emphasis on exporting and supporting the refurbishment of storage facilities, improve quality and standard concerns by expanding the services of Uganda National Bureau Standards, establishment of Border Export Zones at key strategic border points to increase cross border trade, enhance value addition and industrialization and promotion of Cooperative Movement by mobilizing collective resources through cooperatives and strengthen supervisory and management of Cooperatives through District Commercial Extension Services etc.

P2: Highlights Of Programme Projected Performance

Table P2.1 Programme Outcomes Indicators

**Programme Outcome**	Increased number of jobs in the economy					
**Programme Objectives contributed to by the Intermediate Outcome**						
Develop the requisite infrastructure to support manufacturing in line with Uganda's planned growth corridors (triangle)						
			Performance Targets			
**Programme Outcome Indicators**	**Base Year**	**Base Line**	**2023/24**	**2024/25**	**2025/26**	**2026/27**
Contribution of manufacturing to industrial GDP (%)	2020	15.5%	22%	23%	25%	27%
Industrial sector contribution to GDP (%)	2020	27.1%	30%	31%	32%	33%

MANUFACTURING

Table P2.2: Intermediate Outcomes Indicators

Sub-Programme Name:	Industrial and Technological Development					
**Intermediate Outcome Indicators:**	Enhanced industrial facilitation, promotion and cluster					
	Performance Targets					
**Indicators**	**Base Year**	**Base Line**	**2023/24**	**2024/25**	**2025/26**	**2026/27**
Number of standards and guidelines for industrial parks developed or updated	2020	0	1	1	1	1

P3: Medium Term Budget Allocations by Sub-Programme and Vote

Table P3.1: Proposed Budget Allocations and Medium Term Projections by Sub-Programme

Billion Uganda Shillings	FY2022/23	2023/24	Medium Term Projections			
	Approved Budget	Proposed Budget	2024/25	2025/26	2026/27	2027/28
01 Industrial and Technological Development	333.525	252.311	20.432	23.928	33.628	35.731
03 Enabling Environment	82.628	16.001	15.025	18.403	22.938	20.834
**Total for the Programme**	**418.920**	**268.400**	**35.537**	**42.431**	**56.711**	**56.711**

Table P3.2: Proposed Budget Allocations and Medium Term Projections by Vote

Billion Uganda Shillings	FY2022/23		2023/24	Medium Term Projections			
	Approved Budget	Spent by End Sep	Proposed Budget	2024/25	2025/26	2026/27	2027/28
015 Ministry of Trade, Industry and Co-operatives	124.987	4.087	34.697	35.447	42.324	56.566	56.566
138 Uganda Investment Authority (UIA)	287.611	0	233.615	0.000	0.000	0.000	0.000
154 Uganda National Bureau of Standards (UNBS)	4.234	1.055	0.088	0.090	0.108	0.145	0.145
**Total for the Programme**	**418.920**	**5.483**	**268.400**	**35.537**	**42.431**	**56.711**	**56.711**

P4: PROGRAMME PRIORITIES IN LINE WITH INTERVENTIONS FOR FY2023/24

Programme Priorities FY2023/24	NDP III Programme Intervention
Consumer Protection Bill Gazetted and Printed. Implementation of the Competition and Consumer Protection Policy and Law.	Enforce the laws on counterfeits and poor-quality products
Prepare and consulte the AfCFTA, CFTA Tripartite (EACCOMESA- SADC) stakeholder with the aim of harmonizing their various development levels including its implementation strategy.	Sign bilateral agreements to guarantee market access

MANUFACTURING

Programme Priorities FY2023/24	NDP III Programme Intervention
Amendment of Regulations for the Industrial Licensing Act.<br>Development of Regulations to the Accreditation Services Act.<br>Implementation of the National Industrial Policy.	Upgrade industries to make them sustainable, with increased resource-use efficiency and greater adoption of clean and environmentally sound technologies and industrial processes

P5: GENDER AND EQUITY ISSUES AND INTERVENTIONS FOR FY2023/24

As an issue of concern, there is need to Mainstream Gender and provide Equal Opportunities to all men, women, youth, elderly persons and PWDs in both rural and urban areas across the Country and in all activities implemented by the MDAs in the Manufacturing Programme.

The following interventions have been planned to be implemented in response to the above issue,
a) Training of staff in Gender issues and Gender awareness creation for the Ministry Staff.
b) Training MDAs staff in Gender and Equity Budgeting.
c) Data collection across all MDAs to be broken into women men youth.
d) Ensure Gender and Equity mainstreaming in all activities of all MDAs within the Manufacturing Programme in collaboration with EOC and MoGLSD.

TOURISM DEVELOPMENT

Foreword

In line with Section 9 (13) of the Public Financial Management (PFM) Act, 2015(Amended) and the 1st Budget Call Circular (BCC1) issued by the Permanent Secretary/Secretary to the Treasury, I am pleased to present the Budget Framework Paper for the Financial Year 2023/24 for the Tourism Development Programme.

The Budget Framework Paper preparation process involved consultation with relevant stake holders and took into consideration the Governments commitments towards balanced development as well as gender and equity responsiveness.

The GOU MTEF totals to Ushs. 89.293 billion and distributed to the various MDAs through two Votes namely; Vote 022 Ministry of Tourism, Wildlife and Antiquities-UShs. 85.019 billion; and Vote 117 Uganda Tourism Board-UShs. 4.300 billion. The Ushs 85.019 billion under Vote 022 Ministry of Tourism, Wildlife and Antiquities covers the following Subvention Agencies:
Uganda Wildlife Authority(UWA)-UShs. 21.350 billion
Uganda Wildlife Conservation Education Centre (UWEC) - UShs. 1.900 billion
Uganda Hotel and Tourism Training Institute (UHTTI)- UShs. 1.246 billion
Uganda Wildlife Research and Training Institute (UWRTI)- UShs. 0.697 billion

This proposal of Ushs 89.293 billion is way below the FY 2022/23 approved budget of Ushs. 194,677 billion.

The deficit will adversely affect the implementation of the various programmes and attainment of NDP III targets as well as recovery efforts of the Tourism Development Programme.
It is very critical that the Programme is availed with additional UShs. 211.307 billion to enable it to implement the key interventions enumerated in the Budget Strategy for the FY 2023/24 and the NDP III.

There is a need for a special and dedicated focus of the National Budget towards the realization of accelerated development of Tourism. Scaling up the funding will increase the velocity of realization of the return on investment, not only in Tourism Development but for all sectors of the economy. Tourism will generate and fund other government priorities.

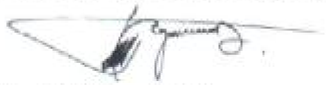

Tom R. Butime (MP)

MINISTER OF TOURISM, WILDLIFE AND ANTIQUITIES

TOURISM DEVELOPMENT

Abbrevations and Acronyms

ACRONYM	ACRONYM NAME
AUTO	Association of Uganda Tour Operators
CAA	Civil Aviation Authority
CITES	Convention of International Trade in Endangered Species of wild fauna and flora
CoP-	Conference of Parties
IAS	Invasive Alien Species
IGP	Inspector General of Police
KTWR	Katonga Wildlife Reserve
KVNP	Kidepo Valley National Park
MDR	Market Destination Representative Firms
MECA	Mount Elgon Conservation Area
MFPA	Murchison Falls Protected Area
MICE	Meetings, Incentives, Conventions, and Exhibitions.
MTWA	Ministry of Tourism, Wildlife and Antiquities
TORs	Terms of Reference
TSA	Tourism Satellite Account
UCOTA	Uganda Community Tourism Association
UNRA	Uganda National Roads Authority
UWEC	Uganda Wildlife Conservation Education Centre

TOURISM DEVELOPMENT

P1: PROGRAMME OVERVIEW

Snapshot of Medium Term Budget Allocations

Table P1.1 Overview of Programme Expenditure and Medium Term Allocations (Ush Billion)

Billion Uganda Shillings		FY2022/23		FY2023/24	MTEF Budget Projections			
		Approved Budget	Spent by End Sep	Proposed Budget	2024/25	2025/26	2026/27	2027/28
Recurrent	Wage	4.226	0.897	4.226	4.438	4.881	5.369	5.369
	Non Wage	178.511	41.781	32.330	32.977	39.572	53.422	53.422
Devt.	GoU	11.940	0.000	52.740	52.740	63.288	88.603	88.603
	ExtFin	0.000	0.000	0.000	0.000	0.000	0.000	0.000
	**GoU Total**	**194.677**	**42.678**	**89.296**	**90.154**	**107.741**	**147.395**	**147.395**
	**Total GoU+Ext Fin (MTEF)**	**194.677**	**42.678**	**89.296**	**90.154**	**107.741**	**147.395**	**147.395**
	A.I.A	0.000	0.000	0.000	0.000	0.000	0.000	0.000
	**Grand Total**	**194.677**	**42.678**	**89.296**	**90.154**	**107.741**	**147.395**	**147.395**

Programme Strategy and linkage to the National Development Plan

The goal of the Ministry over the next five years is to increase Uganda's attractiveness as a preferred tourist destination. The following strategic objectives guide the implementation process:

a. Objective 1: Promote domestic and inbound tourism;
b. Objective 2: To develop a pool of skilled personnel along the tourism value chain and ensure decent working conditions;
c. Objective 3: Develop, conserve and diversify tourism produts;
d. Objective 4: Increase the stock and quality of tourism infrastructure;
e. Objective 5: Enhance regulation, coordination and management of the tourism Program;

P2: Highlights Of Programme Projected Performance

Table P2.1 Programme Outcomes Indicators

**Programme Outcome**	Increased employment/ jobs created along the tourism value chain					
**Programme Objectives contributed to by the Intermediate Outcome**						
Develop a pool of skilled personnel along the tourism value chain and ensure decent working conditions						
			Performance Targets			
**Programme Outcome Indicators**	**Base Year**	**Base Line**	**2023/24**	**2024/25**	**2025/26**	**2026/27**
Contribution of tourism to total employment (%)	2019	5.8%	8.0%	8.5%	8.5%	9.0%
Number of people directly employed along the tourism value chain	2019	200,000	320000	350000	400000	450000
Visitor satisfaction (%)	2019	73	79%	79%	80%	80%

TOURISM DEVELOPMENT

**Programme Outcome**	Increased employment/ jobs created along the tourism value chain					
**Programme Objectives contributed to by the Intermediate Outcome**						
Develop, conserve and diversify tourism products						
			**Performance Targets**			
**Programme Outcome Indicators**	**Base Year**	**Base Line**	**2023/24**	**2024/25**	**2025/26**	**2026/27**
Contribution of tourism to total employment (%)	2019	5.8	8.0%	8.5	8.5	9.0
Number of people directly employed along the tourism value chain	2019	200000	320000	350000	400000	450000
Visitor satisfaction (%)	2019	73	79%	79	80	80
**Programme Outcome**	Improved Wildlife Ecosystems					
**Programme Objectives contributed to by the Intermediate Outcome**						
Develop, conserve and diversify tourism products						
			**Performance Targets**			
**Programme Outcome Indicators**	**Base Year**	**Base Line**	**2023/24**	**2024/25**	**2025/26**	**2026/27**
Incidences of human Wildlife Conflicts (number)	2019	4073	5779	5201.1	4681	4213
Number of visitors to Museums and cultural sites	2019	55,426	167821	302,078	320000	335000
Number of visitors to National Parks and UWEC	2019	707,259	933138	1,007,789	1,087,789	1,182,789
Population of Antelopes	2019	127196	168184	173230	200000	250000
Population of Elephants	2019	5739	7588	7816	7916	7916
Population of Lions	2019	493	652	671	692	692
Population of Mountain Gorillas	2019	459	586	644	650	650
**Programme Outcome**	Improved compliance to Tourism service standards					
**Programme Objectives contributed to by the Intermediate Outcome**						
Enhance regulation, coordination and management of the tourism						
			**Performance Targets**			
**Programme Outcome Indicators**	**Base Year**	**Base Line**	**2023/24**	**2024/25**	**2025/26**	**2026/27**
Level of Compliance to Tourism Service Standards (% enterprises)	2019	35	55%	55	60	60
Visitor satisfaction (%)	2019	73%	79%	79%	80%	80%
**Programme Outcome**	Improved accessibility to tourism goods and services					
**Programme Objectives contributed to by the Intermediate Outcome**						
Increase the stock and quality of tourism infrastructure						
			**Performance Targets**			
**Programme Outcome Indicators**	**Base Year**	**Base Line**	**2023/24**	**2024/25**	**2025/26**	**2026/27**
Accommodation Capacity (No. of rooms)[3]	2019	133,191	177278	195005	195005	200000
Length of stay/ overnights in all types of accommodation	2019	8.3	9	9.1	9.3	9.5
Proportion of leisure to total tourists (%)	2019	19.3	18%	20.1	22	24

TOURISM DEVELOPMENT

Programme Outcome	Improved accessibility to tourism goods and services					
**Programme Objectives contributed to by the Intermediate Outcome**						
Increase the stock and quality of tourism infrastructure						
	**Performance Targets**					
**Programme Outcome Indicators**	**Base Year**	**Base Line**	**2023/24**	**2024/25**	**2025/26**	**2026/27**
Tourist accommodation capacity (No. of beds)	2019	1129	1164	1199	1234	1269
**Programme Outcome**	Increased tourism receipts					
**Programme Objectives contributed to by the Intermediate Outcome**						
Promote domestic and inbound tourism						
	**Performance Targets**					
**Programme Outcome Indicators**	**Base Year**	**Base Line**	**2023/24**	**2024/25**	**2025/26**	**2026/27**
Annual Foreign exchange earnings (USD - Bn)	2019	1.6	1.774	1.862	1.862	1.862
Average annual Hotel occupancy rate (room occupancy rate, %)	2019	51.9	45%	50	52	55
Average Inbound tourism revenues per leisure tourist (USD)	2019	1052	1431	1500	1500	1500
Contribution of Tourism to GDP (%)	2019	5.6	8.1%	8.5	8.5	9.0
Number of direct flight routes to Europe and Asia	2019	6	14	15	15	15
Number of International Tourist arrivals from the U.S., Europe and China[1]	2019	212,603	281760	301483	301483	301483
Number of Ugandans visiting key tourist attractions[2]	2019	434,000	568885	651317	850000	1,000,435
Tourism arrivals	2019	1,542,620	1200000	1440000	1500000	1650000

Table P2.2: Intermediate Outcomes Indicators

Sub-Programme Name:	Marketing and Promotion					
**Intermediate Outcome Indicators:**	Increased tourism arrivals					
	**Performance Targets**					
**Indicators**	**Base Year**	**Base Line**	**2023/24**	**2024/25**	**2025/26**	**2026/27**
No. of domestic visitors to Uganda's key tourist destinations	2019	434,000	568885	651317	850000	1000435
No. of inbound visitor arrivals	2019	1,542,620	1200000	1440000	1500000	1650000
**Sub-Programme Name:**	**Infrastructure, Product Development and Conservation**					
**Intermediate Outcome Indicators:**	Improved Heritage Conservation and Tourism Growth					
	**Performance Targets**					
**Indicators**	**Base Year**	**Base Line**	**2023/24**	**2024/25**	**2025/26**	**2026/27**
Population of Antelopes	2019	127196	168184	173230	200000	250000
Population of Elephants	2019	5739	7588	7816	7916	7916
Population of Lions	2019	493	652	671	692	692
Population of Mountain Gorillas	2019	459	586	644	650	650

TOURISM DEVELOPMENT

Sub-Programme Name:	Infrastructure, Product Development and Conservation					
Intermediate Outcome Indicators:	Increased private investment in tourism infrastructure					
	Performance Targets					
Indicators	Base Year	Base Line	2023/24	2024/25	2025/26	2026/27
Accommodation Capacity (No. of rooms)	2019	133,191	177278	195005	195005	200000
Proportion of leisure to total tourists, %	2019	19.3%	18%	20.1%	22%	24%
Sub-Programme Name:	Regulation and Skills Development					
Intermediate Outcome Indicators:	Personnel trained					
	Performance Targets					
Indicators	Base Year	Base Line	2023/24	2024/25	2025/26	2026/27
UHTTI transformed into a centre of excellence	2019	10%	60%	100%	100%	100%
Intermediate Outcome Indicators:	Sound management of the tourism resources					
	Performance Targets					
Indicators	Base Year	Base Line	2023/24	2024/25	2025/26	2026/27
Level of compliance of planning and budgeting instruments to NDPIII	2019	35%	55%	55%	60%	60%
Level of tourist satisfaction (%)	2019	73	79%	79%	80%	80%

P3: Medium Term Budget Allocations by Sub-Programme and Vote

Table P3.1: Proposed Budget Allocations and Medium Term Projections by Sub-Programme

Billion Uganda Shillings	FY2022/23	2023/24	Medium Term Projections			
	Approved Budget	Proposed Budget	2024/25	2025/26	2026/27	2027/28
01 Marketing and Promotion	23.993	4.665	4.711	30.611	6.460	6.460
02 Infrastructure, Product Development and Conservation	139.067	74.625	74.625	49.325	74.625	74.625
03 Regulation and Skills Development	27.296	10.006	10.818	27.805	66.310	66.310
**Total for the Programme**	**194.677**	**89.296**	**90.154**	**107.741**	**147.395**	**147.395**

Table P3.2: Proposed Budget Allocations and Medium Term Projections by Vote

Billion Uganda Shillings	FY2022/23		2023/24	Medium Term Projections			
	Approved Budget	Spent by End Sep	Proposed Budget	2024/25	2025/26	2026/27	2027/28
022 Ministry of Tourism, Wildlife and Antiquities	166.144	**41.345**	84.991	85.709	102.602	140.986	140.986
117 Uganda Tourism Board (UTB)	23.841	**0.762**	4.305	4.445	5.139	6.408	6.408
**Total for the Programme**	**194.677**	**42.678**	**89.296**	**90.154**	**107.741**	**147.395**	**147.395**

TOURISM DEVELOPMENT

P4: PROGRAMME PRIORITIES IN LINE WITH INTERVENTIONS FOR FY2023/24

Programme Priorities FY2023/24	NDP III Programme Intervention
Conduct 2 tourism research studies and surveys for tourism data collection and strategy development	Develop a more robust public/private sector system to collect and analyse information on the industry in a timely fashion. In particular, establish partnerships with domestic, regional and international airlines/carriers.
An online Tourism Information Management System (TIMS) developed, validated and launched.	Develop a Tourism information Management System
Improved Rwenzori mountaineering infrastructure and increase the number of visitors to Rwenzori Mountain National Park from 5,146 in 2018 to 15,000 by the year 2026: The Rwenzori John Matte tourist camp developed with cold-proof facilities including 40 pax tourist accommodation, 60 pax porters and guides accommodation, rescue and first aid block; 2 dining structures, 2 kitchens, power and water supply and walk ways.<br>The Rwenzori Bujuku tourist camp developed with cold-proof facilities including 40 pax tourist accommodation, 60 pax porters and guides accommodation, rescue and first aid block; 2 dining structures, 2 kitchens, power and water supply and walk ways.<br>Elena tourist camp development completed along the Rwenzori Central Circuit.<br>A total of 1,000 meters of Boardwalks and climbing ladders installed long the Rwenzori Kilembe Trail.<br>Capacity building conducted for Rwenzori stakeholders, service providers and training community associations including 300 porters and guides.	Develop new tourist attraction sites profiled by region to include new products such as: Community tourism; Adventure tourism further enhanced by developing hiking, climbing and cable cars in the Rwenzori Mountains; Water-based (marine) tourism; e.g. from Semuliki National Park to East Madi wildlife reserve through Lake Albert, Semuliki river and River Nile; MICE; Agro-tourism.

TOURISM DEVELOPMENT

Programme Priorities FY2023/24	NDP III Programme Intervention
Fort Portal Regional Museum and handcraft center established and added onto tourism products along the western tourism circuit. Will support handicraft and souvenir industry through the provision of training facilities workshops and selling platform. Kikorongo Equator monument completed.<br><br>Landscaping, Pavers planters roadmaking and painting. Karamoja Museum and handicraft centre reconstructed and equipped. This will add on the tourism products along the Karamoja tourism circuit and will in addition enhance the handicraft and souvenir industry training facilities workshops and market access. Napak Open Air Museum interpretation centred established and equipped. This will add on the tourism products along the Karamoja tourism circuit and enhance tourism activity and benefits for the host communities. Kibiro community and interpretation center constructed in an effort to conserve and develop Kibiro salt village into a viable cultural tourism product that economically transforms the host communities.<br><br>Cultural heritage sites branded and marketed through production and dissemination of video documentary and 10 000 brochures.<br><br>Kabalega and Mwanga cultural heritage sites in Dokolo appraised for upgrade and development. Rock Art file completed through research and archaeological excavations of sites and submitted to UNESCO in an effort to nominate Uganda s Rock art sites into the World Heritage List.	Diversify tourism products (eg cultural) and map potential across the country including conducting hazard risk and vulnerability mapping for tourism areas

TOURISM DEVELOPMENT

Programme Priorities FY2023/24	NDP III Programme Intervention
Classify/grade 120 tourism facilities.	Establish quality marks/standards for grading of tourism-related facilities such as accommodation, attractions, beaches, restaurants and travel
Build capacity of actors along the tourism value chain in quality assurance of tourism service standards	Nurture local hospitality sector enterprises for participation in local, regional and global tourism value chains.
Roll out the destination brand in the domestic and global key source markets for increased competitiveness and attractiveness in the global picture.<br><br>Train 250 Ugandan Embassies and Mission staff in tourism marketing and handling.<br><br>Undertake digital destination marketing through social media marketing, mobile marketing, online PR, etc.<br><br>Bid for 5 international Meetings, Conferences and Events to be hosted in Uganda.<br><br>Undertake domestic tourism marketing promotions.<br><br>Attend 3 international leisure and MICE expos for increased destination awareness and business linkage creation.<br><br>Develop and profile two new tourism products.<br><br>Develop two regional tourism product portfolios	Review and implement a national tourism marketing strategy targeting both elite and mass tourism segments by:
Register and train 370 tour and travel agents<br>License 300 tour guides<br>Register and inspect 1,000 accommodation and restaurant facilities	Strengthen inspection and enforcement of service standards for tourism facilities and tour operators

TOURISM DEVELOPMENT

Programme Priorities FY2023/24	NDP III Programme Intervention
A modern pier established at the Source of the Nile including a dock for 19 boats a viewing bridge 2 water fountains outdoor restaurant sanitary vehicle parking area, relaxing gardens staff station and offices security station and waiting lobbies.<br>One observation deck constructed 500 meters of modern walkways and trails established and safety equipment handrails and cliff barriers at the banks of the river installed covering 1km. Zipline infrastructure developed connecting the eastern to the western banks of the river. A total of 100 Solar lights, hotspot wifi infrastructure 10 resting shades and 10 garbage collection pits 8 modern directional and 10 informational signage installed at the Source of the Nile.<br>An access road of 1 km upgraded to facilitate access to the Source of the Nile. Tourism service providers and other stakeholders supported with capacity building on quality standards control tourist handling, safety and security, products development including handicrafts and souvenirs.<br><br>Resettlement action plan for Source of the Nile finalized.	Upgrade, maintain and redevelop existing tourist attraction sites profiled by region to include new products like dark tourism, culinary tourism, adventure tourism, wellness 'tourism, war tourism

P5: GENDER AND EQUITY ISSUES AND INTERVENTIONS FOR FY2023/24

Human Wildlife Conflict:
Increased patrol of the 22 Wildlife Protected Area boundaries to minimize illegal activities and entry. 646kms of boundary to be maintained.
85 kms of electric fence constructed and operationalized in Queen and Murchison falls National Parks. Communities in Bwindi supported to establish a buffer of 800 acres of tea.
A total of 36 Ranger posts established and maintained in bwindi, Mgahinga, Queen, Kibaale, Rwenzori, Semliki, Tooro-Semliki, and Kabwoya wildlife.
Eight (08) Fire observation towers established and operationalised in Wildlife PAs.
A total of 4,090 hectares cleared of invasive species (MGNP, KNP, QENP, BINP, MFNP and the Reserves) in an effort to restore pasture lands for grazers.
Wildlife host communities (40 groups) engaged and Collaborative Resource Management agreements signed and livelihood projects done.

TOURISM DEVELOPMENT

Desegregation of tourism information by gender:
The data capture forms at tourism sites such as regional museums and National Parks have been modified to capture gender attributes such as sex and age. The analysis and reporting provide the disaggregation.

Unequal access to employment opportunities:
A total of 500 students will be enrolled (including 250 new ones) at UHTTI and 280 students (including 120 new ones) at UWRTI. Students from underserved regions especially Busoga, Teso, Bukedi and Karamoja shall be the target. In partnership with other actors such as DIT, certification will be conducted for skilled individuals that don't have formal education to enable them participate in more decent employment.

Limited quality in private tourism training institutions:
Support supervision conducted to improve quality of trainings.

Equal opportunity for recruitment:
Accommodation facilities maintained (with capacity to accommodate 1,381 staff) in Protected Areas to provide conducive environment for female Rangers.

Lack of adequately customized (language, region) tourism information on product offerings, investment opportunities, service providers:
UTB tourism promotional materials translated in local and select foreign languages and made accessible in print, digital and broadcast media.

Inadequate benefits derived from conservation by host communities:
Revenue sharing 20% of National Park entry fees shared with host communities. Communities supported with grants (US$200,000) towards the development of community tourism products.

Un/under developed tourism products:
Eight tourism products developed in FY 2023/24 including Rwenzori Mountains in Kasese, the Source of the Nile in Jinja, Fort Portal Regional Museum and handcraft center, Kikorongo Equator monument, Karamoja Museum and handicraft centre, Napak Open Air Museum interpretation centre established, and Kibiro community and interpretation center.
UWA tourism products improved through construction and equipment of 5 visitor centres in Bwindi, Murchison falls, Kibaale, Semliki and Rwenzori;
Maintenance of a picnic site at the waterfalls in Buhoma, canopy walk in Sebitoli, as well as camping sites, 3-peak volcano hiking trail.

These products will add added onto tourism products along their respective host tourism circuit and will add on both domestic and international tourist experience.

Limited accessibility to tourism sites:
Ladders (1,000) and 1,700 metres of board walks constructed in Sabinyo, Muhavura, Gahinga and Kirumia in Mgahinga and Semliki National parks.
Six (6) entrance gates and associated facilities constructed and operated at the National Parks/Wildlife Reserves of Bwindi, Mgahinga, Kibaale, Semliki, Queen and Murchison.
A modern pier established at the Source of the Nile including a dock for 19 boats, a viewing bridge, 2 water fountains, outdoor restaurant, sanitary, vehicle parking area, relaxing gardens, staff station and offices, security station and waiting lobbies. One observation deck constructed, 500 meters of modern walkways and trails established and safety equipment (hand rails and cliff barriers) at the banks of the river installed covering 1km.
Zip line infrastructure developed connecting the eastern to the western banks of the river.

TOURISM DEVELOPMENT

[illegible]

A total of 100 Solar lights, hotspot wifi infrastructure, 10 resting shades and 10 garbage collection pits, 8 modern directional and 10 informational signage installed.

An access road (1 km) upgraded to facilitate access to the Source of the Nile.

Tourism service providers and other stakeholders supported with capacity building on quality standards control, tourist handling, safety and security, products development (including handicrafts and souvenirs), etc.

The Rwenzori John Matte and Bujuku tourist camps developed with cold-proof facilities each including 40 pax tourist accommodation, 60 pax porters and guides accommodation, rescue and first aid block; 2 dining structures, 2 kitchens, power and water supply and walk ways.

A total of 1,000 meters of Boardwalks and climbing ladders installed long the Rwenzori Kilembe trail

NATURAL RESOURCES, ENVIRONMENT, CLIMATE CHANGE, LAND AND WATER

Foreword

The Programme Budget Framework Paper FY 2023-24 has been prepared in line with the theme of the budget FY 2023-24 "Full Monetization of Uganda's Economy through Commercial Agriculture, Industrialization, Expanding and Broadening Services, Digital Transformation and Market Access". This has been by done through addressing the critical issues on environmental protection, restoration of wetlands, forestry management, water resource protection and sustainable land management.

The objectives of the programme are to; (i). Ensure availability of adequate and reliable quality fresh water resources for all uses. (ii). Increase forest tree and wetland coverage restore bare hills and protect mountainous areas and rangelands (iii). Strengthen land use and management. (iv). Maintain and or restore a clean healthy and productive environment (v). Promote inclusive climate resilient and low emissions development at all levels. (vi). Reduce human and economic loss from natural hazards and disasters. (vii). Increase incomes and employment through sustainable use and value addition to water forests and other natural resources.

The Programme on Natural Resources, Environment, Climate Change, Land and Water Management is intended to contribute to the realization of the sustainable industrialization agenda of the NDPIII. It contributes directly to the NDPIII objective of Enhancing Value Addition through managing water resources for generation of electricity, provision of land for industries, regulation of pollution and provision of raw materials.
The Program Budget Frame Work paper 2023/24 is composed of the following votes;

1) Vote 019 Mistry of Water and Environment
2) Vote 150 National Environment Management Authority
3) Vote 157 National Forestry Authority
4) Vote 109 Uganda National Meteorological Authority
5) Vote 122 Kampala Capital City Authority
6) Vote 500-6137 Local Governments
7) Vote 012 Ministry of Lands Housing and Urban Development
8) Vote 003 Office of the Prime Minster, Disaster Preparedness Subprogram,

Over the years, there has been massive degradation of water catchments that generate and replenish water resources. This has fundamentally affected the quantity and quality of water resources and indeed the whole hydrological cycle. Poor land use and agricultural practices have led to massive soil erosion, pollution and loss of arable land. In addition, climate change has hit hard and destabilized the global hydrological cycle leading to frequent unpredictable weather patterns in form of floods, droughts and cyclones. The continued environmental degradation will affect water availability, increase ecosystem imbalances and loss of biodiversity.

The Programme advocates for government to recognize the critical role natural resources play in the national economy and hence the need to be given due attention to sustainably contribute to economic development of the country. The natural resources support the growing need for industrialization, transportation, and consumption of everyday goods and services hence the attention it deserves. There is need for increased financing for the programme to be able to contribute to pillars one, two, four and seven of the PDM strategy

The Plans for the Programme were drawn up within the MTEF and are linked to the NDPIII targets. The annual financial projections for the Programme have been adjusted to match the funds available for each year in the MTEF.
The Votes mapped under the Natural Resources, Environment, Climate Change, Land and Water Management Programme have a total approved budget of UShs 547.362bn of which UShs 42.66bn is wage UShs 79.650bn is nonwage recurrent while UShs 146.4bn is domestic development and UShs 278.646bn is external financing.

I therefore call upon all stakeholders to support the programme to implement its plans as outlined recognizing its key role

NATURAL RESOURCES, ENVIRONMENT, CLIMATE CHANGE, LAND AND WATER

I therefore call upon all stakeholders to support the programme to implement its plans as outlined recognizing its key role towards the achievement of the NDPIII targets and its contribution to the Sustainable Development Goals.

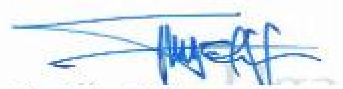

Alfred Okot Okidi

Permanent Secretary, Ministry of Water and Environment

NATURAL RESOURCES, ENVIRONMENT, CLIMATE CHANGE, LAND AND WATER

Abbrevations and Acronyms

ACRONYM	ACRONYM NAME
CBMS	Community Based Maintenance System
CBO	Community Based Organisation
CDM	Clean Development Mechanism
CFA	Cooperative Framework Agreement
CMO	Catchment Management Organisation
DESS	Department of Environment Services
HIP	Hygiene Improvement Programme
WMZ	Water Management Zones

NATURAL RESOURCES, ENVIRONMENT, CLIMATE CHANGE, LAND AND WATER

P1: PROGRAMME OVERVIEW

Snapshot of Medium Term Budget Allocations

Table P1.1 Overview of Programme Expenditure and Medium Term Allocations (Ush Billion)

Billion Uganda Shillings		FY2022/23		FY2023/24	MTEF Budget Projections			
		Approved Budget	Spent by End Sep	Proposed Budget	2024/25	2025/26	2026/27	2027/28
Recurrent	Wage	43.441	8.930	42.666	44.799	49.279	54.207	54.207
	Non Wage	97.505	7.767	79.650	81.243	97.492	131.614	131.614
Devt.	GoU	208.794	0.627	146.400	146.400	175.680	245.952	245.952
	ExtFin	285.402	33.165	278.646	171.816	84.835	0.000	0.000
	**GoU Total**	**349.740**	**17.324**	**268.716**	**272.442**	**322.451**	**431.773**	**431.773**
**Total GoU+Ext Fin (MTEF)**		**635.142**	**50.489**	**547.362**	**444.259**	**407.286**	**431.773**	**431.773**
	A.I.A	0.000	0.003	0.000	0.000	0.000	0.000	0.000
	**Grand Total**	**635.142**	**50.491**	**547.362**	**444.259**	**407.286**	**431.773**	**431.773**

Programme Strategy and linkage to the National Development Plan

As indicated in the NDPIII water and environment are central in contributing towards the countrys drive in increasing household incomes improved quality of life of the population through sustainable industrialization for inclusive-growth employment and wealth creation. Ugandas economy will continue to depend on the available stock of water environmental and natural resources to produce goods and deliver services

Broadly the availability of adequate water resources is critical to life and sustainability of socioeconomic activities critical national infrastructure such as hydropower development projects agro industrialization livestock fisheries health industrial development tourism development etc

Similarly a healthy clean and productive environment is essential in boosting wealth creation for social transformation and sustainable development because it reflects the balance between the demand and supply of natural resources on the one hand and the absorption and supply of waste products on the other hand in the development process

Thus considering that water and environment resources are at the core of sustainable development and are critical for socio-economic transformation and human survival the ministry and its autonomous institutions will focus on the achievement of the NDPIII programme interventions as well as the NRM Manifesto commitments in the period of 2021 2026 and beyond

P2: Highlights Of Programme Projected Performance

Table P2.1 Programme Outcomes Indicators

**Programme Outcome**	Adequate Quantity and Improved Quality of Water Resources for all uses					
**Programme Objectives contributed to by the Intermediate Outcome**						
Assure availability of adequate and reliable quality fresh water resources for all uses						
			Performance Targets			
**Programme Outcome Indicators**	**Base Year**	**Base Line**	**2023/24**	**2024/25**	**2025/26**	**2026/27**
Compliance to abstraction permit conditions - Ground water	2017/18	76	80%	81%	83%	85%

NATURAL RESOURCES, ENVIRONMENT, CLIMATE CHANGE, LAND AND WATER

**Programme Outcome**	Adequate Quantity and Improved Quality of Water Resources for all uses					
**Programme Objectives contributed to by the Intermediate Outcome**						
Assure availability of adequate and reliable quality fresh water resources for all uses						
			**Performance Targets**			
**Programme Outcome Indicators**	**Base Year**	**Base Line**	**2023/24**	**2024/25**	**2025/26**	**2026/27**
Compliance to abstraction permit conditions - Surface water	2017/18	78%	80.5%	82%	83%	85%
Compliance to waste water discharge permit conditions	2017/18	63%	67%	68%	70%	73%
**Programme Outcome**	Increased protection and productivity of the environment and natural resources					
**Programme Objectives contributed to by the Intermediate Outcome**						
Increase forest, tree and wetland coverage and restore and protect hilly and mountainous areas and rangelands						
			**Performance Targets**			
**Programme Outcome Indicators**	**Base Year**	**Base Line**	**2023/24**	**2024/25**	**2025/26**	**2026/27**
% of land area covered by forests	2017/18	12.4%	13.9%	14%	14.1%	14.5%
% of land area covered by wetlands.	2017/18	8.9%	9%	9.1%	9.2%	9.3%
o/w - natural forests	2017/18	9.1%	9.6%	9.7%	9.8%	9.9%
o/w – plantations	2017/18	0.4	3.5%	4%	4.5%	5
**Programme Outcome**	Increased incomes and employment from natural resources					
**Programme Objectives contributed to by the Intermediate Outcome**						
Increase incomes and employment through sustainable use and value addition to water resources, forests, rangelands and other natural resources						
			**Performance Targets**			
**Programme Outcome Indicators**	**Base Year**	**Base Line**	**2023/24**	**2024/25**	**2025/26**	**2026/27**
Proportion of green jobs to total jobs	2017/18	25%	36%	38%	40%	43%
**Programme Outcome**	Clean and productive environment					
**Programme Objectives contributed to by the Intermediate Outcome**						
Maintain and/or restore a clean, healthy, and productive environment						
			**Performance Targets**			
**Programme Outcome Indicators**	**Base Year**	**Base Line**	**2023/24**	**2024/25**	**2025/26**	**2026/27**
% of key biodiversity areas covered by protected areas	2017/18	28%	37.6%	40%	42%	45%
% of Municipal solid waste disposed off safely	2017/18	45%	69%	75%	77%	79%
% of permit holders complying with ESIA conditions at the time of spot check	2017/18	40%	80%	84%	87%	90%
Air Quality Index PM2.5	2017/18	147	145	140	138	135
Percentage area of degraded catchment areas protected	201/18	30%	43%	45%	47%	49%

NATURAL RESOURCES, ENVIRONMENT, CLIMATE CHANGE, LAND AND WATER

**Programme Outcome**	Strengthened, coordination, resilience, adaptive and mitigation capacity to climate change						
**Programme Objectives contributed to by the Intermediate Outcome**							
Promote inclusive climate resilient and low emissions development at all levels							
			**Performance Targets**				
**Programme Outcome Indicators**	**Base Year**	**Base Line**	**2023/24**	**2024/25**	**2025/26**	**2026/27**	
% of Automation of Weather and Climate Network	2017/18	30%	70%	80%	83%	85%	
% of sectors integrating climate change in their development plans	2017/18	30	35%	40%	45%	50%	
Accuracy of Meteorological Information (%)	2017/1/	60%	80%	83%	85%	88%	
Average Annual Change in a Green House Gas (GHG) emissions (MtCO2e)	2017/18	1.39	1.07%	1	0.9	0.8	
Climate Change Vulnerability Index	2017/18	2.5	4	4.5%	4.6%	4.9%	
**Programme Outcome**	Reduced human and economic loss from natural hazards and disaster						
**Programme Objectives contributed to by the Intermediate Outcome**							
Reduce human and economic loss from natural hazards and disasters							
			**Performance Targets**				
**Programme Outcome Indicators**	**Base Year**	**Base Line**	**2023/24**	**2024/25**	**2025/26**	**2026/27**	
Economic loss (USD incurred per disaster as a % of GDP)	2017/18	7.5%	5.5%	5%	4%	3%	
No of deaths and missing persons and directly affected persons attributed to disasters per 100,000 population	2017/18	150	70	50	30	15	
Percentage automation of weather and climate network	2017/18	56%	91%	91.2%	95%	100%	
**Programme Outcome**	Improved productivity of land resources						
**Programme Objectives contributed to by the Intermediate Outcome**							
Strengthen land use and management							
			**Performance Targets**				
**Programme Outcome Indicators**	**Base Year**	**Base Line**	**2023/24**	**2024/25**	**2025/26**	**2026/27**	
Percentage of Government Land titled	2017/18	22%	44.4%	50%	55%	58%	
Percentage of titled land	2017/18	21%	35%	40%	45%	50%	
Portfolio of land and properties valued in trillions UGX (e.g. projects and programs, etc)	2017/18	2	5.6	6.1	6.5	6.9	
Proportion of LGs with approved PDPs, %	2017/18	43.9%	80%	85%	87%	89%	
Turnaround time for titling of land (days)	2017/18	14	7	5	4	3	

Table P2.2: Intermediate Outcomes Indicators

NATURAL RESOURCES, ENVIRONMENT, CLIMATE CHANGE, LAND AND WATER

**Sub-Programme Name:**	**Environment and Natural Resources Management**					
**Intermediate Outcome Indicators:**	Enhanced value addition to conserved natural resources					
	**Performance Targets**					
**Indicators**	**Base Year**	**Base Line**	**2023/24**	**2024/25**	**2025/26**	**2026/27**
No. of Beneficiaries from ENR enterprises.	2017/18	20000	200000	250000	300000	350000
Trends in Forest Livelihood support initiatives.	2017/18	4	8	9	10	10
**Intermediate Outcome Indicators:**	Enhanced sustainable Waste Management in urban areas					
	**Performance Targets**					
**Indicators**	**Base Year**	**Base Line**	**2023/24**	**2024/25**	**2025/26**	**2026/27**
Number of Municipalities/ cities with Sustainable Waste Management Facilities	2017/18	12	27	32	35	40
**Intermediate Outcome Indicators:**	Fragile ecosystems restored					
	**Performance Targets**					
**Indicators**	**Base Year**	**Base Line**	**2023/24**	**2024/25**	**2025/26**	**2026/27**
Trends in fragile ecosystems protected and restored(Hectares)	2017/18	0	6500	7500	8500	9500
**Intermediate Outcome Indicators:**	Economic valuation of Natural Resources undertaken					
	**Performance Targets**					
**Indicators**	**Base Year**	**Base Line**	**2023/24**	**2024/25**	**2025/26**	**2026/27**
Trends in natural capital accounts developed.	2017/18	1	7	8	9	10
**Intermediate Outcome Indicators:**	National Green House Gas emissions effectively monitored.					
	**Performance Targets**					
**Indicators**	**Base Year**	**Base Line**	**2023/24**	**2024/25**	**2025/26**	**2026/27**
A functional GHG Monitoring, Reporting and Verification system.	2017/18	0	1	0	0	0
**Intermediate Outcome Indicators:**	Reduced human and economic loss from natural hazards and disasters					
	**Performance Targets**					
**Indicators**	**Base Year**	**Base Line**	**2023/24**	**2024/25**	**2025/26**	**2026/27**
Number of disaster risk assessments conducted	2017/18	200	400	450	470	490
Number of people supplied with relief items	2017/18	300000	500000	600000	650000	700000
Proportion of disaster risk and vulnerability assessments carried out, %	2017/18	90%	100%	100%	100%	100%
Average response time to disasters (Hrs)	2017/18	48	24	24	24	24
**Sub-Programme Name:**	**Water Resources Management**					
**Intermediate Outcome Indicators:**	Improved Catchment Based Water Resources Management					
	**Performance Targets**					
**Indicators**	**Base Year**	**Base Line**	**2023/24**	**2024/25**	**2025/26**	**2026/27**
Frequency of Water Quantity updates	2017/18	1	2	2	2	2

NATURAL RESOURCES, ENVIRONMENT, CLIMATE CHANGE, LAND AND WATER

**Sub-Programme Name:**	**Water Resources Management**					
**Intermediate Outcome Indicators:**	Improved Catchment Based Water Resources Management					
	**Performance Targets**					
**Indicators**	**Base Year**	**Base Line**	**2023/24**	**2024/25**	**2025/26**	**2026/27**
Percentage of planned CMP Interventions implemented	2017/18	70	90%	95%	97%	99%
**Intermediate Outcome Indicators:**	Improved Water Quality Monitoring					
	**Performance Targets**					
**Indicators**	**Base Year**	**Base Line**	**2023/24**	**2024/25**	**2025/26**	**2026/27**
No. of permit holders complying to permit conditions.	2017/18	804	910	920	930	950
No. of user permits issued.	2017/18	296	336	346	366	386
No. of Water User Permit holders monitored.	2017/18	1130	1530	1630	1730	1830
**Intermediate Outcome Indicators:**	Increased hectares if ecosystems under Approved Management Plans					
	**Performance Targets**					
**Indicators**	**Base Year**	**Base Line**	**2023/24**	**2024/25**	**2025/26**	**2026/27**
Area of wetland restored and maintained	2017/18	10500	22000	28500	30500	35500
Forestry area under approved Management Plans (%)	2017/18	36%	55%	65%	68%	70%
Land area covered by forests, %	2017/18	12.4%	13.9%	14%	14.2%	14.4%
Land area covered by wetlands, %	2017/18	8.9%	9%	9.1%	9.2%	9.57%
Survival Rate of tree Seedlings beyond 3 years, %	2017/18	76%	83%	84%	85%	87%
Wetlands Area under approved Management Plans (%)	2017/18	11.3%	19.5%	21.5%	23%	25%
**Intermediate Outcome Indicators:**	Demarcated and recorded customary land					
	**Performance Targets**					
**Indicators**	**Base Year**	**Base Line**	**2023/24**	**2024/25**	**2025/26**	**2026/27**
No. of Customary Certificate of Occupancy issued	2017/18	14000	20000	22000	24000	26000
**Intermediate Outcome Indicators:**	Land consolidation, titling and banking					
	**Performance Targets**					
**Indicators**	**Base Year**	**Base Line**	**2023/24**	**2024/25**	**2025/26**	**2026/27**
Acreage of land titled, consolidated and banked.	2017/18	0	100000	100000	100000	100000
**Intermediate Outcome Indicators:**	Reduced land related conflicts					
	**Performance Targets**					
**Indicators**	**Base Year**	**Base Line**	**2023/24**	**2024/25**	**2025/26**	**2026/27**
No. of overlapping surveys and titles harmonized	2017/18	0	200000	500000	600000	700000
No. of titles processed for bona fide occupants	2017/18	0	2500	3000	3500	4000
Percentage of customers reporting satisfaction with the Land Registry Services	2017/18	70%	83%	85%	86%	89%
Percentage of land conflicts/ disputes mediated	2017/18	65	25%	20%	19%	18%

NATURAL RESOURCES, ENVIRONMENT, CLIMATE CHANGE, LAND AND WATER

**Sub-Programme Name:**	**Water Resources Management**					
**Intermediate Outcome Indicators:**	Reduced land related conflicts					
			**Performance Targets**			
**Indicators**	**Base Year**	**Base Line**	**2023/24**	**2024/25**	**2025/26**	**2026/27**
Percentage of land registered under the 4 different tenure systems (disaggregated)	2017/18	21	40%	43%	45%	47%
Percentage of public projects acquiring land in the stipulated time	2017/18	60%	73%	75%	77%	80%
Average days taken to register land	2017/18	12	7	5	4	3

P3: Medium Term Budget Allocations by Sub-Programme and Vote

Table P3.1: Proposed Budget Allocations and Medium Term Projections by Sub-Programme

Billion Uganda Shillings	**FY2022/23**	**2023/24**	**Medium Term Projections**			
	**Approved Budget**	**Proposed Budget**	**2024/25**	**2025/26**	**2026/27**	**2027/28**
01 Environment and Natural Resources Management	374.260	332.477	326.106	277.283	277.379	269.247
02 Land Management	155.323	125.325	55.633	66.142	88.043	93.175
03 Water Resources Management	87.706	89.560	62.520	63.861	66.351	69.351
**Total for the Programme**	**635.142**	**547.362**	**444.259**	**407.286**	**431.773**	**431.773**

Table P3.2: Proposed Budget Allocations and Medium Term Projections by Vote

Billion Uganda Shillings	**FY2022/23**		**2023/24**	**Medium Term Projections**			
	**Approved Budget**	**Spent by End Sep**	**Proposed Budget**	**2024/25**	**2025/26**	**2026/27**	**2027/28**
003 Office of the Prime Minister	20.805	0.387	17.747	17.918	21.466	29.465	29.465
012 Ministry of Lands, Housing & Urban Development	123.659	2.663	97.442	27.673	32.293	41.669	41.669
019 Ministry of Water and Environment	259.927	36.293	319.239	283.606	217.916	181.839	181.839
109 Uganda National Meteorological Authority (UNMA)	17.656	1.558	17.143	17.587	20.326	25.663	25.663
122 Kampala Capital City Authority (KCCA)	20.556	3.073	18.220	18.578	22.293	30.116	30.116
150 National Environment Management Authority (NEMA)	18.943	2.489	19.772	20.289	23.641	30.215	30.215
156 Uganda Land Commission (ULC)	31.663	0.151	27.882	27.960	33.440	46.374	46.374
157 National Forestry Authority (NFA)	29.242	2.366	26.416	27.079	31.627	40.648	40.648
606 Local Governments 06	94.837	1.500	3.500	3.570	4.284	5.783	5.783
**Total for the Programme**	**635.142**	**50.491**	**547.362**	**444.259**	**407.286**	**431.773**	**431.773**

NATURAL RESOURCES, ENVIRONMENT, CLIMATE CHANGE, LAND AND WATER

P4: PROGRAMME PRIORITIES IN LINE WITH INTERVENTIONS FOR FY2023/24

Programme Priorities FY2023/24	NDP III Programme Intervention
- National Land Information System (NLIS) enhancements developed and rolled out<br>- 62.5 bn revenue generated<br>- Additional floor at the National Land Information Centre and new Archival Centre Constructed<br>- LIS maintained in the 22 MZOs and other LIS sites<br>- National Atlas revised<br>- SLAAC Data Capturing and Processing Software upgraded and maintained<br>- Boundary separation for 10 combined blocks in Wakiso and Bukalasa carried out.<br>- Government Cadastre Data Inventory and Consolidation for 2 MZOs (Wakiso and Luwero MZOs) prepared<br>- Resurvey and Coordination of Cadastre Blocks and Insets for 2 MZOS (Wakiso and Luwero) carried out<br>- 54 Topographic maps revised for 6 cities (Amolatar,, Kiryandongo, Kole, Dokolo, Lamwo and Bugiri)<br>- 50 rectifications of surveys and mapping data made across the 22 MZOs.<br>- 114 NLIC staff and LIS Users trained on LIS<br>- 72 Rapid Physical Planning Appraisal (RAPPA) Plans produced and disseminated.<br>- Institute of Surveys and Land Management (ISLM) upgraded and supported<br>- Curriculum for Master of Science Course in Land Management and Administration at Makerere University supported	Complete the rollout and integration of the Land Management Information System with other systems.

NATURAL RESOURCES, ENVIRONMENT, CLIMATE CHANGE, LAND AND WATER

Programme Priorities FY2023/24	NDP III Programme Intervention
Promoting Sustainable development of Oil and Gas. NEMA will :<br>1. Undertake environment monitoring inspections and enforcement activities in Oil and gas exploration areas, including new planned basins and production areas.<br>2. Respond to reported environment pollution and degradation incidences in the Oil and Gas areas, both exploration and development areas<br>3. Undertake Baseline verification Inspections<br>Undertake joint review of environmental and social impact assessment (ESIA) submissions, and baseline data collections to formulate baseline monitoring plans and sensitivity atlases<br>4. Train DLGS in Albertine Graben & related basins on effecive monitoring and enforcement of oil and gas development	Develop and implement a framework that reduces adverse per capita environmental impact of cities (air quality and waste management practises)
- Countrywide land market values compiled<br>- Land values collection software developed<br>- 60 land acquisitions for Government projects supervised<br>- Annual Property index data 2022/23 compiled<br>- Databank for compensation rates updated to promote fair compensation rates and land market for all including the elderly, women and PWDs<br>- 30,000 property valuations carried out and supervised<br>- National Valuation Standards and Guidelines developed<br>- Property indices for taxation and valuation purposes developed and published<br>- Compensation rates for 135 districts reviewed and approved	Develop and implement a Land Valuation Management Information System (LAVMIS);
Public awareness on weather and climate issues raised through media<br>engagements, digital campaigns and talk shows<br>4 seasonal climate outlooks issued countrywide with advisories to key	Enhance access and uptake of meteorological information

NATURAL RESOURCES, ENVIRONMENT, CLIMATE CHANGE, LAND AND WATER

Programme Priorities FY2023/24	NDP III Programme Intervention
sectors of the economy for all the 16 climatological zones<br>4 Seasonal rainfall performance evaluations conducted in four regions of<br>the country<br>4 seasonal forecasts disseminated by conducting 20 radio talk shows<br>across the country<br>Vulnerability maps produced for 4 regions to identify climate change<br>hotspot areas to support planning and decision making<br>Pilot study on the utilisation of daily weather forecasts and impacts of<br>severe weather conditions conducted in central and eastern regions.<br>Sensitization and feedback on Marine forecasts obtained for Lakes<br>Albert, Kyoga and Victoria<br>6,570 Terminal Aerodrome Forecasts, 810 SIGMETs and 65,700 METARs and 35,570 Flight folders issued for Entebbe and Soroti<br>(operating 24hrs) ; Gulu, Arua, Kasese,Kajjasi, Kabaale, Jinja and<br>Tororo (operating 12hours) Aerodromes.<br>Daily forecasts disseminated to 3 media houses of UBC TV, Star TV,<br>Bukedde 1 T.V after the newscasts in Luganda, Swahili and English<br>4 Community engagements undertaken to expand Marine weather<br>services to cover Lake Albert (Butyaba and Wanseko) and Kyoga<br>(Namasale and Lwampanga)<br>Sensitisation programs on the importance and use of meteorological<br>services developed and disseminated in 30 primary and 30 secondary<br>schools in Bulisa, Kikube, Kiryandongo, Masindi, Amolatar, Dokolo,<br>Alebtong districts as per WMO's Public Education and Outreach Programs<br>14 Parish Weather Clinics implemented to support the PDM of Government in districts of Mbarara, Bushenyi, Otuke, Alebtong, Pakwach, Nebbi and Nwoya<br>Research on impacts of severe weather events on People with Disabilities (PWD) carried out in Bududa, Kasese, Kampala, Soroti,<br>Kaberamaido, Dokolo, Lira.<br>The Annual State of the Climate report for Uganda for 2023 produced<br>and provisional state of climate report of Uganda for 2024	

NATURAL RESOURCES, ENVIRONMENT, CLIMATE CHANGE, LAND AND WATER

Programme Priorities FY2023/24	NDP III Programme Intervention
[illegible]<br>and provisional state of climate report of Uganda for 2024	
1. 132 preparedness assessments undertaken and 60 Needs assessments conducted to collect Pre and post disaster risk information across the country<br>2. Rapid emergency and disaster response enhanced through:<br>(i) Equipping NECOC with assorted software and hardware<br>(ii) Conducting 4 Quarterly training/ simulation of NECOC staff<br>(iii) Activating 5 new DECOCs to enhance emergency preparedness and response, establishment of Incident Command Systems during disasters,<br>(iv) Eight (8) emergency disaster responses coordinated,<br>(iv) Production of 12 monthly timely situation reports,<br>(vi) Strengthening Early Warning systems and tools,<br>(vii) Conducting of 4 Quarterly search and rescue operations,<br>(viii) Procurement of 3 transport equipment (2 trailers and 1 Double Cabin pickup).<br>3. Two hundred fifty thousand (250,000) households (out of which 70% are women and children) supported with food and non-food items across the country<br>4. A security and retaining wall constructed around 4 acre Namanve relief stores land	Enhance capacities for storage, management and distribution of relief commodities.

NATURAL RESOURCES, ENVIRONMENT, CLIMATE CHANGE, LAND AND WATER

Programme Priorities FY2023/24	NDP III Programme Intervention
1. One hundred (100) households displaced by landslides and floods in Bududa, Manafwa, Bulambuli, Namisindwa and Sironko permanently resettled<br>2. Funds transferred to Uganda Red Cross Society to support disaster victims<br>3. Three (03) Basic amenities (i.e. water, electricity, access roads) supported for resettled households.<br>4. Disaster incidents or events (e.g. landslides, flooding, drought etc.) assessed in 80 districts across the country.<br>5. Cash transfers for IDPs to buy land and relocate from disaster prone areas.<br>6. Resettlement in Kayunga and Kasese districts	Enhance the capacity for resettlement of persons at risk of disasters.
-Trustee incorporation reviewed and trustees regulation formulated<br>- Policy and Legal Frameworks reviewed and developed	Fast-track the formulation, review, harmonisation, and implementation of land laws, policies regulations, standards and guidelines.
oWater vessel Jetty Constructed.<br>oLira and Mbale Regional laboratories furnished.<br>o1New Rating curve developed<br>4 Rating curves updated and compiled into a report<br>oAquifer system in 1 cattle corridor district in KWMZ Nakasongola characterized<br>oConfiguration and calibration 8 surface water and 8 groundwater telemetric stations<br>oCatchment management measures Soil and Water management conservation measures restore deforested and degraded communal and individual land restore degraded stretches of the rivers treat and restore degraded stretches of gulleys to control erosion siltation and destruction of property implemented in Lwakhakha, Aswa II Kochi and Middle Awoja sub-catchments upstream midstream and downstream of river Nyamwamba catchment and Micro-catchments of Ora Anyau Nyimur and Mutunda and Laropi Ayugi in the Refugee Settlements to 100 percent.<br>o100 percent of the Groundwater study to assess available resources and demand undertaken.	Improve coordination, planning, regulation and monitoring of water resources at catchment level

NATURAL RESOURCES, ENVIRONMENT, CLIMATE CHANGE, LAND AND WATER

Programme Priorities FY2023/24	NDP III Programme Intervention
15 million assorted quality exotic seedlings (5million-Indigenous and 10-fast growing) supplied from verified seed sources, 2,000kg of assorted indigenous tree seed and other planting materials supplied and raised from 32 nurseries across the country	Increase funding for promoting non-consumptive uses of the natural resources
20 Barometers and 20 Thermometers calibrated<br>Sanitation for 4 manual weather stations of Mubende, Bududa, Kotido,<br>Wadelai improved<br>100 Automatic Weather Stations, 38 Manual Weather Stations and 150<br>rainfall Stations maintained across the country.<br>15 Manual Weather Stations equipped with 15 digital thermometers<br>Functionality and Operation of 100 ADCON Automatic Weather Stations, 53 Manual Stations and 200 Rainfall Stations maintained across<br>the Country<br>Transmission of Weather data maintained from upcountry stations<br>Office Shelters Raised for 2 Weather Stations of Kibanda and Wadelai	Install new and adequately equip and maintain existing automatic weather stations to ensure maximum functionality
1. A comprehensive national disaster risk management plan developed<br>2. DRR day and Peace day organized and celebrated<br>3. Twelve (12) monthly National Disaster monitoring, early warning and disaster reports produced<br>4. Annual state of disaster report disseminated.<br>5. Thirty-six (36) District Disaster Management Committees (DDMC) resilience and contingency planning Trainings conducted to support disaster risk informed planning, preparedness and response.	Institutionalize disaster risk planning in Programmes.

NATURAL RESOURCES, ENVIRONMENT, CLIMATE CHANGE, LAND AND WATER

Programme Priorities FY2023/24	NDP III Programme Intervention
2 wetland wise use models established in Sezibwa and Aswa wetland system<br>4 wetland management plans implemented in Mayanja and Aswa Wetland resource based enterprises Eco tourism sites and craft centers developed and completed in Mayanja and Aswa Eco tourism sites and craft centers identified and developed in Sezibwa<br>A strategy for sound management of chemicals to curtail air land and water pollution in urban areas prepared	Mainstream environment and natural resources management in policies, programmes and budgets with clear budget lines and performance indicators.
Blue page register updated<br>- 19,000 land searches conducted<br>- 20 trustees registered<br>- 100 land titles issued in fragile ecosystems (wetlands and forest reserves) cancelled<br>- 400 affidavits commissioned<br>- 400 court cases facilitated<br>- 103,440 titles issued to men and women<br>- Inspection and Support Supervision in 22 MZOs conducted<br>- 90,000 files Committed into the UgNLIS<br>- 200 Land Disputes Resolved<br>- 600 CLAs formed and registered<br>- 50,000 CCOs processed<br>- 460,000 new parcels adjudicated and demarcated out of which 178,000 are for women individually or jointly owned.<br>- 200,000 SLAAC Titles processed and issued out of which 80,000 are owned by women individually or jointly.<br>- Gender Strategy interventions implemented<br>- 600 CLAs formed and registered<br>- 1,200 CLA land demarcated	Promote land consolidation, titling and banking.

NATURAL RESOURCES, ENVIRONMENT, CLIMATE CHANGE, LAND AND WATER

Programme Priorities FY2023/24	NDP III Programme Intervention
900 Km of conserved and degraded wetland systems demarcated (in Central Uganda-start demarcation in Kiboga and Mpigi (Mayanja wetland system), Kayunga, Luwero (Sezibwa/Lwajjali wetland System). Northern- Gulu, (Aswa wetland System), Kibaale, Kyenjojo (Muzizi). Eastern: Bugweri and Luuka (Lumbuye)<br><br>13,900 Ha of degraded wetland sections restored (in Central Uganda-start demarcation in Kiboga and Mpigi (Mayanja wetland system), Kayunga, Luwero (Sezibwa/Lwajjali wetland System). Northern- Gulu, (Aswa wetland System), Kibaale, Kyenjojo (Muzizi). Eastern: Bugweri and Luuka (Lumbuye)<br><br>4,000 wetland systems gazetted and publicized; Green parks/protected belts gazetted in Cities/Towns<br><br>100Ha of mountain ecosystems restored and 50Km of natural water bodies, reservoirs, river banks, Lakeshores surveyed and demarcated.<br>450 ha of degraded river banks and lakeshores restored and maintained<br><br>300Km of conserved and degraded wetlands demarcated with pillars and live markers in the GCF districts; 3,665 Wetland systems gazetted	Strengthen conservation, restoration of forests, wetlands and water catchments and hilly and mountainous areas:

NATURAL RESOURCES, ENVIRONMENT, CLIMATE CHANGE, LAND AND WATER

Programme Priorities FY2023/24	NDP III Programme Intervention
300Km of conserved and degraded wetlands demarcated with pillars and live markers in the GCF project districts.<br>Environment Protection Police supported.<br>55,000,000 seedlings of assorted tree species procured and distributed to farmers in project target districts and refugee hosting communities in the Albertine and West Nile Regions as well as individual farmers and District local governments<br>Equipment for establishing a wood processing hub procured and installed at Nyabyeya Forestry College<br>Wetlands.900 Km of conserved and degraded wetland systems demarcated in Kiboga and Mpigi (Mayanja wetland system), Kayunga, Luwero (Sezibwa/Lwajjali wetland System); Gulu, (Aswa wetland System); Kibaale, Kyenjojo (Muzizi), Bugweri & Luuka (Lumbuye wetland Systm)<br>13,900 Ha of degraded wetland sections restored in in Kiboga and Mpigi (Mayanja wetland system), Kayunga, Luwero (Sezibwa/<br>Lwajjali wetland System). Gulu, (Aswa wetland System), Kibaale, Kyenjojo (Muzizi wetland System), Bugweri and Luuka (Lumbuye).<br>2 wetland wise use models established in Sezibwa and Aswa wetland systems.<br>4 wetland management plans implemented in Mayanja and Aswa (Wetland resource-based enterprises).<br>Eco-tourism sites and craft centers developed and completed in Mayanja and Aswa.<br>Eco-tourism sites and craft centers identified and developed in Sezibwa.<br>10 wetland inventory reports for the districts of Kyenjojo, Kyegegwa, Kagadi, Kabarole, Kibaale, Abim, Rubirizi, Kamwenge, Bunyagabu and Agago reviewed and printed.<br>Ecological and socio-economic studies to inform designation of Lubigi Wetland as a wetland park conducted.<br>3,665 Wetland systems gazetted.<br>110km of degraded river banks demarcated and planted with pillars	Strengthen conservation, restoration of forests, wetlands and water catchments and hilly and mountainous areas:
Undertake environment monitoring inspections and enforcement activities<br>Respond to reported environment pollution and degredation incidences<br>Undertake joint review of environmental and social impact assessment (ESIA) submissions<br>Represent NEMA in Civil Litigation matters<br>Undertake prosecutions	Strengthen conservation, restoration of forests, wetlands and water catchments and hilly and mountainous areas:

NATURAL RESOURCES, ENVIRONMENT, CLIMATE CHANGE, LAND AND WATER

Programme Priorities FY2023/24	NDP III Programme Intervention
4000 water supplies and industries monitored for compliance to national standards.<br>4000 boreholes, shallow wells, protected springs and piped water systems monitored, samples collected for compliance to drinking water standards<br>55 stations on Lake Victoria, 8 Kyoga, 7 Albert, 7 Edward and 10 George monitored for compliance to ambient water quality.<br>50 industries supported to implement Resource Efficiency and Cleaner Production best practices<br>One Water Resources assessment undertaken to 100 percent<br>300 Applications for water permits including drilling, construction, water abstraction and waste water discharge permits assessed.<br>500 permits 250 New and 250 renewals; ground water, surface water abstraction, drilling, hydraulic constructions, and dredging and waste water discharge issued.<br>400 Permit holders monitored and assessed for compliance to permit conditions	Strengthen enforcement capacity for improved compliance levels:
50 DLBs, 50 DLOs and 140 ALCs trained in land management<br>- 35 DLOs, 35 DLBs, and 22MZOs supervised, monitored and technically supported<br>- The role of 3 traditional institutions in land administration strengthened<br>- 10 Public sensitizations on Land matters Undertaken in 10 subregions of Acholi, Ankole, Buganda, Bugisu, Busoga, Karamoja, Kigezi, Teso, Toro, and West Nile ensuring representation of all groups especially women and the vulnerable<br>- 8 technical staff trained in specialized short courses on Land Management and Administration	Strengthen the capacity of land management institutions in executing their mandate geared towards securing land rights.

P5: GENDER AND EQUITY ISSUES AND INTERVENTIONS FOR FY2023/24

Gender Equality and Women Empowerment
The advance effects of environment degradation are experienced by both men and women but because of the women triple roles which put her so close to the environment women and children tend to face a lot of challenges Women and girls in Uganda as in other sub Saharan African countries are the major users and managers of the environment in homes they therefore bear the impact of environmental degradation.
In order to address these issues, ENR sub sector has developed a gender strategy so as to reduce unsustainable environmental resources management as well as reduce poverty resulting from inequalities in environmental resource use, access and management.

Engender ENR policies plans and implementation guidelines coordinate the development of guidelines for improved access

and control of ENR resources for men women and vulnerable groups support ENR agencies and department to design criteria for equal participation in planning and sustainable management of resources support ENR Sub sector department and agencies to establish mechanism for equitable sharing of resources.

Develop capacity of ENR sub sector departments and agencies and stakeholders to mainstream gender in environmental management

Wetland Management Department
Assessing the level of compliance in gender mainstreaming for wetland management assess levels of access and control regimes of wetland resources; promote equal representation of both men and women in decision making for wetland management develop and disseminate mechanism for equitable sharing of wetland products and services by men and women through the value chain and public private partnerships build and strengthen capacity for gender mainstreaming for wetland management staff and stakeholders Department Of Environment Support Services Integrate gender into the environmental management policies legislations plans budgets and programs support the development of Management plans for equitable opportunities for access and control of environment resources; strengthen the environment management committees at all levels with a gender lens assess the gender disparities in the benefits sharing of environment resources build and strengthen the capacity of DESS in gender mainstreaming

Climate change department
Ensuring that Gender is integrated in the Implementation of the National Climate Change Policy and its costed implementation strategy improving capacity of relevant stake holders to achieve gender responsive outcomes of the national climate change policy
Support department to design criteria for equal participation in planning and sustainable management of resources establishing a mechanism for equitable sharing of resources establishing acknowledge base for gender climate change adaptation and mitigation.

National Forestry Authority
Mainstreaming gender in all NFA policies and guidelines
promote equal access of both men and women to Natural Forest resources involve both men and women in planning forestry activities establish mechanisms for equitable natural forests resource sharing; impart gender mainstreaming skills among NFA staff and other stakeholders National Environment Management Authority Integrate gender issues into NEMA management policies and procedures review NEMAs governance and management structures to include men and appraise to make them all-inclusive gender inclusion in planning, budgeting implementation and management of ENR develop gender inclusive public awareness and communication strategy ensure all NEMA Policies and guidelines have mechanisms and strategies for sharing benefits from ENR build capacity of NEMA and all their structures for gender mainstreaming.

Uganda National Meteorological Authority
To identify gender issues and popularize ENR gender mainstreaming to promote equitable access and opportunities of men and women to weather and climate information promote affirmative action for recruitment training of both male and female workers disseminate gender responsive weather climate information warnings and alerts strengthen capacity for gender mainstreaming for Met staff stake holders and popularize Meteorology in schools and at all levels

Forestry Support Service Department
To integrate gender issues into Forestry policies reforms planning implementation monitoring and evaluation to promote equitable access to and control of forest resources and opportunities for both men women and vulnerable groups to promote equitable participation of men women and vulnerable groups in planning implementation and management of forestry resources at all levels to promote equitable benefits sharing of forestry resources between men women and vulnerable groups to build capacity for gender mainstreaming for forestry subsector actors and stakeholders at all levels for sustainable

NATURAL RESOURCES, ENVIRONMENT, CLIMATE CHANGE, LAND AND WATER

[illegible]
resources management

Due to high poverty levels in the areas served by the Special programs attributed to the legacy of conflict and violence experienced over time

PRIVATE SECTOR DEVELOPMENT

Foreword

This Private Sector Development (PSD) Programme Budget Framework Paper (PBFP) consolidates gains from implementing the PSD PIAP for the previous two years of the NDP III. The Government's medium term goals under the programme are outlined in its NDP III objectives namely "to increase competitiveness of the private sector to drive sustainable inclusive growth". The focus of implementation has been and continues to be building and strengthening the resilience of enterprises for them to withstand the persistent shocks that economies have experienced. Member agencies working with the private sector are responsible for exploring non-conventional means of expanding market opportunities and building the capacity of local business to take advantage of the opportunities despite the shocks.

The PSD Programme BFP therefore provides the practical approach to prioritising and sequencing key interventions that are crucial for delivering the Programme objectives; for allocating resources to these priorities for the FY2023/24 and projections for FY2024/25. The PSD Programme BFP derives from the budget framework papers of the votes contributing to the PSD programme and is aligned to the PSD Programme Implementation Action Plan. Through a series of Programme Working Group Consultations, member institutions agreed on a number of priorities to guide their individual Vote BFPs which have been considered in this BFP preparation process. Therefore, completeness, ownership and accuracy have been key principles in the development of this PBFP.

The execution of the PSD BFP will build on existing systems and structures and efforts and strengthen practical interactions with the private sector entities through PWGs and TWGs.
Efforts towards aligning the NDP III to the Programme Budgeting System (PBS) have enabled the BFPs for FY 2023/24 to be prepared on the system and this ensures alignment of the budget to the NDP III. As we continue with the programmatic approach to planning and budgeting, we are convinced that the unlocking of the private sector will only be achieved through continuous engagement with the private sector players at all levels and stages of implementing the PSD PIAP, which we have used to guide the development of this year's Budget Framework Paper.

Ramathan Ggoobi

Permanent Secretary/Secretary to the Treasury

PRIVATE SECTOR DEVELOPMENT

P1: PROGRAMME OVERVIEW

Snapshot of Medium Term Budget Allocations

Table P1.1 Overview of Programme Expenditure and Medium Term Allocations (Ush Billion)

Billion Uganda Shillings		FY2022/23		FY2023/24	MTEF Budget Projections			
		Approved Budget	Spent by End Sep	Proposed Budget	2024/25	2025/26	2026/27	2027/28
Recurrent	Wage	53.644	12.375	53.819	56.510	62.161	68.377	68.377
	Non Wage	1,486.678	90.649	1,470.002	1,499.372	1,798.945	2,428.048	2,428.048
Devt.	GoU	42.236	1.633	16.204	16.204	19.445	27.223	27.223
	ExtFin	71.352	6.690	257.950	438.098	359.808	38.194	0.000
	**GoU Total**	**1,582.559**	**104.656**	**1,540.025**	**1,572.086**	**1,880.551**	**2,523.648**	**2,523.648**
	**Total GoU+Ext Fin (MTEF)**	**1,653.911**	**111.346**	**1,797.975**	**2,010.184**	**2,240.359**	**2,561.842**	**2,523.648**
	A.I.A	0.000	0.000	0.000	0.000	0.000	0.000	0.000
	**Grand Total**	**1,653.911**	**111.346**	**1,797.975**	**2,010.184**	**2,240.359**	**2,561.842**	**2,523.648**

Programme Strategy and linkage to the National Development Plan

This Programme directly contributes to the second objective of the NDP III of "Strengthening the Private Sector to create jobs". The PSD Programme strategy is adequately reflected in the agreed on interventions to address each of the 5 objectives of the programme as follows;

1. Sustainably lower the costs of doing business; The planned strategic interventions to implement under this objective include;
a) Lowering the cost of doing business through continuous capitalization of government's development financial institutions;
b) Establishment (construct) Regional OSCs starting with Mbale, Mbarara, Gulu, Arua
c) Address non-financial factors (power, transport, business processes, capacitating UWRS etc.) leading to high costs of doing business

2. Promote local content in public programmes; The planned strategic interventions to implement under this objective include;
a) Establishing and operationalizing structures for implementation of the local content framework, a Local Content Monitoring Committee at entity and National level
b) Build the capacity of local firms to benefit from public investments

3. Strengthen the enabling environment and enforcement of standards; The planned strategic interventions to implement under this objective include;
a) Rationalize and harmonize standards institutions, and policies at local and regional level
b) Market and attract developers and operators in the Public and Private Free Zones and industrial parks

4. Strengthen the role of government in unlocking investment in strategic economic sectors; The planned strategic interventions to implement under this objective include;
a) Operationalization of the Parish Development Model;
b) Strengthen research and innovation capacity in support of private and public investment

PRIVATE SECTOR DEVELOPMENT

[illegible]
b) Strengthen research and innovation capacity in support of private and public investment
c) Conduct feasibility studies in strategic NDPIII areas for government and private sector investment

5. Strengthen the organisational and institutional capacity of the private sector to drive growth. The planned strategic interventions to implement under this objective include:
a) BDSs provided with analysed national and international market information for rational enterprise decision-making in prioritized growth activities.

P2: Highlights Of Programme Projected Performance

Table P2.1 Programme Outcomes Indicators

**Programme Outcome**	Increased local firms' participation in public investment programmes across sectors					
**Programme Objectives contributed to by the Intermediate Outcome**						
Promote local content in public programmes						
	**Performance Targets**					
**Programme Outcome Indicators**	**Base Year**	**Base Line**	**2023/24**	**2024/25**	**2025/26**	**2026/27**
Proportion of jobs taken on by Ugandans, %	2017/18	0	10%	15%	15%	15%
Proportion of the total procurement value awarded to local contractors, %	2017/18	30%	75%	80%	80%	80%
Proportion of Ugandan goods and services utilized by foreign firms in public projects	2017/18	0	35%	40%	40%	40%
**Programme Outcome**	Standards developed and/or enforced					
**Programme Objectives contributed to by the Intermediate Outcome**						
Strengthen the enabling environment and enforcement of standards						
	**Performance Targets**					
**Programme Outcome Indicators**	**Base Year**	**Base Line**	**2023/24**	**2024/25**	**2025/26**	**2026/27**
Annual change in products certified by UNBS (%)	2017/18	24%	10.00%	10.00%	10.00%	10.00%
Number of certified products accessing foreign markets	2017/18	259	5500	6000	6000	6000
Number of counterfeits tracked and destroyed (No. of seizures)	2017/18	252	1100	1200	1200	1200
**Programme Outcome**	Increased accessibility to export processing zones					
**Programme Objectives contributed to by the Intermediate Outcome**						
Strengthen the enabling environment and enforcement of standards						
	**Performance Targets**					
**Programme Outcome Indicators**	**Base Year**	**Base Line**	**2023/24**	**2024/25**	**2025/26**	**2026/27**
Number of firms accessing the export free zones	2017/18	0	35	40	40	40

PRIVATE SECTOR DEVELOPMENT

**Programme Outcome**	Adequate legal and regulatory frameworks in place, that removes restrictions					
**Programme Objectives contributed to by the Intermediate Outcome**						
Strengthen the enabling environment and enforcement of standards						
	**Performance Targets**					
**Programme Outcome Indicators**	**Base Year**	**Base Line**	**2023/24**	**2024/25**	**2025/26**	**2026/27**
Legal and regulatory framework in place, that removes restrictions	2017/18	3	3	3	3	3
**Programme Outcome**	Increased formalization of businesses					
**Programme Objectives contributed to by the Intermediate Outcome**						
Strengthen the enabling environment and enforcement of standards						
	**Performance Targets**					
**Programme Outcome Indicators**	**Base Year**	**Base Line**	**2023/24**	**2024/25**	**2025/26**	**2026/27**
% change in tax payer register	2017/18	0	15%	15%	15%	15%
% contribution of informal sector to GDP	2017/18	0	56%	56%	56%	56%
Proportion of total businesses operating in the informal sector	2017/18	0	25%	28	28	28
**Programme Outcome**	Improved availability of private sector data					
**Programme Objectives contributed to by the Intermediate Outcome**						
Strengthen the enabling environment and enforcement of standards						
	**Performance Targets**					
**Programme Outcome Indicators**	**Base Year**	**Base Line**	**2023/24**	**2024/25**	**2025/26**	**2026/27**
Number of data requests to the MSME database	2017/18	0	300	400	400	400
Number of reports and policy briefs developed	2017/18	0	75	75	75	75
**Programme Outcome**	Adequate system for private sector complaints resolution in place					
**Programme Objectives contributed to by the Intermediate Outcome**						
Strengthen the enabling environment and enforcement of standards						
	**Performance Targets**					
**Programme Outcome Indicators**	**Base Year**	**Base Line**	**2023/24**	**2024/25**	**2025/26**	**2026/27**
% of private sector complaints resolved	2017/18	0	55%	55	55	55
**Programme Outcome**	Improved business capacity and local entrepreneurship skills enhanced					
**Programme Objectives contributed to by the Intermediate Outcome**						
Strengthen the organisational and institutional capacity of the private sector to drive growth						
	**Performance Targets**					
**Programme Outcome Indicators**	**Base Year**	**Base Line**	**2023/24**	**2024/25**	**2025/26**	**2026/27**
% change in annual turnover	2017/28	0	25%	30	30	30
% of businesses having a business expansion plan	2017/18	0	20%	25	25	25
% of existing businesses expanded	2017/18	0	25%	28	28	28

PRIVATE SECTOR DEVELOPMENT

Programme Outcome	Improved business capacity and local entrepreneurship skills enhanced					
**Programme Objectives contributed to by the Intermediate Outcome**						
Strengthen the organisational and institutional capacity of the private sector to drive growth						
	**Performance Targets**					
**Programme Outcome Indicators**	**Base Year**	**Base Line**	**2023/24**	**2024/25**	**2025/26**	**2026/27**
Average life of business	2017/18	0	2.6%	4.2	4.2	4.2
Global Competitiveness Index	2017/18	48.9%	54.6%	55%	55%	55%
Global Competitiveness Index, ranking	2017/18	115/140	110/141	108/141	108/141	108/141

Programme Outcome	Increased membership in chambers of commerce and trade unions					
**Programme Objectives contributed to by the Intermediate Outcome**						
Strengthen the organisational and institutional capacity of the private sector to drive growth						
	**Performance Targets**					
**Programme Outcome Indicators**	**Base Year**	**Base Line**	**2023/24**	**2024/25**	**2025/26**	**2026/27**
% of the informal sector	2017/18	51.6%	48.1%	45.00	45.00	45.00
Number of firms that are registered members of chambers of commerce	2017/18	0	800	800	800	800
Number of members in trade unions	2017/18	0	550000	750000	750000	750000

Programme Outcome	Strengthened linkages to regional and global markets					
**Programme Objectives contributed to by the Intermediate Outcome**						
Strengthen the organisational and institutional capacity of the private sector to drive growth						
	**Performance Targets**					
**Programme Outcome Indicators**	**Base Year**	**Base Line**	**2023/24**	**2024/25**	**2025/26**	**2026/27**
Value of merchandise exports (Million USD)	2017/18	0	4716.8	4973.1	4973.1	4973.1

Programme Outcome	Increased use of research and innovation instruments by the private sector					
**Programme Objectives contributed to by the Intermediate Outcome**						
Strengthen the organisational and institutional capacity of the private sector to drive growth						
	**Performance Targets**					
**Programme Outcome Indicators**	**Base Year**	**Base Line**	**2023/24**	**2024/25**	**2025/26**	**2026/27**
% of MSMEs utilizing the services of Research and innovation facilities	2017/18	0	20%	20%	20%	20%
Proportion of SMEs using digital solutions for key business processes	2017/18	0	38%	42%	42%	42%

PRIVATE SECTOR DEVELOPMENT

Programme Outcome	Increased research and innovation within the private sector					
**Programme Objectives contributed to by the Intermediate Outcome**						
Strengthen the organisational and institutional capacity of the private sector to drive growth						
	Performance Targets					
**Programme Outcome Indicators**	**Base Year**	**Base Line**	**2023/24**	**2024/25**	**2025/26**	**2026/27**
% of businesses undertaking research and development activities in the past year	2017/18	0	10%	25	25	25
% of innovative firms in manufacturing	2017/18	0	88%	90	90	90

Programme Outcome	Increased access and use of market information system by the private sector					
**Programme Objectives contributed to by the Intermediate Outcome**						
Strengthen the organisational and institutional capacity of the private sector to drive growth						
	Performance Targets					
**Programme Outcome Indicators**	**Base Year**	**Base Line**	**2023/24**	**2024/25**	**2025/26**	**2026/27**
Number of firms using market information systems	2017/18	0	5500	6000	6000	6000

Programme Outcome	Increased access and use of incubation centres by the private sector					
**Programme Objectives contributed to by the Intermediate Outcome**						
Strengthen the organisational and institutional capacity of the private sector to drive growth						
	Performance Targets					
**Programme Outcome Indicators**	**Base Year**	**Base Line**	**2023/24**	**2024/25**	**2025/26**	**2026/27**
Number of firms accessing these centres	2017/18	0	1000	1000	1000	1000

Programme Outcome	Simplified system for starting a business					
**Programme Objectives contributed to by the Intermediate Outcome**						
Strengthen the organisational and institutional capacity of the private sector to drive growth						
	Performance Targets					
**Programme Outcome Indicators**	**Base Year**	**Base Line**	**2023/24**	**2024/25**	**2025/26**	**2026/27**
Cost required to complete each procedure (% of income per capita)	2017/18	6	5%	4	4	4
Procedures to legally start and formally operate a company (number)	2017/18	8	3	3	3	3
Time required to complete each procedure (calendar days)	2017/18	4	0.1	0.1	0.1	0.1

Programme Outcome	Regionally balanced key strategic public investments planned and developed to spur private investment in key growth areas					
**Programme Objectives contributed to by the Intermediate Outcome**						
Strengthen the role of government in unlocking investment in strategic economic sectors						
	Performance Targets					
**Programme Outcome Indicators**	**Base Year**	**Base Line**	**2023/24**	**2024/25**	**2025/26**	**2026/27**
Number of private investments by UDC	2017/18	12	12	12	12	12
Proportion of Domestic private sector investment, %	2017/18	0	50.8%	50.8%	50.8%	50.8%

PRIVATE SECTOR DEVELOPMENT

Programme Outcome	Regionally balanced key strategic public investments planned and developed to spur private investment in key growth areas					
**Programme Objectives contributed to by the Intermediate Outcome**						
Strengthen the role of government in unlocking investment in strategic economic sectors						
			Performance Targets			
**Programme Outcome Indicators**	**Base Year**	**Base Line**	**2023/24**	**2024/25**	**2025/26**	**2026/27**
Total private sector investments facilitated by PPPs arrangements	2017/18	0	10	10	10	10

Programme Outcome	Increased lending to key growth sectors					
**Programme Objectives contributed to by the Intermediate Outcome**						
Sustainably lower the costs of doing business						
			Performance Targets			
**Programme Outcome Indicators**	**Base Year**	**Base Line**	**2023/24**	**2024/25**	**2025/26**	**2026/27**
% of MSMEs with an outstanding credit at a financial service provider	207/18	9.7	19.1%	23	23	23
% of SME borrowers as a share of total borrowers (Tier 1-3)	2017/18	0.75	2.5%	3.1	3.1	3.1
Non-commercial lending to the Private Sector in the key growth sectors as a % of GDP	2017/18	0	2.4%	3	3	3
Private sector credit as a % of GDP	2017/18	13.4	21.8%	27.4	27.4	27.4
Share of domestic credit to key growth sectors in total private sector credit[1]	2017/18	27.3	32.5%	33.8	33.8	33.8

Programme Outcome	Increased long-term financing to the private sector by Government owned financial institutions					
**Programme Objectives contributed to by the Intermediate Outcome**						
Sustainably lower the costs of doing business						
			Performance Targets			
**Programme Outcome Indicators**	**Base Year**	**Base Line**	**2023/24**	**2024/25**	**2025/26**	**2026/27**
Total value (UGX billions) of outstanding long-term loans (maturity above 5 years) at DFI	2017/18	556.65	832	998	998	998
Total value of private equity investments by government-owned financial institutions (UDB)- UGX billions	2017/18	0.225	0.32	0.38	0.38	0.38

Programme Outcome	Increased financing through capital markets					
**Programme Objectives contributed to by the Intermediate Outcome**						
Sustainably lower the costs of doing business						
			Performance Targets			
**Programme Outcome Indicators**	**Base Year**	**Base Line**	**2023/24**	**2024/25**	**2025/26**	**2026/27**
CIS assets under management (in UGX Trillion)	2017/18	0	0.95	1.2%	1.2%	1.2%
Domestic market capitalization due to new listings-(UGX, Billion)	2017/18	0	5.4	5.8	5.8	5.8
Domestic market capitalization to GDP	2017/18	4.45%	4.4%	5.1%	5.1%	5.1%

PRIVATE SECTOR DEVELOPMENT

Programme Outcome	Increased value of formal financial sector savings for private sector investment					
**Programme Objectives contributed to by the Intermediate Outcome**						
Sustainably lower the costs of doing business						
			Performance Targets			
**Programme Outcome Indicators**	**Base Year**	**Base Line**	**2023/24**	**2024/25**	**2025/26**	**2026/27**
Deposits in supervised financial institutions to GDP, %	2017/18	17.25	27.5%	29.50%	29.50%	29.50%
Life insurance assets to GDP, %	2017/18	0.37	0.8%	0.99	0.99	0.99
Retirement Assets to GDP	2017/18	9.47	17.1%	19.20	19.20	19.20
**Programme Outcome**	Increased access and usage of non-financial resources (certification, ICT, warehouse information system, etc.)					
**Programme Objectives contributed to by the Intermediate Outcome**						
Sustainably lower the costs of doing business						
			Performance Targets			
**Programme Outcome Indicators**	**Base Year**	**Base Line**	**2023/24**	**2024/25**	**2025/26**	**2026/27**
No. of warehouse receipt discounted at financial institutions	2017/18	0	5480	6600	6600	6600
No. of warehouse receipt traded at commodity exchanges	2017/18	0	10985	12000	12000	12000
Proportion of Key business processes automated and integrated on Government platforms	2017/18	20	65	70	70	70

Table P2.2: Intermediate Outcomes Indicators

Sub-Programme Name:	Enabling Environment					
**Intermediate Outcome Indicators:**	Growth in Private Sector Investment and Jobs in Uganda					
			Performance Targets			
**Indicators**	**Base Year**	**Base Line**	**2023/24**	**2024/25**	**2025/26**	**2026/27**
No. of Job opportunities created	2017/18	35000	51000	55250	55250	55250
**Intermediate Outcome Indicators:**	Import substitution and Export promotion					
			Performance Targets			
**Indicators**	**Base Year**	**Base Line**	**2023/24**	**2024/25**	**2025/26**	**2026/27**
New products introduced in the market	217/18	250	850	1000	1000	1000
**Intermediate Outcome Indicators:**	Increased access of certified goods to regional and international markets					
			Performance Targets			
**Indicators**	**Base Year**	**Base Line**	**2023/24**	**2024/25**	**2025/26**	**2026/27**
Number of certification permits issued	2017/18	2705	10000	12500	12500	12500
Number of product samples tested	2017/18	19796	34560	41472	41472	41472
Number of standards developed	2017/18	505	900	1030	1030	1030

PRIVATE SECTOR DEVELOPMENT

**Sub-Programme Name:**	**Enabling Environment**					
**Intermediate Outcome Indicators:**	Increased revenue to Government through taxes					
	**Performance Targets**					
**Indicators**	**Base Year**	**Base Line**	**2023/24**	**2024/25**	**2025/26**	**2026/27**
Revenue collection from Industrial parks (Billion UGX)	2017/18	400	3200	4000	4000	4000
**Intermediate Outcome Indicators:**	Improved competitiveness of the Private Sector through increased formalization					
	**Performance Targets**					
**Indicators**	**Base Year**	**Base Line**	**2023/24**	**2024/25**	**2025/26**	**2026/27**
No. of Companies registered	2017/18	23167	30000	34000	34000	34000
No. of One –Stop centers established and operationalized	2017/18	43	47	47	47	47
**Intermediate Outcome Indicators:**	Credible, safe and sound financial markets and systems					
	**Performance Targets**					
**Indicators**	**Base Year**	**Base Line**	**2023/24**	**2024/25**	**2025/26**	**2026/27**
% of Saving to GDP ratio	2017/18	16	18.5%	19.91	19.91	19.91
%ge of financially included adults (=16 years	2017/18	78	83%	85	85	85
Domestic Equity market capitalization to GDP	2017/18	4.8	4.4%	5.1	5.1	5.1
Percentage of Private Sector Credit	2017/18	13.752	18%	20	20	20
**Intermediate Outcome Indicators:**	Financial Services improved					
	**Performance Targets**					
**Indicators**	**Base Year**	**Base Line**	**2023/24**	**2024/25**	**2025/26**	**2026/27**
Interest rate spreads in Banking Sector, %	2017/18	16	12%	12	12	12
**Intermediate Outcome Indicators:**	Non-financial services support infrastructure improved					
	**Performance Targets**					
**Indicators**	**Base Year**	**Base Line**	**2023/24**	**2024/25**	**2025/26**	**2026/27**
No. of security interests registered at the movable property registry	2017/18	0	4909	5694	5694	5694
Energy losses in the electricity network, %	2017/18	16.50	13.9%	13.22%	13.22%	13.22%
**Sub-Programme Name:**	**Strengthening Private Sector Institutional and Organizational Capacity**					
**Intermediate Outcome Indicators:**	Nationals and Local Firms' Participation in Public investment programs improved					
	**Performance Targets**					
**Indicators**	**Base Year**	**Base Line**	**2023/24**	**2024/25**	**2025/26**	**2026/27**
Contractual value in public investment procurements awarded to Nationals	2017/18	0	50	50	50	50
No of Public Private Partnerships undertaken by Government	2017/18	0	2	2	2	2

P3: Medium Term Budget Allocations by Sub-Programme and Vote

PRIVATE SECTOR DEVELOPMENT

Table P3.1: Proposed Budget Allocations and Medium Term Projections by Sub-Programme

Billion Uganda Shillings	FY2022/23	2023/24	Medium Term Projections			
	Approved Budget	Proposed Budget	2024/25	2025/26	2026/27	2027/28
01 Enabling Environment	1,475.379	1,620.947	1,822.376	1,963.529	2,177.285	2,138.211
02 Strengthening Private Sector Institutional and Organizational Capacity	178.532	177.028	187.808	276.830	384.557	385.438
**Total for the Programme**	**1,653.911**	**1,797.975**	**2,010.184**	**2,240.359**	**2,561.842**	**2,523.648**

Table P3.2: Proposed Budget Allocations and Medium Term Projections by Vote

Billion Uganda Shillings	FY2022/23		2023/24	Medium Term Projections			
	Approved Budget	Spent by End Sep	Proposed Budget	2024/25	2025/26	2026/27	2027/28
008 Ministry of Finance, Planning and Economic Development	1,510.277	**94.849**	1,675.433	1,883.895	2,094.733	2,380.389	2,342.196
015 Ministry of Trade, Industry and Co-operatives	1.967	**0.439**	2.142	2.220	2.543	3.102	3.102
021 Ministry of East African Community Affairs	1.731	**0.069**	1.731	1.766	2.119	2.860	2.860
108 National Planning Authority (NPA)	0.250	**0**	0.250	0.255	0.306	0.413	0.413
119 Uganda Registration Services Bureau (URSB)	6.673	**1.296**	6.940	7.204	8.189	9.815	9.815
123 National Lotteries and Gaming Regulatory Board	0.490	**0.013**	0.490	0.499	0.599	0.809	0.809
136 Uganda Export Promotion Board (UEPB)	8.428	**0.315**	8.432	8.676	10.143	12.955	12.955
138 Uganda Investment Authority (UIA)	17.403	**1.713**	16.463	16.969	19.646	24.633	24.633
140 Capital Markets Authority	8.570	**0.675**	8.570	8.846	10.250	12.834	12.834
153 Public Procurement & Disposal of Public Assets (PPDA)	1.325	**0.228**	1.225	1.277	1.434	1.665	1.665
154 Uganda National Bureau of Standards (UNBS)	35.936	**7.859**	36.395	37.738	42.781	51.169	51.169
161 Uganda Free Zones Authority	28.357	**1.041**	11.050	11.235	13.230	17.494	17.494
162 Uganda Microfinance Regulatory Authority	15.140	**1.022**	11.490	11.749	13.871	18.207	18.207
163 Uganda Retirement Benefits Regulatory Authority	13.625	**1.505**	13.625	14.071	16.277	20.301	20.301
506 Uganda High Commission in Tanzania, Dar es Salaam	0.200	**0.050**	0.200	0.200	0.200	0.200	0.200
510 Uganda Embassy in the United States, Washington	0.790	**0.147**	0.790	0.790	0.790	0.790	0.790
515 Uganda Embassy in Japan, Tokyo	0.021	**0**	0.021	0.021	0.021	0.021	0.021
523 Uganda Embassy in Germany, Berlin	0.085	**0.003**	0.085	0.085	0.085	0.085	0.085
527 Uganda Embassy in South Sudan, Juba	0.150	**0.050**	0.150	0.150	0.150	0.150	0.150

PRIVATE SECTOR DEVELOPMENT

Billion Uganda Shillings	FY2022/23		2023/24	Medium Term Projections			
	Approved Budget	Spent by End Sep	Proposed Budget	2024/25	2025/26	2026/27	2027/28
531 Uganda Embassy in Turkey, Ankara	0.210	0.053	0.210	0.210	0.210	0.210	0.210
532 Uganda Embassy in Somalia, Mogadishu	0.050	0.020	0.050	0.050	0.050	0.050	0.050
607 Local Governments 07	2.232	0	2.232	2.277	2.732	3.689	3.689
**Total for the Programme**	**1,653.911**	**111.346**	**1,797.975**	**2,010.184**	**2,240.359**	**2,561.842**	**2,523.648**

P4: PROGRAMME PRIORITIES IN LINE WITH INTERVENTIONS FOR FY2023/24

Programme Priorities FY2023/24	NDP III Programme Intervention
Capacity building to the local contractors to enable them to participate in high-value contract Procurements.	Develop and implement a holistic local content policy, legal and institutional framework
Development of Industrial Parks across all regions in Uganda	Fully service the industrial parks and increase access to them by the local private players
Lowering Interest rates	Increase access to affordable credit largely targeting MSMEs
Reduction of certification cost. Fastracking the standing PIRT recommendation to reduce the fees from 500,000 to 100,000.	Rationalize and harmonize standards institutions, and policies at local and regional level;
The full operationalization of UIRIs Machining Manufacturing Industrial Skills Development Centre	Strengthening research and innovation capacity in support of private and public investment

P5: GENDER AND EQUITY ISSUES AND INTERVENTIONS FOR FY2023/24

Gender & Equity Issues of Concern:

1. Limited capacity for mainstreaming gender and equity issues in policies, programs and projects.
2. Limited use and availability for gender dis-aggregated data.
3. Due to Covid 19 pandemic, MSEs, which are predominantly occupied, by women and other vulnerable groups, are facing worse credit and liquidity constraints than they did prior to the pandemic;
4. Timely and expensive process of starting a small business hindering vulnerable groups especially women, PWDs, youth and other vulnerable groups from doing business

SUSTAINABLE ENERGY DEVELOPMENT

Foreword

The National Development Plan (NDPIII) (2020/21 – 2024/25) defines the broad direction for the country and sets key objectives, interventions and targets for sustainable socioeconomic transformation of Uganda while promoting a coordinated approach to achievement of the development objectives.

The Sustainable Energy Development Programme is among the programmes that have been earmarked for implementation of NDP III. The goal of the Sustainable Energy Development Programme is to meet the energy needs of the Country by providing adequate, affordable, clean and reliable energy for sustainable socio-economic growth and development. It contributes to the NDP III objective of consolidating and increasing the stock and quality of productive infrastructure. The specific objectives of the programme are;

i. Increase access and utilization of electricity;
ii. Increase generation capacity of electricity;
iii. Increase adoption and use of clean energy; and
iv. Promote utilization of energy efficient practices and technologies.

I wish to thank all the Programme Working Group stakeholders for your contribution during the preparation of this Budget Framework Paper. I call upon you all to work together to ensure the sustainable development and utilization of the energy resources for economic development and transformation of the country.

For God and My Country

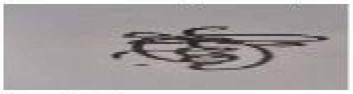

Irene Bateebe

Permanent Secretary

SUSTAINABLE ENERGY DEVELOPMENT

Abbrevations and Acronyms

ACRONYM	ACRONYM NAME
ABCs	Aerial Bundled Cables
AEC	Atomic Energy Council
CDAP	Community Development Action Plan
CGV	Chief Government Valuer
CIA	Cumulative Impact Assessment
DFID	Department for International Development
DRC	Democratic Republic of Congo
EDT	Electricity Disputes Tribunal
EMDSP	Energy and Mineral Development Strategic Plan
EPC	Engineering, Procurement and Construction
ERA	Electricity Regulatory Authority
ERD	Energy Resources Directorate
ESCOs	Energy Service Companies
ESIA	Environmental and Social Impact Assessment
GDP	Gross Domestic Product
GIZ	German Technical Cooperation
HPPs	Hydro Power Plants
HSE	Health, Safety and Environment
IAEA	International Atomic Energy Agency
IDA	International Development Agency
IFC	International Finance Cooperation
kWh	Kilowatt Hour
MEMD	Ministry of Energy and Mineral Development
MEPS	Minimum Energy Performance Standards
MFPED	Ministry of Finance Planning and Economic Development
MLHUD	Ministry of Lands, Housing and Urban Development
MoICT	Ministry of Information, Communication and Technology
MoU	Memorandum of Understanding
MW	Mega Watt
MWE	Ministry of Water and Environment
NED	Nuclear Energy Department
NEMA	National Environment Management Authority
PAPs	Project Affected Persons

SUSTAINABLE ENERGY DEVELOPMENT

ACRONYM	ACRONYM NAME
PIAPS	Programme Implementation Action Plans
PPP	Public Private Partnership
RAP	Resettlement Action Plan
REA	Rural Electrification Agency
REB	Rural Electrification Board
RESP	Rural Electrification Strategic Plan
RETs	Renewable Energy Technologies
SCADA	Supervisory Control and Data Acquisition
SDGs	Sustainable Development Goals
SHA	Shareholders Agreement
SHPP	Small Hydropower Plant
SIA	Social Impact Assessment
SUT	Supply Use Tables
UEGCL	Uganda Electricity Generation Company Limited
UETCL	Uganda Electricity Transmission Company
UIA	Uganda Investment Authority
UNBS	Uganda National Bureau of Standards
UNCST	Uganda National Council of Science and Technology
UNDP	United Nations Development Program
UNHS	Uganda National Household Survey

SUSTAINABLE ENERGY DEVELOPMENT

P1: PROGRAMME OVERVIEW

Snapshot of Medium Term Budget Allocations

Table P1.1 Overview of Programme Expenditure and Medium Term Allocations (Ush Billion)

Billion Uganda Shillings		FY2022/23		FY2023/24	MTEF Budget Projections			
		Approved Budget	Spent by End Sep	Proposed Budget	2024/25	2025/26	2026/27	2027/28
Recurrent	Wage	25.231	4.314	15.800	16.590	18.249	20.074	20.074
	Non Wage	11.009	1.177	67.220	68.433	82.579	112.747	112.747
Devt.	GoU	509.945	47.333	466.390	466.390	559.668	783.535	783.535
	ExtFin	1,030.924	0.000	673.512	1,154.885	1,398.196	1,169.630	271.941
	**GoU Total**	**546.185**	**52.824**	**549.410**	**551.413**	**660.496**	**916.355**	**916.355**
**Total GoU+Ext Fin (MTEF)**		**1,577.108**	**52.824**	**1,222.922**	**1,706.297**	**2,058.692**	**2,085.985**	**1,188.296**
	A.I.A	0.000	0.000	0.000	0.000	0.000	0.000	0.000
	**Grand Total**	**1,577.108**	**52.824**	**1,222.922**	**1,706.297**	**2,058.692**	**2,085.985**	**1,188.296**

Programme Strategy and linkage to the National Development Plan

The goal of the Sustainable Energy Development Programme is to meet the energy needs of the Country by providing adequate, affordable, clean and reliable energy for sustainable socio-economic growth and development. The Theme of NDPIII is ''Sustainable Industrialisation for Inclusive growth, employment and wealth creation''. This programme contributes to the consolidation and increasing the stock and quality of productive infrastructure as envisaged in the NDP III. The Investments in the Sustainable Energy Development Programme during the NDPIII are envisaged to drive Ugandan's Industrialization strategy since electricity has strong forward and backward linkages with agro-processing, food production, manufacturing and services. The other NDPIII Programmes use electricity as an input and would therefore benefit from increased electricity supply for agro-processing manufacturing and value addition.

P2: Highlights Of Programme Projected Performance

Table P2.1 Programme Outcomes Indicators

**Programme Outcome**	Increased electricity access					
**Programme Objectives contributed to by the Intermediate Outcome**<br>Increase access and utilization of electricity						
	Performance Targets					
**Programme Outcome Indicators**	**Base Year**	**Base Line**	**2023/24**	**2024/25**	**2025/26**	**2026/27**
Grid reliability (%)	2019/20	88%	90%	95%	98%	100%
Percentage of households with access to electricity	2019/20	24%	50%	60%	80%	90%
Primary energy consumption (million tonnes of oil equivalent)	2019/20	15.20	19	21.74	23	25
Transmission capacity of High voltage[1] transmission lines (km)	2019/20	2,354	3800	4354	4550	4600

SUSTAINABLE ENERGY DEVELOPMENT

Programme Outcome	Increased electricity consumption					
**Programme Objectives contributed to by the Intermediate Outcome**						
Increase access and utilization of electricity						
	Performance Targets					
**Programme Outcome Indicators**	**Base Year**	**Base Line**	**2023/24**	**2024/25**	**2025/26**	**2026/27**
Electricity consumption per capita (kwh per capita)	2019/20	100	400	578	600	610
Unit cost of power (USD) - cents - Extra-large	2019/20	8	5	5	5	5
Unit cost of power (USD) - cents - Large industrial consumers	2019/20	9.8	5.5	5	5	5
Unit cost of power (USD) - cents - Medium industrial consumers	2019/20	15.6	7	5	5	5
**Programme Outcome**	Increased consumption of alternative clean cooking energy					
**Programme Objectives contributed to by the Intermediate Outcome**						
Increase adoption and use of clean energy						
	Performance Targets					
**Programme Outcome Indicators**	**Base Year**	**Base Line**	**2023/24**	**2024/25**	**2025/26**	**2026/27**
% of households and institutions cooking with: (LPG, Biogas, Solar thermal applications, etc.)	2019/20	24%	40%	50%	55%	60%
Share of biomass Energy used for cooking (%)	2019/20	80%	60%	50%	45%	40%
Share of clean energy used for cooking	2019/20	15	40	50	60	70
**Programme Outcome**	Increased energy generation capacity					
**Programme Objectives contributed to by the Intermediate Outcome**						
Increase electricity generation capacity						
	Performance Targets					
**Programme Outcome Indicators**	**Base Year**	**Base Line**	**2023/24**	**2024/25**	**2025/26**	**2026/27**
Energy generation capacity (MW)	2019/20	984	2997	3500	3600	3700
**Programme Outcome**	Efficient energy utilization					
**Programme Objectives contributed to by the Intermediate Outcome**						
Promote utilization of energy efficient practices and technologies						
	Performance Targets					
**Programme Outcome Indicators**	**Base Year**	**Base Line**	**2023/24**	**2024/25**	**2025/26**	**2026/27**
% of energy wasted (Transmission and Distribution)	2019/20	19.6	14%	13%	12%	10%
Energy losses (%): Transmission and Distribution	2019/20	19.6	14%	13%	12%	10%
MW of energy saved	2019/20	6.4	13	16	19	21

Table P2.2: Intermediate Outcomes Indicators

SUSTAINABLE ENERGY DEVELOPMENT

**Sub-Programme Name:**	**Generation**					
**Intermediate Outcome Indicators:**	Increased electricity generation capacity					
	**Performance Targets**					
**Indicators**	**Base Year**	**Base Line**	**2023/24**	**2024/25**	**2025/26**	**2026/27**
Electricity generation capacity (MW)	2019/20	984	2997	3500	3600	3700
**Sub-Programme Name:**	**Transmission and Distribution**					
**Intermediate Outcome Indicators:**	Increased electricity access					
	**Performance Targets**					
**Indicators**	**Base Year**	**Base Line**	**2023/24**	**2024/25**	**2025/26**	**2026/27**
Primary energy consumption (million tonnes of oil equivalent)	2019/20	15.2	19	22	25	30
Transmission capacity of High voltage[3] transmission lines (km)	2019/20	2354	3800	4354	4500	4600
Grid reliability (%)	2019/20	90	90%	98%	99%	100%
Percentage of households with access to electricity	2019/20	24	50%	60%	80%	90%
**Intermediate Outcome Indicators:**	Reduction in Electricity Cost					
	**Performance Targets**					
**Indicators**	**Base Year**	**Base Line**	**2023/24**	**2024/25**	**2025/26**	**2026/27**
Unit cost of power (USD)- cents -Medium industrial consumers	2019/20	15.6	7	5	5	5
Unit cost of power (USD)- cents-Extra-large	2019/20	8	5	5	5	5
Unit cost of power (USD)- cents-Large industrial consumers	2019/20	9.8	5.5	5	5	5
**Sub-Programme Name:**	**Renewable Energy Development**					
**Intermediate Outcome Indicators:**	Increased consumption of alternative clean cooking energy					
	**Performance Targets**					
**Indicators**	**Base Year**	**Base Line**	**2023/24**	**2024/25**	**2025/26**	**2026/27**
% of households and institutions cooking with: (LPG, Biogas, Solar thermal applications, etc.)	2019/20	0.8	8%	10%	15%	20%
Share of biomass Energy used for cooking (%)	2019/20	85	60%	50%	45%	40%
Share of clean energy used for cooking	2019/20	15	40%	50%	60%	70%
**Intermediate Outcome Indicators:**	Increased Electricity access in rural and					
	**Performance Targets**					
**Indicators**	**Base Year**	**Base Line**	**2023/24**	**2024/25**	**2025/26**	**2026/27**
No of Sub counties electrified	2019/20	50	55	60	65	70
**Sub-Programme Name:**	**Energy Efficiency**					
**Intermediate Outcome Indicators:**	Efficient energy utilization					
	**Performance Targets**					
**Indicators**	**Base Year**	**Base Line**	**2023/24**	**2024/25**	**2025/26**	**2026/27**
MW of energy saved	2019/20	6.4	14	16	18	20

SUSTAINABLE ENERGY DEVELOPMENT

Sub-Programme Name:	Energy Efficiency					
Intermediate Outcome Indicators:	Efficient energy utilization					
	Performance Targets					
Indicators	Base Year	Base Line	2023/24	2024/25	2025/26	2026/27
Energy losses (%): Transmission and Distribution	2019/20	19.6	14%	13%	12%	10%

P3: Medium Term Budget Allocations by Sub-Programme and Vote

Table P3.1: Proposed Budget Allocations and Medium Term Projections by Sub-Programme

Billion Uganda Shillings	FY2022/23	2023/24	Medium Term Projections			
	Approved Budget	Proposed Budget	2024/25	2025/26	2026/27	2027/28
01 Generation	390.470	69.016	70.810	61.612	115.026	215.026
02 Transmission and Distribution	1,185.358	1,143.925	1,619.652	1,897.008	1,833.835	836.146
03 Renewable Energy Development	0.680	5.468	7.926	40.260	42.997	42.997
04 Energy Efficiency	0.600	4.513	7.910	59.812	94.126	94.126
**Total for the Programme**	**1,577.108**	**1,222.922**	**1,706.297**	**2,058.692**	**2,085.985**	**1,188.296**

Table P3.2: Proposed Budget Allocations and Medium Term Projections by Vote

Billion Uganda Shillings	FY2022/23		2023/24	Medium Term Projections			
	Approved Budget	Spent by End Sep	Proposed Budget	2024/25	2025/26	2026/27	2027/28
005 Ministry of Public Service		0	0.500	0.510	0.612	0.826	0.826
006 Ministry of Foreign Affairs		0	0.500	0.510	0.612	0.826	0.826
007 Ministry of Justice and Constitutional Affairs		0	1.180	1.204	1.444	1.950	1.950
008 Ministry of Finance, Planning and Economic Development		0	1.090	1.112	1.334	1.801	1.801
012 Ministry of Lands, Housing & Urban Development		0	0.500	0.500	0.600	0.840	0.840
017 Ministry of Energy and Mineral Development	1,577.108	52.824	1,211.702	1,694.945	2,045.069	2,067.318	1,169.629
150 National Environment Management Authority (NEMA)		0	2.500	2.500	3.000	4.200	4.200
154 Uganda National Bureau of Standards (UNBS)		0	4.950	5.017	6.020	8.224	8.224
**Total for the Programme**	**1,577.108**	**52.824**	**1,222.922**	**1,706.297**	**2,058.692**	**2,085.985**	**1,188.296**

P4: PROGRAMME PRIORITIES IN LINE WITH INTERVENTIONS FOR FY2023/24

SUSTAINABLE ENERGY DEVELOPMENT

Programme Priorities FY2023/24	NDP III Programme Intervention
a)Identify and Sensitize 10 up country town councils and package them for demonstration of the solar street lights.<br>b) Collect data and Develop a strategy for solar street lighting for Uganda<br>c)40 solar street lighting systems procured and installed in 10 up country towns<br>d)Conduct surveys and develop a data base for the medium scale farmers suitable for irrigation<br>e)Mobilize and support farmers with 10 solar water pumping systems<br>f)Develop a strategy for training and dissemination of the solar water pumping technologies<br>g)Conduct sensitization and awareness on solar water pumping technologies in 10 towns<br>h) 15 demonstration solar water pumping systems procured and disseminated to small scale farmers<br>i) Train technician on the installation and maintenance of solar water pumping technologies<br>j)Monitor the performance of the solar driers installed and Conduct awareness of the solar drying technologies<br>k)Conduct training of artisans on the construction of the solar driers and Carry out further research and development in the solar drying systems<br>l)Monitor the performance of the existing large solar water heating systems installed under government programmes and Identify institutions for possible demonstration of the large scale water heating systems.<br>m)Carry out awareness on the solar water heating systems and install demonstration solar water heating systems<br>n)Procuring of 2 large solar watering systems and Conduct surveys and identify communities with potential and select one community for development of the pico hydro systems.	Build local technical capacity in renewable energy solutions
Developing Net metering framework	Develop a framework for net metering
-Conducting Surveillance testing of lighting appliances .<br>-Creating Awareness on Minimum Energy Performance Standards (MEPS)<br>-Developing of Five (5) new MEPS .	Develop and enforce standards on quality of service in the energy industry
-Operationalizing of the Independent Power Transmitter.<br>-Introduction of lifeline tariff for all customers across Country.<br>-Operationalize the Direct Purchase framework for industrial customers.	Establish mechanisms to reduce the end-user tariffs.

SUSTAINABLE ENERGY DEVELOPMENT

Programme Priorities FY2023/24	NDP III Programme Intervention
Grid Expansion through the ongoing Rural electrification projects, the ministry intends to increase the national grid by constructing 1,686kms of MV and 2,747 kms of LV in the FY2023/24.<br>The Ministry is currently preparing the Electricity Access Scale-up Project that is expected to increase grid access from 19% to 44% over its five-year duration.<br>The ministry plans to implement the Grid Densification Programme Phase II different service territories. Physical Investment for schemes are projected to be at 30% completion of works.<br>An annual target of 300,000 consumer connections will be implemented in the FY2023/24<br>The Ministry is implementing the installation of a substation and the associated evacuation lines under the Rural Electrification project Muzizi B substation and Bukinda-Muzizi B, Nkusi-Muzizi evacuation lines.<br>Achieve 80% DLP monitoring for the construction of Muzizi B substation and power evacuation lines for Bukinda and lower Nkusi mini-hydropower projects in Kibaale district.<br>The Ministry intends to undertake system improvement activities like network refurbishment and continued support to service providers. Civil works under the Maintenance of Mpanga Switching Station -Phase II targets to achieve 80% physical performance in the FY2023/24.<br>The Ministry will commence implementation of a priority project to supply power to Small and Medium Enterprises in FY 2023/24	Expand and rehabilitate the distribution network including rural and hard-to-reach areas (grid expansion and densification, last mile connections, evacuation of small generation plants, quality of supply projects)
a) Construct Kabaale Substation in the Albertine Region to feed Kabaale Industrial Park<br>b) Construct Mbale, Kapeeka and Sukulu Substations and Associated Lines to feed respective Industrial Parks<br>c) Construct Njeru Substation to augment power supply to Jinja Industrial Area and its environs<br>d) Add transformation capacity at Kawanda and Mbarara substations for augmenting power supply to respective industrials Areas	Expand the transmission network to key growth economic zones (industrial and science parks, mining areas and free zones, etc.)
Surveillance testing of lighting appliances conducted;<br>Awareness creation on Minimum Energy Performance Standards (MEPS)<br>Five (5) new MEPS developed	Introduce Minimum Performance Standards for selected electrical appliances.
Awareness coverage mapped and information disseminated<br>LPG cylinders and accessories distributed<br>Construction of LPG main and regional plants completed<br>LPG wagons for Lake and rail transport acquired	Invest in LPG infrastructure

SUSTAINABLE ENERGY DEVELOPMENT

Programme Priorities FY2023/24	NDP III Programme Intervention
Conduct comparison tests of cooking technologies on Ugandan market<br>Conduct awareness on efficient cooking technologies<br>Conduct energy assessments of industrial energy consumers<br>Provide technical assistance and capacity building for Energy Management Systems among the different sectors of the economy<br>Conduct market development and awareness campaigns on efficient energy use<br>Develop the electric mobility and fuel efficiency country program<br>Develop a harmonized framework and standard for electric vehicle charging infrastructure<br>Develop complementary policy tools to promote electric mobility.	Promote the use of energy efficient equipment for both industrial and residential consumers;
Increasing the Number of households using improved cook stoves<br>Standards developed and reviewed<br>Increase the Proportion of population using alternative and efficient cooking technologies	Promote uptake of alternative and efficient cooking technologies including rural areas (electric cooking, domestic and institutional biogas and LPG);

SUSTAINABLE ENERGY DEVELOPMENT

Programme Priorities FY2023/24	NDP III Programme Intervention
Identify and Sensitize 10 up country town councils and package them for demonstration of the solar street lights.<br>Collect data and Develop a strategy for solar street lighting for Uganda<br>40 solar street lighting systems procured and installed in 10 up country towns<br>Conduct surveys and develop a data base for the medium scale farmers suitable for irrigation<br>Mobilize and support farmers with 10 solar water pumping systems<br>Develop a strategy for training and dissemination of the solar water pumping technologies<br>Conduct sensitization and awareness on solar water pumping technologies in 10 towns<br>15 demonstration solar water pumping systems procured and disseminated to small scale farmers<br>Train technician on the installation and maintenance of solar water pumping technologies<br>Monitor the performance of the solar driers installed and Conduct awareness of the solar drying technologies<br>Conduct training of artisans on the construction of the solar driers and Carry out further research and development in the solar drying systems<br>Monitor the performance of the existing large solar water heating systems installed under government programmes and Identify institutions for possible demonstration of the large scale water heating systems.<br>Carry out awareness on the solar water heating systems and install demonstration solar water heating systems<br>Procuring of 2 large solar watering systems and Conduct surveys and identify communities with potential and select one community for development of the pico hydro systems.	Promote use of new renewable energy solutions (solar water heating, solar drying, solar cookers, wind water pumping solutions, solar water pumping solutions)
Atomic Energy Act 2008 Amended<br>Energy Efficiency and Conservation Act enacted	Review the existing Acts Electricity Act 1999 and Atomic Energy Act 2008 and develop legislation for geothermal to promote exploration development and utilization of Ugandas geothermal resources for social and economic transformation and energy efficiency

SUSTAINABLE ENERGY DEVELOPMENT

Programme Priorities FY2023/24	NDP III Programme Intervention
Finalize the amendment of Atomic Energy Act, 2008.<br>Fast tracking preparatory activities for construction of the nuclear power plant including; establishment of the Centre for nuclear science and technology at Soroti University, acquisition of 30 square km of land at Buyende District.<br>Strengthen international, regional cooperation on nuclear energy.<br>Develop regulations on siting, construction, operation of nuclear power plants and for other non-nuclear power applications putting issues of gender and equity into consideration.<br>Develop and adopt international industrial codes for construction and operation of nuclear power plants putting issues of gender and equity into consideration.<br>Train Nuclear scientists Environment and Social Impact Assessment (ESIA) for the Centre for Nuclear Science and Technology<br>Develop practice specific codes of practice for operation of nuclear power plants and other non-nuclear power applications<br>Finalize a nuclear fuel supply strategy.	Seek approvals for construction of a nuclear power generation plant
Commence Technical, ESIA and RAP feasibility studies for one power plant	Undertake preliminary development of large generation plants (construction for Ayago 840 MW, feasibility for Kiba 330 MW and Oriang 392 MW)

P5: GENDER AND EQUITY ISSUES AND INTERVENTIONS FOR FY2023/24

There is unequal access to electricity and information by women, the youth and persons with disabilities in Uganda. The planned interventions include the following:

i. Continued Staff training and sensitization on gender and equity issues
ii. Allocate budgets to address the different needs of men and women, PWDs, older persons, youth and other marginalized groups.
iii. Sustain a good performance rating mark of the gender and equity provision
iv. Handling RAP issues equitably.

INTEGRATED TRANSPORT INFRASTRUCTURE AND SERVICES

Foreword

The Integrated Transport Infrastructure and Services (ITIS) Programme Budget Framework Paper (BFP) has been based on the programme priority interventions in NDP III, BCC, the ruling Government's Manifesto and the requirements outlined in the Public Finance Management Act, 2015. The preparation of this paper has been guided fully by the ITIS Program Working Group from allocation of resources up to identification of priority areas to focus on during FY 2023-24. The process of the BFP was very consultative and all program stakeholders were involved.

This BFP presents information relating to Vote 016, Vote 113, Vote 118, Vote 609 and Vote 122 with resources as detailed below;

Vote 016 - MoWT is UGX: 2,053.095
Vote 113 - UNRA is UGX: 1,911.785
Vote 118 - URF is UGX: 467.952
Vote 122 - KCCA is UGX: 196.006
Vote 609 - LGs is UGX: 27.653
Overall total UGX : 4,656.491

During FY 2023/2024, the programme will focus on delivering the critical transport infrastructure and services necessary to realize the goal of NDP III. The programme will aim at consolidating and increasing the stock and quality of productive infrastructure through reducing the average travel time, reducing freight transportation costs; increasing the stock of transport infrastructure; increasing average infrastructure life span and reducing fatality and causalities from transport accidents.

I wish to take this opportunity to thank the Government of Uganda, Development Partners and all Stakeholders for the continued support to the Programme

Bageya Waiswa

PERMANENT SECRETARY

INTEGRATED TRANSPORT INFRASTRUCTURE AND SERVICES

Abbrevations and Acronyms

ACRONYM	ACRONYM NAME
AASHTO	American Association of State Highway and Transportation Officials
AFCAC	African Civil Aviation Commission
ASTM	American Society for Testing and Materials
B2P	Bridges to Prosperity
BRT	Bus Rapid Transit
BS -EN	British Standard European Norm
CARs	Community Access Roads
CCTV	Closed-Circuit Television
CMT	Contract Management Team
CPDs	Continuing Professional Developments
DBST	Double Bituminous Surface Treatment
DLGs	District Local Governments
DLP	Defects Liability Period
DUCAR	District, Urban and Community Access Roads
EACAA	East African Civil Aviation Academy
EASA	European Union Aviation Safety Agency
ESIS	Environmental and Social Impact Screening
FTI	Fisheries Training Institute
GROW	Green Right of Way
ICAO	International Civil Aviation Organization
IWT	Inland Water Transport
KIS	Kalangala Infrastructure Services
LCS	Low Cost Sealing
MCs	Municipal Councils
MES	Mechanical Engineering Services
NBRB	National Building Review Board
NITMP	National Integrated Transport Master Plan
NMT	Non-Motorized Transport
OSBP	One Stop Border Post
PSV	Passenger Service Vehicle
PTC	Primary Teachers College
RCDS	Road Crash Database System
ROW	Right of Way

INTEGRATED TRANSPORT INFRASTRUCTURE AND SERVICES

ACRONYM	ACRONYM NAME
SAR	Search and Rescue
SEA	Strategic Environment Assessment
TC	Town Council
UCAA	Uganda Civil Aviation Academy
UCDP	Uganda Computerized Driving Permits
UIPE	Uganda Institute of Professional Engineers
URC	Uganda Railways Corporation
URF	Uganda Road Fund
VOIP	Voice over IP

INTEGRATED TRANSPORT INFRASTRUCTURE AND SERVICES

P1: PROGRAMME OVERVIEW

Snapshot of Medium Term Budget Allocations

Table P1.1 Overview of Programme Expenditure and Medium Term Allocations (Ush Billion)

Billion Uganda Shillings		FY2022/23		FY2023/24	MTEF Budget Projections			
		Approved Budget	Spent by End Sep	Proposed Budget	2024/25	2025/26	2026/27	2027/28
Recurrent	Wage	87.531	21.576	87.531	91.907	101.098	111.208	111.208
	Non Wage	649.467	76.222	595.637	607.550	729.060	984.231	984.231
Devt.	GoU	2,154.202	26.489	817.953	817.953	981.544	1,374.161	1,374.161
	ExtFin	1,409.450	178.600	3,155.372	4,203.112	6,510.105	6,537.466	2,365.730
	**GoU Total**	**2,891.200**	**124.288**	**1,501.121**	**1,517.410**	**1,811.701**	**2,469.600**	**2,469.600**
**Total GoU+Ext Fin (MTEF)**		**4,300.650**	**302.888**	**4,656.493**	**5,720.522**	**8,321.807**	**9,007.066**	**4,835.330**
	A.I.A	0.000	0.000	0.000	0.000	0.000	0.000	0.000
	**Grand Total**	**4,300.650**	**302.888**	**4,656.493**	**5,720.522**	**8,321.807**	**9,007.066**	**4,835.330**

Programme Strategy and linkage to the National Development Plan

Integrated transport infrastructure and services program contributes to the third Strategic objective under NDP3; Consolidate & increase stock and quality of Productive Infrastructure. Sustainable transport infrastructure and services plays an important role in contributing to a higher rate of economic growth leading to improvement of the country's standard of living. Infrastructure is key to integration in global and domestic trade and market systems. Productivity in virtually every sector of the economy is affected by the quality and performance of the country's transportation, water, power supply and other types of infrastructure. Therefore, access to and efficiency of transport infrastructure and services is critical to Uganda's competitiveness and ability to harness its regional and globalization potential.

With the above background, the program strategy adopted for FY 2023-24 picked directly from Strategic National Intervention Areas communicated in the 1st BCC. Specifically, the program has prioritized activities or interventions that are contributing to 'consolidating infrastructure development to reduce the cost of doing business' focus area. Care has been taken to ensure that priorities considered in the BFP are in line with reprioritized PIAP of the program. Some of the key planned outputs for FY 2023-24 include:

Continuing with DUCAR rehabilitation activities, acquisition of Right of Way for SGR, progressing with rehabilitation projects for MGR and progressing of ongoing projects under national roads.

P2: Highlights Of Programme Projected Performance

Table P2.1 Programme Outcomes Indicators

INTEGRATED TRANSPORT INFRASTRUCTURE AND SERVICES

**Programme Outcome**	Increased access to regional and international markets					
**Programme Objectives contributed to by the Intermediate Outcome**						
Increase transport interconnectivity to promote inter and intra-regional trade and reduce poverty						
	**Performance Targets**					
**Programme Outcome Indicators**	**Base Year**	**Base Line**	**2023/24**	**2024/25**	**2025/26**	**2026/27**
Ratio of Exports to GDP (%)	2021/22	15.69	15.9%	16.00	16.50	17.10
Value of exports to the region (Thousand USD) - Congo	2020/2021	317,004	330,000	340,000	350,000	370,000
Value of exports to the region (Thousand USD) - Kenya	2021/2022	390,803	500,000	600,000	700,000	800,000
Value of exports to the region (Thousand USD) - Rwanda	2021/22	974	277,000	300,000	350,000	400,000
Value of exports to the region (Thousand USD) - South Sudan	2021/2022	280,235	500,000	600,000	700,000	800,000
Value of exports to the region (Thousand USD) - Tanzania	2021/2022	94,371	120,000	150,000	170,000	200,000
**Programme Outcome**	Improved accessibility to goods and services					
**Programme Objectives contributed to by the Intermediate Outcome**						
Optimize transport infrastructure and services investment across all modes						
	**Performance Targets**					
**Programme Outcome Indicators**	**Base Year**	**Base Line**	**2023/24**	**2024/25**	**2025/26**	**2026/27**
Construction of domestic (Ro'Pax) passenger ferries	2021/2022	2	4	6	8	10
Freight Cargo -Central Corridor for Import	2021/2022	17,279.92	20000	22000	24000	26000
Freight Cargo -Central Corridor for Export	2021/2022	54,704.53	60000	70000	80000	100000
Freight Cargo Northern Corridor - Export	2021/2022	20,467.52	22,000	24,000	26,000	30,000
Freight Cargo Northern Corridor for Import	2021/2022	149,577.88	160,000	170,000	190,000	200,000
Freight transportation costs (per ton per km) - From coast (MW) to Kampala (PB) on water -US	2021/2022	0.0063	0.052	0.048	0.04	0.038
Freight transportation costs (per ton per km) - From coast to Kampala (on Rail) (in USD)	2021/2022	0.0068	0.042	0.04	0.038	0.035
Freight transportation costs (per ton per km) - From coast to Kampala (on Road) - USD	2021/2022	0.77	0.702	0.68	0.66	0.64
Freight transportation costs (per ton per km) - Inland (on Rail): USD	2021/2022	0.0035	0.0033	0.0031	0.003	0.0028
Freight transportation costs (per ton per km) - Inland (on Road): Murrum (UGX)	2021/2022	1,120	1,080	1,020	1,000	1,000
Freight transportation costs (per ton per km) - Inland (on Road): tarmac	2021/2022	681.2	620	610	600	600
Freight transportation costs (per ton per km) - Inland (on water): UGX	2021/2022	460	440	420	400	380
Number of Roll-on Roll-off vessels (international)	2021/2022	2	2	2	3	3
Paved National Roads (km)	2021/2022	5,878.50	6163	6190	7000	7100
Paved urban roads (km)	2021/2022	725	800	900	1000	1100
Permanent way /railway road (Km)	2021/2022	270	290	310	330	350

INTEGRATED TRANSPORT INFRASTRUCTURE AND SERVICES

Programme Outcome	Improved accessibility to goods and services					
**Programme Objectives contributed to by the Intermediate Outcome**						
Optimize transport infrastructure and services investment across all modes						
			**Performance Targets**			
**Programme Outcome Indicators**	**Base Year**	**Base Line**	**2023/24**	**2024/25**	**2025/26**	**2026/27**
Reduce average travel time (min/km) for district Roads	2021/2022	2.4	2.2	2.1	2.0	1.9
Reduce average travel time (min/km) for freight rail services (Days) - Mombasa -Kampala	2021/2022	14	13	12	11	10
Reduce average travel time (min/km) for Freight rail services (Days) - Southern route	2021/2022	12	11	10	9	8
Reduce average travel time (min/km) for Inland water transport (MW to PB in Hrs)	2021/2022	12.5	12	11.5	11.0	10.8
Reduce average travel time (min/km) for national roads	2021/2022	1.17	1.00	0.98	0.97	0.96
Reduce average travel time (min/km) for passenger rail services	2021/2022	0.45	0.43	0.41	0.40	0.40
Reduce average travel time (min/km) within GKMA	2021/2022	3.86	3.75	3.7	3.65	3.6
Reduce average travel time (min/km) within other Cities	2021/2022	2.57	2.5	2.4	2.3	2.2
**Programme Outcome**	Reduced cost of transport infrastructure and services					
**Programme Objectives contributed to by the Intermediate Outcome**						
Prioritize transport asset management						
			**Performance Targets**			
**Programme Outcome Indicators**	**Base Year**	**Base Line**	**2023/24**	**2024/25**	**2025/26**	**2026/27**
Development of Aids to Navigation	2021/2022	0	2	2	2	3
Development of search and rescue facilities (USD)	2021/2022	192,000	190,000	188,000	185,000	183,000
Unit cost of building transport infrastructure - Average cost for construction of unpaved/ gravel road (in million)	2021/2022	60	55	50	45	40
Unit cost of building transport infrastructure - Rehabilitation of metre gauge rail infrastructure (Bn/ Km)	2021/2022	5.6	5	4.8	4.6	4
Unit cost of building transport infrastructure - Rehabilitation/ reconstruction of paved roads (Mn/per Km)	2021/2022	1,960	1,900	1,850	1,800	1,700
Unit cost of building transport infrastructure, per Km - Upgrading roads to paved standard (Mn/per Km)	2021/2022	3,280	3,100	3,050	3,000	2,900
**Programme Outcome**	Improved National transport planning					
**Programme Objectives contributed to by the Intermediate Outcome**						
Promote integrated land use and transport planning						
			**Performance Targets**			
**Programme Outcome Indicators**	**Base Year**	**Base Line**	**2023/24**	**2024/25**	**2025/26**	**2026/27**
% Actual progress vs. planned implementation of the Programme	2021/2022	35	40%	45	50	55

INTEGRATED TRANSPORT INFRASTRUCTURE AND SERVICES

**Programme Outcome**	Longer service life of transport investment					
**Programme Objectives contributed to by the Intermediate Outcome**						
Reduce the cost of transport infrastructure and services						
	**Performance Targets**					
**Programme Outcome Indicators**	**Base Year**	**Base Line**	**2023/24**	**2024/25**	**2025/26**	**2026/27**
Average infrastructure life span (First class murram (years))	2021/2022	2	2%	2	2	2
Average infrastructure life span (Tarmac roads – (years))	2021/2022	20	20%	20	21	22
**Programme Outcome**	Improved safety of transport services					
**Programme Objectives contributed to by the Intermediate Outcome**						
Strengthen and harmonize policy, legal, regulatory and institutional framework for infrastructure and services						
	**Performance Targets**					
**Programme Outcome Indicators**	**Base Year**	**Base Line**	**2023/24**	**2024/25**	**2025/26**	**2026/27**
Total Fatalities on road transport	2021/2022	757	700	600	500	400
Fatality per 100,000 vehicles (road transport)	2021/2022	24	20	18	15	10
Serious Injuries on road transport	2021/2022	9070	9000	8500	8000	7200
Total fatalities (Water transport)	2021/2022	92	85	80	70	65
Total Fatalities on Railway Transport	2021/2022	0	0	0	0	0
**Programme Outcome**	Improved coordination and implementation of infrastructure and services					
**Programme Objectives contributed to by the Intermediate Outcome**						
Strengthen and harmonize policy, legal, regulatory and institutional framework for infrastructure and services						
	**Performance Targets**					
**Programme Outcome Indicators**	**Base Year**	**Base Line**	**2023/24**	**2024/25**	**2025/26**	**2026/27**
% of LGs in compliance to road standards	2021/2022	100	100%	100	100	100

Table P2.2: Intermediate Outcomes Indicators

**Sub-Programme Name:**	**Transport Regulation**					
**Intermediate Outcome Indicators:**	Improved compliance in the construction industry					
	**Performance Targets**					
**Indicators**	**Base Year**	**Base Line**	**2023/24**	**2024/25**	**2025/26**	**2026/27**
% of LGs in compliance to road standards	2021/2022	100	100%	100	100	100
**Intermediate Outcome Indicators:**	Improved safety of transport services					
	**Performance Targets**					
**Indicators**	**Base Year**	**Base Line**	**2023/24**	**2024/25**	**2025/26**	**2026/27**
Serious Injuries on road transport	2021/2022	9070	9000	8500	8000	7200
Total Fatalities on Railway Transport	2021/2022	0	0	0	0	0
Total Fatalities on road transport	2021/2022	757	700	600	500	400

INTEGRATED TRANSPORT INFRASTRUCTURE AND SERVICES

**Sub-Programme Name:**	**Transport Regulation**					
**Intermediate Outcome Indicators:**	Improved safety of transport services					
	**Performance Targets**					
**Indicators**	**Base Year**	**Base Line**	**2023/24**	**2024/25**	**2025/26**	**2026/27**
Total fatalities Water transport	2021/2022	92	85	80	70	65
Fatality per 100,000 vehicles (road transport)	2021/2022	24	20	18	15	10
**Sub-Programme Name:**	**Land Use and Transport Planning**					
**Intermediate Outcome Indicators:**	Improved transport planning					
	**Performance Targets**					
**Indicators**	**Base Year**	**Base Line**	**2023/24**	**2024/25**	**2025/26**	**2026/27**
% alignment of the National Transport Masterplan to the National Physical Development Plan	2021/2022	0	70%	100	100	100
% SEA Sector Level Framework recommendations applied in all transport plans and strategies	2021/22	20	30%	40	50	70
Level of implementation of the NITMP	2021/22	1	5%	10	10	10
**Sub-Programme Name:**	**Transport Infrastructure and Services Development**					
**Intermediate Outcome Indicators:**	Increased stock of transport infrastructure					
	**Performance Targets**					
**Indicators**	**Base Year**	**Base Line**	**2023/24**	**2024/25**	**2025/26**	**2026/27**
Km of National Roads Network maintained Periodic Paved	2021/2022	20	40	60	70	80
No. of domestic	2021/2022	2	4	6	8	10
No. of kms of National Roads Network maintained Periodic un Paved	2021/2022	219.74	300	350	500	600
Paved National Roads (km)	2021/2022	5878.50	6000	6050	6100	6200
Paved urban roads (km)	2021/2022	784	850	900	980	1030
Permanent way /railway road (Km)	2021/2022	270	285	300	310	325
Construction of Roll-on Roll-off vessels (international)	2021/2022	0	1	1	2	2
**Intermediate Outcome Indicators:**	Reduced average travel time					
	**Performance Targets**					
**Indicators**	**Base Year**	**Base Line**	**2023/24**	**2024/25**	**2025/26**	**2026/27**
Average travel time on district Roads (min/km)	2021/2022	2.4	2.2	2.1	2.1	2.0
Average travel time on national roads (min/km)	2021/2022	1.17	1.14	1.11	1.07	1.00
Average travel time within GKMA (min/km)	2021/2022	3.86	3.75	3.70	3.65	3.60
Average travel time within other Cities (min/km)	2021/2022	2.57	2.50	2.45	2.40	2.38

INTEGRATED TRANSPORT INFRASTRUCTURE AND SERVICES

Sub-Programme Name:	Transport Infrastructure and Services Development					
**Intermediate Outcome Indicators:**	Reduced freight transportation costs					
	Performance Targets					
**Indicators**	**Base Year**	**Base Line**	**2023/24**	**2024/25**	**2025/26**	**2026/27**
Average travel time freight rail services Mombasa -K'la (days)	2021/2022	14	13	12	11	10
Average travel time freight rail services Southern route (days)	2021/2022	12	11	10	9	8
Average travel time on inland water transport (MW to PB in Hrs.)	2021/2022	12	11	10	9	8
Average travel time on passenger rail services	2021/2022	0.45	0.43	0.42	0.40	0.38
**Intermediate Outcome Indicators:**	Reduced unit cost of building transport infrastructure					
	Performance Targets					
**Indicators**	**Base Year**	**Base Line**	**2023/24**	**2024/25**	**2025/26**	**2026/27**
Rehabilitation of meter gauge rail infrastructure (Bn/ Km)	2021/2022	5.6	5	4.8	4.6	4
Rehabilitation/ reconstruction of paved roads (Bn/per Km)	2021/2022	1960	1900	1850	1800	1700
Upgrading roads to paved standard (Bn/per Km)	2021/2022	3280	3100	3050	3000	2900
Establishment of search and rescue facilities (USD)	2021/2022	192000	190000	188,000	185,000	183,000
Average cost for construction of unpaved/ gravel road (in mn)	2021/2022	60	55	50	45	40
Establishment of Aids to Navigation	2021/2022	0	2	2	2	2
**Sub-Programme Name:**	**Transport Asset Management**					
**Intermediate Outcome Indicators:**	Increased average infrastructure life span					
	Performance Targets					
**Indicators**	**Base Year**	**Base Line**	**2023/24**	**2024/25**	**2025/26**	**2026/27**
First class murram (years)	2021/2022	2	2	2	2	2
Tarmac roads – (years)	2021/2022	20	20	20	20	20
**Intermediate Outcome Indicators:**	Increased district equipment in good working condition					
	Performance Targets					
**Indicators**	**Base Year**	**Base Line**	**2023/24**	**2024/25**	**2025/26**	**2026/27**
%ge of district equipment in good working condition	2021/2022	50	65%	70	75	80

P3: Medium Term Budget Allocations by Sub-Programme and Vote

Table P3.1: Proposed Budget Allocations and Medium Term Projections by Sub-Programme

Billion Uganda Shillings	FY2022/23	2023/24	Medium Term Projections			
	Approved Budget	Proposed Budget	2024/25	2025/26	2026/27	2027/28
01 Transport Regulation	16.553	21.692	11.420	15.420	19.200	19.200
02 Land Use and Transport Planning	549.348	326.173	514.953	836.623	2,211.522	2,084.893

INTEGRATED TRANSPORT INFRASTRUCTURE AND SERVICES

Billion Uganda Shillings	FY2022/23	2023/24	Medium Term Projections			
	Approved Budget	Proposed Budget	2024/25	2025/26	2026/27	2027/28
03 Transport Infrastructure and Services Development	2,373.256	3,569.626	4,165.527	6,264.866	5,614.619	1,569.504
04 Transport Asset Management	1,361.492	739.002	1,028.623	1,204.897	1,161.725	1,161.732
**Total for the Programme**	**4,300.650**	**4,656.493**	**5,720.522**	**8,321.807**	**9,007.066**	**4,835.330**

Table P3.2: Proposed Budget Allocations and Medium Term Projections by Vote

Billion Uganda Shillings	FY2022/23		2023/24	Medium Term Projections			
	Approved Budget	Spent by End Sep	Proposed Budget	2024/25	2025/26	2026/27	2027/28
016 Ministry of Works and Transport	876.840	**34.931**	2,053.096	2,117.773	2,953.382	2,800.394	486.137
113 Uganda National Roads Authority (UNRA)	2,687.984	**199.664**	1,911.785	2,627.984	4,349.073	5,086.534	3,332.109
118 Uganda Road Fund (URF)	487.953	**68.292**	467.952	477.391	572.590	772.226	772.226
122 Kampala Capital City Authority (KCCA)	218.220	**0**	196.006	469.720	413.578	301.455	198.401
609 Local Governments 09	29.653	**0**	27.653	27.653	33.184	46.457	46.457
**Total for the Programme**	**4,300.650**	**302.888**	**4,656.493**	**5,720.522**	**8,321.807**	**9,007.066**	**4,835.330**

P4: PROGRAMME PRIORITIES IN LINE WITH INTERVENTIONS FOR FY2023/24

Programme Priorities FY2023/24	NDP III Programme Intervention
Acquisition of right of way for SGR and for MGR projects will continue during the financial year. Additionally, 1,058 Hectares of land on 20 ongoing and planned Road Projects in the NDP III will be acquired by UNRA.	Acquire infrastructure/ utility corridors

INTEGRATED TRANSPORT INFRASTRUCTURE AND SERVICES

Programme Priorities FY2023/24	NDP III Programme Intervention
The Program will promote research and use of cost-efficient technologies through the following:<br><br>a) Low-Cost sealing trial contracts on a section of Kachumbala - Komuge, Komuge-Kakolo and Kachumbala-Atugoi roads in Bukedea District will be awarded.<br>b) Design and construction of training small bridges (2 box culverts of 2 & 6 cells respectively) on Komuge-Kakolo road in Bukedea District and Namisuni bridge on river Sironko in Gombe Budadili will be undertaken.<br>c) Training roads in Namutumba District (Magada Health Centre III to Magada Trading Centre among other road sections) will be constructed<br>d) Construction of 1 km cobblestone trial section in Bwodeya - Kota - Kiboko in Budadili Sironko District (pavement layers and paving of surfacing) will be done.<br>e) Study into the piloting of use of cobblestones for construction of roads in Uganda will be conducted.	Adopt cost-efficient technologies to reduce maintenance backlog

INTEGRATED TRANSPORT INFRASTRUCTURE AND SERVICES

Programme Priorities FY2023/24	NDP III Programme Intervention
Complete works for Entebbe Airport rehabilitation phase 1, studies and PAPs compensation for Bukasa Port and maintain upcountry aerodromes (Kidepo, Arua,Gulu, Kasese,Pakuba, Kisoro). The following outputs will be delivered:<br><br>1)Construction of 230 km equivalents on the ongoing road upgrading projects<br>2) Substantially complete the upgrading of 278 km of the following road projects and hence adding them to the paved stock of National roads.<br>a) Critical Oil Road Package 5- Masindi-Biiso, Hohwa-Nyairongo-Kyarusheesha-Butoole, And Kabaale-Kiziranfumbi Roads (97km)<br>b) Kapchorwa-Suam(73 Km)<br>c)Atiak-Laropi (66km)<br>d)Moroto-Lokitanyala Road (42km)<br>3)Commence the upgrading/construction of 302.4 km of NDP III road projects including<br>a)Kabale-Lake Bunyonyi Road(15.1km) and Kisoro-Mgahinga Road(18.1km)<br>b) Critical Oil Roads Package 6A-Kabwoya-Buhuka Road (43 km)<br>c)Critical Oil Roads Package 6B-Karugutu-Ntoroko Road(56.5Km), Link to Rwebisengo(8.2Km) and 3.3Km of Town Roads in Ntoroko<br>5)Continue with the tree planting program dubbed Green Right of Way (GROW) along national roads.<br>6)Ensure that Environmental and Social Safeguards Management in the preparation and implementation of projects meets the national and DFI standards.<br><br>The upgrading of road projects are being constructed to include Non-Motorised Traffic especially in urban areas - cycling lanes and road accesses to the main road for communities (especially cyclists, children, pregnant women, the elderly and PWDs), implementation of traffic safety plans-traffic calming like rumble strips and humps to control over speed (especially in urban areas, towns, around schools, markets and hospitals), zebra crossings around schools, hospitals, markets and in town centres, road signs to guide and warn road users, and streetlights for some urban centres. These road features lead to increased convenience and reduced security and safety risks for the communities especially the elderly, PWD, women and girls.	Construct, upgrade and climate proof strategic transport infrastructure (tourism, oil, minerals and agriculture)

INTEGRATED TRANSPORT INFRASTRUCTURE AND SERVICES

Programme Priorities FY2023/24	NDP III Programme Intervention
The program will complete and launch the National Integrated Transport Master Plan (2021-2040). Acquisition of a number of transport planning tools such as the National Transport Multi-modal transport model, modelling software, transport data system e.t.c will be done. Intense data collection for transport planning is the main focus for FY 2023-24 through surveys and development of the transport works and transport statistical system .	Develop and strengthen transport planning capacity
under local construction development, a study into the piloting of use of cobblestones for construction of roads in Uganda will be conducted; Consultancy services into the Review and update of the General specifications for Roads and Bridges will be undertaken; The use of cobblestone technology for construction of roads in Uganda will be piloted; Research study into the use of consolid technology for soil stabilization shall be undertaken; Research study into the use of road rapid soil stabilizers for construction of roads in Uganda will also be undertaken; Consultancy services for the Review and update of social safeguards policy statements and guidelines concluded and assessment of building resistance to earthquakes shall also be conducted.	Develop local construction hire pools
The program will complete and launch the National Integrated Transport Master Plan (2021-2040). Acquisition of a number of transport planning tools such as the National Transport Multi-modal transport model, modelling software, transport data system will be done.	Develop the National Transport Masterplan aligned to the National Physical Development Plan
Rail, road and water transport Safety awareness Campaigns will be conducted; Mandatory Motor Vehicle Inspections shall be conducted and transport licenses issued.	Enforce relevant transport infrastructure and services policy, legal, regulatory and institutional frameworks

INTEGRATED TRANSPORT INFRASTRUCTURE AND SERVICES

Programme Priorities FY2023/24	NDP III Programme Intervention
During FY 2023/24 Feasibility Studies and Detailed Engineering Designs of the following road upgrading projects will be undertaken in preparation for civil works: Matugga-Wakiso-Buloba (18km), Kabwohe-Bugongi-Kitagata-Kabira-Rukungiri/Ruhinda-Kashenshero-Mitooma Road (99.2km), Kapeeka Roads (188km), Kamuli-Kaliro-Pallisa (90Km), Corner Kilak-Patongo-Abim-Kotido (110Km), Arua-Ure (54Km) Noko-Obongi-Ajumani (78Km), Owaffa-Kubala-Kulikulinga (54km), Capacity Improvement and Signalisation of Namungoona-Wakiso-Kakiri Road (30km), and Capacity Improvement of Bwaise-Matugga (13km).<br><br>2) Prepare Feasibility Studies and Detailed Engineering Designs of the following road rehabilitation projects will be undertaken in preparation for civil works: Nebbi-Arua (80km), Mbarara-Ishaka (59km), and Kasese-Kilembe Mines (12km).<br><br>3) Periodic Maintenance Designs of the following road rehabilitation projects will be undertaken in preparation for civil works: Fort Portal-Bundibugyo (103km), Kampala-Gayaza-Kalagi (37km), and Hoima - Kaiso-Tonya (91km).<br><br>4) Undertake condition assessment for Kafu-Masindi (52km), Malaba/Busia-Jinja (175km), Namugongo-Seeta (15km), and Kabale-Kisoro (76km) roads<br><br>5) Feasibility Studies and Detailed Engineering Designs of the following Bridges projects will be undertaken in preparation for civil works: New Katunguru Bridge, Karuma Bridge and Masindi Port Bridge.	Implement a transport infrastructure planning and PIM system
Maintenance of 13 Aerodromes i.e Arua, Gulu, Pakuba, Mansidi, Lira, Kidepo, Moroto, Soroti, Tororo, Jinja, Mbarara, Kasese, and Kisoro will be undertaken. Final report of the feasibility study for Majanji Port and SGR Spur approved. Further, the program will continue implementing preparatory activities for SGR i.e update of feasibility studies for western and eastern routes and undertaking Environment, Social economic Impact Assessments. Additionally, 30% swamp removal, dredging and reclamation works at Bukasa Port development will be completed and Construction works on the Passenger Terminal Building under Entebbe Airport Rehabilitation phase 1 will be completed.	Implement an integrated multi-modal transportation hub (air, rail, road, water)

INTEGRATED TRANSPORT INFRASTRUCTURE AND SERVICES

Programme Priorities FY2023/24	NDP III Programme Intervention
Study into the piloting of use of cobblestones for construction of roads in Uganda will be conducted; Consultancy services into the Review and update of the General specifications for Roads and Bridges undertaken; The use of cobblestone technology for construction of roads in Uganda piloted; Research study into the use of consolid technology for soil stabilization undertaken; Research study into the use of road rapid soil stabilizers for construction of roads in Uganda undertaken; Consultancy services for the Review and update of social safeguards policy statements and guidelines concluded and assessment of building to resistance to earthquakes conducted.	Implement cost-efficient technologies for provision of transport infrastructure and services
The following Ferries Development/ construction will be undertaken<br><br>a) Substantial completion of two Bukungu-Kagwara-Kaberamaido (BKK) ferries and their landing sites<br>b) Construction of two Lake Bunyonyi Ferries and their landing sites.<br>c) Procurement and delivery of Rescue Boats<br>d) Substantial completion of construction of the landing sites for Sigulu Ferry<br><br>2)Continue to operate the eleven (11) ferries namely Nakiwogo, Kiyindi, Bisina, Masindi, Kyoga-I, Kyoga-II, Albert Nile-1, Laropi, Obongi, Amuru, and Sigulu. UNRA will ensure that the level of compliance to published ferries schedules is at least 90%.<br>3)Construction of the new Kamengo and Kamdini Weigh Stations, including High Speed Weigh-in-Motion (HSWIM) lanes.<br>UNRA Ferry services will continue to provide improved access to critical social services such as markets, hospitals and schools by the communities especially the elderly, pregnant women, children and PWDs. They also serve to link remote Island areas such as Sigulu and Lolwe to the main land, towns and cities and provide a major link between rural/ Island communities and social services providers on the main land. They therefore serve to promote equity in access to services to Island communities.<br><br>By constructing Ferries and their Landing sites, Government of Uganda, through UNRA also seeks to promote equity in development and access to critical social services such as markets, schools and hospitals by the people living on Islands and the refugees in West Nile sub region.	Increase capacity of existing transport infrastructure and services
ITIS Programme Policies will be formulated, reviewed and updated and Regulatory Impact Assessment on key ITIS Programme thematic areas will be conducted.	Monitor and evaluate transport infrastructure and services policy, legal and regulatory framework

INTEGRATED TRANSPORT INFRASTRUCTURE AND SERVICES

Programme Priorities FY2023/24	NDP III Programme Intervention
The upgrading of road projects are being constructed to include Non-Motorised Traffic especially in urban areas-cycling lanes and road accesses to the main road for communities (especially cyclists, children, pregnant women, the elderly and PWDs), implementation of traffic safety plans-traffic calming like rumble strips and humps to control over speed (especially in urban areas, towns, around schools, markets and hospitals), zebra crossings around schools, hospitals, markets and in town centres, road signs to guide and warn road users, and streetlights for some urban centres.	Provide Non-Motorized Transport infrastructure within urban areas
Under DUCAR, rehabilitation of District. Urban and Community access roads will continue under the Ministry of Works and Transport. For National Roads, focus will be put on;<br><br>1)Continue with the rehabilitation of 413 km on five road projects including<br>a) Kampala-Jinja Highway (72 Km)<br>b)Busunju-Kiboga-Hoima(145 km)<br>c) Mityana-Mubende Road (86Km) & Mityana Town Roads (14Km)<br>d) Alwii-Nebbi (33Km) and Upgrading of Packwach and Nebbi Town Roads<br>e) Olwiyo-Pakwach Road (62.5 km)<br><br>2) Procurement of civil works contractors for the following rehabilitation projects<br>a) Mbarara-Ishaka (62km)<br>b) Mbarara-Ibanda (65km)<br>c) Kikorongo-Mpondwe (38.3km)<br>d) Mubende-Kyegegwa-Kyenjojojo (89.3Km), and<br>e) Nebbi-Arua road (64km)<br>The rehabilitation road projects will be constructed to include cycling lanes and road accesses to the main road for communities (especially cyclists, children, pregnant women, the elderly and PWDs), rumble strips and humps to control over speed (especially in urban areas, towns, around schools, markets and hospitals), zebra crossings around schools, hospitals, markets and in town centres, road signs to guide and warn road users, and streetlights for some urban centres. These road features will lead to increased convenience and reduced security and safety risks for the communities especially the elderly, PWD, women and girls. The rehabilitation projects will also provide employment to the local communities including women and the youth and improve access to critical social services such as markets, hospitals and schools by the communities	Rehabilitate and maintain transport infrastructure
ITIS Programme Policies will be formulated, reviewed and updated and Regulatory Impact Assessment on key ITIS Programme thematic areas will be conducted	Review, update and develop transport infrastructure and services policies, regulations and standards and laws

INTEGRATED TRANSPORT INFRASTRUCTURE AND SERVICES

Programme Priorities FY2023/24	NDP III Programme Intervention
To conclude the approval process of the Strategic Environment Assessment (SESA) for National Integrated Transport Master Plan 2021-2040 and mainstream SESA in the Ministry and ITIS program activities.<br><br>The program will step up compliance monitoring for social issues during the planning period.<br><br>OHS communication strategy for the program will be put in place to combat diseases like COVID-19 and Ebola	Strengthen existing mechanisms to deal with negative social and environmental effects
Monitored performance of local content will be undertaken and support shall be provided to UIPE and ERB. construction and upgrade of local laboratories will continue (Hoima, Moroto and Central Materials lab in Kireka	Strengthen local construction capacity (industries, construction companies, access to finance, human resource etc.)
The program will support Kalangala Infrastructure Services and MV Kalangala operational will be kept operation during the plan period. Additionally, the following Ferries Development/ construction will be undertaken:<br><br>a) Substantial completion of two Bukungu-Kagwara-Kaberamaido (BKK) ferries and their landing sites<br>b) Construction of two Lake Bunyonyi Ferries and their landing sites.<br>c) Procurement and delivery of Rescue Boats<br>d) Substantial completion of construction of the landing sites for Sigulu Ferry<br>The program will continue to operate the eleven (11) ferries namely Nakiwogo, Kiyindi, Bisina, Masindi, Kyoga-I, Kyoga-II, Albert Nile-1, Laropi, Obongi, Amuru, and Sigulu	Upgrade transport infrastructure around L. Kyoga, Albert, Victoria and River Nile to facilitate connections

P5: GENDER AND EQUITY ISSUES AND INTERVENTIONS FOR FY2023/24

The upgrading of road projects being constructed will include Non-Motorised Traffic especially in urban areas - cycling lanes and road accesses to the main road for communities (especially cyclists, children, pregnant women, the elderly and PWDs). The program will emphasize implementation of traffic safety plans – traffic calming like rumble strips and humps to control over speed (especially in urban areas, towns, around schools, markets and hospitals), zebra crossings around schools, hospitals, markets and in town centres, road signs to guide and warn road users, and streetlights for some urban centres to ensure safety of all users. These road features lead to increased convenience and reduced security and safety risks for the communities especially the elderly, PWD, women and girls. The program projects will provide employment to the local communities including women and the youth and improve access to critical social services such as markets, hospitals and schools by the communities.

SUSTAINABLE URBANISATION AND HOUSING

Foreword

The Government of Uganda has adopted the Programme approach to planning, budgeting and implementation of government programmes effective financial year 2020.21 in the quest to achieve the NDP III goal of increasing household income and improve quality of life of Ugandans.

Under the Programme arrangement, the Ministry of Lands, Housing and Urban Development is privileged to be the Chair or Lead agency for the Sustainable Urbanization and Housing Programme.

The Budget Framework Paper is aligned to the ruling Governments Manifesto and to the Third National Planning Framework 2020.21 to 2024.25 whose Goal is "Increased Household Incomes and Improved Quality of Life of Ugandans" . The Plan presents continued commitment by the Government of Uganda to create wealth for all citizens and eradicate extreme poverty and hunger from the face of the Country by building partnerships with the Government and other Development Partners. The Budget Framework Paper shall form the basis for the preparation of the detailed annual work plans and budgets of the programme for the FY2023.24.

The Programme budget for the FY 2023/24 is Ushs 104.924bn i.e wage - Ushs 4.599bn, non-wage - Ushs 18.800bn, GoU Devt - Ushs 5.60bn and Donor Funding - Ushs 75.925bn.

It should be noted that the proposal of Ushs 104.924 billion is far below the FY 2022/23 reprioritized NDPIII programme budget of Ushs 522.17 billion and original programme budget of Ushs 840 billion.

The programme development aspirations are guided by the NDPIII and the Budget theme for FY2023.24 i.e Full Monetization of Ugandas Economy through Commercial Agriculture, Industrialization, Expanding and Broadening Services, Digital Transformation and Market Access.

During the FY 2023/24 the programme will focus on.
a) Developing and implementing integrated physical and economic development plans in the new cities and other urban areas
b) Developing, promoting, and enforcing building codes/standards
c) Implementing participatory and all-inclusive planning and implementation mechanism to enforce the implementation of land use regulatory and compliance frameworks
d) Scaling up the physical planning and urban management information system.
e) Continued implementation of infrastructure in 22 USMID MLGs and Cities and 8 Refugee hosting Districts.
f) Reviewing and enforcing urban development policies, laws, regulations, standards, and guidelines.
g) Conserving and restoring urban natural resource assets and increase urban carbon sinks

The following interventions under the Programme still remain unfunded. Capital call for Capitalization of the NHCCL (UGX 88.1bn), Development of Physical development Plans (UGX 100.00 bn), Construction of Low-cost Housing for all including Capitalisation of NHCC (UGX 350.00bn) Promote condominium property development especially in the newly created 16 Cities, KCCA and 31 Municipalities (UGX 10.00 bn), Establish a housing research and demonstration Park(UGX 15.00), Plan and develop nucleated settlements (UGX 10.00), Establishment and operationalization of Physical Planners registration board (UGX 5bn), Implementation of the NPDP (UGX 7bn), Conditional Grant for Implementation of PDPs in LGs (UGX 37bn) for 128 Districts, 580 TCs and 31 MCs; Undertake construction and rehabilitation of Public Buildings (UGX 5bn), Integrate NMT facilities in 16 cities and 31 MCs, Implement drainage master plans for 16 cities and 31 municipalities (UGX 15bn) Acquire land for devt of low cost residential houses for industrial workers (UGX 25.5bn) and Establish business engagement centres-incubators at KCCA and all the GKMA LGs (UGX 5bn), Compensation for katosi Government land(UGX 6bn), Shelter Afrique subscription (UGx 1.5bn), Operations and Functions of the NPPB (UGX 5.02bn), and Land compensation payments(UGX 372bn).

SUSTAINABLE URBANISATION AND HOUSING

[illegible]

I therefore call upon all stakeholders Political, Technical, Civil Society, Development Partners, Private Sector, Academia and well wishers to embrace this Budget Framework Paper which must guide the allocation and utilization of resources for successful implementation of the FY2023.24 work plans and request for additional budget for implementation of the above key unfunded interventions.

I thank all stakeholders for their active participation in the preparation of this Budget Framework Paper for FY 2023.24 and commend them for their commitment.

The Programme advocates for government to recognize its critical role in the national economy and therefore the need to be [illegible]

Judith Nabakooba

Minister of Lands, Housing and Urban Development

SUSTAINABLE URBANISATION AND HOUSING

Abbrevations and Acronyms

ACRONYM	ACRONYM NAME
BOU	Bank of Uganda
CEDP	Competitiveness and Enterprise Development Project
DDEG	Discretionary Development Equalization Grant
DPP	Directorate of Public Prosecution
DPPUD	Directorate of Physical Planning and Urban Development
DRMS	Domestic Revenue Mobilization Strategy
EDV	Earthquake Disaster Victims
ESIAs	Environmental, Social and Impact Assessments
GKMA	Greater Kampala Metropolitan Area
KIIDP	Kampala Capital Authority Infrastructure Development Project
km	Kilometre
LED	Local Economic Development
LIS	Land Information System
MDFs	Municipal Development Forums
NH&CC	National Housing and Construction Company
NHCC	National Housing Construction Company
NIMES	National Integrated Monitoring and Evaluation Strategy
NITA	National Information Technology Authority
NPDP	National Physical Development Plan
NPPB	National Physical Planning Board
PAP	Project Affected Persons
PPC	Physical Planning Committee
PPUMIS	Physical Planning and Urban Management Information System
PSFU	Private Sector Foundation Uganda
PST	Program Support Team
RHD	Refugee Hosting District
SMEs	Small and Medium Enterprises
SU&HP	SUSTAINABLE URBANISATION AND HOUSING
UF	Urban Forums
USMID	Uganda Support to Municipal Infrastructure Development

SUSTAINABLE URBANISATION AND HOUSING

P1: PROGRAMME OVERVIEW

Snapshot of Medium Term Budget Allocations

Table P1.1 Overview of Programme Expenditure and Medium Term Allocations (Ush Billion)

Billion Uganda Shillings		FY2022/23		FY2023/24	MTEF Budget Projections			
		Approved Budget	Spent by End Sep	Proposed Budget	2024/25	2025/26	2026/27	2027/28
Recurrent	Wage	4.599	0.941	4.599	4.829	5.312	5.843	5.843
	Non Wage	63.674	7.810	18.800	19.176	23.011	31.065	31.065
Devt.	GoU	1.908	0.010	5.600	5.600	6.720	9.408	9.408
	ExtFin	304.446	79.058	75.925	0.000	0.000	0.000	0.000
	**GoU Total**	**70.182**	**8.761**	**28.999**	**29.605**	**35.043**	**46.316**	**46.316**
**Total GoU+Ext Fin (MTEF)**		**374.628**	**87.820**	**104.924**	**29.605**	**35.043**	**46.316**	**46.316**
	A.I.A	0.000	0.000	0.000	0.000	0.000	0.000	0.000
	**Grand Total**	**374.628**	**87.820**	**104.924**	**29.605**	**35.043**	**46.316**	**46.316**

Programme Strategy and linkage to the National Development Plan

The Programme strategy is to Leverage urbanization for social economic transformation aimed at improving the underdeveloped Infrastructure to attain inclusive, productive and liveable urban areas for socioeconomic development. In FY 2023/24, the programme has prioritised the following areas guided by the Third National Development Plan and the NRM Manifesto, Presidential directives and the NDP III Mid Term Review priority areas.

i. Complete the ongoing rehabilitation and development of key urban infrastructure in all major cities and municipalities under the Uganda Support to Municipal Infrastructure Development Programme
ii. Support housing development through the dissemination of free prototype housing plans
iii. Improving physical planning and development through designation and gazetting of infrastructure corridors as well as development and enforcement of Physical Development Plans for all cities, municipalities, town councils, rural growth centres and rural settings
iv. Development of decent housing facilities starting with institutions such as Uganda Police, Uganda Peoples Defense Forces, schools and health facilities.

The above are aimed at achieving the following key results over the NDPIII timeframe are:
i. Decrease the urban unemployment rate from 14.4 percent to 9.4 percent
ii. Reduce the acute housing deficit of 2.2 million by 20 percent
iii. Decrease the percentage of urban dwellers living in slums and informal settlements from 60 percent to 40 percent
iv. Decrease the average travel time per km in GKMA from 4.1 minutes per km to 3.5 minutes per km
v. Increase the proportion of tarmacked roads in the total urban road network from 1,229.7 km to 2,459.4 km
vi. Improve the efficiency of solid waste collection from 30 percent to 50 percent

Therefore, the programme strategy is in line with the Budget strategy for FY2023.24 focused on boosting economic recovery and Enterprise resilience, facilitating economic growth and competitiveness, enhancing social development and protecting vulnerable communities, and enhancing security and good governance.

SUSTAINABLE URBANISATION AND HOUSING

P2: Highlights Of Programme Projected Performance

Table P2.1 Programme Outcomes Indicators

**Programme Outcome**	Organized urban development					
**Programme Objectives contributed to by the Intermediate Outcome**<br>Enable balanced and productive national urban system						
			**Performance Targets**			
**Programme Outcome Indicators**	**Base Year**	**Base Line**	**2023/24**	**2024/25**	**2025/26**	**2026/27**
Integrated physical and economic development plans for Cities	2019/20	1	10	12	15	15
Integrated physical and economic development plans for Districts	2019/20	6	11	30	50	70
Integrated physical and economic development plans for Municipalities	2019/20	14	22	25	31	31
Integrated physical and economic development plans for Regions	2019/20	3	4	4	4	4
Level of compliance of development projects to GKMA arrangement. %	2019/20	10%	25%	30%	35%	40%
Proportion of LG plans aligned to the National Physical Development plan	2019/20	0%	8%	22%	37%	51.8%
Ratio of land consumption rate to population growth rate	2019/20	0.38	0.4	0.42	0.44	0.46
**Programme Outcome**	Sustainable, liveable and inclusive cities					
**Programme Objectives contributed to by the Intermediate Outcome**<br>Promote green and inclusive cities and urban areas						
			**Performance Targets**			
**Programme Outcome Indicators**	**Base Year**	**Base Line**	**2023/24**	**2024/25**	**2025/26**	**2026/27**
% of Municipal solid waste disposed off safely	2019/20	45%	50%	56%	60%	70%
**Programme Outcome**	Access to decent housing					
**Programme Objectives contributed to by the Intermediate Outcome**<br>Promote urban housing market and provide decent housing for all						
			**Performance Targets**			
**Programme Outcome Indicators**	**Base Year**	**Base Line**	**2023/24**	**2024/25**	**2025/26**	**2026/27**
Housing deficit (Million)	2019/20	2,200,000	2,024,000	2000000	1800000	1650000
**Programme Outcome**	Orderly, secure and safe urban areas					
**Programme Objectives contributed to by the Intermediate Outcome**<br>Strengthen urban policies, governance, planning and finance						
			**Performance Targets**			
**Programme Outcome Indicators**	**Base Year**	**Base Line**	**2023/24**	**2024/25**	**2025/26**	**2026/27**
Compliance to physical planning regulatory framework in the urban areas (%)	2019/20	48%	58%	69.2%	75%	80%
Percentage of housing units with approved housing plans	2019/20	18%	28%	33%	38%	43%

SUSTAINABLE URBANISATION AND HOUSING

Table P2.2: Intermediate Outcomes Indicators

**Sub-Programme Name:**	**Physical Planning and Urbanization;**					
**Intermediate Outcome Indicators:**	Improved capacity of urban stakeholders in physical planning & land use, solid waste management, slum redevelopment, climate change and development control					
	**Performance Targets**					
**Indicators**	**Base Year**	**Base Line**	**2023/24**	**2024/25**	**2025/26**	**2026/27**
Number of stakeholder capacities built in core urban management practices	2020/21	30	160	260	360	460
**Intermediate Outcome Indicators:**	Integrated Regional, District, Urban and Local Physical Development Plans developed					
	**Performance Targets**					
**Indicators**	**Base Year**	**Base Line**	**2023/24**	**2024/25**	**2025/26**	**2026/27**
Number of Integrated Regional, District, Urban and Local Physical Development Plans developed	2020/21	94	120	140	160	200
**Intermediate Outcome Indicators:**	Increased compliance to the Land Use Regulatory Framework					
	**Performance Targets**					
**Indicators**	**Base Year**	**Base Line**	**2023/24**	**2024/25**	**2025/26**	**2026/27**
Level of compliance to the land use regulatory framework, %	2020/21	48%	58%	62%	65%	70%
**Intermediate Outcome Indicators:**	Increased compliance to building codes and decent housing					
	**Performance Targets**					
**Indicators**	**Base Year**	**Base Line**	**2023/24**	**2024/25**	**2025/26**	**2026/27**
Percentage compliance to building codes/standards	2021-2022	22.5%	50%	60%	70%	80%
**Intermediate Outcome Indicators:**	Increased mortgage reach					
	**Performance Targets**					
**Indicators**	**Base Year**	**Base Line**	**2023/24**	**2024/25**	**2025/26**	**2026/27**
Percentage increase in mortgage reach	2019/20	2%	11%	16%	21%	25%
**Intermediate Outcome Indicators:**	Reduced cost of housing construction					
	**Performance Targets**					
**Indicators**	**Base Year**	**Base Line**	**2023/24**	**2024/25**	**2025/26**	**2026/27**
Proportion of population adopting the new cost-efficient building technologies	2019/20	0%	10%	15%	20%	25%
**Sub-Programme Name:**	**Institutional Coordination**					
**Intermediate Outcome Indicators:**	Automated programme service delivery systems for improved planning, budgeting and financial management in all interventions					
	**Performance Targets**					
**Indicators**	**Base Year**	**Base Line**	**2023/24**	**2024/25**	**2025/26**	**2026/27**
Number of programme interventions digitally implemented	2019/20	1	6	6	8	10

SUSTAINABLE URBANISATION AND HOUSING

**Sub-Programme Name:**	**Institutional Coordination**					
**Intermediate Outcome Indicators:**	Efficient and effective programme service delivery					
	**Performance Targets**					
**Indicators**	**Base Year**	**Base Line**	**2023/24**	**2024/25**	**2025/26**	**2026/27**
Number of staff capacities built	2019/20	0	36	38	40	60
% of approved staff structure filled (441/818)	2019/20	54%	70%	75%	80%	90%
**Intermediate Outcome Indicators:**	Improved coordination of programme Plans, policies, laws and regulations with stakeholders					
	**Performance Targets**					
**Indicators**	**Base Year**	**Base Line**	**2023/24**	**2024/25**	**2025/26**	**2026/27**
Number of programme Plans and policy documents produced (BFP, MPS, Plan & Budget)	2019/20	4	4	4	4	4
**Intermediate Outcome Indicators:**	Regular and improved monitoring, supervision and evaluation of programme activities					
	**Performance Targets**					
**Indicators**	**Base Year**	**Base Line**	**2023/24**	**2024/25**	**2025/26**	**2026/27**
% compliance levels to implementation of plans and budgets	2019/20	50%	80%	85%	90%	95%
**Intermediate Outcome Indicators:**	Researches and programme performance reviews undertaken for enhanced programme performance					
	**Performance Targets**					
**Indicators**	**Base Year**	**Base Line**	**2023/24**	**2024/25**	**2025/26**	**2026/27**
Number of reforms undertaken arising from the programme researches and reviews conducted	2019/20	1	2	2	2	2

P3: Medium Term Budget Allocations by Sub-Programme and Vote

Table P3.1: Proposed Budget Allocations and Medium Term Projections by Sub-Programme

Billion Uganda Shillings	FY2022/23	2023/24	Medium Term Projections			
	Approved Budget	Proposed Budget	2024/25	2025/26	2026/27	2027/28
01 Physical Planning and Urbanization;	312.352	85.863	10.311	10.932	17.838	17.838
02 Housing Development	12.970	3.140	3.210	4.119	5.462	5.462
**Total for the Programme**	**374.628**	**104.924**	**29.605**	**35.043**	**46.316**	**46.316**

Table P3.2: Proposed Budget Allocations and Medium Term Projections by Vote

Billion Uganda Shillings	FY2022/23		2023/24	Medium Term Projections			
	Approved Budget	Spent by End Sep	Proposed Budget	2024/25	2025/26	2026/27	2027/28
011 Ministry of Local Government	1.376	0.218	1.111	1.167	1.283	1.412	1.412

SUSTAINABLE URBANISATION AND HOUSING

Billion Uganda Shillings	FY2022/23		2023/24	Medium Term Projections			
	Approved Budget	Spent by End Sep	Proposed Budget	2024/25	2025/26	2026/27	2027/28
012 Ministry of Lands, Housing & Urban Development	144.502	13.732	70.081	23.788	28.284	37.801	37.801
016 Ministry of Works and Transport	11.830	1.691	2.000	2.070	2.379	2.923	2.923
023 Ministry of Kampala Capital City and Metropolitan Affairs	0.500	0.040	2.000	2.040	2.448	3.305	3.305
161 Uganda Free Zones Authority		0	0.530	0.541	0.649	0.876	0.876
610 Local Governments 10	216.420	72.138	29.202	0.000	0.000	0.000	0.000
**Total for the Programme**	**374.628**	**87.820**	**104.924**	**29.605**	**35.043**	**46.316**	**46.316**

P4: PROGRAMME PRIORITIES IN LINE WITH INTERVENTIONS FOR FY2023/24

Programme Priorities FY2023/24	NDP III Programme Intervention
- 50 ULGs monitored and supervised to ensure wetlands and forests are preserved.<br>- 10 cities and 40 Urban LGs offered technical support and guidance on preservation issues of wetlands.<br>- Solid waste management strategy disseminated to the 22 program Municipalities and 15 Cities	Conserve and restore urban natural resource assets and increase urban carbon sinks
- Housing needs assessment carried out in 4 selected cities to guide on appropriate housing developments<br>- Institutional housing project proposal for public servants in 6 hard to reach districts i.e. Bundibudgyo, Kanungu, Adjumani, Kaabong, Amuria, Bukwo designed and developed	Design and build inclusive housing units for government workers (civil servants, police and army)

SUSTAINABLE URBANISATION AND HOUSING

Programme Priorities FY2023/24	NDP III Programme Intervention
- Housing needs assessment carried out in 4 selected cities to guide on appropriate housing developments taking into account the elderly,<br>PWDs, women, and other vulnerable groups.<br>- 1 Affordable housing project proposal for industrial workers designed and developed considering the elderly, PWDs, women, and other vulnerable groups.<br>- Implementation of the condominium property law and regulations monitored and promoted in 6 Municipalities to promote high-rise building.<br>- 24 Condominium plans vetted.<br>- Free, low-cost Prototype plans prepared and disseminated to 12 selected districts across all regions considering the elderly, PWDs, women, and other vulnerable groups.<br>- Land for construction of a housing Innovations Support Center to conduct housing research and promote alternative housing technologies identified.<br>- Slums in 4 cities (Mbale, Mbarara, Hoima and Gulu) and design strategies for redevelopment identified, mapped, and profiled<br>- 12 communities in 4 Cities (Mbale, Mbarara, Hoima and Gulu) mobilized into housing savings groups & housing cooperatives and supported	Develop and implement an investment plan for adequate and affordable housing

SUSTAINABLE URBANISATION AND HOUSING

Programme Priorities FY2023/24	NDP III Programme Intervention
- PDP for Hoima District developed and aligned to the NPDP<br>- 3 Town Council PDPs for Bulindi TC, Kitooba TC and Pakwach TC developed and aligned to the NPDP<br>- PDPs for 2 Urban centers developed i.e Kidooma Urban Centre & Katanga Urban Growth Centre<br>- Detailed plan for the area around Kabaale industrial park<br>- Hoima District prepared<br>- Kaiso-Kibiro corridor action area plan developed<br>- Physical Planning Act 2010 as amended and Physical Planning Regulations disseminated in 20 districts i.e 12 districts (Kayunga, Kalungu, Kyotera, Namisindwa, Manafwa, Tororo, Kamwenge, Kyenjojo, Rubirizi, Omoro, Nwoya and Kole)<br>- Guidelines for preparation and implementation of Physical Development Plans disseminated to 16 Districts of Bugiri, Butaleja, Kibuku, Butebo, Mityana, Kassanda, Mubende, Kyegegwa, Pader, Gulu, Amuru, Nwoya, Kumi, Ngora, Katakwi, Soroti, and Serere.<br>- 4 Stakeholder sensitizations on physical planning undertaken in 4 regions during the Ministry barazas/open days with participation of the vulnerable groups.<br>- Physical planning committees in 12 districts (Kayunga, Kalungu, Kyotera, Namisindwa, Manafwa, Tororo, Kamwenge, Kyenjojo, Rubirizi, Omoro, Nwoya and Kole) trained on various physical planning aspects.<br>- Capacity of leaders in 12 cities/urban areas built on physical planning aspects.<br>- Action area plans to protect and preserve the fragile eco systems in Mbale prepared.<br>- Implementation and development of Physical Development Plans monitored and inspected in 20 districts/DLGs (Kabale, Amuru, Oyam, Kyankwanzi, Mayuge, Lira, Mityana, Mbale, Kaliro, Iganga, Masaka, Rukungiri, Nakapiripiriti, Gulu, Masindi, Hoima, Pakwach, Gomba, Dokolo and Kapchorwa) to ensure inclusion of amenities for PWDs, children, Women and the elderly.	Develop and implement integrated physical and economic development plans in the new cities and other urban areas
- Real Estate Bill which aims to introduce the regulatory framework for the Real Estate Sector developed<br>- Architects Registration Act reviewed and amended.<br>- Budgetary Support to the Architects Registration Board (ARB) provided and monitored<br>- Subscription for staff to professional bodies of ARB, USA, SRB, ISU, ERB, UPIE, RICS, AfRES paid<br>- Sensitization on Human settlement standards conducted in 20 Selected Local Governments in the 4 regions of the country<br>- Local Government staff in 20 selected LGs in the 4 regions trained on National Housing Policy implementation strategies including implementation of the building codes and standards<br>- World Habitat Day 2023 Commemorated	Develop, promote and enforce building codes/standards

SUSTAINABLE URBANISATION AND HOUSING

Programme Priorities FY2023/24	NDP III Programme Intervention
- Capacity building of Stakeholders from 50 Urban LGs across the 4 regions on implementation of LURF carried out.<br>- 4 stakeholder engagements on the Land Use regulatory framework undertaken in the 4 regions.<br>- Land Use regulatory framework disseminated to 40 LGs in 4 regions.<br>- Training manual for inspection of land use compliance developed for law enforcement officers, physical planning committee and political leaders.<br>- Training manual for inspection of land use compliance disseminated to 40 districts across the 4 regions Monitored and evaluated compliance to the Albertine physical development plan and Northern economic corridor plan in Hoima, Kikuube, and Buliisa.<br>- Disseminated physical planning guidelines and standards in 40 Districts across all regions<br>- Toolkit/ manual for subdivisions developed<br>- Publish and disseminate the State of Land Use Compliance report 2022<br>in 40 Districts<br>- Implementation of the Land Use Regulatory Framework in 40 urban councils in the 4 regions monitored and assessed.<br>- Coordinate the preparation of GKMA Integrated Urban Development Master Plan (IUDMP).<br>- Undertake development of drainage master plan for GKMA.<br>- Coordinate implementation of actions that promote environmental Conservation and protection in GKMA.<br>- Undertake monitoring of access to social services in GKMA slums with a spatial aspect.	Implement participatory and all-inclusive planning and implementation mechanism to enforce the implementation of land use regulatory and compliance frameworks
- Support establishment of labor-intensive manufacturing, services, and projects for employment creation including development of bankable business plans.<br>- Monitoring and evaluation and surveys of GKMA activities and programs<br>- Undertake development of drainage master plan for GKMA<br>- Coordinate implementation of actions that promote environmental Conservation and protection in GKMA<br>- Undertake monitoring of access to social services in GKMA slums with a spatial aspect.	Implement the Greater Kampala Metropolitan Area Economic Development Strategy

SUSTAINABLE URBANISATION AND HOUSING

Programme Priorities FY2023/24	NDP III Programme Intervention
-Capacity of 160 Urban Managers built in best Urban Practices, Integrated Urban Development guidelines and Municipal Wide Development Strategies in 6 sub regions<br>- Build capacity of 160 urban councils in waste management in 6sub regions<br>- National Urban Transport Strategy, Street Naming & addressing guidelines to 80 Town Councils in 4 regions<br>- E-governance framework disseminated to 80 Town councils in 4 regions<br>- National Urban Sector report disseminated to 80 Town Councils in 4 regions	Review, develop and enforce urban development policies, laws, regulations, standards and guidelines
- Own Source Revenue databases rolled out to 22 MLGs<br>- Integrated revenue administration system rolled out in the 22 Municipalities (continued provision of technical support)<br>- GIS based PPUMIS implemented in the 22 Cities & MCs	Scale up the physical planning and urban management information system
- Improve Urban waste management services and associated infrastructure in the GKMA.<br>- Undertake waste to wealth initiatives which promote a circular economy in the GKMA.	Undertake waste (including faecal matter) to wealth initiatives which promote a circular economy

P5: GENDER AND EQUITY ISSUES AND INTERVENTIONS FOR FY2023/24

Issues
a) Limited mainstreaming of Gender and Equity in Program Planning, Budgeting and resource allocation.
b)Hard to reach areas hindering access and service delivery.
c)Knowledge gap in mainstreaming of Gender and Equity in the programme undertakings among staff.
d)Inadequate budget allocation for IEC materials, M&E of planned interventions and training of staff in gender mainstreaming

Interventions
1. Conducting the awareness campaigns on G&E mainstreaming
2. Develop a gender and equity profile for the profile
4. Undertake gender and equity assessment of Programs and Projects under the programme.
5. Design an assessment tool for profiling key intervention areas on G&E compliance for the programme
6. Development of administrative data systems for profiling G&E data
7. Developing the G&E strategy for the program

DIGITAL TRANSFORMATION

Foreword

I have the Honour to present the Budget Framework Paper (BFP) for the Digital Transformation Programme for FY 2023/24 as required under the Public Finance Management Act (2015).

Broadly, this BFP captures activities on Programme contributions to the Third National Development Plan (NDP III), Medium Term Policy Objectives and key priority intervention areas for FY 2022/23. It is anchored on the medium-term growth and development objectives of the Third National Development Plan which aims at consolidating the cumulative development gains of NDPs I and II.

In line with the outcome of the Midterm Review of the Third National Development Plan, the Budget Framework Paper for FY 2023/24 has been prepared to provide strategic budget interventions to implement the reprioritized PIAPs. The Budget Strategy has considered the policy interventions required to sustain recovery from the socioeconomic setback caused by COVID-19 and other natural disasters as well as harness the opportunities that come along.

This BFP articulates the Programme's contribution to the National Development Plan III Objectives the Medium-Term Sector Policy Objectives Programme challenges the performance for first half quarter FY 2022/23 summary of performance for FY 2021/22 outcome indicators and projections for the medium term and Planned Outputs for FY 2023/24.

The Programme contributes towards realization of the above aspiration through the promotion of ICT innovation and adoption of the Fourth Industrial Revolution Technologies, including artificial intelligence, internet of things and robotics. These will in essence create efficiency and effectiveness in production of goods and services. This can further be actualized through reduction of costs of doing business; increased Government revenue generation; and creation of employment opportunities in the sector.

During the FY 2023/24 the main thrust will be put promoting ICT research, innovation, and adoption of the Fourth Industrial Revolution Technologies, including Artificial Intelligence, Internet of Things and Robotics, enhancing ICT skills and vocational development, promoting development-oriented mind-set and increase government participation in strategic sectors, Extending broadband ICT infrastructure coverage countrywide in partnership with the private sector and implement last mile connectivity in public service delivery areas leveraging existing infrastructure by government and private sector players and Mainstreaming ICT in all sectors of the economy and digitizing service delivery.

It is my pleasure to present the Digital Transformation Programme Budget Framework Paper for FY 2023/2024.

Dr. Aminah Zawedde

PERMANENT SECRETARY

DIGITAL TRANSFORMATION

Abbrevations and Acronyms

ACRONYM	ACRONYM NAME
4IR	Fourth Industrial Revolution
ATIA	Access to Information Act
BUBU	Buy Uganda Build Uganda
C&ID	Communication and Information Dissemination
CCTLD	Country Code Top Level Domain
CTO	Commonwealth Telecommunications Organization
DNE	Data Networks Engineering
DTH	Direct-To-Home
DTT	Digital Terrestrial Television
EMF	Electromagnetic Field
GCIC	Government Citizen interaction Centre
GCOF	Government Communication Officers'' Forum
ICA	Integrated and Comprehensive Approach
IID	ICT Infrastructure Development
ITU	International Telecommunications Union
MCU	Media Council Uganda
NG	National Guidance
UIXP	Uganda Internet Exchange Point

DIGITAL TRANSFORMATION

P1: PROGRAMME OVERVIEW

Snapshot of Medium Term Budget Allocations

Table P1.1 Overview of Programme Expenditure and Medium Term Allocations (Ush Billion)

Billion Uganda Shillings		FY2022/23		FY2023/24	MTEF Budget Projections			
		Approved Budget	Spent by End Sep	Proposed Budget	2024/25	2025/26	2026/27	2027/28
Recurrent	Wage	16.922	2.823	16.922	17.768	19.545	21.500	21.500
	Non Wage	64.660	1.466	55.685	56.799	68.158	92.014	92.014
Devt.	GoU	38.956	2.962	24.336	24.336	29.204	40.885	40.885
	ExtFin	3.688	0.000	79.818	134.397	210.114	164.275	164.315
	**GoU Total**	**120.538**	**7.251**	**96.943**	**98.903**	**116.907**	**154.398**	**154.398**
**Total GoU+Ext Fin (MTEF)**		**124.226**	7.251	**176.762**	**233.300**	**327.021**	**318.673**	**318.713**
	A.I.A	0.000	0.000	0.000	0.000	0.000	0.000	0.000
	**Grand Total**	**124.226**	**7.251**	**176.762**	**233.300**	**327.021**	**318.673**	**318.713**

Programme Strategy and linkage to the National Development Plan

Digital Transformation (Programme 11) aims to increase ICT penetration and use of ICT services for social and economic development. The expected results relate to: increasing ICT penetration; reducing cost of ICT devices and services; creating more direct jobs in the sector; and increasing government services online.

The Programme will be pursuing the following strategies to contribute towards the NDPIII: to promote ICT innovation, to enhance ICT skills and vocational development, to promote development-oriented mind-set and to increase government participation in strategic sectors.

The Budget Framework Paper for FY 2023/24 has been prepared as guided by Vision 2040 aligned to the National Development Plan III, Presidential Manifesto (2021- 2026) and Other Planning Guidelines i.e. Gender and Equity Sustainable Development Goals. It has been prepared within the context of programmatic planning and budgeting under the NDP III and in line with the Overall Budget strategy for FY 2023/24. In line with the third National Development Plan, the Budget Framework Paper for FY 2023/24 has been prepared to provide strategic budget interventions to implement the plan. The Budget Strategy has considered the policy interventions required to sustain recovery from the socioeconomic setback caused by COVID-19 and other natural disasters as well as harness the opportunities that come along.

The Programme contributes towards realization of the above aspiration through the promotion of ICT innovation and adoption of the Fourth Industrial Revolution Technologies, including artificial intelligence, internet of things and robotics. These will in essence create efficiency and effectiveness in production of goods and services. This can further be actualized through reduction of costs of doing business; increased Government revenue generation; and creation of employment opportunities in the sector.

P2: Highlights Of Programme Projected Performance

Table P2.1 Programme Outcomes Indicators

DIGITAL TRANSFORMATION

Programme Outcome	Increased ICT usage					
**Programme Objectives contributed to by the Intermediate Outcome**						
Enhance usage of ICT in national development and service delivery						
	Performance Targets					
**Programme Outcome Indicators**	**Base Year**	**Base Line**	**2023/24**	**2024/25**	**2025/26**	**2026/27**
ICT contribution to GDP	2021/22	3.0%	3.5%	3.6%	3.8%	4.0%
National broadband coverage with minimum speed of 8 Mbps, %	2021/22	41%	50%	60%	70%	90%
Proportion of government services online	2021/22	25	35	40	80	100
**Programme Outcome**	Enhanced efficiency and productivity in service delivery					
**Programme Objectives contributed to by the Intermediate Outcome**						
Increase the ICT human resource capital						
	Performance Targets					
**Programme Outcome Indicators**	**Base Year**	**Base Line**	**2023/24**	**2024/25**	**2025/26**	**2026/27**
ICT Development Index (IDI value)	2021/22	2	2.5	3.0	3.5	4.0
ICT directly created jobs ('000s)	2021/22	30	40	50	70	90
**Programme Outcome**	Increased ICT penetration					
**Programme Objectives contributed to by the Intermediate Outcome**						
Increase the national ICT infrastructure coverage						
	Performance Targets					
**Programme Outcome Indicators**	**Base Year**	**Base Line**	**2023/24**	**2024/25**	**2025/26**	**2026/27**
Digital Terrestrial TV signal coverage	2017	50%	83%	87%	95%	98%
Fixed broad band connectivity	2017	8868	15255	17848	20882	20999
Internet penetration	2017	25%	43%	46%	50%	60%
Population covered by broadband services (%)	2017	74%	83%	87%	90%	95%
Radio signal coverage (%)	2017	80%	90%	95%	98%	98%
Unit cost of 1Mbps/month of internet	2017	237	100	90	70	50
**Programme Outcome**	Reduced costs of ICT services					
**Programme Objectives contributed to by the Intermediate Outcome**						
Promote ICT research, innovation and commercialisation of indigenous knowledge products						
	Performance Targets					
**Programme Outcome Indicators**	**Base Year**	**Base Line**	**2023/24**	**2024/25**	**2025/26**	**2026/27**
Cost of a computer -(UGX '000s)	2021/22	1550	1500	1450	1400	1400
Unit cost of 1Mbps/month of internet	2021/22	205	200	190	150	100
Unit cost of low entry smart phones (UGX '000s)	2021/22	50	50	50	50	50

DIGITAL TRANSFORMATION

**Programme Outcome**	Effective regulatory framework					
**Programme Objectives contributed to by the Intermediate Outcome**						
Strengthen the policy, legal and regulatory framework						
			**Performance Targets**			
**Programme Outcome Indicators**	**Base Year**	**Base Line**	**2023/24**	**2024/25**	**2025/26**	**2026/27**
No. of Legal and regulatory framework in place	2021/22	2	3	4	5	5
Proportion of licensees adhering to requirements (%)	2021/22	58%	60%	65%	75%	90%

Table P2.2: Intermediate Outcomes Indicators

**Sub-Programme Name:**	**ICT Infrastructure**					
**Intermediate Outcome Indicators:**	Increased coverage and access to ICTs					
			**Performance Targets**			
**Indicators**	**Base Year**	**Base Line**	**2023/24**	**2024/25**	**2025/26**	**2026/27**
Percentage of districts headquarters connected to the NBI	FY 2017/18	40%	60%	70%	90%	100%
Percentage of Parishes with broadband connectivity	2018	65%	85%	90%	90%	95%
**Sub-Programme Name:**	**E-Services**					
**Intermediate Outcome Indicators:**	Improved service delivery					
			**Performance Targets**			
**Indicators**	**Base Year**	**Base Line**	**2023/24**	**2024/25**	**2025/26**	**2026/27**
Number of transactions conducted through the shared public service delivery system (Million).	2018	0	50000000	60000000	80000000	90000000
**Intermediate Outcome Indicators:**	Increased quality of e-services					
			**Performance Targets**			
**Indicators**	**Base Year**	**Base Line**	**2023/24**	**2024/25**	**2025/26**	**2026/27**
%age of beneficiaries satisfied with the QOS over the NBI	2018	0	80%	90%	95%	95%
**Intermediate Outcome Indicators:**	Increased usage of e-services					
			**Performance Targets**			
**Indicators**	**Base Year**	**Base Line**	**2023/24**	**2024/25**	**2025/26**	**2026/27**
No. of transactions conducted through the shared public service delivery system, (mns)	2018	0	50000000	80000000	90000000	100000000
**Sub-Programme Name:**	**Research, Innovation and ICT skills development**					
**Intermediate Outcome Indicators:**	Increased research and innovation products					
			**Performance Targets**			
**Indicators**	**Base Year**	**Base Line**	**2023/24**	**2024/25**	**2025/26**	**2026/27**
Number of Innovations supported by Government and commercialized	2019	72	42	40	50	60
Number of Regional Hubs established by GOU	2021	1	4	4	6	8

DIGITAL TRANSFORMATION

Sub-Programme Name:	Enabling Environment					
Intermediate Outcome Indicators:	Increased ICT human resource capacity					
	Performance Targets					
Indicators	Base Year	Base Line	2023/24	2024/25	2025/26	2026/27
ICT directly created jobs	2021/2022	30	35	40	50	60

P3: Medium Term Budget Allocations by Sub-Programme and Vote

Table P3.1: Proposed Budget Allocations and Medium Term Projections by Sub-Programme

Billion Uganda Shillings	FY2022/23	2023/24	Medium Term Projections			
	Approved Budget	Proposed Budget	2024/25	2025/26	2026/27	2027/28
01 ICT Infrastructure	29.133	102.813	157.591	238.612	197.646	200.142
02 E-Services	12.252	9.195	9.781	12.430	8.103	15.328
03 Research, Innovation and ICT skills development	39.108	16.110	16.210	16.240	19.210	19.509
04 Enabling Environment	43.733	48.645	49.718	59.739	93.714	83.735
**Total for the Programme**	**124.226**	**176.762**	**233.300**	**327.021**	**318.673**	**318.713**

Table P3.2: Proposed Budget Allocations and Medium Term Projections by Vote

Billion Uganda Shillings	FY2022/23		2023/24	Medium Term Projections			
	Approved Budget	Spent by End Sep	Proposed Budget	2024/25	2025/26	2026/27	2027/28
020 Ministry of ICT and National Guidance	69.400	4.505	46.105	46.807	55.569	74.542	74.542
126 National Information Technologies Authority	49.977	2.746	130.657	186.493	271.452	244.131	244.171
**Total for the Programme**	**124.226**	**7.251**	**176.762**	**233.300**	**327.021**	**318.673**	**318.713**

P4: PROGRAMME PRIORITIES IN LINE WITH INTERVENTIONS FOR FY2023/24

Programme Priorities FY2023/24	NDP III Programme Intervention
undertake ICT training for professionally certified courses and specialized short courses at UICT.	Develop a well-grounded ICT professional workforce
Certification framework to regulate ICT professional standards developed	Develop an ICT professional's quality assurance framework
Conduct consultations on ICT research needs and priorities	Develop and implement ICT Research and Innovation ecosystem
Ensure Personal data protection and privacy. Conduct Personal data protection and privacy awareness.	Develop and implement the Data Protection and Privacy Programme
Enhance the infrastructure capacity of the institute to support specialised ICT training.	Develop ICT centres of excellence and vocational institutions

DIGITAL TRANSFORMATION

Programme Priorities FY2023/24	NDP III Programme Intervention
Establish supporting Infrastructure for the National ICT Park which include Internet	Develop Innovation and incubation Centres
Upgrade of existing National Data Centre Infrastructure and Disaster Recovery site	Establish and enhance national common core infrastructure (data centres, high power computing centres, specialized labs)
Implement Last mile connectivity and Uganda Digital Acceleration Program to expand access to affordable high-speed internet through the NBI	Extend broadband ICT infrastructure coverage countrywide in partnership with the private sector and all Government entities and implement last mile connectivity to key areas (Districts, sub-counties, schools, hospitals, post offices, tourism sites, police, LGs etc.)
Review the national postcode system to upgrade it to a digital layout.<br>Training and awareness on the system.<br>Rollout the addressing system to facilitate service delivery (postal, ecommerce, ambulance services, fire brigade, security patrol, tax collection)	Implement the national addressing system
Services (government & non-government) provided through the postal outlets. Expand provision of all e-government services through postal outlets.	Leverage the existing Government infrastructure to deliver public and private services
Carry out ICT needs assessment/mapping carried in key Sector of Education, Health, Trade and Industry, Gender and Agriculture	Mainstream ICT in all sectors of the economy and digitize service delivery
Operationalise the ICT infrastructure blueprint<br>Carry out a comprehensive ICT infrastructure Audit that takes into account infrastructure of Utility providers	Regulate, coordinate and harmonize ICT infrastructure planning, sharing and deployment within the public and private sector
Carry out review and develop appropriate policies, strategies, standards and regulations that respond to industry needs	Review and develop appropriate policies, strategies, standards and regulations that respond to industry needs
Conduct Cyber Security awareness in entities<br>Conduct Information security assurance in MDAs/LGs<br>Implement Information risk management	Strengthen Cyber Security in the country
Establish supporting Infrastructure for the National ICT Park which include Internet connectivity, Roads and Utilities (Water, Sewerage, electricity)	Support local innovation and promote export of knowledge products

P5: GENDER AND EQUITY ISSUES AND INTERVENTIONS FOR FY2023/24

The Programme will prioritize participation of women and differently abled persons in ICT capacity building activities, Coordinate Gender training and awareness campaigns on cyber security, Gender and Equity issues will be taken care of in the segmented groups during sensitization and work shop awareness activity programs.

As regards to equity, the programme is set to Extend broadband ICT infrastructure coverage countrywide in partnership with the private sector and all Government entities and implement last-mile connectivity to key areas (Districts, sub-counties, schools, hospitals, post offices, tourism sites, police, LGs etc.). will also deploy Wi-Fi hotspots to cover the fifty (50) sites across the country.

HUMAN CAPITAL DEVELOPMENT

Foreword

The Human Capital Development Programme Budget Framework Paper (HCDP BFP) is an amalgamation of BFPs for Votes under the Human Capital Development Programme which include: 013 - Education and Sports and votes under it; 014 – Health and its other votes; 018 - Gender Labour and Social Development and its other Votes; and 019 – Water and Environment and its other Votes for FY 2023/24.
The Human Capital Development Programme primarily contributes to the National Development Plan III objective 4 which focuses on "Enhancing the Productivity and Social Well-being of the Population". The programme also contributes to NDP III objectives 1 and 2.
Accordingly, the programme BFP for FY 2023/24 has been aligned to the 11 programme strategic result areas highlighted in the NDP III and interventions of the Programme Implementation Action Plan (PIAP).

In FY 2023/24, the Votes under the Human Capital Development Programme have a Projected budget of Ushs. 9,005.61bn: of which Ushs. 3,966.19bn is wage, Ushs. 1,969.58bn is non-Wage recurrent, while Ushs. 1,005.56bn is domestic development and Ushs. 2,064.27bn is external financing.

During the 1st Quarter, the Votes under the Programme had spent a total of UShs 1,194.03bn of the Ushs. 1,379.38bn released. Of the expenditure Ushs. 800.37bn was wage, Ushs. 390.80bn was non-wage recurrent, while Ushs. 2.86bn was domestic development.

For FY 2023/24, the total Programme budget allocation is Ushs. 9,005.61bn compared to the current allocation of Ushs.9,089.54bn implying an overall reduction of Ushs. 83.94bn.

With the above budget allocation, the Programme will focus on the following key priorities aligned to Programme Implementation Action Plan result areas:

Education, Sports and Skills Sub Programme will focus on the following among others: establishment of the National Teachers' Council; Construction, expansion and equipment of Technical Institutes; Roll out of the redeveloped EMIS; dissemination of the National Physical Education and Sports Policy; Operationalization of the High-Altitude Training Center, Teryet; and, Finalisation of the National School Feeding policy.

Health Sub Programme will focus on the following among others: Mobilization of resources for Immunization; Development and dissemination of the Public-Private Partnerships for Health Strategic plan 2020-2023; Sustainability of maternal HIV testing and maternal ART coverage at over 95%; Increased retention on the treatment of HIV positive pregnant and lactating women; Prevention of new infections among young previously HIV negative women; Improving adolescent health services and Fast tracking of the approval & implementation of the Adolescent Health Policy; Continued training of health workers on Integrated Management of Childhood Illnesses (IMCI); maintenance of medical equipment and recruitment of Biomedical Engineers and improvement of infrastructure including staff accommodation.

Gender, Labour and Social Development Sub Programme will focus on the following among others: Development of the Uganda National Policy on Labour Productivity; Development of the Uganda National Labour Productivity Enhancement Strategy and Plan, 2022-2032; Review of Worker's Compensations Regulations; Empowerment of 21,356 youth units to participate in Parish Development Model and other government development; Conducting of 960 Labour Inspections in all sectors of the Economy on compliance to Labour Standards and Implementation of the National Employment Strategy in 60 local governments.

Water and Environment Sub Programme will focus on the Completion of construction of Kyenshama & Kyemamba multi-purpose dams in Mbarara and Lyantonde; Geregere multi-purpose dam in Agago District to 10% progress; a multi-purpose earth dam in Eastern Region to 30%; Kawumu irrigation scheme in Luweero District to 40% and Construction of Six (6)

HUMAN CAPITAL DEVELOPMENT

[illegible] earth dam in Eastern Region to 30%; Kawumu irrigation scheme in Luweero District to 40% and Construction of Six (6) Medium Scale Irrigation Schemes in Western, Central and Northern Regions, Busoga, Bukedi and Teso Sub-regions to 30% cumulative progress.

The programme suffered an 80% budget cut on subventions translating into a shortfall of Ushs. 382.54bn. This budget cut will affect the implementation of key interventions.
I, therefore, submit the Programme Budget Framework Paper for FY 2023/24 on behalf of the Votes under Human Capital Development Programme for approval. I also appeal that the Ushs. 382.54bn for is reinstated to enable the facilitation of [illegible]

Ketty Lamaro

Permanent Secretary

HUMAN CAPITAL DEVELOPMENT

Abbrevations and Acronyms

ACRONYM	ACRONYM NAME
ACP	AIDS Control Programme
ADB	African Development Bank
AHSPR	Annual Health Sector Performance Report
AIM	AIDS Integrated Management
CHEWS	Community Health Extension Workers
CME	Continuing Medical Education
COCTU	Coordinating Officer for the Control of Trypanosomiasis in Uganda
CPD	Continuing Professional Development
CRCs	Child Rights Clubs
CRPD	Convention on the Rights of Persons with Disabilities
CSO	Civil society organizations
CYP	Couple Years of Protection
DANIDA	Danish International Development Assistance
DBFP	District Budget Framework Paper
DDT	Dichlorodiphenyltrichloroethane
DHT	District Health Team
EDP	Epidemic and Disease Prevention, Preparedness and Response
EIA	Environmental Impact Assessment
EMS	Emergency Medical Services
EPI	Expanded Programme on Immunization
GAIN	Global Alliance for Improvement of Nutrition
GAM	Global Acute Malnutrition
GDF	Global Drug Fund
HC	Health Centre
HCT	HIV/AIDS Counseling and Testing
HFs	Health Facilities
HQs	Headquarters
HRHIS	Human Resource Information System
HSV 2	Herpes Simplex Virus type 2
HTC	Health Training College
HTIs	Health Training Institutions
ICN	International Council of Nursing
ICU	Intensive Care Unit

HUMAN CAPITAL DEVELOPMENT

ACRONYM	ACRONYM NAME
ILO	International LabourOrganisation
IMCI	Integrated Management of Childhood Illness
IMR	Infant Mortality Rate
IPFs	Indicative Planning Figures
IPT	Intermittent Preventive Treatment
IRS	Indoor Residual Spraying
ISCC	Inter-Ministerial Standing Coordinating Committee (education and health)
ISH	Integrated Sanitation and Hygiene
ISS	Integrated Support Supervision
IST	In-service training
IT	Information Technology
ITA	International Training Advice
ITNs	Insecticide Treated Nets
IUCEA	Inter University Council for East Africa
IVM	Integrated Vector Management
IYCF	Infant and Young Child Feeding
JAB	Joint Admissions Board
JAF	Joint Assessment Framework
JICA	Japan International Cooperation Agencies
JMS	Joint Medical Stores
JRM	Joint Review Missions
KCC	Kampala City Council
KCCA,	Kampala Capital City Council Authority
KIU	Kampala International University
KNRC	Kids in Need Resource Centre
LLITNs	Long Lasting Insecticide Treated Nets
LRA	Lords Resistance Army
LTIA	Long Term Institutional Arrangements
MC	Municipal Council
MCP	Malaria Control Programme
MDGs	Millennium Development Goals
MGLSD	Ministry of Gender Labour and Social Development
MR	Mortality Rate
MUK	Makerere University
NAPBHRs	National Action Plan on Business and Human Rights

HUMAN CAPITAL DEVELOPMENT

ACRONYM	ACRONYM NAME
NCD	Non-Communicable Diseases
NDA	National Drug Authority
NHIS	National Health Insurance Scheme
NHP	National Health Policy
NTLP	National Tuberculosis and Leprosy Control Program
NWC	National Women Council
OFID	OPEC Fund for International Development
PC	Partnership Committee
PFMA	Public Finance Management Act
PFMA	Public finance and Management
PHP	Private Health Practitioners
PMI	Presidential Malaria Initiative
PWDs	People With Disabilities
RMNCAH	Reproductive maternal, newborn, child and adolescent health
SAGE	Social Assistance Grants Empowerment
SOPs	Standard Operating Procedures
STEM/STEI	Science Technology Engineering Mathematics/Science Technology Engineering and Innovation
STI	Sexually Transmitted Infection
SWAP	Sector-Wide Approach
TB	Tuberculosis
THETA	Traditional Healers and Medical Practitioners Together Against HIV/AIDS
UBTS	Uganda Blood Transfusion Services
UCE	Uganda Certificate of education
UCG	Uganda Clinical Guidelines
UDHS	Uganda Demographic and Health Survey
UNF	Uganda National Formulary
UNSA	Uganda National Students Association
UPOLET	Universal Post Ordinary Level Education and Training
URCI	Urban Rabies Control Initiative
UVRI	Uganda Virus Research Institute
VAW/G	Violence Against Women and Girls
WFP	World Food Programme

HUMAN CAPITAL DEVELOPMENT

P1: PROGRAMME OVERVIEW

Snapshot of Medium Term Budget Allocations

Table P1.1 Overview of Programme Expenditure and Medium Term Allocations (Ush Billion)

Billion Uganda Shillings		FY2022/23		FY2023/24	MTEF Budget Projections			
		Approved Budget	Spent by End Sep	Proposed Budget	2024/25	2025/26	2026/27	2027/28
Recurrent	Wage	3,385.865	800.365	3,966.192	4,322.629	4,754.891	5,230.381	5,230.381
	Non Wage	2,354.079	390.799	1,969.576	2,696.190	3,260.748	4,406.048	4,406.048
Devt.	GoU	1,127.102	2.864	1,005.563	1,138.545	1,366.254	1,912.755	1,912.755
	ExtFin	2,222.493	81.913	2,064.273	935.382	849.496	236.346	57.510
	**GoU Total**	**6,867.046**	**1,194.029**	**6,941.332**	**8,157.363**	**9,381.893**	**11,549.184**	**11,549.184**
	**Total GoU+Ext Fin (MTEF)**	**9,089.539**	**1,275.942**	**9,005.605**	**9,092.745**	**10,231.390**	**11,785.530**	**11,606.694**
	A.I.A	0.000	0.000	0.000	0.000	0.000	0.000	0.000
	**Grand Total**	**9,089.539**	**1,275.942**	**9,005.605**	**9,092.745**	**10,231.390**	**11,785.530**	**11,606.694**

Programme Strategy and linkage to the National Development Plan

The Human Capital Development Program (HCDP) mainly contributes to the NDPIII objective four which is to: enhance the productivity and social wellbeing of the population. It also contributes to other objectives including (1), which is to: Enhance value addition in Key Growth Opportunities and (2) which is to: Strengthen private sector capacity to drive growth and create jobs among others.

Objective 4 of the NDP III emphasizes the role of the four thematic areas of health, education and Sports, Water and Gender towards increasing productivity, inclusiveness and wellbeing of population for wealth creation. The programme goal is to increase productivity of the population through strategic investment in the people to enable them work productively and competitively to achieve a rising quality of life for all, in line with the Vision 2040.
Specifically, the programme contributes to the following NDPIII key strategic results:

a) Increased youth employment
b) Increased employer satisfaction with the TVET training
c) Increased ratio of STEI/STEM graduates to Humanities
d) Increased proportion of training institutions meeting the basic requirements and minimum standards
e) Increased life expectancy
f) Reduced neonatal, infant, under 5 and maternal mortality rates
g) Reduced fertility rate
h) Increased primary and secondary school survival and transition rates
i) Increased quality adjusted years of schooling
j) Increased literacy rate
k) Increased proportion of the population participating in sports and physical exercises

A reprioritized Programme Implementation Action Plan (PIAP) details actions to deliver the NDP III interventions towards each of the six objectives under the four subprogrammes of Education, Sports and Skills Development; Population health, safety and management; Labour and employment services and Gender and social protection. The Planned outputs and proposed allocations for the FY 2023/24 have been aligned towards achievement of the actions in the revised PIAP.

HUMAN CAPITAL DEVELOPMENT

proposed allocations for the FY 2023/24 have been aligned towards achievement of the actions in the revised PIAP.

The Programme has also costed its unfunded areas with an aim of meeting the targets set in the National Development Plan

P2: Highlights Of Programme Projected Performance

Table P2.1 Programme Outcomes Indicators

**Programme Outcome**	Improved Learning outcomes						
**Programme Objectives contributed to by the Intermediate Outcome**							
To improve the foundations for human capital development							
	**Performance Targets**						
**Programme Outcome Indicators**	**Base Year**	**Base Line**	**2023/24**	**2024/25**	**2025/26**	**2026/27**	
Average years of schooling	2020/21	6.2	60%	7.3	8	8.5	
Gross Enrolment Ratio - Pre-Primary	2020/21	16.6	25.5	26.5	27	28.5	
Gross Enrolment Ratio - Primary	2020/21	111	106.6	105.4	104.2	102	
Gross Enrolment Ratio - Secondary	2020/21	26	41.25	44.6	47.95	51.4	
Net Enrolment Ratio - Pre-Primary	2021/22	17.8	21.7	24.1	26.5	29.0	
Net Enrolment Ratio - Primary	2021/22	94.9	96.2	97.0	97.9	98.7	
Net Enrolment Ratio - Secondary	2021/22	32.12	36.07	39.1	42.0	45.1	
Proficiency in Literacy, % - P.3	2021/22	58.2	60.9%	63.2	65.4	67.6	
Proficiency in Literacy, % - P.6	2021/22	62.04	65.4%	68.1	70.7	73.4	
Proficiency in Numeracy, % - P.3	2021/22	62.88	65.7%	67.8	70.0	72.2	
Proficiency in Numeracy, % - P.6	2021/22	62.4	66.9%	70.2	73.6	77.1	
Proportion of schools/ training institutions and programmes attaining the BRMS[1], % - Pre-Primary (registered)	2021/22	34.4	39.9%	44.3	48.7	53.3	
Proportion of schools/ training institutions and programmes attaining the BRMS[1], % - Primary (SACMEQ)	2021/22	60.5	64.3%	67.2	70.2	73.2	
Proportion of schools/ training institutions and programmes attaining the BRMS[1], % - Secondary (DES)	2021/22	52.8	53.2%	54.2	55.6	56.9	
Proportion of schools/ training institutions and programmes attaining the BRMS[1], % - Universities (accredited programmes)	2021/22	58.9	64.2%	68.5	72.9	77.4	
Quality adjusted years of schooling	2020/21	4.6	4.8%	5.1	5.3	5.5	
Science pass rates[2] (O-level)	2021/22	57.0	57.9%	58.1	58.3	58.5	
Survival rates, % - Primary	2021/22	38.9	41.6%	43.0	44.5	46.0	
Survival rates, % - Secondary	2021/22	82.1	85.1%	87.1	89.2	91.3	
Transition from P.7 to S.1	2022/23	68	70.6	72.7	75	77.2	

HUMAN CAPITAL DEVELOPMENT

**Programme Outcome**	Child development in learning health and psychological wellbeing improved					
**Programme Objectives contributed to by the Intermediate Outcome**						
To improve the foundations for human capital development						
	**Performance Targets**					
**Programme Outcome Indicators**	**Base Year**	**Base Line**	**2023/24**	**2024/25**	**2025/26**	**2026/27**
Percentage of children aged 5-17 years engaged in child labour	2021	40	38%	37.5	37	36.5
Prevalence of under 5 Stunting, %	2020/21	29%	21%	19%	19%	19%
**Programme Outcome**	Improved health, income and national image					
**Programme Objectives contributed to by the Intermediate Outcome**						
To promote sports, recreation, and physical education						
	**Performance Targets**					
**Programme Outcome Indicators**	**Base Year**	**Base Line**	**2023/24**	**2024/25**	**2025/26**	**2026/27**
Uganda's ranking in niche sports (football, athletics, netball, boxing, etc.) - Athletics	2021/22	16	14	12	10	9
Uganda's ranking in niche sports (football, athletics, netball, boxing, etc.) - Football	2021/22	90	85	80	75	70
Uganda's ranking in niche sports (football, athletics, netball, boxing, etc.) - Netball	2021/22	6	5	5	4	4
Uganda's ranking in niche sports (football, athletics, netball, boxing, etc.) - Rugby	2021/22	44	35	30	35	30
**Programme Outcome**	Reduced Morbidity and Mortality of the population					
**Programme Objectives contributed to by the Intermediate Outcome**						
Improve population health, safety and management						
	**Performance Targets**					
**Programme Outcome Indicators**	**Base Year**	**Base Line**	**2023/24**	**2024/25**	**2025/26**	**2026/27**
Annual Cancer Incident Cases	2020/21	80000	40000	40000	40000	40000
Annual Cardiovascular Incident cases	2020/21	7000	4000	4000	4000	4000
Hepatitis B incidence per 100,000 population	2020/21	60	35	30	30	30
Incidence of Road accidents per 1,000	2020/21	2348	1400	1200	1200	1200
Malaria incidence per 1,000 population	2020/21	293	170	147	147	147
Maternal Mortality ratio (per 100,000)	2020/21	336	315	299	299	299
Mortality rate Attributed to unsafe water, unsafe sanitation, and lack of hygiene (per 100,000)	2020/21	54	45	43	43	43
Neonatal Mortality Rate (per 1,000)	2020/21	27	13	10	10	10
Number of new HIV infections per 1,000 susceptible population	2020/21	60	40	30	30	30
Reduce mortality due to AIDS	2020/21	60	40	30	30	30
Reduce mortality due to Malaria	2020/21	60	40	30	30	30
Reduce mortality due to TB	2020/21	60	40	30	30	30

HUMAN CAPITAL DEVELOPMENT

**Programme Outcome**	Reduced Morbidity and Mortality of the population						
**Programme Objectives contributed to by the Intermediate Outcome**							
Improve population health, safety and management							
	**Performance Targets**						
**Programme Outcome Indicators**	**Base Year**	**Base Line**	**2023/24**	**2024/25**	**2025/26**	**2026/27**	
Reduce NCDs Rates - Cancers	2020/21	1.3	1.4	1.2	1.2	1.2	
Reduce NCDs Rates - Diabetic	2020/21	2.5	2.1	2	2	2	
Reduce NCDs Rates - Hypertension rate	2020/21	3.2	2.4	2.5	2.5	2.5	
Tuberculosis incidence per 100,000 population	2020/21	234	104	77	77	77	
Under 5 illnesses attributed to Diarrheal diseases, %	2020/21	69%	35%	30%	30%	30%	
Under Five Mortality Rate (Per 1,000)	2020/21	64	33	30	30	30	
**Programme Outcome**	Improvement in the social determinants of health and safety						
**Programme Objectives contributed to by the Intermediate Outcome**							
Improve population health, safety and management							
	**Performance Targets**						
**Programme Outcome Indicators**	**Base Year**	**Base Line**	**2023/24**	**2024/25**	**2025/26**	**2026/27**	
Access to basic sanitation - Improved handwashing facility	2020/21	34%	46%	50%	50%	50%	
Access to basic sanitation - Improved sanitation coverage (toilet)	2020/21	19%	37%	45%	45%	45%	
Access to safe water supply - Rural	2017/18	73%	69%	70%	72%	74%	
Access to safe water supply - Urban	2021/22	72.1%	75%	76%	78%	80%	
Alcohol abuse Rate	2020/21	5.8	5.0	4.8	4.8	4.8	
Mortality attributed to Injuries (%)	2020/21	13%	9%	8%	8%	8%	
Prevalence of child disability	2017/18	13	9	8	7.5	7	
Prevalence of Malnutrition in the population, %	2020/21	40%	24%	20%	20%	20%	
Prevalence of overweight/ obesity, % - Children U5	2020/21	7%	4%	3.7%	3.7%	3.7%	
Prevalence of teenage Pregnancy	2020/21	25	16	14	14	14	
**Programme Outcome**	Reduced fertility and dependence ratio						
**Programme Objectives contributed to by the Intermediate Outcome**							
Improve population health, safety and management							
	**Performance Targets**						
**Programme Outcome Indicators**	**Base Year**	**Base Line**	**2023/24**	**2024/25**	**2025/26**	**2026/27**	
Adolescent fertility rate (Birth rate per 1,000 adolescent women aged 10 – 14 years, aged 15-19 years)	2020/21	132	126	125	125	125	
Total Fertility Rate	2020/21	5.4	4.6	4.5	4.5	4.5	
Unmet need for Family Planning	2020/21	28	14	10	10	10	

HUMAN CAPITAL DEVELOPMENT

**Programme Outcome**	Universal Health Coverage					
**Programme Objectives contributed to by the Intermediate Outcome**						
Improve population health, safety and management						
			**Performance Targets**			
**Programme Outcome Indicators**	**Base Year**	**Base Line**	**2023/24**	**2024/25**	**2025/26**	**2026/27**
% readiness capacity of health facilities to provide general services	2020/21	72%	68%	76%	76%	76%
Out of pocket health expenditure (financial protection for ill health)	2020/21	42	26	20	20	20
Proportion of the population accessing health Insurance	2020/21	22	17	25	25	25
**Programme Outcome**	Occupational safety and health management improved					
**Programme Objectives contributed to by the Intermediate Outcome**						
Improve population health, safety and management						
			**Performance Targets**			
**Programme Outcome Indicators**	**Base Year**	**Base Line**	**2023/24**	**2024/25**	**2025/26**	**2026/27**
Proportion of workplaces with occupational health services	2017/18	20%	40%	45%	48%	50%
**Programme Outcome**	All key forms of inequalities reduced					
**Programme Objectives contributed to by the Intermediate Outcome**						
Reduce vulnerability and gender inequality along the lifecycle						
			**Performance Targets**			
**Programme Outcome Indicators**	**Base Year**	**Base Line**	**2023/24**	**2024/25**	**2025/26**	**2026/27**
GBV prevalence	2019/20	51%	45%	44%	43.5%	43%
Gender gap index	2017/18	0.523	70%	0.8	0.82	0.85
Gender inequality index	2017/18	0.523	50.5%	0.5	0.45	0.4
Proportion of the population accessing Universal health care, (Universal Health Coverage Index), %	2020/21	69	78%	80%	80%	80%
**Programme Outcome**	Increased coverage of social protection					
**Programme Objectives contributed to by the Intermediate Outcome**						
Reduce vulnerability and gender inequality along the lifecycle						
			**Performance Targets**			
**Programme Outcome Indicators**	**Base Year**	**Base Line**	**2023/24**	**2024/25**	**2025/26**	**2026/27**
Proportion of population with access to social insurance, %	2017/18	5%	15%	20%	25%	28%
Proportion of the population with access to Direct income support, %	2017/18	0.5%	6%	8%	8.5%	9%
Proportion of the population with access to social care services, %	2017/18	5%	12.5%	15%	20%	25%

HUMAN CAPITAL DEVELOPMENT

Programme Outcome	Increased Labour force in decent employment					
**Programme Objectives contributed to by the Intermediate Outcome**						
Produce appropriate knowledgeable, skilled and ethical labour force (with strong emphasis on science and technology, TVET and Sports)						
	Performance Targets					
**Programme Outcome Indicators**	**Base Year**	**Base Line**	**2023/24**	**2024/25**	**2025/26**	**2026/27**
Employment to population ratio (EPR)[3]	2017/18	47.5	64.6	69.8	70.2	73
Unemployment rate, %	2021	11.9%	11%	10.8%	10.2%	9.98%
Unionization density, %	2019/20	6%	11%	12%	13%	14%
**Programme Outcome**	Increased employability of the labor force					
**Programme Objectives contributed to by the Intermediate Outcome**						
Produce appropriate knowledgeable, skilled and ethical labour force (with strong emphasis on science and technology, TVET and Sports)						
	Performance Targets					
**Programme Outcome Indicators**	**Base Year**	**Base Line**	**2023/24**	**2024/25**	**2025/26**	**2026/27**
Employers satisfied with the training provided by the TVET institutions, %	2020/21	44	48.4%	48.5	52.5	56.7
School to work transition rate (%)	2020/21	34.3	36.5%	37.4	38.7	39.9
TVET to work transition rate (%)	2020/21	68	69%	69.7	70.4	71.2
**Programme Outcome**	Improved Skills Mix					
**Programme Objectives contributed to by the Intermediate Outcome**						
Produce appropriate knowledgeable, skilled and ethical labour force (with strong emphasis on science and technology, TVET and Sports)						
	Performance Targets					
**Programme Outcome Indicators**	**Base Year**	**Base Line**	**2023/24**	**2024/25**	**2025/26**	**2026/27**
Ratio of STEI/ STEM graduates to Humanities	2021/22	2	3	3	4	4
Ratio of Technicians to Engineers	2021/22	5	6	6	7	7
Ratio of TVET graduates to University graduates, %	2021/22	60	70%	72	74	75

Table P2.2: Intermediate Outcomes Indicators

**Sub-Programme Name:**	**Education,Sports and skills**					
**Intermediate Outcome Indicators:**	Improved learning outcomes					
	Performance Targets					
**Indicators**	**Base Year**	**Base Line**	**2023/24**	**2024/25**	**2025/26**	**2026/27**
Survival rate to grade 5	2021/22	55.7	60%	59	60.1	61.8
Pupil Classroom ratio	2020/21	54	53	53	53	50
Pupil Desk Ratio	2020/22	7	6	5	5	4
Pupil textbook Ratio	2020/21	5	4	4	4	4
Pupil to toilet stance ratio	2020/21	71	69	68	66	64

HUMAN CAPITAL DEVELOPMENT

**Sub-Programme Name:**	**Education,Sports and skills**					
**Intermediate Outcome Indicators:**	Increased number of Schools equipped and supported to meet the Basic Requirements and Minimum standards					
	**Performance Targets**					
**Indicators**	**Base Year**	**Base Line**	**2023/24**	**2024/25**	**2025/26**	**2026/27**
No. of government schools receiving textbooks	2020/21	1266	1286	1286	1286	1286
No. of Private schools receiving textbooks	2020/21	1860	1880	1880	1880	1880
No. of Secondary Government school laboratories equipped with 100 computers	2020/21	200	320	380	400	450
No. of secondary schools constructed under UGIFT	2021/22	101	115	27	58	58
No. of traditional Government secondary schools rehabilitated and made disability friendly	2020/21	10	28	30	35	40
Percentage of Pre-primary (registered) schools meeting the BRMS	2020/21	22	30%	35	40	45
Proportion of primary schools (SACMEQ) attaining the BRMS, %	2020/21	54	58%	60	63	65
Proportion of Secondary schools (SACMEQ) attaining the BRMS, %	2020/21	47	50%	51,4	52.8	54.2
**Intermediate Outcome Indicators:**	Improved assessing and certifying the competences acquired by the trainee beneficiaries					
	**Performance Targets**					
**Indicators**	**Base Year**	**Base Line**	**2023/24**	**2024/25**	**2025/26**	**2026/27**
Number of accredited work-based training providers	2020/21	10	11	11	12	14
Number of STEM/STEI programmers accredited	2020/21	8	8	9	9	10
**Intermediate Outcome Indicators:**	Streamlined STEI/ STEM in the education system.					
	**Performance Targets**					
**Indicators**	**Base Year**	**Base Line**	**2023/24**	**2024/25**	**2025/26**	**2026/27**
Percent of Teachers/ Instructors oriented on the new curriculum	2020/21	22	32%	35	40	45
**Sub-Programme Name:**	**Population Health, Safety and Management**					
**Intermediate Outcome Indicators:**	Increased access to quality safe water supply and sanitation facilities in rural areas					
	**Performance Targets**					
**Indicators**	**Base Year**	**Base Line**	**2023/24**	**2024/25**	**2025/26**	**2026/27**
% of households with access to improved water supply facilities within 1000 metres	2021	68%	71%	72%	74%	76%
% of point water sources that are functional (active) at the time of spot check	2021	85%	88%	89%	90%	90%
% of population with access to basic sanitation (Improved toilet not shared with other households)	2017/18	36.3%	40%	45%	50%	55%

HUMAN CAPITAL DEVELOPMENT

Sub-Programme Name:	Population Health, Safety and Management					
**Intermediate Outcome Indicators:**	Increased access to safe water and sanitation facilities in urban areas					
	**Performance Targets**					
**Indicators**	**Base Year**	**Base Line**	**2023/24**	**2024/25**	**2025/26**	**2026/27**
% of people with acess to sewerage services (urban areas – NWSC)	2021	33%	34%	37%	40%	43%
**Intermediate Outcome Indicators:**	Quality of Health Care and Patient Safety					
	**Performance Targets**					
**Indicators**	**Base Year**	**Base Line**	**2023/24**	**2024/25**	**2025/26**	**2026/27**
% work places with breast feeding corners	2020/21	25%	20%	30%	30%	30%
ART Coverage	2020/21	95%	95%	95%	95%	95%
DPT3 Coverage	2020/21	98.5%	98%	99%	99%	99%
Under 5 Vitamin A second dose supplement, %	2020/21	50%	46%	55%	55%	55%
Infant Mortality rate per 1000	2020/21	30.5	32.5	25	25	25
Maternal Mortality per 100,000	2020/21	211	236	190	190	190
Under five mortality rate per 1000	2020/21	33	35	30	30	30
**Sub-Programme Name:**	**Gender and Social Protection**					
**Intermediate Outcome Indicators:**	Improved gender equality in participating and benefiting from all development opportunities at all levels					
	**Performance Targets**					
**Indicators**	**Base Year**	**Base Line**	**2023/24**	**2024/25**	**2025/26**	**2026/27**
Prevalence rate of GBV	2019/20	51%	45%	44%	43.5%	43%
Proportion of women owning businesses	2019/20	51%	45%	44%	43.5%	43%
Proportion of women participating in decision making at all levels (National, Parliament, LG, Businesses and Managerial positions in formal sector)	2019/20	35%	40%	42%	42.5%	44%
**Intermediate Outcome Indicators:**	Improved income security of vulnerable groups					
	**Performance Targets**					
**Indicators**	**Base Year**	**Base Line**	**2023/24**	**2024/25**	**2025/26**	**2026/27**
Access to livelihood support by eligible vulnerable persons	2019/20	2.8%	3.7%	4%	4.1%	4.3%
Functional social care and support services system in place	2022/23	1	0	0	0	0
**Intermediate Outcome Indicators:**	Resilience to shocks across the lifecycle by vulnerable groups improved					
	**Performance Targets**					
**Indicators**	**Base Year**	**Base Line**	**2023/24**	**2024/25**	**2025/26**	**2026/27**
Eligible vulnerable persons accessing social care and support services	2019/20	1%	3%	4%	4.5%	5%
Proportion of the population with access to social security	2019/20	4.5%	4.9%	5%	5.1%	5.2%

HUMAN CAPITAL DEVELOPMENT

Sub-Programme Name:	Labour and employment services					
Intermediate Outcome Indicators:	Decent employment improved					
	Performance Targets					
Indicators	Base Year	Base Line	2023/24	2024/25	2025/26	2026/27
Proportion of Labour frce transiting into decent employment	2019/20	34.5%	39.5%	41.5%	42%	42.1%
Unionization density	2019/20	6%	11%	12%	13%	14%

P3: Medium Term Budget Allocations by Sub-Programme and Vote

Table P3.1: Proposed Budget Allocations and Medium Term Projections by Sub-Programme

Billion Uganda Shillings	FY2022/23	2023/24	Medium Term Projections			
	Approved Budget	Proposed Budget	2024/25	2025/26	2026/27	2027/28
01 Education,Sports and skills	3,913.308	4,503.441	5,224.915	5,896.302	7,161.288	7,161.288
02 Population Health, Safety and Management	4,739.061	4,179.236	3,408.885	3,946.673	4,264.841	4,130.545
03 Gender and Social Protection	198.684	42.237	44.424	45.358	47.454	50.364
04 Labour and employment services	238.485	280.692	414.521	343.056	311.948	264.498
**Total for the Programme**	**9,089.539**	**9,005.605**	**9,092.745**	**10,231.390**	**11,785.530**	**11,606.694**

Table P3.2: Proposed Budget Allocations and Medium Term Projections by Vote

Billion Uganda Shillings	FY2022/23		2023/24	Medium Term Projections			
	Approved Budget	Spent by End Sep	Proposed Budget	2024/25	2025/26	2026/27	2027/28
011 Ministry of Local Government	0.040	0	0.040	0.041	0.049	0.066	0.066
013 Ministry of Education and Sports	661.318	36.613	669.405	686.962	591.683	628.863	628.863
014 Ministry of Health	1,583.949	54.267	1,245.293	229.335	272.929	368.746	368.746
018 Ministry of Gender, Labour and Social Development	206.881	37.588	162.082	260.834	176.595	116.117	74.226
019 Ministry of Water and Environment	1,027.783	56.585	785.392	717.460	909.782	618.841	510.629
107 Uganda Aids Commission (UAC)	17.918	1.909	17.199	19.425	22.789	29.404	29.404
108 National Planning Authority (NPA)	8.933	2.541	8.933	11.202	13.442	18.147	18.147
111 National Curriculum Development Centre (NCDC)	22.125	1.393	23.599	36.096	42.417	54.995	54.995
114 Uganda Cancer Institute (UCI)	63.878	6.369	28.901	33.902	39.707	50.962	50.962
115 Uganda Heart Institute (UHI)	23.338	3.487	25.942	69.501	116.252	132.731	103.996
116 Uganda National Medical Stores	513.090	124.498	513.695	723.215	865.810	1,163.612	1,163.612
122 Kampala Capital City Authority (KCCA)	70.911	10.701	62.858	68.975	78.007	92.401	92.401

HUMAN CAPITAL DEVELOPMENT

Billion Uganda Shillings	FY2022/23		2023/24	Medium Term Projections			
	Approved Budget	Spent by End Sep	Proposed Budget	2024/25	2025/26	2026/27	2027/28
124 Equal Opportunities Commission	0.770	**0.130**	0.770	0.965	1.158	1.564	1.564
127 Uganda Virus Research Institute (UVRI)	10.132	**0.835**	7.732	13.392	15.838	20.740	20.740
128 Uganda National Examination Board (UNEB)	126.730	**7.293**	144.828	169.924	202.611	271.842	271.842
132 Education Service Commission (ESC)	14.446	**2.103**	11.830	13.579	15.992	20.902	20.902
134 Health Service Commission (HSC)	13.962	**1.158**	11.967	13.579	16.040	20.954	20.954
149 National Population Council	12.259	**0.915**	11.439	13.847	16.303	21.147	21.147
151 Uganda Blood Transfusion Service (UBTS)	23.657	**4.635**	21.899	35.371	41.805	54.753	54.753
164 National Council for Higher Education	19.805	**1.641**	14.805	18.667	21.850	27.985	27.985
165 Uganda Business and Technical Examination Board	33.733	**5.934**	30.787	44.277	52.489	69.289	69.289
166 National Council of Sports	47.812	**7.642**	17.387	17.783	21.171	28.117	28.117
301 Makerere University	366.345	**76.640**	358.486	423.863	486.693	597.358	597.358
302 Mbarara University	57.782	**12.557**	58.513	84.155	96.785	119.334	119.334
303 Makerere University Business School	107.310	**21.712**	105.806	118.707	135.871	165.465	165.465
304 Kyambogo University	138.010	**28.912**	136.909	168.672	195.983	247.015	247.015
305 Busitema University	60.058	**10.219**	54.475	59.591	67.975	82.430	82.430
306 Muni University	25.125	**4.574**	25.931	27.963	31.907	38.828	38.828
307 Kabale University	63.111	**8.665**	55.944	64.717	73.629	88.471	88.471
308 Soroti University	24.640	**3.522**	23.989	28.067	32.037	38.806	38.806
309 Gulu University	74.751	**12.046**	63.608	69.697	79.645	96.671	96.671
310 Lira University	26.977	**4.915**	27.301	33.249	38.266	47.468	47.468
312 Uganda Management Institute	35.630	**7.502**	36.350	51.579	59.925	75.563	75.563
313 Mountains of the Moon University	37.648	**2.233**	32.235	33.539	37.938	44.867	44.867
401 Mulago National Referral Hospital	98.304	**12.052**	93.404	113.964	131.950	165.229	165.229
402 Butabika Hospital	20.839	**3.782**	21.068	33.536	39.291	50.575	50.575
403 Arua Hospital	17.444	**2.362**	10.884	13.353	15.205	18.281	18.281
404 Fort Portal Hospital	11.838	**2.495**	11.758	14.440	16.383	19.528	19.528
405 Gulu Hospital	16.618	**2.318**	14.722	20.889	24.215	30.356	30.356
406 Hoima Hospital	16.379	**2.444**	10.729	12.597	14.209	16.696	16.696
407 Jinja Hospital	20.389	**3.673**	20.774	27.794	32.031	39.615	39.615
408 Kabale Hospital	12.551	**2.017**	11.683	13.766	15.856	19.588	19.588
409 Masaka Hospital	13.551	**2.383**	10.991	13.348	15.156	18.098	18.098
410 Mbale Hospital	21.452	**2.051**	17.103	23.966	27.778	34.807	34.807
411 Soroti Hospital	11.879	**2.208**	10.729	13.506	15.390	18.536	18.536

HUMAN CAPITAL DEVELOPMENT

Billion Uganda Shillings	FY2022/23		2023/24	Medium Term Projections			
	Approved Budget	Spent by End Sep	Proposed Budget	2024/25	2025/26	2026/27	2027/28
412 Lira Hospital	17.000	3.487	17.215	23.697	27.559	34.801	34.801
413 Mbarara Regional Hospital	19.900	3.705	18.991	25.905	30.157	38.164	38.164
414 Mubende Regional Referral Hospital	10.030	2.157	9.580	11.497	12.971	15.249	15.249
415 Moroto Regional Referral Hospital	10.836	2.363	10.900	13.667	15.650	19.071	19.071
416 Naguru National Referral Hospital	11.737	2.171	11.777	13.340	14.948	17.279	17.279
417 Kiruddu National Referral Hospital	26.364	4.651	26.394	35.231	41.209	52.785	52.785
418 Kawempe National Referral Hospital	16.595	2.994	16.595	20.308	23.382	28.903	28.903
419 Entebbe Regional Referral Hospital	7.768	1.703	7.768	9.184	10.537	12.946	12.946
420 Mulago Specialized Women and Neonatal Hospital	26.268	5.681	26.768	37.031	43.271	55.344	55.344
421 Kayunga Referral Hospital	11.161	0.937	11.161	16.482	19.387	25.097	25.097
422 Yumbe Referral Hospital	10.247	0.880	10.247	11.003	12.765	16.025	16.025
515 Uganda Embassy in Japan, Tokyo	0.044	0.001	0.044	0.044	0.044	0.044	0.044
516 Uganda Embassy in Saudi Arabia, Riyadh	0.001	0	0.001	0.001	0.001	0.001	0.001
519 Uganda Embassy in Italy, Rome	0.040	0	0.040	0.040	0.040	0.040	0.040
524 Uganda Embassy in Iran, Tehran	0.025	0	0.025	0.025	0.025	0.025	0.025
525 Uganda Embassy in Russia, Moscow	0.080	0.003	0.080	0.080	0.080	0.080	0.080
531 Uganda Embassy in Turkey, Ankara	0.150	0.019	0.150	0.150	0.150	0.150	0.150
612 Local Governments 12	3,139.194	645.669	3,807.694	4,211.773	4,760.378	5,583.761	5,583.761
**Total for the Programme**	**9,089.539**	**1,275.942**	**9,005.605**	**9,092.745**	**10,231.390**	**11,785.530**	**11,606.694**

P4: PROGRAMME PRIORITIES IN LINE WITH INTERVENTIONS FOR FY2023/24

Programme Priorities FY2023/24	NDP III Programme Intervention
Implement a National Central Admission System for Universities.<br><br>Sponsor 3 staff of Lira University (2 male and 1 female) for PhD programmes in Uganda.	Accelerate the acquisition of urgently needed skills in key growth areas.
Introduce 3 new science based academic programmes at Lira University	Adopt science project-based assessment in the education curricular

HUMAN CAPITAL DEVELOPMENT

Programme Priorities FY2023/24	NDP III Programme Intervention
The Programme will:<br>i. Disseminate the National Physical Education and Sports Policy to Sports Officers and Sports Associations.<br>ii. Review guidelines for streamlining organization and management of PE and Sports in EIs.<br>iii. Conduct a Needs assessment in 16 schools to determine the best locations for the Regional Centres of Excellence<br>iv. Develop Education Institutions play grounds maintenance guidelines.	Develop a framework for talent identification in Sports, Performing and creative Arts
Roll out Open Distance Learning (ODeL) to 80% of Higher Education Institutions.<br><br>Monitor Higher Education Institutions for ODeL.<br><br>Training of 700 students and 160 teaching staff at Lira University on Open Distance Learning (ODeL) to facilitate both online and face to face learning and assessment of all students particularly in the era of Covid-19 global pandemic and other epidemics such as Ebola.<br><br>Mbarara University of Science and Technology plans to develop 5 self paced online programmes.	Develop and implement a distance learning strategy
Facilitate and participate in 10 inter university games and 5 internal sports leagues.<br><br>MUST plans to waiver tuition fees for 45 outstanding sports personalities.	Develop and implement professional sports club structures to promote formal sports participation
Makerere University plans to continue with digitalisation of university systems and programmes including students learning through MUELE e-learning platform, Academic Records management through ACMIS, Human Resource Management though eHRM; and 3,000education resources repository in the Makerere University Library.<br><br>Makerere University Business School plans to develop 5 online self paced programmes.	Develop digital learning materials and operationalize Digital Repository

HUMAN CAPITAL DEVELOPMENT

Programme Priorities FY2023/24	NDP III Programme Intervention
The Programme plans to<br>-Develop 14 demand driven industrial led occupational standards<br>-Establish the National Teacher Council<br>-Conduct Studies to inform capacity needs for standards and implementation of Education for Sustainable Development and other SDG targets within the education system.<br>-Print 1,000,000 EGR materials<br>-Print 2,212,653 copies of P1 - P4 and procure 3000 metallic cabinets<br>-Procure 3,410 Physics, 4,420 Chemistry and 100 biology textbooks for 230 schools<br>-Procure 42,857 practical science students manuals books<br>-Procure 300 cartons of braille papers, 100 cartons of embossing papers, 30 orbit readers and Assorted materials for learners with intellectual impairment and Albinism<br>-Retool 150 Secondary teachers in the subjects that were affected by the curriculum review<br>-Sensitize 100 private secondary school nonfunctional boards on their roles<br>-Sensitize 200 school leaders on establishing effective school security systems<br>-Sensitize 300 teachers about utilization of the TMIS<br>-Conduct Capacity development for 20 TVET ToTs on preparation and delivery under CBET<br>-Monitor schools and institutions to ensure provision of psychosocial services, Compliance to BRMS in public and private schools and institutions, Development of improvement plans for secondary schools, Adherence to planning, Inspection and accountability guidelines in LGs, Proper use of the TELA system in schools and follow up inspection<br>-Operationalize 7 AEP Centers<br>-Train headteachers and deputy headteachers in Leadership and management<br>-Construct, expand and equip 8 TIs<br>-Carry out expansion works at 9 existing TIs<br>-Develop and review minimum standards for University Programmes<br>-Offer exceptional help to students and staff according to the need for example recess semesters to increase on the graduation numbers as well as offering Skills Development Programme	Equip and support all lagging primary, secondary schools and higher education institutions to meet the basic requirements and minimum standards
Conduct Labour productivity assessment in major sectors of economy.<br><br>Operationalize Minimum Wages Advisory Board to discuss wages proposals and position papers and a functional Labour Advisory Board.<br><br>Launch report on the Assessment of COVID-19 Impact on Labour, Employment and Productivity.	Establish a functional labour market

HUMAN CAPITAL DEVELOPMENT

Programme Priorities FY2023/24	NDP III Programme Intervention
Develop the Uganda National Labour Productivity Enhancement Strategy and Plan, 2022-2032.<br><br>Operationalize Medical Arbitration Board to dispose of disputes of assessment of permanent incapacities between workers and employers.<br><br>Compensate 50 Government Workers for work related injuries and diseases.<br><br>Conduct 960 Labour Inspections in all sectors of the Economy on compliance to Labour Standards.<br><br>Conduct capacity building of 80 workers across all sectors on labour, Industrial Relations and productivity.<br><br>Print and disseminate an annual National Labour Inspection Report FY 2022/23<br><br>Review and register Labour unions returns.<br><br>Inspect 120 Infrastructure development projects for Social Safe guards compliance in all sub-regions and sensitize 60 Workers and contractors on social safeguards.<br><br>Inspect 100 private recruitment agencies on adherence to labour standards across the country; 80 pre-departure orientation and training centers; and 200 external recruitment agencies on compliance for safe labour migration.<br><br>Conduct capacity building on the generation and utilization of Labour Management Information System for 10 generating entities.<br><br>Clear 36,000 Migrant workers at Entebbe International Airport for safe labour migration<br><br>Conduct sensitizations on reintegration of return migrant workers and the general public on the fight against trafficking in persons.<br><br>Conduct capacity building of pre-departure training institutions on standards and compliance.<br><br>Develop and print Jobseekers' career magazine.<br><br>Disseminate the Internship strategy.<br><br>Compile and disseminate quarterly labour market bulletins	

HUMAN CAPITAL DEVELOPMENT

Programme Priorities FY2023/24	NDP III Programme Intervention
[illegible] across the country<br>Review National Employment Policy.	
Organize mentorship sessions on marketable skills for the youth.<br><br>Sensitize of internal private recruitment agencies on effective job matching.<br><br>Organise pre-licensing seminars for external recruitment agencies.<br><br>Conduct capacity building of district labour officers on internal and external employment and stakeholders on the use of the digital job matching tool.<br><br>Carryout Radio, TV media sensitizations on safe labour migration and prevention of trafficking in persons.<br><br>Review worker's Compensations Principles and its regulations.<br><br>Develop principles for amendment of Labour Unions Act and its regulations, principles to amend the Employment Act, (2006), and the Uganda National Policy on Labour Productivity.<br><br>Conduct capacity building of 120 hotel apprenticeship instructors on the green apprenticeship in the hotel industry and Green Skills Needs Assessment in the Agricultural sector.<br><br>Register and settle 500 labour complaints and disputes.<br><br>Assess 10 District Local Governments and enhance Stakeholders awareness on the implementation of the National Employment Strategy.<br><br>Review the international standard classification of occupation (ISCO).	Establish a functional labour market

HUMAN CAPITAL DEVELOPMENT

Programme Priorities FY2023/24	NDP III Programme Intervention
Carry out Stakeholder consultations, engage consultants, development, printing, dissemination, and implementation of the Public Private Partnerships for Health Strategic plan 2020-2023.<br><br>Stakeholder consultations, engage consultants, development, printing, and dissemination of the health sector Integrated Refugee Response Plan.<br><br>Hold quarterly health partners engagement meetings/ workshops.<br><br>Annual documentation of non-state actor contribution to health system investments.<br><br>Integration of refugee response activities into the government health systems while preserving the historical gains of peaceful co-existence of refugees and host communities.<br><br>Develop a multi sectoral framework, compact and accountability framework for joint planning, coordination of common deliverables and performance indicators.	Establish and operationalize mechanisms for effective collaboration and partnership for UHC at all levels
The Programme will:<br>i. Hold the 2023 National Sports Competition<br>ii. Develop the Education Institutions National Annual Sports Calendar<br>iii. Procure and distribute 5,000 assorted equipment and materials for Educational Institutions Physical Education and Sports Activities<br>iv. Facilitate rehabilitation of PES facilities for 4 educational institutions<br>v. Support 4 regional Education Institutions sports competitions<br>vi. Train 150 Physical Education teachers, instructors and tutors coaches.	Establish regional sports-focused schools/sports academies to support early talent identification and development, and the training of requisite human resources for the sports sub-sector

HUMAN CAPITAL DEVELOPMENT

Programme Priorities FY2023/24	NDP III Programme Intervention
Verify 2,656 Groups of Persons with Disabilities.<br><br>Conduct documentation and Publication on achievements of the Disability Special Grant.<br><br>Provide 20% Operation funds to 181 Local Governments.<br><br>Verify 1,600 Groups of Older Persons.<br><br>Develop and submit the second report on the implementation of Convention of the rights of persons with disabilities (CRPD) to the UN committee of experts.<br><br>Develop report on the implementation of Commitments of Uganda Government 2022 disability global summit.<br><br>Conduct 4 SEGOP National Steering Committee Meetings.<br><br>Conduct SEGOP and Age-related issues Training of Trainers.<br><br>Renovate and Operationalize 7 Vocational rehabilitation centers and sheltered workshops (Masaka, Lweza, Jinja Sheltered, Mbale Sheltered, Ocoko, Mpumudde, Kireka)<br><br>Procure Food and Fire wood for 6 Vocational Rehabilitation Centres and Homes for Elderly Persons.<br><br>Procure training Materials for 6 Rehabilitation Centres.<br><br>Provide 1,600 Groups of Older Persons with Special Enterprise Grant, for income generation.<br><br>Conduct 4 Regional annual Special Disability Grant Review with LGs Council of PWDs.<br><br>Conduct 4 Regional Political Support Supervision and Monitoring exercises of the Programme.	Expand livelihood support, public works, and labour market programs to promote green and resilient growth
Commemorate International Days on Disability and Ageing<br><br>Roll out Disability Management Information System to 81 LGs<br><br>Provide care and protection to abandoned, lost and children at Naguru Reception Centre<br><br>Reactivate Uganda as beneficiary of Commonwealth Youth program<br><br>Build capacity of 650 disadvantaged youth in non-formal vocational training for self-employment and wealth creation	Expand scope and coverage of care, support and social protection services of the most vulnerable groups and disaster-prone communities

HUMAN CAPITAL DEVELOPMENT

Programme Priorities FY2023/24	NDP III Programme Intervention
[illegible]<br><br>Conduct capacity building of 1,668 youth in entrepreneurial and life skills for livelihood<br><br>Mentor and monitor 100 beneficiaries of the Youth Venture Capital Fund<br><br>Timely manage and appropriately rehabilitate of juvenile capital offenders at KNRC<br><br>Support to youth empowerment and development through celebration of International Youth Day on 12th August, 2022 enhance.<br><br>Support National Children Authority Secretariat to carry out its legal functions in line with the National Children Act (CAP 59) as amended<br><br>Support 149 District LGs in the development of Child Rights responsive Plans and Budgets<br><br>Enhance care and protection for children especially the girl child through celebrating the International Day of the Girl Child on 11th October 2023<br><br>Increase awareness on children issues through marking the Day of the African Child on 16th June, 2023<br><br>Provide technical support supervision to six (6) children programs at the local governments (YVCF, IECD, Alternative Care, Child Helpline, OVC and Youth Skills Development) to ensure quality delivery of services<br><br>Improve access to basic rights of 1,500 children in conflict with the law through the provision of food and non-food items to the Ministry institutions<br><br>Improve access to justice by 1,500 children in conflict with the law through attendance of courts of law<br><br>Conduct stakeholders quarterly review meetings for equity and social inclusion implementers<br><br>Capacity building on Human Rights Based Approach to Programming for 30 stakeholders in three (3) Local Governments of Kabale, Rukungiri and Rubanda<br><br>Capacity Building of Focal Point Officers o [illegible]	

HUMAN CAPITAL DEVELOPMENT

Programme Priorities FY2023/24	NDP III Programme Intervention
TheProgramme will:<br>i. Approve and digitize 15 Academic programmes for different levels of Education (Secondary and Education) and Competence Based Assessment<br>(CBA)<br>ii. Train 8000 teacher Educators on management of UNITE Academic Programmes<br>iii. Develop UNITE programmes, policies, Strategic plan and Master plan<br>iv. Support supervise 80 special schools, units and inclusive schools on identification of learners with special needs, subvention grant<br>use of assistive materials and provisions of pedagogical skills<br>ix. Orient 100 teachers, caregivers, teacher educators, education managers on Accelerated Education Programme curriculum and guidelines<br>x. Print and roll out Learner identification tool and Continuous Professional Development guidelines<br>xi. Disseminate Guidelines for physical access for buildings and facilities to district councils and other relevant institutions	Implement an incentive structure for the recruitment, training, and retention of the best brains into the teaching profession across the entire education system
The Programme will:<br>-Procure computers, Software licenses, Word Processing software, Local Area Network, Computer Security Software, ICT consumables, IP Phones Upgrading PBX for 5 secondary schools to develop E-Learning infrastructure (ICT)<br>-Replace batteries in 115 PPT institutions in Western and Central Uganda<br>-Carry out maintenance in 143 post primary education institutions in Eastern, Northern and Western region and of solar systems to power computers in 60 schools<br>-Ensure that data and internet connectivity is evenly distributed, internet bandwidth increased, users trained and supported on Open, Distance and Electronic Learning (ODeL) and other ICT systems and services at all Public Universities.<br>-Equip existing computer laboratories at MUST with 200 computers.	Implement an integrated ICT enabled teaching, school level inspection and supervision
Develop Popular Version of revised Gender Policy.	Implement the Uganda Gender Policy Action Plan

HUMAN CAPITAL DEVELOPMENT

Programme Priorities FY2023/24	NDP III Programme Intervention
Sustaining maternal HIV testing and maternal ART coverage at over 95%.<br><br>Increasing retention on treatment of HIV positive pregnant and lactating women.<br><br>Prevention of new infections among young previously HIV negative women.<br>Improve adolescent health services and Fast track the approval &implementation of the Adolescent Health Policy.<br><br>Continued training of health workers on Integrated Management of Childhood Illnesses (IMCI)<br><br>Scale up implementation of the maternal and newborn health package of evidence based high impact interventions at Higher Level Health Facilities.<br><br>Continued training and re orientation of health workers in adolescent and youth friendly Health services.<br><br>Improved access to immunization coverage for children against childhood diseases.<br><br>Increase funding for RMNCAH through advocacy meetings with the RMNCAH Parliamentary Forum.	Improve maternal, adolescent and child health services at all levels of care

HUMAN CAPITAL DEVELOPMENT

Programme Priorities FY2023/24	NDP III Programme Intervention
Collaborate and link with relevant sectors and line ministries which support nutrition-sensitive interventions that target and benefit infants, young children, adolescents, pregnant and lactating mothers.<br><br>Conduct integrated support supervision for nutrition service delivery for 5 high volume refugee settings, nutrition assessment counseling &support implementation in selected regions, ART facilities and poorly performing districts.<br><br>Conduct regional Nutrition Data and indicator performance Reviews.<br><br>Increase coverage of nutrition specific services to at least 80% of infants, young children, pregnant and lactating mothers in normal/stable and difficult/emergency circumstances.<br><br>Increase access to nutrition-specific services to at least 80% of adolescents.<br><br>Improve nutrition and food safety with emphasis on children under 5, children, adolescents, pregnant & lactating women, and vulnerable groups.<br><br>Continue to strengthen multi-sectoral and multi program linkages to reduce malnutrition e.g. Nutrition Committee.	Improve nutrition and food safety with emphasis on children aged under 5, school children, adolescents, pregnant and lactating women and vulnerable groups
The staffing norms for the health facility staff to be revised to take into consideration the growing population and range of services provided.<br><br>Increased focus on maintenance of medical equipment including training and recruitment of Biomedical Engineers.<br><br>Continue to improve infrastructure including staff accommodation.<br><br>Emphasize data use at sub national level and point of collection through training and mentorship and in with collaboration with the Division of Health Information.	Improve the functionality of the health system to deliver quality and affordable preventive, promotive, curative and palliative health care services focusing on:

HUMAN CAPITAL DEVELOPMENT

Programme Priorities FY2023/24	NDP III Programme Intervention
Improving the functionality of health facilities at all levels through,<br>Recruiting more Human Resources to push the health service structures to at least 75%.<br><br>Medicines & commodity supply and management including availability of Essential Medicines & Health Supplies at all care levels.<br><br>Continued improvement of health infrastructure through equipping, equipment maintenance, health facility construction, rehabilitation, and upgrade at all levels of care.<br><br>Implementation of health support systems through improved quality assurance, performance management, M&E, financing, policy, and regulation.	Improve the functionality of the health system to deliver quality and affordable preventive, promotive, curative and palliative health care services focusing on:

HUMAN CAPITAL DEVELOPMENT

Programme Priorities FY2023/24	NDP III Programme Intervention
Develop the National Occupational Safety and Health Strategy.<br><br>Review regulations on Statutory fees for Plant examination and Workplace registration.<br><br>Inspect 1,400 workplaces for compliance with OSH standards.<br><br>Examine and certify 600 statutory equipment.<br><br>Align 50 EIA and Audit projects with Social Safety and Health Safeguard Guidelines<br><br>Conduct capacity building of 600 employers on best practices of Occupational Safety and Health management.<br><br>Build capacity of eight (8) OSH inspectors in accredited courses, 20 OSH in CPDs and 176 labour officers in enforcement of OSH laws from all local governments.<br><br>Commemorate World Day for Safety and Health on 28th April 2023<br><br>Domesticate International Occupational safety and health standards<br><br>Finalise the National CBRNE Emergency Response<br><br>Promote safety of women against chemical exposure in workplaces<br><br>Conduct chemical Risk assessment by inspecting 200 workplaces<br><br>Develop Risk assessment tool<br><br>Undertake capacity building of 60 workers, employers and Labour officers on investigation and response to chemical events.<br><br>Attend International and National meetings on Chemical Weapons Convention.<br><br>Conduct Research on chemical poisoning in agriculture.	Improving Occupational Safety and Health (OSH) management

HUMAN CAPITAL DEVELOPMENT

Programme Priorities FY2023/24	NDP III Programme Intervention
Improve multi sectoral coordination for health promotion and safe workplaces to reduce occupational health hazards and diseases.<br><br>Reduce disease burden and trauma cases in Uganda attributed to occupational health and safety through adherence to Occupational Safety and health management systems.	Improving Occupational Safety and Health (OSH) management
Continue advocacy and resource mobilization for Immunization, forecast and procure vaccines, training and supervision in EPI management for the target population to be fully immunized.<br><br>Scale up vaccination for Yellow fever, TB booster doses, MR etc.<br><br>Supplement and scale up all Immunization activities conducted for measles-Rubella, Polio, Yellow fever, Covid-19 etc.	Increase access to immunization against childhood diseases
Construct new water and harvesting sources for health facilities and communities.<br><br>Functionalize water user committees for water source sustainability at HFs and local government levels.<br><br>Continued sensitization and Social behavior change communication for water treatment and availability of safe water to reduce some of the Communicable diseases.<br><br>Social behavior change communication through CHEWS and VHTs for construction and use of improved sanitation facilities, advocacy for creation of a sanitation fund at sub-county level for household sanitation facilities.	Increase access to inclusive safe water, sanitation and hygiene (WASH) with emphasis on increasing coverage of improved toilet facilities and handwashing practices

HUMAN CAPITAL DEVELOPMENT

Programme Priorities FY2023/24	NDP III Programme Intervention
Rural Water and Sanitation:<br><br>construct 20 No. Solar systems and sanitation facilities in Refugee Hosting District Local Governments. The systems will cover a total of 139 villages from 36 parishes; Construction of 25 Solar powered piped systems in Rural Growth Centers.<br><br>Construct 25 large and Medium solar powered piped systems targeting sub counties below 50% coverage.<br><br>Construct 5 Piped Water Supply systems in rural areas: - Isingiro WSS serving 126 parishes and 902 villages in Isingiro district.<br><br>Construct Ala-Ora WSS based on River Anyau and River Ora. - serving Madi-Okollo, Terego and Yumbe districts- covering 32 parishes and 100 villages; Bitysa WSS in Buhweju covering 6 parishes and 51 villages; Nyamugasani GFS in Kasese district covering 7 parishes and 34 villages; Mpungu GFS in Kanungu district; Construct 450-point water sources across the country.<br><br>Rehabilitate 1200 chronically broken point water sources across the country.	Increase access to inclusive safe water, sanitation and hygiene (WASH) with emphasis on increasing coverage of improved toilet facilities and handwashing practices

HUMAN CAPITAL DEVELOPMENT

Programme Priorities FY2023/24	NDP III Programme Intervention
Urban Water supply and sanitation:<br><br>Complete Construction of Kamuli, Kapchorwa, Buikwe, Bundibugyo Piped WSSs.<br><br>Complete Busia WSS and Commence Namasale, Kaliro Namungarwe, Budaka-Kadama Kibuku Tirinyi, Kyegegwa MparaRuyonza, Butalejja Busolwe WSSs.<br><br>Construction of piped water supply and sanitation systems in Bibia Elegu, Odramacaku, Atiak, Lacekocot, Rhino camp, Arra Dufile, Amuru TC, Kole TC, Omoro TC, Barr, Otwal, Alangi and Zeu. Okokoro, Kati, Parabong, Keri Oraba, Zombo TC, Palabek Kal, Lamwo TC and Obongi TC.<br><br>Construction of WSS in Goma, Expansion of Kiboga, Zigoti and Busiika Piped Water Supply and Sanitation Project, WSS constructed for Kasawo, Lunya, Nkooko, Nyanseke.<br><br>50 towns rehabilitated and improved in all the selected towns; Solar packages provided for 40 towns.<br><br>Construction of 240 stances public toilets in 17 Government Schools, 80 toilet stances in 7 KCCA public toilets and 5 non KCCA public toilets.<br><br>Construction of Gulu- Karuma Water Treatment Plant.	Increase access to inclusive safe water, sanitation and hygiene (WASH) with emphasis on increasing coverage of improved toilet facilities and handwashing practices

HUMAN CAPITAL DEVELOPMENT

Programme Priorities FY2023/24	NDP III Programme Intervention
Develop and disseminate the Family Planning Implementation Plan.<br><br>Forecast and procure family planning commodities for use by the community.<br><br>Train health workers in provision and counselling for family planning.<br><br>Promote and nurture change in social and individual behavior to address myths, misconceptions, and side effects and improve acceptance and continued use of family planning to prevent unintended pregnancies.<br><br>Provide age-appropriate quality SRH information and services to all age groups as part of the Minimum Health Care Package.<br><br>Improving services for prevention, treatment and management of obstetric fistula in Uganda.<br><br>Promote and increase access to the management of infertility by integrating the management of infertility into existing SRHR services.<br><br>Integrate appropriate services for menopause/andropause into the existing sexual and reproductive health service delivery.<br><br>Roll out the National Male Engagement strategy in health in all LGs.	Increase access to Sexual Reproductive Health (SRH) and Rights with special focus to family planning services and harmonised information
Conduct stakeholder engagement meetings to fast track passing of the National Health Insurance Scheme Bill.<br><br>Fast track NHIS capitalization by government.<br><br>Provider Payment Mechanisms under NHIS developed including Capitation, user fees, diagnostic related groups (DRGs) etc. developed.<br><br>Dissemination of the NHIS Act to the various stakeholders.<br><br>Develop NHIS accreditation criteria developed.	Increase financial risk protection for health with emphasis on implementing the national health insurance scheme

HUMAN CAPITAL DEVELOPMENT

Programme Priorities FY2023/24	NDP III Programme Intervention
The Programme will:<br>i. Print, distribute and disseminate ECCE Policy and Guidelines<br>ii. Develop ECCE MDD & WASH Guidelines<br>iii. Map out Partners/Civil society/ organizations that support ECCE activities in 176 Local Governments<br>iv. Hold Dialogue on the Licensing, registration of ECCE<br>v. Orient ECCE teachers on parenting education in 4 Local Governments<br>vi. Map out and profile ECCE centres in 176 LGs	Institutionalize training of ECD caregivers at Public PTCs and enforce the regulatory and quality assurance system of ECD standards
The Programme will conduct studies to inform capacity needs for standards and implementation of Education for Sustainable Development (ESD) and other SDG targets	Integrate Education for Sustainable Development (ESD) into the school curriculum
The Programme will:<br>i. Qualify 120 Education Institutions sports administrators and technical officials<br>ii. Train 150 Physical Education teachers, instructors and tutors coaches	Introduce accredited sports and physical education as stand-alone curricular subject(s) in schools and for sports coaches, administrators, and technical officials
The Programme will sign 4 MOUs with governmental Units, intergovernmental and Private<br>Sector to improve technical capacity in Physical Education and Sports, talent identification and development	Leverage public private partnerships for funding of sports and recreation programmes
Implement the Multi- sectoral NCD Strategic Plan.<br><br>Continued Scaling up of awareness of NCDs through NCD campaign days for example Mental Health Day, World Tobacco Day, World Cancer Day.<br><br>Continued Implementation of integrated education and community sensitization on healthy eating and lifestyle.<br><br>Community sensitization, resource mobilization for vaccines, capacity building.<br><br>Continued improvement of the EMS systems to reduce trauma fatalities and equipping of emergency and causality units in hospitals.	Prevent and control Non-Communicable Diseases with specific focus on cancer, cardiovascular diseases and trauma
Remodeling of health facilities to ensure they cater for disability issues.<br><br>Provide assistive & rehabilitative equipment for PWDs.<br><br>Train staff in special needs communication.	Promote delivery of disability friendly health services including physical accessibility and appropriate equipment

HUMAN CAPITAL DEVELOPMENT

Programme Priorities FY2023/24	NDP III Programme Intervention
Improve health information, innovation and research through the e-Health Policy and National Health, Research & Innovation Strategy.<br><br>Accelerate uptake of health & health systems research, innovation and technology development at all healthcare levels.	Promote health research, innovation and technology uptake
The Programme will:<br>i. Carry out school visits to popularize STEM/STEI.<br>ii. Sponsor 200 learners in oil and gas courses for improved and increased quality of skilled personnel in the oil and gas sector.<br>iii. Conduct regional and national school science and technology fairs.<br>iv. Provide 640 more scholarships and bursaries that target STEM/STEI at Makerere University.	Promote STEM/STEI focused strategic alliances between schools, training institutions, high calibre scientists and industry
Organise and hold National and regional Women Leaders Forum.<br><br>International Women Day on 8th March, 2024.<br><br>Commemorate international Rural Womens Day 2023.	Promote Women's economic empowerment, leadership and participation in decision making through investment in entrepreneurship programs, business centres
The Programme will:<br>i. Procure and distribute at least 183 sets of mini-laboratories to 183 Primary schools in 10 LGs.<br>ii. Build capacity of 500 science teachers in schools for 10 LGs.	Provide early exposure of STEM/STEI to children (eg introduction of innovative science projects primary schools)
The Programme will:<br>i. Pay scholarships for 82 TVET students including 10 SNE students<br>ii. Conduct decentralized admissions at 5 regional centers for 40,000 students.<br>iii. Pay scholarships for 4 TVET trainers and/or managers	Provide incentives to increase enrolment in skills-scarce TVET programmes to reverse the currently inverted skills triangle
The Programme will embark on rationalization of Teachers to the student to teacher ratio not more than 50 to 1 and review of staff establishments for Tertiary Institutions	Provide the critical physical and virtual science infrastructure in all secondary schools and training institutions

HUMAN CAPITAL DEVELOPMENT

Programme Priorities FY2023/24	NDP III Programme Intervention
The Programme will:<br>i. Construct all inclusive lecture facilities, residential, non residential and office accommodation with provision for PWDs and people with Special Needs, procure inclusive instructional materials for all categories of learners, installation of ramps and lifts in high rise buildings at Lira University.<br>ii. Train 60 teachers (at least 40 % male) in Sign language, braille and pedagogy to support learners with special educational needs<br>iii. Train 30 vocational instructors in specialised skills.<br>iv. Procure 300 cartons of braille papers, 100 cartons of embossing papers, 30 orbit readers and assorted materials for learners with intellectual impairment and Albinism.	Provide the required physical infrastructure, instruction materials and human resources for Higher Education Institutions including Special Needs Education
Carryout mass LLIN campaign and distribution.<br><br>Carryout Mass Intermittent Preventive Treatment for malaria countrywide during the National Malaria days twice a year.<br><br>Increase the number of MDR-TB initiating hospitals to 20 and build capacity of HCWs.<br><br>Improve MDR support systems for example enablers for both DS TB and MDR TB, Forecast, and facilitate procurement of HIV testing kits.<br><br>Increase the number of MDR TB initiating Hospitals and build capacity of Health workers through training on contact investigation, screening, diagnosis, lab reporting, etc.<br><br>Forecast, procure and distribute TB /Leprosy medicines.<br><br>Scale up IRS in high transmission districts in West Nile, Acholi, Lango, Teso, Bukedi and Busoga regions.<br><br>Implementation of the National NTD control and elimination plan towards intensified and integrated vector control programming and NTD case management.<br><br>Design and implement/scale up innovative HIV prevention programs to improve comprehensive HIV knowledge, impart life skills, reduce risky sexual behaviors, address gender- based violence and improve sexual and reproductive health status among in and out- of- school children and youth.	Reduce the burden of communicable diseases with focus on high burden diseases (Malaria, HIV/AIDS, TB, Neglected Tropical Diseases, Hepatitis), epidemic prone diseases and malnutrition across all age groups emphasizing Primary Health Care Approach

HUMAN CAPITAL DEVELOPMENT

Programme Priorities FY2023/24	NDP III Programme Intervention
The Programme will monitor 1,000 students undergoing Workplace learning and Industrial Training.	Refocus and support Vocational Training Institutions (schools, institutes and colleges) to deliver a dual training system for TVET (i.e. 80 percent training in industry and 20 percent learning in the institution) and Universities (ie 40 percent training in industry and 60 percent training in institution).
The Programme will:<br>i. Roll-out of the EGRA and EGMA methodologies to P.1 and P.2 teachers in 4 Local Governments of Kalaki, Kaberamaido, Amudat and Tororo<br>ii. Monitor and support 100 primary schools in 10 least performing Local Governments<br>iii. Procure 1,000,000 EGR materials	Roll out Early Grade Reading (EGR) and Early Grade Maths (EGM) in all primary schools to enhance proficiency in literacy and numeracy
The Programme will:<br>a) Inspect 44 Private TVET providers for accreditation and registration<br>b) Monitor and support supervise 193 TVET (143 public and 50 private) institutions<br>c) Train 270 staff on modularized curricula.	Roll out the modularised TVET curricula for all formal TVET programmes as to attain a flexible demand driven TVET system in Uganda
Conduct and strengthen compliance to GBV Shelter Guidelines, 2020 by service providers.<br><br>Conduct 16 Days of Activism against VAW/G.<br><br>Enhance capacity of LG stakeholder in NGBVD management.<br><br>Develop popular Version of GBV Policy 2016.<br><br>Revise the National Action Plan on Elimination of GBV.	Scale up Gender Based Violence (GBV) interventions at all levels
Conduct technical backstopping and support supervision on Gender and Equity mainstreaming in 20 LGs conducted.<br><br>Print and disseminate Gender mainstreaming guidelines.	Support Gender equality and Equity Responsive Budgeting in all sectors and LGs
The Programme will train 50 TVET Trainers (35 male; 15 female) from 4 TVET institutions in 4 regions to meet requirements of international accreditation.	Support the TVET institutions that have the minimum requisite standards to acquire International accreditation Status
The Programme will:<br>i. Roll out and manage the redeveloped EMIS<br>ii. Conduct capacity building for EMIS users<br>iii. Undertake Data Validation/verification exercises	Upgrade the Education Management Information System to include functions for tracking enrolment, drop-out, retention, and uniquely identify learners, teachers, and institutions

P5: GENDER AND EQUITY ISSUES AND INTERVENTIONS FOR FY2023/24

The HCDP continues to make purposive investments to promote Gender and Equity in Service delivery.
These Investments are targeted to benefit all Ugandans previously left out including People with disability, the vulnerable, the older persons, ethnic minorities, refugees, and the populations in hard to reach and hard to stay areas.
The designs for the programme infrastructure have been reviewed to make structural considerations for categorises of persons such as pregnant mothers and those mothers and learners with disabilities, the children, infants and the Adolescents to

HUMAN CAPITAL DEVELOPMENT

[illegible]
promote equal access, participation and service utilization.
In addition, the programme policies and strategies have been developed to address gender and equity issues while in other policies, the programme has integrated issues affecting the vulnerable, refugee and those internally displaced persons.

In particular, the Programme will:
i. Train staff on identify gender and equity issues; gender mainstreaming and developing and monitoring gender and equity indicators;
ii. Engage the community on gender issues;
iii. Provide facilities and services that are gender responsive and accessible to all.
iv. Continue review of curricula to be gender responsive.
v. Maintain the affirmative action of 1.5 points to female students and people with special needs.
vi. Continue implementing interventions such as access to capital, empowerment and livelihood programmes for women, youth, persons with special need, eldery, street children among others.
v. Provide child friendly spaces in workplaces, health centres, schools and education institutions.

The programme recognize that several factors affect the population and lead to inequity such as malaria, HIV AIDS, chronic diseases, mental health among others. The programme will continue implementing the running interventions to address such factors, carry out sensitization exercises and provide psychosocial services to the target and most prone groups.

INNOVATION, TECHNOLOGY DEVELOPMENT AND TRANSFER

Foreword

Countries that invest in research and innovation as well as those that support regulated technology importation and adoption are able to transform faster. STI, together with ICT, enable the acceleration of the entire economy and this supports the digital transformation and the move towards digital (smart) health, digital (smart) agriculture, digital (smart) manufacturing, digital (smart) cities, among others.

A correlation of STI and economic growth can e observed with countries such as South Korea, Israel, Singapore, Germany and Switzerland. In the 1960s, South Korea's GDP per capita ranged between USD 100 and USD 250 but grew to over USD 31,000 by 2020. South Korea's leap in economic fortunes was propelled by purposeful STI human capital development through implementation of education, state-led and corporate research and development of a robust science and technology capacity. As a consequence, South Korea became home to highly innovative companies in the fields of Electronics (Samsung), Automotive (Hyundai), Cosmetics (AmorePacific), Digital (Kakao), e-Commerce (Coupang), among others.

Innovation, technology development and transfer increase productivity, inclusiveness and wellbeing of the population. It enables transformation of ideas into usable products, eases work processes and methods, enhances access to information and knowledge ultimately improving competitiveness in the economy. Innovation, technology development and transfer have the potential to address key development challenges facing the country including: low industrialization and value addition; infectious disease outbreaks; limited application of ICT; low research and innovation in industry and academia; climate change issues; and low quality of life.

This Programme aims to work towards bridging the gap that has existed in the country as far as industrial development is concerned, on the basis of the country being left behind in terms of use and application of STI in national development. The country has barely been part or benefited from the innovations that have developed other economies through the 3 industrial revolutions and yet the 4th Industrial Revolution is already moving ahead. The concept of the 'Loop and Leap' is thus being used to enable catching up while at the same time moving with the current trend of industrial development.

The Loop intends to strategically look back into the 3 industrial revolution technologies and pick up what works for the current level of economic development and apply these, while the Leap focuses on moving forward with the development and application of the 41R technologies, to ensure that the country is not left behind again in terms of technological and industrial development and advancement.

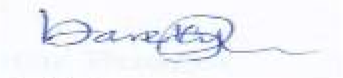

Jane Barekye

State House Comptroller

INNOVATION, TECHNOLOGY DEVELOPMENT AND TRANSFER

P1: PROGRAMME OVERVIEW

Snapshot of Medium Term Budget Allocations

Table P1.1 Overview of Programme Expenditure and Medium Term Allocations (Ush Billion)

Billion Uganda Shillings		FY2022/23		FY2023/24	MTEF Budget Projections			
		Approved Budget	Spent by End Sep	Proposed Budget	2024/25	2025/26	2026/27	2027/28
Recurrent	Wage	15.467	2.563	11.000	11.550	12.705	13.976	13.976
	Non Wage	73.756	15.797	18.000	18.357	22.005	29.665	29.665
Devt.	GoU	140.947	31.412	23.000	23.000	27.600	38.640	38.640
	ExtFin	44.250	0.000	125.568	0.000	0.000	0.000	0.000
	**GoU Total**	**230.170**	**49.773**	**52.000**	**52.907**	**62.310**	**82.281**	**82.281**
	**Total GoU+Ext Fin (MTEF)**	**274.420**	**49.773**	**177.568**	**52.907**	**62.310**	**82.281**	**82.281**
	A.I.A	0.000	0.000	0.000	0.000	0.000	0.000	0.000
	**Grand Total**	**274.420**	**49.773**	**177.568**	**52.907**	**62.310**	**82.281**	**82.281**

Programme Strategy and linkage to the National Development Plan

Uganda has not adequately prepared to use STI to industrialize. This is because;
a) there are no formal mechanisms put in place to facilitate technology transfer;
b) the country's investment on STI is currently very small;
c) the country is yet to establish any incubation and technology parks to facilitate innovation and technology development; and
d) there are no formal established mechanisms linking universities and research institutions with industry to facilitate development and commercialization of new innovations

The goal of this programme, therefore, is to increase the application of appropriate technology in the production and service delivery processes through the development of a well-coordinated STI eco-system.

This will be done through developing Value Chains in eight (8) prioritized areas that have been identified. The Value chains are : Pathogen Economy, Mobility, Aeronautics and space, Industry 4.0, Infrastructure Innovations, Productivity Acceleration, Import Substitution and STI Value Added Exports. All Programme activities have been mapped along these 8 Value Chains, which have been mapped to the NDPIII objectives and interventions.

P2: Highlights Of Programme Projected Performance

Table P2.1 Programme Outcomes Indicators

INNOVATION, TECHNOLOGY DEVELOPMENT AND TRANSFER

Programme Outcome	Enhanced development of appropriate technologies					
**Programme Objectives contributed to by the Intermediate Outcome**						
Build institutional and human resource capacity in STI						
	Performance Targets					
**Programme Outcome Indicators**	**Base Year**	**Base Line**	**2023/24**	**2024/25**	**2025/26**	**2026/27**
Number of intellectual properties registered	2022	2	5	10	10	15
Proportion of the population using appropriate technologies	2020	50	70	60	70	80
Value of International payments for the use of intellectual property - Payments - (USD Mn)	2018	20	300.0	411.7	500	600
Value of International payments for the use of intellectual property - Receipts - (USD Mn)	2017	1.8	20.0	46.6	55	70

Programme Outcome	Increased innovation in all sectors of the economy					
**Programme Objectives contributed to by the Intermediate Outcome**						
Develop requisite STI infrastructure						
	Performance Targets					
**Programme Outcome Indicators**	**Base Year**	**Base Line**	**2023/24**	**2024/25**	**2025/26**	**2026/27**
No. of incubators established and operationalized	2017	2	4	5	5	7
No. of laboratories/ R&D facilities improved or established	2017	0	3	4	5	6
No. of Science and Technology Parks established and operationalized	2020	0	1	1	1	2
No. of technology transfer centres established and operationalized	2020	0	1	1	2	2

Programme Outcome	Increased utilization of appropriate technologies					
**Programme Objectives contributed to by the Intermediate Outcome**						
Increase development, transfer and adoption of appropriate technologies and innovations						
	Performance Targets					
**Programme Outcome Indicators**	**Base Year**	**Base Line**	**2023/24**	**2024/25**	**2025/26**	**2026/27**
No. of firms graduating from incubators	2020	10	15	20	25	30
No. of firms graduating to S&T parks	2022	0	0	0	4	4
No. of new technologies adopted	2020	5	10	20	40	60
Percentage of firms using innovative technologies	2020	10	12%	15	20	25
Percentage of new technologies or research results commercialized	2020	5	7%	10	12	15

INNOVATION, TECHNOLOGY DEVELOPMENT AND TRANSFER

Programme Outcome	Improved legal and regulatory framework					
**Programme Objectives contributed to by the Intermediate Outcome**						
To improve the legal, institutional and regulatory framework						
			Performance Targets			
**Programme Outcome Indicators**	**Base Year**	**Base Line**	**2023/24**	**2024/25**	**2025/26**	**2026/27**
No. of ST&I Laws and Regulations drafted and submitted to cabinet/ parliament	2022	2	3	3	4	4
Percentage of inspected entities that are compliant to ST&I regulations	2020	0	5%	5	7	10
**Programme Outcome**	Increased R&D activities					
**Programme Objectives contributed to by the Intermediate Outcome**						
To strengthen R&D capacities and applications						
			Performance Targets			
**Programme Outcome Indicators**	**Base Year**	**Base Line**	**2023/24**	**2024/25**	**2025/26**	**2026/27**
Business enterprise sector spending on R&D (% of GDP)	2017	0.01	0.1%	0.21	0.3	0.6
Global Innovation Index (%)	2017	25.32	33%	35	37	40
Gross Expenditure on R&D (GERD) as a % of GDP	2017	0.4	0.8%	1.0	1.2	1.5
Number of applications for IP protections per annum	2017	200	300	400	500	600
Researchers in R&D (per million people)	2017	50	100	200	250	300
Technicians in R&D (per million people)	2017	20	50	80	120	160

Table P2.2: Intermediate Outcomes Indicators

Sub-Programme Name:	Research and Development					
**Intermediate Outcome Indicators:**	Increased Biosciences R&D					
			Performance Targets			
**Indicators**	**Base Year**	**Base Line**	**2023/24**	**2024/25**	**2025/26**	**2026/27**
Number of biosciences products developed	2020	5	10	15	20	25
Proportion of R&D projects in new and emerging areas	2017	0	3%	5	10	15
**Intermediate Outcome Indicators:**	Increased Research and Development					
			Performance Targets			
**Indicators**	**Base Year**	**Base Line**	**2023/24**	**2024/25**	**2025/26**	**2026/27**
Number of research projects cleared	2020	100	150	200	300	400
Percentage of research products commercialised	2020	5	10%	15	20	25
National Research Agenda in place	2020	0	1	1	1	1
National STEI surveys conducted	2020	0	1	1	2	2
STEI information management system developed	2020	0	1	1	1	1

INNOVATION, TECHNOLOGY DEVELOPMENT AND TRANSFER

**Sub-Programme Name:**	**STI Ecosystem Development**					
**Intermediate Outcome Indicators:**	Enhanced development of appropriate technologies					
	**Performance Targets**					
**Indicators**	**Base Year**	**Base Line**	**2023/24**	**2024/25**	**2025/26**	**2026/27**
Number of Technology Skills Development Initiatives undertaken	2020	10	20	30	40	50
Number of technicians skilled	2020	1000	3000	4000	5000	7000
Percentage of MDAs integrating STEI	2020	40	70%	80	90	100
Innovative strategies for implementing SDGs developed	2020	1	1	1	1	1
National apprenticeship program established	2020	0	1	1	1	1
STEI integration guidelines developed	2020	1	1	1	1	1
STEI policy developed	2020	1	1	1	1	1
**Intermediate Outcome Indicators:**	Increased innovation in biosciences					
	**Performance Targets**					
**Indicators**	**Base Year**	**Base Line**	**2023/24**	**2024/25**	**2025/26**	**2026/27**
Number of biosciences centres established	2020	0	0	1	1	1
Number of R&D laboratory centres established	2020	2	2	3	4	5
Number of R&D laboratory centres rehabilitated	2020	1	1	1	2	4
**Intermediate Outcome Indicators:**	Increased Research, Innovations and development of appropriate Technologies					
	**Performance Targets**					
**Indicators**	**Base Year**	**Base Line**	**2023/24**	**2024/25**	**2025/26**	**2026/27**
Proportion of prototypes developed into products	2020	5	30%	32	34	40
Proportion of scientists/innovators supported through the National Research and Innovation Fund	2020	5	20%	24	26	28
**Intermediate Outcome Indicators:**	Increased utilisation of appropriate technologies					
	**Performance Targets**					
**Indicators**	**Base Year**	**Base Line**	**2023/24**	**2024/25**	**2025/26**	**2026/27**
Number of technologies developed	2020	2	4	6	8	10
Number of technologies transferred (national and local)	2020	2	3	5	7	8
Number of technology needs assessments undertaken	2020	0	5	10	15	20
Technology adoption rate	2020	10	20	24	27	30
Functional STEI think tank established	2022	2	8	8	10	15
National Technology transfer strategy in place	2022	0	1	1	1	1
**Intermediate Outcome Indicators:**	Increased utilization of appropriate technologies					
	**Performance Targets**					
**Indicators**	**Base Year**	**Base Line**	**2023/24**	**2024/25**	**2025/26**	**2026/27**
Number of Intellectual Property Rights acquired	2017	2	10	15	20	25

INNOVATION, TECHNOLOGY DEVELOPMENT AND TRANSFER

Sub-Programme Name:	STI Ecosystem Development					
Intermediate Outcome Indicators:	Increased utilization of appropriate technologies					
	Performance Targets					
Indicators	Base Year	Base Line	2023/24	2024/25	2025/26	2026/27
% uptake of new and	2020	5	8%	10	12	14
Rate of adoption of locally developed technologies	2020	4	6	8	10	12
Royalties received (in USD) for use of IPRs	2017	0.1	0.5	0.7	1	2

P3: Medium Term Budget Allocations by Sub-Programme and Vote

Table P3.1: Proposed Budget Allocations and Medium Term Projections by Sub-Programme

Billion Uganda Shillings	FY2022/23	2023/24	Medium Term Projections			
	Approved Budget	Proposed Budget	2024/25	2025/26	2026/27	2027/28
03 STI Ecosystem Development	26.732	19.840	14.556	17.725	22.462	22.462
**Total for the Programme**	**274.420**	**177.568**	**52.907**	**62.310**	**82.281**	**82.281**

Table P3.2: Proposed Budget Allocations and Medium Term Projections by Vote

Billion Uganda Shillings	FY2022/23		2023/24	Medium Term Projections			
	Approved Budget	Spent by End Sep	Proposed Budget	2024/25	2025/26	2026/27	2027/28
006 Ministry of Foreign Affairs		0	0.581	0.593	0.711	0.960	0.960
110 Uganda Industrial Research Institute (UIRI)	25.502	3.400	7.360	7.683	8.604	9.924	9.924
119 Uganda Registration Services Bureau (URSB)	1.112	0.249	1.110	1.162	1.291	1.460	1.460
167 Science, Technology and Innovation		0	168.398	43.351	51.585	69.818	69.818
525 Uganda Embassy in Russia, Moscow	0.119	0.000	0.119	0.119	0.119	0.119	0.119
**Total for the Programme**	**274.420**	**49.773**	**177.568**	**52.907**	**62.310**	**82.281**	**82.281**

P4: PROGRAMME PRIORITIES IN LINE WITH INTERVENTIONS FOR FY2023/24

Programme Priorities FY2023/24	NDP III Programme Intervention
Operationalise the space programme<br><br>Training personnel in the area<br><br>Bring together all stakeholders in the Industry 4.0 space and support them to develop the required capacity to contribute to national development	Create capacity on application of drones, satellite imagery through GIS, real- time disaster modelling, and widespread connectedness, improve emergency response and production;

INNOVATION, TECHNOLOGY DEVELOPMENT AND TRANSFER

Programme Priorities FY2023/24	NDP III Programme Intervention
Completing and operationalising the skilling centres in Namanve and Rwebitete<br><br>Supporting training programmes at graduate level	Design and conduct practical skills development programmes
Operationalising the space programme; making the Mpoma satellite earth station operation, developing the aerospace strategy and policy,<br><br>Developing the Industry 4.0 Strategy and Policy<br><br>Establishing the a materials and nano-technology laboratory<br><br>Training personnel in the emerging areas of STI	Design and implement special programmes for Nano technology, space exploration, nuclear technology, bio sciences, ICT and engineering;
Strengthening the national ST&I Advisory role<br><br>Setting up think tanks for the different prioritised value chains and making them operational	Develop a framework for promotion of multi-sectoral and multilateral collabourations
Develop and implement the strategy	Develop and implement a National Science and Technology Innovation Strategy;
Develop the database for the STI national information management system<br><br>Conduct a survey to collect all STI related data from the stakeholders<br><br>Sensitise and popularise the system and train stakeholders on its use	Develop and maintain a national STI Information Management System (including a database of new and on-going Scientific Research, technologies innovations and indigenous knowledge from public and private sectors);
Identify priority areas were technology is needed<br><br>Develop the necessary frameworks for technology development and acquisition	Develop policies, laws and regulations for technology development, transfer and market development and attraction of private funding and FDI in STI.
Identification of gaps and local players in the STI ecosystem that can benefits from technology transfer<br><br>Identification of partners that have the requisite technology to be transferred<br><br>Establishing appropriate frameworks to facilitate effective technology transfer	Develop strategic local and international partnerships and cooperation on technology transfer and adoption;
Identify the necessary policies that support STI development and review, develop or amend these	Develop, review and amend policies to promote the development and uptake of technologies
Training of personnel in the identified areas	Establish a material science, nano & bio science technology centres, Space Science and Aeronautics Technology Institute

INNOVATION, TECHNOLOGY DEVELOPMENT AND TRANSFER

Programme Priorities FY2023/24	NDP III Programme Intervention
Expand the activities of the national science week and involve more stakeholders to foster greater participation and interaction among the stakeholders<br><br>Hold forums that bring together the different stakeholders	Establish platforms for the interaction between the academia, research institutions, industry and state and non-state actors.
Identify potential partners and areas of interest<br><br>Develop frameworks for cooperation	Establish research collaborations at local, regional and international level;
Mobilisation of funding for the identified areas	Increase investment in R & D in key priority sectors like; agriculture, Oil & Gas, Minerals, Energy, Health, Transport;
Facilitate the establishment of processing industries in the different prioritised value chains<br><br>Facilitate IP acquisition<br><br>Establish and operationalise machine centres where machine parts can be produced and reverse engineering done as well as train personnel for requisite skills	Strengthen the function of technology acquisition, promotion as well as transfer and adoption
Establish a working relation with Uganda Service Registrations Bureau<br><br>Train and sensitise stakeholders on IPs<br><br>Support the innovators to undertake the process of IP registration	Strengthen the Intellectual Property (IP) value chain management;
Support the acquisition of necessary research equipment and research linkages and the establishment of centres of excellence in research institutions	Support academia and research institutions to acquire R&D infrastructure;
Acquisition of land for the S&T, development of plans for the park and sourcing of funding	Support the establishment and operations of Science and Technology Parks to facilitate commercialization;
Forging collaboration with potential partners (local and international) to aid the establishment of the centres<br><br>Identification of places for the centres and mapping the necessary priority areas to be handled<br><br>Development of online software and establishment of online platforms to aid the running of the centres	Support the establishment and operations of Technology & Business incubators and Technology Transfer centres

P5: GENDER AND EQUITY ISSUES AND INTERVENTIONS FOR FY2023/24

Gender and Equity: The objective is to promote STI among the youth so that they can embrace the new technologies, This is due to the limited appreciation of STI among the young generation. Science outreach programmes in schools are going to be conducted and also youth will be involved in the science week activities.

HIV/AIDS: Contribute to the fight against the spread of HIV/AIDS among the population. There is continued spread of [illegible]

INNOVATION, TECHNOLOGY DEVELOPMENT AND TRANSFER

HIV/AIDS in the population especially among the youths. There is going to be sensitisation, on behaviour that keep the youths safe from HIV/AIDS, during the STI school outreaches

Environment: Promote R&D in environmental preservation ventures. The major concern is the continued degradation of environment. Using the satellite, data will be provided to track the preservation or degradation of the environment and thus enable policy decisions to be made

COVID: Conduct R&D in COVID-19 vaccine development. There is still a need to develop a COVID vaccine and research in its development is ongoing

PUBLIC SECTOR TRANSFORMATION

Foreword

The Public Sector Transformation Programme (PSTP) led by Ministry of Public Service is one of the 20 Priority Programmes of the 3rd (NDP III) FY 2020/21-2024/25.
The PSTP focuses on transforming the way the Public Service conducts its business and the Public Service culture while harnessing talent management and fostering innovation to address the challenge of Public Sector policy lags.
The key Objectives of the Programme are;
a)Strengthening accountability for results across Government.
b)Streamlining Government structures and institutions for efficient and effective service delivery.
c)Strengthening Strategic Human Resource Management function of Government for improved Service Delivery.
d)Deepening decentralization and citizen participation in local development.
e)Increasing transparency and eliminate corruption in the delivery of services.

The Programme registered several key milestones in the FY2021/22. These include:
a)Rationalization of Agencies: Sixty-nine (69) Government Agencies have been rationalized and guidelines to support the implementation process developed.
b)Automation of Human Resource Management Functions: . These include; establishment management control; human resource planning; recruitment management; on boarding and off boarding; payroll and deductions management; performance management; leave and absence management; disciplinary procedure; grievance handling; training and development; talent management and succession planning, Benefits Management; retirement and pension management; time and attendance and employee life cycle.
c)Implementation of the Pay Policy: National Service Commissions; Vice Chancellors, Professors, and Associate Professors of public universities; Justices, Judges and Judicial Officers; Health Workers and Other Scientists have been considered for pay enhancement and the rest of the civil servants will be enhanced in phased manner.
d)Government concluded 90% investigation of breaches into the Leadership Code out of targeted 100% of which 80% were related to non-declaration while 10% were in relation to inconsistent declarations.
e)Upgrade of E- recruitment systems: In a bid to review and develop management and operational structures, systems and standards, the Public Service Commission upgraded the E Recruitment system.
f)Development of a common public data/information sharing platform was completed in FY 2021/22 and to date 83 MDAs have been put on it. About 7.0 million transactions have been registered on the shared platform.
g)Under the Local Government Finance commission, the Grants allocation formulae and models were reviewed in line with the program approach to make them responsive to line Ministries policies and to in-build crosscutting issues.
During the FY 2023/24 the programme will focus on;
a)Continued rationalization of Government institutions by merging some Ministries,Departments and Agencies to reduce and save on costs of public administration and associated expenditures.
b)Phased salary enhancement for scientists, health workers and all other categories in the public service
c)Funding intervention that leverage on ICT to increase scope of work completed (E- inspection).
d)Automation of Performance Management processes.
e)Reform of the Public Service Pension scheme.
f)Enforcement of service and service delivery standards.
g)Promotion of local economic development through full scale implementation of the Parish Development Model.
Unfunded priorities
a)Inadequate budget for implementation of RAPEX reform: Shs.4.61Bn .
b)Shs.1,078 bn is required to cover commitment towards the UPDF and the phased salary enhancement plan for FY 2023/2024.
c)Establishment and Operationalization of the Adhoc National Emoluments Review Board (ANERB) requires Shs.5.3bn as directed by Cabinet and Parliament.
d)Operationalising Mandatory training at the Civil Service College:Shs.2bn
e)Professionalisation of all cadres in Uganda Public service: Shs 0.3m

PUBLIC SECTOR TRANSFORMATION

[illegible]
e)Professionalisation of all cadres in Uganda Public service: Shs.0.3m
f)Implement prereform activities for the Establishment of the Public Service Pension Fund requires Shs. 5.14bn.
g)Procurement of Vehicles for former leaders at Shs.1.6bn:
h)Disseminate the Public service Standing Order and Public Service Act at Shs.0.700m.
i)National IDs renewal: Mass Enrollment of the 17.2M citizens and renewal of 15M National identification Cards due to expire will require acquisition of new system, mass enrollment and renewal by NIRA.
j)Roll out of E-inspection: Overall the E-Inspection requires 7.8Bn over the medium term.
k)Wage deficit for UBC: Shs.6.5bn is required in wage for the Uganda Broadcasting Services a subvention under Ministry of ICT and national guidance.
l)Roll out of E-inspection: Overall the E-Inspection requires 7.8Bn over the medium term. Shs.2Bn for FY is required for training and bringing users on board.
m)Restructuring of Inspectorate of Government. Shs.3bn for recruiting and wage for the additional staff.
[illegible]

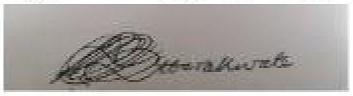

Catherine Bitarakwate Musingwiire (Mrs.)

Permanent Secretary

PUBLIC SECTOR TRANSFORMATION

Abbrevations and Acronyms

ACRONYM	ACRONYM NAME
AAPAM	Association of Africa Public Administration and Management
AIDS	Aquired Immunodeficiency Syndrome
BFP	Budget Framework Paper
BSC	Balanced Scorecard
CNA	Capacity Needs Assessment
COVID	Corona Virus Infectious Disease
COVID-19	Coronavirus Disease of 2019
CSCU	Civil Service College Uganda
CSCU	Civil Service College Uganda
CSOs	Civil Society Organisation
DP	Development Partners
EDRMS	Electronic Document Record Management System
EOC	Equal Opportunities Commission
ESAMI	Eastern & Southern Africa Management Institute FY Financial Year
F&A	Finance and Administration
FY	Financial Year
GAPR	Government Annual Performnace
GBV	Gender Based Violence
GOU	Government of Uganda
HCM	Human capital Management
HIV	Human Immunodeficiency Virus
HoD	Head of Department HR Human Resource
HR	Human Resource
HRD	Human Resource Department
HRDP	Human Resource Development Plans
HRM	Human Resource Management
HRMS	Human Resource Management System
HRP	Human Resource Plannning
HRP&P	Human Resource Policy and Procedure
HRPD	Human Resource Planning and Development
HRPDF	Human Resource Planning and Development Framework
I.A	Instititutionall Assessment
ICT	Information Communication Technology

PUBLIC SECTOR TRANSFORMATION

ACRONYM	ACRONYM NAME
LGs	Local Governmengt
M&E	Monitoring and Evaluation
MDA	Ministries Departments and Agenices
MoFPED	Ministry of Finance, Planning and Economic Development.
MoPS	Ministry of Public Service
MTEF	Medium Term Expenditure Framework
NDP	National Development Plan
NPA	National Planning Authority
NRCA	National Records Centre For Archives
NRH	National Referral Hospital
NSDS	National Service Delivery Survey
NTR	Non Tax Revenue
P&P	Policy and Planning
PDM	Parish Development Model
PIAPAS	Pearl of Aftrica Institutional Performance Assessment Scorecard
PM	Performance Management
PS	Permanent Secretary
PSI	Public Service Inspection
PSM	Public Sector Management
R&S	Research and Standards
RAPEX	Rationalistion of Agencies and Public Expenditure
RIM	Records and Information Management
RRH	Regional Referral Hospital
SDS	Service Delivery Standards
SHRM	Strategic Human Resource Management
SMT	Senior Management Team
SUCs	Service Uganda Centers
TMT	Top Management Team
TNA	Technical Needs Assessment
TSMT	Technical Senior Management Team
USAID	United States Agencies for International Deveolpment

PUBLIC SECTOR TRANSFORMATION

P1: PROGRAMME OVERVIEW

Snapshot of Medium Term Budget Allocations

Table P1.1 Overview of Programme Expenditure and Medium Term Allocations (Ush Billion)

Billion Uganda Shillings		FY2022/23		FY2023/24	MTEF Budget Projections			
		Approved Budget	Spent by End Sep	Proposed Budget	2024/25	2025/26	2026/27	2027/28
Recurrent	Wage	117.312	24.291	117.312	123.178	135.495	149.045	149.045
	Non Wage	93.586	10.372	74.155	75.639	90.766	122.534	122.534
Devt.	GoU	11.794	0.000	16.700	16.700	20.040	28.056	28.056
	ExtFin	0.000	0.000	0.000	0.000	0.000	0.000	0.000
	**GoU Total**	**222.692**	**34.664**	**208.167**	**215.516**	**246.302**	**299.635**	**299.635**
	**Total GoU+Ext Fin (MTEF)**	**222.692**	**34.664**	**208.167**	**215.516**	**246.302**	**299.635**	**299.635**
	A.I.A	0.000	0.000	0.000	0.000	0.000	0.000	0.000
	**Grand Total**	**222.692**	**34.664**	**208.167**	**215.516**	**246.302**	**299.635**	**299.635**

Programme Strategy and linkage to the National Development Plan

The PSTP contributes to the NDPIII objective five which aims at strengthening the role of the state in guiding and facilitating development . The goal of the Programme is to improve public sector response to the needs of the citizens and the private sector. The goal will be pursued under the NDPIII theme of Sustainable Industrialization for inclusive growth, employment and sustainable wealth creation. The key result areas of the programme are to increase Government effectiveness index from -0.52 to 0.01, reduce corruption as measured by the corruption perception index from 27% to 30%, increase the attractiveness of Uganda as an investment destination as measured by the global competitiveness index from 48.9 to 55.
The programme will achieve these results through Continued rationalization of Government institutions by merging some Ministries and Agencies to reduce and save on costs of public administration and associated expenditures Phased salary enhancement for scientists, health workers and all other categories in the public service, enhanced capacity building of the public service, full scale implementation of parish development model and support Government Institutions to develop, document and disseminate Service Delivery Standards.

P2: Highlights Of Programme Projected Performance

Table P2.1 Programme Outcomes Indicators

**Programme Outcome**	Increased local participation in the economy					
**Programme Objectives contributed to by the Intermediate Outcome**						
Deepen decentralization and citizen participation in local development						
			Performance Targets			
**Programme Outcome Indicators**	**Base Year**	**Base Line**	**2023/24**	**2024/25**	**2025/26**	**2026/27**
Proportion of Local Government Development Plans aligned to the NDP, %	2020/21	45%	100%	100%	100%	100%
Proportion of MDA Plans aligned to the NDP, %	2020/21	59%	85%	100%	100%	100%

PUBLIC SECTOR TRANSFORMATION

**Programme Outcome**	Reduced corruption incidences					
**Programme Objectives contributed to by the Intermediate Outcome**						
Increase accountability and transparency in the delivery of services						
	**Performance Targets**					
**Programme Outcome Indicators**	**Base Year**	**Base Line**	**2023/24**	**2024/25**	**2025/26**	**2026/27**
Corruption perception index	2020/21	27%	30%	32%	34%	36%
**Programme Outcome**	Improved government effectiveness					
**Programme Objectives contributed to by the Intermediate Outcome**						
Streamline Government architecture for efficient and effective service delivery						
	**Performance Targets**					
**Programme Outcome Indicators**	**Base Year**	**Base Line**	**2023/24**	**2024/25**	**2025/26**	**2026/27**
Government effectiveness index	2020/21	-0.58%	-0.2%	0.05%	0.08%	0.1%
**Programme Outcome**	Improved accountability for results across government					
**Programme Objectives contributed to by the Intermediate Outcome**						
Strengthen accountability for results across government						
	**Performance Targets**					
**Programme Outcome Indicators**	**Base Year**	**Base Line**	**2023/24**	**2024/25**	**2025/26**	**2026/27**
Proportion of the population satisfied with their last experience of public services	2018/19	40%	65%	70%	75%	80%
**Programme Outcome**	Improved public service productivity					
**Programme Objectives contributed to by the Intermediate Outcome**						
Strengthen human resource management function of Government for improved service delivery						
	**Performance Targets**					
**Programme Outcome Indicators**	**Base Year**	**Base Line**	**2023/24**	**2024/25**	**2025/26**	**2026/27**
Global competitiveness index	2020/21	48.94%	49.1%	50.2%	51.2%	53.2%

Table P2.2: Intermediate Outcomes Indicators

**Sub-Programme Name:**	**Strengthening Accountability**					
**Intermediate Outcome Indicators:**	Increased awareness about public services					
	**Performance Targets**					
**Indicators**	**Base Year**	**Base Line**	**2023/24**	**2024/25**	**2025/26**	**2026/27**
Level of client satisfaction with the client feedback mechanism	2020/21	57%	72%	77%	85%	90%
Percentage of population knowledgeable about public services	2020/21	50%	70%	80%	90%	100%

PUBLIC SECTOR TRANSFORMATION

**Sub-Programme Name:**	**Strengthening Accountability**					
**Intermediate Outcome Indicators:**	Reduced incidences of infollution					
	**Performance Targets**					
**Indicators**	**Base Year**	**Base Line**	**2023/24**	**2024/25**	**2025/26**	**2026/27**
% of information published that comply with the media quality management standards.	2020/21	60%	90%	100%	100%	100%
**Sub-Programme Name:**	**Government Structures and Systems**					
**Intermediate Outcome Indicators:**	Improved Efficiency of Service delivery structures and systems of government					
	**Performance Targets**					
**Indicators**	**Base Year**	**Base Line**	**2023/24**	**2024/25**	**2025/26**	**2026/27**
% of MDAs & LGs with structures aligned to their mandate and the National Development Plan	2020/21	70%	100%	100%	100%	100%
% of structures void of overlaps and duplications	2020/21	50%	100%	100%	100%	100%
Level of satisfaction of clients with the re-engineered systems' turnaround time	2020/21	50%	65%	70%	75%	85%
**Sub-Programme Name:**	**Human Resource Management**					
**Intermediate Outcome Indicators:**	Improved affordability and sustainability of the pension scheme					
	**Performance Targets**					
**Indicators**	**Base Year**	**Base Line**	**2023/24**	**2024/25**	**2025/26**	**2026/27**
Percentage of retirees accessing retirement benefits on the due date	2020/21	70%	95%	95%	100%	100%
Percentage reduction in accumulated pension and gratuity arrears	2020/21	65%	77%	85%	90%	100%
**Intermediate Outcome Indicators:**	Improved alignment of employees' competences and qualifications with job roles					
	**Performance Targets**					
**Indicators**	**Base Year**	**Base Line**	**2023/24**	**2024/25**	**2025/26**	**2026/27**
Percentage age of Public officers whose Qualification and competences are aligned to their jobs	2020/21	80%	100%	100%	100%	100%
**Intermediate Outcome Indicators:**	Improved compliance to recruitment guidelines by service commissions					
	**Performance Targets**					
**Indicators**	**Base Year**	**Base Line**	**2023/24**	**2024/25**	**2025/26**	**2026/27**
Level of compliance to recruitment guidelines by service commissions	2020/21	90%	100%	100%	100%	100%
**Intermediate Outcome Indicators:**	Improved effectiveness of performance and work place dispute management systems					
	**Performance Targets**					
**Indicators**	**Base Year**	**Base Line**	**2023/24**	**2024/25**	**2025/26**	**2026/27**
Level of client satisfaction with the client feedback mechanism	2020/21	65%	72%	77%	80%	100%
Percentage of employee grievances resulting into industrial action	2020/21	0%	0%	0%	0%	0%
Percentage of Organizations achieving	2020/21	60%	75%	85%	100%	100%

PUBLIC SECTOR TRANSFORMATION

Sub-Programme Name:	Human Resource Management					
**Intermediate Outcome Indicators:**	Improved effectiveness of performance and work place dispute management systems					
	Performance Targets					
**Indicators**	**Base Year**	**Base Line**	**2023/24**	**2024/25**	**2025/26**	**2026/27**
Percentage reduction in absenteeism rate in the Public Service	2020/21	10%	5%	5%	5%	5%
**Intermediate Outcome Indicators:**	Improved efficiency and effectiveness in Payroll management in the Public Service					
	Performance Targets					
**Indicators**	**Base Year**	**Base Line**	**2023/24**	**2024/25**	**2025/26**	**2026/27**
% of employees earning salary according to their salary scales	2020/21	100%	100%	100%	100%	100%
% of MDAs & LGs paying salary and pension by 28th	2020/21	80%	100%	100%	100%	100%
% of Public Officers receiving salary according to the approved pay plan	2020/21	70%	75%	100%	100%	100%
% of retired staff accessing the pension payroll within 30 days after	2020/21	70%	95%	100%	100%	100%
% of staff accessing payroll within 30 days after assumption of duty	2020/21	100%	100%	100%	100%	100%
% reduction in MDAs and LGs requesting for wage, gratuity and	2020/21	5%	5%	0%	0%	0%
**Intermediate Outcome Indicators:**	Improved efficiency and effectiveness of the decentralised recruitment function					
	Performance Targets					
**Indicators**	**Base Year**	**Base Line**	**2023/24**	**2024/25**	**2025/26**	**2026/27**
% of LGs with fully constituted service commissions	2020/21	60%	65%	70%	75%	85%
**Intermediate Outcome Indicators:**	Improved Quality of service delivered and compliance to service delivery standards					
	Performance Targets					
**Indicators**	**Base Year**	**Base Line**	**2023/24**	**2024/25**	**2025/26**	**2026/27**
Level of compliance with SDS in MDAs and LGs	2020/21	60%	65%	70%	75%	80%
Level of satisfaction by the service beneficiaries	2020/21	60%	65%	70%	80%	100%
**Intermediate Outcome Indicators:**	Improved Quality of the Civil Service					
	Performance Targets					
**Indicators**	**Base Year**	**Base Line**	**2023/24**	**2024/25**	**2025/26**	**2026/27**
% of advertised positions filled with skilled & competent staff	2020/21	56%	74%	80%	85%	90%
% of employees leaving the service on grounds other than due to retirement or dismissal	2020/21	0.06%	0.04%	0.04%	0.04%	0.04%
Percentage of professional Public Servants	2020/21	16%	64%	80%	85%	90%
Salary compression ratio of the Public Service	2020/21	1:4	1:24	1:18	1:16	1:14
**Intermediate Outcome Indicators:**	Improved Timeliness in implementing approved structures					
	Performance Targets					
**Indicators**	**Base Year**	**Base Line**	**2023/24**	**2024/25**	**2025/26**	**2026/27**
Timeliness in filling declared vacant positions (Months)	2020/21	4 months	3 months	3 months	3 months	3 months

PUBLIC SECTOR TRANSFORMATION

**Sub-Programme Name:**	**Human Resource Management**					
**Intermediate Outcome Indicators:**	Increased adoption of electronic document management systems					
	**Performance Targets**					
**Indicators**	**Base Year**	**Base Line**	**2023/24**	**2024/25**	**2025/26**	**2026/27**
% of Archives reference materials accessible on line	2020/21	25%	20%	30%	50%	70%
Percentage of records lost due to poor storage conditions	2020/21	24%	6%	0%	0%	0%
Percentage uptake of the automated RIM (EDRMS) system	2020/21	5%	40%	51%	70%	100%
Average process turnaround time for retrieval of records (Minutes)	2020/21	49 min	16 min	5 min	5 min	5 min
**Intermediate Outcome Indicators:**	Increased compliance to RIM processes and standards by MDAs and LGs					
	**Performance Targets**					
**Indicators**	**Base Year**	**Base Line**	**2023/24**	**2024/25**	**2025/26**	**2026/27**
Level of compliance to RIM processes	2020/21	56%	65%	70%	80%	100%
**Sub-Programme Name:**	**Decentralization and Local Economic Development**					
**Intermediate Outcome Indicators:**	A conducive environment to facilitate Private Sector participation in investment in the local economy provided					
	**Performance Targets**					
**Indicators**	**Base Year**	**Base Line**	**2023/24**	**2024/25**	**2025/26**	**2026/27**
% of District Private forums that are functional	2020/21	98%	100%	100%	100%	100%
**Intermediate Outcome Indicators:**	Critical positions at in Local Governments filled					
	**Performance Targets**					
**Indicators**	**Base Year**	**Base Line**	**2023/24**	**2024/25**	**2025/26**	**2026/27**
% of approved critical positions in	2020/21	78%	100%	100%	100%	100%
**Intermediate Outcome Indicators:**	Districts with functional LED resource teams/ for a					
	**Performance Targets**					
**Indicators**	**Base Year**	**Base Line**	**2023/24**	**2024/25**	**2025/26**	**2026/27**
Proportion of districts with functional	2020/21	90%	100%	100%	100%	100%
**Intermediate Outcome Indicators:**	Effective and efficient allocation and utilization of public resources					
	**Performance Targets**					
**Indicators**	**Base Year**	**Base Line**	**2023/24**	**2024/25**	**2025/26**	**2026/27**
Budget alignment to NDP (%)	2020/21	100%	100%	100%	100%	100%
Percentage of budget released against originally approved budget.	2020/21	100%	100%	100%	100%	100%
Percentage of funds absorbed against funds released.	2020/21	100%	100%	100%	100%	100%
Proportion of LG plans aligned to the NDP	2020/21	100%	100%	100%	100%	100%
Proportion of MDA plans aligned to the NDP	2020/21	100%	100%	100%	100%	100%

PUBLIC SECTOR TRANSFORMATION

**Sub-Programme Name:**	**Decentralization and Local Economic Development**					
**Intermediate Outcome Indicators:**	Improved commitment of government in financing the delivery of decentralized services					
	**Performance Targets**					
**Indicators**	**Base Year**	**Base Line**	**2023/24**	**2024/25**	**2025/26**	**2026/27**
Percentage share of the National budget between Central and LGs	2020/21	22%	20%	25%	30%	35%
**Intermediate Outcome Indicators:**	Improved fiscal sustainability of local governments					
	**Performance Targets**					
**Indicators**	**Base Year**	**Base Line**	**2023/24**	**2024/25**	**2025/26**	**2026/27**
Increase in local revenue mobilization, %	2020/21	30%	35%	37%	40%	45%
**Intermediate Outcome Indicators:**	Increased Domestic Tax					
	**Performance Targets**					
**Indicators**	**Base Year**	**Base Line**	**2023/24**	**2024/25**	**2025/26**	**2026/27**
Domestic Tax Revenue collection to target	2020/21	100%	100%	100%	100%	100%
Average filing ratio (PAYE & VAT)	2020/21	85%	90%	90%	90%	90%
**Sub-Programme Name:**	**Business Process Re-engineering and Information Management**					
**Intermediate Outcome Indicators:**	Enhanced Identification & Registration services					
	**Performance Targets**					
**Indicators**	**Base Year**	**Base Line**	**2023/24**	**2024/25**	**2025/26**	**2026/27**
% increase in number of government MDA systems interfacing with the NIRA	2020/21	50%	67%	70%	80%	100%
Turnaround time for issuance of Birth, Death and Adoption Orders certificates (in days)	2020/21	1 Day	1 Day	1 Day	1 Day	1 day
Turnaround time for production & Issuance of NID Cards (in days)	2020/21	14 Days	14 Days	14 Days	14 days	14 Days
**Intermediate Outcome Indicators:**	Improved capacity of the IG to respond to citizen's complaints concerning Maladministration and administrative injustice					
	**Performance Targets**					
**Indicators**	**Base Year**	**Base Line**	**2023/24**	**2024/25**	**2025/26**	**2026/27**
% of MDLGs with functional systems for resolving ombudsman Complaints	2020/21	65%	80%	90%	100%	100%
**Intermediate Outcome Indicators:**	Improved efficiency and effectiveness in the implementation of government programmes					
	**Performance Targets**					
**Indicators**	**Base Year**	**Base Line**	**2023/24**	**2024/25**	**2025/26**	**2026/27**
Percentage of high profile/ syndicated corruption cases investigated and completed within 9 months from the time of registration	2020/21	70%	75%	80%	85%	90%
Percentage of IG recommendations implemented.	2020/21	52%	65%	70%	75%	80%

PUBLIC SECTOR TRANSFORMATION

Sub-Programme Name:	Business Process Re-engineering and Information Management					
Intermediate Outcome Indicators:	Improved recovery of illicitly acquired wealth					
	Performance Targets					
Indicators	Base Year	Base Line	2023/24	2024/25	2025/26	2026/27
Value of assets and funds recovered by the IG (Bn)	2020/21	2.7bn	4.0bn	5.0bn	5.0bn	5.0bn
Value of assets and funds recovered by the IG.	2020/21	40bn	50bn	60bn	70bn	80bn
Value of money saved as a result of IG interventions (Bn)	2020/21	30bn	40bn	50bn	50bn	50bn
Intermediate Outcome Indicators:	Increased NBI coverage					
	Performance Targets					
Indicators	Base Year	Base Line	2023/24	2024/25	2025/26	2026/27
%age of districts headquarters connected to the NBI.	2020/21	70%	90%	95%	100%	100%
Intermediate Outcome Indicators:	Increased public participation in the fight against corruption					
	Performance Targets					
Indicators	Base Year	Base Line	2023/24	2024/25	2025/26	2026/27
Percentage of IG complaints registered and managed through integrated ICT platform including feedback	2020/21	75%	90%	80%	85%	90%
Intermediate Outcome Indicators:	Reduced costs of service delivery					
	Performance Targets					
Indicators	Base Year	Base Line	2023/24	2024/25	2025/26	2026/27
Proportion of government services provided online (%)	2020/21	30%	72%	80%	85%	90%

P3: Medium Term Budget Allocations by Sub-Programme and Vote

Table P3.1: Proposed Budget Allocations and Medium Term Projections by Sub-Programme

Billion Uganda Shillings	FY2022/23	2023/24	Medium Term Projections			
	Approved Budget	Proposed Budget	2024/25	2025/26	2026/27	2027/28
01 Strengthening Accountability	75.935	54.285	61.330	65.674	171.193	96.577
02 Government Structures and Systems	10.815	33.134	36.827	33.739	33.935	34.335
03 Human Resource Management	122.603	105.923	103.893	126.734	67.728	140.844
04 Decentralization and Local Economic Development	8.489	11.305	9.875	15.846	20.963	22.063
05 Business Process Re-engineering and Information Management		3.520	3.590	4.308	5.816	5.816
**Total for the Programme**	**222.692**	**208.167**	**215.516**	**246.302**	**299.635**	**299.635**

Table P3.2: Proposed Budget Allocations and Medium Term Projections by Vote

PUBLIC SECTOR TRANSFORMATION

Billion Uganda Shillings	FY2022/23		2023/24	Medium Term Projections			
	Approved Budget	Spent by End Sep	Proposed Budget	2024/25	2025/26	2026/27	2027/28
005 Ministry of Public Service	29.640	2.461	32.560	33.278	39.476	52.225	52.225
011 Ministry of Local Government	32.680	2.923	42.393	43.666	50.073	61.924	61.924
020 Ministry of ICT and National Guidance		0	2.440	2.489	2.987	4.032	4.032
023 Ministry of Kampala Capital City and Metropolitan Affairs	9.665	0.711	1.280	1.320	1.533	1.928	1.928
103 Inspectorate of Government (IG)	7.549	1.102	7.360	7.622	8.744	10.695	10.695
122 Kampala Capital City Authority (KCCA)	118.335	23.243	96.500	100.818	112.624	129.059	129.059
123 National Lotteries and Gaming Regulatory Board	4.828	0.887	2.484	2.608	2.869	3.156	3.156
126 National Information Technologies Authority		0	0.810	0.826	0.991	1.338	1.338
137 National Identification and Registration Authority (NIRA)		0	0.270	0.275	0.330	0.446	0.446
146 Public Service Commission (PSC)	11.487	2.247	12.890	13.218	15.517	20.082	20.082
147 Local Government Finance Commission (LGFC)	3.657	0.941	9.180	9.395	11.158	14.750	14.750
**Total for the Programme**	**222.692**	**34.664**	**208.167**	**215.516**	**246.302**	**299.635**	**299.635**

P4: PROGRAMME PRIORITIES IN LINE WITH INTERVENTIONS FOR FY2023/24

Programme Priorities FY2023/24	NDP III Programme Intervention
1. Local revenue enhancement management plans enhanced through the LG TPCs capacities to partake strategies that lead to self-reliance for LGs<br><br>2. Grants allocation formula and models in light of sector policies reviewed to in-build crosscutting issues like poverty, HIV/AIDS, gender and environment.<br><br>3.Technical support in identified areas of weaknesses in compliance with legal requirements LGs undertaken	Build LG fiscal decentralization and self-reliance capacity
1. Rewards and Sanctions Committees Constituted and operationalized in MDAs and LGs.<br><br>2. Sensitise MDAs and LGs on the rewards and sanctions framework and provide technical support to form the committees.<br><br>3. Hold Rewards and Sanctions committee meetings.	Design and implement a rewards and sanctions system

PUBLIC SECTOR TRANSFORMATION

Programme Priorities FY2023/24	NDP III Programme Intervention
1.Content data on NDP III Planning, Implementation, and performance monitoring and evaluation by various MDAs activities collected by UBC.<br><br>2.MDAs NDP III digital content (documentaries, feature stories, talk shows, and promotion materials) developed and broadcast by UBC.<br><br>3.Materials translated in selected languages.	Design and implement electronic citizen (e-citizen) system
1. Service Delivery Standards developed and disseminated in 8 MDAs and 24 LGs.<br><br>2. Compliance inspections undertaken in 16 MDAs and 40 LGs for Compliance to Service Delivery Standards.<br><br>3. Investigative inspections undertaken in 4 public institutions.<br><br>4. National service Delivery Survey Disseminated to 5 DLGs.<br><br>5. 4 Quarterly forum for key inspectorate agencies organized (1).<br><br>6. E-inspection tool piloted in 8MDAs and 10 LGs.<br><br>7. Investigative inspections undertaken in 4 public institutions.<br><br>8. Pearl of Africa Institutional Performance Assessment Scorecard(PAIPAS) administered in 16 MDAs and 40 LGs.<br><br>9.Verify contents of declarations for 800 Leaders and Public Officials.<br><br>10 .Investigate 100 cases of breach of the Leadership Code and make recommendations to LCT.<br><br>11.Resolve 560 Ombudsman complaints in MDALGs and make appropriate recommendations.<br><br>12. 56 urban councils selected from all the different regions inspected for compliance with existing laws and regulations and reports<br>produced.	Develop and enforce service and service delivery standards
Electronic Document and Records Management System (EDRMS) developed and rolled out to MDAs and LGs through Creating accounts and trainings for records managers in use of Electronic Document and Records Management System (EDRMS).	Develop and operationalize an e-document management system

PUBLIC SECTOR TRANSFORMATION

Programme Priorities FY2023/24	NDP III Programme Intervention
1.Talent Management Frame work disseminated and technical support provided to 40 MDAs and 60 LGs on the implementation of the framework.<br><br>2. Guidelines on professionalization of all cadres in Public Service disseminated to 24 MDAs.<br><br>3. A champion team on professionalization of all cadres in Public Service trained.<br><br>4. Payroll Mangers trained in Wage & Payroll Analysis in 100 LGS & 10 MDAs.<br><br>5. Guidelines on Management of Salary, Wage, Pension and gratuity developed and issued to the entire service.<br><br>6. Recruitment Plans from MDAs & LGs consolidated and staffing levels tracked 80 Votes.<br><br>7. Salaries Review Body established and operationalized.<br><br>8. Wage, pension and gratuity estimates from all MDAs and LGs prepared and submitted to MoFPED.<br><br>9. Curriculum on Pre & Post Retirement reviewed.<br><br>10. Training of Trainers on survival skills after retirement to ensure descent life provided to 30 HR Officers.<br><br>11. Technical & Functional Support to 10 MDAs and 100 LGs on Wage, Pension & Gratuity provided.	Empower MDAs to customize talent management (Attract, retain and motivate public servants)

PUBLIC SECTOR TRANSFORMATION

Programme Priorities FY2023/24	NDP III Programme Intervention
1. Records and information management policy and regulatory framework reviewed and developed including the NRIM Policy and NAM Policies developed, E records Management Guidelines and Disaster Management guidelines developed, Access and Use of Archives Regulation and Fees structure for services at NRCA regulations developed.<br><br>2. NRA Act, 2001 Retention and Disposal Schedule, Records Management Procedures Manual reviewed.<br><br>3. Compliance to RIM standards in 20 MDAs and 40 LGs assessed and technical support provided to address the identified gaps.<br><br>4. Capacity of 250 records officers built in records and Information Management.<br><br>5. Partnership in designing of training programmes established with 10 training institutions.<br><br>6. Public procurement compliance inspection on policy, Act and Regulations undertaken in selected 40 LGs from across all regions<br><br>7.Dissemination of Procurement Risk Identification Manual in 139 LGs under taken.<br><br>8.Compliance/spot check inspections carried out in 2 MDAs and reports disseminated to stake holders.<br>9. 560 citizens complaints concerning maladministration in public offices investigated and addressed	Enforce compliance to the rules and regulations

PUBLIC SECTOR TRANSFORMATION

Programme Priorities FY2023/24	NDP III Programme Intervention
1. MER strategy and system for for UBC and MDAs content development, broadcasting, promotion, and preservation activities formulated and operationalized<br><br>2. Local content for universal access to electronic Governance (e-Governance) services, e-citizen portal services, other private sector e-services, other communication channels developed and packaged (programmed)<br><br>3. Integrated data sharing bus developed and implemented<br><br>4. Data and information sharing platform developed (e.g. radios and TV, websites, web portals, dashboards and social media platforms<br><br>5.60 Government Ministries Departments engaged and sensitized on establishment of service Uganda Centers	Improve access to timely, accurate and comprehensible public information
1. Monitoring and evaluation of implementation of the parish model undertaken.<br><br>2. Documentaries on the status of implementation of the parish model and its impact on the beneficiary communities developed and broadcasted to the citizens.<br><br>3. Content data on NDP III Planning, Implementation, and performance monitoring and evaluation by various MDAs activities collected.	Operationalize the parish model
Public Service Act reviewed	Rationalize and harmonize policies to support public service delivery
1. Capacity building in e-government; Training, skilling and capacity building in the Public Service.<br><br>2. Identify and Integrate additional e-services onto the e-Citizens Portal.<br><br>3.Stakeholders sensitized on business process improvement<br><br>4. Four Government business processes Re-engineered. i.e. Management and Supply of Drugs System under MoH, Land Registration System under MoLHUD, Civil Marriages and Management System and Supply and Distribution of Agricultural inputs system under Agriculture	Reengineer public service delivery business processes

PUBLIC SECTOR TRANSFORMATION

Programme Priorities FY2023/24	NDP III Programme Intervention
1. A Transitional Framework/Plan for the implementation of the recommended structures under Rationalization developed and Implemented.<br><br>2. Comprehensive restructuring of 2 Ministries 4 Agencies and 11 LGs under taken and report produced.<br><br>3.Job Descriptions and Person Specifications of LGs and MDAs arising<br>out of Rationalization reviewed and Developed<br><br>4.Schemes of Service for 16 Cadres in Public Service Developed.<br><br>5.Records management systems set up in 5 MDAs and 35 LGs where they are lacking.	Review and develop management and operational structures, systems and standards
1.Provide technical support to MDAs and LGs to develop client charters	Review and strengthen the client chatter feedback mechanism to enhance the public demand for accountability
1.Existing Laws; Establishing the Salaries Review body reviewed<br><br>2.Institutional framework for implementation of "The Public Service (Negotiating, Consultative And Disputes Settlement Machinery) Act, 2008 established and operationalized.<br><br>3 .Recruitment and deployment guidelines of PWDs developed and operationalized.<br><br>4. Support Supervision on implementation of HR Policies and Procedures in 10 MDAs and 45 LGs provided.<br><br>5. Technical guidance on HR Policies and Procedures provided to 20 MDAs and 40 LGs.<br><br>6. Consultative Committees established and supported in 40 LGs<br><br>7. 100% of decisions of Appointing Authorities implemented.<br><br>8. 100% of Grievances and complaints from Public Service Labour Unions and individuals handled.	Review the existing legal, policy, regulatory and institutional frameworks to standardise regulation and benefits in the public service

PUBLIC SECTOR TRANSFORMATION

Programme Priorities FY2023/24	NDP III Programme Intervention
1.Reconfigure and Rollout the Human Resource Management System in support of the Programme approach to 88 MDAs and LGs.<br>2. Fully operationalise e-recruitment system and roll out to DSC across government.<br>3. Early Life Support to 160 HCM sites undertaken.<br>4. IPPS Recurrent Costs sustained.<br>5. Functional and technical Support provided to 30 problematic Votes.<br>6. Alignment of MDA establishment carried out<br>7. Targeted HCM users in 100 votes trained & HRs certified<br>8. Stakeholder Engagement and Change Management conducted<br>9. Employee master data, establishment and transaction data for 88 votes cleaned	Roll out the Human Resource Management System (Payroll management, productivity management, work leave, e-inspection)
1.Promotion of Local Economic Development through Implementation of the Local Economic Development Strategy<br><br>2. Local Government investment or economic profiles developed	Strengthen collaboration of all stakeholders to promote local economic development;
1.Annual surveys on status of development and implementation of client charter undertaken and report produced<br><br>2.Performance contracts for senior officers and implementation guidelines developed ,approved and disseminated as per the policy.<br><br>3.The long term pay policy of the Public Service implemented<br><br>4. Performance management tools reviewed and aligned to organizational plans using the balanced scorecard framework<br><br>5.Refresher training in performance Management conducted in 10 MDAs and 40 LGs<br><br>6. Refresher Training on the development of Client Charters and feedback mechanisms in 5 MDAs and 40 LGs carried out<br><br>7.Enforcement of Service and service delivery standards<br><br>8. Rewards and Sanctions Committees oriented in 10 MDAs and 40LGs in all regions	Strengthening public sector performance management

PUBLIC SECTOR TRANSFORMATION

Programme Priorities FY2023/24	NDP III Programme Intervention
1. Capacity of Human Resource Managers in the Public Service built in Strategic Human Resource Management.<br><br>2. Mind Set Change Program developed integrated in all training programs.<br><br>3. The Uganda Public Service Capacity Building Plan disseminated.<br><br>4. Technical support to 20 MDAs and 48 LGs to develop and implement Capacity building institutional plans provided.<br><br>5. Guidelines on professionalization of all cadres in Public Service disseminated to 24 MDAs	Undertake nurturing of civil servants through patriotic and long-term national service training

P5: GENDER AND EQUITY ISSUES AND INTERVENTIONS FOR FY2023/24

The Programme is also mindful of the gender and equity requirements as we finalize our Budget Framework Paper (BFP) for FY2023/24. In this context, its focus will be ;

Gender and Equity

1. Design and implement targeted corruption prevention and educational programs for youths, women, the elderly and Person with Disabilities
2. Ensure that Gender mainstreaming activities are conducted in MDAs.
3. ICT capacity building activities for women and differently abled persons.
4. Continued integration of GBV concerns in performance management
5. Coordination of Gender training and awareness campaigns on cyber security.
6. Extend broadband ICT infrastructure coverage countrywide in partnership with the private sector and all Government entities to implement last-mile connectivity to key areas (Districts, sub-counties, schools, hospitals, post offices, tourism sites, police)

HIV/AIDS

1. Provide psychosocial support to all HIV/AIDS affected Staff in partnership with HIV/AIDS organizations

Environment

1. Records management systems set up in 43 MDAs and LGs where they are lacking
2. Coordination of stakeholders in the implementation of the e-waste strategy undertaken
3. Awareness and sensitization carried out on proper disposal of electronic equipment

Covid-19

1. Develop and roll out specific e-Government services to support the fight against COVID-19 pandemic.
2. Provide technical support to entities with zoom licenses to reduce physical engagement

COMMUNITY MOBILIZATION AND MINDSET CHANGE

Foreword

The Ministry of Gender, Labour and Social Development (MoGLSD) is the lead implementing MDA of the Community Mobilization and Mind-set Change Programme (CMMCP) under a programme-based approach to planning that has been adopted by Government in line with the third National Development Plan (NDPIII). The CMMCP seeks to empower citizens, families and communities for increased responsibility and effective participation in sustainable national development; strengthen institutional capacity of central, local government and non-state actors for effective mobilization of communities; Promote and inculcate the National Vision and value system; and reduce negative cultural practices and attitudes.

The Programme directly contributes to the NDP III goal to increase household incomes and improved quality of life through increasing participation, productivity, inclusiveness and social well-being of the population. The programme derives its mandate from Chapter 4 Articles 31 – 40 and Chapter 16 Article 246 of the Constitution of the Republic of Uganda, which focus on empowerment of citizens, families and communities for increased responsibility and effective participation for sustainable national development.

The Programme registered some key milestones in the FY2021/22 and these include but not limited to 68 percent of the households participated in public development initiatives such as Emyooga, YLP, UWEP, OWC activates, among others; the percentage of vulnerable and marginalized persons empowered increased from 2.8 percent in FY 2020/21 to 3.7 percent in the FY 2021/22; the proportion of the youth engaged in national service also increased from 29.5 percent in FY 2020/21 to 34 percent 2021/22 ; the adult literacy rate has improved from 70.2 percent as the Plan as of 2017/18 to 76 percent in FY 2021/22 (MGLSD quarterly reports) also higher than the 74 percent of the FY 2020/21 attributed ICOLEW programme
The Pprogramme also reached out to 176 Local Governments to sensitize them on matters partnering the Parish Development Model in order to increase uptake of the Programme
Notwithstanding the above achievements, the Programme has faced a number of performance challenges. This includes; Inadequate budget, in addition to budget cuts affected implementation of some of the planned interventions thus impacting outcome performance; Effects of the COVID-19. These include but not limited to GBV, Child abuse among others. These effects are placing a heavy burden on the Ministry on the programme and its cadre structures in the Local Government due to inadequate resources;Some of the MDAs like, MoES, MoLG, MoFA, OPM were not allocated funds under the programme to implement the interventions; Limited use of ICT for execution of programme interventions among others.
It should be noted that much as the programme is expected to deliver key outputs during the NDP period, a sharp budget cut in which the Budget of the Programme reduced from Shs 75 Billion to 22 Billion in the next FY 2023/2024 is likely to negatively reverse the efforts already put in place in a bid to attain the core objectives of the programme

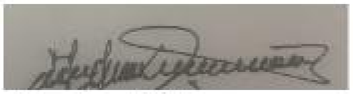

Aggrey David Kibenge

Permanent Secretary, Ministry of Gender, Labour and Social Development

COMMUNITY MOBILIZATION AND MINDSET CHANGE

Abbrevations and Acronyms

ACRONYM	ACRONYM NAME
CAOs	Chief Adminstrative Officers
CDOs	Community Development Officers
PLU	Public Library of Uganda
RFBOs	Religious and Faith Organisations

COMMUNITY MOBILIZATION AND MINDSET CHANGE

P1: PROGRAMME OVERVIEW

Snapshot of Medium Term Budget Allocations

Table P1.1 Overview of Programme Expenditure and Medium Term Allocations (Ush Billion)

Billion Uganda Shillings		FY2022/23		FY2023/24	MTEF Budget Projections			
		Approved Budget	Spent by End Sep	Proposed Budget	2024/25	2025/26	2026/27	2027/28
Recurrent	Wage	4.119	0.880	3.999	4.199	4.618	5.080	5.080
	Non Wage	67.459	7.056	13.000	13.260	15.912	21.481	21.481
Devt.	GoU	3.406	0.085	5.000	5.000	6.000	8.400	8.400
	ExtFin	0.000	0.000	0.000	0.000	0.000	0.000	0.000
	**GoU Total**	**74.984**	**8.020**	**21.999**	**22.459**	**26.530**	**34.962**	**34.962**
	**Total GoU+Ext Fin (MTEF)**	**74.984**	**8.020**	**21.999**	**22.459**	**26.530**	**34.962**	**34.962**
	A.I.A	0.000	0.000	0.000	0.000	0.000	0.000	0.000
	**Grand Total**	**74.984**	**8.020**	**21.999**	**22.459**	**26.530**	**34.962**	**34.962**

Programme Strategy and linkage to the National Development Plan

Programme Goal: To empower families, communities and citizens to embrace national values and actively participate in sustainable development

Objectives

1. Enhance effective mobilization of families, communities and citizens for national development;
2. Strengthen institutional capacity of central and local government and non-state actors for effective mobilization of communities;
3. Promote and inculcate the National Vision and value system; and
4. Reduce negative cultural practices and attitudes

The Programme contributes meaningfully to the attainment of all the NDP programs as it supports Government in its efforts to ensure increased uptake of Government development efforts

P2: Highlights Of Programme Projected Performance

Table P2.1 Programme Outcomes Indicators

**Programme Outcome**	Informed and active citizenry and uptake of development interventions					
**Programme Objectives contributed to by the Intermediate Outcome**						
Enhance effective mobilization of families, communities and citizens for national development.						
	Performance Targets					
**Programme Outcome Indicators**	**Base Year**	**Base Line**	**2023/24**	**2024/25**	**2025/26**	**2026/27**
Adult literacy rate (%) - Total - Female	2020	80%	70%	60%	65%	70%
Adult literacy rate (%) - Total - Male	2020	80	68%	72%	75%	79.5
Adult literacy rate (%) - Total - Total	2020	72.2	72%	75%	77.5%	80%

COMMUNITY MOBILIZATION AND MINDSET CHANGE

**Programme Outcome**	Informed and active citizenry and uptake of development interventions					
**Programme Objectives contributed to by the Intermediate Outcome**						
Enhance effective mobilization of families, communities and citizens for national development.						
	**Performance Targets**					
**Programme Outcome Indicators**	**Base Year**	**Base Line**	**2023/24**	**2024/25**	**2025/26**	**2026/27**
Percentage of Households participating in public development initiatives	2020	30%	33%	34%	35%	38%
Proportion of the population informed about national programmes	2020	60%	63%	64%	66%	70%
**Programme Outcome**	Increased household saving					
**Programme Objectives contributed to by the Intermediate Outcome**						
Enhance effective mobilization of families, communities and citizens for national development.						
	**Performance Targets**					
**Programme Outcome Indicators**	**Base Year**	**Base Line**	**2023/24**	**2024/25**	**2025/26**	**2026/27**
Households participation in a saving schemes (%)	2020	30%	32%	33%	35%	40%
**Programme Outcome**	Increased investment in family and parenting agenda					
**Programme Objectives contributed to by the Intermediate Outcome**						
Enhance effective mobilization of families, communities and citizens for national development.						
	**Performance Targets**					
**Programme Outcome Indicators**	**Base Year**	**Base Line**	**2023/24**	**2024/25**	**2025/26**	**2026/27**
Proportion of population accessing the minimum family care package	2020	1%	2%	2.5%	3%	4%
**Programme Outcome**	Improved morals, positive mindsets, attitudes and patriotism					
**Programme Objectives contributed to by the Intermediate Outcome**						
Promote and inculcate the national Vision and value system						
	**Performance Targets**					
**Programme Outcome Indicators**	**Base Year**	**Base Line**	**2023/24**	**2024/25**	**2025/26**	**2026/27**
A shared national value system in place	2020	In Place	In Place	In Place	In Place	In Place
**Programme Outcome**	Kiswahili as an official language in Uganda promoted					
**Programme Objectives contributed to by the Intermediate Outcome**						
Promote and inculcate the national Vision and value system						
	**Performance Targets**					
**Programme Outcome Indicators**	**Base Year**	**Base Line**	**2023/24**	**2024/25**	**2025/26**	**2026/27**
Proportion of population able to speak, write and read Kiswahili	2020	10%	14%	15%	20%	25%

COMMUNITY MOBILIZATION AND MINDSET CHANGE

**Programme Outcome**	Enhanced social cohesion and participation in cultural life						
**Programme Objectives contributed to by the Intermediate Outcome**							
Reduce negative cultural practices and attitudes							
			**Performance Targets**				
**Programme Outcome Indicators**	**Base Year**	**Base Line**	**2023/24**	**2024/25**	**2025/26**	**2026/27**	
Proportion of population practicing negative cultural practices	2020	0.3	0.35	0.4	0.45	0.5	
**Programme Outcome**	Empowered communities for participation in the development process						
**Programme Objectives contributed to by the Intermediate Outcome**							
Strengthen institutional capacity of central and local governments and non-state actors for effective mobilization of communities							
			**Performance Targets**				
**Programme Outcome Indicators**	**Base Year**	**Base Line**	**2023/24**	**2024/25**	**2025/26**	**2026/27**	
% of vulnerable and marginalized persons empowered	2020	15%	18%	20%	25%	30%	

Table P2.2: Intermediate Outcomes Indicators

**Sub-Programme Name:**	**Community sensitization and empowerment**					
**Intermediate Outcome Indicators:**	Improved uptake of Government programmes by residents, families, communities and citizens f					
			**Performance Targets**			
**Indicators**	**Base Year**	**Base Line**	**2023/24**	**2024/25**	**2025/26**	**2026/27**
Proportion of households participating in development initiatives increased	2020	25%	30%	35%	40%	45%
Proportion of the population informed about national programmes	2020	30%	25%	35%	40%	45%
**Sub-Programme Name:**	**Strengthening institutional support**					
**Intermediate Outcome Indicators:**	Community Development Initiatives in place					
			**Performance Targets**			
**Indicators**	**Base Year**	**Base Line**	**2023/24**	**2024/25**	**2025/26**	**2026/27**
Response rate to development initiatives (%)	2020	30%	20%	25%	30%	35%
**Intermediate Outcome Indicators:**	Empowered communities for participation					
			**Performance Targets**			
**Indicators**	**Base Year**	**Base Line**	**2023/24**	**2024/25**	**2025/26**	**2026/27**
% of vulnerable and marginalized persons empowered	2020	35%	40%	40%	45%	47%

P3: Medium Term Budget Allocations by Sub-Programme and Vote

Table P3.1: Proposed Budget Allocations and Medium Term Projections by Sub-Programme

COMMUNITY MOBILIZATION AND MINDSET CHANGE

Billion Uganda Shillings	FY2022/23	2023/24	Medium Term Projections			
	Approved Budget	Proposed Budget	2024/25	2025/26	2026/27	2027/28
02 Strengthening institutional support	16.473	16.295	15.907	17.236	22.986	21.786
**Total for the Programme**	**74.984**	**21.999**	**22.459**	**26.530**	**34.962**	**34.962**

Table P3.2: Proposed Budget Allocations and Medium Term Projections by Vote

Billion Uganda Shillings	FY2022/23		2023/24	Medium Term Projections			
	Approved Budget	Spent by End Sep	Proposed Budget	2024/25	2025/26	2026/27	2027/28
018 Ministry of Gender, Labour and Social Development	24.319	2.979	18.369	18.707	22.200	29.586	29.586
020 Ministry of ICT and National Guidance	24.768	1.369	1.020	1.071	1.178	1.296	1.296
119 Uganda Registration Services Bureau (URSB)	0.685	0.093	0.300	0.315	0.347	0.381	0.381
124 Equal Opportunities Commission	0.782	0.135	2.000	2.040	2.448	3.305	3.305
149 National Population Council	1.475	0.109	0.310	0.326	0.358	0.394	0.394
**Total for the Programme**	**74.984**	**8.020**	**21.999**	**22.459**	**26.530**	**34.962**	**34.962**

P4: PROGRAMME PRIORITIES IN LINE WITH INTERVENTIONS FOR FY2023/24

**Programme Priorities FY2023/24**	**NDP III Programme Intervention**
Kiswahili Council operationalized	Conduct awareness campaigns and enforce laws enacted against negative and/or harmful religious, traditional/cultural practices and beliefs.
Revised National Culture policy disseminated to 80 Local Governments	Conduct awareness campaigns and enforce laws enacted against negative and/or harmful religious, traditional/cultural practices and beliefs.
80 Local Governments monitored and provided with technical support backstopping on the implementation of the National Culture priority interventions and family and parenting guidelines	Conduct awareness campaigns and enforce laws enacted against negative and/or harmful religious. traditional/cultural practices and beliefs.
14 Cultural Leaders supported with monthly emoluments to mobilize communities for uptake of government programmes.	Conduct awareness campaigns and enforce laws enacted against negative and/or harmful religious, traditional/cultural practices and beliefs.
Uganda National Cultural Center supported with Monthly wage subvention during FY 2023/24	Conduct awareness campaigns and enforce laws enacted against negative and/or harmful religious, traditional/cultural practices and beliefs.
Mapping Study Report of the Culture and Creative Sector prepared and disseminated to 80 Local Governments.	Conduct awareness campaigns and enforce laws enacted against negative and/or harmful religious, traditional/cultural practices and beliefs.

COMMUNITY MOBILIZATION AND MINDSET CHANGE

Programme Priorities FY2023/24	NDP III Programme Intervention
Capacity building of 176 DCDOs and 50 National Facilitators on implementation of Parish Development Model conducted at National Level.	Equip and operationalize Community Mobilization and Empowerment (CME) institutions/structures of central, local government and non-state actors for effective citizen mobilization and dissemination of information to guide and shape the mindsets/attitudes of the population
Mapping of NGOs implementing Social Development Sector activities undertaken in 20 Local Governments.	Equip and operationalize Community Mobilization and Empowerment (CME) institutions/structures of central, local government and non-state actors for effective citizen mobilization and dissemination of information to guide and shape the mindsets/attitudes of the population
Creating awareness on adult learning and education in Uganda to increase participation and uptake of Government programmes through commemoration of the International Literacy Day on 8th September, 2023	Equip and operationalize Community Mobilization and Empowerment (CME) institutions/structures of central, local government and non-state actors for effective citizen mobilization and dissemination of information to guide and shape the mindsets/attitudes of the population
Joint Monitoring of the performance of CMMC Pillar of the Parish Development Model conducted in 176 Local Governments to harmonize implementation and improve performance of Government programmes.	Equip and operationalize Community Mobilization and Empowerment (CME) institutions/structures of central, local government and non-state actors for effective citizen mobilization and dissemination of information to guide and shape the mindsets/attitudes of the population
Learning programmes for RTCs and 10 Community Development Centers as hubs of service delivery and form management Committees for their operationalization developed	Equip and operationalize Community Mobilization and Empowerment (CME) institutions/structures of central, local government and non-state actors for effective citizen mobilization and dissemination of information to guide and shape the mindsets/attitudes of the population
Budget Framework Paper for FY 2023/2024 prepared and submitted to relevant authorities.	Equip and operationalize Community Mobilization and Empowerment (CME) institutions/structures of central, local government and non-state actors for effective citizen mobilization and dissemination of information to guide and shape the mindsets/attitudes of the population
A report on emerging issues on the Budget for FY 2024/2025 prepared for PACOB, Inter Ministerial and Parliament	Equip and operationalize Community Mobilization and Empowerment (CME) institutions/structures of central, local government and non-state actors for effective citizen mobilization and dissemination of information to guide and shape the mindsets/attitudes of the population
Ministerial Policy statement for FY 2023/2024 prepared, printed and submitted to relevant authorities.	Equip and operationalize Community Mobilization and Empowerment (CME) institutions/structures of central, local government and non-state actors for effective citizen mobilization and dissemination of information to guide and shape the mindsets/attitudes of the population
Budget for FY 2023/2024 prepared and submitted to relevant authorities.	Equip and operationalize Community Mobilization and Empowerment (CME) institutions/structures of central, local government and non-state actors for effective citizen mobilization and dissemination of information to guide and shape the mindsets/attitudes of the population
Quarterly performance progress reports prepared and submitted to Ministry of Finance and Economic Development (MoFPED).	Equip and operationalize Community Mobilization and Empowerment (CME) institutions/structures of central, local government and non-state actors for effective citizen mobilization and dissemination of information to guide and shape the mindsets/attitudes of the population
Ministry Departments and agencies supported on Strategic Planning, Budgeting Monitoring and Evaluation.	Equip and operationalize Community Mobilization and Empowerment (CME) institutions/structures of central, local government and non-state actors for effective citizen mobilization and dissemination of information to guide and shape the mindsets/attitudes of the population

COMMUNITY MOBILIZATION AND MINDSET CHANGE

Programme Priorities FY2023/24	NDP III Programme Intervention
Regulatory Impact Assessment reports prepared	Equip and operationalize Community Mobilization and Empowerment (CME) institutions/structures of central, local government and non-state actors for effective citizen mobilization and dissemination of information to guide and shape the mindsets/attitudes of the population
Implementation status of Cabinet decisions/ directives and Sectoral public policies in the MDA monitored and evaluated	Equip and operationalize Community Mobilization and Empowerment (CME) institutions/structures of central, local government and non-state actors for effective citizen mobilization and dissemination of information to guide and shape the mindsets/attitudes of the population
Public Policy Research Agenda compiled and updated	Equip and operationalize Community Mobilization and Empowerment (CME) institutions/structures of central, local government and non-state actors for effective citizen mobilization and dissemination of information to guide and shape the mindsets/attitudes of the population
Annual Performance Assessment Report on MGLSD Strategic Plan 2020/21-24/25 prepaared	Equip and operationalize Community Mobilization and Empowerment (CME) institutions/structures of central, local government and non-state actors for effective citizen mobilization and dissemination of information to guide and shape the mindsets/attitudes of the population
Draft Budget estimates for FY 2024/25 prepared, submitted to MoFPED and other relevant authorities	Equip and operationalize Community Mobilization and Empowerment (CME) institutions/structures of central, local government and non-state actors for effective citizen mobilization and dissemination of information to guide and shape the mindsets/attitudes of the population
Ministry Client charter finalized	Equip and operationalize Community Mobilization and Empowerment (CME) institutions/structures of central, local government and non-state actors for effective citizen mobilization and dissemination of information to guide and shape the mindsets/attitudes of the population
Performance Management Initiatives coordinated	Equip and operationalize Community Mobilization and Empowerment (CME) institutions/structures of central, local government and non-state actors for effective citizen mobilization and dissemination of information to guide and shape the mindsets/attitudes of the population
Quarterly Audit reports prepared and submitted to relevant authorities	Equip and operationalize Community Mobilization and Empowerment (CME) institutions/structures of central, local government and non-state actors for effective citizen mobilization and dissemination of information to guide and shape the mindsets/attitudes of the population
Quarterly reports of political Monitoring and Oversight activities prepared	Equip and operationalize Community Mobilization and Empowerment (CME) institutions/structures of central, local government and non-state actors for effective citizen mobilization and dissemination of information to guide and shape the mindsets/attitudes of the population
Strategic collaborations and partnerships facilitated	Equip and operationalize Community Mobilization and Empowerment (CME) institutions/structures of central, local government and non-state actors for effective citizen mobilization and dissemination of information to guide and shape the mindsets/attitudes of the population

P5: GENDER AND EQUITY ISSUES AND INTERVENTIONS FOR FY2023/24

Key Issues
1. Knowledge gap in mainstreaming gender and equity issues in MDAs plans
2. Declining performance of MDAs and LG on Gender and Equity Planning and Budgeting

COMMUNITY MOBILIZATION AND MINDSET CHANGE

Key Interventions
1. Capacity building on gender and equity mainstreaming

GOVERNANCE AND SECURITY

Foreword

Rt. Honorable Speaker, in line with Section 9(1) of the Public Finance Management Act, 2015 as amended and as guided by paragraph 2 of the First Budget Call Circular (BCC) issued by the Ministry of Finance, Planning and Economic Development (MFPED) on September 15th, 2022, I have the honor to present to you the Governance and Security Program (GSP) Budget Frame Work Paper (BFP) for Financial Year 2023/2024.

The GSP, comprises of thirty-one (31) Institutions which contribute to the NDP III objective five which is; to strengthen the role of the State in development. The Program has sole responsibility for upholding the rule of law, ensuring security, maintaining law and order, public policy governance, administration of justice, promoting human rights, accountability and transparency.

Rt. Honorable Speaker, the preparation and presentation of this BFP has similarly been aligned to; the Programmatic approach; the NRM Manifesto 2021/26; as well as the Parish Development Model (PDM) - which is the last mile service delivery vehicle to the citizenry. Specifically, to two critical PDM Pillars namely; Governance & Administration, and Mindset Change.

The program BFP provides information on registered achievements during the first half of FY 2022/2023, the planned interventions for FY 2023/2024 and the associated resources required to implement our work plan for the coming Financial Year and medium term.

The Program is committed to ensuring that all the activities to be undertaken significantly contribute towards the transformation of the country over the long term into a prosperous and modern society as envisaged in Vision 2040.

Hon. Speaker and Members, permit me therefore to thank Parliament and other stakeholders for the valuable support extended to the GSP which has enabled us to implement our mandate during the FY 2022/23.

In the same vain, I appeal to the honorable Members of the 11th Parliament to support and approve the proposed budget of UGX 6.795Bn to enable the Program to execute its mandate during FY 2023/24.
For God and My Country

Haji Yunus Kakande
SECRETARY-OFFICE OF THE PRESIDENCY

GOVERNANCE AND SECURITY

Abbrevations and Acronyms

ACRONYM	ACRONYM NAME
ACD	Anti-Corruption Division
ADC	Austrian Development Cooperation
ADR	Alternative Dispute Resolution
ASTU	Anti-Stock Theft Unit
BDR	Birth and Death Registration
CAO	Chief Administrative Officer
CCAS	Court Case automation system
CDO	Community Development Officer
CFPOs	Child and Family Protection Officers
CFPU	Child and Family Protection Unit
CID	Criminal Investigations Directorate
DCI	Directorate of Crime Intelligence
DCIC	Directorate of Citizenship and Immigration Control
DCS	Directorate of Community Service
DCSC	District Community Service Committee
DEI	Directorate for Ethics and Integrity
DGAL	Directorate of Government Analytical Laboratory
DLAS	Directorate of Legal Advisory Services
DNA	Deoxyribonucleic Acid
DPC	District Police Commander
EAC	East African Community
EC	Electoral Commission
ESO	External Security Organization
FAL	Functional Adult Literacy
FIA	Finance Intelligence Authority
HRBA	Human Rights Based Approach
IEC	Information Education and Communication
IFMS	Integrated Financial Management System
IGG	Inspectorate of Government
ISO	Internal Security Organization
J4C	Justice for Children
JCU	Justice Centers Uganda
JLOS	Justice Law and Order Sector

GOVERNANCE AND SECURITY

ACRONYM	ACRONYM NAME
JLOSIC	Justice, Law and Order Sector Integrity Committee
JSC	Judicial Service Commission
KCCA	Kampala City Council Authority
KMP	Kampala Metropolitan
LASP	Legal Aid Service Provider
LASPNET	Legal Aid Service Providers Network
LCC	Local Council Court
LCCA	Local Council Courts Act
LCV	Local Council Five
LDC	Law Development Centre
MDAs	Ministries, Agencies and Departments
MEACA	Ministry of East African Community Affairs
MIA	Ministry of Internal Affairs
MoDVA	Ministry of Defense and Veteran Affairs
MoFA	Ministry of Foreign Affairs
MoGLSD	Ministry of Gender, Labour and Social Development
MoJCA	Ministry of Justice and Constitutional Affairs
MoLG	Ministry of Local Government
MPS	Ministry of Public Service
NCIC	National Citizenship and Immigration Control
NFP	National Focal Point (For Light Arms and Small Weapons)
NGOs	Non-Governmental Organization
NIRA	National Identification and Registration Authority
OAG	Office of the Auditor General
ODPP	Office of the Directorate of Public Prosecutions
OP	Office of the President
OPM	Office of the Prime Minister
PDU	Procurement and Disposal Unit
PPDA	Public Procurement and Disposal of Public Assets Authority
PPTA	Prevention and Prohibition of Torture Act
PPU	Policy and Planning Units
PWD	Persons with Disabilities
RCC	Regional Chain-linked Committee
RIA	Regulatory Impact Assessment
RLP	Refugee Law Project

GOVERNANCE AND SECURITY

ACRONYM	ACRONYM NAME
RSA	Resident State Attorney
SALW	Small Arms and Light Weapons
SGBV	Sexual and Gender Based Violence
STA	Senior Technical Advisor
TAT	Tax Appeals Tribunal
TIA	Trial on Indictments Act
UGX	Uganda Shillings
UHRC	Uganda Human Rights Commission
ULRC	Uganda Law Reform Commission
ULS	Uganda Law Society
UPDF	Uganda Peoples' Defence Force
UPF	Uganda Police Force
UPS	Uganda Prisons Service
URA	Uganda Revenue Authority
URSB	Uganda Registration Services Bureau
VAC	Violence Against Children

GOVERNANCE AND SECURITY

P1: PROGRAMME OVERVIEW

Snapshot of Medium Term Budget Allocations

Table P1.1 Overview of Programme Expenditure and Medium Term Allocations (Ush Billion)

Billion Uganda Shillings		FY2022/23		FY2023/24	MTEF Budget Projections			
		Approved Budget	Spent by End Sep	Proposed Budget	2024/25	2025/26	2026/27	2027/28
Recurrent	Wage	1,476.633	472.455	1,477.956	1,561.275	1,714.485	1,883.017	1,883.017
	Non Wage	2,772.333	546.006	2,768.932	2,821.428	3,304.707	4,547.246	4,547.246
Devt.	GoU	2,447.897	493.213	2,344.384	2,344.384	2,802.683	3,285.548	3,285.548
	ExtFin	470.453	3.423	233.615	0.000	0.000	0.000	0.000
	**GoU Total**	**6,696.862**	**1,511.675**	**6,591.272**	**6,727.087**	**7,821.876**	**9,715.811**	**9,715.811**
**Total GoU+Ext Fin (MTEF)**		**7,167.315**	**1,515.097**	**6,824.887**	**6,727.087**	**7,821.876**	**9,715.811**	**9,715.811**
	A.I.A	0.000	0.000	0.000	0.000	0.000	0.000	0.000
	**Grand Total**	**7,167.315**	**1,515.097**	**6,824.887**	**6,727.087**	**7,821.876**	**9,715.811**	**9,715.811**

Programme Strategy and linkage to the National Development Plan

The Governance and Security Programme (GSP) brings together 32 Government Agencies responsible for upholding the rule of law, ensuring security, maintaining law and order, public policy governance, administration of justice, promoting human rights, accountability and transparency. This program contributes to the National Development plan (NDPIII) objective five which is; to strengthen the role of the state in Development.

The Vision of the program is "An integrated people-centered governance and security service delivery system for economic and social transformation. The Mission is to "Promote the rule of law and ensure peace and security in Uganda."

Good governance is essential for enabling development in economic, political, environmental and social sectors. Efficient political and economic strands promote social order thus a stable, predictable and secure political environment is a prerequisite for socioeconomic development. This involves protecting gains from internal instability and external aggression. The society fabric at the individual, household, community and national levels must be at peace for any development to take place.

The rule of law is the foundation of a free society that places limits on Government authority such that all citizens of that society including the rulers, lawmakers, Judges, and members of all social classes are equally subject to a common set of laws and by implication, are not subject to Government action that is unsupported by the law.

The 2030 Agenda (SDG 16) recognizes the need to build peaceful, just and inclusive societies that provide equal access to justice and strong institutions. Aspiration 3 of Agenda 2063 is to have an Africa of good governance, democracy, and respect for human rights, justice and the rule of law. Pillar 3.6 of the EAC Vision 2050, calls for democratic values, human rights, access to justice and the rule of law entrenched in all East African Partner States. According to the Uganda Vision 2040, the tenets of good governance include constitutional democracy, protection of human rights, rule of law, political and electoral processes, transparency and accountability, government effectiveness and regulatory quality and security. Particularly Vision 2040 identified human rights observance as a critical feature of good governance and the rule of law.

GOVERNANCE AND SECURITY

[illegible]

However, as highlighted in the NDP III, weak adherence to the rule of law and the existence of internal and external security threats are still major challenges. This is due to (i) weak policy, legal and regulatory frameworks for effective governance; (ii) weak business support environment; (iii) low respect for and observance of human rights and fundamental freedoms; (iv) limited access to and affordability of justice; (v) high crime rates (vi) weak societal security structure and (vii) low recovery

P2: Highlights Of Programme Projected Performance

Table P2.1 Programme Outcomes Indicators

**Programme Outcome**	Enhanced National response to refugee protection and management					
**Programme Objectives contributed to by the Intermediate Outcome**						
Enhance Refugee protection and Migration Management						
			**Performance Targets**			
**Programme Outcome Indicators**	**Base Year**	**Base Line**	**2023/24**	**2024/25**	**2025/26**	**2026/27**
Level of implementation of the Settlement Transformative Agenda, %	2018	40%	90%	90%	100%	100%
**Programme Outcome**	Effective and efficient JLOS business processes					
**Programme Objectives contributed to by the Intermediate Outcome**						
Reform and strengthen JLOS business processes to facilitate private sector development						
			**Performance Targets**			
**Programme Outcome Indicators**	**Base Year**	**Base Line**	**2023/24**	**2024/25**	**2025/26**	**2026/27**
Conviction rate	2018	61%	62%	62%	63%	63%
Percentage of districts with one stop frontline JLOS service points	2018	67.5%	75%	75.5%	77%	78%
Public satisfaction in the Justice system	40%	2018	70%	70%	70%	70%
**Programme Outcome**	Effective citizen participation in the governance and democratic processes					
**Programme Objectives contributed to by the Intermediate Outcome**						
Strengthen citizen participation and engagement in the democratic processes						
			**Performance Targets**			
**Programme Outcome Indicators**	**Base Year**	**Base Line**	**2023/24**	**2024/25**	**2025/26**	**2026/27**
Democratic index	6.5%	2018	7.9%	8.6%	8.8%	9.0%
**Programme Outcome**	Free and fair elections					
**Programme Objectives contributed to by the Intermediate Outcome**						
Strengthen citizen participation and engagement in the democratic processes						
			**Performance Targets**			
**Programme Outcome Indicators**	**Base Year**	**Base Line**	**2023/24**	**2024/25**	**2025/26**	**2026/27**
% of citizens engaged in electoral process	80%	2018	40%	50%	60%	60%
Proportion of eligible voters registered	62.5%	2018	80%	90%	95%	95%
Proportion of registered election disputes analysed and resolved	75%	2018	84%	84%	82%	84%

GOVERNANCE AND SECURITY

**Programme Outcome**	Increased observance of Human Rights					
**Programme Objectives contributed to by the Intermediate Outcome**						
Strengthen compliance with the Uganda Bill of Rights						
	**Performance Targets**					
**Programme Outcome Indicators**	**Base Year**	**Base Line**	**2023/24**	**2024/25**	**2025/26**	**2026/27**
Disposal rate of Huma Rights cases, %	30%	2018	40%	40%	45%	45%
Proportion of human rights recommendations implemented	60%	2018	90%	95%	95%	95%
Proportion of remand prisoners	48.0	2018	48.5	48.5	48.5	48
**Programme Outcome**	Increased access to Justice					
**Programme Objectives contributed to by the Intermediate Outcome**						
Strengthen people centered delivery of security, justice, law and order services						
	**Performance Targets**					
**Programme Outcome Indicators**	**Base Year**	**Base Line**	**2023/24**	**2024/25**	**2025/26**	**2026/27**
% of backlog cases in the system	2018	18	32%	32%	30%	28%
Rate of recidivism	2018	17.2	14%	14%	13.8%	13.6%
**Programme Outcome**	Effective governance and security					
**Programme Objectives contributed to by the Intermediate Outcome**						
Strengthen policy, legal, regulatory and Institutional frameworks for effective governance and security						
	**Performance Targets**					
**Programme Outcome Indicators**	**Base Year**	**Base Line**	**2023/24**	**2024/25**	**2025/26**	**2026/27**
Laws enacted as a % of those presented	2018	65%	85%	90%	90%	92%
**Programme Outcome**	Efficiency and effectiveness of institutions responsible for security, law, and order					
**Programme Objectives contributed to by the Intermediate Outcome**						
Strengthen the capacity of security agencies to address emerging security threats						
	**Performance Targets**					
**Programme Outcome Indicators**	**Base Year**	**Base Line**	**2023/24**	**2024/25**	**2025/26**	**2026/27**
Level of Combat readiness, %	2018	High	100%	High	High	High
Level of public trust in JLOS, %	2018	59%	65%	65%	67%	67%
Proportion of annually planned equipment acquired, %	2018	33	60%	70%	70%	75%
Proportion of security personnel with advanced training, %	2018	5	30%	45%	45%	50%
Turnaround time (hours/days)	2018	12	3	2	2	2

GOVERNANCE AND SECURITY

**Programme Outcome**	Increased peace and stability					
**Programme Objectives contributed to by the Intermediate Outcome**						
Strengthen the capacity of security agencies to address emerging security threats						
			**Performance Targets**			
**Programme Outcome Indicators**	**Base Year**	**Base Line**	**2023/24**	**2024/25**	**2025/26**	**2026/27**
Enrolment in the National service	2018	0%	0%	2%	3%	5%
Level of public confidence in the security system, %	2018	High	100%	High	High	High
Level of response to emerging security threats	2018	High	High	High	High	High
Peace Index	2018	2.196	1.8%	1.72	1.7	1.7
Proportion of armed or organized criminal groups (%)	2018	100%	100%	100%	100%	100%
**Programme Outcome**	Increased safety of person and security of property					
**Programme Objectives contributed to by the Intermediate Outcome**						
Strengthen the capacity of security agencies to address emerging security threats						
			**Performance Targets**			
**Programme Outcome Indicators**	**Base Year**	**Base Line**	**2023/24**	**2024/25**	**2025/26**	**2026/27**
Absence of IDPs due to conflicts	2018	0	0	0	0	0
Crime rate	2018	667	400%	372	372	372
Incidence of crime committed using small arms and light weapons	2018	262	232	232	232	232
**Programme Outcome**	Reduced corruption					
**Programme Objectives contributed to by the Intermediate Outcome**						
Strengthen transparency, accountability and anti-corruption systems						
			**Performance Targets**			
**Programme Outcome Indicators**	**Base Year**	**Base Line**	**2023/24**	**2024/25**	**2025/26**	**2026/27**
Corruption Perception Index	2018	26	30.1%	30.1%	30.1%	30.1%
**Programme Outcome**	Increased transparency and accountability					
**Programme Objectives contributed to by the Intermediate Outcome**						
Strengthen transparency, accountability and anti-corruption systems						
			**Performance Targets**			
**Programme Outcome Indicators**	**Base Year**	**Base Line**	**2023/24**	**2024/25**	**2025/26**	**2026/27**
ACD conviction rate of Corruption cases	2018	57%	74%	75%	75%	75%
IG conviction rate of Corruption cases	2018	73.5%	50%	50%	50%	50%
ODDP conviction rate of Corruption cases	2018	74%	80%	80%	80%	80%

Table P2.2: Intermediate Outcomes Indicators

GOVERNANCE AND SECURITY

**Sub-Programme Name:**	**Security**					
**Intermediate Outcome Indicators:**	Reduced incidences of trafficking persons					
	**Performance Targets**					
**Indicators**	**Base Year**	**Base Line**	**2023/24**	**2024/25**	**2025/26**	**2026/27**
Incidences of trafficking in persons	2018	165	135	135	135	135
**Intermediate Outcome Indicators:**	Timely and Reliable External Intelligence					
	**Performance Targets**					
**Indicators**	**Base Year**	**Base Line**	**2023/24**	**2024/25**	**2025/26**	**2026/27**
Number of days taken to generate an intelligence report	2018	1	1	1	1	1
Number of Intelligence reports generated	2018	780	780	780	780	780
Level of combat readiness	2018	Medium	High	High	High	High
**Sub-Programme Name:**	**Access to Justice**					
**Intermediate Outcome Indicators:**	Increased observance of Human Rights					
	**Performance Targets**					
**Indicators**	**Base Year**	**Base Line**	**2023/24**	**2024/25**	**2025/26**	**2026/27**
Disposal rate of Human Rights cases, %	2018	30	40%	40%	45%	50%
Proportion of citizens aware of the provisions of the bill of rights	2018	30%	75%	75%	75%	75%
Proportion of remand prisoners	2018	48%	45.5%	45%	45%	45%
**Intermediate Outcome Indicators:**	Enhanced access to travel documents					
	**Performance Targets**					
**Indicators**	**Base Year**	**Base Line**	**2023/24**	**2024/25**	**2025/26**	**2026/27**
Proportion of work permits issued for investments, %	2018	10	22%	25%	25%	27%
Average time taken to issue passports (days)	2018	14	2	2	2	2
**Intermediate Outcome Indicators:**	Increased access to Justice					
	**Performance Targets**					
**Indicators**	**Base Year**	**Base Line**	**2023/24**	**2024/25**	**2025/26**	**2026/27**
Level of public awareness about laws and administration of justice, %	2018	23	42%	42%	43%	45%
Recidivism rate	2018	17.2	13.2%	13.2%	13%	13%
**Intermediate Outcome Indicators:**	Reduced crime					
	**Performance Targets**					
**Indicators**	**Base Year**	**Base Line**	**2023/24**	**2024/25**	**2025/26**	**2026/27**
Crime Rate	2018	551	400%	372	372	372
Proportion of reported cases taken to court	2018	28%	55%	55%	66%	66%
Detective case load	2018	1:42	1:29	1:25	1:25	1:25

GOVERNANCE AND SECURITY

P3: Medium Term Budget Allocations by Sub-Programme and Vote

Table P3.1: Proposed Budget Allocations and Medium Term Projections by Sub-Programme

Billion Uganda Shillings	FY2022/23	2023/24	Medium Term Projections			
	Approved Budget	Proposed Budget	2024/25	2025/26	2026/27	2027/28
01 Institutional Coordination	655.127	634.530	688.069	836.924	1,027.716	1,015.676
02 Security	4,915.241	4,661.554	4,550.268	5,268.702	6,412.266	6,426.894
03 Policy and Legislation Processes	491.565	478.504	490.783	591.569	808.934	810.714
04 Access to Justice	607.514	601.970	577.323	636.365	848.531	846.331
05 Anti-Corruption and Accountability	272.035	261.621	268.864	310.218	388.633	386.355
06 Democratic Processes	147.113	146.718	150.704	176.829	228.059	228.169
07 Refugee Protection & Migration Management	78.720	39.991	1.076	1.269	1.672	1.672
**Total for the Programme**	**7,167.315**	**6,824.887**	**6,727.087**	**7,821.876**	**9,715.811**	**9,715.811**

Table P3.2: Proposed Budget Allocations and Medium Term Projections by Vote

Billion Uganda Shillings	FY2022/23		2023/24	Medium Term Projections			
	Approved Budget	Spent by End Sep	Proposed Budget	2024/25	2025/26	2026/27	2027/28
001 Office of the President	195.511	**27.586**	189.566	193.669	229.745	304.189	304.189
002 State House	434.419	**109.238**	419.938	428.581	511.920	685.859	685.859
003 Office of the Prime Minister	78.720	**3.571**	39.991	1.076	1.269	1.672	1.672
004 Ministry of Defence	3,758.773	**934.993**	3,560.247	3,422.632	3,987.210	4,835.977	4,835.977
006 Ministry of Foreign Affairs	34.508	**2.302**	28.288	29.019	34.192	44.470	44.470
007 Ministry of Justice and Constitutional Affairs	148.630	**8.440**	150.108	153.001	182.461	244.489	244.489
008 Ministry of Finance, Planning and Economic Development	2.020	**0.210**	2.020	2.060	2.472	3.338	3.338
009 Ministry of Internal Affairs	60.543	**10.741**	59.896	61.107	73.071	98.119	98.119
011 Ministry of Local Government	0.754	**0.072**	0.754	0.777	0.903	1.138	1.138
018 Ministry of Gender, Labour and Social Development	4.140	**0.825**	4.140	4.226	5.060	6.802	6.802
021 Ministry of East African Community Affairs	37.285	**1.580**	38.185	38.961	46.619	62.645	62.645
102 Electoral Commission (EC)	146.297	**28.715**	146.297	150.279	176.379	227.459	227.459
103 Inspectorate of Government (IG)	71.819	**7.255**	71.819	73.539	86.190	111.612	111.612
105 Law Reform Commission (LRC)	18.150	**2.219**	18.450	18.933	22.292	28.943	28.943
106 Uganda Human Rights Commission (UHRC)	19.672	**2.458**	19.572	20.180	23.419	29.454	29.454
112 Directorate of Ethics and Integrity (DEI)	14.501	**0.651**	15.101	15.580	18.032	22.557	22.557

GOVERNANCE AND SECURITY

Billion Uganda Shillings	FY2022/23		2023/24	Medium Term Projections			
	Approved Budget	Spent by End Sep	Proposed Budget	2024/25	2025/26	2026/27	2027/28
119 Uganda Registration Services Bureau (URSB)	25.747	**3.529**	25.777	26.504	30.994	39.672	39.672
120 National Citizenship and Immigration Control (NCIC)	119.831	**30.237**	115.615	117.959	140.997	189.203	189.203
124 Equal Opportunities Commission	1.467	**0.323**	1.467	1.497	1.796	2.425	2.425
129 Financial Intelligence Authority (FIA)	26.651	**3.471**	27.251	28.070	32.676	41.386	41.386
131 Office of the Auditor General (OAG)	98.762	**18.869**	98.762	101.968	117.791	146.676	146.676
133 Directorate of Public Prosecution (DPP)	94.973	**8.997**	75.735	77.632	90.629	116.416	116.416
135 Directorate of Government Analytical Laboratory (DGAL)	36.466	**1.216**	33.386	34.061	40.525	54.028	54.028
137 National Identification and Registration Authority (NIRA)	60.886	**6.530**	59.466	61.205	71.311	90.578	90.578
144 Uganda Police Force	876.439	**159.402**	822.729	847.990	977.369	1,216.902	1,216.902
145 Uganda Prisons Service	308.720	**43.823**	308.720	317.236	370.642	474.335	474.335
153 Public Procurement & Disposal of Public Assets (PPDA)	21.802	**2.431**	21.805	22.512	25.858	31.907	31.907
158 Internal Security Organization (ISO)	156.052	**29.620**	148.938	153.277	178.423	226.364	226.364
159 External Security Organization (ESO)	72.215	**14.280**	72.879	74.866	87.916	113.458	113.458
311 Law Development Centre	28.730	**3.985**	28.594	29.301	34.324	44.346	44.346
501 Uganda Mission at the United Nations, New York	16.559	**4.140**	17.612	17.612	17.612	17.612	17.612
502 Uganda High Commission in the United Kingdom	4.011	**1.003**	6.124	6.124	6.124	6.124	6.124
503 Uganda High Commission in Canada, Ottawa	5.114	**1.084**	13.614	13.614	13.614	13.614	13.614
504 Uganda High Commission in India, New Delhi	2.583	**0.237**	2.853	2.853	2.853	2.853	2.853
505 Uganda High Commission in Kenya, Nairobi	13.693	**1.526**	10.393	10.393	10.393	10.393	10.393
506 Uganda High Commission in Tanzania, Dar es Salaam	8.749	**0.937**	9.177	9.177	9.177	9.177	9.177
507 Uganda High Commission in Nigeria, Abuja	5.910	**0.540**	18.922	18.922	18.922	18.922	18.922
508 Uganda High Commission in South Africa, Pretoria	3.079	**0.770**	3.469	3.469	3.469	3.469	3.469
509 Uganda High Commission in Rwanda, Kigali	2.937	**0.670**	3.361	3.361	3.361	3.361	3.361
510 Uganda Embassy in the United States, Washington	9.585	**2.311**	7.505	7.505	7.505	7.505	7.505
511 Uganda Embassy in Egypt, Cairo	2.822	**0.666**	2.992	2.992	2.992	2.992	2.992
512 Uganda Embassy in Ethiopia, Addis Ababa	2.819	**0.711**	2.569	2.569	2.569	2.569	2.569
513 Uganda Embassy in China, Beijing	4.023	**1.031**	4.081	4.081	4.081	4.081	4.081
514 Uganda Embassy in Switzerland, Geneva	6.501	**1.624**	6.601	6.601	6.601	6.601	6.601

GOVERNANCE AND SECURITY

Billion Uganda Shillings	FY2022/23		2023/24	Medium Term Projections			
	Approved Budget	Spent by End Sep	Proposed Budget	2024/25	2025/26	2026/27	2027/28
515 Uganda Embassy in Japan, Tokyo	5.179	**1.039**	5.179	5.179	5.179	5.179	5.179
516 Uganda Embassy in Saudi Arabia, Riyadh	6.041	**1.022**	5.441	5.441	5.441	5.441	5.441
517 Uganda Embassy in Denmark, Copenhagen	6.158	**1.344**	5.748	5.748	5.748	5.748	5.748
518 Uganda Embassy in Belgium, Brussels	4.169	**1.166**	4.549	4.549	4.549	4.549	4.549
519 Uganda Embassy in Italy, Rome	4.713	**0.849**	4.477	4.477	4.477	4.477	4.477
520 Uganda Embassy in DRC, Kinshasa	7.589	**0.949**	6.190	6.190	6.190	6.190	6.190
521 Uganda Embassy in Sudan, Khartoum	4.188	**1.582**	4.358	4.358	4.358	4.358	4.358
522 Uganda Embassy in France, Paris	19.533	**4.814**	6.458	6.458	6.458	6.458	6.458
523 Uganda Embassy in Germany, Berlin	7.571	**1.050**	6.601	6.601	6.601	6.601	6.601
524 Uganda Embassy in Iran, Tehran	2.758	**0.910**	2.758	2.758	2.758	2.758	2.758
525 Uganda Embassy in Russia, Moscow	3.550	**0.511**	3.722	3.722	3.722	3.722	3.722
526 Uganda Embassy in Australia, Canberra	10.163	**6.791**	4.113	4.113	4.113	4.113	4.113
527 Uganda Embassy in South Sudan, Juba	5.064	**1.369**	3.664	3.664	3.664	3.664	3.664
528 Uganda Embassy in United Arab Emirates, Abudhabi	4.665	**0.925**	4.315	4.315	4.315	4.315	4.315
529 Uganda Embassy in Burundi, Bujumbura	2.832	**0.583**	2.982	2.982	2.982	2.982	2.982
530 Uganda Consulate in China, Guangzhou	4.250	**0.846**	9.900	9.900	9.900	9.900	9.900
531 Uganda Embassy in Turkey, Ankara	4.418	**1.172**	4.808	4.808	4.808	4.808	4.808
532 Uganda Embassy in Somalia, Mogadishu	3.282	**0.792**	4.243	4.243	4.243	4.243	4.243
533 Uganda Embassy in Malaysia, Kuala Lumpur	3.378	**0.819**	3.378	3.378	3.378	3.378	3.378
534 Uganda Consulate in Kenya, Mombasa	6.306	**0.849**	4.706	4.706	4.706	4.706	4.706
535 Uganda Embassy in Algeria, Algiers	5.689	**0.897**	3.689	3.689	3.689	3.689	3.689
536 Uganda Embassy in Qatar, Doha	2.939	**0**	2.842	2.842	2.842	2.842	2.842
537 Uganda Mission in Havana, Cuba		**0**	3.000	3.000	3.000	3.000	3.000
538 Uganda Mission in Luanda, Angola		**0**	3.000	3.000	3.000	3.000	3.000
**Total for the Programme**	**7,167.315**	**1,515.097**	**6,824.887**	**6,727.087**	**7,821.876**	**9,715.811**	**9,715.811**

P4: PROGRAMME PRIORITIES IN LINE WITH INTERVENTIONS FOR FY2023/24

GOVERNANCE AND SECURITY

Programme Priorities FY2023/24	NDP III Programme Intervention
-Provide security at refugee entry points, reception centres, transit routes and camps<br>-Receive, document and vet refugees and asylum seekers<br>-Participate in the monitoring of refugee camps and report the same<br>-Acquisition of 13,000 Hectares of Kyangwali Refugee Settlement Land from Bunyoro Kitara Kingdom for hosting refugees and other programs of Government.<br>-Develop migration policy	Coordinating responses that address refugee protection and assistance
Support MoDVA production activities<br>Support UACC to achieve on its mandate<br>Food production to reduce tax payers burden of looking after offenders in custody	Engage in productive activities in line with strengthening capacity of Security Agencies to counter prevailing and emerging threats.
conduct home visits, prepare social inquiry reports<br>Establish, train and facilitate DCSC<br>Supervise offenders, monitor community service programs and conduct compliance checks<br>Train and sensitize stakeholders<br>Counsel and train/empower of offenders	Enhance implementation of community service as a sentence
Engage communities in community policing mechanisms<br>Deploy and train dog handlers as well as detectives<br>Promote patriotism amongst police officers	Enhance crime prevention and strengthen community policing

GOVERNANCE AND SECURITY

Programme Priorities FY2023/24	NDP III Programme Intervention
Increase personnel enrollment into welfare schemes<br>Acquire land for policing purposes<br>Construct and renovate staff houses for the police<br>Provide medical facilities (Police health facilities & Hospital), health personnel, medicines, specialized services etc<br>Provide education services to UPF staff 's children<br>Support welfare schemes<br>Expanding the Prisons Duty Free Shop, Prisons SACCO, funeral management scheme and staff spouses empowerment projects<br>Constructing and equipping the staff hospital<br>Construction of Jumbo Medical facility<br>Provide health services to prisons officers and their families.<br>Provide health services to security officers and their families.<br>Purchase medical equipment<br>Nutrition supplementation to all Prisons staff known to be living with HIV/AIDS per year<br>Provide Health Services to UPDF staff and their families and surrounding communities<br>Provide construction, non-construction and consumable items to UPDF soldiers<br>Accompany the president in the foreign visits to provide protocol services and offer foreign policy coherence services<br>provide protocol services at national functions, international conferences and summits<br>process authorisations for diplomats<br>Handle diplomatic requests<br>Coordinate presentations of letters of credence<br>Facilitate appointments for foreign dignitaries with HE. The president and other government officials sought	Enhance the welfare and housing of security sector personnel

GOVERNANCE AND SECURITY

Programme Priorities FY2023/24	NDP III Programme Intervention
Sensitise and create awareness among police officers on the Uganda Bill of Rights<br>Remodel police detention facilities to appropriate sanitation and hygiene<br>Enhance supervision of police detention facilities for compliance to 48hr rule<br>Hold dialogue meetings with MDAs with a view to reduce Human Rights cases.<br>Translate and disseminate the bill of rights in local languages<br>Conduct trainings<br>Framework for recognizing outstanding human rights personalities<br>Nomination and Assessment of personalities<br>Capacity building undertaken<br>Visits to detention facilities<br>Meetings to discuss recommendations<br>Visits to selected entities<br>conduct field compliance visits<br>Human rights desk in the Ministry in place and functional (preparing annual human rights reports, attending regional and international human rights assemblies, and addressing human rights issues in-country)<br>Defend human rights cases<br>Build the capacity of the Advisory Committee on the Prerogative of Mercy to carry out its mandate, obtain case information, conduct meetings, and advise the President.<br>Facilitating & Conducting Induction Training of all Groups of Visiting Justices<br>Monitoring court operations; Transporting prisoners to court and linking remand prisoners with other actors in the Criminal Justice System; Sureties, lawyers, benefactors, family, etc.<br>Installation of clean water systems (Gravity flow, boreholes, shallow wells and rain water harvesting systems) in prisons<br>Improve domestic and medical waste management in prisons<br>Acquire efficient and clean cooking systems in prisons Including LPG systems<br>Establish and equip human rights committees in all prisons and prisons regions<br>Conduct refresher training for 200 staff annually<br>Assorted DNA extraction equipment acquired to facilitate Collection and analysis of forensic evidence in Regional laboratories (DGAL) to improve access by vulnerable persons<br>Improve access to forensic evidence essential in SGBV cases for court users categorized as poor	Finalize and Implement the Uganda National Action Plan on Human Rights and adopt the National Action Plan on Business and Human Rights

GOVERNANCE AND SECURITY

Programme Priorities FY2023/24	NDP III Programme Intervention
Able bodied Ugandans recruited across the Country on District Quota system<br>Advanced training<br>Carry out refresher training of in-service immigration officers to adapt to changing dimension of migration management<br>Conduct refresher and specialized in-service training for police officers<br>Conduct regular recruitment to fill vacant positions in the organogram and training of staff<br>Conduct specialized training for career development (Criminal Law, Management, Patriotism, Fire Fighting, Mechanical, Defensive Driving, CT & use of IBIS etc)<br>Deploy Human and non-human resourses at Sub-county level Police Stations<br>Develop and strengthen cooperation frameworks in areas of Political, Peace and Security<br>Develop, coordinate, monitor and evaluate police operation doctrines and policies<br>Process submissions received from the Uganda Police Force<br>Process submissions received from the Uganda Prisons Service<br>Professionally manage public disorders, civil disturbances and riots<br>Promote citizens roles and responsibilities for maintenance of law and order<br>Provide security to Oil & Gas and Railway infrastructure<br>Provide strategic training for FIA staff to effectively and efficiently address emerging ML/TF/PF trends and techniques and methods<br>Provide training for KCCA law enforcement personnel.<br>Recruit and train more CID staff<br>Recruit, train ans deploy police personnel.<br>Recruitment and training of security personnel in basic advanced and specialized courses<br>Restructure and implement the new structure<br>Strengthen collaboration with state and non-state actors to promote observence of law and order<br>Strengthen UPF institutional capacity for human rights based approach to law and order enhancement<br>Strengthening the capacity of Prisons Safety and Security Unit<br>Training and re-training of personnel<br>Training of scientists in digital forensics to match emerging trends and sophisticated digital and fraud crimes<br>UPDF personnel trained for both combat and operations other than war.	Improve the capacity and capability of the Security Sector through training and equipping personnel.

GOVERNANCE AND SECURITY

Programme Priorities FY2023/24	NDP III Programme Intervention
Mainstream and implement Cross-cutting issues (Gender & equity,COVID-19, Heamophragic fever, HIV/AIds, Malaria, Environment and climate change, Population and urbanization, nutrition and sanitation. Human rights) in Planning and budgeting<br>Provision of 3 wholesome meals, beddings, uniform and other utilities to all prisoners<br>Strengthen mainstreaming and implementation of Cross-cutting issues (Gender, HIV, Environment and Human rights) in Planning and implementation<br>Coordinate activities of refugee management<br>Receive, document, settle, and protect refugees.<br>Participate in building capacity of MDAs & LGs to effectively formulate HR compliant policies and laws."	Integrate HRBA in policies, legislation, plans and programmes

GOVERNANCE AND SECURITY

Programme Priorities FY2023/24	NDP III Programme Intervention
Provide child friendly services at police stations<br>Build and improve the capacity of UPF Child and Family protection services<br>Establish Child reception centres at UPF police stations<br>Enhance Gender & Equity friendly services at police units<br>Legal aid clinics to be established and mainatained in Lira, Mbale, Kamuli, Masindi, Arua, Hoima<br>Supervise Legal Aid Service providers<br>Fast track hearing of complaints involving children<br>Resolve family disputes through mediation and arbitrations<br>Prosecute cases in special child/juvenile court sessions<br>Train prosecutors in child friendly procedures<br>Distributing child victim emergency kits.<br>Establish& equip child friendly rooms in all ODPP regional offices<br>Prosecute cases in special child/juvenile court sessions<br>Train prosecutors in child friendly procedures<br>Distributing child victim emergency kits.<br>Establish& equip child friendly rooms in all ODPP regional offices<br>Conduct EOC Pre-Tribunal sessions<br>Conduct Mobile Legal Aid Clinics in the four regions of the country<br>Hold disciplinary committee sittings to handle complaints against Advocates<br>Review and assessment of bills, laws for compliance with Equal Opportunities<br>Conduct mobile complaints handling clinics<br>Strengthen follow-up mechanisms of complaints referred<br>Fully investigate human rights cases<br>Perusal and making prosecutorial decisions, stakeholder engagements, prosecution-led-investigations<br>Research, pre-trial case management meetings, pre-trial disclosures, drafting and filling court documents, interfacing with witnesses<br>Conduct hand-on training of prosecutors and investigators, equip officers with laws guidelines manuals and other reference material, hold stakeholder engagements, outreach and public awareness	Promote equitable access to justice through legal aid services

GOVERNANCE AND SECURITY

Programme Priorities FY2023/24	NDP III Programme Intervention
Weed out backlog cases in the police system<br>Procure and install video conferencing facilities in 50 prisons to facilitate access to justice<br>Attend scheduled Court and effectively represent Government and Allied Institutions<br>Payment of Court Award arrears<br>Payment of war debt and other claimants and other compensations<br>Establish and regularly update a list of Court Awards against each MDA<br>Develop Application Programming Interfaces for integration of eservices across government<br>Digitize manual files and integrate electronic document management system in operations<br>Integrate information Systems to achieve the once only principle for inter institutional sharing of information<br>Develop Integrated Management System with five modules<br>Implement e-library and automated services, to strengthen access to legal reference materials<br>Equip the MOJCA Records and Registry Unit with High capacity equipment including scanners, photocopiers, digitization machine.<br>Procure systems and hardware for the DCIC Command Center<br>Equip duty bearers for enhanced accomplishment of their roles<br>Engage and sensitize all duty bearers and stakeholders for marriage registration<br>Rollout the National Marriage Registration System<br>Conduct the survey on licensing of churches, marriage registration and filing of marriage returns<br>Automate all Registration services and integrate with relevant Agencies<br>Establish a Non Individual Entities Register<br>Establish and operationalize pending Registers<br>Establish a Divorce Register<br>Establish a Geographical Indications Register<br>Strengthen Compliance and Enforcement<br>Implement Data clean up and update of business and civil registers project<br>Implement Data conversion of administrative support units and directorate records project	Re-engineer business processes to reduce red tape in service delivery especially regarding commercial and land dispute resolution

GOVERNANCE AND SECURITY

Programme Priorities FY2023/24	NDP III Programme Intervention
Undertake research on the development process of regulation of legal profession and understanding unmet need in the Legal Aid service provision<br>-Quality assurance of submissions to Cabinet for Compliance with RBP/RIA<br>Capacity building for staff of D PD&CB in Policy Management<br>Capacity building for Policy Analyst Cadre and stakeholders in M&E and preparation of policy briefs<br>Provision of guidance on policy development to MDAs<br>Review Bills from MDAs and place them on the Agenda of Cabinet<br>Review Policy submissions from MDAs and place them on the Agenda of Cabinet	Review, and develop appropriate policies for effective governance and security
Expand the National CCTV surveillance project to subsidiary cities, municipalities, major towns, black spots and highways<br>Maintenance costs for the national CCTV project	Rollout CCTV surveillance project
Prepare and support police officers for life in retirement.<br>Establish and operationalize district retired police officers coordination offices<br>Establish and ensure accessibility of welfare schemes to registered retired police officers<br>Mobilize and prepare for retirement<br>Empower security retirees with vocational skills for self-reliance<br>Empower Military Veterans with revolving capital, startup kit and skills for self-reliance to increase income at household levels.<br>Clear Pensions, gratuity and survivors benefits backlog	Seamlessly transition, resettle and reintegrate veterans into productive civilian livelihoods

GOVERNANCE AND SECURITY

Programme Priorities FY2023/24	NDP III Programme Intervention
Set up police establishments at border points.<br>Extend the i24/7 System to border points<br>Coverage of Border points<br>Deploy officers at border points<br>"1) Host and/participate in summit & ministerial meetings<br>2) Host and/participate in command post exercises & training exercises for peace & security<br>3) Participate in border community peace & security cluster meetings<br>4) Participate in policy organs<br>"<br>Coordinate with other security agencies at border points to enforce declaration of cash and bearer negotiable instruments<br>Coordinate cross border meetings with neighbouring countries<br>Coordinate inspection of border points by all stakeholders<br>Coordinate the demacation of Uganda's border points<br>Hold cross border meetings and Joint Commissioners meetings<br>Equip all 53 border posts with e-immigation system to facilitate full automatio and enhanced traveller clearance and verifications procurement of e-consumables , AFIS licenses, operation and maintenance service<br>Establish fully functional document laboratories at selected border posts<br>Manage immigration custody facilities<br>Provide legal advisory on immigration services<br>Carry out enforcements and investigations for compliance<br>Conduct prosecution of offenders of immigration laws, policies and regulations<br>Equip 19 missions abroad with e-immigration system for personalisation of immigration facilities<br>Conduct regular patrols at all gazetted border posts and illegal entry routes<br>Replace old personalisation equipment for e-immigration system participate in cross boarder crime prevention meetings	Strengthen border control and security

GOVERNANCE AND SECURITY

Programme Priorities FY2023/24	NDP III Programme Intervention
Acquire and maintain equipment for policing.<br>Equip UPF Regional Forensic Referral Centre (RFRC) of excellence<br>Develop and equip police training schools and college<br>Recruit, train & Equip Police Crime Intelligence personnel<br>Provide logistics to UPF personnel<br>Purchase of specialized modern equipment and classified assets<br>Acquire modern equipment and reengineer systems and business processes to increase efficiency and effectiveness in detection and prevention of financial crimes<br>Acquire transport equipment to support operations o FIA<br>Acquire Logistical and technical equipment (communication, transport, specialized, training, medical and strategic facilities)<br>Acquisition of modern cyber scientific equipment for DGAL.<br>Acquisition of military equipment in sync with technological advancement<br>Acquire equipment to enhance combat service support<br>Establish and maintain foreign and strategic stations.<br>Purchase transport equipment.<br>Construct and fully equip Katonga International Technical Communication center<br>Construct and fully equip ESO Headquarters<br>Construct, equip and operationalise the Car tracking facility<br>Provision of working uniform and accessories to all Prisons staff<br>Enhancement of Early warning and response mechanisms country wide<br>Provide well balanced feeding diet to security personnel<br>Provide fuel for transportation of troops, equipment and training of personnel<br>Fully dress all UPDF personnel<br>Construct and equip the ISS - U<br>Operationalise ISS-U<br>Construct and equip a National Intelligence Academy<br>Construct the Strategic Headquarters	Strengthen capacity and handle emerging and prevailing sophisticated crimes such as cyber-crimes
Integrate the Birth/Death and Identification registration systems<br>Decentralise the registration services to subnational levels<br>Review the NIRA structure and populate it to subnational levels<br>Certifying and issuing of birth certificates<br>Strengthen partnerships with other MDAs i.e. MOH and MOLG<br>Process, verify and grant citizenship for eligible persons	Strengthen citizenship identification, registration, preservation and control

GOVERNANCE AND SECURITY

Programme Priorities FY2023/24	NDP III Programme Intervention
Establish and operationalize situation rooms (Acquiring equipment, recruiting data analysts, preparing conflict early warning reports)<br>Establish, train, and revive committees<br>Restructure of the CEWER unit, recruit staff and equip<br>Conducting radio/tv talk shows, print media, awareness weeks & sharing reports<br>Establish and operationalize modern situation rooms and centers<br>Hold monthly security meetings<br>Sensitize local communities<br>Coordinate security agencies<br>coordinate the reduction of Cross boarder Crimes	Strengthen conflict early warning and response mechanisms
Invest in counter terrorism measures (sensitization, training, equipment, intelligence)<br>Meetings, training, operations, Reports.<br>Meetings, trainings, Reports.<br>Equip ODPP with skills in handling terrorism crimes<br>Equip ODPP with tools to handle terrorism crime cases<br>Conduct security assesements<br>Conduct security inspections<br>Establish and sustain a coorinating mechanism for PCVE<br>Implement robust systems to identify and prevent financing of terrorism in our economy<br>Participate in Intra EAC COUNTER terrorism Alerts	Strengthen counter terrorism

GOVERNANCE AND SECURITY

Programme Priorities FY2023/24	NDP III Programme Intervention
Provide training to security personnel deploy on election day<br>Ensure law and order before, during and after elections<br>Conduct voter Education consultations and outreaches through institutions, organizations, national events, roadshows, mobile vans,<br>Conduct sensitization through electronic media and billboards,<br>Commission hour on regional televisions<br>Conduct Voter education radio and TV talk shows<br>Produce and disseminate voter education IEC materials, popular celebrities,<br>Conduct sensitization workshops<br>Sensitization through electronic media, road shows, drama competitions and institutions<br>Carry out nomination of Candidates<br>Supervise and coordinate campaign programmes<br>Carry out training of election officials (update, display, polling, tallying)<br>Procure training materials<br>Procure Generic Polling materials<br>Disseminate Election results<br>Gazette Election events, date and results<br>respond to and handling election petitions<br>Organise and conduct by-elections as and when they occur<br>Timely delivery and retrieval of of polling materials and services and payment of polling official and polling day costs<br>Monitoring, review and evaluate every stage of the electoral process	Strengthen democracy and electoral processes
Identify, register and assign NINs to all citizens into the National Identification Register<br>Decentralize registration services to sub-national levels.<br>Registration of legally resident aliens<br>Mass renewal of all first generation IDs<br>Carry on the Tax Payer Registration Expansion Project (TREP), collection of 3rd party information, Standardization of data, Single National Identifier<br>Stock passport booklets and accessories<br>extrend e-passport enrollment centres to regional offices and missions abroad<br>Maintain the e-immigration system and stock immigration saleable documents<br>Provide the required immigration services to diaspora individuals<br>Provide immigration services to diaspora community<br>Provide conventional travel documents to resident refugees<br>Enhance coverage for refugee Camps, Immigration, registration services and persons	Strengthen identification and registration of persons' services

GOVERNANCE AND SECURITY

Programme Priorities FY2023/24	NDP III Programme Intervention
Train & Equip police officers on management of explosives to avert terrorism activities<br>procure license books, carry out due diligence on applicants for permits and licenses	Strengthen management of commercial explosives
Enhance UPF Capacity to effectively detect and conclusively investigate crime<br>Build UPF crime fighting capacity<br>strengthen Use of scientific evidence(forensic) in crime management<br>Improve coordination in response to crime by crime fighting agencies<br>Induct 4,428 personnel into CID to reduce Case load per detective<br>Expand Coverage and range of canine services<br>Conduct ambushes, snap check operations, strategic visibility deployments and patrols for recovery of suspected stolen animals.<br>New detaches, foot and motorized patrols in the ASTU deployments conducted to enhance strategic visibility;<br>Promote application of science in crime investigation<br>Timely analysis of received forensic cases<br>Acquire Critical modern scientific Machinery & Equipment<br>Calibrate and maintain Scientific Equipment<br>Acquire Reagents, Chemicals & Consumables for forensic case analysis.<br>Train Scene of Crime Officers SOCOs on crime scene evidence recovery & preservation<br>Fast track Implementation of Laboratory Quality management systems<br>04 Regional forensic laboratories operationalized in a forensic manner<br>Develop comprehensive standards; Development of minimum performance service delivery standards (DGAL)<br>Develop occupational health and safety SOPs for protection forensic scientists against health hazards in the workplace (DGAL)<br>Acquire protective gears and Equipment against epidemics (DGAL)	Strengthen response to crime

GOVERNANCE AND SECURITY

Programme Priorities FY2023/24	NDP III Programme Intervention
Enhance crime intelligence services<br>Meetings, consultation<br>Management of formal education and FAL programs<br>Training of offenders in Industrial skills<br>Infrastructure for offender vocational skills training in Agriculture<br>Conduct investigations, carryout research, hold stakeholder meetings, preparation and transmission of requests and responses. Service of international Court documents<br>Carryout research, investigations, prosecution and repatriation of fugitives<br>Lead investigations, perusal, case management meetings, stakeholder engagement meetings, drafting court documents, disclose and service.<br>Addressing public complaints on prosecution processes<br>Support legal reviews and a national Policy on Business formalization	Strengthen transitional justice and informal justice processes

P5: GENDER AND EQUITY ISSUES AND INTERVENTIONS FOR FY2023/24

Gender and equity are very critical in service delivery. The Governance and Security Program has prioritized various interventions to ensure equitable access to services. In FY2023/24, the program will undertake following interventions to further enhance gender and equity:

i. Ensure affirmative action in promotion, recruitment, training and deployment as well as protect staff to carry out their duties without fear and prejudice;

ii. Enhance production for wealth creation and self-sustainability to create income generating activities for wives and spouses of UPDF, UPF and UPS and female combatants, such as soap making, tailoring projects, poultry and bee keeping;

iii. Reconfiguration of institutional infrastructure to accommodate gender and equity requirements such as ramps, special toilets, reserved parking areas for access and use to persons with disabilities;

iv. Provide psycho-social support to UPDF, UPF, UPS spouses and their children and victims in human trafficking;

v. Provide Legal Aid Services to the vulnerable groups including PWDs, women, indigent veterans, refugees among others;

vi. Registration of persons including male, females, PWDs, youths and children for inclusion into the National Identification Register;

vii. Provision of prosthesis like artificial limbs, orthopedic shoes, wheelchairs, elbow crutches, hearing aids and other assistive devices e.g. Mubende Rehabilitation Centre (MRC);

viii. Conduct vocational skilling for persons living with disabilities for example special needs training;

ix. Detection, interception and prevention of Trafficking in Persons;

x. Create more projects for demonstration and training for security personnel veterans and persons living with disabilities such as rearing animals, gardening, and quality metal products among others;

xi. Enhance Rehabilitation and Psych-social centers for security veterans with disabilities;

xii. Conduct outreaches to deliver registration services in hard to reach areas and prisons and

xiii. Ensure gender documentation, reporting and monitoring;

REGIONAL BALANCED DEVELOPMENT

Foreword

The Rt. Honourable Speaker of Parliament,
The Regional Development Programme (RDP) whose goal is to accelerate equitable, regional economic growth and development is an affirmative action on the eight (8) poorest sub regions of Karamoja, Teso, Bukedi, Bugisu, Busoga, Acholi, Bunyoro and West Nile based on the Uganda National Household Survey (UNHS) 2016/17. In spite of the challenges occasioned by the COVID-19 pandemic, the UNHS 2019/20 estimates that the percentage of the population living below the official national poverty negligibly reduced from 21.4 percent in 2016/17 to 20.3 percent in 2019/20 with poverty remaining high in the subject sub regions.

Madam speaker, as you are aware, the programmatic approach of planning and budgeting commenced with the implementation of the National Development Plan III. In FY 2021/22, the RDP realized the following key achievements: An industrial hub was constructed in Soroti DLG, value addition equipment was provided to RDP beneficiary LGs as follows 7 maize milling, 12 Milk coolers were also provided in Rwenzori Sub region under Agri-LED, 541 leaders were inducted in the LGs of Arua, Gulu, Soroti, Moroto and Kamuli, 5,220 parishes are currently connected to motorable community access roads and more roads will be connected with the implementation of NOSP and Rural Development and food security in Northern Uganda and 3 irrigation schemes have been rehabilitated in Kumi (Kajamaka Irrigation Scheme), Kibuku (Kapyani Rice Irrigation Scheme) and Katakwi (Agule Irrigation Scheme).

Madam Speaker, in FY 2023/24, the Programme will prioritize 6 priority areas of Extend power transmission lines to key growth opportunity areas of the sub-regions; Develop and implement evidence based Regional Development Plans, Construction of roads leading to key growth opportunity areas, particularly tourism sites, Design and construct infrastructure facilities (Roads, Hydropower, solid waste management/water waste system, ICT/CCTV, service ducts, etc), Distribute quality inputs, livestock, storage facilities and processing equipment's to farmer groups, and Procurement and distribution of tractors, ox-ploughs, identification and training of beneficiary cooperatives, monitoring of beneficiary cooperatives.

Madam Speaker, as with any other wide-ranging initiatives of this magnitude, implementation of the RDP in FY 2021/22 was faced with a number of challenges such as drought, inadequate financing of the MoLG Budget, staffing gaps/ recruitment challenges by the LGs, and coordination challenges by the Secretariat.
I now have the honor to present the FY 2023/24 Budget Framework Paper for the Regional Development Programme and I pray for your support to help the Ministry together with the other contributing MDAs to successfully implement this programme for the achievement of balanced development of our country.

For God and My Country

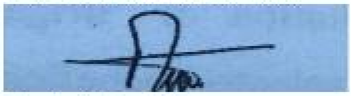

Raphael Magyezi

Hon. Minister of Local Government

REGIONAL BALANCED DEVELOPMENT

Abbrevations and Acronyms

ACRONYM	ACRONYM NAME
CAR	Community Access Road
DEC	District Executive Committee
FYs	Financial Years
IGFTR	Intergovernmental Fiscal Transfer Reform
LGMSD	Local Government Management Service Delivery Program
LLGs	Lower Local Governments
OTIMS	Online Transfer Information Management System.
PCR	Project Completion Report
PIP	Performance Improvement Plan
RGCs	Rural Growth Centres
SAS	Senior Assistant Secretary
TPC	Technical Planning Committee

REGIONAL BALANCED DEVELOPMENT

P1: PROGRAMME OVERVIEW

Snapshot of Medium Term Budget Allocations

Table P1.1 Overview of Programme Expenditure and Medium Term Allocations (Ush Billion)

Billion Uganda Shillings		FY2022/23		FY2023/24	MTEF Budget Projections			
		Approved Budget	Spent by End Sep	Proposed Budget	2024/25	2025/26	2026/27	2027/28
Recurrent	Wage	423.158	105.756	423.158	444.316	488.747	537.622	537.622
	Non Wage	547.342	125.435	145.100	148.002	177.602	239.763	239.763
Devt.	GoU	115.295	24.192	13.900	13.900	16.680	23.352	23.352
	ExtFin	71.685	10.790	52.524	9.141	0.000	0.000	0.000
	**GoU Total**	**1,085.796**	**255.384**	**582.158**	**606.218**	**683.030**	**800.737**	**800.737**
	**Total GoU+Ext Fin (MTEF)**	**1,157.481**	**266.174**	**634.682**	**615.359**	**683.030**	**800.737**	**800.737**
	A.I.A	238.500	0.000	238.000	238.000	238.000	238.000	238.000
	**Grand Total**	**1,395.980**	**266.174**	**872.682**	**853.359**	**921.030**	**1,038.737**	**1,038.737**

Programme Strategy and linkage to the National Development Plan

Regional Development Programme aims to accelerate equitable regional economic growth and development. The key results to be achieved over the next five years are to reduce poverty in the lagging sub-regions lagging behind the national poverty line of Karamoja, Bukedi, Bugisu, Busoga, West Nile, Acholi, Teso and Bunyoro.

This program contributes to four NDPIII objectives, which are

i. Enhance value addition in key growth opportunities ii. Consolidate and increase the stock and quality of productive infrastructure iii. Enhance the productivity and social wellbeing of the population iv. Strengthen the role of the state in guiding and facilitating development.

The goal of the regional development programme is to accelerate equitable, regional economic growth and development and in so doing, the Program is poised to contribute to the goal of the Third National Development Plan to increase average household income and improve the quality of lives of Ugandans.

The key results to be achieved over the next five years are to reduce poverty in the lagging regions.

This programme focuses on accelerating poverty reduction in the regions that are lagging behind the national poverty line or are facing poverty reversal by, providing affirmative actions.

The objectives of this programme are to

a. Stimulate the growth potential of the sub-regions in the key growth opportunities (Agri-business, Tourism, Minerals and Manufacturing);

b. Close regional infrastructure gaps for exploitation of local economic potential;

c. Strengthen and develop regional based value chains for LED;

d. Strengthen the performance measurement and management frameworks for local leadership and public sector management.

The expected key results are

a. Increased production capacity of key growth opportunities (Agri-business, Tourism, Minerals and Manufacturing)

b. Increased household earnings in the sub-regions from ATM

c. Increased market access and value addition

d. Enhanced agro-LED business

e. Improved leadership capacity for transformative rural development

REGIONAL BALANCED DEVELOPMENT

P2: Highlights Of Programme Projected Performance

Table P2.1 Programme Outcomes Indicators

**Programme Outcome**	Increased market access and value addition					
**Programme Objectives contributed to by the Intermediate Outcome**<br>Close regional infrastructure gaps for exploitation of local economic potential						
	**Performance Targets**					
**Programme Outcome Indicators**	**Base Year**	**Base Line**	**2023/24**	**2024/25**	**2025/26**	**2026/27**
Agricultural households accessing local produce markets	2021/22	51	61%	65	70	75
Coverage of the District road network, %	2015	53.1%	75%	78%	82%	85%
**Programme Outcome**	Increased production capacity of key growth opportunities					
**Programme Objectives contributed to by the Intermediate Outcome**<br>Stimulate the growth potential of the sub-regions through area-based agribusiness LED initiatives						
	**Performance Targets**					
**Programme Outcome Indicators**	**Base Year**	**Base Line**	**2023/24**	**2024/25**	**2025/26**	**2026/27**
Average farm size for selected enterprises (ha)	2016/17	2.5	2%	2.5	3	3.5
Average monthly household income (UGX)	2016/17	266250	300000	350000	400000	450000
Irrigated agriculture land as a total cultivated land (%).	2016/17	1.3	3.5%	4	4.5	5
Percentage of households involved in commercial scale agriculture	2016/17	30	35%	40	45	50
Proportion of smallholder farmers accessing credits (%)	2016/17	7	15%	20	24	29
Regional poverty rates - 1. Bukedi	2019/20	34.7%	32.9%	31%	29%	25%
Regional poverty rates - 2. Busoga	2019/20	29.4	26.4	27	23	20
Regional poverty rates - 3. Bugisu	2019/20	13.2	12.6	12.1	11.5	10.6
Regional poverty rates - 4. West Nile	2019/20	16.9	16.5	15.8	14.1	11.1
Regional poverty rates - 5. Karamoja	2019/20	65.7	60.1	55.4	50.7	46
Regional poverty rates - 6. Acholi	2019/20	67.7	65	55.8	51.2	48
Regional poverty rates - 7. Teso	2019/20	21.9	20.4	19.3	18.5	17.6
Regional poverty rates - 8. Bunyoro	2019/20	9.8	9.1	8.4	7.7	7
Registered customary tenure land (%)	2016/17	20	35%	40	45	50
Share of private sector credit for the selected agricultural enterprises (%)	2016/17	17	25%	28	35	40

REGIONAL BALANCED DEVELOPMENT

**Programme Outcome**	Increased household earnings in the sub-regions from Agri-business, Tourism, Minerals (ATM)					
**Programme Objectives contributed to by the Intermediate Outcome**<br>Stimulate the growth potential of the sub-regions through area-based agribusiness LED initiatives						
	**Performance Targets**					
**Programme Outcome Indicators**	**Base Year**	**Base Line**	**2023/24**	**2024/25**	**2025/26**	**2026/27**
Proportion of farmers whose agricultural income has increased, %	2016/17	25	30%	35	40	45
Share of earnings from mining in household income	2016/17	0	2%	3	5	7
Share of tourism earnings in household income	2016/17	0	2%	3	5	7
**Programme Outcome**	Enhanced agro-LED business					
**Programme Objectives contributed to by the Intermediate Outcome**<br>Strengthen and develop regional based value chains for LED;						
	**Performance Targets**					
**Programme Outcome Indicators**	**Base Year**	**Base Line**	**2023/24**	**2024/25**	**2025/26**	**2026/27**
% increase in number of commercial farmers	2016/17	2.5	3.5%	4	5	6
% increase in number of Agro – LED Enterprises	2016/17	0	20%	30	30	40
Proportion of processed agricultural outputs, %	2016/17	25	30%	35	40	45
**Programme Outcome**	Improved leadership capacity for transformative rural development					
**Programme Objectives contributed to by the Intermediate Outcome**<br>Strengthen the performance measurement and management frameworks for local leadership and public sector management.						
	**Performance Targets**					
**Programme Outcome Indicators**	**Base Year**	**Base Line**	**2023/24**	**2024/25**	**2025/26**	**2026/27**
% increase in Community Participation in the planning process	2016/17	85	90%	92	95	97
% of LGs with Unqualified Annual Audit Reports from the OAG	2016/17	95%	97%	98%		99%
Average score of LGs in service delivery performance	2016/17	0	71	75	80	84

Table P2.2: Intermediate Outcomes Indicators

**Sub-Programme Name:**	**Production and productivity**					
**Intermediate Outcome Indicators:**	Increased acreage of agricultural land					
	**Performance Targets**					
**Indicators**	**Base Year**	**Base Line**	**2023/24**	**2024/25**	**2025/26**	**2026/27**
% of households accessing valley dams in the targeted regions	2016/17	6	5%	7	8	9
Irrigated agriculture as a total cultivated land (%).	2016/17	3.9	3.9%	4.1	4.6	4.9

REGIONAL BALANCED DEVELOPMENT

Sub-Programme Name:	Production and productivity					
Intermediate Outcome Indicators:	Increased household earnings from agriculture					
	Performance Targets					
Indicators	Base Year	Base Line	2023/24	2024/25	2025/26	2026/27
Proportion of household incomes from Agriculture in the targeted regions	2016/17	43	53%	60%	65%	70%
Intermediate Outcome Indicators:	Increase production & productivity of the sub region in the Key Growth Opportunities for LED					
	Performance Targets					
Indicators	Base Year	Base Line	2023/24	2024/25	2025/26	2026/27
Proportion of house hold incomes from tourism in the targeted regions	2016/17	0	15%	20%	25%	30%
Sub-Programme Name:	Infrastructure Development					
Intermediate Outcome Indicators:	Increased Market Share of the Key Enterprises in the regions					
	Performance Targets					
Indicators	Base Year	Base Line	2023/24	2024/25	2025/26	2026/27
% Average increase in motorable network of community access roads in the targeted regions	2016/17	0	1%	1%	1%	1%
% Increase in access to electricity by the population in the targeted regions	2016/17	0	5%	5%	5%	5%
% increase in motorable network in the targeted regions	2016/17	0	1%	1%	1%	1%
% increase in the mobile wireless internet subscription	2016/17	1%	1.5%	2%	2.5%	3%
Sub-Programme Name:	Capacity Building of Leaders					
Intermediate Outcome Indicators:	Improved delivery of Services by LG and Community structures within the Regions					
	Performance Targets					
Indicators	Base Year	Base Line	2023/24	2024/25	2025/26	2026/27
% of community leaders participating in Accountability Platforms in the targeted regions	2016/17	0	70%	73	75	79
% of LGs in the targeted regions meeting the minimum performance measures	2016/17	0	70%	73	75	79

P3: Medium Term Budget Allocations by Sub-Programme and Vote

Table P3.1: Proposed Budget Allocations and Medium Term Projections by Sub-Programme

Billion Uganda Shillings	FY2022/23	2023/24	Medium Term Projections			
	Approved Budget	Proposed Budget	2024/25	2025/26	2026/27	2027/28
01 Production and productivity	1,325.908	599.877	588.895	651.399	757.431	757.431
02 Infrastructure Development	67.400	26.101	3.612	6.734	10.301	9.301
03 Capacity Building of Leaders	2.673	8.704	22.851	24.897	33.005	34.005

REGIONAL BALANCED DEVELOPMENT

Billion Uganda Shillings	FY2022/23	2023/24	Medium Term Projections			
	Approved Budget	Proposed Budget	2024/25	2025/26	2026/27	2027/28
Total for the Programme	1,395.980	634.682	615.359	683.030	800.737	800.737

Table P3.2: Proposed Budget Allocations and Medium Term Projections by Vote

Billion Uganda Shillings	FY2022/23		2023/24	Medium Term Projections			
	Approved Budget	Spent by End Sep	Proposed Budget	2024/25	2025/26	2026/27	2027/28
003 Office of the Prime Minister	72.302	3.468	3.047	3.068	3.645	4.971	4.971
010 Ministry of Agriculture, Animal Industry and Fisheries		0	0.300	0.306	0.367	0.496	0.496
011 Ministry of Local Government	70.273	9.139	79.071	35.992	32.217	44.163	44.163
015 Ministry of Trade, Industry and Co-operatives		0	0.100	0.102	0.122	0.165	0.165
016 Ministry of Works and Transport		0	0.400	0.408	0.490	0.661	0.661
017 Ministry of Energy and Mineral Development		0	0.300	0.306	0.367	0.496	0.496
020 Ministry of ICT and National Guidance		0	0.200	0.204	0.245	0.330	0.330
022 Ministry of Tourism, Wildlife and Antiquities		0	0.100	0.102	0.122	0.165	0.165
108 National Planning Authority (NPA)	0.635	0	0.200	0.204	0.245	0.330	0.330
142 National Agricultural Research Organization (NARO)		0	0.100	0.102	0.122	0.165	0.165
147 Local Government Finance Commission (LGFC)		0	0.100	0.102	0.122	0.165	0.165
617 Local Governments 17	1,014.271	253.567	550.764	574.462	644.965	748.629	748.629
Total for the Programme	1,157.481	266.174	634.682	615.359	683.030	800.737	800.737

P4: PROGRAMME PRIORITIES IN LINE WITH INTERVENTIONS FOR FY2023/24

Programme Priorities FY2023/24	NDP III Programme Intervention
Develop and implement evidence based Regional Development Plans	Develop and implement regional specific development plans
Distribute quality inputs livestock storage facilities and processing equipments to farmer groups	Establish post-harvest handling, storage and processing infrastructure including silos, dryers, warehouses, cold rooms and a warehouse receipt system for farmers in those regions
Design and construct infrastructure facilities (Roads, Hydropower, solid waste management/water waste system, ICT/CCTV, service ducts, etc)	Increase ICT interconnectivity in these programme regions
Construction of roads leading to key growth opportunity areas, particularly tourism sites	Increase transport interconnectivity in these programme regions to promote intra-regional trade and reduce poverty

REGIONAL BALANCED DEVELOPMENT

Programme Priorities FY2023/24	NDP III Programme Intervention
Extend power transmission lines to key growth opportunity areas of the sub-regions	Operationalize the Industrial and Business Parks situated in the target regions
Procurement and distribution of tractors, ox-ploughs, identification and training of beneficiary cooperatives, monitoring of beneficiary cooperatives	Organize farmers into cooperatives at district level

P5: GENDER AND EQUITY ISSUES AND INTERVENTIONS FOR FY2023/24

A) Supporting and conducting compliance inspections in all the Local Governments especially those in the hard to reach areas such as Karamoja due to insecurity. Special joint compliance inspections will be made to the hard to reach Local Governments.
B) Reliance on donor support for implementation of interventions related to combating gender-based violence an issue that affects the self-esteem of the affected. Deliberate efforts will be made by budgeting for interventions aimed at combatting gender-based violence.
C) Limited involvement of women in leadership especially on committees like School Management Committees, Health Unit Management Committees. Sensitization exercises will be conducted to create opportunities for women on the various committees.
D) Special interest groups lack capacity in representation of their interest, even where there are leaders representing the groups, facilities that do not cater for their needs get approvals. The Programme Secretariat will build the capacity of the various special interest groups with support from the different stakeholders.
E) Inadequate support in mainstreaming Gender and Equity issues in the various programme member interventions. The Programme Secretariat will support the various programme members to help ensure Gender and Equity budgeting.

DEVELOPMENT PLAN IMPLEMENTATION

Foreword

The goal of the DPI Programme is to improve efficiency in the implementation of the NDP III and to make the plan (NDP III) more effective in achieving its goal. There are two aspects to this goal; on the one hand, taking lessons from the NDP II, it requires us to strengthen coordination and oversight over the NDP III. On the other hand, to improve financial management systems in support of the NDP III implementation. This makes DPI unique as its responsibilities extend across to all other NDP III programmes as well.

This Programme BFP provides an implementation framework for prioritising and sequencing key interventions that are crucial for delivering the Programme objectives, by allocating financing to the Programme priorities for the FY2023/24 and making projections for FY2024/25 to 2025/26. It is derived from the budget framework papers of the votes contributing to the DPI programme and is aligned to the reprioritised Programme Implementation Action Plan (PIAP). This BFP shall provide the basic guidelines for preparing annual Vote budgets for the FY2023/24 for all contributing votes – in relation to the DPI programmes.

Therefore, completeness, inclusion and accuracy have been key principles in the development of this PBFP.
Past performance of the DPI Programme and the economy as a whole has greatly suffered setbacks from global economic shocks like COVID 19 and the Russia Ukraine war. These continue to be a major concern going forward because of their direct impact on DPI targets such as GDP growth and revenue to DGP performance.

Our strategy in this BPF is to give priority to Strengthening budgeting and resource mobilization which is the anchor for NDP III success and to interventions most relevant to economic recovery and to the implementation of the Parish Development Model (PDM). PDM is a key Government intervention designed to improve planning and services delivery at the lowest levels of society and has the potential to greatly impact the NDP III's goal of "Increased Household Incomes and Improved Quality of Life of Ugandans".

Additionally, we will strengthen capacity for coordination, oversight, monitoring and reporting by strengthening the APEX Platform – a high level Executive Organ to strengthen the oversight roles of the Presidency.

Ramathan Ggoobi

Permanent Secretary/Secretary to the Treasury

DEVELOPMENT PLAN IMPLEMENTATION

P1: PROGRAMME OVERVIEW

Snapshot of Medium Term Budget Allocations

Table P1.1 Overview of Programme Expenditure and Medium Term Allocations (Ush Billion)

Billion Uganda Shillings		FY2022/23		FY2023/24	MTEF Budget Projections			
		Approved Budget	Spent by End Sep	Proposed Budget	2024/25	2025/26	2026/27	2027/28
Recurrent	Wage	257.622	56.878	259.422	272.394	299.633	329.596	329.596
	Non Wage	15,771.989	6,154.205	18,365.695	20,469.641	20,405.604	24,969.168	25,037.381
Devt.	GoU	240.970	8.617	256.607	256.607	307.928	431.100	431.100
	ExtFin	27.965	0.593	7.787	0.000	0.000	0.000	0.000
	**GoU Total**	**16,270.582**	**6,219.700**	**18,881.724**	**20,998.642**	**21,013.165**	**25,729.864**	**25,798.077**
**Total GoU+Ext Fin (MTEF)**		**16,298.547**	**6,220.293**	**18,889.511**	**20,998.642**	**21,013.165**	**25,729.864**	**25,798.077**
	A.I.A	0.000	0.000	0.000	0.000	0.000	0.000	0.000
	**Grand Total**	**16,298.547**	**6,220.293**	**18,889.511**	**20,998.642**	**21,013.165**	**25,729.864**	**25,798.077**

Programme Strategy and linkage to the National Development Plan

The goal of the Development Plan Implementation (DPI) Programme is to increase efficiency and effectiveness in the implementation of the National Development Plan. It therefore contributes to the entire NDP III by putting in place mechanisms that will enable its effective implementation and which in turn will ensure that at least 80% of the plan's targets are achieved. Its strategic objectives in this regards include

(i) Objective 1: Strengthening capacity for development planning
(ii) Objective 2: Strengthening budgeting and resource mobilization
(iii) Objective 3: Strengthening capacity for implementation to ensure a focus on results
(iv) Objective 4: Strengthening coordination, monitoring, reporting frameworks and systems
(v) Objective 5: Strengthening the capacity of the national statistics system to generate data for National Development
(vi) Objective 6: Strengthening the research and evaluation function to better inform planning and plan implementation

The Programme will give priority to Strengthening budgeting and resource mobilization which is the anchor for NDP III success. This objective aims at mobilising resources needed to implement the NDP III. The aggregate cost of financing all the NDPIII programme interventions over the 5-year period is estimated at around UGX 411.681 trillion . Of this cost, UGX 276.878 trillion (67.3 percent) is contribution by the Public (incl. off budget), while UGX 134.803 trillion (32.7 percent) is private sector contribution (less recurrent private sector expenditure). The key areas of focus will be;

1. Deepening the reduction of informality and streamlining taxation at national and local government levels.
2. Amending and developing relevant legal frameworks to facilitate resource mobilization and budget execution.
3. Strengthening the alignment of the Programmes, MDA and LG Plans to the NDP III

Under this Budget Framework Paper, we continue building on ongoing interventions, but will give priority interventions most relevant to economic recovery and to the implementation of the Parish Development Model (PDM). PDM is a key Government intervention designed to improve planning and services delivery at the lowest levels of society and has the potential to greatly impact the NDP III's goal of "Increased Household Incomes and Improved Quality of Life of Ugandans". We will also give priority to strengthening capacity for coordination, oversight, monitoring and reporting by strengthening the

DEVELOPMENT PLAN IMPLEMENTATION

[illegible]
We will also give priority to strengthening capacity for coordination, oversight, monitoring and reporting by strengthening the APEX Platform – a high level Executive Organ to strengthen the oversight roles of the Presidency over the NDP III. This will be supplemented with matching efforts for capacity strengthening within the other coordination structures, including at the Office of the Prime Minister, the Ministry of Finance, Planning and Economic Development, the National Planning Authority,

P2: Highlights Of Programme Projected Performance

Table P2.1 Programme Outcomes Indicators

**Programme Outcome**	Sustainable economic growth and stability						
**Programme Objectives contributed to by the Intermediate Outcome**							
Strengthen budgeting and resource mobilization							
	**Performance Targets**						
**Programme Outcome Indicators**	**Base Year**	**Base Line**	**2023/24**	**2024/25**	**2025/26**	**2026/27**	
GDP growth rate	2017-18	6.2%	7%	7.2%	7.2%	7.2%	
Nominal Debt to GDP ratio	2017-18	40.6	48.4	46.71	40	40	
**Programme Outcome**	Increased Budget self sufficiency						
**Programme Objectives contributed to by the Intermediate Outcome**							
Strengthen budgeting and resource mobilization							
	**Performance Targets**						
**Programme Outcome Indicators**	**Base Year**	**Base Line**	**2023/24**	**2024/25**	**2025/26**	**2026/27**	
Domestic revenue to GDP (%)	2017-18	13.36%	14.4%	15.39%	15.39%	15.39%	
Revenue to GDP ratio	2017-18	13.36%	14.49%	15.39%	15.39%	15.39%	
**Programme Outcome**	Effective and efficient allocation and utilization of public resources						
**Programme Objectives contributed to by the Intermediate Outcome**							
Strengthen capacity for development planning							
	**Performance Targets**						
**Programme Outcome Indicators**	**Base Year**	**Base Line**	**2023/24**	**2024/25**	**2025/26**	**2026/27**	
Percentage of budget released against originally approved budget.	2017-18	108.2%	100%	100%	100%	100%	
Percentage of funds absorbed against funds released.	2017-18	99.2%	100%	100%	100%	100%	
**Programme Outcome**	Improved alignment of the plans and budgets						
**Programme Objectives contributed to by the Intermediate Outcome**							
Strengthen capacity for development planning							
	**Performance Targets**						
**Programme Outcome Indicators**	**Base Year**	**Base Line**	**2023/24**	**2024/25**	**2025/26**	**2026/27**	
Budget compliance to the NDP, %	2017-18	60%	100%	100%	100%	100%	

DEVELOPMENT PLAN IMPLEMENTATION

**Programme Outcome**	Improved development results					
**Programme Objectives contributed to by the Intermediate Outcome**						
Strengthen capacity for implementation to ensure a focus on results						
	**Performance Targets**					
**Programme Outcome Indicators**	**Base Year**	**Base Line**	**2023/24**	**2024/25**	**2025/26**	**2026/27**
Proportion of NDP results on target	2017-18	NA	100%	100%	100%	100%
**Programme Outcome**	Statistical programmes aligned to National, regional and international development frameworks					
**Programme Objectives contributed to by the Intermediate Outcome**						
Strengthen capacity for implementation to ensure a focus on results						
	**Performance Targets**					
**Programme Outcome Indicators**	**Base Year**	**Base Line**	**2023/24**	**2024/25**	**2025/26**	**2026/27**
World Bank Statistical Capacity Indicator (WBSCI) score	2017-18	74.4%	78.9%	80%	100%	100%
**Programme Outcome**	Improved budget credibility					
**Programme Objectives contributed to by the Intermediate Outcome**						
Strengthen coordination, monitoring and reporting frameworks and systems						
	**Performance Targets**					
**Programme Outcome Indicators**	**Base Year**	**Base Line**	**2023/24**	**2024/25**	**2025/26**	**2026/27**
Arrears as a percentage of total expenditure for FY N-1	2017-18	1%	0.4%	0.2%	0.2%	0.2%
Budget transparency index	2017-18	60%	72%	75%	80%	85%
National Budget compliance to Gender and equity	2017-18	55%	90%	97%	100%	100%
Supplementary expenditure as a percentage of the initial approved budget	2017-18	5.9%	3%	3%	3%	3%
**Programme Outcome**	Evidence based decision making					
**Programme Objectives contributed to by the Intermediate Outcome**						
Strengthen the capacity of the statistical system to generate data for national development						
	**Performance Targets**					
**Programme Outcome Indicators**	**Base Year**	**Base Line**	**2023/24**	**2024/25**	**2025/26**	**2026/27**
Proportion of key indicators up-to-date with periodic data	2017-18	40	90	100	100	100
Proportion of NDPIII baseline indicators up-to-date & updated	2017-18	60	90	100	100	100
**Programme Outcome**	Enhanced use of data for evidence-based policy and decision making					
**Programme Objectives contributed to by the Intermediate Outcome**						
Strengthen the capacity of the statistical system to generate data for national development						
	**Performance Targets**					
**Programme Outcome Indicators**	**Base Year**	**Base Line**	**2023/24**	**2024/25**	**2025/26**	**2026/27**
Proportion of NDP results framework informed by official statistics	2017-18	30	100	100	100	100

DEVELOPMENT PLAN IMPLEMENTATION

Table P2.2: Intermediate Outcomes Indicators

**Sub-Programme Name:**	**Development Planning, Research, Evaluation and Statistics**					
**Intermediate Outcome Indicators:**	Effective and efficient allocation and utilization of public resources					
	**Performance Targets**					
**Indicators**	**Base Year**	**Base Line**	**2023/24**	**2024/25**	**2025/26**	**2026/27**
Budget alignment to NDP (%)	2017-18	60%	90%	100%	100%	100%
Percentage of budget released against originally approved budget.	2017-18	108.2%	100%	100%	100%	100%
Percentage of funds absorbed against funds released.	2017-18	99.2%	100%	100%	100%	100%
Proportion of LG plans aligned to the NDP	2017-18	94.5%	80%	85%	90%	100%
Proportion of MDA plans aligned to the NDP	2017-18	60%	95%	100%	100%	100%
**Intermediate Outcome Indicators:**	Effective Public Investment Management					
	**Performance Targets**					
**Indicators**	**Base Year**	**Base Line**	**2023/24**	**2024/25**	**2025/26**	**2026/27**
Gross Capital formation (% of GDP)	2017-18	24.2%	27.4%	27.7%	28%	28.5%
Share of PIP Projects implemented on time (%)	2017-18	60%	95%	100%	100%	100%
Share of PIP Projects implemented within the approved budget	2017-18	60%	80%	90%	100%	100%
**Intermediate Outcome Indicators:**	Enhanced use of data for evidence-based policy and decision making					
	**Performance Targets**					
**Indicators**	**Base Year**	**Base Line**	**2023/24**	**2024/25**	**2025/26**	**2026/27**
Proportion of key indicators up-to-date with periodic data	2017-18	60%	90%	100%	100%	100%
Proportion of NDP results framework informed by Official Statistics	2017-18	30%	100%	100%	100%	100%
Proportion of NDPIII baseline indicators up-to-date & updated	2017-18	40%	95%	100%	100%	100%
**Sub-Programme Name:**	**Resource Mobilization and Budgeting**					
**Intermediate Outcome Indicators:**	Fiscal credibility and Sustainability					
	**Performance Targets**					
**Indicators**	**Base Year**	**Base Line**	**2023/24**	**2024/25**	**2025/26**	**2026/27**
Domestic revenue to GDP (%)	2017-18	12.95%	14.3%	15.43%	16%	16.5%
External resource envelope as a percentage of the National Budget.	2017-18	20%	15%	12%	10%	8%
Proportion of direct budget transfers to local government	2017-18	12.25%	29.1%	30%	35%	35%
Nominal Debt to GDP ratio	2017-18	40.06	44.73	41.38	40	38
Revenue to GDP ratio	2017-18	12.95	14.49	15.01	15.5	16.0
Tax revenue to GDP ratio	2017-18	12.58	13.32	13.8	14.2	14.5

DEVELOPMENT PLAN IMPLEMENTATION

**Sub-Programme Name:**	**Resource Mobilization and Budgeting**					
**Intermediate Outcome Indicators:**	Improved Budget Credibility					
	**Performance Targets**					
**Indicators**	**Base Year**	**Base Line**	**2023/24**	**2024/25**	**2025/26**	**2026/27**
Arrears as a percentage of total expenditure for FY N-1	2017-18	1%	0.4%	0.2%	0.1%	0%
Budget transparency index	2017-18	60%	72%	75%	80%	85%
Compliance of the National Budget to NDP (%)	2017-18	60%	90%	100%	100%	100%
National Budget compliance to Gender and equity (%)	2017-18	55%	90%	97%	100%	100%
Supplementary as a percentage of the Initial budget	2017-18	5.89%	3%	3%	2%	1%
Green Economy (GE)Public expenditure Review (PER) grading	2017-18	Average	Above Average	Above Average	Above Average	Above Average
**Sub-Programme Name:**	**Oversight, Implementation, Coordination and Monitoring**					
**Intermediate Outcome Indicators:**	Improved development results					
	**Performance Targets**					
**Indicators**	**Base Year**	**Base Line**	**2023/24**	**2024/25**	**2025/26**	**2026/27**
Proportion of NDP results on target	2017-18	50%	90%	100%	100%	100%
**Intermediate Outcome Indicators:**	Improved Service Delivery					
	**Performance Targets**					
**Indicators**	**Base Year**	**Base Line**	**2023/24**	**2024/25**	**2025/26**	**2026/27**
Government Effectiveness index	2017-18	-0.5	0.004%	0.01	0.05	0.1
Level of satisfaction of public Service by Service (Administrative and Legal Services)	2017=18	60	97	100	100	100
Level of satisfaction of public Service by Service (Electricity)	2017-18	61.8	96	100	100	100
Level of satisfaction of public Service by Service (Extension services)	2017-18	75	95	100	100	100
Level of satisfaction of public Service by Service (Water transport)	2017-18	69	92	100	100	100
**Sub-Programme Name:**	**Accountability Systems and Service Delivery**					
**Intermediate Outcome Indicators:**	Improved compliance with accountability rules and regulations					
	**Performance Targets**					
**Indicators**	**Base Year**	**Base Line**	**2023/24**	**2024/25**	**2025/26**	**2026/27**
External auditor ratings (unqualified)	2017-18	40%	87%	95%	100%	100%
Percentage of internal audit recommendations implemented	2017-18	65.5%	100%	100%	100%	100%
Proportion of prior year external audit recommendations implemented, %	2017-18	31%	52%	55%	60%	65%

P3: Medium Term Budget Allocations by Sub-Programme and Vote

Table P3.1: Proposed Budget Allocations and Medium Term Projections by Sub-Programme

DEVELOPMENT PLAN IMPLEMENTATION

Billion Uganda Shillings	FY2022/23	2023/24	Medium Term Projections			
	Approved Budget	Proposed Budget	2024/25	2025/26	2026/27	2027/28
01 Development Planning, Research, Evaluation and Statistics	215.422	396.989	433.919	518.344	694.589	694.589
02 Resource Mobilization and Budgeting	15,825.060	18,236.341	20,310.443	20,184.170	24,630.855	24,699.061
03 Oversight, Implementation, Coordination and Monitoring	4.788	14.185	14.687	19.611	33.121	33.121
04 Accountability Systems and Service Delivery	253.277	241.997	239.593	291.041	371.298	371.305
**Total for the Programme**	**16,298.547**	**18,889.511**	**20,998.642**	**21.013.165**	**25,729.864**	**25,798.077**

Table P3.2: Proposed Budget Allocations and Medium Term Projections by Vote

Billion Uganda Shillings	FY2022/23		2023/24	Medium Term Projections			
	Approved Budget	Spent by End Sep	Proposed Budget	2024/25	2025/26	2026/27	2027/28
001 Office of the President	14.558	**1.413**	14.558	14.857	17.799	23.948	23.948
003 Office of the Prime Minister	51.403	**7.740**	52.404	53.473	63.830	85.469	85.469
006 Ministry of Foreign Affairs	0.346	**0**	0.346	0.353	0.424	0.572	0.572
008 Ministry of Finance, Planning and Economic Development	374.166	**36.702**	353.988	349.784	418.797	573.610	573.610
011 Ministry of Local Government	19.537	**0.943**	19.537	19.986	23.778	31.536	31.536
023 Ministry of Kampala Capital City and Metropolitan Affairs	0.050	**0**	0.050	0.051	0.061	0.083	0.083
103 Inspectorate of Government (IG)		**0**	9.397	9.397	11.276	15.787	15.787
108 National Planning Authority (NPA)	28.605	**5.271**	31.515	32.525	37.496	46.575	46.575
122 Kampala Capital City Authority (KCCA)	2.532	**0.311**	2.531	2.573	3.088	4.194	4.194
123 National Lotteries and Gaming Regulatory Board	0.342	**0.023**	0.342	0.349	0.419	0.565	0.565
124 Equal Opportunities Commission	11.082	**1.254**	11.082	11.388	13.354	17.185	17.185
130 Treasury Operations	15,174.174	**6,027.298**	17,565.632	19,653.738	19,428.133	23,652.405	23,720.618
131 Office of the Auditor General (OAG)		**0**	1.000	1.020	1.224	1.652	1.652
141 Uganda Revenue Authority (URA)	539.834	**129.508**	567.140	583.741	678.912	859.914	859.914
143 Uganda Bureau of Statistics (UBOS)	72.055	**8.041**	249.587	254.951	303.695	404.619	404.619
147 Local Government Finance Commission (LGFC)	1.798	**0.019**	1.798	1.849	2.165	2.774	2.774
149 National Population Council		**0**	0.541	0.541	0.649	0.909	0.909
501 Uganda Mission at the United Nations, New York	0.148	**0.037**	0.148	0.148	0.148	0.148	0.148
502 Uganda High Commission in the United Kingdom	1.277	**0.319**	1.277	1.277	1.277	1.277	1.277

DEVELOPMENT PLAN IMPLEMENTATION

Billion Uganda Shillings	FY2022/23		2023/24	Medium Term Projections			
	Approved Budget	Spent by End Sep	Proposed Budget	2024/25	2025/26	2026/27	2027/28
503 Uganda High Commission in Canada, Ottawa	0.110	0	0.110	0.110	0.110	0.110	0.110
504 Uganda High Commission in India, New Delhi	1.000	0.083	1.000	1.000	1.000	1.000	1.000
505 Uganda High Commission in Kenya, Nairobi	0.088	0.047	0.088	0.088	0.088	0.088	0.088
506 Uganda High Commission in Tanzania, Dar es Salaam	0.301	0.075	0.301	0.301	0.301	0.301	0.301
510 Uganda Embassy in the United States, Washington	0.736	0.114	0.736	0.736	0.736	0.736	0.736
511 Uganda Embassy in Egypt, Cairo	0.129	0.082	0.129	0.129	0.129	0.129	0.129
512 Uganda Embassy in Ethiopia, Addis Ababa	0.489	0.354	0.489	0.489	0.489	0.489	0.489
513 Uganda Embassy in China, Beijing	0.266	0	0.266	0.266	0.266	0.266	0.266
514 Uganda Embassy in Switzerland, Geneva	0.343	0.087	0.343	0.343	0.343	0.343	0.343
515 Uganda Embassy in Japan, Tokyo	0.100	0	0.100	0.100	0.100	0.100	0.100
516 Uganda Embassy in Saudi Arabia, Riyadh	0.520	0	0.520	0.520	0.520	0.520	0.520
517 Uganda Embassy in Denmark, Copenhagen	0.161	0.026	0.161	0.161	0.161	0.161	0.161
519 Uganda Embassy in Italy, Rome	0.035	0	0.035	0.035	0.035	0.035	0.035
523 Uganda Embassy in Germany, Berlin	0.053	0.006	0.053	0.053	0.053	0.053	0.053
524 Uganda Embassy in Iran, Tehran	0.252	0	0.252	0.252	0.252	0.252	0.252
527 Uganda Embassy in South Sudan, Juba	0.155	0.038	0.155	0.155	0.155	0.155	0.155
528 Uganda Embassy in United Arab Emirates, Abudhabi	1.600	0.347	1.600	1.600	1.600	1.600	1.600
535 Uganda Embassy in Algeria, Algiers	0.197	0.153	0.197	0.197	0.197	0.197	0.197
536 Uganda Embassy in Qatar, Doha	0.104	0	0.104	0.104	0.104	0.104	0.104
**Total for the Programme**	**16,298.547**	**6,220.293**	**18,889.511**	**20,998.642**	**21,013.165**	**25,729.864**	**25,798.077**

P4: PROGRAMME PRIORITIES IN LINE WITH INTERVENTIONS FOR FY2023/24

Programme Priorities FY2023/24	NDP III Programme Intervention
Undertake long-term censuses and surveys integrating NDPIII, Agenda 2063, SDGs data requirements	Align and synchronize national survey and census programmes to NDPIII, Africa Agenda 2063, SDGs and other development framework data requirements;
testing	Alignment of budgets to development plans at national and sub-national levels
Review and Amend/develop the different legal frameworks to facilitate effective and efficient Resource Mobilization and Budget execution.	Amend and develop relevant legal frameworks to facilitate resource mobilisation and budget execution.
Implement Domestic Revenue mobilization, update the existing tax payer register; automate revenue administration processes	Deepening the reduction of informality and streamlining taxation at national and local government levels

DEVELOPMENT PLAN IMPLEMENTATION

Programme Priorities FY2023/24	NDP III Programme Intervention
Consultations and development of NDP IV	Strengthen capacity for development planning at the sector, MDAs and local government levels
Allocate financial resources to the Programme Secretariat through the Programme lead Agency to be able to facilitate the PWGs to be able to execute their roles as guided by the NDPIII programme Guidelines	Strengthen implementation, monitoring and reporting of local governments
Support the preparation of the pre-feasibility, feasibility and value chain studies in priority NDP III projects/areas	Strengthen Public Investment Management across the entire government to be able to develop bankable projects on time
Assess the compliance of the MDA and LG Plans and Budgets to NDPIII programmes	Strengthen the alignment of the Sector, MDA and LG Plans to the NDP III
Monitor the implementation of SDGs and facilitate meetings to fast track the SDGs.	Strengthen the follow up mechanism to streamline the roles of the relevant oversight committees to avoid duplication of roles
Equip and resource parishes to operationalize service delivery structures.	Strengthen the planning and development function at the parish level to bring delivery of services closer to the people;

P5: GENDER AND EQUITY ISSUES AND INTERVENTIONS FOR FY2023/24

Gender and Equity Issues

1. Gender and Equity policy not yet operationalized.
2. Limited capacity for mainstreaming gender and equity issues in policies, programs and projects.
3. Limited use and availability for gender dis-aggregated data

HIV/AIDS and other health related issues.

1. Limited access to medical services at the workplace including Medicare for staff living with HIV/AIDs
2. Limited access to health information by staff especially non communicable diseases like cancer, Diabetes

ADMINISTRATION OF JUSTICE

Foreword

Administration of Justice contributes directly to the structural transformation of an economy by promoting the rule of law through effective regulation of economic activity, clarification and affirmation of rights, and strengthening laws, regulations and institutional frameworks. It is the process by which the legal system of Government is executed to provide justice for all.

There remains limited access to justice occasioned by the existence of weak and outdated laws, inadequate number and skills of the human resource, high volume and sophistication of crime, limited use of technology, inadequate institutional infrastructure, corruption, limited awareness of the Administration of Justice services, and high cost of accessing Justice.

The goal of the Programme is to strengthen Access to Justice for all. The key results to be achieved are –Increased public trust in the justice system, Reduced lead times in the delivery of justice services, Increase in the index of Judicial independence and Improve the corruption perception index.

The Programme key objectives are the following- Strengthen people-centered Justice service delivery system, Reform and strengthen Justice business processes, Strengthen the fight against corruption, Strengthen Legal, regulatory and institutional frameworks for effective and efficient delivery of Justice.

Programme Working Group Composition includes the following- The Judiciary (Lead Institution), Judicial Service Commission, Uganda Police Force, Uganda Prisons Service, Ministry of Gender, Labour and Social Development, Law Development Centre, Ministry of Finance, Planning and Economic Development (Tax Appeal Tribunal) & Office of the Director of Public Prosecution.

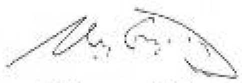

Alfonse Chigamoy Owiny-Dollo

Chief Justice

ADMINISTRATION OF JUSTICE

Abbrevations and Acronyms

ACRONYM	ACRONYM NAME
AJA	Administration of Judiciary Act, 2020
AJP	Administration of Justice Programme
CM	Chief Magistrate
COA	Court of Appeal
DCC	District Chain Linked Committee
ECCMIS	Electronic Court Case Management Information System
ICD	International Crimes Division
JTI	Judicial Training Institute
LAN/WAN	Local Area Network/Wide Area Network
PET	Performance Enhancement Tool
SCP	Small Claims Procedure

ADMINISTRATION OF JUSTICE

P1: PROGRAMME OVERVIEW

Snapshot of Medium Term Budget Allocations

Table P1.1 Overview of Programme Expenditure and Medium Term Allocations (Ush Billion)

Billion Uganda Shillings		FY2022/23		FY2023/24	MTEF Budget Projections			
		Approved Budget	Spent by End Sep	Proposed Budget	2024/25	2025/26	2026/27	2027/28
Recurrent	Wage	112.429	19.386	116.283	122.097	134.306	147.737	147.737
	Non Wage	224.117	46.508	240.290	245.096	294.115	397.055	397.055
Devt.	GoU	63.473	0.000	66.223	66.223	79.468	111.255	111.255
	ExtFin	0.000	0.000	0.000	0.000	0.000	0.000	0.000
	**GoU Total**	**400.019**	**65.894**	**422.796**	**433.416**	**507.889**	**656.047**	**656.047**
	**Total GoU+Ext Fin (MTEF)**	**400.019**	**65.894**	**422.796**	**433.416**	**507.889**	**656.047**	**656.047**
	A.I.A	0.000	0.000	0.000	0.000	0.000	0.000	0.000
	**Grand Total**	**400.019**	**65.894**	**422.796**	**433.416**	**507.889**	**656.047**	**656.047**

Programme Strategy and linkage to the National Development Plan

NDP III recognizes good governance as the panacea to achieving accelerated development and the rule of law as the foundation of a free society that places limits on government authority such that all citizens are equally subject to a common set of laws.

The NDP III identifies the challenges of delayed delivery of justice leading to a growing case backlog. It recognizes the need to strengthen the capacity and operations of the commercial justice institutions to provide fast and effective dispute resolution in all the specialized areas and in Alternative Dispute Resolution (ADR). The plan further observes that while implementation of the sector anti-corruption strategy is on track with the corruption perception index improving from 0.25 to 0.26, there is a need for further improvement.

The NDP III emphasizes that sustainable management of the environment and natural resources together with tackling climate change are key issues on Uganda's development agenda.

The Administration of Justice Programme has a role to play by providing equal access to justice and timely disposal of cases. The Programme will also support the Natural Resource, Environment, Climate Change, Land and Water Resources Management Programme by conducting special sessions relating to natural resources, the environment, climate change, land and water management among other key priority areas.

P2: Highlights Of Programme Projected Performance

Table P2.1 Programme Outcomes Indicators

ADMINISTRATION OF JUSTICE

**Programme Outcome**	Strengthen people centred justice service delivery system					
**Programme Objectives contributed to by the Intermediate Outcome**						
Strengthen people centered Justice service delivery system;						
			**Performance Targets**			
**Programme Outcome Indicators**	**Base Year**	**Base Line**	**2023/24**	**2024/25**	**2025/26**	**2026/27**
Judicial Independence Index	2017-2018	3.41	3.7%	3.8	3.82	3.84
Level of public trust in the Justice system	2017-2018	N/A	62.0%	67.0%	72.0%	77.0%
Proportion of districts with a complete administration of Justice Service delivery point (%)	2017-2018	67.5%	85.9%	87.6%	89.3%	91.0%
**Programme Outcome**	Reduced case backlog					
**Programme Objectives contributed to by the Intermediate Outcome**						
Reform and strengthen Justice business processes;						
			**Performance Targets**			
**Programme Outcome Indicators**	**Base Year**	**Base Line**	**2023/24**	**2024/25**	**2025/26**	**2026/27**
Percentage of backlog cases in the Administration of Justice system (%)	2017-2018	N/A	27.5%	27%	26.5%	26.0%
**Programme Outcome**	Reduced Lead Time /Turnaround Time					
**Programme Objectives contributed to by the Intermediate Outcome**						
Reform and strengthen Justice business processes;						
			**Performance Targets**			
**Programme Outcome Indicators**	**Base Year**	**Base Line**	**2023/24**	**2024/25**	**2025/26**	**2026/27**
Average length of stay on remand (months) for capital offenders	2017-2018	18.3 months	14.1 months	13.2 months	12.3 months	11.4 months
Lead time /Turnaround time(days)	2017-2018	N/A	870 days	860 days	850 days	840 days
Proportion of prison population on remand	2017-2018	51.4%	43.3%	42.2%	41.1%	40.0%
**Programme Outcome**	Effective and Efficient administration of Justice system					
**Programme Objectives contributed to by the Intermediate Outcome**						
Strengthen regulatory and institutional frameworks for effective and efficient delivery of Justice						
			**Performance Targets**			
**Programme Outcome Indicators**	**Base Year**	**Base Line**	**2023/24**	**2024/25**	**2025/26**	**2026/27**
Conviction Rate	FY 2017-2018	60.0%	75.0%	78.0%	81.0%	84.0%
Judicial Officer to population ratio	FY 2017-2018	1:116,808	1:91,490	1:81,490	1:71,490	1:61,490
Level of public satisfaction in the administration of Justice system	FY 2017-2018	40.0%	72.0%	75.0%	78.0%	81.0%
Prosecutor to case ratio	FY 2017/18	1:338	1:248	1:227	1:206	1:185

Table P2.2: Intermediate Outcomes Indicators

ADMINISTRATION OF JUSTICE

Sub-Programme Name:	Institutional Coordination					
**Intermediate Outcome Indicators:**	Improved M&E Systems					
	**Performance Targets**					
**Indicators**	**Base Year**	**Base Line**	**2023/24**	**2024/25**	**2025/26**	**2026/27**
Proportion of Justice Delivery institutions with existing statistics strategies (%)	2017-2018	25.0%	85.0%	100.0%	100.0%	100.0%
Proportion of Justice Delivery institutions with functional M&E Units (%)	2017-2018	25.0%	85.0%	100.0%	100.0%	100.0%
**Intermediate Outcome Indicators:**	Increased funding to the Judiciary					
	**Performance Targets**					
**Indicators**	**Base Year**	**Base Line**	**2023/24**	**2024/25**	**2025/26**	**2026/27**
Percentage of the Country's National Budget allocated to the Judiciary (%).	2017-2018	0.5%	2.2%	2.3%	2.4%	2.5%
**Intermediate Outcome Indicators:**	Increased presence and functionality of Justice service points					
	**Performance Targets**					
**Indicators**	**Base Year**	**Base Line**	**2023/24**	**2024/25**	**2025/26**	**2026/27**
Proportion of designated regions with at least an operation JSC Office (%)	2017-2018	20.0%	50.0%	60.0%	70.0%	80.0%
Proportion of districts with at least an operational Chief Magistrate Court (%)	2017-2018	54.0%	76.0%	77.0%	78.0%	79.0%
Proportion of operational Courts with facilities (physical and functional) for people with special needs (%)	2017-2018	N/A	23.0%	25.0%	27.0%	29.0%
**Intermediate Outcome Indicators:**	Improved staffing in the Justice Institutions					
	**Performance Targets**					
**Indicators**	**Base Year**	**Base Line**	**2023/24**	**2024/25**	**2025/26**	**2026/27**
Proportion of the approved staff structure filled for Judicial Officers (%)	2017-2018	68.0%	77.0%	90.0%	100.0%	100.0%
Proportion of the approved staff structure filled for staff of the Judiciary (%)	2017-2018	46.0%	70.0%	80.0%	90%	100%
**Intermediate Outcome Indicators:**	Improved adherence to the Code of Conduct by Judicial and Staff of the Judiciary					
	**Performance Targets**					
**Indicators**	**Base Year**	**Base Line**	**2023/24**	**2024/25**	**2025/26**	**2026/27**
Number of complaints registered at JSC	2017-2018	124	80	85	90	95
Proportion of Justice Delivery Institutions with documented Service Delivery Standards (%)	FY2017-2018	0.0%	87.5%	100.0%	100.0%	100.0%
Proportion of Justice Delivery Institutions with existing Anti-Corruption Strategies (%)	FY2017-2018	0.0%	95.0%	100.0%	100.0%	100.0%

ADMINISTRATION OF JUSTICE

Sub-Programme Name:	Civil and Criminal Justice					
Intermediate Outcome Indicators:	Increased disposal of Criminal cases					
	Performance Targets					
**Indicators**	**Base Year**	**Base Line**	**2023/24**	**2024/25**	**2025/26**	**2026/27**
Disposal Rate of juvenile cases (%)	2017-2018	N/A	70.0%	71.0%	72.0%	73.0%
Disposal Rate of Refugee related cases (%)	2017-2018	N/A	43.0%	44.0%	45.0%	46.0%
Rate of Cybercrime cases prosecuted	2017-2018	N/A	70.0%	75.0%	80.0%	85.0%
Case disposal Rate of Criminal Cases (%)	2017-2018	N/A	62.0%	64.0%	66.0%	68.0%
Clearance Rate of Corruption Cases (%)	2017-2018	N/A	106.3%	108.0%	110.0%	112.0%
Clearance Rate of Criminal Cases (%)	2017-2018	N/A	91.0%	92.0%	93.0%	94.0%
Clearance Rate of International Crimes (%)	2017-2018	N/A	27.0%	29.0%	31.0%	33.0%
Disposal Rate of Cases on Environmental Justice and Climate Change (%)	2017-2018	N/A	109.0%	110.0%	111.0%	112.0%
Disposal Rate of Corruption Cases (%)	2017-2018	N/A	55.0%	57.0%	59.0%	61.0%
Disposal Rate of Gender Based Violence cases (%)	2017-2018	N/A	43.0%	44.0%	45.0%	46.0%
**Intermediate Outcome Indicators:**	Increased number of complaints against Judicial Officers and Staff of the Judiciary disposed of					
	Performance Targets					
**Indicators**	**Base Year**	**Base Line**	**2023/24**	**2024/25**	**2025/26**	**2026/27**
Complaints disposal rate (%)	2017-2018	N/A	77.0%	78.0%	79.0%	80.0%
**Intermediate Outcome Indicators:**	Increased Rate of disposal of Civil cases					
	Performance Targets					
**Indicators**	**Base Year**	**Base Line**	**2023/24**	**2024/25**	**2025/26**	**2026/27**
Clearance Rate of Civil Cases (%)	2017-2018	N/A	93.0%	95.0%	97.0%	99.0%
Disposal Rate of Commercial Cases	2017-2018	N/A	39.0%	41.0%	43.0%	45.0%
Disposal Rate of Civil Cases (%)	2017-2018	N/A	53.0%	55.0%	57.0%	59.0%
Disposal Rate of Family Cases (%)	2017-2018	N/A	51.0%	53.0%	55.0%	57.0%
Disposal Rate of Land Cases (%)	2017-2018	N/A	41.0%	43.0%	45.0%	47.0%
Disposal Rate of Tax disputes (%)	2017-2018	70.0%	90.0%	95.0%	96.5%	98.0%
**Intermediate Outcome Indicators:**	Reduced backlog in Civil Cases					
	Performance Targets					
**Indicators**	**Base Year**	**Base Line**	**2023/24**	**2024/25**	**2025/26**	**2026/27**
Disposal Rate of Labour Disputes (%)	2017-2018	8.0%	40.0%	50.0%	55.0%	60.0%
Percentage of Backlog in Civil Cases (%)	2017-2018	N/A	28.0%	26.0%	24.0%	22.0%
Percentage of Backlog in Commercial Cases (%)	2017-2018	N/A	30.0%	28.0%	26.0%	24.0%
Percentage of Backlog in Family Cases (%)	2017-2018	N/A	31.0%	29.0%	27.0%	25.0%
Percentage of Backlog in Land Cases (%)	2017-2018	N/A	30.0%	28.0%	26.0%	24.0%

ADMINISTRATION OF JUSTICE

Sub-Programme Name:	Civil and Criminal Justice					
**Intermediate Outcome Indicators:**	Reduced backlog in Civil Cases					
			Performance Targets			
**Indicators**	**Base Year**	**Base Line**	**2023/24**	**2024/25**	**2025/26**	**2026/27**
Percentage of cases pending beyond 2 years at JSC (%)	2017-2018	N/A	20.0%	18.0%	16.0%	14.0%
**Intermediate Outcome Indicators:**	Increase Rate of Sanctioning Anti-Corruption Case Files					
			Performance Targets			
**Indicators**	**Base Year**	**Base Line**	**2023/24**	**2024/25**	**2025/26**	**2026/27**
Percentage of corruption cases committed within 120 days(%).	2017-2018	N/A	35.0%	40.0%	45.0%	50.0%
**Intermediate Outcome Indicators:**	Increased No of people accessing legal aid					
			Performance Targets			
**Indicators**	**Base Year**	**Base Line**	**2023/24**	**2024/25**	**2025/26**	**2026/27**
Proportion of eligible persons accessing legal aid (%)	2017-2018	N/A	32.0%	37.0%	42.0%	47.0%
**Intermediate Outcome Indicators:**	Reduced backlog in Criminal Cases					
			Performance Targets			
**Indicators**	**Base Year**	**Base Line**	**2023/24**	**2024/25**	**2025/26**	**2026/27**
Percentage of backlog in Anti-Corruption Cases (%)	2017-2018	N/A	32.0%	30.0%	28.0%	26.0%
Percentage of backlog in Criminal Cases (%)	2017-2018	N/A	20.0%	18.0%	16.0%	14.0%
Percentage of prisoners on remand beyond 2 years after committal.	2017-2018	39.4%	30.0%	28.0%	26.0%	24.0%
Proportion of corruption backlog cases prosecuted	2017-2018	N/A	70.0%	75.0%	80.0%	85.0%
Proportion of corruption cases concluded beyond 2 years after registration in court	2017-2018	N/A	62.0%	64.0%	66.0%	68.0%
**Intermediate Outcome Indicators:**	Set standards and statutory timelines adhered to					
			Performance Targets			
**Indicators**	**Base Year**	**Base Line**	**2023/24**	**2024/25**	**2025/26**	**2026/27**
Percentage of capital cases committed within 120 days (%).	2017-2018	N/A	78.0%	80.0%	82.0%	84.0%
Percentage of case files sanctioned within 2 working days (%).	2017-2018	N/A	65.0%	70.0%	75.0%	80.0%
Percentage of casefiles prosecution decision made within 20 business working days (%).	2017-2018	N/A	72.0%	75.0%	80.0%	85.0%
Percentage of Judgements delivered within 60 days after close of hearing. (%)	2017-2018	N/A	27.0%	30.0%	33.0%	36.0%
Percentage of prosecution led investigations into general crimes casefiles concluded within 60 business days.	2017-2018	N/A	35.0%	40.0%	45.0%	50.0%

ADMINISTRATION OF JUSTICE

**Sub-Programme Name:**	**Legal Education, Training and Research**					
**Intermediate Outcome Indicators:**	Increased public awareness of Justice Service					
	Performance Targets					
**Indicators**	**Base Year**	**Base Line**	**2023/24**	**2024/25**	**2025/26**	**2026/27**
Percentage of Administration of Justice Delivery points with customer care desks (%)	2017-2018	53.0%	85.0%	87.0%	89.0%	91.0%
Percentage of Administration of Justice Institutions with existing Client Charters (%)	2017-2018	100.0%	85.0%	100.0%	100.0%	100.0%
Percentage of Administration of Justice Institutions with operational call centres (%)	2017-2018	25.0%	62.5%	87.5%	100%	100%
**Intermediate Outcome Indicators:**	Increased capacity of legal practitioners					
	Performance Targets					
**Indicators**	**Base Year**	**Base Line**	**2023/24**	**2024/25**	**2025/26**	**2026/27**
Number Legal Aid Practitioners trained	2017-2018	20	150	200	250	300
Percentage of forensic scientists and scene of crime officers trained(%).	2017-2018	N/A	55.0%	70.0%	80.0%	90.0%
Percentage of Judicial Officers in the management of causes related to GBV, Juveniles, and Refugees, the elderly and other vulnerable groups (%)	2017-2018	N/A	60.0%	70.0%	80.0%	90.0%
Percentage of Justice Actors trained in customer care (%)	2017-2018	N/A	40.0%	50.0%	60.0%	70.0%
Percentage of Labour Officers trained in Conciliation, Mediation and Arbitration of labour complaints and disputes settlement (%)	2017-2018	5.0%	40.0%	50.0%	60.0%	70.0%
**Intermediate Outcome Indicators:**	Increased legal research					
	Performance Targets					
**Indicators**	**Base Year**	**Base Line**	**2023/24**	**2024/25**	**2025/26**	**2026/27**
Number of Legal Publications	2017-2018	300	2000	2600	3200	3800

P3: Medium Term Budget Allocations by Sub-Programme and Vote

Table P3.1: Proposed Budget Allocations and Medium Term Projections by Sub-Programme

Billion Uganda Shillings	**FY2022/23**	**2023/24**	**Medium Term Projections**			
	**Approved Budget**	**Proposed Budget**	**2024/25**	**2025/26**	**2026/27**	**2027/28**
01 Institutional Coordination	205.613	213.552	214.564	231.061	265.342	264.101
02 Civil and Criminal Justice	186.925	200.502	210.351	268.124	380.087	380.778
03 Legal Education, Training and Research	7.481	8.742	8.501	8.705	10.619	11.169
**Total for the Programme**	**400.019**	**422.796**	**433.416**	**507.889**	**656.047**	**656.047**

Table P3.2: Proposed Budget Allocations and Medium Term Projections by Vote

ADMINISTRATION OF JUSTICE

Billion Uganda Shillings	FY2022/23		2023/24	Medium Term Projections			
	Approved Budget	Spent by End Sep	Proposed Budget	2024/25	2025/26	2026/27	2027/28
018 Ministry of Gender, Labour and Social Development		0	0.500	0.510	0.612	0.826	0.826
101 Judiciary (Courts of Judicature)	381.607	63.821	387.541	397.295	465.333	600.574	600.574
133 Directorate of Public Prosecution (DPP)		0	11.843	12.196	14.230	18.098	18.098
144 Uganda Police Force		0	1.000	1.020	1.224	1.652	1.652
145 Uganda Prisons Service		0	1.000	1.000	1.200	1.680	1.680
148 Judicial Service Commission (JSC)	17.568	1.941	19.568	20.016	23.684	31.169	31.169
311 Law Development Centre	0.844	0.131	1.344	1.380	1.606	2.048	2.048
**Total for the Programme**	**400.019**	**65.894**	**422.796**	**433.416**	**507.889**	**656.047**	**656.047**

P4: PROGRAMME PRIORITIES IN LINE WITH INTERVENTIONS FOR FY2023/24

Programme Priorities FY2023/24	NDP III Programme Intervention
a) ECCMIS rolled out to Court Stations of Jinja HC, Jinja CM, Kamuli CM Bugembe G1, Kakira G1 and Kagoma G1;<br><br>b) 9 ECCMIS Change Management sessions provided for 9 ECCMIS Court Stations of Jinja HC, Jinja CM, Kamuli CM Bugembe G1, Kakira G1 and Kagoma G1;<br><br>c) 11 CCTV Cameras installed in Court Stations (Registry of Planning & Development, Fort Portal HC & CM, Mukono HC & CM, Lira HC &CM);<br><br>d) 10 Biometric Machines installed in Court Stations (FortPortal HC & CM, Mukono HC & CM, Lira HC & CM Arua HC & CM, Masaka HC&CM);<br><br>e) 500 Desktop computers procured for 250 Court stations;<br><br>f) 300 laptops procured for new recruited Judicial Officers;<br><br>g) 15 Heavy Duty Photocopiers procured for 15 High Court Stations of Mbarara, Mbale, Jinja, Soroti, Tororo, Moroto, Rukungiri, FortPortal, Masaka, Masindi, Luwero, Lira, Gulu, Iganga and Hoima;<br><br>h) Video conferencing system rolled out to Tororo High Court & the respective Prison;<br><br>i) 5 Video conferencing systems rolled out to Court Hall in each Division (Lands, Civil, ACD, ICD, & JTI);<br><br>[illegible]	Automate and Integrate information management systems

ADMINISTRATION OF JUSTICE

Programme Priorities FY2023/24	NDP III Programme Intervention
j) Subscriptions for LEXISNEXIS for 500 Judicial Officers renewed;<br><br>k) Subscriptions for AFRICAN LII renewed;<br><br>l) Court files digitized for 4 ECCMIS Courts (Commercial Division, Land Division, Civil Division and Mengo CM);<br><br>m) 24 LAN/WAN infrastructure installed in Court Stations (Buhweju, Pallisa, Isingiro, Kiruhura, Kamwenge, Patongo, Bubulo, Budaka, Bugiri, Bullisa, Bundibugyo, Dokolo, Kaberamaido, Kajjansi, Kalangala, Kamuli, Kanungu, Kibale CM, Kira CM);<br><br>n) ISP UTL for 14 Court Stations (Family Division, Nabweru CM, Yumbe CM, Kisoro CM, Iganga CM, Rukungiri CM, Kiboga CM, Kayunga CM, Butambala CM, Lugazi CM, Sironko CM, Kakira G1, Bugembe GI & Kotido CM);<br><br>o) ISP (MTN) procured for 10 Court Stations (Mukono HC, Kabale CM, LDC, Court of Appeal -2nd Link, Kalangala, [illegible]	
p) Mobile Internet procured for 11 Justices of the Supreme Court, 20 Justices of the Court of Appeal, 7 Members of the Contracts Committee, 65 ECCMIS Users - Judicial Officers, 19 for ECCMIS Phase 1 Court Stations & 1 for ICT Department;<br><br>q) Annual ICT audit conducted;<br><br>r) 4 ICT security systems installed and maintained (Antivirus, CISCO Firepower, Mail flitter and Backups);<br><br>s) 8 computers pocured for the Registries in 6 ECCMIS Court Stations of Jinja HCT, Kamuli CM, Bugembe G1, Kakira G1 and Kagoma G1);<br><br>t) Cabled Internet subscribed for 17 Links for Ssembabule CM, Apac CM, Nsangi G1, Kamuli CM, Mayuge G1, Buhweju CM, Pallisa CM, Isingiro CM, Kiruhura CM, Kamwenge CM, Patongo CM, Bubulo CM, Budaka CM, Bugiri CM, Bullisa CM, and Bundibugyo CM;<br><br>u) ICT infrastructure routinely maintained and Serviced including LAN/WAN infrastructure, Judiciary computer hardware, computer printers and photocopier.	Automate and Integrate information management systems

ADMINISTRATION OF JUSTICE

Programme Priorities FY2023/24	NDP III Programme Intervention
a) Construction of Supreme Court and Court of Appeal building completed;<br><br>b) 2 Regional Courts of Appeal buildings constructed (Gulu and Mbarara);<br><br>c) Construction of Soroti, Tororo and Rukungiri High Court buildings completed;<br><br>d) Construction of Alebtong, Budaka and Lyatonde Chief Magistrate Court buildings completed;<br><br>e) Construction of Abim, Patongo and Karenga Magistrate Grade I Court buildings completed;<br><br>f) Moroto, Mpigi, Iganga, Mubende, Luwero and Kitgum High Court buildings constructed;<br><br>g) Kira, Dokolo and Sironko Chief Magistrates buildings constructed;<br><br>h) Omoro, Kaliro, Bududa and Kyazanga Magistrate Grade One Court buildings constructed;<br><br>i) 3 Institutional houses constructed at Nakapiripirit, Moyo, Bukwo & Buhweju;<br><br>j) Payment of contractual obligations for constructions of formerly funded JLOS projects paid (Mukono High Court, Kamwenge CM, Butambala CM, Mayuge CM and 11 Justice Centres at Sembabule, Buhweju, Buyende, Rubirizi, Kole, Nwoya, Sheema, Maracha, Namayingo, Kibaale and Nakaseke);<br><br>k) 40 land titles processed and titled;<br><br>l) Regional JSC Offices in the districts of Masaka, Arua, and Mbarara established and operationalized.	Construct and equip additional Administration of Justice service delivery points

ADMINISTRATION OF JUSTICE

Programme Priorities FY2023/24	NDP III Programme Intervention
a) A mobile Court established in Refugee centres;<br><br>b) 8 Breastfeeding and children's playrooms established;<br><br>c) 55 Judiciary Service staff that have declared living with HIV/AIDS supported financially;<br><br>d) 4 HIV awareness campaigns conducted and HIV/AIDs policy popularized among staff;<br><br>e) Judiciary Anti-Sexual Harassment policy awareness campaigns conducted in Karamoja, West Nile, Tororo and Lango sub-regions.	Implement special programmes that promote equal opportunities to reduce vulnerability

ADMINISTRATION OF JUSTICE

Programme Priorities FY2023/24	NDP III Programme Intervention
120 cases disposed of cases at Supreme Court as follows;<br>a) 45 Criminal cases disposed of;<br>b) 55 Civil cases disposed of;<br>c) 20 Constitutional Applications disposed of;<br><br>1,509 Cases disposed of at Court of Appeal as follows;<br>a) 616 Criminal cases;<br>b) 474 Civil cases disposed of;<br>c) 303 Constitutional cases;<br>d) 16 Taxation Applications disposed of;<br>e) 100 Appellate Mediation Cases disposed of.<br><br>Disposal of cases at High Court Divisions:<br>a) 369 Anti-Corruption cases disposed of at the Anti-Corruption Division;<br>b) 3,322 Civil cases disposed of at the Civil Division<br>c) 2,990 Commercial cases disposed of at the Commercial Division<br>d) 1,514 cases disposed of at Criminal Division;<br>e) 600 cases disposed of through Plea-Bargaining<br>f) 3,949 Family cases disposed of at the Family Division;<br>g) 6,381 Land cases disposed of at the Land Division;<br>h) International Crimes cases disposed of as follows;<br>1) Trial in HCT Uganda versus Kwoyelo Thomas alias Latoni conducted;<br>2) Out-reach conducted in Jinja, Mayuge, Kayunga, Bugiri, Nar and Mbale for the case of Uganda versus Ali Kabambwe & 37 Or Mukulu case)<br>3) Trial in HCT for Uganda Versus Kabambwe & 37 Others (Jamil Mukulu case) conducted;<br>4) Pre-Trial in HCT for Uganda; Mumbere Charles Wesley & 2 (others conducted;<br>5) 48 Pre-trials conducted;<br>6) 24 Trials conducted;<br><br>17,236 cases disposed of at the Court Circuits as follows:<br>a) 7,079 Civil cases disposed of;<br>b) 7,737 Criminal cases disposed of;<br>c) 1,419 Land cases disposed;<br>d) 400 Execution & Bailiffs cases disposed of;<br>e) 601 Family cases disposed.<br><br>216,228 cases disposed of at Magistrate Courts as follows;<br>a) 143,180 Cases disposed of a Chief Magistrates Courts;<br>b) 70,824 Cases disposed of at Magistrates Grade I Courts;<br>c) 2,224 Cases disposed of at Magistrate Grade 2 Courts.	Increase efficiency of Justice delivery Processes
a) Awareness campaigns about the Judiciary and its services conducted in 20 High Court Circuit;	Increase public awareness and advocacy on Justice services.

ADMINISTRATION OF JUSTICE

Programme Priorities FY2023/24	NDP III Programme Intervention
b) Functional information desks at courts established: 6 Divisions, 19 High court Circuits;<br><br>c) 20 Public Awareness campaigns on Mediation conducted;<br><br>d) 20 Public Awareness campaigns on Small Claims Procedure conducted;<br><br>e) National Court Open Day and Justice Week conducted at Judiciary headquarters;<br><br>f) 12 national wide Court Open Day conducted at 12 High Courts (Masaka, Masindi, Luweero, Iganga, Moroto, Lira, Soroti, Kabale, Mukono, Mbale, Gulu and Mbarara);<br><br>g) 3 Information Sharing platforms updated and maintained (Judiciary website, Facebook and Twitter);<br><br>h) 40,800 IEC Materials published;<br><br>i) 36 Radio/TV shows conducted;<br><br>j) 12 Media management/ Campaigns held;<br><br>k) New Law Year ceremony held;<br><br>l) Benedicto Kiwanuka Memorial Lecture held;<br><br>m) Annual Judges' Conference held;<br><br>n) Annual Registrars and Magistrates' Conference held.;<br><br>l) Messages recorded and aired by JSC on various Radios (100) and television (48) on anti-corruption, land justice, money lending, Trial procedure, bail and police bond, children's rights, domestic violence, mandate of JSC, succession and marriage laws to be conducted around the country;<br><br>m) 36 engagements with court users conducted at various court halls about mandate of JSC and administration of justice;<br><br>n) 8 media engagements on access to justice and administration of justice conducted in the respective local areas by JSC;<br><br>o) 1,000 copies of the Citizen's Handbook printed and disseminated;<br><br>p) 5,000 copies IEC Materials on court procedures and initiatives to improve administration of justice printed and disseminated by JSC.	

ADMINISTRATION OF JUSTICE

Programme Priorities FY2023/24	NDP III Programme Intervention
a) Regulations under the Administration of Judiciary Act, 2020 developed, gazzetted, printed and disseminated;<br><br>b) Judges' retreat held on international Crimes Rule and Evidence Procedure held;<br><br>c) 12 Rules Committee meetings held;<br><br>d) Rules Committee retreat held;<br><br>e) Law Reforms Committee retreat held.	Reform rules and procedures
a) 4 High Courts renovated (Kampala, Mbarara, FortPortal and Jinja);<br><br>b) 8 Chief Magistrates Courts renovated (Entebbe, Busia, Nabweru, Kapchorwa, Kabale, Kasese, Kaberamaido and Yumbe);<br><br>c) 8 Magistrates Grade I Courts renovated (Ngora, Amuru, Apala, Aduku, Adjumani, Koboko, Sironko and Amolatar);<br><br>d) 21 standard signage installed at Courts ((Masaka, Mbale, Mbarara, Fortportal, Gulu, Arua, Jinja, Pallisa, Iganga, Kamuli, Dokolo, Soroti, Moroto, Kotido, Kitgum, Buyende, Kamuli, Registry of Planning, Otuke, Apala and Aduku).	Rehabilitate Justice service delivery points
a) 100 vehicles procured for Judicial Officers and supervision (Justices of Supreme Court (3), Justices of Court of Appeal (5), Judges of the High Court (17), Registrars (4), Chief Magistrates (40), Magistrates Grade 1 (20), Inspectorate of Courts (2), 14 seater Mini-bus (2), Supervision (5), specialized van for the Registry of Public Relations and Communications, and specialized van for a Mobile Court in Refugee camps;<br><br>b) 100 motorcycles procured for Process Service for Courts;<br><br>c) 2 boats procured for Courts on islands to facilitate locus-in-quo visits and Process Service for Courts;<br><br>d) 4 Air conditioners procured and installed in 4 High court circuits (Hoima, Tororo, Rukungiri and Soroti);<br><br>e) Water harvesting tanks constructed at 11 Courts (Kabale High Court, Kanungu CM, Gulu High Court, Kitgum CM, Isingiro CM, Apala, Kiruhura, Otuke, Aduku, Masindi and Bundibugyo);<br><br>f) 200 Fire Extinguishers procured for Courts;<br><br>g) Tents procured for the Judicial Training Institute;	Retool institutions in the delivery of Justice

ADMINISTRATION OF JUSTICE

Programme Priorities FY2023/24	NDP III Programme Intervention
[illegible]<br><br>h) 39 containers (40ft) procured for 39 courts to provide Archives space at 12 High Courts and library space at 27 Courts;<br><br>i) 27 tents for waiting area procured for 27 Courts;<br><br>j) 100 desk phones procured for 100 SCP Courts;<br><br>k) 40 desk phones procured for 40 Information desks at Courts;<br><br>l) 50 desktop computers procured for 50 SCP Courts;<br><br>m) 40 desktop computers procured for 40 Information desks;<br><br>n) 2 sets of professional video camera procured;<br><br>o) 2 sets of still professional photo camera procured;<br><br>p) 3 laptop computers procured for Senior Assistant Secretaries;<br><br>q) A luggage scanner procured for High Court;<br><br>r) 50 under carriage security scanners procured for Courts;<br><br>s) 80 walk talkies procured for Courts;<br><br>t) 20 walk though machines procured for Courts.;<br><br>u) 17 solar systems procured and installed at Chief Magistrate Courts (Ibanda, Kiryandongo, Kiruhura, Kasangati, Butambala, Budaka, Patongo, Pader, Wakiso, Mayuge, Nakapiripit, Moroto,	

ADMINISTRATION OF JUSTICE

Programme Priorities FY2023/24	NDP III Programme Intervention
v) Furniture procured for 27 Courts (Amuru, Bushenyi, Mbarara, Yumbe, Moyo, Adjumani, Aduku, Kanoni, Nyimbwa, Goma, Nyanga, Kalangala, Pader, Otuke, Nwoya, Lamwo, Nakaloke, Nakisunga, Kalongo, Atanga, Apac, Kagadi, Bukomero, Kibaale, Buseruka, Kyangwali and High Court Kampala offices);<br><br>w) Furniture procured for 18 Justices and 65 Registrars and Magistrates;<br><br>x) 100 Executive Training chairs procured for JTI;<br><br>y) Furniture procured for 40 information desks and 12 libraries at Courts;<br><br>z) 15 Orthopedic Office chairs and 15 Office Tables/Training Tables procured for JTI;<br><br>aa) 120 file shelves procured for 20 High Courts and Divisions;<br><br>bb) Furniture procured to establish and operationalize Inspectorate of Courts Regional Offices in Mbarara and Gulu;<br><br>cc) 6 double cabin pickups procured to effectively implement the field activities of the JSC;<br><br>dd) New JSC offices partitioned;	Retool institutions in the delivery of Justice
a) Regulations under AJA 2020, developed gazetted, printed and disseminated;<br><br>b) Judiciary ICT policy reviewed;<br><br>c) The Judiciary Service Occupational Health and Safety Policy developed;<br><br>d) Human Resource Open Registry manual developed.	Review and develop relevant legal, regulatory and policy frameworks
a) 100 Appellate Mediation causes disposed of;<br><br>b) 12 Mediation support supervision visits conducted;<br><br>c) 160 Mediators trained and accredited;<br><br>d) 4 Mediation sensitization and awareness campaigns conducted.	Roll out alternative dispute resolution
a) Judiciary Council retreat held;<br><br>b) 3 Judiciary Council meetings held;	Strengthen capacity of duty bearers

ADMINISTRATION OF JUSTICE

Programme Priorities FY2023/24	NDP III Programme Intervention
c) 223 Judicial officers trained on the Law and practice in succession and administration causes;<br><br>d) 240 Magistrates trained in Juvenile Justice;<br><br>e) 30 Justices of appellate courts inducted;<br><br>f) 223 Judicial officers trained in Taxation and Execution of Court orders;<br><br>g) 100 Judicial officers trained in cybercrime and digital electronic evidence;<br><br>h) 118 Court Clerks trained in court interpretation and case management in Kampala Metropolitan;<br><br>i) 20 High Court Judges inducted;<br><br>j) 40 Chief Magistrates inducted;<br><br>k) 70 Magistrates Grade One inducted;<br><br>l) 170 Magistrates trained in Land Justice in Kampala Metropolitan;<br><br>m) 100 staff trained on pre-retirement;<br>n) 948 Judiciary staff trained on the Human Capital Management System in the Central, Eastern, Western and Northern Regions;<br><br>o) 30 Judiciary Senior Managers trained on the Human Capital Management System;<br><br>p) 240 Office Attendants trained in Performance, Basic Office Management and Communication in Central, Western, Northern and North Eastern Regions;<br><br>q) 50 Secretaries trained in Performance, Basic Office Management and Communication in Western Region;<br><br>r) 400 non judicial staff inducted;<br><br>s) 8 Policy and Planning Unit staff trained in programme based management;<br><br>t) The Judiciary Training Calendar developed;<br><br>u) Vital decisions and laws to Judicial Officers compiled and disseminated;	

ADMINISTRATION OF JUSTICE

Programme Priorities FY2023/24	NDP III Programme Intervention
v) The Judiciary Performance Enhancement Tool operationalized;<br><br>w) 4 Joint Monitoring and Evaluation Visits on the Judiciary Strategic Plan V conducted;<br><br>x) 2 Administration of Justice Programme Leadership Committee meetings held;<br><br>y) 4 quarterly Technical Working group meetings held;<br><br>z) 4 quarterly Programme Working Group meeting held;<br><br>aa) Judiciary Performance Report for FY 2022 2023 prepared;<br><br>bb) Court Case File Census conducted;<br><br>cc) Annual financial statement prepared and submitted to [illegible]	
dd) Annual board of survey conducted and a report submitted to MOFPED;<br><br>ee) 238 Court premises maintained;<br><br>ff) 366 Vehicles and 138 motorcycles maintained;<br><br>gg) 26 Judiciary Libraries managed;<br><br>hh) 220 drivers trained in practical defensive skills;<br><br>ii) Professional attire for 88 Judicial Officers and staff uniforms for 200 non judicial staff procured;<br><br>jj) Legal reference materials procured for libraries at Court of Appeal, High Court Divisions and Circuits, Chief Magistrate Courts and Magistrate Grade One Courts;	Strengthen capacity of duty bearers
a) File Conservation & Preservation exercise conducted;<br><br>b) Registry Audit conducted;<br><br>c) 12 High Courts and 16 Registries in Magistrate Courts reorganized.	Strengthen case and records management systems

ADMINISTRATION OF JUSTICE

Programme Priorities FY2023/24	NDP III Programme Intervention
a) Small Claims Procedure (SCP) rolled out to 36 Magistrates Courts;<br><br>b) 32 SCP coaching sessions conducted in 32 Magistrate Courts;<br><br>c) 3 SCP Quarterly Performance review meetings held;<br><br>d) SCP Annual Performance review meeting held;<br><br>e) 40 SCP monitoring and evaluation visits conducted in 40 Magistrate Courts;<br><br>f) 228 Judicial and non-Judicial officers trained in Small Claims Procedure;<br><br>g) SCP case data migrated to CCAS/ ECCMIS in 36 Courts.	Strengthen Courts to resolve disputes in special areas including; land, Commercial, Family disputes, Environment, Standards and Utilities; and Tax disputes
a) 24 Regional Chain linked Committees (RCC) meetings held;<br><br>b) 222 Chain linked Committee meetings held;<br><br>c) 22 Court User Committee meetings held;<br><br>d) Support supervision for 88 Local Council Courts conducted.	Strengthen human resource in the delivery of Justice
a) 400 Bailiffs managed;<br><br>b) 12 Bailiffs Disciplinary Committee Meetings held.	Strengthen implementation of Court decisions.
a) Four On-spot/mobile regional complaints receipt handling conducted;<br><br>b) Investigations of 120 complaints conducted;<br><br>c) One Covert investigation conducted;<br><br>d) 24 Disciplinary Committee meetings held.	Strengthen prevention, detection/investigation and response/ adjudication of corruption cases
State brief provided at all courts adjudicating Criminal cases	Strengthen provision of legal aid services and state brief scheme.

ADMINISTRATION OF JUSTICE

Programme Priorities FY2023/24	NDP III Programme Intervention
a) 2 Regional Inspectorate of Court offices established and operationalized in Mbarara and Gulu;<br><br>b) 20 Field visits conducted by the Chief Inspector of Courts;<br><br>c) 180 Countrywide routine field inspections conducted;<br><br>d) 36 adhoc field inspections conducted;<br><br>e) 36 monitoring field visits on compliance with Service Delivery Standards and the Judiciary Client Charter conducted;<br><br>f) Judiciary Annual Court Inspection Plan developed;<br><br>g) 12 administrative meetings held;<br><br>h) 36 field Monitoring Visits on the Judiciary Anti-Corruption Strategy conducted;<br><br>i) 2 Retreats on monitoring and specialized investigation skills targeting Inspectors, Circuit Registrars and Chief Magistrates conducted;<br><br>j) Judiciary Anti-Corruption Strategy Disseminated;<br><br>k) 4 Integrity Committee meetings conducted;<br><br>l) Judiciary Countrywide Integrity Survey conducted;<br><br>m) 32 Quarterly court inspections conducted by JSC;<br><br>n) 32 radio talk shows on anti-corruption conducted by JSC;<br><br>o) 8 Anti-corruption sensitization workshops conducted by JSC.	Strengthen the inspectorate functions in the Justice systems
a) Court user satisfaction survey conducted;<br><br>b) Research on Innovation and strategies for better delivery of justice conducted.	Undertake Research and Development in improved delivery of Justice

P5: GENDER AND EQUITY ISSUES AND INTERVENTIONS FOR FY2023/24

HIV/AIDS

Issue of Concern: Discrimination and limited access to medicine affect staff living with HIV/AIDs socially and financially at the workplace. This, in turn, affect their performance.

Planned Interventions:

a) Provide financial support to Judiciary Service staff that have declared living with HIV/AIDS

b) Undertake awareness campaigns

ADMINISTRATION OF JUSTICE

GENDER
Issue of Concern: There is need to have gender lenses in the office environment to maximize performance from both men and women
Planned Interventions:
a) Popularization of the Judiciary Service Gender and Equity Policy among staff

ENVIRONMENT
Issue of Concern: Preserving the environment is the key to the overall goal of waning of the effects of the climate change.
Planned Interventions:
a) Equip courts to maintain environment

COVID-19
Issue of Concern: The COVID-19 has greatly affected court operations with some staff losing their lives or their loved ones. Therefore, there is need to test staff and financially support those affected by COVID-19 pandemic.
Planned Interventions:
a) Carry out periodic testing of staff as a preventive measure
b) Provide financial support to the Judiciary Service staff affected by COVID-19

LEGISLATION, OVERSIGHT AND REPRESENTATION

Foreword

The legislature plays a key role in ensuring realization of the NDPIII goal of increasing household income and improving the quality of lives of Ugandans.

The Legislation, Oversight and Representation Programme was developed under the NDPIII framework which aims to strengthen the role of the Legislature in promoting constitutional democracy and good governance in Uganda. The Four (4) key objectives which were selected to deliver this programme
1.Strengthening the institutional capacity of Parliament to independently undertake its constitutional mandates effectively and efficiently,
2.Increasing efficiency and effectiveness in the enactment of legislation for improved democracy and good governance in Uganda,
3.Strengthening transparency, accountability and budget scrutiny for equitable allocation and sustainable development,
4.Strengthening representation and working relationship between Parliament and Local Governments.

In order to effectively implement the desired key NDPIII programme Targets, a number of interventions were identified along with Programme Implementation Action Plans (PIAPS) for all the participating entities in the programme. In this programme, Parliamentary Commission is the lead Agency.

The focus during the development of the programme PIAPs was anchored on the key functions of Parliament namely, legislation, oversight, representation and budget scrutiny and appropriation. In view of the above, the Programme Budget Framework paper for FY 2023/24 and over the medium term captures the costed actions, outputs and outcomes to be delivered during the FY 2023/24 and over the medium term. This is to further submit that the resource requirement for implantation of the costed actions and priorities for Fy2023/34 exceeds the MTEF allocated to the programme for FY 2023/24 as per the First Budget Call Circular.

This is therefore to submit the Programme and the Vote Budget framework Paper for FY2023 and further request that additional resources be provided to enable proper implementation of the planned priorities for FY 2023/24

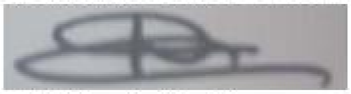

Adolf Mwesige Kasaija

Clerk to Parliament

LEGISLATION, OVERSIGHT AND REPRESENTATION

Abbrevations and Acronyms

ACRONYM	ACRONYM NAME
GE	Gender Equality
PAC	Public Accounts Committee

LEGISLATION, OVERSIGHT AND REPRESENTATION

P1: PROGRAMME OVERVIEW

Snapshot of Medium Term Budget Allocations

Table P1.1 Overview of Programme Expenditure and Medium Term Allocations (Ush Billion)

Billion Uganda Shillings		FY2022/23		FY2023/24	MTEF Budget Projections			
		Approved Budget	Spent by End Sep	Proposed Budget	2024/25	2025/26	2026/27	2027/28
Recurrent	Wage	112.636	27.281	112.525	118.151	129.966	142.963	142.963
	Non Wage	734.932	150.479	739.963	754.763	905.715	1,222.715	1,222.715
Devt.	GoU	67.491	0.000	67.491	67.491	80.989	113.385	113.385
	ExtFin	0.000	0.000	0.000	0.000	0.000	0.000	0.000
	**GoU Total**	**915.059**	**177.759**	**919.979**	**940.405**	**1,116.671**	**1,479.063**	**1,479.063**
**Total GoU+Ext Fin (MTEF)**		**915.059**	**177.759**	**919.979**	**940.405**	**1,116.671**	**1,479.063**	**1,479.063**
	A.I.A	0.000	0.000	0.000	0.000	0.000	0.000	0.000
	**Grand Total**	**915.059**	**177.759**	**919.979**	**940.405**	**1,116.671**	**1,479.063**	**1,479.063**

Programme Strategy and linkage to the National Development Plan

The Legislation, Oversight and Representation (LOR)Programme is one of the 20 National Development (NDP) III developed to contribute to the achievement of the NDIII goal of increasing House hold incomes and improved quality of life of Ugandans. The aim of the LOR Programme is to strengthen the role of the Legislature in promoting constitutional democracy and good governance in Uganda. Its on the basis of the above programme goal that four key results were identified to be achieved over the NDPIII period.

1. Reduced corruption as measured by the Corruption Perception Index from 35% (year 2020) to 26% (year 2025)
2. Improved Constitutional Democracy as measured by the Democracy Index from 4.94 (year 2020) to 6.50 (year 2025)
3. Improved alignment between the annual budgets and the NDP III from 60% to 85%
4. Increased citizens participation in parliamentary business as measured by the citizen scorecard from 50% (year 2021) to 70% (year 2025)

The achievement of the key NDPIII targets will be guided by the strategic plan that spells out the objectives and corresponding interventions over the five year period. The objectives are intended to address development constraints like prevalence of corruption, slow implementation of core development projects which adversely affect growth and job creation, weak implementation of public policies, weak enforcement of laws and regulations for effective governance among others. The objectives of this programme are to:

1. Increase effectiveness and efficiency in the enactment of legislation for improved democracy and good governance.
2. Strengthening transparency, accountability and budget scrutiny for equitable allocation and sustainable development
3. Strengthen representation at local, national, regional and international levels and
4. Strengthen the institutional capacity of Parliament and Local Government Councils to independently undertake their constitutional mandates effectively and efficiently.

The first objective will be attained by improving legislative processes in Parliament and strengthening citizen engagement and participation in Parliamentary business and the following activities will be undertaken

1. Conduct pre and post legislative scrutiny studies
2. Establish a parliamentary consultative framework

LEGISLATION, OVERSIGHT AND REPRESENTATION

2. Establish a parliamentary consultative framework
3. Automate Parliamentary Bill tracking system and Develop a system to track LG Council proceedings over the medium term and
4. Hold committee meetings and plenary sittings to consider the bills and other business brought to Parliament.

The second objective above contributes to achievement of the NDPIII target of reduced corruption and Iimproved alignment between the annual budgets and the NDP III to 85%.To attain the above objective, Parliament will strive to strengthen the oversight role of Parliament over the executive, enhance mechanisms for clearing backlog of constitutional reports, enhance inclusive public participation in Parliamentary oversight, enforce sanctions to MDAs over delayed response to Parliament and strengthen Parliament to effectively play its role in the national budget process for proper implementation of NDPIII. The actions include,
1. Undertaking parliamentary oversight study visits
2. Timely Consideration and disposal of constitutional and statutory reports
3. Holding Committee outreach across the country
4. Build Capacity among Members of Parliaments and staff on Public Finance Management reforms under NDP III
5. Facilitate exposure visits of LG Councilors to Parliament Proceedings and
6. Conducting Assessments of Compliance of the MDAs & LG annual work plans and Budgets to NDPIII programmes.

Strengthening representation at local, national, regional and international levels is another objective to be implemented to achieve the NDPIII programme target of increasing citizens participation in parliamentary business in the short and medium term. This is to be attained through,
1. Attending international and regional Parliamentary engagements
2. Establishing a strong framework for engagement between Parliament, Local Government Councils and the electorate
3. Upgrade and maintain the biometric system to track Members attendance
4. Facilitate MPs consultations with the electorate and
5. Conduct outreach programs for inclusive participation

The fourth objective is intended to deliver on the fourth NDPIII programme target of improving Constitutional Democracy from the measured index of 4.94 in 2020/21 to 6.50. The interventions include developing appropriate infrastructure for legislation, representation, oversight and appropriation, strengthening research, statistical production and evidence use in Parliament and LG councils. The actions to be undertaken:
1. Construct, equip and furnish the new chamber of Parliament over the medium term
2. Undertake retooling of Parliament
3. Conduct research for development
4. Hold sensitization meetings to enhance uptake and use of evidence

P2: Highlights Of Programme Projected Performance

Table P2.1 Programme Outcomes Indicators

**Programme Outcome**	Improved legal framework for implementation of national development priorities					
**Programme Objectives contributed to by the Intermediate Outcome**						
Increase effectiveness and efficiency in the enactment of legislation for improved democracy and good governance.						
	**Performance Targets**					
**Programme Outcome Indicators**	**Base Year**	**Base Line**	**2023/24**	**2024/25**	**2025/26**	**2026/27**
Proportion of laws enacted against planned	2021	90%	95%	100%	100%	100%

LEGISLATION, OVERSIGHT AND REPRESENTATION

**Programme Outcome**	Efficient allocation and utilization of public resources					
**Programme Objectives contributed to by the Intermediate Outcome**						
Strengthen oversight, budget scrutiny and appropriation.						
	**Performance Targets**					
**Programme Outcome Indicators**	**Base Year**	**Base Line**	**2023/24**	**2024/25**	**2025/26**	**2026/27**
Budget alignment to NDP (%)	2018/19	60%	80%	85%	85%	85%
Compliance to gender and equity requirements, %	2018/19	55	90%	97%	97%	97%
Proportion of Parliamentary recommendations on the budget implemented	2018/19	0	75%	80%	80%	80%
**Programme Outcome**	Improved compliance with accountability rules and regulations					
**Programme Objectives contributed to by the Intermediate Outcome**						
Strengthen oversight, budget scrutiny and appropriation.						
	**Performance Targets**					
**Programme Outcome Indicators**	**Base Year**	**Base Line**	**2023/24**	**2024/25**	**2025/26**	**2026/27**
Proportion of Parliamentary recommendations on accountability implemented	2018/19	0	100%	100%	100%	100%
**Programme Outcome**	Improved representation by elected leaders					
**Programme Objectives contributed to by the Intermediate Outcome**						
Strengthen representation at local, regional and international level						
	**Performance Targets**					
**Programme Outcome Indicators**	**Base Year**	**Base Line**	**2023/24**	**2024/25**	**2025/26**	**2026/27**
No. of Parliamentary regional/international bodies[1] with Ugandan representatives	2018/19	12	12	12	12	12
Proportion of representatives aged between 18-35 years in Parliament	2018/19	0	11%	11%	11%	11%
Proportion of women representatives in Parliament	2018/19	35%	%%	34%	34%	35%
**Programme Outcome**	Improved Member participation in Parliamentary and LG Council business					
**Programme Objectives contributed to by the Intermediate Outcome**						
Strengthen representation at local, regional and international level						
	**Performance Targets**					
**Programme Outcome Indicators**	**Base Year**	**Base Line**	**2023/24**	**2024/25**	**2025/26**	**2026/27**
Attendance rates (%) of Parliamentary Committees	2018/19	70%	70%	75%	75%	80%
Attendance rates (%) of Parliamentary sessions by MPs	2018/19	40%	55%	60%	65%	70%

LEGISLATION, OVERSIGHT AND REPRESENTATION

**Programme Outcome**	Effective and efficient transaction of Parliamentary and LG Council business					
**Programme Objectives contributed to by the Intermediate Outcome**						
Strengthen the institutional capacity of Parliament and Local Government Councils to independently undertake their constitutional mandates effectively and efficiently						
	**Performance Targets**					
**Programme Outcome Indicators**	**Base Year**	**Base Line**	**2023/24**	**2024/25**	**2025/26**	**2026/27**
Proportion of parliamentary business processed in time	2018/19	50%	60%	65%	65%	70%
Proportion of Parliamentary processes automated	2018/19	5%	25%	30%	35%	50%

Table P2.2: Intermediate Outcomes Indicators

**Sub-Programme Name:**	**Legislation**					
**Intermediate Outcome Indicators:**	Quality of legislation					
	**Performance Targets**					
**Indicators**	**Base Year**	**Base Line**	**2023/24**	**2024/25**	**2025/26**	**2026/27**
Proportion of legislation challenged in court within six months	2018/19	0%	0%	0%	0%	0%
**Intermediate Outcome Indicators:**	Timely enactment of laws					
	**Performance Targets**					
**Indicators**	**Base Year**	**Base Line**	**2023/24**	**2024/25**	**2025/26**	**2026/27**
Percentage of bills processed by committees within 45 days	60%	50%	%%	60%	65%	65%
**Intermediate Outcome Indicators:**	Improved attendance of Parliamentary and LG Council sessions					
	**Performance Targets**					
**Indicators**	**Base Year**	**Base Line**	**2023/24**	**2024/25**	**2025/26**	**2026/27**
Average attendance of Parliamentary Committee activities	2018/19	16	22	25	25	25
Average attendance of plenary sittings	2018/19	200	330	477	477	500
**Intermediate Outcome Indicators:**	Improved response to citizen's concerns					
	**Performance Targets**					
**Indicators**	**Base Year**	**Base Line**	**2023/24**	**2024/25**	**2025/26**	**2026/27**
Proportion of petitions concluded against submitted	2018/19	40%	70%	70%	75%	80%
Proportion of urgent questions responded to against those raised	2018/19	60%	70%	72%	75%	80%
**Sub-Programme Name:**	**Institutional Capacity**					
**Intermediate Outcome Indicators:**	Improved Parliamentary and LG Council business processes					
	**Performance Targets**					
**Indicators**	**Base Year**	**Base Line**	**2023/24**	**2024/25**	**2025/26**	**2026/27**
Government annual performance score for Parliament	2018/19	100%	100%	100%	100%	100%
LG performance assessment score	2018/19	0%	50%	60%	70%	75%

LEGISLATION, OVERSIGHT AND REPRESENTATION

Sub-Programme Name:	Institutional Capacity					
Intermediate Outcome Indicators:	Improved Parliamentary and LG Council business processes					
	Performance Targets					
Indicators	Base Year	Base Line	2023/24	2024/25	2025/26	2026/27
Percentage of committee business disposed as referred by plenary	2018/19	50%	58%	60%	65%	70%
Proportion of PC targets achieved	2018/19	50%	60%	65%	70%	80%
Proportion of processes automated	2018/19	5%	50%	60%	60%	60%

P3: Medium Term Budget Allocations by Sub-Programme and Vote

Table P3.1: Proposed Budget Allocations and Medium Term Projections by Sub-Programme

Billion Uganda Shillings	FY2022/23	2023/24	Medium Term Projections			
	Approved Budget	Proposed Budget	2024/25	2025/26	2026/27	2027/28
01 Legislation	822.556	827.484	847.910	1,004.626	1,327.615	1,326.615
04 Institutional Capacity	92.503	92.496	92.495	112.045	151.448	152.448
**Total for the Programme**	**915.059**	**919.979**	**940.405**	**1,116.671**	**1,479.063**	**1,479.063**

Table P3.2: Proposed Budget Allocations and Medium Term Projections by Vote

Billion Uganda Shillings	FY2022/23		2023/24	Medium Term Projections			
	Approved Budget	Spent by End Sep	Proposed Budget	2024/25	2025/26	2026/27	2027/28
007 Ministry of Justice and Constitutional Affairs		0	0.100	0.102	0.122	0.165	0.165
011 Ministry of Local Government	0.080	0	0.100	0.102	0.122	0.165	0.165
104 Parliamentary Commission	914.979	177.759	919.779	940.201	1,116.426	1,478.733	1,478.733
**Total for the Programme**	**915.059**	**177.759**	**919.979**	**940.405**	**1,116.671**	**1,479.063**	**1,479.063**

P4: PROGRAMME PRIORITIES IN LINE WITH INTERVENTIONS FOR FY2023/24

Programme Priorities FY2023/24	NDP III Programme Intervention
i) Automate the Parliamentary Bill tracking system (Uganda Bungen) that enables the public to search bills by status, committees and legislators and also make comments while the bill is still in Committees<br>ii) Upgrade the KOHA system for uploading Parliamentary documents to version 21.05 to make it compatible with the integrated library system functionalities for improved service delivery	Develop and upgrade systems essential for fast tracking Parliamentary and LG Council business.

LEGISLATION, OVERSIGHT AND REPRESENTATION

Programme Priorities FY2023/24	NDP III Programme Intervention
i) Develop and install integrated ICT systems<br>ii) Equip and furnish Parliamentary committees for recording, broadcasting and publishing committee proceedings to ease information dissemination and attain inclusive participation in parliamentary business	Develop appropriate infrastructure for legislation, representation, oversight and appropriation.
i) Enhance engagement between Parliament and LGs where inputs from LGs are taken on board by MPs<br>Conduct Parliamentary outreaches to LGs	Establish a strong framework for engagement between Parliament, Local Government Councils and the electorate
i) Conduct Eight (8) pre-and post-legislative scrutiny studies-Research to provide for gender and equity in legislation<br>ii) Support members interested in initiating Private Members Bills<br>iii) Expedite the commenced process of establishing a gender desk in Parliament<br>iv) Fast track works on the new chamber of Parliament to 50% completion to minimize on pressure put on the available physical space within the precincts of Parliament and secure the much needed facilities for PWDs in Parliament and the visiting public<br>v) Ensure adherence to the legislative time table as guided by the Speaker on the flow of Parliamentary Business(where the First meeting after the budget is dedicated for Bills, followed by reports and petitions and eventually the budget process<br>vi) Continuous human capacity building development for Members and Staff of Parliament, to improve on their legislative efficiency and also on gender capacity development skills to foster a uniform approach and understanding of Gender equality and mainstreaming in the Parliament as highlighted in the Gender and Equity Compact for the Legislature 2019/20-2024/25<br>vii) Improve on party whipping system to increase members attendance of Parliamentary activities through strategic engagement with Regional and committee whips to plan for critical activities to be delivered which is informed by the outcome of performance review retreats of Committee leadership<br>viii) Expedite institutionalization of gender equality to facilitate the integration of gender equality issues and concerns in planning, budgeting, implementation and in legislation	Improve legislative processes in Parliament and LG Councils to ensure enhanced scrutiny and quality of legislation.

LEGISLATION, OVERSIGHT AND REPRESENTATION

Programme Priorities FY2023/24	NDP III Programme Intervention
i) Establish a national consultative framework for Parliament on all the bills and other business before Parliament<br>ii) Hold regular outreach programs across all the regions to even cover the marginalized communities<br>iii) Facilitate MPs to undertake consultations with the electorate including the marginalized groups for inclusive legislation<br>iv) Development of a communication strategy within Parliament geared towards increasing public participation in Parliamentary legislative, oversight, budgetar5r and representative processes. provision of timely briefs on key emerging issues, timely provision of public information, education about Parliamentary processes, through the various communication Channels<br>v) Facilitated committees to carry out more consultations, Evaluation of evidence, for use in both Committees and House for evidence based legislation<br>vi) Fast truck regular attendance and Participation of Members in Committees and the House	Strengthen citizen engagement and participation in legislative processes
i) Consider compliance of MDAs & LGs annual work plans and budgets to PFMA<br>ii) Undertake Capacity building of MPs and Staff on PFM reforms under NDP III and other emerging issues<br>iii) Compile Parliamentary recommendations on the budget for follow-up	Strengthen Parliament to effectively play its role in the national budget processes for proper implementation of NDPIII priorities.
i) Automate and maintain systems for data production and development<br>Organise Sensitization meetings to enhance uptake and use of evidence	Strengthen research, statistical production and evidence use in Parliament and LG councils.
i) Undertake parliamentary oversight study visits to gather evidence<br>ii) Establish a clear framework for the Executive to report to Parliament on International engagements.<br>iii) Digitalise and install a tracker for accountability committees for improved committee performance<br>iv) Compile Parliamentary recommendation to form a structure for follow-up on implementation by the Executive<br>v) Enhance Mechanism for clearing Backlog of Constitutional reports and comply the reporting timeframe of six months as per article 163(5) of the Constitution.<br>vi) Ensure Regular reporting by committees on their undertakings to guide the legislative business<br>vii) Support the equal opportunities committee to monitor and promote measures designed to enhance the equalization of opportunities and improvement in the quality of life and status of all people including marginalized groups on the basis of Gender, Age disability etc for purpose of redressing imbalances and report to the House twice in a year.	Strengthen the oversight role of the legislature over the executive.

LEGISLATION, OVERSIGHT AND REPRESENTATION

Programme Priorities FY2023/24	NDP III Programme Intervention
i) Enhance party whipping system and debating process by undertaking training and holding retreats for regional whips and committee leadership<br>ii) Increase sensitization sessions for MPs on Multiparty democracy. The awareness sessions should be extended grassroots to even include the marginalized groups	Strengthen the representative role of MPs.
i) Facilitate training in cross cutting issues including gender and equity , SDGs and Parish Development Model(PDM)and for proper implementation<br>ii) Facilitate Members and Staff with tools necessary to deliver on its mandate under the retooling project	Undertake capacity building and develop systems necessary for optimizing efficiency of Parliament and LG councils.

P5: GENDER AND EQUITY ISSUES AND INTERVENTIONS FOR FY2023/24

The Programme plays an important role to eliminate of all forms of discrimination and inequalities and addressing the different development concerns of the marginalized and vulnerable groups across the country which include women, youth, older persons, ethnic minority and the rural and urban poor in performing its functions of Legislation, Representation, oversight and budget scrutiny and appropriation.
It is also important to note that realization of gender equality and the empowerment of women and girls will make a crucial contribution to progress across all the goals and targets of a country. This is well articulated under the Sustainable Development Goal five(SDG5) to achieve gender equality and empower all women and girls by 2030.The same (SDG5)places a lot of responsibility to Parliament to consider and strengthen sound policies and enforceable legislation for the promotion of gender equality and the empowerment of all women and girls at all levels.
In view of the above, the following gender and equity issues to be addressed during the planning period
Limited appreciation and incorporation of gender and equity statements in strategic plans and programmes of various government agencies
1.Weak institutionalization of a gender focal points within the Parliament and other MDAs to be able to link agencies to the work being done on gender equality and mainstreaming in Uganda
2.Limited capacity for Members and staff of Parliament to be able to address issues and concerns of gender equality and mainstreaming especially during budget scrutiny
4.Limited synergy between MDAs and private entities that have made strides on gender and equality policies and programmes that would provide the necessary data on gender equality and mainstreaming to inform decision making
5.Limited use the Gender and Equity Compacts, the Gender and Equity issues paper for NDP III and other G& E planning frameworks
6.Limited resources to undertake the planned G&E prgrammes because of competing priorities

Interventions
1.Support the creation of sound policy frameworks at national levels based on pro-poor and gender sensitive strategies
2.Improve citizen engagement and participation to increase input by the population, especially the
vulnerable groups, in legislative processes. The planned expenditure is 0.3bn
3.Planned facilitation of training of Members and staff of Parliament in cross cutting issues including gender and equity , SDGs and PDM workshop planned to cost Shs.0.468bn
4.Facilitate the Equal Opportunities and HIVAIDS Committees to Conduct Oversight in hard to reach areas for evidence based oversight
5.Production and dissemination of Gender and Equality (G&E) strategy for Parliament at accost of Shs.0.01bn
6.Undertake Short term Consultancy services to guide parliament in capacity development in G& E main streaming
7.Continuous improvement of the breastfeeding facility that has turned out to be a benchmarking centre by internal and

LEGISLATION, OVERSIGHT AND REPRESENTATION

7.Continuous improvement of the breastfeeding facility that has turned out to be a benchmarking centre by internal and external agencies and persons. The annual expenditure is Shs.0.012bn
8.Hold the annual youth Parliament, regional Parliament outreaches school educational outreaches National School debate This activity draws participants from various regions after regional engagements aimed at preparing the youth for leadership
9.Planned provision of facilities in the Chamber for People With Disability
10.Planned Formation of an internal and external coordination team composed of male and female staff to serve as focal points within the Parliament and other MDAs on gender equality and mainstreaming.
11.Expedite The development of the Gender Strategy for the Parliament
12.Integrate the efforts of entities which are outside the main structures of government for data and information on Gender equality so as to develop comprehensive policies on gender equality and mainstreaming.
13.Encourage continuous use of Gender and Equity compacts, frameworks and reports for continuous improvement in gender and equity mainstreaming agenda.
14.At the onset of her ascendance to Office, the Rt. Hon. Speaker prioritized interventions to uplift and improve the plight of the Girl Child. The Girl Child Initiative will be carried out in one district in each of the original sub-regions of Uganda, including: Acholi, Ankole, Buganda, Bugisu, Bukedi, Bunyoro, Busoga, Karamoja, Kigezi, Lango, Madi, Sebei, Teso, Toro, and West Nile. This is planned to cost UGX1.029bn
15.Carry out continues engagement with development partners, to specifically mobilize resource to support implementation of

ANNEX 1: PROGRAMME ALLOCATIONS FOR FY2023/24 (Excl. Arrears and AIA)

Billion Uganda Shillings	2023/24 Budget		
	GoU	External Fin.	Total Budget
01 AGRO-INDUSTRIALIZATION	694.362	804.967	**1,499.329**
02 MINERAL DEVELOPMENT	38.500	0.000	**38.500**
03 SUSTAINABLE PETROLEUM DEVELOPMENT	539.210	0.000	**539.210**
04 MANUFACTURING	34.785	233.615	**268.400**
05 TOURISM DEVELOPMENT	89.296	0.000	**89.296**
06 NATURAL RESOURCES, ENVIRONMENT, CLIMATE CHANGE, LAND AND WATER	268.716	278.646	**547.362**
07 PRIVATE SECTOR DEVELOPMENT	1,540.125	257.950	**1,798.075**
08 SUSTAINABLE ENERGY DEVELOPMENT	549.410	673.512	**1,222.922**
09 INTEGRATED TRANSPORT INFRASTRUCTURE AND SERVICES	1,501.121	3,155.372	**4,656.493**
10 SUSTAINABLE URBANISATION AND HOUSING	28.999	75.925	**104.924**
11 DIGITAL TRANSFORMATION	96.943	79.818	**176.762**
12 HUMAN CAPITAL DEVELOPMENT	6,941.332	2,064.273	**9,005.605**
13 INNOVATION, TECHNOLOGY DEVELOPMENT AND TRANSFER	52.000	125.568	**177.568**
14 PUBLIC SECTOR TRANSFORMATION	208.167	0.000	**208.167**
15 COMMUNITY MOBILIZATION AND MINDSET CHANGE	21.999	0.000	**21.999**
16 GOVERNANCE AND SECURITY	6,591.272	233.615	**6,824.887**
17 REGIONAL BALANCED DEVELOPMENT	582.158	52.524	**634.682**
18 DEVELOPMENT PLAN IMPLEMENTATION	18,881.724	7.787	**18,889.511**
19 ADMINISTRATION OF JUSTICE	422.796	0.000	**422.796**
20 LEGISLATION, OVERSIGHT AND REPRESENTATION	919.979	0.000	**919.979**
**Grand Total**	**40,002.893**	**8,043.573**	**48,046.466**

ANNEX 2: MEDIUM TERM EXPENDITURE FRAMEWORK (MTEF)2022/23 - 2024/25(Excl. Arrears and AIA)

Billion Uganda Shillings	FY2022/23 Approved Budget						FY2023/24 Budget						FY2024/25 Budget Projections					
PROGRAMME/VOTE	Wage	Non-Wage Recurrent	Domestic Dev	External Financing	Total excl. External Financing	Total incl. External Financing	Wage	Non-Wage Recurrent	Domestic Dev	External Financing	Total excl. External Financing	Total incl. External Financing	Wage	Non-Wage Recurrent	Domestic Dev	External Financing	Total excl. External Financing	Total incl. External Financing
**01 AGRO-INDUSTRIALIZATION**																		
010 Ministry of Agriculture, Animal Industry and Fisheries	19.407	36.433	131.762	376.784	**187.602**	**564.386**	19.407	36.430	139.100	423.260	**194.937**	**618.197**	20.377	37.159	139.100	484.089	**196.636**	**680.725**
011 Ministry of Local Government	0.120	0.167	1.500	47.446	**1.787**	**49.233**	0.120	0.140	0.000	0.000	**0.260**	**0.260**	0.126	0.143	0.000	0.000	**0.269**	**0.269**
015 Ministry of Trade, Industry and Co-operatives	0.000	10.150	0.000	0.000	**10.150**	**10.150**	0.000	9.840	0.000	0.000	**9.840**	**9.840**	0.000	10.037	0.000	0.000	**10.037**	**10.037**
019 Ministry of Water and Environment	0.480	0.035	83.312	121.210	**83.828**	**205.038**	1.248	0.040	83.170	381.707	**84.458**	**466.165**	1.310	0.041	83.170	293.259	**84.521**	**377.780**
021 Ministry of East African Community Affairs	0.000	0.251	0.000	0.000	**0.251**	**0.251**	0.000	0.250	0.000	0.000	**0.250**	**0.250**	0.000	0.255	0.000	0.000	**0.255**	**0.255**
108 National Planning Authority (NPA)	0.000	0.937	0.000	0.000	**0.937**	**0.937**	0.000	0.800	0.000	0.000	**0.800**	**0.800**	0.000	0.816	0.000	0.000	**0.816**	**0.816**
121 Dairy Development Authority (DDA)	4.218	6.468	6.553	0.000	**17.239**	**17.239**	4.218	8.240	0.760	0.000	**13.218**	**13.218**	4.429	8.405	0.760	0.000	**13.594**	**13.594**
122 Kampala Capital City Authority (KCCA)	0.000	0.854	6.334	0.000	**7.188**	**7.188**	0.000	0.350	0.000	0.000	**0.350**	**0.350**	0.000	0.357	0.000	0.000	**0.357**	**0.357**
125 National Animal Genetic Resource Centre and Data Bank (NAGRC&DB)	5.736	5.462	81.971	0.000	**93.169**	**93.169**	5.736	7.860	66.760	0.000	**80.356**	**80.356**	6.023	8.017	66.760	0.000	**80.800**	**80.800**
142 National Agricultural Research Organization (NARO)	38.811	29.397	48.575	0.000	**116.783**	**116.783**	38.811	26.590	35.970	0.000	**101.371**	**101.371**	40.752	27.122	35.970	0.000	**103.843**	**103.843**
152 National Agricultural Advisory Services (NAADS)	2.185	42.643	13.236	0.000	**58.065**	**58.065**	3.309	30.026	0.480	0.000	**33.815**	**33.815**	3.475	30.626	0.480	0.000	**34.581**	**34.581**
154 Uganda National Bureau of Standards (UNBS)	0.000	1.110	0.000	0.000	**1.110**	**1.110**	0.000	0.940	0.000	0.000	**0.940**	**0.940**	0.000	0.959	0.000	0.000	**0.959**	**0.959**
155 Cotton Development Organization	2.013	3.793	1.927	0.000	**7.733**	**7.733**	2.013	3.220	0.300	0.000	**5.533**	**5.533**	2.114	3.284	0.300	0.000	**5.698**	**5.698**
160 Uganda Coffee Development Authority (UCDA)	10.125	50.542	4.273	0.000	**64.940**	**64.940**	10.125	35.360	2.020	0.000	**47.505**	**47.505**	10.631	36.067	2.020	0.000	**48.719**	**48.719**
502 Uganda High Commission in the United Kingdom	0.000	0.863	0.000	0.000	**0.863**	**0.863**	0.000	0.000	0.000	0.000	**0.000**	**0.000**	0.000	0.000	0.000	0.000	**0.000**	**0.000**
504 Uganda High Commission in India, New Delhi	0.000	1.252	0.000	0.000	**1.252**	**1.252**	0.000	0.000	0.000	0.000	**0.000**	**0.000**	0.000	0.000	0.000	0.000	**0.000**	**0.000**
505 Uganda High Commission in Kenya, Nairobi	0.000	0.169	0.000	0.000	**0.169**	**0.169**	0.000	0.000	0.000	0.000	**0.000**	**0.000**	0.000	0.000	0.000	0.000	**0.000**	**0.000**

ANNEX 2: MEDIUM TERM EXPENDITURE FRAMEWORK (MTEF)2022/23 - 2024/25(Excl. Arrears and AIA)

Billion Uganda Shillings	FY2022/23 Approved Budget						FY2023/24 Budget						FY2024/25 Budget Projections					
PROGRAMME/VOTE	Wage	Non-Wage Recurrent	Domestic Dev	External Financing	Total excl. External Financing	Total incl. External Financing	Wage	Non-Wage Recurrent	Domestic Dev	External Financing	Total excl. External Financing	Total incl. External Financing	Wage	Non-Wage Recurrent	Domestic Dev	External Financing	Total excl. External Financing	Total incl. External Financing
**01 AGRO-INDUSTRIALIZATION**																		
507 Uganda High Commission in Nigeria, Abuja	0.000	0.147	0.000	0.000	0.147	0.147	0.000	0.000	0.000	0.000	0.000	0.000	0.000	0.000	0.000	0.000	0.000	0.000
508 Uganda High Commission in South Africa, Pretoria	0.000	0.054	0.000	0.000	0.054	0.054	0.000	0.000	0.000	0.000	0.000	0.000	0.000	0.000	0.000	0.000	0.000	0.000
511 Uganda Embassy in Egypt, Cairo	0.000	0.316	0.000	0.000	0.316	0.316	0.000	0.000	0.000	0.000	0.000	0.000	0.000	0.000	0.000	0.000	0.000	0.000
512 Uganda Embassy in Ethiopia, Addis Ababa	0.000	0.195	0.000	0.000	0.195	0.195	0.000	0.000	0.000	0.000	0.000	0.000	0.000	0.000	0.000	0.000	0.000	0.000
513 Uganda Embassy in China, Beijing	0.000	0.465	0.000	0.000	0.465	0.465	0.000	0.000	0.000	0.000	0.000	0.000	0.000	0.000	0.000	0.000	0.000	0.000
514 Uganda Embassy in Switzerland, Geneva	0.000	0.378	0.000	0.000	0.378	0.378	0.000	0.000	0.000	0.000	0.000	0.000	0.000	0.000	0.000	0.000	0.000	0.000
515 Uganda Embassy in Japan, Tokyo	0.000	0.036	0.000	0.000	0.036	0.036	0.000	0.000	0.000	0.000	0.000	0.000	0.000	0.000	0.000	0.000	0.000	0.000
517 Uganda Embassy in Denmark, Copenhagen	0.000	0.132	0.000	0.000	0.132	0.132	0.000	0.000	0.000	0.000	0.000	0.000	0.000	0.000	0.000	0.000	0.000	0.000
518 Uganda Embassy in Belgium, Brussels	0.000	0.600	0.000	0.000	0.600	0.600	0.000	0.000	0.000	0.000	0.000	0.000	0.000	0.000	0.000	0.000	0.000	0.000
519 Uganda Embassy in Italy, Rome	0.000	0.068	0.000	0.000	0.068	0.068	0.000	0.000	0.000	0.000	0.000	0.000	0.000	0.000	0.000	0.000	0.000	0.000
520 Uganda Embassy in DRC, Kinshasa	0.000	0.167	0.000	0.000	0.167	0.167	0.000	0.000	0.000	0.000	0.000	0.000	0.000	0.000	0.000	0.000	0.000	0.000
523 Uganda Embassy in Germany, Berlin	0.000	0.085	0.000	0.000	0.085	0.085	0.000	0.000	0.000	0.000	0.000	0.000	0.000	0.000	0.000	0.000	0.000	0.000
524 Uganda Embassy in Iran, Tehran	0.000	0.144	0.000	0.000	0.144	0.144	0.000	0.000	0.000	0.000	0.000	0.000	0.000	0.000	0.000	0.000	0.000	0.000
525 Uganda Embassy in Russia, Moscow	0.000	0.258	0.000	0.000	0.258	0.258	0.000	0.000	0.000	0.000	0.000	0.000	0.000	0.000	0.000	0.000	0.000	0.000
526 Uganda Embassy in Australia, Canberra	0.000	0.070	0.000	0.000	0.070	0.070	0.000	0.000	0.000	0.000	0.000	0.000	0.000	0.000	0.000	0.000	0.000	0.000
527 Uganda Embassy in South Sudan, Juba	0.000	0.350	0.000	0.000	0.350	0.350	0.000	0.000	0.000	0.000	0.000	0.000	0.000	0.000	0.000	0.000	0.000	0.000
528 Uganda Embassy in United Arab Emirates, Abudhabi	0.000	0.900	0.000	0.000	0.900	0.900	0.000	0.000	0.000	0.000	0.000	0.000	0.000	0.000	0.000	0.000	0.000	0.000
529 Uganda Embassy in Burundi, Bujumbura	0.000	0.039	0.000	0.000	0.039	0.039	0.000	0.000	0.000	0.000	0.000	0.000	0.000	0.000	0.000	0.000	0.000	0.000
530 Uganda Consulate in China, Guangzhou	0.000	0.061	0.000	0.000	0.061	0.061	0.000	0.000	0.000	0.000	0.000	0.000	0.000	0.000	0.000	0.000	0.000	0.000
531 Uganda Embassy in Turkey, Ankara	0.000	0.205	0.000	0.000	0.205	0.205	0.000	0.000	0.000	0.000	0.000	0.000	0.000	0.000	0.000	0.000	0.000	0.000
534 Uganda Consulate in Kenya, Mombasa	0.000	0.530	0.000	0.000	0.530	0.530	0.000	0.000	0.000	0.000	0.000	0.000	0.000	0.000	0.000	0.000	0.000	0.000

ANNEX 2: MEDIUM TERM EXPENDITURE FRAMEWORK (MTEF)2022/23 - 2024/25(Excl. Arrears and AIA)

Billion Uganda Shillings	FY2022/23 Approved Budget						FY2023/24 Budget						FY2024/25 Budget Projections					
PROGRAMME/VOTE	Wage	Non-Wage Recurrent	Domestic Dev	External Financing	Total excl. External Financing	Total incl. External Financing	Wage	Non-Wage Recurrent	Domestic Dev	External Financing	Total excl. External Financing	Total incl. External Financing	Wage	Non-Wage Recurrent	Domestic Dev	External Financing	Total excl. External Financing	Total incl. External Financing
**01 AGRO-INDUSTRIALIZATION**																		
535 Uganda Embassy in Algeria, Algiers	0.000	0.282	0.000	0.000	**0.282**	**0.282**	0.000	0.000	0.000	0.000	**0.000**	**0.000**	0.000	0.000	0.000	0.000	**0.000**	**0.000**
536 Uganda Embassy in Qatar, Doha	0.000	0.330	0.000	0.000	**0.330**	**0.330**	0.000	0.000	0.000	0.000	**0.000**	**0.000**	0.000	0.000	0.000	0.000	**0.000**	**0.000**
601 Local Governments 01	120.729	44.268	80.496	0.000	**245.493**	**245.493**	120.729	0.000	0.000	0.000	**120.729**	**120.729**	126.765	0.000	0.000	0.000	**126.765**	**126.765**
**Sub Total For: AGRO-INDUSTRIALIZATION**	203.824	240.606	459.940	545.440	**904.370**	**1,449.811**	205.716	160.086	328.560	804.967	**694.362**	**1,499.329**	216.002	163.287	328.560	777.348	**707.849**	**1,485.197**
**02 MINERAL DEVELOPMENT**																		
017 Ministry of Energy and Mineral Development	0.000	5.056	17.420	7.375	**22.476**	**29.851**	5.500	11.000	22.000	0.000	**38.500**	**38.500**	5.775	11.220	22.000	0.000	**38.995**	**38.995**
108 National Planning Authority (NPA)	0.000	1.885	0.000	0.000	**1.885**	**1.885**	0.000	0.000	0.000	0.000	**0.000**	**0.000**	0.000	0.000	0.000	0.000	**0.000**	**0.000**
530 Uganda Consulate in China, Guangzhou	0.000	0.050	0.000	0.000	**0.050**	**0.050**	0.000	0.000	0.000	0.000	**0.000**	**0.000**	0.000	0.000	0.000	0.000	**0.000**	**0.000**
**Sub Total For: MINERAL DEVELOPMENT**	0.000	6.991	17.420	7.375	**24.411**	**31.786**	5.500	11.000	22.000	0.000	**38.500**	**38.500**	5.775	11.220	22.000	0.000	**38.995**	**38.995**
**03 SUSTAINABLE PETROLEUM DEVELOPMENT**																		
006 Ministry of Foreign Affairs	0.000	0.000	0.000	0.000	**0.000**	**0.000**	0.000	0.695	0.000	0.000	**0.695**	**0.695**	0.000	0.709	0.000	0.000	**0.709**	**0.709**
008 Ministry of Finance, Planning and Economic Development	0.000	720.350	0.000	0.000	**720.350**	**720.350**	0.000	281.350	0.000	0.000	**281.350**	**281.350**	0.000	286.977	0.000	0.000	**286.977**	**286.977**
013 Ministry of Education and Sports	0.000	0.000	0.000	0.000	**0.000**	**0.000**	0.000	1.000	0.000	0.000	**1.000**	**1.000**	0.000	1.020	0.000	0.000	**1.020**	**1.020**
017 Ministry of Energy and Mineral Development	0.000	1.920	83.020	0.000	**84.940**	**84.940**	3.700	29.985	126.360	0.000	**160.045**	**160.045**	3.885	30.585	126.360	596.135	**160.830**	**756.965**
139 Petroleum Authority of Uganda (PAU)	28.718	21.350	13.653	0.000	**63.720**	**63.720**	28.718	37.705	27.992	0.000	**94.415**	**94.415**	30.154	38.459	27.992	0.000	**96.605**	**96.605**
150 National Environment Management Authority (NEMA)	0.000	0.000	0.000	0.000	**0.000**	**0.000**	0.000	0.500	0.000	0.000	**0.500**	**0.500**	0.000	0.510	0.000	0.000	**0.510**	**0.510**
154 Uganda National Bureau of Standards (UNBS)	0.000	0.000	0.000	0.000	**0.000**	**0.000**	0.000	0.900	0.000	0.000	**0.900**	**0.900**	0.000	0.918	0.000	0.000	**0.918**	**0.918**
506 Uganda High Commission in Tanzania, Dar es Salaam	0.000	0.305	0.000	0.000	**0.305**	**0.305**	0.000	0.305	0.000	0.000	**0.305**	**0.305**	0.000	0.305	0.000	0.000	**0.305**	**0.305**

ANNEX 2: MEDIUM TERM EXPENDITURE FRAMEWORK (MTEF)2022/23 - 2024/25(Excl. Arrears and AIA)

Billion Uganda Shillings	FY2022/23 Approved Budget						FY2023/24 Budget						FY2024/25 Budget Projections					
PROGRAMME/VOTE	Wage	Non-Wage Recurrent	Domestic Dev	External Financing	Total excl. External Financing	Total incl. External Financing	Wage	Non-Wage Recurrent	Domestic Dev	External Financing	Total excl. External Financing	Total incl. External Financing	Wage	Non-Wage Recurrent	Domestic Dev	External Financing	Total excl. External Financing	Total incl. External Financing
**Sub Total For: SUSTAINABLE PETROLEUM DEVELOPMENT**	28.718	743.925	96.673	0.000	**869.316**	**869.316**	32.418	352.440	154.352	0.000	**539.210**	**539.210**	34.039	359.483	154.352	596.135	**547.874**	**1,144.008**
**04 MANUFACTURING**																		
006 Ministry of Foreign Affairs	0.000	0.265	0.000	0.000	**0.265**	**0.265**	0.000	0.000	0.000	0.000	**0.000**	**0.000**	0.000	0.000	0.000	0.000	**0.000**	**0.000**
015 Ministry of Trade, Industry and Co-operatives	2.201	99.731	23.055	0.000	**124.987**	**124.987**	2.026	32.445	0.226	0.000	**34.697**	**34.697**	2.127	33.094	0.226	0.000	**35.447**	**35.447**
138 Uganda Investment Authority (UIA)	0.000	0.000	66.360	221.251	**66.360**	**287.611**	0.000	0.000	0.000	233.615	**0.000**	**233.615**	0.000	0.000	0.000	0.000	**0.000**	**0.000**
154 Uganda National Bureau of Standards (UNBS)	0.000	4.234	0.000	0.000	**4.234**	**4.234**	0.000	0.088	0.000	0.000	**0.088**	**0.088**	0.000	0.090	0.000	0.000	**0.090**	**0.090**
506 Uganda High Commission in Tanzania, Dar es Salaam	0.000	0.338	0.000	0.000	**0.338**	**0.338**	0.000	0.000	0.000	0.000	**0.000**	**0.000**	0.000	0.000	0.000	0.000	**0.000**	**0.000**
508 Uganda High Commission in South Africa, Pretoria	0.000	0.079	0.000	0.000	**0.079**	**0.079**	0.000	0.000	0.000	0.000	**0.000**	**0.000**	0.000	0.000	0.000	0.000	**0.000**	**0.000**
515 Uganda Embassy in Japan, Tokyo	0.000	0.164	0.000	0.000	**0.164**	**0.164**	0.000	0.000	0.000	0.000	**0.000**	**0.000**	0.000	0.000	0.000	0.000	**0.000**	**0.000**
516 Uganda Embassy in Saudi Arabia, Riyadh	0.000	0.134	0.000	0.000	**0.134**	**0.134**	0.000	0.000	0.000	0.000	**0.000**	**0.000**	0.000	0.000	0.000	0.000	**0.000**	**0.000**
518 Uganda Embassy in Belgium, Brussels	0.000	0.165	0.000	0.000	**0.165**	**0.165**	0.000	0.000	0.000	0.000	**0.000**	**0.000**	0.000	0.000	0.000	0.000	**0.000**	**0.000**
519 Uganda Embassy in Italy, Rome	0.000	0.063	0.000	0.000	**0.063**	**0.063**	0.000	0.000	0.000	0.000	**0.000**	**0.000**	0.000	0.000	0.000	0.000	**0.000**	**0.000**
520 Uganda Embassy in DRC, Kinshasa	0.000	0.230	0.000	0.000	**0.230**	**0.230**	0.000	0.000	0.000	0.000	**0.000**	**0.000**	0.000	0.000	0.000	0.000	**0.000**	**0.000**
525 Uganda Embassy in Russia, Moscow	0.000	0.187	0.000	0.000	**0.187**	**0.187**	0.000	0.000	0.000	0.000	**0.000**	**0.000**	0.000	0.000	0.000	0.000	**0.000**	**0.000**
527 Uganda Embassy in South Sudan, Juba	0.000	0.200	0.000	0.000	**0.200**	**0.200**	0.000	0.000	0.000	0.000	**0.000**	**0.000**	0.000	0.000	0.000	0.000	**0.000**	**0.000**
529 Uganda Embassy in Burundi, Bujumbura	0.000	0.025	0.000	0.000	**0.025**	**0.025**	0.000	0.000	0.000	0.000	**0.000**	**0.000**	0.000	0.000	0.000	0.000	**0.000**	**0.000**
530 Uganda Consulate in China, Guangzhou	0.000	0.139	0.000	0.000	**0.139**	**0.139**	0.000	0.000	0.000	0.000	**0.000**	**0.000**	0.000	0.000	0.000	0.000	**0.000**	**0.000**
532 Uganda Embassy in Somalia, Mogadishu	0.000	0.050	0.000	0.000	**0.050**	**0.050**	0.000	0.000	0.000	0.000	**0.000**	**0.000**	0.000	0.000	0.000	0.000	**0.000**	**0.000**
533 Uganda Embassy in Malaysia, Kuala Lumpur	0.000	0.050	0.000	0.000	**0.050**	**0.050**	0.000	0.000	0.000	0.000	**0.000**	**0.000**	0.000	0.000	0.000	0.000	**0.000**	**0.000**
**Sub Total For: MANUFACTURING**	2.201	106.054	89.415	221.251	**197.670**	**418.920**	2.026	32.533	0.226	233.615	**34.785**	**268.400**	2.127	33.184	0.226	0.000	**35.537**	**35.537**

ANNEX 2: MEDIUM TERM EXPENDITURE FRAMEWORK (MTEF)2022/23 - 2024/25(Excl. Arrears and AIA)

Billion Uganda Shillings	FY2022/23 Approved Budget						FY2023/24 Budget						FY2024/25 Budget Projections					
PROGRAMME/VOTE	Wage	Non-Wage Recurrent	Domestic Dev	External Financing	Total excl. External Financing	Total incl. External Financing	Wage	Non-Wage Recurrent	Domestic Dev	External Financing	Total excl. External Financing	Total incl. External Financing	Wage	Non-Wage Recurrent	Domestic Dev	External Financing	Total excl. External Financing	Total incl. External Financing
**05 TOURISM DEVELOPMENT**																		
022 Ministry of Tourism, Wildlife and Antiquities	2.371	151.926	11.847	0.000	**166.144**	**166.144**	2.371	29.980	52.640	0.000	**84.991**	**84.991**	2.489	30.580	52.640	0.000	**85.709**	**85.709**
117 Uganda Tourism Board (UTB)	1.855	21.893	0.093	0.000	**23.841**	**23.841**	1.855	2.350	0.100	0.000	**4.305**	**4.305**	1.948	2.397	0.100	0.000	**4.445**	**4.445**
122 Kampala Capital City Authority (KCCA)	0.000	0.091	0.000	0.000	**0.091**	**0.091**	0.000	0.000	0.000	0.000	**0.000**	**0.000**	0.000	0.000	0.000	0.000	**0.000**	**0.000**
501 Uganda Mission at the United Nations, New York	0.000	0.380	0.000	0.000	**0.380**	**0.380**	0.000	0.000	0.000	0.000	**0.000**	**0.000**	0.000	0.000	0.000	0.000	**0.000**	**0.000**
505 Uganda High Commission in Kenya, Nairobi	0.000	0.072	0.000	0.000	**0.072**	**0.072**	0.000	0.000	0.000	0.000	**0.000**	**0.000**	0.000	0.000	0.000	0.000	**0.000**	**0.000**
508 Uganda High Commission in South Africa, Pretoria	0.000	0.054	0.000	0.000	**0.054**	**0.054**	0.000	0.000	0.000	0.000	**0.000**	**0.000**	0.000	0.000	0.000	0.000	**0.000**	**0.000**
509 Uganda High Commission in Rwanda, Kigali	0.000	0.042	0.000	0.000	**0.042**	**0.042**	0.000	0.000	0.000	0.000	**0.000**	**0.000**	0.000	0.000	0.000	0.000	**0.000**	**0.000**
513 Uganda Embassy in China, Beijing	0.000	0.366	0.000	0.000	**0.366**	**0.366**	0.000	0.000	0.000	0.000	**0.000**	**0.000**	0.000	0.000	0.000	0.000	**0.000**	**0.000**
515 Uganda Embassy in Japan, Tokyo	0.000	0.221	0.000	0.000	**0.221**	**0.221**	0.000	0.000	0.000	0.000	**0.000**	**0.000**	0.000	0.000	0.000	0.000	**0.000**	**0.000**
516 Uganda Embassy in Saudi Arabia, Riyadh	0.000	0.183	0.000	0.000	**0.183**	**0.183**	0.000	0.000	0.000	0.000	**0.000**	**0.000**	0.000	0.000	0.000	0.000	**0.000**	**0.000**
517 Uganda Embassy in Denmark, Copenhagen	0.000	0.200	0.000	0.000	**0.200**	**0.200**	0.000	0.000	0.000	0.000	**0.000**	**0.000**	0.000	0.000	0.000	0.000	**0.000**	**0.000**
518 Uganda Embassy in Belgium, Brussels	0.000	0.170	0.000	0.000	**0.170**	**0.170**	0.000	0.000	0.000	0.000	**0.000**	**0.000**	0.000	0.000	0.000	0.000	**0.000**	**0.000**
519 Uganda Embassy in Italy, Rome	0.000	0.051	0.000	0.000	**0.051**	**0.051**	0.000	0.000	0.000	0.000	**0.000**	**0.000**	0.000	0.000	0.000	0.000	**0.000**	**0.000**
522 Uganda Embassy in France, Paris	0.000	0.084	0.000	0.000	**0.084**	**0.084**	0.000	0.000	0.000	0.000	**0.000**	**0.000**	0.000	0.000	0.000	0.000	**0.000**	**0.000**
523 Uganda Embassy in Germany, Berlin	0.000	0.085	0.000	0.000	**0.085**	**0.085**	0.000	0.000	0.000	0.000	**0.000**	**0.000**	0.000	0.000	0.000	0.000	**0.000**	**0.000**
524 Uganda Embassy in Iran, Tehran	0.000	0.239	0.000	0.000	**0.239**	**0.239**	0.000	0.000	0.000	0.000	**0.000**	**0.000**	0.000	0.000	0.000	0.000	**0.000**	**0.000**
525 Uganda Embassy in Russia, Moscow	0.000	1.051	0.000	0.000	**1.051**	**1.051**	0.000	0.000	0.000	0.000	**0.000**	**0.000**	0.000	0.000	0.000	0.000	**0.000**	**0.000**
526 Uganda Embassy in Australia, Canberra	0.000	0.459	0.000	0.000	**0.459**	**0.459**	0.000	0.000	0.000	0.000	**0.000**	**0.000**	0.000	0.000	0.000	0.000	**0.000**	**0.000**
527 Uganda Embassy in South Sudan, Juba	0.000	0.300	0.000	0.000	**0.300**	**0.300**	0.000	0.000	0.000	0.000	**0.000**	**0.000**	0.000	0.000	0.000	0.000	**0.000**	**0.000**
529 Uganda Embassy in Burundi, Bujumbura	0.000	0.027	0.000	0.000	**0.027**	**0.027**	0.000	0.000	0.000	0.000	**0.000**	**0.000**	0.000	0.000	0.000	0.000	**0.000**	**0.000**

ANNEX 2: MEDIUM TERM EXPENDITURE FRAMEWORK (MTEF)2022/23 - 2024/25(Excl. Arrears and AIA)

Billion Uganda Shillings	FY2022/23 Approved Budget						FY2023/24 Budget						FY2024/25 Budget Projections					
PROGRAMME/VOTE	Wage	Non-Wage Recurrent	Domestic Dev	External Financing	Total excl. External Financing	Total incl. External Financing	Wage	Non-Wage Recurrent	Domestic Dev	External Financing	Total excl. External Financing	Total incl. External Financing	Wage	Non-Wage Recurrent	Domestic Dev	External Financing	Total excl. External Financing	Total incl. External Financing
**05 TOURISM DEVELOPMENT**																		
530 Uganda Consulate in China, Guangzhou	0.000	0.061	0.000	0.000	**0.061**	**0.061**	0.000	0.000	0.000	0.000	**0.000**	**0.000**	0.000	0.000	0.000	0.000	**0.000**	**0.000**
531 Uganda Embassy in Turkey, Ankara	0.000	0.315	0.000	0.000	**0.315**	**0.315**	0.000	0.000	0.000	0.000	**0.000**	**0.000**	0.000	0.000	0.000	0.000	**0.000**	**0.000**
533 Uganda Embassy in Malaysia, Kuala Lumpur	0.000	0.043	0.000	0.000	**0.043**	**0.043**	0.000	0.000	0.000	0.000	**0.000**	**0.000**	0.000	0.000	0.000	0.000	**0.000**	**0.000**
534 Uganda Consulate in Kenya, Mombasa	0.000	0.200	0.000	0.000	**0.200**	**0.200**	0.000	0.000	0.000	0.000	**0.000**	**0.000**	0.000	0.000	0.000	0.000	**0.000**	**0.000**
**Sub Total For: TOURISM DEVELOPMENT**	4.226	178.511	11.940	0.000	**194.677**	**194.677**	4.226	32.330	52.740	0.000	**89.296**	**89.296**	4.438	32.977	52.740	0.000	**90.154**	**90.154**
**06 NATURAL RESOURCES, ENVIRONMENT, CLIMATE CHANGE, LAND AND WATER**																		
003 Office of the Prime Minister	0.337	9.660	10.808	0.000	**20.805**	**20.805**	0.337	7.680	9.730	0.000	**17.747**	**17.747**	0.354	7.834	9.730	0.000	**17.918**	**17.918**
011 Ministry of Local Government	0.000	0.000	0.300	17.406	**0.300**	**17.706**	0.000	0.000	0.000	0.000	**0.000**	**0.000**	0.000	0.000	0.000	0.000	**0.000**	**0.000**
012 Ministry of Lands, Housing & Urban Development	8.706	10.093	12.670	92.190	**31.469**	**123.659**	8.706	8.580	9.780	70.377	**27.066**	**97.442**	9.141	8.752	9.780	0.000	**27.673**	**27.673**
019 Ministry of Water and Environment	10.935	12.978	60.209	175.805	**84.121**	**259.927**	10.160	15.590	85.220	208.269	**110.970**	**319.239**	10.668	15.902	85.220	171.816	**111.790**	**283.606**
108 National Planning Authority (NPA)	0.000	0.097	0.000	0.000	**0.097**	**0.097**	0.000	0.000	0.000	0.000	**0.000**	**0.000**	0.000	0.000	0.000	0.000	**0.000**	**0.000**
109 Uganda National Meteorological Authority (UNMA)	7.413	3.686	6.557	0.000	**17.656**	**17.656**	7.413	3.670	6.060	0.000	**17.143**	**17.143**	7.784	3.743	6.060	0.000	**17.587**	**17.587**
122 Kampala Capital City Authority (KCCA)	0.000	20.211	0.345	0.000	**20.556**	**20.556**	0.000	17.880	0.340	0.000	**18.220**	**18.220**	0.000	18.238	0.340	0.000	**18.578**	**18.578**
150 National Environment Management Authority (NEMA)	6.722	8.971	3.250	0.000	**18.943**	**18.943**	6.722	9.030	4.020	0.000	**19.772**	**19.772**	7.058	9.211	4.020	0.000	**20.289**	**20.289**
156 Uganda Land Commission (ULC)	1.062	0.935	29.666	0.000	**31.663**	**31.663**	1.062	1.220	25.600	0.000	**27.882**	**27.882**	1.116	1.244	25.600	0.000	**27.960**	**27.960**
157 National Forestry Authority (NFA)	8.266	15.324	5.652	0.000	**29.242**	**29.242**	8.266	12.500	5.650	0.000	**26.416**	**26.416**	8.679	12.750	5.650	0.000	**27.079**	**27.079**
505 Uganda High Commission in Kenya, Nairobi	0.000	0.050	0.000	0.000	**0.050**	**0.050**	0.000	0.000	0.000	0.000	**0.000**	**0.000**	0.000	0.000	0.000	0.000	**0.000**	**0.000**
606 Local Governments 06	0.000	15.500	79.337	0.000	**94.837**	**94.837**	0.000	3.500	0.000	0.000	**3.500**	**3.500**	0.000	3.570	0.000	0.000	**3.570**	**3.570**
**Sub Total For: NATURAL RESOURCES, ENVIRONMENT, CLIMATE CHANGE, LAND AND WATER**	43.441	97.505	208.794	285.402	**349.740**	**635.142**	42.666	79.650	146.400	278.646	**268.716**	**547.362**	44.799	81.243	146.400	171.816	**272.442**	**444.259**

ANNEX 2: MEDIUM TERM EXPENDITURE FRAMEWORK (MTEF)2022/23 - 2024/25(Excl. Arrears and AIA)

Billion Uganda Shillings	FY2022/23 Approved Budget						FY2023/24 Budget						FY2024/25 Budget Projections					
PROGRAMME/VOTE	Wage	Non-Wage Recurrent	Domestic Dev	External Financing	Total excl. External Financing	Total incl. External Financing	Wage	Non-Wage Recurrent	Domestic Dev	External Financing	Total excl. External Financing	Total incl. External Financing	Wage	Non-Wage Recurrent	Domestic Dev	External Financing	Total excl. External Financing	Total incl. External Financing
**07 PRIVATE SECTOR DEVELOPMENT**																		
008 Ministry of Finance, Planning and Economic Development	0.301	1,431.523	7.102	71.352	**1,438.925**	**1,510.277**	0.301	1,414.946	2.236	257.950	**1,417.483**	**1,675.433**	0.316	1,443.245	2.236	438.098	**1,445.797**	**1,883.895**
015 Ministry of Trade, Industry and Co-operatives	0.973	0.994	0.000	0.000	**1.967**	**1.967**	1.148	0.994	0.000	0.000	**2.142**	**2.142**	1.206	1.014	0.000	0.000	**2.220**	**2.220**
021 Ministry of East African Community Affairs	0.000	1.731	0.000	0.000	**1.731**	**1.731**	0.000	1.731	0.000	0.000	**1.731**	**1.731**	0.000	1.766	0.000	0.000	**1.766**	**1.766**
108 National Planning Authority (NPA)	0.000	0.250	0.000	0.000	**0.250**	**0.250**	0.000	0.250	0.000	0.000	**0.250**	**0.250**	0.000	0.255	0.000	0.000	**0.255**	**0.255**
119 Uganda Registration Services Bureau (URSB)	4.349	2.325	0.000	0.000	**6.673**	**6.673**	4.349	2.325	0.267	0.000	**6.940**	**6.940**	4.566	2.371	0.267	0.000	**7.204**	**7.204**
123 National Lotteries and Gaming Regulatory Board	0.000	0.490	0.000	0.000	**0.490**	**0.490**	0.000	0.490	0.000	0.000	**0.490**	**0.490**	0.000	0.499	0.000	0.000	**0.499**	**0.499**
136 Uganda Export Promotion Board (UEPB)	2.561	5.833	0.034	0.000	**8.428**	**8.428**	2.561	5.833	0.037	0.000	**8.432**	**8.432**	2.689	5.950	0.037	0.000	**8.676**	**8.676**
138 Uganda Investment Authority (UIA)	6.833	8.226	2.344	0.000	**17.403**	**17.403**	6.833	8.226	1.404	0.000	**16.463**	**16.463**	7.174	8.391	1.404	0.000	**16.969**	**16.969**
140 Capital Markets Authority	3.475	5.095	0.000	0.000	**8.570**	**8.570**	3.475	5.095	0.000	0.000	**8.570**	**8.570**	3.648	5.197	0.000	0.000	**8.846**	**8.846**
153 Public Procurement & Disposal of Public Assets (PPDA)	0.940	0.385	0.000	0.000	**1.325**	**1.325**	0.940	0.385	0.000	0.000	**1.325**	**1.325**	0.987	0.393	0.000	0.000	**1.379**	**1.379**
154 Uganda National Bureau of Standards (UNBS)	23.856	7.489	4.592	0.000	**35.936**	**35.936**	23.856	7.489	5.051	0.000	**36.395**	**36.395**	25.048	7.638	5.051	0.000	**37.738**	**37.738**
161 Uganda Free Zones Authority	2.393	3.248	22.716	0.000	**28.357**	**28.357**	2.393	3.248	5.409	0.000	**11.050**	**11.050**	2.512	3.313	5.409	0.000	**11.235**	**11.235**
162 Uganda Microfinance Regulatory Authority	2.171	7.519	5.450	0.000	**15.140**	**15.140**	2.171	7.519	1.800	0.000	**11.490**	**11.490**	2.279	7.670	1.800	0.000	**11.749**	**11.749**
163 Uganda Retirement Benefits Regulatory Authority	5.794	7.831	0.000	0.000	**13.625**	**13.625**	5.794	7.831	0.000	0.000	**13.625**	**13.625**	6.084	7.987	0.000	0.000	**14.071**	**14.071**
506 Uganda High Commission in Tanzania, Dar es Salaam	0.000	0.200	0.000	0.000	**0.200**	**0.200**	0.000	0.200	0.000	0.000	**0.200**	**0.200**	0.000	0.200	0.000	0.000	**0.200**	**0.200**
510 Uganda Embassy in the United States, Washington	0.000	0.790	0.000	0.000	**0.790**	**0.790**	0.000	0.790	0.000	0.000	**0.790**	**0.790**	0.000	0.790	0.000	0.000	**0.790**	**0.790**

ANNEX 2: MEDIUM TERM EXPENDITURE FRAMEWORK (MTEF)2022/23 - 2024/25(Excl. Arrears and AIA)

Billion Uganda Shillings	FY2022/23 Approved Budget						FY2023/24 Budget						FY2024/25 Budget Projections					
PROGRAMME/VOTE	Wage	Non-Wage Recurrent	Domestic Dev	External Financing	Total excl. External Financing	Total incl. External Financing	Wage	Non-Wage Recurrent	Domestic Dev	External Financing	Total excl. External Financing	Total incl. External Financing	Wage	Non-Wage Recurrent	Domestic Dev	External Financing	Total excl. External Financing	Total incl. External Financing
**07 PRIVATE SECTOR DEVELOPMENT**																		
515 Uganda Embassy in Japan, Tokyo	0.000	0.021	0.000	0.000	**0.021**	**0.021**	0.000	0.021	0.000	0.000	**0.021**	**0.021**	0.000	0.021	0.000	0.000	**0.021**	**0.021**
523 Uganda Embassy in Germany, Berlin	0.000	0.085	0.000	0.000	**0.085**	**0.085**	0.000	0.085	0.000	0.000	**0.085**	**0.085**	0.000	0.085	0.000	0.000	**0.085**	**0.085**
527 Uganda Embassy in South Sudan, Juba	0.000	0.150	0.000	0.000	**0.150**	**0.150**	0.000	0.150	0.000	0.000	**0.150**	**0.150**	0.000	0.150	0.000	0.000	**0.150**	**0.150**
531 Uganda Embassy in Turkey, Ankara	0.000	0.210	0.000	0.000	**0.210**	**0.210**	0.000	0.210	0.000	0.000	**0.210**	**0.210**	0.000	0.210	0.000	0.000	**0.210**	**0.210**
532 Uganda Embassy in Somalia, Mogadishu	0.000	0.050	0.000	0.000	**0.050**	**0.050**	0.000	0.050	0.000	0.000	**0.050**	**0.050**	0.000	0.050	0.000	0.000	**0.050**	**0.050**
607 Local Governments 07	0.000	2.232	0.000	0.000	**2.232**	**2.232**	0.000	2.232	0.000	0.000	**2.232**	**2.232**	0.000	2.277	0.000	0.000	**2.277**	**2.277**
**Sub Total For: PRIVATE SECTOR DEVELOPMENT**	53.644	1,486.678	42.236	71.352	**1,582.559**	**1,653.911**	53.819	1,470.102	16.204	257.950	**1,540.125**	**1,798.075**	56.510	1,499.473	16.204	438.098	**1,572.188**	**2,010.286**
**08 SUSTAINABLE ENERGY DEVELOPMENT**																		
005 Ministry of Public Service	0.000	0.000	0.000	0.000	**0.000**	**0.000**	0.000	0.500	0.000	0.000	**0.500**	**0.500**	0.000	0.510	0.000	0.000	**0.510**	**0.510**
006 Ministry of Foreign Affairs	0.000	0.000	0.000	0.000	**0.000**	**0.000**	0.000	0.500	0.000	0.000	**0.500**	**0.500**	0.000	0.510	0.000	0.000	**0.510**	**0.510**
007 Ministry of Justice and Constitutional Affairs	0.000	0.000	0.000	0.000	**0.000**	**0.000**	0.000	1.180	0.000	0.000	**1.180**	**1.180**	0.000	1.204	0.000	0.000	**1.204**	**1.204**
008 Ministry of Finance, Planning and Economic Development	0.000	0.000	0.000	0.000	**0.000**	**0.000**	0.000	1.090	0.000	0.000	**1.090**	**1.090**	0.000	1.112	0.000	0.000	**1.112**	**1.112**
012 Ministry of Lands, Housing & Urban Development	0.000	0.000	0.000	0.000	**0.000**	**0.000**	0.000	0.000	0.500	0.000	**0.500**	**0.500**	0.000	0.000	0.500	0.000	**0.500**	**0.500**
017 Ministry of Energy and Mineral Development	25.231	11.009	509.945	1,030.924	**546.185**	**1,577.108**	15.800	60.600	461.790	673.512	**538.190**	**1,211.702**	16.590	61.681	461.790	1,154.885	**540.060**	**1,694.945**
150 National Environment Management Authority (NEMA)	0.000	0.000	0.000	0.000	**0.000**	**0.000**	0.000	0.000	2.500	0.000	**2.500**	**2.500**	0.000	0.000	2.500	0.000	**2.500**	**2.500**
154 Uganda National Bureau of Standards (UNBS)	0.000	0.000	0.000	0.000	**0.000**	**0.000**	0.000	3.350	1.600	0.000	**4.950**	**4.950**	0.000	3.417	1.600	0.000	**5.017**	**5.017**
**Sub Total For: SUSTAINABLE ENERGY DEVELOPMENT**	25.231	11.009	509.945	1,030.924	**546.185**	**1,577.108**	15.800	67.220	466.390	673.512	**549.410**	**1,222.922**	16.590	68.433	466.390	1,154.885	**551.413**	**1,706.297**
**09 INTEGRATED TRANSPORT INFRASTRUCTURE AND SERVICES**																		
016 Ministry of Works and Transport	13.758	90.754	528.270	244.057	**632.783**	**876.840**	13.758	90.225	190.220	1,758.892	**294.203**	**2,053.096**	14.446	92.030	190.220	1,821.078	**296.696**	**2,117.773**

ANNEX 2: MEDIUM TERM EXPENDITURE FRAMEWORK (MTEF)2022/23 - 2024/25(Excl. Arrears and AIA)

Billion Uganda Shillings	FY2022/23 Approved Budget						FY2023/24 Budget						FY2024/25 Budget Projections					
PROGRAMME/VOTE	Wage	Non-Wage Recurrent	Domestic Dev	External Financing	Total excl. External Financing	Total incl. External Financing	Wage	Non-Wage Recurrent	Domestic Dev	External Financing	Total excl. External Financing	Total incl. External Financing	Wage	Non-Wage Recurrent	Domestic Dev	External Financing	Total excl. External Financing	Total incl. External Financing
**09 INTEGRATED TRANSPORT INFRASTRUCTURE AND SERVICES**																		
113 Uganda National Roads Authority (UNRA)	71.105	73.127	1,518.042	1,025.710	**1,662.274**	**2,687.984**	71.105	40.127	590.080	1,210.473	**701.312**	**1,911.785**	74.660	40.930	590.080	1,922.314	**705.670**	**2,627.984**
118 Uganda Road Fund (URF)	2.667	485.285	0.000	0.000	**487.953**	**487.953**	2.667	465.285	0.000	0.000	**467.952**	**467.952**	2.801	474.591	0.000	0.000	**477.391**	**477.391**
122 Kampala Capital City Authority (KCCA)	0.000	0.300	78.237	139.683	**78.537**	**218.220**	0.000	0.000	10.000	186.006	**10.000**	**196.006**	0.000	0.000	10.000	459.720	**10.000**	**469.720**
609 Local Governments 09	0.000	0.000	29.653	0.000	**29.653**	**29.653**	0.000	0.000	27.653	0.000	**27.653**	**27.653**	0.000	0.000	27.653	0.000	**27.653**	**27.653**
**Sub Total For: INTEGRATED TRANSPORT INFRASTRUCTURE AND SERVICES**	87.531	649.467	2,154.202	1,409.450	**2,891.200**	**4,300.650**	87.531	595.637	817.953	3,155.372	**1,501.121**	**4,656.493**	91.907	607.550	817.953	4,203.112	**1,517.410**	**5,720.522**
**10 SUSTAINABLE URBANISATION AND HOUSING**																		
011 Ministry of Local Government	1.111	0.265	0.000	0.000	**1.376**	**1.376**	1.111	0.000	0.000	0.000	**1.111**	**1.111**	1.167	0.000	0.000	0.000	**1.167**	**1.167**
012 Ministry of Lands, Housing & Urban Development	2.488	52.079	1.908	88.026	**56.476**	**144.502**	2.488	15.270	5.600	46.723	**23.358**	**70.081**	2.612	15.575	5.600	0.000	**23.788**	**23.788**
016 Ministry of Works and Transport	1.000	10.830	0.000	0.000	**11.830**	**11.830**	1.000	1.000	0.000	0.000	**2.000**	**2.000**	1.050	1.020	0.000	0.000	**2.070**	**2.070**
023 Ministry of Kampala Capital City and Metropolitan Affairs	0.000	0.500	0.000	0.000	**0.500**	**0.500**	0.000	2.000	0.000	0.000	**2.000**	**2.000**	0.000	2.040	0.000	0.000	**2.040**	**2.040**
161 Uganda Free Zones Authority	0.000	0.000	0.000	0.000	**0.000**	**0.000**	0.000	0.530	0.000	0.000	**0.530**	**0.530**	0.000	0.541	0.000	0.000	**0.541**	**0.541**
610 Local Governments 10	0.000	0.000	0.000	216.420	**0.000**	**216.420**	0.000	0.000	0.000	29.202	**0.000**	**29.202**	0.000	0.000	0.000	0.000	**0.000**	**0.000**
**Sub Total For: SUSTAINABLE URBANISATION AND HOUSING**	4.599	63.674	1.908	304.446	**70.182**	**374.628**	4.599	18.800	5.600	75.925	**28.999**	**104.924**	4.829	19.176	5.600	0.000	**29.605**	**29.605**
**11 DIGITAL TRANSFORMATION**																		
020 Ministry of ICT and National Guidance	5.711	30.009	33.680	0.000	**69.400**	**69.400**	5.711	20.846	19.548	0.000	**46.105**	**46.105**	5.996	21.263	19.548	0.000	**46.807**	**46.807**
122 Kampala Capital City Authority (KCCA)	0.000	2.557	0.000	0.000	**2.557**	**2.557**	0.000	0.000	0.000	0.000	**0.000**	**0.000**	0.000	0.000	0.000	0.000	**0.000**	**0.000**
123 National Lotteries and Gaming Regulatory Board	0.000	2.292	0.000	0.000	**2.292**	**2.292**	0.000	0.000	0.000	0.000	**0.000**	**0.000**	0.000	0.000	0.000	0.000	**0.000**	**0.000**
126 National Information Technologies Authority	11.211	29.802	5.276	3.688	**46.289**	**49.977**	11.211	34.839	4.788	79.818	**50.839**	**130.657**	11.772	35.536	4.788	134.397	**52.096**	**186.493**

ANNEX 2: MEDIUM TERM EXPENDITURE FRAMEWORK (MTEF)2022/23 - 2024/25(Excl. Arrears and AIA)

Billion Uganda Shillings	FY2022/23 Approved Budget						FY2023/24 Budget						FY2024/25 Budget Projections					
PROGRAMME/VOTE	Wage	Non-Wage Recurrent	Domestic Dev	External Financing	Total excl. External Financing	Total incl. External Financing	Wage	Non-Wage Recurrent	Domestic Dev	External Financing	Total excl. External Financing	Total incl. External Financing	Wage	Non-Wage Recurrent	Domestic Dev	External Financing	Total excl. External Financing	Total incl. External Financing
**Sub Total For: DIGITAL TRANSFORMATION**	16.922	64.660	38.956	3.688	**120.538**	**124.226**	16.922	55.685	24.336	79.818	**96.943**	**176.762**	17.768	56.799	24.336	134.397	**98.903**	**233.300**
**12 HUMAN CAPITAL DEVELOPMENT**																		
011 Ministry of Local Government	0.000	0.040	0.000	0.000	**0.040**	**0.040**	0.000	0.040	0.000	0.000	**0.040**	**0.040**	0.000	0.041	0.000	0.000	**0.041**	**0.041**
013 Ministry of Education and Sports	124.598	245.095	101.704	189.922	**471.396**	**661.318**	124.598	139.371	42.506	362.931	**306.475**	**669.405**	130.828	189.114	97.736	269.284	**417.678**	**686.962**
014 Ministry of Health	21.645	138.787	89.977	1,333.540	**250.408**	**1,583.949**	21.645	64.922	54.477	1,104.249	**141.043**	**1,245.293**	22.727	97.581	109.027	0.000	**229.335**	**229.335**
018 Ministry of Gender, Labour and Social Development	1.530	205.350	0.000	0.000	**206.881**	**206.881**	1.530	43.744	0.000	116.807	**45.274**	**162.082**	1.607	44.619	0.000	214.609	**46.225**	**260.834**
019 Ministry of Water and Environment	4.736	0.350	343.947	678.750	**349.033**	**1,027.783**	4.736	0.350	300.020	480.286	**305.106**	**785.392**	4.973	0.357	300.020	412.110	**305.350**	**717.460**
107 Uganda Aids Commission (UAC)	4.964	12.014	0.940	0.000	**17.918**	**17.918**	4.964	11.014	1.221	0.000	**17.199**	**17.199**	5.212	12.992	1.221	0.000	**19.425**	**19.425**
108 National Planning Authority (NPA)	0.000	8.933	0.000	0.000	**8.933**	**8.933**	0.000	8.933	0.000	0.000	**8.933**	**8.933**	0.000	11.202	0.000	0.000	**11.202**	**11.202**
111 National Curriculum Development Centre (NCDC)	8.554	11.671	1.900	0.000	**22.125**	**22.125**	8.554	11.671	3.374	0.000	**23.599**	**23.599**	8.982	23.740	3.374	0.000	**36.096**	**36.096**
114 Uganda Cancer Institute (UCI)	9.293	18.930	15.374	20.281	**43.596**	**63.878**	9.293	18.930	0.679	0.000	**28.901**	**28.901**	9.757	23.466	0.679	0.000	**33.902**	**33.902**
115 Uganda Heart Institute (UHI)	6.614	10.404	6.320	0.000	**23.338**	**23.338**	6.614	10.404	8.924	0.000	**25.942**	**25.942**	6.944	14.254	8.924	39.379	**30.122**	**69.501**
116 Uganda National Medical Stores	17.403	489.640	6.047	0.000	**513.090**	**513.090**	19.500	487.543	6.652	0.000	**513.695**	**513.695**	20.475	696.088	6.652	0.000	**723.215**	**723.215**
122 Kampala Capital City Authority (KCCA)	45.360	14.335	11.216	0.000	**70.911**	**70.911**	45.360	14.335	3.164	0.000	**62.858**	**62.858**	47.628	18.183	3.164	0.000	**68.975**	**68.975**
124 Equal Opportunities Commission	0.000	0.770	0.000	0.000	**0.770**	**0.770**	0.000	0.770	0.000	0.000	**0.770**	**0.770**	0.000	0.965	0.000	0.000	**0.965**	**0.965**
127 Uganda Virus Research Institute (UVRI)	2.218	5.514	2.400	0.000	**10.132**	**10.132**	2.218	5.514	0.000	0.000	**7.732**	**7.732**	2.329	11.063	0.000	0.000	**13.392**	**13.392**
128 Uganda National Examination Board (UNEB)	12.360	101.044	13.326	0.000	**126.730**	**126.730**	12.360	101.044	31.424	0.000	**144.828**	**144.828**	12.978	125.522	31.424	0.000	**169.924**	**169.924**
132 Education Service Commission (ESC)	2.884	6.509	5.053	0.000	**14.446**	**14.446**	2.884	6.509	2.436	0.000	**11.830**	**11.830**	3.029	8.114	2.436	0.000	**13.579**	**13.579**
134 Health Service Commission (HSC)	2.433	9.481	2.048	0.000	**13.962**	**13.962**	2.433	9.481	0.053	0.000	**11.967**	**11.967**	2.555	10.972	0.053	0.000	**13.579**	**13.579**
149 National Population Council	2.987	8.452	0.820	0.000	**12.259**	**12.259**	2.987	8.452	0.000	0.000	**11.439**	**11.439**	3.137	10.711	0.000	0.000	**13.847**	**13.847**
151 Uganda Blood Transfusion Service (UBTS)	6.091	14.574	2.992	0.000	**23.657**	**23.657**	6.091	14.574	1.234	0.000	**21.899**	**21.899**	6.396	27.741	1.234	0.000	**35.371**	**35.371**
164 National Council for Higher Education	5.240	9.565	5.000	0.000	**19.805**	**19.805**	5.240	9.565	0.000	0.000	**14.805**	**14.805**	5.502	13.165	0.000	0.000	**18.667**	**18.667**

ANNEX 2: MEDIUM TERM EXPENDITURE FRAMEWORK (MTEF)2022/23 - 2024/25(Excl. Arrears and AIA)

Billion Uganda Shillings	FY2022/23 Approved Budget						FY2023/24 Budget						FY2024/25 Budget Projections					
PROGRAMME/VOTE	Wage	Non-Wage Recurrent	Domestic Dev	External Financing	Total excl. External Financing	Total incl. External Financing	Wage	Non-Wage Recurrent	Domestic Dev	External Financing	Total excl. External Financing	Total incl. External Financing	Wage	Non-Wage Recurrent	Domestic Dev	External Financing	Total excl. External Financing	Total incl. External Financing
**12 HUMAN CAPITAL DEVELOPMENT**																		
165 Uganda Business and Technical Examination Board	4.895	22.592	6.246	0.000	**33.733**	**33.733**	6.125	21.362	3.300	0.000	**30.787**	**30.787**	6.431	34.545	3.300	0.000	**44.277**	**44.277**
166 National Council of Sports	1.609	46.204	0.000	0.000	**47.812**	**47.812**	1.609	15.779	0.000	0.000	**17.387**	**17.387**	1.689	16.094	0.000	0.000	**17.783**	**17.783**
301 Makerere University	208.970	144.311	13.064	0.000	**366.345**	**366.345**	208.970	138.483	11.033	0.000	**358.486**	**358.486**	219.419	193.411	11.033	0.000	**423.863**	**423.863**
302 Mbarara University	40.006	15.044	2.732	0.000	**57.782**	**57.782**	40.006	14.743	3.764	0.000	**58.513**	**58.513**	42.007	38.384	3.764	0.000	**84.155**	**84.155**
303 Makerere University Business School	62.645	41.036	3.629	0.000	**107.310**	**107.310**	62.645	41.036	2.126	0.000	**105.806**	**105.806**	65.777	50.805	2.126	0.000	**118.707**	**118.707**
304 Kyambogo University	61.172	74.048	2.790	0.000	**138.010**	**138.010**	61.172	74.048	1.690	0.000	**136.909**	**136.909**	64.230	102.752	1.690	0.000	**168.672**	**168.672**
305 Busitema University	33.657	14.434	11.968	0.000	**60.058**	**60.058**	33.657	14.434	6.384	0.000	**54.475**	**54.475**	35.340	17.866	6.384	0.000	**59.591**	**59.591**
306 Muni University	15.694	5.541	3.890	0.000	**25.125**	**25.125**	15.694	5.485	4.752	0.000	**25.931**	**25.931**	16.479	6.732	4.752	0.000	**27.963**	**27.963**
307 Kabale University	38.386	15.093	9.631	0.000	**63.111**	**63.111**	38.386	14.971	2.587	0.000	**55.944**	**55.944**	40.306	21.825	2.587	0.000	**64.717**	**64.717**
308 Soroti University	15.651	7.085	1.905	0.000	**24.640**	**24.640**	15.651	7.085	1.254	0.000	**23.989**	**23.989**	16.433	10.380	1.254	0.000	**28.067**	**28.067**
309 Gulu University	38.014	25.577	11.160	0.000	**74.751**	**74.751**	38.014	23.473	2.121	0.000	**63.608**	**63.608**	39.914	27.661	2.121	0.000	**69.697**	**69.697**
310 Lira University	15.553	6.814	4.610	0.000	**26.977**	**26.977**	15.553	6.748	5.000	0.000	**27.301**	**27.301**	16.331	11.918	5.000	0.000	**33.249**	**33.249**
312 Uganda Management Institute	18.754	16.276	0.600	0.000	**35.630**	**35.630**	18.754	16.276	1.320	0.000	**36.350**	**36.350**	19.692	30.567	1.320	0.000	**51.579**	**51.579**
313 Mountains of the Moon University	21.990	10.245	5.413	0.000	**37.648**	**37.648**	21.990	10.245	0.000	0.000	**32.235**	**32.235**	23.089	10.450	0.000	0.000	**33.539**	**33.539**
401 Mulago National Referral Hospital	45.779	42.444	10.082	0.000	**98.304**	**98.304**	45.779	42.365	5.260	0.000	**93.404**	**93.404**	48.067	60.636	5.260	0.000	**113.964**	**113.964**
402 Butabika Hospital	9.071	9.483	2.285	0.000	**20.839**	**20.839**	9.071	9.483	2.513	0.000	**21.068**	**21.068**	9.525	21.498	2.513	0.000	**33.536**	**33.536**
403 Arua Hospital	7.799	2.965	6.680	0.000	**17.444**	**17.444**	7.799	2.965	0.120	0.000	**10.884**	**10.884**	8.189	5.044	0.120	0.000	**13.353**	**13.353**
404 Fort Portal Hospital	8.993	2.646	0.200	0.000	**11.838**	**11.838**	8.993	2.646	0.120	0.000	**11.758**	**11.758**	9.442	4.877	0.120	0.000	**14.440**	**14.440**
405 Gulu Hospital	8.110	7.518	0.990	0.000	**16.618**	**16.618**	8.110	6.491	0.120	0.000	**14.722**	**14.722**	8.516	12.253	0.120	0.000	**20.889**	**20.889**
406 Hoima Hospital	8.638	1.971	5.770	0.000	**16.379**	**16.379**	8.638	1.971	0.120	0.000	**10.729**	**10.729**	9.069	3.407	0.120	0.000	**12.597**	**12.597**
407 Jinja Hospital	12.585	7.604	0.200	0.000	**20.389**	**20.389**	12.585	8.069	0.120	0.000	**20.774**	**20.774**	13.214	14.460	0.120	0.000	**27.794**	**27.794**
408 Kabale Hospital	6.316	5.114	1.120	0.000	**12.551**	**12.551**	6.316	5.247	0.120	0.000	**11.683**	**11.683**	6.632	7.014	0.120	0.000	**13.766**	**13.766**
409 Masaka Hospital	8.206	2.665	2.680	0.000	**13.551**	**13.551**	8.206	2.665	0.120	0.000	**10.991**	**10.991**	8.617	4.611	0.120	0.000	**13.348**	**13.348**
410 Mbale Hospital	9.351	8.283	3.817	0.000	**21.452**	**21.452**	9.351	7.632	0.120	0.000	**17.103**	**17.103**	9.819	14.027	0.120	0.000	**23.966**	**23.966**

ANNEX 2: MEDIUM TERM EXPENDITURE FRAMEWORK (MTEF)2022/23 - 2024/25(Excl. Arrears and AIA)

Billion Uganda Shillings	FY2022/23 Approved Budget						FY2023/24 Budget						FY2024/25 Budget Projections					
PROGRAMME/VOTE	Wage	Non-Wage Recurrent	Domestic Dev	External Financing	Total excl. External Financing	Total incl. External Financing	Wage	Non-Wage Recurrent	Domestic Dev	External Financing	Total excl. External Financing	Total incl. External Financing	Wage	Non-Wage Recurrent	Domestic Dev	External Financing	Total excl. External Financing	Total incl. External Financing
**12 HUMAN CAPITAL DEVELOPMENT**																		
411 Soroti Hospital	7.785	2.825	1.270	0.000	**11.879**	**11.879**	7.785	2.825	0.120	0.000	**10.729**	**10.729**	8.174	5.213	0.120	0.000	**13.506**	**13.506**
412 Lira Hospital	8.352	8.448	0.200	0.000	**17.000**	**17.000**	8.352	8.742	0.120	0.000	**17.215**	**17.215**	8.770	14.807	0.120	0.000	**23.697**	**23.697**
413 Mbarara Regional Hospital	8.848	9.382	1.670	0.000	**19.900**	**19.900**	8.848	10.024	0.120	0.000	**18.991**	**18.991**	9.290	16.495	0.120	0.000	**25.905**	**25.905**
414 Mubende Regional Referral Hospital	7.862	1.568	0.600	0.000	**10.030**	**10.030**	7.862	1.568	0.150	0.000	**9.580**	**9.580**	8.255	3.091	0.150	0.000	**11.497**	**11.497**
415 Moroto Regional Referral Hospital	7.145	3.490	0.200	0.000	**10.836**	**10.836**	7.145	3.635	0.120	0.000	**10.900**	**10.900**	7.503	6.044	0.120	0.000	**13.667**	**13.667**
416 Naguru National Referral Hospital	10.093	1.443	0.200	0.000	**11.737**	**11.737**	10.093	1.443	0.240	0.000	**11.777**	**11.777**	10.598	2.501	0.240	0.000	**13.340**	**13.340**
417 Kiruddu National Referral Hospital	10.176	14.688	1.500	0.000	**26.364**	**26.364**	10.176	14.688	1.530	0.000	**26.394**	**26.394**	10.685	23.016	1.530	0.000	**35.231**	**35.231**
418 Kawempe National Referral Hospital	9.407	6.288	0.900	0.000	**16.595**	**16.595**	9.407	6.288	0.900	0.000	**16.595**	**16.595**	9.877	9.530	0.900	0.000	**20.308**	**20.308**
419 Entebbe Regional Referral Hospital	4.614	2.254	0.900	0.000	**7.768**	**7.768**	4.614	2.254	0.900	0.000	**7.768**	**7.768**	4.845	3.440	0.900	0.000	**9.184**	**9.184**
420 Mulago Specialized Women and Neonatal Hospital	11.107	13.393	1.768	0.000	**26.268**	**26.268**	11.107	13.393	2.268	0.000	**26.768**	**26.768**	11.662	23.100	2.268	0.000	**37.031**	**37.031**
421 Kayunga Referral Hospital	3.724	7.437	0.000	0.000	**11.161**	**11.161**	3.724	7.437	0.000	0.000	**11.161**	**11.161**	3.910	12.572	0.000	0.000	**16.482**	**16.482**
422 Yumbe Referral Hospital	4.182	6.065	0.000	0.000	**10.247**	**10.247**	4.182	6.065	0.000	0.000	**10.247**	**10.247**	4.392	6.612	0.000	0.000	**11.003**	**11.003**
515 Uganda Embassy in Japan, Tokyo	0.000	0.044	0.000	0.000	**0.044**	**0.044**	0.000	0.044	0.000	0.000	**0.044**	**0.044**	0.000	0.044	0.000	0.000	**0.044**	**0.044**
516 Uganda Embassy in Saudi Arabia, Riyadh	0.000	0.001	0.000	0.000	**0.001**	**0.001**	0.000	0.001	0.000	0.000	**0.001**	**0.001**	0.000	0.001	0.000	0.000	**0.001**	**0.001**
519 Uganda Embassy in Italy, Rome	0.000	0.040	0.000	0.000	**0.040**	**0.040**	0.000	0.040	0.000	0.000	**0.040**	**0.040**	0.000	0.040	0.000	0.000	**0.040**	**0.040**
524 Uganda Embassy in Iran, Tehran	0.000	0.025	0.000	0.000	**0.025**	**0.025**	0.000	0.025	0.000	0.000	**0.025**	**0.025**	0.000	0.025	0.000	0.000	**0.025**	**0.025**
525 Uganda Embassy in Russia, Moscow	0.000	0.080	0.000	0.000	**0.080**	**0.080**	0.000	0.080	0.000	0.000	**0.080**	**0.080**	0.000	0.080	0.000	0.000	**0.080**	**0.080**
531 Uganda Embassy in Turkey, Ankara	0.000	0.150	0.000	0.000	**0.150**	**0.150**	0.000	0.150	0.000	0.000	**0.150**	**0.150**	0.000	0.150	0.000	0.000	**0.150**	**0.150**
612 Local Governments 12	2,311.816	430.010	397.368	0.000	**3,139.194**	**3,139.194**	2,888.816	430.010	488.868	0.000	**3,807.694**	**3,807.694**	3,191.383	508.320	512.070	0.000	**4,211.773**	**4,211.773**
**Sub Total For: HUMAN CAPITAL DEVELOPMENT**	3,385.865	2,354.079	1,127.102	2,222.493	**6,867.046**	**9,089.539**	3,966.192	1,969.576	1,005.563	2,064.273	**6,941.332**	**9,005.605**	4,322.629	2,696.190	1,138.545	935.382	**8,157.363**	**9,092.745**
**13 INNOVATION, TECHNOLOGY DEVELOPMENT AND TRANSFER**																		
002 State House	4.159	61.131	138.148	44.250	**203.438**	**247.688**	0.000	0.000	0.000	0.000	**0.000**	**0.000**	0.000	0.000	0.000	0.000	**0.000**	**0.000**
006 Ministry of Foreign Affairs	0.000	0.000	0.000	0.000	**0.000**	**0.000**	0.000	0.581	0.000	0.000	**0.581**	**0.581**	0.000	0.593	0.000	0.000	**0.593**	**0.593**

ANNEX 2: MEDIUM TERM EXPENDITURE FRAMEWORK (MTEF)2022/23 - 2024/25(Excl. Arrears and AIA)

Billion Uganda Shillings	FY2022/23 Approved Budget						FY2023/24 Budget						FY2024/25 Budget Projections					
PROGRAMME/VOTE	Wage	Non-Wage Recurrent	Domestic Dev	External Financing	Total excl. External Financing	Total incl. External Financing	Wage	Non-Wage Recurrent	Domestic Dev	External Financing	Total excl. External Financing	Total incl. External Financing	Wage	Non-Wage Recurrent	Domestic Dev	External Financing	Total excl. External Financing	Total incl. External Financing
**13 INNOVATION, TECHNOLOGY DEVELOPMENT AND TRANSFER**																		
110 Uganda Industrial Research Institute (UIRI)	10.326	12.376	2.800	0.000	25.502	25.502	5.860	1.500	0.000	0.000	7.360	7.360	6.153	1.530	0.000	0.000	7.683	7.683
119 Uganda Registration Services Bureau (URSB)	0.982	0.130	0.000	0.000	1.112	1.112	0.980	0.130	0.000	0.000	1.110	1.110	1.029	0.133	0.000	0.000	1.162	1.162
167 Science, Technology and Innovation	0.000	0.000	0.000	0.000	0.000	0.000	4.160	15.670	23.000	125.568	42.830	168.398	4.368	15.983	23.000	0.000	43.351	43.351
525 Uganda Embassy in Russia, Moscow	0.000	0.119	0.000	0.000	0.119	0.119	0.000	0.119	0.000	0.000	0.119	0.119	0.000	0.119	0.000	0.000	0.119	0.119
**Sub Total For: INNOVATION, TECHNOLOGY DEVELOPMENT AND TRANSFER**	15.467	73.756	140.947	44.250	230.170	274.420	11.000	18.000	23.000	125.568	52.000	177.568	11.550	18.357	23.000	0.000	52.907	52.907
**14 PUBLIC SECTOR TRANSFORMATION**																		
005 Ministry of Public Service	4.361	22.331	2.948	0.000	29.640	29.640	4.361	24.999	3.200	0.000	32.560	32.560	4.579	25.499	3.200	0.000	33.278	33.278
011 Ministry of Local Government	22.148	8.531	2.000	0.000	32.680	32.680	22.148	8.245	12.000	0.000	42.393	42.393	23.256	8.410	12.000	0.000	43.666	43.666
020 Ministry of ICT and National Guidance	0.000	0.000	0.000	0.000	0.000	0.000	0.000	2.440	0.000	0.000	2.440	2.440	0.000	2.489	0.000	0.000	2.489	2.489
023 Ministry of Kampala Capital City and Metropolitan Affairs	0.490	9.175	0.000	0.000	9.665	9.665	0.490	0.790	0.000	0.000	1.280	1.280	0.515	0.806	0.000	0.000	1.320	1.320
103 Inspectorate of Government (IG)	3.840	3.709	0.000	0.000	7.549	7.549	3.840	3.520	0.000	0.000	7.360	7.360	4.032	3.590	0.000	0.000	7.622	7.622
108 National Planning Authority (NPA)	0.000	0.902	3.948	0.000	4.850	4.850	0.000	0.000	0.000	0.000	0.000	0.000	0.000	0.000	0.000	0.000	0.000	0.000
122 Kampala Capital City Authority (KCCA)	79.596	37.216	1.523	0.000	118.335	118.335	79.596	16.904	0.000	0.000	96.500	96.500	83.575	17.243	0.000	0.000	100.818	100.818
123 National Lotteries and Gaming Regulatory Board	2.484	2.344	0.000	0.000	4.828	4.828	2.484	0.000	0.000	0.000	2.484	2.484	2.608	0.000	0.000	0.000	2.608	2.608
126 National Information Technologies Authority	0.000	0.000	0.000	0.000	0.000	0.000	0.000	0.810	0.000	0.000	0.810	0.810	0.000	0.826	0.000	0.000	0.826	0.826
137 National Identification and Registration Authority (NIRA)	0.000	0.000	0.000	0.000	0.000	0.000	0.000	0.270	0.000	0.000	0.270	0.270	0.000	0.275	0.000	0.000	0.275	0.275
146 Public Service Commission (PSC)	3.289	6.918	1.281	0.000	11.487	11.487	3.289	8.201	1.400	0.000	12.890	12.890	3.453	8.365	1.400	0.000	13.218	13.218
147 Local Government Finance Commission (LGFC)	1.104	2.459	0.094	0.000	3.657	3.657	1.104	7.976	0.100	0.000	9.180	9.180	1.159	8.136	0.100	0.000	9.395	9.395

ANNEX 2: MEDIUM TERM EXPENDITURE FRAMEWORK (MTEF)2022/23 - 2024/25(Excl. Arrears and AIA)

Billion Uganda Shillings	FY2022/23 Approved Budget						FY2023/24 Budget						FY2024/25 Budget Projections					
PROGRAMME/VOTE	Wage	Non-Wage Recurrent	Domestic Dev	External Financing	Total excl. External Financing	Total incl. External Financing	Wage	Non-Wage Recurrent	Domestic Dev	External Financing	Total excl. External Financing	Total incl. External Financing	Wage	Non-Wage Recurrent	Domestic Dev	External Financing	Total excl. External Financing	Total incl. External Financing
**Sub Total For: PUBLIC SECTOR TRANSFORMATION**	117.312	93.586	11.794	0.000	**222.692**	**222.692**	117.312	74.155	16.700	0.000	**208.167**	**208.167**	123.178	75.639	16.700	0.000	**215.516**	**215.516**
**15 COMMUNITY MOBILIZATION AND MINDSET CHANGE**																		
001 Office of the President	0.000	9.100	0.000	0.000	**9.100**	**9.100**	0.000	0.000	0.000	0.000	**0.000**	**0.000**	0.000	0.000	0.000	0.000	**0.000**	**0.000**
006 Ministry of Foreign Affairs	0.000	0.399	0.000	0.000	**0.399**	**0.399**	0.000	0.000	0.000	0.000	**0.000**	**0.000**	0.000	0.000	0.000	0.000	**0.000**	**0.000**
018 Ministry of Gender, Labour and Social Development	2.369	20.033	1.918	0.000	**24.319**	**24.319**	2.369	11.000	5.000	0.000	**18.369**	**18.369**	2.487	11.220	5.000	0.000	**18.707**	**18.707**
020 Ministry of ICT and National Guidance	1.068	23.700	0.000	0.000	**24.768**	**24.768**	1.020	0.000	0.000	0.000	**1.020**	**1.020**	1.071	0.000	0.000	0.000	**1.071**	**1.071**
112 Directorate of Ethics and Integrity (DEI)	0.000	0.760	0.000	0.000	**0.760**	**0.760**	0.000	0.000	0.000	0.000	**0.000**	**0.000**	0.000	0.000	0.000	0.000	**0.000**	**0.000**
119 Uganda Registration Services Bureau (URSB)	0.339	0.346	0.000	0.000	**0.685**	**0.685**	0.300	0.000	0.000	0.000	**0.300**	**0.300**	0.315	0.000	0.000	0.000	**0.315**	**0.315**
122 Kampala Capital City Authority (KCCA)	0.000	0.561	1.488	0.000	**2.049**	**2.049**	0.000	0.000	0.000	0.000	**0.000**	**0.000**	0.000	0.000	0.000	0.000	**0.000**	**0.000**
123 National Lotteries and Gaming Regulatory Board	0.000	0.116	0.000	0.000	**0.116**	**0.116**	0.000	0.000	0.000	0.000	**0.000**	**0.000**	0.000	0.000	0.000	0.000	**0.000**	**0.000**
124 Equal Opportunities Commission	0.000	0.782	0.000	0.000	**0.782**	**0.782**	0.000	2.000	0.000	0.000	**2.000**	**2.000**	0.000	2.040	0.000	0.000	**2.040**	**2.040**
149 National Population Council	0.344	1.131	0.000	0.000	**1.475**	**1.475**	0.310	0.000	0.000	0.000	**0.310**	**0.310**	0.326	0.000	0.000	0.000	**0.326**	**0.326**
503 Uganda High Commission in Canada, Ottawa	0.000	0.165	0.000	0.000	**0.165**	**0.165**	0.000	0.000	0.000	0.000	**0.000**	**0.000**	0.000	0.000	0.000	0.000	**0.000**	**0.000**
506 Uganda High Commission in Tanzania, Dar es Salaam	0.000	0.058	0.000	0.000	**0.058**	**0.058**	0.000	0.000	0.000	0.000	**0.000**	**0.000**	0.000	0.000	0.000	0.000	**0.000**	**0.000**
507 Uganda High Commission in Nigeria, Abuja	0.000	0.100	0.000	0.000	**0.100**	**0.100**	0.000	0.000	0.000	0.000	**0.000**	**0.000**	0.000	0.000	0.000	0.000	**0.000**	**0.000**
508 Uganda High Commission in South Africa, Pretoria	0.000	0.030	0.000	0.000	**0.030**	**0.030**	0.000	0.000	0.000	0.000	**0.000**	**0.000**	0.000	0.000	0.000	0.000	**0.000**	**0.000**
509 Uganda High Commission in Rwanda, Kigali	0.000	0.357	0.000	0.000	**0.357**	**0.357**	0.000	0.000	0.000	0.000	**0.000**	**0.000**	0.000	0.000	0.000	0.000	**0.000**	**0.000**
515 Uganda Embassy in Japan, Tokyo	0.000	0.021	0.000	0.000	**0.021**	**0.021**	0.000	0.000	0.000	0.000	**0.000**	**0.000**	0.000	0.000	0.000	0.000	**0.000**	**0.000**
518 Uganda Embassy in Belgium, Brussels	0.000	0.360	0.000	0.000	**0.360**	**0.360**	0.000	0.000	0.000	0.000	**0.000**	**0.000**	0.000	0.000	0.000	0.000	**0.000**	**0.000**

ANNEX 2: MEDIUM TERM EXPENDITURE FRAMEWORK (MTEF)2022/23 - 2024/25(Excl. Arrears and AIA)

Billion Uganda Shillings	FY2022/23 Approved Budget						FY2023/24 Budget						FY2024/25 Budget Projections					
PROGRAMME/VOTE	Wage	Non-Wage Recurrent	Domestic Dev	External Financing	Total excl. External Financing	Total incl. External Financing	Wage	Non-Wage Recurrent	Domestic Dev	External Financing	Total excl. External Financing	Total incl. External Financing	Wage	Non-Wage Recurrent	Domestic Dev	External Financing	Total excl. External Financing	Total incl. External Financing
**15 COMMUNITY MOBILIZATION AND MINDSET CHANGE**																		
519 Uganda Embassy in Italy, Rome	0.000	0.050	0.000	0.000	0.050	0.050	0.000	0.000	0.000	0.000	0.000	0.000	0.000	0.000	0.000	0.000	0.000	0.000
522 Uganda Embassy in France, Paris	0.000	0.084	0.000	0.000	0.084	0.084	0.000	0.000	0.000	0.000	0.000	0.000	0.000	0.000	0.000	0.000	0.000	0.000
523 Uganda Embassy in Germany, Berlin	0.000	0.082	0.000	0.000	0.082	0.082	0.000	0.000	0.000	0.000	0.000	0.000	0.000	0.000	0.000	0.000	0.000	0.000
525 Uganda Embassy in Russia, Moscow	0.000	0.010	0.000	0.000	0.010	0.010	0.000	0.000	0.000	0.000	0.000	0.000	0.000	0.000	0.000	0.000	0.000	0.000
526 Uganda Embassy in Australia, Canberra	0.000	0.064	0.000	0.000	0.064	0.064	0.000	0.000	0.000	0.000	0.000	0.000	0.000	0.000	0.000	0.000	0.000	0.000
527 Uganda Embassy in South Sudan, Juba	0.000	0.050	0.000	0.000	0.050	0.050	0.000	0.000	0.000	0.000	0.000	0.000	0.000	0.000	0.000	0.000	0.000	0.000
528 Uganda Embassy in United Arab Emirates, Abudhabi	0.000	1.135	0.000	0.000	1.135	1.135	0.000	0.000	0.000	0.000	0.000	0.000	0.000	0.000	0.000	0.000	0.000	0.000
529 Uganda Embassy in Burundi, Bujumbura	0.000	0.044	0.000	0.000	0.044	0.044	0.000	0.000	0.000	0.000	0.000	0.000	0.000	0.000	0.000	0.000	0.000	0.000
531 Uganda Embassy in Turkey, Ankara	0.000	0.259	0.000	0.000	0.259	0.259	0.000	0.000	0.000	0.000	0.000	0.000	0.000	0.000	0.000	0.000	0.000	0.000
533 Uganda Embassy in Malaysia, Kuala Lumpur	0.000	0.022	0.000	0.000	0.022	0.022	0.000	0.000	0.000	0.000	0.000	0.000	0.000	0.000	0.000	0.000	0.000	0.000
615 Local Governments 15	0.000	7.640	0.000	0.000	7.640	7.640	0.000	0.000	0.000	0.000	0.000	0.000	0.000	0.000	0.000	0.000	0.000	0.000
**Sub Total For: COMMUNITY MOBILIZATION AND MINDSET CHANGE**	4.119	67.459	3.406	0.000	74.984	74.984	3.999	13.000	5.000	0.000	21.999	21.999	4.199	13.260	5.000	0.000	22.459	22.459
**16 GOVERNANCE AND SECURITY**																		
001 Office of the President	25.307	135.896	34.308	0.000	195.511	195.511	25.310	141.896	22.360	0.000	189.566	189.566	26.576	144.733	22.360	0.000	193.669	193.669
002 State House	22.637	375.579	36.203	0.000	434.419	434.419	22.637	375.579	21.722	0.000	419.938	419.938	23.769	383.090	21.722	0.000	428.581	428.581
003 Office of the Prime Minister	0.214	0.500	0.569	77.438	1.283	78.720	0.214	0.500	0.342	38.936	1.056	39.991	0.224	0.510	0.342	0.000	1.076	1.076
004 Ministry of Defence	617.579	765.262	1,982.917	393.015	3,365.758	3,758.773	617.579	765.262	1,982.727	194.679	3,365.568	3,560.247	659.338	780.567	1,982.727	0.000	3,422.632	3,422.632
006 Ministry of Foreign Affairs	6.009	28.071	0.428	0.000	34.508	34.508	6.009	21.509	0.770	0.000	28.288	28.288	6.310	21.939	0.770	0.000	29.019	29.019
007 Ministry of Justice and Constitutional Affairs	10.865	116.025	21.740	0.000	148.630	148.630	10.865	117.502	21.740	0.000	150.108	150.108	11.409	119.853	21.740	0.000	153.001	153.001
008 Ministry of Finance, Planning and Economic Development	0.000	2.020	0.000	0.000	2.020	2.020	0.000	2.020	0.000	0.000	2.020	2.020	0.000	2.060	0.000	0.000	2.060	2.060

ANNEX 2: MEDIUM TERM EXPENDITURE FRAMEWORK (MTEF)2022/23 - 2024/25(Excl. Arrears and AIA)

Billion Uganda Shillings	FY2022/23 Approved Budget						FY2023/24 Budget						FY2024/25 Budget Projections					
PROGRAMME/VOTE	Wage	Non-Wage Recurrent	Domestic Dev	External Financing	Total excl. External Financing	Total incl. External Financing	Wage	Non-Wage Recurrent	Domestic Dev	External Financing	Total excl. External Financing	Total incl. External Financing	Wage	Non-Wage Recurrent	Domestic Dev	External Financing	Total excl. External Financing	Total incl. External Financing
**16 GOVERNANCE AND SECURITY**																		
009 Ministry of Internal Affairs	2.448	54.447	3.647	0.000	60.543	60.543	2.448	54.447	3.000	0.000	59.896	59.896	2.571	55.536	3.000	0.000	61.107	61.107
011 Ministry of Local Government	0.283	0.471	0.000	0.000	0.754	0.754	0.283	0.471	0.000	0.000	0.754	0.754	0.297	0.480	0.000	0.000	0.777	0.777
018 Ministry of Gender, Labour and Social Development	0.101	4.039	0.000	0.000	4.140	4.140	0.101	4.039	0.000	0.000	4.140	4.140	0.106	4.119	0.000	0.000	4.226	4.226
021 Ministry of East African Community Affairs	1.280	35.580	0.425	0.000	37.285	37.285	1.280	35.580	1.325	0.000	38.185	38.185	1.344	36.291	1.325	0.000	38.961	38.961
102 Electoral Commission (EC)	37.667	104.910	3.720	0.000	146.297	146.297	37.667	104.910	3.720	0.000	146.297	146.297	39.550	107.009	3.720	0.000	150.279	150.279
103 Inspectorate of Government (IG)	24.113	32.506	15.200	0.000	71.819	71.819	24.113	32.506	15.200	0.000	71.819	71.819	25.319	33.020	15.200	0.000	73.539	73.539
105 Law Reform Commission (LRC)	4.073	13.957	0.120	0.000	18.150	18.150	4.073	13.957	0.420	0.000	18.450	18.450	4.277	14.236	0.420	0.000	18.933	18.933
106 Uganda Human Rights Commission (UHRC)	7.595	11.446	0.631	0.000	19.672	19.672	7.595	11.446	0.531	0.000	19.572	19.572	7.975	11.675	0.531	0.000	20.180	20.180
112 Directorate of Ethics and Integrity (DEI)	6.323	7.824	0.354	0.000	14.501	14.501	6.323	8.124	0.654	0.000	15.101	15.101	6.639	8.287	0.654	0.000	15.580	15.580
119 Uganda Registration Services Bureau (URSB)	7.723	17.054	0.970	0.000	25.747	25.747	7.723	17.054	1.000	0.000	25.777	25.777	8.109	17.395	1.000	0.000	26.504	26.504
120 National Citizenship and Immigration Control (NCIC)	5.274	104.021	10.536	0.000	119.831	119.831	5.274	104.021	6.320	0.000	115.615	115.615	5.538	106.101	6.320	0.000	117.959	117.959
123 National Lotteries and Gaming Regulatory Board	0.000	0.015	0.000	0.000	0.015	0.015	0.000	0.000	0.000	0.000	0.000	0.000	0.000	0.000	0.000	0.000	0.000	0.000
124 Equal Opportunities Commission	0.000	1.467	0.000	0.000	1.467	1.467	0.000	1.467	0.000	0.000	1.467	1.467	0.000	1.497	0.000	0.000	1.497	1.497
129 Financial Intelligence Authority (FIA)	9.594	16.928	0.129	0.000	26.651	26.651	9.594	16.928	0.729	0.000	27.251	27.251	10.074	17.267	0.729	0.000	28.070	28.070
131 Office of the Auditor General (OAG)	43.525	51.477	3.760	0.000	98.762	98.762	43.525	51.477	3.760	0.000	98.762	98.762	45.701	52.507	3.760	0.000	101.968	101.968
133 Directorate of Public Prosecution (DPP)	24.179	44.858	25.935	0.000	94.973	94.973	24.089	34.606	17.041	0.000	75.735	75.735	25.293	35.298	17.041	0.000	77.632	77.632
135 Directorate of Government Analytical Laboratory (DGAL)	3.318	25.448	7.700	0.000	36.466	36.466	3.318	25.448	4.620	0.000	33.386	33.386	3.484	25.957	4.620	0.000	34.061	34.061
137 National Identification and Registration Authority (NIRA)	20.335	36.131	4.420	0.000	60.886	60.886	20.335	36.131	3.000	0.000	59.466	59.466	21.352	36.854	3.000	0.000	61.205	61.205
144 Uganda Police Force	383.034	305.434	187.971	0.000	876.439	876.439	383.034	305.434	134.261	0.000	822.729	822.729	402.186	311.542	134.261	0.000	847.990	847.990

ANNEX 2: MEDIUM TERM EXPENDITURE FRAMEWORK (MTEF)2022/23 - 2024/25(Excl. Arrears and AIA)

Billion Uganda Shillings	FY2022/23 Approved Budget						FY2023/24 Budget						FY2024/25 Budget Projections					
PROGRAMME/VOTE	Wage	Non-Wage Recurrent	Domestic Dev	External Financing	Total excl. External Financing	Total incl. External Financing	Wage	Non-Wage Recurrent	Domestic Dev	External Financing	Total excl. External Financing	Total incl. External Financing	Wage	Non-Wage Recurrent	Domestic Dev	External Financing	Total excl. External Financing	Total incl. External Financing
**16 GOVERNANCE AND SECURITY**																		
145 Uganda Prisons Service	95.630	186.719	26.371	0.000	**308.720**	**308.720**	95.630	186.719	26.371	0.000	**308.720**	**308.720**	100.412	190.453	26.371	0.000	**317.236**	**317.236**
153 Public Procurement & Disposal of Public Assets (PPDA)	11.015	7.790	2.996	0.000	**21.802**	**21.802**	11.015	7.790	3.000	0.000	**21.805**	**21.805**	11.566	7.946	3.000	0.000	**22.512**	**22.512**
158 Internal Security Organization (ISO)	52.461	85.797	17.795	0.000	**156.052**	**156.052**	52.461	85.797	10.680	0.000	**148.938**	**148.938**	55.084	87.513	10.680	0.000	**153.277**	**153.277**
159 External Security Organization (ESO)	17.813	53.562	0.839	0.000	**72.215**	**72.215**	18.313	53.562	1.003	0.000	**72.879**	**72.879**	19.229	54.634	1.003	0.000	**74.866**	**74.866**
311 Law Development Centre	7.975	15.419	5.336	0.000	**28.730**	**28.730**	7.975	15.419	5.200	0.000	**28.594**	**28.594**	8.373	15.728	5.200	0.000	**29.301**	**29.301**
501 Uganda Mission at the United Nations, New York	1.951	14.607	0.000	0.000	**16.559**	**16.559**	1.951	14.607	1.053	0.000	**17.612**	**17.612**	1.951	14.607	1.053	0.000	**17.612**	**17.612**
502 Uganda High Commission in the United Kingdom	1.397	2.614	0.000	0.000	**4.011**	**4.011**	1.397	2.614	2.114	0.000	**6.124**	**6.124**	1.397	2.614	2.114	0.000	**6.124**	**6.124**
503 Uganda High Commission in Canada, Ottawa	1.175	3.938	0.000	0.000	**5.114**	**5.114**	1.175	3.938	8.500	0.000	**13.614**	**13.614**	1.175	3.938	8.500	0.000	**13.614**	**13.614**
504 Uganda High Commission in India, New Delhi	0.306	2.277	0.000	0.000	**2.583**	**2.583**	0.306	2.277	0.270	0.000	**2.853**	**2.853**	0.306	2.277	0.270	0.000	**2.853**	**2.853**
505 Uganda High Commission in Kenya, Nairobi	0.339	3.354	10.000	0.000	**13.693**	**13.693**	0.339	3.354	6.700	0.000	**10.393**	**10.393**	0.339	3.354	6.700	0.000	**10.393**	**10.393**
506 Uganda High Commission in Tanzania, Dar es Salaam	0.603	4.646	3.500	0.000	**8.749**	**8.749**	0.603	4.646	3.928	0.000	**9.177**	**9.177**	0.603	4.646	3.928	0.000	**9.177**	**9.177**
507 Uganda High Commission in Nigeria, Abuja	0.386	1.774	3.750	0.000	**5.910**	**5.910**	0.386	2.336	16.200	0.000	**18.922**	**18.922**	0.386	2.336	16.200	0.000	**18.922**	**18.922**
508 Uganda High Commission in South Africa, Pretoria	0.440	2.638	0.000	0.000	**3.079**	**3.079**	0.440	2.638	0.390	0.000	**3.469**	**3.469**	0.440	2.638	0.390	0.000	**3.469**	**3.469**
509 Uganda High Commission in Rwanda, Kigali	0.529	2.232	0.176	0.000	**2.937**	**2.937**	0.529	2.232	0.600	0.000	**3.361**	**3.361**	0.529	2.232	0.600	0.000	**3.361**	**3.361**
510 Uganda Embassy in the United States, Washington	1.362	5.603	2.620	0.000	**9.585**	**9.585**	1.362	5.603	0.540	0.000	**7.505**	**7.505**	1.362	5.603	0.540	0.000	**7.505**	**7.505**
511 Uganda Embassy in Egypt, Cairo	0.544	2.278	0.000	0.000	**2.822**	**2.822**	0.544	2.278	0.170	0.000	**2.992**	**2.992**	0.544	2.278	0.170	0.000	**2.992**	**2.992**

ANNEX 2: MEDIUM TERM EXPENDITURE FRAMEWORK (MTEF)2022/23 - 2024/25(Excl. Arrears and AIA)

Billion Uganda Shillings	FY2022/23 Approved Budget						FY2023/24 Budget						FY2024/25 Budget Projections					
PROGRAMME/VOTE	Wage	Non-Wage Recurrent	Domestic Dev	External Financing	Total excl. External Financing	Total incl. External Financing	Wage	Non-Wage Recurrent	Domestic Dev	External Financing	Total excl. External Financing	Total incl. External Financing	Wage	Non-Wage Recurrent	Domestic Dev	External Financing	Total excl. External Financing	Total incl. External Financing
**16 GOVERNANCE AND SECURITY**																		
512 Uganda Embassy in Ethiopia, Addis Ababa	0.508	1.910	0.400	0.000	2.819	2.819	0.508	1.910	0.150	0.000	2.569	2.569	0.508	1.910	0.150	0.000	2.569	2.569
513 Uganda Embassy in China, Beijing	0.388	3.592	0.042	0.000	4.023	4.023	0.388	3.592	0.100	0.000	4.081	4.081	0.388	3.592	0.100	0.000	4.081	4.081
514 Uganda Embassy in Switzerland, Geneva	1.631	4.869	0.000	0.000	6.501	6.501	1.631	4.869	0.100	0.000	6.601	6.601	1.631	4.869	0.100	0.000	6.601	6.601
515 Uganda Embassy in Japan, Tokyo	1.510	3.669	0.000	0.000	5.179	5.179	1.510	3.669	0.000	0.000	5.179	5.179	1.510	3.669	0.000	0.000	5.179	5.179
516 Uganda Embassy in Saudi Arabia, Riyadh	0.999	4.441	0.600	0.000	6.041	6.041	0.999	4.441	0.000	0.000	5.441	5.441	0.999	4.441	0.000	0.000	5.441	5.441
517 Uganda Embassy in Denmark, Copenhagen	0.763	4.885	0.510	0.000	6.158	6.158	0.763	4.885	0.100	0.000	5.748	5.748	0.763	4.885	0.100	0.000	5.748	5.748
518 Uganda Embassy in Belgium, Brussels	1.099	3.070	0.000	0.000	4.169	4.169	1.099	3.070	0.380	0.000	4.549	4.549	1.099	3.070	0.380	0.000	4.549	4.549
519 Uganda Embassy in Italy, Rome	0.848	3.629	0.236	0.000	4.713	4.713	0.848	3.629	0.000	0.000	4.477	4.477	0.848	3.629	0.000	0.000	4.477	4.477
520 Uganda Embassy in DRC, Kinshasa	0.658	3.458	3.473	0.000	7.589	7.589	0.658	3.458	2.074	0.000	6.190	6.190	0.658	3.458	2.074	0.000	6.190	6.190
521 Uganda Embassy in Sudan, Khartoum	0.609	3.189	0.390	0.000	4.188	4.188	0.609	3.189	0.560	0.000	4.358	4.358	0.609	3.189	0.560	0.000	4.358	4.358
522 Uganda Embassy in France, Paris	0.951	5.506	13.075	0.000	19.533	19.533	0.951	5.506	0.000	0.000	6.458	6.458	0.951	5.506	0.000	0.000	6.458	6.458
523 Uganda Embassy in Germany, Berlin	1.132	5.468	0.970	0.000	7.571	7.571	1.132	5.468	0.000	0.000	6.601	6.601	1.132	5.468	0.000	0.000	6.601	6.601
524 Uganda Embassy in Iran, Tehran	0.756	2.002	0.000	0.000	2.758	2.758	0.756	2.002	0.000	0.000	2.758	2.758	0.756	2.002	0.000	0.000	2.758	2.758
525 Uganda Embassy in Russia, Moscow	0.610	2.940	0.000	0.000	3.550	3.550	0.610	2.940	0.172	0.000	3.722	3.722	0.610	2.940	0.172	0.000	3.722	3.722
526 Uganda Embassy in Australia, Canberra	0.989	2.974	6.200	0.000	10.163	10.163	0.989	2.974	0.150	0.000	4.113	4.113	0.989	2.974	0.150	0.000	4.113	4.113
527 Uganda Embassy in South Sudan, Juba	0.423	3.241	1.400	0.000	5.064	5.064	0.423	3.241	0.000	0.000	3.664	3.664	0.423	3.241	0.000	0.000	3.664	3.664
528 Uganda Embassy in United Arab Emirates, Abudhabi	0.765	3.550	0.350	0.000	4.665	4.665	0.765	3.550	0.000	0.000	4.315	4.315	0.765	3.550	0.000	0.000	4.315	4.315
529 Uganda Embassy in Burundi, Bujumbura	0.556	2.275	0.000	0.000	2.832	2.832	0.556	2.275	0.150	0.000	2.982	2.982	0.556	2.275	0.150	0.000	2.982	2.982
530 Uganda Consulate in China, Guangzhou	0.419	3.281	0.550	0.000	4.250	4.250	0.419	3.281	6.200	0.000	9.900	9.900	0.419	3.281	6.200	0.000	9.900	9.900
531 Uganda Embassy in Turkey, Ankara	0.695	3.723	0.000	0.000	4.418	4.418	0.695	3.723	0.390	0.000	4.808	4.808	0.695	3.723	0.390	0.000	4.808	4.808
532 Uganda Embassy in Somalia, Mogadishu	0.134	2.509	0.639	0.000	3.282	3.282	0.134	2.509	1.600	0.000	4.243	4.243	0.134	2.509	1.600	0.000	4.243	4.243

ANNEX 2: MEDIUM TERM EXPENDITURE FRAMEWORK (MTEF)2022/23 - 2024/25(Excl. Arrears and AIA)

Billion Uganda Shillings	FY2022/23 Approved Budget						FY2023/24 Budget						FY2024/25 Budget Projections					
PROGRAMME/VOTE	Wage	Non-Wage Recurrent	Domestic Dev	External Financing	Total excl. External Financing	Total incl. External Financing	Wage	Non-Wage Recurrent	Domestic Dev	External Financing	Total excl. External Financing	Total incl. External Financing	Wage	Non-Wage Recurrent	Domestic Dev	External Financing	Total excl. External Financing	Total incl. External Financing
**16 GOVERNANCE AND SECURITY**																		
533 Uganda Embassy in Malaysia, Kuala Lumpur	0.580	2.698	0.100	0.000	**3.378**	**3.378**	0.580	2.698	0.100	0.000	**3.378**	**3.378**	0.580	2.698	0.100	0.000	**3.378**	**3.378**
534 Uganda Consulate in Kenya, Mombasa	0.747	3.960	1.600	0.000	**6.306**	**6.306**	0.747	3.960	0.000	0.000	**4.706**	**4.706**	0.747	3.960	0.000	0.000	**4.706**	**4.706**
535 Uganda Embassy in Algeria, Algiers	0.915	2.774	2.000	0.000	**5.689**	**5.689**	0.915	2.774	0.000	0.000	**3.689**	**3.689**	0.915	2.774	0.000	0.000	**3.689**	**3.689**
536 Uganda Embassy in Qatar, Doha	0.541	2.103	0.295	0.000	**2.939**	**2.939**	0.541	2.103	0.198	0.000	**2.842**	**2.842**	0.541	2.103	0.198	0.000	**2.842**	**2.842**
537 Uganda Mission in Havana, Cuba	0.000	0.000	0.000	0.000	**0.000**	**0.000**	0.326	2.674	0.000	0.000	**3.000**	**3.000**	0.326	2.674	0.000	0.000	**3.000**	**3.000**
538 Uganda Mission in Luanda, Angola	0.000	0.000	0.000	0.000	**0.000**	**0.000**	0.585	2.415	0.000	0.000	**3.000**	**3.000**	0.585	2.415	0.000	0.000	**3.000**	**3.000**
**Sub Total For: GOVERNANCE AND SECURITY**	1,476.633	2,772.333	2,447.897	470.453	**6,696.862**	**7,167.315**	1,477.956	2,768.932	2,344.384	233.615	**6,591.272**	**6,824.887**	1,561.275	2,821.428	2,344.384	0.000	**6,727.087**	**6,727.087**
**17 REGIONAL BALANCED DEVELOPMENT**																		
003 Office of the Prime Minister	0.347	50.433	8.837	12.685	**59.617**	**72.302**	0.347	0.200	2.500	0.000	**3.047**	**3.047**	0.364	0.204	2.500	0.000	**3.068**	**3.068**
010 Ministry of Agriculture, Animal Industry and Fisheries	0.000	0.000	0.000	0.000	**0.000**	**0.000**	0.000	0.300	0.000	0.000	**0.300**	**0.300**	0.000	0.306	0.000	0.000	**0.306**	**0.306**
011 Ministry of Local Government	0.047	0.191	11.034	59.000	**11.273**	**70.273**	0.047	15.100	11.400	52.524	**26.547**	**79.071**	0.049	15.402	11.400	9.141	**26.851**	**35.992**
015 Ministry of Trade, Industry and Co-operatives	0.000	0.000	0.000	0.000	**0.000**	**0.000**	0.000	0.100	0.000	0.000	**0.100**	**0.100**	0.000	0.102	0.000	0.000	**0.102**	**0.102**
016 Ministry of Works and Transport	0.000	0.000	0.000	0.000	**0.000**	**0.000**	0.000	0.400	0.000	0.000	**0.400**	**0.400**	0.000	0.408	0.000	0.000	**0.408**	**0.408**
017 Ministry of Energy and Mineral Development	0.000	0.000	0.000	0.000	**0.000**	**0.000**	0.000	0.300	0.000	0.000	**0.300**	**0.300**	0.000	0.306	0.000	0.000	**0.306**	**0.306**
020 Ministry of ICT and National Guidance	0.000	0.000	0.000	0.000	**0.000**	**0.000**	0.000	0.200	0.000	0.000	**0.200**	**0.200**	0.000	0.204	0.000	0.000	**0.204**	**0.204**
022 Ministry of Tourism, Wildlife and Antiquities	0.000	0.000	0.000	0.000	**0.000**	**0.000**	0.000	0.100	0.000	0.000	**0.100**	**0.100**	0.000	0.102	0.000	0.000	**0.102**	**0.102**
108 National Planning Authority (NPA)	0.000	0.635	0.000	0.000	**0.635**	**0.635**	0.000	0.200	0.000	0.000	**0.200**	**0.200**	0.000	0.204	0.000	0.000	**0.204**	**0.204**
142 National Agricultural Research Organization (NARO)	0.000	0.000	0.000	0.000	**0.000**	**0.000**	0.000	0.100	0.000	0.000	**0.100**	**0.100**	0.000	0.102	0.000	0.000	**0.102**	**0.102**
147 Local Government Finance Commission (LGFC)	0.000	0.000	0.000	0.000	**0.000**	**0.000**	0.000	0.100	0.000	0.000	**0.100**	**0.100**	0.000	0.102	0.000	0.000	**0.102**	**0.102**

ANNEX 2: MEDIUM TERM EXPENDITURE FRAMEWORK (MTEF)2022/23 - 2024/25(Excl. Arrears and AIA)

Billion Uganda Shillings	FY2022/23 Approved Budget						FY2023/24 Budget						FY2024/25 Budget Projections					
PROGRAMME/VOTE	Wage	Non-Wage Recurrent	Domestic Dev	External Financing	Total excl. External Financing	Total incl. External Financing	Wage	Non-Wage Recurrent	Domestic Dev	External Financing	Total excl. External Financing	Total incl. External Financing	Wage	Non-Wage Recurrent	Domestic Dev	External Financing	Total excl. External Financing	Total incl. External Financing
**17 REGIONAL BALANCED DEVELOPMENT**																		
617 Local Governments 17	422.764	496.083	95.424	0.000	**1,014.271**	**1,014.271**	422.764	128.000	0.000	0.000	**550.764**	**550.764**	443.902	130.560	0.000	0.000	**574.462**	**574.462**
**Sub Total For: REGIONAL BALANCED DEVELOPMENT**	423.158	547.342	115.295	71.685	**1,085.796**	**1,157.481**	423.158	145.100	13.900	52.524	**582.158**	**634.682**	444.316	148.002	13.900	9.141	**606.218**	**615.359**
**18 DEVELOPMENT PLAN IMPLEMENTATION**																		
001 Office of the President	0.281	14.277	0.000	0.000	**14.558**	**14.558**	0.281	14.277	0.000	0.000	**14.558**	**14.558**	0.295	14.563	0.000	0.000	**14.857**	**14.857**
003 Office of the Prime Minister	3.213	44.415	3.776	0.000	**51.403**	**51.403**	3.213	45.415	3.776	0.000	**52.404**	**52.404**	3.373	46.323	3.776	0.000	**53.473**	**53.473**
006 Ministry of Foreign Affairs	0.000	0.346	0.000	0.000	**0.346**	**0.346**	0.000	0.346	0.000	0.000	**0.346**	**0.346**	0.000	0.353	0.000	0.000	**0.353**	**0.353**
008 Ministry of Finance, Planning and Economic Development	8.995	156.664	180.542	27.965	**346.201**	**374.166**	8.995	156.664	180.542	7.787	**346.201**	**353.988**	9.445	159.797	180.542	0.000	**349.784**	**349.784**
011 Ministry of Local Government	0.156	19.381	0.000	0.000	**19.537**	**19.537**	1.956	17.581	0.000	0.000	**19.537**	**19.537**	2.054	17.933	0.000	0.000	**19.986**	**19.986**
023 Ministry of Kampala Capital City and Metropolitan Affairs	0.000	0.050	0.000	0.000	**0.050**	**0.050**	0.000	0.050	0.000	0.000	**0.050**	**0.050**	0.000	0.051	0.000	0.000	**0.051**	**0.051**
103 Inspectorate of Government (IG)	0.000	0.000	0.000	0.000	**0.000**	**0.000**	0.000	0.000	9.397	0.000	**9.397**	**9.397**	0.000	0.000	9.397	0.000	**9.397**	**9.397**
108 National Planning Authority (NPA)	14.611	13.994	0.000	0.000	**28.605**	**28.605**	14.611	13.994	2.910	0.000	**31.515**	**31.515**	15.341	14.274	2.910	0.000	**32.525**	**32.525**
122 Kampala Capital City Authority (KCCA)	0.000	2.096	0.435	0.000	**2.532**	**2.532**	0.000	2.096	0.435	0.000	**2.531**	**2.531**	0.000	2.138	0.435	0.000	**2.573**	**2.573**
123 National Lotteries and Gaming Regulatory Board	0.000	0.342	0.000	0.000	**0.342**	**0.342**	0.000	0.342	0.000	0.000	**0.342**	**0.342**	0.000	0.349	0.000	0.000	**0.349**	**0.349**
124 Equal Opportunities Commission	2.967	7.899	0.216	0.000	**11.082**	**11.082**	2.967	7.899	0.216	0.000	**11.082**	**11.082**	3.115	8.057	0.216	0.000	**11.388**	**11.388**
130 Treasury Operations	0.000	15,174.174	0.000	0.000	**15,174.174**	**15,174.174**	0.000	17,565.632	0.000	0.000	**17,565.632**	**17,565.632**	0.000	19,653.738	0.000	0.000	**19,653.738**	**19,653.738**
131 Office of the Auditor General (OAG)	0.000	0.000	0.000	0.000	**0.000**	**0.000**	0.000	1.000	0.000	0.000	**1.000**	**1.000**	0.000	1.020	0.000	0.000	**1.020**	**1.020**
141 Uganda Revenue Authority (URA)	205.495	290.276	44.063	0.000	**539.834**	**539.834**	205.495	316.325	45.320	0.000	**567.140**	**567.140**	215.770	322.651	45.320	0.000	**583.741**	**583.741**
143 Uganda Bureau of Statistics (UBOS)	21.391	38.726	11.938	0.000	**72.055**	**72.055**	21.391	214.726	13.470	0.000	**249.587**	**249.587**	22.460	219.021	13.470	0.000	**254.951**	**254.951**
147 Local Government Finance Commission (LGFC)	0.515	1.283	0.000	0.000	**1.798**	**1.798**	0.515	1.283	0.000	0.000	**1.798**	**1.798**	0.541	1.309	0.000	0.000	**1.849**	**1.849**
149 National Population Council	0.000	0.000	0.000	0.000	**0.000**	**0.000**	0.000	0.000	0.541	0.000	**0.541**	**0.541**	0.000	0.000	0.541	0.000	**0.541**	**0.541**

Billion Uganda Shillings	FY2022/23 Approved Budget						FY2023/24 Budget						FY2024/25 Budget Projections					
PROGRAMME/VOTE	Wage	Non-Wage Recurrent	Domestic Dev	External Financing	Total excl. External Financing	Total incl. External Financing	Wage	Non-Wage Recurrent	Domestic Dev	External Financing	Total excl. External Financing	Total incl. External Financing	Wage	Non-Wage Recurrent	Domestic Dev	External Financing	Total excl. External Financing	Total incl. External Financing
**18 DEVELOPMENT PLAN IMPLEMENTATION**																		
501 Uganda Mission at the United Nations, New York	0.000	0.148	0.000	0.000	0.148	0.148	0.000	0.148	0.000	0.000	0.148	0.148	0.000	0.148	0.000	0.000	0.148	0.148
502 Uganda High Commission in the United Kingdom	0.000	1.277	0.000	0.000	1.277	1.277	0.000	1.277	0.000	0.000	1.277	1.277	0.000	1.277	0.000	0.000	1.277	1.277
503 Uganda High Commission in Canada, Ottawa	0.000	0.110	0.000	0.000	0.110	0.110	0.000	0.110	0.000	0.000	0.110	0.110	0.000	0.110	0.000	0.000	0.110	0.110
504 Uganda High Commission in India, New Delhi	0.000	1.000	0.000	0.000	1.000	1.000	0.000	1.000	0.000	0.000	1.000	1.000	0.000	1.000	0.000	0.000	1.000	1.000
505 Uganda High Commission in Kenya, Nairobi	0.000	0.088	0.000	0.000	0.088	0.088	0.000	0.088	0.000	0.000	0.088	0.088	0.000	0.088	0.000	0.000	0.088	0.088
506 Uganda High Commission in Tanzania, Dar es Salaam	0.000	0.301	0.000	0.000	0.301	0.301	0.000	0.301	0.000	0.000	0.301	0.301	0.000	0.301	0.000	0.000	0.301	0.301
510 Uganda Embassy in the United States, Washington	0.000	0.736	0.000	0.000	0.736	0.736	0.000	0.736	0.000	0.000	0.736	0.736	0.000	0.736	0.000	0.000	0.736	0.736
511 Uganda Embassy in Egypt, Cairo	0.000	0.129	0.000	0.000	0.129	0.129	0.000	0.129	0.000	0.000	0.129	0.129	0.000	0.129	0.000	0.000	0.129	0.129
512 Uganda Embassy in Ethiopia, Addis Ababa	0.000	0.489	0.000	0.000	0.489	0.489	0.000	0.489	0.000	0.000	0.489	0.489	0.000	0.489	0.000	0.000	0.489	0.489
513 Uganda Embassy in China, Beijing	0.000	0.266	0.000	0.000	0.266	0.266	0.000	0.266	0.000	0.000	0.266	0.266	0.000	0.266	0.000	0.000	0.266	0.266
514 Uganda Embassy in Switzerland, Geneva	0.000	0.343	0.000	0.000	0.343	0.343	0.000	0.343	0.000	0.000	0.343	0.343	0.000	0.343	0.000	0.000	0.343	0.343
515 Uganda Embassy in Japan, Tokyo	0.000	0.100	0.000	0.000	0.100	0.100	0.000	0.100	0.000	0.000	0.100	0.100	0.000	0.100	0.000	0.000	0.100	0.100
516 Uganda Embassy in Saudi Arabia, Riyadh	0.000	0.520	0.000	0.000	0.520	0.520	0.000	0.520	0.000	0.000	0.520	0.520	0.000	0.520	0.000	0.000	0.520	0.520
517 Uganda Embassy in Denmark, Copenhagen	0.000	0.161	0.000	0.000	0.161	0.161	0.000	0.161	0.000	0.000	0.161	0.161	0.000	0.161	0.000	0.000	0.161	0.161
519 Uganda Embassy in Italy, Rome	0.000	0.035	0.000	0.000	0.035	0.035	0.000	0.035	0.000	0.000	0.035	0.035	0.000	0.035	0.000	0.000	0.035	0.035
523 Uganda Embassy in Germany, Berlin	0.000	0.053	0.000	0.000	0.053	0.053	0.000	0.053	0.000	0.000	0.053	0.053	0.000	0.053	0.000	0.000	0.053	0.053
524 Uganda Embassy in Iran, Tehran	0.000	0.252	0.000	0.000	0.252	0.252	0.000	0.252	0.000	0.000	0.252	0.252	0.000	0.252	0.000	0.000	0.252	0.252

ANNEX 2: MEDIUM TERM EXPENDITURE FRAMEWORK (MTEF)2022/23 - 2024/25(Excl. Arrears and AIA)

Billion Uganda Shillings	FY2022/23 Approved Budget						FY2023/24 Budget						FY2024/25 Budget Projections					
PROGRAMME/VOTE	Wage	Non-Wage Recurrent	Domestic Dev	External Financing	Total excl. External Financing	Total incl. External Financing	Wage	Non-Wage Recurrent	Domestic Dev	External Financing	Total excl. External Financing	Total incl. External Financing	Wage	Non-Wage Recurrent	Domestic Dev	External Financing	Total excl. External Financing	Total incl. External Financing
**18 DEVELOPMENT PLAN IMPLEMENTATION**																		
527 Uganda Embassy in South Sudan, Juba	0.000	0.155	0.000	0.000	**0.155**	**0.155**	0.000	0.155	0.000	0.000	**0.155**	**0.155**	0.000	0.155	0.000	0.000	**0.155**	**0.155**
528 Uganda Embassy in United Arab Emirates, Abudhabi	0.000	1.600	0.000	0.000	**1.600**	**1.600**	0.000	1.600	0.000	0.000	**1.600**	**1.600**	0.000	1.600	0.000	0.000	**1.600**	**1.600**
535 Uganda Embassy in Algeria, Algiers	0.000	0.197	0.000	0.000	**0.197**	**0.197**	0.000	0.197	0.000	0.000	**0.197**	**0.197**	0.000	0.197	0.000	0.000	**0.197**	**0.197**
536 Uganda Embassy in Qatar, Doha	0.000	0.104	0.000	0.000	**0.104**	**0.104**	0.000	0.104	0.000	0.000	**0.104**	**0.104**	0.000	0.104	0.000	0.000	**0.104**	**0.104**
**Sub Total For: DEVELOPMENT PLAN IMPLEMENTATION**	257.622	15,771.989	240.970	27.965	**16,270.582**	**16,298.547**	259.422	18,365.695	256.607	7.787	**18,881.724**	**18,889.511**	272.394	20,469.641	256.607	0.000	**20,998.642**	**20,998.642**
**19 ADMINISTRATION OF JUSTICE**																		
018 Ministry of Gender, Labour and Social Development	0.000	0.000	0.000	0.000	**0.000**	**0.000**	0.000	0.500	0.000	0.000	**0.500**	**0.500**	0.000	0.510	0.000	0.000	**0.510**	**0.510**
101 Judiciary (Courts of Judicature)	108.767	209.830	63.010	0.000	**381.607**	**381.607**	108.767	215.764	63.010	0.000	**387.541**	**387.541**	114.205	220.080	63.010	0.000	**397.295**	**397.295**
133 Directorate of Public Prosecution (DPP)	0.000	0.000	0.000	0.000	**0.000**	**0.000**	3.854	7.989	0.000	0.000	**11.843**	**11.843**	4.047	8.149	0.000	0.000	**12.196**	**12.196**
144 Uganda Police Force	0.000	0.000	0.000	0.000	**0.000**	**0.000**	0.000	1.000	0.000	0.000	**1.000**	**1.000**	0.000	1.020	0.000	0.000	**1.020**	**1.020**
145 Uganda Prisons Service	0.000	0.000	0.000	0.000	**0.000**	**0.000**	0.000	0.000	1.000	0.000	**1.000**	**1.000**	0.000	0.000	1.000	0.000	**1.000**	**1.000**
148 Judicial Service Commission (JSC)	3.194	13.911	0.464	0.000	**17.568**	**17.568**	3.194	14.411	1.964	0.000	**19.568**	**19.568**	3.353	14.699	1.964	0.000	**20.016**	**20.016**
311 Law Development Centre	0.468	0.376	0.000	0.000	**0.844**	**0.844**	0.468	0.626	0.250	0.000	**1.344**	**1.344**	0.491	0.638	0.250	0.000	**1.380**	**1.380**
**Sub Total For: ADMINISTRATION OF JUSTICE**	112.429	224.117	63.473	0.000	**400.019**	**400.019**	116.283	240.290	66.223	0.000	**422.796**	**422.796**	122.097	245.096	66.223	0.000	**433.416**	**433.416**
**20 LEGISLATION, OVERSIGHT AND REPRESENTATION**																		
007 Ministry of Justice and Constitutional Affairs	0.000	0.000	0.000	0.000	**0.000**	**0.000**	0.000	0.100	0.000	0.000	**0.100**	**0.100**	0.000	0.102	0.000	0.000	**0.102**	**0.102**
011 Ministry of Local Government	0.000	0.080	0.000	0.000	**0.080**	**0.080**	0.000	0.100	0.000	0.000	**0.100**	**0.100**	0.000	0.102	0.000	0.000	**0.102**	**0.102**
104 Parliamentary Commission	112.636	734.852	67.491	0.000	**914.979**	**914.979**	112.525	739.763	67.491	0.000	**919.779**	**919.779**	118.151	754.559	67.491	0.000	**940.201**	**940.201**
**Sub Total For: LEGISLATION, OVERSIGHT AND REPRESENTATION**	112.636	734.932	67.491	0.000	**915.059**	**915.059**	112.525	739.963	67.491	0.000	**919.979**	**919.979**	118.151	754.763	67.491	0.000	**940.405**	**940.405**
**Grand Total**	**6,375.578**	**26,288.674**	**7,849.806**	**6,716.172**	**40,514.057**	**47,230.229**	**6,959.070**	**27,210.194**	**5,833.629**	**8,043.573**	**40,002.893**	**48,046.466**	**7,474.572**	**30,175.200**	**5,966.611**	**8,420.314**	**43,616.382**	**52,036.696**

ANNEX 2: MEDIUM TERM EXPENDITURE FRAMEWORK (MTEF)2025/26 - 2027/28(Excl. Arrears and AIA)

Billion Uganda Shillings	FY2025/26 Budget Projections						FY2026/27 Budget Projections						FY2027/28 Budget Projections					
PROGRAMME/VOTE	Wage	Non-Wage Recurrent	Domestic Dev	External Financing	Total excl. External Financing	Total incl. External Financing	Wage	Non-Wage Recurrent	Domestic Dev	External Financing	Total excl. External Financing	Total incl. External Financing	Wage	Non-Wage Recurrent	Domestic Dev	External Financing	Total excl. External Financing	Total incl. External Financing
**01 AGRO-INDUSTRIALIZATION**																		
010 Ministry of Agriculture, Animal Industry and Fisheries	22.415	44.590	166.920	482.582	**233.925**	**716.508**	24.656	60.197	233.688	302.882	**318.541**	**621.423**	24.656	60.197	233.688	302.955	**318.541**	**621.497**
011 Ministry of Local Government	0.139	0.171	0.000	0.000	**0.310**	**0.310**	0.152	0.231	0.000	0.000	**0.384**	**0.384**	0.152	0.231	0.000	0.000	**0.384**	**0.384**
015 Ministry of Trade, Industry and Co-operatives	0.000	12.044	0.000	0.000	**12.044**	**12.044**	0.000	16.260	0.000	0.000	**16.260**	**16.260**	0.000	16.260	0.000	0.000	**16.260**	**16.260**
019 Ministry of Water and Environment	1.441	0.049	99.804	0.000	**101.294**	**101.294**	1.586	0.066	139.726	0.000	**141.377**	**141.377**	1.586	0.066	139.726	0.000	**141.377**	**141.377**
021 Ministry of East African Community Affairs	0.000	0.306	0.000	0.000	**0.306**	**0.306**	0.000	0.413	0.000	0.000	**0.413**	**0.413**	0.000	0.413	0.000	0.000	**0.413**	**0.413**
108 National Planning Authority (NPA)	0.000	0.979	0.000	0.000	**0.979**	**0.979**	0.000	1.322	0.000	0.000	**1.322**	**1.322**	0.000	1.322	0.000	0.000	**1.322**	**1.322**
121 Dairy Development Authority (DDA)	4.872	10.086	0.912	0.000	**15.870**	**15.870**	5.359	13.616	1.277	0.000	**20.252**	**20.252**	5.359	13.616	1.277	0.000	**20.252**	**20.252**
122 Kampala Capital City Authority (KCCA)	0.000	0.428	0.000	0.000	**0.428**	**0.428**	0.000	0.578	0.000	0.000	**0.578**	**0.578**	0.000	0.578	0.000	0.000	**0.578**	**0.578**
125 National Animal Genetic Resource Centre and Data Bank (NAGRC&DB)	6.625	9.621	80.112	0.000	**96.358**	**96.358**	7.288	12.988	112.157	0.000	**132.432**	**132.432**	7.288	12.988	112.157	0.000	**132.432**	**132.432**
142 National Agricultural Research Organization (NARO)	44.827	32.546	43.164	0.000	**120.537**	**120.537**	49.310	43.937	60.430	0.000	**153.676**	**153.676**	49.310	43.937	60.430	0.000	**153.676**	**153.676**
152 National Agricultural Advisory Services (NAADS)	3.822	36.752	0.576	0.000	**41.150**	**41.150**	4.204	49.615	0.806	0.000	**54.625**	**54.625**	4.204	49.615	0.806	0.000	**54.625**	**54.625**
154 Uganda National Bureau of Standards (UNBS)	0.000	1.151	0.000	0.000	**1.151**	**1.151**	0.000	1.553	0.000	0.000	**1.553**	**1.553**	0.000	1.553	0.000	0.000	**1.553**	**1.553**
155 Cotton Development Organization	2.325	3.941	0.360	0.000	**6.627**	**6.627**	2.558	5.321	0.504	0.000	**8.383**	**8.383**	2.558	5.321	0.504	0.000	**8.383**	**8.383**
160 Uganda Coffee Development Authority (UCDA)	11.694	43.281	2.424	0.000	**57.399**	**57.399**	12.864	58.429	3.394	0.000	**74.686**	**74.686**	12.864	58.429	3.394	0.000	**74.686**	**74.686**
502 Uganda High Commission in the United Kingdom	0.000	0.000	0.000	0.000	**0.000**	**0.000**	0.000	0.000	0.000	0.000	**0.000**	**0.000**	0.000	0.000	0.000	0.000	**0.000**	**0.000**
504 Uganda High Commission in India, New Delhi	0.000	0.000	0.000	0.000	**0.000**	**0.000**	0.000	0.000	0.000	0.000	**0.000**	**0.000**	0.000	0.000	0.000	0.000	**0.000**	**0.000**
505 Uganda High Commission in Kenya, Nairobi	0.000	0.000	0.000	0.000	**0.000**	**0.000**	0.000	0.000	0.000	0.000	**0.000**	**0.000**	0.000	0.000	0.000	0.000	**0.000**	**0.000**
507 Uganda High Commission in Nigeria, Abuja	0.000	0.000	0.000	0.000	**0.000**	**0.000**	0.000	0.000	0.000	0.000	**0.000**	**0.000**	0.000	0.000	0.000	0.000	**0.000**	**0.000**

ANNEX 2: MEDIUM TERM EXPENDITURE FRAMEWORK (MTEF)2025/26 - 2027/28(Excl. Arrears and AIA)

Billion Uganda Shillings	FY2025/26 Budget Projections						FY2026/27 Budget Projections						FY2027/28 Budget Projections					
PROGRAMME/VOTE	Wage	Non-Wage Recurrent	Domestic Dev	External Financing	Total excl. External Financing	Total incl. External Financing	Wage	Non-Wage Recurrent	Domestic Dev	External Financing	Total excl. External Financing	Total incl. External Financing	Wage	Non-Wage Recurrent	Domestic Dev	External Financing	Total excl. External Financing	Total incl. External Financing
**01 AGRO-INDUSTRIALIZATION**																		
508 Uganda High Commission in South Africa, Pretoria	0.000	0.000	0.000	0.000	**0.000**	**0.000**	0.000	0.000	0.000	0.000	**0.000**	**0.000**	0.000	0.000	0.000	0.000	**0.000**	**0.000**
511 Uganda Embassy in Egypt, Cairo	0.000	0.000	0.000	0.000	**0.000**	**0.000**	0.000	0.000	0.000	0.000	**0.000**	**0.000**	0.000	0.000	0.000	0.000	**0.000**	**0.000**
512 Uganda Embassy in Ethiopia, Addis Ababa	0.000	0.000	0.000	0.000	**0.000**	**0.000**	0.000	0.000	0.000	0.000	**0.000**	**0.000**	0.000	0.000	0.000	0.000	**0.000**	**0.000**
513 Uganda Embassy in China, Beijing	0.000	0.000	0.000	0.000	**0.000**	**0.000**	0.000	0.000	0.000	0.000	**0.000**	**0.000**	0.000	0.000	0.000	0.000	**0.000**	**0.000**
514 Uganda Embassy in Switzerland, Geneva	0.000	0.000	0.000	0.000	**0.000**	**0.000**	0.000	0.000	0.000	0.000	**0.000**	**0.000**	0.000	0.000	0.000	0.000	**0.000**	**0.000**
515 Uganda Embassy in Japan, Tokyo	0.000	0.000	0.000	0.000	**0.000**	**0.000**	0.000	0.000	0.000	0.000	**0.000**	**0.000**	0.000	0.000	0.000	0.000	**0.000**	**0.000**
517 Uganda Embassy in Denmark, Copenhagen	0.000	0.000	0.000	0.000	**0.000**	**0.000**	0.000	0.000	0.000	0.000	**0.000**	**0.000**	0.000	0.000	0.000	0.000	**0.000**	**0.000**
518 Uganda Embassy in Belgium, Brussels	0.000	0.000	0.000	0.000	**0.000**	**0.000**	0.000	0.000	0.000	0.000	**0.000**	**0.000**	0.000	0.000	0.000	0.000	**0.000**	**0.000**
519 Uganda Embassy in Italy, Rome	0.000	0.000	0.000	0.000	**0.000**	**0.000**	0.000	0.000	0.000	0.000	**0.000**	**0.000**	0.000	0.000	0.000	0.000	**0.000**	**0.000**
520 Uganda Embassy in DRC, Kinshasa	0.000	0.000	0.000	0.000	**0.000**	**0.000**	0.000	0.000	0.000	0.000	**0.000**	**0.000**	0.000	0.000	0.000	0.000	**0.000**	**0.000**
523 Uganda Embassy in Germany, Berlin	0.000	0.000	0.000	0.000	**0.000**	**0.000**	0.000	0.000	0.000	0.000	**0.000**	**0.000**	0.000	0.000	0.000	0.000	**0.000**	**0.000**
524 Uganda Embassy in Iran, Tehran	0.000	0.000	0.000	0.000	**0.000**	**0.000**	0.000	0.000	0.000	0.000	**0.000**	**0.000**	0.000	0.000	0.000	0.000	**0.000**	**0.000**
525 Uganda Embassy in Russia, Moscow	0.000	0.000	0.000	0.000	**0.000**	**0.000**	0.000	0.000	0.000	0.000	**0.000**	**0.000**	0.000	0.000	0.000	0.000	**0.000**	**0.000**
526 Uganda Embassy in Australia, Canberra	0.000	0.000	0.000	0.000	**0.000**	**0.000**	0.000	0.000	0.000	0.000	**0.000**	**0.000**	0.000	0.000	0.000	0.000	**0.000**	**0.000**
527 Uganda Embassy in South Sudan, Juba	0.000	0.000	0.000	0.000	**0.000**	**0.000**	0.000	0.000	0.000	0.000	**0.000**	**0.000**	0.000	0.000	0.000	0.000	**0.000**	**0.000**
528 Uganda Embassy in United Arab Emirates, Abudhabi	0.000	0.000	0.000	0.000	**0.000**	**0.000**	0.000	0.000	0.000	0.000	**0.000**	**0.000**	0.000	0.000	0.000	0.000	**0.000**	**0.000**
529 Uganda Embassy in Burundi, Bujumbura	0.000	0.000	0.000	0.000	**0.000**	**0.000**	0.000	0.000	0.000	0.000	**0.000**	**0.000**	0.000	0.000	0.000	0.000	**0.000**	**0.000**
530 Uganda Consulate in China, Guangzhou	0.000	0.000	0.000	0.000	**0.000**	**0.000**	0.000	0.000	0.000	0.000	**0.000**	**0.000**	0.000	0.000	0.000	0.000	**0.000**	**0.000**
531 Uganda Embassy in Turkey, Ankara	0.000	0.000	0.000	0.000	**0.000**	**0.000**	0.000	0.000	0.000	0.000	**0.000**	**0.000**	0.000	0.000	0.000	0.000	**0.000**	**0.000**
534 Uganda Consulate in Kenya, Mombasa	0.000	0.000	0.000	0.000	**0.000**	**0.000**	0.000	0.000	0.000	0.000	**0.000**	**0.000**	0.000	0.000	0.000	0.000	**0.000**	**0.000**
535 Uganda Embassy in Algeria, Algiers	0.000	0.000	0.000	0.000	**0.000**	**0.000**	0.000	0.000	0.000	0.000	**0.000**	**0.000**	0.000	0.000	0.000	0.000	**0.000**	**0.000**
536 Uganda Embassy in Qatar, Doha	0.000	0.000	0.000	0.000	**0.000**	**0.000**	0.000	0.000	0.000	0.000	**0.000**	**0.000**	0.000	0.000	0.000	0.000	**0.000**	**0.000**
601 Local Governments 01	139.442	0.000	0.000	0.000	**139.442**	**139.442**	153.386	0.000	0.000	0.000	**153.386**	**153.386**	153.386	0.000	0.000	0.000	**153.386**	**153.386**

ANNEX 2: MEDIUM TERM EXPENDITURE FRAMEWORK (MTEF)2025/26 - 2027/28(Excl. Arrears and AIA)

Billion Uganda Shillings	FY2025/26 Budget Projections						FY2026/27 Budget Projections						FY2027/28 Budget Projections					
PROGRAMME/VOTE	Wage	Non-Wage Recurrent	Domestic Dev	External Financing	Total excl. External Financing	Total incl. External Financing	Wage	Non-Wage Recurrent	Domestic Dev	External Financing	Total excl. External Financing	Total incl. External Financing	Wage	Non-Wage Recurrent	Domestic Dev	External Financing	Total excl. External Financing	Total incl. External Financing
**Sub Total For: AGRO-INDUSTRIALIZATION**	237.602	195.945	394.272	482.582	**827.819**	**1,310.401**	261.362	264.526	551.981	302.882	**1,077.869**	**1,380.750**	261.362	264.526	551.981	302.955	**1,077.869**	**1,380.824**
**02 MINERAL DEVELOPMENT**																		
017 Ministry of Energy and Mineral Development	6.353	13.464	26.400	0.000	**46.217**	**46.217**	6.988	18.176	36.960	0.000	**62.124**	**62.124**	6.988	18.176	36.960	0.000	**62.124**	**62.124**
108 National Planning Authority (NPA)	0.000	0.000	0.000	0.000	**0.000**	**0.000**	0.000	0.000	0.000	0.000	**0.000**	**0.000**	0.000	0.000	0.000	0.000	**0.000**	**0.000**
530 Uganda Consulate in China, Guangzhou	0.000	0.000	0.000	0.000	**0.000**	**0.000**	0.000	0.000	0.000	0.000	**0.000**	**0.000**	0.000	0.000	0.000	0.000	**0.000**	**0.000**
**Sub Total For: MINERAL DEVELOPMENT**	6.353	13.464	26.400	0.000	**46.217**	**46.217**	6.988	18.176	36.960	0.000	**62.124**	**62.124**	6.988	18.176	36.960	0.000	**62.124**	**62.124**
**03 SUSTAINABLE PETROLEUM DEVELOPMENT**																		
006 Ministry of Foreign Affairs	0.000	0.851	0.000	0.000	**0.851**	**0.851**	0.000	1.148	0.000	0.000	**1.148**	**1.148**	0.000	1.148	0.000	0.000	**1.148**	**1.148**
008 Ministry of Finance, Planning and Economic Development	0.000	344.372	0.000	0.000	**344.372**	**344.372**	0.000	464.903	0.000	0.000	**464.903**	**464.903**	0.000	464.903	0.000	0.000	**464.903**	**464.903**
013 Ministry of Education and Sports	0.000	1.224	0.000	0.000	**1.224**	**1.224**	0.000	1.652	0.000	0.000	**1.652**	**1.652**	0.000	1.652	0.000	0.000	**1.652**	**1.652**
017 Ministry of Energy and Mineral Development	4.274	36.702	151.632	909.642	**192.607**	**1,102.249**	4.701	49.547	212.285	431.221	**266.533**	**697.754**	4.701	49.547	212.285	0.000	**266.533**	**266.533**
139 Petroleum Authority of Uganda (PAU)	33.169	46.151	33.590	0.000	**112.911**	**112.911**	36.486	62.304	47.027	0.000	**145.817**	**145.817**	36.486	62.304	47.027	0.000	**145.817**	**145.817**
150 National Environment Management Authority (NEMA)	0.000	0.612	0.000	0.000	**0.612**	**0.612**	0.000	0.826	0.000	0.000	**0.826**	**0.826**	0.000	0.826	0.000	0.000	**0.826**	**0.826**
154 Uganda National Bureau of Standards (UNBS)	0.000	1.102	0.000	0.000	**1.102**	**1.102**	0.000	1.487	0.000	0.000	**1.487**	**1.487**	0.000	1.487	0.000	0.000	**1.487**	**1.487**
506 Uganda High Commission in Tanzania, Dar es Salaam	0.000	0.305	0.000	0.000	**0.305**	**0.305**	0.000	0.305	0.000	0.000	**0.305**	**0.305**	0.000	0.305	0.000	0.000	**0.305**	**0.305**
**Sub Total For: SUSTAINABLE PETROLEUM DEVELOPMENT**	37.443	431.318	185.222	909.642	**653.983**	**1,563.625**	41.187	582.173	259.311	431.221	**882.671**	**1,313.893**	41.187	582.173	259.311	0.000	**882.671**	**882.671**
**04 MANUFACTURING**																		
006 Ministry of Foreign Affairs	0.000	0.000	0.000	0.000	**0.000**	**0.000**	0.000	0.000	0.000	0.000	**0.000**	**0.000**	0.000	0.000	0.000	0.000	**0.000**	**0.000**
015 Ministry of Trade, Industry and Co-operatives	2.340	39.713	0.271	0.000	**42.324**	**42.324**	2.574	53.613	0.379	0.000	**56.566**	**56.566**	2.574	53.613	0.379	0.000	**56.566**	**56.566**
138 Uganda Investment Authority (UIA)	0.000	0.000	0.000	0.000	**0.000**	**0.000**	0.000	0.000	0.000	0.000	**0.000**	**0.000**	0.000	0.000	0.000	0.000	**0.000**	**0.000**

ANNEX 2: MEDIUM TERM EXPENDITURE FRAMEWORK (MTEF)2025/26 - 2027/28(Excl. Arrears and AIA)

Billion Uganda Shillings	FY2025/26 Budget Projections						FY2026/27 Budget Projections						FY2027/28 Budget Projections					
PROGRAMME/VOTE	Wage	Non-Wage Recurrent	Domestic Dev	External Financing	Total excl. External Financing	Total incl. External Financing	Wage	Non-Wage Recurrent	Domestic Dev	External Financing	Total excl. External Financing	Total incl. External Financing	Wage	Non-Wage Recurrent	Domestic Dev	External Financing	Total excl. External Financing	Total incl. External Financing
**04 MANUFACTURING**																		
154 Uganda National Bureau of Standards (UNBS)	0.000	0.108	0.000	0.000	**0.108**	**0.108**	0.000	0.145	0.000	0.000	**0.145**	**0.145**	0.000	0.145	0.000	0.000	**0.145**	**0.145**
506 Uganda High Commission in Tanzania, Dar es Salaam	0.000	0.000	0.000	0.000	**0.000**	**0.000**	0.000	0.000	0.000	0.000	**0.000**	**0.000**	0.000	0.000	0.000	0.000	**0.000**	**0.000**
508 Uganda High Commission in South Africa, Pretoria	0.000	0.000	0.000	0.000	**0.000**	**0.000**	0.000	0.000	0.000	0.000	**0.000**	**0.000**	0.000	0.000	0.000	0.000	**0.000**	**0.000**
515 Uganda Embassy in Japan, Tokyo	0.000	0.000	0.000	0.000	**0.000**	**0.000**	0.000	0.000	0.000	0.000	**0.000**	**0.000**	0.000	0.000	0.000	0.000	**0.000**	**0.000**
516 Uganda Embassy in Saudi Arabia, Riyadh	0.000	0.000	0.000	0.000	**0.000**	**0.000**	0.000	0.000	0.000	0.000	**0.000**	**0.000**	0.000	0.000	0.000	0.000	**0.000**	**0.000**
518 Uganda Embassy in Belgium, Brussels	0.000	0.000	0.000	0.000	**0.000**	**0.000**	0.000	0.000	0.000	0.000	**0.000**	**0.000**	0.000	0.000	0.000	0.000	**0.000**	**0.000**
519 Uganda Embassy in Italy, Rome	0.000	0.000	0.000	0.000	**0.000**	**0.000**	0.000	0.000	0.000	0.000	**0.000**	**0.000**	0.000	0.000	0.000	0.000	**0.000**	**0.000**
520 Uganda Embassy in DRC, Kinshasa	0.000	0.000	0.000	0.000	**0.000**	**0.000**	0.000	0.000	0.000	0.000	**0.000**	**0.000**	0.000	0.000	0.000	0.000	**0.000**	**0.000**
525 Uganda Embassy in Russia, Moscow	0.000	0.000	0.000	0.000	**0.000**	**0.000**	0.000	0.000	0.000	0.000	**0.000**	**0.000**	0.000	0.000	0.000	0.000	**0.000**	**0.000**
527 Uganda Embassy in South Sudan, Juba	0.000	0.000	0.000	0.000	**0.000**	**0.000**	0.000	0.000	0.000	0.000	**0.000**	**0.000**	0.000	0.000	0.000	0.000	**0.000**	**0.000**
529 Uganda Embassy in Burundi, Bujumbura	0.000	0.000	0.000	0.000	**0.000**	**0.000**	0.000	0.000	0.000	0.000	**0.000**	**0.000**	0.000	0.000	0.000	0.000	**0.000**	**0.000**
530 Uganda Consulate in China, Guangzhou	0.000	0.000	0.000	0.000	**0.000**	**0.000**	0.000	0.000	0.000	0.000	**0.000**	**0.000**	0.000	0.000	0.000	0.000	**0.000**	**0.000**
532 Uganda Embassy in Somalia, Mogadishu	0.000	0.000	0.000	0.000	**0.000**	**0.000**	0.000	0.000	0.000	0.000	**0.000**	**0.000**	0.000	0.000	0.000	0.000	**0.000**	**0.000**
533 Uganda Embassy in Malaysia, Kuala Lumpur	0.000	0.000	0.000	0.000	**0.000**	**0.000**	0.000	0.000	0.000	0.000	**0.000**	**0.000**	0.000	0.000	0.000	0.000	**0.000**	**0.000**
**Sub Total For: MANUFACTURING**	2.340	39.821	0.271	0.000	**42.431**	**42.431**	2.574	53.758	0.379	0.000	**56.711**	**56.711**	2.574	53.758	0.379	0.000	**56.711**	**56.711**
**05 TOURISM DEVELOPMENT**																		
022 Ministry of Tourism, Wildlife and Antiquities	2.738	36.696	63.168	0.000	**102.602**	**102.602**	3.012	49.539	88.435	0.000	**140.986**	**140.986**	3.012	49.539	88.435	0.000	**140.986**	**140.986**
117 Uganda Tourism Board (UTB)	2.143	2.876	0.120	0.000	**5.139**	**5.139**	2.357	3.883	0.168	0.000	**6.408**	**6.408**	2.357	3.883	0.168	0.000	**6.408**	**6.408**
122 Kampala Capital City Authority (KCCA)	0.000	0.000	0.000	0.000	**0.000**	**0.000**	0.000	0.000	0.000	0.000	**0.000**	**0.000**	0.000	0.000	0.000	0.000	**0.000**	**0.000**
501 Uganda Mission at the United Nations, New York	0.000	0.000	0.000	0.000	**0.000**	**0.000**	0.000	0.000	0.000	0.000	**0.000**	**0.000**	0.000	0.000	0.000	0.000	**0.000**	**0.000**
505 Uganda High Commission in Kenya, Nairobi	0.000	0.000	0.000	0.000	**0.000**	**0.000**	0.000	0.000	0.000	0.000	**0.000**	**0.000**	0.000	0.000	0.000	0.000	**0.000**	**0.000**

ANNEX 2: MEDIUM TERM EXPENDITURE FRAMEWORK (MTEF)2025/26 - 2027/28(Excl. Arrears and AIA)

Billion Uganda Shillings	FY2025/26 Budget Projections						FY2026/27 Budget Projections						FY2027/28 Budget Projections					
PROGRAMME/VOTE	Wage	Non-Wage Recurrent	Domestic Dev	External Financing	Total excl. External Financing	Total incl. External Financing	Wage	Non-Wage Recurrent	Domestic Dev	External Financing	Total excl. External Financing	Total incl. External Financing	Wage	Non-Wage Recurrent	Domestic Dev	External Financing	Total excl. External Financing	Total incl. External Financing
**05 TOURISM DEVELOPMENT**																		
508 Uganda High Commission in South Africa, Pretoria	0.000	0.000	0.000	0.000	0.000	0.000	0.000	0.000	0.000	0.000	0.000	0.000	0.000	0.000	0.000	0.000	0.000	0.000
509 Uganda High Commission in Rwanda, Kigali	0.000	0.000	0.000	0.000	0.000	0.000	0.000	0.000	0.000	0.000	0.000	0.000	0.000	0.000	0.000	0.000	0.000	0.000
513 Uganda Embassy in China, Beijing	0.000	0.000	0.000	0.000	0.000	0.000	0.000	0.000	0.000	0.000	0.000	0.000	0.000	0.000	0.000	0.000	0.000	0.000
515 Uganda Embassy in Japan, Tokyo	0.000	0.000	0.000	0.000	0.000	0.000	0.000	0.000	0.000	0.000	0.000	0.000	0.000	0.000	0.000	0.000	0.000	0.000
516 Uganda Embassy in Saudi Arabia, Riyadh	0.000	0.000	0.000	0.000	0.000	0.000	0.000	0.000	0.000	0.000	0.000	0.000	0.000	0.000	0.000	0.000	0.000	0.000
517 Uganda Embassy in Denmark, Copenhagen	0.000	0.000	0.000	0.000	0.000	0.000	0.000	0.000	0.000	0.000	0.000	0.000	0.000	0.000	0.000	0.000	0.000	0.000
518 Uganda Embassy in Belgium, Brussels	0.000	0.000	0.000	0.000	0.000	0.000	0.000	0.000	0.000	0.000	0.000	0.000	0.000	0.000	0.000	0.000	0.000	0.000
519 Uganda Embassy in Italy, Rome	0.000	0.000	0.000	0.000	0.000	0.000	0.000	0.000	0.000	0.000	0.000	0.000	0.000	0.000	0.000	0.000	0.000	0.000
522 Uganda Embassy in France, Paris	0.000	0.000	0.000	0.000	0.000	0.000	0.000	0.000	0.000	0.000	0.000	0.000	0.000	0.000	0.000	0.000	0.000	0.000
523 Uganda Embassy in Germany, Berlin	0.000	0.000	0.000	0.000	0.000	0.000	0.000	0.000	0.000	0.000	0.000	0.000	0.000	0.000	0.000	0.000	0.000	0.000
524 Uganda Embassy in Iran, Tehran	0.000	0.000	0.000	0.000	0.000	0.000	0.000	0.000	0.000	0.000	0.000	0.000	0.000	0.000	0.000	0.000	0.000	0.000
525 Uganda Embassy in Russia, Moscow	0.000	0.000	0.000	0.000	0.000	0.000	0.000	0.000	0.000	0.000	0.000	0.000	0.000	0.000	0.000	0.000	0.000	0.000
526 Uganda Embassy in Australia, Canberra	0.000	0.000	0.000	0.000	0.000	0.000	0.000	0.000	0.000	0.000	0.000	0.000	0.000	0.000	0.000	0.000	0.000	0.000
527 Uganda Embassy in South Sudan, Juba	0.000	0.000	0.000	0.000	0.000	0.000	0.000	0.000	0.000	0.000	0.000	0.000	0.000	0.000	0.000	0.000	0.000	0.000
529 Uganda Embassy in Burundi, Bujumbura	0.000	0.000	0.000	0.000	0.000	0.000	0.000	0.000	0.000	0.000	0.000	0.000	0.000	0.000	0.000	0.000	0.000	0.000
530 Uganda Consulate in China, Guangzhou	0.000	0.000	0.000	0.000	0.000	0.000	0.000	0.000	0.000	0.000	0.000	0.000	0.000	0.000	0.000	0.000	0.000	0.000
531 Uganda Embassy in Turkey, Ankara	0.000	0.000	0.000	0.000	0.000	0.000	0.000	0.000	0.000	0.000	0.000	0.000	0.000	0.000	0.000	0.000	0.000	0.000
533 Uganda Embassy in Malaysia, Kuala Lumpur	0.000	0.000	0.000	0.000	0.000	0.000	0.000	0.000	0.000	0.000	0.000	0.000	0.000	0.000	0.000	0.000	0.000	0.000
534 Uganda Consulate in Kenya, Mombasa	0.000	0.000	0.000	0.000	0.000	0.000	0.000	0.000	0.000	0.000	0.000	0.000	0.000	0.000	0.000	0.000	0.000	0.000
**Sub Total For: TOURISM DEVELOPMENT**	4.881	39.572	63.288	0.000	107.741	107.741	5.369	53.422	88.603	0.000	147.395	147.395	5.369	53.422	88.603	0.000	147.395	147.395
**06 NATURAL RESOURCES, ENVIRONMENT, CLIMATE CHANGE, LAND AND WATER**																		
003 Office of the Prime Minister	0.389	9.400	11.676	0.000	21.466	21.466	0.428	12.690	16.346	0.000	29.465	29.465	0.428	12.690	16.346	0.000	29.465	29.465
011 Ministry of Local Government	0.000	0.000	0.000	0.000	0.000	0.000	0.000	0.000	0.000	0.000	0.000	0.000	0.000	0.000	0.000	0.000	0.000	0.000

ANNEX 2: MEDIUM TERM EXPENDITURE FRAMEWORK (MTEF)2025/26 - 2027/28(Excl. Arrears and AIA)

Billion Uganda Shillings	FY2025/26 Budget Projections						FY2026/27 Budget Projections						FY2027/28 Budget Projections					
PROGRAMME/VOTE	Wage	Non-Wage Recurrent	Domestic Dev	External Financing	Total excl. External Financing	Total incl. External Financing	Wage	Non-Wage Recurrent	Domestic Dev	External Financing	Total excl. External Financing	Total incl. External Financing	Wage	Non-Wage Recurrent	Domestic Dev	External Financing	Total excl. External Financing	Total incl. External Financing
**06 NATURAL RESOURCES, ENVIRONMENT, CLIMATE CHANGE, LAND AND WATER**																		
012 Ministry of Lands, Housing & Urban Development	10.055	10.502	11.736	0.000	**32.293**	**32.293**	11.061	14.178	16.430	0.000	**41.669**	**41.669**	11.061	14.178	16.430	0.000	**41.669**	**41.669**
019 Ministry of Water and Environment	11.735	19.082	102.264	84.835	**133.081**	**217.916**	12.908	25.761	143.170	0.000	**181.839**	**181.839**	12.908	25.761	143.170	0.000	**181.839**	**181.839**
108 National Planning Authority (NPA)	0.000	0.000	0.000	0.000	**0.000**	**0.000**	0.000	0.000	0.000	0.000	**0.000**	**0.000**	0.000	0.000	0.000	0.000	**0.000**	**0.000**
109 Uganda National Meteorological Authority (UNMA)	8.562	4.492	7.272	0.000	**20.326**	**20.326**	9.418	6.064	10.181	0.000	**25.663**	**25.663**	9.418	6.064	10.181	0.000	**25.663**	**25.663**
122 Kampala Capital City Authority (KCCA)	0.000	21.885	0.408	0.000	**22.293**	**22.293**	0.000	29.545	0.571	0.000	**30.116**	**30.116**	0.000	29.545	0.571	0.000	**30.116**	**30.116**
150 National Environment Management Authority (NEMA)	7.764	11.053	4.824	0.000	**23.641**	**23.641**	8.540	14.921	6.754	0.000	**30.215**	**30.215**	8.540	14.921	6.754	0.000	**30.215**	**30.215**
156 Uganda Land Commission (ULC)	1.227	1.493	30.720	0.000	**33.440**	**33.440**	1.350	2.016	43.008	0.000	**46.374**	**46.374**	1.350	2.016	43.008	0.000	**46.374**	**46.374**
157 National Forestry Authority (NFA)	9.547	15.300	6.780	0.000	**31.627**	**31.627**	10.501	20.655	9.492	0.000	**40.648**	**40.648**	10.501	20.655	9.492	0.000	**40.648**	**40.648**
505 Uganda High Commission in Kenya, Nairobi	0.000	0.000	0.000	0.000	**0.000**	**0.000**	0.000	0.000	0.000	0.000	**0.000**	**0.000**	0.000	0.000	0.000	0.000	**0.000**	**0.000**
606 Local Governments 06	0.000	4.284	0.000	0.000	**4.284**	**4.284**	0.000	5.783	0.000	0.000	**5.783**	**5.783**	0.000	5.783	0.000	0.000	**5.783**	**5.783**
**Sub Total For: NATURAL RESOURCES, ENVIRONMENT, CLIMATE CHANGE, LAND AND WATER**	49.279	97.492	175.680	84.835	**322.451**	**407.286**	54.207	131.614	245.952	0.000	**431.773**	**431.773**	54.207	131.614	245.952	0.000	**431.773**	**431.773**
**07 PRIVATE SECTOR DEVELOPMENT**																		
008 Ministry of Finance, Planning and Economic Development	0.347	1,731.894	2.683	359.808	**1,734.925**	**2,094.733**	0.382	2,338.057	3.756	38.194	**2,342.196**	**2,380.389**	0.382	2,338.057	3.756	0.000	**2,342.196**	**2,342.196**
015 Ministry of Trade, Industry and Co-operatives	1.326	1.217	0.000	0.000	**2.543**	**2.543**	1.459	1.643	0.000	0.000	**3.102**	**3.102**	1.459	1.643	0.000	0.000	**3.102**	**3.102**
021 Ministry of East African Community Affairs	0.000	2.119	0.000	0.000	**2.119**	**2.119**	0.000	2.860	0.000	0.000	**2.860**	**2.860**	0.000	2.860	0.000	0.000	**2.860**	**2.860**
108 National Planning Authority (NPA)	0.000	0.306	0.000	0.000	**0.306**	**0.306**	0.000	0.413	0.000	0.000	**0.413**	**0.413**	0.000	0.413	0.000	0.000	**0.413**	**0.413**
119 Uganda Registration Services Bureau (URSB)	5.023	2.845	0.320	0.000	**8.189**	**8.189**	5.525	3.841	0.449	0.000	**9.815**	**9.815**	5.525	3.841	0.449	0.000	**9.815**	**9.815**
123 National Lotteries and Gaming Regulatory Board	0.000	0.599	0.000	0.000	**0.599**	**0.599**	0.000	0.809	0.000	0.000	**0.809**	**0.809**	0.000	0.809	0.000	0.000	**0.809**	**0.809**

ANNEX 2: MEDIUM TERM EXPENDITURE FRAMEWORK (MTEF)2025/26 - 2027/28(Excl. Arrears and AIA)

Billion Uganda Shillings	FY2025/26 Budget Projections						FY2026/27 Budget Projections						FY2027/28 Budget Projections					
PROGRAMME/VOTE	Wage	Non-Wage Recurrent	Domestic Dev	External Financing	Total excl. External Financing	Total incl. External Financing	Wage	Non-Wage Recurrent	Domestic Dev	External Financing	Total excl. External Financing	Total incl. External Financing	Wage	Non-Wage Recurrent	Domestic Dev	External Financing	Total excl. External Financing	Total incl. External Financing
**07 PRIVATE SECTOR DEVELOPMENT**																		
136 Uganda Export Promotion Board (UEPB)	2.958	7.140	0.044	0.000	**10.143**	**10.143**	3.254	9.639	0.062	0.000	**12.955**	**12.955**	3.254	9.639	0.062	0.000	**12.955**	**12.955**
138 Uganda Investment Authority (UIA)	7.892	10.069	1.685	0.000	**19.646**	**19.646**	8.681	13.593	2.359	0.000	**24.633**	**24.633**	8.681	13.593	2.359	0.000	**24.633**	**24.633**
140 Capital Markets Authority	4.013	6.237	0.000	0.000	**10.250**	**10.250**	4.415	8.419	0.000	0.000	**12.834**	**12.834**	4.415	8.419	0.000	0.000	**12.834**	**12.834**
153 Public Procurement & Disposal of Public Assets (PPDA)	1.085	0.471	0.000	0.000	**1.556**	**1.556**	1.194	0.636	0.000	0.000	**1.830**	**1.830**	1.194	0.636	0.000	0.000	**1.830**	**1.830**
154 Uganda National Bureau of Standards (UNBS)	27.553	9.166	6.061	0.000	**42.781**	**42.781**	30.309	12.374	8.486	0.000	**51.169**	**51.169**	30.309	12.374	8.486	0.000	**51.169**	**51.169**
161 Uganda Free Zones Authority	2.764	3.976	6.491	0.000	**13.230**	**13.230**	3.040	5.367	9.087	0.000	**17.494**	**17.494**	3.040	5.367	9.087	0.000	**17.494**	**17.494**
162 Uganda Microfinance Regulatory Authority	2.507	9.204	2.160	0.000	**13.871**	**13.871**	2.758	12.425	3.024	0.000	**18.207**	**18.207**	2.758	12.425	3.024	0.000	**18.207**	**18.207**
163 Uganda Retirement Benefits Regulatory Authority	6.693	9.585	0.000	0.000	**16.277**	**16.277**	7.362	12.939	0.000	0.000	**20.301**	**20.301**	7.362	12.939	0.000	0.000	**20.301**	**20.301**
506 Uganda High Commission in Tanzania, Dar es Salaam	0.000	0.200	0.000	0.000	**0.200**	**0.200**	0.000	0.200	0.000	0.000	**0.200**	**0.200**	0.000	0.200	0.000	0.000	**0.200**	**0.200**
510 Uganda Embassy in the United States, Washington	0.000	0.790	0.000	0.000	**0.790**	**0.790**	0.000	0.790	0.000	0.000	**0.790**	**0.790**	0.000	0.790	0.000	0.000	**0.790**	**0.790**
515 Uganda Embassy in Japan, Tokyo	0.000	0.021	0.000	0.000	**0.021**	**0.021**	0.000	0.021	0.000	0.000	**0.021**	**0.021**	0.000	0.021	0.000	0.000	**0.021**	**0.021**
523 Uganda Embassy in Germany, Berlin	0.000	0.085	0.000	0.000	**0.085**	**0.085**	0.000	0.085	0.000	0.000	**0.085**	**0.085**	0.000	0.085	0.000	0.000	**0.085**	**0.085**
527 Uganda Embassy in South Sudan, Juba	0.000	0.150	0.000	0.000	**0.150**	**0.150**	0.000	0.150	0.000	0.000	**0.150**	**0.150**	0.000	0.150	0.000	0.000	**0.150**	**0.150**
531 Uganda Embassy in Turkey, Ankara	0.000	0.210	0.000	0.000	**0.210**	**0.210**	0.000	0.210	0.000	0.000	**0.210**	**0.210**	0.000	0.210	0.000	0.000	**0.210**	**0.210**
532 Uganda Embassy in Somalia, Mogadishu	0.000	0.050	0.000	0.000	**0.050**	**0.050**	0.000	0.050	0.000	0.000	**0.050**	**0.050**	0.000	0.050	0.000	0.000	**0.050**	**0.050**
607 Local Governments 07	0.000	2.732	0.000	0.000	**2.732**	**2.732**	0.000	3.689	0.000	0.000	**3.689**	**3.689**	0.000	3.689	0.000	0.000	**3.689**	**3.689**
**Sub Total For: PRIVATE SECTOR DEVELOPMENT**	62.161	1,799.067	19.445	359.808	**1,880.673**	**2,240.481**	68.377	2,428.213	27.223	38.194	**2,523.813**	**2,562.007**	68.377	2,428.213	27.223	0.000	**2,523.813**	**2,523.813**
**08 SUSTAINABLE ENERGY DEVELOPMENT**																		
005 Ministry of Public Service	0.000	0.612	0.000	0.000	**0.612**	**0.612**	0.000	0.826	0.000	0.000	**0.826**	**0.826**	0.000	0.826	0.000	0.000	**0.826**	**0.826**
006 Ministry of Foreign Affairs	0.000	0.612	0.000	0.000	**0.612**	**0.612**	0.000	0.826	0.000	0.000	**0.826**	**0.826**	0.000	0.826	0.000	0.000	**0.826**	**0.826**
007 Ministry of Justice and Constitutional Affairs	0.000	1.444	0.000	0.000	**1.444**	**1.444**	0.000	1.950	0.000	0.000	**1.950**	**1.950**	0.000	1.950	0.000	0.000	**1.950**	**1.950**

ANNEX 2: MEDIUM TERM EXPENDITURE FRAMEWORK (MTEF)2025/26 - 2027/28(Excl. Arrears and AIA)

Billion Uganda Shillings	FY2025/26 Budget Projections						FY2026/27 Budget Projections						FY2027/28 Budget Projections					
PROGRAMME/VOTE	Wage	Non-Wage Recurrent	Domestic Dev	External Financing	Total excl. External Financing	Total incl. External Financing	Wage	Non-Wage Recurrent	Domestic Dev	External Financing	Total excl. External Financing	Total incl. External Financing	Wage	Non-Wage Recurrent	Domestic Dev	External Financing	Total excl. External Financing	Total incl. External Financing
**08 SUSTAINABLE ENERGY DEVELOPMENT**																		
008 Ministry of Finance, Planning and Economic Development	0.000	1.334	0.000	0.000	1.334	1.334	0.000	1.801	0.000	0.000	1.801	1.801	0.000	1.801	0.000	0.000	1.801	1.801
012 Ministry of Lands, Housing & Urban Development	0.000	0.000	0.600	0.000	0.600	0.600	0.000	0.000	0.840	0.000	0.840	0.840	0.000	0.000	0.840	0.000	0.840	0.840
017 Ministry of Energy and Mineral Development	18.249	74.477	554.148	1,398.196	646.873	2,045.069	20.074	101.808	775.807	1,169.630	897.688	2,067.318	20.074	101.808	775.807	271.941	897.688	1,169.629
150 National Environment Management Authority (NEMA)	0.000	0.000	3.000	0.000	3.000	3.000	0.000	0.000	4.200	0.000	4.200	4.200	0.000	0.000	4.200	0.000	4.200	4.200
154 Uganda National Bureau of Standards (UNBS)	0.000	4.100	1.920	0.000	6.020	6.020	0.000	5.536	2.688	0.000	8.224	8.224	0.000	5.536	2.688	0.000	8.224	8.224
**Sub Total For: SUSTAINABLE ENERGY DEVELOPMENT**	18.249	82.579	559.668	1,398.196	660.496	2,058.692	20.074	112.747	783.535	1,169.630	916.355	2,085.985	20.074	112.747	783.535	271.941	916.355	1,188.296
**09 INTEGRATED TRANSPORT INFRASTRUCTURE AND SERVICES**																		
016 Ministry of Works and Transport	15.891	110.435	228.264	2,598.792	354.590	2,953.382	17.480	149.088	319.570	2,314.257	486.137	2,800.394	17.480	149.088	319.570	0.000	486.137	486.137
113 Uganda National Roads Authority (UNRA)	82.126	49.115	708.096	3,509.735	839.338	4,349.073	90.339	66.306	991.334	3,938.554	1,147.979	5,086.534	90.339	66.306	991.334	2,184.130	1,147.979	3,332.109
118 Uganda Road Fund (URF)	3.081	569.509	0.000	0.000	572.590	572.590	3.389	768.837	0.000	0.000	772.226	772.226	3.389	768.837	0.000	0.000	772.226	772.226
122 Kampala Capital City Authority (KCCA)	0.000	0.000	12.000	401.578	12.000	413.578	0.000	0.000	16.800	284.655	16.800	301.455	0.000	0.000	16.800	181.601	16.800	198.401
609 Local Governments 09	0.000	0.000	33.184	0.000	33.184	33.184	0.000	0.000	46.457	0.000	46.457	46.457	0.000	0.000	46.457	0.000	46.457	46.457
**Sub Total For: INTEGRATED TRANSPORT INFRASTRUCTURE AND SERVICES**	101.098	729.060	981.544	6,510.105	1,811.701	8,321.807	111.208	984.231	1,374.161	6,537.466	2,469.600	9,007.066	111.208	984.231	1,374.161	2,365.730	2,469.600	4,835.330
**10 SUSTAINABLE URBANISATION AND HOUSING**																		
011 Ministry of Local Government	1.283	0.000	0.000	0.000	1.283	1.283	1.412	0.000	0.000	0.000	1.412	1.412	1.412	0.000	0.000	0.000	1.412	1.412
012 Ministry of Lands, Housing & Urban Development	2.873	18.690	6.720	0.000	28.284	28.284	3.161	25.232	9.408	0.000	37.801	37.801	3.161	25.232	9.408	0.000	37.801	37.801
016 Ministry of Works and Transport	1.155	1.224	0.000	0.000	2.379	2.379	1.271	1.652	0.000	0.000	2.923	2.923	1.271	1.652	0.000	0.000	2.923	2.923
023 Ministry of Kampala Capital City and Metropolitan Affairs	0.000	2.448	0.000	0.000	2.448	2.448	0.000	3.305	0.000	0.000	3.305	3.305	0.000	3.305	0.000	0.000	3.305	3.305

ANNEX 2: MEDIUM TERM EXPENDITURE FRAMEWORK (MTEF)2025/26 - 2027/28(Excl. Arrears and AIA)

Billion Uganda Shillings	FY2025/26 Budget Projections						FY2026/27 Budget Projections						FY2027/28 Budget Projections					
PROGRAMME/VOTE	Wage	Non-Wage Recurrent	Domestic Dev	External Financing	Total excl. External Financing	Total incl. External Financing	Wage	Non-Wage Recurrent	Domestic Dev	External Financing	Total excl. External Financing	Total incl. External Financing	Wage	Non-Wage Recurrent	Domestic Dev	External Financing	Total excl. External Financing	Total incl. External Financing
**10 SUSTAINABLE URBANISATION AND HOUSING**																		
161 Uganda Free Zones Authority	0.000	0.649	0.000	0.000	**0.649**	**0.649**	0.000	0.876	0.000	0.000	**0.876**	**0.876**	0.000	0.876	0.000	0.000	**0.876**	**0.876**
610 Local Governments 10	0.000	0.000	0.000	0.000	**0.000**	**0.000**	0.000	0.000	0.000	0.000	**0.000**	**0.000**	0.000	0.000	0.000	0.000	**0.000**	**0.000**
**Sub Total For: SUSTAINABLE URBANISATION AND HOUSING**	5.312	23.011	6.720	0.000	**35.043**	**35.043**	5.843	31.065	9.408	0.000	**46.316**	**46.316**	5.843	31.065	9.408	0.000	**46.316**	**46.316**
**11 DIGITAL TRANSFORMATION**																		
020 Ministry of ICT and National Guidance	6.596	25.515	23.457	0.000	**55.569**	**55.569**	7.256	34.446	32.840	0.000	**74.542**	**74.542**	7.256	34.446	32.840	0.000	**74.542**	**74.542**
122 Kampala Capital City Authority (KCCA)	0.000	0.000	0.000	0.000	**0.000**	**0.000**	0.000	0.000	0.000	0.000	**0.000**	**0.000**	0.000	0.000	0.000	0.000	**0.000**	**0.000**
123 National Lotteries and Gaming Regulatory Board	0.000	0.000	0.000	0.000	**0.000**	**0.000**	0.000	0.000	0.000	0.000	**0.000**	**0.000**	0.000	0.000	0.000	0.000	**0.000**	**0.000**
126 National Information Technologies Authority	12.949	42.643	5.746	210.114	**61.338**	**271.452**	14.244	57.568	8.045	164.275	**79.857**	**244.131**	14.244	57.568	8.045	164.315	**79.857**	**244.171**
**Sub Total For: DIGITAL TRANSFORMATION**	19.545	68.158	29.204	210.114	**116.907**	**327.021**	21.500	92.014	40.885	164.275	**154.398**	**318.673**	21.500	92.014	40.885	164.315	**154.398**	**318.713**
**12 HUMAN CAPITAL DEVELOPMENT**																		
011 Ministry of Local Government	0.000	0.049	0.000	0.000	**0.049**	**0.049**	0.000	0.066	0.000	0.000	**0.066**	**0.066**	0.000	0.066	0.000	0.000	**0.066**	**0.066**
013 Ministry of Education and Sports	143.910	226.937	117.283	103.552	**488.131**	**591.683**	158.301	306.365	164.197	0.000	**628.863**	**628.863**	158.301	306.365	164.197	0.000	**628.863**	**628.863**
014 Ministry of Health	25.000	117.097	130.832	0.000	**272.929**	**272.929**	27.499	158.081	183.165	0.000	**368.746**	**368.746**	27.499	158.081	183.165	0.000	**368.746**	**368.746**
018 Ministry of Gender, Labour and Social Development	1.767	53.543	0.000	121.286	**55.310**	**176.595**	1.944	72.282	0.000	41.890	**74.226**	**116.117**	1.944	72.282	0.000	0.000	**74.226**	**74.226**
019 Ministry of Water and Environment	5.470	0.428	360.024	543.859	**365.923**	**909.782**	6.017	0.578	504.034	108.212	**510.629**	**618.841**	6.017	0.578	504.034	0.000	**510.629**	**510.629**
107 Uganda Aids Commission (UAC)	5.733	15.590	1.465	0.000	**22.789**	**22.789**	6.307	21.047	2.051	0.000	**29.404**	**29.404**	6.307	21.047	2.051	0.000	**29.404**	**29.404**
108 National Planning Authority (NPA)	0.000	13.442	0.000	0.000	**13.442**	**13.442**	0.000	18.147	0.000	0.000	**18.147**	**18.147**	0.000	18.147	0.000	0.000	**18.147**	**18.147**
111 National Curriculum Development Centre (NCDC)	9.880	28.488	4.049	0.000	**42.417**	**42.417**	10.868	38.459	5.668	0.000	**54.995**	**54.995**	10.868	38.459	5.668	0.000	**54.995**	**54.995**
114 Uganda Cancer Institute (UCI)	10.733	28.160	0.814	0.000	**39.707**	**39.707**	11.806	38.015	1.140	0.000	**50.962**	**50.962**	11.806	38.015	1.140	0.000	**50.962**	**50.962**
115 Uganda Heart Institute (UHI)	7.639	17.104	10.709	80.800	**35.452**	**116.252**	8.403	23.091	14.993	86.244	**46.486**	**132.731**	8.403	23.091	14.993	57.510	**46.486**	**103.996**
116 Uganda National Medical Stores	22.523	835.305	7.982	0.000	**865.810**	**865.810**	24.775	1,127.662	11.175	0.000	**1,163.612**	**1,163.612**	24.775	1,127.662	11.175	0.000	**1,163.612**	**1,163.612**
122 Kampala Capital City Authority (KCCA)	52.390	21.820	3.797	0.000	**78.007**	**78.007**	57.629	29.457	5.315	0.000	**92.401**	**92.401**	57.629	29.457	5.315	0.000	**92.401**	**92.401**

ANNEX 2: MEDIUM TERM EXPENDITURE FRAMEWORK (MTEF)2025/26 - 2027/28(Excl. Arrears and AIA)

Billion Uganda Shillings	FY2025/26 Budget Projections						FY2026/27 Budget Projections						FY2027/28 Budget Projections					
PROGRAMME/VOTE	Wage	Non-Wage Recurrent	Domestic Dev	External Financing	Total excl. External Financing	Total incl. External Financing	Wage	Non-Wage Recurrent	Domestic Dev	External Financing	Total excl. External Financing	Total incl. External Financing	Wage	Non-Wage Recurrent	Domestic Dev	External Financing	Total excl. External Financing	Total incl. External Financing
**12 HUMAN CAPITAL DEVELOPMENT**																		
124 Equal Opportunities Commission	0.000	1.158	0.000	0.000	1.158	1.158	0.000	1.564	0.000	0.000	1.564	1.564	0.000	1.564	0.000	0.000	1.564	1.564
127 Uganda Virus Research Institute (UVRI)	2.562	13.276	0.000	0.000	15.838	15.838	2.818	17.922	0.000	0.000	20.740	20.740	2.818	17.922	0.000	0.000	20.740	20.740
128 Uganda National Examination Board (UNEB)	14.276	150.627	37.709	0.000	202.611	202.611	15.703	203.346	52.792	0.000	271.842	271.842	15.703	203.346	52.792	0.000	271.842	271.842
132 Education Service Commission (ESC)	3.331	9.737	2.924	0.000	15.992	15.992	3.665	13.145	4.093	0.000	20.902	20.902	3.665	13.145	4.093	0.000	20.902	20.902
134 Health Service Commission (HSC)	2.810	13.166	0.063	0.000	16.040	16.040	3.091	17.774	0.089	0.000	20.954	20.954	3.091	17.774	0.089	0.000	20.954	20.954
149 National Population Council	3.451	12.853	0.000	0.000	16.303	16.303	3.796	17.351	0.000	0.000	21.147	21.147	3.796	17.351	0.000	0.000	21.147	21.147
151 Uganda Blood Transfusion Service (UBTS)	7.035	33.289	1.481	0.000	41.805	41.805	7.739	44.941	2.073	0.000	54.753	54.753	7.739	44.941	2.073	0.000	54.753	54.753
164 National Council for Higher Education	6.052	15.798	0.000	0.000	21.850	21.850	6.657	21.328	0.000	0.000	27.985	27.985	6.657	21.328	0.000	0.000	27.985	27.985
165 Uganda Business and Technical Examination Board	7.074	41.454	3.960	0.000	52.489	52.489	7.782	55.963	5.544	0.000	69.289	69.289	7.782	55.963	5.544	0.000	69.289	69.289
166 National Council of Sports	1.858	19.313	0.000	0.000	21.171	21.171	2.044	26.073	0.000	0.000	28.117	28.117	2.044	26.073	0.000	0.000	28.117	28.117
301 Makerere University	241.361	232.093	13.239	0.000	486.693	486.693	265.497	313.326	18.535	0.000	597.358	597.358	265.497	313.326	18.535	0.000	597.358	597.358
302 Mbarara University	46.207	46.061	4.517	0.000	96.785	96.785	50.828	62.183	6.324	0.000	119.334	119.334	50.828	62.183	6.324	0.000	119.334	119.334
303 Makerere University Business School	72.355	60.966	2.551	0.000	135.871	135.871	79.590	82.304	3.571	0.000	165.465	165.465	79.590	82.304	3.571	0.000	165.465	165.465
304 Kyambogo University	70.653	123.302	2.028	0.000	195.983	195.983	77.718	166.458	2.839	0.000	247.015	247.015	77.718	166.458	2.839	0.000	247.015	247.015
305 Busitema University	38.874	21.440	7.661	0.000	67.975	67.975	42.762	28.943	10.725	0.000	82.430	82.430	42.762	28.943	10.725	0.000	82.430	82.430
306 Muni University	18.127	8.078	5.702	0.000	31.907	31.907	19.940	10.905	7.983	0.000	38.828	38.828	19.940	10.905	7.983	0.000	38.828	38.828
307 Kabale University	44.336	26.190	3.104	0.000	73.629	73.629	48.770	35.356	4.345	0.000	88.471	88.471	48.770	35.356	4.345	0.000	88.471	88.471
308 Soroti University	18.076	12.456	1.505	0.000	32.037	32.037	19.884	16.815	2.107	0.000	38.806	38.806	19.884	16.815	2.107	0.000	38.806	38.806
309 Gulu University	43.906	33.194	2.545	0.000	79.645	79.645	48.297	44.812	3.563	0.000	96.671	96.671	48.297	44.812	3.563	0.000	96.671	96.671
310 Lira University	17.964	14.302	6.000	0.000	38.266	38.266	19.761	19.307	8.400	0.000	47.468	47.468	19.761	19.307	8.400	0.000	47.468	47.468
312 Uganda Management Institute	21.661	36.680	1.584	0.000	59.925	59.925	23.827	49.518	2.218	0.000	75.563	75.563	23.827	49.518	2.218	0.000	75.563	75.563
313 Mountains of the Moon University	25.398	12.540	0.000	0.000	37.938	37.938	27.938	16.929	0.000	0.000	44.867	44.867	27.938	16.929	0.000	0.000	44.867	44.867
401 Mulago National Referral Hospital	52.874	72.763	6.312	0.000	131.950	131.950	58.162	98.230	8.837	0.000	165.229	165.229	58.162	98.230	8.837	0.000	165.229	165.229
402 Butabika Hospital	10.477	25.798	3.016	0.000	39.291	39.291	11.525	34.827	4.222	0.000	50.575	50.575	11.525	34.827	4.222	0.000	50.575	50.575
403 Arua Hospital	9.008	6.052	0.144	0.000	15.205	15.205	9.909	8.171	0.202	0.000	18.281	18.281	9.909	8.171	0.202	0.000	18.281	18.281

ANNEX 2: MEDIUM TERM EXPENDITURE FRAMEWORK (MTEF)2025/26 - 2027/28(Excl. Arrears and AIA)

Billion Uganda Shillings	FY2025/26 Budget Projections						FY2026/27 Budget Projections						FY2027/28 Budget Projections					
PROGRAMME/VOTE	Wage	Non-Wage Recurrent	Domestic Dev	External Financing	Total excl. External Financing	Total incl. External Financing	Wage	Non-Wage Recurrent	Domestic Dev	External Financing	Total excl. External Financing	Total incl. External Financing	Wage	Non-Wage Recurrent	Domestic Dev	External Financing	Total excl. External Financing	Total incl. External Financing
**12 HUMAN CAPITAL DEVELOPMENT**																		
404 Fort Portal Hospital	10.386	5.853	0.144	0.000	16.383	16.383	11.425	7.902	0.202	0.000	19.528	19.528	11.425	7.902	0.202	0.000	19.528	19.528
405 Gulu Hospital	9.367	14.704	0.144	0.000	24.215	24.215	10.304	19.850	0.202	0.000	30.356	30.356	10.304	19.850	0.202	0.000	30.356	30.356
406 Hoima Hospital	9.976	4.089	0.144	0.000	14.209	14.209	10.974	5.520	0.202	0.000	16.696	16.696	10.974	5.520	0.202	0.000	16.696	16.696
407 Jinja Hospital	14.535	17.352	0.144	0.000	32.031	32.031	15.989	23.425	0.202	0.000	39.615	39.615	15.989	23.425	0.202	0.000	39.615	39.615
408 Kabale Hospital	7.295	8.416	0.144	0.000	15.856	15.856	8.025	11.362	0.202	0.000	19.588	19.588	8.025	11.362	0.202	0.000	19.588	19.588
409 Masaka Hospital	9.478	5.534	0.144	0.000	15.156	15.156	10.426	7.470	0.202	0.000	18.098	18.098	10.426	7.470	0.202	0.000	18.098	18.098
410 Mbale Hospital	10.801	16.833	0.144	0.000	27.778	27.778	11.881	22.725	0.202	0.000	34.807	34.807	11.881	22.725	0.202	0.000	34.807	34.807
411 Soroti Hospital	8.991	6.255	0.144	0.000	15.390	15.390	9.890	8.444	0.202	0.000	18.536	18.536	9.890	8.444	0.202	0.000	18.536	18.536
412 Lira Hospital	9.647	17.769	0.144	0.000	27.559	27.559	10.612	23.988	0.202	0.000	34.801	34.801	10.612	23.988	0.202	0.000	34.801	34.801
413 Mbarara Regional Hospital	10.219	19.794	0.144	0.000	30.157	30.157	11.241	26.722	0.202	0.000	38.164	38.164	11.241	26.722	0.202	0.000	38.164	38.164
414 Mubende Regional Referral Hospital	9.081	3.710	0.180	0.000	12.971	12.971	9.989	5.008	0.252	0.000	15.249	15.249	9.989	5.008	0.252	0.000	15.249	15.249
415 Moroto Regional Referral Hospital	8.253	7.253	0.144	0.000	15.650	15.650	9.078	9.792	0.202	0.000	19.071	19.071	9.078	9.792	0.202	0.000	19.071	19.071
416 Naguru National Referral Hospital	11.658	3.002	0.288	0.000	14.948	14.948	12.824	4.052	0.403	0.000	17.279	17.279	12.824	4.052	0.403	0.000	17.279	17.279
417 Kiruddu National Referral Hospital	11.754	27.619	1.836	0.000	41.209	41.209	12.929	37.286	2.570	0.000	52.785	52.785	12.929	37.286	2.570	0.000	52.785	52.785
418 Kawempe National Referral Hospital	10.865	11.437	1.080	0.000	23.382	23.382	11.951	15.439	1.512	0.000	28.903	28.903	11.951	15.439	1.512	0.000	28.903	28.903
419 Entebbe Regional Referral Hospital	5.329	4.127	1.080	0.000	10.537	10.537	5.862	5.572	1.512	0.000	12.946	12.946	5.862	5.572	1.512	0.000	12.946	12.946
420 Mulago Specialized Women and Neonatal Hospital	12.829	27.720	2.722	0.000	43.271	43.271	14.111	37.423	3.810	0.000	55.344	55.344	14.111	37.423	3.810	0.000	55.344	55.344
421 Kayunga Referral Hospital	4.301	15.086	0.000	0.000	19.387	19.387	4.731	20.366	0.000	0.000	25.097	25.097	4.731	20.366	0.000	0.000	25.097	25.097
422 Yumbe Referral Hospital	4.831	7.934	0.000	0.000	12.765	12.765	5.314	10.711	0.000	0.000	16.025	16.025	5.314	10.711	0.000	0.000	16.025	16.025
515 Uganda Embassy in Japan, Tokyo	0.000	0.044	0.000	0.000	0.044	0.044	0.000	0.044	0.000	0.000	0.044	0.044	0.000	0.044	0.000	0.000	0.044	0.044
516 Uganda Embassy in Saudi Arabia, Riyadh	0.000	0.001	0.000	0.000	0.001	0.001	0.000	0.001	0.000	0.000	0.001	0.001	0.000	0.001	0.000	0.000	0.001	0.001
519 Uganda Embassy in Italy, Rome	0.000	0.040	0.000	0.000	0.040	0.040	0.000	0.040	0.000	0.000	0.040	0.040	0.000	0.040	0.000	0.000	0.040	0.040
524 Uganda Embassy in Iran, Tehran	0.000	0.025	0.000	0.000	0.025	0.025	0.000	0.025	0.000	0.000	0.025	0.025	0.000	0.025	0.000	0.000	0.025	0.025
525 Uganda Embassy in Russia, Moscow	0.000	0.080	0.000	0.000	0.080	0.080	0.000	0.080	0.000	0.000	0.080	0.080	0.000	0.080	0.000	0.000	0.080	0.080
531 Uganda Embassy in Turkey, Ankara	0.000	0.150	0.000	0.000	0.150	0.150	0.000	0.150	0.000	0.000	0.150	0.150	0.000	0.150	0.000	0.000	0.150	0.150
612 Local Governments 12	3,510.522	635.373	614.484	0.000	4,760.378	4,760.378	3,861.574	861.910	860.277	0.000	5,583.761	5,583.761	3,861.574	861.910	860.277	0.000	5,583.761	5,583.761

ANNEX 2: MEDIUM TERM EXPENDITURE FRAMEWORK (MTEF)2025/26 - 2027/28(Excl. Arrears and AIA)

Billion Uganda Shillings	FY2025/26 Budget Projections						FY2026/27 Budget Projections						FY2027/28 Budget Projections					
PROGRAMME/VOTE	Wage	Non-Wage Recurrent	Domestic Dev	External Financing	Total excl. External Financing	Total incl. External Financing	Wage	Non-Wage Recurrent	Domestic Dev	External Financing	Total excl. External Financing	Total incl. External Financing	Wage	Non-Wage Recurrent	Domestic Dev	External Financing	Total excl. External Financing	Total incl. External Financing
**Sub Total For: HUMAN CAPITAL DEVELOPMENT**	4,754.891	3,260.748	1,366.254	849.496	**9,381.893**	**10,231.390**	5,230.381	4,406.048	1,912.755	236.346	**11,549.184**	**11,785.530**	5,230.381	4,406.048	1,912.755	57.510	**11,549.184**	**11,606.694**
**13 INNOVATION, TECHNOLOGY DEVELOPMENT AND TRANSFER**																		
002 State House	0.000	0.000	0.000	0.000	**0.000**	**0.000**	0.000	0.000	0.000	0.000	**0.000**	**0.000**	0.000	0.000	0.000	0.000	**0.000**	**0.000**
006 Ministry of Foreign Affairs	0.000	0.711	0.000	0.000	**0.711**	**0.711**	0.000	0.960	0.000	0.000	**0.960**	**0.960**	0.000	0.960	0.000	0.000	**0.960**	**0.960**
110 Uganda Industrial Research Institute (UIRI)	6.768	1.836	0.000	0.000	**8.604**	**8.604**	7.445	2.479	0.000	0.000	**9.924**	**9.924**	7.445	2.479	0.000	0.000	**9.924**	**9.924**
119 Uganda Registration Services Bureau (URSB)	1.132	0.159	0.000	0.000	**1.291**	**1.291**	1.245	0.215	0.000	0.000	**1.460**	**1.460**	1.245	0.215	0.000	0.000	**1.460**	**1.460**
167 Science, Technology and Innovation	4.805	19.180	27.600	0.000	**51.585**	**51.585**	5.285	25.893	38.640	0.000	**69.818**	**69.818**	5.285	25.893	38.640	0.000	**69.818**	**69.818**
525 Uganda Embassy in Russia, Moscow	0.000	0.119	0.000	0.000	**0.119**	**0.119**	0.000	0.119	0.000	0.000	**0.119**	**0.119**	0.000	0.119	0.000	0.000	**0.119**	**0.119**
**Sub Total For: INNOVATION, TECHNOLOGY DEVELOPMENT AND TRANSFER**	12.705	22.005	27.600	0.000	**62.310**	**62.310**	13.976	29.665	38.640	0.000	**82.281**	**82.281**	13.976	29.665	38.640	0.000	**82.281**	**82.281**
**14 PUBLIC SECTOR TRANSFORMATION**																		
005 Ministry of Public Service	5.037	30.598	3.840	0.000	**39.476**	**39.476**	5.541	41.308	5.376	0.000	**52.225**	**52.225**	5.541	41.308	5.376	0.000	**52.225**	**52.225**
011 Ministry of Local Government	25.581	10.092	14.400	0.000	**50.073**	**50.073**	28.139	13.624	20.160	0.000	**61.924**	**61.924**	28.139	13.624	20.160	0.000	**61.924**	**61.924**
020 Ministry of ICT and National Guidance	0.000	2.987	0.000	0.000	**2.987**	**2.987**	0.000	4.032	0.000	0.000	**4.032**	**4.032**	0.000	4.032	0.000	0.000	**4.032**	**4.032**
023 Ministry of Kampala Capital City and Metropolitan Affairs	0.566	0.967	0.000	0.000	**1.533**	**1.533**	0.623	1.305	0.000	0.000	**1.928**	**1.928**	0.623	1.305	0.000	0.000	**1.928**	**1.928**
103 Inspectorate of Government (IG)	4.435	4.308	0.000	0.000	**8.744**	**8.744**	4.879	5.816	0.000	0.000	**10.695**	**10.695**	4.879	5.816	0.000	0.000	**10.695**	**10.695**
108 National Planning Authority (NPA)	0.000	0.000	0.000	0.000	**0.000**	**0.000**	0.000	0.000	0.000	0.000	**0.000**	**0.000**	0.000	0.000	0.000	0.000	**0.000**	**0.000**
122 Kampala Capital City Authority (KCCA)	91.933	20.691	0.000	0.000	**112.624**	**112.624**	101.126	27.933	0.000	0.000	**129.059**	**129.059**	101.126	27.933	0.000	0.000	**129.059**	**129.059**
123 National Lotteries and Gaming Regulatory Board	2.869	0.000	0.000	0.000	**2.869**	**2.869**	3.156	0.000	0.000	0.000	**3.156**	**3.156**	3.156	0.000	0.000	0.000	**3.156**	**3.156**
126 National Information Technologies Authority	0.000	0.991	0.000	0.000	**0.991**	**0.991**	0.000	1.338	0.000	0.000	**1.338**	**1.338**	0.000	1.338	0.000	0.000	**1.338**	**1.338**
137 National Identification and Registration Authority (NIRA)	0.000	0.330	0.000	0.000	**0.330**	**0.330**	0.000	0.446	0.000	0.000	**0.446**	**0.446**	0.000	0.446	0.000	0.000	**0.446**	**0.446**
146 Public Service Commission (PSC)	3.799	10.038	1.680	0.000	**15.517**	**15.517**	4.178	13.552	2.352	0.000	**20.082**	**20.082**	4.178	13.552	2.352	0.000	**20.082**	**20.082**

ANNEX 2: MEDIUM TERM EXPENDITURE FRAMEWORK (MTEF)2025/26 - 2027/28(Excl. Arrears and AIA)

Billion Uganda Shillings	FY2025/26 Budget Projections						FY2026/27 Budget Projections						FY2027/28 Budget Projections					
PROGRAMME/VOTE	Wage	Non-Wage Recurrent	Domestic Dev	External Financing	Total excl. External Financing	Total incl. External Financing	Wage	Non-Wage Recurrent	Domestic Dev	External Financing	Total excl. External Financing	Total incl. External Financing	Wage	Non-Wage Recurrent	Domestic Dev	External Financing	Total excl. External Financing	Total incl. External Financing
**14 PUBLIC SECTOR TRANSFORMATION**																		
147 Local Government Finance Commission (LGFC)	1.275	9.763	0.120	0.000	**11.158**	**11.158**	1.403	13.180	0.168	0.000	**14.750**	**14.750**	1.403	13.180	0.168	0.000	**14.750**	**14.750**
**Sub Total For: PUBLIC SECTOR TRANSFORMATION**	135.495	90.766	20.040	0.000	**246.302**	**246.302**	149.045	122.534	28.056	0.000	**299.635**	**299.635**	149.045	122.534	28.056	0.000	**299.635**	**299.635**
**15 COMMUNITY MOBILIZATION AND MINDSET CHANGE**																		
001 Office of the President	0.000	0.000	0.000	0.000	**0.000**	**0.000**	0.000	0.000	0.000	0.000	**0.000**	**0.000**	0.000	0.000	0.000	0.000	**0.000**	**0.000**
006 Ministry of Foreign Affairs	0.000	0.000	0.000	0.000	**0.000**	**0.000**	0.000	0.000	0.000	0.000	**0.000**	**0.000**	0.000	0.000	0.000	0.000	**0.000**	**0.000**
018 Ministry of Gender, Labour and Social Development	2.736	13.464	6.000	0.000	**22.200**	**22.200**	3.009	18.176	8.400	0.000	**29.586**	**29.586**	3.009	18.176	8.400	0.000	**29.586**	**29.586**
020 Ministry of ICT and National Guidance	1.178	0.000	0.000	0.000	**1.178**	**1.178**	1.296	0.000	0.000	0.000	**1.296**	**1.296**	1.296	0.000	0.000	0.000	**1.296**	**1.296**
112 Directorate of Ethics and Integrity (DEI)	0.000	0.000	0.000	0.000	**0.000**	**0.000**	0.000	0.000	0.000	0.000	**0.000**	**0.000**	0.000	0.000	0.000	0.000	**0.000**	**0.000**
119 Uganda Registration Services Bureau (URSB)	0.347	0.000	0.000	0.000	**0.347**	**0.347**	0.381	0.000	0.000	0.000	**0.381**	**0.381**	0.381	0.000	0.000	0.000	**0.381**	**0.381**
122 Kampala Capital City Authority (KCCA)	0.000	0.000	0.000	0.000	**0.000**	**0.000**	0.000	0.000	0.000	0.000	**0.000**	**0.000**	0.000	0.000	0.000	0.000	**0.000**	**0.000**
123 National Lotteries and Gaming Regulatory Board	0.000	0.000	0.000	0.000	**0.000**	**0.000**	0.000	0.000	0.000	0.000	**0.000**	**0.000**	0.000	0.000	0.000	0.000	**0.000**	**0.000**
124 Equal Opportunities Commission	0.000	2.448	0.000	0.000	**2.448**	**2.448**	0.000	3.305	0.000	0.000	**3.305**	**3.305**	0.000	3.305	0.000	0.000	**3.305**	**3.305**
149 National Population Council	0.358	0.000	0.000	0.000	**0.358**	**0.358**	0.394	0.000	0.000	0.000	**0.394**	**0.394**	0.394	0.000	0.000	0.000	**0.394**	**0.394**
503 Uganda High Commission in Canada, Ottawa	0.000	0.000	0.000	0.000	**0.000**	**0.000**	0.000	0.000	0.000	0.000	**0.000**	**0.000**	0.000	0.000	0.000	0.000	**0.000**	**0.000**
506 Uganda High Commission in Tanzania, Dar es Salaam	0.000	0.000	0.000	0.000	**0.000**	**0.000**	0.000	0.000	0.000	0.000	**0.000**	**0.000**	0.000	0.000	0.000	0.000	**0.000**	**0.000**
507 Uganda High Commission in Nigeria, Abuja	0.000	0.000	0.000	0.000	**0.000**	**0.000**	0.000	0.000	0.000	0.000	**0.000**	**0.000**	0.000	0.000	0.000	0.000	**0.000**	**0.000**
508 Uganda High Commission in South Africa, Pretoria	0.000	0.000	0.000	0.000	**0.000**	**0.000**	0.000	0.000	0.000	0.000	**0.000**	**0.000**	0.000	0.000	0.000	0.000	**0.000**	**0.000**
509 Uganda High Commission in Rwanda, Kigali	0.000	0.000	0.000	0.000	**0.000**	**0.000**	0.000	0.000	0.000	0.000	**0.000**	**0.000**	0.000	0.000	0.000	0.000	**0.000**	**0.000**
515 Uganda Embassy in Japan, Tokyo	0.000	0.000	0.000	0.000	**0.000**	**0.000**	0.000	0.000	0.000	0.000	**0.000**	**0.000**	0.000	0.000	0.000	0.000	**0.000**	**0.000**

ANNEX 2: MEDIUM TERM EXPENDITURE FRAMEWORK (MTEF)2025/26 - 2027/28(Excl. Arrears and AIA)

Billion Uganda Shillings	FY2025/26 Budget Projections						FY2026/27 Budget Projections						FY2027/28 Budget Projections					
PROGRAMME/VOTE	Wage	Non-Wage Recurrent	Domestic Dev	External Financing	Total excl. External Financing	Total incl. External Financing	Wage	Non-Wage Recurrent	Domestic Dev	External Financing	Total excl. External Financing	Total incl. External Financing	Wage	Non-Wage Recurrent	Domestic Dev	External Financing	Total excl. External Financing	Total incl. External Financing
**15 COMMUNITY MOBILIZATION AND MINDSET CHANGE**																		
518 Uganda Embassy in Belgium, Brussels	0.000	0.000	0.000	0.000	**0.000**	**0.000**	0.000	0.000	0.000	0.000	**0.000**	**0.000**	0.000	0.000	0.000	0.000	**0.000**	**0.000**
519 Uganda Embassy in Italy, Rome	0.000	0.000	0.000	0.000	**0.000**	**0.000**	0.000	0.000	0.000	0.000	**0.000**	**0.000**	0.000	0.000	0.000	0.000	**0.000**	**0.000**
522 Uganda Embassy in France, Paris	0.000	0.000	0.000	0.000	**0.000**	**0.000**	0.000	0.000	0.000	0.000	**0.000**	**0.000**	0.000	0.000	0.000	0.000	**0.000**	**0.000**
523 Uganda Embassy in Germany, Berlin	0.000	0.000	0.000	0.000	**0.000**	**0.000**	0.000	0.000	0.000	0.000	**0.000**	**0.000**	0.000	0.000	0.000	0.000	**0.000**	**0.000**
525 Uganda Embassy in Russia, Moscow	0.000	0.000	0.000	0.000	**0.000**	**0.000**	0.000	0.000	0.000	0.000	**0.000**	**0.000**	0.000	0.000	0.000	0.000	**0.000**	**0.000**
526 Uganda Embassy in Australia, Canberra	0.000	0.000	0.000	0.000	**0.000**	**0.000**	0.000	0.000	0.000	0.000	**0.000**	**0.000**	0.000	0.000	0.000	0.000	**0.000**	**0.000**
527 Uganda Embassy in South Sudan, Juba	0.000	0.000	0.000	0.000	**0.000**	**0.000**	0.000	0.000	0.000	0.000	**0.000**	**0.000**	0.000	0.000	0.000	0.000	**0.000**	**0.000**
528 Uganda Embassy in United Arab Emirates, Abudhabi	0.000	0.000	0.000	0.000	**0.000**	**0.000**	0.000	0.000	0.000	0.000	**0.000**	**0.000**	0.000	0.000	0.000	0.000	**0.000**	**0.000**
529 Uganda Embassy in Burundi, Bujumbura	0.000	0.000	0.000	0.000	**0.000**	**0.000**	0.000	0.000	0.000	0.000	**0.000**	**0.000**	0.000	0.000	0.000	0.000	**0.000**	**0.000**
531 Uganda Embassy in Turkey, Ankara	0.000	0.000	0.000	0.000	**0.000**	**0.000**	0.000	0.000	0.000	0.000	**0.000**	**0.000**	0.000	0.000	0.000	0.000	**0.000**	**0.000**
533 Uganda Embassy in Malaysia, Kuala Lumpur	0.000	0.000	0.000	0.000	**0.000**	**0.000**	0.000	0.000	0.000	0.000	**0.000**	**0.000**	0.000	0.000	0.000	0.000	**0.000**	**0.000**
615 Local Governments 15	0.000	0.000	0.000	0.000	**0.000**	**0.000**	0.000	0.000	0.000	0.000	**0.000**	**0.000**	0.000	0.000	0.000	0.000	**0.000**	**0.000**
**Sub Total For: COMMUNITY MOBILIZATION AND MINDSET CHANGE**	4.618	15.912	6.000	0.000	**26.530**	**26.530**	5.080	21.481	8.400	0.000	**34.962**	**34.962**	5.080	21.481	8.400	0.000	**34.962**	**34.962**
**16 GOVERNANCE AND SECURITY**																		
001 Office of the President	29.233	173.680	26.832	0.000	**229.745**	**229.745**	32.156	234.468	37.565	0.000	**304.189**	**304.189**	32.156	234.468	37.565	0.000	**304.189**	**304.189**
002 State House	26.145	459.708	26.066	0.000	**511.920**	**511.920**	28.760	620.606	36.493	0.000	**685.859**	**685.859**	28.760	620.606	36.493	0.000	**685.859**	**685.859**
003 Office of the Prime Minister	0.247	0.612	0.410	0.000	**1.269**	**1.269**	0.271	0.826	0.575	0.000	**1.672**	**1.672**	0.271	0.826	0.575	0.000	**1.672**	**1.672**
004 Ministry of Defence	725.272	882.665	2,379.272	0.000	**3,987.210**	**3,987.210**	797.799	1,324.250	2,713.928	0.000	**4,835.977**	**4,835.977**	797.799	1,324.250	2,713.928	0.000	**4,835.977**	**4,835.977**
006 Ministry of Foreign Affairs	6.941	26.327	0.924	0.000	**34.192**	**34.192**	7.635	35.542	1.294	0.000	**44.470**	**44.470**	7.635	35.542	1.294	0.000	**44.470**	**44.470**
007 Ministry of Justice and Constitutional Affairs	12.549	143.823	26.088	0.000	**182.461**	**182.461**	13.804	194.161	36.523	0.000	**244.489**	**244.489**	13.804	194.161	36.523	0.000	**244.489**	**244.489**
008 Ministry of Finance, Planning and Economic Development	0.000	2.472	0.000	0.000	**2.472**	**2.472**	0.000	3.338	0.000	0.000	**3.338**	**3.338**	0.000	3.338	0.000	0.000	**3.338**	**3.338**
009 Ministry of Internal Affairs	2.828	66.643	3.600	0.000	**73.071**	**73.071**	3.111	89.968	5.040	0.000	**98.119**	**98.119**	3.111	89.968	5.040	0.000	**98.119**	**98.119**

ANNEX 2: MEDIUM TERM EXPENDITURE FRAMEWORK (MTEF)2025/26 - 2027/28(Excl. Arrears and AIA)

Billion Uganda Shillings	FY2025/26 Budget Projections						FY2026/27 Budget Projections						FY2027/28 Budget Projections					
PROGRAMME/VOTE	Wage	Non-Wage Recurrent	Domestic Dev	External Financing	Total excl. External Financing	Total incl. External Financing	Wage	Non-Wage Recurrent	Domestic Dev	External Financing	Total excl. External Financing	Total incl. External Financing	Wage	Non-Wage Recurrent	Domestic Dev	External Financing	Total excl. External Financing	Total incl. External Financing
**16 GOVERNANCE AND SECURITY**																		
011 Ministry of Local Government	0.327	0.576	0.000	0.000	0.903	0.903	0.360	0.778	0.000	0.000	1.138	1.138	0.360	0.778	0.000	0.000	1.138	1.138
018 Ministry of Gender, Labour and Social Development	0.117	4.943	0.000	0.000	5.060	5.060	0.128	6.674	0.000	0.000	6.802	6.802	0.128	6.674	0.000	0.000	6.802	6.802
021 Ministry of East African Community Affairs	1.479	43.550	1.590	0.000	46.619	46.619	1.627	58.792	2.226	0.000	62.645	62.645	1.627	58.792	2.226	0.000	62.645	62.645
102 Electoral Commission (EC)	43.505	128.410	4.464	0.000	176.379	176.379	47.855	173.354	6.250	0.000	227.459	227.459	47.855	173.354	6.250	0.000	227.459	227.459
103 Inspectorate of Government (IG)	27.850	40.099	18.240	0.000	86.190	86.190	30.635	55.440	25.536	0.000	111.612	111.612	30.635	55.440	25.536	0.000	111.612	111.612
105 Law Reform Commission (LRC)	4.705	17.083	0.504	0.000	22.292	22.292	5.175	23.062	0.706	0.000	28.943	28.943	5.175	23.062	0.706	0.000	28.943	28.943
106 Uganda Human Rights Commission (UHRC)	8.772	14.010	0.637	0.000	23.419	23.419	9.649	18.913	0.892	0.000	29.454	29.454	9.649	18.913	0.892	0.000	29.454	29.454
112 Directorate of Ethics and Integrity (DEI)	7.303	9.944	0.785	0.000	18.032	18.032	8.034	13.425	1.099	0.000	22.557	22.557	8.034	13.425	1.099	0.000	22.557	22.557
119 Uganda Registration Services Bureau (URSB)	8.920	20.874	1.200	0.000	30.994	30.994	9.812	28.179	1.680	0.000	39.672	39.672	9.812	28.179	1.680	0.000	39.672	39.672
120 National Citizenship and Immigration Control (NCIC)	6.092	127.322	7.584	0.000	140.997	140.997	6.701	171.884	10.618	0.000	189.203	189.203	6.701	171.884	10.618	0.000	189.203	189.203
123 National Lotteries and Gaming Regulatory Board	0.000	0.000	0.000	0.000	0.000	0.000	0.000	0.000	0.000	0.000	0.000	0.000	0.000	0.000	0.000	0.000	0.000	0.000
124 Equal Opportunities Commission	0.000	1.796	0.000	0.000	1.796	1.796	0.000	2.425	0.000	0.000	2.425	2.425	0.000	2.425	0.000	0.000	2.425	2.425
129 Financial Intelligence Authority (FIA)	11.082	20.720	0.875	0.000	32.676	32.676	12.190	27.972	1.225	0.000	41.386	41.386	12.190	27.972	1.225	0.000	41.386	41.386
131 Office of the Auditor General (OAG)	50.271	63.008	4.512	0.000	117.791	117.791	55.298	85.061	6.317	0.000	146.676	146.676	55.298	85.061	6.317	0.000	146.676	146.676
133 Directorate of Public Prosecution (DPP)	27.822	42.358	20.449	0.000	90.629	90.629	30.604	57.183	28.629	0.000	116.416	116.416	30.604	57.183	28.629	0.000	116.416	116.416
135 Directorate of Government Analytical Laboratory (DGAL)	3.832	31.149	5.544	0.000	40.525	40.525	4.215	42.051	7.762	0.000	54.028	54.028	4.215	42.051	7.762	0.000	54.028	54.028
137 National Identification and Registration Authority (NIRA)	23.487	44.224	3.600	0.000	71.311	71.311	25.835	59.703	5.040	0.000	90.578	90.578	25.835	59.703	5.040	0.000	90.578	90.578
144 Uganda Police Force	442.405	373.851	161.113	0.000	977.369	977.369	486.645	504.699	225.558	0.000	1,216.902	1,216.902	486.645	504.699	225.558	0.000	1,216.902	1,216.902
145 Uganda Prisons Service	110.453	228.544	31.645	0.000	370.642	370.642	121.498	308.534	44.303	0.000	474.335	474.335	121.498	308.534	44.303	0.000	474.335	474.335
153 Public Procurement & Disposal of Public Assets (PPDA)	12.722	9.535	3.600	0.000	25.858	25.858	13.995	12.873	5.040	0.000	31.907	31.907	13.995	12.873	5.040	0.000	31.907	31.907

ANNEX 2: MEDIUM TERM EXPENDITURE FRAMEWORK (MTEF)2025/26 - 2027/28(Excl. Arrears and AIA)

Billion Uganda Shillings	FY2025/26 Budget Projections						FY2026/27 Budget Projections						FY2027/28 Budget Projections					
PROGRAMME/VOTE	Wage	Non-Wage Recurrent	Domestic Dev	External Financing	Total excl. External Financing	Total incl. External Financing	Wage	Non-Wage Recurrent	Domestic Dev	External Financing	Total excl. External Financing	Total incl. External Financing	Wage	Non-Wage Recurrent	Domestic Dev	External Financing	Total excl. External Financing	Total incl. External Financing
**16 GOVERNANCE AND SECURITY**																		
158 Internal Security Organization (ISO)	60.592	105.015	12.816	0.000	**178.423**	**178.423**	66.651	141.771	17.942	0.000	**226.364**	**226.364**	66.651	141.771	17.942	0.000	**226.364**	**226.364**
159 External Security Organization (ESO)	21.152	65.560	1.204	0.000	**87.916**	**87.916**	23.267	88.506	1.685	0.000	**113.458**	**113.458**	23.267	88.506	1.685	0.000	**113.458**	**113.458**
311 Law Development Centre	9.211	18.873	6.240	0.000	**34.324**	**34.324**	10.132	25.479	8.736	0.000	**44.346**	**44.346**	10.132	25.479	8.736	0.000	**44.346**	**44.346**
501 Uganda Mission at the United Nations, New York	1.951	14.607	1.053	0.000	**17.612**	**17.612**	1.951	14.607	1.053	0.000	**17.612**	**17.612**	1.951	14.607	1.053	0.000	**17.612**	**17.612**
502 Uganda High Commission in the United Kingdom	1.397	2.614	2.114	0.000	**6.124**	**6.124**	1.397	2.614	2.114	0.000	**6.124**	**6.124**	1.397	2.614	2.114	0.000	**6.124**	**6.124**
503 Uganda High Commission in Canada, Ottawa	1.175	3.938	8.500	0.000	**13.614**	**13.614**	1.175	3.938	8.500	0.000	**13.614**	**13.614**	1.175	3.938	8.500	0.000	**13.614**	**13.614**
504 Uganda High Commission in India, New Delhi	0.306	2.277	0.270	0.000	**2.853**	**2.853**	0.306	2.277	0.270	0.000	**2.853**	**2.853**	0.306	2.277	0.270	0.000	**2.853**	**2.853**
505 Uganda High Commission in Kenya, Nairobi	0.339	3.354	6.700	0.000	**10.393**	**10.393**	0.339	3.354	6.700	0.000	**10.393**	**10.393**	0.339	3.354	6.700	0.000	**10.393**	**10.393**
506 Uganda High Commission in Tanzania, Dar es Salaam	0.603	4.646	3.928	0.000	**9.177**	**9.177**	0.603	4.646	3.928	0.000	**9.177**	**9.177**	0.603	4.646	3.928	0.000	**9.177**	**9.177**
507 Uganda High Commission in Nigeria, Abuja	0.386	2.336	16.200	0.000	**18.922**	**18.922**	0.386	2.336	16.200	0.000	**18.922**	**18.922**	0.386	2.336	16.200	0.000	**18.922**	**18.922**
508 Uganda High Commission in South Africa, Pretoria	0.440	2.638	0.390	0.000	**3.469**	**3.469**	0.440	2.638	0.390	0.000	**3.469**	**3.469**	0.440	2.638	0.390	0.000	**3.469**	**3.469**
509 Uganda High Commission in Rwanda, Kigali	0.529	2.232	0.600	0.000	**3.361**	**3.361**	0.529	2.232	0.600	0.000	**3.361**	**3.361**	0.529	2.232	0.600	0.000	**3.361**	**3.361**
510 Uganda Embassy in the United States, Washington	1.362	5.603	0.540	0.000	**7.505**	**7.505**	1.362	5.603	0.540	0.000	**7.505**	**7.505**	1.362	5.603	0.540	0.000	**7.505**	**7.505**
511 Uganda Embassy in Egypt, Cairo	0.544	2.278	0.170	0.000	**2.992**	**2.992**	0.544	2.278	0.170	0.000	**2.992**	**2.992**	0.544	2.278	0.170	0.000	**2.992**	**2.992**
512 Uganda Embassy in Ethiopia, Addis Ababa	0.508	1.910	0.150	0.000	**2.569**	**2.569**	0.508	1.910	0.150	0.000	**2.569**	**2.569**	0.508	1.910	0.150	0.000	**2.569**	**2.569**
513 Uganda Embassy in China, Beijing	0.388	3.592	0.100	0.000	**4.081**	**4.081**	0.388	3.592	0.100	0.000	**4.081**	**4.081**	0.388	3.592	0.100	0.000	**4.081**	**4.081**
514 Uganda Embassy in Switzerland, Geneva	1.631	4.869	0.100	0.000	**6.601**	**6.601**	1.631	4.869	0.100	0.000	**6.601**	**6.601**	1.631	4.869	0.100	0.000	**6.601**	**6.601**
515 Uganda Embassy in Japan, Tokyo	1.510	3.669	0.000	0.000	**5.179**	**5.179**	1.510	3.669	0.000	0.000	**5.179**	**5.179**	1.510	3.669	0.000	0.000	**5.179**	**5.179**
516 Uganda Embassy in Saudi Arabia, Riyadh	0.999	4.441	0.000	0.000	**5.441**	**5.441**	0.999	4.441	0.000	0.000	**5.441**	**5.441**	0.999	4.441	0.000	0.000	**5.441**	**5.441**

ANNEX 2: MEDIUM TERM EXPENDITURE FRAMEWORK (MTEF)2025/26 - 2027/28(Excl. Arrears and AIA)

Billion Uganda Shillings	FY2025/26 Budget Projections						FY2026/27 Budget Projections						FY2027/28 Budget Projections					
PROGRAMME/VOTE	Wage	Non-Wage Recurrent	Domestic Dev	External Financing	Total excl. External Financing	Total incl. External Financing	Wage	Non-Wage Recurrent	Domestic Dev	External Financing	Total excl. External Financing	Total incl. External Financing	Wage	Non-Wage Recurrent	Domestic Dev	External Financing	Total excl. External Financing	Total incl. External Financing
**16 GOVERNANCE AND SECURITY**																		
517 Uganda Embassy in Denmark, Copenhagen	0.763	4.885	0.100	0.000	5.748	5.748	0.763	4.885	0.100	0.000	5.748	5.748	0.763	4.885	0.100	0.000	5.748	5.748
518 Uganda Embassy in Belgium, Brussels	1.099	3.070	0.380	0.000	4.549	4.549	1.099	3.070	0.380	0.000	4.549	4.549	1.099	3.070	0.380	0.000	4.549	4.549
519 Uganda Embassy in Italy, Rome	0.848	3.629	0.000	0.000	4.477	4.477	0.848	3.629	0.000	0.000	4.477	4.477	0.848	3.629	0.000	0.000	4.477	4.477
520 Uganda Embassy in DRC, Kinshasa	0.658	3.458	2.074	0.000	6.190	6.190	0.658	3.458	2.074	0.000	6.190	6.190	0.658	3.458	2.074	0.000	6.190	6.190
521 Uganda Embassy in Sudan, Khartoum	0.609	3.189	0.560	0.000	4.358	4.358	0.609	3.189	0.560	0.000	4.358	4.358	0.609	3.189	0.560	0.000	4.358	4.358
522 Uganda Embassy in France, Paris	0.951	5.506	0.000	0.000	6.458	6.458	0.951	5.506	0.000	0.000	6.458	6.458	0.951	5.506	0.000	0.000	6.458	6.458
523 Uganda Embassy in Germany, Berlin	1.132	5.468	0.000	0.000	6.601	6.601	1.132	5.468	0.000	0.000	6.601	6.601	1.132	5.468	0.000	0.000	6.601	6.601
524 Uganda Embassy in Iran, Tehran	0.756	2.002	0.000	0.000	2.758	2.758	0.756	2.002	0.000	0.000	2.758	2.758	0.756	2.002	0.000	0.000	2.758	2.758
525 Uganda Embassy in Russia, Moscow	0.610	2.940	0.172	0.000	3.722	3.722	0.610	2.940	0.172	0.000	3.722	3.722	0.610	2.940	0.172	0.000	3.722	3.722
526 Uganda Embassy in Australia, Canberra	0.989	2.974	0.150	0.000	4.113	4.113	0.989	2.974	0.150	0.000	4.113	4.113	0.989	2.974	0.150	0.000	4.113	4.113
527 Uganda Embassy in South Sudan, Juba	0.423	3.241	0.000	0.000	3.664	3.664	0.423	3.241	0.000	0.000	3.664	3.664	0.423	3.241	0.000	0.000	3.664	3.664
528 Uganda Embassy in United Arab Emirates, Abudhabi	0.765	3.550	0.000	0.000	4.315	4.315	0.765	3.550	0.000	0.000	4.315	4.315	0.765	3.550	0.000	0.000	4.315	4.315
529 Uganda Embassy in Burundi, Bujumbura	0.556	2.275	0.150	0.000	2.982	2.982	0.556	2.275	0.150	0.000	2.982	2.982	0.556	2.275	0.150	0.000	2.982	2.982
530 Uganda Consulate in China, Guangzhou	0.419	3.281	6.200	0.000	9.900	9.900	0.419	3.281	6.200	0.000	9.900	9.900	0.419	3.281	6.200	0.000	9.900	9.900
531 Uganda Embassy in Turkey, Ankara	0.695	3.723	0.390	0.000	4.808	4.808	0.695	3.723	0.390	0.000	4.808	4.808	0.695	3.723	0.390	0.000	4.808	4.808
532 Uganda Embassy in Somalia, Mogadishu	0.134	2.509	1.600	0.000	4.243	4.243	0.134	2.509	1.600	0.000	4.243	4.243	0.134	2.509	1.600	0.000	4.243	4.243
533 Uganda Embassy in Malaysia, Kuala Lumpur	0.580	2.698	0.100	0.000	3.378	3.378	0.580	2.698	0.100	0.000	3.378	3.378	0.580	2.698	0.100	0.000	3.378	3.378
534 Uganda Consulate in Kenya, Mombasa	0.747	3.960	0.000	0.000	4.706	4.706	0.747	3.960	0.000	0.000	4.706	4.706	0.747	3.960	0.000	0.000	4.706	4.706
535 Uganda Embassy in Algeria, Algiers	0.915	2.774	0.000	0.000	3.689	3.689	0.915	2.774	0.000	0.000	3.689	3.689	0.915	2.774	0.000	0.000	3.689	3.689
536 Uganda Embassy in Qatar, Doha	0.541	2.103	0.198	0.000	2.842	2.842	0.541	2.103	0.198	0.000	2.842	2.842	0.541	2.103	0.198	0.000	2.842	2.842
537 Uganda Mission in Havana, Cuba	0.326	2.674	0.000	0.000	3.000	3.000	0.326	2.674	0.000	0.000	3.000	3.000	0.326	2.674	0.000	0.000	3.000	3.000
538 Uganda Mission in Luanda, Angola	0.585	2.415	0.000	0.000	3.000	3.000	0.585	2.415	0.000	0.000	3.000	3.000	0.585	2.415	0.000	0.000	3.000	3.000
**Sub Total For: GOVERNANCE AND SECURITY**	1,714.485	3,304.707	2,802.683	0.000	7,821.876	7,821.876	1,883.017	4,547.246	3,285.548	0.000	9,715.811	9,715.811	1,883.017	4,547.246	3,285.548	0.000	9,715.811	9,715.811

ANNEX 2: MEDIUM TERM EXPENDITURE FRAMEWORK (MTEF)2025/26 - 2027/28(Excl. Arrears and AIA)

Billion Uganda Shillings	FY2025/26 Budget Projections						FY2026/27 Budget Projections						FY2027/28 Budget Projections					
PROGRAMME/VOTE	Wage	Non-Wage Recurrent	Domestic Dev	External Financing	Total excl. External Financing	Total incl. External Financing	Wage	Non-Wage Recurrent	Domestic Dev	External Financing	Total excl. External Financing	Total incl. External Financing	Wage	Non-Wage Recurrent	Domestic Dev	External Financing	Total excl. External Financing	Total incl. External Financing
**17 REGIONAL BALANCED DEVELOPMENT**																		
003 Office of the Prime Minister	0.401	0.245	3.000	0.000	3.645	3.645	0.441	0.330	4.200	0.000	4.971	4.971	0.441	0.330	4.200	0.000	4.971	4.971
010 Ministry of Agriculture, Animal Industry and Fisheries	0.000	0.367	0.000	0.000	0.367	0.367	0.000	0.496	0.000	0.000	0.496	0.496	0.000	0.496	0.000	0.000	0.496	0.496
011 Ministry of Local Government	0.054	18.482	13.680	0.000	32.217	32.217	0.060	24.951	19.152	0.000	44.163	44.163	0.060	24.951	19.152	0.000	44.163	44.163
015 Ministry of Trade, Industry and Co-operatives	0.000	0.122	0.000	0.000	0.122	0.122	0.000	0.165	0.000	0.000	0.165	0.165	0.000	0.165	0.000	0.000	0.165	0.165
016 Ministry of Works and Transport	0.000	0.490	0.000	0.000	0.490	0.490	0.000	0.661	0.000	0.000	0.661	0.661	0.000	0.661	0.000	0.000	0.661	0.661
017 Ministry of Energy and Mineral Development	0.000	0.367	0.000	0.000	0.367	0.367	0.000	0.496	0.000	0.000	0.496	0.496	0.000	0.496	0.000	0.000	0.496	0.496
020 Ministry of ICT and National Guidance	0.000	0.245	0.000	0.000	0.245	0.245	0.000	0.330	0.000	0.000	0.330	0.330	0.000	0.330	0.000	0.000	0.330	0.330
022 Ministry of Tourism, Wildlife and Antiquities	0.000	0.122	0.000	0.000	0.122	0.122	0.000	0.165	0.000	0.000	0.165	0.165	0.000	0.165	0.000	0.000	0.165	0.165
108 National Planning Authority (NPA)	0.000	0.245	0.000	0.000	0.245	0.245	0.000	0.330	0.000	0.000	0.330	0.330	0.000	0.330	0.000	0.000	0.330	0.330
142 National Agricultural Research Organization (NARO)	0.000	0.122	0.000	0.000	0.122	0.122	0.000	0.165	0.000	0.000	0.165	0.165	0.000	0.165	0.000	0.000	0.165	0.165
147 Local Government Finance Commission (LGFC)	0.000	0.122	0.000	0.000	0.122	0.122	0.000	0.165	0.000	0.000	0.165	0.165	0.000	0.165	0.000	0.000	0.165	0.165
617 Local Governments 17	488.293	156.672	0.000	0.000	644.965	644.965	537.122	211.507	0.000	0.000	748.629	748.629	537.122	211.507	0.000	0.000	748.629	748.629
**Sub Total For: REGIONAL BALANCED DEVELOPMENT**	488.747	177.602	16.680	0.000	683.030	683.030	537.622	239.763	23.352	0.000	800.737	800.737	537.622	239.763	23.352	0.000	800.737	800.737
**18 DEVELOPMENT PLAN IMPLEMENTATION**																		
001 Office of the President	0.324	17.475	0.000	0.000	17.799	17.799	0.357	23.591	0.000	0.000	23.948	23.948	0.357	23.591	0.000	0.000	23.948	23.948
003 Office of the Prime Minister	3.711	55.588	4.531	0.000	63.830	63.830	4.082	75.044	6.344	0.000	85.469	85.469	4.082	75.044	6.344	0.000	85.469	85.469
006 Ministry of Foreign Affairs	0.000	0.424	0.000	0.000	0.424	0.424	0.000	0.572	0.000	0.000	0.572	0.572	0.000	0.572	0.000	0.000	0.572	0.572
008 Ministry of Finance, Planning and Economic Development	10.389	191.757	216.650	0.000	418.797	418.797	11.428	258.872	303.311	0.000	573.610	573.610	11.428	258.872	303.311	0.000	573.610	573.610
011 Ministry of Local Government	2.259	21.519	0.000	0.000	23.778	23.778	2.485	29.051	0.000	0.000	31.536	31.536	2.485	29.051	0.000	0.000	31.536	31.536
023 Ministry of Kampala Capital City and Metropolitan Affairs	0.000	0.061	0.000	0.000	0.061	0.061	0.000	0.083	0.000	0.000	0.083	0.083	0.000	0.083	0.000	0.000	0.083	0.083

ANNEX 2: MEDIUM TERM EXPENDITURE FRAMEWORK (MTEF)2025/26 - 2027/28(Excl. Arrears and AIA)

Billion Uganda Shillings	FY2025/26 Budget Projections						FY2026/27 Budget Projections						FY2027/28 Budget Projections					
PROGRAMME/VOTE	Wage	Non-Wage Recurrent	Domestic Dev	External Financing	Total excl. External Financing	Total incl. External Financing	Wage	Non-Wage Recurrent	Domestic Dev	External Financing	Total excl. External Financing	Total incl. External Financing	Wage	Non-Wage Recurrent	Domestic Dev	External Financing	Total excl. External Financing	Total incl. External Financing
**18 DEVELOPMENT PLAN IMPLEMENTATION**																		
103 Inspectorate of Government (IG)	0.000	0.000	11.276	0.000	**11.276**	**11.276**	0.000	0.000	15.787	0.000	**15.787**	**15.787**	0.000	0.000	15.787	0.000	**15.787**	**15.787**
108 National Planning Authority (NPA)	16.875	17.129	3.492	0.000	**37.496**	**37.496**	18.563	23.124	4.889	0.000	**46.575**	**46.575**	18.563	23.124	4.889	0.000	**46.575**	**46.575**
122 Kampala Capital City Authority (KCCA)	0.000	2.566	0.522	0.000	**3.088**	**3.088**	0.000	3.463	0.731	0.000	**4.194**	**4.194**	0.000	3.463	0.731	0.000	**4.194**	**4.194**
123 National Lotteries and Gaming Regulatory Board	0.000	0.419	0.000	0.000	**0.419**	**0.419**	0.000	0.565	0.000	0.000	**0.565**	**0.565**	0.000	0.565	0.000	0.000	**0.565**	**0.565**
124 Equal Opportunities Commission	3.427	9.668	0.259	0.000	**13.354**	**13.354**	3.769	13.052	0.363	0.000	**17.185**	**17.185**	3.769	13.052	0.363	0.000	**17.185**	**17.185**
130 Treasury Operations	0.000	19,428.133	0.000	0.000	**19,428.133**	**19,428.133**	0.000	23,652.405	0.000	0.000	**23,652.405**	**23,652.405**	0.000	23,720.618	0.000	0.000	**23,720.618**	**23,720.618**
131 Office of the Auditor General (OAG)	0.000	1.224	0.000	0.000	**1.224**	**1.224**	0.000	1.652	0.000	0.000	**1.652**	**1.652**	0.000	1.652	0.000	0.000	**1.652**	**1.652**
141 Uganda Revenue Authority (URA)	237.347	387.182	54.384	0.000	**678.912**	**678.912**	261.082	522.695	76.138	0.000	**859.914**	**859.914**	261.082	522.695	76.138	0.000	**859.914**	**859.914**
143 Uganda Bureau of Statistics (UBOS)	24.706	262.825	16.164	0.000	**303.695**	**303.695**	27.177	354.813	22.630	0.000	**404.619**	**404.619**	27.177	354.813	22.630	0.000	**404.619**	**404.619**
147 Local Government Finance Commission (LGFC)	0.595	1.570	0.000	0.000	**2.165**	**2.165**	0.654	2.120	0.000	0.000	**2.774**	**2.774**	0.654	2.120	0.000	0.000	**2.774**	**2.774**
149 National Population Council	0.000	0.000	0.649	0.000	**0.649**	**0.649**	0.000	0.000	0.909	0.000	**0.909**	**0.909**	0.000	0.000	0.909	0.000	**0.909**	**0.909**
501 Uganda Mission at the United Nations, New York	0.000	0.148	0.000	0.000	**0.148**	**0.148**	0.000	0.148	0.000	0.000	**0.148**	**0.148**	0.000	0.148	0.000	0.000	**0.148**	**0.148**
502 Uganda High Commission in the United Kingdom	0.000	1.277	0.000	0.000	**1.277**	**1.277**	0.000	1.277	0.000	0.000	**1.277**	**1.277**	0.000	1.277	0.000	0.000	**1.277**	**1.277**
503 Uganda High Commission in Canada, Ottawa	0.000	0.110	0.000	0.000	**0.110**	**0.110**	0.000	0.110	0.000	0.000	**0.110**	**0.110**	0.000	0.110	0.000	0.000	**0.110**	**0.110**
504 Uganda High Commission in India, New Delhi	0.000	1.000	0.000	0.000	**1.000**	**1.000**	0.000	1.000	0.000	0.000	**1.000**	**1.000**	0.000	1.000	0.000	0.000	**1.000**	**1.000**
505 Uganda High Commission in Kenya, Nairobi	0.000	0.088	0.000	0.000	**0.088**	**0.088**	0.000	0.088	0.000	0.000	**0.088**	**0.088**	0.000	0.088	0.000	0.000	**0.088**	**0.088**
506 Uganda High Commission in Tanzania, Dar es Salaam	0.000	0.301	0.000	0.000	**0.301**	**0.301**	0.000	0.301	0.000	0.000	**0.301**	**0.301**	0.000	0.301	0.000	0.000	**0.301**	**0.301**
510 Uganda Embassy in the United States, Washington	0.000	0.736	0.000	0.000	**0.736**	**0.736**	0.000	0.736	0.000	0.000	**0.736**	**0.736**	0.000	0.736	0.000	0.000	**0.736**	**0.736**
511 Uganda Embassy in Egypt, Cairo	0.000	0.129	0.000	0.000	**0.129**	**0.129**	0.000	0.129	0.000	0.000	**0.129**	**0.129**	0.000	0.129	0.000	0.000	**0.129**	**0.129**

ANNEX 2: MEDIUM TERM EXPENDITURE FRAMEWORK (MTEF)2025/26 - 2027/28(Excl. Arrears and AIA)

Billion Uganda Shillings	FY2025/26 Budget Projections						FY2026/27 Budget Projections						FY2027/28 Budget Projections					
PROGRAMME/VOTE	Wage	Non-Wage Recurrent	Domestic Dev	External Financing	Total excl. External Financing	Total incl. External Financing	Wage	Non-Wage Recurrent	Domestic Dev	External Financing	Total excl. External Financing	Total incl. External Financing	Wage	Non-Wage Recurrent	Domestic Dev	External Financing	Total excl. External Financing	Total incl. External Financing
**18 DEVELOPMENT PLAN IMPLEMENTATION**																		
512 Uganda Embassy in Ethiopia, Addis Ababa	0.000	0.489	0.000	0.000	0.489	0.489	0.000	0.489	0.000	0.000	0.489	0.489	0.000	0.489	0.000	0.000	0.489	0.489
513 Uganda Embassy in China, Beijing	0.000	0.266	0.000	0.000	0.266	0.266	0.000	0.266	0.000	0.000	0.266	0.266	0.000	0.266	0.000	0.000	0.266	0.266
514 Uganda Embassy in Switzerland, Geneva	0.000	0.343	0.000	0.000	0.343	0.343	0.000	0.343	0.000	0.000	0.343	0.343	0.000	0.343	0.000	0.000	0.343	0.343
515 Uganda Embassy in Japan, Tokyo	0.000	0.100	0.000	0.000	0.100	0.100	0.000	0.100	0.000	0.000	0.100	0.100	0.000	0.100	0.000	0.000	0.100	0.100
516 Uganda Embassy in Saudi Arabia, Riyadh	0.000	0.520	0.000	0.000	0.520	0.520	0.000	0.520	0.000	0.000	0.520	0.520	0.000	0.520	0.000	0.000	0.520	0.520
517 Uganda Embassy in Denmark, Copenhagen	0.000	0.161	0.000	0.000	0.161	0.161	0.000	0.161	0.000	0.000	0.161	0.161	0.000	0.161	0.000	0.000	0.161	0.161
519 Uganda Embassy in Italy, Rome	0.000	0.035	0.000	0.000	0.035	0.035	0.000	0.035	0.000	0.000	0.035	0.035	0.000	0.035	0.000	0.000	0.035	0.035
523 Uganda Embassy in Germany, Berlin	0.000	0.053	0.000	0.000	0.053	0.053	0.000	0.053	0.000	0.000	0.053	0.053	0.000	0.053	0.000	0.000	0.053	0.053
524 Uganda Embassy in Iran, Tehran	0.000	0.252	0.000	0.000	0.252	0.252	0.000	0.252	0.000	0.000	0.252	0.252	0.000	0.252	0.000	0.000	0.252	0.252
527 Uganda Embassy in South Sudan, Juba	0.000	0.155	0.000	0.000	0.155	0.155	0.000	0.155	0.000	0.000	0.155	0.155	0.000	0.155	0.000	0.000	0.155	0.155
528 Uganda Embassy in United Arab Emirates, Abudhabi	0.000	1.600	0.000	0.000	1.600	1.600	0.000	1.600	0.000	0.000	1.600	1.600	0.000	1.600	0.000	0.000	1.600	1.600
535 Uganda Embassy in Algeria, Algiers	0.000	0.197	0.000	0.000	0.197	0.197	0.000	0.197	0.000	0.000	0.197	0.197	0.000	0.197	0.000	0.000	0.197	0.197
536 Uganda Embassy in Qatar, Doha	0.000	0.104	0.000	0.000	0.104	0.104	0.000	0.104	0.000	0.000	0.104	0.104	0.000	0.104	0.000	0.000	0.104	0.104
**Sub Total For: DEVELOPMENT PLAN IMPLEMENTATION**	299.633	20,405.604	307.928	0.000	21,013.165	21,013.165	329.596	24,969.168	431.100	0.000	25,729.864	25,729.864	329.596	25,037.381	431.100	0.000	25,798.077	25,798.077
**19 ADMINISTRATION OF JUSTICE**																		
018 Ministry of Gender, Labour and Social Development	0.000	0.612	0.000	0.000	0.612	0.612	0.000	0.826	0.000	0.000	0.826	0.826	0.000	0.826	0.000	0.000	0.826	0.826
101 Judiciary (Courts of Judicature)	125.626	264.096	75.611	0.000	465.333	465.333	138.188	356.529	105.856	0.000	600.574	600.574	138.188	356.529	105.856	0.000	600.574	600.574
133 Directorate of Public Prosecution (DPP)	4.451	9.779	0.000	0.000	14.230	14.230	4.897	13.201	0.000	0.000	18.098	18.098	4.897	13.201	0.000	0.000	18.098	18.098
144 Uganda Police Force	0.000	1.224	0.000	0.000	1.224	1.224	0.000	1.652	0.000	0.000	1.652	1.652	0.000	1.652	0.000	0.000	1.652	1.652
145 Uganda Prisons Service	0.000	0.000	1.200	0.000	1.200	1.200	0.000	0.000	1.680	0.000	1.680	1.680	0.000	0.000	1.680	0.000	1.680	1.680
148 Judicial Service Commission (JSC)	3.689	17.639	2.356	0.000	23.684	23.684	4.058	23.813	3.299	0.000	31.169	31.169	4.058	23.813	3.299	0.000	31.169	31.169
311 Law Development Centre	0.541	0.766	0.300	0.000	1.606	1.606	0.595	1.034	0.420	0.000	2.048	2.048	0.595	1.034	0.420	0.000	2.048	2.048

ANNEX 2: MEDIUM TERM EXPENDITURE FRAMEWORK (MTEF)2025/26 - 2027/28(Excl. Arrears and AIA)

Billion Uganda Shillings	FY2025/26 Budget Projections						FY2026/27 Budget Projections						FY2027/28 Budget Projections					
PROGRAMME/VOTE	Wage	Non-Wage Recurrent	Domestic Dev	External Financing	Total excl. External Financing	Total incl. External Financing	Wage	Non-Wage Recurrent	Domestic Dev	External Financing	Total excl. External Financing	Total incl. External Financing	Wage	Non-Wage Recurrent	Domestic Dev	External Financing	Total excl. External Financing	Total incl. External Financing
**Sub Total For: ADMINISTRATION OF JUSTICE**	134.306	294.115	79.468	0.000	**507.889**	**507.889**	147.737	397.055	111.255	0.000	**656.047**	**656.047**	147.737	397.055	111.255	0.000	**656.047**	**656.047**
**20 LEGISLATION, OVERSIGHT AND REPRESENTATION**																		
007 Ministry of Justice and Constitutional Affairs	0.000	0.122	0.000	0.000	**0.122**	**0.122**	0.000	0.165	0.000	0.000	**0.165**	**0.165**	0.000	0.165	0.000	0.000	**0.165**	**0.165**
011 Ministry of Local Government	0.000	0.122	0.000	0.000	**0.122**	**0.122**	0.000	0.165	0.000	0.000	**0.165**	**0.165**	0.000	0.165	0.000	0.000	**0.165**	**0.165**
104 Parliamentary Commission	129.966	905.470	80.989	0.000	**1,116.426**	**1,116.426**	142.963	1,222.385	113.385	0.000	**1,478.733**	**1,478.733**	142.963	1,222.385	113.385	0.000	**1,478.733**	**1,478.733**
**Sub Total For: LEGISLATION, OVERSIGHT AND REPRESENTATION**	129.966	905.715	80.989	0.000	**1,116.671**	**1,116.671**	142.963	1,222.715	113.385	0.000	**1,479.063**	**1,479.063**	142.963	1,222.715	113.385	0.000	**1,479.063**	**1,479.063**
**Grand Total**	**8,219.112**	**31,996.662**	**7,149.355**	**10,804.778**	**47,365.129**	**58,169.907**	**9,038.106**	**40,707.615**	**9,370.889**	**8,880.014**	**59,116.610**	**67,996.624**	**9,038.106**	**40,775.828**	**9,370.889**	**3,162.452**	**59,184.822**	**62,347.274**

ANNEX 3: SUB SUBPROGRAMME ALLOCATIONS FOR FY2023/24

	Recurrent				Development					
Billion Uganda Shillings	Wage	Non-Wage	AIA	Total	GoU Dev't	Ext. Fin	AIA	Total	Total Budget	Total Excl AIA
**Vote: 001 Office of the President**	25.591	156.173	0.000	**181.763**	22.360	0.000	0.000	22.360	**204.123**	204.123
01 Cabinet Support and Policy Development	0.000	4.171	0.000	**4.171**	0.000	0.000	0.000	**0.000**	**4.171**	**4.171**
02 Policy, planning and support services	25.310	32.311	0.000	**57.621**	22.360	0.000	0.000	**22.360**	**79.981**	**79.981**
03 Government Mobilisation,Monitoring and people centred security	0.000	54.000	0.000	**54.000**	0.000	0.000	0.000	**0.000**	**54.000**	**54.000**
04 Security Administration	0.000	24.701	0.000	**24.701**	0.000	0.000	0.000	**0.000**	**24.701**	**24.701**
05 Effective Security Management	0.000	26.713	0.000	**26.713**	0.000	0.000	0.000	**0.000**	**26.713**	**26.713**
08 Socio-Economic Monitoring and Research	0.281	10.277	0.000	**10.558**	0.000	0.000	0.000	**0.000**	**10.558**	**10.558**
09 Manifesto Monitoring and Evaluation	0.000	4.000	0.000	**4.000**	0.000	0.000	0.000	**0.000**	**4.000**	**4.000**
**Vote: 002 State House**	22.637	375.579	0.000	**398.216**	21.722	0.000	0.000	21.722	**419.938**	419.938
01 Logistical and Administrative Support to the Presidency	7.755	164.648	0.000	**172.404**	0.000	0.000	0.000	**0.000**	**172.404**	**172.404**
02 Policy, Planning and Support Services	12.495	141.135	0.000	**153.630**	21.722	0.000	0.000	**21.722**	**175.352**	**175.352**
03 Presidential Initiatives	2.386	69.795	0.000	**72.182**	0.000	0.000	0.000	**0.000**	**72.182**	**72.182**
**Vote: 003 Office of the Prime Minister**	4.110	53.795	0.000	**57.905**	16.348	38.936	0.000	55.284	**113.189**	113.189
01 Administration and Support Services	1.356	16.653	0.000	**18.009**	3.776	0.000	0.000	**3.776**	**21.785**	**21.785**
02 Affirmative Action Programs	0.347	0.200	0.000	**0.547**	2.500	0.000	0.000	**2.500**	**3.047**	**3.047**
03 Disaster Preparedness and Refugee Management	0.551	8.180	0.000	**8.731**	10.072	38.936	0.000	**49.008**	**57.739**	**57.739**
04 Executive Governance	1.190	19.712	0.000	**20.902**	0.000	0.000	0.000	**0.000**	**20.902**	**20.902**
05 Monitoring and Evaluation	0.362	5.429	0.000	**5.791**	0.000	0.000	0.000	**0.000**	**5.791**	**5.791**
06 Strategic Coordination and Implementation	0.305	3.621	0.000	**3.926**	0.000	0.000	0.000	**0.000**	**3.926**	**3.926**
**Vote: 004 Ministry of Defence**	617.579	765.262	0.000	**1,382.841**	1,982.727	194.679	0.000	2,177.406	**3,560.247**	3,560.247
01 National Defence (UPDF)	615.735	592.816	0.000	**1,208.551**	1,980.636	194.679	0.000	**2,175.316**	**3,383.867**	**3,383.867**
02 Policy, Planning and Support Services	1.844	172.446	0.000	**174.290**	2.090	0.000	0.000	**2.090**	**176.381**	**176.381**
**Vote: 005 Ministry of Public Service**	4.361	25.499	0.000	**29.860**	3.200	0.000	0.000	3.200	**33.060**	33.060
01 Human Resource Management	0.850	7.159	0.000	**8.009**	0.000	0.000	0.000	**0.000**	**8.009**	**8.009**
02 Inspection and Quality Assurance	0.374	1.206	0.000	**1.580**	0.000	0.000	0.000	**0.000**	**1.580**	**1.580**
03 Management Services	0.298	4.344	0.000	**4.642**	0.000	0.000	0.000	**0.000**	**4.642**	**4.642**
04 Policy, Planning and Support Services	2.839	12.790	0.000	**15.629**	3.200	0.000	0.000	**3.200**	**18.829**	**18.829**
**Vote: 006 Ministry of Foreign Affairs**	6.009	23.631	0.000	**29.640**	0.770	0.000	0.000	0.770	**30.410**	30.410
01 Policy, Planning and Support Services	6.009	18.635	0.000	**24.644**	0.770	0.000	0.000	**0.770**	**25.414**	**25.414**
02 Protocol and Public Diplomacy	0.000	1.051	0.000	**1.051**	0.000	0.000	0.000	**0.000**	**1.051**	**1.051**
03 Regional and International Economic Affairs	0.000	2.302	0.000	**2.302**	0.000	0.000	0.000	**0.000**	**2.302**	**2.302**

ANNEX 3: SUB SUBPROGRAMME ALLOCATIONS FOR FY2023/24

	Recurrent				Development					
Billion Uganda Shillings	Wage	Non-Wage	AIA	Total	GoU Dev't	Ext. Fin	AIA	Total	Total Budget	Total Excl AIA
**Vote: 006 Ministry of Foreign Affairs**	6.009	23.631	0.000	**29.640**	0.770	0.000	0.000	**0.770**	**30.410**	**30.410**
04 Regional and International Political Affairs	0.000	1.643	0.000	**1.643**	0.000	0.000	0.000	**0.000**	**1.643**	**1.643**
**Vote: 007 Ministry of Justice and Constitutional Affairs**	10.865	118.782	0.000	**129.648**	21.740	0.000	0.000	**21.740**	**151.388**	**151.388**
01 Administration of Estates/Property of the Deceased	1.494	0.785	0.000	**2.279**	0.000	0.000	0.000	**0.000**	**2.279**	**2.279**
02 Civil Litigation	1.894	1.343	0.000	**3.237**	0.000	0.000	0.000	**0.000**	**3.237**	**3.237**
03 Legal Advisory and Consultancy Services	1.571	1.209	0.000	**2.780**	0.000	0.000	0.000	**0.000**	**2.780**	**2.780**
04 First Parliamentary Counsel	1.186	3.202	0.000	**4.388**	0.000	0.000	0.000	**0.000**	**4.388**	**4.388**
05 Policy, Planning and Support Services	3.986	111.423	0.000	**115.409**	21.740	0.000	0.000	**21.740**	**137.149**	**137.149**
06 Regulation of the Legal Profession	0.734	0.821	0.000	**1.555**	0.000	0.000	0.000	**0.000**	**1.555**	**1.555**
**Vote: 008 Ministry of Finance, Planning and Economic Development**	9.296	1,856.070	0.000	**1,865.366**	182.778	265.737	0.000	**448.515**	**2,313.881**	**2,313.881**
01 Budget Preparation, Execution and Monitoring	1.208	34.918	0.000	**36.125**	99.337	1.585	0.000	**100.922**	**137.048**	**137.048**
02 Deficit Financing and Cash Management	0.711	8.348	0.000	**9.059**	0.298	0.000	0.000	**0.298**	**9.357**	**9.357**
03 Development Policy and Investment Promotion	0.226	26.285	0.000	**26.511**	2.236	257.950	0.000	**260.186**	**286.697**	**286.697**
04 Financial Sector Development	0.301	1,394.275	0.000	**1,394.575**	0.000	0.000	0.000	**0.000**	**1,394.575**	**1,394.575**
05 Internal Oversight and Advisory Services	0.441	7.806	0.000	**8.247**	0.000	0.000	0.000	**0.000**	**8.247**	**8.247**
06 Macroeconomic Policy and Management	0.557	18.497	0.000	**19.054**	1.590	0.220	0.000	**1.810**	**20.863**	**20.863**
07 Policy, Planning and Support Services	2.859	35.379	0.000	**38.237**	58.130	0.368	0.000	**58.498**	**96.736**	**96.736**
08 Public Financial Management	2.994	330.564	0.000	**333.558**	21.187	5.614	0.000	**26.801**	**360.358**	**360.358**
**Vote: 009 Ministry of Internal Affairs**	2.448	54.447	0.000	**56.896**	3.000	0.000	0.000	**3.000**	**59.896**	**59.896**
01 Combat Trafficking in Persons	0.000	0.317	0.000	**0.317**	0.000	0.000	0.000	**0.000**	**0.317**	**0.317**
02 Directorate of Community Service	0.000	3.598	0.000	**3.598**	0.000	0.000	0.000	**0.000**	**3.598**	**3.598**
03 Internal Security, Coordination and Advisory Services	0.000	15.896	0.000	**15.896**	0.000	0.000	0.000	**0.000**	**15.896**	**15.896**
04 Policy, Planning and Support Services	2.448	25.701	0.000	**28.150**	3.000	0.000	0.000	**3.000**	**31.150**	**31.150**
06 NGO Regulation	0.000	4.400	0.000	**4.400**	0.000	0.000	0.000	**0.000**	**4.400**	**4.400**
07 Peace Building	0.000	2.751	0.000	**2.751**	0.000	0.000	0.000	**0.000**	**2.751**	**2.751**
08 Police and Prisons Supervision	0.000	1.784	0.000	**1.784**	0.000	0.000	0.000	**0.000**	**1.784**	**1.784**
**Vote: 010 Ministry of Agriculture, Animal Industry and Fisheries**	19.407	36.730	0.000	**56.137**	139.100	423.260	0.000	**562.360**	**618.497**	**618.497**
01 Agriculture Extension Services	0.000	1.440	0.000	**1.440**	2.130	0.000	0.000	**2.130**	**3.570**	**3.570**
02 Agriculture Infrastructure and Mechanization Development	0.000	3.335	0.000	**3.335**	104.440	135.280	0.000	**239.720**	**243.055**	**243.055**
03 Animal Resources	0.000	4.829	0.000	**4.829**	0.000	0.000	0.000	**0.000**	**4.829**	**4.829**

ANNEX 3: SUB SUBPROGRAMME ALLOCATIONS FOR FY2023/24

	Recurrent				Development					
Billion Uganda Shillings	Wage	Non-Wage	AIA	Total	GoU Dev't	Ext. Fin	AIA	Total	Total Budget	Total Excl AIA
**Vote: 010 Ministry of Agriculture, Animal Industry and Fisheries**	19.407	36.730	0.000	**56.137**	139.100	423.260	0.000	**562.360**	**618.497**	**618.497**
04 Crop Resources	0.000	4.137	0.000	**4.137**	16.118	214.980	0.000	**231.098**	**235.235**	**235.235**
05 Fisheries Resources	0.000	2.715	0.000	**2.715**	4.241	0.000	0.000	**4.241**	**6.956**	**6.956**
06 Policy, Planning and Support Services	19.407	20.274	0.000	**39.681**	12.171	73.000	0.000	**85.171**	**124.852**	**124.852**
**Vote: 011 Ministry of Local Government**	25.665	41.677	0.000	**67.342**	23.400	52.524	0.000	**75.924**	**143.267**	**143.267**
01 Local Government Administration and Development	22.054	5.062	0.000	**27.116**	1.000	37.923	0.000	**38.923**	**66.039**	**66.039**
02 Local Government Inspection and Assessment	0.639	2.853	0.000	**3.492**	12.400	14.601	0.000	**27.001**	**30.492**	**30.492**
03 Policy, Planning and Support Services	2.972	33.763	0.000	**36.735**	10.000	0.000	0.000	**10.000**	**46.735**	**46.735**
**Vote: 012 Ministry of Lands, Housing & Urban Development**	11.194	23.850	0.000	**35.044**	15.880	117.100	0.000	**132.980**	**168.023**	**168.023**
01 Housing	0.368	0.772	0.000	**1.140**	0.000	0.000	0.000	**0.000**	**1.140**	**1.140**
02 Land, Administration and Management	8.706	8.580	0.000	**17.286**	10.280	70.377	0.000	**80.657**	**97.942**	**97.942**
03 Physical Planning and Urban Development	0.794	2.053	0.000	**2.847**	4.200	46.723	0.000	**50.923**	**53.770**	**53.770**
04 Policy, Planning and Support Services	1.326	12.445	0.000	**13.771**	1.400	0.000	0.000	**1.400**	**15.171**	**15.171**
**Vote: 013 Ministry of Education and Sports**	124.598	140.371	0.000	**264.969**	42.506	362.931	0.000	**405.437**	**670.405**	**670.405**
01 Career Guidance, Counselling and Placement	0.165	0.752	0.000	**0.917**	0.000	0.000	0.000	**0.000**	**0.917**	**0.917**
02 Higher Education	8.910	30.454	0.000	**39.364**	0.296	116.807	0.000	**117.103**	**156.467**	**156.467**
03 Sports and PE	0.125	6.537	0.000	**6.662**	0.000	0.000	0.000	**0.000**	**6.662**	**6.662**
04 Policy, Planning and Support Services	98.858	53.023	0.000	**151.882**	2.430	0.000	0.000	**2.430**	**154.312**	**154.312**
05 Basic and Secondary Education	1.879	15.787	0.000	**17.666**	25.400	161.584	0.000	**186.984**	**204.650**	**204.650**
06 Quality and Standards	1.599	2.809	0.000	**4.407**	0.000	0.000	0.000	**0.000**	**4.407**	**4.407**
07 Technical Vocational Education and Training	12.856	29.992	0.000	**42.848**	14.381	84.539	0.000	**98.920**	**141.768**	**141.768**
08 Special Needs Education	0.204	1.017	0.000	**1.221**	0.000	0.000	0.000	**0.000**	**1.221**	**1.221**
**Vote: 014 Ministry of Health**	21.645	64.922	0.000	**86.567**	54.477	1,104.249	0.000	**1,158.726**	**1,245.293**	**1,245.293**
01 Curative Services	4.929	28.212	0.000	**33.141**	0.000	0.000	0.000	**0.000**	**33.141**	**33.141**
02 Strategy, Policy and Development	1.427	7.193	0.000	**8.621**	33.535	46.480	0.000	**80.015**	**88.636**	**88.636**
03 Support Services	10.303	18.370	0.000	**28.673**	0.272	0.000	0.000	**0.272**	**28.945**	**28.945**
04 Health Governance and Regulation	0.793	1.641	0.000	**2.433**	0.000	0.000	0.000	**0.000**	**2.433**	**2.433**
05 Public Health Services	4.193	9.506	0.000	**13.699**	20.669	1,057.770	0.000	**1,078.438**	**1,092.138**	**1,092.138**
**Vote: 015 Ministry of Trade, Industry and Co-operatives**	3.174	43.380	0.000	**46.553**	0.226	0.000	0.000	**0.226**	**46.779**	**46.779**
01 Trade Development	0.414	0.423	0.000	**0.837**	0.000	0.000	0.000	**0.000**	**0.837**	**0.837**

ANNEX 3: SUB SUBPROGRAMME ALLOCATIONS FOR FY2023/24

	Recurrent				Development					
Billion Uganda Shillings	Wage	Non-Wage	AIA	Total	GoU Dev't	Ext. Fin	AIA	Total	Total Budget	Total Excl AIA
**Vote: 015 Ministry of Trade, Industry and Co-operatives**	3.174	43.380	0.000	**46.553**	0.226	0.000	0.000	**0.226**	**46.779**	**46.779**
02 Regulation and Management of Cooperatives	0.213	12.249	0.000	**12.462**	0.000	0.000	0.000	**0.000**	**12.462**	**12.462**
03 Policy, Planning and Support Services	0.968	12.285	0.000	**13.253**	0.226	0.000	0.000	**0.226**	**13.479**	**13.479**
04 Industrial and Technological Development	0.844	17.852	0.000	**18.696**	0.000	0.000	0.000	**0.000**	**18.696**	**18.696**
05 MSME Development	0.734	0.571	0.000	**1.305**	0.000	0.000	0.000	**0.000**	**1.305**	**1.305**
**Vote: 016 Ministry of Works and Transport**	14.758	91.625	0.000	**106.383**	190.220	1,758.892	0.000	**1,949.112**	**2,055.496**	**2,055.496**
01 Construction Standards and Quality Assurance	2.100	1.500	0.000	**3.600**	15.000	0.000	0.000	**15.000**	**18.600**	**18.600**
02 District, Urban and Community Access Roads	3.300	6.520	0.000	**9.820**	115.000	0.000	0.000	**115.000**	**124.820**	**124.820**
03 Mechanical Equipment, Plant and Ferry Services	2.020	38.000	0.000	**40.020**	0.000	0.000	0.000	**0.000**	**40.020**	**40.020**
04 Policy, Planning and Support Services	1.266	12.905	0.000	**14.171**	2.720	0.000	0.000	**2.720**	**16.891**	**16.891**
05 Multimodal Transport Regulation	5.572	1.700	0.000	**7.272**	15.700	0.000	0.000	**15.700**	**22.972**	**22.972**
06 Rail, Air and Inland Water Transport	0.500	31.000	0.000	**31.500**	41.800	1,758.892	0.000	**1,800.692**	**1,832.192**	**1,832.192**
**Vote: 017 Ministry of Energy and Mineral Development**	25.000	101.885	0.000	**126.885**	610.150	673.512	0.000	**1,283.662**	**1,410.547**	**1,410.547**
01 Mineral Exploration, Development & Value Addition	5.500	11.000	0.000	**16.500**	22.000	0.000	0.000	**22.000**	**38.500**	**38.500**
02 Energy Planning, Management & Infrastructure Dev't	6.047	10.300	0.000	**16.347**	449.630	673.512	0.000	**1,123.142**	**1,139.488**	**1,139.488**
03 Policy, Planning and Support Services	9.753	50.600	0.000	**60.353**	12.160	0.000	0.000	**12.160**	**72.513**	**72.513**
04 Petroleum Exploration, Development, Production, Value Addition and Distribution and Petroleum Products	3.700	29.985	0.000	**33.685**	126.360	0.000	0.000	**126.360**	**160.045**	**160.045**
**Vote: 018 Ministry of Gender, Labour and Social Development**	4.000	59.283	0.000	**63.283**	5.000	116.807	0.000	**121.807**	**185.090**	**185.090**
01 Adminstration, Planning and support services	1.868	9.427	0.000	**11.295**	5.000	0.000	0.000	**5.000**	**16.295**	**16.295**
02 Community Mobilisation, Culture and Empowermen	0.500	1.573	0.000	**2.073**	0.000	0.000	0.000	**0.000**	**2.073**	**2.073**
03 Gender and social protection	0.860	40.607	0.000	**41.467**	0.000	0.000	0.000	**0.000**	**41.467**	**41.467**
04 Labour and Employment services	0.771	7.676	0.000	**8.447**	0.000	116.807	0.000	**116.807**	**125.255**	**125.255**
**Vote: 019 Ministry of Water and Environment**	16.144	15.980	0.000	**32.124**	468.410	1,070.262	0.000	**1,538.672**	**1,570.797**	**1,570.797**
01 Directorate of Environmental Affairs	3.907	3.100	0.000	**7.007**	28.613	162.119	0.000	**190.732**	**197.739**	**197.739**
02 Directorate of Water Resources Management	4.384	1.520	0.000	**5.904**	40.956	42.700	0.000	**83.656**	**89.560**	**89.560**
03 Directorate of Water Development	5.984	0.390	0.000	**6.374**	383.190	861.993	0.000	**1,245.183**	**1,251.557**	**1,251.557**
04 Policy, Planning and Support Services	1.869	10.970	0.000	**12.839**	15.651	3.450	0.000	**19.101**	**31.940**	**31.940**

ANNEX 3: SUB SUBPROGRAMME ALLOCATIONS FOR FY2023/24

	Recurrent				Development					
Billion Uganda Shillings	Wage	Non-Wage	AIA	Total	GoU Dev't	Ext. Fin	AIA	Total	Total Budget	Total Excl AIA
**Vote: 020 Ministry of ICT and National Guidance**	6.731	23.486	0.000	**30.217**	19.548	0.000	0.000	**19.548**	**49.765**	**49.765**
01 Effective Communication and National Guidance	1.020	1.098	0.000	**2.118**	0.000	0.000	0.000	**0.000**	**2.118**	**2.118**
02 Enabling enviroment for ICT Development and Regulation	0.733	6.660	0.000	**7.393**	0.000	0.000	0.000	**0.000**	**7.393**	**7.393**
03 Policy, Planning and Support Services	4.978	15.727	0.000	**20.705**	19.548	0.000	0.000	**19.548**	**40.253**	**40.253**
**Vote: 021 Ministry of East African Community Affairs**	1.280	37.561	0.000	**38.841**	1.325	0.000	0.000	**1.325**	**40.166**	**40.166**
01 Regional Integration	0.000	3.467	0.000	**3.467**	0.000	0.000	0.000	**0.000**	**3.467**	**3.467**
02 Policy, Planning and Support Services	1.280	34.094	0.000	**35.374**	1.325	0.000	0.000	**1.325**	**36.700**	**36.700**
**Vote: 022 Ministry of Tourism, Wildlife and Antiquities**	2.371	30.080	0.000	**32.451**	52.640	0.000	0.000	**52.640**	**85.091**	**85.091**
01 Policy, Planning and Support Services	0.755	4.508	0.000	**5.263**	17.900	0.000	0.000	**17.900**	**23.163**	**23.163**
02 Tourism, Wildlife Conservation and Museums	1.616	25.572	0.000	**27.188**	34.740	0.000	0.000	**34.740**	**61.928**	**61.928**
**Vote: 023 Ministry of Kampala Capital City and Metropolitan Affairs**	0.490	2.840	0.000	**3.330**	0.000	0.000	0.000	**0.000**	**3.330**	**3.330**
01 General Management, Administration and Corporate Planning	0.490	0.840	0.000	**1.330**	0.000	0.000	0.000	**0.000**	**1.330**	**1.330**
02 Economic Development	0.000	1.250	0.000	**1.250**	0.000	0.000	0.000	**0.000**	**1.250**	**1.250**
03 Spatial Planning	0.000	0.750	0.000	**0.750**	0.000	0.000	0.000	**0.000**	**0.750**	**0.750**
**Vote: 101 Judiciary (Courts of Judicature)**	108.767	215.764	0.000	**324.531**	63.010	0.000	0.000	**63.010**	**387.541**	**387.541**
01 Case Management	98.933	86.175	0.000	**185.108**	0.000	0.000	0.000	**0.000**	**185.108**	**185.108**
02 Judiciary General Administration	9.077	124.650	0.000	**133.727**	63.010	0.000	0.000	**63.010**	**196.737**	**196.737**
03 Capacity Building	0.757	4.939	0.000	**5.696**	0.000	0.000	0.000	**0.000**	**5.696**	**5.696**
**Vote: 102 Electoral Commission (EC)**	37.667	104.910	0.000	**142.577**	3.720	0.000	0.000	**3.720**	**146.297**	**146.297**
01 Operations	0.000	11.068	0.000	**11.068**	0.000	0.000	0.000	**0.000**	**11.068**	**11.068**
02 Technical Support Services	0.000	8.672	0.000	**8.672**	0.000	0.000	0.000	**0.000**	**8.672**	**8.672**
03 General Administration and Support Services	37.667	85.170	0.000	**122.837**	3.720	0.000	0.000	**3.720**	**126.557**	**126.557**
**Vote: 103 Inspectorate of Government (IG)**	27.953	36.026	0.000	**63.979**	24.597	0.000	0.000	**24.597**	**88.576**	**88.576**
01 Anti-Corruption	18.968	20.292	0.000	**39.260**	0.000	0.000	0.000	**0.000**	**39.260**	**39.260**
02 General Administration and Support Services	6.878	14.417	0.000	**21.294**	24.597	0.000	0.000	**24.597**	**45.891**	**45.891**
03 Ombudsman	2.108	1.317	0.000	**3.425**	0.000	0.000	0.000	**0.000**	**3.425**	**3.425**
**Vote: 104 Parliamentary Commission**	112.525	739.763	0.000	**852.288**	67.491	0.000	0.000	**67.491**	**919.779**	**919.779**
01 Corporate Affairs	0.000	56.033	0.000	**56.033**	0.000	0.000	0.000	**0.000**	**56.033**	**56.033**
02 General Administration and support to Parliament	38.025	159.521	0.000	**197.546**	67.491	0.000	0.000	**67.491**	**265.037**	**265.037**
03 Parliamentary Affairs	74.500	524.209	0.000	**598.709**	0.000	0.000	0.000	**0.000**	**598.709**	**598.709**

ANNEX 3: SUB SUBPROGRAMME ALLOCATIONS FOR FY2023/24

	Recurrent				Development					
Billion Uganda Shillings	Wage	Non-Wage	AIA	Total	GoU Dev't	Ext. Fin	AIA	Total	Total Budget	Total Excl AIA
**Vote: 105 Law Reform Commission (LRC)**	4.073	13.957	0.000	**18.030**	0.420	0.000	0.000	**0.420**	**18.450**	**18.450**
01 Advocay for law reform	0.000	0.806	0.000	**0.806**	0.000	0.000	0.000	**0.000**	**0.806**	**0.806**
02 General administration and support services	1.753	3.547	0.000	**5.300**	0.420	0.000	0.000	**0.420**	**5.720**	**5.720**
03 Translate, simplify and disseminate laws	0.826	1.549	0.000	**2.375**	0.000	0.000	0.000	**0.000**	**2.375**	**2.375**
04 Reform of laws	1.494	3.443	0.000	**4.937**	0.000	0.000	0.000	**0.000**	**4.937**	**4.937**
05 Publications	0.000	4.612	0.000	**4.612**	0.000	0.000	0.000	**0.000**	**4.612**	**4.612**
**Vote: 106 Uganda Human Rights Commission (UHRC)**	7.595	11.446	0.000	**19.041**	0.531	0.000	0.000	**0.531**	**19.572**	**19.572**
01 General Administration and Support Services	7.595	11.046	0.000	**18.641**	0.531	0.000	0.000	**0.531**	**19.172**	**19.172**
02 Protection and Promotion of Human Rights	0.000	0.400	0.000	**0.400**	0.000	0.000	0.000	**0.000**	**0.400**	**0.400**
**Vote: 107 Uganda Aids Commission (UAC)**	4.964	11.014	0.000	**15.978**	1.221	0.000	0.000	**1.221**	**17.199**	**17.199**
01 National HIV&AIDS Response Coordination	4.964	11.014	0.000	**15.978**	1.221	0.000	0.000	**1.221**	**17.199**	**17.199**
**Vote: 108 National Planning Authority (NPA)**	14.611	24.177	0.000	**38.788**	2.910	0.000	0.000	**2.910**	**41.698**	**41.698**
01 Development Planning	0.000	10.383	0.000	**10.383**	0.000	0.000	0.000	**0.000**	**10.383**	**10.383**
02 Development Performance	0.000	0.200	0.000	**0.200**	0.000	0.000	0.000	**0.000**	**0.200**	**0.200**
03 General administration and support services	14.611	13.594	0.000	**28.205**	2.910	0.000	0.000	**2.910**	**31.115**	**31.115**
**Vote: 109 Uganda National Meteorological Authority (UNMA)**	7.413	3.670	0.000	**11.083**	6.060	0.000	0.000	**6.060**	**17.143**	**17.143**
01 National Meteorological Services	7.413	3.670	0.000	**11.083**	6.060	0.000	0.000	**6.060**	**17.143**	**17.143**
**Vote: 110 Uganda Industrial Research Institute (UIRI)**	5.860	1.500	0.000	**7.360**	0.000	0.000	0.000	**0.000**	**7.360**	**7.360**
01 Industrial Research	5.860	1.500	0.000	**7.360**	0.000	0.000	0.000	**0.000**	**7.360**	**7.360**
**Vote: 111 National Curriculum Development Centre (NCDC)**	8.554	11.671	0.000	**20.225**	3.374	0.000	0.000	**3.374**	**23.599**	**23.599**
01 Curriculum and Instructional Materials Development	0.000	7.216	0.000	**7.216**	0.000	0.000	0.000	**0.000**	**7.216**	**7.216**
02 General Administration and Support Services	8.554	3.586	0.000	**12.140**	3.374	0.000	0.000	**3.374**	**15.514**	**15.514**
03 Research, Consultancy and Library Services	0.000	0.868	0.000	**0.868**	0.000	0.000	0.000	**0.000**	**0.868**	**0.868**
**Vote: 112 Directorate of Ethics and Integrity (DEI)**	6.323	8.124	0.000	**14.447**	0.654	0.000	0.000	**0.654**	**15.101**	**15.101**
01 Ethics and Integrity	6.323	8.124	0.000	**14.447**	0.654	0.000	0.000	**0.654**	**15.101**	**15.101**
**Vote: 113 Uganda National Roads Authority (UNRA)**	71.105	40.127	0.000	**111.232**	590.080	1,210.473	0.000	**1,800.553**	**1,911.785**	**1,911.785**
01 National Roads Maintenance and Construction	71.105	40.127	0.000	**111.232**	590.080	1,210.473	0.000	**1,800.553**	**1,911.785**	**1,911.785**
**Vote: 114 Uganda Cancer Institute (UCI)**	9.293	18.930	0.000	**28.222**	0.679	0.000	0.000	**0.679**	**28.901**	**28.901**
01 Cancer Services	9.293	18.930	0.000	**28.222**	0.679	0.000	0.000	**0.679**	**28.901**	**28.901**

ANNEX 3: SUB SUBPROGRAMME ALLOCATIONS FOR FY2023/24

	Recurrent				Development					
Billion Uganda Shillings	Wage	Non-Wage	AIA	Total	GoU Dev't	Ext. Fin	AIA	Total	Total Budget	Total Excl AIA
**Vote: 115 Uganda Heart Institute (UHI)**	6.614	10.404	0.000	**17.018**	8.924	0.000	0.000	**8.924**	**25.942**	**25.942**
01 Heart Services	6.614	10.404	0.000	**17.018**	8.924	0.000	0.000	**8.924**	**25.942**	**25.942**
**Vote: 116 Uganda National Medical Stores**	19.500	487.543	0.000	**507.043**	6.652	0.000	0.000	**6.652**	**513.695**	**513.695**
01 Pharmaceutical and Medical Supplies	19.500	487.543	0.000	**507.043**	6.652	0.000	0.000	**6.652**	**513.695**	**513.695**
**Vote: 117 Uganda Tourism Board (UTB)**	1.855	2.350	0.000	**4.205**	0.100	0.000	0.000	**0.100**	**4.305**	**4.305**
01 Quality Assurance, Research and Planning	0.180	0.000	0.000	**0.180**	0.000	0.000	0.000	**0.000**	**0.180**	**0.180**
02 Marketing and Product Development	0.499	0.000	0.000	**0.499**	0.000	0.000	0.000	**0.000**	**0.499**	**0.499**
03 General Administration and Support Services	1.176	2.350	0.000	**3.526**	0.100	0.000	0.000	**0.100**	**3.626**	**3.626**
**Vote: 118 Uganda Road Fund (URF)**	2.667	465.285	0.000	**467.952**	0.000	0.000	0.000	**0.000**	**467.952**	**467.952**
01 National and District Road Maintenance	2.667	465.285	0.000	**467.952**	0.000	0.000	0.000	**0.000**	**467.952**	**467.952**
**Vote: 119 Uganda Registration Services Bureau (URSB)**	13.352	19.508	0.000	**32.860**	1.267	0.000	0.000	**1.267**	**34.127**	**34.127**
01 General administration, planning, policy and support services	9.339	17.304	0.000	**26.642**	1.000	0.000	0.000	**1.000**	**27.642**	**27.642**
02 Lawful Registration Services	4.013	2.205	0.000	**6.218**	0.267	0.000	0.000	**0.267**	**6.485**	**6.485**
**Vote: 120 National Citizenship and Immigration Control (NCIC)**	5.274	104.021	0.000	**109.295**	6.320	0.000	0.000	**6.320**	**115.615**	**115.615**
01 Citizenship and Immigration Services	0.000	76.842	0.000	**76.842**	0.000	0.000	0.000	**0.000**	**76.842**	**76.842**
02 General administration, planning, policy and support services	5.274	27.179	0.000	**32.453**	6.320	0.000	0.000	**6.320**	**38.773**	**38.773**
**Vote: 121 Dairy Development Authority (DDA)**	4.218	8.240	0.000	**12.458**	0.760	0.000	0.000	**0.760**	**13.218**	**13.218**
01 Dairy Development and Regulation	4.218	8.240	0.000	**12.458**	0.760	0.000	0.000	**0.760**	**13.218**	**13.218**
**Vote: 122 Kampala Capital City Authority (KCCA)**	124.955	51.565	0.000	**176.521**	13.939	186.006	0.000	**199.945**	**376.466**	**376.466**
01 Community Health Management	8.433	4.415	0.000	**12.849**	0.938	0.000	0.000	**0.938**	**13.786**	**13.786**
02 Economic Policy Monitoring,Evaluation & Inspection	79.596	17.746	0.000	**97.341**	0.422	0.000	0.000	**0.422**	**97.763**	**97.763**
03 Education and Social Services	36.926	9.920	0.000	**46.846**	2.226	0.000	0.000	**2.226**	**49.072**	**49.072**
07 Revenue collection and mobilisation	0.000	1.255	0.000	**1.255**	0.013	0.000	0.000	**0.013**	**1.268**	**1.268**
08 Sanitation and Environmental Services	0.000	16.395	0.000	**16.395**	0.340	0.000	0.000	**0.340**	**16.735**	**16.735**
11 Urban Commercial and Production Services	0.000	0.350	0.000	**0.350**	0.000	0.000	0.000	**0.000**	**0.350**	**0.350**
12 Urban Planning, Security and Land Use	0.000	1.485	0.000	**1.485**	0.000	0.000	0.000	**0.000**	**1.485**	**1.485**
13 Urban Road Network Development	0.000	0.000	0.000	**0.000**	10.000	186.006	0.000	**196.006**	**196.006**	**196.006**
**Vote: 123 National Lotteries and Gaming Regulatory Board**	2.484	0.832	0.000	**3.316**	0.000	0.000	0.000	**0.000**	**3.316**	**3.316**
01 Legal and Board Affairs	0.000	0.490	0.000	**0.490**	0.000	0.000	0.000	**0.000**	**0.490**	**0.490**

ANNEX 3: SUB SUBPROGRAMME ALLOCATIONS FOR FY2023/24

	Recurrent				Development					
Billion Uganda Shillings	Wage	Non-Wage	AIA	Total	GoU Dev't	Ext. Fin	AIA	Total	Total Budget	Total Excl AIA
**Vote: 123 National Lotteries and Gaming Regulatory Board**	2.484	0.832	0.000	**3.316**	0.000	0.000	0.000	**0.000**	**3.316**	**3.316**
02 Policy, Planning and Support Services	2.484	0.342	0.000	**2.826**	0.000	0.000	0.000	**0.000**	**2.826**	**2.826**
**Vote: 124 Equal Opportunities Commission**	2.967	12.136	0.000	**15.103**	0.216	0.000	0.000	**0.216**	**15.319**	**15.319**
01 Gender and Equity	0.000	2.770	0.000	**2.770**	0.000	0.000	0.000	**0.000**	**2.770**	**2.770**
02 Redressing imbalances and promoting equal opportunites	2.967	9.366	0.000	**12.333**	0.216	0.000	0.000	**0.216**	**12.549**	**12.549**
**Vote: 125 National Animal Genetic Resource Centre and Data Bank (NAGRC&DB)**	5.736	7.860	0.000	**13.596**	66.760	0.000	0.000	**66.760**	**80.356**	**80.356**
01 Breeding and Genetic Improvement	5.736	7.860	0.000	**13.596**	66.760	0.000	0.000	**66.760**	**80.356**	**80.356**
**Vote: 126 National Information Technologies Authority**	11.211	35.649	0.000	**46.860**	4.788	79.818	0.000	**84.607**	**131.467**	**131.467**
01 Data protection and privacy	0.000	0.365	0.000	**0.365**	0.000	0.000	0.000	**0.000**	**0.365**	**0.365**
02 General Administration and support services	11.211	10.477	0.000	**21.688**	0.324	0.000	0.000	**0.324**	**22.013**	**22.013**
03 Electronic Public Services Delivery	0.000	8.028	0.000	**8.028**	0.000	0.000	0.000	**0.000**	**8.028**	**8.028**
04 National Cyber Security	0.000	0.645	0.000	**0.645**	0.000	0.000	0.000	**0.000**	**0.645**	**0.645**
05 IT infrastructure	0.000	16.134	0.000	**16.134**	4.464	79.818	0.000	**84.282**	**100.416**	**100.416**
**Vote: 127 Uganda Virus Research Institute (UVRI)**	2.218	5.514	0.000	**7.732**	0.000	0.000	0.000	**0.000**	**7.732**	**7.732**
01 Virus Research	2.218	5.514	0.000	**7.732**	0.000	0.000	0.000	**0.000**	**7.732**	**7.732**
**Vote: 128 Uganda National Examination Board (UNEB)**	12.360	101.044	0.000	**113.404**	31.424	0.000	0.000	**31.424**	**144.828**	**144.828**
01 National Examinations Assessment and Certification	0.000	85.812	0.000	**85.812**	0.000	0.000	0.000	**0.000**	**85.812**	**85.812**
02 General Administration and Support Services	12.360	15.231	0.000	**27.591**	31.424	0.000	0.000	**31.424**	**59.015**	**59.015**
**Vote: 129 Financial Intelligence Authority (FIA)**	9.594	16.928	0.000	**26.522**	0.729	0.000	0.000	**0.729**	**27.251**	**27.251**
01 Directorate of Finance and Administration	9.594	9.089	0.000	**18.683**	0.729	0.000	0.000	**0.729**	**19.412**	**19.412**
02 Directorate of Internal Audit	0.000	0.052	0.000	**0.052**	0.000	0.000	0.000	**0.000**	**0.052**	**0.052**
03 Directorate of Systems Administration and Security	0.000	1.651	0.000	**1.651**	0.000	0.000	0.000	**0.000**	**1.651**	**1.651**
04 Directorate of Analysis and Monitoring	0.000	4.877	0.000	**4.877**	0.000	0.000	0.000	**0.000**	**4.877**	**4.877**
05 Directorate of Compliance and Training	0.000	1.162	0.000	**1.162**	0.000	0.000	0.000	**0.000**	**1.162**	**1.162**
06 Directorate of Legal, Corporate Services and International Relations	0.000	0.097	0.000	**0.097**	0.000	0.000	0.000	**0.000**	**0.097**	**0.097**
**Vote: 130 Treasury Operations**	0.000	17,565.632	0.000	**17,565.632**	0.000	0.000	0.000	**0.000**	**17,565.632**	**17,565.632**
01 Treasury Operations	0.000	17,565.632	0.000	**17,565.632**	0.000	0.000	0.000	**0.000**	**17,565.632**	**17,565.632**
**Vote: 131 Office of the Auditor General (OAG)**	43.525	52.477	0.000	**96.002**	3.760	0.000	0.000	**3.760**	**99.762**	**99.762**
01 External Audit Services	33.295	21.421	0.000	**54.715**	0.000	0.000	0.000	**0.000**	**54.715**	**54.715**

ANNEX 3: SUB SUBPROGRAMME ALLOCATIONS FOR FY2023/24

Billion Uganda Shillings	Recurrent				Development					
	Wage	Non-Wage	AIA	Total	GoU Dev't	Ext. Fin	AIA	Total	Total Budget	Total Excl AIA
**Vote: 131 Office of the Auditor General (OAG)**	43.525	52.477	0.000	**96.002**	3.760	0.000	0.000	**3.760**	**99.762**	**99.762**
02 Support to Audit services	10.230	31.056	0.000	**41.287**	3.760	0.000	0.000	**3.760**	**45.047**	**45.047**
**Vote: 132 Education Service Commission (ESC)**	2.884	6.509	0.000	**9.393**	2.436	0.000	0.000	**2.436**	**11.830**	**11.830**
01 General Administration and Support Services	2.884	3.631	0.000	**6.516**	0.000	0.000	0.000	**0.000**	**6.516**	**6.516**
02 Management of Education Service Personnel	0.000	2.263	0.000	**2.263**	2.436	0.000	0.000	**2.436**	**4.699**	**4.699**
03 Research, Policy and Management Services	0.000	0.615	0.000	**0.615**	0.000	0.000	0.000	**0.000**	**0.615**	**0.615**
**Vote: 133 Directorate of Public Prosecution (DPP)**	27.943	42.595	0.000	**70.538**	17.041	0.000	0.000	**17.041**	**87.579**	**87.579**
01 Inspection and Quality Assurance Services	0.310	1.874	0.000	**2.183**	0.000	0.000	0.000	**0.000**	**2.183**	**2.183**
02 International Affairs	1.608	2.895	0.000	**4.502**	0.000	0.000	0.000	**0.000**	**4.502**	**4.502**
03 Management and Support Services	22.171	29.838	0.000	**52.009**	17.041	0.000	0.000	**17.041**	**69.050**	**69.050**
04 Prosecution	3.854	7.989	0.000	**11.843**	0.000	0.000	0.000	**0.000**	**11.843**	**11.843**
**Vote: 134 Health Service Commission (HSC)**	2.433	9.481	0.000	**11.914**	0.053	0.000	0.000	**0.053**	**11.967**	**11.967**
01 Human Resource Management for Health	2.433	9.481	0.000	**11.914**	0.053	0.000	0.000	**0.053**	**11.967**	**11.967**
**Vote: 135 Directorate of Government Analytical Laboratory (DGAL)**	3.318	25.448	0.000	**28.766**	4.620	0.000	0.000	**4.620**	**33.386**	**33.386**
01 Forensic and General Scientific Services	3.318	25.448	0.000	**28.766**	4.620	0.000	0.000	**4.620**	**33.386**	**33.386**
**Vote: 136 Uganda Export Promotion Board (UEPB)**	2.561	5.833	0.000	**8.395**	0.037	0.000	0.000	**0.037**	**8.432**	**8.432**
01 Export Market Development, Export Promotion and Customized Advisory Services	2.561	5.833	0.000	**8.395**	0.037	0.000	0.000	**0.037**	**8.432**	**8.432**
**Vote: 137 National Identification and Registration Authority (NIRA)**	20.335	36.401	0.000	**56.736**	3.000	0.000	0.000	**3.000**	**59.736**	**59.736**
01 Identification and Registration Services	13.517	14.018	0.000	**27.535**	0.000	0.000	0.000	**0.000**	**27.535**	**27.535**
02 Policy, Planning and Support Services	6.818	22.383	0.000	**29.201**	3.000	0.000	0.000	**3.000**	**32.201**	**32.201**
**Vote: 138 Uganda Investment Authority (UIA)**	6.833	8.226	0.000	**15.059**	1.404	233.615	0.000	**235.019**	**250.078**	**250.078**
01 Investment Promotion and Facilitation	0.000	2.000	0.000	**2.000**	0.000	233.615	0.000	**233.615**	**235.615**	**235.615**
02 General Administration and Support Services	6.833	6.226	0.000	**13.059**	1.404	0.000	0.000	**1.404**	**14.463**	**14.463**
**Vote: 139 Petroleum Authority of Uganda (PAU)**	28.718	37.705	0.000	**66.423**	27.992	0.000	0.000	**27.992**	**94.415**	**94.415**
01 Petroleum Regulation and Monitoring	17.372	15.965	0.000	**33.337**	22.446	0.000	0.000	**22.446**	**55.784**	**55.784**
02 Policy, Planning and Support Services	11.346	21.740	0.000	**33.086**	5.546	0.000	0.000	**5.546**	**38.631**	**38.631**
**Vote: 140 Capital Markets Authority**	3.475	5.095	0.000	**8.570**	0.000	0.000	0.000	**0.000**	**8.570**	**8.570**
01 Investor Protection, Supervision, Research and Market Development	1.170	1.154	0.000	**2.324**	0.000	0.000	0.000	**0.000**	**2.324**	**2.324**
02 General Administration and Support Services	2.305	3.941	0.000	**6.246**	0.000	0.000	0.000	**0.000**	**6.246**	**6.246**

ANNEX 3: SUB SUBPROGRAMME ALLOCATIONS FOR FY2023/24

	Recurrent				Development					
Billion Uganda Shillings	Wage	Non-Wage	AIA	Total	GoU Dev't	Ext. Fin	AIA	Total	Total Budget	Total Excl AIA
**Vote: 141 Uganda Revenue Authority (URA)**	205.495	316.325	0.000	521.820	45.320	0.000	0.000	45.320	567.140	567.140
01 Administration and Support Services	51.200	171.610	0.000	222.810	45.320	0.000	0.000	45.320	268.130	268.130
02 Revenue Collection & Administration	154.295	144.715	0.000	299.010	0.000	0.000	0.000	0.000	299.010	299.010
**Vote: 142 National Agricultural Research Organization (NARO)**	38.811	26.690	0.000	65.501	35.970	0.000	0.000	35.970	101.471	101.471
01 Agricultural Research	38.811	26.690	0.000	65.501	35.970	0.000	0.000	35.970	101.471	101.471
**Vote: 143 Uganda Bureau of Statistics (UBOS)**	21.391	214.726	0.000	236.117	13.470	0.000	0.000	13.470	249.587	249.587
01 Corporate Services	6.934	13.360	0.000	20.294	13.470	0.000	0.000	13.470	33.764	33.764
02 Digital Solutions and Data Capability	2.342	1.949	0.000	4.291	0.000	0.000	0.000	0.000	4.291	4.291
03 Economic Statistics	5.157	9.858	0.000	15.015	0.000	0.000	0.000	0.000	15.015	15.015
04 Methodology and Statistical Coordination Services	4.100	184.009	0.000	188.109	0.000	0.000	0.000	0.000	188.109	188.109
05 Population and Social Statistics	2.856	5.551	0.000	8.407	0.000	0.000	0.000	0.000	8.407	8.407
**Vote: 144 Uganda Police Force**	383.034	306.434	0.000	689.468	134.261	0.000	0.000	134.261	823.729	823.729
01 Crime Prevention and Investigation Management	80.400	61.300	0.000	141.700	0.000	0.000	0.000	0.000	141.700	141.700
02 Emergency Response & Specialized policing	37.432	28.765	0.000	66.197	0.000	0.000	0.000	0.000	66.197	66.197
03 General Administration and Support Services	136.044	177.199	0.000	313.244	134.261	0.000	0.000	134.261	447.505	447.505
04 Territorial Policing	129.158	39.170	0.000	168.328	0.000	0.000	0.000	0.000	168.328	168.328
**Vote: 145 Uganda Prisons Service**	95.630	186.719	0.000	282.349	27.371	0.000	0.000	27.371	309.720	309.720
01 Management and Administration	31.378	40.738	0.000	72.116	0.841	0.000	0.000	0.841	72.957	72.957
02 Safety and Security	3.033	8.658	0.000	11.691	0.000	0.000	0.000	0.000	11.691	11.691
03 Human Rights and Welfare	14.573	128.491	0.000	143.064	0.000	0.000	0.000	0.000	143.064	143.064
04 Prisons Production	0.000	0.000	0.000	0.000	25.530	0.000	0.000	25.530	25.530	25.530
05 Rehabilitation and re-integration of Offenders	0.641	4.557	0.000	5.198	0.000	0.000	0.000	0.000	5.198	5.198
06 Prisoners Management	46.004	4.275	0.000	50.279	1.000	0.000	0.000	1.000	51.279	51.279
**Vote: 146 Public Service Commission (PSC)**	3.289	8.201	0.000	11.490	1.400	0.000	0.000	1.400	12.890	12.890
01 Public Service Selection and Recruitment	3.289	8.201	0.000	11.490	1.400	0.000	0.000	1.400	12.890	12.890
**Vote: 147 Local Government Finance Commission (LGFC)**	1.619	9.359	0.000	10.978	0.100	0.000	0.000	0.100	11.078	11.078
01 Finance and Administration	0.890	5.837	0.000	6.727	0.100	0.000	0.000	0.100	6.827	6.827
02 Local Government Financing	0.729	3.522	0.000	4.250	0.000	0.000	0.000	0.000	4.250	4.250
**Vote: 148 Judicial Service Commission (JSC)**	3.194	14.411	0.000	17.605	1.964	0.000	0.000	1.964	19.568	19.568
01 Complaints, Investigation and Disciplinary Affairs	1.308	1.604	0.000	2.913	0.000	0.000	0.000	0.000	2.913	2.913
02 General administration and support services	0.949	11.697	0.000	12.646	1.964	0.000	0.000	1.964	14.610	14.610

ANNEX 3: SUB SUBPROGRAMME ALLOCATIONS FOR FY2023/24

	Recurrent				Development					
Billion Uganda Shillings	Wage	Non-Wage	AIA	Total	GoU Dev't	Ext. Fin	AIA	Total	Total Budget	Total Excl AIA
**Vote: 148 Judicial Service Commission (JSC)**	3.194	14.411	0.000	**17.605**	1.964	0.000	0.000	**1.964**	**19.568**	**19.568**
03 Legal Education, Public Affairs and research	0.936	1.109	0.000	**2.045**	0.000	0.000	0.000	**0.000**	**2.045**	**2.045**
**Vote: 149 National Population Council**	3.297	8.452	0.000	**11.749**	0.541	0.000	0.000	**0.541**	**12.290**	**12.290**
01 Policy, Planning and Support Services	2.169	4.684	0.000	**6.853**	0.541	0.000	0.000	**0.541**	**7.394**	**7.394**
02 Population Advocacy, Family Health and Communication	1.129	3.768	0.000	**4.896**	0.000	0.000	0.000	**0.000**	**4.896**	**4.896**
**Vote: 150 National Environment Management Authority (NEMA)**	6.722	9.530	0.000	**16.252**	6.520	0.000	0.000	**6.520**	**22.772**	**22.772**
01 Environmental Management	6.722	9.530	0.000	**16.252**	6.520	0.000	0.000	**6.520**	**22.772**	**22.772**
**Vote: 151 Uganda Blood Transfusion Service (UBTS)**	6.091	14.574	0.000	**20.665**	1.234	0.000	0.000	**1.234**	**21.899**	**21.899**
01 Safe Blood Provision	6.091	14.574	0.000	**20.665**	1.234	0.000	0.000	**1.234**	**21.899**	**21.899**
**Vote: 152 National Agricultural Advisory Services (NAADS)**	3.309	30.026	0.000	**33.335**	0.480	0.000	0.000	**0.480**	**33.815**	**33.815**
01 Agricultural Value Chain & Agribusiness Development	3.309	30.026	0.000	**33.335**	0.480	0.000	0.000	**0.480**	**33.815**	**33.815**
**Vote: 153 Public Procurement & Disposal of Public Assets (PPDA)**	11.955	8.175	0.000	**20.130**	3.000	0.000	0.000	**3.000**	**23.130**	**23.130**
01 Regulation of the Procurement and Disposal System	7.412	3.146	0.000	**10.558**	0.000	0.000	0.000	**0.000**	**10.558**	**10.558**
02 General Administration and Support Services	4.543	5.029	0.000	**9.572**	3.000	0.000	0.000	**3.000**	**12.572**	**12.572**
**Vote: 154 Uganda National Bureau of Standards (UNBS)**	23.856	12.767	0.000	**36.622**	6.651	0.000	0.000	**6.651**	**43.273**	**43.273**
01 General Administration and Support Services	23.856	7.489	0.000	**31.344**	0.000	0.000	0.000	**0.000**	**31.344**	**31.344**
02 Standards and Measurements enforcement	0.000	0.500	0.000	**0.500**	0.000	0.000	0.000	**0.000**	**0.500**	**0.500**
03 Standards development	0.000	2.350	0.000	**2.350**	0.000	0.000	0.000	**0.000**	**2.350**	**2.350**
04 Standards and Measurement Systems' promotion	0.000	2.428	0.000	**2.428**	6.651	0.000	0.000	**6.651**	**9.079**	**9.079**
**Vote: 155 Cotton Development Organization**	2.013	3.220	0.000	**5.233**	0.300	0.000	0.000	**0.300**	**5.533**	**5.533**
01 Cotton Development	2.013	3.220	0.000	**5.233**	0.300	0.000	0.000	**0.300**	**5.533**	**5.533**
**Vote: 156 Uganda Land Commission (ULC)**	1.062	1.220	0.000	**2.282**	25.600	0.000	0.000	**25.600**	**27.882**	**27.882**
01 General Administration and Support Services	1.062	0.870	0.000	**1.932**	25.600	0.000	0.000	**25.600**	**27.532**	**27.532**
02 Government Land Administration	0.000	0.350	0.000	**0.350**	0.000	0.000	0.000	**0.000**	**0.350**	**0.350**
**Vote: 157 National Forestry Authority (NFA)**	8.266	12.500	0.000	**20.766**	5.650	0.000	0.000	**5.650**	**26.416**	**26.416**
01 Forest Management	0.000	4.816	0.000	**4.816**	0.000	0.000	0.000	**0.000**	**4.816**	**4.816**
02 Institutional Development	8.266	7.684	0.000	**15.950**	5.650	0.000	0.000	**5.650**	**21.600**	**21.600**

ANNEX 3: SUB SUBPROGRAMME ALLOCATIONS FOR FY2023/24

	Recurrent				Development					
Billion Uganda Shillings	Wage	Non-Wage	AIA	Total	GoU Dev't	Ext. Fin	AIA	Total	Total Budget	Total Excl AIA
**Vote: 158 Internal Security Organization (ISO)**	52.461	85.797	0.000	**138.258**	10.680	0.000	0.000	**10.680**	**148.938**	**148.938**
01 Strengthening Internal security	52.461	85.797	0.000	**138.258**	10.680	0.000	0.000	**10.680**	**148.938**	**148.938**
**Vote: 159 External Security Organization (ESO)**	18.313	53.562	0.000	**71.876**	1.003	0.000	0.000	**1.003**	**72.879**	**72.879**
01 Strengthening External Security	18.313	53.562	0.000	**71.876**	1.003	0.000	0.000	**1.003**	**72.879**	**72.879**
**Vote: 160 Uganda Coffee Development Authority (UCDA)**	10.125	35.360	0.000	**45.485**	2.020	0.000	0.000	**2.020**	**47.505**	**47.505**
01 Coffee Development	10.125	35.360	0.000	**45.485**	2.020	0.000	0.000	**2.020**	**47.505**	**47.505**
**Vote: 161 Uganda Free Zones Authority**	2.393	3.778	0.000	**6.171**	5.409	0.000	0.000	**5.409**	**11.580**	**11.580**
01 Business Development and Investor Support	0.000	0.400	0.000	**0.400**	0.000	0.000	0.000	**0.000**	**0.400**	**0.400**
02 Legal and Board Affairs	0.000	0.630	0.000	**0.630**	0.000	0.000	0.000	**0.000**	**0.630**	**0.630**
03 General Administration and Support Services	2.393	2.748	0.000	**5.141**	5.409	0.000	0.000	**5.409**	**10.550**	**10.550**
**Vote: 162 Uganda Microfinance Regulatory Authority**	2.171	7.519	0.000	**9.690**	1.800	0.000	0.000	**1.800**	**11.490**	**11.490**
01 General Administration and Support Services	0.887	5.594	0.000	**6.481**	1.800	0.000	0.000	**1.800**	**8.281**	**8.281**
02 Supervision and Regulation	1.284	1.925	0.000	**3.209**	0.000	0.000	0.000	**0.000**	**3.209**	**3.209**
**Vote: 163 Uganda Retirement Benefits Regulatory Authority**	5.794	7.831	0.000	**13.625**	0.000	0.000	0.000	**0.000**	**13.625**	**13.625**
01 General Administration and Support Services	3.412	4.841	0.000	**8.253**	0.000	0.000	0.000	**0.000**	**8.253**	**8.253**
02 Regulation and Supervision	1.808	2.194	0.000	**4.002**	0.000	0.000	0.000	**0.000**	**4.002**	**4.002**
03 Research and Strategy	0.575	0.795	0.000	**1.370**	0.000	0.000	0.000	**0.000**	**1.370**	**1.370**
**Vote: 164 National Council for Higher Education**	5.240	9.565	0.000	**14.805**	0.000	0.000	0.000	**0.000**	**14.805**	**14.805**
01 Higher Education Quality, Standard and Accreditation	0.000	4.701	0.000	**4.701**	0.000	0.000	0.000	**0.000**	**4.701**	**4.701**
02 General Administration and support services	5.240	4.864	0.000	**10.104**	0.000	0.000	0.000	**0.000**	**10.104**	**10.104**
**Vote: 165 Uganda Business and Technical Examination Board**	6.125	21.362	0.000	**27.487**	3.300	0.000	0.000	**3.300**	**30.787**	**30.787**
01 Technical and Vocational Examination Assessment and Certification	6.125	21.362	0.000	**27.487**	3.300	0.000	0.000	**3.300**	**30.787**	**30.787**
**Vote: 166 National Council of Sports**	1.609	15.779	0.000	**17.387**	0.000	0.000	0.000	**0.000**	**17.387**	**17.387**
01 Delivery of Sports Services	0.000	12.143	0.000	**12.143**	0.000	0.000	0.000	**0.000**	**12.143**	**12.143**
02 General Administration and Support Services	1.609	3.636	0.000	**5.245**	0.000	0.000	0.000	**0.000**	**5.245**	**5.245**
**Vote: 167 Science, Technology and Innovation**	4.160	15.670	0.000	**19.830**	23.000	125.568	0.000	**148.568**	**168.398**	**168.398**
01 Industrial Value Chain	1.500	5.000	0.000	**6.500**	23.000	125.568	0.000	**148.568**	**155.068**	**155.068**
02 Support Centres	1.500	9.170	0.000	**10.670**	0.000	0.000	0.000	**0.000**	**10.670**	**10.670**
03 Support Services	1.160	1.500	0.000	**2.660**	0.000	0.000	0.000	**0.000**	**2.660**	**2.660**

ANNEX 3: SUB SUBPROGRAMME ALLOCATIONS FOR FY2023/24

	Recurrent				Development					
Billion Uganda Shillings	Wage	Non-Wage	AIA	Total	GoU Dev't	Ext. Fin	AIA	Total	Total Budget	Total Excl AIA
**Vote: 301 Makerere University**	208.970	138.483	0.000	**347.453**	11.033	0.000	0.000	**11.033**	**358.486**	**358.486**
01 Delivery of Tertiary Education	0.000	29.611	0.000	**29.611**	0.000	0.000	0.000	**0.000**	**29.611**	**29.611**
02 Support Services	208.970	108.872	0.000	**317.842**	11.033	0.000	0.000	**11.033**	**328.875**	**328.875**
**Vote: 302 Mbarara University**	40.006	14.743	0.000	**54.749**	3.764	0.000	0.000	**3.764**	**58.513**	**58.513**
01 Delivery of Tertiary Education	32.811	7.422	0.000	**40.233**	0.000	0.000	0.000	**0.000**	**40.233**	**40.233**
02 General Administration and Support Services	7.195	7.321	0.000	**14.516**	3.764	0.000	0.000	**3.764**	**18.280**	**18.280**
**Vote: 303 Makerere University Business School**	62.645	41.036	0.000	**103.681**	2.126	0.000	0.000	**2.126**	**105.806**	**105.806**
01 Delivery of Tertiary Education Programme	0.000	3.055	0.000	**3.055**	0.000	0.000	0.000	**0.000**	**3.055**	**3.055**
02 General Administration and support services	62.645	37.981	0.000	**100.626**	2.126	0.000	0.000	**2.126**	**102.751**	**102.751**
**Vote: 304 Kyambogo University**	61.172	74.048	0.000	**135.219**	1.690	0.000	0.000	**1.690**	**136.909**	**136.909**
01 Delivery of Tertiary Education	37.034	23.261	0.000	**60.295**	0.000	0.000	0.000	**0.000**	**60.295**	**60.295**
02 General Administration and support services	24.138	50.787	0.000	**74.925**	1.690	0.000	0.000	**1.690**	**76.615**	**76.615**
**Vote: 305 Busitema University**	33.657	14.434	0.000	**48.091**	6.384	0.000	0.000	**6.384**	**54.475**	**54.475**
01 Delivery of Tertiary Education Programme	24.066	4.781	0.000	**28.847**	0.000	0.000	0.000	**0.000**	**28.847**	**28.847**
02 General Administration and Support Services	9.592	9.652	0.000	**19.244**	6.384	0.000	0.000	**6.384**	**25.628**	**25.628**
**Vote: 306 Muni University**	15.694	5.485	0.000	**21.179**	4.752	0.000	0.000	**4.752**	**25.931**	**25.931**
01 Delivery of Tertiary Education	0.000	1.541	0.000	**1.541**	0.000	0.000	0.000	**0.000**	**1.541**	**1.541**
02 General Administration and Support Services	15.694	3.944	0.000	**19.638**	4.752	0.000	0.000	**4.752**	**24.390**	**24.390**
**Vote: 307 Kabale University**	38.386	14.971	0.000	**53.358**	2.587	0.000	0.000	**2.587**	**55.944**	**55.944**
01 Delivery of Tertiary Education	0.000	3.051	0.000	**3.051**	0.000	0.000	0.000	**0.000**	**3.051**	**3.051**
02 General Administration and Support Services	38.386	11.920	0.000	**50.307**	2.587	0.000	0.000	**2.587**	**52.893**	**52.893**
**Vote: 308 Soroti University**	15.651	7.085	0.000	**22.735**	1.254	0.000	0.000	**1.254**	**23.989**	**23.989**
01 Delivery of Tertiary Education Programme	8.654	2.779	0.000	**11.432**	0.000	0.000	0.000	**0.000**	**11.432**	**11.432**
02 General Administration and support services	6.997	4.306	0.000	**11.303**	1.254	0.000	0.000	**1.254**	**12.557**	**12.557**
**Vote: 309 Gulu University**	38.014	23.473	0.000	**61.487**	2.121	0.000	0.000	**2.121**	**63.608**	**63.608**
01 Delivery of Tertiary Education	29.892	6.799	0.000	**36.690**	0.000	0.000	0.000	**0.000**	**36.690**	**36.690**
02 General Administration and support services	8.122	16.675	0.000	**24.797**	2.121	0.000	0.000	**2.121**	**26.918**	**26.918**
**Vote: 310 Lira University**	15.553	6.748	0.000	**22.301**	5.000	0.000	0.000	**5.000**	**27.301**	**27.301**
01 Delivery of Tertiary Education	10.258	1.126	0.000	**11.384**	0.000	0.000	0.000	**0.000**	**11.384**	**11.384**
02 General Administration and Support Services	5.295	5.622	0.000	**10.917**	5.000	0.000	0.000	**5.000**	**15.917**	**15.917**
**Vote: 311 Law Development Centre**	8.443	16.045	0.000	**24.487**	5.450	0.000	0.000	**5.450**	**29.937**	**29.937**
01 Legal Training	8.443	16.045	0.000	**24.487**	5.450	0.000	0.000	**5.450**	**29.937**	**29.937**

ANNEX 3: SUB SUBPROGRAMME ALLOCATIONS FOR FY2023/24

	Recurrent				Development					
Billion Uganda Shillings	Wage	Non-Wage	AIA	Total	GoU Dev't	Ext. Fin	AIA	Total	Total Budget	Total Excl AIA
**Vote: 312 Uganda Management Institute**	18.754	16.276	0.000	**35.030**	1.320	0.000	0.000	**1.320**	**36.350**	**36.350**
01 Delivery of Tertiary Education	0.000	3.228	0.000	**3.228**	0.000	0.000	0.000	**0.000**	**3.228**	**3.228**
02 General Administration and support services	18.754	13.048	0.000	**31.802**	1.320	0.000	0.000	**1.320**	**33.122**	**33.122**
**Vote: 313 Mountains of the Moon University**	21.990	10.245	0.000	**32.235**	0.000	0.000	0.000	**0.000**	**32.235**	**32.235**
01 Delivery of Tertiary Education Programme	14.477	3.546	0.000	**18.023**	0.000	0.000	0.000	**0.000**	**18.023**	**18.023**
02 Support Services Programme	7.513	6.699	0.000	**14.212**	0.000	0.000	0.000	**0.000**	**14.212**	**14.212**
**Vote: 401 Mulago National Referral Hospital**	45.779	42.365	0.000	**88.144**	5.260	0.000	0.000	**5.260**	**93.404**	**93.404**
01 National Referral Hospital Services	45.779	42.365	0.000	**88.144**	5.260	0.000	0.000	**5.260**	**93.404**	**93.404**
**Vote: 402 Butabika Hospital**	9.071	9.483	0.000	**18.554**	2.513	0.000	0.000	**2.513**	**21.068**	**21.068**
01 Provision of Specialised Mental Health Services	9.071	9.483	0.000	**18.554**	2.513	0.000	0.000	**2.513**	**21.068**	**21.068**
**Vote: 403 Arua Hospital**	7.799	2.965	0.000	**10.764**	0.120	0.000	0.000	**0.120**	**10.884**	**10.884**
01 Regional Referral Hospital Services	7.799	2.965	0.000	**10.764**	0.120	0.000	0.000	**0.120**	**10.884**	**10.884**
**Vote: 404 Fort Portal Hospital**	8.993	2.646	0.000	**11.638**	0.120	0.000	0.000	**0.120**	**11.758**	**11.758**
01 Regional Referral Hospital Services	8.993	2.646	0.000	**11.638**	0.120	0.000	0.000	**0.120**	**11.758**	**11.758**
**Vote: 405 Gulu Hospital**	8.110	6.491	0.000	**14.602**	0.120	0.000	0.000	**0.120**	**14.722**	**14.722**
01 Regional Referral Hospital Services	8.110	6.491	0.000	**14.602**	0.120	0.000	0.000	**0.120**	**14.722**	**14.722**
**Vote: 406 Hoima Hospital**	8.638	1.971	0.000	**10.609**	0.120	0.000	0.000	**0.120**	**10.729**	**10.729**
01 Regional Referral Hospital Services	8.638	1.971	0.000	**10.609**	0.120	0.000	0.000	**0.120**	**10.729**	**10.729**
**Vote: 407 Jinja Hospital**	12.585	8.069	0.000	**20.654**	0.120	0.000	0.000	**0.120**	**20.774**	**20.774**
01 Regional Referral Hospital Services	12.585	8.069	0.000	**20.654**	0.120	0.000	0.000	**0.120**	**20.774**	**20.774**
**Vote: 408 Kabale Hospital**	6.316	5.247	0.000	**11.563**	0.120	0.000	0.000	**0.120**	**11.683**	**11.683**
01 Regional Referral Hospital Services	6.316	5.247	0.000	**11.563**	0.120	0.000	0.000	**0.120**	**11.683**	**11.683**
**Vote: 409 Masaka Hospital**	8.206	2.665	0.000	**10.871**	0.120	0.000	0.000	**0.120**	**10.991**	**10.991**
01 Regional Referral Hospital Services	8.206	2.665	0.000	**10.871**	0.120	0.000	0.000	**0.120**	**10.991**	**10.991**
**Vote: 410 Mbale Hospital**	9.351	7.632	0.000	**16.983**	0.120	0.000	0.000	**0.120**	**17.103**	**17.103**
01 Regional Referral Hospital Services	9.351	7.632	0.000	**16.983**	0.120	0.000	0.000	**0.120**	**17.103**	**17.103**
**Vote: 411 Soroti Hospital**	7.785	2.825	0.000	**10.609**	0.120	0.000	0.000	**0.120**	**10.729**	**10.729**
01 Regional Referral Hospital Services	7.785	2.825	0.000	**10.609**	0.120	0.000	0.000	**0.120**	**10.729**	**10.729**
**Vote: 412 Lira Hospital**	8.352	8.742	0.000	**17.095**	0.120	0.000	0.000	**0.120**	**17.215**	**17.215**
01 Regional Referral Hospital Services	8.352	8.742	0.000	**17.095**	0.120	0.000	0.000	**0.120**	**17.215**	**17.215**
**Vote: 413 Mbarara Regional Hospital**	8.848	10.024	0.000	**18.871**	0.120	0.000	0.000	**0.120**	**18.991**	**18.991**
01 Regional Referral Hospital Services	8.848	10.024	0.000	**18.871**	0.120	0.000	0.000	**0.120**	**18.991**	**18.991**

ANNEX 3: SUB SUBPROGRAMME ALLOCATIONS FOR FY2023/24

	Recurrent				Development					
Billion Uganda Shillings	Wage	Non-Wage	AIA	Total	GoU Dev't	Ext. Fin	AIA	Total	Total Budget	Total Excl AIA
**Vote: 414 Mubende Regional Referral Hospital**	7.862	1.568	0.000	**9.430**	0.150	0.000	0.000	**0.150**	**9.580**	**9.580**
01 Regional Referral Hospital Services	7.862	1.568	0.000	**9.430**	0.150	0.000	0.000	**0.150**	**9.580**	**9.580**
**Vote: 415 Moroto Regional Referral Hospital**	7.145	3.635	0.000	**10.780**	0.120	0.000	0.000	**0.120**	**10.900**	**10.900**
01 Regional Referral Hospital Services	7.145	3.635	0.000	**10.780**	0.120	0.000	0.000	**0.120**	**10.900**	**10.900**
**Vote: 416 Naguru National Referral Hospital**	10.093	1.443	0.000	**11.537**	0.240	0.000	0.000	**0.240**	**11.777**	**11.777**
01 Regional Referral Hospital Services	10.093	1.443	0.000	**11.537**	0.240	0.000	0.000	**0.240**	**11.777**	**11.777**
**Vote: 417 Kiruddu National Referral Hospital**	10.176	14.688	0.000	**24.864**	1.530	0.000	0.000	**1.530**	**26.394**	**26.394**
01 Regional Referral Hospital Services	10.176	14.688	0.000	**24.864**	1.530	0.000	0.000	**1.530**	**26.394**	**26.394**
**Vote: 418 Kawempe National Referral Hospital**	9.407	6.288	0.000	**15.695**	0.900	0.000	0.000	**0.900**	**16.595**	**16.595**
01 Regional Referral Hospital Services	9.407	6.288	0.000	**15.695**	0.900	0.000	0.000	**0.900**	**16.595**	**16.595**
**Vote: 419 Entebbe Regional Referral Hospital**	4.614	2.254	0.000	**6.868**	0.900	0.000	0.000	**0.900**	**7.768**	**7.768**
01 Regional Referral Hospital Services	4.614	2.254	0.000	**6.868**	0.900	0.000	0.000	**0.900**	**7.768**	**7.768**
**Vote: 420 Mulago Specialized Women and Neonatal Hospital**	11.107	13.393	0.000	**24.500**	2.268	0.000	0.000	**2.268**	**26.768**	**26.768**
01 Mulago Specialized Women and Neonatal Hospital Services	11.107	13.393	0.000	**24.500**	2.268	0.000	0.000	**2.268**	**26.768**	**26.768**
**Vote: 421 Kayunga Referral Hospital**	3.724	7.437	0.000	**11.161**	0.000	0.000	0.000	**0.000**	**11.161**	**11.161**
01 Regional Referral Hospital Services	3.724	7.437	0.000	**11.161**	0.000	0.000	0.000	**0.000**	**11.161**	**11.161**
**Vote: 422 Yumbe Referral Hospital**	4.182	6.065	0.000	**10.247**	0.000	0.000	0.000	**0.000**	**10.247**	**10.247**
01 Regional Referral Hospital Services	4.182	6.065	0.000	**10.247**	0.000	0.000	0.000	**0.000**	**10.247**	**10.247**
**Vote: 501 Uganda Mission at the United Nations, New York**	1.951	14.755	0.000	**16.707**	1.053	0.000	0.000	**1.053**	**17.760**	**17.760**
01 Overseas Mission Services	1.951	14.755	0.000	**16.707**	1.053	0.000	0.000	**1.053**	**17.760**	**17.760**
**Vote: 502 Uganda High Commission in the United Kingdom**	1.397	3.890	0.000	**5.288**	2.114	0.000	0.000	**2.114**	**7.401**	**7.401**
01 Overseas Mission Services	1.397	3.890	0.000	**5.288**	2.114	0.000	0.000	**2.114**	**7.401**	**7.401**
**Vote: 503 Uganda High Commission in Canada, Ottawa**	1.175	4.048	0.000	**5.224**	8.500	0.000	0.000	**8.500**	**13.724**	**13.724**
01 Overseas Mission Services	1.175	4.048	0.000	**5.224**	8.500	0.000	0.000	**8.500**	**13.724**	**13.724**
**Vote: 504 Uganda High Commission in India, New Delhi**	0.306	3.277	0.000	**3.583**	0.270	0.000	0.000	**0.270**	**3.853**	**3.853**
01 Overseas Mission Services	0.306	3.277	0.000	**3.583**	0.270	0.000	0.000	**0.270**	**3.853**	**3.853**
**Vote: 505 Uganda High Commission in Kenya, Nairobi**	0.339	3.442	0.000	**3.781**	6.700	0.000	0.000	**6.700**	**10.481**	**10.481**
01 Overseas Mission Services	0.339	3.442	0.000	**3.781**	6.700	0.000	0.000	**6.700**	**10.481**	**10.481**

ANNEX 3: SUB SUBPROGRAMME ALLOCATIONS FOR FY2023/24

	Recurrent				Development					
Billion Uganda Shillings	Wage	Non-Wage	AIA	Total	GoU Dev't	Ext. Fin	AIA	Total	Total Budget	Total Excl AIA
**Vote: 506 Uganda High Commission in Tanzania, Dar es Salaam**	0.603	5.452	0.000	**6.055**	3.928	0.000	0.000	**3.928**	**9.983**	9.983
01 Overseas Mission Services	0.603	5.452	0.000	**6.055**	3.928	0.000	0.000	**3.928**	**9.983**	9.983
**Vote: 507 Uganda High Commission in Nigeria, Abuja**	0.386	2.336	0.000	**2.722**	16.200	0.000	0.000	**16.200**	**18.922**	18.922
01 Overseas Mission Services	0.386	2.336	0.000	**2.722**	16.200	0.000	0.000	**16.200**	**18.922**	18.922
**Vote: 508 Uganda High Commission in South Africa, Pretoria**	0.440	2.638	0.000	**3.079**	0.390	0.000	0.000	**0.390**	**3.469**	3.469
01 Overseas Mission Services	0.440	2.638	0.000	**3.079**	0.390	0.000	0.000	**0.390**	**3.469**	3.469
**Vote: 509 Uganda High Commission in Rwanda, Kigali**	0.529	2.232	0.000	**2.761**	0.600	0.000	0.000	**0.600**	**3.361**	3.361
01 Overseas Mission Services	0.529	2.232	0.000	**2.761**	0.600	0.000	0.000	**0.600**	**3.361**	3.361
**Vote: 510 Uganda Embassy in the United States, Washington**	1.362	7.130	0.000	**8.492**	0.540	0.000	0.000	**0.540**	**9.032**	9.032
01 Overseas Mission Services	1.362	7.130	0.000	**8.492**	0.540	0.000	0.000	**0.540**	**9.032**	9.032
**Vote: 511 Uganda Embassy in Egypt, Cairo**	0.544	2.407	0.000	**2.951**	0.170	0.000	0.000	**0.170**	**3.121**	3.121
01 Overseas Mission Services	0.544	2.407	0.000	**2.951**	0.170	0.000	0.000	**0.170**	**3.121**	3.121
**Vote: 512 Uganda Embassy in Ethiopia, Addis Ababa**	0.508	2.399	0.000	**2.907**	0.150	0.000	0.000	**0.150**	**3.057**	3.057
01 Overseas Mission Services	0.508	2.399	0.000	**2.907**	0.150	0.000	0.000	**0.150**	**3.057**	3.057
**Vote: 513 Uganda Embassy in China, Beijing**	0.388	3.859	0.000	**4.247**	0.100	0.000	0.000	**0.100**	**4.347**	4.347
01 Overseas Mission Services	0.388	3.859	0.000	**4.247**	0.100	0.000	0.000	**0.100**	**4.347**	4.347
**Vote: 514 Uganda Embassy in Switzerland, Geneva**	1.631	5.213	0.000	**6.844**	0.100	0.000	0.000	**0.100**	**6.944**	6.944
01 Overseas Mission Services	1.631	5.213	0.000	**6.844**	0.100	0.000	0.000	**0.100**	**6.944**	6.944
**Vote: 515 Uganda Embassy in Japan, Tokyo**	1.510	3.834	0.000	**5.344**	0.000	0.000	0.000	**0.000**	**5.344**	5.344
01 Overseas Mission Services	1.510	3.834	0.000	**5.344**	0.000	0.000	0.000	**0.000**	**5.344**	5.344
**Vote: 516 Uganda Embassy in Saudi Arabia, Riyadh**	0.999	4.962	0.000	**5.962**	0.000	0.000	0.000	**0.000**	**5.962**	5.962
01 Overseas Mission Services	0.999	4.962	0.000	**5.962**	0.000	0.000	0.000	**0.000**	**5.962**	5.962
**Vote: 517 Uganda Embassy in Denmark, Copenhagen**	0.763	5.046	0.000	**5.809**	0.100	0.000	0.000	**0.100**	**5.909**	5.909
01 Overseas Mission Services	0.763	5.046	0.000	**5.809**	0.100	0.000	0.000	**0.100**	**5.909**	5.909
**Vote: 518 Uganda Embassy in Belgium, Brussels**	1.099	3.070	0.000	**4.169**	0.380	0.000	0.000	**0.380**	**4.549**	4.549
01 Overseas Mission Services	1.099	3.070	0.000	**4.169**	0.380	0.000	0.000	**0.380**	**4.549**	4.549
**Vote: 519 Uganda Embassy in Italy, Rome**	0.848	3.704	0.000	**4.552**	0.000	0.000	0.000	**0.000**	**4.552**	4.552
01 Overseas Mission Services	0.848	3.704	0.000	**4.552**	0.000	0.000	0.000	**0.000**	**4.552**	4.552

ANNEX 3: SUB SUBPROGRAMME ALLOCATIONS FOR FY2023/24

	Recurrent				Development					
Billion Uganda Shillings	Wage	Non-Wage	AIA	Total	GoU Dev't	Ext. Fin	AIA	Total	Total Budget	Total Excl AIA
**Vote: 520 Uganda Embassy in DRC, Kinshasa**	0.658	3.458	0.000	4.116	2.074	0.000	0.000	2.074	6.190	6.190
01 Overseas Mission Services	0.658	3.458	0.000	4.116	2.074	0.000	0.000	2.074	6.190	6.190
**Vote: 521 Uganda Embassy in Sudan, Khartoum**	0.609	3.189	0.000	3.798	0.560	0.000	0.000	0.560	4.358	4.358
01 Overseas Mission Services	0.609	3.189	0.000	3.798	0.560	0.000	0.000	0.560	4.358	4.358
**Vote: 522 Uganda Embassy in France, Paris**	0.951	5.506	0.000	6.458	0.000	0.000	0.000	0.000	6.458	6.458
01 Overseas Mission Services	0.951	5.506	0.000	6.458	0.000	0.000	0.000	0.000	6.458	6.458
**Vote: 523 Uganda Embassy in Germany, Berlin**	1.132	5.606	0.000	6.739	0.000	0.000	0.000	0.000	6.739	6.739
01 Overseas Mission Services	1.132	5.606	0.000	6.739	0.000	0.000	0.000	0.000	6.739	6.739
**Vote: 524 Uganda Embassy in Iran, Tehran**	0.756	2.278	0.000	3.034	0.000	0.000	0.000	0.000	3.034	3.034
01 Overseas Mission Services	0.756	2.278	0.000	3.034	0.000	0.000	0.000	0.000	3.034	3.034
**Vote: 525 Uganda Embassy in Russia, Moscow**	0.610	3.139	0.000	3.749	0.172	0.000	0.000	0.172	3.921	3.921
01 Overseas Mission Services	0.610	3.139	0.000	3.749	0.172	0.000	0.000	0.172	3.921	3.921
**Vote: 526 Uganda Embassy in Australia, Canberra**	0.989	2.974	0.000	3.963	0.150	0.000	0.000	0.150	4.113	4.113
01 Overseas Mission Services	0.989	2.974	0.000	3.963	0.150	0.000	0.000	0.150	4.113	4.113
**Vote: 527 Uganda Embassy in South Sudan, Juba**	0.423	3.546	0.000	3.969	0.000	0.000	0.000	0.000	3.969	3.969
01 Overseas Mission Services	0.423	3.546	0.000	3.969	0.000	0.000	0.000	0.000	3.969	3.969
**Vote: 528 Uganda Embassy in United Arab Emirates, Abudhabi**	0.765	5.150	0.000	5.915	0.350	0.000	0.000	0.350	6.265	6.265
01 Overseas Mission Services	0.765	5.150	0.000	5.915	0.350	0.000	0.000	0.350	6.265	6.265
**Vote: 529 Uganda Embassy in Burundi, Bujumbura**	0.556	2.275	0.000	2.832	0.150	0.000	0.000	0.150	2.982	2.982
01 Overseas Mission Services	0.556	2.275	0.000	2.832	0.150	0.000	0.000	0.150	2.982	2.982
**Vote: 530 Uganda Consulate in China, Guangzhou**	0.419	3.281	0.000	3.700	6.200	0.000	0.000	6.200	9.900	9.900
01 Overseas Mission Services	0.419	3.281	0.000	3.700	6.200	0.000	0.000	6.200	9.900	9.900
**Vote: 531 Uganda Embassy in Turkey, Ankara**	0.695	4.083	0.000	4.778	0.390	0.000	0.000	0.390	5.168	5.168
01 Overseas Mission Services	0.695	4.083	0.000	4.778	0.390	0.000	0.000	0.390	5.168	5.168
**Vote: 532 Uganda Embassy in Somalia, Mogadishu**	0.134	2.559	0.000	2.693	1.600	0.000	0.000	1.600	4.293	4.293
01 Overseas Mission Services	0.134	2.559	0.000	2.693	1.600	0.000	0.000	1.600	4.293	4.293
**Vote: 533 Uganda Embassy in Malaysia, Kuala Lumpur**	0.580	2.698	0.000	3.278	0.100	0.000	0.000	0.100	3.378	3.378
01 Overseas Mission Services	0.580	2.698	0.000	3.278	0.100	0.000	0.000	0.100	3.378	3.378
**Vote: 534 Uganda Consulate in Kenya, Mombasa**	0.747	3.960	0.000	4.706	0.000	0.000	0.000	0.000	4.706	4.706
01 Overseas Mission Services	0.747	3.960	0.000	4.706	0.000	0.000	0.000	0.000	4.706	4.706

ANNEX 3: SUB SUBPROGRAMME ALLOCATIONS FOR FY2023/24

	Recurrent				Development					
Billion Uganda Shillings	Wage	Non-Wage	AIA	Total	GoU Dev't	Ext. Fin	AIA	Total	Total Budget	Total Excl AIA
**Vote: 535 Uganda Embassy in Algeria, Algiers**	0.915	2.972	0.000	**3.887**	0.000	0.000	0.000	**0.000**	**3.887**	**3.887**
01 Overseas Mission Services	0.915	2.972	0.000	**3.887**	0.000	0.000	0.000	**0.000**	**3.887**	**3.887**
**Vote: 536 Uganda Embassy in Qatar, Doha**	0.541	2.207	0.000	**2.749**	0.198	0.000	0.000	**0.198**	**2.947**	**2.947**
01 Overseas Mission Services	0.541	2.207	0.000	**2.749**	0.198	0.000	0.000	**0.198**	**2.947**	**2.947**
**Vote: 537 Uganda Mission in Havana, Cuba**	0.326	2.674	0.000	**3.000**	0.000	0.000	0.000	**0.000**	**3.000**	**3.000**
01 Overseas Mission Services	0.326	2.674	0.000	**3.000**	0.000	0.000	0.000	**0.000**	**3.000**	**3.000**
**Vote: 538 Uganda Mission in Luanda, Angola**	0.585	2.415	0.000	**3.000**	0.000	0.000	0.000	**0.000**	**3.000**	**3.000**
01 Overseas Mission Services	0.585	2.415	0.000	**3.000**	0.000	0.000	0.000	**0.000**	**3.000**	**3.000**
**Vote: 601 Local Governments 01**	120.729	0.000	0.000	**120.729**	0.000	0.000	0.000	**0.000**	**120.729**	**120.729**
01 District Production Services	120.729	0.000	0.000	**120.729**	0.000	0.000	0.000	**0.000**	**120.729**	**120.729**
**Vote: 606 Local Governments 06**	0.000	3.500	0.000	**3.500**	0.000	0.000	0.000	**0.000**	**3.500**	**3.500**
02 District Natural Resources	0.000	3.500	0.000	**3.500**	0.000	0.000	0.000	**0.000**	**3.500**	**3.500**
**Vote: 607 Local Governments 07**	0.000	2.232	0.000	**2.232**	0.000	0.000	0.000	**0.000**	**2.232**	**2.232**
03 District Commercial Services	0.000	2.232	0.000	**2.232**	0.000	0.000	0.000	**0.000**	**2.232**	**2.232**
**Vote: 609 Local Governments 09**	0.000	0.000	0.000	**0.000**	27.653	0.000	0.000	**27.653**	**27.653**	**27.653**
04 District , Urban and Community Access Roads	0.000	0.000	0.000	**0.000**	27.653	0.000	0.000	**27.653**	**27.653**	**27.653**
**Vote: 610 Local Governments 10**	0.000	0.000	0.000	**0.000**	0.000	29.202	0.000	**29.202**	**29.202**	**29.202**
10 Physical Planning and Urban Development	0.000	0.000	0.000	**0.000**	0.000	29.202	0.000	**29.202**	**29.202**	**29.202**
**Vote: 612 Local Governments 12**	2,888.816	430.010	0.000	**3,318.826**	488.868	0.000	0.000	**488.868**	**3,807.694**	**3,807.694**
02 District Natural Resources	0.000	0.000	0.000	**0.000**	91.500	0.000	0.000	**91.500**	**91.500**	**91.500**
05 Education and Sports	2,140.477	335.133	0.000	**2,475.609**	252.368	0.000	0.000	**252.368**	**2,727.977**	**2,727.977**
06 Primary Health Care	748.339	94.878	0.000	**843.217**	145.000	0.000	0.000	**145.000**	**988.217**	**988.217**
**Vote: 617 Local Governments 17**	422.764	128.000	238.000	**788.764**	0.000	0.000	0.000	**0.000**	**788.764**	**550.764**
09 District and Urban Administration	422.764	128.000	238.000	**788.764**	0.000	0.000	0.000	**0.000**	**788.764**	**550.764**
**Grand Total**	6,959.070	27,210.194	238.000	**34,407.264**	5,833.979	8,043.573	0.000	**13,877.552**	**48,284.816**	**48,046.816**

ANNEX 4: APPROVED BUDGET AND QUARTER ONE OUTTURNS FOR FY2022/23 BY SUB SUBPROGRAMME (Excl. Arrears and AIA)

	Approved Estimates						Outturn by End September						Performance
Billion Uganda Shillings	Wage	Non-Wage	Gou Dev	Ext. Fin	GoU Total	Total GoU + Ext. Fin	Wage	NonWage	GoU Dev	Ext. Fin	Total GoU	Total GoU + Ext. Fin	%GoU Budget Spent
**Vote: 001 Office of the President**	25.588	159.273	34.308	0.000	**219.168**	**219.168**	4.926	25.963	0.000	0.000	**30.889**	**30.889**	**14.09%**
01 Cabinet Support and Policy Development	0.000	3.871	0.000	0.000	**3.871**	**3.871**	0.000	0.760	0.000	0.000	**0.760**	**0.760**	**19.62%**
02 Policy, planning and support services	25.307	30.470	34.308	0.000	**90.085**	**90.085**	4.926	5.012	0.000	0.000	**9.938**	**9.938**	**11.03%**
03 Government Mobilisation,Monitoring and people centred security	0.000	50.101	0.000	0.000	**50.101**	**50.101**	0.000	5.726	0.000	0.000	**5.726**	**5.726**	**11.43%**
04 Security Administration	0.000	24.741	0.000	0.000	**24.741**	**24.741**	0.000	6.670	0.000	0.000	**6.670**	**6.670**	**26.96%**
05 Effective Security Management	0.000	26.713	0.000	0.000	**26.713**	**26.713**	0.000	4.493	0.000	0.000	**4.493**	**4.493**	**16.82%**
06 Civic Education and Patriotism Services	0.000	7.461	0.000	0.000	**7.461**	**7.461**	0.000	1.774	0.000	0.000	**1.774**	**1.774**	**23.78%**
07 Government Mobilisation, Monitoring and Awards	0.000	1.639	0.000	0.000	**1.639**	**1.639**	0.000	0.115	0.000	0.000	**0.115**	**0.115**	**7.00%**
08 Socio-Economic Monitoring and Research	0.281	10.260	0.000	0.000	**10.541**	**10.541**	0.000	1.058	0.000	0.000	**1.058**	**1.058**	**10.04%**
09 Manifesto Monitoring and Evaluation	0.000	4.017	0.000	0.000	**4.017**	**4.017**	0.000	0.355	0.000	0.000	**0.355**	**0.355**	**8.84%**
**Vote: 002 State House**	26.796	436.710	174.351	44.250	**637.856**	**682.107**	5.406	118.545	31.412	0.000	**155.362**	**155.362**	**24.36%**
01 Logistical and Administrative Support to the Presidency	7.755	164.648	0.000	0.000	**172.404**	**172.404**	1.675	38.278	0.000	0.000	**39.952**	**39.952**	**23.17%**
02 Policy, Planning and Support Services	12.495	141.135	36.203	0.000	**189.833**	**189.833**	3.119	53.736	0.000	0.000	**56.855**	**56.855**	**29.95%**
03 Presidential Initiatives	2.386	69.795	0.000	0.000	**72.182**	**72.182**	0.557	11.873	0.000	0.000	**12.431**	**12.431**	**17.22%**
04 STI Support Services	4.159	61.131	138.148	44.250	**203.438**	**247.688**	0.055	14.658	31.412	0.000	**46.124**	**46.124**	**22.67%**
**Vote: 003 Office of the Prime Minister**	4.110	105.008	23.990	90.123	**133.108**	**223.230**	0.760	8.782	0.270	0.000	**9.812**	**9.812**	**7.37%**
01 Administration and Support Services	1.356	15.477	3.776	0.000	**20.608**	**20.608**	0.306	3.191	0.200	0.000	**3.696**	**3.696**	**17.94%**
02 Affirmative Action Programs	0.347	50.433	8.837	12.685	**59.617**	**72.302**	0.060	1.406	0.070	0.000	**1.536**	**1.536**	**2.58%**
03 Disaster Preparedness and Refugee Management	0.551	10.160	11.377	77.438	**22.088**	**99.525**	0.108	0.428	0.000	0.000	**0.535**	**0.535**	**2.42%**
04 Executive Governance	1.190	20.888	0.000	0.000	**22.078**	**22.078**	0.220	2.963	0.000	0.000	**3.183**	**3.183**	**14.42%**
05 Monitoring and Evaluation	0.362	5.429	0.000	0.000	**5.791**	**5.791**	0.042	0.524	0.000	0.000	**0.566**	**0.566**	**9.78%**
06 Strategic Coordination and Implementation	0.305	2.621	0.000	0.000	**2.926**	**2.926**	0.024	0.270	0.000	0.000	**0.294**	**0.294**	**10.05%**
**Vote: 004 Ministry of Defence**	617.579	765.262	1,982.917	393.015	**3,365.758**	**3,758.773**	267.649	197.707	469.637	0.000	**934.993**	**934.993**	**27.78%**
01 National Defence (UPDF)	615.735	607.789	1,980.826	393.015	**3,204.351**	**3,597.365**	267.211	129.303	469.637	0.000	**866.152**	**866.152**	**27.03%**
02 Policy, Planning and Support Services	1.844	157.473	2.090	0.000	**161.408**	**161.408**	0.438	68.404	0.000	0.000	**68.842**	**68.842**	**42.65%**
**Vote: 005 Ministry of Public Service**	4.361	22.331	2.948	0.000	**29.640**	**29.640**	0.811	1.651	0.000	0.000	**2.461**	**2.461**	**8.30%**
01 Human Resource Management	0.850	5.231	0.000	0.000	**6.081**	**6.081**	0.185	0.115	0.000	0.000	**0.300**	**0.300**	**4.93%**
02 Inspection and Quality Assurance	0.374	0.808	0.000	0.000	**1.182**	**1.182**	0.080	0.046	0.000	0.000	**0.126**	**0.126**	**10.68%**
03 Management Services	0.298	4.179	0.000	0.000	**4.478**	**4.478**	0.063	0.188	0.000	0.000	**0.250**	**0.250**	**5.59%**

ANNEX 4: APPROVED BUDGET AND QUARTER ONE OUTTURNS FOR FY2022/23 BY SUB SUBPROGRAMME (Excl. Arrears and AIA)

	Approved Estimates						Outturn by End September						Performance
Billion Uganda Shillings	Wage	Non-Wage	Gou Dev	Ext. Fin	GoU Total	Total GoU + Ext. Fin	Wage	NonWage	GoU Dev	Ext. Fin	Total GoU	Total GoU + Ext. Fin	%GoU Budget Spent
**Vote: 005 Ministry of Public Service**	4.361	22.331	2.948	0.000	**29.640**	**29.640**	0.811	1.651	0.000	0.000	**2.461**	**2.461**	**8.30%**
04 Policy, Planning and Support Services	2.839	12.113	2.948	0.000	**17.900**	**17.900**	0.484	1.301	0.000	0.000	**1.785**	**1.785**	**9.97%**
**Vote: 006 Ministry of Foreign Affairs**	6.009	29.080	0.428	0.000	**35.517**	**35.517**	1.161	1.141	0.000	0.000	**2.302**	**2.302**	**6.48%**
01 Policy, Planning and Support Services	6.009	25.680	0.428	0.000	**32.118**	**32.118**	1.161	1.141	0.000	0.000	**2.302**	**2.302**	**7.17%**
02 Protocol and Public Diplomacy	0.000	0.995	0.000	0.000	**0.995**	**0.995**	0.000	0.000	0.000	0.000	**0.000**	**0.000**	**0.00%**
03 Regional and International Economic Affairs	0.000	0.772	0.000	0.000	**0.772**	**0.772**	0.000	0.000	0.000	0.000	**0.000**	**0.000**	**0.00%**
04 Regional and International Political Affairs	0.000	1.634	0.000	0.000	**1.634**	**1.634**	0.000	0.000	0.000	0.000	**0.000**	**0.000**	**0.00%**
**Vote: 007 Ministry of Justice and Constitutional Affairs**	10.865	116.025	21.740	0.000	**148.630**	**148.630**	2.025	6.415	0.000	0.000	**8.440**	**8.440**	**5.68%**
01 Administration of Estates/Property of the Deceased	1.494	0.785	0.000	0.000	**2.279**	**2.279**	0.225	0.067	0.000	0.000	**0.293**	**0.293**	**12.84%**
02 Civil Litigation	1.894	1.343	0.000	0.000	**3.237**	**3.237**	0.325	0.125	0.000	0.000	**0.450**	**0.450**	**13.91%**
03 Legal Advisory and Consultancy Services	1.571	0.421	0.000	0.000	**1.992**	**1.992**	0.303	0.038	0.000	0.000	**0.341**	**0.341**	**17.10%**
04 First Parliamentary Counsel	1.186	2.302	0.000	0.000	**3.488**	**3.488**	0.233	0.072	0.000	0.000	**0.305**	**0.305**	**8.74%**
05 Policy, Planning and Support Services	3.986	110.353	21.740	0.000	**136.079**	**136.079**	0.815	6.082	0.000	0.000	**6.897**	**6.897**	**5.07%**
06 Regulation of the Legal Profession	0.734	0.821	0.000	0.000	**1.555**	**1.555**	0.123	0.031	0.000	0.000	**0.154**	**0.154**	**9.91%**
**Vote: 008 Ministry of Finance, Planning and Economic Development**	9.296	2,310.558	187.644	99.317	**2,507.497**	**2,606.814**	1.752	205.069	10.047	0.000	**216.868**	**216.868**	**8.65%**
01 Budget Preparation, Execution and Monitoring	1.208	34.918	100.036	5.834	**136.161**	**141.995**	0.234	7.473	2.472	0.000	**10.179**	**10.179**	**7.48%**
02 Deficit Financing and Cash Management	0.711	7.348	0.358	2.573	**8.417**	**10.991**	0.124	0.823	0.000	0.000	**0.947**	**0.947**	**11.25%**
03 Development Policy and Investment Promotion	0.226	26.285	2.249	66.928	**28.760**	**95.688**	0.039	8.231	0.937	0.000	**9.207**	**9.207**	**32.01%**
04 Financial Sector Development	0.301	1,410.852	4.852	4.424	**1,416.004**	**1,420.428**	0.056	79.484	0.695	0.000	**80.235**	**80.235**	**5.67%**
05 Internal Oversight and Advisory Services	0.441	7.806	0.000	0.000	**8.247**	**8.247**	0.058	1.014	0.000	0.000	**1.071**	**1.071**	**12.99%**
06 Macroeconomic Policy and Management	0.557	17.907	0.755	1.294	**19.219**	**20.513**	0.139	3.254	0.167	0.000	**3.560**	**3.560**	**18.52%**
07 Policy, Planning and Support Services	2.859	35.440	59.335	0.747	**97.633**	**98.381**	0.484	5.361	2.798	0.000	**8.643**	**8.643**	**8.85%**
08 Public Financial Management	2.994	770.003	20.058	17.516	**793.055**	**810.571**	0.618	99.430	2.978	0.000	**103.026**	**103.026**	**12.99%**
**Vote: 009 Ministry of Internal Affairs**	2.448	54.447	3.647	0.000	**60.543**	**60.543**	0.360	10.380	0.000	0.000	**10.741**	**10.741**	**17.74%**
01 Combat Trafficking in Persons	0.000	0.317	0.000	0.000	**0.317**	**0.317**	0.000	0.050	0.000	0.000	**0.050**	**0.050**	**15.80%**
02 Directorate of Community Service	0.000	3.598	0.000	0.000	**3.598**	**3.598**	0.000	0.602	0.000	0.000	**0.602**	**0.602**	**16.73%**
03 Internal Security, Coordination and Advisory Services	0.000	16.096	0.000	0.000	**16.096**	**16.096**	0.000	3.579	0.000	0.000	**3.579**	**3.579**	**22.23%**
04 Policy, Planning and Support Services	2.448	25.701	3.647	0.000	**31.797**	**31.797**	0.360	5.299	0.000	0.000	**5.659**	**5.659**	**17.80%**
06 NGO Regulation	0.000	4.400	0.000	0.000	**4.400**	**4.400**	0.000	0.468	0.000	0.000	**0.468**	**0.468**	**10.63%**

ANNEX 4: APPROVED BUDGET AND QUARTER ONE OUTTURNS FOR FY2022/23 BY SUB SUBPROGRAMME (Excl. Arrears and AIA)

	Approved Estimates						Outturn by End September						Performance
Billion Uganda Shillings	Wage	Non-Wage	Gou Dev	Ext. Fin	GoU Total	Total GoU + Ext. Fin	Wage	NonWage	GoU Dev	Ext. Fin	Total GoU	Total GoU + Ext. Fin	%GoU Budget Spent
**Vote: 009 Ministry of Internal Affairs**	2.448	54.447	3.647	0.000	**60.543**	**60.543**	0.360	10.380	0.000	0.000	**10.741**	**10.741**	**17.74%**
07 Peace Building	0.000	2.751	0.000	0.000	**2.751**	**2.751**	0.000	0.152	0.000	0.000	**0.152**	**0.152**	**5.54%**
08 Police and Prisons Supervision	0.000	1.584	0.000	0.000	**1.584**	**1.584**	0.000	0.231	0.000	0.000	**0.231**	**0.231**	**14.55%**
**Vote: 010 Ministry of Agriculture, Animal Industry and Fisheries**	19.407	36.433	131.762	376.784	**187.602**	**564.386**	4.536	4.571	1.685	0.000	**10.791**	**10.791**	**5.75%**
01 Agriculture Extension Services	0.620	1.190	2.330	0.000	**4.140**	**4.140**	0.120	0.060	0.006	0.000	**0.186**	**0.186**	**4.49%**
02 Agriculture Infrastructure and Mechanization Development	0.557	3.165	62.420	37.210	**66.142**	**103.352**	0.139	0.005	0.524	0.000	**0.668**	**0.668**	**1.01%**
03 Animal Resources	3.047	4.121	22.513	18.440	**29.681**	**48.121**	0.675	0.171	0.116	0.000	**0.962**	**0.962**	**3.24%**
04 Crop Resources	3.108	3.165	17.906	247.384	**24.179**	**271.564**	0.737	0.101	0.295	0.000	**1.133**	**1.133**	**4.69%**
05 Fisheries Resources	1.866	3.465	7.422	0.000	**12.753**	**12.753**	0.455	0.354	0.378	0.000	**1.187**	**1.187**	**9.31%**
06 Policy, Planning and Support Services	10.210	21.327	19.171	73.750	**50.707**	**124.457**	2.410	3.880	0.366	0.000	**6.655**	**6.655**	**13.13%**
**Vote: 011 Ministry of Local Government**	23.865	29.127	14.834	123.852	**67.826**	**191.678**	1.992	2.208	0.298	0.000	**4.497**	**4.497**	**6.63%**
01 Local Government Administration and Development	22.054	1.301	2.100	114.852	**25.455**	**140.307**	1.651	0.060	0.031	0.000	**1.742**	**1.742**	**6.84%**
02 Local Government Inspection and Assessment	0.639	0.759	2.620	9.000	**4.018**	**13.018**	0.113	0.042	0.000	0.000	**0.156**	**0.156**	**3.87%**
03 Policy, Planning and Support Services	1.172	27.066	10.114	0.000	**38.353**	**38.353**	0.228	2.105	0.267	0.000	**2.600**	**2.600**	**6.78%**
**Vote: 012 Ministry of Lands, Housing & Urban Development**	11.194	62.173	14.578	180.216	**87.945**	**268.161**	2.476	6.606	0.030	0.000	**9.112**	**9.112**	**10.36%**
01 Housing	0.368	0.772	0.000	0.000	**1.140**	**1.140**	0.064	0.024	0.000	0.000	**0.087**	**0.087**	**7.66%**
02 Land, Administration and Management	8.706	10.093	12.670	92.190	**31.469**	**123.659**	1.986	0.293	0.021	0.000	**2.299**	**2.299**	**7.31%**
03 Physical Planning and Urban Development	0.794	5.127	0.508	88.026	**6.430**	**94.456**	0.165	0.130	0.000	0.000	**0.294**	**0.294**	**4.58%**
04 Policy, Planning and Support Services	1.326	46.180	1.400	0.000	**48.906**	**48.906**	0.262	6.159	0.010	0.000	**6.431**	**6.431**	**13.15%**
**Vote: 013 Ministry of Education and Sports**	124.598	245.095	101.704	189.922	**471.396**	**661.318**	4.157	26.676	0.233	0.000	**31.067**	**31.067**	**6.59%**
01 Career Guidance, Counselling and Placement	0.165	0.707	0.000	0.000	**0.872**	**0.872**	0.041	0.000	0.000	0.000	**0.041**	**0.041**	**4.74%**
02 Higher Education	8.910	65.825	0.296	9.661	**75.031**	**84.692**	1.815	4.662	0.002	0.000	**6.479**	**6.479**	**8.63%**
03 Sports and PE	0.125	14.993	0.000	0.000	**15.118**	**15.118**	0.031	1.500	0.000	0.000	**1.531**	**1.531**	**10.13%**
04 Policy, Planning and Support Services	98.858	50.252	48.503	0.000	**197.613**	**197.613**	0.746	5.030	0.000	0.000	**5.776**	**5.776**	**2.92%**
05 Basic and Secondary Education	1.879	35.534	43.062	33.188	**80.476**	**113.664**	0.416	0.125	0.041	0.000	**0.582**	**0.582**	**0.72%**
06 Quality and Standards	1.599	2.789	0.000	0.000	**4.387**	**4.387**	0.000	0.081	0.000	0.000	**0.081**	**0.081**	**1.84%**
07 Technical Vocational Education and Training	12.856	74.023	7.145	147.073	**94.024**	**241.097**	1.057	15.278	0.190	0.000	**16.525**	**16.525**	**17.58%**
08 Special Needs Education	0.204	0.972	2.698	0.000	**3.874**	**3.874**	0.051	0.000	0.000	0.000	**0.051**	**0.051**	**1.32%**

ANNEX 4: APPROVED BUDGET AND QUARTER ONE OUTTURNS FOR FY2022/23 BY SUB SUBPROGRAMME (Excl. Arrears and AIA)

	Approved Estimates						Outturn by End September						Performance
Billion Uganda Shillings	Wage	Non-Wage	Gou Dev	Ext. Fin	GoU Total	Total GoU + Ext. Fin	Wage	NonWage	GoU Dev	Ext. Fin	Total GoU	Total GoU + Ext. Fin	%GoU Budget Spent
**Vote: 014 Ministry of Health**	21.645	138.787	89.977	1,333.540	**250.408**	**1,583.949**	5.276	25.815	0.415	0.000	**31.506**	**31.506**	**12.58%**
01 Curative Services	4.929	98.733	0.000	0.000	**103.662**	**103.662**	1.213	21.971	0.000	0.000	**23.183**	**23.183**	**22.36%**
02 Strategy, Policy and Development	1.427	8.671	69.035	152.240	**79.134**	**231.374**	0.325	0.204	0.000	0.000	**0.530**	**0.530**	**0.67%**
03 Support Services	10.303	18.386	0.272	0.000	**28.961**	**28.961**	2.529	2.806	0.000	0.000	**5.335**	**5.335**	**18.42%**
04 Health Governance and Regulation	0.793	3.250	0.000	0.000	**4.043**	**4.043**	0.182	0.342	0.000	0.000	**0.524**	**0.524**	**12.97%**
05 Public Health Services	4.193	9.746	20.669	1,181.301	**34.608**	**1,215.909**	1.027	0.492	0.415	0.000	**1.933**	**1.933**	**5.59%**
**Vote: 015 Ministry of Trade, Industry and Co-operatives**	3.174	110.876	23.055	0.000	**137.105**	**137.105**	0.716	3.810	0.000	0.000	**4.526**	**4.526**	**3.30%**
01 Trade Development	0.489	0.779	0.000	0.000	**1.269**	**1.269**	0.100	0.172	0.000	0.000	**0.272**	**0.272**	**21.47%**
02 Regulation and Management of Cooperatives	0.229	49.121	0.000	0.000	**49.350**	**49.350**	0.037	0.055	0.000	0.000	**0.092**	**0.092**	**0.19%**
[truncated: 559,756 more chars]
